# DARS-RNP and QUASI-RNP: New statistical potentials for protein-RNA docking

**DOI:** 10.1186/1471-2105-12-348

**Published:** 2011-08-18

**Authors:** Irina Tuszynska, Janusz M Bujnicki

**Affiliations:** 1 Laboratory of Bioinformatics and Protein Engineering, International Institute of Molecular and Cell Biology, ul. Ks. Trojdena 4, PL-02-109 Warsaw, Poland; 2 Institute of Molecular Biology and Biotechnology, Adam Mickiewicz University, ul. Umultowska 89, PL-61-614 Poznan, Poland

**Keywords:** RNA, protein, RNP, macromolecular docking, complex modeling, structural bioinformatics

## Abstract

**Background:**

Protein-RNA interactions play fundamental roles in many biological processes. Understanding the molecular mechanism of protein-RNA recognition and formation of protein-RNA complexes is a major challenge in structural biology. Unfortunately, the experimental determination of protein-RNA complexes is tedious and difficult, both by X-ray crystallography and NMR. For many interacting proteins and RNAs the individual structures are available, enabling computational prediction of complex structures by computational docking. However, methods for protein-RNA docking remain scarce, in particular in comparison to the numerous methods for protein-protein docking.

**Results:**

We developed two medium-resolution, knowledge-based potentials for scoring protein-RNA models obtained by docking: the quasi-chemical potential (QUASI-RNP) and the Decoys As the Reference State potential (DARS-RNP). Both potentials use a coarse-grained representation for both RNA and protein molecules and are capable of dealing with RNA structures with posttranscriptionally modified residues. We compared the discriminative power of DARS-RNP and QUASI-RNP for selecting rigid-body docking poses with the potentials previously developed by the Varani and Fernandez groups.

**Conclusions:**

In both bound and unbound docking tests, DARS-RNP showed the highest ability to identify native-like structures. Python implementations of DARS-RNP and QUASI-RNP are freely available for download at http://iimcb.genesilico.pl/RNP/

## Background

Protein-RNA interactions play fundamental roles in many biological processes, such as regulation of gene expression, RNA splicing, protein synthesis, replication of viral RNAs, and virus assembly (review: [1]). Defects of protein-RNA interactions are responsible for many human diseases ranging from neurological disorders to cancer [2]. The understanding of these processes improves as new structures of protein-RNA complexes are solved and the molecular details of interactions analyzed. Unfortunately, the experimental determination of protein-RNA complexes is a slow and difficult process [3,4]. The ability to predict structures of protein-RNA complexes computationally would greatly help us study protein-RNA interactions. However, while there is a growing number of methods for modeling protein and RNA structures (reviews: [5,6]), the number of methods for modeling protein-RNA complexes remains scarce.

Docking methods are widely used to predict structures of macromolecular complexes, starting from structures of the individual components [7]. All docking methods face two main challenges: to search the space of possible orientations and conformations (poses) of the components and to identify near-native structures among the alternative complex models (decoys) generated. An ideal docking method should be able to reliably reconstruct a native complex from its 'bound' components, and score it significantly better than any non-native decoys. In real life, the structure of the complex is unknown, and while the structures of binding partners are solved in isolation, the task of the 'unbound' docking experiment is not only to assemble them into a complex, but also to take into account possible conformational changes upon binding. Conformational changes are either modeled explicitly at the atomic level (which makes such analyses computationally very demanding), or a certain level of 'fuzziness' is introduced e.g. by allowing for some extent of steric overlaps between atoms or by 'coarse-graining' of the representation i.e. by neglecting some atoms or grouping them into 'united atoms' to be considered jointly (reviews: [7,8]).

One interesting and frequently neglected aspect of RNA structure and interactions is the presence of posttranscriptional modifications, which increase the basic set of four nucleotides (A, U, G, C) to more than 100 variants with altered base and/or ribose moieties [9]. Modified residues in RNA are involved in many processes, including RNA folding and RNA-RNA interactions (reviews: [10]), but also specific RNA-protein recognition and binding [11,12]. To our knowledge, among freely available macromolecular docking methods only three are suitable for handling post-transcriptional nucleotide modifications in RNA without the need of 'demodification'. HADDOCK accepts RNA as a part of the complex to be predicted, but the user is required to provide force field parameters for all modified nucleotides [13]. GRAMM [14] and HEX [15] can also perform protein-RNA docking for RNA structures with modified nucleotides, but the scoring functions of these programs have been developed to evaluate protein-protein complexes, and while they can generate the poses for protein-RNA complexes, they are unable to identify near-native variants from a set of decoys. A useful extension of the latter kind of methods would be the development of a potential for scoring protein-RNA complexes.

Recently, new statistical potentials for scoring protein-RNA complexes have been proposed: a distance-dependent all-atom potential developed by the Varani group [16], and a residue-level potential developed by the Fernandez group [17]. The Varani potential performs well in discriminating models of protein-RNA complexes that are very close to the native structure, i.e. with the root mean square deviation (RMSD) < 5 Å [16]. However, during a real (unbound) docking experiment it is difficult to obtain many decoys with RMSD < 5 Å. The Fernandez potential was designed to improve the discriminative power of the FTDock potential and is not available as a standalone program. The FTDock program [18] was developed for protein-protein and protein-DNA docking, but it accepts only conventional RNA molecules (without modified nucleotides).

In this article we introduce two new, medium-resolution, knowledge-based potentials for scoring protein-RNA models: the quasi-chemical potential (QUASI-RNP) and the Decoys As the Reference State [19] potential (DARS-RNP). These potentials are based on a reduced representation of protein and RNA, use the same mathematical base but differ in their reference state.

We compare the discriminative power of our new statistical potentials to the Varani and Fernandez potentials. For the bound docking test set our potentials discriminated native-like (with RMSD < 10 Å) structures of protein-RNA complexes, the potential developed by the Varani group recognized structures very close to the native (RMSD < 5 Å), whereas the Fernandez potential recognized near native structures only for some protein-RNA complexes. For the unbound docking test set, our potentials have the highest discriminative ability of alternative models. Our new knowledge-based potentials are a useful tool for scoring protein-RNA complexes generated by macromolecular docking methods, such as GRAMM or Hex.

### Implementation

#### Docking

To search the space of possible orientations and conformations of the components, we employed the GRAMM method for medium- to low-resolution docking [20]. As opposed to high-resolution methods that typically operate in the continuous space, GRAMM discretizes the system (thereby lowers its resolution) by projecting the macromolecular structures on a grid and allows for imprecise fit by 'softening' the van der Waals interactions and permitting some degree of steric conflicts. One of the components of a binary complex, referred to as the "receptor", is fixed, while the other component, referred to as the "ligand", is rotated and translated around the receptor to obtain geometrically compatible poses.

The van der Waals radius was used as a projection of an atom. The value of the grid step was set to 1.7 Å for complexes from the bound docking set and to the minimal value allowed by the program for complexes from the unbound docking set, a repulsion parameter to 10 (attraction is always -1) and attraction double range to 0. Ligand structures were rotated by 10° angle intervals. If significant steric clashes were observed in a large fraction of models obtained in preliminary GRAMM runs, as assessed by visual inspection, the repulsion parameter was increased to decrease the volume of steric conflict, until docking decoys reached physically reasonable geometry (Additional file 1, Table S1). We defined "native-like" poses as those with the ligand RMSD < 10 Å from the native complex structure. According to our experience, this value is appropriate for consideration of medium- and low-resolution docking experiments. The distance between the experimentally determined and decoy complex structure was calculated as the RMSD of all heavy atoms of ligands, following optimal superposition of the receptor structures.

The resulting sets of decoys are supposed to approximate a broad distribution of structures that exhibit relatively good spatial complementarity between the receptor and the ligand, regardless of other interactions that contribute to the observed strength of binding. Such sets may be enriched into native-like decoys comparably to the completely random models (and in fact, in some cases they are), but "spatially complementary" models are much better approximation of the real-life sampling. The false models were not selected by the GRAMM scores, as we found that the GRAMM score alone is a poor predictor of the complex native-likeness (data not shown). We treated all 10000 decoys per complex as equal, both for training of our potential, and for evaluation with all four potentials analyzed in this work.

#### Statistical potentials used

The distance and orientation-dependent knowledge-based potentials described in this article (DARS-RNP and QUASI-RNP) were generated using reverse Boltzmann statistics, where the interaction energy *ε *between the united atom type from the protein *i *and the united atom type from the RNA *j *is calculated according to Formula (1)

(1) $$\epsilon \left(\text{i,j,d}\right)=-RTln\frac{Nobs\left(i,j,d\right)}{Nexp\left(i,j,d\right)}$$

where *R *is the gas constant, *T *is the temperature, *N_obs _(i, j, d) *is the number of contacts between atom types *i *and *j *observed in the training set in a distance/angular bin *d*, while *N_exp _(i, j, d) *is the expected number of contacts at the same distance/angle in the reference state. There are two types of bins, connected with different terms of the potential: a distance bin (1 Å) used in the distance-dependent term, and an angular bin (20°) used in the angular-dependent term. The energy is calculated for each pair of protein-RNA united atoms that are within the distance of 9 Å from each other. This threshold parameter was chosen based on the analysis carried out by the Shakhnovich group [21], who identified 7 Å as the optimal threshold for evaluating protein-nucleic acid interactions with all-atom multiple bin potentials. For our reduced representation potentials we have tested five distance thresholds (7 Å, 8 Å, 9 Å, 10 Å and 11 Å) by calculating correlation coefficients and a position of the native structure in the decoys ranking. The best results was for 9 Å for both DARS and QUASI potentials (slightly better than for 7 and 8), and it was not statistically distinguishable from 10 Å and 11 Å (data not shown).

Our two statistical potentials calculate the reference state in different ways. The reference state for the QUASI-RNP potential is calculated using mole fractions of residues:

(2) $$N_{\exp}\left(i,j,d\right)=X_{i}*X_{j}*N_{obs}\left(d\right),$$

where *X_i _*and *X_j _*are the mole fractions of atom type *i *and *j *respectively, while *N_obs _(d) *is the total number of contacts in bin *d*. On the other hand, in the DARS approach [19]*N_exp _(i, j, d) *is a normalized number of contacts between atom types i and j in a set of decoy structures that are considered as random models. 1000 decoys were generated for each native structure of protein-RNA complex from the training set by using the GRAMM docking program [20], with the following parameters: values for repulsion and attraction were 15 and 0, respectively, while a grid step used the minimal value allowed by the program, depending on the size of the components to be docked. In a few cases where default values led to generation of decoys with significant steric clashes, the repulsion value was increased stepwise, until physically realistic decoys were obtained. It must be emphasized that these decoys maximize the geometric fit between the protein and RNA molecule, but do not take any interaction energies into account.

**Figure 1:**
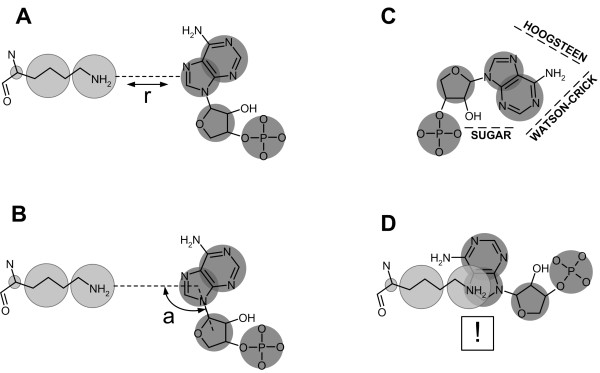
**Schematic representation of four terms used in DARS-RNP and QUASI-RNP statistical potentials: A, a distance-dependent term; B, an angular-dependent term; C, an edge-dependent term as a site term; D, a penalty for steric clashes**. United atoms of nucleotide and amino acid residues are colored in dark and light gray, respectively.

Both statistical potentials comprise a distance-dependent energy term (E_d_), an angular-dependent energy term (E_a_), a site-dependent energy term (E_s_), and a penalty for steric clashes (E_p_) (Figure 1):

(3) E=Ed+Ea+Es+Ep

The site-dependent term assesses the probability of interaction of amino acid residues with edges of nucleotide residues: Watson-Crick, Sugar and Hoogsteen, as defined by Leontis at.al [22] (Figure 1).

**Figure 2:**
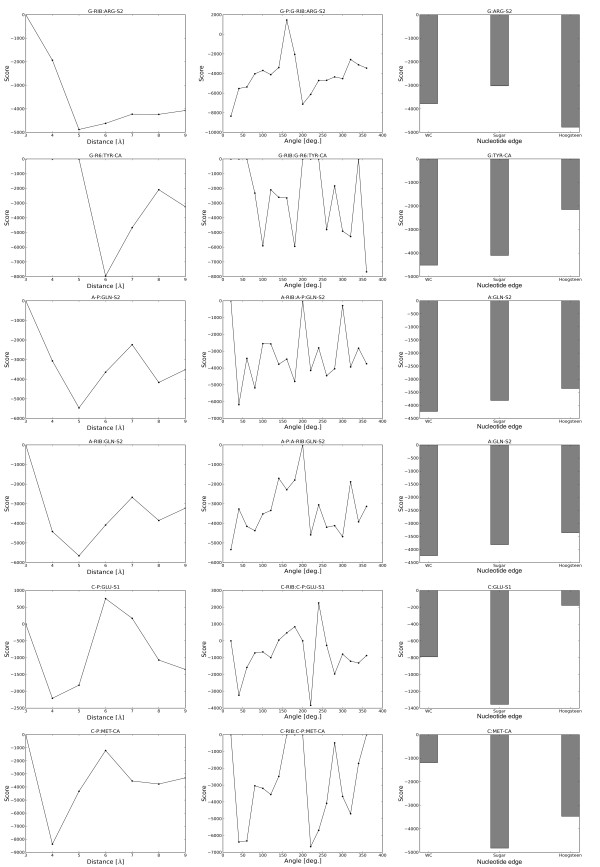
**Examples of value distributions for three terms of the DARS-RNP potential that describe interactions between six arbitrarily selected pars of united atoms**.

**Figure 3:**
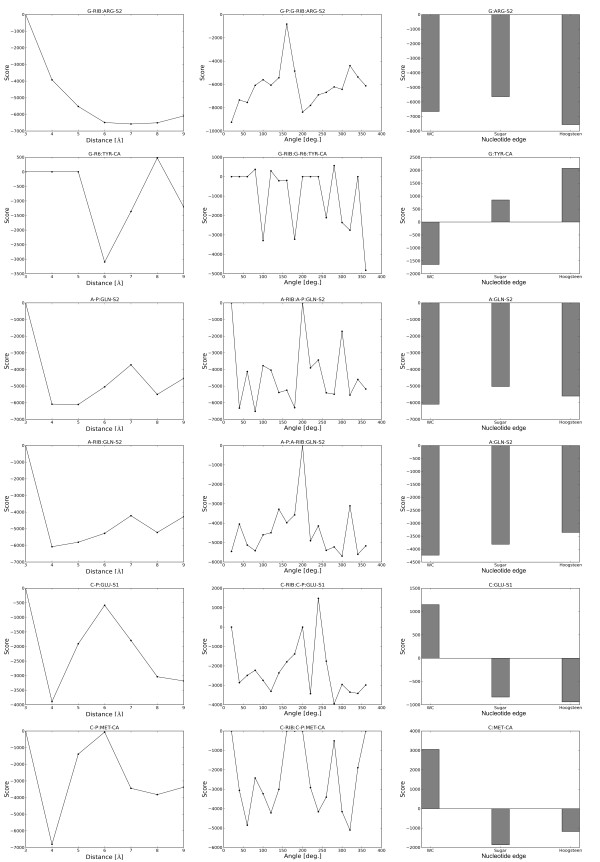
**Examples of value distributions for three terms of the QUASI-RNP potential that describe interactions between six arbitrarily selected pars of united atoms**.

All four terms of the energy function exhibit comparable values (see Figure 2 and 3 for graphical presentation of examples and Additional File 2 and Additional File 3 for the list of all values). Hence, we assigned equal weights to all four terms and have not optimized them further in any way. Additional file 1, Figures S5, S6 and S7 show selected graphs of N_obs _common for DARS-RNP and QUASI-RNP, N_exp _for DARS-RNP, and N_exp _for QUASI-RNP, respectively, as a function of distance, angle and nucleotide edges, while Additional Files 4, 5 and 6 include the list of values for all cases.

#### Representation of molecules

To calculate the parameters of QUASI-RNP and DARS-RNP potentials, the all-atom representation of all macromolecular structures was transformed into a reduced representation. The atoms of each amino acid or nucleotide residue were replaced by a number of united atoms depending on the residue type. For amino acid residues, we used the representation used in the REFINER program [23,24], which involves one to three united atoms per residue, depending on the size of the residue. For nucleotide residues we employed the representation used in the RedRNA method, currently under development in our laboratory (Michal Boniecki and J.M.B. unpublished data). The RNA backbone was represented by two united atoms, one for the phosphate group (P) and one for the ribose (RIB), while the pyrimidine and purine rings were represented by one and two atoms, respectively (Figure 1). All united atoms and alpha carbons from the reduced representation in every residue were considered for the potentials as separate atom types, e.g. the alpha carbon of alanine and alpha carbon of lysine had different types, because they represented different type of residues.

#### Training set for developing the statistical potentials

In order to compare our QUASI-RNP and DARS-RNP potentials to the previously published Varani potential we used the same training set as the Varani group [16]. There are 72 protein-RNA complexes in the training set taken from crystal structures of protein-RNA complexes downloaded from the Protein Data Bank (PDB codes: 1a1v, 1a34, 1b2m, 1c0a, 1d9d, 1dfu, 1di2, 1bu1, 1ec6, 1f7u, 1feu, 1ffy, 1fxl, 1gtf, 1gtr, 1hq1, 1j1u, 1jbs, 1jid, 1jj2 - 50S ribosome structure, 1k8w, 1knz, 1lng, 1m8w, 1mji, 1msw, 1n35, 1n78, 1ooa, 1qln, 1r3e, 1r9f, 1rc7, 1sds, 1tfw, 1u0b, 1urn, 1uvj, 1wsu, 1yvp, 1zbi, 1a8v, 2bgg, 2bh2) [25]. One of these is the 50S ribosomal subunit, which comprises of 28 individual peptide chains in complex with RNA. Due to the limited number of protein-RNA complexes in this set, we performed a "leave one out" cross-validation, in which the potential was recalculated for testing of each structure, based on a training set with the tested structure excluded.

Our software uses an in-house modified variant of PDBParser from Biopython (BrutePDBParser developed by Michal Pietal in IIMCB) to parse PDB files and ignore information about e.g. atom occupancy.

#### Testing statistical potentials for protein-RNA docking

To test the discriminatory power of the QUASI-RNP and DARS-RNP knowledge-based potentials we compared them with two existing statistical potentials developed by the Varani [16] and Fernandez [17] groups. Software to calculate the Varani potential has been kindly provided by the Author (Gabriele Varani, personal communication). Since the Fernandez potential is not available as a standalone software, the Authors have kindly provided raw statistical data for each amino acid-nucleotide pair calculated based on their training set (Juan Fernandez-Recio, personal communication), which we used to calculate our local implementation of their scoring function, independent of the FTDock program.

Two types of test sets were used, based on bound structures (components isolated from complex structures), or on unbound structures (counterparts of complex components, in which one structure or both were solved in isolation from each other).

##### Bound docking test sets

We used one set of molecules with unmodified (bound) conformations of entire RNA molecules and protein backbones, but with optimized protein side chains. This set was developed by the Varani group to perform decoy-discrimination tests of their all-atom potentials [16,26]. Their decoys were obtained by modifying five native protein-RNA complexes (PDB codes: 1cvj, 1ec6, 1fxl, 1jid, 1urn) using the docking module of ROSETTA, through the use of the protein side chain repacking algorithm [27,28]. For each protein complex they have generated 2000 structures with the RMSD from the native complex structure ranging from 0.2 Å to about 30 Å. The RMSD of two complexes was calculated based on ligand heavy atoms, after superposition of receptors.

The second set contains all molecules in unmodified (bound) conformations, and was generated by ourselves using the high-resolution mode of the GRAMM docking program. For each of the five protein-RNA complexes from the Varani test set 10,000 alternative docking decoys were generated according to the procedure described above in the Docking section.

##### Unbound docking test set

The unbound docking test set was based on twelve native protein-RNA complexes (PDB codes: 2rkj, 1wsu, 1ooa, 2r8s, 2pjp, 1lng, 2pxv, 1e7k, 1wpu, 3bso, 2qux, 2jea), previously used by the Fernandez group to test their potential [17]. For each component of these complexes, at least one independently solved 3D structure per complex is available. The GRAMM program was used to generate 10,000 docking decoys for each complex. We used the same parameters as in the bound docking procedure. With these settings, the GRAMM program generated at least five native-like structures (RMSD < 10 Å) for eight out of twelve protein-RNA complexes. Only these eight decoy sets with native-like structures were considered.

#### Clustering the best scored decoys

Critical assessment of protein structure predictions (in particular the CASP experiment) has demonstrated that the scoring functions alone may not be the best discriminators of native-like structures, and better results may be achieved by clustering well-scored suboptimal structures [29,30]. We have applied this approach, in particular using the clustering algorithm proposed by Baker and coworkers [31], which has worked very well in protein structure prediction. First, an RMSD is calculated for all pairs of structures and stored in a distance matrix. Then, the row of the distance matrix with the largest number of RMSD values smaller than a cutoff (default 5 Å) is selected. Structures in that row with RMSD below the cutoff value are assigned to one cluster and excluded from the matrix. The process is iterated until all structures with RMSD smaller than the cutoff are assigned to clusters. Three biggest clusters are then considered as candidates for groups of potentially native-like structures, from which then the lowest-energy decoys are identified. As a rule of thumb, the larger the cluster and the better score of its decoys - the higher chance that it contains a native-like structure.

## Results

In order to compare the ability to identify near-native protein-RNA complex structures by our two potentials (QUASI-RNP and DARS-RNP), the potential developed by Varani et al. [16], and the potential developed by Fernandez et al. [17], we used test sets, obtained by bound and unbound docking. To give all methods equal chances in finding structures close to the native one, we included the clustering procedure implemented in our potentials as an additional step in the scoring performed by both the Varani and Fernandez potentials. We have calculated correlation coefficients for decoys with RMSD in the range of 0-5 Å, 0-10 Å and 0-20 Å. The RMSD range < 5 Å was chosen because the Varani potential has a recognition area of near-native structures around 5 Å. The RMSD threshold 10 Å is a "golden standard" of near to native decoys definition used in the CAPRI (Critical Assessment of PRediction of Interactions) experiment. The generous 20 Å threshold was used because we observed that for large structures there exist decoys with a biologically reasonable match of the binding sites and a number of native-like protein-RNA contacts, despite the global RMSD of the ligand in the range of 10-20 Å. Such deviations are typically due to a rotation of the ligand that retains contacts in the binding site, but moves around atoms that are far away from the binding site, as shown e.g. in Additional file 1, Figures S1 and S2.

### Decoy discrimination for the bound docking test set

**Figure 4:**
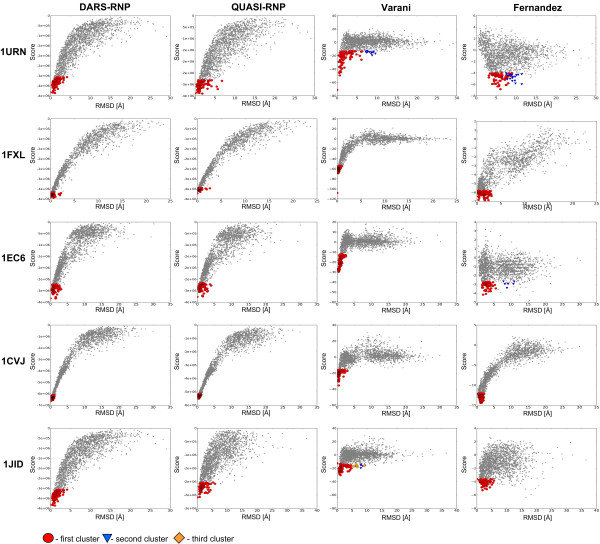
**Score-RMSD dependence for the Varani bound docking set**. The three best clusters (1^st^, 2^nd^, 3^rd^) are colored in orange, violet, and blue, and the corresponding points are marked as squares, triangles, and rounds, respectively.

We tested the DARS-RNP, QUASI-RNP, Varani, and Fernandez potentials for scoring of RNA-protein complexes on two bound test sets, and examined which potential gives the best correlation coefficient between scores and RMSD of the corresponding decoys. For the Varani test set, the DARS-RNP and QUASI-RNP potentials recognized most structures with ligand RMSD < 10 Å. We found strong correlation between DARS-RNP scores and RMSD, as well between QUASI-RNP scores and RMSD for models up to 10 Å from the native structure (Figure 4, Table 1). The Varani potential discriminates models with ligand RMSD < 5 Å, but generally fails to distinguish structures with 5-10 Å RMSD from the native and random structures with larger RMSD values (Figure 4 and Additional file 1, Figure S3). The Fernandez potential fails to recognize individual near-native decoys from the Varani test set; and its scores exhibit positive correlation coefficients with RMSDs only for decoys from three complexes in five (Table 1). The clustering procedure improves the results obtained by the Fernendez potential, as the biggest clusters contain structures with RMSD < 5 Å for all complexes considered. Nonetheless, the native structures and structures with the smallest RMSD are scored too poorly by the Fernandez potential to be included in the top clusters for two of five complexes - 1URN and 1EC6 (Figure 4).

**Table 1:** Results of scoring the decoys in the Varani bound docking set

Complex PDB code	RMSD threshold (decoy vs native)	Number of decoys below threshold (per 2,000)	Correlation coefficient			
			
			DARSI-RNP potential (std. error)	QUASI-RNP potential (std. error)	Varani potential (std. error)	Fernandez potential (std. error)
1urn	5	726	0.77 (0.024)	0.7 (0.027)	0.37 (0.035)	-0.56 (0.031)
	
	10	1457	0.83 (0.015)	0.79 (0.016)	0.27 (0.025)	-0.24 (0.025)
	
	20	1967	0.81 (0.013)	0.79 (0.014)	0.21 (0.022)	-0.1 (0.022)

1ec6	5	907	0.81 (0.019)	0.79 (0.020)	0.57 (0.027)	-0.08 (0.033)
	
	10	1446	0.9 (0.011)	0.89 (0.012)	0.38 (0.024)	-0.05 (0.026)
	
	20	1979	0.87 (0.011)	0.86 (0.011)	0.31 (0.021)	-0.02 (0.022)

1fxl	5	881	0.94 (0.012)	0.95 (0.011)	0.87 (0.017)	0.61 (0.027)
	
	10	1366	0.96 (0.008)	0.96 (0.008)	0.82 (0.015)	0.74 (0.018)
	
	20	1758	0.93 (0.009)	0.94 (0.008)	0.7 (0.017)	0.83 (0.013)

1cvj	5	936	0.96 (0.009)	0.96 (0.009)	0.5 (0.028)	0.85 (0.017)
	
	10	1217	0.97 (0.007)	0.97 (0.007)	0.61 (0.023)	0.92 (0.011)
	
	20	1947	0.93 (0.008)	0.93 (0.008)	0.46 (0.020)	0.9 (0.010)

1jid	5	828	0.58 (0.028)	0.58 (0.028)	0.35 (0.033)	0.33 (0.033)
	
	10	1485	0.72 (0.018)	0.71 (0.018)	0.3 (0.025)	0.39 (0.024)
	
	20	1978	0.7 (0.016)	0.69 (0.016)	0.27 (0.022)	0.29 (0.022)

Mean	5	855.6	0.81	0.8	0.53	0.23
	
	10	1394.2	0.88	0.87	0.47	0.35
	
	20	1925.8	0.85	0.84	0.39	0.38

Correlation coefficients were calculated for scatter plots from Figure 4.

**Figure 5:**
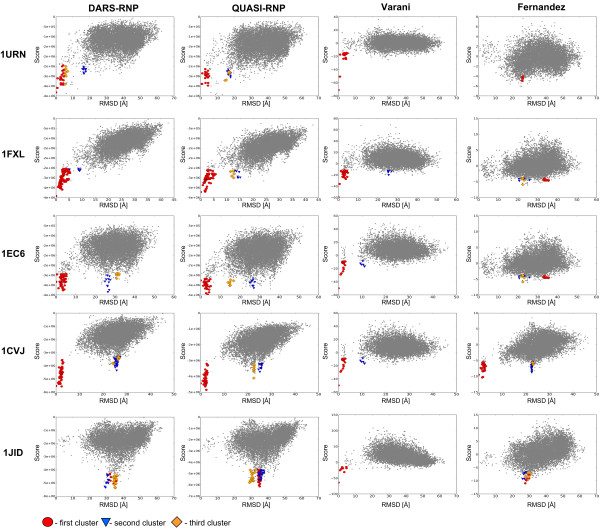
**Score-RMSD dependence for the GRAMM-generated bound docking set**. The three best clusters (1^st^, 2^nd^, 3^rd^) are colored in orange, violet, and blue, and the corresponding points are marked as squares, triangles, and rounds, respectively.

In the bound docking test set generated by the GRAMM program, there is a smaller number of near-native structures than in the Varani set, and some of them exhibit steric clashes. For this test set, our DARS-RNP and QUASI-RNP potentials exhibit lower values of the correlation coefficient than for the Varani test set (Additional file 1, Figure S3). The Varani and Fernanez potentials discriminate GRAMM decoys better than Varani decoys for three complexes, and worse for two complexes (1urn and 1jid) (Figure 5) (Table 1 and Table 2).

**Table 2:** Results of scoring the decoys in the GRAMM-generated bound docking set

Complex PDB code	RMSD threshold (decoy vs native)	Number of decoys below threshold (per 10,000)	Correlation coefficient			
	
			DARS-RNP potential (std. error)	QUASI-RNP potential (std. error)	Varani potential (std. error)	Fernandez potential (std. error)
1urn	5	21	0.38 (0.212)	0.25 (0.222)	0.45 (0.205)	-0.15 (0.227)
	
	10	72	0.51 (0.103)	0.41 (0.109)	0.46 (0.106)	-0.01 (0.120)
	
	20	352	0.6 (0.043)	0.45 (0.048)	0.29 (0.051)	-0.42 (0.049)

1ec6	5	87	0.3 (0.103)	0.28 (0.104)	0.4 (0.099)	-0.12 (0.108)
	
	10	155	0.7 (0.058)	0.68 (0.059)	0.53 (0.069)	0.03 (0.081)
	
	20	1079	0.65 (0.023)	0.56 (0.025)	0.31 (0.029)	0.18 (0.030)

1fxl	5	81	0.62 (0.088)	0.64 (0.086)	0.62 (0.088)	0.46 (0.100)
	
	10	143	0.81 (0.049)	0.82 (0.048)	0.61 (0.067)	0.62 (0.066)
	
	20	1732	0.61 (0.019)	0.52 (0.021)	0.3 (0.023)	0.29 (0.023)

1cvj	5	38	0.62 (0.131)	0.49 (0.145)	0.55 (0.139)	0.34 (0.157)
	
	10	76	0.95 (0.036)	0.91 (0.048)	0.48 (0.102)	0.72 (0.081)
	
	20	1638	0.49 (0.022)	0.43 (0.022)	0.18 (0.024)	0.5 (0.021)

1jid	5	16	0.13 (0.265)	0.18 (0.263)	0.73 (0.183)	0.54 (0.225)
	
	10	29	0.13 (0.191)	0.09 (0.192)	0.59 (0.155)	0.28 (0.185)
	
	20	175	-0.06 (0.076)	0.04 (0.076)	0.58 (0.062)	0.2 (0.074)

Mean	5	49	0.41	0.37	0.55	0.22
	
	10	95	0.62	0.58	0.54	0.33
	
	20	995	0.46	0.4	0.33	0.15

Correlation coefficients were calculated for scatter plots from Figure 5.

Summarizing, the DARS-RNP and QUASI-RNP scores exhibit the highest correlation coefficients for all cutoffs in the Varani test set, and for 10 Å and 20 Å thresholds in the GRAMM test set (Additional file 1, Figure S3). Thus, DARS-RNP and QUASI-RNP potentials can be declared as "winners" of the bound docking test, except for the structures that are very close to the native complexes, where they are outperformed by the Varani potential.

### Decoy discrimination for the unbound docking set

**Figure 6:**
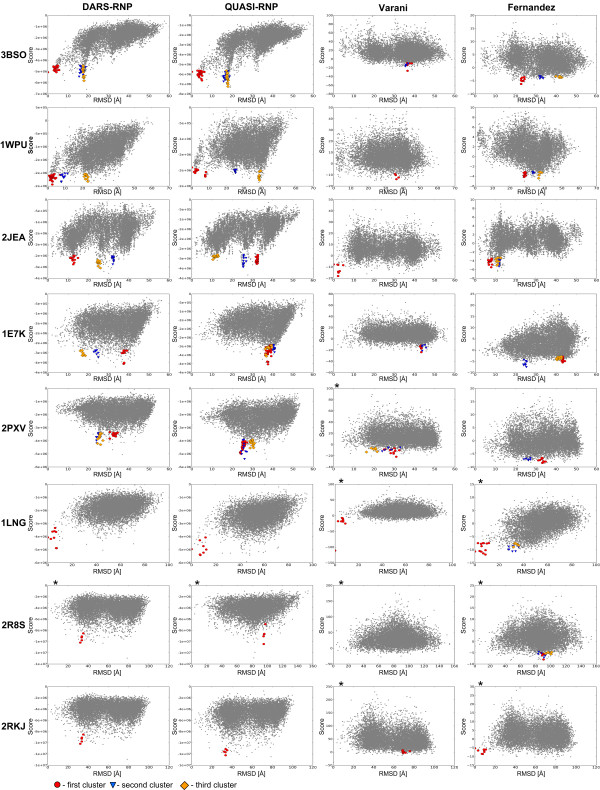
**Score-RMSD dependence for the GRAMM-generated unbound docking set as results for clustering of 100 best-scored docking decoys with the RMSD threshold of 5 Å**. * - no clusters found for 100 best scored decoys and 5 Å threshold, results reported for 200 best scored decoys and the RMSD threshold of 10 Å. The three best clusters (1^st^, 2^nd^, 3^rd^) are colored in red, blue, and orange, and the corresponding points are marked as squares, triangles, and rounds, respectively.

**Figure 7:**
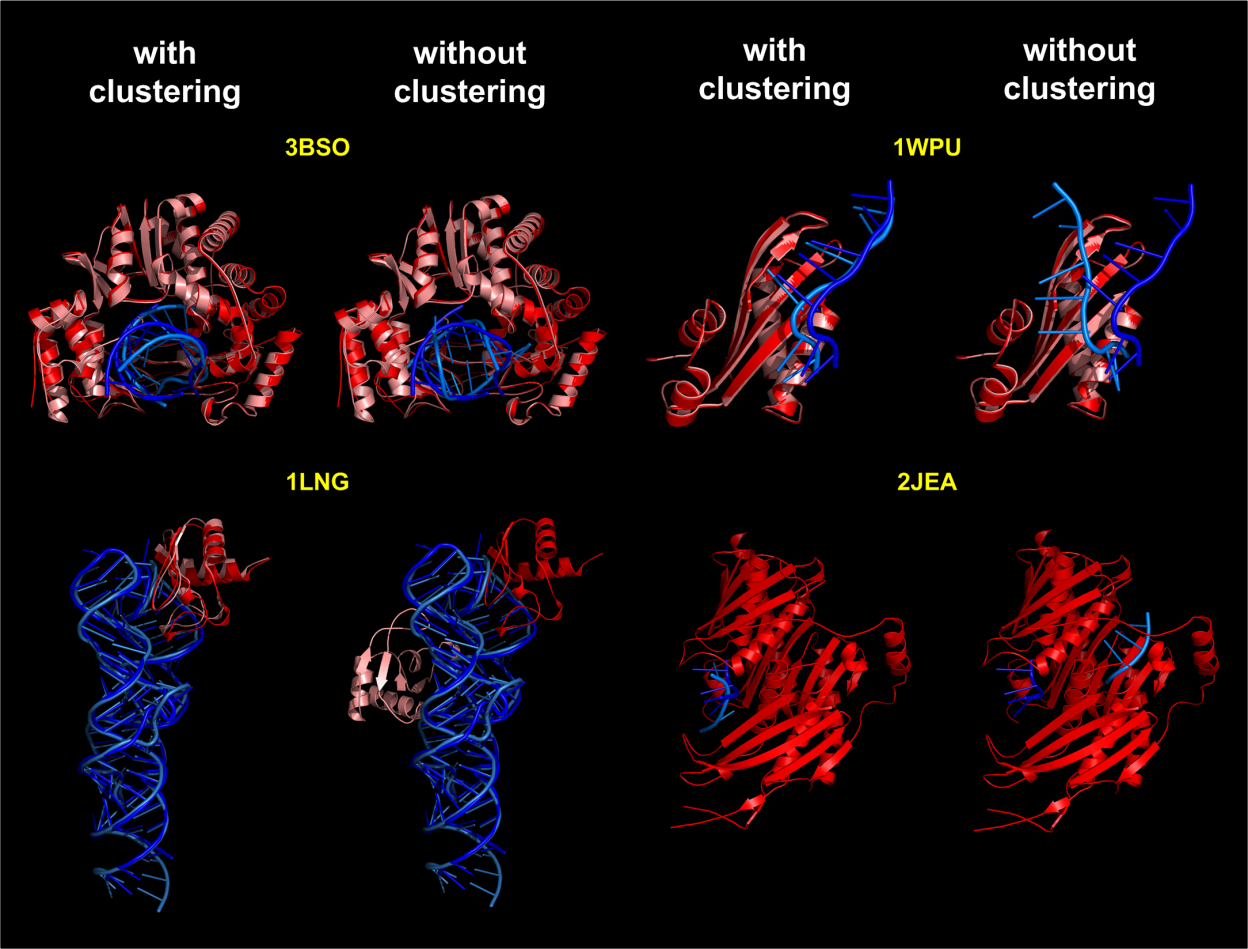
**Examples demonstrating the utility of clustering the best-scored decoys for the identification of native-like RNP structures**. Four cases scored by the DARS-RNP potential have been selected from the analysis presented in Figure 6. The structure with the lowest energy (score) is selected as a representative of a cluster. Native structures are in dark colors (blue -RNA and red - protein), while decoys are indicated by light colors (light blue - RNA and salmon pink - protein). Bigger components are superimposed.

In an analogous way, we examined the discriminatory power of the DARS-RNP, QUASI-RNP, Varani, and Fernandez potentials for decoys of the unbound docking test set. The assessment of unbound docking results reveals, expectedly, that all potentials exhibit worse results than for the bound docking set (Figure 6). Here, the best results are obtained by our DARS-RNP potential, followed closely by the QUASI-RNP potential. These potentials recognized native-like structures for four out of eight complexes from the unbound test set (Figure 6 and 7), while the Varani and Fernandez potentials recognized native-like structures only for one complex in this set with the default options of clustering, and two and three complexes respectively after increasing both number of clustering structures and RMSD threshold to 200 and 10 Å (Figure 6). The correlation coefficients between the model score and RMSD are relatively low for the Varani potential (0.06, 0.06, and 0.01 for RMSD thresholds of 5, 10, and 20 Å, respectively) and for the Fernandez potential (-0.13, -0.04 and 0.13), while the DARS-RNP/QUASI-RNP potentials exhibit correlation coefficients of 0.44/0.48, 0.25/0.23, and 0.37/0.33 for decoys with RMSD from the native structure lesser than 5, 10, and 20 Å, respectively (Table 3) and (Additional file 1, Figure S4).

**Table 3:** Results of scoring the decoys in the GRAMM-generated unbound docking set

Complex PDB code	Protein/ RNA PDB codes	RMSD threshold (decoy vs native)	Number of decoys below threshold (per 10,000)	Correlation coefficient			
	
				DARS-RNP potential (std. error)	QUASI-RNP potential (std. error)	Varani potential (std. error)	Fernandez potential (std. error)
2rkj	1y42/ 1y0q	5	4	-0.58 (0.576)	0.17 (0.697)	-0.6 (0.566)	1 (0.000)
	
		10	15	-0.25 (0.269)	-0.11 (0.276)	0.34 (0.261)	0.31 (0.264)
	
		20	87	0.09 (0.108)	0.21 (0.106)	0.06 (0.108)	0.51 (0.093)

2r8s	2r8s/ 1hr2	5	3	1 (0.000)	1 (0.000)	-1 (0.000)	-1 (0.000)
	
		10	6	0.7 (0.357)	0.73 (0.342)	-0.6 (0.400)	-0.33 0.472)
	
		20	13	0.72 (0.209)	0.64 (0.232)	-0.47 (0.266)	-0.43 (0.272)

1lng	1lng/ 1z43	5	2	-	-	-	-
	
		10	18	0.1 (0.249)	-0.15 (0.247)	0.1 (0.249)	0.14 (0.248)
	
		20	48	0.73 (0.101)	0.71 (0.104)	0.4 (0.135)	0.53 (0.125)

2pxv	2pxv/ 1cql	5	14	0.69 (0.209)	0.02 (0.289)	0.28 (0.277)	-0.19 (0.283)
	
		10	131	-0.04 (0.088)	0.02 (0.088)	0.14 (0.087)	-0.03 (0.088)
	
		20	948	-0.08 (0.032)	-0.02 (0.033)	0.13 (0.032)	0.17 (0.032)

1e7k	2jnb/ 1e7k	5	7	-0.08 (0.446)	0.09 (0.445)	0.6 (0.358)	0.11 (0.444)
	
		10	46	0.31 (0.143)	0.39 (0.139)	0.26 (0.146)	0.2 (0.148)
	
		20	938	0.19 (0.032)	0.17 (0.032)	0.14 (0.032)	0.17 (0.032)

1wpu	1wpv/ 1wpu	5	125	0.63 (0.070)	0.67 (0.067)	-0.08 (0.090)	-0.3 (0.086)
	
		10	227	0.42 (0.061)	0.34 (0.063)	0.02 (0.067)	-0.4 (0.061)
	
		20	1165	0.5 (0.025)	0.37 (0.027)	0.02 (0.029)	-0.21(0.029)

3bso	1sh0/ 3bso	5	34	0.48 (0.155)	0.32 (0.167)	-0.1 (0.176)	-0.09 (0.176)
	
		10	104	0.52 (0.085)	0.49 (0.086)	-0.03 (0.099)	-0.32 (0.094)
	
		20	1212	0.42 (0.026)	0.41 (0.026)	-0.21 (0.028)	0.09 (0.029)

2jea	2je6/ 2jea	5	35	0.16 (0.172)	0.22 (0.170)	0.34 (0.164)	0.44 (0.156)
	
		10	440	0.3 (0.046)	0.21 (0.047)	0.29 (0.046)	0.13 (0.047)
	
		20	3290	0.41 (0.016)	0.42 (0.016)	0.04 (0.017)	0.18 (0.017)
	
		5	28	0.32	0.26	0.21	-0.01

Mean		10	123.4	0.26	0.23	0.06	-0.04
	
		20	962.6	0.37	0.34	0.01	0.13

Correlation coefficients were calculated for scatter plots from Figure 6.

### Clustering of the best-scored decoys

The application of clustering to identify groups of similar structures among the top-scored decoys improves the predictive power of all statistical potentials analyzed in this work. It helps Fernandez potential to recognize near-native structures in the Varani set for bound docking with optimization of side chains (Figure 4), our DARS-RNP and QUASI-RNP potentials in the GRAMM bound docking test set (Figure 5), and all potentials in the unbound docking test set (Figure 6). Figure 7 shows examples of four complexes where the native-structure was found owing to the clustering of well-scored models identified by the DARS-RNP potential.

## Discussion

The QUASI-RNP and DARS-RNP potentials described in this work exhibit the highest discriminatory power for the bound Varani set, where there are many near-native structures without steric clashes. Likewise, our methods performed well for another set of decoys generated for the same RNA-protein complex structures with the GRAMM method. Our potentials failed to recognize native-like structures generated by GRAMM only for the 1JID structure (human SRP19 protein in complex with helix 6 of human SRP RNA). Both DARS-RNP and QUASI-RNP favored a structure that is very different from the native complex, even though they were able to recognize native-like structures for the same complex generated by Varani. The visual examination of models for the 1JID complex that were best-scored by our potentials revealed structures, in which the RNA backbone has entered a very deep and narrow groove on the protein structure far from the true RNA-binding site, leading to decoys with very big area of protein-RNA interactions, and hence with significantly more contacts than in the native structure (solvent-accessible surface area buried upon complex formation: ~2600 Å^2 ^vs ~1600 Å^2 ^for the misleading decoy and the native structure, respectively). One way to avoid such situations is to identify (or predict) the RNA-binding site on the protein surface and filter the initial decoys (e, g. using our method FILTREST3D [32]) to remove those with RNA away from the binding site.

It is worth to mention that the five structures in the bound docking test sets were excluded from the training set only for the QUASI-RNP and DARS-RNP potentials. The training set of the Fernandez potential contained three out of five assessed complexes, and the Varani training set contained all five complexes. Therefore, the ability of the Varani and Fernandez potentials to discriminate native-like models for these structures may be overestimated. In particular, the Varani potential has the best results for those decoys from the GRAMM-generated unbound set that are very close to native structure (RMSD < 5 Å). There, the Varani potential easily recognizes the structures close to those in its training set. Interestingly, for decoys from the same set, with RMSD up to 10 or 20 Å, our potentials still exhibit better results than the Varani potential, suggesting that they do have a power to discriminate between these medium-quality decoys and those that are totally native-unlike.

The results of tests for the unbound docking set are more objective due to a complete separation of training and testing data, and because they simulated the predictive power of the potentials in a real docking experiment, where the bound conformations of components are unknown. Among the four methods tested, QUASI-RNP and DARS-RNP potentials have the biggest average correlation coefficients between the scores and the RMSD of the model from the native structure. However, it must be emphasized that even these "winners" of our benchmark were able to identify native-like structures only for four out of eight tested cases (Figure 6 and 7). As expected, our potentials recognized near native structures only for these complexes, whose components exhibit relatively small structural changes (RMSD < 3 Å) during complex formation (Table 4). Still, in our hands, the Fernandez and Varani potentials recognized near-native structures for one complex only with the default options of clustering (100 best-scored decoys, RMSD threshold of 5 Å), and three and two complexes, respectively, after increasing the number of clustered best-scored decoys to 200 and the RMSD threshold to 10 Å (Figure 6 and Table 3). Such "relaxed" clusters are of course more heterogeneous. For two complexes (2JEA and 1LNG) all four potentials considered in this work have recognized native-like structures. For both of these complexes only one component of the complex was solved in isolation from the other (hence it was actually half-bound/half-unbound docking) and that 'unbound' component underwent only a very minor conformational change with respect to the bound form (RMSD < 3 Å) (Table 4). Hence, these two targets must be considered as very easy.

**Table 4:** Complexes considered in the unbound docking experiment, for which GRAMM produced near-native structures, with their PDB and chain identifiers

Recognition by DARS-RNP and QUASI-RNP	Molecule name	Complex PDB code	Receptor PDB code (and its RMSD vs the bound structure)	Ligand PDB code (and its RMSD vs the bound structure)
	Norwalk Virus polymerase with CTP/RNA primer	3bso_a:p	1sh0_b (1.3)	3bso_p (0.0)
	
Successful	HutP/Hut mRNA	1wpu_a:c	1wpv_a (0.2)	1wpu_c (0.0)
	
	9-subunit archaeal exosome/RNA	2jea_a, b:c	2je6_a, b (0.4)	2jea_c (0.0)
	
	SRP 19/7S.S SRP RNA	1lng_a:b	1lng_a (0.0)	1z43_a (2.1)

	Tyrosyl - tRNA synthetase splicing factor/group I intron RNA	2rkj_b:c	1y0q_a (3.0)	1y42_x (0.9)
	
Unsuccessful	SRP C-termina domain/4.5 S RNA	2pxv_a:b	1cql_a (8.1)	2pxv_a (0.0)
	
	spliceosomal 15.5 K protein/U4 snRNA fragment	1e7k_a:c	2jnb_a (3.2)	1e7k_c (0.0)
	
	Synthetic Fab/P4-P6 ribozyme domain	2r8s_l, h:r	1hr2_a (4.3)	2r8s_l, h (0.0)

Among the four knowledge-based potentials tested here for their ability to identify native-like protein-RNA docking models, the high-resolution Varani potential exhibited the best ability to recognize models closest to the native structure (RMSD < 5 Å). This potential appears as the method of choice for high-accuracy docking methods that are able to generate structures very close to the native ones. It must be emphasized, however, that there are no computational tools, with which to reliably predict conformational changes upon binding. Therefore high-resolution docking is only applicable in situations, where the receptor and ligand structures exhibit very little conformational change between the bound and unbound forms. Unfortunately, whether the conformational change occurs or not cannot be reliably predicted. In most cases of protein-RNA binding, moderate conformational changes of protein and/or RNA molecules occur upon complex formation. There, low resolution methods that apply a coarse-grained energy model to a coarse-grained representation (i.e. without looking at the atomic details that change upon binding) have a chance to be practically useful.

Among the four potentials tested in this work, the Fernandez potential has the weakest discriminatory power for identification of near-native structures, but we believe this potential may perform much better when combined with the FTDock scoring function, as originally intended by the Fernandez group. However, the combination of Fernandez and FTDock potentials is possible only for FTDock decoys, because the FTDock potential is not available as a standalone program, hence we could not apply it to the decoys generated in our study. FTDock is also unable to deal with modified residues in the RNA, which precludes it from applicability to many RNA-protein complexes, where RNA modifications play a critical role (e.g. interactions of tRNA with aminoacyl-tRNA synthetases).

The main difference between all potentials considered in this article is the (sub)set of atoms taken into consideration. The Varani potential considers interactions between all atoms, with chemically similar atoms (based on the CHARMM atom definition) treated in the same way. It contains only a distance-dependent multiple bin term. The Fernandez potential calculates interactions between entire residues represented as single interaction centers, using only one bin (i.e. the interaction is either present or absent). Our potentials use multiple bins for distance as well as orientation, hence they take into account more information about the possible arrangements of amino acid and nucleotide residues, even though they use less atoms than the Varani method.

In our study we have used both bound and unbound conformations for docking (with bound structures either completely unmodified, as in the GRAMM experiment, or with side-chains repacked, as in the Varani experiment). The shapes of score-RMSD dependence plots show differences associated not only with the methods, but also with the type of docking. As expected, all potentials exhibit best results for bound docking experiment, where there are many near-native structures without steric clashes. This observation underlines the influence of decoy generation method on the ability to successfully identify native-like decoys in the generated dataset.

Our study allows for direct comparison of the decoy-based and quasi-chemical approaches for calculating statistical potentials. The small, but significant average advantage of the DARS-RNP potential over the QUASI-RNP potential can be explained by the more realistic treatment of "random" protein-RNA interactions. In the DARS-based approach, the statistics of amino acid-nucleotide contacts are inferred from geometrically plausible, but biologically irrelevant decoys (pseudo-complexes), while the quasi-chemical approach predicts the occurrence of such contacts based on the frequency of individual residues. We expect this advantage of the DARS approach to hold also for other types of docking. The calculation of a DARS-based potential requires, however, the calculation of a large number of decoys for each complex in the training set, hence it requires considerably bigger computational effort, which may be prohibitive in case of large training sets.

By definition, none of the rigid-body docking methods analyzed here is capable of predicting the structures of complexes that involve large conformational changes. We also found that the presence of extensive steric clashes in decoys deteriorates the discriminatory power of all potentials tested in our benchmark. Thus, we propose that the next step in the development of methods for modeling of protein-RNA complexes should be taken towards algorithms that enable simultaneous docking and (re)folding of protein and RNA components. Recently, a number of methods for modeling of RNA 3D structures have been reported that utilize very similar methodology to that used for protein modeling [6,33]. This suggests that the combination of comparative modeling and "de novo" folding should be possible not only for proteins and RNA separately, but also as components of the same molecular system.

## Conclusions

Among the four potentials tested in this work the QUASI-RNP and DARS-RNP potentials exhibit the highest discriminatory power for both bound and unbound docking experiments. The small average advantage of the DARS-RNP potential over the QUASI-RNP potential can be caused by the more realistic treatment of "random" protein-RNA interactions. None of the rigid-body docking methods analyzed here is capable of predicting the structures of complexes that involve large conformational changes. Our potentials recognized near native structures only for these complexes, whose components exhibit relatively small structural changes (RMSD < 3 Å) during formation of the protein-RNA complex.

### Availability and requirements

Project name: Statistical potentials for Protein-RNA docking; Project home page: http://iimcb.genesilico.pl/RNP/; Operating system: Linux; Programming language: Python; Other requirements: Biopython and NumPy libraries; Licence: GNU GPL; No restrictions to use by non-academics.

## List of abbreviations

RMSD: Root Mean Square Deviation; RNP: RNA-protein complexes; QUASI-RNP: QUASI-chemical potential for RNA-Protein complexes; DARS-RNP: Decoys As Reference State potential for RNA-Protein complexes.

## Authors' contributions

IT developed statistical potentials, performed testing procedures, drafted the manuscript and prepared the figures. JMB conceived of the project and edited the manuscript. Both authors analyzed and interpreted the data. All authors read and approved the final manuscript.

## Supplementary Material

Additional file 1 **Additional_figures_and_table.pdf**. Files and tables with additional data illustrating the details of implementation, ordered according to their appearance in the text. Click here for file

Additional file 2 **Energy_DARS.pdf**. Energy for each distance, angle, and site bin, for each pair wise interaction in the DARS-RNP potential. Click here for file

Additional file 3 **Energy_QUASI.pdf**. Energy for each distance, angle, and site bin, for each pair wise interaction in the QUASI-RNP potential. Click here for file

Additional file 4 **Observed.pdf**. Observed number of contacts in each distance, angle, and site bin, for each pair wise interaction. Click here for file

Additional file 5 **Expected_DARS.pdf**. Expected number of contacts in each distance, angle, and site bin, for each pair wise interaction in the DARS-RNP potential. Click here for file

Additional file 6 **Expected_QUASI.pdf**. Expected number of contacts in each distance, angle, and site bin, for each pair wise interaction in the QUASI-RNP potential. Click here for file
