# Supplementary material for: DARS-RNP and QUASI-RNP: New statistical potentials for protein-RNA docking
Source: BMC Bioinformatics. 2011 Aug 18;12:348. doi: 10.1186/1471-2105-12-348 (PMC3179970; doi:10.1186/1471-2105-12-348)
Supplement: Additional file 1 — Additional_figures_and_table.pdf. Files and tables with additional data illustrating the details of implementation, ordered according to their appearance in the text. [file 1471-2105-12-348-S1.PDF]

Supplementary Figure Legends

Figure S1. An example of a biologically reasonable (and functionally native-like) complex with ligand RMSD around 20 Å. An RNA molecule of the native 1LNG complex is colored dark blue, while the docked RNA in with RMSD of 19.56 Å to the native complex is colored light blue.

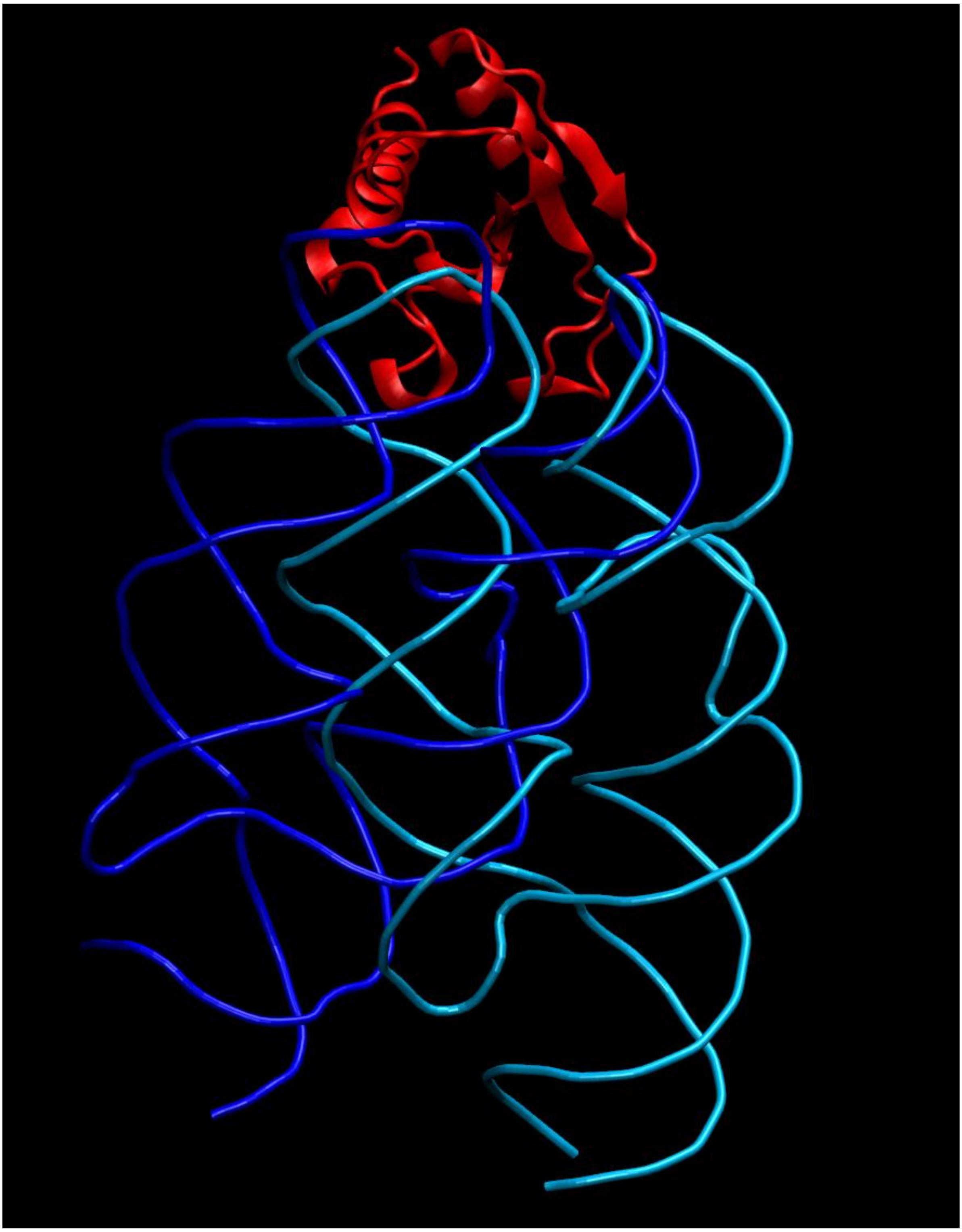

Figure S2. The relationship between the fraction of native ligand-receptor contacts and the ligand RMSD for decoys obtained for the 1LNG complex. Only decoys with RMSD < 20 Å have been considered.

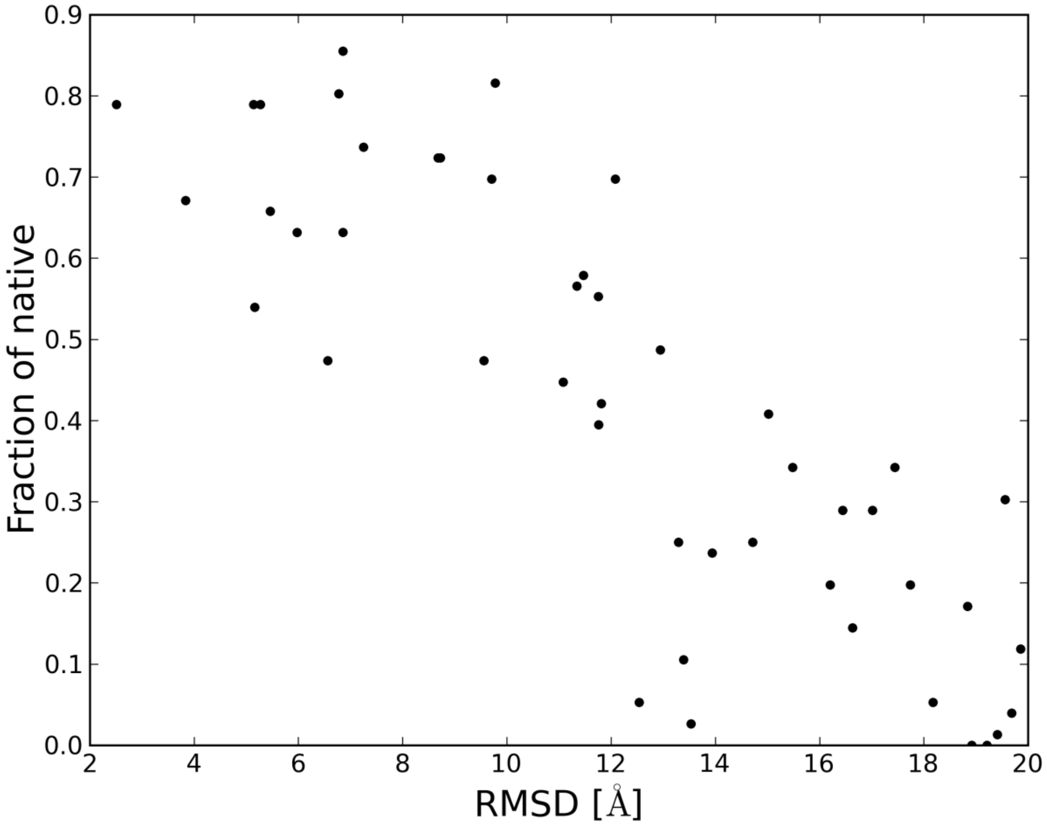

Figure S3. Correlation coefficients for the bound docking test (with GRAMM-generated and Varani decoy sets) for all protein-RNA statistical potentials analyzed in this work.

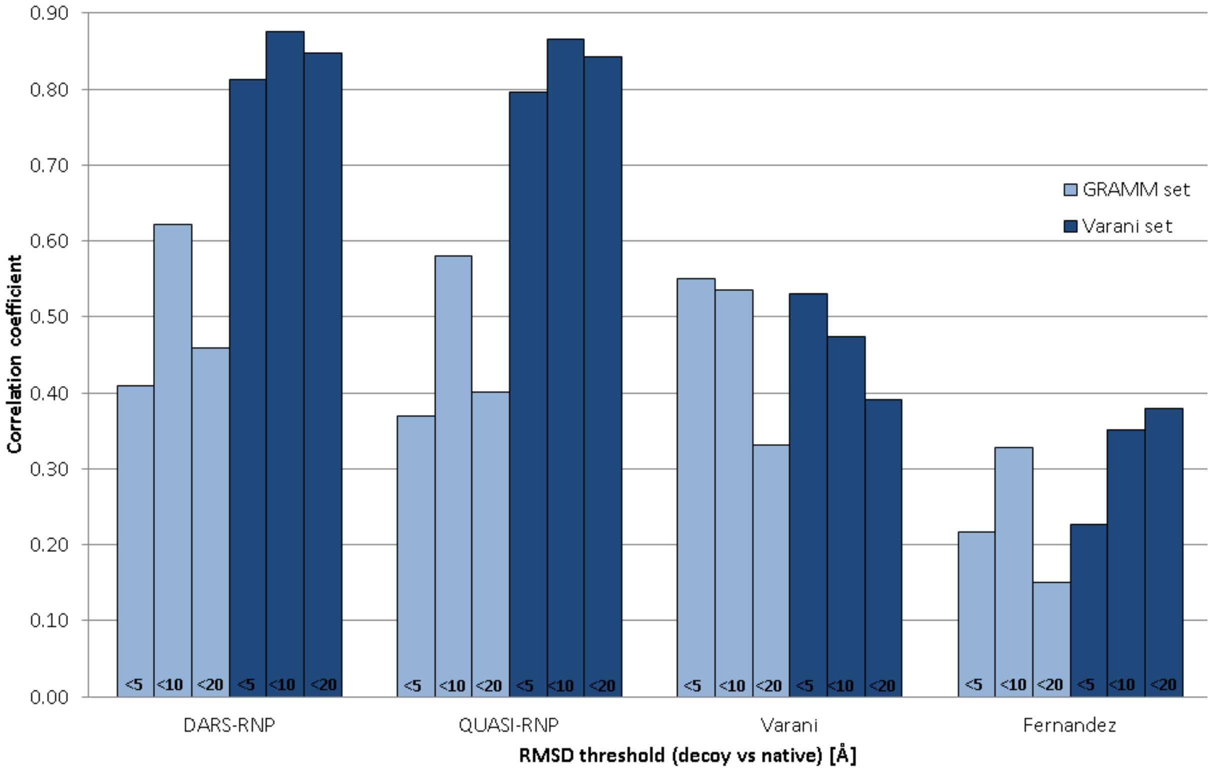

Figure S4. Correlation coefficients for the GRAMM-generated unbound docking test for all protein-RNA statistical potentials analyzed in this work.

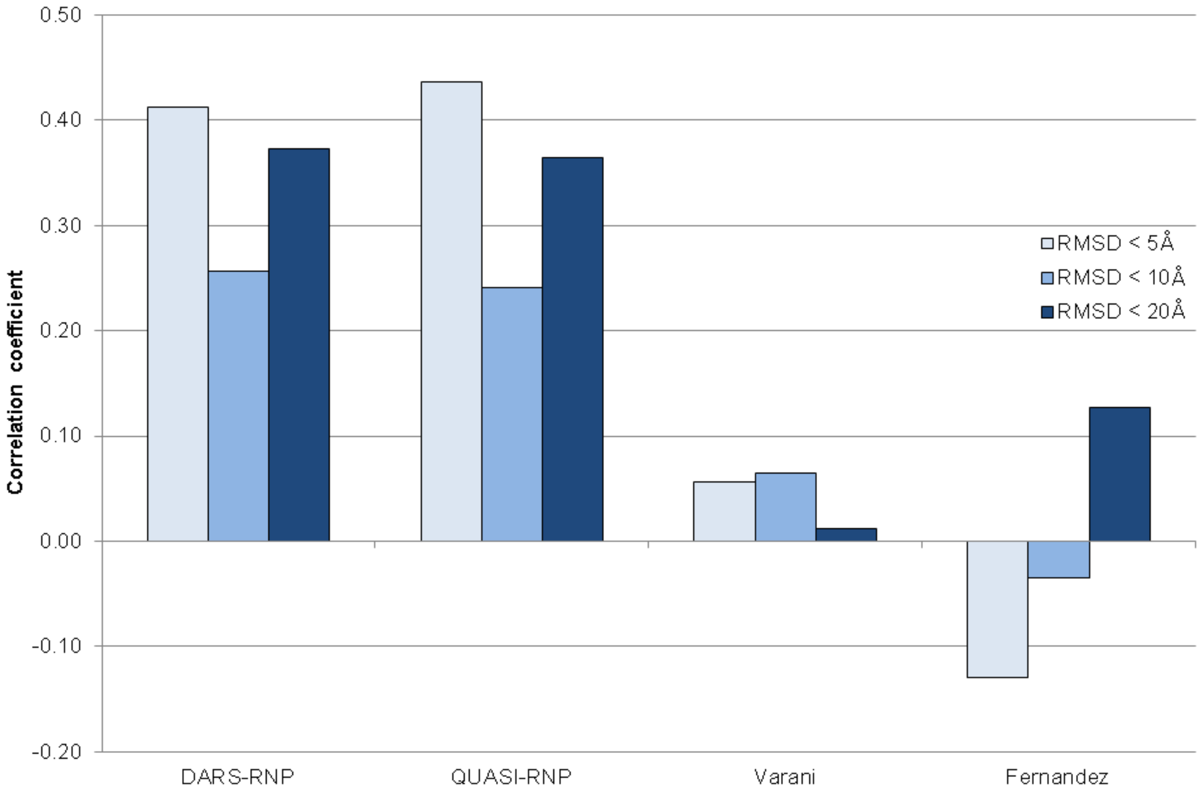

Figure S5. Distance, angle and site distributions for selected pairs of united atoms.

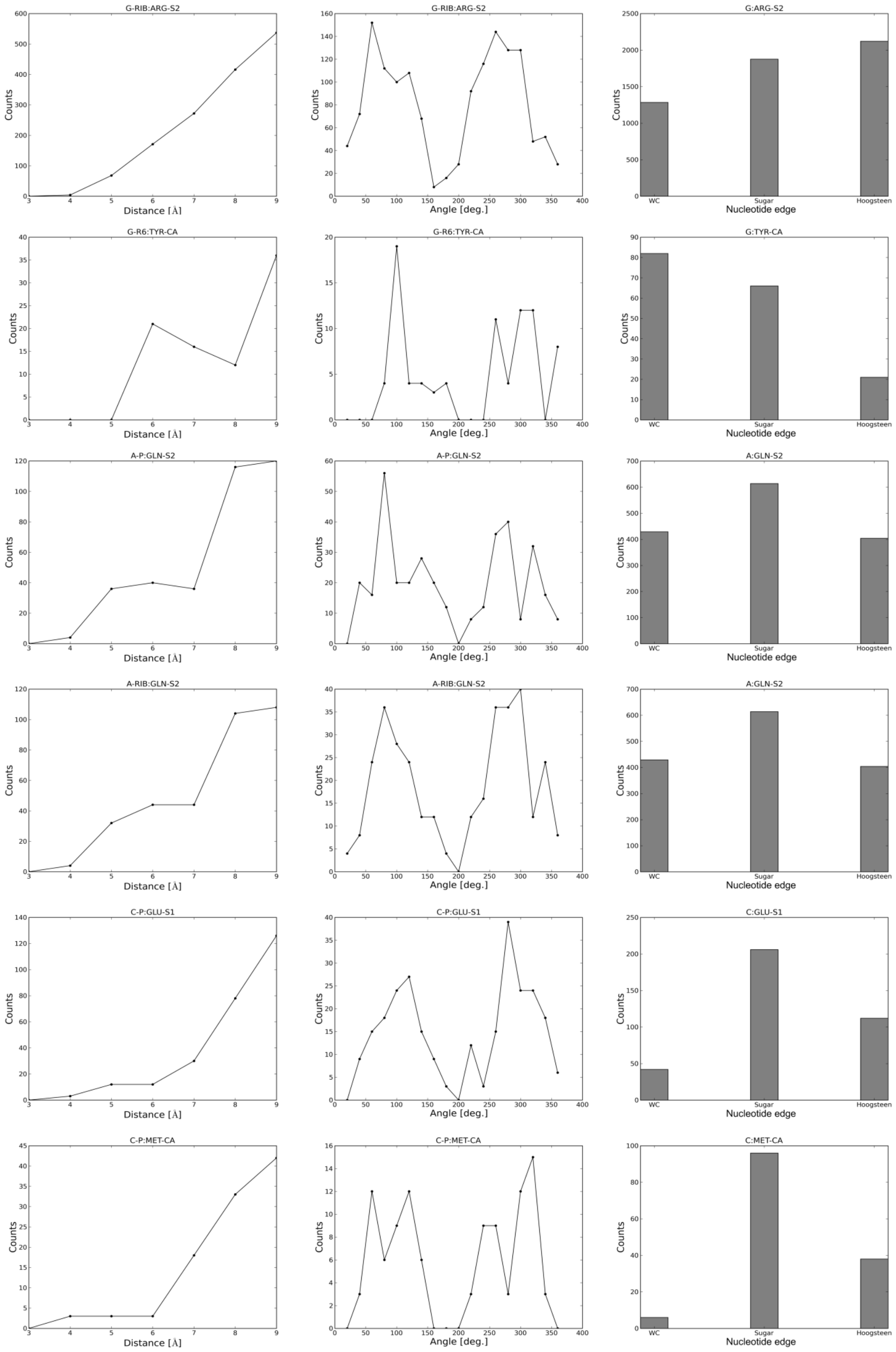

Figure S6. Expected number of selected pairs of united atoms as a function of distance, angle and site of nucleotide for the DARS-RNP potential.

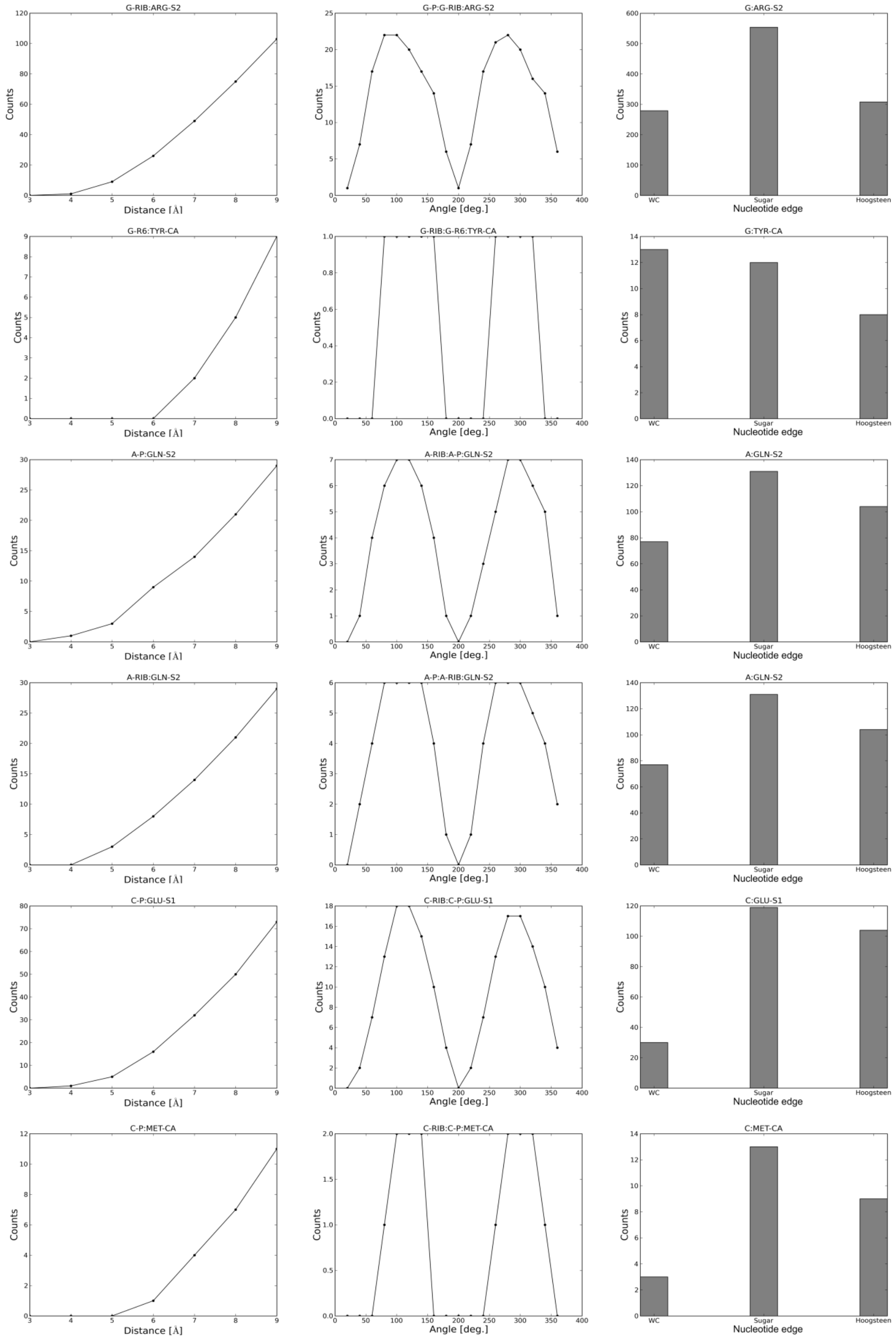

Figure S7. Expected number of selected pairs of united atoms as a function of distance, angle and site of nucleotide for the QUASI-RNP potential.

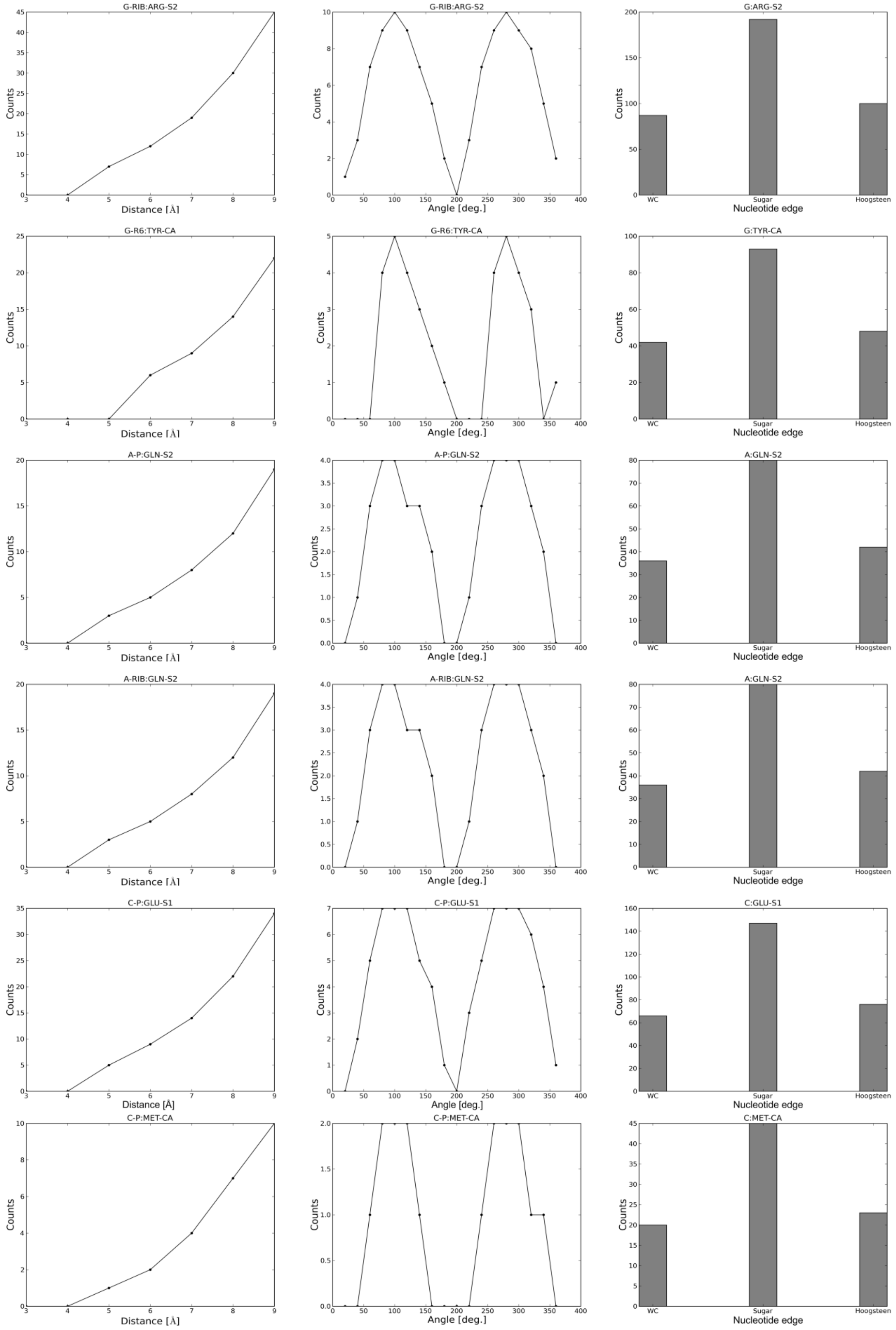

Table S1. Parameters for GRAMM docking used in the rpar.gr GRAMM file. mmode – matching mode; eta – grid step; ro – repulsion; fr – attraction double range; crang – potential range type; ccti – projection; crep – representation; maxm – number of matches to output; ai – angle for rotations

Type of docking set	Complex PDB code	mmode	eta	ro	fr	crang	ccti	crep	maxm	ai
bound	1urn	generic	1.7	10	0	atom_radius	gray	all	10000	10
	1ec6	generic	1.7	10	0	atom_radius	gray	all	10000	10
	1fxl	generic	1.7	10	0	atom_radius	gray	all	10000	10
	1cvj	generic	1.7	10	0	atom_radius	gray	all	10000	10
	1jid	generic	1.7	10	0	atom_radius	gray	all	10000	10
unbound	3bso	generic	1.8	10	0	atom_radius	gray	all	10000	10
	1wpu	generic	1.7	10	0	atom_radius	gray	all	10000	10
	2jea	generic	2.2	20	0	atom_radius	gray	all	10000	10
	1e7k	generic	1.7	10	0	atom_radius	gray	all	10000	10
	2pxv	generic	1.8	10	0	atom_radius	gray	all	10000	10
	1lng	generic	2.3	20	0	atom_radius	gray	all	10000	10
	2r8s	generic	2.8	25	0	atom_radius	gray	all	10000	10
	2rkj	generic	2.9	25	0	atom_radius	gray	all	10000	10
