# Supplementary material for: DARS-RNP and QUASI-RNP: New statistical potentials for protein-RNA docking
Source: BMC Bioinformatics. 2011 Aug 18;12:348. doi: 10.1186/1471-2105-12-348 (PMC3179970; doi:10.1186/1471-2105-12-348)
Supplement: Additional file 2 — Energy_DARS.pdf. Energy for each distance, angle, and site bin, for each pair wise interaction in the DARS-RNP potential. [file 1471-2105-12-348-S2.PDF]

U-P:CYS-S1
EN 3 0
EN 4 0
EN 5 0
EN 6 0
EN 7 -5680
EN 8 -2380
EN 9 -2763
A-RIB:GLN-S2
EN 3 0
EN 4 -4419
EN 5 -5656
EN 6 -4086
EN 7 -2673
EN 8 -3855
EN 9 -3227
QUO-M6:GLU-S2
EN 3 0
EN 4 0
EN 5 -3434
EN 6 0
EN 7 0
EN 8 0
EN 9 0
FHU-MY:ALA-S1
EN 3 0
EN 4 0
EN 5 0
EN 6 0
EN 7 -10760
EN 8 -8528
EN 9 -7186
DA-RIB:ALA-S1
EN 3 0
EN 4 0
EN 5 0
EN 6 0
EN 7 -6734
EN 8 0
EN 9 0
G-P:MET-CA
EN 3 0
EN 4 0
EN 5 -3598
EN 6 -2384
EN 7 -1661
EN 8 -3461
EN 9 -2974
G-R6:GLN-S2
EN 3 0
EN 4 0
EN 5 -6483
EN 6 -4718
EN 7 -4648
EN 8 -3345
EN 9 -2470
A-R5:TYR-CA
EN 3 0

EN 4 0
EN 5 0
EN 6 -10201
EN 7 -6641
EN 8 -5807
EN 9 -5542
G-RIB:GLN-CA
EN 3 0
EN 4 0
EN 5 -6129
EN 6 -6391
EN 7 -2875
EN 8 -4366
EN 9 -3670
M2G-P:GLU-CA
EN 3 0
EN 4 0
EN 5 0
EN 6 0
EN 7 0
EN 8 -9571
EN 9 0
G-P:PHE-S1
EN 3 0
EN 4 0
EN 5 -6672
EN 6 -2426
EN 7 -3342
EN 8 -3797
EN 9 -3585
U31-RIB:PHE-CA
EN 3 0
EN 4 0
EN 5 0
EN 6 0
EN 7 0
EN 8 0
EN 9 -5884
FHU-P:SER-S1
EN 3 0
EN 4 0
EN 5 0
EN 6 0
EN 7 0
EN 8 -7683
EN 9 0
U-Y:GLU-S1
EN 3 0
EN 4 0
EN 5 -3843
EN 6 0
EN 7 1645
EN 8 -28
EN 9 -530
IU-RIB:VAL-S1
EN 3 0
EN 4 0
EN 5 0

EN 6 0
EN 7 0
EN 8 0
EN 9 -7683
C31-MY:LEU-S1
EN 3 0
EN 4 0
EN 5 0
EN 6 -6289
EN 7 0
EN 8 0
EN 9 0
G-RIB:ILE-S1
EN 3 0
EN 4 0
EN 5 -5098
EN 6 -3482
EN 7 -2755
EN 8 -2007
EN 9 -2210
OMC-P:LYS-S1
EN 3 0
EN 4 0
EN 5 0
EN 6 0
EN 7 -5274
EN 8 0
EN 9 0
M2G-P:SER-S1
EN 3 0
EN 4 0
EN 5 0
EN 6 0
EN 7 0
EN 8 0
EN 9 -13127
C-RIB:ILE-S1
EN 3 0
EN 4 0
EN 5 -5358
EN 6 -5443
EN 7 -3765
EN 8 -1884
EN 9 -2853
U-P:MET-CA
EN 3 0
EN 4 0
EN 5 -8829
EN 6 -3857
EN 7 -2121
EN 8 -2937
EN 9 -3325
A-RIB:GLY-CA
EN 3 0
EN 4 -5932
EN 5 -7107
EN 6 -4113
EN 7 -4191

EN 8 -3775
EN 9 -4319
C-Y:HIS-CA
EN 3 0
EN 4 0
EN 5 0
EN 6 -5405
EN 7 -6387
EN 8 -4765
EN 9 -4412
C-RIB:MET-S2
EN 3 0
EN 4 -6078
EN 5 -5840
EN 6 -5882
EN 7 -4914
EN 8 -3190
EN 9 -3569
C-P:GLU-S1
EN 3 0
EN 4 -2209
EN 5 -1819
EN 6 757
EN 7 170
EN 8 -1071
EN 9 -1351
H2U-MY:GLY-CA
EN 3 0
EN 4 0
EN 5 0
EN 6 0
EN 7 -4063
EN 8 0
EN 9 0
C-RIB:LEU-S2
EN 3 0
EN 4 0
EN 5 -4382
EN 6 -5070
EN 7 -4769
EN 8 -3069
EN 9 -2799
A-P:TRP-CA
EN 3 0
EN 4 0
EN 5 -9190
EN 6 -3695
EN 7 -1319
EN 8 -5199
EN 9 -2219
G-R5:TRP-CA
EN 3 0
EN 4 0
EN 5 0
EN 6 -8306
EN 7 -4970
EN 8 -5082
EN 9 -4850

C-RIB:SER-S1
EN 3 0
EN 4 0
EN 5 -5506
EN 6 -4708
EN 7 -3400
EN 8 -3686
EN 9 -3489
A-RIB:ASP-S2
EN 3 0
EN 4 -2497
EN 5 -2905
EN 6 -2757
EN 7 -1267
EN 8 -1661
EN 9 -1489
C31-RIB:GLN-S2
EN 3 0
EN 4 0
EN 5 0
EN 6 0
EN 7 0
EN 8 0
EN 9 -5446
PSU-RIB:ARG-S2
EN 3 0
EN 4 0
EN 5 0
EN 6 0
EN 7 0
EN 8 0
EN 9 -7097
A-P:ASN-S1
EN 3 0
EN 4 -6336
EN 5 -6261
EN 6 -1951
EN 7 -3504
EN 8 -3658
EN 9 -3855
IU-MY:LEU-CA
EN 3 0
EN 4 0
EN 5 0
EN 6 0
EN 7 0
EN 8 -7498
EN 9 -6536
U-P:ALA-S1
EN 3 0
EN 4 -5516
EN 5 -6334
EN 6 -2764
EN 7 -3835
EN 8 -3344
EN 9 -3602
A-RIB:THR-CA
EN 3 0

EN 4 -8772
EN 5 -5491
EN 6 -5485
EN 7 -5952
EN 8 -3562
EN 9 -3300
U-RIB:CYS-S1
EN 3 0
EN 4 0
EN 5 0
EN 6 -6507
EN 7 -2966
EN 8 -4127
EN 9 -2780
U-RIB:HIS-S2
EN 3 0
EN 4 -7472
EN 5 -6962
EN 6 -6098
EN 7 -3869
EN 8 -3769
EN 9 -3284
C-RIB:GLU-S2
EN 3 0
EN 4 0
EN 5 -2699
EN 6 -936
EN 7 698
EN 8 -246
EN 9 -788
C-P:GLU-CA
EN 3 0
EN 4 0
EN 5 -4667
EN 6 1167
EN 7 2314
EN 8 -500
EN 9 -1745
H2U-RIB:PHE-S2
EN 3 0
EN 4 0
EN 5 0
EN 6 -2722
EN 7 0
EN 8 0
EN 9 0
G-R5:ASP-S2
EN 3 0
EN 4 0
EN 5 -411
EN 6 -1141
EN 7 -3681
EN 8 -2409
EN 9 -2361
G-P:HIS-S2
EN 3 0
EN 4 0
EN 5 -5316

EN 6 -5311
EN 7 -3629
EN 8 -3155
EN 9 -3424
U-P:GLU-S1
EN 3 0
EN 4 0
EN 5 -2239
EN 6 -328
EN 7 -47
EN 8 -1437
EN 9 -591
C-RIB:ASP-CA
EN 3 0
EN 4 0
EN 5 -5606
EN 6 -3779
EN 7 -2696
EN 8 -2369
EN 9 -1695
U-RIB:ALA-CA
EN 3 0
EN 4 0
EN 5 -5106
EN 6 -3385
EN 7 -4319
EN 8 -3231
EN 9 -3936
C-P:LYS-S2
EN 3 0
EN 4 -6069
EN 5 -6750
EN 6 -2787
EN 7 -3472
EN 8 -3234
EN 9 -3249
C-RIB:PRO-S1
EN 3 0
EN 4 0
EN 5 -5249
EN 6 -4410
EN 7 -3297
EN 8 -3445
EN 9 -2946
QUO-M5:PHE-CA
EN 3 0
EN 4 0
EN 5 0
EN 6 0
EN 7 0
EN 8 0
EN 9 -3434
C31-RIB:PHE-CA
EN 3 0
EN 4 0
EN 5 0
EN 6 0
EN 7 0

EN 8 -8102
EN 9 0
C-P:GLN-S1
EN 3 0
EN 4 0
EN 5 -6743
EN 6 -3105
EN 7 -3845
EN 8 -4469
EN 9 -3885
U-RIB:PHE-CA
EN 3 0
EN 4 0
EN 5 -8934
EN 6 -4624
EN 7 -4810
EN 8 -4253
EN 9 -4262
QUO-RIB:ASN-S2
EN 3 0
EN 4 0
EN 5 -3434
EN 6 0
EN 7 0
EN 8 0
EN 9 0
G-P:VAL-CA
EN 3 0
EN 4 0
EN 5 0
EN 6 -2880
EN 7 -2168
EN 8 -2387
EN 9 -1738
U31-P:ASP-S2
EN 3 0
EN 4 0
EN 5 0
EN 6 -5584
EN 7 0
EN 8 -4667
EN 9 -3695
FHU-RIB:THR-S1
EN 3 0
EN 4 0
EN 5 0
EN 6 0
EN 7 -9042
EN 8 -7278
EN 9 -7012
U-P:GLU-CA
EN 3 0
EN 4 0
EN 5 -4733
EN 6 176
EN 7 -812
EN 8 -1050
EN 9 -1214

FMU-MY:GLU-S2
EN 3 0
EN 4 0
EN 5 -11410
EN 6 0
EN 7 0
EN 8 0
EN 9 0
A-R5:CYS-S1
EN 3 0
EN 4 0
EN 5 -13680
EN 6 -8168
EN 7 -7397
EN 8 -5970
EN 9 -1721
G-RIB:ARG-S2
EN 3 0
EN 4 -1934
EN 5 -4878
EN 6 -4618
EN 7 -4227
EN 8 -4235
EN 9 -4072
A-RIB:GLN-S1
EN 3 0
EN 4 0
EN 5 0
EN 6 -5797
EN 7 -3760
EN 8 -2674
EN 9 -3906
M2G-P:GLU-S2
EN 3 0
EN 4 0
EN 5 0
EN 6 0
EN 7 -9456
EN 8 0
EN 9 0
QUO-RIB:LEU-S1
EN 3 0
EN 4 0
EN 5 0
EN 6 0
EN 7 0
EN 8 -16110
EN 9 0
C-P:TRP-S1
EN 3 0
EN 4 0
EN 5 0
EN 6 -2039
EN 7 -4976
EN 8 -4853
EN 9 -1539
G-RIB:LEU-CA
EN 3 0

EN 4 0
EN 5 -4434
EN 6 -5358
EN 7 -3956
EN 8 -3248
EN 9 -3391
G-P:TRP-CA
EN 3 0
EN 4 0
EN 5 -8528
EN 6 -5099
EN 7 -2430
EN 8 -4599
EN 9 -2901
A-R6:HIS-S1
EN 3 0
EN 4 -9819
EN 5 -7589
EN 6 -5646
EN 7 -4629
EN 8 -4996
EN 9 -3884
U-Y:ASP-S1
EN 3 0
EN 4 0
EN 5 -2461
EN 6 662
EN 7 -775
EN 8 -1358
EN 9 -1711
A-P:THR-CA
EN 3 0
EN 4 0
EN 5 -7249
EN 6 -5136
EN 7 -3633
EN 8 -4115
EN 9 -4250
G-R6:TYR-S2
EN 3 0
EN 4 -11741
EN 5 -7987
EN 6 -6672
EN 7 -1456
EN 8 -4228
EN 9 -4739
U-RIB:LEU-S2
EN 3 0
EN 4 0
EN 5 -8093
EN 6 -5252
EN 7 -4356
EN 8 -1952
EN 9 -2538
C31-RIB:PHE-S1
EN 3 0
EN 4 0
EN 5 0

EN 6 -8452
EN 7 -6166
EN 8 0
EN 9 0
A-RIB:ASN-S2
EN 3 0
EN 4 0
EN 5 -6639
EN 6 -4412
EN 7 -2677
EN 8 -3007
EN 9 -4397
U31-P:ARG-S2
EN 3 0
EN 4 0
EN 5 0
EN 6 0
EN 7 0
EN 8 -4871
EN 9 0
U-RIB:GLN-S2
EN 3 0
EN 4 -4189
EN 5 -5607
EN 6 -3340
EN 7 -3261
EN 8 -3057
EN 9 -3072
U-RIB:TYR-CA
EN 3 0
EN 4 0
EN 5 -7012
EN 6 -3002
EN 7 -4472
EN 8 -4404
EN 9 -4034
DA-M6:SER-CA
EN 3 0
EN 4 0
EN 5 -7739
EN 6 0
EN 7 0
EN 8 0
EN 9 -4587
FHU-P:LEU-CA
EN 3 0
EN 4 0
EN 5 0
EN 6 -9571
EN 7 0
EN 8 -8815
EN 9 -7076
A-RIB:SER-S1
EN 3 0
EN 4 0
EN 5 -5029
EN 6 -4814
EN 7 -3085

EN 8 -3611
EN 9 -3802
C-Y:GLU-S1
EN 3 0
EN 4 0
EN 5 -3697
EN 6 1318
EN 7 -1342
EN 8 -380
EN 9 -1083
U-Y:TRP-CA
EN 3 0
EN 4 0
EN 5 0
EN 6 -6363
EN 7 -6867
EN 8 -1648
EN 9 -3200
A-R6:ARG-S1
EN 3 0
EN 4 0
EN 5 -6387
EN 6 -5465
EN 7 -2920
EN 8 -2861
EN 9 -4224
U-P:PHE-S2
EN 3 0
EN 4 0
EN 5 0
EN 6 -6240
EN 7 -2291
EN 8 -3852
EN 9 -4056
G-RIB:ASP-S1
EN 3 0
EN 4 0
EN 5 -4340
EN 6 -2542
EN 7 -987
EN 8 -686
EN 9 -2553
C-Y:ALA-CA
EN 3 0
EN 4 0
EN 5 -5701
EN 6 -5055
EN 7 -4014
EN 8 -3332
EN 9 -3258
A-R5:SER-S1
EN 3 0
EN 4 0
EN 5 -3577
EN 6 -4613
EN 7 -4649
EN 8 -3520
EN 9 -4043

G-RIB:GLN-S1
EN 3 0
EN 4 0
EN 5 -6715
EN 6 -5382
EN 7 -4004
EN 8 -3051
EN 9 -2933
G-R5:ARG-CA
EN 3 0
EN 4 0
EN 5 -6212
EN 6 -5986
EN 7 -3416
EN 8 -3743
EN 9 -4359
C31-P:ASP-S1
EN 3 0
EN 4 0
EN 5 0
EN 6 -6849
EN 7 -6258
EN 8 0
EN 9 -3567
C-P:LEU-CA
EN 3 0
EN 4 0
EN 5 -5966
EN 6 -1257
EN 7 -3389
EN 8 -3490
EN 9 -2768
A-RIB:LYS-S2
EN 3 0
EN 4 0
EN 5 -4004
EN 6 -3698
EN 7 -4409
EN 8 -3709
EN 9 -3724
C31-P:ALA-CA
EN 3 0
EN 4 0
EN 5 0
EN 6 0
EN 7 0
EN 8 -5514
EN 9 0
C-RIB:PRO-CA
EN 3 0
EN 4 -7278
EN 5 -5021
EN 6 -4791
EN 7 -4229
EN 8 -2891
EN 9 -2699
C-Y:ILE-S1
EN 3 0

EN 4 0
EN 5 -5323
EN 6 -3791
EN 7 -3919
EN 8 -3751
EN 9 -3658
U-Y:ARG-CA
EN 3 0
EN 4 -11174
EN 5 -5139
EN 6 -3840
EN 7 -5001
EN 8 -3587
EN 9 -4130
C-Y:SER-S1
EN 3 0
EN 4 0
EN 5 -5607
EN 6 -5191
EN 7 -3429
EN 8 -3682
EN 9 -3820
C31-MY:PHE-CA
EN 3 0
EN 4 0
EN 5 0
EN 6 0
EN 7 -7141
EN 8 0
EN 9 0
U31-MY:PHE-S1
EN 3 0
EN 4 0
EN 5 0
EN 6 0
EN 7 -6484
EN 8 0
EN 9 0
C-Y:TYR-CA
EN 3 0
EN 4 0
EN 5 -8402
EN 6 -9118
EN 7 -5275
EN 8 -4222
EN 9 -4234
U-P:LYS-CA
EN 3 0
EN 4 0
EN 5 -5699
EN 6 -4055
EN 7 -4021
EN 8 -4162
EN 9 -3954
U-Y:ILE-S1
EN 3 0
EN 4 0
EN 5 -6648

EN 6 -4573
EN 7 -2708
EN 8 -2880
EN 9 -2985
C-P:ILE-CA
EN 3 0
EN 4 0
EN 5 0
EN 6 -2875
EN 7 -3578
EN 8 -3657
EN 9 -3348
U-P:GLU-S2
EN 3 0
EN 4 0
EN 5 0
EN 6 255
EN 7 -1666
EN 8 -819
EN 9 -1529
A-R6:THR-S1
EN 3 0
EN 4 -4717
EN 5 -5004
EN 6 -4866
EN 7 -2926
EN 8 -2517
EN 9 -3824
U-P:MET-S2
EN 3 0
EN 4 0
EN 5 0
EN 6 -4502
EN 7 -4339
EN 8 -4561
EN 9 -2892
DA-M5:TYR-S1
EN 3 0
EN 4 0
EN 5 0
EN 6 0
EN 7 0
EN 8 -8903
EN 9 0
C-P:GLN-S2
EN 3 0
EN 4 0
EN 5 -5980
EN 6 -4758
EN 7 -2707
EN 8 -3910
EN 9 -3345
U-RIB:GLU-S1
EN 3 0
EN 4 0
EN 5 -3247
EN 6 -1276
EN 7 -450

EN 8 1480
EN 9 1098
U-P:HIS-S1
EN 3 0
EN 4 0
EN 5 0
EN 6 -4878
EN 7 -3474
EN 8 -4868
EN 9 -4376
QUO-RIB:PHE-S2
EN 3 0
EN 4 0
EN 5 0
EN 6 -3434
EN 7 0
EN 8 0
EN 9 -3434
C31-P:ALA-S1
EN 3 0
EN 4 0
EN 5 0
EN 6 0
EN 7 0
EN 8 -4998
EN 9 0
A-P:CYS-CA
EN 3 0
EN 4 0
EN 5 -8102
EN 6 -4961
EN 7 -3058
EN 8 -1432
EN 9 -4998
QUO-M6:GLN-S1
EN 3 0
EN 4 0
EN 5 0
EN 6 0
EN 7 0
EN 8 0
EN 9 -15398
DA-M6:TYR-S1
EN 3 0
EN 4 0
EN 5 0
EN 6 0
EN 7 -10023
EN 8 0
EN 9 0
C-Y:ASP-S1
EN 3 0
EN 4 0
EN 5 -5801
EN 6 -2868
EN 7 -3452
EN 8 -2561
EN 9 -2535

A-P:PRO-S1
EN 3 0
EN 4 0
EN 5 -5388
EN 6 -3579
EN 7 -2208
EN 8 -3307
EN 9 -3998
U-Y:GLN-CA
EN 3 0
EN 4 0
EN 5 -6553
EN 6 -2354
EN 7 -2694
EN 8 -3479
EN 9 -3469
U-Y:MET-S2
EN 3 0
EN 4 0
EN 5 -5281
EN 6 -4432
EN 7 -4732
EN 8 -1795
EN 9 -4289
C-P:THR-S1
EN 3 0
EN 4 0
EN 5 -6247
EN 6 -3278
EN 7 -3032
EN 8 -3920
EN 9 -3663
IU-P:SER-CA
EN 3 0
EN 4 0
EN 5 0
EN 6 0
EN 7 0
EN 8 0
EN 9 -7854
C-RIB:HIS-S1
EN 3 0
EN 4 -8414
EN 5 -6166
EN 6 -5813
EN 7 -3192
EN 8 -4294
EN 9 -3982
A-R6:TYR-CA
EN 3 0
EN 4 0
EN 5 -7549
EN 6 -7452
EN 7 -5524
EN 8 -5445
EN 9 -5002
U-Y:THR-CA
EN 3 0

EN 4 0
EN 5 -6479
EN 6 -5780
EN 7 -3227
EN 8 -749
EN 9 -2626
C-P:VAL-S1
EN 3 0
EN 4 0
EN 5 -2980
EN 6 -4950
EN 7 -2871
EN 8 -3855
EN 9 -4102
G-RIB:TRP-S1
EN 3 0
EN 4 -8528
EN 5 -6320
EN 6 -4048
EN 7 -4074
EN 8 -5525
EN 9 -1954
U34-P:TYR-CA
EN 3 0
EN 4 0
EN 5 0
EN 6 0
EN 7 0
EN 8 0
EN 9 -10489
U-P:SER-S1
EN 3 0
EN 4 -6352
EN 5 -5548
EN 6 -2528
EN 7 -4164
EN 8 -3488
EN 9 -3115
A-P:TYR-S1
EN 3 0
EN 4 0
EN 5 -7934
EN 6 -4434
EN 7 -5277
EN 8 -5502
EN 9 -4425
G-R5:LYS-CA
EN 3 0
EN 4 -10023
EN 5 -4276
EN 6 -4002
EN 7 -3664
EN 8 -3505
EN 9 -4114
A-RIB:PRO-CA
EN 3 0
EN 4 0
EN 5 -5302

EN 6 -4475
EN 7 -4243
EN 8 -2815
EN 9 -3477
U-RIB:LEU-S1
EN 3 0
EN 4 0
EN 5 -7758
EN 6 -5164
EN 7 -3746
EN 8 -3166
EN 9 -2280
FHU-RIB:SER-CA
EN 3 0
EN 4 0
EN 5 0
EN 6 0
EN 7 0
EN 8 -10405
EN 9 0
GTP-RIB:ARG-CA
EN 3 0
EN 4 0
EN 5 0
EN 6 0
EN 7 0
EN 8 -6166
EN 9 0
GTP-M5:ASN-S1
EN 3 0
EN 4 0
EN 5 0
EN 6 0
EN 7 0
EN 8 0
EN 9 -5074
A-RIB:TRP-S2
EN 3 0
EN 4 0
EN 5 -8378
EN 6 -5424
EN 7 -6000
EN 8 -2217
EN 9 -4493
G-R6:PRO-S1
EN 3 0
EN 4 0
EN 5 -5276
EN 6 -4778
EN 7 -2907
EN 8 -2621
EN 9 -1995
A-R6:CYS-CA
EN 3 0
EN 4 0
EN 5 -9456
EN 6 0
EN 7 -3601

EN 8 -3902
EN 9 -2691
H2U-P:PRO-S1
EN 3 0
EN 4 0
EN 5 0
EN 6 0
EN 7 0
EN 8 0
EN 9 -4907
QUO-M5:ASP-CA
EN 3 0
EN 4 0
EN 5 0
EN 6 0
EN 7 0
EN 8 0
EN 9 -17115
FMU-P:ARG-S1
EN 3 0
EN 4 0
EN 5 0
EN 6 0
EN 7 0
EN 8 -9139
EN 9 -11537
QUO-M6:LYS-S2
EN 3 0
EN 4 0
EN 5 0
EN 6 0
EN 7 0
EN 8 0
EN 9 -3434
G-RIB:GLN-S2
EN 3 0
EN 4 -3434
EN 5 -6414
EN 6 -3837
EN 7 -3370
EN 8 -2969
EN 9 -2654
FHU-P:LYS-CA
EN 3 0
EN 4 0
EN 5 -12294
EN 6 -8949
EN 7 0
EN 8 -7739
EN 9 -6196
G-P:CYS-CA
EN 3 0
EN 4 0
EN 5 0
EN 6 0
EN 7 0
EN 8 -2864
EN 9 -2421

G-R6:SER-CA
EN 3 0
EN 4 0
EN 5 -3234
EN 6 -4075
EN 7 -3281
EN 8 -3802
EN 9 -1914
G-RIB:GLU-CA
EN 3 0
EN 4 0
EN 5 -4029
EN 6 776
EN 7 64
EN 8 -479
EN 9 -1052
G-R6:LEU-S1
EN 3 0
EN 4 -9571
EN 5 -4591
EN 6 -5343
EN 7 -3752
EN 8 -3760
EN 9 -2720
A-R6:CYS-S1
EN 3 0
EN 4 0
EN 5 0
EN 6 -10000
EN 7 -4003
EN 8 -4361
EN 9 0
QUO-M5:ARG-CA
EN 3 0
EN 4 0
EN 5 0
EN 6 0
EN 7 0
EN 8 0
EN 9 -3434
DA-M6:GLN-S1
EN 3 0
EN 4 0
EN 5 0
EN 6 0
EN 7 0
EN 8 0
EN 9 -5446
A-R5:VAL-S1
EN 3 0
EN 4 -8168
EN 5 -7753
EN 6 -5671
EN 7 -4486
EN 8 -3482
EN 9 -3456
QUO-M6:LEU-S2
EN 3 0

```
EN 4  0
EN 5  0
EN 6  -3434
EN 7  0
EN 8  0
EN 9  -15398
A-R5:PHE-S1
EN 3  0
EN 4  0
EN 5  -9755
EN 6  -6472
EN 7  -3807
EN 8  -2081
EN 9  -4800
G-R6:PRO-CA
EN 3  0
EN 4  0
EN 5  -4124
EN 6  -5610
EN 7  -2432
EN 8  -3628
EN 9  -2288
C-RIB:PHE-S2
EN 3  0
EN 4  0
EN 5  -6911
EN 6  -5309
EN 7  -4691
EN 8  -2765
EN 9  -1663
U-Y:LEU-S1
EN 3  0
EN 4  0
EN 5  0
EN 6  -4994
EN 7  -5675
EN 8  -3980
EN 9  -4059
C-P:PRO-S1
EN 3  0
EN 4  -3024
EN 5  -5713
EN 6  -3652
EN 7  -2563
EN 8  -3291
EN 9  -2977
U-Y:VAL-CA
EN 3  0
EN 4  0
EN 5  0
EN 6  -3686
EN 7  -2253
EN 8  -3627
EN 9  -2819
A-R5:MET-S1
EN 3  0
EN 4  -12294
EN 5  -6970
```

EN 6 -5975
EN 7 -4971
EN 8 -5275
EN 9 -3053
U31-RIB:ASP-S2
EN 3 0
EN 4 0
EN 5 0
EN 6 -9571
EN 7 -5093
EN 8 -4167
EN 9 -3041
C-Y:MET-CA
EN 3 0
EN 4 0
EN 5 -7854
EN 6 0
EN 7 -5440
EN 8 -2692
EN 9 -3467
U-Y:ASP-S2
EN 3 0
EN 4 0
EN 5 -894
EN 6 1691
EN 7 -1807
EN 8 -2320
EN 9 -1217
A-R6:LEU-CA
EN 3 0
EN 4 0
EN 5 -5761
EN 6 -5556
EN 7 -5098
EN 8 -5274
EN 9 -3505
G-R6:ARG-S1
EN 3 0
EN 4 -8972
EN 5 -1847
EN 6 -3410
EN 7 -4489
EN 8 -3425
EN 9 -3700
A-R5:LEU-CA
EN 3 0
EN 4 0
EN 5 0
EN 6 -6849
EN 7 -5726
EN 8 -5015
EN 9 -3883
C-Y:ASN-S1
EN 3 0
EN 4 0
EN 5 -3688
EN 6 -3653
EN 7 -3704

EN 8 -3133
EN 9 -3956
C-P:GLN-CA
EN 3 0
EN 4 0
EN 5 -7422
EN 6 -4823
EN 7 -3954
EN 8 -4337
EN 9 -4230
U31-MY:ILE-CA
EN 3 0
EN 4 0
EN 5 0
EN 6 0
EN 7 -8168
EN 8 0
EN 9 0
U-Y:ASN-CA
EN 3 0
EN 4 0
EN 5 -5755
EN 6 -1691
EN 7 -3656
EN 8 -4147
EN 9 -4500
A-R6:GLY-CA
EN 3 0
EN 4 -3420
EN 5 -3975
EN 6 -4468
EN 7 -3675
EN 8 -4023
EN 9 -3752
A-P:HIS-S2
EN 3 0
EN 4 0
EN 5 -6371
EN 6 -5082
EN 7 -4547
EN 8 -4184
EN 9 -3414
U31-P:GLN-S2
EN 3 0
EN 4 0
EN 5 -11671
EN 6 0
EN 7 0
EN 8 0
EN 9 0
C-Y:LYS-S2
EN 3 0
EN 4 -2432
EN 5 -4985
EN 6 -3554
EN 7 -2637
EN 8 -2333
EN 9 -4022

FHU-RIB:THR-CA
EN 3 0
EN 4 0
EN 5 0
EN 6 -12294
EN 7 -13680
EN 8 0
EN 9 0
A-R6:HIS-CA
EN 3 0
EN 4 0
EN 5 -6289
EN 6 -7683
EN 7 -3874
EN 8 -3840
EN 9 -5384
DA-RIB:LYS-S2
EN 3 0
EN 4 0
EN 5 0
EN 6 0
EN 7 0
EN 8 -5380
EN 9 0
A-R6:ASP-CA
EN 3 0
EN 4 -8470
EN 5 -2993
EN 6 -2036
EN 7 -1501
EN 8 -1760
EN 9 -1341
U-Y:TRP-S1
EN 3 0
EN 4 0
EN 5 0
EN 6 -8080
EN 7 -2907
EN 8 -3206
EN 9 -3661
U-P:ASP-CA
EN 3 0
EN 4 0
EN 5 -5237
EN 6 -1926
EN 7 -510
EN 8 -1962
EN 9 -1798
G-R6:LEU-S2
EN 3 0
EN 4 -6929
EN 5 -7719
EN 6 -4009
EN 7 -553
EN 8 -2712
EN 9 -3296
H2U-MY:LEU-S2
EN 3 0

EN 4 0
EN 5 0
EN 6 0
EN 7 -6137
EN 8 0
EN 9 0
U-Y:ASP-CA
EN 3 0
EN 4 0
EN 5 0
EN 6 -841
EN 7 -901
EN 8 -1259
EN 9 -1309
G-P:LYS-CA
EN 3 0
EN 4 0
EN 5 -7242
EN 6 -4560
EN 7 -3372
EN 8 -4441
EN 9 -3463
C-P:ASP-CA
EN 3 0
EN 4 0
EN 5 -3814
EN 6 -827
EN 7 -1095
EN 8 -2616
EN 9 -2499
C-P:ARG-S2
EN 3 0
EN 4 -3198
EN 5 -8354
EN 6 -5135
EN 7 -3572
EN 8 -4018
EN 9 -4083
C-Y:GLN-CA
EN 3 0
EN 4 0
EN 5 0
EN 6 -5975
EN 7 -3023
EN 8 -3027
EN 9 -4305
A-RIB:HIS-CA
EN 3 0
EN 4 0
EN 5 -5705
EN 6 -7393
EN 7 -5839
EN 8 -4800
EN 9 -4327
DA-M6:TYR-CA
EN 3 0
EN 4 0
EN 5 0

EN 6 -9571
EN 7 0
EN 8 -7325
EN 9 0
A-P:PHE-S2
EN 3 0
EN 4 0
EN 5 0
EN 6 -4525
EN 7 -4411
EN 8 -4997
EN 9 -4127
FMU-MY:GLN-CA
EN 3 0
EN 4 0
EN 5 0
EN 6 0
EN 7 -10760
EN 8 0
EN 9 0
C-RIB:ALA-S1
EN 3 0
EN 4 -3058
EN 5 -5849
EN 6 -4797
EN 7 -2559
EN 8 -2350
EN 9 -3186
IU-MY:LYS-S1
EN 3 0
EN 4 0
EN 5 0
EN 6 -9346
EN 7 -7262
EN 8 -7503
EN 9 -6690
QUO-P:LEU-CA
EN 3 0
EN 4 0
EN 5 0
EN 6 0
EN 7 0
EN 8 0
EN 9 -17115
A-RIB:HIS-S2
EN 3 0
EN 4 0
EN 5 -6546
EN 6 -6501
EN 7 -5244
EN 8 -3681
EN 9 -4125
FHU-MY:TYR-CA
EN 3 0
EN 4 0
EN 5 -17115
EN 6 0
EN 7 0

EN 8 0
EN 9 -10245
C-RIB:ARG-S2
EN 3 0
EN 4 -4134
EN 5 -5137
EN 6 -5603
EN 7 -4342
EN 8 -4302
EN 9 -4596
DA-M6:LYS-S1
EN 3 0
EN 4 0
EN 5 0
EN 6 0
EN 7 -7575
EN 8 0
EN 9 0
U-Y:MET-CA
EN 3 0
EN 4 0
EN 5 -9042
EN 6 -6496
EN 7 -5380
EN 8 -2395
EN 9 -3714
A-P:LEU-S1
EN 3 0
EN 4 0
EN 5 -5730
EN 6 -4532
EN 7 -2546
EN 8 -2951
EN 9 -3021
A-RIB:LEU-S1
EN 3 0
EN 4 0
EN 5 -5932
EN 6 -5589
EN 7 -4414
EN 8 -3585
EN 9 -3017
QUO-M6:GLU-CA
EN 3 0
EN 4 0
EN 5 0
EN 6 0
EN 7 0
EN 8 0
EN 9 -3434
G-R5:HIS-S2
EN 3 0
EN 4 -10405
EN 5 0
EN 6 -6243
EN 7 -4490
EN 8 -4771
EN 9 -4425

G-P:GLU-CA
EN 3 0
EN 4 0
EN 5 199
EN 6 688
EN 7 2699
EN 8 -468
EN 9 -1327
C-Y:PHE-S2
EN 3 0
EN 4 -14011
EN 5 -6553
EN 6 -4390
EN 7 -5051
EN 8 -3546
EN 9 -3534
FMU-MY:ASN-CA
EN 3 0
EN 4 0
EN 5 0
EN 6 0
EN 7 0
EN 8 0
EN 9 -10958
FMU-MY:SER-CA
EN 3 0
EN 4 0
EN 5 0
EN 6 0
EN 7 -10405
EN 8 0
EN 9 0
C-RIB:LYS-S2
EN 3 0
EN 4 0
EN 5 -2759
EN 6 -4173
EN 7 -3751
EN 8 -3402
EN 9 -3388
U34-RIB:GLY-CA
EN 3 0
EN 4 0
EN 5 0
EN 6 0
EN 7 0
EN 8 -8528
EN 9 0
A-RIB:ARG-CA
EN 3 0
EN 4 0
EN 5 -6250
EN 6 -5977
EN 7 -4497
EN 8 -4275
EN 9 -4782
GTP-RIB:THR-S1
EN 3 0

EN 4 0
EN 5 0
EN 6 0
EN 7 0
EN 8 0
EN 9 -5680
G-R5:VAL-CA
EN 3 0
EN 4 0
EN 5 0
EN 6 -5117
EN 7 -3330
EN 8 -1846
EN 9 -2503
A-RIB:MET-S2
EN 3 0
EN 4 0
EN 5 -6608
EN 6 -3781
EN 7 -3391
EN 8 -3255
EN 9 -2728
G-R5:MET-CA
EN 3 0
EN 4 0
EN 5 -8185
EN 6 -4408
EN 7 -4562
EN 8 -4593
EN 9 -4167
H2U-P:THR-S1
EN 3 0
EN 4 0
EN 5 0
EN 6 0
EN 7 0
EN 8 0
EN 9 -9042
U-Y:SER-CA
EN 3 0
EN 4 0
EN 5 -3124
EN 6 -4067
EN 7 -3516
EN 8 -2810
EN 9 -3460
G-R6:LYS-S1
EN 3 0
EN 4 -7385
EN 5 -5789
EN 6 -1596
EN 7 -3092
EN 8 -3144
EN 9 -2646
C-RIB:TRP-S1
EN 3 0
EN 4 0
EN 5 -7397

EN 6 -4054
EN 7 -4233
EN 8 -4367
EN 9 -2602
U-RIB:GLN-CA
EN 3 0
EN 4 0
EN 5 0
EN 6 -6734
EN 7 -4137
EN 8 -1509
EN 9 -3326
5BU-P:ILE-CA
EN 3 0
EN 4 0
EN 5 0
EN 6 -2722
EN 7 0
EN 8 -12294
EN 9 0
C31-MY:GLU-S2
EN 3 0
EN 4 0
EN 5 0
EN 6 0
EN 7 -4587
EN 8 0
EN 9 -3774
QUO-M5:ASN-CA
EN 3 0
EN 4 0
EN 5 0
EN 6 0
EN 7 0
EN 8 -3434
EN 9 0
IU-MY:THR-CA
EN 3 0
EN 4 0
EN 5 0
EN 6 0
EN 7 -9190
EN 8 -9206
EN 9 0
G-P:ALA-CA
EN 3 0
EN 4 -2687
EN 5 -4159
EN 6 -3319
EN 7 -1950
EN 8 -2648
EN 9 -2459
FHU-RIB:ALA-S1
EN 3 0
EN 4 0
EN 5 0
EN 6 -12294
EN 7 0

EN 8 -10489
EN 9 -9241
G-RIB:ASN-S1
EN 3 0
EN 4 0
EN 5 -4974
EN 6 -5168
EN 7 -4351
EN 8 -3541
EN 9 -3090
FHU-P:ALA-CA
EN 3 0
EN 4 0
EN 5 0
EN 6 0
EN 7 -8378
EN 8 0
EN 9 0
G-P:ASN-CA
EN 3 0
EN 4 -6484
EN 5 -7513
EN 6 -4973
EN 7 -3535
EN 8 -3276
EN 9 -3205
A-R5:PRO-S1
EN 3 0
EN 4 -7373
EN 5 -6508
EN 6 -3604
EN 7 -3991
EN 8 -3593
EN 9 -3776
C31-P:ASN-S2
EN 3 0
EN 4 0
EN 5 0
EN 6 0
EN 7 0
EN 8 0
EN 9 -4276
U34-P:PHE-S2
EN 3 0
EN 4 0
EN 5 0
EN 6 0
EN 7 0
EN 8 0
EN 9 -12294
G-P:MET-S1
EN 3 0
EN 4 0
EN 5 -3820
EN 6 -155
EN 7 -3191
EN 8 -3011
EN 9 -1790

C-Y:ARG-CA
EN 3 0
EN 4 0
EN 5 0
EN 6 -5887
EN 7 -5576
EN 8 -4952
EN 9 -3260
FHU-MY:ASP-S2
EN 3 0
EN 4 0
EN 5 -12477
EN 6 0
EN 7 0
EN 8 0
EN 9 -6484
C31-RIB:SER-CA
EN 3 0
EN 4 0
EN 5 0
EN 6 0
EN 7 -7629
EN 8 0
EN 9 0
U34-MY:TYR-S2
EN 3 0
EN 4 0
EN 5 0
EN 6 0
EN 7 -11410
EN 8 0
EN 9 0
FMU-MY:GLU-S1
EN 3 0
EN 4 0
EN 5 0
EN 6 0
EN 7 -9819
EN 8 0
EN 9 0
IU-MY:PRO-S1
EN 3 0
EN 4 0
EN 5 0
EN 6 -12123
EN 7 -10351
EN 8 0
EN 9 0
U31-MY:GLU-S2
EN 3 0
EN 4 0
EN 5 0
EN 6 0
EN 7 -4375
EN 8 -4332
EN 9 0
A-R6:GLN-S2
EN 3 0

EN 4 -4754
EN 5 -5454
EN 6 -4744
EN 7 -4400
EN 8 -3155
EN 9 -3146
FHU-P:THR-CA
EN 3 0
EN 4 0
EN 5 0
EN 6 -10576
EN 7 -10023
EN 8 -7325
EN 9 0
C-RIB:GLY-CA
EN 3 0
EN 4 -4971
EN 5 -5903
EN 6 -4299
EN 7 -3905
EN 8 -3526
EN 9 -3524
G-RIB:TRP-S2
EN 3 0
EN 4 0
EN 5 -6258
EN 6 -5964
EN 7 -4876
EN 8 -3642
EN 9 -2908
U-Y:HIS-S1
EN 3 0
EN 4 -10576
EN 5 -4986
EN 6 -4138
EN 7 -4135
EN 8 -4223
EN 9 -3285
C-Y:ASN-S2
EN 3 0
EN 4 0
EN 5 -3718
EN 6 -5494
EN 7 -2349
EN 8 -1192
EN 9 -4399
U-RIB:PRO-S1
EN 3 0
EN 4 0
EN 5 -3831
EN 6 -4059
EN 7 -3189
EN 8 -2376
EN 9 -3497
OMC-RIB:LYS-S2
EN 3 0
EN 4 0
EN 5 0

EN 6 0
EN 7 -4973
EN 8 -4380
EN 9 0
U-Y:LYS-S1
EN 3 0
EN 4 0
EN 5 -5323
EN 6 -2331
EN 7 -3014
EN 8 -2963
EN 9 -3793
G-R6:GLN-CA
EN 3 0
EN 4 0
EN 5 0
EN 6 -2693
EN 7 -1270
EN 8 -2643
EN 9 -3990
C-P:PHE-S1
EN 3 0
EN 4 0
EN 5 0
EN 6 -3954
EN 7 -2566
EN 8 -3469
EN 9 -1970
H2U-RIB:GLU-S1
EN 3 0
EN 4 0
EN 5 0
EN 6 0
EN 7 0
EN 8 -5730
EN 9 0
A-RIB:ILE-S1
EN 3 0
EN 4 0
EN 5 -6005
EN 6 -2244
EN 7 -4133
EN 8 -3098
EN 9 -2518
C31-MY:THR-S1
EN 3 0
EN 4 0
EN 5 0
EN 6 0
EN 7 0
EN 8 0
EN 9 -4141
G-P:ALA-S1
EN 3 0
EN 4 -2769
EN 5 -4450
EN 6 -2955
EN 7 -1591

EN 8 -2492
EN 9 -2138
G-R5:ILE-CA
EN 3 0
EN 4 0
EN 5 0
EN 6 -5394
EN 7 0
EN 8 -3380
EN 9 -2116
A-R6:ALA-S1
EN 3 0
EN 4 -4288
EN 5 -2556
EN 6 -3264
EN 7 1404
EN 8 -2150
EN 9 -2741
FHU-MY:GLN-S2
EN 3 0
EN 4 0
EN 5 0
EN 6 0
EN 7 0
EN 8 0
EN 9 -9456
C-RIB:TRP-CA
EN 3 0
EN 4 0
EN 5 0
EN 6 -4553
EN 7 -5424
EN 8 -4697
EN 9 -1596
G-R6:ASP-S2
EN 3 0
EN 4 0
EN 5 -5662
EN 6 -2900
EN 7 -1585
EN 8 -2753
EN 9 -2605
C-RIB:ASN-CA
EN 3 0
EN 4 0
EN 5 -3337
EN 6 -4891
EN 7 -4229
EN 8 -3558
EN 9 -3564
IU-MY:SER-S1
EN 3 0
EN 4 0
EN 5 -12675
EN 6 0
EN 7 0
EN 8 0
EN 9 -8353

C-RIB:GLU-CA
EN 3 0
EN 4 0
EN 5 -3215
EN 6 -1003
EN 7 -873
EN 8 -1252
EN 9 -1422
U31-RIB:MET-S1
EN 3 0
EN 4 0
EN 5 0
EN 6 -11963
EN 7 0
EN 8 0
EN 9 -4651
U-RIB:HIS-CA
EN 3 0
EN 4 0
EN 5 -8080
EN 6 -5420
EN 7 -5045
EN 8 -3583
EN 9 -3679
FHU-RIB:ALA-CA
EN 3 0
EN 4 0
EN 5 0
EN 6 -13680
EN 7 0
EN 8 0
EN 9 -9346
A-P:PHE-CA
EN 3 0
EN 4 0
EN 5 0
EN 6 -5440
EN 7 -4127
EN 8 -3619
EN 9 -3817
OMC-P:LYS-CA
EN 3 0
EN 4 0
EN 5 0
EN 6 0
EN 7 0
EN 8 0
EN 9 -3890
H2U-RIB:LYS-S2
EN 3 0
EN 4 0
EN 5 0
EN 6 -6889
EN 7 0
EN 8 0
EN 9 0
G-RIB:LYS-S1
EN 3 0

EN 4 0
EN 5 -3844
EN 6 -4072
EN 7 -4226
EN 8 -3305
EN 9 -3285
G-P:THR-S1
EN 3 0
EN 4 -5435
EN 5 -5728
EN 6 -2898
EN 7 -2693
EN 8 -1202
EN 9 -2475
C-P:HIS-S1
EN 3 0
EN 4 0
EN 5 -5158
EN 6 -3756
EN 7 -4052
EN 8 -4830
EN 9 -2593
A-RIB:CYS-S1
EN 3 0
EN 4 0
EN 5 -10095
EN 6 -6417
EN 7 -3611
EN 8 -6101
EN 9 -3282
G-RIB:SER-CA
EN 3 0
EN 4 0
EN 5 -3318
EN 6 -4768
EN 7 -3431
EN 8 -2969
EN 9 -2953
G-RIB:VAL-CA
EN 3 0
EN 4 0
EN 5 -3559
EN 6 -2674
EN 7 -2609
EN 8 -2093
EN 9 -1406
U-P:ARG-S1
EN 3 0
EN 4 0
EN 5 -6537
EN 6 -5375
EN 7 -3918
EN 8 -5101
EN 9 -3728
U-Y:ARG-S2
EN 3 0
EN 4 -10081
EN 5 -5947

EN 6 -6058
EN 7 -4316
EN 8 -2377
EN 9 -3749
C-P:GLY-CA
EN 3 0
EN 4 -5565
EN 5 -6682
EN 6 -2313
EN 7 -3530
EN 8 -4260
EN 9 -3277
G-P:MET-S2
EN 3 0
EN 4 0
EN 5 -4805
EN 6 -3129
EN 7 -2746
EN 8 -2588
EN 9 -662
DA-M6:SER-S1
EN 3 0
EN 4 0
EN 5 -7914
EN 6 0
EN 7 0
EN 8 0
EN 9 0
FMU-RIB:ASN-S1
EN 3 0
EN 4 0
EN 5 0
EN 6 0
EN 7 0
EN 8 0
EN 9 -11671
U-RIB:PHE-S1
EN 3 0
EN 4 0
EN 5 -7934
EN 6 -7596
EN 7 -3958
EN 8 -4556
EN 9 -4446
DA-RIB:ASN-S1
EN 3 0
EN 4 0
EN 5 0
EN 6 -7854
EN 7 0
EN 8 0
EN 9 0
FHU-P:LEU-S2
EN 3 0
EN 4 0
EN 5 0
EN 6 0
EN 7 -7472

EN 8 -7523
EN 9 -5705
A-P:GLN-S1
EN 3 0
EN 4 0
EN 5 -5816
EN 6 -4323
EN 7 -2288
EN 8 -3586
EN 9 -3901
C-RIB:ASN-S2
EN 3 0
EN 4 -3130
EN 5 -5909
EN 6 -2325
EN 7 -3373
EN 8 -3239
EN 9 -3366
C31-P:PHE-S2
EN 3 0
EN 4 0
EN 5 0
EN 6 0
EN 7 0
EN 8 -9755
EN 9 0
G-P:GLU-S2
EN 3 0
EN 4 0
EN 5 344
EN 6 221
EN 7 -802
EN 8 -165
EN 9 -1329
H2U-MY:PRO-CA
EN 3 0
EN 4 0
EN 5 0
EN 6 0
EN 7 0
EN 8 -5253
EN 9 -6289
C31-P:GLN-CA
EN 3 0
EN 4 0
EN 5 0
EN 6 0
EN 7 0
EN 8 -6107
EN 9 0
GTP-RIB:ARG-S1
EN 3 0
EN 4 0
EN 5 0
EN 6 0
EN 7 0
EN 8 -5858
EN 9 0

H2U-RIB:GLU-CA
EN 3 0
EN 4 0
EN 5 0
EN 6 0
EN 7 0
EN 8 0
EN 9 -5112
IU-MY:VAL-S1
EN 3 0
EN 4 0
EN 5 0
EN 6 0
EN 7 0
EN 8 0
EN 9 -9755
U31-MY:GLN-CA
EN 3 0
EN 4 0
EN 5 0
EN 6 0
EN 7 0
EN 8 0
EN 9 -4479
U-Y:HIS-CA
EN 3 0
EN 4 0
EN 5 0
EN 6 -5984
EN 7 -4889
EN 8 -3386
EN 9 -3192
U34-MY:ASN-S1
EN 3 0
EN 4 0
EN 5 0
EN 6 0
EN 7 0
EN 8 -6227
EN 9 -5192
A-RIB:MET-S1
EN 3 0
EN 4 0
EN 5 0
EN 6 -6311
EN 7 -4528
EN 8 -3092
EN 9 -4413
G-RIB:HIS-S1
EN 3 0
EN 4 -7141
EN 5 -6948
EN 6 -5697
EN 7 -3856
EN 8 -4151
EN 9 -2985
G-P:VAL-S1
EN 3 0

EN 4 0
EN 5 -1568
EN 6 -2405
EN 7 -2163
EN 8 -2855
EN 9 -1998
A-P:GLU-S2
EN 3 0
EN 4 0
EN 5 0
EN 6 248
EN 7 -1595
EN 8 -1050
EN 9 -1235
G-RIB:GLY-CA
EN 3 0
EN 4 -4020
EN 5 -6265
EN 6 -4440
EN 7 -3707
EN 8 -3138
EN 9 -3163
A-RIB:PHE-CA
EN 3 0
EN 4 0
EN 5 -5905
EN 6 -5007
EN 7 -3980
EN 8 -5055
EN 9 -3547
A-R5:TRP-S1
EN 3 0
EN 4 0
EN 5 0
EN 6 -4943
EN 7 -2553
EN 8 -2628
EN 9 -5739
IU-MY:LYS-CA
EN 3 0
EN 4 0
EN 5 -15398
EN 6 0
EN 7 -9382
EN 8 -6678
EN 9 -7106
DA-RIB:ASP-CA
EN 3 0
EN 4 0
EN 5 0
EN 6 -7914
EN 7 0
EN 8 0
EN 9 0
C-RIB:GLN-CA
EN 3 0
EN 4 0
EN 5 -4790

EN 6 -5568
EN 7 -5866
EN 8 -4302
EN 9 -3405
U-RIB:MET-S1
EN 3 0
EN 4 0
EN 5 -8521
EN 6 -2343
EN 7 -4573
EN 8 -4853
EN 9 -1704
A-P:PRO-CA
EN 3 0
EN 4 0
EN 5 -3745
EN 6 -2979
EN 7 -3549
EN 8 -4519
EN 9 -3332
G-R6:TRP-CA
EN 3 0
EN 4 0
EN 5 -9139
EN 6 -8502
EN 7 -2832
EN 8 -4070
EN 9 -4356
C-RIB:MET-CA
EN 3 0
EN 4 0
EN 5 -7422
EN 6 -7330
EN 7 -2178
EN 8 -3119
EN 9 -2888
G-R5:SER-S1
EN 3 0
EN 4 0
EN 5 -6274
EN 6 -3483
EN 7 -2874
EN 8 -3133
EN 9 -2888
G-RIB:MET-S2
EN 3 0
EN 4 0
EN 5 -7092
EN 6 -3875
EN 7 -2814
EN 8 -3825
EN 9 -2605
QUO-M6:ARG-S1
EN 3 0
EN 4 0
EN 5 0
EN 6 0
EN 7 -3434

EN 8 0
EN 9 -3434
U-RIB:ARG-S2
EN 3 0
EN 4 0
EN 5 -5646
EN 6 -5722
EN 7 -3969
EN 8 -3720
EN 9 -3870
C-P:ASN-S1
EN 3 0
EN 4 -4063
EN 5 -6412
EN 6 -3448
EN 7 -4601
EN 8 -3885
EN 9 -3448
DA-M5:LEU-S1
EN 3 0
EN 4 0
EN 5 0
EN 6 0
EN 7 0
EN 8 0
EN 9 -4290
C-P:HIS-S2
EN 3 0
EN 4 0
EN 5 -4692
EN 6 -5571
EN 7 -3974
EN 8 -3109
EN 9 -3346
G-RIB:HIS-CA
EN 3 0
EN 4 0
EN 5 -4513
EN 6 -5579
EN 7 -5209
EN 8 -3443
EN 9 -3318
C-RIB:THR-CA
EN 3 0
EN 4 0
EN 5 -5504
EN 6 -5437
EN 7 -5018
EN 8 -3613
EN 9 -3238
FHU-P:TYR-S2
EN 3 0
EN 4 0
EN 5 0
EN 6 0
EN 7 0
EN 8 -11329
EN 9 -8038

FHU-MY:VAL-S1
EN 3 0
EN 4 0
EN 5 0
EN 6 0
EN 7 0
EN 8 -10095
EN 9 -9513
G-P:ILE-S1
EN 3 0
EN 4 0
EN 5 -2807
EN 6 -2267
EN 7 -1803
EN 8 -3069
EN 9 -3249
H2U-RIB:ARG-S1
EN 3 0
EN 4 0
EN 5 0
EN 6 0
EN 7 0
EN 8 0
EN 9 -5656
FHU-P:VAL-CA
EN 3 0
EN 4 0
EN 5 0
EN 6 0
EN 7 0
EN 8 0
EN 9 -8135
C-P:MET-S2
EN 3 0
EN 4 0
EN 5 -3641
EN 6 -3990
EN 7 -4386
EN 8 -4280
EN 9 -2420
FHU-MY:GLY-CA
EN 3 0
EN 4 0
EN 5 0
EN 6 0
EN 7 0
EN 8 0
EN 9 -7629
5BU-MY:PRO-S1
EN 3 0
EN 4 0
EN 5 0
EN 6 0
EN 7 0
EN 8 0
EN 9 -12294
FHU-P:CYS-S1
EN 3 0

EN 4 0
EN 5 0
EN 6 0
EN 7 0
EN 8 0
EN 9 -11671
U34-RIB:ASN-S2
EN 3 0
EN 4 0
EN 5 0
EN 6 -14845
EN 7 0
EN 8 -6050
EN 9 0
U34-P:ARG-S2
EN 3 0
EN 4 0
EN 5 0
EN 6 0
EN 7 -2722
EN 8 0
EN 9 0
G-R5:LEU-S2
EN 3 0
EN 4 0
EN 5 -8723
EN 6 -5568
EN 7 1056
EN 8 -3485
EN 9 -3724
G-RIB:LYS-S2
EN 3 0
EN 4 0
EN 5 -3426
EN 6 -3747
EN 7 -4087
EN 8 -3590
EN 9 -3490
A-R5:ARG-CA
EN 3 0
EN 4 0
EN 5 0
EN 6 -6264
EN 7 -5429
EN 8 -4238
EN 9 -5112
A-R6:VAL-CA
EN 3 0
EN 4 0
EN 5 -4721
EN 6 -5216
EN 7 -5198
EN 8 -3041
EN 9 -2761
H2U-P:GLU-S1
EN 3 0
EN 4 0
EN 5 0

EN 6 0
EN 7 0
EN 8 0
EN 9 -6678
U-P:ILE-CA
EN 3 0
EN 4 0
EN 5 0
EN 6 -994
EN 7 -2605
EN 8 -629
EN 9 -2996
C-P:TYR-S2
EN 3 0
EN 4 0
EN 5 -5884
EN 6 -5827
EN 7 -3979
EN 8 -2354
EN 9 -3491
A-R5:MET-CA
EN 3 0
EN 4 0
EN 5 -9042
EN 6 -6368
EN 7 -6000
EN 8 -3628
EN 9 -5148
U34-P:GLU-CA
EN 3 0
EN 4 0
EN 5 0
EN 6 0
EN 7 0
EN 8 0
EN 9 -6484
C-Y:VAL-CA
EN 3 0
EN 4 0
EN 5 -6227
EN 6 -7485
EN 7 -2918
EN 8 -2476
EN 9 -3563
G-P:ASN-S2
EN 3 0
EN 4 0
EN 5 -5759
EN 6 -3410
EN 7 -3624
EN 8 -3622
EN 9 -3410
FMU-P:PHE-S1
EN 3 0
EN 4 0
EN 5 -2722
EN 6 0
EN 7 0

EN 8 0
EN 9 0
A-P:HIS-CA
EN 3 0
EN 4 0
EN 5 -4154
EN 6 -5116
EN 7 -3182
EN 8 -4818
EN 9 -4207
A-P:ASP-S2
EN 3 0
EN 4 0
EN 5 169
EN 6 -1453
EN 7 -2746
EN 8 -1867
EN 9 -1500
H2U-MY:ARG-CA
EN 3 0
EN 4 0
EN 5 0
EN 6 0
EN 7 0
EN 8 -6227
EN 9 0
U31-MY:ALA-S1
EN 3 0
EN 4 0
EN 5 0
EN 6 0
EN 7 0
EN 8 -5514
EN 9 -4525
G-R5:ASN-CA
EN 3 0
EN 4 0
EN 5 -7984
EN 6 -5470
EN 7 -5196
EN 8 -3962
EN 9 -3629
A-R5:CYS-CA
EN 3 0
EN 4 0
EN 5 0
EN 6 -8102
EN 7 -6540
EN 8 -3127
EN 9 -1222
A-R5:GLN-CA
EN 3 0
EN 4 0
EN 5 -8972
EN 6 -3740
EN 7 -3469
EN 8 -4570
EN 9 -3892

DA-RIB:TYR-S1
EN 3 0
EN 4 0
EN 5 0
EN 6 0
EN 7 0
EN 8 0
EN 9 -5656
C-RIB:ASP-S2
EN 3 0
EN 4 -1737
EN 5 -4864
EN 6 -3221
EN 7 -926
EN 8 -2050
EN 9 -2192
U-P:PRO-S1
EN 3 0
EN 4 0
EN 5 -5301
EN 6 -3786
EN 7 -2799
EN 8 -3892
EN 9 -2787
FMU-MY:MET-S1
EN 3 0
EN 4 0
EN 5 0
EN 6 0
EN 7 0
EN 8 -13127
EN 9 0
5BU-P:THR-CA
EN 3 0
EN 4 0
EN 5 0
EN 6 0
EN 7 0
EN 8 0
EN 9 -7575
U-Y:GLU-CA
EN 3 0
EN 4 0
EN 5 0
EN 6 -1841
EN 7 436
EN 8 2374
EN 9 -1076
U-Y:PRO-S1
EN 3 0
EN 4 -9401
EN 5 -4183
EN 6 -2857
EN 7 -4022
EN 8 -2980
EN 9 -3176
A-R5:HIS-S1
EN 3 0

EN 4 -10245
EN 5 -8688
EN 6 -7638
EN 7 -5661
EN 8 -5229
EN 9 -3705
C31-MY:GLN-S1
EN 3 0
EN 4 0
EN 5 0
EN 6 0
EN 7 0
EN 8 0
EN 9 -5380
C31-RIB:TYR-S1
EN 3 0
EN 4 0
EN 5 0
EN 6 0
EN 7 0
EN 8 0
EN 9 -5514
U-Y:PRO-CA
EN 3 0
EN 4 0
EN 5 -6166
EN 6 -4088
EN 7 -3991
EN 8 -3889
EN 9 -3268
U-RIB:MET-S2
EN 3 0
EN 4 0
EN 5 -5484
EN 6 -3231
EN 7 -4187
EN 8 -2393
EN 9 -3966
H2U-RIB:ASN-S2
EN 3 0
EN 4 0
EN 5 0
EN 6 0
EN 7 0
EN 8 -7854
EN 9 -5680
U-RIB:GLU-CA
EN 3 0
EN 4 -9042
EN 5 -2547
EN 6 -325
EN 7 -1009
EN 8 913
EN 9 -1048
U-RIB:ASN-S1
EN 3 0
EN 4 -6772
EN 5 -4612

EN 6 -5149
EN 7 -4124
EN 8 -3691
EN 9 -3774
G-P:GLN-S2
EN 3 0
EN 4 0
EN 5 -4406
EN 6 -3620
EN 7 -3077
EN 8 -3321
EN 9 -3189
A-R5:TRP-CA
EN 3 0
EN 4 0
EN 5 0
EN 6 -6553
EN 7 -3611
EN 8 -3271
EN 9 -5489
I-RIB:TRP-S1
EN 3 0
EN 4 0
EN 5 0
EN 6 0
EN 7 0
EN 8 0
EN 9 -1717
FHU-MY:ILE-CA
EN 3 0
EN 4 0
EN 5 -18832
EN 6 0
EN 7 0
EN 8 0
EN 9 0
IU-P:LEU-CA
EN 3 0
EN 4 0
EN 5 0
EN 6 0
EN 7 0
EN 8 -6889
EN 9 -6078
DA-M5:VAL-S1
EN 3 0
EN 4 0
EN 5 0
EN 6 0
EN 7 0
EN 8 0
EN 9 -6624
U31-P:ASN-S1
EN 3 0
EN 4 0
EN 5 0
EN 6 -7325
EN 7 0

EN 8 0
EN 9 0
H2U-MY:PHE-CA
EN 3 0
EN 4 0
EN 5 0
EN 6 0
EN 7 -12294
EN 8 0
EN 9 0
G-P:LEU-CA
EN 3 0
EN 4 0
EN 5 -6697
EN 6 -2773
EN 7 -2144
EN 8 -2861
EN 9 -3256
G-R5:ARG-S1
EN 3 0
EN 4 -8528
EN 5 -5426
EN 6 -5336
EN 7 -4014
EN 8 -3735
EN 9 -4093
U-RIB:VAL-CA
EN 3 0
EN 4 0
EN 5 0
EN 6 -3283
EN 7 -4710
EN 8 -1471
EN 9 -1640
U-RIB:ILE-CA
EN 3 0
EN 4 0
EN 5 0
EN 6 0
EN 7 -3534
EN 8 -1508
EN 9 -2838
FMU-RIB:GLN-CA
EN 3 0
EN 4 0
EN 5 0
EN 6 0
EN 7 0
EN 8 -12294
EN 9 0
GTP-M5:THR-CA
EN 3 0
EN 4 0
EN 5 0
EN 6 0
EN 7 0
EN 8 0
EN 9 -4332

DA-M5:SER-S1
EN 3 0
EN 4 0
EN 5 -9139
EN 6 0
EN 7 0
EN 8 0
EN 9 -4717
C-P:ALA-S1
EN 3 0
EN 4 -3623
EN 5 -5088
EN 6 -2720
EN 7 -3680
EN 8 -3720
EN 9 -3183
U-RIB:GLY-CA
EN 3 0
EN 4 -5323
EN 5 -6316
EN 6 -4877
EN 7 -4017
EN 8 -3732
EN 9 -3858
C-Y:TRP-S1
EN 3 0
EN 4 0
EN 5 -9139
EN 6 -8102
EN 7 -4207
EN 8 -3756
EN 9 -3395
C-Y:TRP-CA
EN 3 0
EN 4 0
EN 5 -13228
EN 6 -5607
EN 7 -6676
EN 8 -1318
EN 9 -2229
G-R5:LYS-S1
EN 3 0
EN 4 -8288
EN 5 -7182
EN 6 -3242
EN 7 -3770
EN 8 -2947
EN 9 -3707
H2U-P:ASN-S2
EN 3 0
EN 4 0
EN 5 0
EN 6 -10760
EN 7 -9886
EN 8 0
EN 9 0
A-R6:ASP-S1
EN 3 0

EN 4 -5212
EN 5 -2396
EN 6 -2566
EN 7 867
EN 8 -1684
EN 9 -2024
DA-M5:ASP-S2
EN 3 0
EN 4 0
EN 5 0
EN 6 0
EN 7 0
EN 8 0
EN 9 -3914
A-P:ILE-S1
EN 3 0
EN 4 0
EN 5 -2694
EN 6 -4172
EN 7 -3675
EN 8 -3225
EN 9 -3218
G-R5:VAL-S1
EN 3 0
EN 4 0
EN 5 -5122
EN 6 -3244
EN 7 -1724
EN 8 -1972
EN 9 -2520
FHU-MY:ARG-S1
EN 3 0
EN 4 0
EN 5 0
EN 6 -10857
EN 7 0
EN 8 -8489
EN 9 0
A-RIB:ARG-S2
EN 3 0
EN 4 0
EN 5 -5763
EN 6 -6010
EN 7 -4874
EN 8 -4433
EN 9 -3966
U-P:LYS-S2
EN 3 0
EN 4 -7081
EN 5 -6661
EN 6 -2986
EN 7 -2763
EN 8 -3299
EN 9 -3835
DA-M6:THR-S1
EN 3 0
EN 4 0
EN 5 0

EN 6 0
EN 7 0
EN 8 -5607
EN 9 0
A-P:LYS-CA
EN 3 0
EN 4 0
EN 5 -7645
EN 6 -4043
EN 7 -4380
EN 8 -4150
EN 9 -4117
A-R5:LYS-S1
EN 3 0
EN 4 -7854
EN 5 -6751
EN 6 -3416
EN 7 -1753
EN 8 -3625
EN 9 -4229
DA-M6:LYS-CA
EN 3 0
EN 4 0
EN 5 0
EN 6 0
EN 7 0
EN 8 -5730
EN 9 0
U31-P:GLN-CA
EN 3 0
EN 4 0
EN 5 0
EN 6 0
EN 7 -9042
EN 8 0
EN 9 0
G-P:TYR-S1
EN 3 0
EN 4 0
EN 5 -4248
EN 6 -2999
EN 7 -4266
EN 8 -5047
EN 9 -3287
U31-RIB:GLN-CA
EN 3 0
EN 4 0
EN 5 0
EN 6 0
EN 7 -7629
EN 8 0
EN 9 0
A-R6:GLN-S1
EN 3 0
EN 4 0
EN 5 -5137
EN 6 -5619
EN 7 -3578

EN 8 -3639
EN 9 -3129
IU-RIB:ALA-S1
EN 3 0
EN 4 0
EN 5 0
EN 6 0
EN 7 -8489
EN 8 -8772
EN 9 -8660
U-P:TYR-S2
EN 3 0
EN 4 0
EN 5 0
EN 6 -5575
EN 7 -2904
EN 8 -3231
EN 9 -3187
IU-P:LYS-S1
EN 3 0
EN 4 0
EN 5 0
EN 6 -8102
EN 7 0
EN 8 0
EN 9 -4619
A-RIB:GLU-S2
EN 3 0
EN 4 -4138
EN 5 -2366
EN 6 -1554
EN 7 -1101
EN 8 -137
EN 9 -1035
FMU-MY:ARG-S2
EN 3 0
EN 4 0
EN 5 0
EN 6 0
EN 7 0
EN 8 -7683
EN 9 0
U31-RIB:THR-S1
EN 3 0
EN 4 0
EN 5 0
EN 6 0
EN 7 0
EN 8 0
EN 9 -6385
U-P:GLN-S1
EN 3 0
EN 4 -5491
EN 5 -4242
EN 6 -1786
EN 7 -3795
EN 8 -3255
EN 9 -3143

FMU-P:ILE-CA
EN 3 0
EN 4 0
EN 5 0
EN 6 0
EN 7 0
EN 8 -15398
EN 9 0
G-R5:MET-S1
EN 3 0
EN 4 -11174
EN 5 0
EN 6 -4970
EN 7 -5901
EN 8 -3264
EN 9 -3451
G-R5:THR-S1
EN 3 0
EN 4 -5474
EN 5 -3787
EN 6 -1027
EN 7 -3036
EN 8 -2694
EN 9 -2291
C-P:CYS-CA
EN 3 0
EN 4 0
EN 5 -7796
EN 6 0
EN 7 -2403
EN 8 -3777
EN 9 -3540
C-P:TRP-CA
EN 3 0
EN 4 0
EN 5 0
EN 6 -2934
EN 7 -2476
EN 8 -3673
EN 9 -4014
G-R6:HIS-S2
EN 3 0
EN 4 -10975
EN 5 0
EN 6 -4319
EN 7 -5707
EN 8 -4747
EN 9 -4342
G-P:TRP-S1
EN 3 0
EN 4 0
EN 5 -5755
EN 6 -5337
EN 7 -3679
EN 8 -4088
EN 9 -1939
H2U-RIB:GLY-CA
EN 3 0

EN 4 0
EN 5 0
EN 6 0
EN 7 0
EN 8 -4127
EN 9 0
C-P:LYS-CA
EN 3 0
EN 4 0
EN 5 -6816
EN 6 -5125
EN 7 -3984
EN 8 -4204
EN 9 -3493
H2U-MY:LEU-CA
EN 3 0
EN 4 0
EN 5 0
EN 6 0
EN 7 0
EN 8 0
EN 9 -5074
C-P:ASP-S1
EN 3 0
EN 4 0
EN 5 -3686
EN 6 -423
EN 7 -1168
EN 8 -3014
EN 9 -2672
I-P:TRP-S2
EN 3 0
EN 4 0
EN 5 0
EN 6 0
EN 7 0
EN 8 0
EN 9 -1717
U-RIB:LYS-CA
EN 3 0
EN 4 0
EN 5 -4405
EN 6 -4835
EN 7 -4180
EN 8 -4258
EN 9 -3156
C-Y:THR-S1
EN 3 0
EN 4 0
EN 5 -4973
EN 6 -4446
EN 7 -2820
EN 8 -3167
EN 9 -3685
C-RIB:LEU-CA
EN 3 0
EN 4 0
EN 5 -6122

EN 6 -5430
EN 7 -3306
EN 8 -4502
EN 9 -3814
G-RIB:THR-S1
EN 3 0
EN 4 0
EN 5 -3230
EN 6 -4272
EN 7 -2859
EN 8 -1867
EN 9 -2973
C-RIB:TYR-S1
EN 3 0
EN 4 0
EN 5 -6703
EN 6 -5922
EN 7 -4794
EN 8 -5013
EN 9 -3265
IU-P:HIS-CA
EN 3 0
EN 4 0
EN 5 -17115
EN 6 0
EN 7 0
EN 8 0
EN 9 0
G-R6:TRP-S2
EN 3 0
EN 4 -18832
EN 5 -11788
EN 6 -5997
EN 7 -5735
EN 8 -3413
EN 9 -4275
G-RIB:LYS-CA
EN 3 0
EN 4 0
EN 5 -5266
EN 6 -4565
EN 7 -3772
EN 8 -3676
EN 9 -3515
DA-M5:LYS-S2
EN 3 0
EN 4 0
EN 5 0
EN 6 -7186
EN 7 0
EN 8 0
EN 9 0
FMU-RIB:CYS-CA
EN 3 0
EN 4 0
EN 5 0
EN 6 0
EN 7 -2722

EN 8 0
EN 9 0
FMU-P:ARG-S2
EN 3 0
EN 4 0
EN 5 -17115
EN 6 0
EN 7 0
EN 8 -11174
EN 9 0
FMU-MY:ASP-CA
EN 3 0
EN 4 0
EN 5 0
EN 6 0
EN 7 0
EN 8 -9346
EN 9 0
G-RIB:MET-S1
EN 3 0
EN 4 0
EN 5 0
EN 6 -5196
EN 7 -4993
EN 8 -3697
EN 9 -2774
U-P:ASP-S2
EN 3 0
EN 4 0
EN 5 -1497
EN 6 -2305
EN 7 -1894
EN 8 -1265
EN 9 -1752
C31-RIB:SER-S1
EN 3 0
EN 4 0
EN 5 0
EN 6 0
EN 7 0
EN 8 -6772
EN 9 0
QUO-M6:ARG-S2
EN 3 0
EN 4 0
EN 5 0
EN 6 -3434
EN 7 0
EN 8 -3434
EN 9 0
U-RIB:ARG-S1
EN 3 0
EN 4 0
EN 5 -6130
EN 6 -4210
EN 7 -4062
EN 8 -4239
EN 9 -4221

U-RIB:PRO-CA
EN 3 0
EN 4 0
EN 5 0
EN 6 -3952
EN 7 -3069
EN 8 -4445
EN 9 -2471
A-P:TRP-S1
EN 3 0
EN 4 0
EN 5 -5232
EN 6 -2417
EN 7 -1086
EN 8 -5392
EN 9 -1516
DA-RIB:GLN-CA
EN 3 0
EN 4 0
EN 5 0
EN 6 0
EN 7 0
EN 8 -5295
EN 9 0
DA-M5:ASP-S1
EN 3 0
EN 4 0
EN 5 0
EN 6 0
EN 7 0
EN 8 0
EN 9 -4221
GTP-M6:THR-S1
EN 3 0
EN 4 0
EN 5 0
EN 6 0
EN 7 0
EN 8 0
EN 9 -4101
G-P:LEU-S1
EN 3 0
EN 4 0
EN 5 -5806
EN 6 -3932
EN 7 -932
EN 8 -3197
EN 9 -3219
FHU-MY:TYR-S1
EN 3 0
EN 4 0
EN 5 0
EN 6 -13388
EN 7 0
EN 8 -11812
EN 9 0
A-P:LYS-S1
EN 3 0

EN 4 -4845
EN 5 -6473
EN 6 -5597
EN 7 -4403
EN 8 -3591
EN 9 -3746
C-P:LEU-S2
EN 3 0
EN 4 0
EN 5 -2387
EN 6 -3484
EN 7 -4430
EN 8 -1678
EN 9 -3699
C-P:THR-CA
EN 3 0
EN 4 -6289
EN 5 -5934
EN 6 -3332
EN 7 -3952
EN 8 -3838
EN 9 -3475
GTP-M5:ALA-S1
EN 3 0
EN 4 0
EN 5 0
EN 6 0
EN 7 0
EN 8 -5036
EN 9 0
A-R6:GLU-S2
EN 3 0
EN 4 0
EN 5 -2513
EN 6 -345
EN 7 -2590
EN 8 -625
EN 9 -592
A-P:ASP-S1
EN 3 0
EN 4 0
EN 5 691
EN 6 -981
EN 7 -841
EN 8 -2256
EN 9 -2311
G-RIB:ALA-S1
EN 3 0
EN 4 -2948
EN 5 -3960
EN 6 -3056
EN 7 -1132
EN 8 -2125
EN 9 -3048
A-P:VAL-S1
EN 3 0
EN 4 0
EN 5 -6251

EN 6 -3576
EN 7 -3146
EN 8 -2070
EN 9 -3667
A-RIB:GLU-S1
EN 3 0
EN 4 0
EN 5 -3658
EN 6 -3052
EN 7 -486
EN 8 -669
EN 9 -1151
U-P:PRO-CA
EN 3 0
EN 4 -8414
EN 5 -6945
EN 6 -1977
EN 7 -3822
EN 8 -3366
EN 9 -2521
FHU-RIB:ASP-CA
EN 3 0
EN 4 0
EN 5 0
EN 6 0
EN 7 -10760
EN 8 0
EN 9 0
FHU-P:PRO-S1
EN 3 0
EN 4 0
EN 5 0
EN 6 0
EN 7 0
EN 8 -7523
EN 9 0
G-P:TYR-S2
EN 3 0
EN 4 0
EN 5 -5373
EN 6 -6251
EN 7 -4192
EN 8 -1630
EN 9 -3235
C31-MY:LEU-CA
EN 3 0
EN 4 0
EN 5 0
EN 6 -7054
EN 7 0
EN 8 0
EN 9 0
C-Y:ALA-S1
EN 3 0
EN 4 -4722
EN 5 -5470
EN 6 -3028
EN 7 -3563

EN 8 -3779
EN 9 -3160
U-RIB:LYS-S1
EN 3 0
EN 4 0
EN 5 0
EN 6 -5085
EN 7 -3714
EN 8 -3022
EN 9 -4295
C-Y:ARG-S2
EN 3 0
EN 4 -7546
EN 5 -8429
EN 6 -5607
EN 7 -3952
EN 8 -4124
EN 9 -4396
H2U-MY:PRO-S1
EN 3 0
EN 4 0
EN 5 0
EN 6 0
EN 7 -6352
EN 8 0
EN 9 -4449
G-P:TYR-CA
EN 3 0
EN 4 0
EN 5 -6451
EN 6 -2754
EN 7 -3883
EN 8 -3788
EN 9 -3743
FHU-RIB:GLY-CA
EN 3 0
EN 4 0
EN 5 0
EN 6 -9953
EN 7 -9090
EN 8 -7012
EN 9 0
FHU-RIB:TYR-S1
EN 3 0
EN 4 0
EN 5 0
EN 6 -15398
EN 7 -14393
EN 8 0
EN 9 0
C-P:TYR-S1
EN 3 0
EN 4 0
EN 5 -4479
EN 6 -3535
EN 7 -4426
EN 8 -4581
EN 9 -4253

4SU-P:GLU-S2
EN 3 0
EN 4 0
EN 5 0
EN 6 0
EN 7 0
EN 8 0
EN 9 -2722
C-Y:TRP-S2
EN 3 0
EN 4 -18381
EN 5 -10144
EN 6 -4818
EN 7 -6702
EN 8 -5262
EN 9 -4926
FHU-RIB:ARG-S1
EN 3 0
EN 4 0
EN 5 0
EN 6 0
EN 7 0
EN 8 -6734
EN 9 -8452
U31-P:ASN-CA
EN 3 0
EN 4 0
EN 5 0
EN 6 0
EN 7 -6519
EN 8 0
EN 9 0
DA-M6:HIS-S2
EN 3 0
EN 4 0
EN 5 -9571
EN 6 0
EN 7 0
EN 8 0
EN 9 0
FMU-MY:VAL-CA
EN 3 0
EN 4 0
EN 5 0
EN 6 0
EN 7 0
EN 8 -8378
EN 9 -8306
C31-P:MET-CA
EN 3 0
EN 4 0
EN 5 0
EN 6 0
EN 7 0
EN 8 0
EN 9 -5514
DA-RIB:ARG-S1
EN 3 0

EN 4 0
EN 5 0
EN 6 0
EN 7 0
EN 8 0
EN 9 -4332
C31-MY:GLU-CA
EN 3 0
EN 4 0
EN 5 0
EN 6 0
EN 7 0
EN 8 -5402
EN 9 0
A-RIB:PRO-S1
EN 3 0
EN 4 0
EN 5 -2450
EN 6 -5141
EN 7 -2413
EN 8 -3429
EN 9 -3820
IU-RIB:ARG-S2
EN 3 0
EN 4 0
EN 5 0
EN 6 0
EN 7 0
EN 8 -7278
EN 9 0
H2U-MY:TRP-S2
EN 3 0
EN 4 0
EN 5 -2722
EN 6 0
EN 7 0
EN 8 0
EN 9 0
A-R6:LEU-S2
EN 3 0
EN 4 -9202
EN 5 -5916
EN 6 -7258
EN 7 -3676
EN 8 -4690
EN 9 -4095
G-P:ILE-CA
EN 3 0
EN 4 0
EN 5 -5260
EN 6 0
EN 7 -1454
EN 8 -3304
EN 9 -2578
C-Y:PRO-CA
EN 3 0
EN 4 0
EN 5 -6876

EN 6 -4721
EN 7 -4429
EN 8 -3234
EN 9 -3773
IU-MY:THR-S1
EN 3 0
EN 4 0
EN 5 0
EN 6 0
EN 7 -8688
EN 8 -7611
EN 9 0
A-P:ASN-CA
EN 3 0
EN 4 0
EN 5 -6857
EN 6 -4756
EN 7 -3148
EN 8 -4167
EN 9 -3491
C31-P:GLU-S2
EN 3 0
EN 4 0
EN 5 -8606
EN 6 0
EN 7 0
EN 8 0
EN 9 0
G-R5:ASN-S1
EN 3 0
EN 4 0
EN 5 -8348
EN 6 -4548
EN 7 -3995
EN 8 -3395
EN 9 -4096
DA-RIB:ASN-CA
EN 3 0
EN 4 0
EN 5 0
EN 6 0
EN 7 0
EN 8 -5705
EN 9 0
A-R6:SER-CA
EN 3 0
EN 4 0
EN 5 -2219
EN 6 -5130
EN 7 -2832
EN 8 -3526
EN 9 -2827
A-R6:TRP-CA
EN 3 0
EN 4 0
EN 5 0
EN 6 0
EN 7 -5260

EN 8 -3719
EN 9 -3624
QUO-M5:LYS-S2
EN 3 0
EN 4 0
EN 5 0
EN 6 0
EN 7 0
EN 8 0
EN 9 -17115
FMU-RIB:PHE-S2
EN 3 0
EN 4 0
EN 5 0
EN 6 -4439
EN 7 0
EN 8 0
EN 9 0
A-P:ALA-S1
EN 3 0
EN 4 -1388
EN 5 -5640
EN 6 -4431
EN 7 -3630
EN 8 -2312
EN 9 -2961
G-R5:PRO-CA
EN 3 0
EN 4 0
EN 5 -6324
EN 6 -2837
EN 7 -4864
EN 8 -2711
EN 9 -2478
GTP-RIB:ALA-S1
EN 3 0
EN 4 0
EN 5 0
EN 6 0
EN 7 0
EN 8 0
EN 9 -3673
C-P:MET-S1
EN 3 0
EN 4 0
EN 5 -4784
EN 6 -3238
EN 7 -3106
EN 8 -5023
EN 9 -3622
G-RIB:TYR-CA
EN 3 0
EN 4 0
EN 5 0
EN 6 -3623
EN 7 -4659
EN 8 -2018
EN 9 -2376

A-R5:GLU-S2
EN 3 0
EN 4 0
EN 5 -1541
EN 6 -1538
EN 7 -1799
EN 8 -1928
EN 9 -1032
U31-RIB:TYR-S2
EN 3 0
EN 4 0
EN 5 0
EN 6 0
EN 7 -6137
EN 8 0
EN 9 -5295
U-Y:LYS-CA
EN 3 0
EN 4 0
EN 5 -7131
EN 6 -3933
EN 7 -1305
EN 8 -3447
EN 9 -2705
U31-RIB:ASP-CA
EN 3 0
EN 4 0
EN 5 0
EN 6 0
EN 7 -8606
EN 8 -4925
EN 9 -4180
A-RIB:VAL-CA
EN 3 0
EN 4 0
EN 5 -4322
EN 6 -2053
EN 7 -4474
EN 8 -4120
EN 9 -3038
QUO-RIB:LYS-S2
EN 3 0
EN 4 0
EN 5 0
EN 6 0
EN 7 0
EN 8 -17115
EN 9 0
G-R6:ARG-CA
EN 3 0
EN 4 -9513
EN 5 -6442
EN 6 -4874
EN 7 -2441
EN 8 -3739
EN 9 -3362
U-P:LEU-S1
EN 3 0

EN 4 0
EN 5 0
EN 6 -2087
EN 7 -3846
EN 8 -3662
EN 9 -3700
A-R5:PHE-CA
EN 3 0
EN 4 0
EN 5 0
EN 6 -8091
EN 7 -3920
EN 8 -1887
EN 9 -4841
C-Y:PRO-S1
EN 3 0
EN 4 -8168
EN 5 -4595
EN 6 -5014
EN 7 -3121
EN 8 -2701
EN 9 -3423
U-Y:ASN-S2
EN 3 0
EN 4 -7515
EN 5 -3451
EN 6 -5000
EN 7 -4345
EN 8 -3510
EN 9 -4168
G-RIB:ILE-CA
EN 3 0
EN 4 0
EN 5 -6277
EN 6 -4019
EN 7 -2400
EN 8 -3374
EN 9 -1913
U31-P:MET-S2
EN 3 0
EN 4 0
EN 5 0
EN 6 -11174
EN 7 0
EN 8 0
EN 9 -5680
G-P:SER-S1
EN 3 0
EN 4 -4329
EN 5 -5092
EN 6 -2867
EN 7 -2879
EN 8 -3162
EN 9 -3573
C-Y:HIS-S1
EN 3 0
EN 4 0
EN 5 -5087

EN 6 -7323
EN 7 -5094
EN 8 -4744
EN 9 -4941
FMU-MY:PRO-CA
EN 3 0
EN 4 0
EN 5 0
EN 6 0
EN 7 0
EN 8 -11963
EN 9 0
DA-M5:MET-S2
EN 3 0
EN 4 0
EN 5 0
EN 6 0
EN 7 0
EN 8 -8452
EN 9 0
G-R5:PHE-S1
EN 3 0
EN 4 0
EN 5 0
EN 6 -6046
EN 7 -5476
EN 8 -2567
EN 9 -4416
C31-RIB:LEU-S1
EN 3 0
EN 4 0
EN 5 0
EN 6 0
EN 7 -6107
EN 8 0
EN 9 0
IU-MY:LYS-S2
EN 3 0
EN 4 0
EN 5 0
EN 6 -8437
EN 7 -7278
EN 8 -6270
EN 9 0
U-Y:LEU-S2
EN 3 0
EN 4 0
EN 5 -4281
EN 6 -5542
EN 7 -6163
EN 8 -2727
EN 9 -3155
U-RIB:HIS-S1
EN 3 0
EN 4 0
EN 5 -6780
EN 6 -5749
EN 7 -4621

EN 8 -4313
EN 9 -3485
G-R5:MET-S2
EN 3 0
EN 4 -8378
EN 5 -4571
EN 6 -5930
EN 7 -4595
EN 8 -4662
EN 9 -3136
U-RIB:TYR-S1
EN 3 0
EN 4 0
EN 5 0
EN 6 -4584
EN 7 -4905
EN 8 -4726
EN 9 -3582
C-RIB:GLU-S1
EN 3 0
EN 4 -4037
EN 5 -2650
EN 6 -1270
EN 7 -726
EN 8 -134
EN 9 -800
U-P:ALA-CA
EN 3 0
EN 4 0
EN 5 -6297
EN 6 -4830
EN 7 -2596
EN 8 -3962
EN 9 -3911
IU-MY:ARG-S1
EN 3 0
EN 4 0
EN 5 -12967
EN 6 0
EN 7 0
EN 8 0
EN 9 -8124
U-RIB:MET-CA
EN 3 0
EN 4 0
EN 5 -9494
EN 6 -6722
EN 7 -4797
EN 8 -3299
EN 9 -2308
A-P:ILE-CA
EN 3 0
EN 4 0
EN 5 -4405
EN 6 -4453
EN 7 -1436
EN 8 -3726
EN 9 -2669

IU-MY:GLU-S2
EN 3 0
EN 4 0
EN 5 0
EN 6 0
EN 7 0
EN 8 -5572
EN 9 0
FHU-P:TYR-CA
EN 3 0
EN 4 0
EN 5 0
EN 6 0
EN 7 0
EN 8 -8452
EN 9 -10169
G-R5:PHE-CA
EN 3 0
EN 4 0
EN 5 0
EN 6 0
EN 7 -4014
EN 8 -3735
EN 9 -4526
C-Y:LEU-S1
EN 3 0
EN 4 0
EN 5 -5600
EN 6 -6382
EN 7 -4198
EN 8 -3442
EN 9 -4382
IU-MY:ARG-S2
EN 3 0
EN 4 0
EN 5 -13482
EN 6 0
EN 7 -8980
EN 8 0
EN 9 -9139
C-Y:ARG-S1
EN 3 0
EN 4 0
EN 5 -5799
EN 6 -5768
EN 7 -5445
EN 8 -4594
EN 9 -4891
IU-MY:HIS-CA
EN 3 0
EN 4 0
EN 5 0
EN 6 0
EN 7 0
EN 8 0
EN 9 -11511
A-R6:ASN-S1
EN 3 0

EN 4 0
EN 5 -6069
EN 6 -4588
EN 7 -4343
EN 8 -3164
EN 9 -2530
A-R6:PRO-S1
EN 3 0
EN 4 0
EN 5 -5900
EN 6 -5666
EN 7 -1865
EN 8 -4235
EN 9 -3268
A-RIB:TYR-CA
EN 3 0
EN 4 0
EN 5 -7278
EN 6 -6122
EN 7 -7100
EN 8 -3085
EN 9 -4092
H2U-MY:ASN-CA
EN 3 0
EN 4 0
EN 5 0
EN 6 -10760
EN 7 0
EN 8 0
EN 9 -4717
G-R6:MET-S2
EN 3 0
EN 4 0
EN 5 -5748
EN 6 -6612
EN 7 -4377
EN 8 -3645
EN 9 -3178
FHU-MY:LEU-S2
EN 3 0
EN 4 0
EN 5 -15398
EN 6 0
EN 7 -10169
EN 8 0
EN 9 0
A-RIB:ASN-CA
EN 3 0
EN 4 0
EN 5 -5698
EN 6 -5785
EN 7 -4504
EN 8 -3194
EN 9 -4093
C-Y:PHE-S1
EN 3 0
EN 4 0
EN 5 -7012

EN 6 -3866
EN 7 -3772
EN 8 -4015
EN 9 -4395
G-P:SER-CA
EN 3 0
EN 4 -3797
EN 5 -5673
EN 6 -3793
EN 7 -3676
EN 8 -3330
EN 9 -3236
A-R6:ARG-S2
EN 3 0
EN 4 -8624
EN 5 -6172
EN 6 -4263
EN 7 -3521
EN 8 -3369
EN 9 -4132
G-P:HIS-CA
EN 3 0
EN 4 -8688
EN 5 0
EN 6 -3492
EN 7 -4149
EN 8 -3943
EN 9 -3922
IU-RIB:PRO-CA
EN 3 0
EN 4 0
EN 5 0
EN 6 0
EN 7 0
EN 8 -8080
EN 9 -6810
QUO-M5:GLN-S2
EN 3 0
EN 4 0
EN 5 0
EN 6 0
EN 7 0
EN 8 -17115
EN 9 0
IU-RIB:SER-CA
EN 3 0
EN 4 0
EN 5 0
EN 6 0
EN 7 -7796
EN 8 -6830
EN 9 0
FMU-RIB:ALA-S1
EN 3 0
EN 4 0
EN 5 0
EN 6 0
EN 7 0

EN 8 -9241
EN 9 0
IU-P:ILE-CA
EN 3 0
EN 4 0
EN 5 0
EN 6 0
EN 7 0
EN 8 -9953
EN 9 0
A-P:LYS-S2
EN 3 0
EN 4 -6712
EN 5 -6687
EN 6 -3917
EN 7 -3213
EN 8 -3662
EN 9 -3625
G-RIB:LEU-S2
EN 3 0
EN 4 0
EN 5 -6190
EN 6 -4775
EN 7 -3605
EN 8 -2339
EN 9 -3578
A-R5:ARG-S1
EN 3 0
EN 4 0
EN 5 -7341
EN 6 -5788
EN 7 -4952
EN 8 -3885
EN 9 -4186
A-P:THR-S1
EN 3 0
EN 4 -3189
EN 5 -6574
EN 6 -4121
EN 7 -3701
EN 8 -4529
EN 9 -3397
QUO-M6:ASN-CA
EN 3 0
EN 4 0
EN 5 0
EN 6 0
EN 7 -3434
EN 8 0
EN 9 0
DA-M6:LEU-S2
EN 3 0
EN 4 0
EN 5 0
EN 6 0
EN 7 0
EN 8 0
EN 9 -6181

FMU-MY:GLU-CA
EN 3 0
EN 4 0
EN 5 0
EN 6 0
EN 7 0
EN 8 -8949
EN 9 0
U34-P:TYR-S1
EN 3 0
EN 4 0
EN 5 0
EN 6 0
EN 7 0
EN 8 0
EN 9 -9346
U34-P:GLU-S1
EN 3 0
EN 4 0
EN 5 0
EN 6 0
EN 7 0
EN 8 -7683
EN 9 0
A-R6:LYS-S2
EN 3 0
EN 4 0
EN 5 -3869
EN 6 -3856
EN 7 -2803
EN 8 -2488
EN 9 -2749
H2U-MY:ARG-S1
EN 3 0
EN 4 0
EN 5 0
EN 6 -8236
EN 7 0
EN 8 0
EN 9 0
A-RIB:SER-CA
EN 3 0
EN 4 0
EN 5 -5615
EN 6 -3655
EN 7 -4157
EN 8 -2888
EN 9 -3788
GTP-RIB:ASN-CA
EN 3 0
EN 4 0
EN 5 0
EN 6 -10760
EN 7 0
EN 8 0
EN 9 0
A-R5:PHE-S2
EN 3 0

EN 4 -12675
EN 5 -9414
EN 6 -6527
EN 7 -4495
EN 8 -5639
EN 9 -4242
C31-RIB:LEU-S2
EN 3 0
EN 4 0
EN 5 0
EN 6 -6849
EN 7 0
EN 8 0
EN 9 0
DA-M5:GLU-S1
EN 3 0
EN 4 0
EN 5 0
EN 6 0
EN 7 0
EN 8 0
EN 9 -3866
GTP-RIB:ASN-S2
EN 3 0
EN 4 0
EN 5 0
EN 6 -8452
EN 7 0
EN 8 0
EN 9 0
IU-MY:LEU-S1
EN 3 0
EN 4 0
EN 5 0
EN 6 0
EN 7 -11671
EN 8 0
EN 9 0
U-P:THR-CA
EN 3 0
EN 4 -6660
EN 5 -6528
EN 6 -3504
EN 7 -2123
EN 8 -3468
EN 9 -3399
C-Y:GLU-S2
EN 3 0
EN 4 0
EN 5 598
EN 6 -1109
EN 7 -739
EN 8 -1022
EN 9 -1403
A-R6:ASP-S2
EN 3 0
EN 4 0
EN 5 473

EN 6 -2371
EN 7 -1329
EN 8 -1702
EN 9 -2455
FMU-P:ASP-S2
EN 3 0
EN 4 0
EN 5 0
EN 6 0
EN 7 0
EN 8 0
EN 9 -9456
U31-P:LEU-S1
EN 3 0
EN 4 0
EN 5 0
EN 6 0
EN 7 0
EN 8 0
EN 9 -4667
C-Y:ILE-CA
EN 3 0
EN 4 0
EN 5 0
EN 6 -3659
EN 7 -2456
EN 8 -3352
EN 9 -4608
U-Y:ARG-S1
EN 3 0
EN 4 0
EN 5 -7750
EN 6 -5893
EN 7 -3804
EN 8 -3984
EN 9 -3626
FMU-P:CYS-S1
EN 3 0
EN 4 0
EN 5 0
EN 6 0
EN 7 0
EN 8 0
EN 9 -15398
C31-MY:PHE-S1
EN 3 0
EN 4 0
EN 5 -9346
EN 6 0
EN 7 0
EN 8 0
EN 9 -7231
U-Y:VAL-S1
EN 3 0
EN 4 0
EN 5 -5212
EN 6 -3722
EN 7 -3996

EN 8 -2391
EN 9 -1550
U31-RIB:GLN-S2
EN 3 0
EN 4 0
EN 5 0
EN 6 0
EN 7 -7975
EN 8 0
EN 9 0
FHU-P:PRO-CA
EN 3 0
EN 4 0
EN 5 0
EN 6 0
EN 7 -8528
EN 8 0
EN 9 0
DA-M6:GLN-CA
EN 3 0
EN 4 0
EN 5 0
EN 6 0
EN 7 0
EN 8 -6107
EN 9 0
C-RIB:VAL-CA
EN 3 0
EN 4 0
EN 5 -6549
EN 6 -4730
EN 7 -4683
EN 8 -4188
EN 9 -3019
IU-RIB:SER-S1
EN 3 0
EN 4 0
EN 5 -12477
EN 6 0
EN 7 0
EN 8 -7739
EN 9 0
U31-RIB:GLU-CA
EN 3 0
EN 4 0
EN 5 0
EN 6 0
EN 7 0
EN 8 0
EN 9 -4127
A-R5:THR-CA
EN 3 0
EN 4 0
EN 5 0
EN 6 -6566
EN 7 -4737
EN 8 -4203
EN 9 -4183

QUO-M5:LEU-S1
EN 3 0
EN 4 0
EN 5 0
EN 6 0
EN 7 -3434
EN 8 0
EN 9 -17115
G-P:ARG-S2
EN 3 0
EN 4 -32
EN 5 -8041
EN 6 -4134
EN 7 -3567
EN 8 -3418
EN 9 -3574
C-P:ASN-CA
EN 3 0
EN 4 -6588
EN 5 -4925
EN 6 -5064
EN 7 -4157
EN 8 -3694
EN 9 -3849
G-R5:TRP-S2
EN 3 0
EN 4 0
EN 5 -11764
EN 6 -8043
EN 7 -2604
EN 8 -6475
EN 9 -2163
U-P:ASN-S1
EN 3 0
EN 4 -6863
EN 5 -6348
EN 6 -4254
EN 7 -3052
EN 8 -4118
EN 9 -2357
H2U-MY:GLN-S2
EN 3 0
EN 4 0
EN 5 0
EN 6 -9571
EN 7 0
EN 8 0
EN 9 0
C-Y:MET-S2
EN 3 0
EN 4 0
EN 5 -5172
EN 6 -3683
EN 7 -3890
EN 8 -4677
EN 9 -4751
C31-P:TYR-S1
EN 3 0

EN 4 0
EN 5 0
EN 6 0
EN 7 0
EN 8 -5560
EN 9 -5093
A-R5:ILE-CA
EN 3 0
EN 4 0
EN 5 0
EN 6 -4517
EN 7 -2746
EN 8 -3604
EN 9 -3165
A-R6:LEU-S1
EN 3 0
EN 4 0
EN 5 -6181
EN 6 -7202
EN 7 -5178
EN 8 -4110
EN 9 -4539
FMU-MY:CYS-CA
EN 3 0
EN 4 0
EN 5 -2722
EN 6 0
EN 7 0
EN 8 0
EN 9 0
C31-MY:TYR-S2
EN 3 0
EN 4 0
EN 5 0
EN 6 0
EN 7 0
EN 8 -5832
EN 9 -6320
QUO-M5:LYS-S1
EN 3 0
EN 4 0
EN 5 0
EN 6 0
EN 7 0
EN 8 0
EN 9 -3434
C-RIB:HIS-CA
EN 3 0
EN 4 0
EN 5 -8470
EN 6 -6294
EN 7 -4565
EN 8 -3650
EN 9 -3338
QUO-P:ASN-S2
EN 3 0
EN 4 0
EN 5 0

EN 6 0
EN 7 0
EN 8 0
EN 9 -15398
A-RIB:ASN-S1
EN 3 0
EN 4 0
EN 5 -6591
EN 6 -4496
EN 7 -4274
EN 8 -2993
EN 9 -3874
G-R5:ILE-S1
EN 3 0
EN 4 -8202
EN 5 -3822
EN 6 -1865
EN 7 0
EN 8 -1828
EN 9 -1959
A-R5:GLN-S2
EN 3 0
EN 4 0
EN 5 -6116
EN 6 -4163
EN 7 -3730
EN 8 -3737
EN 9 -4153
A-R6:GLU-CA
EN 3 0
EN 4 0
EN 5 -6111
EN 6 -3097
EN 7 -895
EN 8 -1178
EN 9 -2054
DA-M5:HIS-S2
EN 3 0
EN 4 -12675
EN 5 0
EN 6 0
EN 7 0
EN 8 0
EN 9 0
U-Y:TYR-CA
EN 3 0
EN 4 0
EN 5 -8080
EN 6 -8412
EN 7 -4479
EN 8 -4436
EN 9 -4807
C-RIB:LYS-S1
EN 3 0
EN 4 0
EN 5 -3045
EN 6 -4434
EN 7 -3382

EN 8 -3449
EN 9 -3627
G-R5:ASP-CA
EN 3 0
EN 4 0
EN 5 0
EN 6 -2215
EN 7 602
EN 8 -1669
EN 9 -2973
G-RIB:TYR-S1
EN 3 0
EN 4 0
EN 5 -5413
EN 6 -3304
EN 7 -3200
EN 8 -4292
EN 9 -2842
IU-RIB:LYS-CA
EN 3 0
EN 4 0
EN 5 -11963
EN 6 0
EN 7 0
EN 8 -7152
EN 9 -5977
C-RIB:GLN-S2
EN 3 0
EN 4 0
EN 5 -6611
EN 6 -4980
EN 7 -4114
EN 8 -2948
EN 9 -4087
U-RIB:ILE-S1
EN 3 0
EN 4 0
EN 5 -3827
EN 6 -4940
EN 7 -2766
EN 8 -2976
EN 9 -2316
U31-P:ASP-CA
EN 3 0
EN 4 -2722
EN 5 0
EN 6 0
EN 7 0
EN 8 -4961
EN 9 0
QUO-P:LEU-S2
EN 3 0
EN 4 0
EN 5 0
EN 6 0
EN 7 -15398
EN 8 0
EN 9 0

G-R6:LEU-CA
EN 3 0
EN 4 0
EN 5 -6615
EN 6 -2446
EN 7 -5119
EN 8 -4379
EN 9 -3767
G-P:HIS-S1
EN 3 0
EN 4 -5424
EN 5 -3546
EN 6 -3389
EN 7 -4503
EN 8 -4717
EN 9 -1831
A-R5:ASP-S1
EN 3 0
EN 4 0
EN 5 -2658
EN 6 -3195
EN 7 -2065
EN 8 -983
EN 9 -1918
A-R5:GLU-S1
EN 3 0
EN 4 0
EN 5 -2557
EN 6 -1775
EN 7 -2303
EN 8 -1344
EN 9 -1598
C-Y:THR-CA
EN 3 0
EN 4 0
EN 5 0
EN 6 -6145
EN 7 -3858
EN 8 -3336
EN 9 -3015
DA-M6:LEU-S1
EN 3 0
EN 4 0
EN 5 0
EN 6 0
EN 7 0
EN 8 0
EN 9 -5525
GTP-M5:SER-S1
EN 3 0
EN 4 0
EN 5 0
EN 6 0
EN 7 0
EN 8 -5705
EN 9 -4603
U-RIB:GLN-S1
EN 3 0

EN 4 0
EN 5 -4722
EN 6 -5228
EN 7 -3609
EN 8 -2465
EN 9 -3032
C31-MY:SER-CA
EN 3 0
EN 4 0
EN 5 0
EN 6 0
EN 7 0
EN 8 -6289
EN 9 0
A-R5:LEU-S2
EN 3 0
EN 4 -10958
EN 5 -8504
EN 6 -5723
EN 7 -3566
EN 8 -4905
EN 9 -4013
G-R5:TYR-S1
EN 3 0
EN 4 0
EN 5 0
EN 6 -7264
EN 7 -4211
EN 8 -4868
EN 9 -2610
QUO-RIB:ASN-CA
EN 3 0
EN 4 0
EN 5 0
EN 6 0
EN 7 0
EN 8 -3434
EN 9 0
G-R6:ARG-S2
EN 3 0
EN 4 -4479
EN 5 -3313
EN 6 -5571
EN 7 -4175
EN 8 -3279
EN 9 -2899
C-RIB:PHE-S1
EN 3 0
EN 4 0
EN 5 -6778
EN 6 -1853
EN 7 -2801
EN 8 -2859
EN 9 -3798
QUO-M5:PHE-S2
EN 3 0
EN 4 0
EN 5 -3434

EN 6 -3434
EN 7 0
EN 8 0
EN 9 0
A-R5:HIS-CA
EN 3 0
EN 4 0
EN 5 0
EN 6 -8349
EN 7 -5980
EN 8 -5521
EN 9 -5132
DA-RIB:MET-S2
EN 3 0
EN 4 0
EN 5 -11410
EN 6 0
EN 7 0
EN 8 0
EN 9 0
A-R6:TYR-S2
EN 3 0
EN 4 -12891
EN 5 -8118
EN 6 -6327
EN 7 -4332
EN 8 -3481
EN 9 -4228
C-RIB:GLN-S1
EN 3 0
EN 4 0
EN 5 -4008
EN 6 -6187
EN 7 -4637
EN 8 -4076
EN 9 -3607
U-P:TRP-CA
EN 3 0
EN 4 0
EN 5 -8528
EN 6 -5537
EN 7 -6968
EN 8 -3966
EN 9 -4506
C-Y:MET-S1
EN 3 0
EN 4 0
EN 5 -6588
EN 6 -5384
EN 7 -3242
EN 8 -4486
EN 9 -3571
A-R5:TYR-S1
EN 3 0
EN 4 0
EN 5 -13320
EN 6 -6849
EN 7 -6775

EN 8 -6839
EN 9 -4882
U-Y:HIS-S2
EN 3 0
EN 4 -11581
EN 5 -4629
EN 6 -5364
EN 7 -5961
EN 8 -2646
EN 9 -3706
U-RIB:ASN-S2
EN 3 0
EN 4 -4582
EN 5 -5374
EN 6 -4520
EN 7 -3470
EN 8 -4361
EN 9 -3472
H2U-P:PHE-S1
EN 3 0
EN 4 0
EN 5 0
EN 6 0
EN 7 0
EN 8 0
EN 9 -8772
U-Y:CYS-S1
EN 3 0
EN 4 0
EN 5 0
EN 6 -6916
EN 7 0
EN 8 -4815
EN 9 -5990
G-R6:ASN-S2
EN 3 0
EN 4 0
EN 5 -3063
EN 6 -5460
EN 7 -4413
EN 8 -2591
EN 9 -2976
M2G-P:GLY-CA
EN 3 0
EN 4 0
EN 5 0
EN 6 0
EN 7 0
EN 8 -12294
EN 9 0
G-RIB:ASN-CA
EN 3 0
EN 4 0
EN 5 -6912
EN 6 -5205
EN 7 -4272
EN 8 -4237
EN 9 -3491

H2U-MY:PHE-S1
EN 3 0
EN 4 0
EN 5 -2722
EN 6 0
EN 7 0
EN 8 0
EN 9 0
G-R5:THR-CA
EN 3 0
EN 4 0
EN 5 0
EN 6 -4221
EN 7 -3814
EN 8 -1345
EN 9 -3313
A-P:SER-CA
EN 3 0
EN 4 -6734
EN 5 -6842
EN 6 -5697
EN 7 -3589
EN 8 -4460
EN 9 -3307
A-R5:LYS-CA
EN 3 0
EN 4 -12675
EN 5 0
EN 6 -4663
EN 7 -4155
EN 8 -3152
EN 9 -3609
A-P:ARG-S1
EN 3 0
EN 4 0
EN 5 -7952
EN 6 -6583
EN 7 -4061
EN 8 -5237
EN 9 -4530
C31-RIB:THR-CA
EN 3 0
EN 4 0
EN 5 0
EN 6 -10958
EN 7 0
EN 8 0
EN 9 -6385
5BU-P:PRO-CA
EN 3 0
EN 4 0
EN 5 -14393
EN 6 0
EN 7 0
EN 8 0
EN 9 0
QUO-P:LEU-S1
EN 3 0

EN 4 0
EN 5 0
EN 6 0
EN 7 -17115
EN 8 0
EN 9 0
G-P:ASP-S1
EN 3 0
EN 4 -2022
EN 5 -1196
EN 6 -880
EN 7 -1982
EN 8 -2415
EN 9 -2528
QUO-M5:ASP-S2
EN 3 0
EN 4 0
EN 5 0
EN 6 0
EN 7 -15398
EN 8 -3434
EN 9 0
G-R6:ASN-S1
EN 3 0
EN 4 0
EN 5 -4816
EN 6 -5616
EN 7 -4020
EN 8 -4101
EN 9 -2620
IU-P:ASP-S2
EN 3 0
EN 4 0
EN 5 0
EN 6 0
EN 7 0
EN 8 -7523
EN 9 0
G-R6:ILE-CA
EN 3 0
EN 4 0
EN 5 0
EN 6 -2438
EN 7 360
EN 8 -1257
EN 9 -2878
FHU-P:HIS-S2
EN 3 0
EN 4 0
EN 5 0
EN 6 0
EN 7 0
EN 8 0
EN 9 -10095
IU-RIB:GLN-S2
EN 3 0
EN 4 0
EN 5 0

EN 6 0
EN 7 0
EN 8 -8580
EN 9 0
U34-P:HIS-CA
EN 3 0
EN 4 0
EN 5 0
EN 6 0
EN 7 0
EN 8 0
EN 9 -7575
A-P:SER-S1
EN 3 0
EN 4 -5761
EN 5 -7143
EN 6 -4357
EN 7 -2312
EN 8 -3737
EN 9 -3879
C31-MY:ALA-S1
EN 3 0
EN 4 0
EN 5 0
EN 6 0
EN 7 0
EN 8 -5993
EN 9 0
G-RIB:CYS-CA
EN 3 0
EN 4 0
EN 5 -8547
EN 6 0
EN 7 -2403
EN 8 -2522
EN 9 -2210
DA-M5:GLN-CA
EN 3 0
EN 4 0
EN 5 0
EN 6 0
EN 7 0
EN 8 0
EN 9 -5730
U-P:ASN-CA
EN 3 0
EN 4 -7575
EN 5 -7807
EN 6 -3844
EN 7 -3171
EN 8 -3538
EN 9 -3249
5BU-P:ARG-S2
EN 3 0
EN 4 0
EN 5 -12294
EN 6 0
EN 7 0

EN 8 0
EN 9 0
A-RIB:LEU-S2
EN 3 0
EN 4 0
EN 5 -3934
EN 6 -5067
EN 7 -4202
EN 8 -2261
EN 9 -3667
G-P:PHE-CA
EN 3 0
EN 4 0
EN 5 -7054
EN 6 -3637
EN 7 -3092
EN 8 -2655
EN 9 -3649
DA-RIB:MET-S1
EN 3 0
EN 4 0
EN 5 0
EN 6 0
EN 7 -7739
EN 8 0
EN 9 0
U-RIB:SER-CA
EN 3 0
EN 4 0
EN 5 -5512
EN 6 -3693
EN 7 -4855
EN 8 -2650
EN 9 -2976
A-R6:ALA-CA
EN 3 0
EN 4 -6043
EN 5 -5099
EN 6 -617
EN 7 -2383
EN 8 -1119
EN 9 -3292
U31-MY:ASP-S2
EN 3 0
EN 4 -13680
EN 5 0
EN 6 0
EN 7 0
EN 8 -3706
EN 9 -3535
C31-P:SER-S1
EN 3 0
EN 4 0
EN 5 0
EN 6 0
EN 7 0
EN 8 0
EN 9 -4750

U-Y:SER-S1
EN 3 0
EN 4 -4285
EN 5 -5002
EN 6 -4405
EN 7 -2774
EN 8 -2370
EN 9 -3354
U-Y:TYR-S2
EN 3 0
EN 4 -14790
EN 5 -9886
EN 6 -2423
EN 7 -5246
EN 8 -3278
EN 9 -4308
U-Y:PHE-S2
EN 3 0
EN 4 -15264
EN 5 -8489
EN 6 -7709
EN 7 -2936
EN 8 -4785
EN 9 -4215
QUO-M5:ASN-S1
EN 3 0
EN 4 0
EN 5 0
EN 6 0
EN 7 -3434
EN 8 0
EN 9 0
DA-RIB:HIS-S1
EN 3 0
EN 4 0
EN 5 0
EN 6 -9346
EN 7 0
EN 8 -6553
EN 9 0
A-R5:ILE-S1
EN 3 0
EN 4 0
EN 5 -5348
EN 6 -2611
EN 7 -3087
EN 8 -2519
EN 9 -3641
H2U-MY:GLU-S2
EN 3 0
EN 4 0
EN 5 0
EN 6 -7012
EN 7 0
EN 8 0
EN 9 0
A-P:ARG-S2
EN 3 0

EN 4 0
EN 5 -8683
EN 6 -4233
EN 7 -4466
EN 8 -4586
EN 9 -4019
FMU-MY:PHE-CA
EN 3 0
EN 4 0
EN 5 0
EN 6 0
EN 7 0
EN 8 0
EN 9 -10095
FMU-P:PHE-S2
EN 3 0
EN 4 0
EN 5 0
EN 6 -15398
EN 7 0
EN 8 0
EN 9 0
U34-MY:PHE-S1
EN 3 0
EN 4 0
EN 5 0
EN 6 0
EN 7 0
EN 8 -2722
EN 9 0
U-RIB:ASP-CA
EN 3 0
EN 4 0
EN 5 -4561
EN 6 -2373
EN 7 -1878
EN 8 -2095
EN 9 -1707
A-P:TYR-S2
EN 3 0
EN 4 0
EN 5 -5938
EN 6 -7449
EN 7 -5251
EN 8 -4445
EN 9 -3933
IU-RIB:LYS-S2
EN 3 0
EN 4 0
EN 5 0
EN 6 -6428
EN 7 -4419
EN 8 -4613
EN 9 0
C-RIB:ARG-CA
EN 3 0
EN 4 0
EN 5 -6785

EN 6 -4697
EN 7 -4571
EN 8 -4901
EN 9 -4076
DA-M6:MET-S2
EN 3 0
EN 4 0
EN 5 0
EN 6 0
EN 7 0
EN 8 -9042
EN 9 0
A-RIB:LYS-CA
EN 3 0
EN 4 0
EN 5 -5777
EN 6 -4441
EN 7 -4111
EN 8 -3951
EN 9 -3632
FMU-RIB:ILE-CA
EN 3 0
EN 4 0
EN 5 0
EN 6 0
EN 7 0
EN 8 0
EN 9 -13680
FMU-P:ALA-S1
EN 3 0
EN 4 0
EN 5 0
EN 6 0
EN 7 0
EN 8 -10095
EN 9 0
FMU-RIB:VAL-CA
EN 3 0
EN 4 0
EN 5 0
EN 6 0
EN 7 0
EN 8 -10576
EN 9 0
FHU-P:SER-CA
EN 3 0
EN 4 0
EN 5 0
EN 6 0
EN 7 0
EN 8 -7683
EN 9 -6697
G-R6:PHE-CA
EN 3 0
EN 4 0
EN 5 0
EN 6 -5758
EN 7 -3254

EN 8 -4170
EN 9 -2543
G-RIB:VAL-S1
EN 3 0
EN 4 0
EN 5 -2443
EN 6 -3493
EN 7 -1825
EN 8 -1494
EN 9 -2217
FMU-P:ALA-CA
EN 3 0
EN 4 0
EN 5 0
EN 6 0
EN 7 0
EN 8 0
EN 9 -9042
U-P:SER-CA
EN 3 0
EN 4 0
EN 5 -6581
EN 6 -3615
EN 7 -4677
EN 8 -3297
EN 9 -3286
H2U-P:TRP-S1
EN 3 0
EN 4 0
EN 5 0
EN 6 0
EN 7 0
EN 8 -12675
EN 9 0
QUO-M6:PHE-S2
EN 3 0
EN 4 0
EN 5 -3434
EN 6 -3434
EN 7 0
EN 8 0
EN 9 0
GTP-M6:ASN-S1
EN 3 0
EN 4 0
EN 5 0
EN 6 0
EN 7 0
EN 8 0
EN 9 -5402
C-Y:TYR-S1
EN 3 0
EN 4 -11671
EN 5 -11000
EN 6 -6381
EN 7 -4647
EN 8 -4659
EN 9 -4137

A-R5:LYS-S2
EN 3 0
EN 4 0
EN 5 -5275
EN 6 -2574
EN 7 -4084
EN 8 -3295
EN 9 -3606
U31-RIB:MET-CA
EN 3 0
EN 4 0
EN 5 0
EN 6 -13127
EN 7 0
EN 8 0
EN 9 0
FHU-P:GLY-CA
EN 3 0
EN 4 -12294
EN 5 -11410
EN 6 0
EN 7 -7054
EN 8 -5884
EN 9 0
QUO-M6:ASP-S2
EN 3 0
EN 4 0
EN 5 0
EN 6 0
EN 7 -17115
EN 8 -17115
EN 9 0
A-R6:PRO-CA
EN 3 0
EN 4 0
EN 5 -6629
EN 6 -4622
EN 7 -4227
EN 8 -4021
EN 9 -2776
G-R5:CYS-CA
EN 3 0
EN 4 0
EN 5 0
EN 6 0
EN 7 0
EN 8 -3665
EN 9 -3920
U31-MY:MET-S2
EN 3 0
EN 4 -10405
EN 5 0
EN 6 0
EN 7 0
EN 8 0
EN 9 0
U-RIB:ASP-S1
EN 3 0

EN 4 -4847
EN 5 -3291
EN 6 -554
EN 7 -2389
EN 8 -981
EN 9 -1091
U-Y:THR-S1
EN 3 0
EN 4 0
EN 5 -4790
EN 6 -4520
EN 7 -1995
EN 8 -3052
EN 9 -2684
H2U-P:TRP-CA
EN 3 0
EN 4 0
EN 5 0
EN 6 0
EN 7 0
EN 8 0
EN 9 -10958
FMU-RIB:PHE-CA
EN 3 0
EN 4 0
EN 5 0
EN 6 0
EN 7 0
EN 8 0
EN 9 -17115
G-RIB:ASN-S2
EN 3 0
EN 4 -3105
EN 5 -4251
EN 6 -5117
EN 7 -3518
EN 8 -3096
EN 9 -3289
G-R6:GLN-S1
EN 3 0
EN 4 0
EN 5 0
EN 6 -2389
EN 7 -4285
EN 8 -4600
EN 9 -3448
U31-MY:GLU-S1
EN 3 0
EN 4 0
EN 5 0
EN 6 0
EN 7 0
EN 8 -4571
EN 9 0
U31-MY:TYR-S2
EN 3 0
EN 4 0
EN 5 -10576

EN 6 0
EN 7 0
EN 8 0
EN 9 0
C-RIB:LYS-CA
EN 3 0
EN 4 0
EN 5 -4474
EN 6 -4585
EN 7 -3759
EN 8 -3819
EN 9 -3638
FMU-RIB:GLU-S1
EN 3 0
EN 4 0
EN 5 0
EN 6 0
EN 7 0
EN 8 0
EN 9 -9139
IU-P:ARG-S2
EN 3 0
EN 4 0
EN 5 0
EN 6 0
EN 7 0
EN 8 0
EN 9 -6849
A-P:GLN-CA
EN 3 0
EN 4 0
EN 5 -5705
EN 6 -5325
EN 7 -3711
EN 8 -3948
EN 9 -4285
G-R6:ILE-S1
EN 3 0
EN 4 -7289
EN 5 -4000
EN 6 0
EN 7 824
EN 8 -1095
EN 9 -2226
U-RIB:TYR-S2
EN 3 0
EN 4 0
EN 5 -6577
EN 6 -5548
EN 7 -5119
EN 8 -2800
EN 9 -3630
A-R5:THR-S1
EN 3 0
EN 4 -6289
EN 5 -6044
EN 6 -4659
EN 7 -3856

EN 8 -3695
EN 9 -3346
QUO-M5:GLU-S1
EN 3 0
EN 4 0
EN 5 0
EN 6 0
EN 7 0
EN 8 0
EN 9 -15398
C-P:HIS-CA
EN 3 0
EN 4 0
EN 5 -4167
EN 6 -5659
EN 7 -2048
EN 8 -4458
EN 9 -4231
U34-P:ARG-S1
EN 3 0
EN 4 0
EN 5 0
EN 6 0
EN 7 0
EN 8 0
EN 9 -2722
FHU-P:LYS-S2
EN 3 0
EN 4 -12675
EN 5 -9571
EN 6 0
EN 7 0
EN 8 -8271
EN 9 0
U-P:GLN-CA
EN 3 0
EN 4 0
EN 5 -5506
EN 6 -3635
EN 7 -3061
EN 8 -3483
EN 9 -3971
IU-MY:TYR-CA
EN 3 0
EN 4 0
EN 5 0
EN 6 0
EN 7 0
EN 8 0
EN 9 -10666
DA-M6:ASN-S1
EN 3 0
EN 4 0
EN 5 0
EN 6 0
EN 7 -6352
EN 8 0
EN 9 -4818

C-RIB:MET-S1
EN 3 0
EN 4 -8606
EN 5 -7254
EN 6 -6217
EN 7 -4225
EN 8 -3756
EN 9 -2382
C-RIB:THR-S1
EN 3 0
EN 4 0
EN 5 -5109
EN 6 -5029
EN 7 -3394
EN 8 -3704
EN 9 -3187
FHU-P:LEU-S1
EN 3 0
EN 4 0
EN 5 0
EN 6 0
EN 7 -8038
EN 8 0
EN 9 -9346
5BU-RIB:ILE-S1
EN 3 0
EN 4 0
EN 5 0
EN 6 0
EN 7 0
EN 8 0
EN 9 -15398
A-P:MET-S1
EN 3 0
EN 4 0
EN 5 -3567
EN 6 -3092
EN 7 -3808
EN 8 -4657
EN 9 -3846
G-P:LYS-S2
EN 3 0
EN 4 -5358
EN 5 -6174
EN 6 -3676
EN 7 -4125
EN 8 -3383
EN 9 -3619
G-R6:LYS-CA
EN 3 0
EN 4 0
EN 5 -5494
EN 6 -3638
EN 7 -2002
EN 8 -3390
EN 9 -3219
FMU-MY:ILE-CA
EN 3 0

EN 4 0
EN 5 0
EN 6 0
EN 7 0
EN 8 0
EN 9 -11410
5BU-P:ALA-S1
EN 3 0
EN 4 0
EN 5 0
EN 6 0
EN 7 0
EN 8 0
EN 9 -7683
FMU-MY:GLN-S2
EN 3 0
EN 4 0
EN 5 -12294
EN 6 0
EN 7 0
EN 8 0
EN 9 0
IU-RIB:LEU-S1
EN 3 0
EN 4 0
EN 5 -1717
EN 6 0
EN 7 0
EN 8 0
EN 9 0
U-P:PHE-CA
EN 3 0
EN 4 0
EN 5 -5198
EN 6 -1967
EN 7 -3306
EN 8 -2792
EN 9 -3404
G-P:PRO-CA
EN 3 0
EN 4 0
EN 5 -5007
EN 6 -3336
EN 7 -3851
EN 8 -2892
EN 9 -3727
G-R5:TYR-S2
EN 3 0
EN 4 -10958
EN 5 -9130
EN 6 -4479
EN 7 -4436
EN 8 -4228
EN 9 -4227
U-Y:LYS-S2
EN 3 0
EN 4 0
EN 5 0

EN 6 -4370
EN 7 -3601
EN 8 -3430
EN 9 -3994
OMC-RIB:LYS-S1
EN 3 0
EN 4 0
EN 5 0
EN 6 0
EN 7 0
EN 8 -4847
EN 9 0
QUO-M5:ARG-S2
EN 3 0
EN 4 0
EN 5 0
EN 6 0
EN 7 -3434
EN 8 0
EN 9 -3434
G-RIB:PHE-S1
EN 3 0
EN 4 0
EN 5 -6859
EN 6 -5182
EN 7 -5668
EN 8 -3387
EN 9 -3353
5BU-RIB:ARG-S2
EN 3 0
EN 4 0
EN 5 0
EN 6 0
EN 7 0
EN 8 0
EN 9 -6849
FHU-RIB:ARG-CA
EN 3 0
EN 4 0
EN 5 0
EN 6 0
EN 7 0
EN 8 0
EN 9 -8271
C-P:ASP-S2
EN 3 0
EN 4 0
EN 5 -1113
EN 6 -2011
EN 7 -2159
EN 8 -1945
EN 9 -3008
G-R6:MET-S1
EN 3 0
EN 4 0
EN 5 -5680
EN 6 -7675
EN 7 -4133

EN 8 -3228
EN 9 -3825
G-P:GLN-CA
EN 3 0
EN 4 0
EN 5 -7192
EN 6 -2705
EN 7 -3664
EN 8 -4613
EN 9 -2649
U-RIB:TRP-S1
EN 3 0
EN 4 0
EN 5 0
EN 6 -5139
EN 7 -6115
EN 8 -2265
EN 9 -4294
GTP-M5:ASP-S1
EN 3 0
EN 4 0
EN 5 0
EN 6 0
EN 7 0
EN 8 0
EN 9 -4750
G-R6:HIS-CA
EN 3 0
EN 4 0
EN 5 -5918
EN 6 -5763
EN 7 -4296
EN 8 -2183
EN 9 -4685
A-R6:MET-S2
EN 3 0
EN 4 -6050
EN 5 -8347
EN 6 -4145
EN 7 -4018
EN 8 -5368
EN 9 -2431
U-RIB:LYS-S2
EN 3 0
EN 4 -1381
EN 5 -3424
EN 6 -2529
EN 7 -4393
EN 8 -4198
EN 9 -3516
FMU-RIB:GLU-S2
EN 3 0
EN 4 0
EN 5 0
EN 6 0
EN 7 0
EN 8 -9346
EN 9 0

C-Y:GLU-CA
EN 3 0
EN 4 0
EN 5 -6003
EN 6 -3764
EN 7 2418
EN 8 535
EN 9 -1552
DA-RIB:TYR-CA
EN 3 0
EN 4 0
EN 5 0
EN 6 0
EN 7 0
EN 8 0
EN 9 -6417
H2U-MY:TRP-CA
EN 3 0
EN 4 0
EN 5 0
EN 6 0
EN 7 -17115
EN 8 0
EN 9 0
C-RIB:HIS-S2
EN 3 0
EN 4 -8414
EN 5 -5808
EN 6 -5440
EN 7 -3575
EN 8 -4340
EN 9 -3202
C31-RIB:ALA-S1
EN 3 0
EN 4 0
EN 5 0
EN 6 0
EN 7 0
EN 8 0
EN 9 -6909
FMU-RIB:ARG-CA
EN 3 0
EN 4 0
EN 5 0
EN 6 0
EN 7 0
EN 8 -12675
EN 9 0
U31-MY:GLU-CA
EN 3 0
EN 4 0
EN 5 0
EN 6 0
EN 7 0
EN 8 -4127
EN 9 0
A-RIB:TYR-S1
EN 3 0

EN 4 0
EN 5 -9011
EN 6 -8072
EN 7 -6420
EN 8 -3886
EN 9 -3267
C-P:VAL-CA
EN 3 0
EN 4 0
EN 5 -4006
EN 6 -3838
EN 7 -4274
EN 8 -4157
EN 9 -3549
G-R6:ASN-CA
EN 3 0
EN 4 0
EN 5 0
EN 6 -6241
EN 7 -5131
EN 8 -3126
EN 9 -3359
C31-P:TYR-CA
EN 3 0
EN 4 0
EN 5 0
EN 6 0
EN 7 0
EN 8 0
EN 9 -7054
C-P:MET-CA
EN 3 0
EN 4 -8378
EN 5 -4332
EN 6 -1212
EN 7 -3542
EN 8 -3777
EN 9 -3305
QUO-M6:ASN-S1
EN 3 0
EN 4 0
EN 5 0
EN 6 -3434
EN 7 0
EN 8 0
EN 9 0
IU-RIB:ILE-CA
EN 3 0
EN 4 0
EN 5 0
EN 6 -11812
EN 7 -9532
EN 8 0
EN 9 0
DA-M6:GLU-S1
EN 3 0
EN 4 0
EN 5 0

EN 6 0
EN 7 0
EN 8 -4525
EN 9 0
G-RIB:PHE-S2
EN 3 0
EN 4 0
EN 5 -6553
EN 6 -6280
EN 7 -4961
EN 8 -3032
EN 9 -2310
A-RIB:PHE-S1
EN 3 0
EN 4 0
EN 5 -8118
EN 6 -5272
EN 7 -5342
EN 8 -4210
EN 9 -3427
C-P:PHE-CA
EN 3 0
EN 4 0
EN 5 0
EN 6 0
EN 7 -1573
EN 8 -3623
EN 9 -2542
OMC-MY:LYS-S2
EN 3 0
EN 4 0
EN 5 0
EN 6 0
EN 7 0
EN 8 -5884
EN 9 0
C31-MY:THR-CA
EN 3 0
EN 4 0
EN 5 0
EN 6 0
EN 7 0
EN 8 0
EN 9 -4464
FHU-P:ASP-S1
EN 3 0
EN 4 0
EN 5 0
EN 6 0
EN 7 0
EN 8 0
EN 9 -5631
FHU-P:LYS-S1
EN 3 0
EN 4 0
EN 5 -13680
EN 6 -10666
EN 7 -7097

EN 8 0
EN 9 0
FHU-MY:THR-S1
EN 3 0
EN 4 0
EN 5 0
EN 6 0
EN 7 0
EN 8 -7472
EN 9 -10095
A-R6:TRP-S2
EN 3 0
EN 4 0
EN 5 -7683
EN 6 -7536
EN 7 -5594
EN 8 -4291
EN 9 -3266
C31-RIB:THR-S1
EN 3 0
EN 4 0
EN 5 0
EN 6 -9456
EN 7 0
EN 8 0
EN 9 0
H2U-P:ARG-S2
EN 3 0
EN 4 0
EN 5 0
EN 6 -9692
EN 7 0
EN 8 0
EN 9 0
FMU-P:ASP-CA
EN 3 0
EN 4 0
EN 5 0
EN 6 0
EN 7 0
EN 8 -10576
EN 9 0
A-P:GLU-CA
EN 3 0
EN 4 0
EN 5 -2562
EN 6 0
EN 7 368
EN 8 -926
EN 9 -1531
C31-MY:SER-S1
EN 3 0
EN 4 0
EN 5 0
EN 6 0
EN 7 0
EN 8 0
EN 9 -4961

G-R5:SER-CA
EN 3 0
EN 4 0
EN 5 0
EN 6 -5617
EN 7 -3129
EN 8 -3433
EN 9 -2614
C-P:PHE-S2
EN 3 0
EN 4 0
EN 5 0
EN 6 -4784
EN 7 -2052
EN 8 -3167
EN 9 -3844
A-R6:MET-S1
EN 3 0
EN 4 -8949
EN 5 -6697
EN 6 -4127
EN 7 -5930
EN 8 -4479
EN 9 -3909
U-Y:GLN-S2
EN 3 0
EN 4 0
EN 5 -5328
EN 6 -5946
EN 7 -3565
EN 8 -2903
EN 9 -3227
U-P:LEU-CA
EN 3 0
EN 4 0
EN 5 0
EN 6 -532
EN 7 -3882
EN 8 -3571
EN 9 -2814
U-P:THR-S1
EN 3 0
EN 4 -3027
EN 5 -5930
EN 6 -2497
EN 7 -2691
EN 8 -3513
EN 9 -2515
U-Y:ASN-S1
EN 3 0
EN 4 0
EN 5 -5256
EN 6 -3612
EN 7 -4663
EN 8 -4558
EN 9 -3700
U-Y:GLN-S1
EN 3 0

EN 4 0
EN 5 0
EN 6 0
EN 7 -4307
EN 8 -5088
EN 9 -3554
A-RIB:ASP-S1
EN 3 0
EN 4 -4587
EN 5 -3601
EN 6 -1795
EN 7 -2448
EN 8 -848
EN 9 -1836
5BU-P:SER-S1
EN 3 0
EN 4 0
EN 5 0
EN 6 0
EN 7 0
EN 8 -7914
EN 9 0
C-P:GLU-S2
EN 3 0
EN 4 0
EN 5 0
EN 6 -600
EN 7 -1060
EN 8 -1306
EN 9 -1251
C-RIB:ILE-CA
EN 3 0
EN 4 0
EN 5 0
EN 6 -5837
EN 7 -3311
EN 8 -3484
EN 9 -3211
FHU-MY:LYS-S1
EN 3 0
EN 4 0
EN 5 0
EN 6 0
EN 7 0
EN 8 0
EN 9 -8306
G-P:GLU-S1
EN 3 0
EN 4 0
EN 5 413
EN 6 2883
EN 7 755
EN 8 -1248
EN 9 -680
FMU-RIB:ARG-S2
EN 3 0
EN 4 0
EN 5 -12675

EN 6 0
EN 7 0
EN 8 -9401
EN 9 0
DA-M5:TYR-CA
EN 3 0
EN 4 0
EN 5 0
EN 6 0
EN 7 -9139
EN 8 0
EN 9 -5993
U31-MY:THR-S1
EN 3 0
EN 4 0
EN 5 0
EN 6 -9692
EN 7 0
EN 8 -5560
EN 9 -4767
A-R5:ALA-CA
EN 3 0
EN 4 0
EN 5 -5348
EN 6 -2034
EN 7 -3359
EN 8 -2206
EN 9 -2924
C-Y:CYS-CA
EN 3 0
EN 4 0
EN 5 0
EN 6 -7934
EN 7 0
EN 8 0
EN 9 -3250
C-P:ALA-CA
EN 3 0
EN 4 0
EN 5 -6620
EN 6 -3342
EN 7 -2631
EN 8 -3847
EN 9 -3633
H2U-RIB:PHE-S1
EN 3 0
EN 4 0
EN 5 0
EN 6 0
EN 7 0
EN 8 -11410
EN 9 0
H2U-MY:GLN-CA
EN 3 0
EN 4 0
EN 5 0
EN 6 0
EN 7 0

EN 8 0
EN 9 -6660
IU-MY:PRO-CA
EN 3 0
EN 4 0
EN 5 0
EN 6 0
EN 7 0
EN 8 -8859
EN 9 -8402
U-Y:TRP-S2
EN 3 0
EN 4 -17115
EN 5 0
EN 6 -5344
EN 7 -2980
EN 8 0
EN 9 -4956
U31-P:SER-S1
EN 3 0
EN 4 0
EN 5 0
EN 6 0
EN 7 0
EN 8 0
EN 9 -5295
A-P:ASP-CA
EN 3 0
EN 4 0
EN 5 -2576
EN 6 0
EN 7 -396
EN 8 -2655
EN 9 -3015
G-R5:ASP-S1
EN 3 0
EN 4 0
EN 5 0
EN 6 -1525
EN 7 -653
EN 8 -3031
EN 9 -2191
U-P:CYS-CA
EN 3 0
EN 4 0
EN 5 0
EN 6 -5099
EN 7 0
EN 8 -3570
EN 9 -2449
DA-M6:GLU-S2
EN 3 0
EN 4 0
EN 5 -7097
EN 6 0
EN 7 0
EN 8 0
EN 9 0

U31-MY:THR-CA
EN 3 0
EN 4 0
EN 5 0
EN 6 -10760
EN 7 -7914
EN 8 0
EN 9 0
FHU-RIB:ILE-S1
EN 3 0
EN 4 0
EN 5 0
EN 6 0
EN 7 0
EN 8 -8168
EN 9 -7796
G-P:LYS-S1
EN 3 0
EN 4 -3185
EN 5 -5485
EN 6 -5893
EN 7 -3844
EN 8 -3546
EN 9 -2977
U34-RIB:SER-S1
EN 3 0
EN 4 0
EN 5 0
EN 6 0
EN 7 -5468
EN 8 0
EN 9 -4449
IU-MY:MET-CA
EN 3 0
EN 4 0
EN 5 0
EN 6 0
EN 7 0
EN 8 0
EN 9 -8168
A-R6:ASN-CA
EN 3 0
EN 4 0
EN 5 -4907
EN 6 -4325
EN 7 -4550
EN 8 -3338
EN 9 -3607
A-P:ARG-CA
EN 3 0
EN 4 0
EN 5 -6929
EN 6 -5709
EN 7 -5526
EN 8 -5306
EN 9 -4340
G-R6:VAL-S1
EN 3 0

EN 4 0
EN 5 -5124
EN 6 -3204
EN 7 -1926
EN 8 -2282
EN 9 -1955
U31-RIB:ASN-S2
EN 3 0
EN 4 0
EN 5 0
EN 6 0
EN 7 0
EN 8 -4509
EN 9 0
DA-M5:HIS-CA
EN 3 0
EN 4 0
EN 5 0
EN 6 -12675
EN 7 0
EN 8 0
EN 9 0
U34-MY:ASP-S2
EN 3 0
EN 4 0
EN 5 0
EN 6 0
EN 7 0
EN 8 0
EN 9 -7012
FMU-MY:MET-S2
EN 3 0
EN 4 0
EN 5 0
EN 6 0
EN 7 -2722
EN 8 -13680
EN 9 0
U-P:ILE-S1
EN 3 0
EN 4 0
EN 5 -4048
EN 6 -3934
EN 7 -1429
EN 8 -1477
EN 9 -2505
DA-M5:LEU-S2
EN 3 0
EN 4 0
EN 5 0
EN 6 0
EN 7 0
EN 8 0
EN 9 -4000
A-R6:TRP-S1
EN 3 0
EN 4 0
EN 5 -5911

EN 6 -3577
EN 7 -4768
EN 8 0
EN 9 -4222
U-RIB:CYS-CA
EN 3 0
EN 4 0
EN 5 0
EN 6 0
EN 7 0
EN 8 -5787
EN 9 -3288
H2U-RIB:ARG-S2
EN 3 0
EN 4 0
EN 5 0
EN 6 -9241
EN 7 0
EN 8 0
EN 9 -4818
G-RIB:ARG-S1
EN 3 0
EN 4 0
EN 5 -3794
EN 6 -5165
EN 7 -3850
EN 8 -3877
EN 9 -4016
QUO-RIB:ASN-S1
EN 3 0
EN 4 0
EN 5 0
EN 6 0
EN 7 -3434
EN 8 0
EN 9 0
U31-RIB:GLU-S2
EN 3 0
EN 4 0
EN 5 0
EN 6 0
EN 7 -4700
EN 8 0
EN 9 0
U31-P:GLN-S1
EN 3 0
EN 4 0
EN 5 0
EN 6 -10576
EN 7 0
EN 8 0
EN 9 0
C31-RIB:TYR-S2
EN 3 0
EN 4 0
EN 5 0
EN 6 0
EN 7 -8647

EN 8 0
EN 9 0
U34-RIB:SER-CA
EN 3 0
EN 4 0
EN 5 0
EN 6 0
EN 7 0
EN 8 0
EN 9 -3914
H2U-MY:GLU-CA
EN 3 0
EN 4 0
EN 5 0
EN 6 -9346
EN 7 0
EN 8 0
EN 9 0
IU-P:LYS-CA
EN 3 0
EN 4 0
EN 5 0
EN 6 0
EN 7 0
EN 8 -7656
EN 9 0
A-RIB:MET-CA
EN 3 0
EN 4 0
EN 5 -7796
EN 6 -4283
EN 7 -4105
EN 8 -4530
EN 9 -3923
FMU-MY:CYS-S1
EN 3 0
EN 4 -2722
EN 5 0
EN 6 0
EN 7 0
EN 8 0
EN 9 0
DA-RIB:ARG-CA
EN 3 0
EN 4 0
EN 5 0
EN 6 0
EN 7 0
EN 8 0
EN 9 -4556
DA-RIB:MET-CA
EN 3 0
EN 4 0
EN 5 0
EN 6 0
EN 7 0
EN 8 0
EN 9 -6849

GTP-RIB:GLY-CA
EN 3 0
EN 4 0
EN 5 0
EN 6 0
EN 7 -5656
EN 8 0
EN 9 0
A-R5:GLN-S1
EN 3 0
EN 4 0
EN 5 -5237
EN 6 -6229
EN 7 -3348
EN 8 -3293
EN 9 -3789
A-R5:ASN-S2
EN 3 0
EN 4 0
EN 5 -4272
EN 6 -1400
EN 7 -5025
EN 8 -4232
EN 9 -3844
IU-P:SER-S1
EN 3 0
EN 4 0
EN 5 0
EN 6 0
EN 7 0
EN 8 0
EN 9 -9886
G-R5:ARG-S2
EN 3 0
EN 4 -7519
EN 5 -6784
EN 6 -5214
EN 7 -4094
EN 8 -3563
EN 9 -3791
A-R5:TRP-S2
EN 3 0
EN 4 0
EN 5 0
EN 6 -7054
EN 7 -6196
EN 8 -5625
EN 9 -3735
QUO-M6:GLN-S2
EN 3 0
EN 4 0
EN 5 0
EN 6 0
EN 7 -17115
EN 8 0
EN 9 0
C31-MY:ASP-CA
EN 3 0

EN 4 0
EN 5 0
EN 6 0
EN 7 0
EN 8 0
EN 9 -4276
QUO-RIB:PHE-S1
EN 3 0
EN 4 0
EN 5 0
EN 6 0
EN 7 0
EN 8 0
EN 9 -3434
U-P:GLN-S2
EN 3 0
EN 4 0
EN 5 -5573
EN 6 -3026
EN 7 -2469
EN 8 -2761
EN 9 -3029
A-RIB:LYS-S1
EN 3 0
EN 4 0
EN 5 -3042
EN 6 -5126
EN 7 -3896
EN 8 -3285
EN 9 -3784
GTP-M6:ASN-CA
EN 3 0
EN 4 0
EN 5 0
EN 6 0
EN 7 0
EN 8 0
EN 9 -5514
H2U-MY:ASN-S1
EN 3 0
EN 4 0
EN 5 0
EN 6 -11410
EN 7 0
EN 8 -5491
EN 9 -4801
U-P:GLY-CA
EN 3 0
EN 4 -6105
EN 5 -6141
EN 6 -4151
EN 7 -3570
EN 8 -3330
EN 9 -3423
5BU-P:SER-CA
EN 3 0
EN 4 0
EN 5 0

EN 6 0
EN 7 0
EN 8 -8378
EN 9 0
G-RIB:GLU-S2
EN 3 0
EN 4 0
EN 5 -3076
EN 6 -753
EN 7 406
EN 8 -271
EN 9 -348
U-RIB:GLU-S2
EN 3 0
EN 4 0
EN 5 -2271
EN 6 650
EN 7 314
EN 8 231
EN 9 -252
U31-MY:PHE-S2
EN 3 0
EN 4 0
EN 5 0
EN 6 0
EN 7 0
EN 8 0
EN 9 -5537
U-P:TRP-S1
EN 3 0
EN 4 -8306
EN 5 -6943
EN 6 -5702
EN 7 -4431
EN 8 -5465
EN 9 -4818
G-R5:GLY-CA
EN 3 0
EN 4 -5117
EN 5 -6866
EN 6 -4379
EN 7 -3426
EN 8 -4023
EN 9 -3227
A-RIB:THR-S1
EN 3 0
EN 4 0
EN 5 -5856
EN 6 -5465
EN 7 -3438
EN 8 -3997
EN 9 -3526
A-RIB:TRP-CA
EN 3 0
EN 4 0
EN 5 0
EN 6 -6258
EN 7 -3309

EN 8 -5702
EN 9 -3629
U31-MY:VAL-S1
EN 3 0
EN 4 0
EN 5 0
EN 6 0
EN 7 0
EN 8 -7373
EN 9 0
C31-P:MET-S1
EN 3 0
EN 4 0
EN 5 0
EN 6 0
EN 7 0
EN 8 -5755
EN 9 0
U-Y:CYS-CA
EN 3 0
EN 4 0
EN 5 0
EN 6 -8729
EN 7 0
EN 8 -4213
EN 9 -3161
FMU-RIB:MET-S1
EN 3 0
EN 4 0
EN 5 0
EN 6 0
EN 7 0
EN 8 0
EN 9 -17115
H2U-MY:LYS-S1
EN 3 0
EN 4 0
EN 5 0
EN 6 0
EN 7 0
EN 8 0
EN 9 -4405
A-RIB:GLU-CA
EN 3 0
EN 4 0
EN 5 -4944
EN 6 -2467
EN 7 -1488
EN 8 -356
EN 9 -1643
FMU-RIB:ARG-S1
EN 3 0
EN 4 0
EN 5 0
EN 6 -13127
EN 7 0
EN 8 0
EN 9 -9241

QUO-M6:LYS-CA
EN 3 0
EN 4 0
EN 5 0
EN 6 0
EN 7 0
EN 8 0
EN 9 -3434
G-R6:TRP-S1
EN 3 0
EN 4 -10958
EN 5 -9908
EN 6 0
EN 7 -6533
EN 8 -3185
EN 9 -5525
C-Y:GLN-S2
EN 3 0
EN 4 0
EN 5 -5255
EN 6 -5772
EN 7 -4888
EN 8 -4014
EN 9 -3408
A-R5:GLU-CA
EN 3 0
EN 4 0
EN 5 -6368
EN 6 -743
EN 7 -3099
EN 8 -2437
EN 9 -1581
G-R6:SER-S1
EN 3 0
EN 4 0
EN 5 -3265
EN 6 -4572
EN 7 -3010
EN 8 -2605
EN 9 -2953
A-R5:ARG-S2
EN 3 0
EN 4 -10781
EN 5 -6365
EN 6 -5681
EN 7 -3077
EN 8 -3770
EN 9 -4747
5BU-RIB:PRO-S1
EN 3 0
EN 4 0
EN 5 0
EN 6 0
EN 7 -11963
EN 8 0
EN 9 0
G-P:ASN-S1
EN 3 0

EN 4 -3577
EN 5 -6422
EN 6 -3745
EN 7 -4123
EN 8 -3425
EN 9 -3445
U-P:ARG-CA
EN 3 0
EN 4 -6227
EN 5 -6472
EN 6 -5877
EN 7 -3924
EN 8 -4478
EN 9 -4233
FMU-MY:VAL-S1
EN 3 0
EN 4 0
EN 5 0
EN 6 0
EN 7 0
EN 8 -10245
EN 9 0
C-Y:LYS-CA
EN 3 0
EN 4 0
EN 5 0
EN 6 -5993
EN 7 -3522
EN 8 -3073
EN 9 -3730
U-RIB:THR-CA
EN 3 0
EN 4 0
EN 5 0
EN 6 -5839
EN 7 -3592
EN 8 -3917
EN 9 -3084
U31-RIB:ASP-S1
EN 3 0
EN 4 0
EN 5 0
EN 6 -7231
EN 7 -5514
EN 8 -5980
EN 9 0
FHU-RIB:LYS-S2
EN 3 0
EN 4 0
EN 5 0
EN 6 0
EN 7 0
EN 8 0
EN 9 -9382
U34-P:HIS-S2
EN 3 0
EN 4 0
EN 5 -2722

EN 6 0
EN 7 0
EN 8 0
EN 9 -10095
H2U-MY:THR-CA
EN 3 0
EN 4 0
EN 5 0
EN 6 0
EN 7 0
EN 8 0
EN 9 -6624
G-RIB:TYR-S2
EN 3 0
EN 4 0
EN 5 0
EN 6 -6031
EN 7 -3096
EN 8 -3795
EN 9 -3865
IU-MY:SER-CA
EN 3 0
EN 4 0
EN 5 0
EN 6 0
EN 7 -9293
EN 8 0
EN 9 0
IU-P:ILE-S1
EN 3 0
EN 4 0
EN 5 0
EN 6 0
EN 7 0
EN 8 0
EN 9 -8772
QUO-M6:LEU-S1
EN 3 0
EN 4 0
EN 5 0
EN 6 0
EN 7 0
EN 8 -17115
EN 9 0
G-R6:GLU-S1
EN 3 0
EN 4 0
EN 5 0
EN 6 -2511
EN 7 -1745
EN 8 -1275
EN 9 -1030
G-P:ASP-CA
EN 3 0
EN 4 0
EN 5 -3980
EN 6 1217
EN 7 -1643

EN 8 -2505
EN 9 -2563
GTP-M5:GLY-CA
EN 3 0
EN 4 0
EN 5 0
EN 6 0
EN 7 0
EN 8 0
EN 9 -5380
IU-RIB:PRO-S1
EN 3 0
EN 4 0
EN 5 0
EN 6 0
EN 7 -9107
EN 8 -7825
EN 9 0
IU-RIB:LYS-S1
EN 3 0
EN 4 0
EN 5 -10461
EN 6 0
EN 7 0
EN 8 -5439
EN 9 -6017
G-P:GLN-S1
EN 3 0
EN 4 -4221
EN 5 -6120
EN 6 -2563
EN 7 -3726
EN 8 -3444
EN 9 -3461
FHU-RIB:TYR-CA
EN 3 0
EN 4 0
EN 5 0
EN 6 -17115
EN 7 0
EN 8 0
EN 9 0
FHU-P:ALA-S1
EN 3 0
EN 4 0
EN 5 0
EN 6 0
EN 7 -8528
EN 8 0
EN 9 0
IU-RIB:ARG-S1
EN 3 0
EN 4 0
EN 5 0
EN 6 -11581
EN 7 -7711
EN 8 0
EN 9 0

G-R5:HIS-CA
EN 3 0
EN 4 0
EN 5 -7536
EN 6 -4769
EN 7 -4986
EN 8 -3616
EN 9 -4061
G-R5:GLN-S1
EN 3 0
EN 4 0
EN 5 -5012
EN 6 -1184
EN 7 -4429
EN 8 -3518
EN 9 -4119
C-RIB:ARG-S1
EN 3 0
EN 4 0
EN 5 -5828
EN 6 -5255
EN 7 -4537
EN 8 -4643
EN 9 -4451
U-Y:ALA-CA
EN 3 0
EN 4 0
EN 5 -6031
EN 6 -5182
EN 7 -2950
EN 8 -2822
EN 9 -3520
A-R5:ASN-S1
EN 3 0
EN 4 0
EN 5 -4290
EN 6 -4302
EN 7 -3698
EN 8 -4444
EN 9 -3655
C-P:LEU-S1
EN 3 0
EN 4 0
EN 5 -3614
EN 6 -3089
EN 7 -4417
EN 8 -2528
EN 9 -3133
G-RIB:ARG-CA
EN 3 0
EN 4 0
EN 5 -4566
EN 6 -5810
EN 7 -3650
EN 8 -3485
EN 9 -4036
C-Y:PHE-CA
EN 3 0

EN 4 0
EN 5 0
EN 6 -6352
EN 7 -3723
EN 8 -2625
EN 9 -4100
C31-MY:LEU-S2
EN 3 0
EN 4 -9819
EN 5 0
EN 6 0
EN 7 0
EN 8 0
EN 9 0
U-RIB:VAL-S1
EN 3 0
EN 4 0
EN 5 -7030
EN 6 -3583
EN 7 -1204
EN 8 -2617
EN 9 -2657
U34-RIB:TYR-S2
EN 3 0
EN 4 0
EN 5 0
EN 6 0
EN 7 0
EN 8 0
EN 9 -8528
G-R6:ALA-S1
EN 3 0
EN 4 -3182
EN 5 -1715
EN 6 -3003
EN 7 2300
EN 8 -3022
EN 9 -2173
U34-MY:VAL-CA
EN 3 0
EN 4 0
EN 5 0
EN 6 0
EN 7 0
EN 8 0
EN 9 -8949
G-P:ARG-S1
EN 3 0
EN 4 0
EN 5 -6528
EN 6 -5136
EN 7 -3942
EN 8 -4868
EN 9 -3944
C31-P:TYR-S2
EN 3 0
EN 4 0
EN 5 0

EN 6 -8949
EN 7 -7472
EN 8 0
EN 9 0
FHU-RIB:ASP-S1
EN 3 0
EN 4 0
EN 5 0
EN 6 0
EN 7 -9631
EN 8 0
EN 9 0
C-RIB:VAL-S1
EN 3 0
EN 4 0
EN 5 -6050
EN 6 -4891
EN 7 -4687
EN 8 -2841
EN 9 -2982
U-P:TRP-S2
EN 3 0
EN 4 0
EN 5 0
EN 6 -5727
EN 7 -6460
EN 8 -3389
EN 9 -3974
U-P:HIS-CA
EN 3 0
EN 4 0
EN 5 -4371
EN 6 0
EN 7 -4625
EN 8 -3289
EN 9 -5040
C31-RIB:GLU-S1
EN 3 0
EN 4 0
EN 5 0
EN 6 0
EN 7 -4784
EN 8 0
EN 9 0
C31-MY:GLU-S1
EN 3 0
EN 4 0
EN 5 0
EN 6 0
EN 7 0
EN 8 0
EN 9 -3808
DA-M6:ASN-CA
EN 3 0
EN 4 0
EN 5 0
EN 6 0
EN 7 0

EN 8 0
EN 9 -4405
G-R5:PHE-S2
EN 3 0
EN 4 -12294
EN 5 0
EN 6 -4966
EN 7 -3712
EN 8 -3562
EN 9 -3524
G-RIB:THR-CA
EN 3 0
EN 4 0
EN 5 -6020
EN 6 -4778
EN 7 -2900
EN 8 -2527
EN 9 -2733
A-RIB:ASP-CA
EN 3 0
EN 4 0
EN 5 -4009
EN 6 -2538
EN 7 -1994
EN 8 -1679
EN 9 -1955
DA-M5:THR-S1
EN 3 0
EN 4 0
EN 5 0
EN 6 0
EN 7 -6553
EN 8 0
EN 9 0
FHU-MY:PHE-S2
EN 3 0
EN 4 0
EN 5 0
EN 6 0
EN 7 0
EN 8 -10405
EN 9 0
IU-MY:ILE-S1
EN 3 0
EN 4 0
EN 5 0
EN 6 0
EN 7 0
EN 8 -9631
EN 9 0
5BU-P:ARG-CA
EN 3 0
EN 4 0
EN 5 0
EN 6 0
EN 7 0
EN 8 0
EN 9 -9293

U31-P:ASP-S1
EN 3 0
EN 4 -11671
EN 5 0
EN 6 0
EN 7 0
EN 8 -4733
EN 9 0
C-Y:CYS-S1
EN 3 0
EN 4 0
EN 5 -10144
EN 6 0
EN 7 0
EN 8 -3113
EN 9 -4374
A-P:CYS-S1
EN 3 0
EN 4 0
EN 5 -10245
EN 6 0
EN 7 0
EN 8 0
EN 9 -4955
G-R5:GLU-CA
EN 3 0
EN 4 0
EN 5 -4187
EN 6 0
EN 7 -1467
EN 8 -1697
EN 9 -127
A-P:ALA-CA
EN 3 0
EN 4 -3975
EN 5 -5899
EN 6 -3999
EN 7 -4121
EN 8 -3313
EN 9 -2822
U-P:LYS-S1
EN 3 0
EN 4 -2944
EN 5 -5707
EN 6 -5653
EN 7 -3663
EN 8 -3459
EN 9 -3439
FHU-RIB:SER-S1
EN 3 0
EN 4 0
EN 5 0
EN 6 0
EN 7 0
EN 8 -10489
EN 9 -9571
QUO-RIB:LEU-S2
EN 3 0

EN 4 0
EN 5 0
EN 6 0
EN 7 -17115
EN 8 0
EN 9 0
U34-P:ASN-S1
EN 3 0
EN 4 0
EN 5 -2722
EN 6 0
EN 7 0
EN 8 -8567
EN 9 -5584
C-P:TYR-CA
EN 3 0
EN 4 0
EN 5 -4818
EN 6 0
EN 7 -5302
EN 8 -2151
EN 9 -4381
FHU-P:ARG-S2
EN 3 0
EN 4 0
EN 5 0
EN 6 -8606
EN 7 0
EN 8 0
EN 9 -5036
C-P:LYS-S1
EN 3 0
EN 4 -2173
EN 5 -6228
EN 6 -5696
EN 7 -4163
EN 8 -2976
EN 9 -3147
U31-P:ARG-S1
EN 3 0
EN 4 0
EN 5 0
EN 6 0
EN 7 0
EN 8 0
EN 9 -4750
A-R5:ASP-CA
EN 3 0
EN 4 0
EN 5 -6904
EN 6 -883
EN 7 -2771
EN 8 -1899
EN 9 -1621
FMU-P:PHE-CA
EN 3 0
EN 4 0
EN 5 0

EN 6 -2722
EN 7 0
EN 8 0
EN 9 0
H2U-MY:ALA-CA
EN 3 0
EN 4 0
EN 5 0
EN 6 0
EN 7 0
EN 8 0
EN 9 -3926
U-RIB:LEU-CA
EN 3 0
EN 4 0
EN 5 -7944
EN 6 -6413
EN 7 -2486
EN 8 -2306
EN 9 -3357
QUO-M5:LEU-CA
EN 3 0
EN 4 0
EN 5 0
EN 6 0
EN 7 0
EN 8 -17115
EN 9 0
A-R6:MET-CA
EN 3 0
EN 4 0
EN 5 -8070
EN 6 -5088
EN 7 -5345
EN 8 -3074
EN 9 -4799
C31-RIB:PHE-S2
EN 3 0
EN 4 0
EN 5 -10405
EN 6 0
EN 7 -8236
EN 8 0
EN 9 0
U31-RIB:MET-S2
EN 3 0
EN 4 0
EN 5 0
EN 6 -10760
EN 7 0
EN 8 0
EN 9 0
U34-MY:ASN-S2
EN 3 0
EN 4 0
EN 5 0
EN 6 0
EN 7 0

EN 8 -7231
EN 9 0
U-P:PHE-S1
EN 3 0
EN 4 0
EN 5 -5185
EN 6 0
EN 7 -3618
EN 8 -4051
EN 9 -3021
A-P:MET-CA
EN 3 0
EN 4 0
EN 5 -8547
EN 6 -1854
EN 7 -3515
EN 8 -3579
EN 9 -4045
G-R5:ALA-CA
EN 3 0
EN 4 -8528
EN 5 -4727
EN 6 -3138
EN 7 -2220
EN 8 -2961
EN 9 -2355
A-R6:LYS-CA
EN 3 0
EN 4 0
EN 5 -5590
EN 6 -2757
EN 7 -2654
EN 8 -3581
EN 9 -2580
FHU-MY:LEU-S1
EN 3 0
EN 4 0
EN 5 0
EN 6 0
EN 7 -11671
EN 8 -7683
EN 9 -6970
FHU-MY:LEU-CA
EN 3 0
EN 4 0
EN 5 0
EN 6 -11174
EN 7 -9241
EN 8 -9401
EN 9 0
I-RIB:ALA-S1
EN 3 0
EN 4 0
EN 5 0
EN 6 0
EN 7 0
EN 8 -17115
EN 9 0

G-R6:GLU-S2
EN 3 0
EN 4 0
EN 5 -4441
EN 6 -2085
EN 7 -493
EN 8 -1131
EN 9 -720
H2U-P:LYS-S2
EN 3 0
EN 4 0
EN 5 0
EN 6 0
EN 7 0
EN 8 0
EN 9 -6122
A-P:TRP-S2
EN 3 0
EN 4 0
EN 5 0
EN 6 -5980
EN 7 -3398
EN 8 -2165
EN 9 -3377
FHU-RIB:ILE-CA
EN 3 0
EN 4 0
EN 5 0
EN 6 0
EN 7 0
EN 8 -10576
EN 9 0
U-Y:LEU-CA
EN 3 0
EN 4 0
EN 5 0
EN 6 0
EN 7 -5132
EN 8 -4709
EN 9 -3624
G-RIB:ASP-S2
EN 3 0
EN 4 0
EN 5 -4335
EN 6 -1245
EN 7 -1044
EN 8 -1785
EN 9 -2351
U-P:HIS-S2
EN 3 0
EN 4 0
EN 5 -3679
EN 6 -4368
EN 7 -4416
EN 8 -5052
EN 9 -3821
G-RIB:HIS-S2
EN 3 0

EN 4 0
EN 5 -6633
EN 6 -5629
EN 7 -4019
EN 8 -4084
EN 9 -3192
G-R5:GLU-S1
EN 3 0
EN 4 0
EN 5 -3812
EN 6 -248
EN 7 -1496
EN 8 459
EN 9 -1240
C-P:SER-CA
EN 3 0
EN 4 -5911
EN 5 -7573
EN 6 -4243
EN 7 -3996
EN 8 -3259
EN 9 -3546
A-R6:ASN-S2
EN 3 0
EN 4 0
EN 5 -3816
EN 6 -5569
EN 7 -4092
EN 8 -3398
EN 9 -3480
G-P:ASP-S2
EN 3 0
EN 4 0
EN 5 -97
EN 6 -1549
EN 7 -2391
EN 8 -2093
EN 9 -2721
G-R6:ASP-S1
EN 3 0
EN 4 0
EN 5 -3238
EN 6 -3095
EN 7 -3144
EN 8 -1614
EN 9 -2075
G-R6:TYR-CA
EN 3 0
EN 4 0
EN 5 0
EN 6 -7960
EN 7 -4670
EN 8 -2092
EN 9 -3250
C-Y:ASP-S2
EN 3 0
EN 4 -4728
EN 5 -4945

EN 6 -2255
EN 7 -3219
EN 8 -2521
EN 9 -1852
C-Y:SER-CA
EN 3 0
EN 4 0
EN 5 -4145
EN 6 -5308
EN 7 -4653
EN 8 -3983
EN 9 -3505
C-RIB:ASN-S1
EN 3 0
EN 4 -6957
EN 5 -3307
EN 6 -4265
EN 7 -3925
EN 8 -3402
EN 9 -3704
C31-P:PHE-S1
EN 3 0
EN 4 0
EN 5 0
EN 6 0
EN 7 0
EN 8 -7325
EN 9 0
FHU-MY:ARG-CA
EN 3 0
EN 4 0
EN 5 0
EN 6 -11963
EN 7 -8236
EN 8 0
EN 9 -9651
C-P:SER-S1
EN 3 0
EN 4 -6902
EN 5 -6074
EN 6 -4016
EN 7 -3056
EN 8 -3353
EN 9 -3817
G-R6:CYS-S1
EN 3 0
EN 4 0
EN 5 0
EN 6 -5036
EN 7 -3371
EN 8 0
EN 9 -3529
A-RIB:TRP-S1
EN 3 0
EN 4 0
EN 5 -5274
EN 6 -2823
EN 7 -4576

EN 8 -4693
EN 9 -4201
U31-MY:VAL-CA
EN 3 0
EN 4 0
EN 5 0
EN 6 0
EN 7 0
EN 8 0
EN 9 -5537
G-R6:ASP-CA
EN 3 0
EN 4 0
EN 5 0
EN 6 -2541
EN 7 -3463
EN 8 -1856
EN 9 -2022
G-R5:CYS-S1
EN 3 0
EN 4 0
EN 5 0
EN 6 0
EN 7 -3881
EN 8 -2356
EN 9 -730
QUO-M6:LYS-S1
EN 3 0
EN 4 0
EN 5 0
EN 6 0
EN 7 0
EN 8 0
EN 9 -17115
A-R5:TYR-S2
EN 3 0
EN 4 -16110
EN 5 -9755
EN 6 -6796
EN 7 -6576
EN 8 -5754
EN 9 -3612
U34-RIB:ASN-CA
EN 3 0
EN 4 0
EN 5 0
EN 6 0
EN 7 0
EN 8 -11410
EN 9 -9382
A-P:ASN-S2
EN 3 0
EN 4 0
EN 5 -5975
EN 6 -2938
EN 7 -3227
EN 8 -3444
EN 9 -3280

H2U-MY:THR-S1
EN 3 0
EN 4 0
EN 5 0
EN 6 0
EN 7 0
EN 8 0
EN 9 -5584
U-Y:GLY-CA
EN 3 0
EN 4 0
EN 5 -4198
EN 6 -5266
EN 7 -865
EN 8 -3544
EN 9 -3945
C31-RIB:GLU-S2
EN 3 0
EN 4 0
EN 5 0
EN 6 -6320
EN 7 0
EN 8 0
EN 9 -2823
G-R5:LEU-S1
EN 3 0
EN 4 0
EN 5 -8875
EN 6 -6212
EN 7 -3123
EN 8 -3704
EN 9 -3150
A-P:VAL-CA
EN 3 0
EN 4 0
EN 5 -7270
EN 6 -2307
EN 7 -3367
EN 8 -3368
EN 9 -3005
5BU-P:PRO-S1
EN 3 0
EN 4 0
EN 5 0
EN 6 -12294
EN 7 0
EN 8 0
EN 9 0
G-R5:TYR-CA
EN 3 0
EN 4 0
EN 5 -7201
EN 6 0
EN 7 -6320
EN 8 -2359
EN 9 -4030
U34-RIB:ASN-S1
EN 3 0

EN 4 0
EN 5 0
EN 6 0
EN 7 -13680
EN 8 -7054
EN 9 0
IU-RIB:GLN-S1
EN 3 0
EN 4 0
EN 5 0
EN 6 0
EN 7 0
EN 8 0
EN 9 -7040
U34-MY:PHE-CA
EN 3 0
EN 4 0
EN 5 0
EN 6 0
EN 7 0
EN 8 0
EN 9 -10095
U31-MY:GLN-S1
EN 3 0
EN 4 0
EN 5 0
EN 6 0
EN 7 -5705
EN 8 0
EN 9 0
FHU-MY:ASP-S1
EN 3 0
EN 4 0
EN 5 0
EN 6 -11174
EN 7 0
EN 8 0
EN 9 -8489
C-Y:LYS-S1
EN 3 0
EN 4 0
EN 5 -4099
EN 6 -4056
EN 7 -3979
EN 8 -2108
EN 9 -3613
A-R5:SER-CA
EN 3 0
EN 4 0
EN 5 0
EN 6 -3373
EN 7 -4664
EN 8 -3608
EN 9 -3716
C-RIB:PHE-CA
EN 3 0
EN 4 0
EN 5 0

EN 6 -1585
EN 7 -3079
EN 8 -3367
EN 9 -2456
H2U-P:GLU-S2
EN 3 0
EN 4 0
EN 5 0
EN 6 0
EN 7 -6352
EN 8 0
EN 9 -4717
A-P:HIS-S1
EN 3 0
EN 4 0
EN 5 -5302
EN 6 -3318
EN 7 -4886
EN 8 -5042
EN 9 -3910
FMU-MY:ARG-S1
EN 3 0
EN 4 0
EN 5 0
EN 6 0
EN 7 0
EN 8 -8378
EN 9 0
FMU-RIB:CYS-S1
EN 3 0
EN 4 0
EN 5 -2722
EN 6 0
EN 7 0
EN 8 0
EN 9 0
A-RIB:VAL-S1
EN 3 0
EN 4 0
EN 5 -5275
EN 6 -5281
EN 7 -4867
EN 8 -3053
EN 9 -3039
H2U-RIB:LEU-S2
EN 3 0
EN 4 0
EN 5 0
EN 6 0
EN 7 0
EN 8 0
EN 9 -4405
G-P:GLY-CA
EN 3 0
EN 4 -4121
EN 5 -5909
EN 6 -1406
EN 7 -3131

EN 8 -3273
EN 9 -3106
G-P:ARG-CA
EN 3 0
EN 4 0
EN 5 -4953
EN 6 -4258
EN 7 -4335
EN 8 -4725
EN 9 -4323
U-Y:ALA-S1
EN 3 0
EN 4 0
EN 5 -5680
EN 6 -4516
EN 7 -3262
EN 8 -1382
EN 9 -3058
H2U-RIB:PRO-S1
EN 3 0
EN 4 0
EN 5 0
EN 6 0
EN 7 0
EN 8 -6417
EN 9 0
A-R5:GLY-CA
EN 3 0
EN 4 -5662
EN 5 -7365
EN 6 -3971
EN 7 -4407
EN 8 -3668
EN 9 -4233
U34-P:ASN-S2
EN 3 0
EN 4 0
EN 5 -17115
EN 6 0
EN 7 -6889
EN 8 0
EN 9 -5631
DA-M6:LYS-S2
EN 3 0
EN 4 -11671
EN 5 0
EN 6 0
EN 7 0
EN 8 0
EN 9 0
U-P:VAL-S1
EN 3 0
EN 4 0
EN 5 0
EN 6 -3762
EN 7 -3676
EN 8 -2998
EN 9 -3195

C-P:ARG-S1
EN 3 0
EN 4 -2490
EN 5 -8042
EN 6 -6254
EN 7 -3680
EN 8 -4675
EN 9 -4294
C-Y:VAL-S1
EN 3 0
EN 4 0
EN 5 -7607
EN 6 -6139
EN 7 -279
EN 8 -3185
EN 9 -3248
DA-RIB:HIS-CA
EN 3 0
EN 4 0
EN 5 0
EN 6 0
EN 7 -8168
EN 8 -6929
EN 9 0
G-P:LEU-S2
EN 3 0
EN 4 -3385
EN 5 -3693
EN 6 -4359
EN 7 -2131
EN 8 -3278
EN 9 -3067
GTP-RIB:SER-S1
EN 3 0
EN 4 0
EN 5 0
EN 6 0
EN 7 -5966
EN 8 0
EN 9 -4127
FMU-MY:SER-S1
EN 3 0
EN 4 0
EN 5 0
EN 6 0
EN 7 0
EN 8 -9692
EN 9 0
H2U-RIB:PRO-CA
EN 3 0
EN 4 0
EN 5 0
EN 6 0
EN 7 0
EN 8 0
EN 9 -5132
G-P:PHE-S2
EN 3 0

EN 4 0
EN 5 0
EN 6 -4833
EN 7 -4780
EN 8 -2293
EN 9 -2950
A-R6:PHE-S1
EN 3 0
EN 4 -11812
EN 5 -7746
EN 6 0
EN 7 -1483
EN 8 -4199
EN 9 -3277
G-R5:HIS-S1
EN 3 0
EN 4 0
EN 5 -8196
EN 6 -6097
EN 7 -4928
EN 8 -3714
EN 9 -4533
A-R5:HIS-S2
EN 3 0
EN 4 -12414
EN 5 -8414
EN 6 -5476
EN 7 -5761
EN 8 -4499
EN 9 -4624
5BU-P:ILE-S1
EN 3 0
EN 4 0
EN 5 0
EN 6 -17115
EN 7 -15398
EN 8 0
EN 9 0
4SU-P:THR-S1
EN 3 0
EN 4 0
EN 5 0
EN 6 0
EN 7 0
EN 8 -2722
EN 9 0
FMU-MY:PHE-S1
EN 3 0
EN 4 0
EN 5 0
EN 6 0
EN 7 0
EN 8 -10760
EN 9 0
I-RIB:TRP-S2
EN 3 0
EN 4 0
EN 5 0

EN 6 0
EN 7 -1717
EN 8 0
EN 9 0
A-R6:HIS-S2
EN 3 0
EN 4 -12178
EN 5 -5560
EN 6 -5884
EN 7 -4875
EN 8 -4226
EN 9 -3674
U-RIB:ALA-S1
EN 3 0
EN 4 -2772
EN 5 -4032
EN 6 -4040
EN 7 -3794
EN 8 -3306
EN 9 -3615
A-R6:ILE-CA
EN 3 0
EN 4 0
EN 5 -5643
EN 6 -4699
EN 7 -1351
EN 8 -2787
EN 9 -3195
U31-P:ASN-S2
EN 3 0
EN 4 0
EN 5 0
EN 6 -7629
EN 7 0
EN 8 0
EN 9 0
G-R6:THR-CA
EN 3 0
EN 4 0
EN 5 0
EN 6 -2437
EN 7 -2434
EN 8 -2905
EN 9 -2493
A-P:LEU-S2
EN 3 0
EN 4 0
EN 5 -4509
EN 6 -4824
EN 7 -1834
EN 8 -3184
EN 9 -2936
FMU-MY:HIS-S1
EN 3 0
EN 4 0
EN 5 0
EN 6 0
EN 7 0

EN 8 0
EN 9 -11174
G-RIB:MET-CA
EN 3 0
EN 4 -10405
EN 5 -5305
EN 6 0
EN 7 -4411
EN 8 -4262
EN 9 -2980
IU-RIB:HIS-S1
EN 3 0
EN 4 0
EN 5 0
EN 6 0
EN 7 -13334
EN 8 0
EN 9 0
G-R6:LYS-S2
EN 3 0
EN 4 -2088
EN 5 -3872
EN 6 -4699
EN 7 -1744
EN 8 -2842
EN 9 -2721
FHU-RIB:LEU-S2
EN 3 0
EN 4 0
EN 5 0
EN 6 0
EN 7 -9692
EN 8 -9631
EN 9 -8647
FMU-MY:ASN-S1
EN 3 0
EN 4 0
EN 5 0
EN 6 0
EN 7 0
EN 8 0
EN 9 -10760
A-R6:ILE-S1
EN 3 0
EN 4 0
EN 5 -5517
EN 6 -1218
EN 7 569
EN 8 -4221
EN 9 -3444
A-RIB:ALA-S1
EN 3 0
EN 4 -2379
EN 5 -3784
EN 6 -3685
EN 7 -3808
EN 8 -2694
EN 9 -3135

H2U-P:ASN-S1
EN 3 0
EN 4 0
EN 5 0
EN 6 0
EN 7 -8306
EN 8 -9241
EN 9 0
C-RIB:TRP-S2
EN 3 0
EN 4 0
EN 5 -6849
EN 6 -6338
EN 7 -4727
EN 8 -2824
EN 9 -3472
H2U-RIB:ASN-S1
EN 3 0
EN 4 0
EN 5 0
EN 6 0
EN 7 0
EN 8 0
EN 9 -7602
QUO-M6:LEU-CA
EN 3 0
EN 4 0
EN 5 0
EN 6 0
EN 7 0
EN 8 0
EN 9 -15398
IU-RIB:HIS-CA
EN 3 0
EN 4 0
EN 5 0
EN 6 -12477
EN 7 -11764
EN 8 0
EN 9 0
U31-P:SER-CA
EN 3 0
EN 4 0
EN 5 0
EN 6 0
EN 7 0
EN 8 0
EN 9 -6050
A-RIB:ARG-S1
EN 3 0
EN 4 0
EN 5 -5021
EN 6 -5556
EN 7 -4732
EN 8 -4976
EN 9 -4840
A-P:PHE-S1
EN 3 0

EN 4 0
EN 5 -6434
EN 6 -2185
EN 7 -4451
EN 8 -3576
EN 9 -4405
U31-MY:PHE-CA
EN 3 0
EN 4 0
EN 5 0
EN 6 0
EN 7 -6970
EN 8 0
EN 9 0
U34-P:SER-CA
EN 3 0
EN 4 0
EN 5 0
EN 6 0
EN 7 0
EN 8 -4818
EN 9 -3328
U-P:TYR-CA
EN 3 0
EN 4 0
EN 5 0
EN 6 -2270
EN 7 -1947
EN 8 -430
EN 9 -4100
U31-P:HIS-CA
EN 3 0
EN 4 0
EN 5 0
EN 6 0
EN 7 -9241
EN 8 0
EN 9 0
I-RIB:GLY-CA
EN 3 0
EN 4 0
EN 5 0
EN 6 0
EN 7 0
EN 8 0
EN 9 -17115
C31-P:ASN-S1
EN 3 0
EN 4 0
EN 5 0
EN 6 0
EN 7 0
EN 8 -4556
EN 9 0
G-R6:MET-CA
EN 3 0
EN 4 0
EN 5 -7097

EN 6 -5674
EN 7 -5373
EN 8 -5223
EN 9 -2845
C-Y:GLY-CA
EN 3 0
EN 4 -5975
EN 5 -4991
EN 6 -4238
EN 7 -3470
EN 8 -3450
EN 9 -4223
A-RIB:TYR-S2
EN 3 0
EN 4 0
EN 5 -10518
EN 6 -7503
EN 7 -1923
EN 8 -3064
EN 9 -4237
U-Y:PHE-CA
EN 3 0
EN 4 0
EN 5 0
EN 6 -6577
EN 7 -6877
EN 8 -4141
EN 9 -4618
DA-M5:ASN-S1
EN 3 0
EN 4 0
EN 5 0
EN 6 0
EN 7 0
EN 8 -5093
EN 9 0
U31-MY:ASP-CA
EN 3 0
EN 4 0
EN 5 0
EN 6 -8528
EN 7 0
EN 8 -4221
EN 9 0
FMU-MY:ASP-S2
EN 3 0
EN 4 0
EN 5 0
EN 6 -11174
EN 7 0
EN 8 0
EN 9 0
IU-P:LYS-S2
EN 3 0
EN 4 0
EN 5 -8949
EN 6 0
EN 7 0

EN 8 0
EN 9 -4571
G-R5:PRO-S1
EN 3 0
EN 4 0
EN 5 -6372
EN 6 -1350
EN 7 -3733
EN 8 -2475
EN 9 -3714
H2U-RIB:LYS-S1
EN 3 0
EN 4 0
EN 5 0
EN 6 0
EN 7 0
EN 8 0
EN 9 -4961
FMU-RIB:GLN-S2
EN 3 0
EN 4 0
EN 5 0
EN 6 0
EN 7 0
EN 8 -9953
EN 9 0
H2U-RIB:TRP-S2
EN 3 0
EN 4 0
EN 5 0
EN 6 0
EN 7 -17115
EN 8 0
EN 9 0
A-R6:GLU-S1
EN 3 0
EN 4 0
EN 5 -3420
EN 6 -2305
EN 7 -1541
EN 8 -1270
EN 9 -1492
IU-RIB:LEU-CA
EN 3 0
EN 4 0
EN 5 0
EN 6 -13127
EN 7 0
EN 8 0
EN 9 -6879
U-P:ARG-S2
EN 3 0
EN 4 0
EN 5 -7815
EN 6 -5084
EN 7 -2978
EN 8 -4029
EN 9 -4265

G-R6:GLU-CA
EN 3 0
EN 4 0
EN 5 0
EN 6 367
EN 7 -1722
EN 8 -1775
EN 9 -1435
DA-RIB:ASN-S2
EN 3 0
EN 4 0
EN 5 0
EN 6 -7373
EN 7 0
EN 8 0
EN 9 0
U-Y:ILE-CA
EN 3 0
EN 4 0
EN 5 0
EN 6 0
EN 7 -3770
EN 8 -2714
EN 9 -3143
U-RIB:THR-S1
EN 3 0
EN 4 0
EN 5 -5614
EN 6 -3612
EN 7 -3039
EN 8 -2718
EN 9 -3166
G-P:PRO-S1
EN 3 0
EN 4 0
EN 5 -5267
EN 6 -3283
EN 7 -2857
EN 8 -3499
EN 9 -3618
U-P:ASP-S1
EN 3 0
EN 4 -3428
EN 5 -2768
EN 6 -2090
EN 7 -738
EN 8 -1199
EN 9 -1585
G-RIB:PHE-CA
EN 3 0
EN 4 0
EN 5 -5418
EN 6 -6505
EN 7 -3194
EN 8 -4557
EN 9 -2725
FHU-RIB:LEU-S1
EN 3 0

EN 4 0
EN 5 0
EN 6 0
EN 7 -11410
EN 8 -8168
EN 9 0
A-P:LEU-CA
EN 3 0
EN 4 0
EN 5 -5845
EN 6 -4154
EN 7 -3008
EN 8 -2423
EN 9 -3243
DA-M5:LYS-S1
EN 3 0
EN 4 0
EN 5 0
EN 6 0
EN 7 0
EN 8 -6137
EN 9 0
QUO-RIB:ASP-S1
EN 3 0
EN 4 0
EN 5 0
EN 6 0
EN 7 0
EN 8 0
EN 9 -12675
A-R6:SER-S1
EN 3 0
EN 4 0
EN 5 -6412
EN 6 -3431
EN 7 -2876
EN 8 -3288
EN 9 -2898
A-R5:ASN-CA
EN 3 0
EN 4 0
EN 5 0
EN 6 -2562
EN 7 -4382
EN 8 -4173
EN 9 -3526
A-P:GLU-S1
EN 3 0
EN 4 0
EN 5 1011
EN 6 1760
EN 7 344
EN 8 -958
EN 9 -1900
DA-M5:ASN-S2
EN 3 0
EN 4 0
EN 5 0

EN 6 0
EN 7 -5755
EN 8 0
EN 9 0
A-P:GLN-S2
EN 3 0
EN 4 -3067
EN 5 -5461
EN 6 -3640
EN 7 -2238
EN 8 -4165
EN 9 -3510
FMU-MY:PHE-S2
EN 3 0
EN 4 0
EN 5 0
EN 6 -12675
EN 7 -14393
EN 8 0
EN 9 0
IU-RIB:HIS-S2
EN 3 0
EN 4 0
EN 5 0
EN 6 0
EN 7 0
EN 8 -9456
EN 9 -10824
FHU-RIB:ARG-S2
EN 3 0
EN 4 0
EN 5 0
EN 6 0
EN 7 -10207
EN 8 0
EN 9 -5112
QUO-M6:ASP-CA
EN 3 0
EN 4 0
EN 5 0
EN 6 0
EN 7 0
EN 8 -15398
EN 9 0
U-P:MET-S1
EN 3 0
EN 4 0
EN 5 -3793
EN 6 -3364
EN 7 -2882
EN 8 -4844
EN 9 -4226
C31-P:MET-S2
EN 3 0
EN 4 0
EN 5 0
EN 6 0
EN 7 0

EN 8 -5584
EN 9 0
QUO-M6:PHE-S1
EN 3 0
EN 4 0
EN 5 0
EN 6 0
EN 7 -3434
EN 8 0
EN 9 -3434
G-R6:TYR-S1
EN 3 0
EN 4 0
EN 5 -8197
EN 6 -5591
EN 7 -6589
EN 8 665
EN 9 -3933
U-RIB:SER-S1
EN 3 0
EN 4 -5239
EN 5 -4649
EN 6 -4247
EN 7 -3469
EN 8 -2161
EN 9 -2992
C31-P:PHE-CA
EN 3 0
EN 4 0
EN 5 0
EN 6 0
EN 7 0
EN 8 0
EN 9 -7278
GTP-RIB:THR-CA
EN 3 0
EN 4 0
EN 5 0
EN 6 0
EN 7 0
EN 8 0
EN 9 -4141
FHU-MY:SER-S1
EN 3 0
EN 4 0
EN 5 0
EN 6 0
EN 7 0
EN 8 0
EN 9 -7373
U31-P:MET-S1
EN 3 0
EN 4 0
EN 5 0
EN 6 -11410
EN 7 -7854
EN 8 0
EN 9 0

C-P:ILE-S1
EN 3 0
EN 4 0
EN 5 -3495
EN 6 -2805
EN 7 -3489
EN 8 -3760
EN 9 -3499
U31-P:TYR-S1
EN 3 0
EN 4 0
EN 5 0
EN 6 0
EN 7 0
EN 8 0
EN 9 -7141
C31-P:THR-CA
EN 3 0
EN 4 0
EN 5 0
EN 6 0
EN 7 -9953
EN 8 0
EN 9 -6227
A-R5:ASP-S2
EN 3 0
EN 4 0
EN 5 -4272
EN 6 -1499
EN 7 -1660
EN 8 -1858
EN 9 -1882
FHU-MY:TYR-S2
EN 3 0
EN 4 0
EN 5 0
EN 6 -12967
EN 7 -9241
EN 8 0
EN 9 0
FHU-MY:SER-CA
EN 3 0
EN 4 0
EN 5 0
EN 6 0
EN 7 0
EN 8 0
EN 9 -9090
FMU-RIB:HIS-S1
EN 3 0
EN 4 0
EN 5 0
EN 6 0
EN 7 0
EN 8 -12294
EN 9 0
U31-MY:MET-CA
EN 3 0

EN 4 0
EN 5 0
EN 6 -9953
EN 7 0
EN 8 0
EN 9 0
G-RIB:TRP-CA
EN 3 0
EN 4 0
EN 5 -7186
EN 6 -6962
EN 7 -4473
EN 8 -4181
EN 9 -3527
A-R6:THR-CA
EN 3 0
EN 4 0
EN 5 -3941
EN 6 -4730
EN 7 -4854
EN 8 -1548
EN 9 -3386
FMU-RIB:PHE-S1
EN 3 0
EN 4 0
EN 5 0
EN 6 0
EN 7 0
EN 8 -2722
EN 9 -11174
U31-RIB:TYR-S1
EN 3 0
EN 4 0
EN 5 0
EN 6 0
EN 7 0
EN 8 -6734
EN 9 0
H2U-P:GLU-CA
EN 3 0
EN 4 0
EN 5 0
EN 6 0
EN 7 0
EN 8 0
EN 9 -5232
FMU-MY:ARG-CA
EN 3 0
EN 4 0
EN 5 0
EN 6 0
EN 7 0
EN 8 -9241
EN 9 0
FHU-RIB:ASP-S2
EN 3 0
EN 4 0
EN 5 0

EN 6 -11063
EN 7 0
EN 8 0
EN 9 0
C31-P:ASP-S2
EN 3 0
EN 4 0
EN 5 -9139
EN 6 -7422
EN 7 -5491
EN 8 0
EN 9 0
FMU-P:VAL-S1
EN 3 0
EN 4 0
EN 5 0
EN 6 0
EN 7 0
EN 8 0
EN 9 -11671
C31-MY:GLN-S2
EN 3 0
EN 4 0
EN 5 0
EN 6 0
EN 7 -7796
EN 8 0
EN 9 0
QUO-P:PHE-S2
EN 3 0
EN 4 0
EN 5 0
EN 6 0
EN 7 0
EN 8 0
EN 9 -17115
DA-RIB:ASP-S1
EN 3 0
EN 4 0
EN 5 0
EN 6 -7278
EN 7 0
EN 8 0
EN 9 0
DA-M6:HIS-S1
EN 3 0
EN 4 0
EN 5 0
EN 6 -8236
EN 7 0
EN 8 0
EN 9 0
5BU-MY:ARG-S2
EN 3 0
EN 4 0
EN 5 0
EN 6 0
EN 7 0

EN 8 0
EN 9 -9139
FMU-MY:MET-CA
EN 3 0
EN 4 0
EN 5 0
EN 6 0
EN 7 0
EN 8 0
EN 9 -13127
FMU-RIB:ASP-S2
EN 3 0
EN 4 0
EN 5 0
EN 6 0
EN 7 0
EN 8 0
EN 9 -8236
IU-MY:ALA-CA
EN 3 0
EN 4 0
EN 5 -17115
EN 6 0
EN 7 0
EN 8 0
EN 9 -10728
FMU-MY:PRO-S1
EN 3 0
EN 4 0
EN 5 0
EN 6 0
EN 7 0
EN 8 0
EN 9 -9953
C-RIB:CYS-CA
EN 3 0
EN 4 0
EN 5 0
EN 6 -5730
EN 7 0
EN 8 -5529
EN 9 -4409
IU-P:ARG-CA
EN 3 0
EN 4 0
EN 5 0
EN 6 0
EN 7 -9139
EN 8 0
EN 9 -6970
G-RIB:PRO-S1
EN 3 0
EN 4 0
EN 5 -4893
EN 6 -4663
EN 7 -4307
EN 8 -2857
EN 9 -2495

FMU-RIB:GLN-S1
EN 3 0
EN 4 0
EN 5 0
EN 6 0
EN 7 0
EN 8 0
EN 9 -8452
H2U-MY:ARG-S2
EN 3 0
EN 4 0
EN 5 -11963
EN 6 0
EN 7 0
EN 8 -4818
EN 9 -5980
A-RIB:ILE-CA
EN 3 0
EN 4 0
EN 5 -7656
EN 6 -3990
EN 7 -3612
EN 8 -2948
EN 9 -3647
G-R5:LEU-CA
EN 3 0
EN 4 0
EN 5 -7575
EN 6 -6124
EN 7 -3736
EN 8 -5185
EN 9 -3600
C31-RIB:ASP-S1
EN 3 0
EN 4 0
EN 5 0
EN 6 0
EN 7 0
EN 8 0
EN 9 -3395
G-R5:ALA-S1
EN 3 0
EN 4 -4157
EN 5 -3434
EN 6 -2795
EN 7 -2313
EN 8 -2058
EN 9 -2790
GTP-M6:SER-S1
EN 3 0
EN 4 0
EN 5 0
EN 6 0
EN 7 0
EN 8 0
EN 9 -6624
QUO-RIB:GLN-S2
EN 3 0

EN 4 0
EN 5 0
EN 6 0
EN 7 0
EN 8 0
EN 9 -17115
FMU-RIB:HIS-S2
EN 3 0
EN 4 0
EN 5 0
EN 6 0
EN 7 0
EN 8 -11410
EN 9 0
FHU-P:ARG-S1
EN 3 0
EN 4 0
EN 5 0
EN 6 0
EN 7 0
EN 8 -5884
EN 9 -5358
FHU-MY:ALA-CA
EN 3 0
EN 4 0
EN 5 0
EN 6 0
EN 7 0
EN 8 -10576
EN 9 0
G-R5:LYS-S2
EN 3 0
EN 4 -3917
EN 5 -6414
EN 6 -3872
EN 7 -3215
EN 8 -2585
EN 9 -3791
U-RIB:TRP-CA
EN 3 0
EN 4 0
EN 5 0
EN 6 -3862
EN 7 -4025
EN 8 -5235
EN 9 -4393
FHU-MY:THR-CA
EN 3 0
EN 4 0
EN 5 0
EN 6 0
EN 7 -7914
EN 8 -9631
EN 9 -6553
U34-P:TYR-S2
EN 3 0
EN 4 0
EN 5 0

EN 6 -2722
EN 7 0
EN 8 0
EN 9 0
DA-M5:GLU-S2
EN 3 0
EN 4 0
EN 5 0
EN 6 0
EN 7 -5537
EN 8 0
EN 9 0
GTP-RIB:ASN-S1
EN 3 0
EN 4 0
EN 5 -11410
EN 6 0
EN 7 0
EN 8 0
EN 9 0
U31-P:LEU-S2
EN 3 0
EN 4 0
EN 5 0
EN 6 0
EN 7 0
EN 8 0
EN 9 -4332
FMU-P:GLN-CA
EN 3 0
EN 4 0
EN 5 0
EN 6 0
EN 7 0
EN 8 0
EN 9 -10958
IU-MY:ILE-CA
EN 3 0
EN 4 0
EN 5 0
EN 6 0
EN 7 0
EN 8 -9293
EN 9 -7934
A-RIB:GLN-CA
EN 3 0
EN 4 0
EN 5 -7455
EN 6 -3943
EN 7 -3028
EN 8 -3833
EN 9 -3001
C31-P:LEU-S2
EN 3 0
EN 4 0
EN 5 0
EN 6 0
EN 7 0

EN 8 0
EN 9 -3366
G-RIB:LEU-S1
EN 3 0
EN 4 0
EN 5 -6759
EN 6 -3745
EN 7 -4047
EN 8 -3660
EN 9 -2708
DA-RIB:ASP-S2
EN 3 0
EN 4 0
EN 5 0
EN 6 0
EN 7 0
EN 8 -3950
EN 9 0
5BU-MY:PRO-CA
EN 3 0
EN 4 0
EN 5 0
EN 6 0
EN 7 0
EN 8 0
EN 9 -13127
C-RIB:TYR-S2
EN 3 0
EN 4 0
EN 5 -4127
EN 6 -5811
EN 7 -4814
EN 8 -2748
EN 9 -3766
IU-RIB:ILE-S1
EN 3 0
EN 4 0
EN 5 0
EN 6 0
EN 7 -10666
EN 8 -8918
EN 9 0
C-P:CYS-S1
EN 3 0
EN 4 0
EN 5 0
EN 6 -4012
EN 7 -2490
EN 8 -3759
EN 9 -3660
C31-P:ASP-CA
EN 3 0
EN 4 0
EN 5 0
EN 6 0
EN 7 -6553
EN 8 -5656
EN 9 -4000

U34-P:SER-S1
EN 3 0
EN 4 0
EN 5 0
EN 6 0
EN 7 0
EN 8 0
EN 9 -6098
U31-RIB:GLN-S1
EN 3 0
EN 4 0
EN 5 -17115
EN 6 0
EN 7 0
EN 8 0
EN 9 0
U-P:TYR-S1
EN 3 0
EN 4 0
EN 5 0
EN 6 -2454
EN 7 -210
EN 8 -2701
EN 9 -3702
A-R6:PHE-S2
EN 3 0
EN 4 -9456
EN 5 -7777
EN 6 -6688
EN 7 -3287
EN 8 -3442
EN 9 -3989
FHU-RIB:PRO-CA
EN 3 0
EN 4 0
EN 5 0
EN 6 0
EN 7 0
EN 8 0
EN 9 -7097
A-R6:ARG-CA
EN 3 0
EN 4 0
EN 5 -6040
EN 6 -4504
EN 7 -4591
EN 8 -3958
EN 9 -4069
C-P:TRP-S2
EN 3 0
EN 4 0
EN 5 -7012
EN 6 -4790
EN 7 -4714
EN 8 -2943
EN 9 -3923
G-R6:HIS-S1
EN 3 0

EN 4 0
EN 5 -5959
EN 6 -6511
EN 7 -3978
EN 8 -4809
EN 9 -4182
C31-RIB:ASP-S2
EN 3 0
EN 4 0
EN 5 0
EN 6 0
EN 7 0
EN 8 0
EN 9 -5074
DA-M5:HIS-S1
EN 3 0
EN 4 0
EN 5 0
EN 6 -8859
EN 7 0
EN 8 0
EN 9 0
FHU-RIB:PRO-S1
EN 3 0
EN 4 0
EN 5 0
EN 6 0
EN 7 0
EN 8 -7141
EN 9 0
A-RIB:HIS-S1
EN 3 0
EN 4 0
EN 5 -7705
EN 6 -6829
EN 7 -5370
EN 8 -4945
EN 9 -3154
U31-RIB:ASN-CA
EN 3 0
EN 4 0
EN 5 0
EN 6 0
EN 7 0
EN 8 -5337
EN 9 0
C-RIB:LEU-S1
EN 3 0
EN 4 0
EN 5 -4719
EN 6 -5167
EN 7 -4109
EN 8 -4422
EN 9 -3991
H2U-RIB:GLU-S2
EN 3 0
EN 4 0
EN 5 0

EN 6 0
EN 7 0
EN 8 -4871
EN 9 0
IU-MY:LEU-S2
EN 3 0
EN 4 0
EN 5 0
EN 6 0
EN 7 -10169
EN 8 0
EN 9 0
U34-MY:ASP-CA
EN 3 0
EN 4 0
EN 5 0
EN 6 0
EN 7 0
EN 8 0
EN 9 -7854
QUO-M6:GLU-S1
EN 3 0
EN 4 0
EN 5 0
EN 6 0
EN 7 -17115
EN 8 0
EN 9 0
U-RIB:ASN-CA
EN 3 0
EN 4 0
EN 5 -4961
EN 6 -5261
EN 7 -4618
EN 8 -3421
EN 9 -3722
G-P:TRP-S2
EN 3 0
EN 4 0
EN 5 0
EN 6 -4646
EN 7 -4223
EN 8 -4224
EN 9 -4749
U31-P:ARG-CA
EN 3 0
EN 4 0
EN 5 0
EN 6 0
EN 7 0
EN 8 0
EN 9 -5112
A-R6:TYR-S1
EN 3 0
EN 4 -13680
EN 5 -6849
EN 6 -6960
EN 7 -5902

EN 8 -5883
EN 9 -4216
DA-M6:TYR-S2
EN 3 0
EN 4 0
EN 5 0
EN 6 0
EN 7 0
EN 8 0
EN 9 -6196
FHU-RIB:TYR-S2
EN 3 0
EN 4 0
EN 5 -4439
EN 6 0
EN 7 -10760
EN 8 -9819
EN 9 0
G-R5:ASN-S2
EN 3 0
EN 4 0
EN 5 -6194
EN 6 -4642
EN 7 -3867
EN 8 -3346
EN 9 -3327
IU-P:HIS-S2
EN 3 0
EN 4 0
EN 5 0
EN 6 -14393
EN 7 0
EN 8 0
EN 9 0
DA-M5:SER-CA
EN 3 0
EN 4 0
EN 5 0
EN 6 -7629
EN 7 0
EN 8 0
EN 9 -4375
QUO-M5:ASP-S1
EN 3 0
EN 4 0
EN 5 0
EN 6 0
EN 7 0
EN 8 -15398
EN 9 -3434
G-R6:VAL-CA
EN 3 0
EN 4 0
EN 5 0
EN 6 -5985
EN 7 -2989
EN 8 -958
EN 9 -2325

GTP-M5:SER-CA
EN 3 0
EN 4 0
EN 5 0
EN 6 0
EN 7 0
EN 8 -5607
EN 9 0
FMU-MY:ALA-CA
EN 3 0
EN 4 0
EN 5 0
EN 6 -10245
EN 7 0
EN 8 0
EN 9 0
U31-P:ALA-S1
EN 3 0
EN 4 0
EN 5 0
EN 6 0
EN 7 0
EN 8 0
EN 9 -5295
U34-MY:SER-S1
EN 3 0
EN 4 0
EN 5 -13127
EN 6 0
EN 7 -5468
EN 8 0
EN 9 0
U31-MY:GLN-S2
EN 3 0
EN 4 0
EN 5 0
EN 6 0
EN 7 0
EN 8 -5152
EN 9 0
A-P:GLY-CA
EN 3 0
EN 4 -5720
EN 5 -7626
EN 6 -3722
EN 7 -3290
EN 8 -4708
EN 9 -3674
GTP-M6:GLY-CA
EN 3 0
EN 4 0
EN 5 0
EN 6 0
EN 7 0
EN 8 -5680
EN 9 0
G-R6:ALA-CA
EN 3 0

EN 4 0
EN 5 -4761
EN 6 -2981
EN 7 -1690
EN 8 -1863
EN 9 -3064
A-RIB:ALA-CA
EN 3 0
EN 4 0
EN 5 -1225
EN 6 -4492
EN 7 -4185
EN 8 -2182
EN 9 -2695
C31-P:GLU-S1
EN 3 0
EN 4 0
EN 5 0
EN 6 0
EN 7 -5560
EN 8 0
EN 9 0
H2U-MY:ILE-CA
EN 3 0
EN 4 0
EN 5 0
EN 6 0
EN 7 0
EN 8 0
EN 9 -7012
G-R5:GLN-CA
EN 3 0
EN 4 0
EN 5 0
EN 6 0
EN 7 -1848
EN 8 -2686
EN 9 -4710
H2U-MY:TRP-S1
EN 3 0
EN 4 0
EN 5 0
EN 6 0
EN 7 -17115
EN 8 0
EN 9 0
C-RIB:TYR-CA
EN 3 0
EN 4 0
EN 5 0
EN 6 -5178
EN 7 -4918
EN 8 -4833
EN 9 -3527
DA-RIB:ARG-S2
EN 3 0
EN 4 0
EN 5 0

EN 6 0
EN 7 0
EN 8 0
EN 9 -4235
C31-P:GLN-S1
EN 3 0
EN 4 0
EN 5 0
EN 6 0
EN 7 0
EN 8 -5253
EN 9 0
G-R5:GLN-S2
EN 3 0
EN 4 -6451
EN 5 -4706
EN 6 -5410
EN 7 -4346
EN 8 -3393
EN 9 -2838
C-Y:ASN-CA
EN 3 0
EN 4 0
EN 5 -6660
EN 6 -2185
EN 7 -3525
EN 8 -2266
EN 9 -3779
U-P:LEU-S2
EN 3 0
EN 4 0
EN 5 0
EN 6 -2444
EN 7 -3581
EN 8 -4385
EN 9 -3710
U31-MY:ILE-S1
EN 3 0
EN 4 0
EN 5 0
EN 6 0
EN 7 0
EN 8 -8038
EN 9 0
C-RIB:ASP-S1
EN 3 0
EN 4 0
EN 5 -4250
EN 6 -4273
EN 7 -2582
EN 8 -888
EN 9 -2033
A-R6:VAL-S1
EN 3 0
EN 4 0
EN 5 -6897
EN 6 -6474
EN 7 -1821

EN 8 -3829
EN 9 -3691
U34-MY:ASN-CA
EN 3 0
EN 4 0
EN 5 0
EN 6 0
EN 7 0
EN 8 0
EN 9 -5295
U-Y:TYR-S1
EN 3 0
EN 4 0
EN 5 -10475
EN 6 0
EN 7 -6388
EN 8 -4621
EN 9 -3023
C-Y:HIS-S2
EN 3 0
EN 4 -11764
EN 5 0
EN 6 -5716
EN 7 -5873
EN 8 -4884
EN 9 -2987
IU-MY:ALA-S1
EN 3 0
EN 4 0
EN 5 -17115
EN 6 0
EN 7 0
EN 8 -9908
EN 9 0
C31-MY:ALA-CA
EN 3 0
EN 4 0
EN 5 0
EN 6 0
EN 7 0
EN 8 0
EN 9 -5274
5BU-P:ARG-S1
EN 3 0
EN 4 0
EN 5 0
EN 6 0
EN 7 -9571
EN 8 0
EN 9 0
QUO-RIB:LYS-S1
EN 3 0
EN 4 0
EN 5 0
EN 6 0
EN 7 0
EN 8 -3434
EN 9 0

QUO-M5:ASN-S2
EN 3 0
EN 4 0
EN 5 -3434
EN 6 0
EN 7 0
EN 8 0
EN 9 0
U-Y:GLU-S2
EN 3 0
EN 4 0
EN 5 -2717
EN 6 -690
EN 7 1611
EN 8 766
EN 9 -763
A-RIB:LEU-CA
EN 3 0
EN 4 0
EN 5 0
EN 6 -5451
EN 7 -4765
EN 8 -3348
EN 9 -3302
IU-P:LEU-S1
EN 3 0
EN 4 0
EN 5 0
EN 6 0
EN 7 0
EN 8 -7325
EN 9 0
C-Y:LEU-S2
EN 3 0
EN 4 0
EN 5 -7502
EN 6 -3973
EN 7 -4261
EN 8 -2977
EN 9 -3793
G-R6:PHE-S1
EN 3 0
EN 4 0
EN 5 -6493
EN 6 -5115
EN 7 -4014
EN 8 -3059
EN 9 -3973
H2U-MY:GLU-S1
EN 3 0
EN 4 0
EN 5 -12294
EN 6 0
EN 7 0
EN 8 0
EN 9 0
A-P:MET-S2
EN 3 0

EN 4 0
EN 5 -2410
EN 6 -4661
EN 7 -3864
EN 8 -2864
EN 9 -3355
U34-P:GLU-S2
EN 3 0
EN 4 0
EN 5 0
EN 6 -9456
EN 7 0
EN 8 0
EN 9 0
U34-P:PRO-S1
EN 3 0
EN 4 0
EN 5 0
EN 6 0
EN 7 0
EN 8 -14393
EN 9 0
A-R5:ALA-S1
EN 3 0
EN 4 -6933
EN 5 0
EN 6 -1588
EN 7 -3653
EN 8 -2175
EN 9 -3229
IU-RIB:ALA-CA
EN 3 0
EN 4 0
EN 5 0
EN 6 0
EN 7 -9090
EN 8 -8980
EN 9 0
C-Y:ASP-CA
EN 3 0
EN 4 0
EN 5 0
EN 6 -5442
EN 7 -2503
EN 8 -2655
EN 9 -3220
FMU-MY:ALA-S1
EN 3 0
EN 4 0
EN 5 0
EN 6 0
EN 7 -10095
EN 8 0
EN 9 0
FHU-MY:CYS-S1
EN 3 0
EN 4 0
EN 5 0

EN 6 0
EN 7 0
EN 8 0
EN 9 -11963
C31-RIB:ASP-CA
EN 3 0
EN 4 0
EN 5 0
EN 6 0
EN 7 -4871
EN 8 0
EN 9 0
C-Y:GLN-S1
EN 3 0
EN 4 -9042
EN 5 0
EN 6 -1528
EN 7 -4983
EN 8 -3787
EN 9 -4216
H2U-P:TRP-S2
EN 3 0
EN 4 0
EN 5 0
EN 6 -15398
EN 7 0
EN 8 0
EN 9 0
A-R6:LYS-S1
EN 3 0
EN 4 -7638
EN 5 -4270
EN 6 -2938
EN 7 -2369
EN 8 -2523
EN 9 -3382
U31-MY:MET-S1
EN 3 0
EN 4 0
EN 5 -10958
EN 6 0
EN 7 0
EN 8 0
EN 9 0
FHU-MY:PRO-S1
EN 3 0
EN 4 0
EN 5 0
EN 6 0
EN 7 0
EN 8 -7739
EN 9 0
IU-MY:ARG-CA
EN 3 0
EN 4 0
EN 5 0
EN 6 0
EN 7 0

EN 8 -10297
EN 9 -8236
A-P:TYR-CA
EN 3 0
EN 4 0
EN 5 -9066
EN 6 -2456
EN 7 -4744
EN 8 -3391
EN 9 -5296
IU-P:ARG-S1
EN 3 0
EN 4 0
EN 5 0
EN 6 0
EN 7 0
EN 8 -9401
EN 9 0
5BU-RIB:PRO-CA
EN 3 0
EN 4 0
EN 5 0
EN 6 0
EN 7 -13127
EN 8 0
EN 9 0
U31-MY:TYR-S1
EN 3 0
EN 4 0
EN 5 0
EN 6 0
EN 7 0
EN 8 -6166
EN 9 0
U-RIB:ARG-CA
EN 3 0
EN 4 0
EN 5 -6040
EN 6 -5198
EN 7 -3667
EN 8 -3280
EN 9 -3923
DA-M6:ASN-S2
EN 3 0
EN 4 0
EN 5 0
EN 6 -6772
EN 7 0
EN 8 0
EN 9 0
QUO-M6:ASP-S1
EN 3 0
EN 4 0
EN 5 0
EN 6 0
EN 7 -15398
EN 8 0
EN 9 0

C31-MY:PHE-S2
EN 3 0
EN 4 0
EN 5 -10760
EN 6 0
EN 7 0
EN 8 0
EN 9 0
I-RIB:ALA-CA
EN 3 0
EN 4 0
EN 5 0
EN 6 0
EN 7 0
EN 8 0
EN 9 -12675
GTP-RIB:ARG-S2
EN 3 0
EN 4 0
EN 5 0
EN 6 0
EN 7 0
EN 8 0
EN 9 -4361
A-R6:PHE-CA
EN 3 0
EN 4 0
EN 5 -6991
EN 6 -5271
EN 7 -1559
EN 8 -4863
EN 9 -3491
U-P:ASN-S2
EN 3 0
EN 4 -3016
EN 5 -6005
EN 6 -4269
EN 7 -2960
EN 8 -2620
EN 9 -3760
G-R5:TRP-S1
EN 3 0
EN 4 -12675
EN 5 0
EN 6 -7939
EN 7 -5007
EN 8 -3984
EN 9 -5007
FHU-P:TYR-S1
EN 3 0
EN 4 0
EN 5 0
EN 6 0
EN 7 0
EN 8 -9139
EN 9 0
QUO-M5:LEU-S2
EN 3 0

EN 4 0
EN 5 -3434
EN 6 0
EN 7 0
EN 8 0
EN 9 -14393
H2U-MY:GLN-S1
EN 3 0
EN 4 0
EN 5 0
EN 6 0
EN 7 -8859
EN 8 0
EN 9 0
A-R5:LEU-S1
EN 3 0
EN 4 0
EN 5 0
EN 6 -8500
EN 7 -4820
EN 8 -5159
EN 9 -4550
QUO-RIB:LEU-CA
EN 3 0
EN 4 0
EN 5 0
EN 6 0
EN 7 0
EN 8 0
EN 9 -17115
A-R5:MET-S2
EN 3 0
EN 4 0
EN 5 -9746
EN 6 -2326
EN 7 -5416
EN 8 -4907
EN 9 -4034
C31-P:GLN-S2
EN 3 0
EN 4 0
EN 5 0
EN 6 0
EN 7 0
EN 8 0
EN 9 -4304
M2G-P:GLU-S1
EN 3 0
EN 4 0
EN 5 0
EN 6 0
EN 7 0
EN 8 -8606
EN 9 0
FHU-P:ASP-CA
EN 3 0
EN 4 0
EN 5 0

EN 6 0
EN 7 0
EN 8 -8168
EN 9 -6258
OMC-P:LYS-S2
EN 3 0
EN 4 0
EN 5 -7914
EN 6 0
EN 7 0
EN 8 0
EN 9 -3619
IU-RIB:ARG-CA
EN 3 0
EN 4 0
EN 5 0
EN 6 -13127
EN 7 0
EN 8 -8282
EN 9 0
U-RIB:PHE-S2
EN 3 0
EN 4 0
EN 5 -5814
EN 6 -8339
EN 7 -5856
EN 8 -1822
EN 9 -4095
G-R6:GLY-CA
EN 3 0
EN 4 0
EN 5 -5775
EN 6 -4403
EN 7 -3333
EN 8 -3443
EN 9 -3058
QUO-M5:ARG-S1
EN 3 0
EN 4 0
EN 5 0
EN 6 0
EN 7 0
EN 8 0
EN 9 -3434
U31-RIB:PHE-S1
EN 3 0
EN 4 0
EN 5 0
EN 6 0
EN 7 0
EN 8 -5858
EN 9 0
FHU-RIB:LEU-CA
EN 3 0
EN 4 0
EN 5 0
EN 6 -18832
EN 7 0

EN 8 0
EN 9 0
GTP-M5:ASP-S2
EN 3 0
EN 4 0
EN 5 0
EN 6 0
EN 7 0
EN 8 0
EN 9 -5274
U34-MY:PRO-CA
EN 3 0
EN 4 0
EN 5 0
EN 6 0
EN 7 0
EN 8 0
EN 9 -14393
DA-RIB:GLN-S1
EN 3 0
EN 4 0
EN 5 0
EN 6 0
EN 7 0
EN 8 0
EN 9 -6536
IU-MY:VAL-CA
EN 3 0
EN 4 0
EN 5 0
EN 6 0
EN 7 0
EN 8 0
EN 9 -11010
G-P:THR-CA
EN 3 0
EN 4 -5730
EN 5 -6107
EN 6 -4157
EN 7 -3664
EN 8 -2517
EN 9 -2307
C31-RIB:GLU-CA
EN 3 0
EN 4 0
EN 5 0
EN 6 -8949
EN 7 0
EN 8 0
EN 9 0
GTP-RIB:SER-CA
EN 3 0
EN 4 0
EN 5 0
EN 6 0
EN 7 0
EN 8 0
EN 9 -6107

GTP-M5:ALA-CA
EN 3 0
EN 4 0
EN 5 0
EN 6 0
EN 7 0
EN 8 0
EN 9 -4101
H2U-MY:PHE-S2
EN 3 0
EN 4 -2722
EN 5 0
EN 6 0
EN 7 0
EN 8 0
EN 9 0
DA-RIB:SER-S1
EN 3 0
EN 4 0
EN 5 0
EN 6 0
EN 7 0
EN 8 0
EN 9 -5074
QUO-M5:PHE-S1
EN 3 0
EN 4 0
EN 5 0
EN 6 0
EN 7 0
EN 8 -3434
EN 9 -3434
DA-RIB:ALA-CA
EN 3 0
EN 4 0
EN 5 0
EN 6 0
EN 7 -7373
EN 8 0
EN 9 0
FHU-P:THR-S1
EN 3 0
EN 4 0
EN 5 0
EN 6 0
EN 7 -8606
EN 8 -8135
EN 9 0
FMU-MY:ASP-S1
EN 3 0
EN 4 0
EN 5 0
EN 6 0
EN 7 -9456
EN 8 0
EN 9 0
U-P:VAL-CA
EN 3 0

EN 4 0
EN 5 0
EN 6 -2389
EN 7 -2366
EN 8 -3726
EN 9 -2842
FMU-RIB:VAL-S1
EN 3 0
EN 4 0
EN 5 0
EN 6 0
EN 7 -12294
EN 8 0
EN 9 0
5BU-RIB:SER-S1
EN 3 0
EN 4 0
EN 5 0
EN 6 0
EN 7 0
EN 8 0
EN 9 -6417
C31-RIB:LEU-CA
EN 3 0
EN 4 0
EN 5 0
EN 6 -8038
EN 7 0
EN 8 0
EN 9 0
DA-RIB:HIS-S2
EN 3 0
EN 4 0
EN 5 0
EN 6 -8236
EN 7 0
EN 8 0
EN 9 0
U34-P:ASN-CA
EN 3 0
EN 4 0
EN 5 0
EN 6 -17115
EN 7 0
EN 8 0
EN 9 -8715
G-RIB:PRO-CA
EN 3 0
EN 4 -7087
EN 5 -1844
EN 6 -5671
EN 7 -4163
EN 8 -3296
EN 9 -2888
G-RIB:CYS-S1
EN 3 0
EN 4 0
EN 5 0

EN 6 0
EN 7 -3900
EN 8 0
EN 9 -3171
H2U-P:ARG-S1
EN 3 0
EN 4 0
EN 5 0
EN 6 0
EN 7 0
EN 8 -7097
EN 9 0
DA-M6:HIS-CA
EN 3 0
EN 4 0
EN 5 0
EN 6 -10760
EN 7 0
EN 8 0
EN 9 0
G-RIB:ASP-CA
EN 3 0
EN 4 0
EN 5 -3621
EN 6 -2779
EN 7 -2075
EN 8 -1192
EN 9 -2513
U-Y:PHE-S1
EN 3 0
EN 4 0
EN 5 -8402
EN 6 -6501
EN 7 -6874
EN 8 -4962
EN 9 -4660
FHU-MY:ARG-S2
EN 3 0
EN 4 0
EN 5 0
EN 6 -10095
EN 7 0
EN 8 -7629
EN 9 0
C-Y:TYR-S2
EN 3 0
EN 4 -12817
EN 5 -8146
EN 6 -4434
EN 7 -6023
EN 8 -3708
EN 9 -3463
U31-MY:ALA-CA
EN 3 0
EN 4 0
EN 5 0
EN 6 0
EN 7 0

EN 8 -4871
EN 9 0
H2U-P:PHE-S2
EN 3 0
EN 4 0
EN 5 0
EN 6 -2722
EN 7 0
EN 8 0
EN 9 0
G-R6:THR-S1
EN 3 0
EN 4 0
EN 5 0
EN 6 -3164
EN 7 -3172
EN 8 -2634
EN 9 -2404
U31-MY:ASP-S1
EN 3 0
EN 4 0
EN 5 0
EN 6 -7422
EN 7 0
EN 8 -4207
EN 9 -4101
C-RIB:SER-CA
EN 3 0
EN 4 0
EN 5 -6285
EN 6 -5020
EN 7 -4358
EN 8 -3789
EN 9 -2840
FHU-P:ASP-S2
EN 3 0
EN 4 0
EN 5 0
EN 6 0
EN 7 0
EN 8 0
EN 9 -6196
H2U-MY:LEU-S1
EN 3 0
EN 4 0
EN 5 0
EN 6 0
EN 7 0
EN 8 -4651
EN 9 -3684
FMU-RIB:ALA-CA
EN 3 0
EN 4 0
EN 5 0
EN 6 0
EN 7 0
EN 8 -9953
EN 9 0

H2U-P:ASN-CA
EN 3 0
EN 4 0
EN 5 0
EN 6 0
EN 7 0
EN 8 -9190
EN 9 0
U34-MY:SER-CA
EN 3 0
EN 4 0
EN 5 0
EN 6 -13680
EN 7 0
EN 8 0
EN 9 -4114
C-Y:LEU-CA
EN 3 0
EN 4 0
EN 5 -8829
EN 6 -4793
EN 7 -3728
EN 8 -4292
EN 9 -4235
G-RIB:SER-S1
EN 3 0
EN 4 -1981
EN 5 -5191
EN 6 -2531
EN 7 -3269
EN 8 -2718
EN 9 -3258
FHU-RIB:LYS-CA
EN 3 0
EN 4 0
EN 5 0
EN 6 0
EN 7 0
EN 8 0
EN 9 -6451
U31-RIB:ASN-S1
EN 3 0
EN 4 0
EN 5 0
EN 6 0
EN 7 -5491
EN 8 0
EN 9 0
C-RIB:ALA-CA
EN 3 0
EN 4 0
EN 5 -6020
EN 6 -4749
EN 7 -4435
EN 8 -2239
EN 9 -3304
DA-RIB:GLN-S2
EN 3 0

EN 4 0
EN 5 0
EN 6 0
EN 7 0
EN 8 -5806
EN 9 0
C31-P:GLU-CA
EN 3 0
EN 4 0
EN 5 0
EN 6 -8038
EN 7 0
EN 8 0
EN 9 0
U31-P:HIS-S1
EN 3 0
EN 4 0
EN 5 0
EN 6 0
EN 7 0
EN 8 -5911
EN 9 0
DA-RIB:VAL-CA
EN 3 0
EN 4 0
EN 5 0
EN 6 0
EN 7 0
EN 8 -6810
EN 9 0
C31-P:ASN-CA
EN 3 0
EN 4 0
EN 5 0
EN 6 0
EN 7 0
EN 8 0
EN 9 -4221
H2U-RIB:PHE-CA
EN 3 0
EN 4 0
EN 5 0
EN 6 0
EN 7 0
EN 8 0
EN 9 -7975
QUO-P:SER-S1
EN 3 0
EN 4 0
EN 5 0
EN 6 0
EN 7 0
EN 8 0
EN 9 -13127
H2U-P:THR-CA
EN 3 0
EN 4 0
EN 5 0

EN 6 0
EN 7 0
EN 8 0
EN 9 -8815
DA-M5:ASN-CA
EN 3 0
EN 4 0
EN 5 0
EN 6 0
EN 7 0
EN 8 0
EN 9 -4464
FHU-P:VAL-S1
EN 3 0
EN 4 0
EN 5 0
EN 6 0
EN 7 0
EN 8 -9953
EN 9 0
U-RIB:ASP-S2
EN 3 0
EN 4 0
EN 5 -3772
EN 6 -947
EN 7 -275
EN 8 -1067
EN 9 -1323
DA-RIB:VAL-S1
EN 3 0
EN 4 0
EN 5 0
EN 6 0
EN 7 0
EN 8 -7141
EN 9 0
C-P:PRO-CA
EN 3 0
EN 4 0
EN 5 -6723
EN 6 -4779
EN 7 -2525
EN 8 -3655
EN 9 -3144
H2U-MY:ASN-S2
EN 3 0
EN 4 0
EN 5 -11963
EN 6 0
EN 7 -6320
EN 8 0
EN 9 -7278
QUO-M6:PHE-CA
EN 3 0
EN 4 0
EN 5 0
EN 6 0
EN 7 0

EN 8 -3434
EN 9 -3434
DA-M6:THR-CA
EN 3 0
EN 4 0
EN 5 0
EN 6 0
EN 7 0
EN 8 0
EN 9 -6078
A-R6:GLN-CA
EN 3 0
EN 4 0
EN 5 0
EN 6 -6490
EN 7 -4250
EN 8 -2279
EN 9 -3767
U31-MY:TYR-CA
EN 3 0
EN 4 0
EN 5 0
EN 6 0
EN 7 0
EN 8 -5607
EN 9 0
G-R5:GLU-S2
EN 3 0
EN 4 -3209
EN 5 -2839
EN 6 -498
EN 7 -1849
EN 8 -374
EN 9 -537
C-RIB:CYS-S1
EN 3 0
EN 4 0
EN 5 0
EN 6 0
EN 7 -6245
EN 8 -4359
EN 9 -3577
IU-P:ALA-CA
EN 3 0
EN 4 0
EN 5 0
EN 6 0
EN 7 0
EN 8 0
EN 9 -8688
QUO-M5:GLU-S2
EN 3 0
EN 4 0
EN 5 0
EN 6 0
EN 7 -3434
EN 8 0
EN 9 0

A-R5:VAL-CA
EN 3 0
EN 4 0
EN 5 -7739
EN 6 -6258
EN 7 -5884
EN 8 -2511
EN 9 -3250
FMU-RIB:MET-S2
EN 3 0
EN 4 0
EN 5 0
EN 6 0
EN 7 0
EN 8 0
EN 9 -2722
DA-M5:THR-CA
EN 3 0
EN 4 0
EN 5 0
EN 6 0
EN 7 0
EN 8 0
EN 9 -4619
FMU-MY:GLN-S1
EN 3 0
EN 4 0
EN 5 0
EN 6 0
EN 7 -9953
EN 8 0
EN 9 0
IU-RIB:LEU-S2
EN 3 0
EN 4 0
EN 5 0
EN 6 -10760
EN 7 0
EN 8 0
EN 9 0
G-P:CYS-S1
EN 3 0
EN 4 0
EN 5 0
EN 6 -3434
EN 7 -1839
EN 8 0
EN 9 -1951
H2U-MY:LYS-S2
EN 3 0
EN 4 0
EN 5 0
EN 6 0
EN 7 -5537
EN 8 0
EN 9 0
GTP-M5:THR-S1
EN 3 0

EN 4 0
EN 5 0
EN 6 0
EN 7 0
EN 8 0
EN 9 -4375
U31-P:MET-CA
EN 3 0
EN 4 0
EN 5 0
EN 6 -11410
EN 7 0
EN 8 -7097
EN 9 0
IU-P:HIS-S1
EN 3 0
EN 4 0
EN 5 -2722
EN 6 0
EN 7 0
EN 8 0
EN 9 0
G-RIB:GLU-S1
EN 3 0
EN 4 0
EN 5 -2498
EN 6 -820
EN 7 -1001
EN 8 1311
EN 9 -904
C-P:ARG-CA
EN 3 0
EN 4 0
EN 5 -7179
EN 6 -5451
EN 7 -5543
EN 8 -4778
EN 9 -4516
5BU-MY:ILE-S1
EN 3 0
EN 4 0
EN 5 0
EN 6 0
EN 7 -2722
EN 8 0
EN 9 0
H2U-P:LEU-S1
EN 3 0
EN 4 0
EN 5 0
EN 6 0
EN 7 0
EN 8 0
EN 9 -5055
U-Y:MET-S1
EN 3 0
EN 4 0
EN 5 -7171

EN 6 -5759
EN 7 -5518
EN 8 -2244
EN 9 -2669
A-R5:PRO-CA
EN 3 0
EN 4 0
EN 5 -5263
EN 6 -5075
EN 7 -5417
EN 8 -3864
EN 9 -3500
U-RIB:TRP-S2
EN 3 0
EN 4 0
EN 5 -7854
EN 6 -6913
EN 7 -4158
EN 8 -4453
EN 9 -2504
G-RIB:ALA-CA
EN 3 0
EN 4 0
EN 5 -2044
EN 6 -3875
EN 7 -2571
EN 8 -2027
EN 9 -2937
FHU-MY:ILE-S1
EN 3 0
EN 4 0
EN 5 0
EN 6 -12891
EN 7 0
EN 8 0
EN 9 0
FHU-MY:ASP-CA
EN 3 0
EN 4 0
EN 5 0
EN 6 0
EN 7 -10760
EN 8 0
EN 9 0
A-RIB:CYS-CA
EN 3 0
EN 4 0
EN 5 0
EN 6 -5832
EN 7 -6774
EN 8 -4831
EN 9 -2921
U34-P:HIS-S1
EN 3 0
EN 4 0
EN 5 0
EN 6 0
EN 7 0

EN 8 -9346
EN 9 0
G-R6:CYS-CA
EN 3 0
EN 4 0
EN 5 0
EN 6 0
EN 7 -6591
EN 8 -1852
EN 9 -2195
FHU-RIB:LYS-S1
EN 3 0
EN 4 0
EN 5 0
EN 6 0
EN 7 0
EN 8 0
EN 9 -8378
GTP-M6:SER-CA
EN 3 0
EN 4 0
EN 5 0
EN 6 0
EN 7 0
EN 8 0
EN 9 -4587
C31-P:THR-S1
EN 3 0
EN 4 0
EN 5 0
EN 6 0
EN 7 0
EN 8 -8528
EN 9 0
H2U-P:PRO-CA
EN 3 0
EN 4 0
EN 5 0
EN 6 0
EN 7 0
EN 8 0
EN 9 -5358
C-P:ASN-S2
EN 3 0
EN 4 0
EN 5 -6283
EN 6 -4488
EN 7 -3339
EN 8 -3172
EN 9 -3559
FMU-P:ASP-S1
EN 3 0
EN 4 0
EN 5 0
EN 6 0
EN 7 0
EN 8 -11410
EN 9 0

A-RIB:PHE-S2
EN 3 0
EN 4 0
EN 5 -9803
EN 6 -6720
EN 7 -4910
EN 8 -3115
EN 9 -3634
QUO-M6:ASN-S2
EN 3 0
EN 4 0
EN 5 -3434
EN 6 0
EN 7 0
EN 8 0
EN 9 0
H2U-P:LEU-S2
EN 3 0
EN 4 0
EN 5 0
EN 6 0
EN 7 -7141
EN 8 0
EN 9 0
QUO-M6:ARG-CA
EN 3 0
EN 4 0
EN 5 0
EN 6 0
EN 7 -3434
EN 8 0
EN 9 0
G-R6:PHE-S2
EN 3 0
EN 4 -11671
EN 5 0
EN 6 -6277
EN 7 -566
EN 8 -2591
EN 9 -4338
G-RIB:G-R6:TRP-CA
EN 20 0
EN 40 0
EN 60 -4814
EN 80 -8140
EN 100 0
EN 120 -5483
EN 140 -5838
EN 160 0
EN 180 0
EN 200 0
EN 220 -8528
EN 240 0
EN 260 -3402
EN 280 -3420
EN 300 -5207
EN 320 -3990
EN 340 0

EN 360 0
C-RIB:C-Y:MET-CA
EN 20 0
EN 40 -5884
EN 60 -6624
EN 80 -4283
EN 100 -2904
EN 120 -3121
EN 140 0
EN 160 0
EN 180 0
EN 200 0
EN 220 -4509
EN 240 0
EN 260 -3081
EN 280 -5354
EN 300 -3154
EN 320 0
EN 340 0
EN 360 0
U-RIB:U-P:THR-CA
EN 20 0
EN 40 -6289
EN 60 -2680
EN 80 -2054
EN 100 -1234
EN 120 -3769
EN 140 -3021
EN 160 -1568
EN 180 -2345
EN 200 0
EN 220 -6553
EN 240 -5420
EN 260 -4496
EN 280 -3772
EN 300 -2996
EN 320 -3960
EN 340 -3180
EN 360 -3831
U-RIB:U-Y:VAL-CA
EN 20 0
EN 40 -5362
EN 60 -1804
EN 80 -2703
EN 100 -3979
EN 120 0
EN 140 -2634
EN 160 -1893
EN 180 0
EN 200 0
EN 220 0
EN 240 -5007
EN 260 -3732
EN 280 -4446
EN 300 -271
EN 320 -3919
EN 340 -3753
EN 360 0

C-P:C-RIB:HIS-S2
EN 20 -7523
EN 40 -4486
EN 60 -3771
EN 80 -3535
EN 100 -3870
EN 120 -4627
EN 140 -3216
EN 160 -4675
EN 180 -5364
EN 200 0
EN 220 -2784
EN 240 -3872
EN 260 -2976
EN 280 -4538
EN 300 -4039
EN 320 -5295
EN 340 -1675
EN 360 -5337
A-RIB:A-R6:ALA-CA
EN 20 0
EN 40 -4577
EN 60 -3989
EN 80 -1768
EN 100 -2652
EN 120 -2091
EN 140 0
EN 160 -1502
EN 180 -4050
EN 200 0
EN 220 -4412
EN 240 -4156
EN 260 0
EN 280 -2739
EN 300 -2686
EN 320 -4613
EN 340 -2464
EN 360 -2342
G-RIB:G-R6:GLU-S1
EN 20 0
EN 40 -2026
EN 60 -1560
EN 80 -141
EN 100 -749
EN 120 -842
EN 140 305
EN 160 -1439
EN 180 -4066
EN 200 0
EN 220 -2107
EN 240 -2103
EN 260 -1154
EN 280 -167
EN 300 -1173
EN 320 -1917
EN 340 -3227
EN 360 -1377
A-RIB:A-R6:PRO-CA

EN 20 0
EN 40 0
EN 60 -2749
EN 80 -3141
EN 100 -4874
EN 120 -2400
EN 140 -3192
EN 160 -4732
EN 180 -3459
EN 200 0
EN 220 -6493
EN 240 -3247
EN 260 -4107
EN 280 -3437
EN 300 -5046
EN 320 -2901
EN 340 -767
EN 360 -3237
FHU-RIB:FHU-P:LYS-S2
EN 20 0
EN 40 0
EN 60 0
EN 80 0
EN 100 0
EN 120 -8606
EN 140 -7683
EN 160 0
EN 180 0
EN 200 0
EN 220 0
EN 240 0
EN 260 0
EN 280 0
EN 300 -8859
EN 320 0
EN 340 -9953
EN 360 0
C-P:C-RIB:ARG-S2
EN 20 -8251
EN 40 -6546
EN 60 -5460
EN 80 -4265
EN 100 -3676
EN 120 -3815
EN 140 -4294
EN 160 -2545
EN 180 -3664
EN 200 -8210
EN 220 -7046
EN 240 -5790
EN 260 -4434
EN 280 -4358
EN 300 -4375
EN 320 -4295
EN 340 -3769
EN 360 -3987
G-RIB:G-P:ARG-CA
EN 20 0

EN 40 -5945
EN 60 -4390
EN 80 -4575
EN 100 -4235
EN 120 -4532
EN 140 -4129
EN 160 -4897
EN 180 -3405
EN 200 0
EN 220 -2615
EN 240 -4702
EN 260 -3961
EN 280 -4823
EN 300 -3425
EN 320 -4701
EN 340 -5200
EN 360 -4794
G-RIB:G-R6:TYR-CA
EN 20 0
EN 40 0
EN 60 0
EN 80 -2323
EN 100 -5906
EN 120 -2102
EN 140 -2608
EN 160 -2653
EN 180 -5945
EN 200 0
EN 220 0
EN 240 0
EN 260 -4800
EN 280 -1834
EN 300 -4910
EN 320 -5275
EN 340 0
EN 360 -7676
U-P:U-RIB:SER-S1
EN 20 -5132
EN 40 -3041
EN 60 -4213
EN 80 -4226
EN 100 -3463
EN 120 -2803
EN 140 -2696
EN 160 -984
EN 180 -3253
EN 200 -6929
EN 220 -933
EN 240 -3668
EN 260 -3197
EN 280 -3754
EN 300 -1130
EN 320 -2662
EN 340 -3837
EN 360 -1458
G-P:G-RIB:THR-CA
EN 20 -8236
EN 40 -4474

EN 60 -1037
EN 80 -3615
EN 100 -2900
EN 120 -3628
EN 140 -3521
EN 160 -2999
EN 180 -1282
EN 200 -9776
EN 220 -5380
EN 240 -2962
EN 260 -1792
EN 280 -769
EN 300 -2541
EN 320 -2591
EN 340 -2611
EN 360 -4692
G-RIB:G-P:VAL-S1
EN 20 0
EN 40 -2257
EN 60 -1452
EN 80 1784
EN 100 -2348
EN 120 -2832
EN 140 -3937
EN 160 -2139
EN 180 -1249
EN 200 0
EN 220 -4949
EN 240 -2517
EN 260 -1817
EN 280 -1665
EN 300 -276
EN 320 -2107
EN 340 -2479
EN 360 -3922
G-RIB:G-R6:TRP-S1
EN 20 0
EN 40 -6258
EN 60 -4147
EN 80 -6045
EN 100 -5308
EN 120 -6859
EN 140 -4283
EN 160 0
EN 180 0
EN 200 0
EN 220 -8006
EN 240 -5677
EN 260 -3432
EN 280 -2893
EN 300 -7301
EN 320 -3972
EN 340 -6647
EN 360 0
G-RIB:G-R5:ASP-CA
EN 20 -3654
EN 40 -2216
EN 60 -2764

EN 80 -1866
EN 100 -1431
EN 120 0
EN 140 -2839
EN 160 -3879
EN 180 0
EN 200 -3828
EN 220 -3743
EN 240 -2734
EN 260 -1953
EN 280 -2186
EN 300 -3476
EN 320 -1110
EN 340 -3006
EN 360 0
G-RIB:G-P:ASN-S2
EN 20 0
EN 40 -4659
EN 60 -4606
EN 80 -3112
EN 100 -3161
EN 120 -3074
EN 140 -2783
EN 160 -4375
EN 180 -4687
EN 200 0
EN 220 -4149
EN 240 -3914
EN 260 -3697
EN 280 -4830
EN 300 -3743
EN 320 -2599
EN 340 -3095
EN 360 -786
U-RIB:U-P:ARG-S1
EN 20 0
EN 40 -2651
EN 60 -2339
EN 80 -4396
EN 100 -4211
EN 120 -3999
EN 140 -4817
EN 160 -4574
EN 180 -5504
EN 200 -8528
EN 220 -6035
EN 240 -4006
EN 260 -4536
EN 280 -4893
EN 300 -4700
EN 320 -4257
EN 340 -5050
EN 360 -4184
U-RIB:U-P:ASN-CA
EN 20 0
EN 40 -4998
EN 60 -3221
EN 80 -3767

EN 100 -2963
EN 120 -4748
EN 140 -4458
EN 160 -3701
EN 180 -2608
EN 200 0
EN 220 0
EN 240 -3434
EN 260 -4525
EN 280 -3677
EN 300 -3522
EN 320 -2165
EN 340 -3069
EN 360 0
FHU-RIB:FHU-P:TYR-CA
EN 20 0
EN 40 -4439
EN 60 0
EN 80 0
EN 100 0
EN 120 0
EN 140 0
EN 160 0
EN 180 -13680
EN 200 0
EN 220 0
EN 240 0
EN 260 0
EN 280 0
EN 300 0
EN 320 0
EN 340 0
EN 360 0
A-RIB:A-P:PRO-CA
EN 20 0
EN 40 0
EN 60 -3369
EN 80 -3612
EN 100 -4685
EN 120 -2411
EN 140 -2740
EN 160 -3054
EN 180 -5074
EN 200 0
EN 220 -5402
EN 240 -4896
EN 260 -3697
EN 280 -4041
EN 300 -2989
EN 320 -5012
EN 340 -3285
EN 360 -1962
5BU-P:5BU-RIB:ILE-S1
EN 20 0
EN 40 0
EN 60 0
EN 80 0
EN 100 0

EN 120 0
EN 140 0
EN 160 0
EN 180 0
EN 200 0
EN 220 0
EN 240 -2722
EN 260 0
EN 280 0
EN 300 0
EN 320 0
EN 340 0
EN 360 0
G-P:G-RIB:MET-S2
EN 20 0
EN 40 -4810
EN 60 -1204
EN 80 -580
EN 100 -3192
EN 120 -2548
EN 140 -2665
EN 160 -5546
EN 180 0
EN 200 0
EN 220 0
EN 240 -3890
EN 260 -4358
EN 280 -2928
EN 300 -2131
EN 320 -4069
EN 340 -6705
EN 360 -3515
C-RIB:C-Y:ASP-S2
EN 20 -1682
EN 40 -4411
EN 60 27
EN 80 -4194
EN 100 -1687
EN 120 -1039
EN 140 -2280
EN 160 -3865
EN 180 -5027
EN 200 -1476
EN 220 -1831
EN 240 -3093
EN 260 -1252
EN 280 -2256
EN 300 -26
EN 320 -2677
EN 340 -2529
EN 360 0
C-P:C-RIB:MET-S2
EN 20 0
EN 40 0
EN 60 -4337
EN 80 -4939
EN 100 -4184
EN 120 -4687

EN 140 -3645
EN 160 -6050
EN 180 -5142
EN 200 0
EN 220 -3385
EN 240 -3420
EN 260 -4075
EN 280 -4512
EN 300 -3312
EN 320 -5024
EN 340 -4225
EN 360 0
C31-RIB:C31-MY:LEU-CA
EN 20 0
EN 40 0
EN 60 0
EN 80 0
EN 100 0
EN 120 0
EN 140 0
EN 160 0
EN 180 0
EN 200 0
EN 220 0
EN 240 0
EN 260 -7373
EN 280 0
EN 300 0
EN 320 0
EN 340 0
EN 360 0
C-RIB:C-Y:THR-S1
EN 20 -2849
EN 40 -4836
EN 60 -3482
EN 80 -3956
EN 100 -3256
EN 120 -1290
EN 140 -3358
EN 160 -2433
EN 180 -4801
EN 200 -5517
EN 220 -4178
EN 240 -4692
EN 260 -2862
EN 280 -2709
EN 300 458
EN 320 -2608
EN 340 -4740
EN 360 -4517
U-RIB:U-Y:SER-CA
EN 20 0
EN 40 -4479
EN 60 -4733
EN 80 -4576
EN 100 -2382
EN 120 -3187
EN 140 -319

EN 160 -1094
EN 180 0
EN 200 -4871
EN 220 -3321
EN 240 -3058
EN 260 -2916
EN 280 -3097
EN 300 -952
EN 320 -4073
EN 340 -5235
EN 360 -4041
C-RIB:C-Y:GLN-CA
EN 20 0
EN 40 -5963
EN 60 -1678
EN 80 -4865
EN 100 -3028
EN 120 -2796
EN 140 -3630
EN 160 0
EN 180 0
EN 200 -7112
EN 220 -7436
EN 240 -5934
EN 260 -5350
EN 280 -939
EN 300 -2942
EN 320 0
EN 340 -2580
EN 360 0
A-RIB:A-R5:ILE-CA
EN 20 -6929
EN 40 -3808
EN 60 -4970
EN 80 -1605
EN 100 -1414
EN 120 -4329
EN 140 0
EN 160 -5332
EN 180 0
EN 200 0
EN 220 -4088
EN 240 -4990
EN 260 -1465
EN 280 0
EN 300 -5377
EN 320 -2610
EN 340 0
EN 360 0
A-RIB:A-P:HIS-S1
EN 20 0
EN 40 -5112
EN 60 -7218
EN 80 -4743
EN 100 -4736
EN 120 -2584
EN 140 -4719
EN 160 -3950

EN 180 -5212
EN 200 0
EN 220 -7033
EN 240 -5139
EN 260 -2694
EN 280 -5169
EN 300 -1584
EN 320 -3021
EN 340 -5144
EN 360 -5326
QUO-RIB:QUO-M5:GLN-S2
EN 20 0
EN 40 0
EN 60 0
EN 80 0
EN 100 -3434
EN 120 0
EN 140 0
EN 160 0
EN 180 0
EN 200 0
EN 220 0
EN 240 0
EN 260 0
EN 280 0
EN 300 0
EN 320 0
EN 340 0
EN 360 0
G-RIB:G-P:SER-CA
EN 20 0
EN 40 -4342
EN 60 -3473
EN 80 -3047
EN 100 -1809
EN 120 -3490
EN 140 -4743
EN 160 -3976
EN 180 -621
EN 200 0
EN 220 -3469
EN 240 -4316
EN 260 -3619
EN 280 -4240
EN 300 -3105
EN 320 -3029
EN 340 -2326
EN 360 -4770
U-RIB:U-P:ALA-S1
EN 20 0
EN 40 -1918
EN 60 -3371
EN 80 -4791
EN 100 -3122
EN 120 -1519
EN 140 -3578
EN 160 -3385
EN 180 -5243

EN 200 -5884
EN 220 -4514
EN 240 -2687
EN 260 -4664
EN 280 -4341
EN 300 -3144
EN 320 -4668
EN 340 -2552
EN 360 -5705
G-RIB:G-R5:ASN-S2
EN 20 -5360
EN 40 -3358
EN 60 -2378
EN 80 -2674
EN 100 -4110
EN 120 -4411
EN 140 -4522
EN 160 -3393
EN 180 -3079
EN 200 -5231
EN 220 -2390
EN 240 -4347
EN 260 -2570
EN 280 -5199
EN 300 -3989
EN 320 -1594
EN 340 0
EN 360 -4589
U-P:U-RIB:GLN-CA
EN 20 0
EN 40 -6368
EN 60 -3230
EN 80 -283
EN 100 -3883
EN 120 -1682
EN 140 -2176
EN 160 -4733
EN 180 -3609
EN 200 0
EN 220 -5793
EN 240 -4154
EN 260 -308
EN 280 -4688
EN 300 -4228
EN 320 -2003
EN 340 -3789
EN 360 -5122
A-RIB:A-R5:THR-S1
EN 20 -6291
EN 40 -3837
EN 60 -4525
EN 80 -4037
EN 100 -4567
EN 120 -2480
EN 140 -3838
EN 160 -1304
EN 180 -3743
EN 200 -5656

EN 220 -5208
EN 240 -3605
EN 260 -4456
EN 280 -1123
EN 300 -2544
EN 320 -2117
EN 340 -4012
EN 360 0
C-P:C-RIB:CYS-CA
EN 20 0
EN 40 -8528
EN 60 0
EN 80 -4494
EN 100 -4449
EN 120 0
EN 140 -4479
EN 160 -5607
EN 180 0
EN 200 0
EN 220 0
EN 240 0
EN 260 -4419
EN 280 -6970
EN 300 -4318
EN 320 -4717
EN 340 -5832
EN 360 0
A-RIB:A-P:GLN-S2
EN 20 0
EN 40 -6178
EN 60 -3427
EN 80 -5182
EN 100 -2553
EN 120 -2566
EN 140 -3775
EN 160 -3472
EN 180 -4801
EN 200 0
EN 220 -4147
EN 240 -2800
EN 260 -4454
EN 280 -4045
EN 300 -290
EN 320 -3935
EN 340 -2821
EN 360 -3745
C-RIB:C-P:TYR-S2
EN 20 0
EN 40 0
EN 60 -5695
EN 80 -4801
EN 100 -3583
EN 120 -4862
EN 140 51
EN 160 -2647
EN 180 -3444
EN 200 0
EN 220 -4943

EN 240 -3690
EN 260 -3828
EN 280 -3619
EN 300 -2465
EN 320 -4624
EN 340 -2962
EN 360 0
G-RIB:G-R6:ASN-CA
EN 20 0
EN 40 0
EN 60 -5364
EN 80 -4619
EN 100 -2774
EN 120 -4843
EN 140 -2266
EN 160 -2631
EN 180 0
EN 200 0
EN 220 -6278
EN 240 -3661
EN 260 -2571
EN 280 -3957
EN 300 -4320
EN 320 -4049
EN 340 -1358
EN 360 -3762
U-P:U-RIB:ASN-S1
EN 20 -9090
EN 40 0
EN 60 -5587
EN 80 -3008
EN 100 -3347
EN 120 -3699
EN 140 -2408
EN 160 -3731
EN 180 -6184
EN 200 0
EN 220 -5351
EN 240 -4733
EN 260 -3148
EN 280 -4016
EN 300 -3541
EN 320 -2860
EN 340 -5421
EN 360 -5514
U-RIB:U-P:HIS-CA
EN 20 0
EN 40 -10461
EN 60 -5867
EN 80 -3149
EN 100 -2191
EN 120 -3751
EN 140 -2466
EN 160 -5197
EN 180 0
EN 200 0
EN 220 0
EN 240 -6830

EN 260 -5801
EN 280 -4703
EN 300 -3041
EN 320 -2583
EN 340 -5785
EN 360 0
G-P:G-RIB:ASN-S2
EN 20 -6122
EN 40 -5032
EN 60 -4682
EN 80 -3729
EN 100 -3902
EN 120 -1418
EN 140 -4287
EN 160 -3636
EN 180 -3241
EN 200 -6417
EN 220 -3825
EN 240 -3938
EN 260 -3379
EN 280 -2779
EN 300 -4299
EN 320 -78
EN 340 -3132
EN 360 -552
C-RIB:C-P:GLU-S2
EN 20 -3366
EN 40 -551
EN 60 -1853
EN 80 398
EN 100 -1795
EN 120 -1936
EN 140 -374
EN 160 -1727
EN 180 -297
EN 200 0
EN 220 -626
EN 240 557
EN 260 -32
EN 280 -1711
EN 300 -951
EN 320 -111
EN 340 903
EN 360 -1311
G-RIB:G-P:PRO-S1
EN 20 0
EN 40 -4902
EN 60 -5032
EN 80 -2186
EN 100 -3643
EN 120 -2800
EN 140 -2587
EN 160 -3126
EN 180 -3677
EN 200 0
EN 220 -4464
EN 240 -5026
EN 260 -4189

EN 280 -3959
EN 300 -3359
EN 320 -3022
EN 340 -1114
EN 360 -4950
A-RIB:A-R5:TRP-CA
EN 20 0
EN 40 -5780
EN 60 0
EN 80 -4801
EN 100 -4238
EN 120 -4884
EN 140 -4907
EN 160 0
EN 180 0
EN 200 0
EN 220 -6588
EN 240 -6519
EN 260 -7810
EN 280 -4767
EN 300 0
EN 320 -5074
EN 340 0
EN 360 0
A-RIB:A-R6:SER-CA
EN 20 -8772
EN 40 -4548
EN 60 -2963
EN 80 -1889
EN 100 -716
EN 120 -3463
EN 140 -4640
EN 160 -3052
EN 180 0
EN 200 0
EN 220 -5631
EN 240 -4238
EN 260 -3039
EN 280 -2323
EN 300 -3017
EN 320 -2964
EN 340 -4320
EN 360 -4684
U-RIB:U-Y:ARG-CA
EN 20 -5491
EN 40 -2526
EN 60 -5094
EN 80 -6724
EN 100 -4998
EN 120 -3684
EN 140 -4586
EN 160 -1275
EN 180 -3770
EN 200 0
EN 220 -4095
EN 240 -2908
EN 260 -2286
EN 280 -2778

EN 300 -3749
EN 320 -3098
EN 340 -5271
EN 360 0
A-RIB:A-R6:LYS-S2
EN 20 0
EN 40 180
EN 60 -3334
EN 80 -3427
EN 100 -3482
EN 120 -2239
EN 140 -1919
EN 160 -2409
EN 180 -1149
EN 200 -3993
EN 220 -3224
EN 240 -3157
EN 260 -3480
EN 280 -3234
EN 300 -3419
EN 320 -2541
EN 340 -1709
EN 360 0
U-P:U-RIB:GLU-S1
EN 20 0
EN 40 -602
EN 60 1794
EN 80 -1773
EN 100 242
EN 120 1155
EN 140 2531
EN 160 188
EN 180 0
EN 200 -5232
EN 220 0
EN 240 2
EN 260 988
EN 280 1231
EN 300 -470
EN 320 2576
EN 340 -796
EN 360 -106
C-RIB:C-P:ILE-S1
EN 20 0
EN 40 -5884
EN 60 -4805
EN 80 -5006
EN 100 -4163
EN 120 -1803
EN 140 -3193
EN 160 -1986
EN 180 -2925
EN 200 0
EN 220 -4088
EN 240 -4607
EN 260 -3402
EN 280 -3694
EN 300 -2521

EN 320 -4449
EN 340 0
EN 360 -2430
G-P:G-RIB:HIS-CA
EN 20 0
EN 40 -4419
EN 60 -1241
EN 80 -3276
EN 100 -3097
EN 120 326
EN 140 -5788
EN 160 0
EN 180 -5592
EN 200 0
EN 220 -5424
EN 240 -1196
EN 260 -5989
EN 280 -4966
EN 300 -1594
EN 320 -3588
EN 340 -4710
EN 360 -4651
C-RIB:C-P:GLY-CA
EN 20 0
EN 40 -5600
EN 60 -2781
EN 80 -3100
EN 100 -4348
EN 120 -2808
EN 140 -3934
EN 160 -4195
EN 180 -3767
EN 200 0
EN 220 -5560
EN 240 -4987
EN 260 -3403
EN 280 -3712
EN 300 -3208
EN 320 -4449
EN 340 -4028
EN 360 -3056
G-RIB:G-R5:ALA-CA
EN 20 0
EN 40 -2431
EN 60 -673
EN 80 -2135
EN 100 -3396
EN 120 0
EN 140 -2902
EN 160 -1952
EN 180 0
EN 200 0
EN 220 -4504
EN 240 -4269
EN 260 -2915
EN 280 -3201
EN 300 -3348
EN 320 -2876

EN 340 -348
EN 360 -2746
A-P:A-RIB:ASP-CA
EN 20 0
EN 40 0
EN 60 0
EN 80 -2161
EN 100 -2342
EN 120 -531
EN 140 207
EN 160 -380
EN 180 -693
EN 200 0
EN 220 -1907
EN 240 875
EN 260 -2601
EN 280 -2993
EN 300 -3509
EN 320 -2491
EN 340 -4020
EN 360 0
G-RIB:G-R5:ILE-CA
EN 20 -5514
EN 40 0
EN 60 -1447
EN 80 -559
EN 100 -3748
EN 120 0
EN 140 0
EN 160 0
EN 180 0
EN 200 -6336
EN 220 -4682
EN 240 -3236
EN 260 -3076
EN 280 -3875
EN 300 -2855
EN 320 0
EN 340 -4754
EN 360 0
U-RIB:U-Y:THR-CA
EN 20 0
EN 40 -5125
EN 60 -4642
EN 80 -3119
EN 100 -2718
EN 120 -4243
EN 140 -799
EN 160 0
EN 180 -4295
EN 200 -6166
EN 220 0
EN 240 -1265
EN 260 -3474
EN 280 -1876
EN 300 -1901
EN 320 -2105
EN 340 -3393

EN 360 0
U-RIB:U-P:HIS-S2
EN 20 0
EN 40 -6078
EN 60 -3180
EN 80 -5085
EN 100 -3158
EN 120 -1680
EN 140 -3636
EN 160 -3797
EN 180 -7549
EN 200 0
EN 220 -7510
EN 240 -5980
EN 260 -4889
EN 280 -4998
EN 300 -3801
EN 320 -4254
EN 340 -948
EN 360 -3762
U-RIB:U-Y:GLY-CA
EN 20 -7433
EN 40 -3596
EN 60 -5268
EN 80 1465
EN 100 -3787
EN 120 -2736
EN 140 -986
EN 160 -2036
EN 180 -4351
EN 200 -5780
EN 220 -5173
EN 240 -4602
EN 260 -4779
EN 280 -3410
EN 300 -1537
EN 320 879
EN 340 -3573
EN 360 -4169
C-RIB:C-Y:TRP-S1
EN 20 0
EN 40 -6804
EN 60 -3930
EN 80 -2490
EN 100 -5709
EN 120 -5579
EN 140 0
EN 160 -5274
EN 180 0
EN 200 -9042
EN 220 0
EN 240 0
EN 260 -3987
EN 280 -4323
EN 300 0
EN 320 -5212
EN 340 -5680
EN 360 0

G-RIB:G-R5:ASP-S2
EN 20 0
EN 40 -1057
EN 60 -1184
EN 80 -2703
EN 100 -1012
EN 120 -1572
EN 140 -3369
EN 160 -2140
EN 180 -1831
EN 200 0
EN 220 -4309
EN 240 -1137
EN 260 -3792
EN 280 -3714
EN 300 -829
EN 320 -3700
EN 340 -2746
EN 360 -1691
G-RIB:G-P:GLN-CA
EN 20 0
EN 40 -4943
EN 60 -4008
EN 80 -2811
EN 100 -2297
EN 120 -3092
EN 140 -3782
EN 160 -2189
EN 180 -4790
EN 200 0
EN 220 -4961
EN 240 -5066
EN 260 -3835
EN 280 -4289
EN 300 -3204
EN 320 -3864
EN 340 -4881
EN 360 -4733
U-RIB:U-Y:ASP-S1
EN 20 -3839
EN 40 -2356
EN 60 -1903
EN 80 -2376
EN 100 1043
EN 120 -911
EN 140 -3631
EN 160 -555
EN 180 0
EN 200 0
EN 220 -2237
EN 240 -3161
EN 260 -1523
EN 280 0
EN 300 -763
EN 320 0
EN 340 0
EN 360 -2947
U-RIB:U-Y:GLU-CA

EN 20 0
EN 40 -2920
EN 60 215
EN 80 -1852
EN 100 1355
EN 120 1274
EN 140 692
EN 160 -589
EN 180 -2888
EN 200 -5884
EN 220 -1293
EN 240 -1506
EN 260 815
EN 280 -588
EN 300 1126
EN 320 562
EN 340 0
EN 360 0
H2U-RIB:H2U-MY:LEU-CA
EN 20 0
EN 40 0
EN 60 0
EN 80 0
EN 100 0
EN 120 0
EN 140 0
EN 160 -10958
EN 180 0
EN 200 0
EN 220 0
EN 240 0
EN 260 0
EN 280 0
EN 300 0
EN 320 -7472
EN 340 0
EN 360 0
G-RIB:G-R6:LEU-S2
EN 20 -6859
EN 40 -4603
EN 60 -2832
EN 80 -2965
EN 100 -3408
EN 120 -4018
EN 140 -2444
EN 160 -3571
EN 180 0
EN 200 0
EN 220 -4920
EN 240 -4385
EN 260 -3651
EN 280 -2641
EN 300 -4362
EN 320 -2325
EN 340 0
EN 360 0
U-RIB:U-P:MET-CA
EN 20 0

EN 40 -7422
EN 60 -7127
EN 80 0
EN 100 -3185
EN 120 -4750
EN 140 -3712
EN 160 -2731
EN 180 0
EN 200 -12294
EN 220 -8306
EN 240 -6107
EN 260 0
EN 280 -1535
EN 300 -3963
EN 320 -1712
EN 340 0
EN 360 0
C-RIB:C-P:MET-S1
EN 20 0
EN 40 -8967
EN 60 -4235
EN 80 -5286
EN 100 -546
EN 120 -2936
EN 140 -2552
EN 160 -1772
EN 180 0
EN 200 0
EN 220 -5358
EN 240 -6376
EN 260 -4867
EN 280 -4476
EN 300 -2191
EN 320 -2410
EN 340 -5607
EN 360 0
A-RIB:A-R5:ASN-S2
EN 20 -7490
EN 40 -4589
EN 60 -3355
EN 80 -3418
EN 100 -4273
EN 120 -3880
EN 140 -3218
EN 160 -5372
EN 180 0
EN 200 -5031
EN 220 -4812
EN 240 -4677
EN 260 -3973
EN 280 -4133
EN 300 -4750
EN 320 -235
EN 340 -1400
EN 360 -3151
C31-RIB:C31-P:TYR-CA
EN 20 0
EN 40 0

EN 60 0
EN 80 0
EN 100 -7575
EN 120 0
EN 140 0
EN 160 0
EN 180 0
EN 200 0
EN 220 0
EN 240 0
EN 260 0
EN 280 -9456
EN 300 0
EN 320 0
EN 340 0
EN 360 0
G-P:G-RIB:LYS-S1
EN 20 -3567
EN 40 -3719
EN 60 -4247
EN 80 -4229
EN 100 -4179
EN 120 -3343
EN 140 -3278
EN 160 -1497
EN 180 574
EN 200 -7434
EN 220 -3870
EN 240 -5111
EN 260 -4060
EN 280 -2473
EN 300 -3607
EN 320 -2832
EN 340 -1939
EN 360 -2784
A-RIB:A-R6:ILE-CA
EN 20 0
EN 40 -6859
EN 60 -4283
EN 80 -2991
EN 100 0
EN 120 -2396
EN 140 -3501
EN 160 -3621
EN 180 -4167
EN 200 0
EN 220 0
EN 240 -2687
EN 260 -2852
EN 280 -3371
EN 300 -4156
EN 320 -900
EN 340 -3512
EN 360 -4479
G-P:G-RIB:TRP-S2
EN 20 0
EN 40 0
EN 60 -2807

EN 80 -4592
EN 100 -4216
EN 120 -4706
EN 140 -2079
EN 160 -2909
EN 180 -4684
EN 200 0
EN 220 0
EN 240 -3118
EN 260 -4961
EN 280 -1940
EN 300 -5780
EN 320 -5178
EN 340 -2909
EN 360 0
A-P:A-RIB:HIS-S1
EN 20 0
EN 40 -5925
EN 60 -4635
EN 80 -2604
EN 100 -5256
EN 120 -4034
EN 140 -4177
EN 160 -2921
EN 180 -4889
EN 200 0
EN 220 0
EN 240 -5730
EN 260 -4635
EN 280 -5256
EN 300 -5896
EN 320 -6068
EN 340 -3971
EN 360 -3318
A-RIB:A-R6:THR-S1
EN 20 0
EN 40 -4573
EN 60 -2741
EN 80 -4697
EN 100 -4138
EN 120 -3467
EN 140 -1213
EN 160 -3953
EN 180 -3257
EN 200 0
EN 220 -6385
EN 240 -3531
EN 260 -4623
EN 280 -2410
EN 300 -2416
EN 320 -1247
EN 340 -4477
EN 360 -3095
C-P:C-RIB:THR-CA
EN 20 -7422
EN 40 -2497
EN 60 -4991
EN 80 -3500

EN 100 -2357
EN 120 -4977
EN 140 -1932
EN 160 -2756
EN 180 -5232
EN 200 -7854
EN 220 -5295
EN 240 -4216
EN 260 -4390
EN 280 -3714
EN 300 -3903
EN 320 -3645
EN 340 -4334
EN 360 -4464
U-RIB:U-P:LYS-CA
EN 20 0
EN 40 0
EN 60 -4592
EN 80 -5010
EN 100 -3844
EN 120 -2923
EN 140 -4444
EN 160 -5159
EN 180 -4771
EN 200 0
EN 220 -3774
EN 240 -5705
EN 260 -3853
EN 280 -2840
EN 300 -3171
EN 320 -4667
EN 340 -3840
EN 360 -4046
U-P:U-RIB:SER-CA
EN 20 0
EN 40 -5152
EN 60 -4767
EN 80 -3355
EN 100 -4402
EN 120 -3899
EN 140 -761
EN 160 -3687
EN 180 -3395
EN 200 -6889
EN 220 -3902
EN 240 -4082
EN 260 -2843
EN 280 -3290
EN 300 -2734
EN 320 -3535
EN 340 -1461
EN 360 -3439
U-RIB:U-Y:CYS-CA
EN 20 0
EN 40 0
EN 60 -6836
EN 80 0
EN 100 0

EN 120 0
EN 140 0
EN 160 0
EN 180 0
EN 200 0
EN 220 0
EN 240 0
EN 260 -5212
EN 280 0
EN 300 -7575
EN 320 -5849
EN 340 -7422
EN 360 0
U-RIB:U-Y:LEU-CA
EN 20 -7914
EN 40 -6283
EN 60 -6420
EN 80 -3663
EN 100 -2768
EN 120 -4901
EN 140 -1426
EN 160 0
EN 180 -5152
EN 200 0
EN 220 0
EN 240 -4545
EN 260 -1531
EN 280 -895
EN 300 -5571
EN 320 -4735
EN 340 -5278
EN 360 0
U-RIB:U-P:SER-CA
EN 20 0
EN 40 -3337
EN 60 -4268
EN 80 -3307
EN 100 -1589
EN 120 169
EN 140 -4282
EN 160 -3860
EN 180 -2120
EN 200 0
EN 220 -6088
EN 240 -5289
EN 260 -2377
EN 280 -4827
EN 300 -4188
EN 320 -5163
EN 340 -3958
EN 360 -2091
G-RIB:G-R5:PRO-S1
EN 20 -7004
EN 40 -3387
EN 60 -4312
EN 80 -2660
EN 100 -2360
EN 120 -2930

EN 140 -207
EN 160 -2710
EN 180 0
EN 200 -4552
EN 220 -5959
EN 240 -3620
EN 260 -2661
EN 280 -2549
EN 300 -3753
EN 320 -373
EN 340 -3904
EN 360 0
A-RIB:A-R5:TYR-S2
EN 20 -8859
EN 40 -3881
EN 60 -5714
EN 80 -6428
EN 100 -4902
EN 120 -5734
EN 140 -5736
EN 160 -5771
EN 180 0
EN 200 -9692
EN 220 -3966
EN 240 -5147
EN 260 -6855
EN 280 -1718
EN 300 -3893
EN 320 0
EN 340 -3687
EN 360 -7711
C-RIB:C-P:LYS-S2
EN 20 0
EN 40 -3406
EN 60 -3417
EN 80 -2922
EN 100 -2668
EN 120 -4230
EN 140 -3686
EN 160 -3011
EN 180 -4261
EN 200 -4708
EN 220 -3572
EN 240 -3606
EN 260 -3460
EN 280 -2874
EN 300 -4313
EN 320 -4630
EN 340 -4976
EN 360 -4380
G-RIB:G-P:HIS-CA
EN 20 0
EN 40 -5358
EN 60 -5418
EN 80 -6350
EN 100 -3506
EN 120 -3318
EN 140 -3725

EN 160 -3712
EN 180 -4375
EN 200 0
EN 220 0
EN 240 -1767
EN 260 -1634
EN 280 -3878
EN 300 -4439
EN 320 -3892
EN 340 -2102
EN 360 0
C-P:C-RIB:VAL-CA
EN 20 0
EN 40 -5055
EN 60 -2818
EN 80 -4546
EN 100 -3155
EN 120 -4435
EN 140 -4733
EN 160 -3725
EN 180 -1273
EN 200 0
EN 220 -4793
EN 240 -2836
EN 260 -5473
EN 280 -3526
EN 300 -2507
EN 320 -4026
EN 340 -3935
EN 360 0
G-RIB:G-R6:HIS-S1
EN 20 -9819
EN 40 -7108
EN 60 -4185
EN 80 -4889
EN 100 -4686
EN 120 -5595
EN 140 -1571
EN 160 0
EN 180 0
EN 200 0
EN 220 -5613
EN 240 -5322
EN 260 -5550
EN 280 -4221
EN 300 -4715
EN 320 -4047
EN 340 0
EN 360 -5182
C-P:C-RIB:TYR-CA
EN 20 0
EN 40 0
EN 60 -2301
EN 80 -3221
EN 100 -5209
EN 120 -4989
EN 140 0
EN 160 0

EN 180 0
EN 200 0
EN 220 -5468
EN 240 -2800
EN 260 -5228
EN 280 -6058
EN 300 -5596
EN 320 -2285
EN 340 -4163
EN 360 -5212
U-RIB:U-P:ALA-CA
EN 20 0
EN 40 -3118
EN 60 -4907
EN 80 -4207
EN 100 -4022
EN 120 -2099
EN 140 -4495
EN 160 -4933
EN 180 -5525
EN 200 0
EN 220 -5623
EN 240 -2251
EN 260 -4219
EN 280 -3404
EN 300 -4071
EN 320 -3559
EN 340 -3816
EN 360 -3646
G-RIB:G-P:LYS-S1
EN 20 0
EN 40 0
EN 60 -3416
EN 80 -4440
EN 100 -3800
EN 120 -3838
EN 140 -4093
EN 160 -4105
EN 180 -4779
EN 200 0
EN 220 -3605
EN 240 -4538
EN 260 -3940
EN 280 -3594
EN 300 -2882
EN 320 -3708
EN 340 -4166
EN 360 -4774
U-RIB:U-P:CYS-S1
EN 20 0
EN 40 0
EN 60 -7325
EN 80 0
EN 100 0
EN 120 0
EN 140 -4998
EN 160 -6289
EN 180 0

EN 200 0
EN 220 0
EN 240 0
EN 260 0
EN 280 0
EN 300 -5017
EN 320 0
EN 340 -6227
EN 360 0
C-P:C-RIB:VAL-S1
EN 20 0
EN 40 -3356
EN 60 -2989
EN 80 -4114
EN 100 -4117
EN 120 -4047
EN 140 -3987
EN 160 -4309
EN 180 0
EN 200 0
EN 220 0
EN 240 -3975
EN 260 -5156
EN 280 -3028
EN 300 -3219
EN 320 -3953
EN 340 -2581
EN 360 -1253
C-P:C-RIB:LEU-CA
EN 20 0
EN 40 -5668
EN 60 -4703
EN 80 -4072
EN 100 -3596
EN 120 -3174
EN 140 -3074
EN 160 -3174
EN 180 -5680
EN 200 0
EN 220 0
EN 240 -3956
EN 260 -3907
EN 280 -5421
EN 300 -4523
EN 320 -4057
EN 340 -4799
EN 360 0
A-RIB:A-P:GLN-CA
EN 20 -12294
EN 40 0
EN 60 -4678
EN 80 -4804
EN 100 -3841
EN 120 -3753
EN 140 -4112
EN 160 -4082
EN 180 0
EN 200 0

EN 220 -8168
EN 240 -2143
EN 260 -2048
EN 280 -4207
EN 300 -5170
EN 320 -3571
EN 340 -4986
EN 360 -4571
G-RIB:G-R6:ASP-S2
EN 20 -4211
EN 40 -3349
EN 60 -421
EN 80 -1893
EN 100 -2181
EN 120 -2126
EN 140 -2012
EN 160 -3149
EN 180 -4418
EN 200 -6111
EN 220 -3266
EN 240 -2409
EN 260 -2850
EN 280 -3200
EN 300 -2965
EN 320 -2376
EN 340 -4011
EN 360 -3476
A-RIB:A-P:TYR-S1
EN 20 0
EN 40 0
EN 60 -7562
EN 80 -3849
EN 100 -4805
EN 120 -4438
EN 140 -4849
EN 160 -6174
EN 180 0
EN 200 0
EN 220 0
EN 240 -7313
EN 260 -6233
EN 280 -4033
EN 300 -5074
EN 320 -5117
EN 340 -2702
EN 360 0
G-RIB:G-P:MET-S1
EN 20 0
EN 40 0
EN 60 -4108
EN 80 -3438
EN 100 -2854
EN 120 -1754
EN 140 -3154
EN 160 -1634
EN 180 -3663
EN 200 0
EN 220 0

EN 240 -4095
EN 260 -1003
EN 280 -1762
EN 300 -103
EN 320 -3005
EN 340 -2877
EN 360 0
C-RIB:C-P:HIS-S2
EN 20 0
EN 40 -6527
EN 60 -3602
EN 80 -3571
EN 100 -3825
EN 120 -2460
EN 140 -3878
EN 160 -3464
EN 180 0
EN 200 0
EN 220 -7422
EN 240 -2552
EN 260 -4345
EN 280 -3444
EN 300 -3581
EN 320 -3963
EN 340 -1953
EN 360 -6000
U-RIB:U-Y:THR-S1
EN 20 0
EN 40 -4027
EN 60 -4684
EN 80 -1332
EN 100 -4891
EN 120 -3789
EN 140 -2186
EN 160 -4139
EN 180 0
EN 200 -4123
EN 220 -4044
EN 240 -3368
EN 260 -2439
EN 280 164
EN 300 -2566
EN 320 -269
EN 340 0
EN 360 0
G-RIB:G-R6:ILE-S1
EN 20 -8414
EN 40 0
EN 60 -1366
EN 80 0
EN 100 -3334
EN 120 -2190
EN 140 0
EN 160 -2001
EN 180 0
EN 200 0
EN 220 0
EN 240 -3400

EN 260 -1966
EN 280 -2142
EN 300 -1896
EN 320 0
EN 340 0
EN 360 -5026
A-RIB:A-P:ALA-CA
EN 20 0
EN 40 -4325
EN 60 -4351
EN 80 -3957
EN 100 -3859
EN 120 -2541
EN 140 -3420
EN 160 -2929
EN 180 -4840
EN 200 0
EN 220 0
EN 240 -3383
EN 260 -3774
EN 280 -2246
EN 300 -3390
EN 320 -3999
EN 340 -4087
EN 360 -3351
A-P:A-RIB:PHE-CA
EN 20 0
EN 40 0
EN 60 -4810
EN 80 -5487
EN 100 -2618
EN 120 -4339
EN 140 -3918
EN 160 -3706
EN 180 -4619
EN 200 0
EN 220 0
EN 240 -3024
EN 260 -4356
EN 280 -4401
EN 300 -4286
EN 320 -5763
EN 340 -3525
EN 360 -4050
A-RIB:A-P:ASP-S1
EN 20 0
EN 40 -1653
EN 60 -702
EN 80 -2422
EN 100 -2068
EN 120 -1346
EN 140 -1646
EN 160 -731
EN 180 -388
EN 200 0
EN 220 -3390
EN 240 -3043
EN 260 -2612

EN 280 -1088
EN 300 -1730
EN 320 -2161
EN 340 -2594
EN 360 -434
U-RIB:U-Y:GLN-CA
EN 20 0
EN 40 -4449
EN 60 -6243
EN 80 -1769
EN 100 0
EN 120 -1122
EN 140 -3461
EN 160 -5485
EN 180 0
EN 200 -9456
EN 220 0
EN 240 -4095
EN 260 -3204
EN 280 -2725
EN 300 -2665
EN 320 -5088
EN 340 0
EN 360 0
QUO-RIB:QUO-M6:GLU-S1
EN 20 0
EN 40 0
EN 60 0
EN 80 0
EN 100 0
EN 120 0
EN 140 0
EN 160 -3434
EN 180 0
EN 200 0
EN 220 0
EN 240 0
EN 260 0
EN 280 0
EN 300 0
EN 320 0
EN 340 0
EN 360 0
U-P:U-RIB:LYS-S2
EN 20 -6181
EN 40 -5058
EN 60 -3797
EN 80 -3524
EN 100 -3934
EN 120 -3356
EN 140 -1566
EN 160 -710
EN 180 -2797
EN 200 -7134
EN 220 -6477
EN 240 -4430
EN 260 -4312
EN 280 -4399

EN 300 -3156
EN 320 -1865
EN 340 -3152
EN 360 -3533
A-P:A-RIB:ILE-CA
EN 20 0
EN 40 0
EN 60 -4520
EN 80 -5433
EN 100 -4065
EN 120 -2587
EN 140 -2774
EN 160 -2854
EN 180 -3092
EN 200 0
EN 220 -4943
EN 240 0
EN 260 -3382
EN 280 -4530
EN 300 -2596
EN 320 -3593
EN 340 -772
EN 360 -5920
C-RIB:C-Y:LYS-S1
EN 20 -7472
EN 40 -3933
EN 60 -3915
EN 80 -2797
EN 100 -3731
EN 120 -3898
EN 140 -604
EN 160 -3049
EN 180 -3731
EN 200 -4849
EN 220 -2703
EN 240 -2393
EN 260 -3952
EN 280 -3478
EN 300 -1790
EN 320 -2759
EN 340 -2100
EN 360 -4467
A-P:A-RIB:ARG-S1
EN 20 -5938
EN 40 -7469
EN 60 -5730
EN 80 -5415
EN 100 -5183
EN 120 -4389
EN 140 -4045
EN 160 -4854
EN 180 -3665
EN 200 0
EN 220 -6481
EN 240 -5636
EN 260 -4728
EN 280 -5477
EN 300 -3863

EN 320 -4425
EN 340 -3217
EN 360 -5143
G-RIB:G-R6:GLU-CA
EN 20 0
EN 40 -4433
EN 60 -615
EN 80 -1026
EN 100 -200
EN 120 -383
EN 140 1368
EN 160 -2804
EN 180 -3660
EN 200 0
EN 220 -1997
EN 240 -1669
EN 260 -2016
EN 280 799
EN 300 -1441
EN 320 -1405
EN 340 -3867
EN 360 -3293
U-P:U-RIB:ASP-CA
EN 20 0
EN 40 -2777
EN 60 -1298
EN 80 -1246
EN 100 1890
EN 120 -3394
EN 140 -325
EN 160 -2110
EN 180 -3088
EN 200 -8168
EN 220 -2307
EN 240 283
EN 260 1463
EN 280 -2630
EN 300 -3133
EN 320 -2541
EN 340 -3352
EN 360 -2921
QUO-RIB:QUO-M6:LYS-S1
EN 20 0
EN 40 0
EN 60 0
EN 80 -17115
EN 100 0
EN 120 0
EN 140 0
EN 160 0
EN 180 0
EN 200 0
EN 220 0
EN 240 0
EN 260 0
EN 280 0
EN 300 0
EN 320 0

EN 340 0
EN 360 0
A-RIB:A-R5:THR-CA
EN 20 -7739
EN 40 -3077
EN 60 -4732
EN 80 -4032
EN 100 -5131
EN 120 -4290
EN 140 -826
EN 160 -5391
EN 180 0
EN 200 0
EN 220 -6137
EN 240 -5599
EN 260 -3691
EN 280 -4289
EN 300 -3851
EN 320 -2667
EN 340 -5964
EN 360 0
U-P:U-RIB:THR-S1
EN 20 -8606
EN 40 -4853
EN 60 -2345
EN 80 -4003
EN 100 -4067
EN 120 -2265
EN 140 -3699
EN 160 63
EN 180 -4733
EN 200 0
EN 220 -3535
EN 240 -2295
EN 260 -1668
EN 280 -2758
EN 300 -2874
EN 320 -4406
EN 340 -1675
EN 360 0
G-RIB:G-R6:GLN-S1
EN 20 0
EN 40 -7071
EN 60 -4309
EN 80 -5212
EN 100 -2144
EN 120 -4582
EN 140 0
EN 160 -1110
EN 180 0
EN 200 0
EN 220 -7905
EN 240 -3158
EN 260 -5395
EN 280 -3792
EN 300 -2488
EN 320 -2095
EN 340 0

EN 360 0
C-RIB:C-Y:GLN-S2
EN 20 -5777
EN 40 -5336
EN 60 -4516
EN 80 -4827
EN 100 -4507
EN 120 -1937
EN 140 -3150
EN 160 0
EN 180 0
EN 200 -3072
EN 220 -3787
EN 240 -5514
EN 260 -4349
EN 280 -4861
EN 300 -2716
EN 320 0
EN 340 -4696
EN 360 0
A-RIB:A-R6:HIS-CA
EN 20 0
EN 40 0
EN 60 -7012
EN 80 -5766
EN 100 -3032
EN 120 -5342
EN 140 -3478
EN 160 -5319
EN 180 0
EN 200 0
EN 220 0
EN 240 -7460
EN 260 -6138
EN 280 -4743
EN 300 -3161
EN 320 -5085
EN 340 -6037
EN 360 0
C-RIB:C-P:GLN-S1
EN 20 0
EN 40 -5305
EN 60 -3268
EN 80 -4504
EN 100 -3881
EN 120 -3157
EN 140 -1626
EN 160 -3624
EN 180 -3820
EN 200 0
EN 220 -5212
EN 240 -5465
EN 260 -4649
EN 280 -5202
EN 300 -3731
EN 320 -4664
EN 340 -3033
EN 360 -5369

G-P:G-RIB:GLU-S2
EN 20 -1476
EN 40 2149
EN 60 1318
EN 80 314
EN 100 -1041
EN 120 20
EN 140 123
EN 160 -2356
EN 180 189
EN 200 0
EN 220 434
EN 240 -11
EN 260 213
EN 280 -788
EN 300 2734
EN 320 -272
EN 340 -2598
EN 360 -2189
U-RIB:U-Y:SER-S1
EN 20 -5362
EN 40 -5114
EN 60 -3005
EN 80 -3885
EN 100 -3125
EN 120 -2258
EN 140 -1563
EN 160 -888
EN 180 -3274
EN 200 -3107
EN 220 -5671
EN 240 -2360
EN 260 -2489
EN 280 659
EN 300 -3478
EN 320 -3267
EN 340 -4859
EN 360 0
G-RIB:G-R6:ASN-S2
EN 20 0
EN 40 0
EN 60 -3981
EN 80 -3504
EN 100 -3065
EN 120 -3028
EN 140 -4297
EN 160 -1625
EN 180 -4744
EN 200 -5787
EN 220 -4427
EN 240 -4422
EN 260 -2986
EN 280 -3780
EN 300 -4052
EN 320 -3676
EN 340 -2263
EN 360 0
C-P:C-RIB:GLY-CA

EN 20 -6709
EN 40 -5084
EN 60 -4951
EN 80 -3332
EN 100 -4000
EN 120 -3781
EN 140 -3203
EN 160 -3769
EN 180 -3708
EN 200 -3609
EN 220 -5057
EN 240 -4064
EN 260 -3566
EN 280 -3667
EN 300 -3887
EN 320 -3583
EN 340 -3228
EN 360 -2754
U-RIB:U-P:ILE-S1
EN 20 0
EN 40 0
EN 60 -3993
EN 80 -866
EN 100 -1732
EN 120 -1668
EN 140 -294
EN 160 -4097
EN 180 0
EN 200 0
EN 220 -6970
EN 240 0
EN 260 -4276
EN 280 -155
EN 300 -3810
EN 320 0
EN 340 -2724
EN 360 -3630
U-P:U-RIB:ILE-S1
EN 20 -8038
EN 40 0
EN 60 -1737
EN 80 -2651
EN 100 -1918
EN 120 -3075
EN 140 -3987
EN 160 -3105
EN 180 0
EN 200 0
EN 220 -4088
EN 240 -3342
EN 260 -2672
EN 280 -4063
EN 300 0
EN 320 -2320
EN 340 -5098
EN 360 -3831
U-RIB:U-P:LEU-S1
EN 20 0

EN 40 -7012
EN 60 -5674
EN 80 -522
EN 100 -3356
EN 120 -3692
EN 140 -1373
EN 160 -3107
EN 180 0
EN 200 0
EN 220 -8688
EN 240 -3956
EN 260 -3278
EN 280 -5055
EN 300 -2726
EN 320 -3590
EN 340 -3315
EN 360 0
A-RIB:A-R6:GLN-S2
EN 20 0
EN 40 -4044
EN 60 -3497
EN 80 -4188
EN 100 -3912
EN 120 -4553
EN 140 -3690
EN 160 -4142
EN 180 -3295
EN 200 -7097
EN 220 -4000
EN 240 -4013
EN 260 -3220
EN 280 -784
EN 300 -4137
EN 320 -4685
EN 340 -2379
EN 360 -3071
U-RIB:U-P:ARG-S2
EN 20 -7397
EN 40 -5198
EN 60 -4241
EN 80 -4013
EN 100 -4549
EN 120 -3415
EN 140 -5184
EN 160 -5148
EN 180 -5412
EN 200 0
EN 220 -4160
EN 240 -2768
EN 260 -5487
EN 280 -4425
EN 300 -3650
EN 320 -2423
EN 340 -5058
EN 360 -5970
H2U-P:H2U-RIB:ASN-S2
EN 20 0
EN 40 0

EN 60 0
EN 80 0
EN 100 0
EN 120 0
EN 140 0
EN 160 0
EN 180 0
EN 200 0
EN 220 -12675
EN 240 0
EN 260 -10245
EN 280 0
EN 300 -9819
EN 320 0
EN 340 0
EN 360 0
A-RIB:A-P:ASN-CA
EN 20 0
EN 40 -6137
EN 60 -3793
EN 80 -5009
EN 100 -4560
EN 120 -2549
EN 140 -4297
EN 160 -4096
EN 180 -2390
EN 200 0
EN 220 -6624
EN 240 -3290
EN 260 -2876
EN 280 -3688
EN 300 -2683
EN 320 -4748
EN 340 -1724
EN 360 -4082
C-RIB:C-Y:TYR-CA
EN 20 0
EN 40 0
EN 60 -3602
EN 80 -7023
EN 100 -4532
EN 120 -4469
EN 140 -3306
EN 160 -5929
EN 180 0
EN 200 0
EN 220 -4239
EN 240 -4787
EN 260 -7299
EN 280 -2810
EN 300 -5909
EN 320 -3069
EN 340 -3975
EN 360 0
A-P:A-RIB:ASN-S1
EN 20 0
EN 40 -2463
EN 60 -1757

EN 80 -4491
EN 100 -4299
EN 120 -3079
EN 140 -4475
EN 160 -3577
EN 180 -1358
EN 200 0
EN 220 -6208
EN 240 -5086
EN 260 -2303
EN 280 -3881
EN 300 -2973
EN 320 -4779
EN 340 -3952
EN 360 -4884
G-RIB:G-R5:LYS-S2
EN 20 -4907
EN 40 -2232
EN 60 -4092
EN 80 -3694
EN 100 -3583
EN 120 -3070
EN 140 -342
EN 160 -3425
EN 180 -4913
EN 200 -4845
EN 220 -2304
EN 240 -3691
EN 260 -3081
EN 280 -3362
EN 300 -2970
EN 320 -5742
EN 340 -3953
EN 360 -2509
U-RIB:U-Y:GLN-S1
EN 20 0
EN 40 -2591
EN 60 -5927
EN 80 -2145
EN 100 -366
EN 120 -5139
EN 140 -2845
EN 160 -6160
EN 180 -4761
EN 200 0
EN 220 -4484
EN 240 -4389
EN 260 -5197
EN 280 -1577
EN 300 -3731
EN 320 -4441
EN 340 0
EN 360 -6201
G-RIB:G-R5:THR-S1
EN 20 -2760
EN 40 -2724
EN 60 -3016
EN 80 -322

EN 100 -3682
EN 120 -990
EN 140 350
EN 160 -197
EN 180 0
EN 200 -2384
EN 220 -4548
EN 240 -3616
EN 260 -3329
EN 280 -2162
EN 300 -1862
EN 320 -3472
EN 340 -299
EN 360 0
H2U-P:H2U-RIB:ASN-S1
EN 20 0
EN 40 0
EN 60 0
EN 80 0
EN 100 0
EN 120 0
EN 140 0
EN 160 0
EN 180 0
EN 200 0
EN 220 0
EN 240 0
EN 260 -10958
EN 280 0
EN 300 -9819
EN 320 0
EN 340 0
EN 360 0
G-P:G-RIB:GLN-S1
EN 20 -6519
EN 40 -3757
EN 60 -3492
EN 80 -3995
EN 100 -2855
EN 120 -3334
EN 140 -3836
EN 160 -5194
EN 180 -5975
EN 200 0
EN 220 -4509
EN 240 -2114
EN 260 -2966
EN 280 -1794
EN 300 -3339
EN 320 -2958
EN 340 -2989
EN 360 -6656
C-RIB:C-P:ARG-CA
EN 20 0
EN 40 -7501
EN 60 -3515
EN 80 -5569
EN 100 -4613

EN 120 -5295
EN 140 -4382
EN 160 -4842
EN 180 -5876
EN 200 0
EN 220 -2629
EN 240 -4118
EN 260 -4760
EN 280 -4969
EN 300 -4880
EN 320 -5422
EN 340 -4343
EN 360 -5331
U-RIB:U-Y:HIS-CA
EN 20 -9241
EN 40 -8088
EN 60 -4943
EN 80 0
EN 100 -2054
EN 120 -1987
EN 140 -4337
EN 160 -5656
EN 180 0
EN 200 0
EN 220 -6588
EN 240 -3376
EN 260 0
EN 280 -3995
EN 300 -3757
EN 320 -4479
EN 340 0
EN 360 0
C-RIB:C-Y:ILE-CA
EN 20 -7422
EN 40 -5256
EN 60 -5847
EN 80 -1479
EN 100 -4276
EN 120 -4209
EN 140 -4052
EN 160 -3306
EN 180 0
EN 200 0
EN 220 -7243
EN 240 -3563
EN 260 -3429
EN 280 -1491
EN 300 -4797
EN 320 -2008
EN 340 -2802
EN 360 0
C-RIB:C-Y:HIS-S1
EN 20 0
EN 40 -5615
EN 60 -6083
EN 80 -4603
EN 100 -6605
EN 120 -3541

EN 140 -4088
EN 160 -3408
EN 180 -5576
EN 200 0
EN 220 -6024
EN 240 -5302
EN 260 -6758
EN 280 -4678
EN 300 -2054
EN 320 -4163
EN 340 -3598
EN 360 0
A-RIB:A-R5:ASP-CA
EN 20 -6540
EN 40 -4769
EN 60 488
EN 80 -619
EN 100 1112
EN 120 -1942
EN 140 -3380
EN 160 -2698
EN 180 0
EN 200 -4793
EN 220 0
EN 240 -1011
EN 260 -2154
EN 280 -2817
EN 300 791
EN 320 -3408
EN 340 -2631
EN 360 0
G-RIB:G-P:PHE-S1
EN 20 0
EN 40 0
EN 60 -4725
EN 80 -5450
EN 100 -3987
EN 120 -1294
EN 140 -3311
EN 160 -4635
EN 180 0
EN 200 0
EN 220 0
EN 240 -7636
EN 260 -2562
EN 280 -1625
EN 300 -3762
EN 320 -2741
EN 340 -2521
EN 360 -3253
G-RIB:G-P:TRP-S2
EN 20 0
EN 40 -8528
EN 60 -5380
EN 80 -5004
EN 100 -4163
EN 120 -3028
EN 140 -4405

EN 160	-5748
EN 180	0
EN 200	0
EN 220	-7278
EN 240	-3652
EN 260	-3663
EN 280	-4419
EN 300	-3736
EN 320	-4867
EN 340	-2377
EN 360	-4818
U-P:U-RIB:LEU-CA
EN 20 0
EN 40 0
EN 60 -5353
EN 80 0
EN 100	-3271
EN 120	-3647
EN 140	-4186
EN 160	-4123
EN 180	-7108
EN 200	0
EN 220	0
EN 240	-3500
EN 260	-2149
EN 280	-2494
EN 300	-3185
EN 320	-3641
EN 340	-4587
EN 360	0
FHU-P:FHU-RIB:TYR-S2
EN 20 0
EN 40 0
EN 60 0
EN 80 -15398
EN 100	0
EN 120	-11963
EN 140	0
EN 160	0
EN 180	0
EN 200	0
EN 220	0
EN 240	0
EN 260	-10958
EN 280	0
EN 300	-11671
EN 320	0
EN 340	0
EN 360	0
A-RIB:A-R5:ALA-S1
EN 20 -4278
EN 40 -4825
EN 60 -3522
EN 80 -2617
EN 100	-2817
EN 120	-3571
EN 140	-1405
EN 160	-3013

EN 180 -2915
EN 200 -5916
EN 220 -4387
EN 240 -1833
EN 260 -2853
EN 280 -658
EN 300 -2012
EN 320 -1407
EN 340 -2251
EN 360 0
G-RIB:G-R5:ARG-CA
EN 20 -3596
EN 40 -5322
EN 60 -3242
EN 80 -2595
EN 100 -3499
EN 120 -2618
EN 140 -4092
EN 160 -548
EN 180 -6669
EN 200 -6227
EN 220 -5822
EN 240 -3426
EN 260 -4829
EN 280 -4348
EN 300 -4068
EN 320 -5367
EN 340 -5192
EN 360 -3073
A-P:A-RIB:GLU-S1
EN 20 0
EN 40 -252
EN 60 2046
EN 80 -1811
EN 100 -2821
EN 120 -1709
EN 140 -2497
EN 160 614
EN 180 324
EN 200 0
EN 220 0
EN 240 2060
EN 260 -1394
EN 280 -664
EN 300 -1262
EN 320 -1077
EN 340 -1531
EN 360 -1282
G-RIB:G-R6:LYS-S2
EN 20 0
EN 40 -3152
EN 60 -3435
EN 80 -2870
EN 100 -2246
EN 120 -2718
EN 140 -2221
EN 160 -1820
EN 180 -1321

EN 200 0
EN 220 -1499
EN 240 -3328
EN 260 -2741
EN 280 -3915
EN 300 -3647
EN 320 -3127
EN 340 -3429
EN 360 0
C-P:C-RIB:GLU-S1
EN 20 -4750
EN 40 274
EN 60 -2080
EN 80 -1445
EN 100 4007
EN 120 -29
EN 140 -1037
EN 160 -754
EN 180 -1142
EN 200 -4101
EN 220 0
EN 240 143
EN 260 -1063
EN 280 -1150
EN 300 -861
EN 320 249
EN 340 -1323
EN 360 -1971
C-RIB:C-Y:GLU-CA
EN 20 0
EN 40 -2741
EN 60 -2034
EN 80 -1853
EN 100 0
EN 120 -3518
EN 140 -709
EN 160 -1709
EN 180 -2506
EN 200 0
EN 220 -1845
EN 240 -2124
EN 260 1448
EN 280 1424
EN 300 1475
EN 320 0
EN 340 -2821
EN 360 0
A-P:A-RIB:MET-CA
EN 20 0
EN 40 -5993
EN 60 -3110
EN 80 -6005
EN 100 -1742
EN 120 -4814
EN 140 -3663
EN 160 -2622
EN 180 0
EN 200 0

EN 220 -8070
EN 240 -3366
EN 260 -4717
EN 280 -4984
EN 300 -4434
EN 320 -1849
EN 340 -5274
EN 360 0
FHU-RIB:FHU-P:ASP-S2
EN 20 0
EN 40 0
EN 60 0
EN 80 0
EN 100 0
EN 120 0
EN 140 0
EN 160 -8949
EN 180 0
EN 200 0
EN 220 0
EN 240 0
EN 260 0
EN 280 0
EN 300 0
EN 320 0
EN 340 0
EN 360 0
G-RIB:G-R6:LYS-CA
EN 20 0
EN 40 -4961
EN 60 -2095
EN 80 -1529
EN 100 -3733
EN 120 -3136
EN 140 -623
EN 160 296
EN 180 -3929
EN 200 0
EN 220 -2071
EN 240 -4608
EN 260 -3941
EN 280 -3504
EN 300 -1021
EN 320 -4188
EN 340 -4531
EN 360 -3726
U-RIB:U-Y:ASN-CA
EN 20 0
EN 40 -3438
EN 60 -5526
EN 80 -2479
EN 100 -5416
EN 120 -3098
EN 140 -4469
EN 160 -1980
EN 180 0
EN 200 0
EN 220 -5373

EN 240 -4328
EN 260 -5680
EN 280 -4182
EN 300 -1918
EN 320 -4758
EN 340 -4469
EN 360 0
C-P:C-RIB:ILE-CA
EN 20 0
EN 40 -5832
EN 60 -3084
EN 80 -4607
EN 100 -3683
EN 120 -3648
EN 140 554
EN 160 -2036
EN 180 -2644
EN 200 0
EN 220 -4603
EN 240 0
EN 260 -3955
EN 280 -1982
EN 300 -4285
EN 320 -4530
EN 340 -3902
EN 360 -4540
C-P:C-RIB:ALA-CA
EN 20 -5656
EN 40 -2792
EN 60 -4034
EN 80 -3559
EN 100 -4192
EN 120 -1951
EN 140 -4113
EN 160 -3011
EN 180 -2134
EN 200 -5730
EN 220 -4675
EN 240 -4029
EN 260 -3541
EN 280 -2959
EN 300 -3437
EN 320 -3759
EN 340 -4086
EN 360 -3159
C-RIB:C-P:ALA-CA
EN 20 0
EN 40 -5468
EN 60 -4453
EN 80 -3591
EN 100 -3880
EN 120 -2790
EN 140 -2902
EN 160 -4526
EN 180 -2460
EN 200 -6929
EN 220 -4509
EN 240 -4545

EN 260 -3533
EN 280 -3899
EN 300 -4213
EN 320 -3157
EN 340 -3484
EN 360 0
U-P:U-RIB:GLU-CA
EN 20 -7373
EN 40 -3706
EN 60 -944
EN 80 -687
EN 100 -1623
EN 120 -231
EN 140 379
EN 160 1349
EN 180 0
EN 200 0
EN 220 -2191
EN 240 739
EN 260 -1361
EN 280 766
EN 300 763
EN 320 -1183
EN 340 -1974
EN 360 -766
U-P:U-RIB:TRP-CA
EN 20 0
EN 40 0
EN 60 -7854
EN 80 -4332
EN 100 0
EN 120 -3395
EN 140 -3987
EN 160 -4717
EN 180 0
EN 200 0
EN 220 -7683
EN 240 -6753
EN 260 -5767
EN 280 -3525
EN 300 -5274
EN 320 0
EN 340 -4525
EN 360 0
G-RIB:G-R5:SER-CA
EN 20 -5900
EN 40 -3402
EN 60 -4507
EN 80 -3002
EN 100 -753
EN 120 -2831
EN 140 -3398
EN 160 -937
EN 180 -3103
EN 200 -4307
EN 220 -1591
EN 240 -2975
EN 260 -2908

EN 280 -2880
EN 300 -3406
EN 320 -3437
EN 340 -3506
EN 360 0
U-P:U-RIB:GLU-S2
EN 20 0
EN 40 0
EN 60 922
EN 80 1620
EN 100 745
EN 120 -122
EN 140 170
EN 160 721
EN 180 -1278
EN 200 0
EN 220 674
EN 240 -741
EN 260 1618
EN 280 -2262
EN 300 -128
EN 320 1246
EN 340 -1550
EN 360 -1366
U-RIB:U-Y:TYR-S1
EN 20 0
EN 40 0
EN 60 -3659
EN 80 -4616
EN 100 -2825
EN 120 -4257
EN 140 -2762
EN 160 0
EN 180 0
EN 200 0
EN 220 0
EN 240 -5510
EN 260 -3081
EN 280 -5925
EN 300 -7453
EN 320 -5797
EN 340 0
EN 360 0
C-P:C-RIB:LYS-S2
EN 20 -7693
EN 40 -6593
EN 60 -3526
EN 80 -3178
EN 100 -2985
EN 120 -3724
EN 140 -2071
EN 160 -2345
EN 180 -3419
EN 200 -6127
EN 220 -6165
EN 240 -2483
EN 260 -3084
EN 280 -3090

EN 300 -3725
EN 320 -2323
EN 340 -1543
EN 360 -1358
A-P:A-RIB:PHE-S2
EN 20 0
EN 40 -5172
EN 60 -2608
EN 80 -6325
EN 100 -5116
EN 120 -3902
EN 140 -4845
EN 160 -3878
EN 180 -6107
EN 200 0
EN 220 0
EN 240 0
EN 260 -4916
EN 280 -3983
EN 300 -5631
EN 320 0
EN 340 -3944
EN 360 -6849
A-RIB:A-R5:PRO-S1
EN 20 -7234
EN 40 -1206
EN 60 -1518
EN 80 -3913
EN 100 -4482
EN 120 -4037
EN 140 -4653
EN 160 0
EN 180 0
EN 200 0
EN 220 -5067
EN 240 -4790
EN 260 -3285
EN 280 -4454
EN 300 -3897
EN 320 -4122
EN 340 -1676
EN 360 -3914
U-RIB:U-Y:PRO-S1
EN 20 -6849
EN 40 -3434
EN 60 -3771
EN 80 -3899
EN 100 -2759
EN 120 -253
EN 140 -4188
EN 160 -1735
EN 180 -4008
EN 200 0
EN 220 -1314
EN 240 -4165
EN 260 -3888
EN 280 -3053
EN 300 -4827

EN 320 0
EN 340 -3431
EN 360 -4230
U-P:U-RIB:MET-S2
EN 20 0
EN 40 0
EN 60 0
EN 80 -6264
EN 100 -4127
EN 120 -5578
EN 140 0
EN 160 -3207
EN 180 -7447
EN 200 0
EN 220 0
EN 240 -2800
EN 260 -5274
EN 280 -1792
EN 300 -2108
EN 320 -2580
EN 340 -3101
EN 360 0
A-RIB:A-P:CYS-CA
EN 20 0
EN 40 -11174
EN 60 0
EN 80 -4925
EN 100 -5656
EN 120 -3684
EN 140 -4000
EN 160 0
EN 180 0
EN 200 0
EN 220 0
EN 240 -6772
EN 260 -7012
EN 280 -4063
EN 300 0
EN 320 0
EN 340 0
EN 360 -7683
FHU-RIB:FHU-MY:TYR-S2
EN 20 0
EN 40 0
EN 60 -12294
EN 80 0
EN 100 -10958
EN 120 0
EN 140 0
EN 160 0
EN 180 0
EN 200 0
EN 220 0
EN 240 -15398
EN 260 0
EN 280 0
EN 300 -11174
EN 320 0

EN 340 0
EN 360 0
A-RIB:A-P:PHE-S1
EN 20 0
EN 40 0
EN 60 -5263
EN 80 -4706
EN 100 -4357
EN 120 -2355
EN 140 -1245
EN 160 -1978
EN 180 0
EN 200 0
EN 220 -8903
EN 240 -3866
EN 260 -5858
EN 280 -2705
EN 300 -5740
EN 320 -4704
EN 340 -2185
EN 360 -4405
G-RIB:G-P:GLU-S1
EN 20 0
EN 40 -429
EN 60 2370
EN 80 -459
EN 100 -416
EN 120 -897
EN 140 -1289
EN 160 -2107
EN 180 1288
EN 200 0
EN 220 -2980
EN 240 711
EN 260 -1166
EN 280 4760
EN 300 336
EN 320 -47
EN 340 -879
EN 360 -454
A-RIB:A-R6:ASN-CA
EN 20 0
EN 40 0
EN 60 -3118
EN 80 -2633
EN 100 -4167
EN 120 -5187
EN 140 -3141
EN 160 -4593
EN 180 -3914
EN 200 0
EN 220 -7665
EN 240 -1325
EN 260 -4299
EN 280 -4170
EN 300 -3811
EN 320 -328
EN 340 -3187

EN 360 0
U-RIB:U-Y:MET-S1
EN 20 0
EN 40 -4925
EN 60 -6755
EN 80 0
EN 100 -3139
EN 120 0
EN 140 0
EN 160 0
EN 180 0
EN 200 -8306
EN 220 0
EN 240 -5684
EN 260 -3659
EN 280 -5503
EN 300 0
EN 320 -3878
EN 340 -5260
EN 360 0
U-RIB:U-P:MET-S1
EN 20 0
EN 40 -8271
EN 60 -3878
EN 80 -3987
EN 100 -5274
EN 120 -3105
EN 140 0
EN 160 -3008
EN 180 -5253
EN 200 0
EN 220 -8903
EN 240 -6785
EN 260 -2587
EN 280 -3262
EN 300 -3207
EN 320 -4409
EN 340 -4405
EN 360 0
U-RIB:U-P:PRO-CA
EN 20 0
EN 40 -4235
EN 60 -901
EN 80 -3523
EN 100 -4437
EN 120 -3194
EN 140 -1697
EN 160 -4767
EN 180 -5858
EN 200 0
EN 220 -4180
EN 240 -5097
EN 260 251
EN 280 -3416
EN 300 -2328
EN 320 -926
EN 340 -4469
EN 360 0

G-RIB:G-R5:GLU-S1
EN 20 0
EN 40 -2895
EN 60 -1055
EN 80 -442
EN 100 771
EN 120 0
EN 140 -1484
EN 160 -739
EN 180 -1531
EN 200 -1442
EN 220 -1905
EN 240 -558
EN 260 1160
EN 280 -732
EN 300 -1748
EN 320 -2181
EN 340 -1822
EN 360 -1381
G-RIB:G-R5:TRP-S2
EN 20 -7231
EN 40 -4956
EN 60 0
EN 80 -3442
EN 100 -6791
EN 120 -7779
EN 140 -4805
EN 160 -5093
EN 180 0
EN 200 0
EN 220 -5192
EN 240 -4864
EN 260 -4712
EN 280 0
EN 300 -6144
EN 320 -4248
EN 340 0
EN 360 0
G-RIB:G-R5:HIS-S2
EN 20 -6504
EN 40 -2875
EN 60 -6006
EN 80 -5638
EN 100 -5138
EN 120 -5682
EN 140 -1260
EN 160 -3716
EN 180 0
EN 200 0
EN 220 -5741
EN 240 -4884
EN 260 -3338
EN 280 -5882
EN 300 -3463
EN 320 0
EN 340 -4586
EN 360 0
A-RIB:A-R6:LEU-S2

EN 20 0
EN 40 -4025
EN 60 -1509
EN 80 -4916
EN 100 -5587
EN 120 -3467
EN 140 -4519
EN 160 -1664
EN 180 -5868
EN 200 0
EN 220 -3751
EN 240 -4252
EN 260 -5036
EN 280 -5702
EN 300 -6450
EN 320 -4105
EN 340 -4993
EN 360 0
A-RIB:A-P:TYR-S2
EN 20 0
EN 40 -9456
EN 60 -6050
EN 80 -5664
EN 100 -3247
EN 120 -2105
EN 140 -5226
EN 160 -1792
EN 180 -4037
EN 200 0
EN 220 -8918
EN 240 -7465
EN 260 -5081
EN 280 -2433
EN 300 -4581
EN 320 -4536
EN 340 -3872
EN 360 -4479
U-P:U-RIB:ASN-CA
EN 20 -10324
EN 40 -3797
EN 60 -2811
EN 80 -3977
EN 100 -2443
EN 120 -3581
EN 140 -3517
EN 160 -4190
EN 180 -5323
EN 200 -8236
EN 220 -6685
EN 240 -4965
EN 260 -3165
EN 280 -3432
EN 300 -3442
EN 320 -4281
EN 340 -3081
EN 360 -6553
C-RIB:C-Y:ALA-S1
EN 20 -3379

EN 40 -3752
EN 60 -3251
EN 80 -3443
EN 100 -3187
EN 120 -1888
EN 140 -1364
EN 160 -3869
EN 180 0
EN 200 -6563
EN 220 -4440
EN 240 -4500
EN 260 -3786
EN 280 -1361
EN 300 -4382
EN 320 -3610
EN 340 -2365
EN 360 0
C-P:C-RIB:ALA-S1
EN 20 -5619
EN 40 -4127
EN 60 -3273
EN 80 -3028
EN 100 -3485
EN 120 -2427
EN 140 -3202
EN 160 -2534
EN 180 -4506
EN 200 -5243
EN 220 -2836
EN 240 -4007
EN 260 -3792
EN 280 -3230
EN 300 -4078
EN 320 -3150
EN 340 -3041
EN 360 -426
A-RIB:A-P:HIS-CA
EN 20 0
EN 40 -8452
EN 60 -7301
EN 80 -5132
EN 100 -4107
EN 120 -3356
EN 140 -4728
EN 160 -3221
EN 180 -5717
EN 200 0
EN 220 -6810
EN 240 -5552
EN 260 -2854
EN 280 -2966
EN 300 -3997
EN 320 -3271
EN 340 -3071
EN 360 -5525
C-RIB:C-Y:ILE-S1
EN 20 -5158
EN 40 -4080

EN 60 -3332
EN 80 -4276
EN 100 -4179
EN 120 -3483
EN 140 -3894
EN 160 0
EN 180 0
EN 200 0
EN 220 -5112
EN 240 -5163
EN 260 -3142
EN 280 -4931
EN 300 -4174
EN 320 -1813
EN 340 0
EN 360 0
5BU-RIB:5BU-P:ILE-CA
EN 20 0
EN 40 0
EN 60 0
EN 80 0
EN 100 0
EN 120 -12675
EN 140 0
EN 160 0
EN 180 0
EN 200 0
EN 220 0
EN 240 0
EN 260 0
EN 280 0
EN 300 -14393
EN 320 -17115
EN 340 0
EN 360 0
C-P:C-RIB:ASN-CA
EN 20 -9456
EN 40 -3110
EN 60 -3500
EN 80 -5117
EN 100 -4307
EN 120 -3695
EN 140 -3920
EN 160 -2183
EN 180 -4008
EN 200 0
EN 220 -5468
EN 240 -4439
EN 260 -3979
EN 280 -1903
EN 300 -3979
EN 320 -3591
EN 340 -2786
EN 360 -2830
G-P:G-RIB:PHE-S2
EN 20 0
EN 40 0
EN 60 0

EN 80 -625
EN 100 -443
EN 120 -5365
EN 140 -4494
EN 160 -1934
EN 180 -5911
EN 200 0
EN 220 0
EN 240 -4414
EN 260 -4565
EN 280 -5485
EN 300 0
EN 320 -3797
EN 340 -5641
EN 360 -4540
A-P:A-RIB:ASN-S2
EN 20 0
EN 40 -1436
EN 60 -1241
EN 80 -4735
EN 100 -5206
EN 120 -2188
EN 140 -4282
EN 160 -4366
EN 180 -5533
EN 200 -5337
EN 220 -3910
EN 240 -4462
EN 260 -4671
EN 280 -2900
EN 300 -3003
EN 320 -2748
EN 340 -4523
EN 360 -3971
U-RIB:U-P:PHE-S2
EN 20 0
EN 40 -7186
EN 60 -5295
EN 80 0
EN 100 -3706
EN 120 -4889
EN 140 -1414
EN 160 -2120
EN 180 0
EN 200 0
EN 220 -9107
EN 240 -8931
EN 260 -3444
EN 280 -786
EN 300 -4214
EN 320 0
EN 340 0
EN 360 -4525
U-P:U-RIB:HIS-CA
EN 20 0
EN 40 -5560
EN 60 -2665
EN 80 -5241

EN 100 -6882
EN 120 -3372
EN 140 -2680
EN 160 -2161
EN 180 0
EN 200 0
EN 220 0
EN 240 0
EN 260 -4093
EN 280 -3634
EN 300 -2524
EN 320 -4285
EN 340 -5911
EN 360 -4405
C-P:C-RIB:ASP-CA
EN 20 0
EN 40 -1499
EN 60 -3089
EN 80 -96
EN 100 -3403
EN 120 -3298
EN 140 -1384
EN 160 294
EN 180 -3335
EN 200 0
EN 220 -1261
EN 240 -345
EN 260 -2778
EN 280 -1411
EN 300 -2729
EN 320 -2117
EN 340 -3386
EN 360 -3801
G-RIB:G-P:CYS-S1
EN 20 0
EN 40 0
EN 60 0
EN 80 -3598
EN 100 0
EN 120 -2517
EN 140 -2807
EN 160 0
EN 180 0
EN 200 0
EN 220 0
EN 240 0
EN 260 0
EN 280 0
EN 300 0
EN 320 -3415
EN 340 0
EN 360 -5755
A-RIB:A-R6:HIS-S1
EN 20 -10958
EN 40 0
EN 60 -5056
EN 80 -4822
EN 100 -2723

EN 120 -5071
EN 140 -1578
EN 160 -5069
EN 180 0
EN 200 0
EN 220 -5402
EN 240 -7370
EN 260 -4748
EN 280 -5899
EN 300 -1155
EN 320 -5581
EN 340 -2456
EN 360 -7278
U-RIB:U-P:SER-S1
EN 20 -5993
EN 40 -3366
EN 60 -1399
EN 80 -4377
EN 100 -1881
EN 120 -3746
EN 140 -2862
EN 160 -3048
EN 180 -1692
EN 200 0
EN 220 -3567
EN 240 -3667
EN 260 -3976
EN 280 -3721
EN 300 -3449
EN 320 -5115
EN 340 -4063
EN 360 -4550
G-RIB:G-R5:ARG-S2
EN 20 -5028
EN 40 -3739
EN 60 -2877
EN 80 -4082
EN 100 -3814
EN 120 -3998
EN 140 -5576
EN 160 -3548
EN 180 -3856
EN 200 -6936
EN 220 -4490
EN 240 -2837
EN 260 -2944
EN 280 -4807
EN 300 -3210
EN 320 -3308
EN 340 -4718
EN 360 -5258
IU-RIB:IU-MY:LYS-S2
EN 20 0
EN 40 0
EN 60 0
EN 80 0
EN 100 -6772
EN 120 0

EN 140 -8124
EN 160 0
EN 180 -10297
EN 200 0
EN 220 0
EN 240 0
EN 260 -7506
EN 280 0
EN 300 -8306
EN 320 0
EN 340 -8006
EN 360 0
A-RIB:A-P:GLN-S1
EN 20 0
EN 40 -4390
EN 60 -3454
EN 80 -5210
EN 100 -4092
EN 120 -1710
EN 140 -4205
EN 160 -2833
EN 180 0
EN 200 0
EN 220 -6929
EN 240 -3847
EN 260 -3125
EN 280 -2212
EN 300 -3811
EN 320 -4381
EN 340 -4429
EN 360 0
C-RIB:C-P:GLU-CA
EN 20 0
EN 40 -2022
EN 60 -2053
EN 80 301
EN 100 -544
EN 120 -761
EN 140 -410
EN 160 893
EN 180 -3180
EN 200 0
EN 220 -4640
EN 240 1248
EN 260 156
EN 280 -1598
EN 300 -1409
EN 320 -1600
EN 340 -1381
EN 360 118
A-RIB:A-R5:LEU-S2
EN 20 0
EN 40 0
EN 60 -1139
EN 80 -5026
EN 100 -5686
EN 120 -4687
EN 140 -1960

EN 160 0
EN 180 -7203
EN 200 -4853
EN 220 -6140
EN 240 -3982
EN 260 -3514
EN 280 -6819
EN 300 -4022
EN 320 -6093
EN 340 -2879
EN 360 0
A-RIB:A-R5:VAL-CA
EN 20 0
EN 40 -5738
EN 60 -3689
EN 80 -3231
EN 100 -4908
EN 120 -4732
EN 140 -1218
EN 160 -5957
EN 180 0
EN 200 0
EN 220 -2758
EN 240 -2711
EN 260 -635
EN 280 -4345
EN 300 -4090
EN 320 -4173
EN 340 -2548
EN 360 0
U34-RIB:U34-P:ASN-S2
EN 20 0
EN 40 0
EN 60 0
EN 80 0
EN 100 -8772
EN 120 0
EN 140 0
EN 160 0
EN 180 0
EN 200 0
EN 220 0
EN 240 -10095
EN 260 -9692
EN 280 0
EN 300 0
EN 320 0
EN 340 0
EN 360 0
A-RIB:A-R6:ASP-CA
EN 20 0
EN 40 -4878
EN 60 81
EN 80 -391
EN 100 -2733
EN 120 -1145
EN 140 -2336
EN 160 -2601

EN 180	0
EN 200	0
EN 220	0
EN 240	237
EN 260	-1189
EN 280	114
EN 300	-3663
EN 320	-756
EN 340	163
EN 360	-2045
A-RIB:A-R5:LYS-S2
EN 20 -1208
EN 40 -2447
EN 60 -4760
EN 80 -2988
EN 100	-3516
EN 120	-3700
EN 140	-3665
EN 160	-1764
EN 180	0
EN 200	-4884
EN 220	-3365
EN 240	-3836
EN 260	-3433
EN 280	-4299
EN 300	-4326
EN 320	-3870
EN 340	-2392
EN 360	0
G-P:G-RIB:ASN-CA
EN 20 -8859
EN 40 -6505
EN 60 -4851
EN 80 -3576
EN 100	-3575
EN 120	-3328
EN 140	-3060
EN 160	-5448
EN 180	-3140
EN 200	0
EN 220	-5373
EN 240	-5069
EN 260	-2589
EN 280	-3641
EN 300	-3926
EN 320	-3464
EN 340	-4871
EN 360	-3118
C-RIB:C-P:PHE-S1
EN 20 0
EN 40 -6107
EN 60 0
EN 80 -4696
EN 100	0
EN 120	-1540
EN 140	-2985
EN 160	-4000
EN 180	0

EN 200 0
EN 220 0
EN 240 -4517
EN 260 -4190
EN 280 -1815
EN 300 -3407
EN 320 0
EN 340 -3906
EN 360 0
A-RIB:A-P:GLU-S1
EN 20 0
EN 40 -3158
EN 60 -3258
EN 80 -1805
EN 100 1952
EN 120 -1751
EN 140 -1425
EN 160 2579
EN 180 0
EN 200 0
EN 220 -1517
EN 240 -1376
EN 260 -1449
EN 280 -761
EN 300 -1093
EN 320 -638
EN 340 -874
EN 360 249
G-P:G-RIB:GLN-CA
EN 20 0
EN 40 -4871
EN 60 -4503
EN 80 -4968
EN 100 -3664
EN 120 -4217
EN 140 -5434
EN 160 -5375
EN 180 -5871
EN 200 -8688
EN 220 -4793
EN 240 -2085
EN 260 -3759
EN 280 -549
EN 300 -2382
EN 320 -3952
EN 340 -5057
EN 360 -2410
G-RIB:G-P:GLY-CA
EN 20 -4998
EN 40 -4096
EN 60 -3773
EN 80 -4041
EN 100 -2798
EN 120 -2928
EN 140 -2976
EN 160 -2490
EN 180 -4103
EN 200 0

EN 220 -5250
EN 240 -5039
EN 260 -3075
EN 280 -3664
EN 300 -3210
EN 320 -2504
EN 340 -1356
EN 360 -1052
G-RIB:G-R5:TYR-CA
EN 20 0
EN 40 -6089
EN 60 0
EN 80 -6168
EN 100 0
EN 120 -2970
EN 140 -3340
EN 160 -4187
EN 180 -6772
EN 200 -8102
EN 220 0
EN 240 -4521
EN 260 -5576
EN 280 0
EN 300 -4298
EN 320 -6385
EN 340 0
EN 360 0
U-P:U-RIB:GLN-S2
EN 20 0
EN 40 -5108
EN 60 -2270
EN 80 -2934
EN 100 -3914
EN 120 -3498
EN 140 -2798
EN 160 -3482
EN 180 -3582
EN 200 0
EN 220 -3207
EN 240 -4842
EN 260 -2369
EN 280 -3677
EN 300 -2994
EN 320 -2039
EN 340 -4347
EN 360 0
A-RIB:A-R5:HIS-S2
EN 20 -5680
EN 40 -5631
EN 60 -4351
EN 80 -2281
EN 100 -5288
EN 120 -5742
EN 140 -4313
EN 160 -2731
EN 180 0
EN 200 -7186
EN 220 -5387

EN 240 -5533
EN 260 -6500
EN 280 -4802
EN 300 -5822
EN 320 -5810
EN 340 -2809
EN 360 0
A-RIB:A-P:MET-S2
EN 20 -9139
EN 40 -5055
EN 60 -6344
EN 80 -3162
EN 100 -1216
EN 120 -2724
EN 140 -3003
EN 160 -1951
EN 180 0
EN 200 0
EN 220 0
EN 240 -5514
EN 260 -3347
EN 280 -4928
EN 300 -1055
EN 320 -3118
EN 340 -2039
EN 360 0
G-P:G-RIB:LEU-CA
EN 20 0
EN 40 -3271
EN 60 -3075
EN 80 -2317
EN 100 -4419
EN 120 -3205
EN 140 -3783
EN 160 -2685
EN 180 -5905
EN 200 0
EN 220 -3454
EN 240 -4919
EN 260 -766
EN 280 -4977
EN 300 -2587
EN 320 -3384
EN 340 -2724
EN 360 -5351
A-P:A-RIB:TRP-S1
EN 20 0
EN 40 -6352
EN 60 0
EN 80 0
EN 100 -4548
EN 120 -4595
EN 140 -6459
EN 160 -3505
EN 180 -5730
EN 200 0
EN 220 0
EN 240 0

EN 260 -4571
EN 280 -4347
EN 300 -4318
EN 320 -4651
EN 340 -6040
EN 360 0
U31-RIB:U31-MY:ASP-CA
EN 20 0
EN 40 0
EN 60 0
EN 80 -6660
EN 100 -6734
EN 120 -6320
EN 140 0
EN 160 0
EN 180 0
EN 200 0
EN 220 0
EN 240 0
EN 260 0
EN 280 0
EN 300 0
EN 320 0
EN 340 0
EN 360 0
C-P:C-RIB:PRO-CA
EN 20 0
EN 40 -3684
EN 60 -5152
EN 80 -4367
EN 100 -3663
EN 120 -3023
EN 140 -1919
EN 160 -915
EN 180 -2626
EN 200 0
EN 220 -3872
EN 240 -3376
EN 260 -2834
EN 280 -3999
EN 300 -2573
EN 320 -4325
EN 340 -2984
EN 360 -810
G-P:G-RIB:GLU-CA
EN 20 0
EN 40 0
EN 60 2200
EN 80 -744
EN 100 244
EN 120 -1558
EN 140 -1247
EN 160 -949
EN 180 -981
EN 200 0
EN 220 -2257
EN 240 504
EN 260 1669

EN 280 -1510
EN 300 -1929
EN 320 -1290
EN 340 767
EN 360 -2143
U-P:U-RIB:ILE-CA
EN 20 0
EN 40 0
EN 60 0
EN 80 -3471
EN 100 -1712
EN 120 -3921
EN 140 -3723
EN 160 -1074
EN 180 0
EN 200 0
EN 220 -4943
EN 240 0
EN 260 -631
EN 280 -3625
EN 300 -83
EN 320 -3350
EN 340 -1405
EN 360 -3630
A-P:A-RIB:MET-S2
EN 20 0
EN 40 -3987
EN 60 -2108
EN 80 -1673
EN 100 -5397
EN 120 -3216
EN 140 -3395
EN 160 -3914
EN 180 0
EN 200 0
EN 220 -4434
EN 240 -2403
EN 260 -4239
EN 280 -3067
EN 300 -3991
EN 320 -1405
EN 340 -3785
EN 360 0
G-RIB:G-R5:TYR-S2
EN 20 0
EN 40 -2901
EN 60 -5083
EN 80 -3109
EN 100 -7194
EN 120 -4482
EN 140 0
EN 160 0
EN 180 0
EN 200 0
EN 220 0
EN 240 -5304
EN 260 -3219
EN 280 -5633

EN 300 -4648
EN 320 -3883
EN 340 -5045
EN 360 -5680
U-RIB:U-P:LEU-S2
EN 20 -9042
EN 40 -7083
EN 60 -4318
EN 80 -1873
EN 100 538
EN 120 -3950
EN 140 -3596
EN 160 -4324
EN 180 0
EN 200 0
EN 220 -9081
EN 240 -3625
EN 260 -4827
EN 280 -3983
EN 300 -2909
EN 320 -3189
EN 340 -3253
EN 360 0
G-RIB:G-R5:SER-S1
EN 20 -3060
EN 40 -3355
EN 60 -4047
EN 80 -2216
EN 100 -3637
EN 120 -3325
EN 140 -3560
EN 160 -2001
EN 180 -4132
EN 200 -4881
EN 220 -2122
EN 240 -2956
EN 260 -3158
EN 280 -2236
EN 300 -3010
EN 320 -2198
EN 340 -3572
EN 360 -2613
C-RIB:C-Y:PRO-S1
EN 20 -4922
EN 40 -1840
EN 60 -4346
EN 80 -3570
EN 100 -4293
EN 120 -3321
EN 140 -2038
EN 160 -3110
EN 180 0
EN 200 -6527
EN 220 -3187
EN 240 -3089
EN 260 -3345
EN 280 -4299
EN 300 -1782

EN 320 -1895
EN 340 -1455
EN 360 0
A-RIB:A-P:GLY-CA
EN 20 0
EN 40 -5705
EN 60 -5153
EN 80 -3072
EN 100 -4296
EN 120 -3681
EN 140 -3933
EN 160 -3853
EN 180 -2769
EN 200 -5656
EN 220 -7358
EN 240 -3701
EN 260 -4626
EN 280 -5043
EN 300 -3957
EN 320 -3722
EN 340 -4641
EN 360 -4922
U-P:U-RIB:ALA-S1
EN 20 -6501
EN 40 -4635
EN 60 -4688
EN 80 -4644
EN 100 -2919
EN 120 -2446
EN 140 -3268
EN 160 -4867
EN 180 0
EN 200 -4801
EN 220 -4149
EN 240 -4529
EN 260 -3503
EN 280 -4206
EN 300 -1877
EN 320 -2218
EN 340 -2274
EN 360 -3831
U-P:U-RIB:LEU-S1
EN 20 0
EN 40 0
EN 60 -5863
EN 80 -3262
EN 100 -37
EN 120 -3862
EN 140 -2167
EN 160 -3290
EN 180 -4101
EN 200 0
EN 220 0
EN 240 0
EN 260 -3905
EN 280 -2670
EN 300 -4368
EN 320 -3797

EN 340 -4093
EN 360 -3950
A-RIB:A-R6:PHE-CA
EN 20 0
EN 40 -7097
EN 60 -5619
EN 80 -2611
EN 100 -5270
EN 120 -3928
EN 140 -2662
EN 160 0
EN 180 0
EN 200 0
EN 220 0
EN 240 -5471
EN 260 -2809
EN 280 -5685
EN 300 -5215
EN 320 0
EN 340 0
EN 360 0
G-RIB:G-P:THR-CA
EN 20 0
EN 40 -7033
EN 60 -161
EN 80 -2883
EN 100 -2270
EN 120 -801
EN 140 -3998
EN 160 -4074
EN 180 -4871
EN 200 0
EN 220 -6406
EN 240 -2014
EN 260 -3645
EN 280 -2334
EN 300 -1729
EN 320 -3053
EN 340 -3621
EN 360 -2800
C-RIB:C-P:TRP-CA
EN 20 0
EN 40 0
EN 60 -4836
EN 80 -2877
EN 100 0
EN 120 -4262
EN 140 -3636
EN 160 -3084
EN 180 0
EN 200 0
EN 220 0
EN 240 -7171
EN 260 -4375
EN 280 -4728
EN 300 0
EN 320 -3884
EN 340 -4767

EN 360 0
A-P:A-RIB:MET-S1
EN 20 0
EN 40 0
EN 60 0
EN 80 -5933
EN 100 -5509
EN 120 -4271
EN 140 -4484
EN 160 -2559
EN 180 0
EN 200 0
EN 220 -5424
EN 240 -4667
EN 260 -3745
EN 280 -1494
EN 300 -5622
EN 320 -1891
EN 340 -5212
EN 360 -4979
A-RIB:A-R5:LYS-CA
EN 20 -6293
EN 40 -4690
EN 60 -3075
EN 80 -2665
EN 100 -4251
EN 120 -3172
EN 140 -1577
EN 160 -3766
EN 180 -5321
EN 200 -4560
EN 220 -3798
EN 240 -2197
EN 260 -3570
EN 280 -5171
EN 300 -2648
EN 320 -3216
EN 340 -4253
EN 360 0
GTP-RIB:GTP-M5:ASP-S2
EN 20 0
EN 40 0
EN 60 0
EN 80 0
EN 100 0
EN 120 0
EN 140 0
EN 160 0
EN 180 0
EN 200 0
EN 220 0
EN 240 0
EN 260 0
EN 280 0
EN 300 -7975
EN 320 0
EN 340 0
EN 360 0

G-RIB:G-R6:CYS-CA
EN 20 0
EN 40 -9139
EN 60 0
EN 80 -5069
EN 100 0
EN 120 -4262
EN 140 -5364
EN 160 0
EN 180 -7711
EN 200 0
EN 220 0
EN 240 0
EN 260 0
EN 280 0
EN 300 -4671
EN 320 0
EN 340 0
EN 360 0
U-RIB:U-Y:ALA-S1
EN 20 -4922
EN 40 -5613
EN 60 -3370
EN 80 -4514
EN 100 360
EN 120 -3111
EN 140 -3406
EN 160 -3984
EN 180 0
EN 200 -2966
EN 220 0
EN 240 -3779
EN 260 -2337
EN 280 -1329
EN 300 -2365
EN 320 -3388
EN 340 -2511
EN 360 0
U-P:U-RIB:TYR-CA
EN 20 0
EN 40 -6258
EN 60 0
EN 80 -5246
EN 100 -1644
EN 120 -3110
EN 140 0
EN 160 -5253
EN 180 0
EN 200 0
EN 220 0
EN 240 0
EN 260 0
EN 280 -6385
EN 300 -4704
EN 320 -4613
EN 340 -6588
EN 360 -6536
U-RIB:U-Y:MET-S2

EN 20 0
EN 40 -6171
EN 60 -3271
EN 80 -3072
EN 100 -3030
EN 120 -5881
EN 140 -3591
EN 160 0
EN 180 0
EN 200 0
EN 220 -4662
EN 240 -5202
EN 260 -2639
EN 280 -5263
EN 300 0
EN 320 -3967
EN 340 -4998
EN 360 0
FHU-RIB:FHU-P:GLY-CA
EN 20 0
EN 40 0
EN 60 0
EN 80 -8949
EN 100 0
EN 120 0
EN 140 0
EN 160 0
EN 180 0
EN 200 0
EN 220 0
EN 240 0
EN 260 -10576
EN 280 -9819
EN 300 -8772
EN 320 -8236
EN 340 0
EN 360 0
A-RIB:A-P:LYS-S1
EN 20 0
EN 40 -5147
EN 60 -2405
EN 80 -3713
EN 100 -5133
EN 120 -4751
EN 140 -4586
EN 160 -4481
EN 180 -5468
EN 200 0
EN 220 0
EN 240 -3766
EN 260 -4975
EN 280 -3709
EN 300 -3396
EN 320 -4111
EN 340 -2423
EN 360 -5177
C31-P:C31-RIB:SER-CA
EN 20 0

EN 40 0
EN 60 0
EN 80 0
EN 100 0
EN 120 0
EN 140 0
EN 160 0
EN 180 0
EN 200 0
EN 220 0
EN 240 0
EN 260 0
EN 280 0
EN 300 0
EN 320 0
EN 340 -11963
EN 360 0
A-P:A-RIB:ALA-S1
EN 20 0
EN 40 -3468
EN 60 -5013
EN 80 -3110
EN 100 -4107
EN 120 -1356
EN 140 -2522
EN 160 -2209
EN 180 0
EN 200 -4318
EN 220 -5162
EN 240 -3579
EN 260 -3974
EN 280 -3155
EN 300 -2739
EN 320 -3999
EN 340 -421
EN 360 -2842
C-RIB:C-P:LEU-CA
EN 20 0
EN 40 -6606
EN 60 -5085
EN 80 -4532
EN 100 -2449
EN 120 -3923
EN 140 -2213
EN 160 -977
EN 180 0
EN 200 0
EN 220 -6734
EN 240 -3890
EN 260 -4132
EN 280 -3557
EN 300 -2214
EN 320 -1559
EN 340 856
EN 360 -3415
A-RIB:A-P:LEU-S2
EN 20 0
EN 40 -3424

EN 60 -4441
EN 80 -2577
EN 100 -2002
EN 120 -873
EN 140 -2185
EN 160 -2856
EN 180 0
EN 200 0
EN 220 -3878
EN 240 -5249
EN 260 -1531
EN 280 -3632
EN 300 -3053
EN 320 -4202
EN 340 -3715
EN 360 -5337
C-RIB:C-Y:HIS-CA
EN 20 0
EN 40 -6849
EN 60 -2282
EN 80 -4673
EN 100 -3806
EN 120 -3971
EN 140 -4409
EN 160 -3938
EN 180 -6374
EN 200 0
EN 220 -7639
EN 240 -6289
EN 260 -6003
EN 280 -3862
EN 300 -4138
EN 320 -4517
EN 340 0
EN 360 0
G-P:G-RIB:LYS-CA
EN 20 -8688
EN 40 -4657
EN 60 -4331
EN 80 -4298
EN 100 -4504
EN 120 -3271
EN 140 -902
EN 160 -2959
EN 180 -3788
EN 200 -7278
EN 220 -6444
EN 240 -2130
EN 260 -4713
EN 280 -2025
EN 300 -3766
EN 320 -3725
EN 340 -2703
EN 360 -2994
G-RIB:G-R5:ALA-S1
EN 20 0
EN 40 -3833
EN 60 -939

EN 80 -1476
EN 100 -3381
EN 120 1375
EN 140 -2597
EN 160 -1491
EN 180 -4995
EN 200 -2969
EN 220 -3913
EN 240 -3550
EN 260 -3275
EN 280 -2268
EN 300 -2061
EN 320 -1900
EN 340 95
EN 360 -2382
A-P:A-RIB:SER-S1
EN 20 -7523
EN 40 -5236
EN 60 -2562
EN 80 -4419
EN 100 -4127
EN 120 -1543
EN 140 -1742
EN 160 -2219
EN 180 -2569
EN 200 -4587
EN 220 -6492
EN 240 -3880
EN 260 -3554
EN 280 -4304
EN 300 -5070
EN 320 -3447
EN 340 -2789
EN 360 -4483
G-RIB:G-R5:LEU-S1
EN 20 0
EN 40 -1818
EN 60 -3162
EN 80 -4365
EN 100 -4188
EN 120 -2469
EN 140 -3995
EN 160 -4141
EN 180 0
EN 200 -5003
EN 220 -4590
EN 240 -1276
EN 260 -6056
EN 280 -4395
EN 300 -2610
EN 320 -4031
EN 340 -1985
EN 360 0
C-RIB:C-P:PRO-CA
EN 20 0
EN 40 -6943
EN 60 -3283
EN 80 -2866

EN 100 -2499
EN 120 -4043
EN 140 -3808
EN 160 -2955
EN 180 -3050
EN 200 0
EN 220 0
EN 240 -3422
EN 260 -3807
EN 280 -4621
EN 300 -2219
EN 320 -2630
EN 340 -4113
EN 360 -4975
U-RIB:U-P:TYR-S1
EN 20 0
EN 40 0
EN 60 -8195
EN 80 -2155
EN 100 -1358
EN 120 -955
EN 140 -3759
EN 160 0
EN 180 0
EN 200 0
EN 220 0
EN 240 0
EN 260 -5767
EN 280 -2978
EN 300 -3882
EN 320 0
EN 340 0
EN 360 0
A-RIB:A-R5:LEU-CA
EN 20 -8528
EN 40 -3378
EN 60 -4659
EN 80 -6161
EN 100 -5585
EN 120 -4761
EN 140 -2131
EN 160 -3671
EN 180 0
EN 200 0
EN 220 -3246
EN 240 -2128
EN 260 -4771
EN 280 -2870
EN 300 -5237
EN 320 -3542
EN 340 -5998
EN 360 0
C-P:C-RIB:GLU-CA
EN 20 -6553
EN 40 -2724
EN 60 -1676
EN 80 -1506
EN 100 -567

EN 120 1802
EN 140 -2213
EN 160 -1266
EN 180 -1088
EN 200 -6385
EN 220 -2687
EN 240 1711
EN 260 -347
EN 280 -854
EN 300 -2478
EN 320 -753
EN 340 -1899
EN 360 -2171
H2U-P:H2U-RIB:LYS-S2
EN 20 0
EN 40 0
EN 60 0
EN 80 0
EN 100 0
EN 120 0
EN 140 0
EN 160 0
EN 180 0
EN 200 0
EN 220 0
EN 240 0
EN 260 0
EN 280 0
EN 300 -7325
EN 320 0
EN 340 0
EN 360 0
C-RIB:C-P:LYS-S1
EN 20 -5780
EN 40 -2440
EN 60 -4053
EN 80 -2564
EN 100 -3033
EN 120 -2956
EN 140 -4482
EN 160 -2934
EN 180 -4581
EN 200 0
EN 220 -4532
EN 240 -3408
EN 260 -3189
EN 280 -4454
EN 300 -3034
EN 320 -5079
EN 340 -4268
EN 360 -5676
G-RIB:G-R6:ARG-S1
EN 20 0
EN 40 -3622
EN 60 -3351
EN 80 -4608
EN 100 -4028
EN 120 -3418

EN 140 -3424
EN 160 -4294
EN 180 0
EN 200 0
EN 220 -4112
EN 240 -3434
EN 260 -3402
EN 280 -3442
EN 300 -4559
EN 320 -2970
EN 340 -3421
EN 360 -5312
C-RIB:C-P:CYS-CA
EN 20 0
EN 40 0
EN 60 0
EN 80 -6553
EN 100 0
EN 120 0
EN 140 -3207
EN 160 0
EN 180 -6889
EN 200 0
EN 220 0
EN 240 -6772
EN 260 -4750
EN 280 -3706
EN 300 0
EN 320 -6440
EN 340 0
EN 360 0
A-P:A-RIB:PRO-S1
EN 20 0
EN 40 -1787
EN 60 -4993
EN 80 -2315
EN 100 -3853
EN 120 -4905
EN 140 -3761
EN 160 -4406
EN 180 -1472
EN 200 0
EN 220 0
EN 240 -2320
EN 260 -2777
EN 280 -4203
EN 300 -2546
EN 320 -3632
EN 340 -3238
EN 360 -5519
FHU-P:FHU-RIB:ALA-CA
EN 20 0
EN 40 0
EN 60 0
EN 80 -10760
EN 100 0
EN 120 -10760
EN 140 0

EN 160 0
EN 180 0
EN 200 0
EN 220 0
EN 240 0
EN 260 0
EN 280 0
EN 300 -13127
EN 320 0
EN 340 0
EN 360 0
U34-RIB:U34-P:ASN-S1
EN 20 0
EN 40 0
EN 60 -14393
EN 80 0
EN 100 -10095
EN 120 0
EN 140 0
EN 160 0
EN 180 0
EN 200 0
EN 220 0
EN 240 0
EN 260 -8772
EN 280 -9692
EN 300 0
EN 320 0
EN 340 0
EN 360 0
U-RIB:U-Y:GLN-S2
EN 20 -3979
EN 40 -2972
EN 60 -4553
EN 80 -2153
EN 100 -2649
EN 120 -3567
EN 140 -5141
EN 160 -4928
EN 180 -4167
EN 200 -6496
EN 220 -1230
EN 240 -3409
EN 260 -4345
EN 280 -4481
EN 300 -3240
EN 320 -481
EN 340 -3556
EN 360 -3797
C31-P:C31-RIB:PHE-S1
EN 20 0
EN 40 0
EN 60 0
EN 80 0
EN 100 -9571
EN 120 -11671
EN 140 0
EN 160 0

EN 180 0
EN 200 0
EN 220 0
EN 240 0
EN 260 0
EN 280 0
EN 300 0
EN 320 0
EN 340 0
EN 360 0
C-RIB:C-Y:LEU-CA
EN 20 -8528
EN 40 -4807
EN 60 -5004
EN 80 -4598
EN 100 -3570
EN 120 -6150
EN 140 -3957
EN 160 -4176
EN 180 0
EN 200 0
EN 220 -2722
EN 240 -5130
EN 260 -4078
EN 280 -4631
EN 300 -711
EN 320 -1219
EN 340 -4080
EN 360 -4744
A-RIB:A-R5:ARG-CA
EN 20 -4845
EN 40 -5125
EN 60 -5142
EN 80 -5615
EN 100 -5851
EN 120 -1269
EN 140 -5010
EN 160 -2898
EN 180 0
EN 200 -6417
EN 220 -4761
EN 240 -4988
EN 260 -6342
EN 280 -2972
EN 300 -5161
EN 320 -5635
EN 340 -4713
EN 360 -3665
U-RIB:U-Y:PHE-S2
EN 20 0
EN 40 -6929
EN 60 -5391
EN 80 -3008
EN 100 -5310
EN 120 -2961
EN 140 -5023
EN 160 0
EN 180 0

EN 200 0
EN 220 -4973
EN 240 -7145
EN 260 -7780
EN 280 -5658
EN 300 -4703
EN 320 -4833
EN 340 0
EN 360 0
G-RIB:G-R6:HIS-S2
EN 20 -8168
EN 40 -6264
EN 60 -5237
EN 80 -5359
EN 100 -3769
EN 120 -6061
EN 140 -4195
EN 160 -1640
EN 180 0
EN 200 -7990
EN 220 -7113
EN 240 -4383
EN 260 -4038
EN 280 -3025
EN 300 -6113
EN 320 -4155
EN 340 0
EN 360 -4430
C-P:C-RIB:GLN-CA
EN 20 0
EN 40 -5871
EN 60 -4490
EN 80 -4660
EN 100 -2735
EN 120 -4037
EN 140 -3798
EN 160 -2784
EN 180 -4842
EN 200 0
EN 220 -4494
EN 240 -2709
EN 260 -4672
EN 280 -4320
EN 300 -5512
EN 320 -4573
EN 340 -5668
EN 360 -2011
U-RIB:U-Y:ARG-S1
EN 20 -5871
EN 40 -2670
EN 60 -3686
EN 80 -5666
EN 100 -5045
EN 120 -5203
EN 140 -3913
EN 160 -3257
EN 180 -4590
EN 200 0

EN 220 -3633
EN 240 -1458
EN 260 -4428
EN 280 -3063
EN 300 -1845
EN 320 -3143
EN 340 -5393
EN 360 -5615
A-P:A-RIB:LYS-S2
EN 20 -5986
EN 40 -5182
EN 60 -4897
EN 80 -2420
EN 100 -3931
EN 120 -2391
EN 140 -2578
EN 160 -1828
EN 180 -1265
EN 200 -7822
EN 220 -5728
EN 240 -5161
EN 260 -3726
EN 280 -4073
EN 300 -3403
EN 320 -2441
EN 340 -4416
EN 360 -2893
A-RIB:A-P:ASN-S2
EN 20 -6050
EN 40 -1834
EN 60 -2419
EN 80 -4495
EN 100 -4014
EN 120 -4143
EN 140 -2521
EN 160 -4256
EN 180 -1517
EN 200 0
EN 220 0
EN 240 -5431
EN 260 -3161
EN 280 -3019
EN 300 -2663
EN 320 -2542
EN 340 -3710
EN 360 0
A-RIB:A-P:MET-CA
EN 20 0
EN 40 0
EN 60 -5305
EN 80 -4640
EN 100 -4180
EN 120 -3991
EN 140 -3295
EN 160 -2510
EN 180 0
EN 200 0
EN 220 0

EN 240 -5457
EN 260 -6027
EN 280 -4004
EN 300 -3800
EN 320 -4088
EN 340 0
EN 360 0
C-RIB:C-Y:ASP-CA
EN 20 -6064
EN 40 -2387
EN 60 -2794
EN 80 -3057
EN 100 -3627
EN 120 -1730
EN 140 -3654
EN 160 -3307
EN 180 0
EN 200 0
EN 220 -4512
EN 240 -2945
EN 260 -3624
EN 280 1106
EN 300 -2959
EN 320 -4415
EN 340 -3797
EN 360 -3064
U-RIB:U-P:ARG-CA
EN 20 0
EN 40 -4540
EN 60 -3542
EN 80 -3434
EN 100 -4857
EN 120 -4420
EN 140 -5803
EN 160 -4174
EN 180 -4925
EN 200 0
EN 220 -7247
EN 240 -4056
EN 260 -2288
EN 280 -4795
EN 300 -5059
EN 320 -3537
EN 340 -3793
EN 360 -5045
G-RIB:G-R5:LEU-S2
EN 20 -5302
EN 40 -4947
EN 60 -539
EN 80 -3288
EN 100 -3366
EN 120 -4017
EN 140 -3869
EN 160 -2038
EN 180 0
EN 200 -5593
EN 220 -4506
EN 240 -3711

EN 260 -4814
EN 280 -3397
EN 300 -4118
EN 320 -4974
EN 340 0
EN 360 0
A-RIB:A-R6:ARG-CA
EN 20 0
EN 40 0
EN 60 -6146
EN 80 -4941
EN 100 -4139
EN 120 -4276
EN 140 -4497
EN 160 -3367
EN 180 0
EN 200 0
EN 220 -6352
EN 240 -6022
EN 260 -1914
EN 280 -4539
EN 300 -2089
EN 320 -4158
EN 340 -4229
EN 360 0
U-RIB:U-Y:ASN-S1
EN 20 -8102
EN 40 -2116
EN 60 -4760
EN 80 -3348
EN 100 -4380
EN 120 -4139
EN 140 -4202
EN 160 -3234
EN 180 0
EN 200 0
EN 220 -3961
EN 240 -5335
EN 260 -5214
EN 280 -4166
EN 300 -1625
EN 320 -4385
EN 340 -3133
EN 360 -5424
A-P:A-RIB:VAL-S1
EN 20 0
EN 40 -4018
EN 60 -5500
EN 80 -2123
EN 100 -3637
EN 120 -3980
EN 140 -4445
EN 160 -3948
EN 180 -3802
EN 200 0
EN 220 0
EN 240 -3107
EN 260 -2844

EN 280 -5058
EN 300 -2290
EN 320 -3061
EN 340 -3792
EN 360 -5429
G-RIB:G-P:TYR-S1
EN 20 0
EN 40 0
EN 60 -4784
EN 80 -4509
EN 100 -4556
EN 120 -4328
EN 140 -2939
EN 160 -6389
EN 180 0
EN 200 0
EN 220 0
EN 240 -2870
EN 260 -2604
EN 280 -3405
EN 300 -3295
EN 320 -4349
EN 340 -4304
EN 360 -4127
A-RIB:A-P:ILE-CA
EN 20 0
EN 40 0
EN 60 -4000
EN 80 -2417
EN 100 -2670
EN 120 -1251
EN 140 -1757
EN 160 -1014
EN 180 -3987
EN 200 0
EN 220 -5993
EN 240 -2483
EN 260 -5456
EN 280 -3079
EN 300 -1315
EN 320 -2754
EN 340 -4991
EN 360 -3774
U-RIB:U-Y:VAL-S1
EN 20 -5560
EN 40 -4639
EN 60 -2934
EN 80 -2434
EN 100 -3358
EN 120 0
EN 140 -2958
EN 160 0
EN 180 0
EN 200 0
EN 220 -5212
EN 240 -3878
EN 260 -3280
EN 280 -3383

EN 300 0
EN 320 -1372
EN 340 -2079
EN 360 0
C-RIB:C-Y:ALA-CA
EN 20 -4880
EN 40 -4737
EN 60 -3061
EN 80 -4087
EN 100 -3782
EN 120 -2880
EN 140 -1628
EN 160 -4100
EN 180 0
EN 200 -3309
EN 220 -5919
EN 240 -4377
EN 260 -3003
EN 280 -2568
EN 300 -3407
EN 320 -1669
EN 340 -3713
EN 360 0
C-P:C-RIB:PRO-S1
EN 20 -9261
EN 40 -2803
EN 60 -4653
EN 80 -4011
EN 100 -2822
EN 120 -3269
EN 140 -2685
EN 160 -2296
EN 180 -3556
EN 200 0
EN 220 -4544
EN 240 -2882
EN 260 -2979
EN 280 -3524
EN 300 -3329
EN 320 -4904
EN 340 1252
EN 360 -779
U34-P:U34-RIB:ASN-CA
EN 20 0
EN 40 0
EN 60 0
EN 80 -10760
EN 100 0
EN 120 0
EN 140 0
EN 160 0
EN 180 0
EN 200 0
EN 220 0
EN 240 -15398
EN 260 -11963
EN 280 -10760
EN 300 0

EN 320 0
EN 340 0
EN 360 0
C-RIB:C-P:ALA-S1
EN 20 0
EN 40 -6066
EN 60 -3113
EN 80 -3764
EN 100 -3757
EN 120 -1470
EN 140 -3850
EN 160 -2961
EN 180 -3508
EN 200 0
EN 220 -5355
EN 240 -4529
EN 260 -2417
EN 280 -3101
EN 300 -3881
EN 320 -3671
EN 340 -2240
EN 360 -4211
A-RIB:A-P:HIS-S2
EN 20 0
EN 40 -5656
EN 60 -6828
EN 80 -4405
EN 100 -4881
EN 120 -4211
EN 140 -3335
EN 160 -2230
EN 180 -4810
EN 200 0
EN 220 -5402
EN 240 -4063
EN 260 -4456
EN 280 -3890
EN 300 -2754
EN 320 -2535
EN 340 -5093
EN 360 -2925
C-RIB:C-Y:LEU-S1
EN 20 -5514
EN 40 -2441
EN 60 -4100
EN 80 -2859
EN 100 -5239
EN 120 -5718
EN 140 -1410
EN 160 -4189
EN 180 0
EN 200 -5424
EN 220 -5701
EN 240 -6556
EN 260 -3835
EN 280 -4325
EN 300 -2597
EN 320 -1454

EN 340 -2151
EN 360 -4813
C-RIB:C-Y:ARG-S2
EN 20 -5920
EN 40 -3261
EN 60 -4282
EN 80 -5211
EN 100 -5072
EN 120 -5162
EN 140 -3678
EN 160 -4196
EN 180 -5066
EN 200 -6540
EN 220 -4623
EN 240 -3900
EN 260 -4639
EN 280 -4948
EN 300 -4098
EN 320 -3196
EN 340 -1823
EN 360 -7326
U-RIB:U-Y:HIS-S1
EN 20 0
EN 40 -6146
EN 60 -5787
EN 80 -2017
EN 100 -4559
EN 120 -1942
EN 140 -4466
EN 160 -3560
EN 180 0
EN 200 0
EN 220 -7571
EN 240 -2977
EN 260 0
EN 280 -3670
EN 300 -4881
EN 320 -2393
EN 340 -3461
EN 360 0
U-P:U-RIB:GLY-CA
EN 20 -5966
EN 40 -4338
EN 60 -5710
EN 80 -3884
EN 100 -2370
EN 120 -3442
EN 140 -4348
EN 160 -3718
EN 180 -5521
EN 200 -6304
EN 220 -5849
EN 240 -1304
EN 260 -2202
EN 280 -4868
EN 300 -3988
EN 320 -4874
EN 340 -4232

EN 360 -3347
G-RIB:G-P:MET-CA
EN 20 0
EN 40 0
EN 60 -4449
EN 80 -4336
EN 100 -4005
EN 120 142
EN 140 -590
EN 160 0
EN 180 0
EN 200 0
EN 220 -6107
EN 240 -2594
EN 260 -1074
EN 280 -4042
EN 300 63
EN 320 -3606
EN 340 -4920
EN 360 -3706
G-P:G-RIB:ALA-CA
EN 20 -7739
EN 40 -3204
EN 60 -1706
EN 80 -3059
EN 100 -3149
EN 120 -3604
EN 140 -2363
EN 160 -3500
EN 180 -2301
EN 200 -4784
EN 220 -3107
EN 240 -2720
EN 260 -1887
EN 280 -1647
EN 300 -2702
EN 320 -2024
EN 340 -997
EN 360 -4525
C-P:C-RIB:TRP-S1
EN 20 0
EN 40 0
EN 60 -3525
EN 80 -4121
EN 100 -5988
EN 120 -4925
EN 140 0
EN 160 0
EN 180 -5755
EN 200 0
EN 220 0
EN 240 0
EN 260 -4114
EN 280 -5093
EN 300 0
EN 320 0
EN 340 -5797
EN 360 -5656

A-RIB:A-P:MET-S1
EN 20 0
EN 40 0
EN 60 -4898
EN 80 -3690
EN 100 -4110
EN 120 -3012
EN 140 -6078
EN 160 -2397
EN 180 0
EN 200 0
EN 220 0
EN 240 -3309
EN 260 -6783
EN 280 -3922
EN 300 -3105
EN 320 -3003
EN 340 0
EN 360 0
G-RIB:G-P:ARG-S2
EN 20 0
EN 40 -5134
EN 60 -3720
EN 80 -4894
EN 100 -3661
EN 120 -4240
EN 140 -4112
EN 160 -4638
EN 180 -2561
EN 200 0
EN 220 -2976
EN 240 -3348
EN 260 -3661
EN 280 -4542
EN 300 -3876
EN 320 -3731
EN 340 -4889
EN 360 -4290
C-P:C-RIB:LEU-S1
EN 20 0
EN 40 -4889
EN 60 -4494
EN 80 -4834
EN 100 -2665
EN 120 -3041
EN 140 -4514
EN 160 -4380
EN 180 -4044
EN 200 0
EN 220 0
EN 240 -3069
EN 260 -5526
EN 280 -4197
EN 300 -5302
EN 320 -3998
EN 340 -3285
EN 360 -4201
C-P:C-RIB:GLN-S2

EN 20 -4717
EN 40 -6189
EN 60 -5008
EN 80 -4114
EN 100 -3811
EN 120 -3647
EN 140 -1116
EN 160 -5111
EN 180 -897
EN 200 0
EN 220 -2905
EN 240 -3127
EN 260 -3767
EN 280 -3832
EN 300 -3474
EN 320 -4099
EN 340 -5779
EN 360 -5611
G-P:G-RIB:VAL-CA
EN 20 0
EN 40 0
EN 60 -1358
EN 80 -1768
EN 100 -645
EN 120 -2889
EN 140 -2405
EN 160 -2658
EN 180 0
EN 200 0
EN 220 -4075
EN 240 -1257
EN 260 -1723
EN 280 -1296
EN 300 -2219
EN 320 -2653
EN 340 -1918
EN 360 -3067
U-RIB:U-P:ASP-S1
EN 20 -7231
EN 40 0
EN 60 607
EN 80 -800
EN 100 -881
EN 120 -1317
EN 140 -3234
EN 160 0
EN 180 -2830
EN 200 0
EN 220 -4619
EN 240 534
EN 260 -1500
EN 280 -103
EN 300 -238
EN 320 449
EN 340 -3834
EN 360 -3535
A-RIB:A-R6:GLN-S1
EN 20 0

EN 40 -4063
EN 60 -4107
EN 80 -2901
EN 100 -3819
EN 120 -1578
EN 140 -3236
EN 160 -4577
EN 180 -5543
EN 200 0
EN 220 -4221
EN 240 -2826
EN 260 -3971
EN 280 -1532
EN 300 -3827
EN 320 -5565
EN 340 -2786
EN 360 -3606
A-RIB:A-P:LEU-S1
EN 20 0
EN 40 -4318
EN 60 -3454
EN 80 -3429
EN 100 -2054
EN 120 -751
EN 140 -2167
EN 160 -2048
EN 180 -4684
EN 200 0
EN 220 -6484
EN 240 -5055
EN 260 -3344
EN 280 -883
EN 300 -4859
EN 320 -2836
EN 340 -4224
EN 360 0
G-RIB:G-P:SER-S1
EN 20 0
EN 40 -4793
EN 60 -2758
EN 80 -2347
EN 100 -2815
EN 120 -3409
EN 140 -4680
EN 160 -4056
EN 180 -1997
EN 200 0
EN 220 -3863
EN 240 -1862
EN 260 -4104
EN 280 -3813
EN 300 -2901
EN 320 -2796
EN 340 -3849
EN 360 -2059
C-RIB:C-P:ASN-S2
EN 20 0
EN 40 -3804

EN 60 -4084
EN 80 349
EN 100 -4049
EN 120 -1620
EN 140 -4278
EN 160 -3729
EN 180 -3584
EN 200 0
EN 220 -1432
EN 240 -4912
EN 260 -3243
EN 280 -4795
EN 300 -4179
EN 320 -3253
EN 340 -4817
EN 360 -3595
G-RIB:G-R5:HIS-CA
EN 20 -7231
EN 40 -6088
EN 60 -4430
EN 80 -4575
EN 100 -3077
EN 120 -4455
EN 140 -2027
EN 160 0
EN 180 0
EN 200 -6744
EN 220 -6495
EN 240 -3241
EN 260 -5170
EN 280 -3138
EN 300 -3161
EN 320 -3768
EN 340 -4878
EN 360 0
A-P:A-RIB:PHE-S1
EN 20 0
EN 40 0
EN 60 -5055
EN 80 -5268
EN 100 -3016
EN 120 -4536
EN 140 -3149
EN 160 -3831
EN 180 -6715
EN 200 0
EN 220 0
EN 240 -3075
EN 260 -4556
EN 280 -2946
EN 300 -5120
EN 320 -4911
EN 340 -5055
EN 360 0
G-RIB:G-R6:ARG-S2
EN 20 0
EN 40 -3044
EN 60 -3484

EN 80 -3772
EN 100 -3782
EN 120 -4037
EN 140 -3374
EN 160 -2547
EN 180 -5344
EN 200 -6078
EN 220 -2335
EN 240 -3614
EN 260 -3289
EN 280 -3759
EN 300 -4150
EN 320 -3300
EN 340 -3308
EN 360 -2894
C-RIB:C-Y:TRP-CA
EN 20 0
EN 40 0
EN 60 -4489
EN 80 -3092
EN 100 -6904
EN 120 0
EN 140 -4640
EN 160 0
EN 180 0
EN 200 0
EN 220 0
EN 240 -4651
EN 260 0
EN 280 -4479
EN 300 0
EN 320 -5172
EN 340 -5656
EN 360 0
G-RIB:G-R5:ASN-S1
EN 20 -5313
EN 40 -5134
EN 60 -3995
EN 80 -4607
EN 100 -3568
EN 120 -3946
EN 140 -4277
EN 160 -4633
EN 180 -3929
EN 200 -6086
EN 220 -5246
EN 240 -2900
EN 260 -4366
EN 280 -2194
EN 300 -4303
EN 320 -3049
EN 340 -2845
EN 360 -2627
A-P:A-RIB:SER-CA
EN 20 -9776
EN 40 -6560
EN 60 -3158
EN 80 -3134

EN 100 -3915
EN 120 -1247
EN 140 -2096
EN 160 -1940
EN 180 -1172
EN 200 -6697
EN 220 -6594
EN 240 -4457
EN 260 -3497
EN 280 -3862
EN 300 -5070
EN 320 -3118
EN 340 -3145
EN 360 -2846
DA-RIB:DA-M5:SER-CA
EN 20 0
EN 40 0
EN 60 0
EN 80 0
EN 100 0
EN 120 0
EN 140 0
EN 160 0
EN 180 0
EN 200 0
EN 220 0
EN 240 0
EN 260 0
EN 280 0
EN 300 -7231
EN 320 0
EN 340 -8038
EN 360 0
G-RIB:G-P:THR-S1
EN 20 -6078
EN 40 0
EN 60 -2590
EN 80 -2293
EN 100 -2372
EN 120 -1669
EN 140 -2615
EN 160 -3117
EN 180 -4012
EN 200 -6588
EN 220 0
EN 240 -3632
EN 260 -3847
EN 280 -1182
EN 300 -2523
EN 320 -2282
EN 340 -3884
EN 360 -3337
G-P:G-RIB:ARG-S2
EN 20 -8345
EN 40 -5528
EN 60 -5366
EN 80 -4028
EN 100 -3686

EN 120	-4115
EN 140	-3392
EN 160	1461
EN 180	-2049
EN 200	-7122
EN 220	-6125
EN 240	-4713
EN 260	-4693
EN 280	-4345
EN 300	-4519
EN 320	-2571
EN 340	-3110
EN 360	-3446
H2U-RIB:H2U-MY:ASN-S2
EN 20 0
EN 40 0
EN 60 0
EN 80 0
EN 100	-8102
EN 120	0
EN 140	-8528
EN 160	0
EN 180	0
EN 200	0
EN 220	0
EN 240	0
EN 260	-10958
EN 280	0
EN 300	0
EN 320	0
EN 340	0
EN 360	0
A-RIB:A-R6:TRP-S1
EN 20 0
EN 40 0
EN 60 0
EN 80 -5771
EN 100	-3274
EN 120	-3385
EN 140	0
EN 160	0
EN 180	0
EN 200	0
EN 220	0
EN 240	-4540
EN 260	-6156
EN 280	-5245
EN 300	0
EN 320	-3525
EN 340	-4494
EN 360	0
C-RIB:C-Y:PHE-S1
EN 20 0
EN 40 0
EN 60 0
EN 80 -4651
EN 100	-4673
EN 120	-5023

EN 140 -3539
EN 160 -6379
EN 180 0
EN 200 -7201
EN 220 -4474
EN 240 0
EN 260 -5454
EN 280 -5414
EN 300 -3021
EN 320 -3369
EN 340 0
EN 360 -7777
G-P:G-RIB:ASP-CA
EN 20 -6320
EN 40 -848
EN 60 -1834
EN 80 -731
EN 100 -2125
EN 120 -3200
EN 140 -2312
EN 160 -2963
EN 180 -3323
EN 200 -6137
EN 220 -2658
EN 240 -42
EN 260 -2377
EN 280 -1415
EN 300 -1513
EN 320 -1855
EN 340 -2998
EN 360 -1747
A-RIB:A-P:TYR-CA
EN 20 0
EN 40 0
EN 60 -6507
EN 80 -4836
EN 100 -3839
EN 120 -4339
EN 140 -4880
EN 160 -5023
EN 180 0
EN 200 0
EN 220 -7325
EN 240 -5871
EN 260 -6636
EN 280 -4793
EN 300 -3634
EN 320 -5862
EN 340 -2417
EN 360 0
FMU-P:FMU-RIB:VAL-CA
EN 20 0
EN 40 0
EN 60 0
EN 80 0
EN 100 0
EN 120 0
EN 140 0

EN 160 0
EN 180 0
EN 200 0
EN 220 0
EN 240 0
EN 260 0
EN 280 -13680
EN 300 0
EN 320 0
EN 340 0
EN 360 0
FHU-RIB:FHU-P:VAL-CA
EN 20 0
EN 40 0
EN 60 0
EN 80 0
EN 100 0
EN 120 -9346
EN 140 0
EN 160 0
EN 180 0
EN 200 0
EN 220 0
EN 240 0
EN 260 0
EN 280 0
EN 300 0
EN 320 -9139
EN 340 0
EN 360 0
U-P:U-RIB:LYS-CA
EN 20 0
EN 40 -3808
EN 60 439
EN 80 -4039
EN 100 -5153
EN 120 -4282
EN 140 -2015
EN 160 -4717
EN 180 -3230
EN 200 -7975
EN 220 -5911
EN 240 -3774
EN 260 -4710
EN 280 -4362
EN 300 -716
EN 320 -3558
EN 340 -4297
EN 360 -1048
A-RIB:A-R5:CYS-CA
EN 20 0
EN 40 0
EN 60 -6258
EN 80 -5891
EN 100 -5543
EN 120 0
EN 140 0
EN 160 0

EN 180 0
EN 200 0
EN 220 0
EN 240 0
EN 260 0
EN 280 -7549
EN 300 0
EN 320 0
EN 340 0
EN 360 0
A-RIB:A-P:ARG-S1
EN 20 0
EN 40 -5248
EN 60 -5446
EN 80 -4738
EN 100 -5097
EN 120 -5264
EN 140 -4525
EN 160 -5246
EN 180 -5944
EN 200 -7141
EN 220 -3588
EN 240 -5077
EN 260 -5768
EN 280 -5043
EN 300 -5158
EN 320 -4535
EN 340 -5174
EN 360 -5328
G-RIB:G-R6:ASP-S1
EN 20 0
EN 40 -4597
EN 60 -25
EN 80 -1938
EN 100 -717
EN 120 -3006
EN 140 -1217
EN 160 -2257
EN 180 -1989
EN 200 0
EN 220 -3583
EN 240 -1799
EN 260 -2224
EN 280 -3504
EN 300 -1747
EN 320 -220
EN 340 -3925
EN 360 -3575
U-RIB:U-P:ASP-CA
EN 20 0
EN 40 -3505
EN 60 -4162
EN 80 1397
EN 100 -454
EN 120 -3040
EN 140 -2507
EN 160 -2592
EN 180 -3101

EN 200 0
EN 220 0
EN 240 -233
EN 260 -1324
EN 280 587
EN 300 -472
EN 320 -836
EN 340 -3766
EN 360 -1282
C31-RIB:C31-P:GLN-CA
EN 20 0
EN 40 0
EN 60 0
EN 80 0
EN 100 0
EN 120 0
EN 140 0
EN 160 -8772
EN 180 0
EN 200 0
EN 220 0
EN 240 0
EN 260 0
EN 280 0
EN 300 0
EN 320 0
EN 340 0
EN 360 0
G-RIB:G-R6:HIS-CA
EN 20 0
EN 40 0
EN 60 -4874
EN 80 -4311
EN 100 -3269
EN 120 -5136
EN 140 -1847
EN 160 0
EN 180 0
EN 200 0
EN 220 -8157
EN 240 -5234
EN 260 -5976
EN 280 -2234
EN 300 -5348
EN 320 -3306
EN 340 0
EN 360 0
C-P:C-RIB:GLN-S1
EN 20 -9571
EN 40 -5858
EN 60 -3462
EN 80 -5288
EN 100 -4595
EN 120 -2763
EN 140 -2909
EN 160 -3464
EN 180 -4889
EN 200 0

EN 220	-6122
EN 240	-1757
EN 260	-3912
EN 280	-3091
EN 300	-3729
EN 320	-4879
EN 340	-4909
EN 360	-6078
A-P:A-RIB:TRP-CA
EN 20 0
EN 40 0
EN 60 -4571
EN 80 0
EN 100	-4801
EN 120	-6697
EN 140	-4943
EN 160	-3684
EN 180	-6050
EN 200	0
EN 220	0
EN 240	-4419
EN 260	-4889
EN 280	-4611
EN 300	-2846
EN 320	-4742
EN 340	-5380
EN 360	0
QUO-RIB:QUO-M6:ARG-S1
EN 20 0
EN 40 0
EN 60 0
EN 80 0
EN 100	0
EN 120	-3434
EN 140	0
EN 160	0
EN 180	-3434
EN 200	0
EN 220	0
EN 240	0
EN 260	0
EN 280	0
EN 300	0
EN 320	0
EN 340	0
EN 360	0
C-RIB:C-P:ARG-S1
EN 20 -6588
EN 40 -6376
EN 60 -4548
EN 80 -4138
EN 100	-5073
EN 120	-4653
EN 140	-4298
EN 160	-4171
EN 180	-6264
EN 200	0
EN 220	-5043

EN 240 -4250
EN 260 -4482
EN 280 -5478
EN 300 -5359
EN 320 -4159
EN 340 -5078
EN 360 -4278
G-RIB:G-P:TYR-CA
EN 20 0
EN 40 0
EN 60 -5560
EN 80 -4889
EN 100 -3967
EN 120 -2399
EN 140 -2899
EN 160 -3101
EN 180 -5755
EN 200 0
EN 220 0
EN 240 -2942
EN 260 -3684
EN 280 -3241
EN 300 -2605
EN 320 -5135
EN 340 -4849
EN 360 0
A-P:A-RIB:GLY-CA
EN 20 -8747
EN 40 -6078
EN 60 -5007
EN 80 -4288
EN 100 -4616
EN 120 -3403
EN 140 -4666
EN 160 -3651
EN 180 274
EN 200 -4464
EN 220 -6286
EN 240 -4869
EN 260 -2987
EN 280 -3441
EN 300 -4938
EN 320 -4326
EN 340 -3472
EN 360 -3862
U-RIB:U-Y:TRP-S1
EN 20 0
EN 40 0
EN 60 -6836
EN 80 -4979
EN 100 -7299
EN 120 0
EN 140 0
EN 160 0
EN 180 0
EN 200 0
EN 220 -6289
EN 240 0

EN 260 -5093
EN 280 -4706
EN 300 0
EN 320 0
EN 340 -6176
EN 360 0
U-RIB:U-P:GLU-CA
EN 20 0
EN 40 -2974
EN 60 0
EN 80 -698
EN 100 -565
EN 120 -1492
EN 140 -1924
EN 160 -1136
EN 180 -2665
EN 200 0
EN 220 -3154
EN 240 -3248
EN 260 -765
EN 280 -499
EN 300 285
EN 320 -2254
EN 340 -1185
EN 360 0
FHU-P:FHU-RIB:ASP-CA
EN 20 0
EN 40 0
EN 60 0
EN 80 0
EN 100 0
EN 120 0
EN 140 -13127
EN 160 0
EN 180 0
EN 200 0
EN 220 0
EN 240 0
EN 260 0
EN 280 0
EN 300 0
EN 320 -12675
EN 340 0
EN 360 0
A-RIB:A-R6:GLY-CA
EN 20 -5838
EN 40 -6782
EN 60 -3776
EN 80 -3933
EN 100 -3093
EN 120 -3825
EN 140 -2807
EN 160 -3446
EN 180 0
EN 200 -6360
EN 220 -6430
EN 240 -4573
EN 260 -3630

EN 280 -4169
EN 300 -3546
EN 320 -2484
EN 340 -4075
EN 360 -3281
A-RIB:A-R5:GLN-S2
EN 20 -3860
EN 40 -4803
EN 60 -4534
EN 80 -4149
EN 100 -4150
EN 120 -4513
EN 140 -2245
EN 160 -4224
EN 180 0
EN 200 -5540
EN 220 -4315
EN 240 -3651
EN 260 -4392
EN 280 -2856
EN 300 -2693
EN 320 -4863
EN 340 -2997
EN 360 -5531
G-RIB:G-P:PHE-CA
EN 20 0
EN 40 0
EN 60 -4368
EN 80 -4318
EN 100 -4154
EN 120 -951
EN 140 -3671
EN 160 -4603
EN 180 0
EN 200 0
EN 220 -7854
EN 240 -6078
EN 260 -4723
EN 280 -1326
EN 300 -2907
EN 320 -2432
EN 340 -2423
EN 360 0
A-RIB:A-P:SER-CA
EN 20 0
EN 40 -2909
EN 60 -5960
EN 80 -3417
EN 100 -4174
EN 120 -3384
EN 140 -4724
EN 160 -4419
EN 180 -1605
EN 200 0
EN 220 -4548
EN 240 -3617
EN 260 -3177
EN 280 -3695

EN 300 -4282
EN 320 -3778
EN 340 -5596
EN 360 -6098
U-P:U-RIB:HIS-S2
EN 20 0
EN 40 -3515
EN 60 -1121
EN 80 -5881
EN 100 -4656
EN 120 -2901
EN 140 -4928
EN 160 -5545
EN 180 -6451
EN 200 0
EN 220 -3234
EN 240 -1086
EN 260 -4312
EN 280 -4045
EN 300 -3751
EN 320 -3960
EN 340 -1345
EN 360 -5017
DA-RIB:DA-M5:MET-S2
EN 20 0
EN 40 0
EN 60 0
EN 80 0
EN 100 0
EN 120 0
EN 140 0
EN 160 0
EN 180 0
EN 200 0
EN 220 -10958
EN 240 0
EN 260 0
EN 280 0
EN 300 0
EN 320 0
EN 340 0
EN 360 0
A-RIB:A-P:THR-S1
EN 20 -7141
EN 40 -5152
EN 60 -5248
EN 80 -3708
EN 100 -4656
EN 120 -4836
EN 140 -3978
EN 160 -3677
EN 180 -5088
EN 200 0
EN 220 -2365
EN 240 -5474
EN 260 -3233
EN 280 -4037
EN 300 -4344

EN 320 -2916
EN 340 -1922
EN 360 -1568
G-P:G-RIB:SER-S1
EN 20 -6496
EN 40 -3176
EN 60 -3665
EN 80 -4104
EN 100 -3490
EN 120 -1255
EN 140 -3633
EN 160 -2044
EN 180 -2729
EN 200 -6385
EN 220 -4931
EN 240 -1912
EN 260 -2325
EN 280 -3200
EN 300 -2633
EN 320 -3082
EN 340 -3595
EN 360 -4417
U31-RIB:U31-MY:GLN-S1
EN 20 0
EN 40 0
EN 60 -10760
EN 80 0
EN 100 0
EN 120 0
EN 140 0
EN 160 0
EN 180 0
EN 200 0
EN 220 0
EN 240 0
EN 260 0
EN 280 0
EN 300 0
EN 320 0
EN 340 0
EN 360 0
A-RIB:A-P:ILE-S1
EN 20 0
EN 40 -5036
EN 60 -5185
EN 80 -2430
EN 100 -1873
EN 120 -3288
EN 140 -1934
EN 160 -3902
EN 180 -3577
EN 200 0
EN 220 -4509
EN 240 -4801
EN 260 -4044
EN 280 -3469
EN 300 0
EN 320 -4243

EN 340 -3983
EN 360 -5619
C-P:C-RIB:TYR-S2
EN 20 0
EN 40 -4651
EN 60 -3037
EN 80 -3929
EN 100 -4055
EN 120 -2687
EN 140 -4361
EN 160 -1129
EN 180 0
EN 200 0
EN 220 -6612
EN 240 -4136
EN 260 -5082
EN 280 -4765
EN 300 -2680
EN 320 -4276
EN 340 -4449
EN 360 -4228
G-RIB:G-P:GLU-CA
EN 20 0
EN 40 -2079
EN 60 1362
EN 80 1605
EN 100 1540
EN 120 -2116
EN 140 -1408
EN 160 -368
EN 180 650
EN 200 0
EN 220 -4678
EN 240 0
EN 260 -1687
EN 280 235
EN 300 407
EN 320 -439
EN 340 -1682
EN 360 0
C-RIB:C-Y:LYS-S2
EN 20 -4927
EN 40 -3738
EN 60 -3124
EN 80 -4485
EN 100 -3218
EN 120 -2425
EN 140 -2622
EN 160 -3287
EN 180 -2812
EN 200 -4469
EN 220 -3009
EN 240 -3163
EN 260 -2888
EN 280 -3311
EN 300 -3089
EN 320 -3650
EN 340 -4110

EN 360 -2690
U-P:U-RIB:ARG-S2
EN 20 -8168
EN 40 -5165
EN 60 -4760
EN 80 -5352
EN 100 -3339
EN 120 -3765
EN 140 -3841
EN 160 -3208
EN 180 -4497
EN 200 -5435
EN 220 -6064
EN 240 -5323
EN 260 -3946
EN 280 -4045
EN 300 -2622
EN 320 -3249
EN 340 -2500
EN 360 -4446
G-RIB:G-R6:LEU-S1
EN 20 -8793
EN 40 -5806
EN 60 -4673
EN 80 -4066
EN 100 0
EN 120 -4295
EN 140 -3425
EN 160 -3542
EN 180 0
EN 200 0
EN 220 -5894
EN 240 -1561
EN 260 -5172
EN 280 -4708
EN 300 -2972
EN 320 -747
EN 340 0
EN 360 0
DA-RIB:DA-M6:SER-CA
EN 20 0
EN 40 0
EN 60 0
EN 80 0
EN 100 0
EN 120 0
EN 140 0
EN 160 0
EN 180 0
EN 200 0
EN 220 0
EN 240 0
EN 260 0
EN 280 0
EN 300 -6970
EN 320 -6553
EN 340 0
EN 360 0

C-P:C-RIB:ARG-S1
EN 20 0
EN 40 -6919
EN 60 -5644
EN 80 -4968
EN 100 -4551
EN 120 -3656
EN 140 -3932
EN 160 -3583
EN 180 -5214
EN 200 -8253
EN 220 -6834
EN 240 -5372
EN 260 -4319
EN 280 -4255
EN 300 -4605
EN 320 -4273
EN 340 -3769
EN 360 -3527
G-RIB:G-R6:TYR-S1
EN 20 0
EN 40 0
EN 60 -5021
EN 80 -2093
EN 100 -5286
EN 120 -3830
EN 140 0
EN 160 -4779
EN 180 0
EN 200 0
EN 220 -7921
EN 240 0
EN 260 -4067
EN 280 -4953
EN 300 -3706
EN 320 -5312
EN 340 -3551
EN 360 -7517
G-RIB:G-R6:GLY-CA
EN 20 -6064
EN 40 -4652
EN 60 -4482
EN 80 -3699
EN 100 -2823
EN 120 -2888
EN 140 -2774
EN 160 -2262
EN 180 -4068
EN 200 -7251
EN 220 -4924
EN 240 -2665
EN 260 -3914
EN 280 -2920
EN 300 -3001
EN 320 -3483
EN 340 -2381
EN 360 -4119
C-RIB:C-P:GLN-CA

EN 20 0
EN 40 -5380
EN 60 -5675
EN 80 -5007
EN 100 -3345
EN 120 -3564
EN 140 -1877
EN 160 -3512
EN 180 -5198
EN 200 0
EN 220 -6810
EN 240 -4561
EN 260 -4295
EN 280 -5079
EN 300 -4804
EN 320 -4836
EN 340 -4078
EN 360 -4318
G-P:G-RIB:ILE-S1
EN 20 0
EN 40 -2644
EN 60 0
EN 80 -3922
EN 100 -2564
EN 120 -1860
EN 140 -2217
EN 160 0
EN 180 0
EN 200 0
EN 220 -3975
EN 240 0
EN 260 -2923
EN 280 -4298
EN 300 627
EN 320 -3845
EN 340 -2713
EN 360 -3154
C-P:C-RIB:MET-CA
EN 20 0
EN 40 -4700
EN 60 0
EN 80 -4067
EN 100 -5428
EN 120 -933
EN 140 -2731
EN 160 0
EN 180 -5780
EN 200 0
EN 220 0
EN 240 -5011
EN 260 -1366
EN 280 -3495
EN 300 -5036
EN 320 -3835
EN 340 0
EN 360 -6542
G-RIB:G-R6:TYR-S2
EN 20 0

EN 40 0
EN 60 -5344
EN 80 -5014
EN 100 -4236
EN 120 -5721
EN 140 -3263
EN 160 -2685
EN 180 0
EN 200 0
EN 220 -4947
EN 240 -5143
EN 260 -2965
EN 280 -4488
EN 300 -5002
EN 320 -5739
EN 340 -4544
EN 360 -4875
C-P:C-RIB:TRP-S2
EN 20 0
EN 40 -5755
EN 60 0
EN 80 -6190
EN 100 -4662
EN 120 -1823
EN 140 0
EN 160 -4494
EN 180 0
EN 200 0
EN 220 0
EN 240 -6166
EN 260 -2333
EN 280 -4830
EN 300 -1940
EN 320 -3956
EN 340 -3075
EN 360 -7119
C-RIB:C-Y:TYR-S2
EN 20 0
EN 40 -5631
EN 60 -3483
EN 80 -5088
EN 100 -5920
EN 120 -3666
EN 140 -2515
EN 160 -3464
EN 180 0
EN 200 0
EN 220 -5051
EN 240 -4224
EN 260 -3771
EN 280 -5404
EN 300 -4646
EN 320 -2632
EN 340 -4898
EN 360 0
U-P:U-RIB:TYR-S2
EN 20 0
EN 40 -5093

EN 60 0
EN 80 -4490
EN 100 -5017
EN 120 -3140
EN 140 -1554
EN 160 -4801
EN 180 -5966
EN 200 0
EN 220 -4907
EN 240 -1962
EN 260 -5737
EN 280 -3481
EN 300 -955
EN 320 -4759
EN 340 -4773
EN 360 -4405
C-RIB:C-Y:MET-S1
EN 20 0
EN 40 -6227
EN 60 -4288
EN 80 -5055
EN 100 -5382
EN 120 0
EN 140 0
EN 160 0
EN 180 0
EN 200 -6553
EN 220 -5584
EN 240 -4530
EN 260 -2707
EN 280 -5281
EN 300 -2864
EN 320 0
EN 340 0
EN 360 0
U-RIB:U-Y:ASP-CA
EN 20 -5446
EN 40 -1719
EN 60 -1896
EN 80 -1399
EN 100 0
EN 120 -1169
EN 140 70
EN 160 -1029
EN 180 0
EN 200 0
EN 220 -3366
EN 240 -217
EN 260 -3120
EN 280 0
EN 300 516
EN 320 12
EN 340 -3666
EN 360 -3075
G-RIB:G-P:ASP-CA
EN 20 0
EN 40 0
EN 60 0

EN 80 -2073
EN 100 -1790
EN 120 -1394
EN 140 -2765
EN 160 -2675
EN 180 -19
EN 200 0
EN 220 -5344
EN 240 -3405
EN 260 -813
EN 280 -2182
EN 300 -3440
EN 320 -2289
EN 340 -2013
EN 360 -3985
G-RIB:G-R5:LYS-S1
EN 20 -4470
EN 40 -4765
EN 60 -3336
EN 80 -2277
EN 100 -3242
EN 120 -3248
EN 140 -2016
EN 160 -2799
EN 180 -5393
EN 200 -6169
EN 220 -719
EN 240 -2508
EN 260 -2341
EN 280 -3725
EN 300 -5148
EN 320 -4713
EN 340 -5630
EN 360 0
A-RIB:A-P:PHE-CA
EN 20 0
EN 40 0
EN 60 -4998
EN 80 -3439
EN 100 -2377
EN 120 -365
EN 140 -1074
EN 160 -5429
EN 180 -4464
EN 200 0
EN 220 -6697
EN 240 -5295
EN 260 -3198
EN 280 -3623
EN 300 -5662
EN 320 -4304
EN 340 -4540
EN 360 0
G-P:G-RIB:TRP-CA
EN 20 0
EN 40 -8135
EN 60 0
EN 80 -4221

EN 100 -3944
EN 120 0
EN 140 -4354
EN 160 -5840
EN 180 0
EN 200 0
EN 220 0
EN 240 -6672
EN 260 -4479
EN 280 -6184
EN 300 -4790
EN 320 -4405
EN 340 0
EN 360 0
G-P:G-RIB:TYR-CA
EN 20 0
EN 40 0
EN 60 -2056
EN 80 -3488
EN 100 -593
EN 120 0
EN 140 -1037
EN 160 -4479
EN 180 0
EN 200 0
EN 220 -6929
EN 240 0
EN 260 -4892
EN 280 -3860
EN 300 -3019
EN 320 0
EN 340 -4257
EN 360 0
U-P:U-RIB:TRP-S1
EN 20 0
EN 40 -8271
EN 60 -4540
EN 80 -6722
EN 100 0
EN 120 0
EN 140 -5337
EN 160 -4375
EN 180 0
EN 200 0
EN 220 0
EN 240 -8006
EN 260 -5435
EN 280 -3464
EN 300 0
EN 320 0
EN 340 -4525
EN 360 0
G-RIB:G-R6:ARG-CA
EN 20 0
EN 40 -4412
EN 60 -3186
EN 80 -3066
EN 100 -3432

EN 120 -2626
EN 140 -3649
EN 160 -3495
EN 180 -2717
EN 200 0
EN 220 -6545
EN 240 -4077
EN 260 -4115
EN 280 -2467
EN 300 -4358
EN 320 -3230
EN 340 -4060
EN 360 0
G-RIB:G-R5:ASP-S1
EN 20 0
EN 40 -1971
EN 60 -1775
EN 80 -1144
EN 100 -2038
EN 120 -1227
EN 140 -2345
EN 160 -3322
EN 180 0
EN 200 -3737
EN 220 -3590
EN 240 -2285
EN 260 -2751
EN 280 -2985
EN 300 -275
EN 320 -3436
EN 340 -1404
EN 360 -2285
U-RIB:U-Y:LEU-S2
EN 20 0
EN 40 -5106
EN 60 -5564
EN 80 -4437
EN 100 -4628
EN 120 -2823
EN 140 -4136
EN 160 -2476
EN 180 -4706
EN 200 0
EN 220 -3213
EN 240 -6580
EN 260 -4215
EN 280 -2909
EN 300 -1144
EN 320 -3278
EN 340 -2356
EN 360 -5119
A-P:A-RIB:HIS-S2
EN 20 0
EN 40 0
EN 60 -5333
EN 80 -3924
EN 100 -4801
EN 120 -3385

EN 140 -4566
EN 160 -3324
EN 180 -5316
EN 200 0
EN 220 -4603
EN 240 -5446
EN 260 -5772
EN 280 -5598
EN 300 -4866
EN 320 -5166
EN 340 -4121
EN 360 -2352
A-P:A-RIB:GLN-S2
EN 20 -5337
EN 40 -3271
EN 60 -4154
EN 80 -4372
EN 100 -3521
EN 120 -3344
EN 140 -1705
EN 160 -2286
EN 180 -1792
EN 200 0
EN 220 -4587
EN 240 -3058
EN 260 -4198
EN 280 -4119
EN 300 -4679
EN 320 -1881
EN 340 -3922
EN 360 -3136
A-RIB:A-R5:ASN-S1
EN 20 -8482
EN 40 -1507
EN 60 -3444
EN 80 -4057
EN 100 -4658
EN 120 -2155
EN 140 -5788
EN 160 -4583
EN 180 0
EN 200 0
EN 220 -4367
EN 240 -5250
EN 260 -2986
EN 280 -2511
EN 300 -2960
EN 320 -2502
EN 340 -2059
EN 360 -5601
FHU-RIB:FHU-P:ARG-S2
EN 20 0
EN 40 0
EN 60 0
EN 80 -7629
EN 100 -7854
EN 120 0
EN 140 0

EN 160 0
EN 180 0
EN 200 0
EN 220 0
EN 240 0
EN 260 0
EN 280 -7141
EN 300 0
EN 320 0
EN 340 0
EN 360 0
FMU-P:FMU-RIB:ASN-S1
EN 20 0
EN 40 0
EN 60 0
EN 80 0
EN 100 0
EN 120 0
EN 140 -14393
EN 160 0
EN 180 0
EN 200 0
EN 220 0
EN 240 0
EN 260 0
EN 280 0
EN 300 0
EN 320 0
EN 340 0
EN 360 0
U-RIB:U-P:MET-S2
EN 20 0
EN 40 0
EN 60 -4898
EN 80 -5187
EN 100 -1620
EN 120 0
EN 140 -4194
EN 160 -4147
EN 180 0
EN 200 0
EN 220 -7301
EN 240 -3535
EN 260 -5680
EN 280 -4332
EN 300 -1463
EN 320 -3271
EN 340 -4154
EN 360 0
H2U-RIB:H2U-MY:ARG-S2
EN 20 0
EN 40 0
EN 60 0
EN 80 0
EN 100 -7278
EN 120 0
EN 140 0
EN 160 0

EN 180	0
EN 200	0
EN 220	0
EN 240	0
EN 260	-9241
EN 280	0
EN 300	-6970
EN 320	0
EN 340	-9241
EN 360	0
G-RIB:G-R5:ILE-S1
EN 20 0
EN 40 -3602
EN 60 -989
EN 80 -244
EN 100	-2969
EN 120	-2895
EN 140	-1721
EN 160	0
EN 180	0
EN 200	-4771
EN 220	-2212
EN 240	-1241
EN 260	-2517
EN 280	-1062
EN 300	-2959
EN 320	0
EN 340	0
EN 360	0
G-RIB:G-R6:ILE-CA
EN 20 0
EN 40 0
EN 60 -1801
EN 80 0
EN 100	-3151
EN 120	-654
EN 140	0
EN 160	-2108
EN 180	-4729
EN 200	0
EN 220	0
EN 240	-4836
EN 260	-3140
EN 280	-2025
EN 300	-3873
EN 320	-1358
EN 340	0
EN 360	0
A-RIB:A-R6:GLU-S2
EN 20 0
EN 40 -3683
EN 60 -1886
EN 80 -1205
EN 100	-135
EN 120	-1711
EN 140	-663
EN 160	125
EN 180	-3571

EN 200 0
EN 220 76
EN 240 -147
EN 260 -691
EN 280 -2478
EN 300 -838
EN 320 952
EN 340 1796
EN 360 -3528
A-RIB:A-R5:MET-S1
EN 20 0
EN 40 0
EN 60 -4880
EN 80 -4775
EN 100 -5433
EN 120 -4661
EN 140 -5232
EN 160 0
EN 180 0
EN 200 0
EN 220 -4556
EN 240 -3313
EN 260 -4767
EN 280 0
EN 300 -6608
EN 320 -3507
EN 340 -7309
EN 360 -6889
G-P:G-RIB:TYR-S2
EN 20 0
EN 40 -3866
EN 60 -3938
EN 80 -4146
EN 100 -4295
EN 120 -2843
EN 140 -3834
EN 160 -3995
EN 180 -4759
EN 200 0
EN 220 -3567
EN 240 -6577
EN 260 -4494
EN 280 -3330
EN 300 7
EN 320 -4224
EN 340 -1086
EN 360 0
FHU-RIB:FHU-MY:ARG-CA
EN 20 0
EN 40 0
EN 60 0
EN 80 0
EN 100 0
EN 120 -9953
EN 140 -12891
EN 160 0
EN 180 0
EN 200 0

EN 220 0
EN 240 0
EN 260 0
EN 280 0
EN 300 -9819
EN 320 -12675
EN 340 0
EN 360 0
C-RIB:C-P:LYS-CA
EN 20 0
EN 40 -4056
EN 60 -3551
EN 80 -4339
EN 100 -3599
EN 120 -3710
EN 140 -4484
EN 160 -4422
EN 180 -3671
EN 200 0
EN 220 -5158
EN 240 -2754
EN 260 -3841
EN 280 -3174
EN 300 -4169
EN 320 -4799
EN 340 -4662
EN 360 -4032
FMU-P:FMU-RIB:GLN-CA
EN 20 0
EN 40 0
EN 60 0
EN 80 0
EN 100 0
EN 120 0
EN 140 0
EN 160 0
EN 180 0
EN 200 0
EN 220 0
EN 240 0
EN 260 0
EN 280 -2722
EN 300 0
EN 320 0
EN 340 0
EN 360 0
U-RIB:U-P:THR-S1
EN 20 0
EN 40 -5145
EN 60 -2985
EN 80 -1618
EN 100 -1761
EN 120 -1125
EN 140 -3644
EN 160 -3511
EN 180 -3630
EN 200 0
EN 220 -5688

EN 240 -4047
EN 260 -3251
EN 280 -2778
EN 300 -1055
EN 320 -3730
EN 340 -4573
EN 360 -5217
C-P:C-RIB:THR-S1
EN 20 -4801
EN 40 -4878
EN 60 -2901
EN 80 -3554
EN 100 -3038
EN 120 -3844
EN 140 -3277
EN 160 -1514
EN 180 -3574
EN 200 -4801
EN 220 -3732
EN 240 -5112
EN 260 -3155
EN 280 -3020
EN 300 -3979
EN 320 -4616
EN 340 -4157
EN 360 -3385
G-RIB:G-R5:LEU-CA
EN 20 0
EN 40 -2777
EN 60 -4503
EN 80 -2880
EN 100 -4150
EN 120 -4712
EN 140 -5591
EN 160 -2606
EN 180 0
EN 200 0
EN 220 -5378
EN 240 -5439
EN 260 -6243
EN 280 -2661
EN 300 -4263
EN 320 -3024
EN 340 0
EN 360 -4763
G-RIB:G-P:GLN-S2
EN 20 0
EN 40 -3212
EN 60 -4786
EN 80 -1834
EN 100 -2189
EN 120 -2854
EN 140 -2929
EN 160 -4482
EN 180 -4180
EN 200 0
EN 220 -5192
EN 240 -3244

EN 260 -2264
EN 280 -3902
EN 300 -2655
EN 320 -2223
EN 340 -3990
EN 360 -4889
G-P:G-RIB:TYR-S1
EN 20 0
EN 40 0
EN 60 -3914
EN 80 -2576
EN 100 -2333
EN 120 -3302
EN 140 -1063
EN 160 -4592
EN 180 0
EN 200 0
EN 220 -5132
EN 240 -2326
EN 260 -4972
EN 280 -5017
EN 300 -2059
EN 320 0
EN 340 -4975
EN 360 0
A-P:A-RIB:PRO-CA
EN 20 0
EN 40 -2983
EN 60 -3731
EN 80 -3231
EN 100 -4034
EN 120 -4806
EN 140 -1927
EN 160 -3565
EN 180 -5031
EN 200 0
EN 220 0
EN 240 -3774
EN 260 -4561
EN 280 -2143
EN 300 -3212
EN 320 -4770
EN 340 -2257
EN 360 0
G-RIB:G-R6:VAL-CA
EN 20 0
EN 40 0
EN 60 -703
EN 80 426
EN 100 -3653
EN 120 -2144
EN 140 -36
EN 160 0
EN 180 -3056
EN 200 0
EN 220 -4079
EN 240 -3206
EN 260 -3029

EN 280 -2844
EN 300 -1995
EN 320 -2777
EN 340 -4911
EN 360 0
IU-RIB:IU-MY:ILE-CA
EN 20 0
EN 40 0
EN 60 -14393
EN 80 0
EN 100 0
EN 120 0
EN 140 0
EN 160 0
EN 180 0
EN 200 0
EN 220 0
EN 240 -11963
EN 260 0
EN 280 0
EN 300 0
EN 320 0
EN 340 0
EN 360 0
A-RIB:A-R5:GLN-CA
EN 20 -7739
EN 40 0
EN 60 -6061
EN 80 -1207
EN 100 -5278
EN 120 -2917
EN 140 -3952
EN 160 -6476
EN 180 0
EN 200 -9042
EN 220 -3388
EN 240 -1893
EN 260 -5218
EN 280 -2919
EN 300 -4786
EN 320 -2078
EN 340 0
EN 360 0
G-RIB:G-R6:MET-S2
EN 20 0
EN 40 -5590
EN 60 -4381
EN 80 -4048
EN 100 -3255
EN 120 -4380
EN 140 0
EN 160 0
EN 180 -7119
EN 200 0
EN 220 0
EN 240 -5993
EN 260 -5542
EN 280 -4131

EN 300 -1648
EN 320 -5246
EN 340 -4194
EN 360 0
A-RIB:A-R6:THR-CA
EN 20 0
EN 40 -4961
EN 60 -4648
EN 80 -1484
EN 100 -4159
EN 120 -3403
EN 140 -3227
EN 160 -2667
EN 180 -5292
EN 200 0
EN 220 -6642
EN 240 -5521
EN 260 -4276
EN 280 -2037
EN 300 -2224
EN 320 -2650
EN 340 -2535
EN 360 0
C-RIB:C-P:HIS-CA
EN 20 0
EN 40 -8580
EN 60 -4063
EN 80 -786
EN 100 -5588
EN 120 -3643
EN 140 -4361
EN 160 -4961
EN 180 -3609
EN 200 0
EN 220 0
EN 240 -2282
EN 260 -5708
EN 280 -2356
EN 300 -3031
EN 320 -5248
EN 340 -1018
EN 360 -3207
U-P:U-RIB:ARG-CA
EN 20 -7914
EN 40 -4318
EN 60 -3890
EN 80 -2762
EN 100 -3879
EN 120 -4809
EN 140 -4419
EN 160 -3698
EN 180 -2245
EN 200 -7575
EN 220 -5416
EN 240 -5938
EN 260 -2331
EN 280 -2435
EN 300 -4401

EN 320 -1559
EN 340 -3838
EN 360 -2215
G-RIB:G-R6:PHE-S2
EN 20 -12675
EN 40 0
EN 60 -4544
EN 80 -5413
EN 100 -5272
EN 120 -1642
EN 140 -2304
EN 160 -3351
EN 180 0
EN 200 0
EN 220 -5980
EN 240 -6221
EN 260 -3871
EN 280 -4299
EN 300 -2029
EN 320 0
EN 340 -2476
EN 360 0
A-RIB:A-R6:VAL-CA
EN 20 0
EN 40 -5537
EN 60 -1422
EN 80 -5583
EN 100 -3721
EN 120 -3211
EN 140 -3575
EN 160 -972
EN 180 -3899
EN 200 0
EN 220 0
EN 240 -1431
EN 260 -4166
EN 280 -3625
EN 300 -3606
EN 320 -3961
EN 340 -2490
EN 360 0
A-RIB:A-R5:TRP-S1
EN 20 -7739
EN 40 -5172
EN 60 0
EN 80 -3938
EN 100 -3779
EN 120 -4283
EN 140 -4619
EN 160 0
EN 180 0
EN 200 0
EN 220 0
EN 240 -7015
EN 260 -5884
EN 280 -7112
EN 300 -4408
EN 320 -4556

EN 340 0
EN 360 0
C31-RIB:C31-P:SER-S1
EN 20 0
EN 40 0
EN 60 0
EN 80 0
EN 100 0
EN 120 0
EN 140 0
EN 160 0
EN 180 0
EN 200 0
EN 220 0
EN 240 0
EN 260 0
EN 280 0
EN 300 0
EN 320 -12675
EN 340 0
EN 360 0
U-RIB:U-P:ASP-S2
EN 20 0
EN 40 0
EN 60 1187
EN 80 -395
EN 100 -1101
EN 120 -3122
EN 140 -976
EN 160 -989
EN 180 -2345
EN 200 0
EN 220 -4552
EN 240 -535
EN 260 -1656
EN 280 -1107
EN 300 -718
EN 320 -1589
EN 340 -3460
EN 360 -3382
U-RIB:U-Y:TRP-S2
EN 20 -10958
EN 40 -6258
EN 60 -7054
EN 80 -4454
EN 100 -4194
EN 120 0
EN 140 0
EN 160 0
EN 180 0
EN 200 -10576
EN 220 0
EN 240 0
EN 260 -6078
EN 280 0
EN 300 -4540
EN 320 -4556
EN 340 0

EN 360 0
G-P:G-RIB:PRO-CA
EN 20 0
EN 40 -1803
EN 60 -4490
EN 80 -5072
EN 100 -4351
EN 120 -2884
EN 140 -3273
EN 160 -4894
EN 180 -3732
EN 200 -6970
EN 220 -4351
EN 240 -2478
EN 260 -2530
EN 280 -3012
EN 300 -3777
EN 320 -2859
EN 340 -2503
EN 360 0
U31-RIB:U31-P:GLN-S1
EN 20 0
EN 40 0
EN 60 0
EN 80 0
EN 100 0
EN 120 0
EN 140 0
EN 160 0
EN 180 0
EN 200 0
EN 220 0
EN 240 -11963
EN 260 0
EN 280 0
EN 300 0
EN 320 0
EN 340 0
EN 360 0
C-RIB:C-Y:LYS-CA
EN 20 -3950
EN 40 -4207
EN 60 -4493
EN 80 -4670
EN 100 -5254
EN 120 -3519
EN 140 -2984
EN 160 -4564
EN 180 -4361
EN 200 -3991
EN 220 -3254
EN 240 -4051
EN 260 -3956
EN 280 1084
EN 300 -2821
EN 320 -1209
EN 340 -1834
EN 360 -4078

A-P:A-RIB:VAL-CA
EN 20 0
EN 40 -6237
EN 60 -4088
EN 80 -3466
EN 100 -2599
EN 120 -3793
EN 140 -3257
EN 160 -5010
EN 180 -2079
EN 200 0
EN 220 0
EN 240 -817
EN 260 -3808
EN 280 -4340
EN 300 -3154
EN 320 -3549
EN 340 -3167
EN 360 -4366
U31-P:U31-RIB:PHE-CA
EN 20 0
EN 40 0
EN 60 0
EN 80 0
EN 100 0
EN 120 -13127
EN 140 0
EN 160 0
EN 180 0
EN 200 0
EN 220 0
EN 240 0
EN 260 0
EN 280 0
EN 300 0
EN 320 0
EN 340 0
EN 360 0
A-RIB:A-R5:ALA-CA
EN 20 -4028
EN 40 -3768
EN 60 -2317
EN 80 -1797
EN 100 -1921
EN 120 -3510
EN 140 -2805
EN 160 -4289
EN 180 0
EN 200 -5671
EN 220 -3630
EN 240 -1543
EN 260 -3384
EN 280 -1995
EN 300 -3563
EN 320 -1897
EN 340 -951
EN 360 -3402
C31-RIB:C31-MY:THR-CA

EN 20 0
EN 40 0
EN 60 0
EN 80 0
EN 100 0
EN 120 0
EN 140 0
EN 160 0
EN 180 0
EN 200 0
EN 220 0
EN 240 0
EN 260 -8772
EN 280 0
EN 300 0
EN 320 0
EN 340 0
EN 360 0
FMU-RIB:FMU-MY:ASP-S1
EN 20 0
EN 40 0
EN 60 0
EN 80 0
EN 100 0
EN 120 0
EN 140 0
EN 160 -12675
EN 180 0
EN 200 0
EN 220 0
EN 240 0
EN 260 0
EN 280 0
EN 300 0
EN 320 0
EN 340 0
EN 360 0
A-P:A-RIB:TYR-CA
EN 20 0
EN 40 0
EN 60 -7163
EN 80 -5619
EN 100 -5478
EN 120 -2983
EN 140 -3299
EN 160 -5087
EN 180 -4405
EN 200 0
EN 220 -6734
EN 240 -3556
EN 260 -4711
EN 280 -4041
EN 300 -5253
EN 320 -4337
EN 340 -6073
EN 360 0
C-P:C-RIB:PHE-CA
EN 20 0

EN 40 0
EN 60 -3515
EN 80 -4072
EN 100 -3318
EN 120 -3295
EN 140 -2173
EN 160 -4132
EN 180 0
EN 200 0
EN 220 0
EN 240 0
EN 260 -3645
EN 280 -3019
EN 300 -193
EN 320 -3309
EN 340 0
EN 360 -4141
OMC-RIB:OMC-P:LYS-S2
EN 20 0
EN 40 0
EN 60 0
EN 80 -7914
EN 100 -6772
EN 120 0
EN 140 0
EN 160 0
EN 180 0
EN 200 0
EN 220 0
EN 240 0
EN 260 0
EN 280 0
EN 300 0
EN 320 0
EN 340 0
EN 360 0
A-RIB:A-R6:LYS-CA
EN 20 0
EN 40 -6326
EN 60 -3667
EN 80 -2274
EN 100 -3128
EN 120 -2779
EN 140 -3143
EN 160 -2157
EN 180 0
EN 200 0
EN 220 -3309
EN 240 -3878
EN 260 -3865
EN 280 -4181
EN 300 -2314
EN 320 -2654
EN 340 600
EN 360 0
C-RIB:C-Y:ASN-CA
EN 20 0
EN 40 -2108

EN 60 -5317
EN 80 -3661
EN 100 -3135
EN 120 -2270
EN 140 -3704
EN 160 0
EN 180 -4207
EN 200 -7406
EN 220 0
EN 240 -2806
EN 260 -3510
EN 280 -3398
EN 300 -2115
EN 320 -3693
EN 340 -1834
EN 360 -5845
FMU-P:FMU-RIB:GLN-S2
EN 20 0
EN 40 0
EN 60 0
EN 80 0
EN 100 0
EN 120 0
EN 140 0
EN 160 0
EN 180 0
EN 200 0
EN 220 0
EN 240 0
EN 260 0
EN 280 0
EN 300 -11410
EN 320 0
EN 340 0
EN 360 0
C-RIB:C-P:MET-S2
EN 20 0
EN 40 -6078
EN 60 -5760
EN 80 -4641
EN 100 -2099
EN 120 -2134
EN 140 -3908
EN 160 0
EN 180 -4194
EN 200 0
EN 220 -4375
EN 240 -5711
EN 260 -4279
EN 280 -4018
EN 300 -458
EN 320 -494
EN 340 -3008
EN 360 -5871
C-RIB:C-Y:VAL-S1
EN 20 -4033
EN 40 -3571
EN 60 -4818

EN 80 -2464
EN 100 -3493
EN 120 -3104
EN 140 -3429
EN 160 0
EN 180 0
EN 200 -5435
EN 220 -3713
EN 240 -4432
EN 260 -3462
EN 280 -4626
EN 300 -3130
EN 320 -3535
EN 340 0
EN 360 0
G-P:G-RIB:PHE-S1
EN 20 -9953
EN 40 -6624
EN 60 0
EN 80 -4372
EN 100 -3834
EN 120 -5463
EN 140 -2746
EN 160 -2114
EN 180 -4276
EN 200 0
EN 220 0
EN 240 -4795
EN 260 -3574
EN 280 -3969
EN 300 -2170
EN 320 -1109
EN 340 -6171
EN 360 -6196
G-P:G-RIB:HIS-S1
EN 20 -7629
EN 40 -5017
EN 60 -855
EN 80 -3687
EN 100 -2008
EN 120 -2175
EN 140 -5320
EN 160 -2298
EN 180 -5925
EN 200 0
EN 220 -3084
EN 240 -4184
EN 260 -4761
EN 280 -3892
EN 300 -3145
EN 320 -4755
EN 340 -5739
EN 360 -2390
G-RIB:G-R6:PRO-S1
EN 20 0
EN 40 -3252
EN 60 -4162
EN 80 -1998

EN 100 -1461
EN 120 -2695
EN 140 -1380
EN 160 -2257
EN 180 0
EN 200 0
EN 220 -5722
EN 240 -2683
EN 260 -4004
EN 280 -2666
EN 300 -627
EN 320 -3228
EN 340 -3580
EN 360 0
C-RIB:C-Y:GLU-S2
EN 20 -375
EN 40 -1979
EN 60 -1837
EN 80 -451
EN 100 -1255
EN 120 -326
EN 140 436
EN 160 -2051
EN 180 -2650
EN 200 -2970
EN 220 -495
EN 240 -670
EN 260 -904
EN 280 -1266
EN 300 2604
EN 320 -1825
EN 340 -346
EN 360 -3711
C-RIB:C-P:THR-CA
EN 20 0
EN 40 -5202
EN 60 -4728
EN 80 -4718
EN 100 -3497
EN 120 -4390
EN 140 -2692
EN 160 -3943
EN 180 -3154
EN 200 0
EN 220 -5631
EN 240 -3668
EN 260 -3902
EN 280 -3801
EN 300 -2912
EN 320 -3312
EN 340 -2978
EN 360 -4058
A-RIB:A-R5:VAL-S1
EN 20 -7145
EN 40 -5127
EN 60 -2364
EN 80 -4940
EN 100 -5786

EN 120 -2197
EN 140 -2852
EN 160 -3764
EN 180 0
EN 200 0
EN 220 -5172
EN 240 -363
EN 260 -2961
EN 280 -3890
EN 300 -5035
EN 320 -4046
EN 340 -2262
EN 360 0
U-RIB:U-P:TRP-S2
EN 20 0
EN 40 0
EN 60 0
EN 80 -6642
EN 100 -2629
EN 120 -4311
EN 140 -3162
EN 160 -3950
EN 180 0
EN 200 0
EN 220 -10095
EN 240 -4635
EN 260 -3474
EN 280 -5178
EN 300 0
EN 320 -5721
EN 340 -6342
EN 360 0
U-RIB:U-P:TYR-CA
EN 20 0
EN 40 -7472
EN 60 -6685
EN 80 -4961
EN 100 -1384
EN 120 0
EN 140 -2901
EN 160 -2257
EN 180 0
EN 200 0
EN 220 0
EN 240 0
EN 260 -4044
EN 280 -4071
EN 300 -3770
EN 320 -1591
EN 340 0
EN 360 0
G-P:G-RIB:GLY-CA
EN 20 -3484
EN 40 -3406
EN 60 -4222
EN 80 -2474
EN 100 -4310
EN 120 -3713

EN 140 -3791
EN 160 -4058
EN 180 -2691
EN 200 -5867
EN 220 -3876
EN 240 -2995
EN 260 -3417
EN 280 -3848
EN 300 -3124
EN 320 -3672
EN 340 -3604
EN 360 -4523
C-RIB:C-P:TRP-S2
EN 20 0
EN 40 0
EN 60 -5596
EN 80 -4088
EN 100 -4920
EN 120 -4535
EN 140 -3814
EN 160 -4845
EN 180 0
EN 200 0
EN 220 0
EN 240 -5549
EN 260 -6245
EN 280 0
EN 300 -4884
EN 320 0
EN 340 -2739
EN 360 0
U-RIB:U-Y:ILE-S1
EN 20 -6484
EN 40 0
EN 60 -2159
EN 80 -4426
EN 100 -4843
EN 120 -5000
EN 140 -1960
EN 160 0
EN 180 0
EN 200 0
EN 220 -3800
EN 240 -3596
EN 260 -1714
EN 280 -1499
EN 300 -1435
EN 320 -4513
EN 340 -2660
EN 360 0
A-P:A-RIB:TYR-S1
EN 20 0
EN 40 -6588
EN 60 -5806
EN 80 -5730
EN 100 -5911
EN 120 -3033
EN 140 -4667

EN 160	-2436
EN 180	-4464
EN 200	0
EN 220	0
EN 240	0
EN 260	-4992
EN 280	-5424
EN 300	-4198
EN 320	-4979
EN 340	-6044
EN 360	-4375
C-P:C-RIB:TRP-CA
EN 20 0
EN 40 0
EN 60 -6021
EN 80 -2746
EN 100	-4949
EN 120	-6196
EN 140	0
EN 160	0
EN 180	-5295
EN 200	0
EN 220	0
EN 240	0
EN 260	0
EN 280	-5219
EN 300	0
EN 320	0
EN 340	-4961
EN 360	-5780
G-RIB:G-R5:MET-S1
EN 20 -5717
EN 40 0
EN 60 -4964
EN 80 -6314
EN 100	0
EN 120	-2545
EN 140	0
EN 160	0
EN 180	0
EN 200	0
EN 220	-5045
EN 240	0
EN 260	-6177
EN 280	-2279
EN 300	-4575
EN 320	-6358
EN 340	-4211
EN 360	0
U34-RIB:U34-MY:SER-CA
EN 20 0
EN 40 0
EN 60 0
EN 80 0
EN 100	0
EN 120	0
EN 140	-10095
EN 160	0

EN 180	0
EN 200	0
EN 220	0
EN 240	0
EN 260	0
EN 280	0
EN 300	-8859
EN 320	0
EN 340	-14393
EN 360	0
G-RIB:G-R5:GLN-CA
EN 20 -5723
EN 40 -6913
EN 60 -6330
EN 80 -2531
EN 100	0
EN 120	0
EN 140	-1433
EN 160	0
EN 180	0
EN 200	-7690
EN 220	-6496
EN 240	-3947
EN 260	-5690
EN 280	-2641
EN 300	-2760
EN 320	-3206
EN 340	0
EN 360	0
FHU-RIB:FHU-MY:ILE-S1
EN 20 0
EN 40 0
EN 60 0
EN 80 0
EN 100	0
EN 120	0
EN 140	-14393
EN 160	0
EN 180	0
EN 200	0
EN 220	0
EN 240	0
EN 260	0
EN 280	0
EN 300	0
EN 320	-11410
EN 340	0
EN 360	0
A-RIB:A-R5:TRP-S2
EN 20 0
EN 40 0
EN 60 -7044
EN 80 0
EN 100	-6533
EN 120	-4339
EN 140	0
EN 160	-7774
EN 180	0

EN 200 0
EN 220 -5491
EN 240 -6035
EN 260 -3751
EN 280 -6630
EN 300 -4279
EN 320 -4509
EN 340 0
EN 360 0
A-RIB:A-R5:GLU-S2
EN 20 0
EN 40 -3225
EN 60 -2237
EN 80 -633
EN 100 -459
EN 120 712
EN 140 -2410
EN 160 -567
EN 180 -1307
EN 200 -1087
EN 220 -1133
EN 240 -1077
EN 260 -2074
EN 280 -1540
EN 300 -2808
EN 320 -826
EN 340 -489
EN 360 -1344
A-RIB:A-P:LEU-CA
EN 20 0
EN 40 -5806
EN 60 -3690
EN 80 -3622
EN 100 -1906
EN 120 -2780
EN 140 -931
EN 160 -3657
EN 180 0
EN 200 0
EN 220 -7209
EN 240 -3636
EN 260 -3244
EN 280 -2476
EN 300 -4377
EN 320 -769
EN 340 -4656
EN 360 -2870
C31-RIB:C31-P:GLU-CA
EN 20 0
EN 40 0
EN 60 0
EN 80 -7523
EN 100 0
EN 120 0
EN 140 0
EN 160 0
EN 180 0
EN 200 0

EN 220 0
EN 240 0
EN 260 0
EN 280 0
EN 300 0
EN 320 0
EN 340 0
EN 360 0
G-P:G-RIB:SER-CA
EN 20 -6417
EN 40 -5236
EN 60 -4836
EN 80 -2056
EN 100 -3442
EN 120 -1976
EN 140 -3811
EN 160 -2524
EN 180 -4259
EN 200 0
EN 220 -6200
EN 240 -2549
EN 260 -3074
EN 280 -3234
EN 300 -3774
EN 320 -2175
EN 340 -1729
EN 360 -3008
G-RIB:G-R6:PHE-S1
EN 20 0
EN 40 0
EN 60 -3575
EN 80 -4519
EN 100 -2008
EN 120 -5612
EN 140 -2360
EN 160 -3318
EN 180 0
EN 200 0
EN 220 -8210
EN 240 -5429
EN 260 -5288
EN 280 -2177
EN 300 -4847
EN 320 0
EN 340 0
EN 360 0
G-RIB:G-R6:SER-CA
EN 20 0
EN 40 -4341
EN 60 -4309
EN 80 -3502
EN 100 -2220
EN 120 -2143
EN 140 -1164
EN 160 -2218
EN 180 0
EN 200 0
EN 220 -3429

EN 240 -785
EN 260 -3300
EN 280 -3079
EN 300 -4397
EN 320 -2851
EN 340 -1998
EN 360 -2704
G-RIB:G-P:LEU-CA
EN 20 0
EN 40 0
EN 60 -4902
EN 80 -776
EN 100 -4402
EN 120 -3365
EN 140 -1862
EN 160 -2293
EN 180 -3434
EN 200 0
EN 220 0
EN 240 -6170
EN 260 -2379
EN 280 -3076
EN 300 -2675
EN 320 -2987
EN 340 -2304
EN 360 -1526
G-RIB:G-P:ILE-CA
EN 20 0
EN 40 0
EN 60 -4746
EN 80 -2330
EN 100 -1978
EN 120 -3091
EN 140 -2668
EN 160 -3641
EN 180 0
EN 200 0
EN 220 0
EN 240 -2966
EN 260 -5110
EN 280 1578
EN 300 -1049
EN 320 -2806
EN 340 -1485
EN 360 0
U-P:U-RIB:ARG-S1
EN 20 -7422
EN 40 -4579
EN 60 -5116
EN 80 -4697
EN 100 -3682
EN 120 -4211
EN 140 -3537
EN 160 -5162
EN 180 -5105
EN 200 0
EN 220 -5228
EN 240 -5008

EN 260 -4525
EN 280 -2909
EN 300 -4085
EN 320 -3862
EN 340 -3808
EN 360 -1236
FHU-RIB:FHU-MY:TYR-S1
EN 20 0
EN 40 0
EN 60 0
EN 80 0
EN 100 -11671
EN 120 0
EN 140 -15398
EN 160 0
EN 180 0
EN 200 0
EN 220 0
EN 240 0
EN 260 0
EN 280 -11963
EN 300 0
EN 320 -10958
EN 340 0
EN 360 0
G-P:G-RIB:LEU-S2
EN 20 0
EN 40 0
EN 60 -4492
EN 80 -4767
EN 100 -4736
EN 120 -297
EN 140 -3753
EN 160 -1682
EN 180 -4194
EN 200 -5514
EN 220 -3593
EN 240 -1556
EN 260 -3787
EN 280 -4126
EN 300 -3589
EN 320 -3968
EN 340 -2836
EN 360 -3837
FHU-RIB:FHU-MY:TYR-CA
EN 20 0
EN 40 0
EN 60 0
EN 80 0
EN 100 -11963
EN 120 0
EN 140 -11671
EN 160 0
EN 180 0
EN 200 0
EN 220 0
EN 240 0
EN 260 0

EN 280 -15398
EN 300 0
EN 320 -13127
EN 340 0
EN 360 0
C-P:C-RIB:PHE-S1
EN 20 0
EN 40 -5253
EN 60 -2173
EN 80 -3595
EN 100 -4199
EN 120 -3970
EN 140 -4088
EN 160 -3551
EN 180 -4571
EN 200 0
EN 220 0
EN 240 0
EN 260 -3855
EN 280 -3268
EN 300 -3256
EN 320 -3663
EN 340 -1983
EN 360 0
U-RIB:U-Y:PHE-S1
EN 20 0
EN 40 -8810
EN 60 -6161
EN 80 -3770
EN 100 0
EN 120 -4798
EN 140 0
EN 160 0
EN 180 0
EN 200 -9692
EN 220 -8734
EN 240 -4561
EN 260 -6377
EN 280 -7659
EN 300 -6816
EN 320 -3556
EN 340 0
EN 360 0
G-RIB:G-R5:PHE-S1
EN 20 -6669
EN 40 0
EN 60 -3311
EN 80 -2830
EN 100 -4530
EN 120 0
EN 140 -2999
EN 160 -4700
EN 180 0
EN 200 0
EN 220 -3908
EN 240 -7702
EN 260 -6320
EN 280 -2386

EN 300 -4470
EN 320 -3575
EN 340 0
EN 360 0
U-RIB:U-Y:LYS-CA
EN 20 -5631
EN 40 -2211
EN 60 -2852
EN 80 -2326
EN 100 -1993
EN 120 -3193
EN 140 248
EN 160 -734
EN 180 -4387
EN 200 -8038
EN 220 -4554
EN 240 -1931
EN 260 -4373
EN 280 -2517
EN 300 -2479
EN 320 -3558
EN 340 -4210
EN 360 0
U-RIB:U-P:LEU-CA
EN 20 0
EN 40 -6258
EN 60 -2173
EN 80 -3210
EN 100 -2627
EN 120 -3493
EN 140 -1934
EN 160 0
EN 180 -4141
EN 200 0
EN 220 -8038
EN 240 -2307
EN 260 -367
EN 280 -4853
EN 300 -4092
EN 320 -3303
EN 340 -336
EN 360 0
A-RIB:A-R5:ASP-S1
EN 20 0
EN 40 -4946
EN 60 1386
EN 80 -2596
EN 100 0
EN 120 -2208
EN 140 -1118
EN 160 -2921
EN 180 0
EN 200 -5125
EN 220 -1744
EN 240 -2562
EN 260 2027
EN 280 -2288
EN 300 1364

EN 320 -2982
EN 340 -2974
EN 360 -2987
G-RIB:G-P:ASP-S1
EN 20 0
EN 40 -999
EN 60 -2243
EN 80 -1176
EN 100 -1791
EN 120 -2543
EN 140 -1805
EN 160 -2011
EN 180 -1282
EN 200 0
EN 220 -3663
EN 240 -2755
EN 260 479
EN 280 -2064
EN 300 -3376
EN 320 -2284
EN 340 -3217
EN 360 -2901
G-P:G-RIB:TRP-S1
EN 20 0
EN 40 -5656
EN 60 -4952
EN 80 -2143
EN 100 -3561
EN 120 -3831
EN 140 -4134
EN 160 -2687
EN 180 0
EN 200 0
EN 220 0
EN 240 -6981
EN 260 -2538
EN 280 -4931
EN 300 -5847
EN 320 -4018
EN 340 -3198
EN 360 0
U-RIB:U-P:TYR-S2
EN 20 0
EN 40 0
EN 60 -5309
EN 80 -4375
EN 100 -3837
EN 120 -2230
EN 140 -2410
EN 160 -3347
EN 180 0
EN 200 0
EN 220 -6021
EN 240 -2784
EN 260 -5043
EN 280 -599
EN 300 -4612
EN 320 -915

EN 340 -3515
EN 360 0
U-RIB:U-P:ASN-S1
EN 20 -7796
EN 40 0
EN 60 -4134
EN 80 644
EN 100 -4428
EN 120 -3895
EN 140 -4554
EN 160 -3933
EN 180 -2358
EN 200 0
EN 220 -5457
EN 240 -3744
EN 260 -4002
EN 280 -3339
EN 300 -2320
EN 320 -2578
EN 340 -4474
EN 360 -2390
G-P:G-RIB:ASP-S2
EN 20 0
EN 40 937
EN 60 -2259
EN 80 -2548
EN 100 -2683
EN 120 -2433
EN 140 -325
EN 160 -2681
EN 180 -1846
EN 200 -2622
EN 220 0
EN 240 -1418
EN 260 -1513
EN 280 -2020
EN 300 -835
EN 320 -2919
EN 340 -2930
EN 360 -3448
C-RIB:C-Y:GLN-S1
EN 20 0
EN 40 -4584
EN 60 -5010
EN 80 -5090
EN 100 -734
EN 120 -4101
EN 140 -1122
EN 160 -1925
EN 180 0
EN 200 -6715
EN 220 -5239
EN 240 -6617
EN 260 -3668
EN 280 -3894
EN 300 0
EN 320 -4416
EN 340 -1945

EN 360 0
G-RIB:G-P:ARG-S1
EN 20 0
EN 40 -6124
EN 60 -3825
EN 80 -4435
EN 100 -5124
EN 120 -3916
EN 140 -4272
EN 160 -4365
EN 180 -5630
EN 200 0
EN 220 -4671
EN 240 -3736
EN 260 -4214
EN 280 -4600
EN 300 -4483
EN 320 -4060
EN 340 -4469
EN 360 -5366
FHU-P:FHU-RIB:ARG-S1
EN 20 0
EN 40 0
EN 60 0
EN 80 -9571
EN 100 0
EN 120 0
EN 140 0
EN 160 0
EN 180 0
EN 200 0
EN 220 0
EN 240 0
EN 260 -8949
EN 280 0
EN 300 0
EN 320 0
EN 340 -13680
EN 360 -2722
C-RIB:C-Y:THR-CA
EN 20 -5902
EN 40 -3777
EN 60 -2813
EN 80 -4222
EN 100 -3042
EN 120 -96
EN 140 -4025
EN 160 -1366
EN 180 -3627
EN 200 -8452
EN 220 -1865
EN 240 -5687
EN 260 -3285
EN 280 -1655
EN 300 -1691
EN 320 -2997
EN 340 -3990
EN 360 0

G-RIB:G-R6:ASP-CA
EN 20 0
EN 40 -2711
EN 60 -3320
EN 80 -1935
EN 100 -1422
EN 120 -154
EN 140 946
EN 160 -3341
EN 180 -2271
EN 200 0
EN 220 -5234
EN 240 -1095
EN 260 -1998
EN 280 -3611
EN 300 -1810
EN 320 -748
EN 340 -3271
EN 360 -3629
IU-RIB:IU-MY:LYS-CA
EN 20 0
EN 40 0
EN 60 -9571
EN 80 0
EN 100 -8647
EN 120 0
EN 140 -12759
EN 160 0
EN 180 0
EN 200 0
EN 220 0
EN 240 0
EN 260 -9042
EN 280 0
EN 300 0
EN 320 0
EN 340 0
EN 360 0
C31-RIB:C31-P:GLN-S2
EN 20 0
EN 40 0
EN 60 0
EN 80 0
EN 100 0
EN 120 0
EN 140 0
EN 160 -9571
EN 180 0
EN 200 0
EN 220 0
EN 240 0
EN 260 0
EN 280 0
EN 300 0
EN 320 0
EN 340 0
EN 360 0
U-RIB:U-P:TRP-S1

EN 20 0
EN 40 0
EN 60 -6553
EN 80 -3751
EN 100 -6849
EN 120 -2862
EN 140 -3092
EN 160 -4114
EN 180 -6849
EN 200 0
EN 220 0
EN 240 0
EN 260 -4025
EN 280 -6328
EN 300 -6865
EN 320 -5122
EN 340 -4434
EN 360 0
C-P:C-RIB:SER-CA
EN 20 -10364
EN 40 -6083
EN 60 -4964
EN 80 -3018
EN 100 -3979
EN 120 -3841
EN 140 -3207
EN 160 -1835
EN 180 -2825
EN 200 0
EN 220 -5261
EN 240 -3823
EN 260 -4108
EN 280 -1680
EN 300 -3091
EN 320 -4167
EN 340 -3478
EN 360 -5602
G-RIB:G-R6:GLN-S2
EN 20 0
EN 40 -4779
EN 60 -5252
EN 80 -4534
EN 100 -3451
EN 120 -4341
EN 140 -2354
EN 160 0
EN 180 -3194
EN 200 0
EN 220 -4137
EN 240 -2549
EN 260 -4235
EN 280 -4098
EN 300 -3393
EN 320 482
EN 340 -2491
EN 360 0
C-P:C-RIB:TYR-S1
EN 20 0

EN 40 0
EN 60 -2658
EN 80 -3313
EN 100 -2524
EN 120 -4670
EN 140 -2604
EN 160 -1828
EN 180 0
EN 200 0
EN 220 -5656
EN 240 0
EN 260 -5328
EN 280 -5399
EN 300 -6082
EN 320 -2517
EN 340 -5646
EN 360 -7325
G-P:G-RIB:PHE-CA
EN 20 0
EN 40 -6678
EN 60 -3768
EN 80 -4050
EN 100 -4781
EN 120 -4499
EN 140 -2336
EN 160 -3469
EN 180 0
EN 200 0
EN 220 0
EN 240 -4767
EN 260 -2500
EN 280 -4164
EN 300 -161
EN 320 -3434
EN 340 -4180
EN 360 -7171
G-P:G-RIB:VAL-S1
EN 20 0
EN 40 -4067
EN 60 -995
EN 80 -2801
EN 100 -2942
EN 120 -1652
EN 140 -1258
EN 160 -910
EN 180 -1436
EN 200 0
EN 220 -3012
EN 240 -2617
EN 260 74
EN 280 -1381
EN 300 -732
EN 320 -3851
EN 340 -1844
EN 360 -4244
A-RIB:A-R5:GLU-CA
EN 20 -6320
EN 40 -3645

EN 60 0
EN 80 -3055
EN 100 -236
EN 120 -3902
EN 140 -3282
EN 160 -496
EN 180 -3267
EN 200 0
EN 220 -4235
EN 240 703
EN 260 1203
EN 280 -2017
EN 300 -1590
EN 320 -2893
EN 340 0
EN 360 -2792
G-RIB:G-P:LYS-S2
EN 20 -2762
EN 40 1139
EN 60 -4227
EN 80 -4589
EN 100 -4602
EN 120 -4080
EN 140 -3415
EN 160 -4332
EN 180 -4195
EN 200 0
EN 220 -2417
EN 240 -4262
EN 260 -3466
EN 280 -4040
EN 300 -3778
EN 320 -3842
EN 340 -3945
EN 360 -3613
G-P:G-RIB:ASP-S1
EN 20 -5596
EN 40 167
EN 60 -2082
EN 80 -198
EN 100 -2129
EN 120 -2399
EN 140 -1172
EN 160 -2081
EN 180 -3368
EN 200 -4361
EN 220 213
EN 240 -369
EN 260 -1852
EN 280 -1811
EN 300 -1932
EN 320 -2491
EN 340 -2506
EN 360 -3882
C-P:C-RIB:ASP-S1
EN 20 0
EN 40 0
EN 60 -2362

EN 80 -874
EN 100 -3110
EN 120 -2484
EN 140 -2597
EN 160 -292
EN 180 -3376
EN 200 0
EN 220 -222
EN 240 -589
EN 260 -3446
EN 280 -2187
EN 300 565
EN 320 -3570
EN 340 -2701
EN 360 -3686
U-RIB:U-P:GLN-S1
EN 20 0
EN 40 0
EN 60 -4379
EN 80 -4119
EN 100 -2342
EN 120 -3355
EN 140 833
EN 160 -2823
EN 180 -2559
EN 200 0
EN 220 -6078
EN 240 -3804
EN 260 -1290
EN 280 -2412
EN 300 -4416
EN 320 -4567
EN 340 -3072
EN 360 -4434
A-RIB:A-R5:HIS-S1
EN 20 -6588
EN 40 -5142
EN 60 -3863
EN 80 -6497
EN 100 -4422
EN 120 -5832
EN 140 0
EN 160 -3474
EN 180 0
EN 200 0
EN 220 -5192
EN 240 -6484
EN 260 -4306
EN 280 -6159
EN 300 -3573
EN 320 -6576
EN 340 0
EN 360 0
C-RIB:C-Y:ARG-S1
EN 20 -5339
EN 40 -4053
EN 60 -4894
EN 80 -5509

EN 100 -6008
EN 120 -3731
EN 140 -5818
EN 160 -3563
EN 180 -7069
EN 200 -4248
EN 220 -5683
EN 240 -4636
EN 260 -5131
EN 280 -5159
EN 300 -4152
EN 320 -3949
EN 340 -3609
EN 360 -4474
G-RIB:G-R6:SER-S1
EN 20 -4438
EN 40 -4261
EN 60 -4539
EN 80 -2716
EN 100 -1860
EN 120 -2478
EN 140 -576
EN 160 46
EN 180 -2691
EN 200 -4386
EN 220 -3454
EN 240 -822
EN 260 -3968
EN 280 -3751
EN 300 -3850
EN 320 -3745
EN 340 39
EN 360 0
A-RIB:A-P:PHE-S2
EN 20 0
EN 40 -8378
EN 60 -5746
EN 80 -4938
EN 100 -3508
EN 120 -2735
EN 140 -3132
EN 160 -1934
EN 180 -4540
EN 200 0
EN 220 -7301
EN 240 -7593
EN 260 -4993
EN 280 -4286
EN 300 -915
EN 320 -5156
EN 340 -3598
EN 360 0
C-P:C-RIB:ILE-S1
EN 20 0
EN 40 -3216
EN 60 -3692
EN 80 -2694
EN 100 -4336

EN 120 -2815
EN 140 -1364
EN 160 -3902
EN 180 0
EN 200 0
EN 220 0
EN 240 -3714
EN 260 -4945
EN 280 -4134
EN 300 -3343
EN 320 -2456
EN 340 -2348
EN 360 0
A-RIB:A-R6:GLU-CA
EN 20 -9819
EN 40 -3084
EN 60 0
EN 80 -2219
EN 100 -1316
EN 120 192
EN 140 -2797
EN 160 -2561
EN 180 0
EN 200 0
EN 220 -5791
EN 240 -2533
EN 260 -976
EN 280 352
EN 300 -3184
EN 320 -3827
EN 340 0
EN 360 0
A-RIB:A-P:ASP-CA
EN 20 -9241
EN 40 0
EN 60 -2447
EN 80 -3326
EN 100 -741
EN 120 -3335
EN 140 754
EN 160 -2357
EN 180 -2384
EN 200 0
EN 220 -4708
EN 240 -3417
EN 260 -2198
EN 280 -2555
EN 300 -2371
EN 320 -356
EN 340 -3320
EN 360 -2552
C-RIB:C-Y:SER-S1
EN 20 -1971
EN 40 -4032
EN 60 -3631
EN 80 -4597
EN 100 -4519
EN 120 -2765

EN 140 -1806
EN 160 -852
EN 180 -3052
EN 200 -5404
EN 220 -4432
EN 240 -4042
EN 260 -3905
EN 280 -4964
EN 300 -3999
EN 320 -1630
EN 340 -4115
EN 360 0
C-RIB:C-P:LEU-S1
EN 20 0
EN 40 -4063
EN 60 -5683
EN 80 -4085
EN 100 -2201
EN 120 -4157
EN 140 -2314
EN 160 -1253
EN 180 -1787
EN 200 0
EN 220 -4114
EN 240 -866
EN 260 -5137
EN 280 -3117
EN 300 -2977
EN 320 -1837
EN 340 -2034
EN 360 -1967
IU-P:IU-RIB:PRO-S1
EN 20 0
EN 40 0
EN 60 0
EN 80 0
EN 100 0
EN 120 -12294
EN 140 0
EN 160 0
EN 180 0
EN 200 0
EN 220 0
EN 240 0
EN 260 0
EN 280 0
EN 300 0
EN 320 0
EN 340 0
EN 360 0
G-RIB:G-R6:TRP-S2
EN 20 0
EN 40 -8054
EN 60 0
EN 80 -4558
EN 100 -4782
EN 120 -8116
EN 140 -3743

EN 160 0
EN 180 -6434
EN 200 -12675
EN 220 -7231
EN 240 -5364
EN 260 -2667
EN 280 -5139
EN 300 -4607
EN 320 -5689
EN 340 0
EN 360 0
U-RIB:U-Y:GLU-S2
EN 20 -1841
EN 40 992
EN 60 -863
EN 80 48
EN 100 -341
EN 120 2043
EN 140 -1052
EN 160 -1832
EN 180 -4886
EN 200 -1851
EN 220 0
EN 240 -170
EN 260 -609
EN 280 -314
EN 300 2086
EN 320 0
EN 340 800
EN 360 0
C-P:C-RIB:LYS-CA
EN 20 -6385
EN 40 -6689
EN 60 -3737
EN 80 -3949
EN 100 -3158
EN 120 -3065
EN 140 -2503
EN 160 -3179
EN 180 -2917
EN 200 -6929
EN 220 -5814
EN 240 -3824
EN 260 -4992
EN 280 -3997
EN 300 -2695
EN 320 -4080
EN 340 -4253
EN 360 -2899
A-RIB:A-P:TRP-S1
EN 20 0
EN 40 -7472
EN 60 0
EN 80 -4667
EN 100 -2251
EN 120 -4877
EN 140 0
EN 160 -6530

EN 180 0
EN 200 0
EN 220 0
EN 240 0
EN 260 0
EN 280 -2483
EN 300 -2417
EN 320 -2854
EN 340 -3855
EN 360 -6849
A-RIB:A-R5:GLN-S1
EN 20 0
EN 40 -5032
EN 60 -3534
EN 80 -3592
EN 100 -3183
EN 120 -4881
EN 140 -4182
EN 160 -4336
EN 180 -6702
EN 200 -5566
EN 220 -5014
EN 240 -2323
EN 260 -4703
EN 280 -392
EN 300 -4127
EN 320 -3991
EN 340 0
EN 360 0
U31-P:U31-RIB:MET-S1
EN 20 0
EN 40 -13127
EN 60 0
EN 80 -12675
EN 100 0
EN 120 0
EN 140 0
EN 160 0
EN 180 0
EN 200 0
EN 220 0
EN 240 0
EN 260 0
EN 280 0
EN 300 0
EN 320 0
EN 340 0
EN 360 0
FHU-RIB:FHU-MY:ALA-S1
EN 20 0
EN 40 -17115
EN 60 0
EN 80 0
EN 100 0
EN 120 -10576
EN 140 0
EN 160 -14393
EN 180 0

EN 200 0
EN 220 0
EN 240 0
EN 260 0
EN 280 0
EN 300 -9819
EN 320 0
EN 340 0
EN 360 0
A-RIB:A-R6:CYS-CA
EN 20 0
EN 40 0
EN 60 -7231
EN 80 -5380
EN 100 0
EN 120 0
EN 140 -5253
EN 160 0
EN 180 0
EN 200 0
EN 220 0
EN 240 -6772
EN 260 0
EN 280 -4591
EN 300 -4647
EN 320 0
EN 340 0
EN 360 0
U31-RIB:U31-MY:THR-S1
EN 20 0
EN 40 0
EN 60 0
EN 80 -8378
EN 100 0
EN 120 0
EN 140 0
EN 160 -9953
EN 180 0
EN 200 0
EN 220 0
EN 240 0
EN 260 0
EN 280 0
EN 300 0
EN 320 -7796
EN 340 0
EN 360 0
C-RIB:C-Y:TRP-S2
EN 20 0
EN 40 -7854
EN 60 -3762
EN 80 -7201
EN 100 -4012
EN 120 -5871
EN 140 -6289
EN 160 -4255
EN 180 0
EN 200 -10405

EN 220 -5446
EN 240 0
EN 260 -3598
EN 280 -7182
EN 300 -6780
EN 320 -4530
EN 340 0
EN 360 0
DA-RIB:DA-M6:SER-S1
EN 20 0
EN 40 0
EN 60 0
EN 80 0
EN 100 0
EN 120 0
EN 140 -7575
EN 160 0
EN 180 0
EN 200 0
EN 220 0
EN 240 0
EN 260 0
EN 280 0
EN 300 0
EN 320 0
EN 340 0
EN 360 0
FHU-P:FHU-RIB:LYS-S1
EN 20 0
EN 40 -12675
EN 60 0
EN 80 0
EN 100 0
EN 120 0
EN 140 0
EN 160 0
EN 180 0
EN 200 0
EN 220 0
EN 240 -11174
EN 260 0
EN 280 0
EN 300 0
EN 320 0
EN 340 0
EN 360 0
U-P:U-RIB:ASN-S2
EN 20 -5274
EN 40 -4603
EN 60 -3295
EN 80 -4514
EN 100 -3769
EN 120 -4196
EN 140 -4154
EN 160 -4736
EN 180 -1876
EN 200 0
EN 220 -5505

EN 240 -4101
EN 260 -2191
EN 280 -3317
EN 300 -2768
EN 320 -4204
EN 340 -5579
EN 360 -4667
DA-RIB:DA-M5:TYR-CA
EN 20 0
EN 40 0
EN 60 0
EN 80 0
EN 100 0
EN 120 0
EN 140 0
EN 160 0
EN 180 0
EN 200 0
EN 220 0
EN 240 0
EN 260 0
EN 280 -8306
EN 300 -8772
EN 320 0
EN 340 0
EN 360 0
A-P:A-RIB:GLN-CA
EN 20 0
EN 40 0
EN 60 -4163
EN 80 -2977
EN 100 -2636
EN 120 -4691
EN 140 -319
EN 160 -869
EN 180 -3024
EN 200 0
EN 220 -6258
EN 240 -5103
EN 260 -5087
EN 280 -3753
EN 300 -2301
EN 320 -1767
EN 340 -2374
EN 360 -5522
A-RIB:A-P:ARG-CA
EN 20 0
EN 40 -5192
EN 60 -4592
EN 80 -4919
EN 100 -4583
EN 120 -5405
EN 140 -4661
EN 160 -4192
EN 180 -5333
EN 200 0
EN 220 -6451
EN 240 -5488

EN 260 -4378
EN 280 -4709
EN 300 -5547
EN 320 -5082
EN 340 -5515
EN 360 -5814
G-RIB:G-R6:GLU-S2
EN 20 -2915
EN 40 -3147
EN 60 -1659
EN 80 -394
EN 100 -1380
EN 120 -752
EN 140 332
EN 160 -3148
EN 180 -2617
EN 200 0
EN 220 1254
EN 240 -2463
EN 260 -1106
EN 280 -2
EN 300 1016
EN 320 -1143
EN 340 -855
EN 360 -2522
C-RIB:C-Y:GLU-S1
EN 20 -1989
EN 40 -1622
EN 60 943
EN 80 1023
EN 100 -2151
EN 120 0
EN 140 -1163
EN 160 -3684
EN 180 -1657
EN 200 0
EN 220 -1724
EN 240 -2471
EN 260 -1819
EN 280 2204
EN 300 0
EN 320 -1844
EN 340 609
EN 360 0
U-P:U-RIB:ASP-S1
EN 20 0
EN 40 -1531
EN 60 -1799
EN 80 318
EN 100 -3032
EN 120 -2258
EN 140 -1662
EN 160 -1565
EN 180 -2403
EN 200 0
EN 220 -2866
EN 240 -666
EN 260 2009

EN 280 443
EN 300 -634
EN 320 -2212
EN 340 -2877
EN 360 -827
G-RIB:G-R5:VAL-CA
EN 20 0
EN 40 -1744
EN 60 -248
EN 80 -196
EN 100 -4182
EN 120 -1891
EN 140 -2333
EN 160 -1524
EN 180 -4430
EN 200 -6523
EN 220 -3365
EN 240 -4007
EN 260 -1706
EN 280 -3939
EN 300 -34
EN 320 -539
EN 340 -3353
EN 360 0
G-P:G-RIB:ARG-S1
EN 20 -7112
EN 40 -4784
EN 60 -4842
EN 80 -4997
EN 100 -2879
EN 120 -3738
EN 140 -2430
EN 160 -3983
EN 180 -1423
EN 200 -7083
EN 220 -3330
EN 240 -5615
EN 260 -4520
EN 280 -4313
EN 300 -4282
EN 320 -3869
EN 340 -1140
EN 360 -3225
FHU-RIB:FHU-MY:LYS-S1
EN 20 0
EN 40 0
EN 60 0
EN 80 -9819
EN 100 0
EN 120 0
EN 140 0
EN 160 0
EN 180 0
EN 200 0
EN 220 0
EN 240 0
EN 260 -8772
EN 280 0

EN 300 0
EN 320 0
EN 340 0
EN 360 0
IU-P:IU-RIB:LYS-S2
EN 20 0
EN 40 0
EN 60 0
EN 80 0
EN 100 0
EN 120 0
EN 140 0
EN 160 -8688
EN 180 0
EN 200 0
EN 220 -11671
EN 240 0
EN 260 0
EN 280 0
EN 300 0
EN 320 0
EN 340 0
EN 360 0
QUO-P:QUO-RIB:PHE-S2
EN 20 0
EN 40 0
EN 60 0
EN 80 0
EN 100 0
EN 120 0
EN 140 0
EN 160 0
EN 180 0
EN 200 0
EN 220 0
EN 240 0
EN 260 0
EN 280 0
EN 300 -5152
EN 320 0
EN 340 0
EN 360 0
C31-RIB:C31-MY:GLN-S2
EN 20 0
EN 40 0
EN 60 0
EN 80 0
EN 100 0
EN 120 0
EN 140 0
EN 160 0
EN 180 0
EN 200 0
EN 220 0
EN 240 0
EN 260 0
EN 280 0
EN 300 -11410

EN 320 0
EN 340 0
EN 360 0
A-RIB:A-P:TRP-CA
EN 20 0
EN 40 0
EN 60 -4961
EN 80 -4943
EN 100 -4095
EN 120 -4998
EN 140 -2815
EN 160 -3641
EN 180 0
EN 200 0
EN 220 0
EN 240 0
EN 260 -3866
EN 280 -2463
EN 300 -2463
EN 320 -3154
EN 340 -4050
EN 360 -7054
G-RIB:G-R5:PRO-CA
EN 20 0
EN 40 -4411
EN 60 -3903
EN 80 -2110
EN 100 -2243
EN 120 -1693
EN 140 -3371
EN 160 0
EN 180 0
EN 200 0
EN 220 -6180
EN 240 -3636
EN 260 -2820
EN 280 -3118
EN 300 -3
EN 320 -2406
EN 340 -1577
EN 360 -6448
U-RIB:U-P:HIS-S1
EN 20 0
EN 40 -7683
EN 60 -5514
EN 80 -4468
EN 100 -2096
EN 120 -2014
EN 140 -4088
EN 160 -5093
EN 180 -5832
EN 200 0
EN 220 0
EN 240 -6484
EN 260 -5934
EN 280 -4207
EN 300 -2901
EN 320 -4587

EN 340 -4489
EN 360 0
U-RIB:U-Y:PRO-CA
EN 20 0
EN 40 -4116
EN 60 -5412
EN 80 -4561
EN 100 -327
EN 120 -574
EN 140 -3703
EN 160 -2235
EN 180 -4375
EN 200 0
EN 220 0
EN 240 -3688
EN 260 -3314
EN 280 -4085
EN 300 -5291
EN 320 -2954
EN 340 -5023
EN 360 -4853
U-RIB:U-P:LYS-S2
EN 20 -5755
EN 40 -1948
EN 60 -2968
EN 80 -4094
EN 100 -4766
EN 120 -3907
EN 140 -4048
EN 160 -4821
EN 180 -108
EN 200 0
EN 220 -3575
EN 240 -3306
EN 260 -3572
EN 280 -4343
EN 300 -3507
EN 320 -4005
EN 340 -3708
EN 360 -3649
U31-RIB:U31-MY:PHE-S2
EN 20 0
EN 40 0
EN 60 0
EN 80 0
EN 100 0
EN 120 0
EN 140 0
EN 160 0
EN 180 0
EN 200 0
EN 220 0
EN 240 0
EN 260 0
EN 280 -9456
EN 300 0
EN 320 0
EN 340 0

EN 360 0
QUO-RIB:QUO-M5:ASP-CA
EN 20 0
EN 40 0
EN 60 0
EN 80 0
EN 100 0
EN 120 -3434
EN 140 0
EN 160 0
EN 180 0
EN 200 0
EN 220 0
EN 240 0
EN 260 0
EN 280 0
EN 300 0
EN 320 -3434
EN 340 0
EN 360 0
G-P:G-RIB:ARG-CA
EN 20 0
EN 40 -1148
EN 60 -4163
EN 80 -3989
EN 100 -4711
EN 120 -3285
EN 140 -4165
EN 160 -4518
EN 180 -3366
EN 200 -6289
EN 220 -2954
EN 240 -5125
EN 260 -4676
EN 280 -4119
EN 300 -2573
EN 320 -4061
EN 340 -4327
EN 360 -2440
FHU-RIB:FHU-P:LEU-S1
EN 20 0
EN 40 0
EN 60 -12294
EN 80 0
EN 100 0
EN 120 0
EN 140 0
EN 160 -9042
EN 180 0
EN 200 0
EN 220 0
EN 240 -14393
EN 260 0
EN 280 0
EN 300 0
EN 320 0
EN 340 -11963
EN 360 0

A-RIB:A-R6:LEU-S1
EN 20 0
EN 40 -7611
EN 60 -3865
EN 80 -5936
EN 100 -5498
EN 120 -4308
EN 140 -698
EN 160 -3408
EN 180 0
EN 200 0
EN 220 -8156
EN 240 -2105
EN 260 -5189
EN 280 -5571
EN 300 -5818
EN 320 -4130
EN 340 -1696
EN 360 -4498
G-RIB:G-R5:VAL-S1
EN 20 0
EN 40 -2150
EN 60 301
EN 80 -2899
EN 100 -2628
EN 120 -2874
EN 140 -587
EN 160 0
EN 180 -5593
EN 200 -4956
EN 220 -3963
EN 240 -2841
EN 260 -1851
EN 280 -2496
EN 300 -2696
EN 320 -3245
EN 340 0
EN 360 0
C-RIB:C-P:ARG-S2
EN 20 -4180
EN 40 -6346
EN 60 -4533
EN 80 -4758
EN 100 -4944
EN 120 -4508
EN 140 -4542
EN 160 -4962
EN 180 -4426
EN 200 0
EN 220 -4241
EN 240 -3977
EN 260 -4839
EN 280 -4848
EN 300 -3675
EN 320 -4502
EN 340 -4384
EN 360 -3553
FMU-RIB:FMU-MY:ASN-S1

EN 20 0
EN 40 0
EN 60 0
EN 80 0
EN 100 -14393
EN 120 0
EN 140 0
EN 160 0
EN 180 0
EN 200 0
EN 220 0
EN 240 0
EN 260 0
EN 280 0
EN 300 0
EN 320 0
EN 340 0
EN 360 0
A-P:A-RIB:LYS-CA
EN 20 -8606
EN 40 -6101
EN 60 -3424
EN 80 -3295
EN 100 -4356
EN 120 -3582
EN 140 -2943
EN 160 -3040
EN 180 -2333
EN 200 0
EN 220 -5692
EN 240 -4492
EN 260 -4168
EN 280 -4686
EN 300 -3942
EN 320 -3593
EN 340 -3817
EN 360 -2413
FMU-RIB:FMU-MY:GLU-S2
EN 20 0
EN 40 0
EN 60 0
EN 80 0
EN 100 0
EN 120 0
EN 140 0
EN 160 0
EN 180 0
EN 200 0
EN 220 0
EN 240 0
EN 260 0
EN 280 0
EN 300 0
EN 320 -11963
EN 340 0
EN 360 0
G-RIB:G-R6:ASN-S1
EN 20 0

EN 40 -2417
EN 60 -4091
EN 80 -4632
EN 100 -3910
EN 120 -4301
EN 140 -4005
EN 160 -2319
EN 180 -3276
EN 200 0
EN 220 -5456
EN 240 -1986
EN 260 -3507
EN 280 -3246
EN 300 -4894
EN 320 -1913
EN 340 -949
EN 360 0
U31-RIB:U31-MY:GLU-S2
EN 20 0
EN 40 0
EN 60 0
EN 80 0
EN 100 0
EN 120 0
EN 140 0
EN 160 -7575
EN 180 0
EN 200 0
EN 220 0
EN 240 0
EN 260 -7186
EN 280 0
EN 300 0
EN 320 0
EN 340 0
EN 360 0
A-RIB:A-P:LYS-S2
EN 20 -5112
EN 40 -3257
EN 60 -2980
EN 80 -4560
EN 100 -4230
EN 120 -3916
EN 140 -3695
EN 160 -4959
EN 180 -4867
EN 200 0
EN 220 -1364
EN 240 -4340
EN 260 -3121
EN 280 -4305
EN 300 -3661
EN 320 -4791
EN 340 -3562
EN 360 -3689
FHU-RIB:FHU-MY:ALA-CA
EN 20 0
EN 40 -17115

EN 60 0
EN 80 0
EN 100 0
EN 120 -10760
EN 140 0
EN 160 0
EN 180 0
EN 200 0
EN 220 0
EN 240 0
EN 260 0
EN 280 0
EN 300 -9953
EN 320 0
EN 340 0
EN 360 0
A-RIB:A-P:PRO-S1
EN 20 0
EN 40 -2233
EN 60 -4436
EN 80 -2723
EN 100 -4212
EN 120 -2277
EN 140 -1282
EN 160 -4517
EN 180 0
EN 200 0
EN 220 -7305
EN 240 -2707
EN 260 -3310
EN 280 -3883
EN 300 -3212
EN 320 -4107
EN 340 -4635
EN 360 -1886
A-RIB:A-R6:VAL-S1
EN 20 0
EN 40 -5152
EN 60 -3761
EN 80 -5792
EN 100 -4306
EN 120 -4141
EN 140 -3731
EN 160 -4643
EN 180 0
EN 200 0
EN 220 -5245
EN 240 -3456
EN 260 -4105
EN 280 -2416
EN 300 -5111
EN 320 -2758
EN 340 -2775
EN 360 0
G-RIB:G-P:LEU-S1
EN 20 0
EN 40 -4390
EN 60 -5097

EN 80 -3970
EN 100 -3316
EN 120 -2832
EN 140 -2129
EN 160 -678
EN 180 -4332
EN 200 -9346
EN 220 -3914
EN 240 -6163
EN 260 -2983
EN 280 -2966
EN 300 -2610
EN 320 -2907
EN 340 -742
EN 360 -1712
U-P:U-RIB:ALA-CA
EN 20 -10144
EN 40 -4000
EN 60 -4801
EN 80 -4441
EN 100 -3815
EN 120 -2636
EN 140 -3792
EN 160 -3321
EN 180 -2371
EN 200 0
EN 220 -4121
EN 240 -3464
EN 260 -4122
EN 280 -4119
EN 300 -3376
EN 320 -3816
EN 340 -3147
EN 360 -4069
C-RIB:C-P:PHE-CA
EN 20 0
EN 40 0
EN 60 -4269
EN 80 -2777
EN 100 -2622
EN 120 416
EN 140 -12
EN 160 -5241
EN 180 0
EN 200 0
EN 220 0
EN 240 -3872
EN 260 -3981
EN 280 -3787
EN 300 -2428
EN 320 0
EN 340 -2765
EN 360 0
A-RIB:A-P:ASN-S1
EN 20 -7854
EN 40 -2917
EN 60 -1962
EN 80 -3788

EN 100 -5083
EN 120 -3215
EN 140 -1517
EN 160 -4797
EN 180 -2185
EN 200 0
EN 220 -5132
EN 240 -5192
EN 260 -3895
EN 280 -3668
EN 300 -2609
EN 320 -3218
EN 340 -4742
EN 360 -2051
A-RIB:A-R6:PRO-S1
EN 20 0
EN 40 -2726
EN 60 -1911
EN 80 -3339
EN 100 -4923
EN 120 -4045
EN 140 -4453
EN 160 -4509
EN 180 -2952
EN 200 0
EN 220 -5192
EN 240 -4427
EN 260 -2063
EN 280 -4709
EN 300 -3573
EN 320 -4088
EN 340 -434
EN 360 -3001
G-RIB:G-R5:TYR-S1
EN 20 0
EN 40 0
EN 60 -2599
EN 80 -5917
EN 100 -2517
EN 120 -2989
EN 140 0
EN 160 -3963
EN 180 -7054
EN 200 0
EN 220 -5637
EN 240 -2836
EN 260 -5312
EN 280 -2583
EN 300 -4847
EN 320 -6345
EN 340 0
EN 360 0
A-RIB:A-R6:ASP-S2
EN 20 0
EN 40 -3473
EN 60 -1844
EN 80 -381
EN 100 -1097

EN 120	-1105
EN 140	-796
EN 160	-2408
EN 180	-1481
EN 200	-5637
EN 220	-2466
EN 240	-3057
EN 260	-371
EN 280	-3265
EN 300	-3156
EN 320	-865
EN 340	-807
EN 360	-3385
G-RIB:G-P:TRP-S1
EN 20 0
EN 40 -6258
EN 60 -6889
EN 80 0
EN 100	-5705
EN 120	0
EN 140	-1668
EN 160	-4479
EN 180	-5036
EN 200	0
EN 220	0
EN 240	-3588
EN 260	-3831
EN 280	-3366
EN 300	-2995
EN 320	-3371
EN 340	0
EN 360	-5232
U31-RIB:U31-P:TYR-S1
EN 20 0
EN 40 0
EN 60 0
EN 80 -9139
EN 100	0
EN 120	0
EN 140	0
EN 160	0
EN 180	0
EN 200	0
EN 220	0
EN 240	0
EN 260	0
EN 280	0
EN 300	0
EN 320	0
EN 340	0
EN 360	0
H2U-RIB:H2U-MY:TRP-S1
EN 20 0
EN 40 0
EN 60 0
EN 80 0
EN 100	0
EN 120	0

EN 140 0
EN 160 0
EN 180 0
EN 200 0
EN 220 0
EN 240 0
EN 260 0
EN 280 0
EN 300 0
EN 320 -17115
EN 340 0
EN 360 0
C-P:C-RIB:ASN-S1
EN 20 0
EN 40 -5906
EN 60 -4718
EN 80 -4314
EN 100 -4632
EN 120 -3493
EN 140 -3287
EN 160 -1645
EN 180 -2507
EN 200 -5584
EN 220 -4818
EN 240 -5577
EN 260 -2788
EN 280 -3228
EN 300 -3347
EN 320 -3238
EN 340 -606
EN 360 -4259
G-P:G-RIB:MET-S1
EN 20 0
EN 40 -3926
EN 60 -1678
EN 80 -2541
EN 100 -2443
EN 120 -838
EN 140 -3991
EN 160 -3718
EN 180 -6984
EN 200 -8236
EN 220 -3546
EN 240 -3212
EN 260 -3451
EN 280 -3145
EN 300 -3225
EN 320 -5316
EN 340 -1972
EN 360 -7819
G-P:G-RIB:MET-CA
EN 20 -10405
EN 40 0
EN 60 -4925
EN 80 -859
EN 100 -2443
EN 120 -3532
EN 140 -2889

EN 160 -6016
EN 180 -6093
EN 200 0
EN 220 0
EN 240 -2062
EN 260 -4405
EN 280 -3244
EN 300 -2273
EN 320 -3553
EN 340 -5440
EN 360 -4000
FMU-P:FMU-RIB:MET-S1
EN 20 0
EN 40 0
EN 60 0
EN 80 0
EN 100 0
EN 120 0
EN 140 0
EN 160 0
EN 180 0
EN 200 0
EN 220 0
EN 240 0
EN 260 0
EN 280 0
EN 300 0
EN 320 -2722
EN 340 0
EN 360 0
G-RIB:G-R5:GLY-CA
EN 20 -5730
EN 40 -4069
EN 60 -4317
EN 80 -4256
EN 100 -2293
EN 120 -3314
EN 140 80
EN 160 -2896
EN 180 -1531
EN 200 -5745
EN 220 -5220
EN 240 -4111
EN 260 -3483
EN 280 -4339
EN 300 -2623
EN 320 -2781
EN 340 -3526
EN 360 -1481
U-RIB:U-Y:GLU-S1
EN 20 -3673
EN 40 -4073
EN 60 -371
EN 80 160
EN 100 96
EN 120 0
EN 140 1129
EN 160 -1397

EN 180 -2054
EN 200 0
EN 220 -21
EN 240 -304
EN 260 1804
EN 280 -28
EN 300 -165
EN 320 0
EN 340 212
EN 360 0
A-RIB:A-R5:MET-S2
EN 20 0
EN 40 -5197
EN 60 -5439
EN 80 -2279
EN 100 -5591
EN 120 -6783
EN 140 -5918
EN 160 -6919
EN 180 0
EN 200 0
EN 220 -3556
EN 240 -2741
EN 260 -4783
EN 280 0
EN 300 -5791
EN 320 -5952
EN 340 0
EN 360 -9190
U-P:U-RIB:VAL-S1
EN 20 0
EN 40 -2709
EN 60 -3159
EN 80 -3680
EN 100 -3855
EN 120 -3971
EN 140 -2519
EN 160 -2423
EN 180 -2559
EN 200 0
EN 220 0
EN 240 -494
EN 260 -3389
EN 280 -2665
EN 300 714
EN 320 -4490
EN 340 -2140
EN 360 0
I-P:I-RIB:GLY-CA
EN 20 0
EN 40 0
EN 60 0
EN 80 0
EN 100 0
EN 120 0
EN 140 0
EN 160 -1717
EN 180 0

EN 200 0
EN 220 0
EN 240 0
EN 260 0
EN 280 0
EN 300 0
EN 320 0
EN 340 0
EN 360 0
A-RIB:A-R6:LEU-CA
EN 20 0
EN 40 -5884
EN 60 -4096
EN 80 -6019
EN 100 -4973
EN 120 -5046
EN 140 -3380
EN 160 0
EN 180 -4898
EN 200 0
EN 220 -7478
EN 240 0
EN 260 -4704
EN 280 -4633
EN 300 -4986
EN 320 -4475
EN 340 -1709
EN 360 0
C-RIB:C-Y:CYS-CA
EN 20 0
EN 40 0
EN 60 -6849
EN 80 0
EN 100 0
EN 120 -6050
EN 140 0
EN 160 0
EN 180 0
EN 200 0
EN 220 0
EN 240 0
EN 260 0
EN 280 0
EN 300 -5461
EN 320 0
EN 340 0
EN 360 0
A-RIB:A-R6:ARG-S1
EN 20 0
EN 40 -6537
EN 60 -5083
EN 80 -4596
EN 100 -4824
EN 120 -3500
EN 140 -3642
EN 160 -4483
EN 180 -3684
EN 200 0

EN 220 -4669
EN 240 -4410
EN 260 -3664
EN 280 -2665
EN 300 -2051
EN 320 -3726
EN 340 -3257
EN 360 -1746
C-P:C-RIB:ASP-S2
EN 20 0
EN 40 -1048
EN 60 -794
EN 80 -2547
EN 100 -1937
EN 120 -1918
EN 140 -2775
EN 160 -3643
EN 180 -2413
EN 200 0
EN 220 -1048
EN 240 -1754
EN 260 -2871
EN 280 -1201
EN 300 -2445
EN 320 -2887
EN 340 -2008
EN 360 -3532
A-P:A-RIB:GLN-S1
EN 20 0
EN 40 -5713
EN 60 -3902
EN 80 -3090
EN 100 -4186
EN 120 -2779
EN 140 -4626
EN 160 -330
EN 180 -4943
EN 200 -7629
EN 220 0
EN 240 -5077
EN 260 -3804
EN 280 -5037
EN 300 -3755
EN 320 -1373
EN 340 -1594
EN 360 -3820
FHU-P:FHU-RIB:ASP-S1
EN 20 0
EN 40 0
EN 60 0
EN 80 0
EN 100 0
EN 120 0
EN 140 0
EN 160 -14393
EN 180 0
EN 200 0
EN 220 0

EN 240 0
EN 260 0
EN 280 0
EN 300 0
EN 320 -11410
EN 340 0
EN 360 0
G-RIB:G-R5:MET-CA
EN 20 -6501
EN 40 0
EN 60 -4544
EN 80 -7289
EN 100 -2685
EN 120 0
EN 140 0
EN 160 0
EN 180 0
EN 200 0
EN 220 0
EN 240 -4710
EN 260 -4529
EN 280 -6581
EN 300 0
EN 320 -6212
EN 340 -4823
EN 360 -8022
A-RIB:A-P:GLU-CA
EN 20 0
EN 40 -5537
EN 60 -1154
EN 80 -1202
EN 100 -750
EN 120 591
EN 140 197
EN 160 -1464
EN 180 0
EN 200 0
EN 220 -4801
EN 240 -2999
EN 260 -2212
EN 280 -888
EN 300 -1049
EN 320 -1066
EN 340 0
EN 360 0
U-RIB:U-P:LYS-S1
EN 20 0
EN 40 -1929
EN 60 -995
EN 80 -5089
EN 100 -4692
EN 120 -4525
EN 140 -3791
EN 160 -4804
EN 180 -4245
EN 200 0
EN 220 -3987
EN 240 -1129

EN 260 -4199
EN 280 -2231
EN 300 -3332
EN 320 -3649
EN 340 -3947
EN 360 -4519
A-RIB:A-R6:TRP-CA
EN 20 0
EN 40 0
EN 60 -5424
EN 80 -4651
EN 100 -3378
EN 120 -3843
EN 140 0
EN 160 0
EN 180 0
EN 200 0
EN 220 0
EN 240 -5584
EN 260 -6648
EN 280 -3569
EN 300 -3347
EN 320 0
EN 340 -4494
EN 360 0
FHU-RIB:FHU-MY:PRO-S1
EN 20 0
EN 40 0
EN 60 0
EN 80 0
EN 100 -9953
EN 120 0
EN 140 0
EN 160 0
EN 180 0
EN 200 0
EN 220 0
EN 240 0
EN 260 0
EN 280 0
EN 300 0
EN 320 0
EN 340 0
EN 360 0
G-RIB:G-P:ASN-S1
EN 20 0
EN 40 -3825
EN 60 -5392
EN 80 -4338
EN 100 -2671
EN 120 -3201
EN 140 -2783
EN 160 -5179
EN 180 -5058
EN 200 0
EN 220 -2702
EN 240 -3673
EN 260 -4568

EN 280 -3592
EN 300 -3250
EN 320 -3502
EN 340 -3180
EN 360 -3886
C-RIB:C-P:LEU-S2
EN 20 0
EN 40 -3405
EN 60 -5427
EN 80 -3746
EN 100 -2741
EN 120 -2880
EN 140 -3171
EN 160 -2676
EN 180 0
EN 200 0
EN 220 -4979
EN 240 -3899
EN 260 -3177
EN 280 -3254
EN 300 -4852
EN 320 -1138
EN 340 -2100
EN 360 -3479
U-RIB:U-Y:TYR-S2
EN 20 0
EN 40 -4509
EN 60 0
EN 80 -5352
EN 100 -3922
EN 120 -3634
EN 140 -3896
EN 160 -3124
EN 180 0
EN 200 0
EN 220 -4414
EN 240 -4885
EN 260 -5314
EN 280 -5698
EN 300 -6681
EN 320 -5920
EN 340 0
EN 360 0
A-RIB:A-R6:PHE-S2
EN 20 0
EN 40 -8387
EN 60 0
EN 80 -4969
EN 100 -5155
EN 120 -4853
EN 140 -2566
EN 160 0
EN 180 0
EN 200 0
EN 220 -7952
EN 240 -3207
EN 260 -6725
EN 280 -2215

EN 300 -2180
EN 320 0
EN 340 -6909
EN 360 0
IU-RIB:IU-MY:LEU-S1
EN 20 0
EN 40 0
EN 60 -13388
EN 80 0
EN 100 0
EN 120 0
EN 140 0
EN 160 0
EN 180 0
EN 200 0
EN 220 0
EN 240 0
EN 260 0
EN 280 0
EN 300 0
EN 320 0
EN 340 0
EN 360 0
A-RIB:A-P:ASP-S2
EN 20 0
EN 40 -4040
EN 60 -1949
EN 80 -1118
EN 100 -1806
EN 120 -2112
EN 140 -2142
EN 160 -2644
EN 180 0
EN 200 0
EN 220 0
EN 240 -2531
EN 260 -2126
EN 280 -1596
EN 300 -1112
EN 320 -1944
EN 340 -490
EN 360 -1775
C31-RIB:C31-MY:THR-S1
EN 20 0
EN 40 0
EN 60 0
EN 80 0
EN 100 0
EN 120 0
EN 140 0
EN 160 0
EN 180 0
EN 200 0
EN 220 -10405
EN 240 0
EN 260 0
EN 280 0
EN 300 0

EN 320 0
EN 340 0
EN 360 0
G-RIB:G-R5:TRP-S1
EN 20 -6859
EN 40 -4318
EN 60 0
EN 80 -5144
EN 100 -8188
EN 120 -5777
EN 140 0
EN 160 0
EN 180 0
EN 200 0
EN 220 -6683
EN 240 -5353
EN 260 0
EN 280 -5062
EN 300 -6606
EN 320 0
EN 340 -5761
EN 360 0
GTP-RIB:GTP-M5:THR-CA
EN 20 0
EN 40 0
EN 60 0
EN 80 0
EN 100 -10576
EN 120 0
EN 140 0
EN 160 0
EN 180 0
EN 200 0
EN 220 0
EN 240 0
EN 260 0
EN 280 0
EN 300 0
EN 320 0
EN 340 0
EN 360 0
U-RIB:U-P:ASN-S2
EN 20 0
EN 40 -3751
EN 60 -1777
EN 80 -3638
EN 100 -4144
EN 120 -3958
EN 140 -4309
EN 160 -3016
EN 180 -2125
EN 200 -6660
EN 220 -5316
EN 240 -3802
EN 260 -4211
EN 280 -3355
EN 300 -3001
EN 320 -4318

EN 340 451
EN 360 -2227
FHU-RIB:FHU-P:LYS-S1
EN 20 0
EN 40 0
EN 60 0
EN 80 0
EN 100 0
EN 120 -8949
EN 140 -8606
EN 160 0
EN 180 0
EN 200 0
EN 220 0
EN 240 0
EN 260 0
EN 280 0
EN 300 -9346
EN 320 -8606
EN 340 0
EN 360 0
C-P:C-RIB:CYS-S1
EN 20 0
EN 40 0
EN 60 -5938
EN 80 0
EN 100 -6035
EN 120 0
EN 140 -4494
EN 160 0
EN 180 0
EN 200 0
EN 220 0
EN 240 0
EN 260 -5938
EN 280 -7781
EN 300 0
EN 320 -4818
EN 340 0
EN 360 0
A-RIB:A-R6:MET-S2
EN 20 0
EN 40 -5021
EN 60 -3058
EN 80 -6590
EN 100 -1982
EN 120 -4525
EN 140 -2363
EN 160 -4902
EN 180 0
EN 200 0
EN 220 -7076
EN 240 -3427
EN 260 -2204
EN 280 -5648
EN 300 -3784
EN 320 -6411
EN 340 -5204

EN 360 0
U-RIB:U-P:GLU-S2
EN 20 0
EN 40 204
EN 60 -473
EN 80 -691
EN 100 -964
EN 120 -2031
EN 140 589
EN 160 -941
EN 180 -3680
EN 200 0
EN 220 -1945
EN 240 -511
EN 260 1643
EN 280 -183
EN 300 -728
EN 320 -2448
EN 340 -845
EN 360 -2542
A-RIB:A-P:ALA-S1
EN 20 0
EN 40 -1212
EN 60 -4441
EN 80 -3726
EN 100 -2893
EN 120 -2417
EN 140 -3651
EN 160 -4148
EN 180 -1140
EN 200 0
EN 220 0
EN 240 -4211
EN 260 -2759
EN 280 -3883
EN 300 -3262
EN 320 -2701
EN 340 -4402
EN 360 -1467
C-RIB:C-P:TRP-S1
EN 20 0
EN 40 0
EN 60 -4167
EN 80 -4154
EN 100 -1762
EN 120 -1697
EN 140 -3588
EN 160 -2999
EN 180 0
EN 200 0
EN 220 -8202
EN 240 -5596
EN 260 -4095
EN 280 -3495
EN 300 0
EN 320 -5816
EN 340 0
EN 360 0

A-RIB:A-R6:ASN-S2
EN 20 0
EN 40 -6125
EN 60 -1521
EN 80 -3477
EN 100 -3875
EN 120 -2559
EN 140 -3963
EN 160 -2306
EN 180 -3105
EN 200 0
EN 220 -5708
EN 240 -4989
EN 260 -4933
EN 280 -4027
EN 300 -1875
EN 320 -3936
EN 340 -3997
EN 360 -4945
U-RIB:U-Y:ILE-CA
EN 20 0
EN 40 0
EN 60 -4099
EN 80 -4439
EN 100 -1153
EN 120 -2612
EN 140 -1545
EN 160 -2988
EN 180 0
EN 200 0
EN 220 0
EN 240 -2463
EN 260 -1740
EN 280 -4096
EN 300 -4549
EN 320 -3075
EN 340 -2503
EN 360 0
GTP-RIB:GTP-M6:SER-S1
EN 20 0
EN 40 0
EN 60 -8236
EN 80 0
EN 100 0
EN 120 0
EN 140 0
EN 160 0
EN 180 0
EN 200 0
EN 220 0
EN 240 0
EN 260 0
EN 280 -8236
EN 300 0
EN 320 0
EN 340 0
EN 360 0
A-RIB:A-R5:ARG-S2

EN 20 -6289
EN 40 -4924
EN 60 -5610
EN 80 -4300
EN 100 -4738
EN 120 -4940
EN 140 -4324
EN 160 -2067
EN 180 -3706
EN 200 -6840
EN 220 -3796
EN 240 -4043
EN 260 -4057
EN 280 -4940
EN 300 -4702
EN 320 -4137
EN 340 -2614
EN 360 -1821
U34-P:U34-RIB:ASN-S2
EN 20 0
EN 40 0
EN 60 -11671
EN 80 -9042
EN 100 0
EN 120 0
EN 140 0
EN 160 0
EN 180 0
EN 200 0
EN 220 0
EN 240 0
EN 260 0
EN 280 0
EN 300 -8452
EN 320 0
EN 340 0
EN 360 0
5BU-RIB:5BU-P:ARG-S1
EN 20 0
EN 40 0
EN 60 0
EN 80 0
EN 100 0
EN 120 0
EN 140 -11671
EN 160 0
EN 180 0
EN 200 0
EN 220 0
EN 240 0
EN 260 0
EN 280 0
EN 300 0
EN 320 0
EN 340 0
EN 360 0
A-P:A-RIB:GLU-S2
EN 20 0

EN 40 1192
EN 60 -406
EN 80 -1157
EN 100 1899
EN 120 -2759
EN 140 -956
EN 160 -2397
EN 180 -1818
EN 200 0
EN 220 0
EN 240 1196
EN 260 -1133
EN 280 -1076
EN 300 -1158
EN 320 -2017
EN 340 -995
EN 360 877
C-P:C-RIB:MET-S1
EN 20 0
EN 40 0
EN 60 0
EN 80 -5789
EN 100 -5028
EN 120 -2720
EN 140 0
EN 160 -4540
EN 180 -6672
EN 200 0
EN 220 0
EN 240 -4998
EN 260 -2987
EN 280 -3542
EN 300 -6160
EN 320 -2991
EN 340 0
EN 360 -3740
C-RIB:C-P:ILE-CA
EN 20 0
EN 40 0
EN 60 -6156
EN 80 -3410
EN 100 -771
EN 120 -4970
EN 140 -2578
EN 160 -1975
EN 180 -2823
EN 200 0
EN 220 0
EN 240 0
EN 260 -5074
EN 280 -1747
EN 300 -3474
EN 320 -3344
EN 340 -3388
EN 360 -2450
C-RIB:C-P:THR-S1
EN 20 -6078
EN 40 -5337

EN 60 -4421
EN 80 -4211
EN 100 -3453
EN 120 -3104
EN 140 -4378
EN 160 -4371
EN 180 -2754
EN 200 0
EN 220 -4514
EN 240 -3379
EN 260 -3862
EN 280 -3176
EN 300 -3962
EN 320 -2253
EN 340 -4012
EN 360 -3812
A-P:A-RIB:THR-CA
EN 20 0
EN 40 -6510
EN 60 -4553
EN 80 -4209
EN 100 -4687
EN 120 -4496
EN 140 -3699
EN 160 -1665
EN 180 -3449
EN 200 0
EN 220 -3154
EN 240 -5554
EN 260 -4272
EN 280 -3053
EN 300 -3758
EN 320 -5175
EN 340 -4091
EN 360 -1860
G-RIB:G-P:LYS-CA
EN 20 0
EN 40 -3673
EN 60 -3320
EN 80 -4118
EN 100 -3299
EN 120 -4493
EN 140 -3448
EN 160 -3514
EN 180 -4805
EN 200 0
EN 220 -4228
EN 240 -5614
EN 260 -3720
EN 280 -3724
EN 300 -3382
EN 320 -4074
EN 340 -5183
EN 360 -4825
G-RIB:G-R6:LEU-CA
EN 20 0
EN 40 -8533
EN 60 -2133

EN 80 -4065
EN 100 -2844
EN 120 -261
EN 140 -3281
EN 160 -5076
EN 180 0
EN 200 0
EN 220 -6924
EN 240 -4552
EN 260 -6521
EN 280 -4230
EN 300 -3628
EN 320 -4717
EN 340 0
EN 360 0
H2U-RIB:H2U-MY:LEU-S1
EN 20 0
EN 40 0
EN 60 0
EN 80 0
EN 100 0
EN 120 0
EN 140 0
EN 160 -11174
EN 180 0
EN 200 0
EN 220 0
EN 240 0
EN 260 0
EN 280 0
EN 300 0
EN 320 -7739
EN 340 0
EN 360 0
A-RIB:A-R6:TYR-S2
EN 20 0
EN 40 -8956
EN 60 -5452
EN 80 -5928
EN 100 -5665
EN 120 -4113
EN 140 -3516
EN 160 -2771
EN 180 0
EN 200 0
EN 220 -7711
EN 240 -5463
EN 260 -4339
EN 280 -2877
EN 300 -1598
EN 320 -1786
EN 340 -5384
EN 360 0
A-RIB:A-P:TRP-S2
EN 20 -12294
EN 40 0
EN 60 -6916
EN 80 -2629

EN 100 -2000
EN 120 0
EN 140 -2658
EN 160 -3495
EN 180 -5656
EN 200 0
EN 220 0
EN 240 -5993
EN 260 0
EN 280 -2215
EN 300 -2045
EN 320 -5185
EN 340 0
EN 360 -5966
FHU-RIB:FHU-MY:LEU-S1
EN 20 0
EN 40 0
EN 60 0
EN 80 -12294
EN 100 0
EN 120 0
EN 140 -11174
EN 160 -11410
EN 180 0
EN 200 0
EN 220 0
EN 240 0
EN 260 -9953
EN 280 0
EN 300 0
EN 320 0
EN 340 -12675
EN 360 0
H2U-RIB:H2U-P:LYS-S2
EN 20 0
EN 40 -11174
EN 60 0
EN 80 0
EN 100 0
EN 120 0
EN 140 0
EN 160 0
EN 180 -11671
EN 200 0
EN 220 0
EN 240 0
EN 260 0
EN 280 0
EN 300 0
EN 320 0
EN 340 0
EN 360 0
A-P:A-RIB:ALA-CA
EN 20 -6660
EN 40 -4504
EN 60 -3484
EN 80 -3734
EN 100 -3305

EN 120 -3247
EN 140 -2423
EN 160 -1714
EN 180 0
EN 200 0
EN 220 -5348
EN 240 -4004
EN 260 -1992
EN 280 -2950
EN 300 -3571
EN 320 -3261
EN 340 -2384
EN 360 0
U-P:U-RIB:PRO-S1
EN 20 -6050
EN 40 0
EN 60 -4818
EN 80 -2707
EN 100 -2934
EN 120 -3060
EN 140 -3370
EN 160 -3368
EN 180 -1918
EN 200 0
EN 220 -3802
EN 240 -1687
EN 260 -3966
EN 280 -3681
EN 300 -1644
EN 320 -4299
EN 340 -1483
EN 360 -4706
C-RIB:C-Y:PRO-CA
EN 20 0
EN 40 -6088
EN 60 -4869
EN 80 -3843
EN 100 -4302
EN 120 -3601
EN 140 -2409
EN 160 -5286
EN 180 0
EN 200 -7439
EN 220 -3118
EN 240 -3541
EN 260 491
EN 280 -3837
EN 300 -4364
EN 320 -2300
EN 340 -2034
EN 360 0
A-RIB:A-P:THR-CA
EN 20 0
EN 40 -4464
EN 60 -3991
EN 80 -4355
EN 100 -5274
EN 120 -4464

EN 140 -5023
EN 160 -3676
EN 180 -1918
EN 200 0
EN 220 -4290
EN 240 -6652
EN 260 -4213
EN 280 -3869
EN 300 -3590
EN 320 -3271
EN 340 302
EN 360 -6553
U-P:U-RIB:TYR-S1
EN 20 0
EN 40 0
EN 60 0
EN 80 -5619
EN 100 -1531
EN 120 -1834
EN 140 -5842
EN 160 -4147
EN 180 0
EN 200 0
EN 220 0
EN 240 0
EN 260 0
EN 280 -6907
EN 300 -1388
EN 320 -2022
EN 340 -6653
EN 360 -5112
H2U-RIB:H2U-MY:TRP-CA
EN 20 0
EN 40 0
EN 60 0
EN 80 0
EN 100 0
EN 120 0
EN 140 0
EN 160 0
EN 180 0
EN 200 0
EN 220 0
EN 240 0
EN 260 0
EN 280 0
EN 300 0
EN 320 -15398
EN 340 0
EN 360 0
FMU-P:FMU-RIB:ARG-S1
EN 20 0
EN 40 0
EN 60 0
EN 80 0
EN 100 0
EN 120 -14393
EN 140 0

EN 160 0
EN 180 0
EN 200 0
EN 220 0
EN 240 -15398
EN 260 0
EN 280 0
EN 300 0
EN 320 0
EN 340 0
EN 360 0
G-P:G-RIB:THR-S1
EN 20 -6709
EN 40 -3047
EN 60 -1297
EN 80 -3378
EN 100 -3824
EN 120 -2175
EN 140 -3609
EN 160 -2841
EN 180 -2538
EN 200 -7083
EN 220 -4470
EN 240 -1291
EN 260 -3244
EN 280 -2546
EN 300 -2162
EN 320 -947
EN 340 -2188
EN 360 -3577
G-RIB:G-R5:THR-CA
EN 20 -4544
EN 40 -3446
EN 60 -136
EN 80 -3617
EN 100 -1334
EN 120 55
EN 140 -305
EN 160 -3512
EN 180 0
EN 200 -4631
EN 220 -5308
EN 240 -4708
EN 260 -3877
EN 280 -3374
EN 300 -2422
EN 320 -3625
EN 340 0
EN 360 0
FHU-RIB:FHU-P:LEU-CA
EN 20 0
EN 40 0
EN 60 -12675
EN 80 0
EN 100 0
EN 120 0
EN 140 0
EN 160 -9456

EN 180 0
EN 200 0
EN 220 0
EN 240 -17115
EN 260 0
EN 280 0
EN 300 0
EN 320 0
EN 340 -9456
EN 360 -11963
G-P:G-RIB:ALA-S1
EN 20 -6212
EN 40 576
EN 60 -1829
EN 80 -3059
EN 100 -2701
EN 120 -3275
EN 140 -3114
EN 160 -2487
EN 180 -3923
EN 200 -4750
EN 220 -4211
EN 240 -2663
EN 260 -2000
EN 280 -1495
EN 300 -2768
EN 320 -836
EN 340 -2912
EN 360 -562
G-RIB:G-R5:LYS-CA
EN 20 -5902
EN 40 -2685
EN 60 -2848
EN 80 -2158
EN 100 -3447
EN 120 -3894
EN 140 -3174
EN 160 -3519
EN 180 -4325
EN 200 -7150
EN 220 -3910
EN 240 -2281
EN 260 -3436
EN 280 -4499
EN 300 -5481
EN 320 -4769
EN 340 -4571
EN 360 -2627
U-RIB:U-Y:HIS-S2
EN 20 -7767
EN 40 0
EN 60 -5434
EN 80 -4419
EN 100 -5605
EN 120 -5459
EN 140 -4929
EN 160 0
EN 180 0

EN 200 0
EN 220 -4675
EN 240 -6227
EN 260 -4016
EN 280 -3118
EN 300 -1555
EN 320 -2129
EN 340 0
EN 360 -6963
A-P:A-RIB:ARG-S2
EN 20 -8038
EN 40 -5940
EN 60 -5808
EN 80 -4804
EN 100 -4728
EN 120 -4495
EN 140 -3892
EN 160 -1747
EN 180 -3234
EN 200 -8750
EN 220 -6166
EN 240 -4720
EN 260 -5531
EN 280 -4258
EN 300 -4254
EN 320 -2950
EN 340 -4032
EN 360 -3908
FHU-RIB:FHU-P:HIS-S2
EN 20 0
EN 40 0
EN 60 0
EN 80 0
EN 100 0
EN 120 0
EN 140 0
EN 160 0
EN 180 0
EN 200 0
EN 220 0
EN 240 0
EN 260 0
EN 280 -12123
EN 300 0
EN 320 0
EN 340 0
EN 360 0
A-RIB:A-R6:GLU-S1
EN 20 0
EN 40 -3189
EN 60 -1342
EN 80 -1921
EN 100 -2375
EN 120 -1488
EN 140 -567
EN 160 -1324
EN 180 -2954
EN 200 0

EN 220 -4240
EN 240 -1308
EN 260 -1441
EN 280 13
EN 300 -2309
EN 320 -569
EN 340 -1339
EN 360 0
G-RIB:G-P:TYR-S2
EN 20 0
EN 40 0
EN 60 -3469
EN 80 -4985
EN 100 -3744
EN 120 -4471
EN 140 -4006
EN 160 -5061
EN 180 -2942
EN 200 0
EN 220 0
EN 240 -3395
EN 260 -4545
EN 280 -1210
EN 300 -2052
EN 320 -3232
EN 340 -3415
EN 360 0
FMU-RIB:FMU-P:PHE-CA
EN 20 0
EN 40 0
EN 60 0
EN 80 0
EN 100 0
EN 120 -17115
EN 140 0
EN 160 0
EN 180 0
EN 200 0
EN 220 0
EN 240 0
EN 260 0
EN 280 0
EN 300 0
EN 320 0
EN 340 0
EN 360 0
U-RIB:U-Y:PHE-CA
EN 20 0
EN 40 0
EN 60 -6263
EN 80 -3874
EN 100 0
EN 120 -4459
EN 140 -3634
EN 160 0
EN 180 0
EN 200 0
EN 220 -8059

EN 240 -4629
EN 260 -5420
EN 280 -7922
EN 300 -6642
EN 320 -3030
EN 340 0
EN 360 0
IU-RIB:IU-MY:PRO-S1
EN 20 0
EN 40 0
EN 60 0
EN 80 -11100
EN 100 0
EN 120 0
EN 140 0
EN 160 0
EN 180 0
EN 200 0
EN 220 0
EN 240 0
EN 260 0
EN 280 0
EN 300 -12477
EN 320 0
EN 340 0
EN 360 0
C31-RIB:C31-MY:GLU-S1
EN 20 0
EN 40 0
EN 60 0
EN 80 0
EN 100 0
EN 120 0
EN 140 -8236
EN 160 0
EN 180 0
EN 200 0
EN 220 0
EN 240 0
EN 260 0
EN 280 0
EN 300 0
EN 320 0
EN 340 0
EN 360 0
C31-RIB:C31-P:THR-CA
EN 20 0
EN 40 0
EN 60 -9139
EN 80 0
EN 100 -9241
EN 120 0
EN 140 0
EN 160 0
EN 180 0
EN 200 0
EN 220 0
EN 240 0

EN 260 0
EN 280 0
EN 300 0
EN 320 0
EN 340 0
EN 360 0
A-P:A-RIB:LEU-CA
EN 20 0
EN 40 -8102
EN 60 -1563
EN 80 236
EN 100 -3330
EN 120 -4434
EN 140 -3628
EN 160 -4525
EN 180 -5529
EN 200 0
EN 220 -4871
EN 240 0
EN 260 -4060
EN 280 -2278
EN 300 -3994
EN 320 -4351
EN 340 -3061
EN 360 -4532
G-RIB:G-R6:VAL-S1
EN 20 0
EN 40 -2078
EN 60 0
EN 80 482
EN 100 -3205
EN 120 -1124
EN 140 0
EN 160 0
EN 180 -5980
EN 200 0
EN 220 -2248
EN 240 -4010
EN 260 -3694
EN 280 -580
EN 300 -3194
EN 320 -3344
EN 340 -931
EN 360 -5174
C-P:C-RIB:HIS-CA
EN 20 0
EN 40 0
EN 60 -1865
EN 80 -5322
EN 100 -4063
EN 120 -1891
EN 140 0
EN 160 -5915
EN 180 -4889
EN 200 0
EN 220 0
EN 240 -4684
EN 260 -766

EN 280	-4133
EN 300	-5898
EN 320	-4310
EN 340	-3532
EN 360	-5789
U-RIB:U-Y:MET-CA
EN 20 0
EN 40 -5858
EN 60 -7529
EN 80 -5647
EN 100	0
EN 120	0
EN 140	0
EN 160	0
EN 180	0
EN 200	0
EN 220	-8624
EN 240	-4608
EN 260	-5845
EN 280	0
EN 300	-3598
EN 320	-3906
EN 340	0
EN 360	0
G-RIB:G-P:ASP-S2
EN 20 0
EN 40 -2447
EN 60 -2504
EN 80 -1175
EN 100	-2794
EN 120	-2375
EN 140	-1539
EN 160	-1666
EN 180	-3189
EN 200	0
EN 220	-3495
EN 240	-1161
EN 260	-1560
EN 280	-2956
EN 300	-2059
EN 320	-1296
EN 340	-3622
EN 360	-2021
QUO-P:QUO-RIB:PHE-S1
EN 20 0
EN 40 0
EN 60 0
EN 80 0
EN 100	0
EN 120	0
EN 140	0
EN 160	0
EN 180	0
EN 200	0
EN 220	0
EN 240	0
EN 260	0
EN 280	0

EN 300 -3434
EN 320 0
EN 340 0
EN 360 0
C-RIB:C-P:ASP-S1
EN 20 0
EN 40 -4815
EN 60 -3385
EN 80 -2406
EN 100 -3175
EN 120 -2002
EN 140 -2529
EN 160 -2722
EN 180 -1685
EN 200 0
EN 220 -4529
EN 240 -3795
EN 260 -1387
EN 280 -1529
EN 300 -2636
EN 320 -309
EN 340 -2103
EN 360 101
G-RIB:G-R6:ALA-CA
EN 20 0
EN 40 -1706
EN 60 -1135
EN 80 -3161
EN 100 -2020
EN 120 -2655
EN 140 -2755
EN 160 -3153
EN 180 -2170
EN 200 0
EN 220 -6205
EN 240 -3193
EN 260 -3176
EN 280 -779
EN 300 -3771
EN 320 -618
EN 340 137
EN 360 0
A-P:A-RIB:HIS-CA
EN 20 0
EN 40 -7777
EN 60 -2423
EN 80 -4464
EN 100 -4632
EN 120 -3508
EN 140 -973
EN 160 -4947
EN 180 -5132
EN 200 0
EN 220 0
EN 240 -4050
EN 260 -6178
EN 280 -4150
EN 300 -6996

EN 320 -5480
EN 340 -6499
EN 360 -3464
A-RIB:A-R6:ASP-S1
EN 20 0
EN 40 -3439
EN 60 -688
EN 80 167
EN 100 -1284
EN 120 -2024
EN 140 -1900
EN 160 -2011
EN 180 0
EN 200 0
EN 220 -3473
EN 240 -1575
EN 260 -1436
EN 280 -2284
EN 300 -2882
EN 320 -1971
EN 340 618
EN 360 -1721
G-RIB:G-P:HIS-S2
EN 20 0
EN 40 -4692
EN 60 -5118
EN 80 -3413
EN 100 -4039
EN 120 -3116
EN 140 -2798
EN 160 -2360
EN 180 0
EN 200 0
EN 220 -5772
EN 240 -2999
EN 260 -3392
EN 280 -5289
EN 300 -3287
EN 320 -3869
EN 340 -3669
EN 360 -3641
G-RIB:G-P:ASN-CA
EN 20 0
EN 40 -3866
EN 60 -4143
EN 80 -3450
EN 100 -3605
EN 120 -3679
EN 140 -3581
EN 160 -1851
EN 180 -6098
EN 200 -13388
EN 220 -3975
EN 240 -4476
EN 260 -4268
EN 280 -2705
EN 300 -3842
EN 320 -3361

EN 340 -3546
EN 360 -3140
A-RIB:A-R5:TYR-CA
EN 20 0
EN 40 -5074
EN 60 -6892
EN 80 -7451
EN 100 -5592
EN 120 -6480
EN 140 -5560
EN 160 0
EN 180 -9164
EN 200 0
EN 220 -6801
EN 240 -6320
EN 260 -6913
EN 280 -5574
EN 300 -4673
EN 320 -6442
EN 340 -4444
EN 360 0
FHU-P:FHU-RIB:ALA-S1
EN 20 0
EN 40 0
EN 60 0
EN 80 0
EN 100 -12891
EN 120 -11174
EN 140 0
EN 160 0
EN 180 0
EN 200 0
EN 220 0
EN 240 0
EN 260 0
EN 280 -10245
EN 300 -12294
EN 320 0
EN 340 0
EN 360 0
QUO-RIB:QUO-M6:LYS-S2
EN 20 0
EN 40 0
EN 60 -3434
EN 80 0
EN 100 0
EN 120 0
EN 140 0
EN 160 0
EN 180 0
EN 200 0
EN 220 0
EN 240 0
EN 260 0
EN 280 0
EN 300 0
EN 320 0
EN 340 0

EN 360 0
G-RIB:G-R5:ARG-S1
EN 20 -6925
EN 40 -4028
EN 60 -3483
EN 80 -3757
EN 100 -3677
EN 120 -3630
EN 140 -4071
EN 160 -5264
EN 180 -4039
EN 200 -6189
EN 220 -828
EN 240 -3323
EN 260 -3848
EN 280 -2953
EN 300 -4782
EN 320 -4947
EN 340 -5191
EN 360 -4911
A-RIB:A-P:LYS-CA
EN 20 0
EN 40 -5639
EN 60 -5407
EN 80 -3974
EN 100 -4284
EN 120 -5040
EN 140 -3957
EN 160 -5544
EN 180 -4532
EN 200 0
EN 220 -3405
EN 240 -5318
EN 260 -3103
EN 280 -3515
EN 300 -3424
EN 320 -2417
EN 340 -4912
EN 360 -5795
A-P:A-RIB:LYS-S1
EN 20 -4961
EN 40 -3395
EN 60 -4267
EN 80 -4260
EN 100 -3943
EN 120 -2126
EN 140 -3707
EN 160 -1569
EN 180 -2794
EN 200 -7834
EN 220 -4537
EN 240 -4940
EN 260 -4667
EN 280 -2111
EN 300 -3233
EN 320 -4660
EN 340 -3797
EN 360 -3354

C-RIB:C-P:CYS-S1
EN 20 0
EN 40 0
EN 60 -6772
EN 80 -6984
EN 100 0
EN 120 -2777
EN 140 0
EN 160 0
EN 180 0
EN 200 0
EN 220 0
EN 240 -6227
EN 260 0
EN 280 -3619
EN 300 -5284
EN 320 0
EN 340 0
EN 360 -7629
5BU-RIB:5BU-MY:ARG-S2
EN 20 0
EN 40 0
EN 60 0
EN 80 0
EN 100 0
EN 120 0
EN 140 0
EN 160 0
EN 180 0
EN 200 0
EN 220 0
EN 240 0
EN 260 -14393
EN 280 0
EN 300 0
EN 320 0
EN 340 0
EN 360 0
C-RIB:C-Y:SER-CA
EN 20 -6496
EN 40 -5163
EN 60 -4350
EN 80 -3920
EN 100 -3978
EN 120 -2515
EN 140 -463
EN 160 -4101
EN 180 -3759
EN 200 0
EN 220 -4527
EN 240 -4147
EN 260 -4619
EN 280 -4149
EN 300 -3169
EN 320 -3065
EN 340 -3862
EN 360 0
U-P:U-RIB:PHE-CA

EN 20 0
EN 40 0
EN 60 -2777
EN 80 -5337
EN 100 -4025
EN 120 -5031
EN 140 -5572
EN 160 -2901
EN 180 0
EN 200 0
EN 220 0
EN 240 -4961
EN 260 0
EN 280 -3101
EN 300 -6311
EN 320 -5253
EN 340 -5631
EN 360 0
A-RIB:A-R6:MET-S1
EN 20 0
EN 40 0
EN 60 -6519
EN 80 -5584
EN 100 -3899
EN 120 -4816
EN 140 -5319
EN 160 -4947
EN 180 0
EN 200 0
EN 220 0
EN 240 -5235
EN 260 0
EN 280 -5075
EN 300 -6208
EN 320 -2581
EN 340 -3902
EN 360 -6467
A-RIB:A-R5:PHE-S1
EN 20 -8378
EN 40 -5279
EN 60 -5402
EN 80 -4581
EN 100 -2879
EN 120 -3127
EN 140 0
EN 160 0
EN 180 0
EN 200 0
EN 220 -7515
EN 240 -3693
EN 260 -4902
EN 280 -5149
EN 300 -6796
EN 320 0
EN 340 0
EN 360 0
G-RIB:G-R5:GLU-S2
EN 20 -1585

EN 40 1015
EN 60 -1236
EN 80 -1597
EN 100 764
EN 120 -1167
EN 140 -2781
EN 160 -16
EN 180 -662
EN 200 238
EN 220 47
EN 240 -1212
EN 260 -875
EN 280 192
EN 300 -1104
EN 320 -1048
EN 340 -953
EN 360 0
G-RIB:G-P:ALA-CA
EN 20 0
EN 40 0
EN 60 -2978
EN 80 -1461
EN 100 -2361
EN 120 -3172
EN 140 -1636
EN 160 -2164
EN 180 0
EN 200 0
EN 220 -5770
EN 240 -3398
EN 260 -2870
EN 280 -2727
EN 300 -2660
EN 320 -2741
EN 340 -3350
EN 360 -1981
FHU-P:FHU-RIB:SER-CA
EN 20 0
EN 40 0
EN 60 0
EN 80 0
EN 100 0
EN 120 0
EN 140 -15398
EN 160 0
EN 180 0
EN 200 0
EN 220 0
EN 240 0
EN 260 0
EN 280 0
EN 300 0
EN 320 -11410
EN 340 0
EN 360 0
G-RIB:G-P:GLN-S1
EN 20 0
EN 40 -7805

EN 60 -3582
EN 80 -3051
EN 100 -2188
EN 120 -2900
EN 140 -3003
EN 160 -2524
EN 180 -6218
EN 200 0
EN 220 -3127
EN 240 -4188
EN 260 -4286
EN 280 -2648
EN 300 -3204
EN 320 -2936
EN 340 -5261
EN 360 -4080
A-RIB:A-R5:LEU-S1
EN 20 0
EN 40 -4552
EN 60 -1675
EN 80 -6156
EN 100 -6285
EN 120 -4222
EN 140 0
EN 160 -3292
EN 180 0
EN 200 0
EN 220 -7769
EN 240 -5232
EN 260 -5287
EN 280 -5586
EN 300 -5363
EN 320 -1978
EN 340 -5869
EN 360 0
FHU-RIB:FHU-P:LYS-CA
EN 20 0
EN 40 0
EN 60 0
EN 80 0
EN 100 -10760
EN 120 -9571
EN 140 0
EN 160 0
EN 180 0
EN 200 0
EN 220 0
EN 240 0
EN 260 0
EN 280 0
EN 300 -9346
EN 320 0
EN 340 0
EN 360 -13127
A-RIB:A-R5:PHE-CA
EN 20 0
EN 40 0
EN 60 -5026

EN 80 0
EN 100 -5662
EN 120 -4818
EN 140 -3978
EN 160 0
EN 180 0
EN 200 -8341
EN 220 -4490
EN 240 -4984
EN 260 -4733
EN 280 -5896
EN 300 -6052
EN 320 0
EN 340 0
EN 360 0
H2U-RIB:H2U-P:THR-CA
EN 20 0
EN 40 0
EN 60 0
EN 80 0
EN 100 0
EN 120 -14393
EN 140 0
EN 160 0
EN 180 0
EN 200 0
EN 220 0
EN 240 0
EN 260 0
EN 280 0
EN 300 0
EN 320 0
EN 340 0
EN 360 0
FHU-RIB:FHU-MY:THR-S1
EN 20 0
EN 40 0
EN 60 0
EN 80 0
EN 100 -9346
EN 120 0
EN 140 0
EN 160 0
EN 180 0
EN 200 0
EN 220 -2722
EN 240 0
EN 260 0
EN 280 -11963
EN 300 0
EN 320 0
EN 340 0
EN 360 0
FMU-RIB:FMU-MY:MET-CA
EN 20 0
EN 40 0
EN 60 0
EN 80 0

EN 100 0
EN 120 0
EN 140 0
EN 160 0
EN 180 0
EN 200 0
EN 220 0
EN 240 0
EN 260 0
EN 280 -14393
EN 300 0
EN 320 0
EN 340 0
EN 360 0
G-RIB:G-P:ILE-S1
EN 20 0
EN 40 -4419
EN 60 -4571
EN 80 -2305
EN 100 -2714
EN 120 -4142
EN 140 -2914
EN 160 -1561
EN 180 0
EN 200 0
EN 220 -5358
EN 240 -4354
EN 260 -2932
EN 280 -2061
EN 300 -2025
EN 320 -2336
EN 340 -1554
EN 360 -2179
G-RIB:G-R5:GLN-S2
EN 20 -2334
EN 40 -3121
EN 60 -5220
EN 80 -4019
EN 100 -4865
EN 120 -3220
EN 140 -109
EN 160 0
EN 180 0
EN 200 -4793
EN 220 271
EN 240 -3529
EN 260 -4932
EN 280 -4315
EN 300 -3075
EN 320 -184
EN 340 -905
EN 360 -3043
U-P:U-RIB:PHE-S2
EN 20 0
EN 40 -5858
EN 60 -2566
EN 80 -3267
EN 100 -6214

EN 120 -6162
EN 140 -5435
EN 160 -2966
EN 180 0
EN 200 0
EN 220 0
EN 240 -5615
EN 260 0
EN 280 -1549
EN 300 -5898
EN 320 -6190
EN 340 -4675
EN 360 -6536
U-RIB:U-P:GLN-S2
EN 20 -6451
EN 40 -3938
EN 60 -3502
EN 80 -2841
EN 100 -3780
EN 120 -2069
EN 140 -1528
EN 160 -2375
EN 180 -3797
EN 200 0
EN 220 -6174
EN 240 -2729
EN 260 -1631
EN 280 -3578
EN 300 -3820
EN 320 -2412
EN 340 -3216
EN 360 0
A-P:A-RIB:GLU-CA
EN 20 0
EN 40 -3136
EN 60 0
EN 80 -3215
EN 100 -2745
EN 120 -2524
EN 140 -1660
EN 160 -41
EN 180 0
EN 200 0
EN 220 0
EN 240 0
EN 260 -1111
EN 280 -676
EN 300 -2096
EN 320 -383
EN 340 -1518
EN 360 -269
C-RIB:C-Y:HIS-S2
EN 20 -5316
EN 40 -5482
EN 60 -3256
EN 80 -4830
EN 100 -6080
EN 120 -3110

EN 140 -3491
EN 160 -2508
EN 180 0
EN 200 0
EN 220 -4487
EN 240 -5956
EN 260 -4814
EN 280 -4939
EN 300 -3282
EN 320 0
EN 340 -2641
EN 360 -5061
FHU-RIB:FHU-P:THR-CA
EN 20 0
EN 40 0
EN 60 -11963
EN 80 -10760
EN 100 0
EN 120 0
EN 140 0
EN 160 0
EN 180 0
EN 200 0
EN 220 0
EN 240 0
EN 260 -11963
EN 280 0
EN 300 0
EN 320 -9819
EN 340 0
EN 360 0
DA-RIB:DA-M5:THR-CA
EN 20 0
EN 40 0
EN 60 0
EN 80 0
EN 100 0
EN 120 0
EN 140 0
EN 160 0
EN 180 0
EN 200 0
EN 220 0
EN 240 0
EN 260 0
EN 280 0
EN 300 0
EN 320 -7796
EN 340 0
EN 360 0
A-RIB:A-P:SER-S1
EN 20 0
EN 40 -5867
EN 60 -4859
EN 80 -4401
EN 100 -2101
EN 120 -4310
EN 140 -4918

EN 160 -2262
EN 180 -5274
EN 200 0
EN 220 -3230
EN 240 -2954
EN 260 -4640
EN 280 -3249
EN 300 -2709
EN 320 -4975
EN 340 -3845
EN 360 -5168
FMU-P:FMU-RIB:HIS-S1
EN 20 0
EN 40 0
EN 60 0
EN 80 0
EN 100 0
EN 120 0
EN 140 0
EN 160 -17115
EN 180 0
EN 200 0
EN 220 0
EN 240 0
EN 260 0
EN 280 0
EN 300 0
EN 320 0
EN 340 0
EN 360 0
FHU-RIB:FHU-MY:ASP-CA
EN 20 0
EN 40 0
EN 60 0
EN 80 0
EN 100 -9953
EN 120 0
EN 140 0
EN 160 0
EN 180 0
EN 200 0
EN 220 0
EN 240 0
EN 260 0
EN 280 -9139
EN 300 0
EN 320 0
EN 340 0
EN 360 0
U34-RIB:U34-P:GLU-CA
EN 20 0
EN 40 0
EN 60 0
EN 80 0
EN 100 0
EN 120 0
EN 140 0
EN 160 0

EN 180 0
EN 200 0
EN 220 0
EN 240 0
EN 260 0
EN 280 0
EN 300 0
EN 320 0
EN 340 0
EN 360 -14393
QUO-RIB:QUO-M5:GLU-S2
EN 20 0
EN 40 0
EN 60 0
EN 80 0
EN 100 0
EN 120 0
EN 140 -3434
EN 160 0
EN 180 0
EN 200 0
EN 220 0
EN 240 0
EN 260 0
EN 280 0
EN 300 0
EN 320 0
EN 340 0
EN 360 0
DA-RIB:DA-M5:GLU-S2
EN 20 0
EN 40 0
EN 60 0
EN 80 0
EN 100 0
EN 120 0
EN 140 0
EN 160 0
EN 180 0
EN 200 0
EN 220 0
EN 240 0
EN 260 0
EN 280 0
EN 300 0
EN 320 -7472
EN 340 0
EN 360 0
G-RIB:G-R6:CYS-S1
EN 20 0
EN 40 0
EN 60 0
EN 80 0
EN 100 0
EN 120 0
EN 140 -6242
EN 160 -6297
EN 180 0

EN 200 0
EN 220 0
EN 240 -5966
EN 260 0
EN 280 0
EN 300 -4853
EN 320 0
EN 340 0
EN 360 0
H2U-RIB:H2U-P:TRP-S1
EN 20 0
EN 40 0
EN 60 0
EN 80 0
EN 100 -17115
EN 120 0
EN 140 0
EN 160 0
EN 180 0
EN 200 0
EN 220 0
EN 240 0
EN 260 0
EN 280 0
EN 300 0
EN 320 0
EN 340 0
EN 360 0
IU-RIB:IU-P:LYS-S2
EN 20 0
EN 40 0
EN 60 0
EN 80 0
EN 100 0
EN 120 0
EN 140 -9241
EN 160 0
EN 180 0
EN 200 0
EN 220 0
EN 240 0
EN 260 0
EN 280 0
EN 300 -8688
EN 320 0
EN 340 0
EN 360 0
U31-P:U31-RIB:ASP-S1
EN 20 0
EN 40 -12294
EN 60 0
EN 80 -8236
EN 100 0
EN 120 0
EN 140 0
EN 160 -7914
EN 180 0
EN 200 0

EN 220 0
EN 240 0
EN 260 0
EN 280 0
EN 300 0
EN 320 -7472
EN 340 0
EN 360 0
U-P:U-RIB:CYS-CA
EN 20 0
EN 40 0
EN 60 0
EN 80 0
EN 100 0
EN 120 0
EN 140 -6588
EN 160 -6166
EN 180 -8306
EN 200 0
EN 220 -9241
EN 240 0
EN 260 -7012
EN 280 0
EN 300 0
EN 320 0
EN 340 0
EN 360 0
G-RIB:G-R5:PHE-CA
EN 20 -9382
EN 40 -3805
EN 60 -4840
EN 80 -5787
EN 100 -4211
EN 120 0
EN 140 0
EN 160 -4430
EN 180 0
EN 200 -7209
EN 220 -3902
EN 240 -5858
EN 260 -5738
EN 280 -2548
EN 300 0
EN 320 -3276
EN 340 0
EN 360 0
U-RIB:U-Y:ASP-S2
EN 20 -2535
EN 40 -1499
EN 60 -1906
EN 80 -2261
EN 100 1471
EN 120 -1535
EN 140 -3264
EN 160 -3001
EN 180 0
EN 200 -2751
EN 220 239

EN 240 -2998
EN 260 -955
EN 280 1492
EN 300 -445
EN 320 755
EN 340 -2735
EN 360 0
C-RIB:C-Y:ASP-S1
EN 20 -5248
EN 40 -3902
EN 60 -2371
EN 80 -2949
EN 100 -3402
EN 120 -392
EN 140 -2624
EN 160 -3553
EN 180 0
EN 200 -4318
EN 220 -3260
EN 240 -2449
EN 260 -3055
EN 280 -1098
EN 300 -359
EN 320 -4328
EN 340 -2691
EN 360 -2580
C-RIB:C-P:VAL-S1
EN 20 0
EN 40 -4063
EN 60 -4779
EN 80 -4349
EN 100 -4653
EN 120 -2713
EN 140 -3904
EN 160 -3515
EN 180 -3376
EN 200 0
EN 220 -5596
EN 240 -5600
EN 260 -4394
EN 280 -2872
EN 300 -2621
EN 320 -4730
EN 340 -1755
EN 360 -1414
C31-P:C31-RIB:TYR-S2
EN 20 0
EN 40 0
EN 60 -10245
EN 80 0
EN 100 0
EN 120 0
EN 140 0
EN 160 0
EN 180 0
EN 200 0
EN 220 0
EN 240 0

EN 260 -7854
EN 280 0
EN 300 0
EN 320 0
EN 340 0
EN 360 0
U31-RIB:U31-MY:GLU-S1
EN 20 0
EN 40 0
EN 60 0
EN 80 0
EN 100 0
EN 120 0
EN 140 -6624
EN 160 0
EN 180 0
EN 200 0
EN 220 0
EN 240 0
EN 260 0
EN 280 0
EN 300 0
EN 320 0
EN 340 0
EN 360 0
A-RIB:A-R5:CYS-S1
EN 20 0
EN 40 -7975
EN 60 0
EN 80 -8477
EN 100 0
EN 120 0
EN 140 0
EN 160 0
EN 180 0
EN 200 0
EN 220 0
EN 240 -7629
EN 260 0
EN 280 -8350
EN 300 -6706
EN 320 0
EN 340 0
EN 360 0
U-RIB:U-Y:CYS-S1
EN 20 0
EN 40 0
EN 60 0
EN 80 -6810
EN 100 0
EN 120 0
EN 140 -5902
EN 160 0
EN 180 0
EN 200 0
EN 220 -9494
EN 240 -6970
EN 260 0

EN 280 -8390
EN 300 -6722
EN 320 -6078
EN 340 0
EN 360 0
U-P:U-RIB:GLN-S1
EN 20 0
EN 40 -5738
EN 60 -3192
EN 80 -2257
EN 100 -2745
EN 120 -2133
EN 140 -3489
EN 160 -3281
EN 180 -4361
EN 200 0
EN 220 -2615
EN 240 -3281
EN 260 -2913
EN 280 -3727
EN 300 -2083
EN 320 -4603
EN 340 -3024
EN 360 -5125
G-RIB:G-P:GLU-S2
EN 20 0
EN 40 -414
EN 60 -16
EN 80 642
EN 100 -1030
EN 120 -839
EN 140 -1259
EN 160 -2117
EN 180 1764
EN 200 0
EN 220 -2262
EN 240 -1748
EN 260 3006
EN 280 -254
EN 300 -874
EN 320 -701
EN 340 -386
EN 360 -976
G-RIB:G-P:PHE-S2
EN 20 0
EN 40 -7767
EN 60 -6372
EN 80 -3975
EN 100 -3924
EN 120 -4149
EN 140 -2559
EN 160 -4760
EN 180 0
EN 200 0
EN 220 -5858
EN 240 -2510
EN 260 -666
EN 280 -1517

EN 300 354
EN 320 -3897
EN 340 -948
EN 360 -3118
FMU-RIB:FMU-MY:GLU-CA
EN 20 0
EN 40 0
EN 60 0
EN 80 0
EN 100 0
EN 120 0
EN 140 0
EN 160 0
EN 180 0
EN 200 0
EN 220 0
EN 240 0
EN 260 0
EN 280 0
EN 300 -11963
EN 320 0
EN 340 0
EN 360 0
A-RIB:A-R6:ALA-S1
EN 20 0
EN 40 -3019
EN 60 -3924
EN 80 -2681
EN 100 -1911
EN 120 -1814
EN 140 -1455
EN 160 -1342
EN 180 0
EN 200 0
EN 220 -3834
EN 240 -3044
EN 260 -521
EN 280 -1975
EN 300 -3574
EN 320 -1427
EN 340 -2362
EN 360 -2048
FHU-P:FHU-RIB:ARG-S2
EN 20 0
EN 40 0
EN 60 -7739
EN 80 0
EN 100 0
EN 120 0
EN 140 0
EN 160 0
EN 180 -2722
EN 200 0
EN 220 0
EN 240 -8102
EN 260 -7796
EN 280 0
EN 300 0

EN 320 0
EN 340 0
EN 360 -14393
G-RIB:G-R5:GLN-S1
EN 20 0
EN 40 -6016
EN 60 -5588
EN 80 -4188
EN 100 -275
EN 120 -552
EN 140 0
EN 160 -1589
EN 180 0
EN 200 -7201
EN 220 -5923
EN 240 -3903
EN 260 -5974
EN 280 -2842
EN 300 -3018
EN 320 0
EN 340 -1761
EN 360 0
U34-RIB:U34-P:TYR-CA
EN 20 0
EN 40 0
EN 60 0
EN 80 -13127
EN 100 0
EN 120 0
EN 140 0
EN 160 0
EN 180 0
EN 200 0
EN 220 0
EN 240 0
EN 260 0
EN 280 0
EN 300 0
EN 320 -15398
EN 340 0
EN 360 0
U31-RIB:U31-MY:PHE-S1
EN 20 0
EN 40 0
EN 60 0
EN 80 0
EN 100 0
EN 120 0
EN 140 0
EN 160 0
EN 180 0
EN 200 0
EN 220 0
EN 240 0
EN 260 0
EN 280 -8378
EN 300 0
EN 320 0

EN 340 0
EN 360 0
U31-P:U31-RIB:PHE-S1
EN 20 0
EN 40 0
EN 60 0
EN 80 0
EN 100 0
EN 120 0
EN 140 0
EN 160 0
EN 180 0
EN 200 0
EN 220 0
EN 240 0
EN 260 0
EN 280 0
EN 300 -9571
EN 320 0
EN 340 0
EN 360 0
A-RIB:A-R6:PHE-S1
EN 20 0
EN 40 -9456
EN 60 -4114
EN 80 0
EN 100 -6422
EN 120 -4061
EN 140 0
EN 160 0
EN 180 0
EN 200 0
EN 220 0
EN 240 0
EN 260 -5582
EN 280 -4425
EN 300 -4226
EN 320 0
EN 340 0
EN 360 0
U-RIB:U-P:PHE-CA
EN 20 0
EN 40 0
EN 60 -5480
EN 80 -3762
EN 100 0
EN 120 -3369
EN 140 -3804
EN 160 -2191
EN 180 -4818
EN 200 0
EN 220 -8528
EN 240 -5337
EN 260 -4592
EN 280 -4259
EN 300 0
EN 320 -2796
EN 340 0

EN 360	-5017
U31-P:U31-RIB:ASP-CA
EN 20 0
EN 40 -13127
EN 60 0
EN 80 -8038
EN 100	0
EN 120	0
EN 140	-11671
EN 160	0
EN 180	0
EN 200	0
EN 220	0
EN 240	0
EN 260	0
EN 280	0
EN 300	0
EN 320	-8306
EN 340	0
EN 360	0
FHU-P:FHU-RIB:TYR-CA
EN 20 0
EN 40 0
EN 60 0
EN 80 0
EN 100	0
EN 120	0
EN 140	0
EN 160	0
EN 180	0
EN 200	0
EN 220	0
EN 240	0
EN 260	0
EN 280	0
EN 300	-14393
EN 320	0
EN 340	0
EN 360	0
G-P:G-RIB:GLN-S2
EN 20 0
EN 40 -3262
EN 60 -4046
EN 80 -1825
EN 100	-3695
EN 120	-2080
EN 140	-3808
EN 160	-3673
EN 180	-5162
EN 200	-6021
EN 220	-3969
EN 240	-1910
EN 260	-1837
EN 280	-4241
EN 300	-3236
EN 320	-1959
EN 340	-3312
EN 360	-6385

H2U-RIB:H2U-MY:ASN-CA
EN 20 0
EN 40 0
EN 60 0
EN 80 0
EN 100 0
EN 120 0
EN 140 -8236
EN 160 0
EN 180 0
EN 200 0
EN 220 0
EN 240 0
EN 260 0
EN 280 -8688
EN 300 0
EN 320 0
EN 340 0
EN 360 0
G-P:G-RIB:GLU-S1
EN 20 0
EN 40 0
EN 60 3129
EN 80 -374
EN 100 157
EN 120 -1376
EN 140 245
EN 160 -880
EN 180 -1230
EN 200 -3774
EN 220 879
EN 240 1405
EN 260 1321
EN 280 -1811
EN 300 41
EN 320 -787
EN 340 -2044
EN 360 -2636
H2U-RIB:H2U-MY:PHE-S2
EN 20 0
EN 40 0
EN 60 0
EN 80 0
EN 100 -11671
EN 120 0
EN 140 0
EN 160 0
EN 180 0
EN 200 0
EN 220 0
EN 240 0
EN 260 0
EN 280 0
EN 300 0
EN 320 0
EN 340 0
EN 360 0
5BU-RIB:5BU-P:ARG-CA

EN 20 0
EN 40 0
EN 60 0
EN 80 0
EN 100 0
EN 120 -10576
EN 140 -11410
EN 160 0
EN 180 0
EN 200 0
EN 220 0
EN 240 0
EN 260 0
EN 280 0
EN 300 0
EN 320 0
EN 340 0
EN 360 0
C-RIB:C-P:PHE-S2
EN 20 0
EN 40 0
EN 60 -6166
EN 80 -2317
EN 100 -1752
EN 120 -3410
EN 140 -2073
EN 160 -3105
EN 180 0
EN 200 0
EN 220 -5192
EN 240 -5938
EN 260 -3619
EN 280 -2815
EN 300 -3420
EN 320 -4482
EN 340 -2803
EN 360 0
U34-RIB:U34-P:TYR-S1
EN 20 0
EN 40 0
EN 60 0
EN 80 0
EN 100 0
EN 120 0
EN 140 0
EN 160 0
EN 180 0
EN 200 0
EN 220 0
EN 240 0
EN 260 0
EN 280 0
EN 300 0
EN 320 -14393
EN 340 0
EN 360 0
U-RIB:U-P:VAL-S1
EN 20 0

EN 40 -3706
EN 60 -2669
EN 80 -3031
EN 100 -2854
EN 120 -2219
EN 140 -2440
EN 160 -3260
EN 180 -2470
EN 200 -8606
EN 220 -7397
EN 240 -3882
EN 260 -3149
EN 280 -3691
EN 300 -3227
EN 320 -3770
EN 340 -1636
EN 360 0
C-P:C-RIB:ASN-S2
EN 20 -4836
EN 40 -3591
EN 60 -4904
EN 80 -4192
EN 100 -4259
EN 120 -3568
EN 140 -2622
EN 160 -3006
EN 180 -2059
EN 200 -4784
EN 220 -3398
EN 240 -4607
EN 260 -1848
EN 280 -3484
EN 300 -2365
EN 320 -2158
EN 340 -2535
EN 360 -3180
A-RIB:A-R5:ARG-S1
EN 20 -6798
EN 40 -5392
EN 60 -4158
EN 80 -5730
EN 100 -4457
EN 120 -4219
EN 140 -4234
EN 160 -2177
EN 180 -2954
EN 200 -6608
EN 220 -1920
EN 240 -4262
EN 260 -5316
EN 280 -3388
EN 300 -4470
EN 320 -4462
EN 340 0
EN 360 -2748
A-P:A-RIB:ILE-S1
EN 20 0
EN 40 0

EN 60 -1559
EN 80 -4587
EN 100 -3726
EN 120 -3985
EN 140 -3551
EN 160 -2735
EN 180 -4898
EN 200 0
EN 220 -5572
EN 240 0
EN 260 -3448
EN 280 -2206
EN 300 -179
EN 320 -3765
EN 340 -973
EN 360 -3271
A-P:A-RIB:CYS-S1
EN 20 0
EN 40 0
EN 60 -8236
EN 80 0
EN 100 -7455
EN 120 -7201
EN 140 0
EN 160 0
EN 180 -8859
EN 200 0
EN 220 0
EN 240 -6660
EN 260 0
EN 280 0
EN 300 -4419
EN 320 0
EN 340 -6166
EN 360 0
G-RIB:G-P:LEU-S2
EN 20 0
EN 40 -3444
EN 60 -5414
EN 80 -2917
EN 100 -3133
EN 120 -2740
EN 140 -2152
EN 160 -722
EN 180 -4746
EN 200 0
EN 220 -5705
EN 240 -4767
EN 260 -4514
EN 280 -3260
EN 300 -1793
EN 320 -2718
EN 340 -2517
EN 360 -1587
A-RIB:A-R6:HIS-S2
EN 20 0
EN 40 -5584
EN 60 -4097

EN 80 -428
EN 100 -4803
EN 120 -5539
EN 140 -4824
EN 160 -3606
EN 180 -6035
EN 200 0
EN 220 -6368
EN 240 -4125
EN 260 -6325
EN 280 -5798
EN 300 -3162
EN 320 -785
EN 340 -4481
EN 360 -4853
U34-RIB:U34-MY:TYR-S2
EN 20 0
EN 40 0
EN 60 0
EN 80 0
EN 100 0
EN 120 0
EN 140 0
EN 160 0
EN 180 0
EN 200 0
EN 220 0
EN 240 0
EN 260 0
EN 280 0
EN 300 -10760
EN 320 0
EN 340 0
EN 360 0
DA-RIB:DA-M5:SER-S1
EN 20 0
EN 40 0
EN 60 0
EN 80 0
EN 100 0
EN 120 0
EN 140 0
EN 160 0
EN 180 0
EN 200 0
EN 220 0
EN 240 0
EN 260 0
EN 280 -8038
EN 300 0
EN 320 0
EN 340 0
EN 360 -17115
C-RIB:C-Y:ASN-S2
EN 20 -6542
EN 40 -4344
EN 60 -3527
EN 80 -3464

EN 100 -4148
EN 120 -3361
EN 140 -1970
EN 160 -4377
EN 180 0
EN 200 -4548
EN 220 -4412
EN 240 -3078
EN 260 -2866
EN 280 -3696
EN 300 -4398
EN 320 -1721
EN 340 -3760
EN 360 0
A-RIB:A-R5:ASP-S2
EN 20 -2571
EN 40 -2070
EN 60 -3169
EN 80 -2039
EN 100 -1358
EN 120 -1106
EN 140 -2411
EN 160 286
EN 180 -2257
EN 200 0
EN 220 -3414
EN 240 -2362
EN 260 -228
EN 280 -1903
EN 300 -121
EN 320 -2821
EN 340 -1529
EN 360 -2177
FHU-RIB:FHU-MY:VAL-S1
EN 20 0
EN 40 0
EN 60 0
EN 80 0
EN 100 -11410
EN 120 0
EN 140 0
EN 160 0
EN 180 -2722
EN 200 0
EN 220 0
EN 240 0
EN 260 0
EN 280 0
EN 300 0
EN 320 0
EN 340 -14393
EN 360 0
IU-RIB:IU-MY:THR-S1
EN 20 0
EN 40 0
EN 60 0
EN 80 0
EN 100 0

EN 120 -11764
EN 140 0
EN 160 0
EN 180 0
EN 200 0
EN 220 0
EN 240 0
EN 260 0
EN 280 0
EN 300 0
EN 320 0
EN 340 -9755
EN 360 0
C-RIB:C-Y:PHE-S2
EN 20 -8271
EN 40 0
EN 60 -4466
EN 80 -4116
EN 100 -2406
EN 120 -5253
EN 140 -3171
EN 160 0
EN 180 0
EN 200 0
EN 220 -3878
EN 240 -5952
EN 260 -5052
EN 280 -5176
EN 300 -4283
EN 320 0
EN 340 0
EN 360 0
C31-P:C31-RIB:GLU-CA
EN 20 0
EN 40 0
EN 60 0
EN 80 0
EN 100 0
EN 120 0
EN 140 0
EN 160 0
EN 180 0
EN 200 0
EN 220 0
EN 240 0
EN 260 -7231
EN 280 0
EN 300 0
EN 320 0
EN 340 0
EN 360 0
G-RIB:G-R5:HIS-S1
EN 20 -6970
EN 40 -6909
EN 60 -5426
EN 80 -4979
EN 100 -5575
EN 120 -4908

EN 140 -3322
EN 160 0
EN 180 0
EN 200 0
EN 220 -3960
EN 240 -3539
EN 260 -5540
EN 280 -2470
EN 300 -4807
EN 320 -5074
EN 340 0
EN 360 0
A-RIB:A-R6:MET-CA
EN 20 0
EN 40 0
EN 60 -5858
EN 80 -4276
EN 100 -6101
EN 120 -4737
EN 140 -5342
EN 160 0
EN 180 -6159
EN 200 0
EN 220 0
EN 240 -4079
EN 260 -2792
EN 280 -2538
EN 300 -5603
EN 320 -5155
EN 340 -3760
EN 360 -5911
C-RIB:C-P:MET-CA
EN 20 0
EN 40 -6385
EN 60 -6320
EN 80 -3041
EN 100 -3192
EN 120 -3561
EN 140 -2483
EN 160 0
EN 180 0
EN 200 0
EN 220 -6660
EN 240 -5688
EN 260 -4084
EN 280 -488
EN 300 -3676
EN 320 -4703
EN 340 -1712
EN 360 0
QUO-RIB:QUO-M6:PHE-CA
EN 20 0
EN 40 0
EN 60 0
EN 80 0
EN 100 0
EN 120 0
EN 140 0

EN 160 0
EN 180 0
EN 200 0
EN 220 0
EN 240 0
EN 260 0
EN 280 -3434
EN 300 0
EN 320 -3434
EN 340 0
EN 360 0
FHU-RIB:FHU-MY:ASP-S1
EN 20 0
EN 40 0
EN 60 0
EN 80 0
EN 100 -9953
EN 120 0
EN 140 -11174
EN 160 0
EN 180 0
EN 200 0
EN 220 0
EN 240 0
EN 260 0
EN 280 -8606
EN 300 0
EN 320 -10405
EN 340 0
EN 360 0
A-RIB:A-R5:SER-CA
EN 20 -4419
EN 40 -4499
EN 60 -4621
EN 80 -3460
EN 100 686
EN 120 -3182
EN 140 -5123
EN 160 -4224
EN 180 -7778
EN 200 0
EN 220 -3821
EN 240 -4199
EN 260 -1054
EN 280 -4279
EN 300 -2570
EN 320 -3818
EN 340 -4263
EN 360 -6057
A-P:A-RIB:ASP-S2
EN 20 0
EN 40 0
EN 60 289
EN 80 -2517
EN 100 -1099
EN 120 -1738
EN 140 2616
EN 160 471

EN 180 -2682
EN 200 0
EN 220 -2463
EN 240 -1272
EN 260 -2331
EN 280 -2841
EN 300 -2887
EN 320 -3060
EN 340 -2157
EN 360 0
C-RIB:C-Y:TYR-S1
EN 20 0
EN 40 0
EN 60 -5267
EN 80 -6232
EN 100 -4512
EN 120 -6260
EN 140 -3312
EN 160 0
EN 180 0
EN 200 0
EN 220 0
EN 240 -4839
EN 260 -6204
EN 280 -5610
EN 300 -6808
EN 320 -4664
EN 340 -4235
EN 360 0
C-RIB:C-P:ASN-CA
EN 20 0
EN 40 -3609
EN 60 -3729
EN 80 -3697
EN 100 -3835
EN 120 -3868
EN 140 -1909
EN 160 -2718
EN 180 -4385
EN 200 0
EN 220 -6863
EN 240 -4461
EN 260 -4801
EN 280 -4455
EN 300 -4507
EN 320 -3802
EN 340 -3855
EN 360 -5300
QUO-RIB:QUO-P:LEU-S2
EN 20 0
EN 40 0
EN 60 0
EN 80 -18832
EN 100 0
EN 120 0
EN 140 0
EN 160 0
EN 180 0

EN 200 0
EN 220 0
EN 240 0
EN 260 0
EN 280 0
EN 300 0
EN 320 0
EN 340 0
EN 360 0
DA-RIB:DA-M6:ASN-S1
EN 20 0
EN 40 0
EN 60 0
EN 80 0
EN 100 0
EN 120 0
EN 140 -6929
EN 160 0
EN 180 0
EN 200 0
EN 220 0
EN 240 -9571
EN 260 0
EN 280 0
EN 300 0
EN 320 0
EN 340 0
EN 360 0
FMU-RIB:FMU-MY:VAL-S1
EN 20 0
EN 40 0
EN 60 0
EN 80 0
EN 100 0
EN 120 0
EN 140 0
EN 160 0
EN 180 0
EN 200 0
EN 220 0
EN 240 -14393
EN 260 0
EN 280 0
EN 300 0
EN 320 0
EN 340 0
EN 360 -17115
G-RIB:G-P:VAL-CA
EN 20 0
EN 40 -3751
EN 60 -1865
EN 80 -1785
EN 100 -1793
EN 120 -1921
EN 140 -3177
EN 160 -2946
EN 180 -3866
EN 200 0

EN 220 -5619
EN 240 0
EN 260 -1785
EN 280 -173
EN 300 -1167
EN 320 -2288
EN 340 -2770
EN 360 0
U-P:U-RIB:HIS-S1
EN 20 0
EN 40 0
EN 60 -4728
EN 80 -6215
EN 100 -5517
EN 120 -3324
EN 140 -5055
EN 160 -4459
EN 180 -5966
EN 200 0
EN 220 0
EN 240 0
EN 260 -5024
EN 280 -4082
EN 300 -722
EN 320 -1224
EN 340 -6131
EN 360 0
G-RIB:G-P:TRP-CA
EN 20 0
EN 40 -8949
EN 60 -6151
EN 80 -4563
EN 100 -4409
EN 120 -1353
EN 140 0
EN 160 -5373
EN 180 0
EN 200 0
EN 220 0
EN 240 -4127
EN 260 -2443
EN 280 -1687
EN 300 -4194
EN 320 -5117
EN 340 0
EN 360 -7186
H2U-P:H2U-RIB:LEU-S2
EN 20 0
EN 40 0
EN 60 0
EN 80 0
EN 100 0
EN 120 0
EN 140 0
EN 160 0
EN 180 0
EN 200 0
EN 220 0

EN 240 -11174
EN 260 0
EN 280 0
EN 300 0
EN 320 0
EN 340 0
EN 360 0
A-RIB:A-P:GLU-S2
EN 20 -3663
EN 40 -2377
EN 60 -2673
EN 80 249
EN 100 1365
EN 120 -315
EN 140 -1728
EN 160 3049
EN 180 -1086
EN 200 0
EN 220 -3742
EN 240 -1072
EN 260 -799
EN 280 202
EN 300 -2094
EN 320 -1052
EN 340 -937
EN 360 674
A-RIB:A-R6:GLN-CA
EN 20 0
EN 40 0
EN 60 -4223
EN 80 -1022
EN 100 -5950
EN 120 -2271
EN 140 -4520
EN 160 -3610
EN 180 -5986
EN 200 -14393
EN 220 0
EN 240 -2334
EN 260 -4287
EN 280 0
EN 300 -3019
EN 320 -4915
EN 340 -3253
EN 360 0
C-P:C-RIB:LEU-S2
EN 20 0
EN 40 -2073
EN 60 -3472
EN 80 -3659
EN 100 -3123
EN 120 -2570
EN 140 -4318
EN 160 -3698
EN 180 -2352
EN 200 -5806
EN 220 0
EN 240 -3376

EN 260 -3418
EN 280 -4527
EN 300 -3809
EN 320 -4066
EN 340 -3285
EN 360 -3837
A-RIB:A-R6:TRP-S2
EN 20 0
EN 40 0
EN 60 -5980
EN 80 -3328
EN 100 -6014
EN 120 -3325
EN 140 0
EN 160 0
EN 180 0
EN 200 0
EN 220 -8038
EN 240 -7422
EN 260 -3262
EN 280 0
EN 300 -7029
EN 320 -3559
EN 340 0
EN 360 -8772
U31-RIB:U31-MY:ASP-S1
EN 20 0
EN 40 0
EN 60 -8452
EN 80 -6050
EN 100 -6772
EN 120 -6227
EN 140 0
EN 160 0
EN 180 0
EN 200 0
EN 220 0
EN 240 0
EN 260 0
EN 280 0
EN 300 0
EN 320 0
EN 340 0
EN 360 0
C-RIB:C-P:GLN-S2
EN 20 -6258
EN 40 -5007
EN 60 -5009
EN 80 -3498
EN 100 -2339
EN 120 -3230
EN 140 -1731
EN 160 -3530
EN 180 -3145
EN 200 0
EN 220 -4952
EN 240 -4653
EN 260 -4147

EN 280 -4605
EN 300 -3889
EN 320 -3427
EN 340 -2495
EN 360 -5363
QUO-RIB:QUO-M5:ASP-S1
EN 20 0
EN 40 0
EN 60 0
EN 80 0
EN 100 0
EN 120 -17115
EN 140 0
EN 160 0
EN 180 0
EN 200 0
EN 220 0
EN 240 0
EN 260 0
EN 280 0
EN 300 0
EN 320 -3434
EN 340 0
EN 360 0
U-RIB:U-P:PRO-S1
EN 20 0
EN 40 -2594
EN 60 -3019
EN 80 -3173
EN 100 -3058
EN 120 -3751
EN 140 -3745
EN 160 -3569
EN 180 -6697
EN 200 0
EN 220 -4556
EN 240 -4413
EN 260 -2711
EN 280 -3436
EN 300 -2697
EN 320 -2456
EN 340 -1403
EN 360 -3820
U-P:U-RIB:PRO-CA
EN 20 -8452
EN 40 -6127
EN 60 -2403
EN 80 -2388
EN 100 -3240
EN 120 -4122
EN 140 363
EN 160 -4646
EN 180 -4012
EN 200 0
EN 220 0
EN 240 -4056
EN 260 -5059
EN 280 -885

EN 300 -4709
EN 320 -2773
EN 340 0
EN 360 -4050
GTP-RIB:GTP-M6:SER-CA
EN 20 0
EN 40 0
EN 60 0
EN 80 0
EN 100 0
EN 120 0
EN 140 0
EN 160 0
EN 180 0
EN 200 0
EN 220 0
EN 240 0
EN 260 -8038
EN 280 0
EN 300 0
EN 320 0
EN 340 0
EN 360 0
U34-RIB:U34-P:GLU-S1
EN 20 0
EN 40 0
EN 60 0
EN 80 0
EN 100 0
EN 120 0
EN 140 0
EN 160 0
EN 180 0
EN 200 0
EN 220 0
EN 240 0
EN 260 0
EN 280 0
EN 300 0
EN 320 0
EN 340 0
EN 360 -15398
U-P:U-RIB:THR-CA
EN 20 0
EN 40 -4692
EN 60 -3902
EN 80 -4468
EN 100 -4328
EN 120 -3136
EN 140 -5141
EN 160 -3704
EN 180 0
EN 200 -8038
EN 220 -5984
EN 240 -2128
EN 260 -2743
EN 280 -1770
EN 300 -4290

EN 320 -3169
EN 340 -599
EN 360 -2209
U-P:U-RIB:ASP-S2
EN 20 0
EN 40 -534
EN 60 -123
EN 80 -1613
EN 100 -234
EN 120 -295
EN 140 -2376
EN 160 -2367
EN 180 -2348
EN 200 0
EN 220 -2065
EN 240 -1099
EN 260 -403
EN 280 -905
EN 300 -1646
EN 320 -1299
EN 340 -1304
EN 360 -2140
U-RIB:U-Y:LYS-S2
EN 20 -4891
EN 40 -1872
EN 60 -3903
EN 80 -3266
EN 100 -4588
EN 120 -1583
EN 140 -1241
EN 160 -4146
EN 180 -5083
EN 200 -4728
EN 220 -3152
EN 240 -4826
EN 260 -3753
EN 280 -3433
EN 300 -3353
EN 320 -2724
EN 340 -4767
EN 360 -4550
G-RIB:G-R6:ALA-S1
EN 20 -3881
EN 40 -183
EN 60 -458
EN 80 -2802
EN 100 730
EN 120 -3029
EN 140 -2881
EN 160 -1308
EN 180 -2139
EN 200 0
EN 220 -4635
EN 240 -3434
EN 260 -2442
EN 280 -2317
EN 300 -689
EN 320 -2540

EN 340 432
EN 360 0
DA-RIB:DA-M5:ASN-CA
EN 20 0
EN 40 0
EN 60 0
EN 80 0
EN 100 0
EN 120 0
EN 140 0
EN 160 0
EN 180 0
EN 200 0
EN 220 0
EN 240 -9953
EN 260 0
EN 280 0
EN 300 0
EN 320 0
EN 340 0
EN 360 0
IU-RIB:IU-MY:HIS-CA
EN 20 0
EN 40 -13680
EN 60 0
EN 80 0
EN 100 0
EN 120 0
EN 140 0
EN 160 0
EN 180 0
EN 200 0
EN 220 -13127
EN 240 0
EN 260 0
EN 280 0
EN 300 0
EN 320 0
EN 340 0
EN 360 0
A-P:A-RIB:TYR-S2
EN 20 0
EN 40 -7472
EN 60 -4318
EN 80 -5970
EN 100 -4723
EN 120 -5288
EN 140 -3870
EN 160 -3561
EN 180 -3630
EN 200 0
EN 220 -5074
EN 240 -4088
EN 260 -6293
EN 280 -3605
EN 300 -5395
EN 320 -4513
EN 340 0

EN 360 0
U-RIB:U-P:GLN-CA
EN 20 0
EN 40 -7472
EN 60 -5590
EN 80 -3706
EN 100 -2974
EN 120 -2660
EN 140 -1232
EN 160 -4066
EN 180 -4810
EN 200 0
EN 220 0
EN 240 -5048
EN 260 0
EN 280 -3857
EN 300 -3284
EN 320 -5664
EN 340 -2555
EN 360 -5055
A-RIB:A-P:VAL-CA
EN 20 -12294
EN 40 0
EN 60 -4591
EN 80 -3932
EN 100 -3330
EN 120 -3872
EN 140 -1381
EN 160 -3110
EN 180 -2073
EN 200 0
EN 220 0
EN 240 -3257
EN 260 -2274
EN 280 -4526
EN 300 -2743
EN 320 -2556
EN 340 -2823
EN 360 -4790
5BU-RIB:5BU-P:PRO-CA
EN 20 0
EN 40 0
EN 60 0
EN 80 0
EN 100 0
EN 120 0
EN 140 0
EN 160 0
EN 180 0
EN 200 0
EN 220 0
EN 240 0
EN 260 0
EN 280 0
EN 300 -10245
EN 320 0
EN 340 0
EN 360 0

G-RIB:G-R6:LYS-S1
EN 20 -6401
EN 40 -2527
EN 60 -3512
EN 80 -1990
EN 100 -3057
EN 120 -2686
EN 140 -1694
EN 160 -3025
EN 180 0
EN 200 0
EN 220 -800
EN 240 -2690
EN 260 -3512
EN 280 -3815
EN 300 -3664
EN 320 -3506
EN 340 -1840
EN 360 -2013
C-P:C-RIB:HIS-S1
EN 20 0
EN 40 -5243
EN 60 -4631
EN 80 -1978
EN 100 -4633
EN 120 -2702
EN 140 -263
EN 160 -5274
EN 180 -6227
EN 200 -8452
EN 220 0
EN 240 -5320
EN 260 -3715
EN 280 -4556
EN 300 -5702
EN 320 -4919
EN 340 -2387
EN 360 -5232
G-RIB:G-P:MET-S2
EN 20 -7422
EN 40 0
EN 60 -4216
EN 80 -4399
EN 100 -1670
EN 120 51
EN 140 -225
EN 160 -2815
EN 180 0
EN 200 0
EN 220 -6035
EN 240 -3751
EN 260 -2257
EN 280 39
EN 300 -2854
EN 320 -2152
EN 340 0
EN 360 0
A-P:A-RIB:LEU-S2

EN 20 0
EN 40 -3347
EN 60 -4304
EN 80 -1441
EN 100 -2838
EN 120 -2813
EN 140 -3125
EN 160 -4725
EN 180 -2983
EN 200 0
EN 220 0
EN 240 -948
EN 260 -2584
EN 280 -5270
EN 300 -2032
EN 320 -4637
EN 340 -4821
EN 360 -5366
H2U-P:H2U-RIB:ARG-S1
EN 20 0
EN 40 0
EN 60 0
EN 80 0
EN 100 0
EN 120 0
EN 140 0
EN 160 0
EN 180 0
EN 200 0
EN 220 0
EN 240 0
EN 260 -9571
EN 280 0
EN 300 0
EN 320 0
EN 340 0
EN 360 0
FHU-P:FHU-RIB:THR-S1
EN 20 0
EN 40 0
EN 60 0
EN 80 -9953
EN 100 0
EN 120 0
EN 140 0
EN 160 0
EN 180 0
EN 200 0
EN 220 0
EN 240 0
EN 260 -10760
EN 280 -10405
EN 300 0
EN 320 0
EN 340 0
EN 360 0
QUO-RIB:QUO-M5:PHE-CA
EN 20 0

EN 40 0
EN 60 0
EN 80 0
EN 100 0
EN 120 0
EN 140 0
EN 160 0
EN 180 0
EN 200 0
EN 220 0
EN 240 0
EN 260 0
EN 280 0
EN 300 -3434
EN 320 0
EN 340 0
EN 360 0
QUO-RIB:QUO-M6:ASP-S2
EN 20 0
EN 40 0
EN 60 0
EN 80 0
EN 100 0
EN 120 -3434
EN 140 0
EN 160 0
EN 180 0
EN 200 0
EN 220 0
EN 240 0
EN 260 0
EN 280 0
EN 300 -17115
EN 320 0
EN 340 0
EN 360 0
A-RIB:A-R6:ILE-S1
EN 20 -9953
EN 40 -6155
EN 60 -4137
EN 80 -2936
EN 100 0
EN 120 -861
EN 140 -3894
EN 160 0
EN 180 -4262
EN 200 0
EN 220 0
EN 240 -2624
EN 260 -3907
EN 280 -554
EN 300 -4691
EN 320 -4514
EN 340 -5185
EN 360 0
A-RIB:A-R6:SER-S1
EN 20 0
EN 40 -4254

EN 60 -3955
EN 80 -1574
EN 100 -3041
EN 120 -348
EN 140 -4722
EN 160 -2849
EN 180 -4139
EN 200 -5705
EN 220 -4885
EN 240 -4024
EN 260 -2143
EN 280 -3973
EN 300 -2138
EN 320 -2526
EN 340 -4778
EN 360 -2481
C-RIB:C-P:SER-S1
EN 20 0
EN 40 -5319
EN 60 -1405
EN 80 -4396
EN 100 -2209
EN 120 -3707
EN 140 -3495
EN 160 -3210
EN 180 -4124
EN 200 0
EN 220 -4512
EN 240 -3702
EN 260 -4484
EN 280 -4628
EN 300 -3494
EN 320 -3085
EN 340 -5305
EN 360 -5333
A-RIB:A-R5:PRO-CA
EN 20 -5093
EN 40 -3866
EN 60 -3912
EN 80 -3930
EN 100 -3645
EN 120 -4436
EN 140 -5156
EN 160 0
EN 180 0
EN 200 -5358
EN 220 -4617
EN 240 -3605
EN 260 -2614
EN 280 -4488
EN 300 -5562
EN 320 -2892
EN 340 -4998
EN 360 0
U-RIB:U-P:CYS-CA
EN 20 0
EN 40 0
EN 60 0

EN 80 -5832
EN 100 -4290
EN 120 -5596
EN 140 0
EN 160 -5358
EN 180 0
EN 200 0
EN 220 0
EN 240 0
EN 260 0
EN 280 0
EN 300 0
EN 320 0
EN 340 0
EN 360 0
G-RIB:G-R5:PHE-S2
EN 20 0
EN 40 -5569
EN 60 -2615
EN 80 -3834
EN 100 -3752
EN 120 0
EN 140 -4240
EN 160 0
EN 180 0
EN 200 0
EN 220 -7355
EN 240 -4074
EN 260 -6678
EN 280 -2461
EN 300 -2825
EN 320 0
EN 340 0
EN 360 0
U31-RIB:U31-MY:MET-S1
EN 20 0
EN 40 0
EN 60 0
EN 80 -9456
EN 100 0
EN 120 0
EN 140 0
EN 160 0
EN 180 0
EN 200 0
EN 220 0
EN 240 0
EN 260 0
EN 280 0
EN 300 0
EN 320 0
EN 340 0
EN 360 0
A-RIB:A-R5:HIS-CA
EN 20 -8306
EN 40 -5755
EN 60 -5310
EN 80 -6602

EN 100 -5483
EN 120 -6605
EN 140 0
EN 160 -4003
EN 180 0
EN 200 0
EN 220 -7825
EN 240 -6560
EN 260 -5599
EN 280 -3634
EN 300 -4006
EN 320 -6828
EN 340 -4235
EN 360 0
C-RIB:C-Y:ARG-CA
EN 20 0
EN 40 -4978
EN 60 -3573
EN 80 -4792
EN 100 -5801
EN 120 -3774
EN 140 -4479
EN 160 -1023
EN 180 -3356
EN 200 -6970
EN 220 -899
EN 240 -4392
EN 260 -4693
EN 280 -4976
EN 300 -2793
EN 320 -4871
EN 340 -3730
EN 360 0
C-RIB:C-P:PRO-S1
EN 20 0
EN 40 -6280
EN 60 -3381
EN 80 -3574
EN 100 -2684
EN 120 -3588
EN 140 -1932
EN 160 -3188
EN 180 0
EN 200 0
EN 220 -3598
EN 240 -4345
EN 260 -2660
EN 280 -3871
EN 300 -2569
EN 320 -1396
EN 340 -4250
EN 360 -4603
IU-RIB:IU-P:SER-S1
EN 20 0
EN 40 0
EN 60 0
EN 80 0
EN 100 -11410

EN 120 0
EN 140 0
EN 160 0
EN 180 0
EN 200 0
EN 220 0
EN 240 0
EN 260 0
EN 280 -12294
EN 300 0
EN 320 0
EN 340 0
EN 360 0
FMU-P:FMU-RIB:PHE-S1
EN 20 0
EN 40 0
EN 60 0
EN 80 0
EN 100 0
EN 120 0
EN 140 0
EN 160 0
EN 180 0
EN 200 0
EN 220 0
EN 240 -2722
EN 260 0
EN 280 0
EN 300 0
EN 320 0
EN 340 -2722
EN 360 0
A-P:A-RIB:CYS-CA
EN 20 0
EN 40 0
EN 60 0
EN 80 -7954
EN 100 -4180
EN 120 -7112
EN 140 -4717
EN 160 0
EN 180 0
EN 200 0
EN 220 0
EN 240 -6258
EN 260 0
EN 280 -3950
EN 300 0
EN 320 -6064
EN 340 0
EN 360 0
U-RIB:U-Y:ARG-S2
EN 20 -4979
EN 40 -4738
EN 60 -4661
EN 80 -4310
EN 100 -5239
EN 120 -4128

EN 140 -3744
EN 160 -3100
EN 180 -6408
EN 200 -7681
EN 220 -1221
EN 240 -3770
EN 260 -2289
EN 280 -3834
EN 300 -1098
EN 320 -4029
EN 340 -4107
EN 360 -1870
A-RIB:A-R6:TYR-S1
EN 20 0
EN 40 0
EN 60 -8205
EN 80 -7293
EN 100 -2201
EN 120 -5434
EN 140 -2241
EN 160 -6139
EN 180 -5774
EN 200 0
EN 220 0
EN 240 -8486
EN 260 -5362
EN 280 -1966
EN 300 -4760
EN 320 -5796
EN 340 -2704
EN 360 -6007
U-P:U-RIB:PHE-S1
EN 20 0
EN 40 0
EN 60 -4853
EN 80 -1972
EN 100 -4889
EN 120 -6480
EN 140 -6672
EN 160 -3271
EN 180 0
EN 200 0
EN 220 0
EN 240 -3598
EN 260 -2227
EN 280 -3351
EN 300 -6171
EN 320 -4717
EN 340 -4708
EN 360 -5424
C-RIB:C-P:VAL-CA
EN 20 0
EN 40 -4101
EN 60 -3751
EN 80 -5186
EN 100 -4216
EN 120 -3874
EN 140 -3191

EN 160 -836
EN 180 -3309
EN 200 0
EN 220 -4494
EN 240 -6059
EN 260 -4053
EN 280 -3594
EN 300 -2877
EN 320 -3825
EN 340 -4479
EN 360 -1610
U34-RIB:U34-P:HIS-S2
EN 20 0
EN 40 0
EN 60 0
EN 80 0
EN 100 0
EN 120 0
EN 140 0
EN 160 -11671
EN 180 0
EN 200 0
EN 220 0
EN 240 0
EN 260 0
EN 280 -11671
EN 300 0
EN 320 0
EN 340 0
EN 360 0
IU-RIB:IU-MY:ARG-CA
EN 20 -16110
EN 40 0
EN 60 0
EN 80 -12817
EN 100 0
EN 120 0
EN 140 0
EN 160 0
EN 180 0
EN 200 0
EN 220 0
EN 240 0
EN 260 0
EN 280 0
EN 300 0
EN 320 0
EN 340 0
EN 360 0
A-P:A-RIB:LEU-S1
EN 20 0
EN 40 -7485
EN 60 -3101
EN 80 -1524
EN 100 -4708
EN 120 -2213
EN 140 -3829
EN 160 -5080

EN 180 -4587
EN 200 0
EN 220 -3820
EN 240 0
EN 260 -3380
EN 280 -3934
EN 300 -2127
EN 320 -4377
EN 340 -4461
EN 360 -4708
U34-RIB:U34-P:ASN-CA
EN 20 0
EN 40 0
EN 60 0
EN 80 -13127
EN 100 -11174
EN 120 0
EN 140 0
EN 160 0
EN 180 0
EN 200 0
EN 220 0
EN 240 0
EN 260 -11410
EN 280 0
EN 300 0
EN 320 0
EN 340 0
EN 360 0
IU-RIB:IU-P:ARG-CA
EN 20 0
EN 40 0
EN 60 -15398
EN 80 0
EN 100 0
EN 120 0
EN 140 0
EN 160 0
EN 180 0
EN 200 0
EN 220 0
EN 240 0
EN 260 0
EN 280 0
EN 300 0
EN 320 -10405
EN 340 0
EN 360 0
C-RIB:C-P:HIS-S1
EN 20 0
EN 40 -7938
EN 60 -3619
EN 80 -2972
EN 100 -5586
EN 120 -2211
EN 140 -2461
EN 160 -4134
EN 180 0

EN 200 0
EN 220 0
EN 240 -3380
EN 260 -4205
EN 280 -3533
EN 300 -1280
EN 320 -5031
EN 340 -3598
EN 360 -3225
U-P:U-RIB:VAL-CA
EN 20 0
EN 40 0
EN 60 -2538
EN 80 -1463
EN 100 -3760
EN 120 -3253
EN 140 -1294
EN 160 0
EN 180 -4325
EN 200 0
EN 220 0
EN 240 0
EN 260 -3814
EN 280 -815
EN 300 -4108
EN 320 -1115
EN 340 -228
EN 360 -2456
FMU-RIB:FMU-MY:VAL-CA
EN 20 0
EN 40 0
EN 60 0
EN 80 0
EN 100 0
EN 120 0
EN 140 0
EN 160 0
EN 180 -15398
EN 200 0
EN 220 0
EN 240 0
EN 260 -11963
EN 280 0
EN 300 0
EN 320 0
EN 340 0
EN 360 0
C-RIB:C-P:ASP-S2
EN 20 0
EN 40 -4558
EN 60 -1061
EN 80 -2886
EN 100 -2937
EN 120 -2286
EN 140 -2708
EN 160 -3601
EN 180 238
EN 200 0

EN 220 -4823
EN 240 -2944
EN 260 -2247
EN 280 -1305
EN 300 -2125
EN 320 -71
EN 340 -752
EN 360 -1394
C-P:C-RIB:GLU-S2
EN 20 0
EN 40 0
EN 60 -734
EN 80 -772
EN 100 50
EN 120 -1016
EN 140 800
EN 160 -2665
EN 180 -601
EN 200 0
EN 220 0
EN 240 -1104
EN 260 -1729
EN 280 -328
EN 300 1610
EN 320 233
EN 340 -192
EN 360 -1401
G-P:G-RIB:ASN-S1
EN 20 -7975
EN 40 -3123
EN 60 -5100
EN 80 -4368
EN 100 -3569
EN 120 -2794
EN 140 -2806
EN 160 -4412
EN 180 0
EN 200 -5514
EN 220 -3054
EN 240 -5137
EN 260 -2946
EN 280 -4301
EN 300 -3571
EN 320 -2677
EN 340 -3268
EN 360 -2784
A-RIB:A-P:VAL-S1
EN 20 0
EN 40 -4667
EN 60 -5997
EN 80 -2118
EN 100 -3595
EN 120 -2575
EN 140 -2276
EN 160 -4154
EN 180 -2197
EN 200 0
EN 220 -3444

EN 240 -4408
EN 260 -2175
EN 280 -4723
EN 300 -2988
EN 320 -3306
EN 340 -1371
EN 360 -1983
U-P:U-RIB:MET-S1
EN 20 0
EN 40 0
EN 60 -3207
EN 80 -2566
EN 100 -6747
EN 120 -5239
EN 140 -2846
EN 160 -3577
EN 180 0
EN 200 0
EN 220 0
EN 240 -4952
EN 260 -3969
EN 280 -3831
EN 300 -2179
EN 320 -4347
EN 340 0
EN 360 -5938
C31-RIB:C31-MY:SER-CA
EN 20 0
EN 40 0
EN 60 0
EN 80 0
EN 100 0
EN 120 0
EN 140 0
EN 160 0
EN 180 0
EN 200 0
EN 220 0
EN 240 -8949
EN 260 0
EN 280 0
EN 300 0
EN 320 0
EN 340 0
EN 360 0
C31-RIB:C31-P:MET-CA
EN 20 0
EN 40 0
EN 60 0
EN 80 0
EN 100 0
EN 120 0
EN 140 0
EN 160 0
EN 180 0
EN 200 0
EN 220 0
EN 240 0

EN 260	0
EN 280	0
EN 300	0
EN 320	-14393
EN 340	0
EN 360	0
DA-RIB:DA-M5:ASN-S1
EN 20 0
EN 40 0
EN 60 0
EN 80 0
EN 100	0
EN 120	0
EN 140	0
EN 160	0
EN 180	0
EN 200	0
EN 220	0
EN 240	-9346
EN 260	0
EN 280	0
EN 300	0
EN 320	0
EN 340	0
EN 360	0
H2U-RIB:H2U-MY:GLU-CA
EN 20 0
EN 40 0
EN 60 0
EN 80 0
EN 100	0
EN 120	0
EN 140	0
EN 160	-9692
EN 180	0
EN 200	0
EN 220	0
EN 240	0
EN 260	0
EN 280	0
EN 300	0
EN 320	0
EN 340	0
EN 360	0
DA-RIB:DA-M6:LEU-S2
EN 20 0
EN 40 0
EN 60 0
EN 80 0
EN 100	-6385
EN 120	0
EN 140	0
EN 160	0
EN 180	0
EN 200	0
EN 220	0
EN 240	0
EN 260	0

EN 280 0
EN 300 -8688
EN 320 0
EN 340 0
EN 360 0
G-RIB:G-R5:TRP-CA
EN 20 -9066
EN 40 -7081
EN 60 0
EN 80 -4322
EN 100 -7217
EN 120 -6025
EN 140 0
EN 160 0
EN 180 0
EN 200 0
EN 220 -9171
EN 240 0
EN 260 -3715
EN 280 -3622
EN 300 -7190
EN 320 0
EN 340 0
EN 360 0
C-RIB:C-Y:ASN-S1
EN 20 -7270
EN 40 -5120
EN 60 -4184
EN 80 -4732
EN 100 0
EN 120 -4226
EN 140 -2375
EN 160 -1417
EN 180 0
EN 200 -7108
EN 220 -547
EN 240 -4023
EN 260 -3198
EN 280 -3880
EN 300 -4015
EN 320 0
EN 340 -3224
EN 360 -3866
C-P:C-RIB:PHE-S2
EN 20 0
EN 40 0
EN 60 -4469
EN 80 -3542
EN 100 -3712
EN 120 -3745
EN 140 -2446
EN 160 -4494
EN 180 -3926
EN 200 0
EN 220 0
EN 240 0
EN 260 -2336
EN 280 -4784

EN 300 -455
EN 320 -3395
EN 340 -4439
EN 360 -4025
G-P:G-RIB:LEU-S1
EN 20 0
EN 40 -2371
EN 60 -4141
EN 80 -3340
EN 100 -4043
EN 120 -3389
EN 140 -1811
EN 160 -4154
EN 180 -2587
EN 200 0
EN 220 -6727
EN 240 -1970
EN 260 -3075
EN 280 -4590
EN 300 -3164
EN 320 -3099
EN 340 -1951
EN 360 -4397
IU-RIB:IU-MY:VAL-S1
EN 20 0
EN 40 0
EN 60 0
EN 80 0
EN 100 -12123
EN 120 0
EN 140 0
EN 160 0
EN 180 0
EN 200 0
EN 220 0
EN 240 0
EN 260 0
EN 280 0
EN 300 0
EN 320 0
EN 340 0
EN 360 0
C-P:C-RIB:LYS-S1
EN 20 -7460
EN 40 -4562
EN 60 -5626
EN 80 -3112
EN 100 -2887
EN 120 -3831
EN 140 -2511
EN 160 -3365
EN 180 -2704
EN 200 -6050
EN 220 -5553
EN 240 -4257
EN 260 -2506
EN 280 -3841
EN 300 -3265

EN 320 -2556
EN 340 -3364
EN 360 -2022
U34-P:U34-RIB:ASN-S1
EN 20 0
EN 40 0
EN 60 -12675
EN 80 -8772
EN 100 0
EN 120 0
EN 140 0
EN 160 0
EN 180 0
EN 200 0
EN 220 0
EN 240 0
EN 260 0
EN 280 -8606
EN 300 0
EN 320 0
EN 340 0
EN 360 0
IU-RIB:IU-MY:ALA-S1
EN 20 0
EN 40 0
EN 60 0
EN 80 0
EN 100 -11174
EN 120 -11537
EN 140 0
EN 160 0
EN 180 0
EN 200 0
EN 220 0
EN 240 0
EN 260 0
EN 280 0
EN 300 0
EN 320 0
EN 340 0
EN 360 0
FHU-P:FHU-RIB:TYR-S1
EN 20 0
EN 40 0
EN 60 0
EN 80 0
EN 100 0
EN 120 0
EN 140 0
EN 160 0
EN 180 0
EN 200 0
EN 220 0
EN 240 0
EN 260 0
EN 280 0
EN 300 -13127
EN 320 -14393

EN 340	0
EN 360	0
QUO-RIB:QUO-M6:GLU-CA
EN 20 0
EN 40 0
EN 60 0
EN 80 0
EN 100	0
EN 120	0
EN 140	-3434
EN 160	0
EN 180	0
EN 200	0
EN 220	0
EN 240	0
EN 260	0
EN 280	0
EN 300	0
EN 320	0
EN 340	0
EN 360	0
U-P:U-RIB:LYS-S1
EN 20 0
EN 40 -4616
EN 60 -3503
EN 80 -3915
EN 100	-3657
EN 120	-4783
EN 140	-703
EN 160	-4370
EN 180	-4121
EN 200	-5295
EN 220	-5691
EN 240	-4992
EN 260	-4904
EN 280	-3587
EN 300	-2148
EN 320	-3677
EN 340	-3433
EN 360	-3353
FMU-RIB:FMU-MY:PHE-S2
EN 20 0
EN 40 0
EN 60 0
EN 80 0
EN 100	0
EN 120	0
EN 140	0
EN 160	0
EN 180	0
EN 200	0
EN 220	0
EN 240	0
EN 260	-17115
EN 280	0
EN 300	0
EN 320	0
EN 340	0

EN 360 0
H2U-RIB:H2U-MY:GLU-S1
EN 20 0
EN 40 0
EN 60 0
EN 80 0
EN 100 0
EN 120 0
EN 140 -8168
EN 160 0
EN 180 0
EN 200 0
EN 220 0
EN 240 0
EN 260 0
EN 280 0
EN 300 0
EN 320 0
EN 340 0
EN 360 0
C31-P:C31-RIB:ALA-S1
EN 20 0
EN 40 0
EN 60 0
EN 80 0
EN 100 0
EN 120 0
EN 140 -9456
EN 160 0
EN 180 0
EN 200 0
EN 220 0
EN 240 0
EN 260 0
EN 280 0
EN 300 -7796
EN 320 0
EN 340 0
EN 360 0
QUO-RIB:QUO-M6:PHE-S2
EN 20 0
EN 40 0
EN 60 0
EN 80 0
EN 100 0
EN 120 0
EN 140 0
EN 160 0
EN 180 0
EN 200 0
EN 220 0
EN 240 0
EN 260 -3434
EN 280 0
EN 300 -3434
EN 320 0
EN 340 0
EN 360 0

FHU-P:FHU-RIB:SER-S1
EN 20 0
EN 40 0
EN 60 0
EN 80 0
EN 100 -12675
EN 120 0
EN 140 -13127
EN 160 0
EN 180 0
EN 200 0
EN 220 0
EN 240 0
EN 260 0
EN 280 0
EN 300 0
EN 320 -11671
EN 340 0
EN 360 0
A-P:A-RIB:ASP-S1
EN 20 0
EN 40 0
EN 60 0
EN 80 -2831
EN 100 -2653
EN 120 -230
EN 140 -1614
EN 160 76
EN 180 0
EN 200 0
EN 220 0
EN 240 -1167
EN 260 -2383
EN 280 -1877
EN 300 -3628
EN 320 -2167
EN 340 -3257
EN 360 -203
C-P:C-RIB:SER-S1
EN 20 -5980
EN 40 -4324
EN 60 -4609
EN 80 -4311
EN 100 -3711
EN 120 -3628
EN 140 -4160
EN 160 -2487
EN 180 87
EN 200 -3987
EN 220 -5438
EN 240 -4629
EN 260 -2668
EN 280 -3141
EN 300 -3703
EN 320 -3874
EN 340 -3061
EN 360 -4288
C-RIB:C-P:ASP-CA

EN 20 -9631
EN 40 0
EN 60 -2940
EN 80 -2836
EN 100 -2100
EN 120 -2673
EN 140 -1841
EN 160 -3341
EN 180 0
EN 200 0
EN 220 -5106
EN 240 -4264
EN 260 -162
EN 280 -2374
EN 300 -2116
EN 320 -1104
EN 340 -1516
EN 360 0
C-RIB:C-P:TYR-S1
EN 20 0
EN 40 -9090
EN 60 -6857
EN 80 -6316
EN 100 -2117
EN 120 -4197
EN 140 -3259
EN 160 -1732
EN 180 -4290
EN 200 0
EN 220 0
EN 240 -3474
EN 260 -3299
EN 280 -4461
EN 300 -4692
EN 320 -2476
EN 340 -4728
EN 360 0
G-RIB:G-R5:ASN-CA
EN 20 0
EN 40 -5880
EN 60 -5086
EN 80 -2530
EN 100 -4182
EN 120 -3782
EN 140 -4908
EN 160 -3516
EN 180 -6070
EN 200 -5243
EN 220 -4786
EN 240 -4866
EN 260 -4876
EN 280 0
EN 300 -3574
EN 320 -2333
EN 340 -4450
EN 360 -3471
U31-P:U31-RIB:ASN-CA
EN 20 0

EN 40 0
EN 60 0
EN 80 0
EN 100 0
EN 120 0
EN 140 0
EN 160 0
EN 180 0
EN 200 0
EN 220 0
EN 240 -8688
EN 260 0
EN 280 0
EN 300 0
EN 320 0
EN 340 0
EN 360 0
U31-RIB:U31-MY:ASP-S2
EN 20 0
EN 40 0
EN 60 0
EN 80 -6320
EN 100 0
EN 120 -6258
EN 140 0
EN 160 0
EN 180 0
EN 200 0
EN 220 0
EN 240 -10405
EN 260 0
EN 280 -5884
EN 300 0
EN 320 0
EN 340 0
EN 360 0
C31-P:C31-RIB:GLU-S2
EN 20 0
EN 40 0
EN 60 -7523
EN 80 0
EN 100 0
EN 120 -6484
EN 140 0
EN 160 0
EN 180 0
EN 200 0
EN 220 0
EN 240 0
EN 260 0
EN 280 0
EN 300 0
EN 320 0
EN 340 0
EN 360 0
C31-P:C31-RIB:TYR-S1
EN 20 0
EN 40 0

EN 60 0
EN 80 0
EN 100 0
EN 120 0
EN 140 0
EN 160 0
EN 180 0
EN 200 0
EN 220 0
EN 240 -8688
EN 260 0
EN 280 0
EN 300 0
EN 320 0
EN 340 0
EN 360 0
C-RIB:C-Y:MET-S2
EN 20 0
EN 40 -4540
EN 60 -4605
EN 80 -5848
EN 100 -3798
EN 120 -4538
EN 140 0
EN 160 0
EN 180 0
EN 200 -8427
EN 220 -7677
EN 240 -1940
EN 260 -3292
EN 280 -5554
EN 300 -4297
EN 320 0
EN 340 0
EN 360 0
C31-RIB:C31-P:LEU-S2
EN 20 0
EN 40 -15398
EN 60 0
EN 80 0
EN 100 0
EN 120 0
EN 140 0
EN 160 0
EN 180 0
EN 200 0
EN 220 0
EN 240 0
EN 260 0
EN 280 0
EN 300 0
EN 320 0
EN 340 0
EN 360 0
A-RIB:A-R5:ILE-S1
EN 20 0
EN 40 -4473
EN 60 -4833

EN 80 -1389
EN 100 0
EN 120 -3609
EN 140 -2321
EN 160 0
EN 180 0
EN 200 -5993
EN 220 0
EN 240 -4889
EN 260 -4715
EN 280 0
EN 300 -4516
EN 320 -3799
EN 340 -5279
EN 360 0
G-P:G-RIB:ILE-CA
EN 20 0
EN 40 -3474
EN 60 0
EN 80 -3345
EN 100 -3756
EN 120 -4154
EN 140 -3890
EN 160 -1978
EN 180 0
EN 200 0
EN 220 -3444
EN 240 0
EN 260 -3039
EN 280 -2495
EN 300 -824
EN 320 -3760
EN 340 -670
EN 360 0
G-RIB:G-P:HIS-S1
EN 20 0
EN 40 -5980
EN 60 -4106
EN 80 -5219
EN 100 -3550
EN 120 -3247
EN 140 -3195
EN 160 -121
EN 180 -2301
EN 200 0
EN 220 -7266
EN 240 -1192
EN 260 -3673
EN 280 -2914
EN 300 -4649
EN 320 -1978
EN 340 -4050
EN 360 -2566
QUO-RIB:QUO-M5:LYS-S1
EN 20 0
EN 40 0
EN 60 0
EN 80 -17115

EN 100 0
EN 120 0
EN 140 0
EN 160 0
EN 180 0
EN 200 0
EN 220 0
EN 240 0
EN 260 0
EN 280 0
EN 300 0
EN 320 0
EN 340 0
EN 360 0
IU-RIB:IU-MY:LYS-S1
EN 20 0
EN 40 0
EN 60 -9382
EN 80 0
EN 100 -8489
EN 120 0
EN 140 0
EN 160 -12846
EN 180 0
EN 200 0
EN 220 0
EN 240 -9419
EN 260 0
EN 280 -8341
EN 300 0
EN 320 0
EN 340 0
EN 360 0
U-RIB:U-P:TRP-CA
EN 20 0
EN 40 0
EN 60 0
EN 80 -6759
EN 100 -4659
EN 120 -5705
EN 140 -5929
EN 160 -4221
EN 180 -8949
EN 200 0
EN 220 0
EN 240 0
EN 260 -4276
EN 280 -5814
EN 300 -6459
EN 320 0
EN 340 -4818
EN 360 0
IU-RIB:IU-MY:ARG-S1
EN 20 0
EN 40 0
EN 60 0
EN 80 0
EN 100 -11250

EN 120 0
EN 140 -11494
EN 160 0
EN 180 0
EN 200 0
EN 220 0
EN 240 0
EN 260 0
EN 280 0
EN 300 0
EN 320 0
EN 340 0
EN 360 0
FHU-P:FHU-RIB:LEU-S2
EN 20 0
EN 40 0
EN 60 0
EN 80 0
EN 100 -12294
EN 120 -11671
EN 140 0
EN 160 0
EN 180 0
EN 200 0
EN 220 0
EN 240 0
EN 260 0
EN 280 -10958
EN 300 -10095
EN 320 0
EN 340 0
EN 360 0
A-RIB:A-R5:GLU-S1
EN 20 0
EN 40 -4098
EN 60 -845
EN 80 -1620
EN 100 -2281
EN 120 -1533
EN 140 -3023
EN 160 396
EN 180 0
EN 200 -2919
EN 220 -3037
EN 240 93
EN 260 -425
EN 280 -567
EN 300 -2313
EN 320 -1549
EN 340 308
EN 360 -2221
IU-RIB:IU-P:ARG-S2
EN 20 0
EN 40 0
EN 60 0
EN 80 0
EN 100 0
EN 120 0

EN 140 0
EN 160 0
EN 180 0
EN 200 0
EN 220 0
EN 240 -13127
EN 260 0
EN 280 0
EN 300 0
EN 320 0
EN 340 0
EN 360 0
G-RIB:G-P:PRO-CA
EN 20 0
EN 40 -2580
EN 60 -4755
EN 80 -2970
EN 100 -2093
EN 120 -3590
EN 140 -2123
EN 160 -3408
EN 180 -5888
EN 200 0
EN 220 -3016
EN 240 -5661
EN 260 -4538
EN 280 -4076
EN 300 -3604
EN 320 -2601
EN 340 -2658
EN 360 -2680
H2U-RIB:H2U-P:PRO-CA
EN 20 0
EN 40 0
EN 60 0
EN 80 0
EN 100 -8102
EN 120 0
EN 140 0
EN 160 0
EN 180 0
EN 200 0
EN 220 0
EN 240 0
EN 260 0
EN 280 0
EN 300 0
EN 320 0
EN 340 0
EN 360 0
H2U-RIB:H2U-P:ASN-CA
EN 20 0
EN 40 0
EN 60 0
EN 80 0
EN 100 0
EN 120 -9953
EN 140 -11410

EN 160 0
EN 180 0
EN 200 0
EN 220 0
EN 240 0
EN 260 0
EN 280 0
EN 300 0
EN 320 0
EN 340 0
EN 360 0
C31-RIB:C31-P:ASP-S2
EN 20 0
EN 40 0
EN 60 0
EN 80 0
EN 100 0
EN 120 -7739
EN 140 0
EN 160 0
EN 180 0
EN 200 0
EN 220 0
EN 240 0
EN 260 0
EN 280 0
EN 300 0
EN 320 -9755
EN 340 0
EN 360 0
DA-RIB:DA-M5:ASN-S2
EN 20 0
EN 40 0
EN 60 0
EN 80 0
EN 100 0
EN 120 0
EN 140 0
EN 160 0
EN 180 0
EN 200 0
EN 220 0
EN 240 -8859
EN 260 0
EN 280 0
EN 300 0
EN 320 0
EN 340 0
EN 360 0
U-RIB:U-Y:ASN-S2
EN 20 -4088
EN 40 -3023
EN 60 -3965
EN 80 -4486
EN 100 -4684
EN 120 -5049
EN 140 -3073
EN 160 -2674

EN 180 0
EN 200 -6278
EN 220 -3847
EN 240 -5167
EN 260 -4528
EN 280 -3745
EN 300 -4042
EN 320 -3688
EN 340 -3464
EN 360 -3363
FHU-RIB:FHU-P:TYR-S1
EN 20 0
EN 40 0
EN 60 0
EN 80 0
EN 100 0
EN 120 0
EN 140 0
EN 160 0
EN 180 -11963
EN 200 0
EN 220 0
EN 240 0
EN 260 0
EN 280 0
EN 300 0
EN 320 0
EN 340 0
EN 360 0
C-RIB:C-P:SER-CA
EN 20 0
EN 40 -4790
EN 60 -4664
EN 80 -4303
EN 100 -3610
EN 120 -1676
EN 140 -2592
EN 160 -4813
EN 180 -4343
EN 200 0
EN 220 -6358
EN 240 -3177
EN 260 -4237
EN 280 -4815
EN 300 -2860
EN 320 -3017
EN 340 -4724
EN 360 -5499
H2U-RIB:H2U-MY:PRO-CA
EN 20 0
EN 40 0
EN 60 0
EN 80 0
EN 100 -8567
EN 120 0
EN 140 -7683
EN 160 0
EN 180 0

EN 200 0
EN 220 0
EN 240 0
EN 260 0
EN 280 0
EN 300 0
EN 320 0
EN 340 0
EN 360 0
A-RIB:A-R5:MET-CA
EN 20 0
EN 40 -4571
EN 60 -6206
EN 80 -5623
EN 100 -6877
EN 120 -3099
EN 140 -3869
EN 160 -5162
EN 180 0
EN 200 0
EN 220 -5003
EN 240 -5471
EN 260 -3371
EN 280 0
EN 300 -4959
EN 320 -5915
EN 340 -6393
EN 360 -7325
IU-RIB:IU-P:LEU-CA
EN 20 0
EN 40 0
EN 60 0
EN 80 0
EN 100 0
EN 120 0
EN 140 -10576
EN 160 0
EN 180 0
EN 200 0
EN 220 0
EN 240 0
EN 260 0
EN 280 0
EN 300 -9042
EN 320 0
EN 340 0
EN 360 0
QUO-RIB:QUO-M5:GLU-S1
EN 20 0
EN 40 0
EN 60 0
EN 80 0
EN 100 0
EN 120 0
EN 140 -3434
EN 160 0
EN 180 0
EN 200 0

EN 220 0
EN 240 0
EN 260 0
EN 280 0
EN 300 0
EN 320 0
EN 340 0
EN 360 0
FHU-RIB:FHU-P:TYR-S2
EN 20 0
EN 40 -15398
EN 60 0
EN 80 -10576
EN 100 0
EN 120 0
EN 140 0
EN 160 0
EN 180 0
EN 200 0
EN 220 -17115
EN 240 0
EN 260 -12675
EN 280 0
EN 300 0
EN 320 0
EN 340 0
EN 360 0
FMU-RIB:FMU-MY:CYS-S1
EN 20 0
EN 40 0
EN 60 0
EN 80 0
EN 100 -2722
EN 120 0
EN 140 0
EN 160 0
EN 180 0
EN 200 0
EN 220 0
EN 240 0
EN 260 0
EN 280 0
EN 300 0
EN 320 0
EN 340 0
EN 360 0
U-P:U-RIB:LEU-S2
EN 20 0
EN 40 0
EN 60 0
EN 80 -6536
EN 100 -2859
EN 120 -4907
EN 140 -3737
EN 160 -1427
EN 180 -3525
EN 200 0
EN 220 -3641

EN 240 -1063
EN 260 -1836
EN 280 -3274
EN 300 -2660
EN 320 -3695
EN 340 -3028
EN 360 -3820
U34-RIB:U34-P:PRO-S1
EN 20 0
EN 40 0
EN 60 0
EN 80 0
EN 100 0
EN 120 0
EN 140 0
EN 160 0
EN 180 0
EN 200 0
EN 220 0
EN 240 0
EN 260 0
EN 280 -14393
EN 300 0
EN 320 0
EN 340 0
EN 360 0
C-RIB:C-P:TYR-CA
EN 20 0
EN 40 0
EN 60 -7201
EN 80 -5564
EN 100 -2039
EN 120 -4439
EN 140 -2170
EN 160 -4975
EN 180 0
EN 200 0
EN 220 0
EN 240 0
EN 260 -5376
EN 280 -3047
EN 300 -3517
EN 320 -2182
EN 340 -1634
EN 360 -5819
A-RIB:A-R5:TYR-S1
EN 20 0
EN 40 -4925
EN 60 -8111
EN 80 -7355
EN 100 -4631
EN 120 -7584
EN 140 -7487
EN 160 0
EN 180 -7975
EN 200 0
EN 220 -7404
EN 240 -3687

EN 260 -6434
EN 280 -4481
EN 300 -2999
EN 320 -6329
EN 340 -4858
EN 360 -7825
GTP-RIB:GTP-M5:ALA-CA
EN 20 0
EN 40 0
EN 60 0
EN 80 0
EN 100 0
EN 120 0
EN 140 0
EN 160 0
EN 180 0
EN 200 0
EN 220 0
EN 240 0
EN 260 0
EN 280 -8038
EN 300 0
EN 320 0
EN 340 0
EN 360 0
U-RIB:U-P:VAL-CA
EN 20 0
EN 40 0
EN 60 -3097
EN 80 -2927
EN 100 -3447
EN 120 -190
EN 140 -2844
EN 160 -1412
EN 180 -2221
EN 200 0
EN 220 -5938
EN 240 -5391
EN 260 -2439
EN 280 -4212
EN 300 -3881
EN 320 -2183
EN 340 -2470
EN 360 -2390
C31-RIB:C31-MY:GLU-S2
EN 20 0
EN 40 -9571
EN 60 0
EN 80 0
EN 100 0
EN 120 0
EN 140 -7975
EN 160 0
EN 180 0
EN 200 0
EN 220 0
EN 240 0
EN 260 0

EN 280 0
EN 300 0
EN 320 0
EN 340 0
EN 360 0
FHU-RIB:FHU-MY:LEU-S2
EN 20 0
EN 40 0
EN 60 0
EN 80 -11963
EN 100 0
EN 120 0
EN 140 0
EN 160 -13127
EN 180 0
EN 200 0
EN 220 0
EN 240 0
EN 260 0
EN 280 -9456
EN 300 0
EN 320 0
EN 340 -12675
EN 360 0
G-P:G-RIB:HIS-S2
EN 20 0
EN 40 -3956
EN 60 -5579
EN 80 -2476
EN 100 -2895
EN 120 -2502
EN 140 -4219
EN 160 -5634
EN 180 -1668
EN 200 -6021
EN 220 -4640
EN 240 -3667
EN 260 -3041
EN 280 -4301
EN 300 -4441
EN 320 -4591
EN 340 -3351
EN 360 -6326
A-RIB:A-P:ARG-S2
EN 20 0
EN 40 -6833
EN 60 -5108
EN 80 -5114
EN 100 -4841
EN 120 -4062
EN 140 -4411
EN 160 -5616
EN 180 -4405
EN 200 0
EN 220 -5399
EN 240 -4907
EN 260 -4932
EN 280 -4794

EN 300 -3999
EN 320 -4545
EN 340 -4998
EN 360 -3529
IU-RIB:IU-P:ILE-S1
EN 20 0
EN 40 0
EN 60 0
EN 80 0
EN 100 0
EN 120 0
EN 140 0
EN 160 0
EN 180 0
EN 200 0
EN 220 0
EN 240 0
EN 260 -14393
EN 280 0
EN 300 0
EN 320 0
EN 340 0
EN 360 0
G-RIB:G-R6:GLN-CA
EN 20 0
EN 40 -6344
EN 60 -3460
EN 80 -5299
EN 100 -3631
EN 120 -281
EN 140 0
EN 160 -3665
EN 180 0
EN 200 0
EN 220 -7889
EN 240 -3421
EN 260 -5707
EN 280 -2832
EN 300 -534
EN 320 0
EN 340 0
EN 360 0
C-RIB:C-P:ASN-S1
EN 20 0
EN 40 -5197
EN 60 -2940
EN 80 -2824
EN 100 -4310
EN 120 -4132
EN 140 -2207
EN 160 -3376
EN 180 -4033
EN 200 0
EN 220 -6498
EN 240 -3180
EN 260 -5029
EN 280 -3673
EN 300 -4255

EN 320 -4292
EN 340 -4544
EN 360 -4088
FHU-P:FHU-RIB:LYS-S2
EN 20 -2722
EN 40 0
EN 60 0
EN 80 0
EN 100 -10095
EN 120 0
EN 140 0
EN 160 0
EN 180 0
EN 200 0
EN 220 -11410
EN 240 0
EN 260 0
EN 280 0
EN 300 0
EN 320 0
EN 340 0
EN 360 0
H2U-RIB:H2U-P:ASN-S2
EN 20 0
EN 40 0
EN 60 0
EN 80 0
EN 100 -9346
EN 120 -9819
EN 140 -10576
EN 160 0
EN 180 0
EN 200 0
EN 220 0
EN 240 0
EN 260 0
EN 280 0
EN 300 0
EN 320 0
EN 340 0
EN 360 0
C31-RIB:C31-P:ASP-CA
EN 20 0
EN 40 0
EN 60 0
EN 80 0
EN 100 0
EN 120 -7975
EN 140 -9346
EN 160 0
EN 180 0
EN 200 0
EN 220 0
EN 240 0
EN 260 0
EN 280 0
EN 300 0
EN 320 -10576

EN 340 0
EN 360 0
G-P:G-RIB:LYS-S2
EN 20 -6103
EN 40 -4731
EN 60 -4570
EN 80 -3192
EN 100 -4115
EN 120 -2501
EN 140 -3161
EN 160 -1808
EN 180 1298
EN 200 -4050
EN 220 -5376
EN 240 -5108
EN 260 -4344
EN 280 -3963
EN 300 -2947
EN 320 -1479
EN 340 -2453
EN 360 -1322
FHU-RIB:FHU-MY:SER-S1
EN 20 0
EN 40 0
EN 60 0
EN 80 0
EN 100 -10405
EN 120 0
EN 140 0
EN 160 0
EN 180 0
EN 200 0
EN 220 0
EN 240 0
EN 260 0
EN 280 0
EN 300 0
EN 320 0
EN 340 0
EN 360 0
FHU-P:FHU-RIB:ARG-CA
EN 20 0
EN 40 0
EN 60 0
EN 80 0
EN 100 0
EN 120 0
EN 140 0
EN 160 0
EN 180 0
EN 200 0
EN 220 0
EN 240 0
EN 260 0
EN 280 0
EN 300 0
EN 320 -13127
EN 340 -15398

EN 360 0
U31-RIB:U31-P:LEU-S1
EN 20 0
EN 40 0
EN 60 0
EN 80 0
EN 100 0
EN 120 0
EN 140 0
EN 160 0
EN 180 0
EN 200 0
EN 220 0
EN 240 0
EN 260 0
EN 280 -7796
EN 300 0
EN 320 0
EN 340 0
EN 360 0
U34-RIB:U34-MY:PHE-S1
EN 20 0
EN 40 0
EN 60 0
EN 80 0
EN 100 0
EN 120 0
EN 140 0
EN 160 0
EN 180 0
EN 200 0
EN 220 0
EN 240 0
EN 260 0
EN 280 0
EN 300 -13680
EN 320 0
EN 340 0
EN 360 0
H2U-RIB:H2U-P:LEU-S2
EN 20 0
EN 40 0
EN 60 0
EN 80 0
EN 100 -9042
EN 120 0
EN 140 0
EN 160 0
EN 180 0
EN 200 0
EN 220 0
EN 240 0
EN 260 0
EN 280 0
EN 300 0
EN 320 0
EN 340 0
EN 360 0

A-P:A-RIB:TRP-S2
EN 20 0
EN 40 -6588
EN 60 -3843
EN 80 0
EN 100 -2436
EN 120 -6882
EN 140 -6086
EN 160 -5274
EN 180 0
EN 200 0
EN 220 0
EN 240 -4050
EN 260 -2538
EN 280 -4108
EN 300 -5288
EN 320 -2724
EN 340 -4970
EN 360 -6849
FMU-RIB:FMU-MY:ALA-CA
EN 20 0
EN 40 0
EN 60 0
EN 80 0
EN 100 -11174
EN 120 0
EN 140 0
EN 160 0
EN 180 0
EN 200 0
EN 220 0
EN 240 0
EN 260 0
EN 280 0
EN 300 0
EN 320 0
EN 340 0
EN 360 0
H2U-RIB:H2U-P:ASN-S1
EN 20 0
EN 40 0
EN 60 0
EN 80 0
EN 100 -9819
EN 120 -9953
EN 140 -10760
EN 160 0
EN 180 0
EN 200 0
EN 220 0
EN 240 0
EN 260 0
EN 280 0
EN 300 0
EN 320 0
EN 340 0
EN 360 0
C-RIB:C-Y:CYS-S1

EN 20 0
EN 40 0
EN 60 0
EN 80 -6957
EN 100 0
EN 120 -6069
EN 140 0
EN 160 0
EN 180 0
EN 200 0
EN 220 0
EN 240 -6916
EN 260 0
EN 280 0
EN 300 -7430
EN 320 0
EN 340 0
EN 360 0
U-RIB:U-P:PHE-S1
EN 20 0
EN 40 -9190
EN 60 -4012
EN 80 -2314
EN 100 -1196
EN 120 -2800
EN 140 -3145
EN 160 -2397
EN 180 -5152
EN 200 0
EN 220 -8102
EN 240 -3938
EN 260 -3636
EN 280 -5066
EN 300 -2524
EN 320 -1224
EN 340 0
EN 360 -4651
C31-P:C31-RIB:LEU-CA
EN 20 0
EN 40 0
EN 60 0
EN 80 0
EN 100 0
EN 120 0
EN 140 0
EN 160 0
EN 180 0
EN 200 0
EN 220 0
EN 240 0
EN 260 0
EN 280 0
EN 300 -7575
EN 320 0
EN 340 0
EN 360 0
U31-RIB:U31-P:ASP-S1
EN 20 0

EN 40 0
EN 60 0
EN 80 0
EN 100 0
EN 120 0
EN 140 0
EN 160 0
EN 180 0
EN 200 0
EN 220 0
EN 240 0
EN 260 -7683
EN 280 0
EN 300 -8606
EN 320 0
EN 340 0
EN 360 0
A-RIB:A-R6:ARG-S2
EN 20 0
EN 40 -5797
EN 60 -3792
EN 80 -4621
EN 100 -3954
EN 120 -4314
EN 140 -4386
EN 160 -4058
EN 180 -2750
EN 200 0
EN 220 -4549
EN 240 -4739
EN 260 -4221
EN 280 -2004
EN 300 -3611
EN 320 -4375
EN 340 -3807
EN 360 -2602
QUO-RIB:QUO-M5:LEU-S2
EN 20 0
EN 40 0
EN 60 0
EN 80 0
EN 100 0
EN 120 0
EN 140 0
EN 160 0
EN 180 0
EN 200 0
EN 220 0
EN 240 -17115
EN 260 0
EN 280 -3434
EN 300 0
EN 320 0
EN 340 0
EN 360 0
FMU-P:FMU-RIB:PHE-CA
EN 20 0
EN 40 0

EN 60 0
EN 80 0
EN 100 0
EN 120 0
EN 140 0
EN 160 0
EN 180 0
EN 200 0
EN 220 -2722
EN 240 0
EN 260 0
EN 280 0
EN 300 0
EN 320 0
EN 340 0
EN 360 0
C-P:C-RIB:ARG-CA
EN 20 -8528
EN 40 -7064
EN 60 -5141
EN 80 -4523
EN 100 -4079
EN 120 -4046
EN 140 -3639
EN 160 -4687
EN 180 -4600
EN 200 -6417
EN 220 -4176
EN 240 -5263
EN 260 -5099
EN 280 -4471
EN 300 -4255
EN 320 -4372
EN 340 -3754
EN 360 -4072
H2U-RIB:H2U-P:PRO-S1
EN 20 0
EN 40 0
EN 60 0
EN 80 0
EN 100 -8528
EN 120 0
EN 140 0
EN 160 0
EN 180 0
EN 200 0
EN 220 0
EN 240 0
EN 260 0
EN 280 0
EN 300 0
EN 320 0
EN 340 0
EN 360 0
FMU-RIB:FMU-P:ASP-CA
EN 20 0
EN 40 0
EN 60 0

EN 80 0
EN 100 0
EN 120 0
EN 140 -11671
EN 160 0
EN 180 0
EN 200 0
EN 220 0
EN 240 0
EN 260 0
EN 280 0
EN 300 0
EN 320 0
EN 340 0
EN 360 0
FHU-RIB:FHU-MY:ASP-S2
EN 20 0
EN 40 0
EN 60 0
EN 80 0
EN 100 -9692
EN 120 0
EN 140 0
EN 160 0
EN 180 0
EN 200 0
EN 220 0
EN 240 0
EN 260 0
EN 280 -8859
EN 300 -8378
EN 320 0
EN 340 0
EN 360 0
G-RIB:G-R5:MET-S2
EN 20 -7706
EN 40 -3883
EN 60 -4016
EN 80 -4936
EN 100 0
EN 120 -2350
EN 140 -3095
EN 160 -4304
EN 180 0
EN 200 -4595
EN 220 -2392
EN 240 -3488
EN 260 -5630
EN 280 -6212
EN 300 0
EN 320 -4635
EN 340 -4416
EN 360 0
G-RIB:G-R6:MET-S1
EN 20 0
EN 40 -4486
EN 60 0
EN 80 -6698

EN 100 -3578
EN 120 -2450
EN 140 0
EN 160 0
EN 180 0
EN 200 0
EN 220 -5117
EN 240 -2915
EN 260 -5556
EN 280 -4573
EN 300 -4845
EN 320 -3016
EN 340 -7101
EN 360 0
H2U-RIB:H2U-P:GLU-S2
EN 20 0
EN 40 0
EN 60 -10095
EN 80 0
EN 100 0
EN 120 0
EN 140 -8236
EN 160 0
EN 180 0
EN 200 0
EN 220 0
EN 240 0
EN 260 0
EN 280 0
EN 300 0
EN 320 0
EN 340 0
EN 360 0
QUO-RIB:QUO-P:PHE-S2
EN 20 0
EN 40 -3434
EN 60 0
EN 80 0
EN 100 0
EN 120 0
EN 140 0
EN 160 0
EN 180 0
EN 200 0
EN 220 0
EN 240 0
EN 260 0
EN 280 0
EN 300 0
EN 320 0
EN 340 0
EN 360 0
U34-RIB:U34-MY:PHE-CA
EN 20 0
EN 40 0
EN 60 0
EN 80 0
EN 100 0

EN 120 -15398
EN 140 0
EN 160 0
EN 180 0
EN 200 0
EN 220 0
EN 240 0
EN 260 0
EN 280 0
EN 300 0
EN 320 0
EN 340 0
EN 360 0
H2U-RIB:H2U-MY:ILE-CA
EN 20 0
EN 40 0
EN 60 0
EN 80 0
EN 100 0
EN 120 0
EN 140 0
EN 160 -11410
EN 180 0
EN 200 0
EN 220 0
EN 240 0
EN 260 0
EN 280 0
EN 300 0
EN 320 0
EN 340 0
EN 360 0
IU-RIB:IU-MY:ARG-S2
EN 20 0
EN 40 0
EN 60 0
EN 80 0
EN 100 0
EN 120 -10890
EN 140 -12608
EN 160 -12967
EN 180 0
EN 200 0
EN 220 0
EN 240 0
EN 260 0
EN 280 0
EN 300 0
EN 320 0
EN 340 0
EN 360 0
IU-RIB:IU-P:LYS-S1
EN 20 -2722
EN 40 0
EN 60 0
EN 80 0
EN 100 -7141
EN 120 0

EN 140 0
EN 160 0
EN 180 0
EN 200 0
EN 220 0
EN 240 0
EN 260 0
EN 280 0
EN 300 0
EN 320 0
EN 340 0
EN 360 0
FHU-P:FHU-RIB:ILE-S1
EN 20 0
EN 40 0
EN 60 0
EN 80 0
EN 100 0
EN 120 0
EN 140 0
EN 160 0
EN 180 0
EN 200 0
EN 220 0
EN 240 0
EN 260 0
EN 280 0
EN 300 -11812
EN 320 0
EN 340 0
EN 360 0
QUO-RIB:QUO-M6:LEU-CA
EN 20 0
EN 40 0
EN 60 0
EN 80 0
EN 100 0
EN 120 0
EN 140 0
EN 160 0
EN 180 0
EN 200 0
EN 220 0
EN 240 0
EN 260 -3434
EN 280 0
EN 300 0
EN 320 0
EN 340 0
EN 360 0
U34-RIB:U34-MY:SER-S1
EN 20 0
EN 40 0
EN 60 0
EN 80 0
EN 100 0
EN 120 0
EN 140 0

EN 160 0
EN 180 0
EN 200 0
EN 220 0
EN 240 0
EN 260 0
EN 280 0
EN 300 -8528
EN 320 0
EN 340 0
EN 360 -2722
FMU-RIB:FMU-P:ARG-S2
EN 20 0
EN 40 0
EN 60 0
EN 80 0
EN 100 0
EN 120 -13680
EN 140 0
EN 160 0
EN 180 0
EN 200 0
EN 220 0
EN 240 -13127
EN 260 0
EN 280 0
EN 300 0
EN 320 0
EN 340 0
EN 360 0
H2U-RIB:H2U-MY:THR-CA
EN 20 0
EN 40 0
EN 60 0
EN 80 0
EN 100 0
EN 120 -7914
EN 140 0
EN 160 0
EN 180 0
EN 200 0
EN 220 0
EN 240 0
EN 260 0
EN 280 0
EN 300 -8306
EN 320 0
EN 340 0
EN 360 0
G-RIB:G-R6:THR-CA
EN 20 0
EN 40 -7676
EN 60 -2369
EN 80 -1873
EN 100 -2870
EN 120 -1919
EN 140 -1456
EN 160 -577

EN 180 0
EN 200 0
EN 220 -6404
EN 240 -2026
EN 260 -3756
EN 280 -2830
EN 300 -3647
EN 320 398
EN 340 0
EN 360 0
G-RIB:G-R6:MET-CA
EN 20 0
EN 40 -6281
EN 60 -5222
EN 80 -6201
EN 100 -1996
EN 120 -3915
EN 140 0
EN 160 0
EN 180 0
EN 200 0
EN 220 0
EN 240 -6112
EN 260 -3953
EN 280 -3618
EN 300 -4993
EN 320 -5122
EN 340 -4290
EN 360 -6899
U34-RIB:U34-P:SER-S1
EN 20 0
EN 40 0
EN 60 -7854
EN 80 0
EN 100 0
EN 120 -8452
EN 140 0
EN 160 0
EN 180 0
EN 200 0
EN 220 0
EN 240 0
EN 260 -6227
EN 280 0
EN 300 0
EN 320 0
EN 340 0
EN 360 0
U31-P:U31-RIB:ASP-S2
EN 20 0
EN 40 -10405
EN 60 0
EN 80 0
EN 100 -8772
EN 120 0
EN 140 0
EN 160 -7796
EN 180 0

EN 200 0
EN 220 0
EN 240 0
EN 260 0
EN 280 0
EN 300 -6624
EN 320 0
EN 340 -7796
EN 360 0
C31-P:C31-RIB:SER-S1
EN 20 0
EN 40 0
EN 60 0
EN 80 0
EN 100 0
EN 120 0
EN 140 0
EN 160 0
EN 180 0
EN 200 0
EN 220 0
EN 240 0
EN 260 0
EN 280 0
EN 300 0
EN 320 -7914
EN 340 0
EN 360 0
G-P:G-RIB:PRO-S1
EN 20 -4509
EN 40 -2413
EN 60 -4717
EN 80 -3934
EN 100 -4287
EN 120 -3032
EN 140 -3884
EN 160 -3727
EN 180 -716
EN 200 -5274
EN 220 -470
EN 240 -3356
EN 260 -2573
EN 280 -2489
EN 300 -3168
EN 320 -2548
EN 340 -3075
EN 360 -3673
FMU-RIB:FMU-P:ALA-CA
EN 20 0
EN 40 0
EN 60 0
EN 80 0
EN 100 0
EN 120 0
EN 140 0
EN 160 0
EN 180 0
EN 200 0

EN 220 0
EN 240 -14393
EN 260 0
EN 280 0
EN 300 0
EN 320 0
EN 340 0
EN 360 0
FHU-RIB:FHU-MY:LEU-CA
EN 20 0
EN 40 0
EN 60 0
EN 80 -11410
EN 100 0
EN 120 0
EN 140 0
EN 160 -11671
EN 180 0
EN 200 0
EN 220 0
EN 240 0
EN 260 -9692
EN 280 0
EN 300 0
EN 320 0
EN 340 -13127
EN 360 0
U31-P:U31-RIB:TYR-S2
EN 20 0
EN 40 0
EN 60 0
EN 80 0
EN 100 0
EN 120 0
EN 140 0
EN 160 -8772
EN 180 0
EN 200 0
EN 220 0
EN 240 0
EN 260 0
EN 280 -8378
EN 300 0
EN 320 0
EN 340 0
EN 360 0
FMU-RIB:FMU-P:CYS-S1
EN 20 0
EN 40 0
EN 60 0
EN 80 0
EN 100 0
EN 120 0
EN 140 0
EN 160 0
EN 180 0
EN 200 -2722
EN 220 0

EN 240 0
EN 260 0
EN 280 0
EN 300 0
EN 320 0
EN 340 0
EN 360 0
A-RIB:A-R5:LYS-S1
EN 20 -5698
EN 40 -1687
EN 60 -3392
EN 80 -4004
EN 100 -4449
EN 120 -2543
EN 140 -4385
EN 160 -143
EN 180 0
EN 200 -6497
EN 220 -5400
EN 240 -4429
EN 260 -3972
EN 280 -2645
EN 300 -5059
EN 320 -3443
EN 340 -1703
EN 360 -2492
A-RIB:A-P:CYS-S1
EN 20 0
EN 40 0
EN 60 -6970
EN 80 0
EN 100 -4248
EN 120 0
EN 140 0
EN 160 0
EN 180 -7796
EN 200 0
EN 220 0
EN 240 0
EN 260 -8502
EN 280 -6242
EN 300 -4114
EN 320 0
EN 340 0
EN 360 0
FHU-P:FHU-RIB:LEU-CA
EN 20 0
EN 40 0
EN 60 0
EN 80 0
EN 100 -11963
EN 120 0
EN 140 0
EN 160 0
EN 180 0
EN 200 0
EN 220 0
EN 240 0

EN 260 0
EN 280 -9241
EN 300 0
EN 320 0
EN 340 0
EN 360 0
C-RIB:C-Y:PHE-CA
EN 20 0
EN 40 0
EN 60 -5249
EN 80 -2952
EN 100 -5836
EN 120 -2717
EN 140 -3309
EN 160 -6176
EN 180 0
EN 200 0
EN 220 -4375
EN 240 0
EN 260 -2692
EN 280 -4260
EN 300 -2636
EN 320 -3186
EN 340 -4054
EN 360 -7325
FMU-P:FMU-RIB:PHE-S2
EN 20 0
EN 40 0
EN 60 0
EN 80 0
EN 100 0
EN 120 0
EN 140 0
EN 160 0
EN 180 0
EN 200 0
EN 220 0
EN 240 0
EN 260 -2722
EN 280 0
EN 300 0
EN 320 0
EN 340 -2722
EN 360 0
IU-P:IU-RIB:ALA-S1
EN 20 0
EN 40 0
EN 60 0
EN 80 0
EN 100 -13127
EN 120 0
EN 140 0
EN 160 0
EN 180 0
EN 200 0
EN 220 0
EN 240 0
EN 260 0

EN 280 0
EN 300 -11671
EN 320 0
EN 340 0
EN 360 0
U31-RIB:U31-MY:ALA-CA
EN 20 0
EN 40 0
EN 60 0
EN 80 0
EN 100 0
EN 120 0
EN 140 0
EN 160 0
EN 180 0
EN 200 0
EN 220 0
EN 240 0
EN 260 0
EN 280 -7373
EN 300 0
EN 320 0
EN 340 0
EN 360 0
U34-P:U34-RIB:TYR-S2
EN 20 0
EN 40 0
EN 60 0
EN 80 0
EN 100 0
EN 120 0
EN 140 0
EN 160 0
EN 180 0
EN 200 0
EN 220 0
EN 240 0
EN 260 0
EN 280 0
EN 300 -14393
EN 320 0
EN 340 0
EN 360 0
FMU-RIB:FMU-P:ARG-S1
EN 20 0
EN 40 0
EN 60 0
EN 80 0
EN 100 -13680
EN 120 -12294
EN 140 0
EN 160 0
EN 180 0
EN 200 0
EN 220 0
EN 240 -14393
EN 260 0
EN 280 0

EN 300 0
EN 320 0
EN 340 0
EN 360 0
FHU-RIB:FHU-P:ARG-S1
EN 20 0
EN 40 0
EN 60 0
EN 80 -9241
EN 100 0
EN 120 0
EN 140 0
EN 160 0
EN 180 0
EN 200 0
EN 220 0
EN 240 0
EN 260 0
EN 280 -8306
EN 300 0
EN 320 0
EN 340 0
EN 360 0
A-RIB:A-R5:ASN-CA
EN 20 -9275
EN 40 -4189
EN 60 -3578
EN 80 -4211
EN 100 -4413
EN 120 -3612
EN 140 -4199
EN 160 0
EN 180 0
EN 200 -6021
EN 220 -2842
EN 240 -2524
EN 260 -5051
EN 280 -1929
EN 300 -723
EN 320 -4065
EN 340 -2559
EN 360 -6357
M2G-RIB:M2G-P:GLU-CA
EN 20 0
EN 40 0
EN 60 0
EN 80 0
EN 100 0
EN 120 0
EN 140 0
EN 160 0
EN 180 0
EN 200 0
EN 220 0
EN 240 0
EN 260 0
EN 280 0
EN 300 0

EN 320 0
EN 340 0
EN 360 -3434
DA-RIB:DA-M6:LYS-S2
EN 20 0
EN 40 0
EN 60 0
EN 80 0
EN 100 0
EN 120 0
EN 140 0
EN 160 0
EN 180 0
EN 200 0
EN 220 0
EN 240 0
EN 260 0
EN 280 0
EN 300 -8378
EN 320 0
EN 340 0
EN 360 0
I-RIB:I-P:TRP-S2
EN 20 0
EN 40 0
EN 60 0
EN 80 0
EN 100 0
EN 120 0
EN 140 0
EN 160 0
EN 180 0
EN 200 0
EN 220 0
EN 240 -1717
EN 260 0
EN 280 0
EN 300 0
EN 320 0
EN 340 0
EN 360 0
QUO-RIB:QUO-P:LEU-S1
EN 20 0
EN 40 0
EN 60 0
EN 80 -17115
EN 100 -3434
EN 120 0
EN 140 0
EN 160 0
EN 180 0
EN 200 0
EN 220 0
EN 240 0
EN 260 0
EN 280 0
EN 300 0
EN 320 0

EN 340 0
EN 360 0
U-RIB:U-P:ILE-CA
EN 20 0
EN 40 0
EN 60 -4339
EN 80 0
EN 100 -3724
EN 120 -1232
EN 140 -3457
EN 160 -1286
EN 180 0
EN 200 0
EN 220 0
EN 240 -2450
EN 260 -2374
EN 280 222
EN 300 352
EN 320 -3931
EN 340 -3598
EN 360 0
H2U-RIB:H2U-MY:GLY-CA
EN 20 0
EN 40 0
EN 60 0
EN 80 0
EN 100 0
EN 120 -5631
EN 140 0
EN 160 0
EN 180 0
EN 200 0
EN 220 0
EN 240 0
EN 260 0
EN 280 0
EN 300 0
EN 320 0
EN 340 0
EN 360 0
IU-P:IU-RIB:ARG-CA
EN 20 0
EN 40 0
EN 60 0
EN 80 0
EN 100 0
EN 120 0
EN 140 0
EN 160 0
EN 180 0
EN 200 0
EN 220 0
EN 240 0
EN 260 0
EN 280 -10095
EN 300 0
EN 320 0
EN 340 0

EN 360 0
G-RIB:G-R5:GLU-CA
EN 20 -3444
EN 40 -1274
EN 60 -52
EN 80 1771
EN 100 1722
EN 120 -166
EN 140 1809
EN 160 -1574
EN 180 -2433
EN 200 0
EN 220 197
EN 240 -1763
EN 260 -953
EN 280 -1433
EN 300 1551
EN 320 -2224
EN 340 -2475
EN 360 -4052
PSU-P:PSU-RIB:ARG-S2
EN 20 0
EN 40 0
EN 60 0
EN 80 0
EN 100 0
EN 120 -11410
EN 140 0
EN 160 0
EN 180 0
EN 200 0
EN 220 0
EN 240 0
EN 260 0
EN 280 0
EN 300 0
EN 320 0
EN 340 0
EN 360 0
A-RIB:A-R6:LYS-S1
EN 20 0
EN 40 -3615
EN 60 -3532
EN 80 -3212
EN 100 -2582
EN 120 -3206
EN 140 -1565
EN 160 -3559
EN 180 0
EN 200 0
EN 220 -4692
EN 240 -2814
EN 260 -4455
EN 280 -4144
EN 300 -2544
EN 320 -2607
EN 340 -610
EN 360 0

C-RIB:C-Y:GLY-CA
EN 20 -6086
EN 40 -4224
EN 60 -4853
EN 80 -3247
EN 100 -4080
EN 120 -2205
EN 140 -2682
EN 160 -4453
EN 180 -3845
EN 200 -6758
EN 220 -4251
EN 240 -4008
EN 260 -4338
EN 280 -4402
EN 300 -3006
EN 320 -3482
EN 340 0
EN 360 -1989
A-P:A-RIB:ASN-CA
EN 20 0
EN 40 -3762
EN 60 -4224
EN 80 -2929
EN 100 -4637
EN 120 -3389
EN 140 -5152
EN 160 -1712
EN 180 -3520
EN 200 0
EN 220 -5243
EN 240 -6111
EN 260 -3609
EN 280 -3505
EN 300 -3166
EN 320 -4891
EN 340 -2622
EN 360 -6368
5BU-P:5BU-RIB:ARG-S2
EN 20 0
EN 40 0
EN 60 0
EN 80 0
EN 100 0
EN 120 0
EN 140 0
EN 160 0
EN 180 0
EN 200 -17115
EN 220 0
EN 240 0
EN 260 0
EN 280 0
EN 300 0
EN 320 0
EN 340 0
EN 360 0
C31-RIB:C31-P:TYR-S1

EN 20 0
EN 40 0
EN 60 0
EN 80 0
EN 100 -7629
EN 120 0
EN 140 0
EN 160 0
EN 180 0
EN 200 0
EN 220 0
EN 240 0
EN 260 0
EN 280 -9456
EN 300 0
EN 320 0
EN 340 0
EN 360 0
FHU-RIB:FHU-MY:ARG-S1
EN 20 0
EN 40 0
EN 60 0
EN 80 0
EN 100 -9042
EN 120 0
EN 140 -10576
EN 160 0
EN 180 0
EN 200 0
EN 220 0
EN 240 0
EN 260 0
EN 280 -8102
EN 300 0
EN 320 -10576
EN 340 0
EN 360 0
H2U-RIB:H2U-MY:ARG-S1
EN 20 0
EN 40 0
EN 60 0
EN 80 0
EN 100 0
EN 120 0
EN 140 0
EN 160 0
EN 180 0
EN 200 0
EN 220 0
EN 240 0
EN 260 0
EN 280 0
EN 300 -7796
EN 320 0
EN 340 0
EN 360 0
H2U-RIB:H2U-P:PHE-S1
EN 20 0

EN 40 0
EN 60 0
EN 80 -17115
EN 100 0
EN 120 0
EN 140 0
EN 160 0
EN 180 0
EN 200 0
EN 220 0
EN 240 0
EN 260 0
EN 280 0
EN 300 0
EN 320 0
EN 340 0
EN 360 0
H2U-RIB:H2U-MY:GLU-S2
EN 20 0
EN 40 0
EN 60 0
EN 80 0
EN 100 -6697
EN 120 0
EN 140 0
EN 160 0
EN 180 0
EN 200 0
EN 220 0
EN 240 0
EN 260 0
EN 280 0
EN 300 0
EN 320 0
EN 340 0
EN 360 0
FHU-RIB:FHU-P:LEU-S2
EN 20 0
EN 40 0
EN 60 0
EN 80 -10405
EN 100 0
EN 120 0
EN 140 0
EN 160 0
EN 180 0
EN 200 0
EN 220 0
EN 240 -15398
EN 260 0
EN 280 0
EN 300 0
EN 320 -9819
EN 340 0
EN 360 0
M2G-RIB:M2G-P:GLU-S1
EN 20 0
EN 40 0

EN 60 0
EN 80 0
EN 100 0
EN 120 0
EN 140 0
EN 160 0
EN 180 0
EN 200 0
EN 220 0
EN 240 0
EN 260 0
EN 280 0
EN 300 0
EN 320 0
EN 340 0
EN 360 -3434
FMU-P:FMU-RIB:CYS-S1
EN 20 0
EN 40 0
EN 60 0
EN 80 0
EN 100 0
EN 120 0
EN 140 0
EN 160 -2722
EN 180 0
EN 200 0
EN 220 0
EN 240 0
EN 260 0
EN 280 0
EN 300 0
EN 320 0
EN 340 0
EN 360 0
U-RIB:U-Y:TYR-CA
EN 20 0
EN 40 0
EN 60 -3645
EN 80 -3434
EN 100 -5504
EN 120 0
EN 140 -4684
EN 160 0
EN 180 0
EN 200 0
EN 220 -5468
EN 240 -3866
EN 260 -4711
EN 280 -8100
EN 300 -5973
EN 320 -5840
EN 340 -4756
EN 360 0
5BU-RIB:5BU-P:ALA-S1
EN 20 0
EN 40 0
EN 60 0

EN 80 0
EN 100 0
EN 120 0
EN 140 0
EN 160 0
EN 180 0
EN 200 0
EN 220 0
EN 240 0
EN 260 0
EN 280 0
EN 300 0
EN 320 -15398
EN 340 0
EN 360 0
C31-RIB:C31-P:GLN-S1
EN 20 0
EN 40 0
EN 60 0
EN 80 0
EN 100 0
EN 120 0
EN 140 0
EN 160 -8528
EN 180 0
EN 200 0
EN 220 0
EN 240 0
EN 260 0
EN 280 0
EN 300 0
EN 320 0
EN 340 0
EN 360 0
A-RIB:A-R6:CYS-S1
EN 20 0
EN 40 0
EN 60 -9346
EN 80 -5446
EN 100 0
EN 120 0
EN 140 0
EN 160 0
EN 180 0
EN 200 0
EN 220 0
EN 240 0
EN 260 -6519
EN 280 -5003
EN 300 0
EN 320 -5602
EN 340 0
EN 360 0
U31-RIB:U31-P:MET-S1
EN 20 0
EN 40 0
EN 60 0
EN 80 0

EN 100 0
EN 120 0
EN 140 0
EN 160 0
EN 180 0
EN 200 0
EN 220 0
EN 240 -13127
EN 260 0
EN 280 0
EN 300 -8378
EN 320 0
EN 340 0
EN 360 0
FHU-P:FHU-RIB:LEU-S1
EN 20 0
EN 40 0
EN 60 0
EN 80 0
EN 100 -12294
EN 120 0
EN 140 0
EN 160 0
EN 180 0
EN 200 0
EN 220 0
EN 240 0
EN 260 0
EN 280 -9819
EN 300 0
EN 320 0
EN 340 0
EN 360 0
QUO-P:QUO-RIB:ASN-S2
EN 20 0
EN 40 0
EN 60 0
EN 80 0
EN 100 0
EN 120 0
EN 140 0
EN 160 -3434
EN 180 0
EN 200 0
EN 220 0
EN 240 0
EN 260 0
EN 280 0
EN 300 0
EN 320 0
EN 340 0
EN 360 0
C31-RIB:C31-MY:ALA-S1
EN 20 0
EN 40 0
EN 60 0
EN 80 0
EN 100 0

EN 120 0
EN 140 0
EN 160 0
EN 180 0
EN 200 0
EN 220 0
EN 240 0
EN 260 0
EN 280 -9953
EN 300 0
EN 320 0
EN 340 0
EN 360 0
GTP-RIB:GTP-M6:ASN-CA
EN 20 0
EN 40 -15398
EN 60 0
EN 80 0
EN 100 0
EN 120 0
EN 140 0
EN 160 0
EN 180 0
EN 200 0
EN 220 0
EN 240 0
EN 260 0
EN 280 0
EN 300 0
EN 320 0
EN 340 0
EN 360 0
FMU-RIB:FMU-MY:GLN-CA
EN 20 0
EN 40 0
EN 60 0
EN 80 0
EN 100 0
EN 120 0
EN 140 0
EN 160 0
EN 180 0
EN 200 0
EN 220 0
EN 240 0
EN 260 0
EN 280 -15398
EN 300 0
EN 320 0
EN 340 0
EN 360 0
FHU-RIB:FHU-MY:GLY-CA
EN 20 0
EN 40 0
EN 60 -9953
EN 80 0
EN 100 0
EN 120 0

EN 140 0
EN 160 0
EN 180 0
EN 200 0
EN 220 0
EN 240 -10760
EN 260 0
EN 280 0
EN 300 0
EN 320 0
EN 340 0
EN 360 0
FHU-RIB:FHU-P:THR-S1
EN 20 0
EN 40 0
EN 60 0
EN 80 -11174
EN 100 0
EN 120 0
EN 140 0
EN 160 0
EN 180 0
EN 200 0
EN 220 0
EN 240 -11174
EN 260 0
EN 280 0
EN 300 0
EN 320 -9456
EN 340 0
EN 360 0
U31-RIB:U31-P:MET-CA
EN 20 0
EN 40 0
EN 60 0
EN 80 0
EN 100 0
EN 120 0
EN 140 0
EN 160 0
EN 180 0
EN 200 0
EN 220 0
EN 240 0
EN 260 -10576
EN 280 0
EN 300 -9571
EN 320 0
EN 340 0
EN 360 0
QUO-RIB:QUO-P:LEU-CA
EN 20 0
EN 40 0
EN 60 0
EN 80 0
EN 100 -5152
EN 120 0
EN 140 0

EN 160 0
EN 180 0
EN 200 0
EN 220 0
EN 240 0
EN 260 0
EN 280 0
EN 300 0
EN 320 0
EN 340 0
EN 360 0
U-RIB:U-Y:ALA-CA
EN 20 -4318
EN 40 -3523
EN 60 -5080
EN 80 -4316
EN 100 -2699
EN 120 -2331
EN 140 -1698
EN 160 -4388
EN 180 0
EN 200 0
EN 220 -1661
EN 240 -4561
EN 260 -5184
EN 280 -2724
EN 300 -2678
EN 320 -2904
EN 340 -3934
EN 360 0
IU-P:IU-RIB:PRO-CA
EN 20 0
EN 40 0
EN 60 0
EN 80 0
EN 100 0
EN 120 0
EN 140 -12294
EN 160 0
EN 180 0
EN 200 0
EN 220 0
EN 240 0
EN 260 0
EN 280 0
EN 300 0
EN 320 0
EN 340 0
EN 360 0
QUO-RIB:QUO-M5:ASN-S2
EN 20 0
EN 40 0
EN 60 0
EN 80 -3434
EN 100 0
EN 120 0
EN 140 0
EN 160 0

EN 180 0
EN 200 0
EN 220 0
EN 240 0
EN 260 0
EN 280 0
EN 300 0
EN 320 0
EN 340 0
EN 360 0
U31-P:U31-RIB:GLN-S1
EN 20 0
EN 40 0
EN 60 0
EN 80 -9456
EN 100 0
EN 120 0
EN 140 0
EN 160 0
EN 180 0
EN 200 0
EN 220 0
EN 240 0
EN 260 0
EN 280 0
EN 300 0
EN 320 0
EN 340 0
EN 360 0
A-P:A-RIB:ARG-CA
EN 20 -10297
EN 40 -6595
EN 60 -4485
EN 80 -4419
EN 100 -5484
EN 120 -3970
EN 140 -4220
EN 160 -5256
EN 180 -2827
EN 200 -7739
EN 220 -6772
EN 240 -4592
EN 260 -5306
EN 280 -4344
EN 300 -4321
EN 320 -4353
EN 340 -3935
EN 360 -5002
A-RIB:A-R5:SER-S1
EN 20 -6030
EN 40 -4205
EN 60 -4738
EN 80 -2842
EN 100 -2897
EN 120 -3502
EN 140 -4271
EN 160 -4559
EN 180 -6304

EN 200 -4428
EN 220 -4491
EN 240 -3850
EN 260 -4162
EN 280 -4753
EN 300 -3253
EN 320 -4003
EN 340 -2717
EN 360 0
5BU-RIB:5BU-P:ILE-S1
EN 20 0
EN 40 0
EN 60 0
EN 80 0
EN 100 0
EN 120 -14393
EN 140 0
EN 160 0
EN 180 0
EN 200 0
EN 220 0
EN 240 0
EN 260 0
EN 280 0
EN 300 0
EN 320 0
EN 340 -15398
EN 360 0
H2U-P:H2U-RIB:GLU-S1
EN 20 0
EN 40 0
EN 60 0
EN 80 0
EN 100 0
EN 120 0
EN 140 0
EN 160 0
EN 180 0
EN 200 0
EN 220 0
EN 240 0
EN 260 0
EN 280 -7914
EN 300 0
EN 320 0
EN 340 0
EN 360 0
U-RIB:U-Y:LEU-S1
EN 20 0
EN 40 -5876
EN 60 -7593
EN 80 -3757
EN 100 -1256
EN 120 -4444
EN 140 -1566
EN 160 -2805
EN 180 -5560
EN 200 0

EN 220 0
EN 240 -6012
EN 260 -5178
EN 280 -4014
EN 300 -4647
EN 320 -3384
EN 340 -4446
EN 360 0
QUO-RIB:QUO-M6:GLU-S2
EN 20 0
EN 40 0
EN 60 0
EN 80 0
EN 100 0
EN 120 0
EN 140 0
EN 160 -3434
EN 180 0
EN 200 0
EN 220 0
EN 240 0
EN 260 0
EN 280 0
EN 300 0
EN 320 0
EN 340 0
EN 360 0
A-RIB:A-R6:ASN-S1
EN 20 -9042
EN 40 0
EN 60 -2093
EN 80 -3333
EN 100 -3327
EN 120 -4790
EN 140 -1810
EN 160 -2501
EN 180 0
EN 200 0
EN 220 -4880
EN 240 -4028
EN 260 -4167
EN 280 -2804
EN 300 -3546
EN 320 -2529
EN 340 -4562
EN 360 -3556
G-RIB:G-R6:PHE-CA
EN 20 0
EN 40 -6242
EN 60 -5587
EN 80 -5059
EN 100 0
EN 120 -1885
EN 140 -4050
EN 160 0
EN 180 0
EN 200 0
EN 220 0

EN 240 -5297
EN 260 -4264
EN 280 -4636
EN 300 -3610
EN 320 0
EN 340 0
EN 360 0
U31-RIB:U31-MY:ALA-S1
EN 20 0
EN 40 0
EN 60 0
EN 80 0
EN 100 0
EN 120 -7097
EN 140 0
EN 160 0
EN 180 0
EN 200 0
EN 220 0
EN 240 0
EN 260 0
EN 280 -7054
EN 300 0
EN 320 0
EN 340 0
EN 360 0
QUO-RIB:QUO-M6:ASP-S1
EN 20 0
EN 40 0
EN 60 0
EN 80 0
EN 100 0
EN 120 -3434
EN 140 0
EN 160 0
EN 180 0
EN 200 0
EN 220 0
EN 240 0
EN 260 0
EN 280 0
EN 300 0
EN 320 0
EN 340 0
EN 360 0
U-RIB:U-P:GLY-CA
EN 20 -6320
EN 40 -5993
EN 60 -4727
EN 80 -2967
EN 100 -1079
EN 120 -2659
EN 140 -5000
EN 160 -4572
EN 180 -3321
EN 200 -7683
EN 220 -6124
EN 240 -2436

EN 260 -4535
EN 280 -4047
EN 300 -3765
EN 320 -879
EN 340 -4408
EN 360 -644
C31-P:C31-RIB:ASP-S2
EN 20 0
EN 40 -13388
EN 60 0
EN 80 0
EN 100 0
EN 120 0
EN 140 0
EN 160 0
EN 180 0
EN 200 0
EN 220 0
EN 240 0
EN 260 0
EN 280 0
EN 300 0
EN 320 0
EN 340 0
EN 360 0
G-P:G-RIB:CYS-S1
EN 20 0
EN 40 0
EN 60 0
EN 80 0
EN 100 0
EN 120 -4037
EN 140 -4700
EN 160 0
EN 180 -7523
EN 200 0
EN 220 0
EN 240 0
EN 260 -3567
EN 280 0
EN 300 -3505
EN 320 -4635
EN 340 0
EN 360 0
DA-RIB:DA-M5:TYR-S1
EN 20 0
EN 40 0
EN 60 0
EN 80 0
EN 100 -8688
EN 120 0
EN 140 0
EN 160 0
EN 180 0
EN 200 0
EN 220 0
EN 240 0
EN 260 0

EN 280 0
EN 300 -8378
EN 320 0
EN 340 0
EN 360 0
G-P:G-RIB:CYS-CA
EN 20 0
EN 40 0
EN 60 0
EN 80 0
EN 100 0
EN 120 -5680
EN 140 0
EN 160 0
EN 180 -7186
EN 200 0
EN 220 0
EN 240 0
EN 260 0
EN 280 -5929
EN 300 -3535
EN 320 0
EN 340 0
EN 360 0
OMC-P:OMC-RIB:LYS-S2
EN 20 0
EN 40 0
EN 60 0
EN 80 0
EN 100 0
EN 120 0
EN 140 0
EN 160 0
EN 180 0
EN 200 0
EN 220 0
EN 240 -7683
EN 260 0
EN 280 -7422
EN 300 0
EN 320 0
EN 340 0
EN 360 0
H2U-P:H2U-RIB:GLU-CA
EN 20 0
EN 40 0
EN 60 0
EN 80 0
EN 100 0
EN 120 0
EN 140 0
EN 160 0
EN 180 0
EN 200 0
EN 220 0
EN 240 0
EN 260 -9042
EN 280 0

EN 300 0
EN 320 0
EN 340 0
EN 360 0
IU-RIB:IU-MY:GLU-S2
EN 20 0
EN 40 0
EN 60 0
EN 80 0
EN 100 0
EN 120 0
EN 140 -8903
EN 160 0
EN 180 0
EN 200 0
EN 220 0
EN 240 0
EN 260 0
EN 280 0
EN 300 0
EN 320 0
EN 340 0
EN 360 0
H2U-P:H2U-RIB:PRO-S1
EN 20 0
EN 40 0
EN 60 0
EN 80 0
EN 100 0
EN 120 0
EN 140 0
EN 160 0
EN 180 0
EN 200 0
EN 220 0
EN 240 0
EN 260 0
EN 280 0
EN 300 -9456
EN 320 0
EN 340 0
EN 360 0
U31-RIB:U31-P:ARG-S2
EN 20 0
EN 40 0
EN 60 0
EN 80 0
EN 100 0
EN 120 0
EN 140 0
EN 160 0
EN 180 0
EN 200 0
EN 220 0
EN 240 0
EN 260 0
EN 280 0
EN 300 -7683

EN 320 0
EN 340 0
EN 360 0
5BU-RIB:5BU-MY:PRO-CA
EN 20 0
EN 40 0
EN 60 -17115
EN 80 0
EN 100 0
EN 120 0
EN 140 0
EN 160 0
EN 180 0
EN 200 0
EN 220 0
EN 240 0
EN 260 0
EN 280 0
EN 300 0
EN 320 0
EN 340 0
EN 360 0
U31-RIB:U31-MY:MET-S2
EN 20 0
EN 40 0
EN 60 0
EN 80 0
EN 100 -8102
EN 120 0
EN 140 0
EN 160 0
EN 180 0
EN 200 0
EN 220 0
EN 240 0
EN 260 0
EN 280 0
EN 300 0
EN 320 0
EN 340 0
EN 360 0
FMU-P:FMU-RIB:ALA-CA
EN 20 0
EN 40 0
EN 60 0
EN 80 0
EN 100 -12675
EN 120 0
EN 140 0
EN 160 0
EN 180 0
EN 200 0
EN 220 0
EN 240 0
EN 260 0
EN 280 0
EN 300 0
EN 320 0

EN 340 0
EN 360 0
DA-RIB:DA-M5:GLU-S1
EN 20 0
EN 40 0
EN 60 0
EN 80 0
EN 100 0
EN 120 0
EN 140 0
EN 160 0
EN 180 0
EN 200 0
EN 220 0
EN 240 0
EN 260 0
EN 280 0
EN 300 0
EN 320 -7325
EN 340 0
EN 360 0
A-RIB:A-R6:TYR-CA
EN 20 0
EN 40 -8038
EN 60 -6663
EN 80 -6926
EN 100 -3925
EN 120 -4602
EN 140 0
EN 160 -5258
EN 180 -5780
EN 200 0
EN 220 -7337
EN 240 -7656
EN 260 -6396
EN 280 -1806
EN 300 -5310
EN 320 -6105
EN 340 -5000
EN 360 -7251
QUO-RIB:QUO-M6:LEU-S2
EN 20 0
EN 40 0
EN 60 0
EN 80 0
EN 100 0
EN 120 0
EN 140 0
EN 160 0
EN 180 0
EN 200 0
EN 220 0
EN 240 -3434
EN 260 -3434
EN 280 0
EN 300 0
EN 320 0
EN 340 0

EN 360 0
QUO-RIB:QUO-M6:PHE-S1
EN 20 0
EN 40 0
EN 60 0
EN 80 0
EN 100 0
EN 120 0
EN 140 0
EN 160 0
EN 180 0
EN 200 0
EN 220 0
EN 240 0
EN 260 -3434
EN 280 0
EN 300 0
EN 320 -3434
EN 340 0
EN 360 0
5BU-RIB:5BU-MY:PRO-S1
EN 20 0
EN 40 0
EN 60 -14393
EN 80 0
EN 100 0
EN 120 0
EN 140 0
EN 160 0
EN 180 0
EN 200 0
EN 220 0
EN 240 0
EN 260 0
EN 280 0
EN 300 0
EN 320 0
EN 340 0
EN 360 0
H2U-RIB:H2U-P:GLU-S1
EN 20 0
EN 40 0
EN 60 0
EN 80 -9042
EN 100 0
EN 120 0
EN 140 -8306
EN 160 0
EN 180 0
EN 200 0
EN 220 0
EN 240 0
EN 260 0
EN 280 0
EN 300 0
EN 320 0
EN 340 0
EN 360 0

A-RIB:A-R5:GLY-CA
EN 20 -7065
EN 40 -5734
EN 60 -3672
EN 80 -4498
EN 100 -3466
EN 120 -1133
EN 140 -2989
EN 160 -3911
EN 180 -2312
EN 200 -7171
EN 220 -4213
EN 240 -4752
EN 260 -4983
EN 280 -2872
EN 300 -4446
EN 320 -3747
EN 340 -3348
EN 360 -5903
FHU-RIB:FHU-MY:SER-CA
EN 20 0
EN 40 0
EN 60 0
EN 80 0
EN 100 0
EN 120 -10576
EN 140 0
EN 160 0
EN 180 0
EN 200 0
EN 220 0
EN 240 -14393
EN 260 0
EN 280 0
EN 300 0
EN 320 0
EN 340 0
EN 360 0
FHU-P:FHU-RIB:GLY-CA
EN 20 0
EN 40 -16110
EN 60 -9692
EN 80 0
EN 100 0
EN 120 0
EN 140 0
EN 160 0
EN 180 0
EN 200 0
EN 220 0
EN 240 -8859
EN 260 0
EN 280 0
EN 300 0
EN 320 0
EN 340 0
EN 360 0
QUO-RIB:QUO-M6:LYS-CA

EN 20 0
EN 40 0
EN 60 0
EN 80 -3434
EN 100 0
EN 120 0
EN 140 0
EN 160 0
EN 180 0
EN 200 0
EN 220 0
EN 240 0
EN 260 0
EN 280 0
EN 300 0
EN 320 0
EN 340 0
EN 360 0
IU-P:IU-RIB:HIS-S2
EN 20 0
EN 40 0
EN 60 0
EN 80 0
EN 100 0
EN 120 0
EN 140 0
EN 160 0
EN 180 0
EN 200 0
EN 220 -15398
EN 240 0
EN 260 0
EN 280 0
EN 300 0
EN 320 0
EN 340 0
EN 360 0
H2U-P:H2U-RIB:TRP-S2
EN 20 0
EN 40 0
EN 60 0
EN 80 0
EN 100 0
EN 120 0
EN 140 0
EN 160 0
EN 180 0
EN 200 0
EN 220 0
EN 240 -17115
EN 260 0
EN 280 0
EN 300 0
EN 320 0
EN 340 0
EN 360 0
FMU-RIB:FMU-MY:MET-S2
EN 20 0

EN 40 0
EN 60 0
EN 80 0
EN 100 0
EN 120 0
EN 140 0
EN 160 0
EN 180 0
EN 200 0
EN 220 0
EN 240 0
EN 260 0
EN 280 0
EN 300 -15398
EN 320 -2722
EN 340 0
EN 360 0
QUO-RIB:QUO-M5:LYS-S2
EN 20 0
EN 40 0
EN 60 0
EN 80 -3434
EN 100 0
EN 120 0
EN 140 0
EN 160 0
EN 180 0
EN 200 0
EN 220 0
EN 240 0
EN 260 0
EN 280 0
EN 300 0
EN 320 0
EN 340 0
EN 360 0
U-P:U-RIB:MET-CA
EN 20 0
EN 40 -8743
EN 60 -3337
EN 80 -2476
EN 100 -4813
EN 120 -5119
EN 140 0
EN 160 -5468
EN 180 0
EN 200 0
EN 220 -7796
EN 240 0
EN 260 -2524
EN 280 -4955
EN 300 -4540
EN 320 0
EN 340 0
EN 360 0
FHU-RIB:FHU-MY:THR-CA
EN 20 0
EN 40 0

EN 60 0
EN 80 -10405
EN 100 0
EN 120 0
EN 140 0
EN 160 0
EN 180 0
EN 200 0
EN 220 -2722
EN 240 0
EN 260 -8859
EN 280 0
EN 300 -10095
EN 320 0
EN 340 0
EN 360 0
5BU-RIB:5BU-P:SER-S1
EN 20 0
EN 40 0
EN 60 0
EN 80 0
EN 100 0
EN 120 0
EN 140 0
EN 160 0
EN 180 0
EN 200 0
EN 220 0
EN 240 0
EN 260 0
EN 280 -11174
EN 300 0
EN 320 0
EN 340 0
EN 360 0
GTP-RIB:GTP-M5:ASN-S1
EN 20 -17115
EN 40 0
EN 60 0
EN 80 0
EN 100 0
EN 120 0
EN 140 0
EN 160 0
EN 180 0
EN 200 0
EN 220 0
EN 240 0
EN 260 0
EN 280 0
EN 300 0
EN 320 0
EN 340 0
EN 360 0
FMU-RIB:FMU-MY:MET-S1
EN 20 0
EN 40 0
EN 60 0

EN 80 0
EN 100 0
EN 120 0
EN 140 0
EN 160 0
EN 180 0
EN 200 0
EN 220 0
EN 240 0
EN 260 0
EN 280 -15398
EN 300 0
EN 320 0
EN 340 0
EN 360 0
U-RIB:U-Y:LYS-S1
EN 20 -5898
EN 40 -576
EN 60 -2455
EN 80 -3109
EN 100 -2019
EN 120 -2147
EN 140 -1872
EN 160 -2695
EN 180 -2529
EN 200 -3967
EN 220 -5040
EN 240 -3844
EN 260 -4049
EN 280 -4210
EN 300 -2889
EN 320 -3594
EN 340 -5287
EN 360 -4004
C31-RIB:C31-P:THR-S1
EN 20 0
EN 40 0
EN 60 -8772
EN 80 0
EN 100 -9241
EN 120 0
EN 140 0
EN 160 0
EN 180 0
EN 200 0
EN 220 0
EN 240 0
EN 260 0
EN 280 0
EN 300 0
EN 320 0
EN 340 0
EN 360 0
U31-RIB:U31-P:ASP-S2
EN 20 0
EN 40 0
EN 60 0
EN 80 0

EN 100 -6588
EN 120 0
EN 140 0
EN 160 0
EN 180 0
EN 200 0
EN 220 0
EN 240 0
EN 260 -7854
EN 280 0
EN 300 -7472
EN 320 0
EN 340 0
EN 360 0
DA-RIB:DA-M5:ASP-S1
EN 20 0
EN 40 0
EN 60 0
EN 80 0
EN 100 0
EN 120 0
EN 140 0
EN 160 -9692
EN 180 0
EN 200 0
EN 220 0
EN 240 0
EN 260 0
EN 280 0
EN 300 0
EN 320 0
EN 340 0
EN 360 0
FHU-RIB:FHU-P:PRO-S1
EN 20 0
EN 40 0
EN 60 0
EN 80 0
EN 100 0
EN 120 -9042
EN 140 0
EN 160 0
EN 180 0
EN 200 0
EN 220 0
EN 240 0
EN 260 0
EN 280 0
EN 300 0
EN 320 0
EN 340 0
EN 360 0
IU-P:IU-RIB:ARG-S2
EN 20 0
EN 40 0
EN 60 0
EN 80 0
EN 100 -11963

EN 120 0
EN 140 0
EN 160 0
EN 180 0
EN 200 0
EN 220 0
EN 240 0
EN 260 0
EN 280 0
EN 300 0
EN 320 0
EN 340 0
EN 360 0
G-RIB:G-P:CYS-CA
EN 20 0
EN 40 0
EN 60 0
EN 80 0
EN 100 -2838
EN 120 -2155
EN 140 -4127
EN 160 0
EN 180 0
EN 200 0
EN 220 -9692
EN 240 0
EN 260 0
EN 280 0
EN 300 0
EN 320 -3309
EN 340 0
EN 360 -6289
QUO-RIB:QUO-M5:PHE-S2
EN 20 0
EN 40 0
EN 60 0
EN 80 0
EN 100 0
EN 120 0
EN 140 0
EN 160 0
EN 180 0
EN 200 0
EN 220 0
EN 240 0
EN 260 -3434
EN 280 0
EN 300 0
EN 320 -3434
EN 340 0
EN 360 0
C-RIB:C-Y:LEU-S2
EN 20 -4262
EN 40 -4285
EN 60 -4189
EN 80 -3267
EN 100 -3944
EN 120 -3552

EN 140 -1307
EN 160 0
EN 180 -4449
EN 200 -4163
EN 220 -5198
EN 240 -4163
EN 260 -4113
EN 280 -6156
EN 300 -4282
EN 320 0
EN 340 0
EN 360 0
IU-P:IU-RIB:GLN-S2
EN 20 0
EN 40 0
EN 60 0
EN 80 0
EN 100 0
EN 120 0
EN 140 0
EN 160 0
EN 180 0
EN 200 0
EN 220 0
EN 240 0
EN 260 0
EN 280 0
EN 300 -14393
EN 320 0
EN 340 0
EN 360 0
QUO-P:QUO-RIB:LEU-CA
EN 20 0
EN 40 0
EN 60 0
EN 80 0
EN 100 0
EN 120 0
EN 140 0
EN 160 0
EN 180 0
EN 200 0
EN 220 0
EN 240 0
EN 260 -5152
EN 280 0
EN 300 0
EN 320 0
EN 340 0
EN 360 0
IU-RIB:IU-MY:ALA-CA
EN 20 0
EN 40 0
EN 60 -15398
EN 80 0
EN 100 0
EN 120 -12759
EN 140 0

EN 160 0
EN 180 0
EN 200 0
EN 220 0
EN 240 0
EN 260 0
EN 280 0
EN 300 0
EN 320 0
EN 340 0
EN 360 0
H2U-RIB:H2U-MY:LEU-S2
EN 20 0
EN 40 0
EN 60 0
EN 80 0
EN 100 0
EN 120 0
EN 140 0
EN 160 0
EN 180 0
EN 200 0
EN 220 0
EN 240 0
EN 260 0
EN 280 0
EN 300 -6970
EN 320 0
EN 340 0
EN 360 0
U-P:U-RIB:CYS-S1
EN 20 0
EN 40 0
EN 60 0
EN 80 0
EN 100 0
EN 120 -5172
EN 140 -5584
EN 160 -8606
EN 180 0
EN 200 0
EN 220 0
EN 240 0
EN 260 0
EN 280 -5152
EN 300 0
EN 320 0
EN 340 0
EN 360 0
GTP-RIB:GTP-M5:GLY-CA
EN 20 0
EN 40 0
EN 60 -8038
EN 80 0
EN 100 0
EN 120 0
EN 140 0
EN 160 0

EN 180	0
EN 200	0
EN 220	0
EN 240	0
EN 260	0
EN 280	0
EN 300	0
EN 320	0
EN 340	0
EN 360	0
U31-RIB:U31-MY:THR-CA
EN 20 0
EN 40 0
EN 60 0
EN 80 0
EN 100	0
EN 120	0
EN 140	0
EN 160	0
EN 180	-11410
EN 200	0
EN 220	0
EN 240	0
EN 260	0
EN 280	0
EN 300	0
EN 320	-8236
EN 340	0
EN 360	0
U34-P:U34-RIB:GLY-CA
EN 20 0
EN 40 0
EN 60 0
EN 80 0
EN 100	0
EN 120	-11174
EN 140	0
EN 160	0
EN 180	0
EN 200	0
EN 220	0
EN 240	0
EN 260	0
EN 280	0
EN 300	0
EN 320	0
EN 340	0
EN 360	0
IU-P:IU-RIB:ARG-S1
EN 20 0
EN 40 0
EN 60 0
EN 80 0
EN 100	0
EN 120	0
EN 140	0
EN 160	0
EN 180	0

EN 200 0
EN 220 0
EN 240 0
EN 260 0
EN 280 -9953
EN 300 0
EN 320 0
EN 340 0
EN 360 0
IU-RIB:IU-MY:SER-S1
EN 20 0
EN 40 0
EN 60 0
EN 80 -9692
EN 100 0
EN 120 -12294
EN 140 0
EN 160 0
EN 180 0
EN 200 0
EN 220 0
EN 240 0
EN 260 0
EN 280 0
EN 300 0
EN 320 0
EN 340 0
EN 360 0
FMU-P:FMU-RIB:ARG-S2
EN 20 0
EN 40 0
EN 60 0
EN 80 0
EN 100 -11963
EN 120 0
EN 140 0
EN 160 0
EN 180 0
EN 200 0
EN 220 -15398
EN 240 0
EN 260 0
EN 280 -12675
EN 300 0
EN 320 0
EN 340 0
EN 360 0
H2U-P:H2U-RIB:PHE-CA
EN 20 0
EN 40 0
EN 60 0
EN 80 0
EN 100 0
EN 120 0
EN 140 0
EN 160 0
EN 180 0
EN 200 0

EN 220 0
EN 240 0
EN 260 0
EN 280 -12675
EN 300 0
EN 320 0
EN 340 0
EN 360 0
FMU-P:FMU-RIB:VAL-S1
EN 20 0
EN 40 0
EN 60 0
EN 80 0
EN 100 0
EN 120 0
EN 140 0
EN 160 0
EN 180 0
EN 200 0
EN 220 0
EN 240 0
EN 260 0
EN 280 -13127
EN 300 0
EN 320 0
EN 340 0
EN 360 0
G-RIB:G-R5:CYS-S1
EN 20 0
EN 40 0
EN 60 0
EN 80 0
EN 100 0
EN 120 -5232
EN 140 0
EN 160 -6071
EN 180 0
EN 200 0
EN 220 -7301
EN 240 0
EN 260 0
EN 280 0
EN 300 0
EN 320 0
EN 340 0
EN 360 0
C31-RIB:C31-P:TYR-S2
EN 20 0
EN 40 0
EN 60 0
EN 80 -7739
EN 100 0
EN 120 0
EN 140 0
EN 160 0
EN 180 0
EN 200 0
EN 220 0

EN 240 0
EN 260 -7575
EN 280 0
EN 300 0
EN 320 0
EN 340 0
EN 360 0
U31-RIB:U31-MY:ILE-CA
EN 20 0
EN 40 0
EN 60 0
EN 80 0
EN 100 0
EN 120 0
EN 140 0
EN 160 0
EN 180 0
EN 200 0
EN 220 0
EN 240 0
EN 260 0
EN 280 0
EN 300 0
EN 320 0
EN 340 0
EN 360 -17115
G-RIB:G-R6:THR-S1
EN 20 0
EN 40 -1640
EN 60 -2147
EN 80 -2575
EN 100 -3552
EN 120 -2943
EN 140 -1929
EN 160 -67
EN 180 0
EN 200 0
EN 220 -4108
EN 240 -4642
EN 260 -1048
EN 280 -2303
EN 300 -3367
EN 320 -2543
EN 340 -71
EN 360 0
FMU-P:FMU-RIB:ALA-S1
EN 20 0
EN 40 0
EN 60 0
EN 80 -12294
EN 100 0
EN 120 0
EN 140 0
EN 160 0
EN 180 0
EN 200 0
EN 220 0
EN 240 0

EN 260 0
EN 280 0
EN 300 0
EN 320 0
EN 340 0
EN 360 0
IU-P:IU-RIB:HIS-CA
EN 20 0
EN 40 0
EN 60 0
EN 80 0
EN 100 0
EN 120 0
EN 140 0
EN 160 0
EN 180 0
EN 200 0
EN 220 0
EN 240 -17115
EN 260 0
EN 280 0
EN 300 0
EN 320 0
EN 340 0
EN 360 0
H2U-P:H2U-RIB:PHE-S1
EN 20 0
EN 40 0
EN 60 0
EN 80 0
EN 100 0
EN 120 0
EN 140 0
EN 160 0
EN 180 0
EN 200 0
EN 220 0
EN 240 0
EN 260 0
EN 280 -2722
EN 300 0
EN 320 0
EN 340 0
EN 360 0
FMU-RIB:FMU-MY:SER-S1
EN 20 0
EN 40 0
EN 60 0
EN 80 0
EN 100 0
EN 120 0
EN 140 -10958
EN 160 0
EN 180 0
EN 200 0
EN 220 0
EN 240 0
EN 260 0

EN 280	0
EN 300	0
EN 320	0
EN 340	0
EN 360	0
FHU-RIB:FHU-MY:ARG-S2
EN 20 0
EN 40 0
EN 60 0
EN 80 -7575
EN 100	-8528
EN 120	0
EN 140	0
EN 160	0
EN 180	0
EN 200	0
EN 220	0
EN 240	0
EN 260	-8306
EN 280	-8306
EN 300	0
EN 320	0
EN 340	0
EN 360	0
U34-RIB:U34-MY:PRO-CA
EN 20 0
EN 40 0
EN 60 0
EN 80 0
EN 100	0
EN 120	0
EN 140	0
EN 160	0
EN 180	0
EN 200	0
EN 220	0
EN 240	0
EN 260	0
EN 280	0
EN 300	-17115
EN 320	0
EN 340	0
EN 360	0
U31-RIB:U31-MY:GLU-CA
EN 20 0
EN 40 0
EN 60 0
EN 80 0
EN 100	0
EN 120	-5806
EN 140	0
EN 160	0
EN 180	0
EN 200	0
EN 220	0
EN 240	0
EN 260	0
EN 280	0

EN 300 0
EN 320 0
EN 340 0
EN 360 0
QUO-RIB:QUO-M5:ASP-S2
EN 20 0
EN 40 0
EN 60 0
EN 80 0
EN 100 0
EN 120 -3434
EN 140 0
EN 160 0
EN 180 0
EN 200 0
EN 220 0
EN 240 0
EN 260 0
EN 280 0
EN 300 0
EN 320 -17115
EN 340 0
EN 360 0
U-RIB:U-Y:TRP-CA
EN 20 0
EN 40 -6759
EN 60 0
EN 80 0
EN 100 -7834
EN 120 0
EN 140 0
EN 160 0
EN 180 0
EN 200 0
EN 220 -6772
EN 240 0
EN 260 -5416
EN 280 0
EN 300 -4673
EN 320 0
EN 340 -6672
EN 360 0
FHU-RIB:FHU-MY:ILE-CA
EN 20 0
EN 40 0
EN 60 0
EN 80 0
EN 100 0
EN 120 0
EN 140 -11963
EN 160 0
EN 180 0
EN 200 0
EN 220 0
EN 240 0
EN 260 0
EN 280 0
EN 300 0

EN 320	-12294
EN 340	0
EN 360	0
FMU-RIB:FMU-MY:ASP-S2
EN 20 0
EN 40 0
EN 60 0
EN 80 0
EN 100	0
EN 120	0
EN 140	0
EN 160	-12675
EN 180	0
EN 200	0
EN 220	0
EN 240	0
EN 260	0
EN 280	0
EN 300	0
EN 320	0
EN 340	0
EN 360	0
FMU-RIB:FMU-P:VAL-S1
EN 20 0
EN 40 0
EN 60 -2722
EN 80 0
EN 100	0
EN 120	0
EN 140	0
EN 160	0
EN 180	0
EN 200	0
EN 220	0
EN 240	0
EN 260	0
EN 280	0
EN 300	0
EN 320	0
EN 340	0
EN 360	0
C-RIB:C-P:GLU-S1
EN 20 0
EN 40 -3234
EN 60 -1582
EN 80 -720
EN 100	-659
EN 120	-1002
EN 140	45
EN 160	474
EN 180	837
EN 200	0
EN 220	-3837
EN 240	2255
EN 260	-270
EN 280	-1968
EN 300	-793
EN 320	-1211

EN 340 -1314
EN 360 -874
FMU-P:FMU-RIB:GLU-S2
EN 20 0
EN 40 0
EN 60 0
EN 80 0
EN 100 0
EN 120 0
EN 140 0
EN 160 0
EN 180 0
EN 200 0
EN 220 0
EN 240 0
EN 260 0
EN 280 0
EN 300 0
EN 320 0
EN 340 -11963
EN 360 0
DA-RIB:DA-M5:HIS-CA
EN 20 0
EN 40 0
EN 60 0
EN 80 0
EN 100 0
EN 120 0
EN 140 0
EN 160 0
EN 180 0
EN 200 0
EN 220 0
EN 240 0
EN 260 -8606
EN 280 0
EN 300 0
EN 320 0
EN 340 0
EN 360 0
U31-P:U31-RIB:TYR-S1
EN 20 0
EN 40 0
EN 60 0
EN 80 0
EN 100 0
EN 120 0
EN 140 0
EN 160 0
EN 180 0
EN 200 0
EN 220 0
EN 240 0
EN 260 0
EN 280 -13127
EN 300 0
EN 320 0
EN 340 0

EN 360 0
C-RIB:C-Y:VAL-CA
EN 20 0
EN 40 -5156
EN 60 -3354
EN 80 -4740
EN 100 -2153
EN 120 -4744
EN 140 -2633
EN 160 -1702
EN 180 0
EN 200 0
EN 220 -4351
EN 240 -4372
EN 260 -1983
EN 280 -4817
EN 300 -4443
EN 320 -2608
EN 340 0
EN 360 0
IU-RIB:IU-P:ILE-CA
EN 20 0
EN 40 0
EN 60 0
EN 80 -13127
EN 100 0
EN 120 0
EN 140 0
EN 160 0
EN 180 0
EN 200 0
EN 220 0
EN 240 0
EN 260 0
EN 280 0
EN 300 0
EN 320 0
EN 340 0
EN 360 0
OMC-RIB:OMC-MY:LYS-S2
EN 20 0
EN 40 0
EN 60 -8633
EN 80 0
EN 100 0
EN 120 0
EN 140 0
EN 160 0
EN 180 0
EN 200 0
EN 220 0
EN 240 0
EN 260 0
EN 280 0
EN 300 0
EN 320 0
EN 340 0
EN 360 0

C31-RIB:C31-MY:PHE-S1
EN 20 0
EN 40 0
EN 60 0
EN 80 0
EN 100 -9346
EN 120 0
EN 140 0
EN 160 0
EN 180 0
EN 200 0
EN 220 -12294
EN 240 0
EN 260 0
EN 280 0
EN 300 0
EN 320 0
EN 340 0
EN 360 0
H2U-RIB:H2U-P:THR-S1
EN 20 0
EN 40 0
EN 60 0
EN 80 0
EN 100 0
EN 120 0
EN 140 0
EN 160 0
EN 180 0
EN 200 0
EN 220 0
EN 240 0
EN 260 0
EN 280 -11410
EN 300 -10576
EN 320 0
EN 340 0
EN 360 0
A-RIB:A-R5:PHE-S2
EN 20 -7810
EN 40 -7134
EN 60 0
EN 80 -4964
EN 100 -4093
EN 120 -6006
EN 140 0
EN 160 0
EN 180 -7447
EN 200 -7753
EN 220 -8309
EN 240 0
EN 260 -6028
EN 280 -6662
EN 300 -4827
EN 320 0
EN 340 0
EN 360 -7683
FHU-RIB:FHU-P:ALA-CA

EN 20 0
EN 40 0
EN 60 0
EN 80 0
EN 100 0
EN 120 0
EN 140 0
EN 160 0
EN 180 0
EN 200 0
EN 220 0
EN 240 -13680
EN 260 0
EN 280 0
EN 300 0
EN 320 0
EN 340 0
EN 360 0
H2U-RIB:H2U-MY:ALA-CA
EN 20 0
EN 40 0
EN 60 0
EN 80 0
EN 100 0
EN 120 0
EN 140 -7575
EN 160 0
EN 180 0
EN 200 0
EN 220 0
EN 240 0
EN 260 0
EN 280 0
EN 300 0
EN 320 0
EN 340 0
EN 360 0
U31-P:U31-RIB:MET-CA
EN 20 0
EN 40 0
EN 60 -10760
EN 80 0
EN 100 0
EN 120 0
EN 140 0
EN 160 0
EN 180 0
EN 200 0
EN 220 0
EN 240 0
EN 260 0
EN 280 0
EN 300 0
EN 320 0
EN 340 0
EN 360 0
FMU-RIB:FMU-P:PHE-S1
EN 20 0

EN 40 0
EN 60 0
EN 80 0
EN 100 -2722
EN 120 0
EN 140 0
EN 160 0
EN 180 0
EN 200 0
EN 220 0
EN 240 0
EN 260 0
EN 280 0
EN 300 0
EN 320 0
EN 340 0
EN 360 0
H2U-RIB:H2U-P:ARG-S2
EN 20 0
EN 40 0
EN 60 0
EN 80 -8528
EN 100 0
EN 120 0
EN 140 0
EN 160 0
EN 180 0
EN 200 0
EN 220 0
EN 240 0
EN 260 0
EN 280 0
EN 300 0
EN 320 0
EN 340 0
EN 360 0
IU-RIB:IU-P:LYS-CA
EN 20 0
EN 40 0
EN 60 0
EN 80 0
EN 100 -7231
EN 120 0
EN 140 0
EN 160 0
EN 180 0
EN 200 0
EN 220 -14393
EN 240 0
EN 260 0
EN 280 0
EN 300 0
EN 320 0
EN 340 0
EN 360 0
IU-RIB:IU-MY:LEU-S2
EN 20 0
EN 40 0

EN 60 -10958
EN 80 0
EN 100 0
EN 120 0
EN 140 0
EN 160 0
EN 180 0
EN 200 0
EN 220 0
EN 240 0
EN 260 0
EN 280 0
EN 300 0
EN 320 0
EN 340 0
EN 360 0
U-RIB:U-P:GLU-S1
EN 20 0
EN 40 -1485
EN 60 1319
EN 80 -25
EN 100 1634
EN 120 -2054
EN 140 -1555
EN 160 -722
EN 180 -193
EN 200 0
EN 220 -1849
EN 240 -488
EN 260 -1308
EN 280 686
EN 300 -653
EN 320 -1486
EN 340 -2376
EN 360 0
IU-RIB:IU-MY:ILE-S1
EN 20 0
EN 40 0
EN 60 0
EN 80 0
EN 100 0
EN 120 0
EN 140 0
EN 160 0
EN 180 0
EN 200 0
EN 220 0
EN 240 -15398
EN 260 0
EN 280 0
EN 300 0
EN 320 0
EN 340 0
EN 360 0
FMU-P:FMU-RIB:ASP-S2
EN 20 0
EN 40 0
EN 60 0

EN 80 0
EN 100 0
EN 120 -12675
EN 140 0
EN 160 0
EN 180 0
EN 200 0
EN 220 0
EN 240 0
EN 260 0
EN 280 0
EN 300 0
EN 320 0
EN 340 0
EN 360 0
H2U-RIB:H2U-MY:PRO-S1
EN 20 0
EN 40 0
EN 60 0
EN 80 0
EN 100 -9190
EN 120 0
EN 140 0
EN 160 0
EN 180 0
EN 200 0
EN 220 0
EN 240 0
EN 260 0
EN 280 0
EN 300 0
EN 320 0
EN 340 0
EN 360 0
QUO-RIB:QUO-M5:LEU-CA
EN 20 0
EN 40 0
EN 60 0
EN 80 0
EN 100 0
EN 120 0
EN 140 0
EN 160 0
EN 180 0
EN 200 0
EN 220 0
EN 240 0
EN 260 0
EN 280 -3434
EN 300 0
EN 320 0
EN 340 0
EN 360 0
C31-RIB:C31-P:MET-S1
EN 20 0
EN 40 0
EN 60 0
EN 80 0

EN 100 0
EN 120 0
EN 140 0
EN 160 0
EN 180 0
EN 200 0
EN 220 0
EN 240 0
EN 260 0
EN 280 0
EN 300 0
EN 320 -10576
EN 340 0
EN 360 0
U34-RIB:U34-P:SER-CA
EN 20 0
EN 40 0
EN 60 0
EN 80 -6021
EN 100 0
EN 120 -9692
EN 140 0
EN 160 0
EN 180 0
EN 200 0
EN 220 0
EN 240 0
EN 260 0
EN 280 0
EN 300 0
EN 320 0
EN 340 0
EN 360 0
QUO-RIB:QUO-M6:ARG-S2
EN 20 0
EN 40 0
EN 60 0
EN 80 0
EN 100 0
EN 120 -3434
EN 140 0
EN 160 0
EN 180 0
EN 200 0
EN 220 0
EN 240 0
EN 260 0
EN 280 0
EN 300 0
EN 320 0
EN 340 0
EN 360 -3434
QUO-P:QUO-RIB:ASP-S1
EN 20 0
EN 40 0
EN 60 0
EN 80 0
EN 100 0

EN 120 0
EN 140 -3434
EN 160 0
EN 180 0
EN 200 0
EN 220 0
EN 240 0
EN 260 0
EN 280 0
EN 300 0
EN 320 0
EN 340 0
EN 360 0
QUO-RIB:QUO-M6:GLN-S2
EN 20 0
EN 40 0
EN 60 0
EN 80 0
EN 100 -3434
EN 120 0
EN 140 0
EN 160 0
EN 180 0
EN 200 0
EN 220 0
EN 240 0
EN 260 0
EN 280 0
EN 300 0
EN 320 0
EN 340 0
EN 360 0
U31-RIB:U31-P:ASN-S1
EN 20 0
EN 40 0
EN 60 0
EN 80 0
EN 100 -6588
EN 120 0
EN 140 0
EN 160 0
EN 180 0
EN 200 0
EN 220 0
EN 240 0
EN 260 0
EN 280 0
EN 300 0
EN 320 0
EN 340 0
EN 360 0
IU-RIB:IU-MY:VAL-CA
EN 20 0
EN 40 0
EN 60 0
EN 80 0
EN 100 -13127
EN 120 -13127

EN 140 0
EN 160 0
EN 180 0
EN 200 0
EN 220 0
EN 240 0
EN 260 0
EN 280 0
EN 300 0
EN 320 0
EN 340 0
EN 360 0
DA-RIB:DA-M6:TYR-CA
EN 20 0
EN 40 0
EN 60 0
EN 80 0
EN 100 0
EN 120 -8949
EN 140 0
EN 160 0
EN 180 0
EN 200 0
EN 220 0
EN 240 0
EN 260 0
EN 280 0
EN 300 -8688
EN 320 0
EN 340 0
EN 360 0
GTP-RIB:GTP-M5:THR-S1
EN 20 0
EN 40 0
EN 60 0
EN 80 0
EN 100 -9692
EN 120 0
EN 140 0
EN 160 0
EN 180 0
EN 200 0
EN 220 0
EN 240 0
EN 260 0
EN 280 0
EN 300 0
EN 320 0
EN 340 0
EN 360 0
C31-P:C31-RIB:ASP-CA
EN 20 0
EN 40 0
EN 60 0
EN 80 0
EN 100 0
EN 120 0
EN 140 0

EN 160 0
EN 180 0
EN 200 0
EN 220 0
EN 240 0
EN 260 0
EN 280 0
EN 300 0
EN 320 -8528
EN 340 0
EN 360 0
C31-RIB:C31-MY:GLN-S1
EN 20 0
EN 40 0
EN 60 0
EN 80 0
EN 100 0
EN 120 0
EN 140 0
EN 160 0
EN 180 0
EN 200 0
EN 220 0
EN 240 0
EN 260 0
EN 280 0
EN 300 -10245
EN 320 0
EN 340 0
EN 360 0
QUO-RIB:QUO-M5:ASN-CA
EN 20 0
EN 40 0
EN 60 0
EN 80 -3434
EN 100 0
EN 120 0
EN 140 0
EN 160 0
EN 180 0
EN 200 0
EN 220 0
EN 240 0
EN 260 0
EN 280 0
EN 300 0
EN 320 0
EN 340 0
EN 360 0
U34-RIB:U34-MY:ASP-CA
EN 20 0
EN 40 0
EN 60 0
EN 80 0
EN 100 -11410
EN 120 0
EN 140 0
EN 160 0

EN 180 0
EN 200 0
EN 220 0
EN 240 0
EN 260 0
EN 280 0
EN 300 0
EN 320 0
EN 340 0
EN 360 0
A-P:A-RIB:THR-S1
EN 20 0
EN 40 -5286
EN 60 -4438
EN 80 -2827
EN 100 -3709
EN 120 -4080
EN 140 -3610
EN 160 -3301
EN 180 -3323
EN 200 0
EN 220 -5970
EN 240 -4367
EN 260 -4952
EN 280 -3506
EN 300 -4378
EN 320 -3288
EN 340 -4372
EN 360 -1601
U34-RIB:U34-MY:ASN-S2
EN 20 0
EN 40 0
EN 60 0
EN 80 0
EN 100 0
EN 120 0
EN 140 0
EN 160 0
EN 180 0
EN 200 0
EN 220 -2722
EN 240 0
EN 260 -8236
EN 280 0
EN 300 0
EN 320 0
EN 340 0
EN 360 0
G-RIB:G-P:ALA-S1
EN 20 0
EN 40 -2048
EN 60 -2443
EN 80 -1651
EN 100 -3131
EN 120 -2191
EN 140 -3053
EN 160 -1130
EN 180 -1821

EN 200 -4405
EN 220 -2273
EN 240 -3231
EN 260 -2781
EN 280 -2508
EN 300 -2170
EN 320 -2212
EN 340 -2908
EN 360 -153
U31-RIB:U31-P:SER-CA
EN 20 0
EN 40 0
EN 60 0
EN 80 0
EN 100 0
EN 120 0
EN 140 0
EN 160 0
EN 180 0
EN 200 0
EN 220 0
EN 240 0
EN 260 0
EN 280 0
EN 300 -7186
EN 320 0
EN 340 0
EN 360 0
FHU-RIB:FHU-P:ASP-CA
EN 20 0
EN 40 0
EN 60 0
EN 80 0
EN 100 0
EN 120 0
EN 140 -9346
EN 160 -10857
EN 180 0
EN 200 0
EN 220 0
EN 240 0
EN 260 0
EN 280 0
EN 300 0
EN 320 0
EN 340 0
EN 360 0
GTP-RIB:GTP-M6:GLY-CA
EN 20 0
EN 40 0
EN 60 0
EN 80 -7683
EN 100 0
EN 120 0
EN 140 0
EN 160 0
EN 180 0
EN 200 0

EN 220	0
EN 240	0
EN 260	0
EN 280	0
EN 300	0
EN 320	0
EN 340	0
EN 360	0
GTP-RIB:GTP-M6:THR-S1
EN 20 0
EN 40 0
EN 60 0
EN 80 -9819
EN 100	0
EN 120	0
EN 140	0
EN 160	0
EN 180	0
EN 200	0
EN 220	0
EN 240	0
EN 260	0
EN 280	0
EN 300	0
EN 320	0
EN 340	0
EN 360	0
H2U-RIB:H2U-MY:ASN-S1
EN 20 0
EN 40 0
EN 60 0
EN 80 0
EN 100	0
EN 120	0
EN 140	0
EN 160	-10095
EN 180	0
EN 200	0
EN 220	0
EN 240	0
EN 260	0
EN 280	-10245
EN 300	0
EN 320	0
EN 340	0
EN 360	0
IU-RIB:IU-MY:TYR-CA
EN 20 0
EN 40 0
EN 60 0
EN 80 0
EN 100	0
EN 120	-1717
EN 140	0
EN 160	0
EN 180	0
EN 200	0
EN 220	0

EN 240	0
EN 260	0
EN 280	0
EN 300	0
EN 320	0
EN 340	0
EN 360	0
U34-RIB:U34-MY:ASP-S2
EN 20 0
EN 40 0
EN 60 0
EN 80 0
EN 100	-9953
EN 120	0
EN 140	0
EN 160	0
EN 180	0
EN 200	0
EN 220	0
EN 240	0
EN 260	0
EN 280	0
EN 300	0
EN 320	0
EN 340	0
EN 360	0
H2U-P:H2U-RIB:GLU-S2
EN 20 0
EN 40 0
EN 60 0
EN 80 0
EN 100	0
EN 120	0
EN 140	0
EN 160	0
EN 180	0
EN 200	0
EN 220	0
EN 240	0
EN 260	0
EN 280	-7914
EN 300	0
EN 320	0
EN 340	0
EN 360	0
DA-RIB:DA-M5:LYS-S1
EN 20 0
EN 40 0
EN 60 0
EN 80 0
EN 100	0
EN 120	0
EN 140	0
EN 160	0
EN 180	0
EN 200	0
EN 220	0
EN 240	0

EN 260 0
EN 280 -8772
EN 300 0
EN 320 0
EN 340 0
EN 360 0
QUO-P:QUO-RIB:ASN-CA
EN 20 0
EN 40 0
EN 60 0
EN 80 0
EN 100 0
EN 120 0
EN 140 0
EN 160 -3434
EN 180 0
EN 200 0
EN 220 0
EN 240 0
EN 260 0
EN 280 0
EN 300 0
EN 320 0
EN 340 0
EN 360 0
M2G-RIB:M2G-P:GLY-CA
EN 20 0
EN 40 0
EN 60 0
EN 80 0
EN 100 0
EN 120 0
EN 140 0
EN 160 -13680
EN 180 0
EN 200 0
EN 220 0
EN 240 0
EN 260 0
EN 280 0
EN 300 0
EN 320 0
EN 340 0
EN 360 0
FHU-P:FHU-RIB:ASP-S2
EN 20 0
EN 40 0
EN 60 0
EN 80 0
EN 100 0
EN 120 0
EN 140 0
EN 160 -15398
EN 180 0
EN 200 0
EN 220 0
EN 240 0
EN 260 0

EN 280 0
EN 300 0
EN 320 0
EN 340 -13127
EN 360 0
OMC-P:OMC-RIB:LYS-S1
EN 20 0
EN 40 0
EN 60 0
EN 80 0
EN 100 0
EN 120 0
EN 140 0
EN 160 0
EN 180 0
EN 200 0
EN 220 0
EN 240 -9346
EN 260 0
EN 280 0
EN 300 0
EN 320 0
EN 340 0
EN 360 0
H2U-RIB:H2U-MY:LYS-S1
EN 20 0
EN 40 0
EN 60 0
EN 80 0
EN 100 0
EN 120 0
EN 140 0
EN 160 0
EN 180 0
EN 200 0
EN 220 0
EN 240 0
EN 260 -8102
EN 280 0
EN 300 0
EN 320 0
EN 340 0
EN 360 0
I-P:I-RIB:TRP-S1
EN 20 0
EN 40 0
EN 60 0
EN 80 0
EN 100 0
EN 120 0
EN 140 -1717
EN 160 0
EN 180 0
EN 200 0
EN 220 0
EN 240 0
EN 260 0
EN 280 0

EN 300 0
EN 320 0
EN 340 0
EN 360 0
IU-RIB:IU-MY:LEU-CA
EN 20 0
EN 40 -1717
EN 60 0
EN 80 0
EN 100 0
EN 120 -10857
EN 140 0
EN 160 0
EN 180 0
EN 200 0
EN 220 0
EN 240 0
EN 260 0
EN 280 0
EN 300 0
EN 320 0
EN 340 0
EN 360 0
IU-RIB:IU-MY:PRO-CA
EN 20 0
EN 40 0
EN 60 0
EN 80 -12414
EN 100 0
EN 120 0
EN 140 0
EN 160 0
EN 180 0
EN 200 0
EN 220 0
EN 240 0
EN 260 0
EN 280 -10760
EN 300 0
EN 320 0
EN 340 0
EN 360 0
FMU-RIB:FMU-MY:PRO-S1
EN 20 0
EN 40 0
EN 60 0
EN 80 0
EN 100 0
EN 120 0
EN 140 -14393
EN 160 0
EN 180 0
EN 200 0
EN 220 0
EN 240 0
EN 260 0
EN 280 0
EN 300 0

EN 320	0
EN 340	0
EN 360	0
DA-RIB:DA-M5:THR-S1
EN 20 0
EN 40 0
EN 60 0
EN 80 0
EN 100	0
EN 120	0
EN 140	0
EN 160	0
EN 180	0
EN 200	0
EN 220	0
EN 240	0
EN 260	0
EN 280	0
EN 300	0
EN 320	-7854
EN 340	0
EN 360	0
QUO-RIB:QUO-M6:ARG-CA
EN 20 0
EN 40 0
EN 60 0
EN 80 0
EN 100	0
EN 120	0
EN 140	-3434
EN 160	0
EN 180	0
EN 200	0
EN 220	0
EN 240	0
EN 260	0
EN 280	0
EN 300	0
EN 320	0
EN 340	0
EN 360	0
U31-RIB:U31-P:ASN-S2
EN 20 0
EN 40 0
EN 60 0
EN 80 0
EN 100	-7186
EN 120	0
EN 140	0
EN 160	0
EN 180	0
EN 200	0
EN 220	0
EN 240	0
EN 260	0
EN 280	0
EN 300	0
EN 320	0

EN 340 0
EN 360 0
QUO-RIB:QUO-M6:LEU-S1
EN 20 0
EN 40 0
EN 60 0
EN 80 0
EN 100 0
EN 120 0
EN 140 0
EN 160 0
EN 180 0
EN 200 0
EN 220 0
EN 240 0
EN 260 -3434
EN 280 0
EN 300 0
EN 320 0
EN 340 0
EN 360 0
G-RIB:G-R5:CYS-CA
EN 20 0
EN 40 -6970
EN 60 0
EN 80 -5268
EN 100 0
EN 120 -6965
EN 140 0
EN 160 -6588
EN 180 0
EN 200 0
EN 220 0
EN 240 0
EN 260 -5132
EN 280 0
EN 300 0
EN 320 0
EN 340 0
EN 360 0
FHU-P:FHU-RIB:THR-CA
EN 20 0
EN 40 0
EN 60 0
EN 80 -10760
EN 100 0
EN 120 0
EN 140 0
EN 160 0
EN 180 0
EN 200 0
EN 220 0
EN 240 0
EN 260 -10245
EN 280 -9953
EN 300 0
EN 320 0
EN 340 0

EN 360 0
C31-RIB:C31-P:ASN-S1
EN 20 0
EN 40 0
EN 60 0
EN 80 0
EN 100 0
EN 120 0
EN 140 -8378
EN 160 0
EN 180 0
EN 200 0
EN 220 0
EN 240 0
EN 260 0
EN 280 0
EN 300 0
EN 320 0
EN 340 0
EN 360 0
DA-RIB:DA-M6:TYR-S2
EN 20 0
EN 40 0
EN 60 0
EN 80 0
EN 100 0
EN 120 -8859
EN 140 0
EN 160 0
EN 180 0
EN 200 0
EN 220 0
EN 240 0
EN 260 0
EN 280 0
EN 300 0
EN 320 0
EN 340 0
EN 360 0
IU-RIB:IU-P:LEU-S1
EN 20 0
EN 40 0
EN 60 0
EN 80 0
EN 100 0
EN 120 0
EN 140 0
EN 160 0
EN 180 0
EN 200 0
EN 220 0
EN 240 0
EN 260 0
EN 280 -11410
EN 300 0
EN 320 0
EN 340 0
EN 360 0

C31-P:C31-RIB:THR-CA
EN 20 0
EN 40 0
EN 60 0
EN 80 0
EN 100 0
EN 120 0
EN 140 0
EN 160 -10405
EN 180 0
EN 200 0
EN 220 0
EN 240 -10958
EN 260 0
EN 280 -6889
EN 300 0
EN 320 0
EN 340 0
EN 360 0
GTP-RIB:GTP-M5:SER-S1
EN 20 0
EN 40 0
EN 60 -8102
EN 80 0
EN 100 0
EN 120 0
EN 140 0
EN 160 0
EN 180 0
EN 200 0
EN 220 0
EN 240 0
EN 260 0
EN 280 0
EN 300 -9241
EN 320 0
EN 340 0
EN 360 0
G-RIB:G-R6:PRO-CA
EN 20 0
EN 40 -6279
EN 60 -4575
EN 80 -3039
EN 100 -698
EN 120 -3659
EN 140 -1782
EN 160 -1064
EN 180 0
EN 200 0
EN 220 -4500
EN 240 -2888
EN 260 -3853
EN 280 -3891
EN 300 0
EN 320 -2892
EN 340 -3790
EN 360 -3649
H2U-RIB:H2U-MY:GLN-S1

EN 20 0
EN 40 0
EN 60 0
EN 80 0
EN 100 0
EN 120 0
EN 140 0
EN 160 0
EN 180 0
EN 200 0
EN 220 0
EN 240 0
EN 260 0
EN 280 0
EN 300 0
EN 320 0
EN 340 0
EN 360 -15398
DA-RIB:DA-M5:HIS-S1
EN 20 0
EN 40 0
EN 60 0
EN 80 0
EN 100 0
EN 120 0
EN 140 0
EN 160 0
EN 180 0
EN 200 0
EN 220 0
EN 240 0
EN 260 -8306
EN 280 0
EN 300 0
EN 320 0
EN 340 0
EN 360 0
H2U-RIB:H2U-P:TRP-S2
EN 20 0
EN 40 0
EN 60 0
EN 80 0
EN 100 -15398
EN 120 0
EN 140 0
EN 160 0
EN 180 0
EN 200 0
EN 220 0
EN 240 0
EN 260 0
EN 280 0
EN 300 0
EN 320 0
EN 340 0
EN 360 0
FHU-RIB:FHU-P:SER-CA
EN 20 0

EN 40 0
EN 60 0
EN 80 0
EN 100 0
EN 120 -9241
EN 140 0
EN 160 -10958
EN 180 0
EN 200 0
EN 220 0
EN 240 0
EN 260 0
EN 280 0
EN 300 0
EN 320 0
EN 340 0
EN 360 0
U31-RIB:U31-P:ARG-CA
EN 20 0
EN 40 0
EN 60 0
EN 80 0
EN 100 0
EN 120 0
EN 140 0
EN 160 0
EN 180 0
EN 200 0
EN 220 0
EN 240 0
EN 260 0
EN 280 0
EN 300 0
EN 320 0
EN 340 -10760
EN 360 0
FHU-RIB:FHU-MY:PHE-S2
EN 20 0
EN 40 0
EN 60 0
EN 80 0
EN 100 0
EN 120 0
EN 140 0
EN 160 0
EN 180 0
EN 200 0
EN 220 0
EN 240 0
EN 260 0
EN 280 0
EN 300 0
EN 320 -2722
EN 340 0
EN 360 0
FHU-RIB:FHU-MY:GLN-S2
EN 20 0
EN 40 0

EN 60 0
EN 80 0
EN 100 -11671
EN 120 0
EN 140 0
EN 160 0
EN 180 0
EN 200 0
EN 220 0
EN 240 0
EN 260 0
EN 280 -10576
EN 300 0
EN 320 0
EN 340 0
EN 360 0
FMU-RIB:FMU-MY:PHE-S1
EN 20 0
EN 40 0
EN 60 0
EN 80 0
EN 100 0
EN 120 0
EN 140 0
EN 160 0
EN 180 0
EN 200 0
EN 220 0
EN 240 0
EN 260 -15398
EN 280 0
EN 300 0
EN 320 0
EN 340 0
EN 360 0
C31-RIB:C31-MY:PHE-CA
EN 20 0
EN 40 0
EN 60 0
EN 80 0
EN 100 -9346
EN 120 0
EN 140 0
EN 160 0
EN 180 0
EN 200 0
EN 220 0
EN 240 0
EN 260 0
EN 280 0
EN 300 0
EN 320 0
EN 340 0
EN 360 0
C31-RIB:C31-P:ASN-CA
EN 20 0
EN 40 0
EN 60 0

EN 80 0
EN 100 0
EN 120 0
EN 140 -8528
EN 160 0
EN 180 0
EN 200 0
EN 220 0
EN 240 0
EN 260 0
EN 280 0
EN 300 0
EN 320 0
EN 340 0
EN 360 0
QUO-P:QUO-RIB:LEU-S2
EN 20 0
EN 40 0
EN 60 0
EN 80 0
EN 100 0
EN 120 0
EN 140 0
EN 160 0
EN 180 0
EN 200 0
EN 220 0
EN 240 0
EN 260 -18832
EN 280 0
EN 300 0
EN 320 0
EN 340 0
EN 360 0
4SU-RIB:4SU-P:THR-S1
EN 20 0
EN 40 0
EN 60 0
EN 80 0
EN 100 0
EN 120 0
EN 140 0
EN 160 -2722
EN 180 0
EN 200 0
EN 220 0
EN 240 0
EN 260 0
EN 280 0
EN 300 0
EN 320 0
EN 340 0
EN 360 0
C31-RIB:C31-P:ASP-S1
EN 20 0
EN 40 0
EN 60 0
EN 80 0

EN 100 0
EN 120 -7975
EN 140 -7914
EN 160 0
EN 180 0
EN 200 0
EN 220 0
EN 240 0
EN 260 0
EN 280 0
EN 300 0
EN 320 -8859
EN 340 0
EN 360 0
4SU-RIB:4SU-P:GLU-S2
EN 20 0
EN 40 0
EN 60 0
EN 80 0
EN 100 0
EN 120 0
EN 140 0
EN 160 0
EN 180 0
EN 200 0
EN 220 0
EN 240 0
EN 260 0
EN 280 0
EN 300 0
EN 320 0
EN 340 -2722
EN 360 0
H2U-RIB:H2U-P:PHE-S2
EN 20 0
EN 40 0
EN 60 0
EN 80 -17115
EN 100 0
EN 120 0
EN 140 0
EN 160 0
EN 180 0
EN 200 0
EN 220 0
EN 240 0
EN 260 0
EN 280 0
EN 300 0
EN 320 0
EN 340 0
EN 360 0
QUO-P:QUO-RIB:LYS-S2
EN 20 0
EN 40 0
EN 60 0
EN 80 0
EN 100 0

EN 120 -3434
EN 140 0
EN 160 0
EN 180 0
EN 200 0
EN 220 0
EN 240 0
EN 260 0
EN 280 0
EN 300 0
EN 320 0
EN 340 0
EN 360 0
H2U-RIB:H2U-MY:TRP-S2
EN 20 0
EN 40 0
EN 60 0
EN 80 0
EN 100 0
EN 120 0
EN 140 0
EN 160 0
EN 180 0
EN 200 0
EN 220 0
EN 240 0
EN 260 0
EN 280 0
EN 300 -13127
EN 320 0
EN 340 0
EN 360 0
QUO-RIB:QUO-M6:ASP-CA
EN 20 0
EN 40 0
EN 60 0
EN 80 0
EN 100 0
EN 120 -3434
EN 140 0
EN 160 0
EN 180 0
EN 200 0
EN 220 0
EN 240 0
EN 260 0
EN 280 0
EN 300 0
EN 320 0
EN 340 0
EN 360 0
DA-RIB:DA-M5:ASP-S2
EN 20 0
EN 40 0
EN 60 0
EN 80 0
EN 100 0
EN 120 0

EN 140 0
EN 160 -9346
EN 180 0
EN 200 0
EN 220 0
EN 240 0
EN 260 0
EN 280 0
EN 300 0
EN 320 0
EN 340 0
EN 360 0
M2G-RIB:M2G-P:SER-S1
EN 20 0
EN 40 0
EN 60 0
EN 80 0
EN 100 0
EN 120 0
EN 140 0
EN 160 0
EN 180 0
EN 200 0
EN 220 0
EN 240 0
EN 260 0
EN 280 0
EN 300 0
EN 320 -13680
EN 340 0
EN 360 0
FMU-RIB:FMU-P:ASP-S2
EN 20 0
EN 40 0
EN 60 0
EN 80 0
EN 100 0
EN 120 0
EN 140 0
EN 160 -14393
EN 180 0
EN 200 0
EN 220 0
EN 240 0
EN 260 0
EN 280 0
EN 300 0
EN 320 0
EN 340 0
EN 360 0
C31-P:C31-RIB:GLU-S1
EN 20 0
EN 40 0
EN 60 0
EN 80 -7231
EN 100 0
EN 120 0
EN 140 0

EN 160 0
EN 180 0
EN 200 0
EN 220 0
EN 240 0
EN 260 0
EN 280 0
EN 300 0
EN 320 0
EN 340 0
EN 360 0
U-P:U-RIB:TRP-S2
EN 20 0
EN 40 0
EN 60 -4375
EN 80 -6186
EN 100 0
EN 120 -5832
EN 140 -3271
EN 160 -3774
EN 180 0
EN 200 0
EN 220 0
EN 240 -4012
EN 260 -6840
EN 280 -5064
EN 300 0
EN 320 0
EN 340 -4025
EN 360 -6107
U34-P:U34-RIB:SER-S1
EN 20 0
EN 40 0
EN 60 0
EN 80 -6519
EN 100 0
EN 120 0
EN 140 0
EN 160 0
EN 180 0
EN 200 0
EN 220 0
EN 240 0
EN 260 0
EN 280 -6451
EN 300 0
EN 320 0
EN 340 0
EN 360 0
QUO-RIB:QUO-P:SER-S1
EN 20 0
EN 40 0
EN 60 0
EN 80 0
EN 100 0
EN 120 -3434
EN 140 0
EN 160 0

EN 180 0
EN 200 0
EN 220 0
EN 240 0
EN 260 0
EN 280 0
EN 300 0
EN 320 0
EN 340 0
EN 360 0
IU-P:IU-RIB:LYS-CA
EN 20 0
EN 40 0
EN 60 0
EN 80 0
EN 100 0
EN 120 -8688
EN 140 0
EN 160 0
EN 180 0
EN 200 0
EN 220 0
EN 240 -9692
EN 260 0
EN 280 0
EN 300 0
EN 320 0
EN 340 0
EN 360 0
FHU-P:FHU-RIB:LYS-CA
EN 20 0
EN 40 0
EN 60 0
EN 80 0
EN 100 0
EN 120 0
EN 140 0
EN 160 0
EN 180 0
EN 200 0
EN 220 -13680
EN 240 0
EN 260 0
EN 280 0
EN 300 0
EN 320 0
EN 340 0
EN 360 0
C31-RIB:C31-MY:PHE-S2
EN 20 0
EN 40 0
EN 60 0
EN 80 0
EN 100 0
EN 120 0
EN 140 0
EN 160 0
EN 180 0

EN 200	0
EN 220	0
EN 240	0
EN 260	-10576
EN 280	0
EN 300	0
EN 320	0
EN 340	0
EN 360	0
C31-RIB:C31-MY:TYR-S2
EN 20 0
EN 40 0
EN 60 -11812
EN 80 0
EN 100	0
EN 120	0
EN 140	0
EN 160	0
EN 180	0
EN 200	0
EN 220	0
EN 240	0
EN 260	0
EN 280	0
EN 300	0
EN 320	0
EN 340	0
EN 360	0
C31-RIB:C31-MY:LEU-S1
EN 20 0
EN 40 0
EN 60 0
EN 80 0
EN 100	0
EN 120	0
EN 140	0
EN 160	0
EN 180	0
EN 200	0
EN 220	0
EN 240	0
EN 260	0
EN 280	-7575
EN 300	0
EN 320	0
EN 340	0
EN 360	0
5BU-P:5BU-RIB:PRO-CA
EN 20 0
EN 40 -13680
EN 60 0
EN 80 0
EN 100	0
EN 120	0
EN 140	0
EN 160	0
EN 180	0
EN 200	0

EN 220	0
EN 240	0
EN 260	0
EN 280	0
EN 300	0
EN 320	0
EN 340	0
EN 360	0
DA-RIB:DA-M6:TYR-S1
EN 20 0
EN 40 0
EN 60 0
EN 80 0
EN 100	-8688
EN 120	0
EN 140	0
EN 160	0
EN 180	0
EN 200	0
EN 220	0
EN 240	0
EN 260	0
EN 280	0
EN 300	-9346
EN 320	0
EN 340	0
EN 360	0
FMU-P:FMU-RIB:MET-S2
EN 20 0
EN 40 0
EN 60 0
EN 80 0
EN 100	0
EN 120	0
EN 140	0
EN 160	0
EN 180	0
EN 200	0
EN 220	0
EN 240	0
EN 260	0
EN 280	0
EN 300	0
EN 320	-2722
EN 340	0
EN 360	0
QUO-P:QUO-RIB:ASN-S1
EN 20 0
EN 40 0
EN 60 0
EN 80 0
EN 100	0
EN 120	0
EN 140	0
EN 160	-3434
EN 180	0
EN 200	0
EN 220	0

EN 240 0
EN 260 0
EN 280 0
EN 300 0
EN 320 0
EN 340 0
EN 360 0
I-P:I-RIB:ALA-S1
EN 20 0
EN 40 0
EN 60 0
EN 80 0
EN 100 0
EN 120 0
EN 140 0
EN 160 -1717
EN 180 0
EN 200 0
EN 220 0
EN 240 0
EN 260 0
EN 280 0
EN 300 0
EN 320 0
EN 340 0
EN 360 0
FHU-RIB:FHU-P:PRO-CA
EN 20 0
EN 40 0
EN 60 0
EN 80 0
EN 100 0
EN 120 -9571
EN 140 0
EN 160 0
EN 180 0
EN 200 0
EN 220 0
EN 240 0
EN 260 0
EN 280 0
EN 300 0
EN 320 0
EN 340 0
EN 360 0
U34-RIB:U34-MY:ASN-S1
EN 20 0
EN 40 0
EN 60 0
EN 80 0
EN 100 0
EN 120 0
EN 140 0
EN 160 0
EN 180 0
EN 200 0
EN 220 0
EN 240 -15398

EN 260 -10576
EN 280 0
EN 300 0
EN 320 0
EN 340 0
EN 360 0
FMU-RIB:FMU-P:ILE-CA
EN 20 0
EN 40 0
EN 60 0
EN 80 0
EN 100 -2722
EN 120 0
EN 140 0
EN 160 0
EN 180 0
EN 200 0
EN 220 0
EN 240 0
EN 260 0
EN 280 0
EN 300 0
EN 320 0
EN 340 0
EN 360 0
U31-RIB:U31-P:ASP-CA
EN 20 0
EN 40 0
EN 60 0
EN 80 0
EN 100 0
EN 120 0
EN 140 0
EN 160 0
EN 180 0
EN 200 0
EN 220 0
EN 240 0
EN 260 -7975
EN 280 0
EN 300 -8102
EN 320 0
EN 340 0
EN 360 0
U31-RIB:U31-P:LEU-S2
EN 20 0
EN 40 0
EN 60 0
EN 80 0
EN 100 0
EN 120 0
EN 140 0
EN 160 0
EN 180 0
EN 200 0
EN 220 0
EN 240 0
EN 260 0

EN 280 0
EN 300 -8378
EN 320 0
EN 340 0
EN 360 0
FHU-RIB:FHU-P:VAL-S1
EN 20 0
EN 40 0
EN 60 0
EN 80 0
EN 100 0
EN 120 -9139
EN 140 0
EN 160 0
EN 180 0
EN 200 0
EN 220 0
EN 240 0
EN 260 0
EN 280 0
EN 300 0
EN 320 -9346
EN 340 0
EN 360 0
U34-RIB:U34-MY:VAL-CA
EN 20 0
EN 40 0
EN 60 0
EN 80 0
EN 100 0
EN 120 0
EN 140 0
EN 160 0
EN 180 0
EN 200 0
EN 220 0
EN 240 0
EN 260 0
EN 280 0
EN 300 0
EN 320 0
EN 340 -15398
EN 360 0
U31-RIB:U31-P:SER-S1
EN 20 0
EN 40 0
EN 60 0
EN 80 0
EN 100 0
EN 120 0
EN 140 0
EN 160 0
EN 180 0
EN 200 0
EN 220 0
EN 240 0
EN 260 0
EN 280 0

EN 300 -7422
EN 320 0
EN 340 0
EN 360 0
DA-RIB:DA-M6:LEU-S1
EN 20 0
EN 40 0
EN 60 0
EN 80 0
EN 100 0
EN 120 -7683
EN 140 0
EN 160 0
EN 180 0
EN 200 0
EN 220 0
EN 240 0
EN 260 0
EN 280 0
EN 300 -6889
EN 320 0
EN 340 0
EN 360 0
DA-RIB:DA-M6:HIS-S2
EN 20 0
EN 40 0
EN 60 0
EN 80 0
EN 100 0
EN 120 0
EN 140 0
EN 160 0
EN 180 0
EN 200 0
EN 220 0
EN 240 0
EN 260 -8038
EN 280 0
EN 300 0
EN 320 0
EN 340 0
EN 360 0
U31-RIB:U31-MY:VAL-CA
EN 20 0
EN 40 0
EN 60 0
EN 80 0
EN 100 0
EN 120 -7422
EN 140 0
EN 160 0
EN 180 0
EN 200 0
EN 220 0
EN 240 0
EN 260 0
EN 280 0
EN 300 0

EN 320 0
EN 340 0
EN 360 0
FHU-RIB:FHU-P:ASP-S1
EN 20 0
EN 40 0
EN 60 0
EN 80 0
EN 100 0
EN 120 0
EN 140 0
EN 160 -8452
EN 180 0
EN 200 0
EN 220 0
EN 240 0
EN 260 0
EN 280 0
EN 300 0
EN 320 0
EN 340 0
EN 360 0
H2U-P:H2U-RIB:PRO-CA
EN 20 0
EN 40 0
EN 60 0
EN 80 0
EN 100 0
EN 120 0
EN 140 0
EN 160 0
EN 180 0
EN 200 0
EN 220 0
EN 240 0
EN 260 0
EN 280 0
EN 300 -9456
EN 320 0
EN 340 0
EN 360 0
U34-RIB:U34-MY:ASN-CA
EN 20 0
EN 40 0
EN 60 0
EN 80 0
EN 100 0
EN 120 0
EN 140 0
EN 160 0
EN 180 0
EN 200 0
EN 220 0
EN 240 -17115
EN 260 0
EN 280 0
EN 300 0
EN 320 0

EN 340 0
EN 360 0
IU-RIB:IU-MY:MET-CA
EN 20 0
EN 40 0
EN 60 0
EN 80 0
EN 100 0
EN 120 0
EN 140 0
EN 160 -17115
EN 180 0
EN 200 0
EN 220 0
EN 240 0
EN 260 0
EN 280 0
EN 300 0
EN 320 0
EN 340 0
EN 360 0
I-P:I-RIB:ALA-CA
EN 20 0
EN 40 0
EN 60 0
EN 80 0
EN 100 0
EN 120 0
EN 140 0
EN 160 -1717
EN 180 0
EN 200 0
EN 220 0
EN 240 0
EN 260 0
EN 280 0
EN 300 0
EN 320 0
EN 340 0
EN 360 0
M2G-RIB:M2G-P:GLU-S2
EN 20 0
EN 40 0
EN 60 0
EN 80 0
EN 100 0
EN 120 0
EN 140 0
EN 160 0
EN 180 0
EN 200 0
EN 220 0
EN 240 0
EN 260 0
EN 280 0
EN 300 0
EN 320 0
EN 340 -13127

EN 360 0
GTP-RIB:GTP-M6:ASN-S1
EN 20 0
EN 40 -11671
EN 60 0
EN 80 0
EN 100 0
EN 120 0
EN 140 0
EN 160 0
EN 180 0
EN 200 0
EN 220 0
EN 240 0
EN 260 0
EN 280 0
EN 300 0
EN 320 0
EN 340 0
EN 360 0
IU-P:IU-RIB:HIS-S1
EN 20 0
EN 40 0
EN 60 0
EN 80 0
EN 100 0
EN 120 0
EN 140 0
EN 160 0
EN 180 0
EN 200 0
EN 220 0
EN 240 -15398
EN 260 0
EN 280 0
EN 300 0
EN 320 0
EN 340 0
EN 360 0
C31-P:C31-RIB:THR-S1
EN 20 0
EN 40 0
EN 60 0
EN 80 0
EN 100 0
EN 120 0
EN 140 0
EN 160 0
EN 180 0
EN 200 0
EN 220 0
EN 240 0
EN 260 0
EN 280 -6810
EN 300 0
EN 320 0
EN 340 0
EN 360 0

H2U-P:H2U-RIB:ARG-S2
EN 20 0
EN 40 0
EN 60 0
EN 80 0
EN 100 0
EN 120 0
EN 140 0
EN 160 0
EN 180 0
EN 200 0
EN 220 0
EN 240 0
EN 260 -8102
EN 280 0
EN 300 0
EN 320 -8528
EN 340 0
EN 360 0
U31-P:U31-RIB:THR-S1
EN 20 0
EN 40 0
EN 60 0
EN 80 0
EN 100 -10958
EN 120 -10760
EN 140 0
EN 160 0
EN 180 0
EN 200 0
EN 220 0
EN 240 0
EN 260 0
EN 280 0
EN 300 0
EN 320 0
EN 340 0
EN 360 0
U31-RIB:U31-MY:GLN-CA
EN 20 0
EN 40 0
EN 60 -11410
EN 80 0
EN 100 0
EN 120 0
EN 140 0
EN 160 0
EN 180 0
EN 200 0
EN 220 0
EN 240 0
EN 260 0
EN 280 0
EN 300 0
EN 320 0
EN 340 0
EN 360 0
U31-P:U31-RIB:GLU-S2

EN 20 0
EN 40 0
EN 60 0
EN 80 0
EN 100 0
EN 120 0
EN 140 0
EN 160 0
EN 180 0
EN 200 0
EN 220 0
EN 240 0
EN 260 0
EN 280 0
EN 300 0
EN 320 0
EN 340 0
EN 360 -10405
FMU-RIB:FMU-P:PHE-S2
EN 20 0
EN 40 0
EN 60 0
EN 80 -2722
EN 100 0
EN 120 0
EN 140 0
EN 160 0
EN 180 0
EN 200 0
EN 220 0
EN 240 0
EN 260 0
EN 280 0
EN 300 0
EN 320 0
EN 340 0
EN 360 0
U31-RIB:U31-MY:PHE-CA
EN 20 0
EN 40 0
EN 60 0
EN 80 0
EN 100 0
EN 120 0
EN 140 0
EN 160 0
EN 180 0
EN 200 0
EN 220 0
EN 240 0
EN 260 0
EN 280 0
EN 300 -9241
EN 320 0
EN 340 0
EN 360 0
QUO-RIB:QUO-P:ASN-S2
EN 20 0

EN 40 0
EN 60 0
EN 80 0
EN 100 0
EN 120 0
EN 140 0
EN 160 0
EN 180 0
EN 200 -3434
EN 220 0
EN 240 0
EN 260 0
EN 280 0
EN 300 0
EN 320 0
EN 340 0
EN 360 0
IU-RIB:IU-MY:THR-CA
EN 20 0
EN 40 0
EN 60 0
EN 80 0
EN 100 0
EN 120 -12294
EN 140 -10169
EN 160 0
EN 180 0
EN 200 0
EN 220 0
EN 240 0
EN 260 0
EN 280 0
EN 300 0
EN 320 0
EN 340 0
EN 360 0
5BU-RIB:5BU-MY:ILE-S1
EN 20 0
EN 40 0
EN 60 0
EN 80 0
EN 100 0
EN 120 0
EN 140 0
EN 160 0
EN 180 0
EN 200 0
EN 220 0
EN 240 0
EN 260 0
EN 280 0
EN 300 -2722
EN 320 0
EN 340 0
EN 360 0
FMU-P:FMU-RIB:GLN-S1
EN 20 0
EN 40 0

EN 60 0
EN 80 0
EN 100 0
EN 120 0
EN 140 0
EN 160 0
EN 180 0
EN 200 0
EN 220 0
EN 240 0
EN 260 0
EN 280 -17115
EN 300 0
EN 320 0
EN 340 0
EN 360 0
DA-RIB:DA-M6:MET-S2
EN 20 0
EN 40 0
EN 60 0
EN 80 0
EN 100 0
EN 120 0
EN 140 0
EN 160 0
EN 180 0
EN 200 0
EN 220 0
EN 240 -11410
EN 260 0
EN 280 0
EN 300 0
EN 320 0
EN 340 0
EN 360 0
C31-P:C31-RIB:LEU-S1
EN 20 0
EN 40 0
EN 60 0
EN 80 0
EN 100 0
EN 120 0
EN 140 0
EN 160 0
EN 180 0
EN 200 0
EN 220 0
EN 240 0
EN 260 0
EN 280 0
EN 300 0
EN 320 -8949
EN 340 0
EN 360 0
DA-RIB:DA-M5:LEU-S1
EN 20 0
EN 40 0
EN 60 0

EN 80 0
EN 100 0
EN 120 -7575
EN 140 0
EN 160 0
EN 180 0
EN 200 0
EN 220 0
EN 240 0
EN 260 0
EN 280 0
EN 300 0
EN 320 0
EN 340 0
EN 360 0
DA-RIB:DA-M5:GLN-CA
EN 20 0
EN 40 0
EN 60 0
EN 80 0
EN 100 0
EN 120 0
EN 140 0
EN 160 0
EN 180 0
EN 200 0
EN 220 0
EN 240 0
EN 260 -8688
EN 280 0
EN 300 0
EN 320 0
EN 340 0
EN 360 0
C31-RIB:C31-P:GLU-S2
EN 20 0
EN 40 0
EN 60 0
EN 80 0
EN 100 0
EN 120 0
EN 140 0
EN 160 0
EN 180 0
EN 200 0
EN 220 0
EN 240 0
EN 260 -6196
EN 280 0
EN 300 0
EN 320 0
EN 340 0
EN 360 0
FMU-RIB:FMU-MY:PHE-CA
EN 20 0
EN 40 0
EN 60 0
EN 80 0

EN 100 0
EN 120 0
EN 140 0
EN 160 0
EN 180 0
EN 200 0
EN 220 0
EN 240 0
EN 260 -17115
EN 280 0
EN 300 0
EN 320 0
EN 340 0
EN 360 0
FMU-RIB:FMU-MY:GLU-S1
EN 20 0
EN 40 0
EN 60 0
EN 80 0
EN 100 0
EN 120 0
EN 140 0
EN 160 0
EN 180 0
EN 200 0
EN 220 0
EN 240 0
EN 260 0
EN 280 0
EN 300 -10760
EN 320 0
EN 340 0
EN 360 0
H2U-RIB:H2U-MY:PHE-CA
EN 20 0
EN 40 0
EN 60 0
EN 80 0
EN 100 0
EN 120 0
EN 140 -10760
EN 160 0
EN 180 0
EN 200 0
EN 220 0
EN 240 0
EN 260 0
EN 280 0
EN 300 0
EN 320 0
EN 340 0
EN 360 0
QUO-RIB:QUO-M5:PHE-S1
EN 20 0
EN 40 0
EN 60 0
EN 80 0
EN 100 0

EN 120 0
EN 140 0
EN 160 0
EN 180 0
EN 200 0
EN 220 0
EN 240 0
EN 260 -3434
EN 280 0
EN 300 0
EN 320 -3434
EN 340 0
EN 360 0
DA-RIB:DA-M5:HIS-S2
EN 20 0
EN 40 0
EN 60 0
EN 80 0
EN 100 0
EN 120 0
EN 140 0
EN 160 0
EN 180 0
EN 200 0
EN 220 0
EN 240 0
EN 260 0
EN 280 -7739
EN 300 0
EN 320 0
EN 340 0
EN 360 0
QUO-RIB:QUO-M5:LEU-S1
EN 20 0
EN 40 0
EN 60 0
EN 80 0
EN 100 0
EN 120 0
EN 140 0
EN 160 0
EN 180 0
EN 200 0
EN 220 0
EN 240 -3434
EN 260 0
EN 280 -3434
EN 300 0
EN 320 0
EN 340 0
EN 360 0
IU-P:IU-RIB:ALA-CA
EN 20 0
EN 40 0
EN 60 0
EN 80 0
EN 100 0
EN 120 0

EN 140 0
EN 160 0
EN 180 0
EN 200 0
EN 220 0
EN 240 0
EN 260 0
EN 280 -11963
EN 300 0
EN 320 0
EN 340 0
EN 360 0
FMU-RIB:FMU-MY:ARG-S2
EN 20 0
EN 40 -2722
EN 60 0
EN 80 0
EN 100 0
EN 120 0
EN 140 0
EN 160 0
EN 180 0
EN 200 0
EN 220 0
EN 240 0
EN 260 0
EN 280 0
EN 300 0
EN 320 0
EN 340 0
EN 360 0
C31-RIB:C31-MY:ALA-CA
EN 20 0
EN 40 0
EN 60 0
EN 80 0
EN 100 0
EN 120 0
EN 140 0
EN 160 0
EN 180 0
EN 200 0
EN 220 0
EN 240 0
EN 260 0
EN 280 -9241
EN 300 0
EN 320 0
EN 340 0
EN 360 0
QUO-RIB:QUO-M5:ARG-S2
EN 20 0
EN 40 0
EN 60 0
EN 80 0
EN 100 -3434
EN 120 0
EN 140 0

EN 160 0
EN 180 -3434
EN 200 0
EN 220 0
EN 240 0
EN 260 0
EN 280 0
EN 300 0
EN 320 0
EN 340 0
EN 360 0
QUO-P:QUO-RIB:LEU-S1
EN 20 0
EN 40 0
EN 60 0
EN 80 0
EN 100 0
EN 120 0
EN 140 0
EN 160 0
EN 180 0
EN 200 0
EN 220 0
EN 240 0
EN 260 -18832
EN 280 0
EN 300 0
EN 320 0
EN 340 0
EN 360 0
5BU-P:5BU-RIB:SER-S1
EN 20 0
EN 40 0
EN 60 -14393
EN 80 0
EN 100 0
EN 120 0
EN 140 0
EN 160 0
EN 180 0
EN 200 0
EN 220 0
EN 240 0
EN 260 0
EN 280 0
EN 300 0
EN 320 0
EN 340 0
EN 360 0
U31-RIB:U31-MY:TYR-S2
EN 20 0
EN 40 0
EN 60 0
EN 80 0
EN 100 0
EN 120 0
EN 140 0
EN 160 0

EN 180 0
EN 200 0
EN 220 0
EN 240 0
EN 260 0
EN 280 -7054
EN 300 0
EN 320 0
EN 340 0
EN 360 0
U31-RIB:U31-MY:ILE-S1
EN 20 0
EN 40 0
EN 60 0
EN 80 0
EN 100 0
EN 120 0
EN 140 0
EN 160 0
EN 180 0
EN 200 0
EN 220 0
EN 240 0
EN 260 0
EN 280 0
EN 300 0
EN 320 0
EN 340 -10576
EN 360 0
U31-P:U31-RIB:GLN-CA
EN 20 0
EN 40 0
EN 60 0
EN 80 -10245
EN 100 0
EN 120 0
EN 140 0
EN 160 0
EN 180 0
EN 200 0
EN 220 0
EN 240 0
EN 260 0
EN 280 0
EN 300 0
EN 320 0
EN 340 0
EN 360 0
QUO-RIB:QUO-M6:ASN-S1
EN 20 0
EN 40 0
EN 60 0
EN 80 -3434
EN 100 0
EN 120 0
EN 140 0
EN 160 0
EN 180 0

EN 200 0
EN 220 0
EN 240 0
EN 260 0
EN 280 0
EN 300 0
EN 320 0
EN 340 0
EN 360 0
FMU-RIB:FMU-MY:ARG-CA
EN 20 0
EN 40 0
EN 60 0
EN 80 -12675
EN 100 0
EN 120 0
EN 140 0
EN 160 0
EN 180 0
EN 200 0
EN 220 0
EN 240 0
EN 260 0
EN 280 0
EN 300 0
EN 320 0
EN 340 0
EN 360 0
C31-RIB:C31-MY:GLU-CA
EN 20 0
EN 40 0
EN 60 0
EN 80 0
EN 100 0
EN 120 -7796
EN 140 0
EN 160 0
EN 180 0
EN 200 0
EN 220 0
EN 240 0
EN 260 0
EN 280 0
EN 300 0
EN 320 0
EN 340 0
EN 360 0
FHU-RIB:FHU-P:SER-S1
EN 20 0
EN 40 0
EN 60 0
EN 80 0
EN 100 0
EN 120 0
EN 140 0
EN 160 0
EN 180 0
EN 200 0

EN 220	0
EN 240	0
EN 260	0
EN 280	0
EN 300	0
EN 320	0
EN 340	-13127
EN 360	0
IU-RIB:IU-P:ASP-S2
EN 20 0
EN 40 0
EN 60 0
EN 80 0
EN 100	0
EN 120	0
EN 140	-10095
EN 160	0
EN 180	0
EN 200	0
EN 220	0
EN 240	0
EN 260	0
EN 280	0
EN 300	0
EN 320	0
EN 340	0
EN 360	0
QUO-RIB:QUO-M5:ARG-CA
EN 20 0
EN 40 0
EN 60 0
EN 80 0
EN 100	0
EN 120	-3434
EN 140	0
EN 160	0
EN 180	0
EN 200	0
EN 220	0
EN 240	0
EN 260	0
EN 280	0
EN 300	0
EN 320	0
EN 340	0
EN 360	0
5BU-RIB:5BU-P:SER-CA
EN 20 0
EN 40 0
EN 60 0
EN 80 0
EN 100	0
EN 120	0
EN 140	0
EN 160	0
EN 180	0
EN 200	0
EN 220	0

EN 240 0
EN 260 0
EN 280 0
EN 300 -11410
EN 320 0
EN 340 0
EN 360 0
C31-RIB:C31-MY:ASP-CA
EN 20 0
EN 40 0
EN 60 0
EN 80 0
EN 100 0
EN 120 0
EN 140 0
EN 160 0
EN 180 0
EN 200 0
EN 220 0
EN 240 -8859
EN 260 0
EN 280 0
EN 300 0
EN 320 0
EN 340 0
EN 360 0
IU-P:IU-RIB:LYS-S1
EN 20 0
EN 40 0
EN 60 0
EN 80 0
EN 100 0
EN 120 0
EN 140 0
EN 160 0
EN 180 0
EN 200 0
EN 220 0
EN 240 -9139
EN 260 0
EN 280 0
EN 300 0
EN 320 0
EN 340 -9692
EN 360 0
H2U-P:H2U-RIB:LYS-S1
EN 20 0
EN 40 0
EN 60 0
EN 80 0
EN 100 0
EN 120 0
EN 140 0
EN 160 0
EN 180 0
EN 200 0
EN 220 0
EN 240 0

EN 260 0
EN 280 -8772
EN 300 0
EN 320 0
EN 340 0
EN 360 0
IU-RIB:IU-P:ALA-CA
EN 20 0
EN 40 0
EN 60 -14393
EN 80 0
EN 100 0
EN 120 0
EN 140 0
EN 160 0
EN 180 0
EN 200 0
EN 220 0
EN 240 0
EN 260 0
EN 280 0
EN 300 0
EN 320 0
EN 340 0
EN 360 0
DA-RIB:DA-M6:THR-CA
EN 20 0
EN 40 0
EN 60 0
EN 80 0
EN 100 0
EN 120 0
EN 140 0
EN 160 0
EN 180 0
EN 200 0
EN 220 0
EN 240 0
EN 260 0
EN 280 0
EN 300 -7325
EN 320 0
EN 340 -9042
EN 360 0
I-P:I-RIB:TRP-S2
EN 20 0
EN 40 0
EN 60 0
EN 80 0
EN 100 0
EN 120 -1717
EN 140 0
EN 160 0
EN 180 0
EN 200 0
EN 220 0
EN 240 0
EN 260 0

EN 280 0
EN 300 0
EN 320 0
EN 340 0
EN 360 0
C31-RIB:C31-P:PHE-S2
EN 20 0
EN 40 0
EN 60 0
EN 80 0
EN 100 0
EN 120 0
EN 140 0
EN 160 0
EN 180 0
EN 200 0
EN 220 -10576
EN 240 -10245
EN 260 0
EN 280 0
EN 300 0
EN 320 0
EN 340 0
EN 360 0
U34-RIB:U34-P:HIS-S1
EN 20 0
EN 40 0
EN 60 0
EN 80 0
EN 100 0
EN 120 0
EN 140 0
EN 160 -14393
EN 180 0
EN 200 0
EN 220 0
EN 240 0
EN 260 0
EN 280 0
EN 300 0
EN 320 0
EN 340 0
EN 360 0
C31-RIB:C31-MY:SER-S1
EN 20 0
EN 40 0
EN 60 0
EN 80 0
EN 100 0
EN 120 0
EN 140 0
EN 160 0
EN 180 0
EN 200 0
EN 220 0
EN 240 0
EN 260 -7739
EN 280 0

EN 300	0
EN 320	0
EN 340	0
EN 360	0
FMU-RIB:FMU-MY:ILE-CA
EN 20 0
EN 40 0
EN 60 0
EN 80 0
EN 100	0
EN 120	0
EN 140	0
EN 160	0
EN 180	0
EN 200	0
EN 220	0
EN 240	0
EN 260	0
EN 280	-15398
EN 300	0
EN 320	0
EN 340	0
EN 360	0
FMU-P:FMU-RIB:ARG-CA
EN 20 0
EN 40 0
EN 60 0
EN 80 0
EN 100	0
EN 120	-17115
EN 140	0
EN 160	0
EN 180	0
EN 200	0
EN 220	0
EN 240	0
EN 260	0
EN 280	0
EN 300	0
EN 320	0
EN 340	0
EN 360	0
C31-RIB:C31-P:ASN-S2
EN 20 0
EN 40 0
EN 60 0
EN 80 0
EN 100	0
EN 120	-8528
EN 140	0
EN 160	0
EN 180	0
EN 200	0
EN 220	0
EN 240	0
EN 260	0
EN 280	0
EN 300	0

EN 320 0
EN 340 0
EN 360 0
FHU-RIB:FHU-P:ALA-S1
EN 20 0
EN 40 0
EN 60 0
EN 80 0
EN 100 0
EN 120 0
EN 140 0
EN 160 0
EN 180 0
EN 200 0
EN 220 0
EN 240 -11671
EN 260 0
EN 280 0
EN 300 0
EN 320 0
EN 340 0
EN 360 0
C31-P:C31-RIB:ASP-S1
EN 20 0
EN 40 0
EN 60 0
EN 80 0
EN 100 0
EN 120 0
EN 140 0
EN 160 0
EN 180 0
EN 200 0
EN 220 0
EN 240 0
EN 260 0
EN 280 0
EN 300 0
EN 320 -8102
EN 340 0
EN 360 0
U31-P:U31-RIB:ASN-S2
EN 20 0
EN 40 0
EN 60 0
EN 80 0
EN 100 0
EN 120 0
EN 140 0
EN 160 0
EN 180 0
EN 200 0
EN 220 0
EN 240 -8168
EN 260 0
EN 280 0
EN 300 0
EN 320 0

EN 340 0
EN 360 0
U31-RIB:U31-P:HIS-CA
EN 20 0
EN 40 0
EN 60 0
EN 80 0
EN 100 0
EN 120 0
EN 140 0
EN 160 0
EN 180 0
EN 200 0
EN 220 0
EN 240 0
EN 260 0
EN 280 0
EN 300 0
EN 320 -9953
EN 340 0
EN 360 0
C31-P:C31-RIB:PHE-S2
EN 20 0
EN 40 0
EN 60 0
EN 80 0
EN 100 -9456
EN 120 -9819
EN 140 0
EN 160 0
EN 180 0
EN 200 0
EN 220 0
EN 240 0
EN 260 0
EN 280 0
EN 300 0
EN 320 0
EN 340 0
EN 360 0
U34-RIB:U34-P:HIS-CA
EN 20 0
EN 40 0
EN 60 0
EN 80 0
EN 100 0
EN 120 0
EN 140 0
EN 160 -17115
EN 180 0
EN 200 0
EN 220 0
EN 240 0
EN 260 0
EN 280 0
EN 300 0
EN 320 0
EN 340 0

EN 360 0
DA-RIB:DA-M5:LYS-S2
EN 20 0
EN 40 0
EN 60 0
EN 80 0
EN 100 0
EN 120 0
EN 140 0
EN 160 0
EN 180 0
EN 200 0
EN 220 0
EN 240 0
EN 260 0
EN 280 -8528
EN 300 0
EN 320 0
EN 340 0
EN 360 0
C31-RIB:C31-P:PHE-CA
EN 20 0
EN 40 0
EN 60 0
EN 80 0
EN 100 0
EN 120 0
EN 140 0
EN 160 0
EN 180 0
EN 200 0
EN 220 0
EN 240 -12294
EN 260 0
EN 280 0
EN 300 0
EN 320 0
EN 340 0
EN 360 0
U31-P:U31-RIB:ASN-S1
EN 20 0
EN 40 0
EN 60 0
EN 80 0
EN 100 0
EN 120 0
EN 140 0
EN 160 0
EN 180 0
EN 200 0
EN 220 0
EN 240 -7472
EN 260 0
EN 280 0
EN 300 0
EN 320 0
EN 340 0
EN 360 0

DA-RIB:DA-M5:LEU-S2
EN 20 0
EN 40 0
EN 60 0
EN 80 0
EN 100 0
EN 120 -8168
EN 140 0
EN 160 0
EN 180 0
EN 200 0
EN 220 0
EN 240 0
EN 260 0
EN 280 0
EN 300 0
EN 320 0
EN 340 0
EN 360 0
QUO-RIB:QUO-M6:GLN-S1
EN 20 0
EN 40 0
EN 60 0
EN 80 0
EN 100 0
EN 120 -3434
EN 140 0
EN 160 0
EN 180 0
EN 200 0
EN 220 0
EN 240 0
EN 260 0
EN 280 0
EN 300 0
EN 320 0
EN 340 0
EN 360 0
C31-RIB:C31-P:PHE-S1
EN 20 0
EN 40 0
EN 60 0
EN 80 0
EN 100 0
EN 120 0
EN 140 0
EN 160 0
EN 180 0
EN 200 0
EN 220 0
EN 240 -10245
EN 260 0
EN 280 0
EN 300 0
EN 320 0
EN 340 0
EN 360 0
U31-RIB:U31-MY:MET-CA

EN 20 0
EN 40 0
EN 60 0
EN 80 -7975
EN 100 0
EN 120 0
EN 140 0
EN 160 0
EN 180 0
EN 200 0
EN 220 0
EN 240 0
EN 260 0
EN 280 0
EN 300 0
EN 320 0
EN 340 0
EN 360 0
U34-RIB:U34-P:GLU-S2
EN 20 0
EN 40 0
EN 60 0
EN 80 0
EN 100 0
EN 120 0
EN 140 0
EN 160 0
EN 180 0
EN 200 0
EN 220 0
EN 240 0
EN 260 0
EN 280 0
EN 300 0
EN 320 0
EN 340 -11174
EN 360 0
DA-RIB:DA-M6:GLN-S1
EN 20 0
EN 40 0
EN 60 0
EN 80 0
EN 100 0
EN 120 0
EN 140 0
EN 160 0
EN 180 0
EN 200 0
EN 220 0
EN 240 0
EN 260 -8949
EN 280 0
EN 300 0
EN 320 0
EN 340 0
EN 360 0
H2U-P:H2U-RIB:GLY-CA
EN 20 0

EN 40 0
EN 60 0
EN 80 0
EN 100 0
EN 120 0
EN 140 0
EN 160 0
EN 180 0
EN 200 0
EN 220 0
EN 240 0
EN 260 0
EN 280 0
EN 300 0
EN 320 -8306
EN 340 0
EN 360 0
OMC-RIB:OMC-P:LYS-S1
EN 20 0
EN 40 0
EN 60 0
EN 80 0
EN 100 -7575
EN 120 0
EN 140 0
EN 160 0
EN 180 0
EN 200 0
EN 220 0
EN 240 0
EN 260 0
EN 280 0
EN 300 0
EN 320 0
EN 340 0
EN 360 0
QUO-P:QUO-RIB:LYS-S1
EN 20 0
EN 40 0
EN 60 0
EN 80 0
EN 100 0
EN 120 0
EN 140 -3434
EN 160 0
EN 180 0
EN 200 0
EN 220 0
EN 240 0
EN 260 0
EN 280 0
EN 300 0
EN 320 0
EN 340 0
EN 360 0
FMU-P:FMU-RIB:GLU-S1
EN 20 0
EN 40 0

EN 60 0
EN 80 0
EN 100 0
EN 120 0
EN 140 0
EN 160 0
EN 180 0
EN 200 0
EN 220 0
EN 240 0
EN 260 0
EN 280 0
EN 300 0
EN 320 0
EN 340 -13127
EN 360 0
IU-RIB:IU-P:ARG-S1
EN 20 0
EN 40 0
EN 60 -12294
EN 80 0
EN 100 0
EN 120 0
EN 140 0
EN 160 0
EN 180 0
EN 200 0
EN 220 0
EN 240 0
EN 260 0
EN 280 0
EN 300 0
EN 320 0
EN 340 -10760
EN 360 0
U34-RIB:U34-P:TYR-S2
EN 20 0
EN 40 0
EN 60 0
EN 80 0
EN 100 0
EN 120 0
EN 140 0
EN 160 0
EN 180 0
EN 200 0
EN 220 0
EN 240 0
EN 260 0
EN 280 0
EN 300 0
EN 320 -13680
EN 340 0
EN 360 0
C31-RIB:C31-P:GLU-S1
EN 20 0
EN 40 0
EN 60 0

EN 80 0
EN 100 0
EN 120 0
EN 140 0
EN 160 0
EN 180 0
EN 200 0
EN 220 0
EN 240 0
EN 260 -6734
EN 280 0
EN 300 0
EN 320 0
EN 340 0
EN 360 0
C31-P:C31-RIB:GLN-S2
EN 20 0
EN 40 0
EN 60 0
EN 80 0
EN 100 0
EN 120 0
EN 140 -9571
EN 160 0
EN 180 0
EN 200 0
EN 220 0
EN 240 0
EN 260 0
EN 280 0
EN 300 0
EN 320 0
EN 340 0
EN 360 0
FMU-RIB:FMU-MY:PRO-CA
EN 20 0
EN 40 0
EN 60 0
EN 80 0
EN 100 0
EN 120 0
EN 140 -14393
EN 160 0
EN 180 0
EN 200 0
EN 220 0
EN 240 0
EN 260 0
EN 280 0
EN 300 0
EN 320 0
EN 340 0
EN 360 0
QUO-RIB:QUO-M5:ASN-S1
EN 20 0
EN 40 0
EN 60 0
EN 80 -3434

EN 100 0
EN 120 0
EN 140 0
EN 160 0
EN 180 0
EN 200 0
EN 220 0
EN 240 0
EN 260 0
EN 280 0
EN 300 0
EN 320 0
EN 340 0
EN 360 0
DA-RIB:DA-M6:LYS-S1
EN 20 0
EN 40 0
EN 60 0
EN 80 0
EN 100 0
EN 120 0
EN 140 0
EN 160 0
EN 180 0
EN 200 0
EN 220 0
EN 240 0
EN 260 0
EN 280 0
EN 300 -8859
EN 320 0
EN 340 0
EN 360 0
GTP-RIB:GTP-M5:ALA-S1
EN 20 0
EN 40 0
EN 60 0
EN 80 0
EN 100 0
EN 120 0
EN 140 0
EN 160 0
EN 180 0
EN 200 0
EN 220 0
EN 240 0
EN 260 0
EN 280 -7523
EN 300 0
EN 320 0
EN 340 0
EN 360 0
U31-RIB:U31-P:GLN-CA
EN 20 0
EN 40 0
EN 60 0
EN 80 0
EN 100 0

EN 120 0
EN 140 0
EN 160 0
EN 180 0
EN 200 0
EN 220 0
EN 240 0
EN 260 -10095
EN 280 0
EN 300 0
EN 320 0
EN 340 0
EN 360 0
C31-RIB:C31-P:MET-S2
EN 20 0
EN 40 0
EN 60 0
EN 80 0
EN 100 0
EN 120 0
EN 140 0
EN 160 0
EN 180 0
EN 200 0
EN 220 0
EN 240 0
EN 260 0
EN 280 0
EN 300 0
EN 320 -9139
EN 340 0
EN 360 0
DA-RIB:DA-M6:GLU-S2
EN 20 0
EN 40 0
EN 60 0
EN 80 0
EN 100 0
EN 120 0
EN 140 0
EN 160 0
EN 180 0
EN 200 0
EN 220 0
EN 240 0
EN 260 0
EN 280 0
EN 300 0
EN 320 0
EN 340 -7975
EN 360 0
QUO-RIB:QUO-M6:ASN-CA
EN 20 0
EN 40 0
EN 60 0
EN 80 -3434
EN 100 0
EN 120 0

EN 140	0
EN 160	0
EN 180	0
EN 200	0
EN 220	0
EN 240	0
EN 260	0
EN 280	0
EN 300	0
EN 320	0
EN 340	0
EN 360	0
FMU-RIB:FMU-MY:ALA-S1
EN 20 0
EN 40 0
EN 60 0
EN 80 0
EN 100	-11410
EN 120	0
EN 140	0
EN 160	0
EN 180	0
EN 200	0
EN 220	0
EN 240	0
EN 260	0
EN 280	0
EN 300	0
EN 320	0
EN 340	0
EN 360	0
IU-RIB:IU-MY:SER-CA
EN 20 0
EN 40 0
EN 60 0
EN 80 -9401
EN 100	0
EN 120	0
EN 140	0
EN 160	0
EN 180	0
EN 200	0
EN 220	0
EN 240	0
EN 260	0
EN 280	0
EN 300	0
EN 320	0
EN 340	0
EN 360	0
FMU-RIB:FMU-P:ALA-S1
EN 20 0
EN 40 0
EN 60 0
EN 80 0
EN 100	0
EN 120	0
EN 140	0

EN 160 0
EN 180 0
EN 200 0
EN 220 0
EN 240 0
EN 260 -11410
EN 280 0
EN 300 0
EN 320 0
EN 340 0
EN 360 0
IU-RIB:IU-P:SER-CA
EN 20 0
EN 40 0
EN 60 0
EN 80 0
EN 100 -12294
EN 120 0
EN 140 0
EN 160 0
EN 180 0
EN 200 0
EN 220 0
EN 240 0
EN 260 0
EN 280 0
EN 300 0
EN 320 0
EN 340 0
EN 360 0
C31-RIB:C31-P:ALA-S1
EN 20 0
EN 40 0
EN 60 0
EN 80 0
EN 100 0
EN 120 0
EN 140 0
EN 160 0
EN 180 0
EN 200 0
EN 220 0
EN 240 0
EN 260 0
EN 280 -8236
EN 300 0
EN 320 0
EN 340 0
EN 360 0
H2U-RIB:H2U-P:LEU-S1
EN 20 0
EN 40 0
EN 60 0
EN 80 0
EN 100 -8306
EN 120 0
EN 140 0
EN 160 0

EN 180 0
EN 200 0
EN 220 0
EN 240 0
EN 260 0
EN 280 0
EN 300 0
EN 320 0
EN 340 0
EN 360 0
FMU-P:FMU-RIB:ILE-CA
EN 20 0
EN 40 0
EN 60 0
EN 80 0
EN 100 0
EN 120 0
EN 140 0
EN 160 0
EN 180 0
EN 200 0
EN 220 0
EN 240 0
EN 260 -2722
EN 280 0
EN 300 0
EN 320 0
EN 340 0
EN 360 0
U34-P:U34-RIB:SER-CA
EN 20 0
EN 40 0
EN 60 0
EN 80 0
EN 100 0
EN 120 0
EN 140 0
EN 160 0
EN 180 0
EN 200 0
EN 220 0
EN 240 0
EN 260 0
EN 280 0
EN 300 -9139
EN 320 0
EN 340 0
EN 360 0
DA-RIB:DA-M6:HIS-CA
EN 20 0
EN 40 0
EN 60 0
EN 80 0
EN 100 0
EN 120 0
EN 140 0
EN 160 0
EN 180 0

EN 200 0
EN 220 0
EN 240 0
EN 260 -9571
EN 280 0
EN 300 0
EN 320 0
EN 340 0
EN 360 0
C31-RIB:C31-P:ALA-CA
EN 20 0
EN 40 0
EN 60 0
EN 80 0
EN 100 0
EN 120 0
EN 140 0
EN 160 0
EN 180 0
EN 200 0
EN 220 0
EN 240 0
EN 260 0
EN 280 0
EN 300 -7975
EN 320 0
EN 340 0
EN 360 0
U31-RIB:U31-MY:TYR-CA
EN 20 0
EN 40 0
EN 60 0
EN 80 0
EN 100 0
EN 120 0
EN 140 0
EN 160 0
EN 180 0
EN 200 0
EN 220 0
EN 240 0
EN 260 0
EN 280 0
EN 300 0
EN 320 -8949
EN 340 0
EN 360 0
FMU-RIB:FMU-MY:CYS-CA
EN 20 0
EN 40 0
EN 60 0
EN 80 0
EN 100 0
EN 120 -2722
EN 140 0
EN 160 0
EN 180 0
EN 200 0

EN 220 0
EN 240 0
EN 260 0
EN 280 0
EN 300 0
EN 320 0
EN 340 0
EN 360 0
5BU-RIB:5BU-P:ARG-S2
EN 20 0
EN 40 0
EN 60 0
EN 80 0
EN 100 0
EN 120 0
EN 140 0
EN 160 -10958
EN 180 0
EN 200 0
EN 220 0
EN 240 0
EN 260 0
EN 280 0
EN 300 0
EN 320 0
EN 340 0
EN 360 0
U31-RIB:U31-P:ASN-CA
EN 20 0
EN 40 0
EN 60 0
EN 80 0
EN 100 -6970
EN 120 0
EN 140 0
EN 160 0
EN 180 0
EN 200 0
EN 220 0
EN 240 0
EN 260 0
EN 280 0
EN 300 0
EN 320 0
EN 340 0
EN 360 0
H2U-RIB:H2U-MY:THR-S1
EN 20 0
EN 40 0
EN 60 0
EN 80 0
EN 100 0
EN 120 0
EN 140 0
EN 160 0
EN 180 0
EN 200 0
EN 220 0

EN 240 0
EN 260 0
EN 280 0
EN 300 0
EN 320 -8606
EN 340 0
EN 360 0
U31-RIB:U31-P:ARG-S1
EN 20 0
EN 40 0
EN 60 0
EN 80 0
EN 100 0
EN 120 0
EN 140 0
EN 160 0
EN 180 0
EN 200 0
EN 220 0
EN 240 0
EN 260 0
EN 280 0
EN 300 0
EN 320 -8859
EN 340 0
EN 360 0
U31-P:U31-RIB:GLN-S2
EN 20 0
EN 40 0
EN 60 -11174
EN 80 0
EN 100 0
EN 120 0
EN 140 0
EN 160 0
EN 180 0
EN 200 0
EN 220 0
EN 240 0
EN 260 0
EN 280 0
EN 300 0
EN 320 0
EN 340 0
EN 360 0
U34-RIB:U34-P:ARG-S2
EN 20 0
EN 40 0
EN 60 0
EN 80 0
EN 100 0
EN 120 0
EN 140 0
EN 160 0
EN 180 0
EN 200 0
EN 220 0
EN 240 0

EN 260 0
EN 280 0
EN 300 -2722
EN 320 0
EN 340 0
EN 360 0
U31-P:U31-RIB:MET-S2
EN 20 0
EN 40 0
EN 60 0
EN 80 0
EN 100 0
EN 120 -8859
EN 140 0
EN 160 0
EN 180 0
EN 200 0
EN 220 0
EN 240 0
EN 260 0
EN 280 0
EN 300 0
EN 320 0
EN 340 0
EN 360 0
U31-RIB:U31-P:ALA-S1
EN 20 0
EN 40 0
EN 60 0
EN 80 0
EN 100 0
EN 120 0
EN 140 0
EN 160 0
EN 180 0
EN 200 0
EN 220 0
EN 240 0
EN 260 0
EN 280 0
EN 300 0
EN 320 0
EN 340 -8236
EN 360 0
FHU-RIB:FHU-MY:CYS-S1
EN 20 0
EN 40 0
EN 60 0
EN 80 0
EN 100 0
EN 120 0
EN 140 0
EN 160 0
EN 180 0
EN 200 0
EN 220 0
EN 240 0
EN 260 0

EN 280 -15398
EN 300 0
EN 320 0
EN 340 0
EN 360 0
IU-RIB:IU-P:HIS-S2
EN 20 0
EN 40 0
EN 60 0
EN 80 0
EN 100 0
EN 120 -14393
EN 140 0
EN 160 0
EN 180 0
EN 200 0
EN 220 0
EN 240 0
EN 260 0
EN 280 0
EN 300 0
EN 320 0
EN 340 0
EN 360 0
DA-RIB:DA-M6:GLN-CA
EN 20 0
EN 40 0
EN 60 0
EN 80 0
EN 100 0
EN 120 0
EN 140 0
EN 160 0
EN 180 0
EN 200 0
EN 220 0
EN 240 0
EN 260 -9241
EN 280 0
EN 300 0
EN 320 0
EN 340 0
EN 360 0
FMU-RIB:FMU-MY:GLN-S1
EN 20 0
EN 40 0
EN 60 0
EN 80 0
EN 100 0
EN 120 0
EN 140 0
EN 160 0
EN 180 0
EN 200 0
EN 220 0
EN 240 0
EN 260 0
EN 280 0

EN 300 -13680
EN 320 0
EN 340 0
EN 360 0
FMU-RIB:FMU-MY:ARG-S1
EN 20 0
EN 40 0
EN 60 -14393
EN 80 0
EN 100 0
EN 120 0
EN 140 0
EN 160 0
EN 180 0
EN 200 0
EN 220 0
EN 240 0
EN 260 0
EN 280 0
EN 300 0
EN 320 0
EN 340 0
EN 360 0
FMU-RIB:FMU-MY:HIS-S1
EN 20 0
EN 40 0
EN 60 0
EN 80 0
EN 100 0
EN 120 0
EN 140 0
EN 160 0
EN 180 0
EN 200 0
EN 220 0
EN 240 -17115
EN 260 0
EN 280 0
EN 300 0
EN 320 0
EN 340 0
EN 360 0
U31-RIB:U31-MY:TYR-S1
EN 20 0
EN 40 0
EN 60 0
EN 80 0
EN 100 0
EN 120 0
EN 140 0
EN 160 0
EN 180 0
EN 200 0
EN 220 0
EN 240 0
EN 260 0
EN 280 0
EN 300 -7231

EN 320 0
EN 340 0
EN 360 0
U31-RIB:U31-MY:VAL-S1
EN 20 0
EN 40 0
EN 60 0
EN 80 0
EN 100 0
EN 120 -7231
EN 140 0
EN 160 0
EN 180 0
EN 200 0
EN 220 0
EN 240 0
EN 260 0
EN 280 0
EN 300 0
EN 320 0
EN 340 0
EN 360 0
FHU-RIB:FHU-P:CYS-S1
EN 20 0
EN 40 0
EN 60 0
EN 80 0
EN 100 -15398
EN 120 0
EN 140 0
EN 160 0
EN 180 0
EN 200 0
EN 220 0
EN 240 0
EN 260 0
EN 280 0
EN 300 0
EN 320 0
EN 340 0
EN 360 0
U34-RIB:U34-P:PHE-S2
EN 20 0
EN 40 0
EN 60 0
EN 80 0
EN 100 0
EN 120 0
EN 140 0
EN 160 0
EN 180 0
EN 200 0
EN 220 0
EN 240 0
EN 260 0
EN 280 0
EN 300 -2722
EN 320 0

EN 340 0
EN 360 0
H2U-P:H2U-RIB:PHE-S2
EN 20 0
EN 40 0
EN 60 0
EN 80 0
EN 100 0
EN 120 0
EN 140 0
EN 160 0
EN 180 0
EN 200 0
EN 220 0
EN 240 0
EN 260 -15398
EN 280 0
EN 300 0
EN 320 0
EN 340 0
EN 360 0
DA-RIB:DA-M5:VAL-S1
EN 20 0
EN 40 0
EN 60 0
EN 80 0
EN 100 0
EN 120 0
EN 140 0
EN 160 0
EN 180 0
EN 200 0
EN 220 0
EN 240 -9241
EN 260 0
EN 280 0
EN 300 0
EN 320 0
EN 340 0
EN 360 0
IU-RIB:IU-P:HIS-S1
EN 20 0
EN 40 0
EN 60 0
EN 80 0
EN 100 -12294
EN 120 0
EN 140 0
EN 160 0
EN 180 0
EN 200 0
EN 220 0
EN 240 0
EN 260 0
EN 280 0
EN 300 0
EN 320 0
EN 340 0

EN 360 0
FMU-P:FMU-RIB:HIS-S2
EN 20 0
EN 40 0
EN 60 0
EN 80 0
EN 100 0
EN 120 0
EN 140 -14393
EN 160 0
EN 180 0
EN 200 0
EN 220 0
EN 240 0
EN 260 0
EN 280 0
EN 300 0
EN 320 0
EN 340 0
EN 360 0
U34-RIB:U34-P:ARG-S1
EN 20 0
EN 40 0
EN 60 0
EN 80 0
EN 100 0
EN 120 0
EN 140 0
EN 160 0
EN 180 0
EN 200 0
EN 220 0
EN 240 0
EN 260 0
EN 280 0
EN 300 0
EN 320 -2722
EN 340 0
EN 360 0
DA-RIB:DA-M6:ASN-S2
EN 20 0
EN 40 0
EN 60 0
EN 80 0
EN 100 0
EN 120 0
EN 140 0
EN 160 0
EN 180 0
EN 200 0
EN 220 0
EN 240 0
EN 260 -9692
EN 280 0
EN 300 0
EN 320 0
EN 340 0
EN 360 0

U31-RIB:U31-P:MET-S2
EN 20 0
EN 40 0
EN 60 0
EN 80 0
EN 100 0
EN 120 0
EN 140 -11671
EN 160 0
EN 180 0
EN 200 0
EN 220 0
EN 240 -17115
EN 260 0
EN 280 0
EN 300 0
EN 320 0
EN 340 0
EN 360 0
IU-RIB:IU-P:HIS-CA
EN 20 0
EN 40 0
EN 60 0
EN 80 0
EN 100 -15398
EN 120 0
EN 140 0
EN 160 0
EN 180 0
EN 200 0
EN 220 0
EN 240 0
EN 260 0
EN 280 0
EN 300 0
EN 320 0
EN 340 0
EN 360 0
H2U-RIB:H2U-P:GLU-CA
EN 20 0
EN 40 0
EN 60 0
EN 80 -9346
EN 100 0
EN 120 0
EN 140 0
EN 160 0
EN 180 0
EN 200 0
EN 220 0
EN 240 0
EN 260 0
EN 280 0
EN 300 0
EN 320 0
EN 340 0
EN 360 0
FHU-P:FHU-RIB:ILE-CA

EN 20 0
EN 40 0
EN 60 0
EN 80 0
EN 100 0
EN 120 0
EN 140 0
EN 160 0
EN 180 0
EN 200 0
EN 220 0
EN 240 0
EN 260 0
EN 280 0
EN 300 -10576
EN 320 -15398
EN 340 0
EN 360 0
QUO-RIB:QUO-M6:ASN-S2
EN 20 0
EN 40 0
EN 60 0
EN 80 -3434
EN 100 0
EN 120 0
EN 140 0
EN 160 0
EN 180 0
EN 200 0
EN 220 0
EN 240 0
EN 260 0
EN 280 0
EN 300 0
EN 320 0
EN 340 0
EN 360 0
FMU-P:FMU-RIB:CYS-CA
EN 20 0
EN 40 0
EN 60 0
EN 80 0
EN 100 0
EN 120 0
EN 140 0
EN 160 -2722
EN 180 0
EN 200 0
EN 220 0
EN 240 0
EN 260 0
EN 280 0
EN 300 0
EN 320 0
EN 340 0
EN 360 0
H2U-RIB:H2U-MY:PHE-S1
EN 20 0

```
EN 40 0
EN 60 0
EN 80 0
EN 100      0
EN 120      0
EN 140      -12294
EN 160      0
EN 180      0
EN 200      0
EN 220      0
EN 240      0
EN 260      0
EN 280      0
EN 300      0
EN 320      0
EN 340      0
EN 360      0
U31-RIB:U31-MY:GLN-S2
EN 20 0
EN 40 0
EN 60 -10958
EN 80 0
EN 100      0
EN 120      0
EN 140      0
EN 160      0
EN 180      0
EN 200      0
EN 220      0
EN 240      0
EN 260      0
EN 280      0
EN 300      0
EN 320      0
EN 340      0
EN 360      0
H2U-RIB:H2U-MY:GLN-S2
EN 20 0
EN 40 0
EN 60 0
EN 80 0
EN 100      0
EN 120      0
EN 140      0
EN 160      0
EN 180      0
EN 200      0
EN 220      0
EN 240      0
EN 260      0
EN 280      0
EN 300      0
EN 320      0
EN 340      0
EN 360      -14393
FMU-RIB:FMU-P:GLN-CA
EN 20 0
EN 40 0
```

EN 60 -2722
EN 80 0
EN 100 0
EN 120 0
EN 140 0
EN 160 0
EN 180 0
EN 200 0
EN 220 0
EN 240 0
EN 260 0
EN 280 0
EN 300 0
EN 320 0
EN 340 0
EN 360 0
IU-P:IU-RIB:GLN-S1
EN 20 0
EN 40 0
EN 60 0
EN 80 0
EN 100 0
EN 120 0
EN 140 0
EN 160 0
EN 180 0
EN 200 0
EN 220 0
EN 240 0
EN 260 0
EN 280 0
EN 300 -11963
EN 320 0
EN 340 0
EN 360 0
FHU-P:FHU-RIB:PRO-S1
EN 20 0
EN 40 0
EN 60 0
EN 80 0
EN 100 0
EN 120 0
EN 140 0
EN 160 -14393
EN 180 0
EN 200 0
EN 220 0
EN 240 0
EN 260 0
EN 280 0
EN 300 0
EN 320 0
EN 340 0
EN 360 0
DA-RIB:DA-M6:GLU-S1
EN 20 0
EN 40 0
EN 60 0

EN 80 0
EN 100 0
EN 120 0
EN 140 0
EN 160 0
EN 180 0
EN 200 0
EN 220 0
EN 240 0
EN 260 0
EN 280 0
EN 300 0
EN 320 0
EN 340 -8378
EN 360 0
IU-P:IU-RIB:ILE-CA
EN 20 0
EN 40 0
EN 60 0
EN 80 0
EN 100 0
EN 120 0
EN 140 0
EN 160 0
EN 180 0
EN 200 0
EN 220 0
EN 240 0
EN 260 0
EN 280 -12675
EN 300 0
EN 320 0
EN 340 0
EN 360 0
C31-P:C31-RIB:PHE-CA
EN 20 0
EN 40 0
EN 60 0
EN 80 0
EN 100 -9819
EN 120 -11671
EN 140 0
EN 160 0
EN 180 0
EN 200 0
EN 220 0
EN 240 0
EN 260 0
EN 280 0
EN 300 0
EN 320 0
EN 340 0
EN 360 0
DA-RIB:DA-M6:HIS-S1
EN 20 0
EN 40 0
EN 60 0
EN 80 0

EN 100 0
EN 120 0
EN 140 0
EN 160 0
EN 180 0
EN 200 0
EN 220 0
EN 240 0
EN 260 -9241
EN 280 0
EN 300 0
EN 320 0
EN 340 0
EN 360 0
FHU-P:FHU-RIB:PRO-CA
EN 20 0
EN 40 0
EN 60 0
EN 80 0
EN 100 0
EN 120 0
EN 140 0
EN 160 0
EN 180 0
EN 200 0
EN 220 0
EN 240 -9346
EN 260 0
EN 280 0
EN 300 0
EN 320 0
EN 340 0
EN 360 0
GTP-RIB:GTP-M5:ASP-S1
EN 20 0
EN 40 0
EN 60 0
EN 80 0
EN 100 0
EN 120 0
EN 140 0
EN 160 0
EN 180 0
EN 200 0
EN 220 0
EN 240 0
EN 260 0
EN 280 0
EN 300 -7914
EN 320 0
EN 340 0
EN 360 0
DA-RIB:DA-M6:THR-S1
EN 20 0
EN 40 0
EN 60 0
EN 80 0
EN 100 0

EN 120 0
EN 140 0
EN 160 0
EN 180 0
EN 200 0
EN 220 0
EN 240 0
EN 260 0
EN 280 -6970
EN 300 0
EN 320 0
EN 340 0
EN 360 0
QUO-RIB:QUO-M5:ARG-S1
EN 20 0
EN 40 0
EN 60 0
EN 80 0
EN 100 0
EN 120 -3434
EN 140 0
EN 160 0
EN 180 0
EN 200 0
EN 220 0
EN 240 0
EN 260 0
EN 280 0
EN 300 0
EN 320 0
EN 340 0
EN 360 0
IU-P:IU-RIB:ILE-S1
EN 20 0
EN 40 0
EN 60 0
EN 80 0
EN 100 -12294
EN 120 0
EN 140 0
EN 160 0
EN 180 0
EN 200 0
EN 220 0
EN 240 0
EN 260 0
EN 280 0
EN 300 0
EN 320 0
EN 340 0
EN 360 0
FMU-RIB:FMU-MY:GLN-S2
EN 20 0
EN 40 0
EN 60 0
EN 80 0
EN 100 0
EN 120 0

EN 140 0
EN 160 0
EN 180 0
EN 200 0
EN 220 0
EN 240 0
EN 260 0
EN 280 0
EN 300 -10245
EN 320 0
EN 340 0
EN 360 0
FMU-RIB:FMU-MY:ASP-CA
EN 20 0
EN 40 0
EN 60 0
EN 80 0
EN 100 0
EN 120 0
EN 140 0
EN 160 -13680
EN 180 0
EN 200 0
EN 220 0
EN 240 0
EN 260 0
EN 280 0
EN 300 0
EN 320 0
EN 340 0
EN 360 0
H2U-RIB:H2U-P:ARG-S1
EN 20 0
EN 40 0
EN 60 0
EN 80 -9241
EN 100 0
EN 120 0
EN 140 0
EN 160 0
EN 180 0
EN 200 0
EN 220 0
EN 240 0
EN 260 0
EN 280 0
EN 300 0
EN 320 0
EN 340 0
EN 360 0
C31-RIB:C31-MY:LEU-S2
EN 20 0
EN 40 0
EN 60 0
EN 80 0
EN 100 0
EN 120 0
EN 140 0

EN 160 0
EN 180 0
EN 200 0
EN 220 0
EN 240 0
EN 260 0
EN 280 -7629
EN 300 0
EN 320 0
EN 340 0
EN 360 0
OMC-RIB:OMC-P:LYS-CA
EN 20 0
EN 40 0
EN 60 0
EN 80 0
EN 100 -7854
EN 120 0
EN 140 0
EN 160 0
EN 180 0
EN 200 0
EN 220 0
EN 240 0
EN 260 0
EN 280 0
EN 300 0
EN 320 0
EN 340 0
EN 360 0
GTP-RIB:GTP-M5:SER-CA
EN 20 0
EN 40 0
EN 60 0
EN 80 0
EN 100 -8949
EN 120 0
EN 140 0
EN 160 0
EN 180 0
EN 200 0
EN 220 0
EN 240 0
EN 260 0
EN 280 0
EN 300 0
EN 320 0
EN 340 0
EN 360 0
DA-RIB:DA-M6:LYS-CA
EN 20 0
EN 40 0
EN 60 0
EN 80 0
EN 100 0
EN 120 0
EN 140 0
EN 160 0

EN 180 0
EN 200 0
EN 220 0
EN 240 0
EN 260 0
EN 280 -9456
EN 300 0
EN 320 0
EN 340 0
EN 360 0
QUO-P:QUO-RIB:GLN-S2
EN 20 0
EN 40 0
EN 60 0
EN 80 0
EN 100 0
EN 120 0
EN 140 0
EN 160 0
EN 180 0
EN 200 0
EN 220 0
EN 240 0
EN 260 0
EN 280 0
EN 300 0
EN 320 0
EN 340 -3434
EN 360 0
H2U-RIB:H2U-MY:LYS-S2
EN 20 0
EN 40 0
EN 60 0
EN 80 0
EN 100 0
EN 120 0
EN 140 0
EN 160 0
EN 180 0
EN 200 0
EN 220 0
EN 240 -8606
EN 260 0
EN 280 0
EN 300 0
EN 320 0
EN 340 0
EN 360 0
U31-P:U31-RIB:GLU-CA
EN 20 0
EN 40 0
EN 60 0
EN 80 0
EN 100 0
EN 120 0
EN 140 0
EN 160 0
EN 180 0

EN 200 0
EN 220 0
EN 240 0
EN 260 0
EN 280 0
EN 300 0
EN 320 0
EN 340 -10095
EN 360 0
DA-RIB:DA-M6:ASN-CA
EN 20 0
EN 40 0
EN 60 0
EN 80 0
EN 100 0
EN 120 0
EN 140 0
EN 160 0
EN 180 0
EN 200 0
EN 220 0
EN 240 -11174
EN 260 0
EN 280 0
EN 300 0
EN 320 0
EN 340 0
EN 360 0
H2U-RIB:H2U-MY:ARG-CA
EN 20 0
EN 40 0
EN 60 0
EN 80 0
EN 100 0
EN 120 0
EN 140 0
EN 160 0
EN 180 0
EN 200 0
EN 220 0
EN 240 0
EN 260 0
EN 280 0
EN 300 0
EN 320 -9139
EN 340 0
EN 360 0
C31-P:C31-RIB:LEU-S2
EN 20 0
EN 40 0
EN 60 0
EN 80 0
EN 100 0
EN 120 0
EN 140 0
EN 160 0
EN 180 0
EN 200 0

EN 220 0
EN 240 0
EN 260 0
EN 280 0
EN 300 -8606
EN 320 0
EN 340 0
EN 360 0
5BU-RIB:5BU-P:THR-CA
EN 20 0
EN 40 0
EN 60 0
EN 80 0
EN 100 0
EN 120 0
EN 140 0
EN 160 -15398
EN 180 0
EN 200 0
EN 220 0
EN 240 0
EN 260 0
EN 280 0
EN 300 0
EN 320 0
EN 340 0
EN 360 0
5BU-P:5BU-RIB:PRO-S1
EN 20 0
EN 40 0
EN 60 -12294
EN 80 0
EN 100 0
EN 120 0
EN 140 0
EN 160 0
EN 180 0
EN 200 0
EN 220 0
EN 240 0
EN 260 0
EN 280 0
EN 300 0
EN 320 0
EN 340 0
EN 360 0
FMU-RIB:FMU-MY:ASN-CA
EN 20 0
EN 40 0
EN 60 0
EN 80 0
EN 100 -13680
EN 120 0
EN 140 0
EN 160 0
EN 180 0
EN 200 0
EN 220 0

EN 240	0
EN 260	0
EN 280	0
EN 300	0
EN 320	0
EN 340	0
EN 360	0
U31-RIB:U31-P:HIS-S1
EN 20 0
EN 40 0
EN 60 0
EN 80 0
EN 100	0
EN 120	0
EN 140	0
EN 160	0
EN 180	0
EN 200	0
EN 220	0
EN 240	0
EN 260	0
EN 280	0
EN 300	0
EN 320	-9042
EN 340	0
EN 360	0
5BU-RIB:5BU-P:PRO-S1
EN 20 0
EN 40 0
EN 60 0
EN 80 0
EN 100	0
EN 120	0
EN 140	0
EN 160	0
EN 180	0
EN 200	0
EN 220	0
EN 240	0
EN 260	0
EN 280	-12675
EN 300	0
EN 320	0
EN 340	0
EN 360	0
H2U-RIB:H2U-P:TRP-CA
EN 20 0
EN 40 0
EN 60 0
EN 80 0
EN 100	-14393
EN 120	0
EN 140	0
EN 160	0
EN 180	0
EN 200	0
EN 220	0
EN 240	0

EN 260 0
EN 280 0
EN 300 0
EN 320 0
EN 340 0
EN 360 0
FMU-RIB:FMU-MY:SER-CA
EN 20 0
EN 40 0
EN 60 0
EN 80 0
EN 100 0
EN 120 0
EN 140 -11174
EN 160 0
EN 180 0
EN 200 0
EN 220 0
EN 240 0
EN 260 0
EN 280 0
EN 300 0
EN 320 0
EN 340 0
EN 360 0
FMU-RIB:FMU-P:ASP-S1
EN 20 0
EN 40 0
EN 60 0
EN 80 0
EN 100 0
EN 120 0
EN 140 -11963
EN 160 0
EN 180 0
EN 200 0
EN 220 0
EN 240 0
EN 260 0
EN 280 0
EN 300 0
EN 320 0
EN 340 0
EN 360 0
U31-RIB:U31-P:GLN-S2
EN 20 0
EN 40 0
EN 60 0
EN 80 0
EN 100 0
EN 120 0
EN 140 0
EN 160 0
EN 180 0
EN 200 0
EN 220 0
EN 240 0
EN 260 0

EN 280	-8168
EN 300	0
EN 320	0
EN 340	0
EN 360	0
H2U-RIB:H2U-MY:GLN-CA
EN 20 0
EN 40 0
EN 60 0
EN 80 0
EN 100	0
EN 120	0
EN 140	0
EN 160	0
EN 180	0
EN 200	0
EN 220	0
EN 240	0
EN 260	0
EN 280	0
EN 300	0
EN 320	0
EN 340	0
EN 360	-13680
G:HIS-S2
EN WoCr	-4633
EN Sug	-5133
EN Hoo	-4183
C:GLU-CA
EN WoCr	-396
EN Sug	-1685
EN Hoo	-1030
A:ASN-CA
EN WoCr	-3652
EN Sug	-4262
EN Hoo	-3042
C:LEU-CA
EN WoCr	-4302
EN Sug	-4556
EN Hoo	-3999
U:ASN-S2
EN WoCr	-4219
EN Sug	-4189
EN Hoo	-4049
G:ARG-CA
EN WoCr	-3795
EN Sug	-4053
EN Hoo	-3867
C:SER-CA
EN WoCr	-3435
EN Sug	-4375
EN Hoo	-3633
A:GLU-CA
EN WoCr	-2272
EN Sug	-1698
EN Hoo	-1970
G:GLN-CA
EN WoCr	658

EN Sug -5572
EN Hoo -2236
C:ASP-S2
EN WoCr -2421
EN Sug -2703
EN Hoo -2126
C:VAL-S1
EN WoCr -3019
EN Sug -4060
EN Hoo -2949
G:ALA-S1
EN WoCr -1305
EN Sug -2542
EN Hoo -3201
C:GLN-S1
EN WoCr -3020
EN Sug -5225
EN Hoo -2986
G:GLN-S1
EN WoCr -2049
EN Sug -5411
EN Hoo -1994
U:LYS-S2
EN WoCr -2913
EN Sug -3411
EN Hoo -4555
C:ASN-S2
EN WoCr -2557
EN Sug -4103
EN Hoo -3607
A:LYS-S2
EN WoCr -2253
EN Sug -2920
EN Hoo -4675
G:LYS-S1
EN WoCr -2849
EN Sug -2238
EN Hoo -4648
C:GLU-S1
EN WoCr -789
EN Sug -1355
EN Hoo -177
G:MET-CA
EN WoCr -4532
EN Sug -5371
EN Hoo -776
C:TRP-S1
EN WoCr -2862
EN Sug -5194
EN Hoo -4264
G:TRP-CA
EN WoCr -4873
EN Sug -5573
EN Hoo -3365
U:GLU-S1
EN WoCr 320
EN Sug -735
EN Hoo 498

G:GLU-S2
EN WoCr -1252
EN Sug -1314
EN Hoo -52
A:PHE-S1
EN WoCr -3108
EN Sug -5191
EN Hoo -3623
G:CYS-CA
EN WoCr -4175
EN Sug -3936
EN Hoo 0
A:ASN-S2
EN WoCr -3782
EN Sug -4531
EN Hoo -3116
G:ALA-CA
EN WoCr -1773
EN Sug -2809
EN Hoo -3341
A:ASN-S1
EN WoCr -3831
EN Sug -3905
EN Hoo -3034
G:LEU-CA
EN WoCr -3547
EN Sug -4590
EN Hoo -4568
G:ASN-CA
EN WoCr -3718
EN Sug -4156
EN Hoo -4004
A:TYR-S1
EN WoCr -5154
EN Sug -6202
EN Hoo -6611
C:LEU-S2
EN WoCr -3135
EN Sug -4443
EN Hoo -3569
G:LYS-CA
EN WoCr -3169
EN Sug -2735
EN Hoo -4729
C:TYR-S1
EN WoCr -5460
EN Sug -4954
EN Hoo -4569
G:TRP-S1
EN WoCr -5723
EN Sug -5313
EN Hoo -4333
U:THR-S1
EN WoCr -2461
EN Sug -3502
EN Hoo -2803
C:MET-CA
EN WoCr -1188

EN Sug -4818
EN Hoo -3467
U:ASN-S1
EN WoCr -3766
EN Sug -4601
EN Hoo -4093
C:LYS-S1
EN WoCr -3198
EN Sug -3543
EN Hoo -3296
A:ILE-S1
EN WoCr -3192
EN Sug -3903
EN Hoo -1827
G:HIS-CA
EN WoCr -3556
EN Sug -4953
EN Hoo -3738
G:GLN-S2
EN WoCr -2868
EN Sug -4338
EN Hoo -3108
C:LEU-S1
EN WoCr -3645
EN Sug -4996
EN Hoo -4015
U:LYS-CA
EN WoCr -1911
EN Sug -3254
EN Hoo -3773
A:MET-CA
EN WoCr -5067
EN Sug -4680
EN Hoo -4782
G:ASP-CA
EN WoCr -2774
EN Sug -2753
EN Hoo -7
C:ALA-S1
EN WoCr -2470
EN Sug -3905
EN Hoo -3840
U:PRO-S1
EN WoCr -3826
EN Sug -3701
EN Hoo -2034
C:PHE-CA
EN WoCr -4225
EN Sug -3119
EN Hoo -4213
G:VAL-S1
EN WoCr -2795
EN Sug -2437
EN Hoo -1301
A:TRP-S1
EN WoCr -3130
EN Sug -4940
EN Hoo -4366

U:GLU-S2
EN WoCr -289
EN Sug -371
EN Hoo 254
U:GLN-S1
EN WoCr -3797
EN Sug -4560
EN Hoo -3590
G:ARG-S2
EN WoCr -3778
EN Sug -3020
EN Hoo -4776
G:TYR-S2
EN WoCr -4997
EN Sug -4459
EN Hoo -3859
G:ASN-S2
EN WoCr -3645
EN Sug -3452
EN Hoo -3707
A:HIS-CA
EN WoCr -4715
EN Sug -5956
EN Hoo -5358
U:SER-S1
EN WoCr -2184
EN Sug -3829
EN Hoo -3420
C:VAL-CA
EN WoCr -3312
EN Sug -4343
EN Hoo -2836
G:GLY-CA
EN WoCr -3119
EN Sug -3914
EN Hoo -3665
C:ASP-S1
EN WoCr -2292
EN Sug -3026
EN Hoo -2980
A:HIS-S2
EN WoCr -4941
EN Sug -4688
EN Hoo -5126
A:LEU-S2
EN WoCr -5173
EN Sug -3789
EN Hoo -5051
U:TRP-CA
EN WoCr -1439
EN Sug -3222
EN Hoo -5595
U:ASP-S2
EN WoCr -1343
EN Sug -2132
EN Hoo -528
A:TRP-CA
EN WoCr -2778

EN Sug -5352
EN Hoo -4800
A:ALA-S1
EN WoCr -1979
EN Sug -2711
EN Hoo -3554
G:HIS-S1
EN WoCr -3988
EN Sug -5297
EN Hoo -4079
A:SER-S1
EN WoCr -3496
EN Sug -3575
EN Hoo -4224
G:GLU-CA
EN WoCr -1660
EN Sug -1161
EN Hoo -495
U:VAL-CA
EN WoCr -1741
EN Sug -3679
EN Hoo -3570
G:ARG-S1
EN WoCr -3603
EN Sug -3139
EN Hoo -5051
C:ASP-CA
EN WoCr -3269
EN Sug -3067
EN Hoo -3074
U:PHE-S2
EN WoCr -5037
EN Sug -6577
EN Hoo 1048
U:TYR-S2
EN WoCr -4236
EN Sug -4921
EN Hoo -4818
U:TYR-CA
EN WoCr -6284
EN Sug -4519
EN Hoo -2529
U:PHE-CA
EN WoCr -5332
EN Sug -6308
EN Hoo -1250
C:LYS-CA
EN WoCr -3541
EN Sug -4069
EN Hoo -3223
U:HIS-CA
EN WoCr -2673
EN Sug -4609
EN Hoo -3975
C:GLU-S2
EN WoCr -1457
EN Sug -1336
EN Hoo -36

U:CYS-S1
EN WoCr -5787
EN Sug -5145
EN Hoo -4628
A:ALA-CA
EN WoCr -2679
EN Sug -2176
EN Hoo -3610
U:CYS-CA
EN WoCr -4704
EN Sug -2767
EN Hoo -4282
U:LEU-S2
EN WoCr -2602
EN Sug -5241
EN Hoo -3909
A:ASP-S2
EN WoCr -1638
EN Sug -2078
EN Hoo -2171
G:MET-S2
EN WoCr -3802
EN Sug -5004
EN Hoo -2824
A:GLN-CA
EN WoCr -3979
EN Sug -3918
EN Hoo -3813
U:ARG-CA
EN WoCr -3407
EN Sug -3806
EN Hoo -5139
U:VAL-S1
EN WoCr -503
EN Sug -3503
EN Hoo -3070
A:PRO-CA
EN WoCr -3936
EN Sug -3331
EN Hoo -4666
U:HIS-S1
EN WoCr -3343
EN Sug -4724
EN Hoo -3754
U:MET-S2
EN WoCr -2580
EN Sug -4384
EN Hoo -4343
A:PRO-S1
EN WoCr -3919
EN Sug -3896
EN Hoo -3706
G:ASP-S1
EN WoCr -2815
EN Sug -2549
EN Hoo -317
A:GLN-S2
EN WoCr -4232

EN Sug -3812
EN Hoo -3354
U:ALA-CA
EN WoCr -2312
EN Sug -3953
EN Hoo -4058
A:ARG-S1
EN WoCr -3496
EN Sug -4278
EN Hoo -5087
U:PHE-S1
EN WoCr -5226
EN Sug -7129
EN Hoo -1451
U:ASN-CA
EN WoCr -3705
EN Sug -4615
EN Hoo -4320
C:GLN-CA
EN WoCr -3230
EN Sug -5346
EN Hoo -1630
G:SER-S1
EN WoCr -2572
EN Sug -3184
EN Hoo -3529
G:PHE-S1
EN WoCr -3916
EN Sug -5149
EN Hoo -1640
G:SER-CA
EN WoCr -2277
EN Sug -3175
EN Hoo -3693
U:MET-CA
EN WoCr -193
EN Sug -6241
EN Hoo -3011
C:ILE-S1
EN WoCr -1606
EN Sug -4185
EN Hoo -4976
A:GLU-S2
EN WoCr -1164
EN Sug -1602
EN Hoo -1130
U:HIS-S2
EN WoCr -3380
EN Sug -4970
EN Hoo -4436
A:ARG-S2
EN WoCr -3950
EN Sug -3910
EN Hoo -5313
A:LEU-CA
EN WoCr -4312
EN Sug -5015
EN Hoo -3961

G:PHE-S2
EN WoCr -2462
EN Sug -5333
EN Hoo -2761
G:CYS-S1
EN WoCr -3665
EN Sug -1899
EN Hoo 0
G:ASP-S2
EN WoCr -2818
EN Sug -2616
EN Hoo -2267
A:PHE-S2
EN WoCr -4063
EN Sug -5468
EN Hoo -4705
C:GLY-CA
EN WoCr -3354
EN Sug -4360
EN Hoo -3816
G:TRP-S2
EN WoCr -5851
EN Sug -4701
EN Hoo -3750
U:GLU-CA
EN WoCr -178
EN Sug -826
EN Hoo 266
G:TYR-S1
EN WoCr -4352
EN Sug -4443
EN Hoo -2994
U:GLN-S2
EN WoCr -3861
EN Sug -3991
EN Hoo -3135
A:HIS-S1
EN WoCr -4169
EN Sug -5623
EN Hoo -4803
U:ILE-CA
EN WoCr -3380
EN Sug -2339
EN Hoo -2877
A:MET-S1
EN WoCr -5243
EN Sug -3637
EN Hoo -4789
C:TRP-CA
EN WoCr -3884
EN Sug -3774
EN Hoo -3321
G:ASN-S1
EN WoCr -3855
EN Sug -3994
EN Hoo -3817
G:PRO-CA
EN WoCr -3000

EN Sug -4004
EN Hoo -749
A:THR-CA
EN WoCr -3230
EN Sug -4275
EN Hoo -4611
G:LEU-S1
EN WoCr -3222
EN Sug -3932
EN Hoo -3848
A:ILE-CA
EN WoCr -3356
EN Sug -3391
EN Hoo -1551
G:ILE-CA
EN WoCr -1477
EN Sug -3052
EN Hoo -2089
A:ARG-CA
EN WoCr -4165
EN Sug -4903
EN Hoo -4848
C:HIS-S2
EN WoCr -3667
EN Sug -5330
EN Hoo -3209
C:ASN-CA
EN WoCr -3368
EN Sug -3742
EN Hoo -2721
U:GLY-CA
EN WoCr -2772
EN Sug -4721
EN Hoo -2575
A:GLY-CA
EN WoCr -3390
EN Sug -4232
EN Hoo -4781
G:PRO-S1
EN WoCr -2770
EN Sug -3768
EN Hoo -2253
C:HIS-CA
EN WoCr -4375
EN Sug -5888
EN Hoo -3201
C:ARG-S1
EN WoCr -4895
EN Sug -4463
EN Hoo -5669
C:THR-S1
EN WoCr -2945
EN Sug -4229
EN Hoo -2664
C:CYS-S1
EN WoCr -2644
EN Sug -4402
EN Hoo -4711

C:HIS-S1
EN WoCr -4218
EN Sug -5898
EN Hoo -4445
A:TYR-CA
EN WoCr -4869
EN Sug -6292
EN Hoo -6546
A:LEU-S1
EN WoCr -4572
EN Sug -5098
EN Hoo -5476
A:CYS-S1
EN WoCr -3184
EN Sug -4279
EN Hoo -6677
A:CYS-CA
EN WoCr -2174
EN Sug -3946
EN Hoo -5450
C:PHE-S2
EN WoCr -2279
EN Sug -5282
EN Hoo -3845
C:CYS-CA
EN WoCr -2535
EN Sug -3019
EN Hoo -2598
G:LEU-S2
EN WoCr -3134
EN Sug -3370
EN Hoo -4362
A:ASP-CA
EN WoCr -1678
EN Sug -1623
EN Hoo -2285
U:LEU-CA
EN WoCr -4056
EN Sug -4602
EN Hoo -3602
G:TYR-CA
EN WoCr -4513
EN Sug -4100
EN Hoo -2149
U:MET-S1
EN WoCr -1838
EN Sug -5444
EN Hoo -1932
U:LYS-S1
EN WoCr -2665
EN Sug -3263
EN Hoo -4207
U:ARG-S2
EN WoCr -3443
EN Sug -3991
EN Hoo -4662
C:ILE-CA
EN WoCr -2484

EN Sug -4798
EN Hoo -4654
G:GLU-S1
EN WoCr -1604
EN Sug -1188
EN Hoo -156
G:ILE-S1
EN WoCr -1766
EN Sug -2370
EN Hoo 106
A:ASP-S1
EN WoCr -1745
EN Sug -1669
EN Hoo -1957
U:SER-CA
EN WoCr -2378
EN Sug -3685
EN Hoo -3797
U:PRO-CA
EN WoCr -4250
EN Sug -3232
EN Hoo -3536
U:ILE-S1
EN WoCr -2565
EN Sug -3065
EN Hoo -4125
U:THR-CA
EN WoCr -1751
EN Sug -3184
EN Hoo -3476
C:LYS-S2
EN WoCr -2895
EN Sug -3028
EN Hoo -4309
C:ARG-S2
EN WoCr -4274
EN Sug -3875
EN Hoo -5728
U:ARG-S1
EN WoCr -3486
EN Sug -3713
EN Hoo -5058
G:THR-S1
EN WoCr -2118
EN Sug -2918
EN Hoo -2628
C:PHE-S1
EN WoCr -4447
EN Sug -3725
EN Hoo -4684
C:TYR-CA
EN WoCr -4469
EN Sug -6142
EN Hoo -3184
A:LYS-S1
EN WoCr -2820
EN Sug -3683
EN Hoo -4158

A:VAL-S1
EN WoCr -4258
EN Sug -4111
EN Hoo -3426
C:THR-CA
EN WoCr -2900
EN Sug -4336
EN Hoo -2412
A:TRP-S2
EN WoCr -4536
EN Sug -5527
EN Hoo -3858
C:PRO-CA
EN WoCr -3203
EN Sug -4318
EN Hoo -3677
C:GLN-S2
EN WoCr -3559
EN Sug -4867
EN Hoo -3025
A:MET-S2
EN WoCr -5450
EN Sug -4374
EN Hoo -3759
C:SER-S1
EN WoCr -3837
EN Sug -3942
EN Hoo -3904
C:ALA-CA
EN WoCr -2285
EN Sug -4172
EN Hoo -3876
G:PHE-CA
EN WoCr -1765
EN Sug -5351
EN Hoo -2471
C:ARG-CA
EN WoCr -3558
EN Sug -3994
EN Hoo -5409
C:MET-S2
EN WoCr -4378
EN Sug -5020
EN Hoo -3394
C:MET-S1
EN WoCr -2740
EN Sug -5180
EN Hoo -2553
A:GLN-S1
EN WoCr -4142
EN Sug -3635
EN Hoo -2919
A:GLU-S1
EN WoCr -1383
EN Sug -1882
EN Hoo -1567
U:ASP-S1
EN WoCr -1031

EN Sug -2429
EN Hoo 704
A:LYS-CA
EN WoCr -3018
EN Sug -3550
EN Hoo -3456
U:ASP-CA
EN WoCr -458
EN Sug -2259
EN Hoo -288
U:TRP-S1
EN WoCr 0
EN Sug -4336
EN Hoo -5851
C:TRP-S2
EN WoCr -5295
EN Sug -5385
EN Hoo -6290
G:MET-S1
EN WoCr -3622
EN Sug -5430
EN Hoo -1091
U:TRP-S2
EN WoCr -746
EN Sug -5345
EN Hoo -4397
C:ASN-S1
EN WoCr -2690
EN Sug -4392
EN Hoo -3071
A:TYR-S2
EN WoCr -3620
EN Sug -5138
EN Hoo -6579
C:TYR-S2
EN WoCr -4236
EN Sug -3922
EN Hoo -4998
U:TYR-S1
EN WoCr -5676
EN Sug -3084
EN Hoo -3547
A:PHE-CA
EN WoCr -4153
EN Sug -4376
EN Hoo -4023
U:GLN-CA
EN WoCr -3146
EN Sug -3928
EN Hoo -3050
U:ALA-S1
EN WoCr -1210
EN Sug -3328
EN Hoo -4071
A:SER-CA
EN WoCr -3833
EN Sug -3301
EN Hoo -3279

G:LYS-S2
EN WoCr -2640
EN Sug -2496
EN Hoo -4523
A:THR-S1
EN WoCr -2756
EN Sug -4062
EN Hoo -4368
G:THR-CA
EN WoCr -1204
EN Sug -3825
EN Hoo -2719
A:VAL-CA
EN WoCr -3667
EN Sug -3130
EN Hoo -4418
U:LEU-S1
EN WoCr -3512
EN Sug -5485
EN Hoo -3876
C:PRO-S1
EN WoCr -2236
EN Sug -3964
EN Hoo -3450
G:VAL-CA
EN WoCr -2824
EN Sug -2282
EN Hoo -2256
