# Supplementary material for: DARS-RNP and QUASI-RNP: New statistical potentials for protein-RNA docking
Source: BMC Bioinformatics. 2011 Aug 18;12:348. doi: 10.1186/1471-2105-12-348 (PMC3179970; doi:10.1186/1471-2105-12-348)
Supplement: Additional file 3 — Energy_QUASI.pdf. Energy for each distance, angle, and site bin, for each pair wise interaction in the QUASI-RNP potential. [file 1471-2105-12-348-S3.PDF]

U-P:CYS-S1
EN 3 0
EN 4 0
EN 5 0
EN 6 0
EN 7 -3668
EN 8 -827
EN 9 -1501
A-RIB:GLN-S2
EN 3 0
EN 4 -6080
EN 5 -5811
EN 6 -5279
EN 7 -4218
EN 8 -5226
EN 9 -4276
QUO-M6:GLU-S2
EN 3 0
EN 4 0
EN 5 -16655
EN 6 0
EN 7 0
EN 8 0
EN 9 0
FHU-MY:ALA-S1
EN 3 0
EN 4 0
EN 5 0
EN 6 0
EN 7 -14183
EN 8 -11342
EN 9 -10299
DA-RIB:ALA-S1
EN 3 0
EN 4 0
EN 5 0
EN 6 0
EN 7 -14183
EN 8 0
EN 9 0
G-P:MET-CA
EN 3 0
EN 4 0
EN 5 -1001
EN 6 -1397
EN 7 -1341
EN 8 -3436
EN 9 -2990
G-R6:GLN-S2
EN 3 0
EN 4 0
EN 5 -4920
EN 6 -4719
EN 7 -5375
EN 8 -4136
EN 9 -3063
A-R5:TYR-CA
EN 3 0

EN 4   0
EN 5   0
EN 6   -4369
EN 7   -2925
EN 8   -3067
EN 9   -3446
G-RIB:GLN-CA
EN 3   0
EN 4   0
EN 5   -1786
EN 6   -4904
EN 7   -2838
EN 8   -4884
EN 9   -4398
M2G-P:GLU-CA
EN 3   0
EN 4   0
EN 5   0
EN 6   0
EN 7   0
EN 8   -13110
EN 9   0
G-P:PHE-S1
EN 3   0
EN 4   0
EN 5   -2695
EN 6   -369
EN 7   -2030
EN 8   -3006
EN 9   -2967
U31-RIB:PHE-CA
EN 3   0
EN 4   0
EN 5   0
EN 6   0
EN 7   0
EN 8   0
EN 9   -13960
FHU-P:SER-S1
EN 3   0
EN 4   0
EN 5   0
EN 6   0
EN 7   0
EN 8   -12347
EN 9   0
U-Y:GLU-S1
EN 3   0
EN 4   0
EN 5   -1103
EN 6   0
EN 7   1279
EN 8   -701
EN 9   -1375
IU-RIB:VAL-S1
EN 3   0
EN 4   0
EN 5   0

EN 6   0
EN 7   0
EN 8   0
EN 9   -8083
C31-MY:LEU-S1
EN 3   0
EN 4   0
EN 5   0
EN 6   -15097
EN 7   0
EN 8   0
EN 9   0
G-RIB:ILE-S1
EN 3   0
EN 4   0
EN 5   -1711
EN 6   -1655
EN 7   -1680
EN 8   -1125
EN 9   -1385
OMC-P:LYS-S1
EN 3   0
EN 4   0
EN 5   0
EN 6   0
EN 7   -11167
EN 8   0
EN 9   0
M2G-P:SER-S1
EN 3   0
EN 4   0
EN 5   0
EN 6   0
EN 7   0
EN 8   0
EN 9   -13021
C-RIB:ILE-S1
EN 3   0
EN 4   0
EN 5   -2114
EN 6   -3776
EN 7   -2715
EN 8   -1038
EN 9   -2138
U-P:MET-CA
EN 3   0
EN 4   0
EN 5   -5740
EN 6   -2702
EN 7   -1641
EN 8   -2788
EN 9   -3200
A-RIB:GLY-CA
EN 3   0
EN 4   -7093
EN 5   -6823
EN 6   -4924
EN 7   -5580

EN 8   -5086
EN 9   -5465
C-Y:HIS-CA
EN 3   0
EN 4   0
EN 5   0
EN 6   -1840
EN 7   -4214
EN 8   -3643
EN 9   -3764
C-RIB:MET-S2
EN 3   0
EN 4   -6809
EN 5   -4823
EN 6   -5772
EN 7   -5163
EN 8   -3326
EN 9   -3548
C-P:GLU-S1
EN 3   0
EN 4   -3892
EN 5   -1906
EN 6   -585
EN 7   -1794
EN 8   -3038
EN 9   -3182
H2U-MY:GLY-CA
EN 3   0
EN 4   0
EN 5   0
EN 6   0
EN 7   -10583
EN 8   0
EN 9   0
C-RIB:LEU-S2
EN 3   0
EN 4   0
EN 5   -982
EN 6   -3096
EN 7   -3536
EN 8   -2005
EN 9   -1783
A-P:TRP-CA
EN 3   0
EN 4   0
EN 5   -4786
EN 6   -1748
EN 7   -686
EN 8   -4715
EN 9   -1954
G-R5:TRP-CA
EN 3   0
EN 4   0
EN 5   0
EN 6   -2903
EN 7   -1841
EN 8   -2988
EN 9   -3662

C-RIB:SER-S1
EN 3 0
EN 4 0
EN 5 -6119
EN 6 -6161
EN 7 -5067
EN 8 -5222
EN 9 -4728
A-RIB:ASP-S2
EN 3 0
EN 4 -4771
EN 5 -3337
EN 6 -4186
EN 7 -3124
EN 8 -3387
EN 9 -2966
C31-RIB:GLN-S2
EN 3 0
EN 4 0
EN 5 0
EN 6 0
EN 7 0
EN 8 0
EN 9 -14114
PSU-RIB:ARG-S2
EN 3 0
EN 4 0
EN 5 0
EN 6 0
EN 7 0
EN 8 0
EN 9 -8533
A-P:ASN-S1
EN 3 0
EN 4 -7526
EN 5 -5540
EN 6 -2502
EN 7 -4544
EN 8 -4756
EN 9 -4717
IU-MY:LEU-CA
EN 3 0
EN 4 0
EN 5 0
EN 6 0
EN 7 0
EN 8 -8688
EN 9 -7644
U-P:ALA-S1
EN 3 0
EN 4 -8189
EN 5 -6043
EN 6 -2834
EN 7 -4112
EN 8 -3596
EN 9 -3796
A-RIB:THR-CA
EN 3 0

EN 4   -5227
EN 5   -1523
EN 6   -3929
EN 7   -5679
EN 8   -3843
EN 9   -3683
U-RIB:CYS-S1
EN 3   0
EN 4   0
EN 5   0
EN 6   -3007
EN 7   -941
EN 8   -2540
EN 9   -1496
U-RIB:HIS-S2
EN 3   0
EN 4   -7806
EN 5   -6372
EN 6   -7419
EN 7   -5708
EN 8   -5418
EN 9   -4705
C-RIB:GLU-S2
EN 3   0
EN 4   0
EN 5   -4199
EN 6   -3330
EN 7   -2053
EN 8   -2757
EN 9   -2935
C-P:GLU-CA
EN 3   0
EN 4   0
EN 5   -2904
EN 6   1138
EN 7   1195
EN 8   -1978
EN 9   -3234
H2U-RIB:PHE-S2
EN 3   0
EN 4   0
EN 5   0
EN 6   -13205
EN 7   0
EN 8   0
EN 9   0
G-R5:ASP-S2
EN 3   0
EN 4   0
EN 5   1209
EN 6   -904
EN 7   -4282
EN 8   -3450
EN 9   -3441
G-P:HIS-S2
EN 3   0
EN 4   0
EN 5   -5906

EN 6   -6784
EN 7   -5572
EN 8   -4874
EN 9   -4962
U-P:GLU-S1
EN 3   0
EN 4   0
EN 5   -2112
EN 6   -1504
EN 7   -1829
EN 8   -3307
EN 9   -2263
C-RIB:ASP-CA
EN 3   0
EN 4   0
EN 5   -3133
EN 6   -3766
EN 7   -3925
EN 8   -3896
EN 9   -3259
U-RIB:ALA-CA
EN 3   0
EN 4   0
EN 5   -2763
EN 6   -2447
EN 7   -4107
EN 8   -3203
EN 9   -4040
C-P:LYS-S2
EN 3   0
EN 4   -11172
EN 5   -9066
EN 6   -5535
EN 7   -6240
EN 8   -5679
EN 9   -5280
C-RIB:PRO-S1
EN 3   0
EN 4   0
EN 5   -4895
EN 6   -5397
EN 7   -4627
EN 8   -4769
EN 9   -4108
QUO-M5:PHE-CA
EN 3   0
EN 4   0
EN 5   0
EN 6   0
EN 7   0
EN 8   0
EN 9   -13960
C31-RIB:PHE-CA
EN 3   0
EN 4   0
EN 5   0
EN 6   0
EN 7   0

EN 8   -16721
EN 9   0
C-P:GLN-S1
EN 3   0
EN 4   0
EN 5   -5925
EN 6   -3600
EN 7   -4969
EN 8   -5639
EN 9   -4945
U-RIB:PHE-CA
EN 3   0
EN 4   0
EN 5   -2990
EN 6   -1669
EN 7   -3330
EN 8   -3211
EN 9   -3694
QUO-RIB:ASN-S2
EN 3   0
EN 4   0
EN 5   -18410
EN 6   0
EN 7   0
EN 8   0
EN 9   0
G-P:VAL-CA
EN 3   0
EN 4   0
EN 5   0
EN 6   -1436
EN 7   -1877
EN 8   -2470
EN 9   -1934
U31-P:ASP-S2
EN 3   0
EN 4   0
EN 5   0
EN 6   -16033
EN 7   0
EN 8   -13848
EN 9   -12804
FHU-RIB:THR-S1
EN 3   0
EN 4   0
EN 5   0
EN 6   0
EN 7   -13710
EN 8   -12586
EN 9   -11543
U-P:GLU-CA
EN 3   0
EN 4   0
EN 5   -2818
EN 6   219
EN 7   -1822
EN 8   -2416
EN 9   -2603

FMU-MY:GLU-S2
EN 3 0
EN 4 0
EN 5 -16655
EN 6 0
EN 7 0
EN 8 0
EN 9 0
A-R5:CYS-S1
EN 3 0
EN 4 0
EN 5 -3561
EN 6 -2240
EN 7 -2896
EN 8 -2777
EN 9 987
G-RIB:ARG-S2
EN 3 0
EN 4 -3927
EN 5 -5526
EN 6 -6490
EN 7 -6579
EN 8 -6508
EN 9 -6097
A-RIB:GLN-S1
EN 3 0
EN 4 0
EN 5 0
EN 6 -5464
EN 7 -4403
EN 8 -3478
EN 9 -4666
M2G-P:GLU-S2
EN 3 0
EN 4 0
EN 5 0
EN 6 0
EN 7 -14272
EN 8 0
EN 9 0
QUO-RIB:LEU-S1
EN 3 0
EN 4 0
EN 5 0
EN 6 0
EN 7 0
EN 8 -14629
EN 9 0
C-P:TRP-S1
EN 3 0
EN 4 0
EN 5 0
EN 6 -1598
EN 7 -4976
EN 8 -5118
EN 9 -1804
G-RIB:LEU-CA
EN 3 0

EN 4   0
EN 5   2146
EN 6   -1354
EN 7   -1628
EN 8   -1861
EN 9   -2444
G-P:TRP-CA
EN 3   0
EN 4   0
EN 5   -5255
EN 6   -3934
EN 7   -1868
EN 8   -4471
EN 9   -3136
A-R6:HIS-S1
EN 3   0
EN 4   -8739
EN 5   -5035
EN 6   -4267
EN 7   -4040
EN 8   -4634
EN 9   -3761
U-Y:ASP-S1
EN 3   0
EN 4   0
EN 5   -72
EN 6   1248
EN 7   -1124
EN 8   -2010
EN 9   -2542
A-P:THR-CA
EN 3   0
EN 4   0
EN 5   -4960
EN 6   -4428
EN 7   -3781
EN 8   -4375
EN 9   -4578
G-R6:TYR-S2
EN 3   0
EN 4   -5734
EN 5   -2583
EN 6   -3431
EN 7   352
EN 8   -2858
EN 9   -3422
U-RIB:LEU-S2
EN 3   0
EN 4   0
EN 5   -4005
EN 6   -2684
EN 7   -2743
EN 8   -499
EN 9   -1173
C31-RIB:PHE-S1
EN 3   0
EN 4   0
EN 5   0

EN 6   -17189
EN 7   -16127
EN 8   0
EN 9   0
A-RIB:ASN-S2
EN 3   0
EN 4   0
EN 5   -6945
EN 6   -5440
EN 7   -4180
EN 8   -4444
EN 9   -5499
U31-P:ARG-S2
EN 3   0
EN 4   0
EN 5   0
EN 6   0
EN 7   0
EN 8   -13564
EN 9   0
U-RIB:GLN-S2
EN 3   0
EN 4   -6847
EN 5   -6578
EN 6   -5257
EN 7   -5523
EN 8   -5082
EN 9   -4751
U-RIB:TYR-CA
EN 3   0
EN 4   0
EN 5   -1635
EN 6   -314
EN 7   -3241
EN 8   -3835
EN 9   -3740
DA-M6:SER-CA
EN 3   0
EN 4   0
EN 5   -17570
EN 6   0
EN 7   0
EN 8   0
EN 9   -13021
FHU-P:LEU-CA
EN 3   0
EN 4   0
EN 5   0
EN 6   -13379
EN 7   0
EN 8   -12912
EN 9   -11868
A-RIB:SER-S1
EN 3   0
EN 4   0
EN 5   -5010
EN 6   -5540
EN 7   -4053

EN 8   -4487
EN 9   -4503
C-Y:GLU-S1
EN 3   0
EN 4   0
EN 5   -179
EN 6   2859
EN 7   -1231
EN 8   -897
EN 9   -1887
U-Y:TRP-CA
EN 3   0
EN 4   0
EN 5   0
EN 6   -2512
EN 7   -4173
EN 8   -327
EN 9   -2006
A-R6:ARG-S1
EN 3   0
EN 4   0
EN 5   -4206
EN 6   -4895
EN 7   -3027
EN 8   -3207
EN 9   -4554
U-P:PHE-S2
EN 3   0
EN 4   0
EN 5   0
EN 6   -4781
EN 7   -1621
EN 8   -3219
EN 9   -3441
G-RIB:ASP-S1
EN 3   0
EN 4   0
EN 5   -3566
EN 6   -3365
EN 7   -2519
EN 8   -2259
EN 9   -4021
C-Y:ALA-CA
EN 3   0
EN 4   0
EN 5   -122
EN 6   -2236
EN 7   -2377
EN 8   -2194
EN 9   -2442
A-R5:SER-S1
EN 3   0
EN 4   0
EN 5   -1283
EN 6   -3689
EN 7   -4479
EN 8   -3690
EN 9   -4277

G-RIB:GLN-S1
EN 3 0
EN 4 0
EN 5 -4890
EN 6 -5415
EN 7 -4981
EN 8 -4221
EN 9 -4027
G-R5:ARG-CA
EN 3 0
EN 4 0
EN 5 -211
EN 6 -2325
EN 7 -1264
EN 8 -2647
EN 9 -3962
C31-P:ASP-S1
EN 3 0
EN 4 0
EN 5 0
EN 6 -15988
EN 7 -14926
EN 8 0
EN 9 -12759
C-P:LEU-CA
EN 3 0
EN 4 0
EN 5 -988
EN 6 1336
EN 7 -2041
EN 8 -2833
EN 9 -2380
A-RIB:LYS-S2
EN 3 0
EN 4 0
EN 5 -5185
EN 6 -5555
EN 7 -6529
EN 8 -5540
EN 9 -5273
C31-P:ALA-CA
EN 3 0
EN 4 0
EN 5 0
EN 6 0
EN 7 0
EN 8 -13060
EN 9 0
C-RIB:PRO-CA
EN 3 0
EN 4 -5269
EN 5 -3283
EN 6 -4818
EN 7 -5124
EN 8 -4001
EN 9 -3807
C-Y:ILE-S1
EN 3 0

EN 4 0
EN 5 607
EN 6 211
EN 7 -997
EN 8 -1330
EN 9 -1552
U-Y:ARG-CA
EN 3 0
EN 4 -5242
EN 5 178
EN 6 -1222
EN 7 -3596
EN 8 -2854
EN 9 -3780
C-Y:SER-S1
EN 3 0
EN 4 0
EN 5 -3397
EN 6 -4443
EN 7 -3566
EN 8 -4159
EN 9 -4381
C31-MY:PHE-CA
EN 3 0
EN 4 0
EN 5 0
EN 6 0
EN 7 -16127
EN 8 0
EN 9 0
U31-MY:PHE-S1
EN 3 0
EN 4 0
EN 5 0
EN 6 0
EN 7 -16127
EN 8 0
EN 9 0
C-Y:TYR-CA
EN 3 0
EN 4 0
EN 5 -711
EN 6 -3830
EN 7 -1764
EN 8 -1645
EN 9 -2319
U-P:LYS-CA
EN 3 0
EN 4 0
EN 5 -4186
EN 6 -4131
EN 7 -5023
EN 8 -5380
EN 9 -5126
U-Y:ILE-S1
EN 3 0
EN 4 0
EN 5 -2033

EN 6   -1717
EN 7   -655
EN 8   -1108
EN 9   -1471
C-P:ILE-CA
EN 3   0
EN 4   0
EN 5   0
EN 6   -803
EN 7   -2724
EN 8   -3191
EN 9   -3108
U-P:GLU-S2
EN 3   0
EN 4   0
EN 5   0
EN 6   -2089
EN 7   -4202
EN 8   -3078
EN 9   -3460
A-R6:THR-S1
EN 3   0
EN 4   -5227
EN 5   -3793
EN 6   -4426
EN 7   -2867
EN 8   -2456
EN 9   -3744
U-P:MET-S2
EN 3   0
EN 4   0
EN 5   0
EN 6   -4431
EN 7   -4374
EN 8   -4516
EN 9   -2589
DA-M5:TYR-S1
EN 3   0
EN 4   0
EN 5   0
EN 6   0
EN 7   0
EN 8   -17084
EN 9   0
C-P:GLN-S2
EN 3   0
EN 4   0
EN 5   -7051
EN 6   -6486
EN 7   -4668
EN 8   -5669
EN 9   -4700
U-RIB:GLU-S1
EN 3   0
EN 4   0
EN 5   -2107
EN 6   -2052
EN 7   -2155

EN 8 -319
EN 9 -541
U-P:HIS-S1
EN 3 0
EN 4 0
EN 5 0
EN 6 -5039
EN 7 -4430
EN 8 -6028
EN 9 -5482
QUO-RIB:PHE-S2
EN 3 0
EN 4 0
EN 5 0
EN 6 -17192
EN 7 0
EN 8 0
EN 9 -13964
C31-P:ALA-S1
EN 3 0
EN 4 0
EN 5 0
EN 6 0
EN 7 0
EN 8 -13060
EN 9 0
A-P:CYS-CA
EN 3 0
EN 4 0
EN 5 -3564
EN 6 -2243
EN 7 -1181
EN 8 -58
EN 9 -3836
QUO-M6:GLN-S1
EN 3 0
EN 4 0
EN 5 0
EN 6 0
EN 7 0
EN 8 0
EN 9 -14083
DA-M6:TYR-S1
EN 3 0
EN 4 0
EN 5 0
EN 6 0
EN 7 -18207
EN 8 0
EN 9 0
C-Y:ASP-S1
EN 3 0
EN 4 0
EN 5 -2583
EN 6 -1815
EN 7 -3475
EN 8 -3185
EN 9 -3336

A-P:PRO-S1
EN 3 0
EN 4 0
EN 5 -5159
EN 6 -4130
EN 7 -3069
EN 8 -4160
EN 9 -4659
U-Y:GLN-CA
EN 3 0
EN 4 0
EN 5 -1395
EN 6 -74
EN 7 -1735
EN 8 -2936
EN 9 -3385
U-Y:MET-S2
EN 3 0
EN 4 0
EN 5 -2312
EN 6 -2708
EN 7 -3364
EN 8 -524
EN 9 -2915
C-P:THR-S1
EN 3 0
EN 4 0
EN 5 -6678
EN 6 -4279
EN 7 -4296
EN 8 -5099
EN 9 -4701
IU-P:SER-CA
EN 3 0
EN 4 0
EN 5 0
EN 6 0
EN 7 0
EN 8 0
EN 9 -10299
C-RIB:HIS-S1
EN 3 0
EN 4 -8583
EN 5 -4879
EN 6 -5828
EN 7 -3883
EN 8 -5233
EN 9 -4870
A-R6:TYR-CA
EN 3 0
EN 4 0
EN 5 -868
EN 6 -3535
EN 7 -2925
EN 8 -3467
EN 9 -3339
U-Y:THR-CA
EN 3 0

```
EN 4   0
EN 5   -2290
EN 6   -3691
EN 7   -2630
EN 8   -502
EN 9   -2620
C-P:VAL-S1
EN 3   0
EN 4   0
EN 5   -1427
EN 6   -4254
EN 7   -2678
EN 8   -3627
EN 9   -3946
G-RIB:TRP-S1
EN 3   0
EN 4   -7927
EN 5   -4223
EN 6   -2903
EN 7   -3559
EN 8   -5355
EN 9   -1944
U34-P:TYR-CA
EN 3   0
EN 4   0
EN 5   0
EN 6   0
EN 7   0
EN 8   0
EN 9   -16040
U-P:SER-S1
EN 3   0
EN 4   -9747
EN 5   -6279
EN 6   -3838
EN 7   -5724
EN 8   -4998
EN 9   -4375
A-P:TYR-S1
EN 3   0
EN 4   0
EN 5   -3593
EN 6   -1267
EN 7   -3310
EN 8   -4075
EN 9   -3341
G-R5:LYS-CA
EN 3   0
EN 4   -3836
EN 5   1585
EN 6   -528
EN 7   -1917
EN 8   -2884
EN 9   -4091
A-RIB:PRO-CA
EN 3   0
EN 4   0
EN 5   -2727
```

EN 6   -3836
EN 7   -4642
EN 8   -3368
EN 9   -4119
U-RIB:LEU-S1
EN 3   0
EN 4   0
EN 5   -2615
EN 6   -1847
EN 7   -1620
EN 8   -1616
EN 9   -986
FHU-RIB:SER-CA
EN 3   0
EN 4   0
EN 5   0
EN 6   0
EN 7   0
EN 8   -14064
EN 9   0
GTP-RIB:ARG-CA
EN 3   0
EN 4   0
EN 5   0
EN 6   0
EN 7   0
EN 8   -13552
EN 9   0
GTP-M5:ASN-S1
EN 3   0
EN 4   0
EN 5   0
EN 6   0
EN 7   0
EN 8   0
EN 9   -13840
A-RIB:TRP-S2
EN 3   0
EN 4   0
EN 5   -4783
EN 6   -4467
EN 7   -5836
EN 8   -2282
EN 9   -4458
G-R6:PRO-S1
EN 3   0
EN 4   0
EN 5   -2880
EN 6   -3829
EN 7   -2768
EN 8   -2607
EN 9   -2057
A-R6:CYS-CA
EN 3   0
EN 4   0
EN 5   -3561
EN 6   0
EN 7   -1179

EN 8   -1773
EN 9   -729
H2U-P:PRO-S1
EN 3   0
EN 4   0
EN 5   0
EN 6   0
EN 7   0
EN 8   0
EN 9   -9472
QUO-M5:ASP-CA
EN 3   0
EN 4   0
EN 5   0
EN 6   0
EN 7   0
EN 8   0
EN 9   -14474
FMU-P:ARG-S1
EN 3   0
EN 4   0
EN 5   0
EN 6   0
EN 7   0
EN 8   -13552
EN 9   -14226
QUO-M6:LYS-S2
EN 3   0
EN 4   0
EN 5   0
EN 6   0
EN 7   0
EN 8   0
EN 9   -12483
G-RIB:GLN-S2
EN 3   0
EN 4   -5520
EN 5   -6969
EN 6   -5446
EN 7   -5260
EN 8   -4666
EN 9   -4058
FHU-P:LYS-CA
EN 3   0
EN 4   0
EN 5   -15261
EN 6   -13940
EN 7   0
EN 8   -11755
EN 9   -10712
G-P:CYS-CA
EN 3   0
EN 4   0
EN 5   0
EN 6   0
EN 7   0
EN 8   -2244
EN 9   -1913

G-R6:SER-CA
EN 3 0
EN 4 0
EN 5 993
EN 6 -2124
EN 7 -2780
EN 8 -3734
EN 9 -2000
G-RIB:GLU-CA
EN 3 0
EN 4 0
EN 5 -773
EN 6 1551
EN 7 -490
EN 8 -1565
EN 9 -2364
G-R6:LEU-S1
EN 3 0
EN 4 -3275
EN 5 2146
EN 6 -520
EN 7 88
EN 8 -504
EN 9 156
A-R6:CYS-S1
EN 3 0
EN 4 0
EN 5 0
EN 6 -4962
EN 7 -1179
EN 8 -1773
EN 9 0
QUO-M5:ARG-CA
EN 3 0
EN 4 0
EN 5 0
EN 6 0
EN 7 0
EN 8 0
EN 9 -12509
DA-M6:GLN-S1
EN 3 0
EN 4 0
EN 5 0
EN 6 0
EN 7 0
EN 8 0
EN 9 -14083
A-R5:VAL-S1
EN 3 0
EN 4 -4274
EN 5 -2287
EN 6 -1971
EN 7 -1914
EN 8 -1503
EN 9 -1846
QUO-M6:LEU-S2
EN 3 0

EN 4 0
EN 5 0
EN 6 -15100
EN 7 0
EN 8 0
EN 9 -11871
A-R5:PHE-S1
EN 3 0
EN 4 0
EN 5 -2223
EN 6 -902
EN 7 158
EN 8 1282
EN 9 -2113
G-R6:PRO-CA
EN 3 0
EN 4 0
EN 5 554
EN 6 -3276
EN 7 -1502
EN 8 -3234
EN 9 -2022
C-RIB:PHE-S2
EN 3 0
EN 4 0
EN 5 -3074
EN 6 -3471
EN 7 -3911
EN 8 -1999
EN 9 -955
U-Y:LEU-S1
EN 3 0
EN 4 0
EN 5 0
EN 6 -582
EN 7 -2242
EN 8 -1119
EN 9 -1651
C-P:PRO-S1
EN 3 0
EN 4 -5279
EN 5 -6015
EN 6 -4694
EN 7 -4014
EN 8 -4608
EN 9 -4117
U-Y:VAL-CA
EN 3 0
EN 4 0
EN 5 0
EN 6 -14
EN 7 42
EN 8 -2053
EN 9 -1607
A-R5:MET-S1
EN 3 0
EN 4 -6955
EN 5 -1534

EN 6   -2935
EN 7   -2586
EN 8   -3472
EN 9   -1424
U31-RIB:ASP-S2
EN 3   0
EN 4   0
EN 5   0
EN 6   -17750
EN 7   -14972
EN 8   -13848
EN 9   -12804
C-Y:MET-CA
EN 3   0
EN 4   0
EN 5   -1377
EN 6   0
EN 7   -2430
EN 8   -593
EN 9   -1980
U-Y:ASP-S2
EN 3   0
EN 4   0
EN 5   -117
EN 6   1203
EN 7   -2887
EN 8   -3481
EN 9   -2437
A-R6:LEU-CA
EN 3   0
EN 4   0
EN 5   1586
EN 6   -527
EN 7   -1475
EN 8   -2451
EN 9   -1255
G-R6:ARG-S1
EN 3   0
EN 4   -5633
EN 5   1505
EN 6   -1994
EN 7   -4087
EN 8   -3497
EN 9   -3994
A-R5:LEU-CA
EN 3   0
EN 4   0
EN 5   0
EN 6   184
EN 7   -852
EN 8   -1446
EN 9   -1159
C-Y:ASN-S1
EN 3   0
EN 4   0
EN 5   -228
EN 6   -2342
EN 7   -3551

EN 8 -3432
EN 9 -4464
C-P:GLN-CA
EN 3 0
EN 4 0
EN 5 -4469
EN 6 -3982
EN 7 -4256
EN 8 -5048
EN 9 -5009
U31-MY:ILE-CA
EN 3 0
EN 4 0
EN 5 0
EN 6 0
EN 7 -15170
EN 8 0
EN 9 0
U-Y:ASN-CA
EN 3 0
EN 4 0
EN 5 -1152
EN 6 168
EN 7 -3209
EN 8 -4356
EN 9 -4829
A-R6:GLY-CA
EN 3 0
EN 4 -4371
EN 5 -2937
EN 6 -4498
EN 7 -4226
EN 8 -4644
EN 9 -4317
A-P:HIS-S2
EN 3 0
EN 4 0
EN 5 -6442
EN 6 -6241
EN 7 -5948
EN 8 -5410
EN 9 -4493
U31-P:GLN-S2
EN 3 0
EN 4 0
EN 5 -18663
EN 6 0
EN 7 0
EN 8 0
EN 9 0
C-Y:LYS-S2
EN 3 0
EN 4 -4292
EN 5 -4812
EN 6 -4420
EN 7 -4147
EN 8 -3952
EN 9 -5525

FHU-RIB:THR-CA
EN 3 0
EN 4 0
EN 5 0
EN 6 -14771
EN 7 -15427
EN 8 0
EN 9 0
A-R6:HIS-CA
EN 3 0
EN 4 0
EN 5 -1601
EN 6 -5724
EN 7 -2653
EN 8 -3247
EN 9 -5100
DA-RIB:LYS-S2
EN 3 0
EN 4 0
EN 5 0
EN 6 0
EN 7 0
EN 8 -13526
EN 9 0
A-R6:ASP-CA
EN 3 0
EN 4 -4724
EN 5 697
EN 6 -703
EN 7 -1359
EN 8 -1953
EN 9 -1622
U-Y:TRP-S1
EN 3 0
EN 4 0
EN 5 0
EN 6 -5234
EN 7 -1451
EN 8 -2045
EN 9 -2719
U-P:ASP-CA
EN 3 0
EN 4 0
EN 5 -3509
EN 6 -2188
EN 7 -1680
EN 8 -3438
EN 9 -3221
G-R6:LEU-S2
EN 3 0
EN 4 -3278
EN 5 -2678
EN 6 29
EN 7 2807
EN 8 204
EN 9 -603
H2U-MY:LEU-S2
EN 3 0

EN 4   0
EN 5   0
EN 6   0
EN 7   -10051
EN 8   0
EN 9   0
U-Y:ASP-CA
EN 3   0
EN 4   0
EN 5   0
EN 6   1251
EN 7   -409
EN 8   -1385
EN 9   -1677
G-P:LYS-CA
EN 3   0
EN 4   0
EN 5   -5985
EN 6   -4890
EN 7   -4568
EN 8   -5824
EN 9   -4841
C-P:ASP-CA
EN 3   0
EN 4   0
EN 5   -2590
EN 6   -1269
EN 7   -2478
EN 8   -4236
EN 9   -4038
C-P:ARG-S2
EN 3   0
EN 4   -7062
EN 5   -9984
EN 6   -7572
EN 7   -6279
EN 8   -6450
EN 9   -6211
C-Y:GLN-CA
EN 3   0
EN 4   0
EN 5   0
EN 6   -1873
EN 7   -811
EN 8   -1787
EN 9   -3691
A-RIB:HIS-CA
EN 3   0
EN 4   0
EN 5   -1601
EN 6   -5985
EN 7   -5574
EN 8   -5115
EN 9   -4820
DA-M6:TYR-CA
EN 3   0
EN 4   0
EN 5   0

EN 6   -17551
EN 7   0
EN 8   -15367
EN 9   0
A-P:PHE-S2
EN 3   0
EN 4   0
EN 5   0
EN 6   -2626
EN 7   -2951
EN 8   -3716
EN 9   -2832
FMU-MY:GLN-CA
EN 3   0
EN 4   0
EN 5   0
EN 6   0
EN 7   -16250
EN 8   0
EN 9   0
C-RIB:ALA-S1
EN 3   0
EN 4   -5543
EN 5   -5566
EN 6   -5059
EN 7   -3042
EN 8   -2773
EN 9   -3498
IU-MY:LYS-S1
EN 3   0
EN 4   0
EN 5   0
EN 6   -11440
EN 7   -11383
EN 8   -11525
EN 9   -10482
QUO-P:LEU-CA
EN 3   0
EN 4   0
EN 5   0
EN 6   0
EN 7   0
EN 8   0
EN 9   -13586
A-RIB:HIS-S2
EN 3   0
EN 4   0
EN 5   -5605
EN 6   -7166
EN 7   -6531
EN 8   -4981
EN 9   -5141
FHU-MY:TYR-CA
EN 3   0
EN 4   0
EN 5   -18872
EN 6   0
EN 7   0

EN 8   0
EN 9   -14323
C-RIB:ARG-S2
EN 3   0
EN 4   -6048
EN 5   -6071
EN 6   -7733
EN 7   -6982
EN 8   -6789
EN 9   -6764
DA-M6:LYS-S1
EN 3   0
EN 4   0
EN 5   0
EN 6   0
EN 7   -14602
EN 8   0
EN 9   0
U-Y:MET-CA
EN 3   0
EN 4   0
EN 5   -2301
EN 6   -2697
EN 7   -2641
EN 8   -512
EN 9   -2191
A-P:LEU-S1
EN 3   0
EN 4   0
EN 5   -1138
EN 6   -1535
EN 7   -473
EN 8   -1265
EN 9   -1525
A-RIB:LEU-S1
EN 3   0
EN 4   0
EN 5   -1135
EN 6   -2245
EN 7   -2188
EN 8   -1777
EN 9   -1407
QUO-M6:GLU-CA
EN 3   0
EN 4   0
EN 5   0
EN 6   0
EN 7   0
EN 8   0
EN 9   -12066
G-R5:HIS-S2
EN 3   0
EN 4   -8196
EN 5   0
EN 6   -5181
EN 7   -4560
EN 8   -5321
EN 9   -5153

G-P:GLU-CA
EN 3 0
EN 4 0
EN 5 1921
EN 6 519
EN 7 1581
EN 8 -1868
EN 9 -2790
C-Y:PHE-S2
EN 3 0
EN 4 -7491
EN 5 -352
EN 6 -749
EN 7 -1958
EN 8 -834
EN 9 -1247
FMU-MY:ASN-CA
EN 3 0
EN 4 0
EN 5 0
EN 6 0
EN 7 0
EN 8 0
EN 9 -13840
FMU-MY:SER-CA
EN 3 0
EN 4 0
EN 5 0
EN 6 0
EN 7 -15188
EN 8 0
EN 9 0
C-RIB:LYS-S2
EN 3 0
EN 4 0
EN 5 -4576
EN 6 -6627
EN 7 -6462
EN 8 -5844
EN 9 -5443
U34-RIB:GLY-CA
EN 3 0
EN 4 0
EN 5 0
EN 6 0
EN 7 0
EN 8 -13448
EN 9 0
A-RIB:ARG-CA
EN 3 0
EN 4 0
EN 5 -2489
EN 6 -4603
EN 7 -4546
EN 8 -4688
EN 9 -5423
GTP-RIB:THR-S1
EN 3 0

EN 4 0
EN 5 0
EN 6 0
EN 7 0
EN 8 0
EN 9 -14978
G-R5:VAL-CA
EN 3 0
EN 4 0
EN 5 0
EN 6 307
EN 7 103
EN 8 393
EN 9 -1037
A-RIB:MET-S2
EN 3 0
EN 4 0
EN 5 -4980
EN 6 -2946
EN 7 -3150
EN 8 -2860
EN 9 -2148
G-R5:MET-CA
EN 3 0
EN 4 0
EN 5 -974
EN 6 346
EN 7 -1314
EN 8 -2289
EN 9 -2581
H2U-P:THR-S1
EN 3 0
EN 4 0
EN 5 0
EN 6 0
EN 7 0
EN 8 0
EN 9 -10990
U-Y:SER-CA
EN 3 0
EN 4 0
EN 5 -333
EN 6 -3000
EN 7 -3301
EN 8 -2914
EN 9 -3692
G-R6:LYS-S1
EN 3 0
EN 4 -5559
EN 5 -2860
EN 6 -534
EN 7 -3058
EN 8 -3502
EN 9 -3132
C-RIB:TRP-S1
EN 3 0
EN 4 0
EN 5 -5339

EN 6   -3306
EN 7   -3962
EN 8   -4450
EN 9   -2799
U-RIB:GLN-CA
EN 3   0
EN 4   0
EN 5   0
EN 6   -5518
EN 7   -4457
EN 8   -2329
EN 9   -4389
5BU-P:ILE-CA
EN 3   0
EN 4   0
EN 5   0
EN 6   -14514
EN 7   0
EN 8   -14047
EN 9   0
C31-MY:GLU-S2
EN 3   0
EN 4   0
EN 5   0
EN 6   0
EN 7   -14272
EN 8   0
EN 9   -12105
QUO-M5:ASN-CA
EN 3   0
EN 4   0
EN 5   0
EN 6   0
EN 7   0
EN 8   -14883
EN 9   0
IU-MY:THR-CA
EN 3   0
EN 4   0
EN 5   0
EN 6   0
EN 7   -11203
EN 8   -11085
EN 9   0
G-P:ALA-CA
EN 3   0
EN 4   -3450
EN 5   -3181
EN 6   -3246
EN 7   -2356
EN 8   -3109
EN 9   -2911
FHU-RIB:ALA-S1
EN 3   0
EN 4   0
EN 5   0
EN 6   -13527
EN 7   0

EN 8   -13060
EN 9   -12016
G-RIB:ASN-S1
EN 3   0
EN 4   0
EN 5   -4049
EN 6   -5666
EN 7   -5586
EN 8   -4746
EN 9   -4155
FHU-P:ALA-CA
EN 3   0
EN 4   0
EN 5   0
EN 6   0
EN 7   -12466
EN 8   0
EN 9   0
G-P:ASN-CA
EN 3   0
EN 4   -5274
EN 5   -5557
EN 6   -5070
EN 7   -4490
EN 8   -4419
EN 9   -4250
A-R5:PRO-S1
EN 3   0
EN 4   -5426
EN 5   -3439
EN 6   -2119
EN 7   -3564
EN 8   -3519
EN 9   -3777
C31-P:ASN-S2
EN 3   0
EN 4   0
EN 5   0
EN 6   0
EN 7   0
EN 8   0
EN 9   -13861
U34-P:PHE-S2
EN 3   0
EN 4   0
EN 5   0
EN 6   0
EN 7   0
EN 8   0
EN 9   -13964
G-P:MET-S1
EN 3   0
EN 4   0
EN 5   -2718
EN 6   319
EN 7   -3058
EN 8   -2939
EN 9   -1604

C-Y:ARG-CA
EN 3   0
EN 4   0
EN 5   0
EN 6   -2729
EN 7   -3810
EN 8   -4030
EN 9   -2935
FHU-MY:ASP-S2
EN 3   0
EN 4   0
EN 5   -17354
EN 6   0
EN 7   0
EN 8   0
EN 9   -11087
C31-RIB:SER-CA
EN 3   0
EN 4   0
EN 5   0
EN 6   0
EN 7   -15188
EN 8   0
EN 9   0
U34-MY:TYR-S2
EN 3   0
EN 4   0
EN 5   0
EN 6   0
EN 7   -16494
EN 8   0
EN 9   0
FMU-MY:GLU-S1
EN 3   0
EN 4   0
EN 5   0
EN 6   0
EN 7   -14240
EN 8   0
EN 9   0
IU-MY:PRO-S1
EN 3   0
EN 4   0
EN 5   0
EN 6   -12464
EN 7   -12407
EN 8   0
EN 9   0
U31-MY:GLU-S2
EN 3   0
EN 4   0
EN 5   0
EN 6   0
EN 7   -14272
EN 8   -13149
EN 9   0
A-R6:GLN-S2
EN 3   0

EN 4   -6080
EN 5   -5098
EN 6   -5279
EN 7   -5296
EN 8   -3863
EN 9   -3653
FHU-P:THR-CA
EN 3   0
EN 4   0
EN 5   0
EN 6   -14771
EN 7   -15427
EN 8   -12586
EN 9   0
C-RIB:GLY-CA
EN 3   0
EN 4   -6936
EN 5   -6088
EN 6   -5638
EN 7   -5729
EN 8   -5260
EN 9   -5131
G-RIB:TRP-S2
EN 3   0
EN 4   0
EN 5   -2506
EN 6   -4620
EN 7   -4945
EN 8   -3822
EN 9   -3109
U-Y:HIS-S1
EN 3   0
EN 4   -9507
EN 5   -2368
EN 6   -2764
EN 7   -3420
EN 8   -4014
EN 9   -3262
C-Y:ASN-S2
EN 3   0
EN 4   0
EN 5   -1967
EN 6   -5283
EN 7   -3019
EN 8   -2094
EN 9   -5291
U-RIB:PRO-S1
EN 3   0
EN 4   0
EN 5   -3494
EN 6   -4895
EN 7   -4546
EN 8   -3621
EN 9   -4566
OMC-RIB:LYS-S2
EN 3   0
EN 4   0
EN 5   0

EN 6 0
EN 7 -10833
EN 8 -9709
EN 9 0
U-Y:LYS-S1
EN 3 0
EN 4 0
EN 5 -3182
EN 6 -1861
EN 7 -3452
EN 8 -3664
EN 9 -4555
G-R6:GLN-CA
EN 3 0
EN 4 0
EN 5 0
EN 6 1252
EN 7 596
EN 8 -1715
EN 9 -3494
C-P:PHE-S1
EN 3 0
EN 4 0
EN 5 0
EN 6 -1760
EN 7 -1411
EN 8 -2794
EN 9 -1514
H2U-RIB:GLU-S1
EN 3 0
EN 4 0
EN 5 0
EN 6 0
EN 7 0
EN 8 -9129
EN 9 0
A-RIB:ILE-S1
EN 3 0
EN 4 0
EN 5 -2271
EN 6 54
EN 7 -2610
EN 8 -1748
EN 9 -1354
C31-MY:THR-S1
EN 3 0
EN 4 0
EN 5 0
EN 6 0
EN 7 0
EN 8 0
EN 9 -13260
G-P:ALA-S1
EN 3 0
EN 4 -6172
EN 5 -4738
EN 6 -3417
EN 7 -2185

EN 8 -2950
EN 9 -2556
G-R5:ILE-CA
EN 3 0
EN 4 0
EN 5 0
EN 6 614
EN 7 0
EN 8 -635
EN 9 -81
A-R6:ALA-S1
EN 3 0
EN 4 -5700
EN 5 -1283
EN 6 -2393
EN 7 2103
EN 8 -1411
EN 9 -2084
FHU-MY:GLN-S2
EN 3 0
EN 4 0
EN 5 0
EN 6 0
EN 7 0
EN 8 0
EN 9 -14114
C-RIB:TRP-CA
EN 3 0
EN 4 0
EN 5 0
EN 6 -2854
EN 7 -4825
EN 8 -4556
EN 9 -1795
G-R6:ASP-S2
EN 3 0
EN 4 0
EN 5 -4495
EN 6 -3174
EN 7 -2564
EN 8 -3711
EN 9 -3471
C-RIB:ASN-CA
EN 3 0
EN 4 0
EN 5 -228
EN 6 -4613
EN 7 -5141
EN 8 -4795
EN 9 -4692
IU-MY:SER-S1
EN 3 0
EN 4 0
EN 5 -13346
EN 6 0
EN 7 0
EN 8 0
EN 9 -9801

C-RIB:GLU-CA
EN 3   0
EN 4   0
EN 5   -172
EN 6   -568
EN 7   -1777
EN 8   -2607
EN 9   -2896
U31-RIB:MET-S1
EN 3   0
EN 4   0
EN 5   0
EN 6   -18217
EN 7   0
EN 8   0
EN 9   -14989
U-RIB:HIS-CA
EN 3   0
EN 4   0
EN 5   -4085
EN 6   -4482
EN 7   -5430
EN 8   -4306
EN 9   -4688
FHU-RIB:ALA-CA
EN 3   0
EN 4   0
EN 5   0
EN 6   -13527
EN 7   0
EN 8   0
EN 9   -12016
A-P:PHE-CA
EN 3   0
EN 4   0
EN 5   0
EN 6   -2622
EN 7   -2565
EN 8   -2446
EN 9   -2979
OMC-P:LYS-CA
EN 3   0
EN 4   0
EN 5   0
EN 6   0
EN 7   0
EN 8   0
EN 9   -8994
H2U-RIB:LYS-S2
EN 3   0
EN 4   0
EN 5   0
EN 6   -11723
EN 7   0
EN 8   0
EN 9   0
G-RIB:LYS-S1
EN 3   0

EN 4   0
EN 5   -2860
EN 6   -4758
EN 7   -5668
EN 8   -4964
EN 9   -4820
G-P:THR-S1
EN 3   0
EN 4   -8129
EN 5   -6142
EN 6   -3893
EN 7   -3910
EN 8   -2305
EN 9   -3460
C-P:HIS-S1
EN 3   0
EN 4   0
EN 5   -4176
EN 6   -4120
EN 7   -4776
EN 8   -5822
EN 9   -3614
A-RIB:CYS-S1
EN 3   0
EN 4   0
EN 5   -5278
EN 6   -2240
EN 7   -1179
EN 8   -4043
EN 9   -1734
G-RIB:SER-CA
EN 3   0
EN 4   0
EN 5   -1728
EN 6   -4980
EN 7   -4498
EN 8   -4068
EN 9   -3943
G-RIB:VAL-CA
EN 3   0
EN 4   0
EN 5   1708
EN 6   -404
EN 7   -1850
EN 8   -1805
EN 9   -1371
U-P:ARG-S1
EN 3   0
EN 4   0
EN 5   -6365
EN 6   -6379
EN 7   -5516
EN 8   -6738
EN 9   -5250
U-Y:ARG-S2
EN 3   0
EN 4   -10076
EN 5   -4985

EN 6   -6553
EN 7   -5524
EN 8   -3750
EN 9   -5074
C-P:GLY-CA
EN 3   0
EN 4   -8211
EN 5   -7466
EN 6   -3836
EN 7   -5367
EN 8   -6027
EN 9   -4883
G-P:MET-S2
EN 3   0
EN 4   0
EN 5   -4447
EN 6   -3126
EN 7   -3069
EN 8   -2658
EN 9   -450
DA-M6:SER-S1
EN 3   0
EN 4   0
EN 5   -17570
EN 6   0
EN 7   0
EN 8   0
EN 9   0
FMU-RIB:ASN-S1
EN 3   0
EN 4   0
EN 5   0
EN 6   0
EN 7   0
EN 8   0
EN 9   -13840
U-RIB:PHE-S1
EN 3   0
EN 4   0
EN 5   -2990
EN 6   -4391
EN 7   -2325
EN 8   -3388
EN 9   -3593
DA-RIB:ASN-S1
EN 3   0
EN 4   0
EN 5   0
EN 6   -17068
EN 7   0
EN 8   0
EN 9   0
FHU-P:LEU-S2
EN 3   0
EN 4   0
EN 5   0
EN 6   0
EN 7   -12321

EN 8   -11197
EN 9   -10154
A-P:GLN-S1
EN 3   0
EN 4   0
EN 5   -4618
EN 6   -4462
EN 7   -3070
EN 8   -4420
EN 9   -4590
C-RIB:ASN-S2
EN 3   0
EN 4   -5671
EN 5   -6604
EN 6   -4081
EN 7   -5410
EN 8   -5086
EN 9   -4941
C31-P:PHE-S2
EN 3   0
EN 4   0
EN 5   0
EN 6   0
EN 7   0
EN 8   -16725
EN 9   0
G-P:GLU-S2
EN 3   0
EN 4   0
EN 5   -1552
EN 6   -2241
EN 7   -3394
EN 8   -2486
EN 9   -3310
H2U-MY:PRO-CA
EN 3   0
EN 4   0
EN 5   0
EN 6   0
EN 7   0
EN 8   -10515
EN 9   -11189
C31-P:GLN-CA
EN 3   0
EN 4   0
EN 5   0
EN 6   0
EN 7   0
EN 8   -15127
EN 9   0
GTP-RIB:ARG-S1
EN 3   0
EN 4   0
EN 5   0
EN 6   0
EN 7   0
EN 8   -13552
EN 9   0

H2U-RIB:GLU-CA
EN 3   0
EN 4   0
EN 5   0
EN 6   0
EN 7   0
EN 8   0
EN 9   -8078
IU-MY:VAL-S1
EN 3   0
EN 4   0
EN 5   0
EN 6   0
EN 7   0
EN 8   0
EN 9   -8083
U31-MY:GLN-CA
EN 3   0
EN 4   0
EN 5   0
EN 6   0
EN 7   0
EN 8   0
EN 9   -14083
U-Y:HIS-CA
EN 3   0
EN 4   0
EN 5   0
EN 6   -3769
EN 7   -3973
EN 8   -2850
EN 9   -2971
U34-MY:ASN-S1
EN 3   0
EN 4   0
EN 5   0
EN 6   0
EN 7   0
EN 8   -14883
EN 9   -13840
A-RIB:MET-S1
EN 3   0
EN 4   0
EN 5   0
EN 6   -4652
EN 7   -3591
EN 8   -2467
EN 9   -3694
G-RIB:HIS-S1
EN 3   0
EN 4   -6462
EN 5   -5480
EN 6   -5604
EN 7   -4600
EN 8   -5165
EN 9   -4005
G-P:VAL-S1
EN 3   0

EN 4 0
EN 5 -37
EN 6 -1820
EN 7 -1878
EN 8 -2688
EN 9 -1843
A-P:GLU-S2
EN 3 0
EN 4 0
EN 5 0
EN 6 -1772
EN 7 -3693
EN 8 -2916
EN 9 -2767
G-RIB:GLY-CA
EN 3 0
EN 4 -5528
EN 5 -6462
EN 6 -5577
EN 7 -5268
EN 8 -4755
EN 9 -4658
A-RIB:PHE-CA
EN 3 0
EN 4 0
EN 5 -505
EN 6 -1907
EN 7 -2111
EN 8 -3869
EN 9 -2666
A-R5:TRP-S1
EN 3 0
EN 4 0
EN 5 0
EN 6 -1745
EN 7 -684
EN 8 -1278
EN 9 -4673
IU-MY:LYS-CA
EN 3 0
EN 4 0
EN 5 -12755
EN 6 0
EN 7 -11377
EN 8 -9249
EN 9 -10476
DA-RIB:ASP-CA
EN 3 0
EN 4 0
EN 5 0
EN 6 -15986
EN 7 0
EN 8 0
EN 9 0
C-RIB:GLN-CA
EN 3 0
EN 4 0
EN 5 -471

EN 6 -3972
EN 7 -5963
EN 8 -4941
EN 9 -4265
U-RIB:MET-S1
EN 3 0
EN 4 0
EN 5 -5736
EN 6 -980
EN 7 -3906
EN 8 -4239
EN 9 -1186
A-P:PRO-CA
EN 3 0
EN 4 0
EN 5 -1725
EN 6 -2674
EN 7 -3980
EN 8 -5165
EN 9 -3885
G-R6:TRP-CA
EN 3 0
EN 4 0
EN 5 -2506
EN 6 -3907
EN 7 -124
EN 8 -2435
EN 9 -3109
C-RIB:MET-CA
EN 3 0
EN 4 0
EN 5 -3094
EN 6 -5500
EN 7 -1717
EN 8 -3023
EN 9 -2984
G-R5:SER-S1
EN 3 0
EN 4 0
EN 5 -3737
EN 6 -2506
EN 7 -2979
EN 8 -3666
EN 9 -3469
G-RIB:MET-S2
EN 3 0
EN 4 0
EN 5 -6137
EN 6 -3652
EN 7 -3042
EN 8 -3834
EN 9 -2377
QUO-M6:ARG-S1
EN 3 0
EN 4 0
EN 5 0
EN 6 0
EN 7 -14676

EN 8   0
EN 9   -12509
U-RIB:ARG-S2
EN 3   0
EN 4   0
EN 5   -6372
EN 6   -7713
EN 7   -6398
EN 8   -6004
EN 9   -5880
C-P:ASN-S1
EN 3   0
EN 4   -5659
EN 5   -6395
EN 6   -4622
EN 7   -6199
EN 8   -5470
EN 9   -4766
DA-M5:LEU-S1
EN 3   0
EN 4   0
EN 5   0
EN 6   0
EN 7   0
EN 8   0
EN 9   -11868
C-P:HIS-S2
EN 3   0
EN 4   0
EN 5   -4905
EN 6   -6859
EN 7   -5627
EN 8   -4675
EN 9   -4690
G-RIB:HIS-CA
EN 3   0
EN 4   0
EN 5   -1041
EN 6   -4541
EN 7   -5489
EN 8   -4245
EN 9   -4233
C-RIB:THR-CA
EN 3   0
EN 4   0
EN 5   -2371
EN 6   -4485
EN 7   -5523
EN 8   -4486
EN 9   -4046
FHU-P:TYR-S2
EN 3   0
EN 4   0
EN 5   0
EN 6   0
EN 7   0
EN 8   -16375
EN 9   -12610

FHU-MY:VAL-S1
EN 3   0
EN 4   0
EN 5   0
EN 6   0
EN 7   0
EN 8   -11633
EN 9   -12307
G-P:ILE-S1
EN 3   0
EN 4   0
EN 5   -733
EN 6   -1129
EN 7   -1073
EN 8   -2379
EN 9   -2632
H2U-RIB:ARG-S1
EN 3   0
EN 4   0
EN 5   0
EN 6   0
EN 7   0
EN 8   0
EN 9   -8521
FHU-P:VAL-CA
EN 3   0
EN 4   0
EN 5   0
EN 6   0
EN 7   0
EN 8   0
EN 9   -12305
C-P:MET-S2
EN 3   0
EN 4   0
EN 5   -3115
EN 6   -4065
EN 7   -4721
EN 8   -4431
EN 9   -2292
FHU-MY:GLY-CA
EN 3   0
EN 4   0
EN 5   0
EN 6   0
EN 7   0
EN 8   0
EN 9   -12404
5BU-MY:PRO-S1
EN 3   0
EN 4   0
EN 5   0
EN 6   0
EN 7   0
EN 8   0
EN 9   -11742
FHU-P:CYS-S1
EN 3   0

EN 4 0
EN 5 0
EN 6 0
EN 7 0
EN 8 0
EN 9 -15298
U34-RIB:ASN-S2
EN 3 0
EN 4 0
EN 5 0
EN 6 -18807
EN 7 0
EN 8 -14904
EN 9 0
U34-P:ARG-S2
EN 3 0
EN 4 0
EN 5 0
EN 6 0
EN 7 -14688
EN 8 0
EN 9 0
G-R5:LEU-S2
EN 3 0
EN 4 0
EN 5 -2678
EN 6 -975
EN 7 4525
EN 8 -508
EN 9 -1181
G-RIB:LYS-S2
EN 3 0
EN 4 0
EN 5 -5007
EN 6 -6026
EN 7 -6612
EN 8 -5873
EN 9 -5420
A-R5:ARG-CA
EN 3 0
EN 4 0
EN 5 0
EN 6 -2554
EN 7 -3381
EN 8 -3207
EN 9 -4690
A-R6:VAL-CA
EN 3 0
EN 4 0
EN 5 1148
EN 6 -1517
EN 7 -3007
EN 8 -1286
EN 9 -1463
H2U-P:GLU-S1
EN 3 0
EN 4 0
EN 5 0

EN 6   0
EN 7   0
EN 8   0
EN 9   -9803
U-P:ILE-CA
EN 3   0
EN 4   0
EN 5   0
EN 6   1000
EN 7   -1926
EN 8   -249
EN 9   -2791
C-P:TYR-S2
EN 3   0
EN 4   0
EN 5   -3447
EN 6   -5110
EN 7   -4048
EN 8   -2372
EN 9   -3598
A-R5:MET-CA
EN 3   0
EN 4   0
EN 5   -1534
EN 6   -1930
EN 7   -3139
EN 8   -1463
EN 9   -3523
U34-P:GLU-CA
EN 3   0
EN 4   0
EN 5   0
EN 6   0
EN 7   0
EN 8   0
EN 9   -12066
C-Y:VAL-CA
EN 3   0
EN 4   0
EN 5   1305
EN 6   -2525
EN 7   253
EN 8   -340
EN 9   -2019
G-P:ASN-S2
EN 3   0
EN 4   0
EN 5   -6743
EN 6   -5091
EN 7   -5466
EN 8   -5314
EN 9   -4810
FMU-P:PHE-S1
EN 3   0
EN 4   0
EN 5   -18510
EN 6   0
EN 7   0

EN 8   0
EN 9   0
A-P:HIS-CA
EN 3   0
EN 4   0
EN 5   -1603
EN 6   -4270
EN 7   -3209
EN 8   -5393
EN 9   -4823
A-P:ASP-S2
EN 3   0
EN 4   0
EN 5   -1070
EN 6   -3184
EN 7   -4629
EN 8   -3615
EN 9   -2969
H2U-MY:ARG-CA
EN 3   0
EN 4   0
EN 5   0
EN 6   0
EN 7   0
EN 8   -9565
EN 9   0
U31-MY:ALA-S1
EN 3   0
EN 4   0
EN 5   0
EN 6   0
EN 7   0
EN 8   -13060
EN 9   -12016
G-R5:ASN-CA
EN 3   0
EN 4   0
EN 5   -1212
EN 6   -1939
EN 7   -3651
EN 8   -3339
EN 9   -3554
A-R5:CYS-CA
EN 3   0
EN 4   0
EN 5   0
EN 6   -2240
EN 7   -2896
EN 8   -55
EN 9   987
A-R5:GLN-CA
EN 3   0
EN 4   0
EN 5   -628
EN 6   692
EN 7   -968
EN 8   -3279
EN 9   -3240

DA-RIB:TYR-S1
EN 3 0
EN 4 0
EN 5 0
EN 6 0
EN 7 0
EN 8 0
EN 9 -14323
C-RIB:ASP-S2
EN 3 0
EN 4 -4614
EN 5 -5903
EN 6 -5294
EN 7 -3349
EN 8 -4274
EN 9 -4075
U-P:PRO-S1
EN 3 0
EN 4 0
EN 5 -5598
EN 6 -4900
EN 7 -4099
EN 8 -5145
EN 9 -3886
FMU-MY:MET-S1
EN 3 0
EN 4 0
EN 5 0
EN 6 0
EN 7 0
EN 8 -16032
EN 9 0
5BU-P:THR-CA
EN 3 0
EN 4 0
EN 5 0
EN 6 0
EN 7 0
EN 8 0
EN 9 -11543
U-Y:GLU-CA
EN 3 0
EN 4 0
EN 5 0
EN 6 224
EN 7 1285
EN 8 2409
EN 9 -1368
U-Y:PRO-S1
EN 3 0
EN 4 -7911
EN 5 -2489
EN 6 -2173
EN 7 -4095
EN 8 -3207
EN 9 -3384
A-R5:HIS-S1
EN 3 0

EN 4   -7022
EN 5   -3318
EN 6   -5101
EN 7   -4371
EN 8   -4634
EN 9   -3406
C31-MY:GLN-S1
EN 3   0
EN 4   0
EN 5   0
EN 6   0
EN 7   0
EN 8   0
EN 9   -14083
C31-RIB:TYR-S1
EN 3   0
EN 4   0
EN 5   0
EN 6   0
EN 7   0
EN 8   0
EN 9   -14323
U-Y:PRO-CA
EN 3   0
EN 4   0
EN 5   -2489
EN 6   -2173
EN 7   -3211
EN 8   -3621
EN 9   -3242
U-RIB:MET-S2
EN 3   0
EN 4   0
EN 5   -4029
EN 6   -2708
EN 7   -3917
EN 8   -2241
EN 9   -3468
H2U-RIB:ASN-S2
EN 3   0
EN 4   0
EN 5   0
EN 6   0
EN 7   0
EN 8   -12634
EN 9   -9873
U-RIB:GLU-CA
EN 3   0
EN 4   -4800
EN 5   621
EN 6   224
EN 7   -1818
EN 8   -312
EN 9   -2372
U-RIB:ASN-S1
EN 3   0
EN 4   -6574
EN 5   -3874

EN 6   -5773
EN 7   -5480
EN 8   -5190
EN 9   -5084
G-P:GLN-S2
EN 3   0
EN 4   0
EN 5   -5570
EN 6   -5515
EN 7   -5166
EN 8   -5129
EN 9   -4654
A-R5:TRP-CA
EN 3   0
EN 4   0
EN 5   0
EN 6   -1745
EN 7   -684
EN 8   -1278
EN 9   -4222
I-RIB:TRP-S1
EN 3   0
EN 4   0
EN 5   0
EN 6   0
EN 7   0
EN 8   0
EN 9   -16521
FHU-MY:ILE-CA
EN 3   0
EN 4   0
EN 5   -17553
EN 6   0
EN 7   0
EN 8   0
EN 9   0
IU-P:LEU-CA
EN 3   0
EN 4   0
EN 5   0
EN 6   0
EN 7   0
EN 8   -10190
EN 9   -9146
DA-M5:VAL-S1
EN 3   0
EN 4   0
EN 5   0
EN 6   0
EN 7   0
EN 8   0
EN 9   -12307
U31-P:ASN-S1
EN 3   0
EN 4   0
EN 5   0
EN 6   -17068
EN 7   0

EN 8   0
EN 9   0
H2U-MY:PHE-CA
EN 3   0
EN 4   0
EN 5   0
EN 6   0
EN 7   -12140
EN 8   0
EN 9   0
G-P:LEU-CA
EN 3   0
EN 4   0
EN 5   -1868
EN 6   5
EN 7   -650
EN 8   -1918
EN 9   -2650
G-R5:ARG-S1
EN 3   0
EN 4   -3915
EN 5   -1216
EN 6   -3114
EN 7   -3273
EN 8   -3725
EN 9   -4541
U-RIB:VAL-CA
EN 3   0
EN 4   0
EN 5   0
EN 6   -1019
EN 7   -3889
EN 8   -1264
EN 9   -1722
U-RIB:ILE-CA
EN 3   0
EN 4   0
EN 5   0
EN 6   0
EN 7   -2373
EN 8   -1108
EN 9   -2636
FMU-RIB:GLN-CA
EN 3   0
EN 4   0
EN 5   0
EN 6   0
EN 7   0
EN 8   -15127
EN 9   0
GTP-M5:THR-CA
EN 3   0
EN 4   0
EN 5   0
EN 6   0
EN 7   0
EN 8   0
EN 9   -13260

DA-M5:SER-S1
EN 3 0
EN 4 0
EN 5 -17570
EN 6 0
EN 7 0
EN 8 0
EN 9 -13021
C-P:ALA-S1
EN 3 0
EN 4 -6558
EN 5 -5124
EN 6 -3035
EN 7 -4198
EN 8 -4109
EN 9 -3508
U-RIB:GLY-CA
EN 3 0
EN 4 -6855
EN 5 -6426
EN 6 -5939
EN 7 -5649
EN 8 -5371
EN 9 -5352
C-Y:TRP-S1
EN 3 0
EN 4 0
EN 5 -4175
EN 6 -4019
EN 7 -2244
EN 8 -2125
EN 9 -2348
C-Y:TRP-CA
EN 3 0
EN 4 0
EN 5 -4175
EN 6 -584
EN 7 -3249
EN 8 596
EN 9 -1082
G-R5:LYS-S1
EN 3 0
EN 4 -5559
EN 5 -3573
EN 6 -1539
EN 7 -3461
EN 8 -3321
EN 9 -4353
H2U-P:ASN-S2
EN 3 0
EN 4 0
EN 5 0
EN 6 -13102
EN 7 -13758
EN 8 0
EN 9 0
A-R6:ASP-S1
EN 3 0

EN 4 -4726
EN 5 -1022
EN 6 -2423
EN 7 355
EN 8 -2337
EN 9 -2629
DA-M5:ASP-S2
EN 3 0
EN 4 0
EN 5 0
EN 6 0
EN 7 0
EN 8 0
EN 9 -12804
A-P:ILE-S1
EN 3 0
EN 4 0
EN 5 448
EN 6 -2218
EN 7 -2321
EN 8 -1750
EN 9 -1871
G-R5:VAL-S1
EN 3 0
EN 4 0
EN 5 -10
EN 6 305
EN 7 654
EN 8 -231
EN 9 -1103
FHU-MY:ARG-S1
EN 3 0
EN 4 0
EN 5 0
EN 6 -15737
EN 7 0
EN 8 -13552
EN 9 0
A-RIB:ARG-S2
EN 3 0
EN 4 0
EN 5 -6086
EN 6 -7674
EN 7 -6988
EN 8 -6448
EN 9 -5693
U-P:LYS-S2
EN 3 0
EN 4 -12091
EN 5 -8877
EN 6 -5499
EN 7 -5292
EN 8 -5495
EN 9 -5668
DA-M6:THR-S1
EN 3 0
EN 4 0
EN 5 0

EN 6 0
EN 7 0
EN 8 -14304
EN 9 0
A-P:LYS-CA
EN 3 0
EN 4 0
EN 5 -5332
EN 6 -3597
EN 7 -4851
EN 8 -4903
EN 9 -4846
A-R5:LYS-S1
EN 3 0
EN 4 -4402
EN 5 -2968
EN 6 -1647
EN 7 -1419
EN 8 -3902
EN 9 -4735
DA-M6:LYS-CA
EN 3 0
EN 4 0
EN 5 0
EN 6 0
EN 7 0
EN 8 -13473
EN 9 0
U31-P:GLN-CA
EN 3 0
EN 4 0
EN 5 0
EN 6 0
EN 7 -16250
EN 8 0
EN 9 0
G-P:TYR-S1
EN 3 0
EN 4 0
EN 5 -335
EN 6 -732
EN 7 -3105
EN 8 -4373
EN 9 -2806
U31-RIB:GLN-CA
EN 3 0
EN 4 0
EN 5 0
EN 6 0
EN 7 -16250
EN 8 0
EN 9 0
A-R6:GLN-S1
EN 3 0
EN 4 0
EN 5 -2346
EN 6 -4460
EN 7 -3398

EN 8   -3661
EN 9   -3240
IU-RIB:ALA-S1
EN 3   0
EN 4   0
EN 5   0
EN 6   0
EN 7   -9959
EN 8   -9840
EN 9   -8797
U-P:TYR-S2
EN 3   0
EN 4   0
EN 5   0
EN 6   -5145
EN 7   -3250
EN 8   -3582
EN 9   -3252
IU-P:LYS-S1
EN 3   0
EN 4   0
EN 5   0
EN 6   -12942
EN 7   0
EN 8   0
EN 9   -9713
A-RIB:GLU-S2
EN 3   0
EN 4   -6794
EN 5   -3090
EN 6   -3271
EN 7   -3138
EN 8   -2014
EN 9   -2609
FMU-MY:ARG-S2
EN 3   0
EN 4   0
EN 5   0
EN 6   0
EN 7   0
EN 8   -13564
EN 9   0
U31-RIB:THR-S1
EN 3   0
EN 4   0
EN 5   0
EN 6   0
EN 7   0
EN 8   0
EN 9   -14978
U-P:GLN-S1
EN 3   0
EN 4   -6821
EN 5   -4122
EN 6   -2801
EN 7   -5373
EN 8   -4764
EN 9   -4509

FMU-P:ILE-CA
EN 3 0
EN 4 0
EN 5 0
EN 6 0
EN 7 0
EN 8 -14047
EN 9 0
G-R5:MET-S1
EN 3 0
EN 4 -6395
EN 5 0
EN 6 -1370
EN 7 -3413
EN 8 -1456
EN 9 -2130
G-R5:THR-S1
EN 3 0
EN 4 -4667
EN 5 -963
EN 6 357
EN 7 -2804
EN 8 -2759
EN 9 -2570
C-P:CYS-CA
EN 3 0
EN 4 0
EN 5 -3414
EN 6 0
EN 7 -1032
EN 8 -2630
EN 9 -2852
C-P:TRP-CA
EN 3 0
EN 4 0
EN 5 0
EN 6 -1598
EN 7 -2254
EN 8 -3853
EN 9 -4311
G-R6:HIS-S2
EN 3 0
EN 4 -9201
EN 5 0
EN 6 -3724
EN 7 -6098
EN 8 -5321
EN 9 -4894
G-P:TRP-S1
EN 3 0
EN 4 0
EN 5 -4251
EN 6 -4647
EN 7 -3586
EN 8 -4180
EN 9 -1971
H2U-RIB:GLY-CA
EN 3 0

EN 4   0
EN 5   0
EN 6   0
EN 7   0
EN 8   -9460
EN 9   0
C-P:LYS-CA
EN 3   0
EN 4   0
EN 5   -5183
EN 6   -5275
EN 7   -5032
EN 8   -5547
EN 9   -4840
H2U-MY:LEU-CA
EN 3   0
EN 4   0
EN 5   0
EN 6   0
EN 7   0
EN 8   0
EN 9   -9598
C-P:ASP-S1
EN 3   0
EN 4   0
EN 5   -3979
EN 6   -1824
EN 7   -3130
EN 8   -4912
EN 9   -4350
I-P:TRP-S2
EN 3   0
EN 4   0
EN 5   0
EN 6   0
EN 7   0
EN 8   0
EN 9   -16521
U-RIB:LYS-CA
EN 3   0
EN 4   0
EN 5   -1459
EN 6   -4126
EN 7   -4782
EN 8   -5249
EN 9   -4248
C-Y:THR-S1
EN 3   0
EN 4   0
EN 5   -2371
EN 6   -3480
EN 7   -2710
EN 8   -3304
EN 9   -3978
C-RIB:LEU-CA
EN 3   0
EN 4   0
EN 5   25

```
EN 6   -2088
EN 7   -1579
EN 8   -3488
EN 9   -3229
G-RIB:THR-S1
EN 3   0
EN 4   0
EN 5   -2680
EN 6   -4794
EN 7   -3883
EN 8   -2901
EN 9   -3882
C-RIB:TYR-S1
EN 3   0
EN 4   0
EN 5   -2429
EN 6   -3378
EN 7   -3376
EN 8   -4225
EN 9   -2872
IU-P:HIS-CA
EN 3   0
EN 4   0
EN 5   -16883
EN 6   0
EN 7   0
EN 8   0
EN 9   0
G-R6:TRP-S2
EN 3   0
EN 4   -9645
EN 5   -5228
EN 6   -2903
EN 7   -4111
EN 8   -2435
EN 9   -3401
G-RIB:LYS-CA
EN 3   0
EN 4   0
EN 5   -2402
EN 6   -3963
EN 7   -4354
EN 8   -4589
EN 9   -4612
DA-M5:LYS-S2
EN 3   0
EN 4   0
EN 5   0
EN 6   -15711
EN 7   0
EN 8   0
EN 9   0
FMU-RIB:CYS-CA
EN 3   0
EN 4   0
EN 5   0
EN 6   0
EN 7   -19183
```

EN 8   0
EN 9   0
FMU-P:ARG-S2
EN 3   0
EN 4   0
EN 5   -17070
EN 6   0
EN 7   0
EN 8   -15282
EN 9   0
FMU-MY:ASP-CA
EN 3   0
EN 4   0
EN 5   0
EN 6   0
EN 7   0
EN 8   -13801
EN 9   0
G-RIB:MET-S1
EN 3   0
EN 4   0
EN 5   0
EN 6   -4092
EN 7   -4533
EN 8   -3409
EN 9   -2581
U-P:ASP-S2
EN 3   0
EN 4   0
EN 5   -2843
EN 6   -4245
EN 7   -4157
EN 8   -3325
EN 9   -3547
C31-RIB:SER-S1
EN 3   0
EN 4   0
EN 5   0
EN 6   0
EN 7   0
EN 8   -14064
EN 9   0
QUO-M6:ARG-S2
EN 3   0
EN 4   0
EN 5   0
EN 6   -15749
EN 7   0
EN 8   -13564
EN 9   0
U-RIB:ARG-S1
EN 3   0
EN 4   0
EN 5   -4642
EN 6   -4657
EN 7   -5208
EN 8   -5634
EN 9   -5665

U-RIB:PRO-CA
EN 3 0
EN 4 0
EN 5 0
EN 6 -3890
EN 7 -3834
EN 8 -5432
EN 9 -3384
A-P:TRP-S1
EN 3 0
EN 4 0
EN 5 -3069
EN 6 -1748
EN 7 -686
EN 8 -5007
EN 9 -1241
DA-RIB:GLN-CA
EN 3 0
EN 4 0
EN 5 0
EN 6 0
EN 7 0
EN 8 -15127
EN 9 0
DA-M5:ASP-S1
EN 3 0
EN 4 0
EN 5 0
EN 6 0
EN 7 0
EN 8 0
EN 9 -12759
GTP-M6:THR-S1
EN 3 0
EN 4 0
EN 5 0
EN 6 0
EN 7 0
EN 8 0
EN 9 -13260
G-P:LEU-S1
EN 3 0
EN 4 0
EN 5 -2320
EN 6 -1712
EN 7 513
EN 8 -2033
EN 9 -2278
FHU-MY:TYR-S1
EN 3 0
EN 4 0
EN 5 0
EN 6 -17551
EN 7 0
EN 8 -15367
EN 9 0
A-P:LYS-S1
EN 3 0

EN 4   -6122
EN 5   -6144
EN 6   -6399
EN 7   -5678
EN 8   -4853
EN 9   -4814
C-P:LEU-S2
EN 3   0
EN 4   0
EN 5   12
EN 6   -2101
EN 7   -3431
EN 8   -705
EN 9   -2797
C-P:THR-CA
EN 3   0
EN 4   -5080
EN 5   -4098
EN 6   -3159
EN 7   -4572
EN 8   -4740
EN 9   -4370
GTP-M5:ALA-S1
EN 3   0
EN 4   0
EN 5   0
EN 6   0
EN 7   0
EN 8   -13060
EN 9   0
A-R6:GLU-S2
EN 3   0
EN 4   0
EN 5   -2638
EN 6   -1317
EN 7   -3927
EN 8   -1854
EN 9   -1524
A-P:ASP-S1
EN 3   0
EN 4   0
EN 5   692
EN 6   -1974
EN 7   -2369
EN 8   -3676
EN 9   -3567
G-RIB:ALA-S1
EN 3   0
EN 4   -5140
EN 5   -3445
EN 6   -3219
EN 7   -1560
EN 8   -2471
EN 9   -3324
A-P:VAL-S1
EN 3   0
EN 4   0
EN 5   -4299

EN 6   -2355
EN 7   -2178
EN 8   -1290
EN 9   -2969
A-RIB:GLU-S1
EN 3   0
EN 4   0
EN 5   -2053
EN 6   -3238
EN 7   -1680
EN 8   -1982
EN 9   -2412
U-P:PRO-CA
EN 3   0
EN 4   -7915
EN 5   -5598
EN 6   -2178
EN 7   -4749
EN 8   -4432
EN 9   -3523
FHU-RIB:ASP-CA
EN 3   0
EN 4   0
EN 5   0
EN 6   0
EN 7   -14924
EN 8   0
EN 9   0
FHU-P:PRO-S1
EN 3   0
EN 4   0
EN 5   0
EN 6   0
EN 7   0
EN 8   -12786
EN 9   0
G-P:TYR-S2
EN 3   0
EN 4   0
EN 5   -3061
EN 6   -5557
EN 7   -4114
EN 8   -1655
EN 9   -3213
C31-MY:LEU-CA
EN 3   0
EN 4   0
EN 5   0
EN 6   -15097
EN 7   0
EN 8   0
EN 9   0
C-Y:ALA-S1
EN 3   0
EN 4   -3826
EN 5   -2844
EN 6   -1523
EN 7   -2732

EN 8   -3155
EN 9   -2664
U-RIB:LYS-S1
EN 3   0
EN 4   0
EN 5   0
EN 6   -5849
EN 7   -5239
EN 8   -4669
EN 9   -5783
C-Y:ARG-S2
EN 3   0
EN 4   -7053
EN 5   -6885
EN 6   -5845
EN 7   -5035
EN 8   -5506
EN 9   -5811
H2U-MY:PRO-S1
EN 3   0
EN 4   0
EN 5   0
EN 6   0
EN 7   -11639
EN 8   0
EN 9   -9472
G-P:TYR-CA
EN 3   0
EN 4   0
EN 5   -2053
EN 6   -732
EN 7   -2774
EN 8   -3185
EN 9   -3329
FHU-RIB:GLY-CA
EN 3   0
EN 4   0
EN 5   0
EN 6   -13915
EN 7   -14571
EN 8   -11730
EN 9   0
FHU-RIB:TYR-S1
EN 3   0
EN 4   0
EN 5   0
EN 6   -15834
EN 7   -14773
EN 8   0
EN 9   0
C-P:TYR-S1
EN 3   0
EN 4   0
EN 5   -721
EN 6   -1118
EN 7   -3160
EN 8   -3925
EN 9   -3830

4SU-P:GLU-S2
EN 3 0
EN 4 0
EN 5 0
EN 6 0
EN 7 0
EN 8 0
EN 9 -12105
C-Y:TRP-S2
EN 3 0
EN 4 -9596
EN 5 -2909
EN 6 -584
EN 7 -4515
EN 8 -3843
EN 9 -4065
FHU-RIB:ARG-S1
EN 3 0
EN 4 0
EN 5 0
EN 6 0
EN 7 0
EN 8 -11835
EN 9 -13513
U31-P:ASN-CA
EN 3 0
EN 4 0
EN 5 0
EN 6 0
EN 7 -16007
EN 8 0
EN 9 0
DA-M6:HIS-S2
EN 3 0
EN 4 0
EN 5 -19622
EN 6 0
EN 7 0
EN 8 0
EN 9 0
FMU-MY:VAL-CA
EN 3 0
EN 4 0
EN 5 0
EN 6 0
EN 7 0
EN 8 -13349
EN 9 -12305
C31-P:MET-CA
EN 3 0
EN 4 0
EN 5 0
EN 6 0
EN 7 0
EN 8 0
EN 9 -14989
DA-RIB:ARG-S1
EN 3 0

EN 4   0
EN 5   0
EN 6   0
EN 7   0
EN 8   0
EN 9   -12509
C31-MY:GLU-CA
EN 3   0
EN 4   0
EN 5   0
EN 6   0
EN 7   0
EN 8   -13110
EN 9   0
A-RIB:PRO-S1
EN 3   0
EN 4   0
EN 5   -1722
EN 6   -5553
EN 7   -3327
EN 8   -4268
EN 9   -4532
IU-RIB:ARG-S2
EN 3   0
EN 4   0
EN 5   0
EN 6   0
EN 7   0
EN 8   -10345
EN 9   0
H2U-MY:TRP-S2
EN 3   0
EN 4   0
EN 5   -17083
EN 6   0
EN 7   0
EN 8   0
EN 9   0
A-R6:LEU-S2
EN 3   0
EN 4   -5555
EN 5   -1139
EN 6   -3634
EN 7   -856
EN 8   -2072
EN 9   -1636
G-P:ILE-CA
EN 3   0
EN 4   0
EN 5   -1738
EN 6   0
EN 7   -621
EN 8   -2805
EN 9   -2340
C-Y:PRO-CA
EN 3   0
EN 4   0
EN 5   -1565

EN 6   -1962
EN 7   -3171
EN 8   -2821
EN 9   -3726
IU-MY:THR-S1
EN 3   0
EN 4   0
EN 5   0
EN 6   0
EN 7   -11203
EN 8   -11085
EN 9   0
A-P:ASN-CA
EN 3   0
EN 4   0
EN 5   -4375
EN 6   -4219
EN 7   -3711
EN 8   -4954
EN 9   -4265
C31-P:GLU-S2
EN 3   0
EN 4   0
EN 5   -16655
EN 6   0
EN 7   0
EN 8   0
EN 9   0
G-R5:ASN-S1
EN 3   0
EN 4   0
EN 5   -4556
EN 6   -2838
EN 7   -3548
EN 8   -3582
EN 9   -4400
DA-RIB:ASN-CA
EN 3   0
EN 4   0
EN 5   0
EN 6   0
EN 7   0
EN 8   -14883
EN 9   0
A-R6:SER-CA
EN 3   0
EN 4   0
EN 5   433
EN 6   -3950
EN 7   -2628
EN 8   -3355
EN 9   -2675
A-R6:TRP-CA
EN 3   0
EN 4   0
EN 5   0
EN 6   0
EN 7   -3406

EN 8   -2282
EN 9   -2504
QUO-M5:LYS-S2
EN 3   0
EN 4   0
EN 5   0
EN 6   0
EN 7   0
EN 8   0
EN 9   -12483
FMU-RIB:PHE-S2
EN 3   0
EN 4   0
EN 5   0
EN 6   -18910
EN 7   0
EN 8   0
EN 9   0
A-P:ALA-S1
EN 3   0
EN 4   -3985
EN 5   -5103
EN 6   -4263
EN 7   -3604
EN 8   -2219
EN 9   -2893
G-R5:PRO-CA
EN 3   0
EN 4   0
EN 5   -1162
EN 6   158
EN 7   -3418
EN 8   -2096
EN 9   -2287
GTP-RIB:ALA-S1
EN 3   0
EN 4   0
EN 5   0
EN 6   0
EN 7   0
EN 8   0
EN 9   -12016
C-P:MET-S1
EN 3   0
EN 4   0
EN 5   -3104
EN 6   -2788
EN 7   -2992
EN 8   -4901
EN 9   -3547
G-RIB:TYR-CA
EN 3   0
EN 4   0
EN 5   0
EN 6   -705
EN 7   -3300
EN 8   -1136
EN 9   -1863

A-R5:GLU-S2
EN 3   0
EN 4   0
EN 5   -368
EN 6   -1769
EN 7   -2807
EN 8   -3120
EN 9   -2174
U31-RIB:TYR-S2
EN 3   0
EN 4   0
EN 5   0
EN 6   0
EN 7   -16494
EN 8   0
EN 9   -14327
U-Y:LYS-CA
EN 3   0
EN 4   0
EN 5   -2464
EN 6   -1856
EN 7   -579
EN 8   -3397
EN 9   -2961
U31-RIB:ASP-CA
EN 3   0
EN 4   0
EN 5   0
EN 6   0
EN 7   -16642
EN 8   -13801
EN 9   -12757
A-RIB:VAL-CA
EN 3   0
EN 4   0
EN 5   1148
EN 6   752
EN 7   -3178
EN 8   -3417
EN 9   -2557
QUO-RIB:LYS-S2
EN 3   0
EN 4   0
EN 5   0
EN 6   0
EN 7   0
EN 8   -13526
EN 9   0
G-R6:ARG-CA
EN 3   0
EN 4   -3915
EN 5   -1216
EN 6   -1994
EN 7   -933
EN 8   -2862
EN 9   -2957
U-P:LEU-S1
EN 3   0

EN 4 0
EN 5 0
EN 6 417
EN 7 -2247
EN 8 -2389
EN 9 -2708
A-R5:PHE-CA
EN 3 0
EN 4 0
EN 5 0
EN 6 -1907
EN 7 158
EN 8 1282
EN 9 -2311
C-Y:PRO-S1
EN 3 0
EN 4 -5269
EN 5 -1565
EN 6 -3679
EN 7 -2910
EN 8 -2821
EN 9 -3642
U-Y:ASN-S2
EN 3 0
EN 4 -8312
EN 5 -2890
EN 6 -5717
EN 7 -5501
EN 8 -4687
EN 9 -5211
G-RIB:ILE-CA
EN 3 0
EN 4 0
EN 5 -706
EN 6 -1102
EN 7 -1046
EN 8 -2644
EN 9 -1422
U31-P:MET-S2
EN 3 0
EN 4 0
EN 5 0
EN 6 -18228
EN 7 0
EN 8 0
EN 9 -15000
G-P:SER-S1
EN 3 0
EN 4 -7889
EN 5 -6053
EN 6 -4251
EN 7 -4419
EN 8 -4540
EN 9 -4709
C-Y:HIS-S1
EN 3 0
EN 4 0
EN 5 -1444

EN 6   -4944
EN 7   -3883
EN 8   -4095
EN 9   -4769
FMU-MY:PRO-CA
EN 3   0
EN 4   0
EN 5   0
EN 6   0
EN 7   0
EN 8   -14503
EN 9   0
DA-M5:MET-S2
EN 3   0
EN 4   0
EN 5   0
EN 6   0
EN 7   0
EN 8   -16043
EN 9   0
G-R5:PHE-S1
EN 3   0
EN 4   0
EN 5   0
EN 6   -342
EN 7   -998
EN 8   837
EN 9   -1703
C31-RIB:LEU-S1
EN 3   0
EN 4   0
EN 5   0
EN 6   0
EN 7   -14035
EN 8   0
EN 9   0
IU-MY:LYS-S2
EN 3   0
EN 4   0
EN 5   0
EN 6   -13758
EN 7   -12696
EN 8   -11573
EN 9   0
U-Y:LEU-S2
EN 3   0
EN 4   0
EN 5   815
EN 6   -1850
EN 7   -3340
EN 8   -117
EN 9   -791
U-RIB:HIS-S1
EN 3   0
EN 4   0
EN 5   -5090
EN 6   -5486
EN 7   -5430

EN 8   -5401
EN 9   -4528
G-R5:MET-S2
EN 3   0
EN 4   -6406
EN 5   -985
EN 6   -3652
EN 7   -3042
EN 8   -3636
EN 9   -2141
U-RIB:TYR-S1
EN 3   0
EN 4   0
EN 5   0
EN 6   -2032
EN 7   -3692
EN 8   -3994
EN 9   -3243
C-RIB:GLU-S1
EN 3   0
EN 4   -3883
EN 5   -1896
EN 6   -2293
EN 7   -2618
EN 8   -2117
EN 9   -2625
U-P:ALA-CA
EN 3   0
EN 4   0
EN 5   -4777
EN 6   -4169
EN 7   -2656
EN 8   -4083
EN 9   -4103
IU-MY:ARG-S1
EN 3   0
EN 4   0
EN 5   -13839
EN 6   0
EN 7   0
EN 8   0
EN 9   -9289
U-RIB:MET-CA
EN 3   0
EN 4   0
EN 5   -5023
EN 6   -4415
EN 7   -3906
EN 8   -2783
EN 9   -2049
A-P:ILE-CA
EN 3   0
EN 4   0
EN 5   448
EN 6   -1665
EN 7   108
EN 8   -2584
EN 9   -1711

IU-MY:GLU-S2
EN 3 0
EN 4 0
EN 5 0
EN 6 0
EN 7 0
EN 8 -8925
EN 9 0
FHU-P:TYR-CA
EN 3 0
EN 4 0
EN 5 0
EN 6 0
EN 7 0
EN 8 -13649
EN 9 -14323
G-R5:PHE-CA
EN 3 0
EN 4 0
EN 5 0
EN 6 0
EN 7 718
EN 8 -427
EN 9 -2106
C-Y:LEU-S1
EN 3 0
EN 4 0
EN 5 1742
EN 6 -923
EN 7 -314
EN 8 -195
EN 9 -1873
IU-MY:ARG-S2
EN 3 0
EN 4 0
EN 5 -13851
EN 6 0
EN 7 -11469
EN 8 0
EN 9 -11019
C-Y:ARG-S1
EN 3 0
EN 4 0
EN 5 -2332
EN 6 -4115
EN 7 -4942
EN 8 -4749
EN 9 -5384
IU-MY:HIS-CA
EN 3 0
EN 4 0
EN 5 0
EN 6 0
EN 7 0
EN 8 0
EN 9 -13102
A-R6:ASN-S1
EN 3 0

EN 4   0
EN 5   -3820
EN 6   -3886
EN 7   -4497
EN 8   -3418
EN 9   -2705
A-R6:PRO-S1
EN 3   0
EN 4   0
EN 5   -4444
EN 6   -5223
EN 7   -2062
EN 8   -4347
EN 9   -3224
A-RIB:TYR-CA
EN 3   0
EN 4   0
EN 5   -868
EN 6   -2982
EN 7   -5196
EN 8   -1802
EN 9   -3188
H2U-MY:ASN-CA
EN 3   0
EN 4   0
EN 5   0
EN 6   -13081
EN 7   0
EN 8   0
EN 9   -9852
G-R6:MET-S2
EN 3   0
EN 4   0
EN 5   -2702
EN 6   -4816
EN 7   -3042
EN 8   -2300
EN 9   -1879
FHU-MY:LEU-S2
EN 3   0
EN 4   0
EN 5   -16421
EN 6   0
EN 7   -14038
EN 8   0
EN 9   0
A-RIB:ASN-CA
EN 3   0
EN 4   0
EN 5   -2103
EN 6   -4769
EN 7   -4872
EN 8   -3899
EN 9   -4782
C-Y:PHE-S1
EN 3   0
EN 4   0
EN 5   -349

EN 6   971
EN 7   315
EN 8   -831
EN 9   -1741
G-P:SER-CA
EN 3   0
EN 4   -4454
EN 5   -4974
EN 6   -4422
EN 7   -4817
EN 8   -4474
EN 9   -4225
A-R6:ARG-S2
EN 3   0
EN 4   -9932
EN 5   -5776
EN 6   -5168
EN 7   -4850
EN 8   -4637
EN 9   -5181
G-P:HIS-CA
EN 3   0
EN 4   -6489
EN 5   0
EN 6   -3182
EN 7   -4842
EN 8   -4984
EN 9   -5105
IU-RIB:PRO-CA
EN 3   0
EN 4   0
EN 5   0
EN 6   0
EN 7   0
EN 8   -11284
EN 9   -9236
QUO-M5:GLN-S2
EN 3   0
EN 4   0
EN 5   0
EN 6   0
EN 7   0
EN 8   -15157
EN 9   0
IU-RIB:SER-CA
EN 3   0
EN 4   0
EN 5   0
EN 6   0
EN 7   -10964
EN 8   -9840
EN 9   0
FMU-RIB:ALA-S1
EN 3   0
EN 4   0
EN 5   0
EN 6   0
EN 7   0

EN 8   -13060
EN 9   0
IU-P:ILE-CA
EN 3   0
EN 4   0
EN 5   0
EN 6   0
EN 7   0
EN 8   -11325
EN 9   0
A-P:LYS-S2
EN 3   0
EN 4   -11162
EN 5   -8407
EN 6   -5966
EN 7   -5311
EN 8   -5454
EN 9   -5112
G-RIB:LEU-S2
EN 3   0
EN 4   0
EN 5   -2678
EN 6   -2477
EN 7   -2013
EN 8   -890
EN 9   -2254
A-R5:ARG-S1
EN 3   0
EN 4   0
EN 5   -3493
EN 6   -3890
EN 7   -4330
EN 8   -3891
EN 9   -4522
A-P:THR-S1
EN 3   0
EN 4   -5229
EN 5   -6518
EN 6   -4644
EN 7   -4446
EN 8   -5181
EN 9   -3922
QUO-M6:ASN-CA
EN 3   0
EN 4   0
EN 5   0
EN 6   0
EN 7   -16007
EN 8   0
EN 9   0
DA-M6:LEU-S2
EN 3   0
EN 4   0
EN 5   0
EN 6   0
EN 7   0
EN 8   0
EN 9   -13589

FMU-MY:GLU-CA
EN 3 0
EN 4 0
EN 5 0
EN 6 0
EN 7 0
EN 8 -13110
EN 9 0
U34-P:TYR-S1
EN 3 0
EN 4 0
EN 5 0
EN 6 0
EN 7 0
EN 8 0
EN 9 -14323
U34-P:GLU-S1
EN 3 0
EN 4 0
EN 5 0
EN 6 0
EN 7 0
EN 8 -13116
EN 9 0
A-R6:LYS-S2
EN 3 0
EN 4 0
EN 5 -4472
EN 6 -5129
EN 7 -4304
EN 8 -3778
EN 9 -3739
H2U-MY:ARG-S1
EN 3 0
EN 4 0
EN 5 0
EN 6 -11750
EN 7 0
EN 8 0
EN 9 0
A-RIB:SER-CA
EN 3 0
EN 4 0
EN 5 -3554
EN 6 -3397
EN 7 -4606
EN 8 -3482
EN 9 -4277
GTP-RIB:ASN-CA
EN 3 0
EN 4 0
EN 5 0
EN 6 -17068
EN 7 0
EN 8 0
EN 9 0
A-R5:PHE-S2
EN 3 0

EN 4 -5930
EN 5 -2227
EN 6 -1910
EN 7 -1562
EN 8 -2945
EN 9 -1901
C31-RIB:LEU-S2
EN 3 0
EN 4 0
EN 5 0
EN 6 -15100
EN 7 0
EN 8 0
EN 9 0
DA-M5:GLU-S1
EN 3 0
EN 4 0
EN 5 0
EN 6 0
EN 7 0
EN 8 0
EN 9 -12073
GTP-RIB:ASN-S2
EN 3 0
EN 4 0
EN 5 0
EN 6 -17089
EN 7 0
EN 8 0
EN 9 0
IU-MY:LEU-S1
EN 3 0
EN 4 0
EN 5 0
EN 6 0
EN 7 -9811
EN 8 0
EN 9 0
U-P:THR-CA
EN 3 0
EN 4 -5999
EN 5 -5017
EN 6 -3696
EN 7 -3016
EN 8 -4494
EN 9 -4455
C-Y:GLU-S2
EN 3 0
EN 4 0
EN 5 1505
EN 6 -1612
EN 7 -2053
EN 8 -2531
EN 9 -2891
A-R6:ASP-S2
EN 3 0
EN 4 0
EN 5 649

EN 6   -3181
EN 7   -2411
EN 8   -2713
EN 9   -3309
FMU-P:ASP-S2
EN 3   0
EN 4   0
EN 5   0
EN 6   0
EN 7   0
EN 8   0
EN 9   -12804
U31-P:LEU-S1
EN 3   0
EN 4   0
EN 5   0
EN 6   0
EN 7   0
EN 8   0
EN 9   -11868
C-Y:ILE-CA
EN 3   0
EN 4   0
EN 5   0
EN 6   1928
EN 7   1272
EN 8   -707
EN 9   -2611
U-Y:ARG-S1
EN 3   0
EN 4   0
EN 5   -4642
EN 6   -4657
EN 7   -3596
EN 8   -4155
EN 9   -4081
FMU-P:CYS-S1
EN 3   0
EN 4   0
EN 5   0
EN 6   0
EN 7   0
EN 8   0
EN 9   -17016
C31-MY:PHE-S1
EN 3   0
EN 4   0
EN 5   -18510
EN 6   0
EN 7   0
EN 8   0
EN 9   -13960
U-Y:VAL-S1
EN 3   0
EN 4   0
EN 5   -1337
EN 6   -1021
EN 7   -2059

EN 8   -814
EN 9   -222
U31-RIB:GLN-S2
EN 3   0
EN 4   0
EN 5   0
EN 6   0
EN 7   -16281
EN 8   0
EN 9   0
FHU-P:PRO-CA
EN 3   0
EN 4   0
EN 5   0
EN 6   0
EN 7   -13909
EN 8   0
EN 9   0
DA-M6:GLN-CA
EN 3   0
EN 4   0
EN 5   0
EN 6   0
EN 7   0
EN 8   -15127
EN 9   0
C-RIB:VAL-CA
EN 3   0
EN 4   0
EN 5   -1416
EN 6   -2525
EN 7   -4080
EN 8   -3997
EN 9   -3023
IU-RIB:SER-S1
EN 3   0
EN 4   0
EN 5   -13346
EN 6   0
EN 7   0
EN 8   -9840
EN 9   0
U31-RIB:GLU-CA
EN 3   0
EN 4   0
EN 5   0
EN 6   0
EN 7   0
EN 8   0
EN 9   -12066
A-R5:THR-CA
EN 3   0
EN 4   0
EN 5   0
EN 6   -3306
EN 7   -2867
EN 8   -3319
EN 9   -3683

QUO-M5:LEU-S1
EN 3 0
EN 4 0
EN 5 0
EN 6 0
EN 7 -14035
EN 8 0
EN 9 -11868
G-P:ARG-S2
EN 3 0
EN 4 -3955
EN 5 -9764
EN 6 -6531
EN 7 -6211
EN 8 -5790
EN 9 -5608
C-P:ASN-CA
EN 3 0
EN 4 -5659
EN 5 -2960
EN 6 -5074
EN 7 -5278
EN 8 -4988
EN 9 -5008
G-R5:TRP-S2
EN 3 0
EN 4 0
EN 5 -5228
EN 6 -3907
EN 7 -124
EN 8 -5157
EN 9 -1391
U-P:ASN-S1
EN 3 0
EN 4 -8296
EN 5 -6309
EN 6 -5280
EN 7 -4480
EN 8 -5627
EN 9 -3627
H2U-MY:GLN-S2
EN 3 0
EN 4 0
EN 5 0
EN 6 -13354
EN 7 0
EN 8 0
EN 9 0
C-Y:MET-S2
EN 3 0
EN 4 0
EN 5 -1388
EN 6 -1785
EN 7 -2441
EN 8 -3587
EN 9 -3708
C31-P:TYR-S1
EN 3 0

EN 4 0
EN 5 0
EN 6 0
EN 7 0
EN 8 -15367
EN 9 -14323
A-R5:ILE-CA
EN 3 0
EN 4 0
EN 5 0
EN 6 1771
EN 7 1115
EN 8 -864
EN 9 -1156
A-R6:LEU-S1
EN 3 0
EN 4 0
EN 5 -131
EN 6 -2798
EN 7 -1736
EN 8 -1263
EN 9 -2120
FMU-MY:CYS-CA
EN 3 0
EN 4 0
EN 5 -21565
EN 6 0
EN 7 0
EN 8 0
EN 9 0
C31-MY:TYR-S2
EN 3 0
EN 4 0
EN 5 0
EN 6 0
EN 7 0
EN 8 -15371
EN 9 -14327
QUO-M5:LYS-S1
EN 3 0
EN 4 0
EN 5 0
EN 6 0
EN 7 0
EN 8 0
EN 9 -12435
C-RIB:HIS-CA
EN 3 0
EN 4 0
EN 5 -4879
EN 6 -5275
EN 7 -4767
EN 8 -4293
EN 9 -4190
QUO-P:ASN-S2
EN 3 0
EN 4 0
EN 5 0

EN 6 0
EN 7 0
EN 8 0
EN 9 -13861
A-RIB:ASN-S1
EN 3 0
EN 4 0
EN 5 -5207
EN 6 -4769
EN 7 -5164
EN 8 -4041
EN 9 -4782
G-R5:ILE-S1
EN 3 0
EN 4 -4410
EN 5 1011
EN 6 2331
EN 7 0
EN 8 656
EN 9 116
A-R5:GLN-S2
EN 3 0
EN 4 0
EN 5 -4094
EN 6 -3777
EN 7 -4218
EN 8 -4448
EN 9 -4847
A-R6:GLU-CA
EN 3 0
EN 4 0
EN 5 -2046
EN 6 -1278
EN 7 -217
EN 8 -1047
EN 9 -2134
DA-M5:HIS-S2
EN 3 0
EN 4 -25043
EN 5 0
EN 6 0
EN 7 0
EN 8 0
EN 9 0
U-Y:TYR-CA
EN 3 0
EN 4 0
EN 5 -1635
EN 6 -4302
EN 7 -1975
EN 8 -2569
EN 9 -3625
C-RIB:LYS-S1
EN 3 0
EN 4 0
EN 5 -2258
EN 6 -5377
EN 7 -5028

EN 8   -5232
EN 9   -5311
G-R5:ASP-CA
EN 3   0
EN 4   0
EN 5   0
EN 6   860
EN 7   1922
EN 8   -1178
EN 9   -3123
G-RIB:TYR-S1
EN 3   0
EN 4   0
EN 5   -308
EN 6   -705
EN 7   -1786
EN 8   -3296
EN 9   -2207
IU-RIB:LYS-CA
EN 3   0
EN 4   0
EN 5   -13759
EN 6   0
EN 7   0
EN 8   -10966
EN 9   -10476
C-RIB:GLN-S2
EN 3   0
EN 4   0
EN 5   -7212
EN 6   -6725
EN 7   -6095
EN 8   -4765
EN 9   -5492
U-RIB:ILE-S1
EN 3   0
EN 4   0
EN 5   -316
EN 6   -3293
EN 7   -1921
EN 8   -2254
EN 9   -1707
U31-P:ASP-CA
EN 3   0
EN 4   -22728
EN 5   0
EN 6   0
EN 7   0
EN 8   -13801
EN 9   0
QUO-P:LEU-S2
EN 3   0
EN 4   0
EN 5   0
EN 6   0
EN 7   -15756
EN 8   0
EN 9   0

G-R6:LEU-CA
EN 3   0
EN 4   0
EN 5   2146
EN 6   3467
EN 7   -915
EN 8   -1057
EN 9   -1178
G-P:HIS-S1
EN 3   0
EN 4   -6489
EN 5   -2785
EN 6   -3735
EN 7   -5555
EN 8   -5989
EN 9   -3057
A-R5:ASP-S1
EN 3   0
EN 4   0
EN 5   695
EN 6   -1971
EN 7   -2074
EN 8   -1504
EN 9   -2524
A-R5:GLU-S1
EN 3   0
EN 4   0
EN 5   1381
EN 6   -19
EN 7   -1941
EN 8   -1651
EN 9   -2141
C-Y:THR-CA
EN 3   0
EN 4   0
EN 5   0
EN 6   -2767
EN 7   -2419
EN 8   -2682
EN 9   -2832
DA-M6:LEU-S1
EN 3   0
EN 4   0
EN 5   0
EN 6   0
EN 7   0
EN 8   0
EN 9   -13586
GTP-M5:SER-S1
EN 3   0
EN 4   0
EN 5   0
EN 6   0
EN 7   0
EN 8   -14064
EN 9   -13021
U-RIB:GLN-S1
EN 3   0

EN 4   0
EN 5   -3113
EN 6   -5518
EN 7   -4954
EN 8   -4046
EN 9   -4467
C31-MY:SER-CA
EN 3   0
EN 4   0
EN 5   0
EN 6   0
EN 7   0
EN 8   -14064
EN 9   0
A-R5:LEU-S2
EN 3   0
EN 4   -5555
EN 5   -1851
EN 6   -1083
EN 7   -22
EN 8   -1931
EN 9   -1410
G-R5:TYR-S1
EN 3   0
EN 4   0
EN 5   0
EN 6   -2091
EN 7   -432
EN 8   -1955
EN 9   -490
QUO-RIB:ASN-CA
EN 3   0
EN 4   0
EN 5   0
EN 6   0
EN 7   0
EN 8   -14883
EN 9   0
G-R6:ARG-S2
EN 3   0
EN 4   -3927
EN 5   -1941
EN 6   -5923
EN 7   -5355
EN 8   -4540
EN 9   -4007
C-RIB:PHE-S1
EN 3   0
EN 4   0
EN 5   -2066
EN 6   971
EN 7   -1401
EN 8   -1995
EN 9   -3222
QUO-M5:PHE-S2
EN 3   0
EN 4   0
EN 5   -18513

EN 6   -17192
EN 7   0
EN 8   0
EN 9   0
A-R5:HIS-CA
EN 3   0
EN 4   0
EN 5   0
EN 6   -4267
EN 7   -4040
EN 8   -4450
EN 9   -4595
DA-RIB:MET-S2
EN 3   0
EN 4   0
EN 5   -19549
EN 6   0
EN 7   0
EN 8   0
EN 9   0
A-R6:TYR-S2
EN 3   0
EN 4   -8011
EN 5   -3594
EN 6   -3991
EN 7   -2929
EN 8   -2188
EN 9   -3033
C-RIB:GLN-S1
EN 3   0
EN 4   0
EN 5   -2189
EN 6   -6170
EN 7   -5748
EN 8   -5309
EN 9   -4801
U-P:TRP-CA
EN 3   0
EN 4   0
EN 5   -5555
EN 6   -4234
EN 7   -6277
EN 8   -3767
EN 9   -4441
C-Y:MET-S1
EN 3   0
EN 4   0
EN 5   -1377
EN 6   -1774
EN 7   -712
EN 8   -2693
EN 9   -2272
A-R5:TYR-S1
EN 3   0
EN 4   0
EN 5   -5308
EN 6   -1265
EN 7   -2925

EN 8   -4072
EN 9   -2813
U-Y:HIS-S2
EN 3   0
EN 4   -10528
EN 5   -2385
EN 6   -5052
EN 7   -6358
EN 8   -3318
EN 9   -4374
U-RIB:ASN-S2
EN 3   0
EN 4   -6594
EN 5   -5994
EN 6   -6207
EN 7   -5501
EN 8   -6149
EN 9   -4967
H2U-P:PHE-S1
EN 3   0
EN 4   0
EN 5   0
EN 6   0
EN 7   0
EN 8   0
EN 9   -9973
U-Y:CYS-S1
EN 3   0
EN 4   0
EN 5   0
EN 6   -3007
EN 7   0
EN 8   -2088
EN 9   -3766
G-R6:ASN-S2
EN 3   0
EN 4   0
EN 5   -1563
EN 6   -5395
EN 7   -5150
EN 8   -3323
EN 9   -3597
M2G-P:GLY-CA
EN 3   0
EN 4   0
EN 5   0
EN 6   0
EN 7   0
EN 8   -13448
EN 9   0
G-RIB:ASN-CA
EN 3   0
EN 4   0
EN 5   -3260
EN 6   -4556
EN 7   -4834
EN 8   -5164
EN 9   -4369

H2U-MY:PHE-S1
EN 3   0
EN 4   0
EN 5   -14522
EN 6   0
EN 7   0
EN 8   0
EN 9   0
G-R5:THR-CA
EN 3   0
EN 4   0
EN 5   0
EN 6   -647
EN 7   -2015
EN 8   -561
EN 9   -3039
A-P:SER-CA
EN 3   0
EN 4   -6707
EN 5   -5274
EN 6   -5543
EN 7   -4206
EN 8   -5075
EN 9   -3828
A-R5:LYS-CA
EN 3   0
EN 4   -4396
EN 5   0
EN 6   -1088
EN 7   -2297
EN 8   -2489
EN 9   -3502
A-P:ARG-S1
EN 3   0
EN 4   0
EN 5   -7223
EN 6   -7055
EN 7   -5183
EN 8   -6469
EN 9   -5711
C31-RIB:THR-CA
EN 3   0
EN 4   0
EN 5   0
EN 6   -16489
EN 7   0
EN 8   0
EN 9   -14978
5BU-P:PRO-CA
EN 3   0
EN 4   0
EN 5   -16291
EN 6   0
EN 7   0
EN 8   0
EN 9   0
QUO-P:LEU-S1
EN 3   0

EN 4   0
EN 5   0
EN 6   0
EN 7   -15753
EN 8   0
EN 9   0
G-P:ASP-S1
EN 3   0
EN 4   -4193
EN 5   -1494
EN 6   -2272
EN 7   -3812
EN 8   -4213
EN 9   -4106
QUO-M5:ASP-S2
EN 3   0
EN 4   0
EN 5   0
EN 6   0
EN 7   -14972
EN 8   -13848
EN 9   0
G-R6:ASN-S1
EN 3   0
EN 4   0
EN 5   -1543
EN 6   -4389
EN 7   -3892
EN 8   -4319
EN 9   -2819
IU-P:ASP-S2
EN 3   0
EN 4   0
EN 5   0
EN 6   0
EN 7   0
EN 8   -11126
EN 9   0
G-R6:ILE-CA
EN 3   0
EN 4   0
EN 5   0
EN 6   2331
EN 7   3393
EN 8   1082
EN 9   -933
FHU-P:HIS-S2
EN 3   0
EN 4   0
EN 5   0
EN 6   0
EN 7   0
EN 8   0
EN 9   -15072
IU-RIB:GLN-S2
EN 3   0
EN 4   0
EN 5   0

EN 6 0
EN 7 0
EN 8 -11938
EN 9 0
U34-P:HIS-CA
EN 3 0
EN 4 0
EN 5 0
EN 6 0
EN 7 0
EN 8 0
EN 9 -15055
A-P:SER-S1
EN 3 0
EN 4 -8425
EN 5 -7337
EN 6 -5117
EN 7 -3343
EN 8 -4571
EN 9 -4611
C31-MY:ALA-S1
EN 3 0
EN 4 0
EN 5 0
EN 6 0
EN 7 0
EN 8 -13060
EN 9 0
G-RIB:CYS-CA
EN 3 0
EN 4 0
EN 5 -3001
EN 6 0
EN 7 -619
EN 8 -1213
EN 9 -1174
DA-M5:GLN-CA
EN 3 0
EN 4 0
EN 5 0
EN 6 0
EN 7 0
EN 8 0
EN 9 -14083
U-P:ASN-CA
EN 3 0
EN 4 -6578
EN 5 -5978
EN 6 -3824
EN 7 -4219
EN 8 -4671
EN 9 -4376
5BU-P:ARG-S2
EN 3 0
EN 4 0
EN 5 -15353
EN 6 0
EN 7 0

EN 8 0
EN 9 0
A-RIB:LEU-S2
EN 3 0
EN 4 0
EN 5 -134
EN 6 -2540
EN 7 -2389
EN 8 -616
EN 9 -2033
G-P:PHE-CA
EN 3 0
EN 4 0
EN 5 -2695
EN 6 -1374
EN 7 -2030
EN 8 -2172
EN 9 -3297
DA-RIB:MET-S1
EN 3 0
EN 4 0
EN 5 0
EN 6 0
EN 7 -17156
EN 8 0
EN 9 0
U-RIB:SER-CA
EN 3 0
EN 4 0
EN 5 -3768
EN 6 -3833
EN 7 -5893
EN 8 -3798
EN 9 -4040
A-R6:ALA-CA
EN 3 0
EN 4 -3982
EN 5 -1996
EN 6 1041
EN 7 -1331
EN 8 -208
EN 9 -2520
U31-MY:ASP-S2
EN 3 0
EN 4 -24493
EN 5 0
EN 6 0
EN 7 0
EN 8 -13848
EN 9 -12804
C31-P:SER-S1
EN 3 0
EN 4 0
EN 5 0
EN 6 0
EN 7 0
EN 8 0
EN 9 -13021

U-Y:SER-S1
EN 3   0
EN 4   -5754
EN 5   -4150
EN 6   -4456
EN 7   -3103
EN 8   -2914
EN 9   -3950
U-Y:TYR-S2
EN 3   0
EN 4   -9783
EN 5   -5074
EN 6   -319
EN 7   -4304
EN 8   -2573
EN 9   -3629
U-Y:PHE-S2
EN 3   0
EN 4   -9420
EN 5   -2994
EN 6   -4253
EN 7   -611
EN 8   -2592
EN 9   -2171
QUO-M5:ASN-S1
EN 3   0
EN 4   0
EN 5   0
EN 6   0
EN 7   -16007
EN 8   0
EN 9   0
DA-RIB:HIS-S1
EN 3   0
EN 4   0
EN 5   0
EN 6   -18284
EN 7   0
EN 8   -16099
EN 9   0
A-R5:ILE-S1
EN 3   0
EN 4   0
EN 5   451
EN 6   1771
EN 7   111
EN 8   -30
EN 9   -1538
H2U-MY:GLU-S2
EN 3   0
EN 4   0
EN 5   0
EN 6   -11346
EN 7   0
EN 8   0
EN 9   0
A-P:ARG-S2
EN 3   0

EN 4 0
EN 5 -9766
EN 6 -6175
EN 7 -6615
EN 8 -6570
EN 9 -5668
FMU-MY:PHE-CA
EN 3 0
EN 4 0
EN 5 0
EN 6 0
EN 7 0
EN 8 0
EN 9 -13960
FMU-P:PHE-S2
EN 3 0
EN 4 0
EN 5 0
EN 6 -17192
EN 7 0
EN 8 0
EN 9 0
U34-MY:PHE-S1
EN 3 0
EN 4 0
EN 5 0
EN 6 0
EN 7 0
EN 8 -15004
EN 9 0
U-RIB:ASP-CA
EN 3 0
EN 4 0
EN 5 -1787
EN 6 -2183
EN 7 -2839
EN 8 -3433
EN 9 -3063
A-P:TYR-S2
EN 3 0
EN 4 0
EN 5 -2592
EN 6 -5909
EN 7 -4434
EN 8 -3908
EN 9 -3345
IU-RIB:LYS-S2
EN 3 0
EN 4 0
EN 5 0
EN 6 -12492
EN 7 -10426
EN 8 -10307
EN 9 0
C-RIB:ARG-CA
EN 3 0
EN 4 0
EN 5 -3719

EN 6   -4115
EN 7   -5178
EN 8   -5882
EN 9   -5226
DA-M6:MET-S2
EN 3   0
EN 4   0
EN 5   0
EN 6   0
EN 7   0
EN 8   -16043
EN 9   0
A-RIB:LYS-CA
EN 3   0
EN 4   0
EN 5   -2409
EN 6   -3359
EN 7   -4361
EN 8   -4546
EN 9   -4309
FMU-RIB:ILE-CA
EN 3   0
EN 4   0
EN 5   0
EN 6   0
EN 7   0
EN 8   0
EN 9   -13003
FMU-P:ALA-S1
EN 3   0
EN 4   0
EN 5   0
EN 6   0
EN 7   0
EN 8   -13060
EN 9   0
FMU-RIB:VAL-CA
EN 3   0
EN 4   0
EN 5   0
EN 6   0
EN 7   0
EN 8   -13349
EN 9   0
FHU-P:SER-CA
EN 3   0
EN 4   0
EN 5   0
EN 6   0
EN 7   0
EN 8   -12347
EN 9   -11303
G-R6:PHE-CA
EN 3   0
EN 4   0
EN 5   0
EN 6   -342
EN 7   718

EN 8   -1171
EN 9   -217
G-RIB:VAL-S1
EN 3   0
EN 4   0
EN 5   -10
EN 6   -2416
EN 7   -1354
EN 8   -1094
EN 9   -1886
FMU-P:ALA-CA
EN 3   0
EN 4   0
EN 5   0
EN 6   0
EN 7   0
EN 8   0
EN 9   -12016
U-P:SER-CA
EN 3   0
EN 4   0
EN 5   -5782
EN 6   -4169
EN 7   -5830
EN 8   -4491
EN 9   -4375
H2U-P:TRP-S1
EN 3   0
EN 4   0
EN 5   0
EN 6   0
EN 7   0
EN 8   -13577
EN 9   0
QUO-M6:PHE-S2
EN 3   0
EN 4   0
EN 5   -18513
EN 6   -17192
EN 7   0
EN 8   0
EN 9   0
GTP-M6:ASN-S1
EN 3   0
EN 4   0
EN 5   0
EN 6   0
EN 7   0
EN 8   0
EN 9   -13840
C-Y:TYR-S1
EN 3   0
EN 4   -6133
EN 5   -4699
EN 6   -2113
EN 7   -1051
EN 8   -2027
EN 9   -2103

A-R5:LYS-S2
EN 3   0
EN 4   0
EN 5   -4733
EN 6   -3151
EN 7   -5309
EN 8   -4662
EN 9   -4850
U31-RIB:MET-CA
EN 3   0
EN 4   0
EN 5   0
EN 6   -18217
EN 7   0
EN 8   0
EN 9   0
FHU-P:GLY-CA
EN 3   0
EN 4   -20657
EN 5   -16953
EN 6   0
EN 7   -12854
EN 8   -11730
EN 9   0
QUO-M6:ASP-S2
EN 3   0
EN 4   0
EN 5   0
EN 6   0
EN 7   -14972
EN 8   -13848
EN 9   0
A-R6:PRO-CA
EN 3   0
EN 4   0
EN 5   -3439
EN 6   -3123
EN 7   -3779
EN 8   -3794
EN 9   -2617
G-R5:CYS-CA
EN 3   0
EN 4   0
EN 5   0
EN 6   0
EN 7   0
EN 8   -1213
EN 9   -1886
U31-MY:MET-S2
EN 3   0
EN 4   -24970
EN 5   0
EN 6   0
EN 7   0
EN 8   0
EN 9   0
U-RIB:ASP-S1
EN 3   0

EN 4   -5493
EN 5   -2794
EN 6   -1473
EN 7   -4045
EN 8   -2723
EN 9   -2684
U-Y:THR-S1
EN 3   0
EN 4   0
EN 5   -3295
EN 6   -4073
EN 7   -2178
EN 8   -3422
EN 9   -3043
H2U-P:TRP-CA
EN 3   0
EN 4   0
EN 5   0
EN 6   0
EN 7   0
EN 8   0
EN 9   -12533
FMU-RIB:PHE-CA
EN 3   0
EN 4   0
EN 5   0
EN 6   0
EN 7   0
EN 8   0
EN 9   -13960
G-RIB:ASN-S2
EN 3   0
EN 4   -5267
EN 5   -4577
EN 6   -6476
EN 7   -5286
EN 8   -4747
EN 9   -4587
G-R6:GLN-S1
EN 3   0
EN 4   0
EN 5   0
EN 6   -465
EN 7   -3627
EN 8   -4538
EN 9   -3494
U31-MY:GLU-S1
EN 3   0
EN 4   0
EN 5   0
EN 6   0
EN 7   0
EN 8   -13116
EN 9   0
U31-MY:TYR-S2
EN 3   0
EN 4   0
EN 5   -18877

EN 6 0
EN 7 0
EN 8 0
EN 9 0
C-RIB:LYS-CA
EN 3 0
EN 4 0
EN 5 -1540
EN 6 -4036
EN 7 -4508
EN 8 -4955
EN 9 -4830
FMU-RIB:GLU-S1
EN 3 0
EN 4 0
EN 5 0
EN 6 0
EN 7 0
EN 8 0
EN 9 -12073
IU-P:ARG-S2
EN 3 0
EN 4 0
EN 5 0
EN 6 0
EN 7 0
EN 8 0
EN 9 -9799
A-P:GLN-CA
EN 3 0
EN 4 0
EN 5 -2348
EN 6 -4131
EN 7 -3693
EN 8 -4286
EN 9 -4745
G-R6:ILE-S1
EN 3 0
EN 4 -4410
EN 5 1011
EN 6 0
EN 7 3393
EN 8 1241
EN 9 -81
U-RIB:TYR-S2
EN 3 0
EN 4 0
EN 5 -3357
EN 6 -4616
EN 7 -5198
EN 8 -2955
EN 9 -3629
A-R5:THR-S1
EN 3 0
EN 4 -5227
EN 5 -3240
EN 6 -3306
EN 7 -3364

EN 8   -3595
EN 9   -3329
QUO-M5:GLU-S1
EN 3   0
EN 4   0
EN 5   0
EN 6   0
EN 7   0
EN 8   0
EN 9   -12073
C-P:HIS-CA
EN 3   0
EN 4   0
EN 5   -1454
EN 6   -4954
EN 7   -2506
EN 8   -5243
EN 9   -5160
U34-P:ARG-S1
EN 3   0
EN 4   0
EN 5   0
EN 6   0
EN 7   0
EN 8   0
EN 9   -12509
FHU-P:LYS-S2
EN 3   0
EN 4   -20736
EN 5   -15315
EN 6   0
EN 7   0
EN 8   -13526
EN 9   0
U-P:GLN-CA
EN 3   0
EN 4   0
EN 5   -3117
EN 6   -3514
EN 7   -3839
EN 8   -4604
EN 9   -5017
IU-MY:TYR-CA
EN 3   0
EN 4   0
EN 5   0
EN 6   0
EN 7   0
EN 8   0
EN 9   -10099
DA-M6:ASN-S1
EN 3   0
EN 4   0
EN 5   0
EN 6   0
EN 7   -16007
EN 8   0
EN 9   -13840

C-RIB:MET-S1
EN 3 0
EN 4 -6798
EN 5 -4812
EN 6 -5208
EN 7 -3816
EN 8 -3576
EN 9 -2272
C-RIB:THR-S1
EN 3 0
EN 4 0
EN 5 -4801
EN 6 -5871
EN 7 -4689
EN 8 -4880
EN 9 -4239
FHU-P:LEU-S1
EN 3 0
EN 4 0
EN 5 0
EN 6 0
EN 7 -12318
EN 8 0
EN 9 -13586
5BU-RIB:ILE-S1
EN 3 0
EN 4 0
EN 5 0
EN 6 0
EN 7 0
EN 8 0
EN 9 -11286
A-P:MET-S1
EN 3 0
EN 4 0
EN 5 -1536
EN 6 -1933
EN 7 -3142
EN 8 -3972
EN 9 -3144
G-P:LYS-S2
EN 3 0
EN 4 -10626
EN 5 -8512
EN 6 -6375
EN 7 -6843
EN 8 -5719
EN 9 -5569
G-R6:LYS-CA
EN 3 0
EN 4 0
EN 5 -132
EN 6 -1081
EN 7 -854
EN 8 -2884
EN 9 -3051
FMU-MY:ILE-CA
EN 3 0

EN 4 0
EN 5 0
EN 6 0
EN 7 0
EN 8 0
EN 9 -13003
5BU-P:ALA-S1
EN 3 0
EN 4 0
EN 5 0
EN 6 0
EN 7 0
EN 8 0
EN 9 -10299
FMU-MY:GLN-S2
EN 3 0
EN 4 0
EN 5 -18663
EN 6 0
EN 7 0
EN 8 0
EN 9 0
IU-RIB:LEU-S1
EN 3 0
EN 4 0
EN 5 -12194
EN 6 0
EN 7 0
EN 8 0
EN 9 0
U-P:PHE-CA
EN 3 0
EN 4 0
EN 5 -1277
EN 6 43
EN 7 -2330
EN 8 -2211
EN 9 -3083
G-P:PRO-CA
EN 3 0
EN 4 0
EN 5 -3911
EN 6 -3595
EN 7 -4748
EN 8 -4039
EN 9 -4806
G-R5:TYR-S2
EN 3 0
EN 4 -5734
EN 5 -2583
EN 6 -709
EN 7 -1918
EN 8 -2512
EN 9 -2992
U-Y:LYS-S2
EN 3 0
EN 4 0
EN 5 0

EN 6   -5636
EN 7   -5252
EN 8   -5026
EN 9   -5431
OMC-RIB:LYS-S1
EN 3   0
EN 4   0
EN 5   0
EN 6   0
EN 7   0
EN 8   -9662
EN 9   0
QUO-M5:ARG-S2
EN 3   0
EN 4   0
EN 5   0
EN 6   0
EN 7   -14688
EN 8   0
EN 9   -12521
G-RIB:PHE-S1
EN 3   0
EN 4   0
EN 5   -1663
EN 6   -2059
EN 7   -3720
EN 8   -2145
EN 9   -2379
5BU-RIB:ARG-S2
EN 3   0
EN 4   0
EN 5   0
EN 6   0
EN 7   0
EN 8   0
EN 9   -10803
FHU-RIB:ARG-CA
EN 3   0
EN 4   0
EN 5   0
EN 6   0
EN 7   0
EN 8   0
EN 9   -12509
C-P:ASP-S2
EN 3   0
EN 4   0
EN 5   -2637
EN 6   -4237
EN 7   -4479
EN 8   -4124
EN 9   -4919
G-R6:MET-S1
EN 3   0
EN 4   0
EN 5   -974
EN 6   -4474
EN 7   -2026

EN 8   -1456
EN 9   -2130
G-P:GLN-CA
EN 3   0
EN 4   0
EN 5   -4917
EN 6   -2209
EN 7   -4252
EN 8   -5468
EN 9   -3618
U-RIB:TRP-S1
EN 3   0
EN 4   0
EN 5   0
EN 6   -4230
EN 7   -5890
EN 8   -2045
EN 9   -4105
GTP-M5:ASP-S1
EN 3   0
EN 4   0
EN 5   0
EN 6   0
EN 7   0
EN 8   0
EN 9   -12759
G-R6:HIS-CA
EN 3   0
EN 4   0
EN 5   -1041
EN 6   -3155
EN 7   -3098
EN 8   -1523
EN 9   -4442
A-R6:MET-S2
EN 3   0
EN 4   -6966
EN 5   -6366
EN 6   -2946
EN 7   -2597
EN 8   -3980
EN 9   -983
U-RIB:LYS-S2
EN 3   0
EN 4   -5216
EN 5   -4947
EN 6   -4829
EN 7   -6881
EN 8   -6461
EN 9   -5390
FMU-RIB:GLU-S2
EN 3   0
EN 4   0
EN 5   0
EN 6   0
EN 7   0
EN 8   -13149
EN 9   0

C-Y:GLU-CA
EN 3 0
EN 4 0
EN 5 -172
EN 6 -568
EN 7 3927
EN 8 1063
EN 9 -1638
DA-RIB:TYR-CA
EN 3 0
EN 4 0
EN 5 0
EN 6 0
EN 7 0
EN 8 0
EN 9 -14323
H2U-MY:TRP-CA
EN 3 0
EN 4 0
EN 5 0
EN 6 0
EN 7 -14700
EN 8 0
EN 9 0
C-RIB:HIS-S2
EN 3 0
EN 4 -8600
EN 5 -5449
EN 6 -6679
EN 7 -5235
EN 8 -5930
EN 9 -4455
C31-RIB:ALA-S1
EN 3 0
EN 4 0
EN 5 0
EN 6 0
EN 7 0
EN 8 0
EN 9 -13733
FMU-RIB:ARG-CA
EN 3 0
EN 4 0
EN 5 0
EN 6 0
EN 7 0
EN 8 -13552
EN 9 0
U31-MY:GLU-CA
EN 3 0
EN 4 0
EN 5 0
EN 6 0
EN 7 0
EN 8 -13110
EN 9 0
A-RIB:TYR-S1
EN 3 0

EN 4 0
EN 5 -3590
EN 6 -4699
EN 7 -4427
EN 8 -2515
EN 9 -2260
C-P:VAL-CA
EN 3 0
EN 4 0
EN 5 -421
EN 6 -2204
EN 7 -3744
EN 8 -4077
EN 9 -3640
G-R6:ASN-CA
EN 3 0
EN 4 0
EN 5 0
EN 6 -3142
EN 7 -4069
EN 8 -2768
EN 9 -3226
C31-P:TYR-CA
EN 3 0
EN 4 0
EN 5 0
EN 6 0
EN 7 0
EN 8 0
EN 9 -16040
C-P:MET-CA
EN 3 0
EN 4 -6808
EN 5 -1387
EN 6 -66
EN 7 -3444
EN 8 -3822
EN 9 -3376
QUO-M6:ASN-S1
EN 3 0
EN 4 0
EN 5 0
EN 6 -17068
EN 7 0
EN 8 0
EN 9 0
IU-RIB:ILE-CA
EN 3 0
EN 4 0
EN 5 0
EN 6 -12008
EN 7 -11951
EN 8 0
EN 9 0
DA-M6:GLU-S1
EN 3 0
EN 4 0
EN 5 0

EN 6   0
EN 7   0
EN 8   -13116
EN 9   0
G-RIB:PHE-S2
EN 3   0
EN 4   0
EN 5   -1667
EN 6   -3780
EN 7   -3672
EN 8   -1887
EN 9   -1341
A-RIB:PHE-S1
EN 3   0
EN 4   0
EN 5   -2223
EN 6   -1907
EN 7   -3275
EN 8   -2705
EN 9   -2311
C-P:PHE-CA
EN 3   0
EN 4   0
EN 5   0
EN 6   0
EN 7   -698
EN 8   -3208
EN 9   -2348
OMC-MY:LYS-S2
EN 3   0
EN 4   0
EN 5   0
EN 6   0
EN 7   0
EN 8   -9709
EN 9   0
C31-MY:THR-CA
EN 3   0
EN 4   0
EN 5   0
EN 6   0
EN 7   0
EN 8   0
EN 9   -13260
FHU-P:ASP-S1
EN 3   0
EN 4   0
EN 5   0
EN 6   0
EN 7   0
EN 8   0
EN 9   -11042
FHU-P:LYS-S1
EN 3   0
EN 4   0
EN 5   -15267
EN 6   -15664
EN 7   -12885

EN 8 0
EN 9 0
FHU-MY:THR-S1
EN 3 0
EN 4 0
EN 5 0
EN 6 0
EN 7 0
EN 8 -12586
EN 9 -14265
A-R6:TRP-S2
EN 3 0
EN 4 0
EN 5 -3066
EN 6 -5180
EN 7 -4671
EN 8 -3548
EN 9 -2504
C31-RIB:THR-S1
EN 3 0
EN 4 0
EN 5 0
EN 6 -16489
EN 7 0
EN 8 0
EN 9 0
H2U-P:ARG-S2
EN 3 0
EN 4 0
EN 5 0
EN 6 -11762
EN 7 0
EN 8 0
EN 9 0
FMU-P:ASP-CA
EN 3 0
EN 4 0
EN 5 0
EN 6 0
EN 7 0
EN 8 -13801
EN 9 0
A-P:GLU-CA
EN 3 0
EN 4 0
EN 5 -331
EN 6 0
EN 7 -219
EN 8 -1818
EN 9 -2492
C31-MY:SER-S1
EN 3 0
EN 4 0
EN 5 0
EN 6 0
EN 7 0
EN 8 0
EN 9 -13021

G-R5:SER-CA
EN 3 0
EN 4 0
EN 5 0
EN 6 -2758
EN 7 -2068
EN 8 -3269
EN 9 -2800
C-P:PHE-S2
EN 3 0
EN 4 0
EN 5 0
EN 6 -3481
EN 7 -1415
EN 8 -2561
EN 9 -3235
A-R6:MET-S1
EN 3 0
EN 4 -6955
EN 5 -3251
EN 6 -1930
EN 7 -4304
EN 8 -2849
EN 9 -2428
U-Y:GLN-S2
EN 3 0
EN 4 0
EN 5 -4861
EN 6 -6759
EN 7 -4749
EN 8 -4077
EN 9 -4257
U-P:LEU-CA
EN 3 0
EN 4 0
EN 5 0
EN 6 2135
EN 7 -2247
EN 8 -2549
EN 9 -2179
U-P:THR-S1
EN 3 0
EN 4 -5999
EN 5 -6519
EN 6 -3696
EN 7 -4136
EN 8 -4840
EN 9 -3571
U-Y:ASN-S1
EN 3 0
EN 4 0
EN 5 -2870
EN 6 -3266
EN 7 -5125
EN 8 -5267
EN 9 -4372
U-Y:GLN-S1
EN 3 0

EN 4 0
EN 5 0
EN 6 0
EN 7 -4457
EN 8 -5510
EN 9 -4097
A-RIB:ASP-S1
EN 3 0
EN 4 -4726
EN 5 -2739
EN 6 -2423
EN 7 -3792
EN 8 -2154
EN 9 -3098
5BU-P:SER-S1
EN 3 0
EN 4 0
EN 5 0
EN 6 0
EN 7 0
EN 8 -12347
EN 9 0
C-P:GLU-S2
EN 3 0
EN 4 0
EN 5 0
EN 6 -3124
EN 7 -3780
EN 8 -3735
EN 9 -3330
C-RIB:ILE-CA
EN 3 0
EN 4 0
EN 5 0
EN 6 -2892
EN 7 -2162
EN 8 -2906
EN 9 -2915
FHU-MY:LYS-S1
EN 3 0
EN 4 0
EN 5 0
EN 6 0
EN 7 0
EN 8 0
EN 9 -12435
G-P:GLU-S1
EN 3 0
EN 4 0
EN 5 196
EN 6 1517
EN 7 -1147
EN 8 -3166
EN 9 -2415
FMU-RIB:ARG-S2
EN 3 0
EN 4 0
EN 5 -17070

EN 6   0
EN 7   0
EN 8   -15282
EN 9   0
DA-M5:TYR-CA
EN 3   0
EN 4   0
EN 5   0
EN 6   0
EN 7   -16490
EN 8   0
EN 9   -14323
U31-MY:THR-S1
EN 3   0
EN 4   0
EN 5   0
EN 6   -16489
EN 7   0
EN 8   -14304
EN 9   -13260
A-R5:ALA-CA
EN 3   0
EN 4   0
EN 5   -279
EN 6   1041
EN 7   -1623
EN 8   -997
EN 9   -1987
C-Y:CYS-CA
EN 3   0
EN 4   0
EN 5   0
EN 6   -2083
EN 7   0
EN 8   0
EN 9   -572
C-P:ALA-CA
EN 3   0
EN 4   0
EN 5   -5284
EN 6   -3035
EN 7   -2902
EN 8   -4169
EN 9   -3968
H2U-RIB:PHE-S1
EN 3   0
EN 4   0
EN 5   0
EN 6   0
EN 7   0
EN 8   -11016
EN 9   0
H2U-MY:GLN-CA
EN 3   0
EN 4   0
EN 5   0
EN 6   0
EN 7   0

EN 8   0
EN 9   -10095
IU-MY:PRO-CA
EN 3   0
EN 4   0
EN 5   0
EN 6   0
EN 7   0
EN 8   -10279
EN 9   -10240
U-Y:TRP-S2
EN 3   0
EN 4   -9255
EN 5   0
EN 6   -2512
EN 7   -1451
EN 8   0
EN 9   -4436
U31-P:SER-S1
EN 3   0
EN 4   0
EN 5   0
EN 6   0
EN 7   0
EN 8   0
EN 9   -13021
A-P:ASP-CA
EN 3   0
EN 4   0
EN 5   -1022
EN 6   0
EN 7   -1362
EN 8   -3774
EN 9   -4075
G-R5:ASP-S1
EN 3   0
EN 4   0
EN 5   0
EN 6   -146
EN 7   -350
EN 8   -3405
EN 9   -2840
U-P:CYS-CA
EN 3   0
EN 4   0
EN 5   0
EN 6   -3012
EN 7   0
EN 8   -2544
EN 9   -1501
DA-M6:GLU-S2
EN 3   0
EN 4   0
EN 5   -16655
EN 6   0
EN 7   0
EN 8   0
EN 9   0

U31-MY:THR-CA
EN 3 0
EN 4 0
EN 5 0
EN 6 -16489
EN 7 -15427
EN 8 0
EN 9 0
FHU-RIB:ILE-S1
EN 3 0
EN 4 0
EN 5 0
EN 6 0
EN 7 0
EN 8 -12329
EN 9 -11286
G-P:LYS-S1
EN 3 0
EN 4 -5586
EN 5 -5870
EN 6 -7352
EN 7 -5807
EN 8 -5396
EN 9 -4628
U34-RIB:SER-S1
EN 3 0
EN 4 0
EN 5 0
EN 6 0
EN 7 -15188
EN 8 0
EN 9 -13021
IU-MY:MET-CA
EN 3 0
EN 4 0
EN 5 0
EN 6 0
EN 7 0
EN 8 0
EN 9 -10765
A-R6:ASN-CA
EN 3 0
EN 4 0
EN 5 -385
EN 6 -2499
EN 7 -3887
EN 8 -3234
EN 9 -3494
A-P:ARG-CA
EN 3 0
EN 4 0
EN 5 -4209
EN 6 -4897
EN 7 -5751
EN 8 -5855
EN 9 -5034
G-R6:VAL-S1
EN 3 0

EN 4   0
EN 5   -1014
EN 6   -406
EN 7   101
EN 8   -613
EN 9   -453
U31-RIB:ASN-S2
EN 3   0
EN 4   0
EN 5   0
EN 6   0
EN 7   0
EN 8   -14904
EN 9   0
DA-M5:HIS-CA
EN 3   0
EN 4   0
EN 5   0
EN 6   -18284
EN 7   0
EN 8   0
EN 9   0
U34-MY:ASP-S2
EN 3   0
EN 4   0
EN 5   0
EN 6   0
EN 7   0
EN 8   0
EN 9   -12804
FMU-MY:MET-S2
EN 3   0
EN 4   0
EN 5   0
EN 6   0
EN 7   -17167
EN 8   -16043
EN 9   0
U-P:ILE-S1
EN 3   0
EN 4   0
EN 5   -2038
EN 6   -2434
EN 7   -660
EN 8   -802
EN 9   -1928
DA-M5:LEU-S2
EN 3   0
EN 4   0
EN 5   0
EN 6   0
EN 7   0
EN 8   0
EN 9   -11871
A-R6:TRP-S1
EN 3   0
EN 4   0
EN 5   -3066

```
EN 6   -1745
EN 7   -3406
EN 8   0
EN 9   -3338
U-RIB:CYS-CA
EN 3   0
EN 4   0
EN 5   0
EN 6   0
EN 7   0
EN 8   -4639
EN 9   -2501
H2U-RIB:ARG-S2
EN 3   0
EN 4   0
EN 5   0
EN 6   -11762
EN 7   0
EN 8   0
EN 9   -8533
G-RIB:ARG-S1
EN 3   0
EN 4   0
EN 5   -2482
EN 6   -5516
EN 7   -5042
EN 8   -5331
EN 9   -5452
QUO-RIB:ASN-S1
EN 3   0
EN 4   0
EN 5   0
EN 6   0
EN 7   -16007
EN 8   0
EN 9   0
U31-RIB:GLU-S2
EN 3   0
EN 4   0
EN 5   0
EN 6   0
EN 7   -14272
EN 8   0
EN 9   0
U31-P:GLN-S1
EN 3   0
EN 4   0
EN 5   0
EN 6   -17312
EN 7   0
EN 8   0
EN 9   0
C31-RIB:TYR-S2
EN 3   0
EN 4   0
EN 5   0
EN 6   0
EN 7   -18212
```

EN 8   0
EN 9   0
U34-RIB:SER-CA
EN 3   0
EN 4   0
EN 5   0
EN 6   0
EN 7   0
EN 8   0
EN 9   -13021
H2U-MY:GLU-CA
EN 3   0
EN 4   0
EN 5   0
EN 6   -11307
EN 7   0
EN 8   0
EN 9   0
IU-P:LYS-CA
EN 3   0
EN 4   0
EN 5   0
EN 6   0
EN 7   0
EN 8   -12468
EN 9   0
A-RIB:MET-CA
EN 3   0
EN 4   0
EN 5   -3251
EN 6   -1930
EN 7   -3139
EN 8   -3733
EN 9   -3340
FMU-MY:CYS-S1
EN 3   0
EN 4   -26987
EN 5   0
EN 6   0
EN 7   0
EN 8   0
EN 9   0
DA-RIB:ARG-CA
EN 3   0
EN 4   0
EN 5   0
EN 6   0
EN 7   0
EN 8   0
EN 9   -12509
DA-RIB:MET-CA
EN 3   0
EN 4   0
EN 5   0
EN 6   0
EN 7   0
EN 8   0
EN 9   -14989

GTP-RIB:GLY-CA
EN 3 0
EN 4 0
EN 5 0
EN 6 0
EN 7 -14571
EN 8 0
EN 9 0
A-R5:GLN-S1
EN 3 0
EN 4 0
EN 5 -628
EN 6 -3747
EN 7 -2234
EN 8 -3064
EN 9 -3848
A-R5:ASN-S2
EN 3 0
EN 4 0
EN 5 -2123
EN 6 -802
EN 7 -5415
EN 8 -4876
EN 9 -4520
IU-P:SER-S1
EN 3 0
EN 4 0
EN 5 0
EN 6 0
EN 7 0
EN 8 0
EN 9 -12016
G-R5:ARG-S2
EN 3 0
EN 4 -6650
EN 5 -4861
EN 6 -5060
EN 7 -5003
EN 8 -4838
EN 9 -5167
A-R5:TRP-S2
EN 3 0
EN 4 0
EN 5 0
EN 6 -3463
EN 7 -4119
EN 8 -4382
EN 9 -2956
QUO-M6:GLN-S2
EN 3 0
EN 4 0
EN 5 0
EN 6 0
EN 7 -16281
EN 8 0
EN 9 0
C31-MY:ASP-CA
EN 3 0

EN 4 0
EN 5 0
EN 6 0
EN 7 0
EN 8 0
EN 9 -12757
QUO-RIB:PHE-S1
EN 3 0
EN 4 0
EN 5 0
EN 6 0
EN 7 0
EN 8 0
EN 9 -13960
U-P:GLN-S2
EN 3 0
EN 4 0
EN 5 -7136
EN 6 -5262
EN 7 -4754
EN 8 -4795
EN 9 -4650
A-RIB:LYS-S1
EN 3 0
EN 4 0
EN 5 -1702
EN 6 -5428
EN 7 -5025
EN 8 -4487
EN 9 -4876
GTP-M6:ASN-CA
EN 3 0
EN 4 0
EN 5 0
EN 6 0
EN 7 0
EN 8 0
EN 9 -13840
H2U-MY:ASN-S1
EN 3 0
EN 4 0
EN 5 0
EN 6 -13081
EN 7 0
EN 8 -10896
EN 9 -9852
U-P:GLY-CA
EN 3 0
EN 4 -9130
EN 5 -7017
EN 6 -5696
EN 7 -5314
EN 8 -5024
EN 9 -4962
5BU-P:SER-CA
EN 3 0
EN 4 0
EN 5 0

EN 6 0
EN 7 0
EN 8 -12347
EN 9 0
G-RIB:GLU-S2
EN 3 0
EN 4 0
EN 5 -4247
EN 6 -2874
EN 7 -2063
EN 8 -2535
EN 9 -2332
U-RIB:GLU-S2
EN 3 0
EN 4 0
EN 5 -3405
EN 6 -1532
EN 7 -2188
EN 8 -2069
EN 9 -2164
U31-MY:PHE-S2
EN 3 0
EN 4 0
EN 5 0
EN 6 0
EN 7 0
EN 8 0
EN 9 -13964
U-P:TRP-S1
EN 3 0
EN 4 -9259
EN 5 -5555
EN 6 -5239
EN 7 -4178
EN 8 -5269
EN 9 -4733
G-R5:GLY-CA
EN 3 0
EN 4 -3811
EN 5 -4330
EN 6 -3607
EN 7 -3666
EN 8 -4673
EN 9 -4066
A-RIB:THR-S1
EN 3 0
EN 4 0
EN 5 -4958
EN 6 -5646
EN 7 -4133
EN 8 -4643
EN 9 -4065
A-RIB:TRP-CA
EN 3 0
EN 4 0
EN 5 0
EN 6 -4467
EN 7 -2401

EN 8   -5004
EN 9   -3338
U31-MY:VAL-S1
EN 3   0
EN 4   0
EN 5   0
EN 6   0
EN 7   0
EN 8   -13351
EN 9   0
C31-P:MET-S1
EN 3   0
EN 4   0
EN 5   0
EN 6   0
EN 7   0
EN 8   -16032
EN 9   0
U-Y:CYS-CA
EN 3   0
EN 4   0
EN 5   0
EN 6   -4725
EN 7   0
EN 8   -2088
EN 9   -1496
FMU-RIB:MET-S1
EN 3   0
EN 4   0
EN 5   0
EN 6   0
EN 7   0
EN 8   0
EN 9   -14989
H2U-MY:LYS-S1
EN 3   0
EN 4   0
EN 5   0
EN 6   0
EN 7   0
EN 8   0
EN 9   -8447
A-RIB:GLU-CA
EN 3   0
EN 4   0
EN 5   -1333
EN 6   -1278
EN 7   -1673
EN 8   -1047
EN 9   -2489
FMU-RIB:ARG-S1
EN 3   0
EN 4   0
EN 5   0
EN 6   -15737
EN 7   0
EN 8   0
EN 9   -12509

QUO-M6:LYS-CA
EN 3   0
EN 4   0
EN 5   0
EN 6   0
EN 7   0
EN 8   0
EN 9   -12429
G-R6:TRP-S1
EN 3   0
EN 4   -7927
EN 5   -5228
EN 6   0
EN 7   -4563
EN 8   -1722
EN 9   -4312
C-Y:GLN-S2
EN 3   0
EN 4   0
EN 5   -3224
EN 6   -5338
EN 7   -5415
EN 8   -4765
EN 9   -4209
A-R5:GLU-CA
EN 3   0
EN 4   0
EN 5   -329
EN 6   2709
EN 7   -1381
EN 8   -1815
EN 9   -1484
G-R6:SER-S1
EN 3   0
EN 4   0
EN 5   -1728
EN 6   -4224
EN 7   -3292
EN 8   -3043
EN 9   -3335
A-R5:ARG-S2
EN 3   0
EN 4   -10429
EN 5   -4771
EN 6   -5514
EN 7   -3979
EN 8   -4992
EN 9   -5970
5BU-RIB:PRO-S1
EN 3   0
EN 4   0
EN 5   0
EN 6   0
EN 7   -13909
EN 8   0
EN 9   0
G-P:ASN-S1
EN 3   0

EN 4   -5274
EN 5   -6391
EN 6   -4688
EN 7   -5543
EN 8   -4774
EN 9   -4622
U-P:ARG-CA
EN 3   0
EN 4   -5247
EN 5   -4647
EN 6   -5666
EN 7   -4605
EN 8   -5460
EN 9   -5294
FMU-MY:VAL-S1
EN 3   0
EN 4   0
EN 5   0
EN 6   0
EN 7   0
EN 8   -15068
EN 9   0
C-Y:LYS-CA
EN 3   0
EN 4   0
EN 5   0
EN 6   -2649
EN 7   -1879
EN 8   -2473
EN 9   -3700
U-RIB:THR-CA
EN 3   0
EN 4   0
EN 5   0
EN 6   -4957
EN 7   -4132
EN 8   -4799
EN 9   -4064
U31-RIB:ASP-S1
EN 3   0
EN 4   0
EN 5   0
EN 6   -15988
EN 7   -14926
EN 8   -15520
EN 9   0
FHU-RIB:LYS-S2
EN 3   0
EN 4   0
EN 5   0
EN 6   0
EN 7   0
EN 8   0
EN 9   -13487
U34-P:HIS-S2
EN 3   0
EN 4   0
EN 5   -19622

EN 6   0
EN 7   0
EN 8   0
EN 9   -15072
H2U-MY:THR-CA
EN 3   0
EN 4   0
EN 5   0
EN 6   0
EN 7   0
EN 8   0
EN 9   -10990
G-RIB:TYR-S2
EN 3   0
EN 4   0
EN 5   0
EN 6   -4634
EN 7   -2751
EN 8   -3598
EN 9   -3713
IU-MY:SER-CA
EN 3   0
EN 4   0
EN 5   0
EN 6   0
EN 7   -10964
EN 8   0
EN 9   0
IU-P:ILE-S1
EN 3   0
EN 4   0
EN 5   0
EN 6   0
EN 7   0
EN 8   0
EN 9   -10281
QUO-M6:LEU-S1
EN 3   0
EN 4   0
EN 5   0
EN 6   0
EN 7   0
EN 8   -12912
EN 9   0
G-R6:GLU-S1
EN 3   0
EN 4   0
EN 5   0
EN 6   -1177
EN 7   -1781
EN 8   -1782
EN 9   -1652
G-P:ASP-CA
EN 3   0
EN 4   0
EN 5   -2757
EN 6   833
EN 7   -2926

EN 8   -4001
EN 9   -4010
GTP-M5:GLY-CA
EN 3   0
EN 4   0
EN 5   0
EN 6   0
EN 7   0
EN 8   0
EN 9   -12404
IU-RIB:PRO-S1
EN 3   0
EN 4   0
EN 5   0
EN 6   0
EN 7   -12407
EN 8   -10279
EN 9   0
IU-RIB:LYS-S1
EN 3   0
EN 4   0
EN 5   -13765
EN 6   0
EN 7   0
EN 8   -10259
EN 9   -10933
G-P:GLN-S1
EN 3   0
EN 4   -5517
EN 5   -5539
EN 6   -3214
EN 7   -5008
EN 8   -4846
EN 9   -4686
FHU-RIB:TYR-CA
EN 3   0
EN 4   0
EN 5   0
EN 6   -17551
EN 7   0
EN 8   0
EN 9   0
FHU-P:ALA-S1
EN 3   0
EN 4   0
EN 5   0
EN 6   0
EN 7   -12466
EN 8   0
EN 9   0
IU-RIB:ARG-S1
EN 3   0
EN 4   0
EN 5   0
EN 6   -12518
EN 7   -10452
EN 8   0
EN 9   0

G-R5:HIS-CA
EN 3   0
EN 4   0
EN 5   -1041
EN 6   -1437
EN 7   -3390
EN 8   -2687
EN 9   -3754
G-R5:GLN-S1
EN 3   0
EN 4   0
EN 5   -68
EN 6   1252
EN 7   -3391
EN 8   -3272
EN 9   -4182
C-RIB:ARG-S1
EN 3   0
EN 4   0
EN 5   -5054
EN 6   -6003
EN 7   -6054
EN 8   -6310
EN 9   -6083
U-Y:ALA-CA
EN 3   0
EN 4   0
EN 5   -2050
EN 6   -2708
EN 7   -1385
EN 8   -1687
EN 9   -2653
A-R5:ASN-S1
EN 3   0
EN 4   0
EN 5   -385
EN 6   -2499
EN 7   -3155
EN 8   -4510
EN 9   -3908
C-P:LEU-S1
EN 3   0
EN 4   0
EN 5   15
EN 6   -933
EN 7   -3180
EN 8   -1630
EN 9   -2380
G-RIB:ARG-CA
EN 3   0
EN 4   0
EN 5   -1216
EN 6   -4832
EN 7   -3881
EN 8   -4249
EN 9   -5010
C-Y:PHE-CA
EN 3   0

EN 4   0
EN 5   0
EN 6   -745
EN 7   315
EN 8   434
EN 9   -1956
C31-MY:LEU-S2
EN 3   0
EN 4   -21842
EN 5   0
EN 6   0
EN 7   0
EN 8   0
EN 9   0
U-RIB:VAL-S1
EN 3   0
EN 4   0
EN 5   -4666
EN 6   -2286
EN 7   -672
EN 8   -2271
EN 9   -2366
U34-RIB:TYR-S2
EN 3   0
EN 4   0
EN 5   0
EN 6   0
EN 7   0
EN 8   0
EN 9   -14327
G-R6:ALA-S1
EN 3   0
EN 4   -3423
EN 5   280
EN 6   -1833
EN 7   2994
EN 8   -2370
EN 9   -1618
U34-MY:VAL-CA
EN 3   0
EN 4   0
EN 5   0
EN 6   0
EN 7   0
EN 8   0
EN 9   -12305
G-P:ARG-S1
EN 3   0
EN 4   0
EN 5   -6496
EN 6   -6277
EN 7   -5625
EN 8   -6593
EN 9   -5573
C31-P:TYR-S2
EN 3   0
EN 4   0
EN 5   0

EN 6   -17556
EN 7   -16494
EN 8   0
EN 9   0
FHU-RIB:ASP-S1
EN 3   0
EN 4   0
EN 5   0
EN 6   0
EN 7   -14926
EN 8   0
EN 9   0
C-RIB:VAL-S1
EN 3   0
EN 4   0
EN 5   -3848
EN 6   -3914
EN 7   -4386
EN 8   -2612
EN 9   -2809
U-P:TRP-S2
EN 3   0
EN 4   0
EN 5   0
EN 6   -5239
EN 7   -6709
EN 8   -3767
EN 9   -4110
U-P:HIS-CA
EN 3   0
EN 4   0
EN 5   -2372
EN 6   0
EN 7   -5142
EN 8   -4311
EN 9   -6079
C31-RIB:GLU-S1
EN 3   0
EN 4   0
EN 5   0
EN 6   0
EN 7   -14240
EN 8   0
EN 9   0
C31-MY:GLU-S1
EN 3   0
EN 4   0
EN 5   0
EN 6   0
EN 7   0
EN 8   0
EN 9   -12073
DA-M6:ASN-CA
EN 3   0
EN 4   0
EN 5   0
EN 6   0
EN 7   0

EN 8   0
EN 9   -13840
G-R5:PHE-S2
EN 3   0
EN 4   -7088
EN 5   0
EN 6   -346
EN 7   -73
EN 8   -883
EN 9   -1105
G-RIB:THR-CA
EN 3   0
EN 4   0
EN 5   -2680
EN 6   -3630
EN 7   -3020
EN 8   -3162
EN 9   -3433
A-RIB:ASP-CA
EN 3   0
EN 4   0
EN 5   -1020
EN 6   -1969
EN 7   -2625
EN 8   -2666
EN 9   -2919
DA-M5:THR-S1
EN 3   0
EN 4   0
EN 5   0
EN 6   0
EN 7   -15427
EN 8   0
EN 9   0
FHU-MY:PHE-S2
EN 3   0
EN 4   0
EN 5   0
EN 6   0
EN 7   0
EN 8   -13290
EN 9   0
IU-MY:ILE-S1
EN 3   0
EN 4   0
EN 5   0
EN 6   0
EN 7   0
EN 8   -9823
EN 9   0
5BU-P:ARG-CA
EN 3   0
EN 4   0
EN 5   0
EN 6   0
EN 7   0
EN 8   0
EN 9   -12509

U31-P:ASP-S1
EN 3 0
EN 4 -22730
EN 5 0
EN 6 0
EN 7 0
EN 8 -13803
EN 9 0
C-Y:CYS-S1
EN 3 0
EN 4 0
EN 5 -3404
EN 6 0
EN 7 0
EN 8 101
EN 9 -1577
A-P:CYS-S1
EN 3 0
EN 4 0
EN 5 -6286
EN 6 0
EN 7 0
EN 8 0
EN 9 -3454
G-R5:GLU-CA
EN 3 0
EN 4 0
EN 5 1948
EN 6 0
EN 7 342
EN 8 -994
EN 9 -170
A-P:ALA-CA
EN 3 0
EN 4 -3985
EN 5 -4008
EN 6 -3184
EN 7 -3840
EN 8 -3131
EN 9 -2678
U-P:LYS-S1
EN 3 0
EN 4 -5174
EN 5 -5909
EN 6 -7018
EN 7 -5443
EN 8 -5125
EN 9 -4945
FHU-RIB:SER-S1
EN 3 0
EN 4 0
EN 5 0
EN 6 0
EN 7 0
EN 8 -14064
EN 9 -13021
QUO-RIB:LEU-S2
EN 3 0

EN 4 0
EN 5 0
EN 6 0
EN 7 -15756
EN 8 0
EN 9 0
U34-P:ASN-S1
EN 3 0
EN 4 0
EN 5 -18389
EN 6 0
EN 7 0
EN 8 -16601
EN 9 -13840
C-P:TYR-CA
EN 3 0
EN 4 0
EN 5 -721
EN 6 0
EN 7 -4280
EN 8 -1655
EN 9 -4147
FHU-P:ARG-S2
EN 3 0
EN 4 0
EN 5 0
EN 6 -15749
EN 7 0
EN 8 0
EN 9 -10803
C-P:LYS-S1
EN 3 0
EN 4 -4255
EN 5 -6492
EN 6 -7052
EN 7 -6144
EN 8 -4867
EN 9 -4846
U31-P:ARG-S1
EN 3 0
EN 4 0
EN 5 0
EN 6 0
EN 7 0
EN 8 0
EN 9 -12509
A-R5:ASP-CA
EN 3 0
EN 4 0
EN 5 -1020
EN 6 2018
EN 7 -1741
EN 8 -1738
EN 9 -1773
FMU-P:PHE-CA
EN 3 0
EN 4 0
EN 5 0

EN 6   -17189
EN 7   0
EN 8   0
EN 9   0
H2U-MY:ALA-CA
EN 3   0
EN 4   0
EN 5   0
EN 6   0
EN 7   0
EN 8   0
EN 9   -8028
U-RIB:LEU-CA
EN 3   0
EN 4   0
EN 5   -898
EN 6   -2681
EN 7   -233
EN 8   -827
EN 9   -2400
QUO-M5:LEU-CA
EN 3   0
EN 4   0
EN 5   0
EN 6   0
EN 7   0
EN 8   -12912
EN 9   0
A-R6:MET-CA
EN 3   0
EN 4   0
EN 5   -3251
EN 6   -1930
EN 7   -3591
EN 8   -1463
EN 9   -3523
C31-RIB:PHE-S2
EN 3   0
EN 4   0
EN 5   -18513
EN 6   0
EN 7   -16131
EN 8   0
EN 9   0
U31-RIB:MET-S2
EN 3   0
EN 4   0
EN 5   0
EN 6   -18228
EN 7   0
EN 8   0
EN 9   0
U34-MY:ASN-S2
EN 3   0
EN 4   0
EN 5   0
EN 6   0
EN 7   0

EN 8 -16622
EN 9 0
U-P:PHE-S1
EN 3 0
EN 4 0
EN 5 -1277
EN 6 0
EN 7 -2330
EN 8 -3216
EN 9 -2433
A-P:MET-CA
EN 3 0
EN 4 0
EN 5 -4971
EN 6 -215
EN 7 -2589
EN 8 -2852
EN 9 -3526
G-R5:ALA-CA
EN 3 0
EN 4 -3423
EN 5 280
EN 6 -115
EN 7 -440
EN 8 -1887
EN 9 -1708
A-R6:LYS-CA
EN 3 0
EN 4 0
EN 5 -1697
EN 6 -1088
EN 7 -2036
EN 8 -3444
EN 9 -2681
FHU-MY:LEU-S1
EN 3 0
EN 4 0
EN 5 0
EN 6 0
EN 7 -15040
EN 8 -11194
EN 9 -10151
FHU-MY:LEU-CA
EN 3 0
EN 4 0
EN 5 0
EN 6 -13379
EN 7 -12318
EN 8 -12912
EN 9 0
I-RIB:ALA-S1
EN 3 0
EN 4 0
EN 5 0
EN 6 0
EN 7 0
EN 8 -13060
EN 9 0

G-R6:GLU-S2
EN 3 0
EN 4 0
EN 5 -3733
EN 6 -2711
EN 7 -1813
EN 8 -2433
EN 9 -1863
H2U-P:LYS-S2
EN 3 0
EN 4 0
EN 5 0
EN 6 0
EN 7 0
EN 8 0
EN 9 -10212
A-P:TRP-S2
EN 3 0
EN 4 0
EN 5 0
EN 6 -5183
EN 7 -3408
EN 8 -2285
EN 9 -3341
FHU-RIB:ILE-CA
EN 3 0
EN 4 0
EN 5 0
EN 6 0
EN 7 0
EN 8 -14047
EN 9 0
U-Y:LEU-CA
EN 3 0
EN 4 0
EN 5 0
EN 6 0
EN 7 -1620
EN 8 -2030
EN 9 -1501
G-RIB:ASP-S2
EN 3 0
EN 4 0
EN 5 -5145
EN 6 -2913
EN 7 -3117
EN 8 -3711
EN 9 -4077
U-P:HIS-S2
EN 3 0
EN 4 0
EN 5 -4107
EN 6 -5890
EN 7 -6164
EN 8 -6652
EN 9 -5263
G-RIB:HIS-S2
EN 3 0

EN 4 0
EN 5 -6210
EN 6 -6864
EN 7 -5658
EN 8 -5763
EN 9 -4581
G-R5:GLU-S1
EN 3 0
EN 4 0
EN 5 223
EN 6 1544
EN 7 -1050
EN 8 72
EN 9 -1997
C-P:SER-CA
EN 3 0
EN 4 -6558
EN 5 -6714
EN 6 -4808
EN 7 -5203
EN 8 -4421
EN 9 -4611
A-R6:ASN-S2
EN 3 0
EN 4 0
EN 5 -3128
EN 6 -5916
EN 7 -4893
EN 8 -4062
EN 9 -3929
G-P:ASP-S2
EN 3 0
EN 4 0
EN 5 -1539
EN 6 -3653
EN 7 -4691
EN 8 -4120
EN 9 -4533
G-R6:ASP-S1
EN 3 0
EN 4 0
EN 5 -462
EN 6 -2245
EN 7 -3382
EN 8 -2069
EN 9 -2622
G-R6:TYR-CA
EN 3 0
EN 4 0
EN 5 0
EN 6 -3096
EN 7 -1361
EN 8 475
EN 9 -1203
C-Y:ASP-S2
EN 3 0
EN 4 -4614
EN 5 -3632

EN 6 -2311
EN 7 -4106
EN 8 -3662
EN 9 -3071
C-Y:SER-CA
EN 3 0
EN 4 0
EN 5 590
EN 6 -2910
EN 7 -3736
EN 8 -3743
EN 9 -3669
C-RIB:ASN-S1
EN 3 0
EN 4 -7367
EN 5 -2950
EN 6 -5263
EN 7 -5505
EN 8 -4889
EN 9 -5055
C31-P:PHE-S1
EN 3 0
EN 4 0
EN 5 0
EN 6 0
EN 7 0
EN 8 -15004
EN 9 0
FHU-MY:ARG-CA
EN 3 0
EN 4 0
EN 5 0
EN 6 -14020
EN 7 -12958
EN 8 0
EN 9 -13513
C-P:SER-S1
EN 3 0
EN 4 -10284
EN 5 -6841
EN 6 -5393
EN 7 -4580
EN 8 -4722
EN 9 -4972
G-R6:CYS-S1
EN 3 0
EN 4 0
EN 5 0
EN 6 -1680
EN 7 -619
EN 8 0
EN 9 -1174
A-RIB:TRP-S1
EN 3 0
EN 4 0
EN 5 -3066
EN 6 -1745
EN 7 -4119

EN 8   -4382
EN 9   -3961
U31-MY:VAL-CA
EN 3   0
EN 4   0
EN 5   0
EN 6   0
EN 7   0
EN 8   0
EN 9   -12305
G-R6:ASP-CA
EN 3   0
EN 4   0
EN 5   0
EN 6   -143
EN 7   -2715
EN 8   -1731
EN 9   -2168
G-R5:CYS-S1
EN 3   0
EN 4   0
EN 5   0
EN 6   0
EN 7   -619
EN 8   504
EN 9   1547
QUO-M6:LYS-S1
EN 3   0
EN 4   0
EN 5   0
EN 6   0
EN 7   0
EN 8   0
EN 9   -12435
A-R5:TYR-S2
EN 3   0
EN 4   -8011
EN 5   -3594
EN 6   -2986
EN 7   -4195
EN 8   -4076
EN 9   -2264
U34-RIB:ASN-CA
EN 3   0
EN 4   0
EN 5   0
EN 6   0
EN 7   0
EN 8   -14883
EN 9   -16562
A-P:ASN-S2
EN 3   0
EN 4   0
EN 5   -6565
EN 6   -4240
EN 7   -4736
EN 8   -4777
EN 9   -4368

H2U-MY:THR-S1
EN 3 0
EN 4 0
EN 5 0
EN 6 0
EN 7 0
EN 8 0
EN 9 -9272
U-Y:GLY-CA
EN 3 0
EN 4 0
EN 5 -2438
EN 6 -4934
EN 7 -1322
EN 8 -4186
EN 9 -4706
C31-RIB:GLU-S2
EN 3 0
EN 4 0
EN 5 0
EN 6 -15334
EN 7 0
EN 8 0
EN 9 -12105
G-R5:LEU-S1
EN 3 0
EN 4 0
EN 5 -1288
EN 6 -520
EN 7 1093
EN 8 -289
EN 9 -324
A-P:VAL-CA
EN 3 0
EN 4 0
EN 5 -3293
EN 6 -254
EN 7 -2412
EN 8 -2770
EN 9 -2647
5BU-P:PRO-S1
EN 3 0
EN 4 0
EN 5 0
EN 6 -14971
EN 7 0
EN 8 0
EN 9 0
G-R5:TYR-CA
EN 3 0
EN 4 0
EN 5 404
EN 6 0
EN 7 -2150
EN 8 475
EN 9 -1863
U34-RIB:ASN-S1
EN 3 0

EN 4   0
EN 5   0
EN 6   0
EN 7   -17724
EN 8   -14883
EN 9   0
IU-RIB:GLN-S1
EN 3   0
EN 4   0
EN 5   0
EN 6   0
EN 7   0
EN 8   0
EN 9   -10864
U34-MY:PHE-CA
EN 3   0
EN 4   0
EN 5   0
EN 6   0
EN 7   0
EN 8   0
EN 9   -13960
U31-MY:GLN-S1
EN 3   0
EN 4   0
EN 5   0
EN 6   0
EN 7   -16250
EN 8   0
EN 9   0
FHU-MY:ASP-S1
EN 3   0
EN 4   0
EN 5   0
EN 6   -15988
EN 7   0
EN 8   0
EN 9   -12759
C-Y:LYS-S1
EN 3   0
EN 4   0
EN 5   -541
EN 6   -2655
EN 7   -3864
EN 8   -2479
EN 9   -4335
A-R5:SER-CA
EN 3   0
EN 4   0
EN 5   0
EN 6   -967
EN 7   -3539
EN 8   -3221
EN 9   -3603
C-RIB:PHE-CA
EN 3   0
EN 4   0
EN 5   0

EN 6   971
EN 7   -1954
EN 8   -2784
EN 9   -2154
H2U-P:GLU-S2
EN 3   0
EN 4   0
EN 5   0
EN 6   0
EN 7   -10285
EN 8   0
EN 9   -8118
A-P:HIS-S1
EN 3   0
EN 4   0
EN 5   -4325
EN 6   -3004
EN 7   -5378
EN 8   -5756
EN 9   -4597
FMU-MY:ARG-S1
EN 3   0
EN 4   0
EN 5   0
EN 6   0
EN 7   0
EN 8   -13552
EN 9   0
FMU-RIB:CYS-S1
EN 3   0
EN 4   0
EN 5   -21565
EN 6   0
EN 7   0
EN 8   0
EN 9   0
A-RIB:VAL-S1
EN 3   0
EN 4   0
EN 5   -2287
EN 6   -3688
EN 7   -3893
EN 8   -2216
EN 9   -2278
H2U-RIB:LEU-S2
EN 3   0
EN 4   0
EN 5   0
EN 6   0
EN 7   0
EN 8   0
EN 9   -7884
G-P:GLY-CA
EN 3   0
EN 4   -7272
EN 5   -6843
EN 6   -3037
EN 7   -5047

EN 8 -5096
EN 9 -4724
G-P:ARG-CA
EN 3 0
EN 4 0
EN 5 -3342
EN 6 -4220
EN 7 -5216
EN 8 -5903
EN 9 -5550
U-Y:ALA-S1
EN 3 0
EN 4 0
EN 5 -3626
EN 6 -3160
EN 7 -2390
EN 8 -644
EN 9 -2400
H2U-RIB:PRO-S1
EN 3 0
EN 4 0
EN 5 0
EN 6 0
EN 7 0
EN 8 -10515
EN 9 0
A-R5:GLY-CA
EN 3 0
EN 4 -4371
EN 5 -4890
EN 6 -3072
EN 7 -4441
EN 8 -4181
EN 9 -4818
U34-P:ASN-S2
EN 3 0
EN 4 0
EN 5 -18410
EN 6 0
EN 7 -16028
EN 8 0
EN 9 -13861
DA-M6:LYS-S2
EN 3 0
EN 4 -22453
EN 5 0
EN 6 0
EN 7 0
EN 8 0
EN 9 0
U-P:VAL-S1
EN 3 0
EN 4 0
EN 5 0
EN 6 -3125
EN 7 -3399
EN 8 -2828
EN 9 -3050

C-P:ARG-S1
EN 3   0
EN 4   -4328
EN 5   -8168
EN 6   -7470
EN 7   -5404
EN 8   -6466
EN 9   -5981
C-Y:VAL-S1
EN 3   0
EN 4   0
EN 5   -2683
EN 6   -2819
EN 7   1968
EN 8   -1545
EN 9   -1915
DA-RIB:HIS-CA
EN 3   0
EN 4   0
EN 5   0
EN 6   0
EN 7   -17223
EN 8   -16099
EN 9   0
G-P:LEU-S2
EN 3   0
EN 4   -3305
EN 5   -1318
EN 6   -2720
EN 7   -945
EN 8   -2147
EN 9   -1921
GTP-RIB:SER-S1
EN 3   0
EN 4   0
EN 5   0
EN 6   0
EN 7   -15188
EN 8   0
EN 9   -13021
FMU-MY:SER-S1
EN 3   0
EN 4   0
EN 5   0
EN 6   0
EN 7   0
EN 8   -14064
EN 9   0
H2U-RIB:PRO-CA
EN 3   0
EN 4   0
EN 5   0
EN 6   0
EN 7   0
EN 8   0
EN 9   -9472
G-P:PHE-S2
EN 3   0

EN 4 0
EN 5 0
EN 6 -3477
EN 7 -3949
EN 8 -1623
EN 9 -2137
A-R6:PHE-S1
EN 3 0
EN 4 -7644
EN 5 -2223
EN 6 0
EN 7 1876
EN 8 -1439
EN 9 -777
G-R5:HIS-S1
EN 3 0
EN 4 0
EN 5 -3763
EN 6 -3707
EN 7 -4033
EN 8 -3476
EN 9 -4540
A-R5:HIS-S2
EN 3 0
EN 4 -9761
EN 5 -5052
EN 6 -4284
EN 7 -5774
EN 8 -4822
EN 9 -5044
5BU-P:ILE-S1
EN 3 0
EN 4 0
EN 5 0
EN 6 -14514
EN 7 -13453
EN 8 0
EN 9 0
4SU-P:THR-S1
EN 3 0
EN 4 0
EN 5 0
EN 6 0
EN 7 0
EN 8 -14304
EN 9 0
FMU-MY:PHE-S1
EN 3 0
EN 4 0
EN 5 0
EN 6 0
EN 7 0
EN 8 -15004
EN 9 0
I-RIB:TRP-S2
EN 3 0
EN 4 0
EN 5 0

EN 6 0
EN 7 -18688
EN 8 0
EN 9 0
A-R6:HIS-S2
EN 3 0
EN 4 -11478
EN 5 -4340
EN 6 -5741
EN 7 -5392
EN 8 -4651
EN 9 -4088
U-RIB:ALA-S1
EN 3 0
EN 4 -4750
EN 5 -3316
EN 6 -3949
EN 7 -3966
EN 8 -3443
EN 9 -3703
A-R6:ILE-CA
EN 3 0
EN 4 0
EN 5 451
EN 6 -950
EN 7 1115
EN 8 -864
EN 9 -1538
U31-P:ASN-S2
EN 3 0
EN 4 0
EN 5 0
EN 6 -17089
EN 7 0
EN 8 0
EN 9 0
G-R6:THR-CA
EN 3 0
EN 4 0
EN 5 0
EN 6 357
EN 7 -1303
EN 8 -2449
EN 9 -2239
A-P:LEU-S2
EN 3 0
EN 4 0
EN 5 -1141
EN 6 -2542
EN 7 -25
EN 8 -1623
EN 9 -1292
FMU-MY:HIS-S1
EN 3 0
EN 4 0
EN 5 0
EN 6 0
EN 7 0

EN 8   0
EN 9   -15055
G-RIB:MET-CA
EN 3   0
EN 4   -6395
EN 5   -974
EN 6   0
EN 7   -3744
EN 8   -4007
EN 9   -2780
IU-RIB:HIS-S1
EN 3   0
EN 4   0
EN 5   0
EN 6   0
EN 7   -15269
EN 8   0
EN 9   0
G-R6:LYS-S2
EN 3   0
EN 4   -3889
EN 5   -3912
EN 6   -5655
EN 7   -3247
EN 8   -4223
EN 9   -3892
FHU-RIB:LEU-S2
EN 3   0
EN 4   0
EN 5   0
EN 6   0
EN 7   -12321
EN 8   -12915
EN 9   -11871
FMU-MY:ASN-S1
EN 3   0
EN 4   0
EN 5   0
EN 6   0
EN 7   0
EN 8   0
EN 9   -13840
A-R6:ILE-S1
EN 3   0
EN 4   0
EN 5   -1266
EN 6   1771
EN 7   2833
EN 8   -2199
EN 9   -1538
A-RIB:ALA-S1
EN 3   0
EN 4   -3982
EN 5   -3001
EN 6   -3397
EN 7   -3838
EN 8   -2599
EN 9   -3025

H2U-P:ASN-S1
EN 3 0
EN 4 0
EN 5 0
EN 6 0
EN 7 -12019
EN 8 -12613
EN 9 0
C-RIB:TRP-S2
EN 3 0
EN 4 0
EN 5 -2909
EN 6 -5405
EN 7 -4966
EN 8 -3391
EN 9 -3804
H2U-RIB:ASN-S1
EN 3 0
EN 4 0
EN 5 0
EN 6 0
EN 7 0
EN 8 0
EN 9 -11570
QUO-M6:LEU-CA
EN 3 0
EN 4 0
EN 5 0
EN 6 0
EN 7 0
EN 8 0
EN 9 -11868
IU-RIB:HIS-CA
EN 3 0
EN 4 0
EN 5 0
EN 6 -14060
EN 7 -14003
EN 8 0
EN 9 0
U31-P:SER-CA
EN 3 0
EN 4 0
EN 5 0
EN 6 0
EN 7 0
EN 8 0
EN 9 -13021
A-RIB:ARG-S1
EN 3 0
EN 4 0
EN 5 -3493
EN 6 -5708
EN 7 -5618
EN 8 -6144
EN 9 -6012
A-P:PHE-S1
EN 3 0

EN 4 0
EN 5 -2225
EN 6 812
EN 7 -2565
EN 8 -2154
EN 9 -3254
U31-MY:PHE-CA
EN 3 0
EN 4 0
EN 5 0
EN 6 0
EN 7 -16127
EN 8 0
EN 9 0
U34-P:SER-CA
EN 3 0
EN 4 0
EN 5 0
EN 6 0
EN 7 0
EN 8 -14064
EN 9 -13021
U-P:TYR-CA
EN 3 0
EN 4 0
EN 5 0
EN 6 -319
EN 7 -975
EN 8 147
EN 9 -3800
U31-P:HIS-CA
EN 3 0
EN 4 0
EN 5 0
EN 6 0
EN 7 -17223
EN 8 0
EN 9 0
I-RIB:GLY-CA
EN 3 0
EN 4 0
EN 5 0
EN 6 0
EN 7 0
EN 8 0
EN 9 -12404
C31-P:ASN-S1
EN 3 0
EN 4 0
EN 5 0
EN 6 0
EN 7 0
EN 8 -14883
EN 9 0
G-R6:MET-CA
EN 3 0
EN 4 0
EN 5 -974

```
EN 6   -1370
EN 7   -2579
EN 8   -3173
EN 9   -1246
C-Y:GLY-CA
EN 3   0
EN 4   -4214
EN 5   -2780
EN 6   -3628
EN 7   -3706
EN 8   -4096
EN 9   -5006
A-RIB:TYR-S2
EN 3   0
EN 4   0
EN 5   -6316
EN 6   -5907
EN 7   -1212
EN 8   -2519
EN 9   -3618
U-Y:PHE-CA
EN 3   0
EN 4   0
EN 5   0
EN 6   -1669
EN 7   -3591
EN 8   -1754
EN 9   -2664
DA-M5:ASN-S1
EN 3   0
EN 4   0
EN 5   0
EN 6   0
EN 7   0
EN 8   -14883
EN 9   0
U31-MY:ASP-CA
EN 3   0
EN 4   0
EN 5   0
EN 6   -17703
EN 7   0
EN 8   -13801
EN 9   0
FMU-MY:ASP-S2
EN 3   0
EN 4   0
EN 5   0
EN 6   -16033
EN 7   0
EN 8   0
EN 9   0
IU-P:LYS-S2
EN 3   0
EN 4   0
EN 5   -14310
EN 6   0
EN 7   0
```

EN 8   0
EN 9   -9761
G-R5:PRO-S1
EN 3   0
EN 4   0
EN 5   -3432
EN 6   158
EN 7   -3219
EN 8   -2478
EN 9   -3957
H2U-RIB:LYS-S1
EN 3   0
EN 4   0
EN 5   0
EN 6   0
EN 7   0
EN 8   0
EN 9   -8447
FMU-RIB:GLN-S2
EN 3   0
EN 4   0
EN 5   0
EN 6   0
EN 7   0
EN 8   -15157
EN 9   0
H2U-RIB:TRP-S2
EN 3   0
EN 4   0
EN 5   0
EN 6   0
EN 7   -14700
EN 8   0
EN 9   0
A-R6:GLU-S1
EN 3   0
EN 4   0
EN 5   -1340
EN 6   -1737
EN 7   -1941
EN 8   -1822
EN 9   -2044
IU-RIB:LEU-CA
EN 3   0
EN 4   0
EN 5   0
EN 6   -10873
EN 7   0
EN 8   0
EN 9   -9362
U-P:ARG-S2
EN 3   0
EN 4   0
EN 5   -9430
EN 6   -7464
EN 7   -5528
EN 8   -6364
EN 9   -6267

G-R6:GLU-CA
EN 3   0
EN 4   0
EN 5   0
EN 6   3269
EN 7   -490
EN 8   -1337
EN 9   -1452
DA-RIB:ASN-S2
EN 3   0
EN 4   0
EN 5   0
EN 6   -17089
EN 7   0
EN 8   0
EN 9   0
U-Y:ILE-CA
EN 3   0
EN 4   0
EN 5   0
EN 6   0
EN 7   -1368
EN 8   -1249
EN 9   -1923
U-RIB:THR-S1
EN 3   0
EN 4   0
EN 5   -5394
EN 6   -4404
EN 7   -4347
EN 8   -3936
EN 9   -4250
G-P:PRO-S1
EN 3   0
EN 4   0
EN 5   -5629
EN 6   -4308
EN 7   -4251
EN 8   -4775
EN 9   -4760
U-P:ASP-S1
EN 3   0
EN 4   -5498
EN 5   -2798
EN 6   -3195
EN 7   -2515
EN 8   -2988
EN 9   -3186
G-RIB:PHE-CA
EN 3   0
EN 4   0
EN 5   54
EN 6   -3446
EN 7   -1551
EN 8   -3460
EN 9   -2064
FHU-RIB:LEU-S1
EN 3   0

EN 4 0
EN 5 0
EN 6 0
EN 7 -12318
EN 8 -11194
EN 9 0
A-P:LEU-CA
EN 3 0
EN 4 0
EN 5 -133
EN 6 -530
EN 7 -855
EN 8 -851
EN 9 -2033
DA-M5:LYS-S1
EN 3 0
EN 4 0
EN 5 0
EN 6 0
EN 7 0
EN 8 -13479
EN 9 0
QUO-RIB:ASP-S1
EN 3 0
EN 4 0
EN 5 0
EN 6 0
EN 7 0
EN 8 0
EN 9 -12759
A-R6:SER-S1
EN 3 0
EN 4 0
EN 5 -5507
EN 6 -3397
EN 7 -3125
EN 8 -3482
EN 9 -2992
A-R5:ASN-CA
EN 3 0
EN 4 0
EN 5 0
EN 6 935
EN 7 -2824
EN 8 -3547
EN 9 -3379
A-P:GLU-S1
EN 3 0
EN 4 0
EN 5 1378
EN 6 982
EN 7 -1060
EN 8 -2410
EN 9 -3148
DA-M5:ASN-S2
EN 3 0
EN 4 0
EN 5 0

EN 6 0
EN 7 -16028
EN 8 0
EN 9 0
A-P:GLN-S2
EN 3 0
EN 4 -6083
EN 5 -6106
EN 6 -5046
EN 7 -3723
EN 8 -5499
EN 9 -4539
FMU-MY:PHE-S2
EN 3 0
EN 4 0
EN 5 0
EN 6 -17192
EN 7 -16131
EN 8 0
EN 9 0
IU-RIB:HIS-S2
EN 3 0
EN 4 0
EN 5 0
EN 6 0
EN 7 0
EN 8 -11892
EN 9 -11853
FHU-RIB:ARG-S2
EN 3 0
EN 4 0
EN 5 0
EN 6 0
EN 7 -16405
EN 8 0
EN 9 -10803
QUO-M6:ASP-CA
EN 3 0
EN 4 0
EN 5 0
EN 6 0
EN 7 0
EN 8 -13801
EN 9 0
U-P:MET-S1
EN 3 0
EN 4 0
EN 5 -2306
EN 6 -2702
EN 7 -2645
EN 8 -4505
EN 9 -3913
C31-P:MET-S2
EN 3 0
EN 4 0
EN 5 0
EN 6 0
EN 7 0

EN 8   -16043
EN 9   0
QUO-M6:PHE-S1
EN 3   0
EN 4   0
EN 5   0
EN 6   0
EN 7   -16127
EN 8   0
EN 9   -13960
G-R6:TYR-S1
EN 3   0
EN 4   0
EN 5   -1695
EN 6   -1258
EN 7   -3370
EN 8   3197
EN 9   -1916
U-RIB:SER-S1
EN 3   0
EN 4   -7472
EN 5   -5033
EN 6   -5367
EN 7   -4970
EN 8   -3645
EN 9   -4211
C31-P:PHE-CA
EN 3   0
EN 4   0
EN 5   0
EN 6   0
EN 7   0
EN 8   0
EN 9   -13960
GTP-RIB:THR-CA
EN 3   0
EN 4   0
EN 5   0
EN 6   0
EN 7   0
EN 8   0
EN 9   -13260
FHU-MY:SER-S1
EN 3   0
EN 4   0
EN 5   0
EN 6   0
EN 7   0
EN 8   0
EN 9   -11303
U31-P:MET-S1
EN 3   0
EN 4   0
EN 5   0
EN 6   -18217
EN 7   -17156
EN 8   0
EN 9   0

C-P:ILE-S1
EN 3 0
EN 4 0
EN 5 -1119
EN 6 -1515
EN 7 -2724
EN 8 -3057
EN 9 -2827
U31-P:TYR-S1
EN 3 0
EN 4 0
EN 5 0
EN 6 0
EN 7 0
EN 8 0
EN 9 -14323
C31-P:THR-CA
EN 3 0
EN 4 0
EN 5 0
EN 6 0
EN 7 -15427
EN 8 0
EN 9 -13260
A-R5:ASP-S2
EN 3 0
EN 4 0
EN 5 -2784
EN 6 -1463
EN 7 -2411
EN 8 -2864
EN 9 -2873
FHU-MY:TYR-S2
EN 3 0
EN 4 0
EN 5 0
EN 6 -18560
EN 7 -14777
EN 8 0
EN 9 0
FHU-MY:SER-CA
EN 3 0
EN 4 0
EN 5 0
EN 6 0
EN 7 0
EN 8 0
EN 9 -13021
FMU-RIB:HIS-S1
EN 3 0
EN 4 0
EN 5 0
EN 6 0
EN 7 0
EN 8 -16099
EN 9 0
U31-MY:MET-CA
EN 3 0

EN 4   0
EN 5   0
EN 6   -18217
EN 7   0
EN 8   0
EN 9   0
G-RIB:TRP-CA
EN 3   0
EN 4   0
EN 5   -2506
EN 6   -5173
EN 7   -3559
EN 8   -3822
EN 9   -3401
A-R6:THR-CA
EN 3   0
EN 4   0
EN 5   194
EN 6   -2924
EN 7   -4133
EN 8   -1121
EN 9   -3025
FMU-RIB:PHE-S1
EN 3   0
EN 4   0
EN 5   0
EN 6   0
EN 7   0
EN 8   -15004
EN 9   -13960
U31-RIB:TYR-S1
EN 3   0
EN 4   0
EN 5   0
EN 6   0
EN 7   0
EN 8   -15367
EN 9   0
H2U-P:GLU-CA
EN 3   0
EN 4   0
EN 5   0
EN 6   0
EN 7   0
EN 8   0
EN 9   -8078
FMU-MY:ARG-CA
EN 3   0
EN 4   0
EN 5   0
EN 6   0
EN 7   0
EN 8   -13552
EN 9   0
FHU-RIB:ASP-S2
EN 3   0
EN 4   0
EN 5   0

EN 6   -16033
EN 7   0
EN 8   0
EN 9   0
C31-P:ASP-S2
EN 3   0
EN 4   0
EN 5   -17354
EN 6   -16033
EN 7   -14972
EN 8   0
EN 9   0
FMU-P:VAL-S1
EN 3   0
EN 4   0
EN 5   0
EN 6   0
EN 7   0
EN 8   0
EN 9   -12307
C31-MY:GLN-S2
EN 3   0
EN 4   0
EN 5   0
EN 6   0
EN 7   -16281
EN 8   0
EN 9   0
QUO-P:PHE-S2
EN 3   0
EN 4   0
EN 5   0
EN 6   0
EN 7   0
EN 8   0
EN 9   -13964
DA-RIB:ASP-S1
EN 3   0
EN 4   0
EN 5   0
EN 6   -15988
EN 7   0
EN 8   0
EN 9   0
DA-M6:HIS-S1
EN 3   0
EN 4   0
EN 5   0
EN 6   -18284
EN 7   0
EN 8   0
EN 9   0
5BU-MY:ARG-S2
EN 3   0
EN 4   0
EN 5   0
EN 6   0
EN 7   0

EN 8   0
EN 9   -10803
FMU-MY:MET-CA
EN 3   0
EN 4   0
EN 5   0
EN 6   0
EN 7   0
EN 8   0
EN 9   -14989
FMU-RIB:ASP-S2
EN 3   0
EN 4   0
EN 5   0
EN 6   0
EN 7   0
EN 8   0
EN 9   -12804
IU-MY:ALA-CA
EN 3   0
EN 4   0
EN 5   -12342
EN 6   0
EN 7   0
EN 8   0
EN 9   -10514
FMU-MY:PRO-S1
EN 3   0
EN 4   0
EN 5   0
EN 6   0
EN 7   0
EN 8   0
EN 9   -13459
C-RIB:CYS-CA
EN 3   0
EN 4   0
EN 5   0
EN 6   -2083
EN 7   0
EN 8   -3886
EN 9   -3294
IU-P:ARG-CA
EN 3   0
EN 4   0
EN 5   0
EN 6   0
EN 7   -11954
EN 8   0
EN 9   -9787
G-RIB:PRO-S1
EN 3   0
EN 4   0
EN 5   -4266
EN 6   -5285
EN 7   -5406
EN 8   -4059
EN 9   -3559

FMU-RIB:GLN-S1
EN 3 0
EN 4 0
EN 5 0
EN 6 0
EN 7 0
EN 8 0
EN 9 -14083
H2U-MY:ARG-S2
EN 3 0
EN 4 0
EN 5 -13083
EN 6 0
EN 7 0
EN 8 -9577
EN 9 -10251
A-RIB:ILE-CA
EN 3 0
EN 4 0
EN 5 -1266
EN 6 -950
EN 7 -1988
EN 8 -1748
EN 9 -2768
G-R5:LEU-CA
EN 3 0
EN 4 0
EN 5 2146
EN 6 744
EN 7 1093
EN 8 -1509
EN 9 -847
C31-RIB:ASP-S1
EN 3 0
EN 4 0
EN 5 0
EN 6 0
EN 7 0
EN 8 0
EN 9 -12759
G-R5:ALA-S1
EN 3 0
EN 4 -3423
EN 5 -723
EN 6 -1120
EN 7 -1503
EN 8 -1515
EN 9 -2399
GTP-M6:SER-S1
EN 3 0
EN 4 0
EN 5 0
EN 6 0
EN 7 0
EN 8 0
EN 9 -14738
QUO-RIB:GLN-S2
EN 3 0

EN 4 0
EN 5 0
EN 6 0
EN 7 0
EN 8 0
EN 9 -14114
FMU-RIB:HIS-S2
EN 3 0
EN 4 0
EN 5 0
EN 6 0
EN 7 0
EN 8 -16116
EN 9 0
FHU-P:ARG-S1
EN 3 0
EN 4 0
EN 5 0
EN 6 0
EN 7 0
EN 8 -11835
EN 9 -10791
FHU-MY:ALA-CA
EN 3 0
EN 4 0
EN 5 0
EN 6 0
EN 7 0
EN 8 -14064
EN 9 0
G-R5:LYS-S2
EN 3 0
EN 4 -5607
EN 5 -6011
EN 6 -4570
EN 7 -4594
EN 8 -4102
EN 9 -5215
U-RIB:TRP-CA
EN 3 0
EN 4 0
EN 5 0
EN 6 -2512
EN 7 -3168
EN 8 -4767
EN 9 -4105
FHU-MY:THR-CA
EN 3 0
EN 4 0
EN 5 0
EN 6 0
EN 7 -13710
EN 8 -14304
EN 9 -11543
U34-P:TYR-S2
EN 3 0
EN 4 0
EN 5 0

EN 6   -17556
EN 7   0
EN 8   0
EN 9   0
DA-M5:GLU-S2
EN 3   0
EN 4   0
EN 5   0
EN 6   0
EN 7   -14272
EN 8   0
EN 9   0
GTP-RIB:ASN-S1
EN 3   0
EN 4   0
EN 5   -18389
EN 6   0
EN 7   0
EN 8   0
EN 9   0
U31-P:LEU-S2
EN 3   0
EN 4   0
EN 5   0
EN 6   0
EN 7   0
EN 8   0
EN 9   -11871
FMU-P:GLN-CA
EN 3   0
EN 4   0
EN 5   0
EN 6   0
EN 7   0
EN 8   0
EN 9   -14083
IU-MY:ILE-CA
EN 3   0
EN 4   0
EN 5   0
EN 6   0
EN 7   0
EN 8   -9823
EN 9   -9784
A-RIB:GLN-CA
EN 3   0
EN 4   0
EN 5   -3350
EN 6   -2029
EN 7   -2685
EN 8   -3992
EN 9   -3374
C31-P:LEU-S2
EN 3   0
EN 4   0
EN 5   0
EN 6   0
EN 7   0

EN 8   0
EN 9   -11871
G-RIB:LEU-S1
EN 3   0
EN 4   0
EN 5   -1841
EN 6   -520
EN 7   -2010
EN 8   -2116
EN 9   -1470
DA-RIB:ASP-S2
EN 3   0
EN 4   0
EN 5   0
EN 6   0
EN 7   0
EN 8   -13848
EN 9   0
5BU-MY:PRO-CA
EN 3   0
EN 4   0
EN 5   0
EN 6   0
EN 7   0
EN 8   0
EN 9   -11742
C-RIB:TYR-S2
EN 3   0
EN 4   0
EN 5   -716
EN 6   -4745
EN 7   -4872
EN 8   -2915
EN 9   -3825
IU-RIB:ILE-S1
EN 3   0
EN 4   0
EN 5   0
EN 6   0
EN 7   -10947
EN 8   -10828
EN 9   0
C-P:CYS-S1
EN 3   0
EN 4   0
EN 5   0
EN 6   -2093
EN 7   -1032
EN 8   -2630
EN 9   -2852
C31-P:ASP-CA
EN 3   0
EN 4   0
EN 5   0
EN 6   0
EN 7   -14924
EN 8   -13801
EN 9   -12757

U34-P:SER-S1
EN 3   0
EN 4   0
EN 5   0
EN 6   0
EN 7   0
EN 8   0
EN 9   -15743
U31-RIB:GLN-S1
EN 3   0
EN 4   0
EN 5   -18632
EN 6   0
EN 7   0
EN 8   0
EN 9   0
U-P:TYR-S1
EN 3   0
EN 4   0
EN 5   0
EN 6   -319
EN 7   741
EN 8   -2122
EN 9   -3446
A-R6:PHE-S2
EN 3   0
EN 4   -5930
EN 5   -3231
EN 6   -3628
EN 7   -849
EN 8   -991
EN 9   -1665
FHU-RIB:PRO-CA
EN 3   0
EN 4   0
EN 5   0
EN 6   0
EN 7   0
EN 8   0
EN 9   -11742
A-R6:ARG-CA
EN 3   0
EN 4   0
EN 5   -1776
EN 6   -2554
EN 7   -3692
EN 8   -3524
EN 9   -3867
C-P:TRP-S2
EN 3   0
EN 4   0
EN 5   -4636
EN 6   -4320
EN 7   -4976
EN 8   -3401
EN 9   -4311
G-R6:HIS-S1
EN 3   0

EN 4   0
EN 5   -2758
EN 6   -4872
EN 7   -3480
EN 8   -4555
EN 9   -4122
C31-RIB:ASP-S2
EN 3   0
EN 4   0
EN 5   0
EN 6   0
EN 7   0
EN 8   0
EN 9   -14522
DA-M5:HIS-S1
EN 3   0
EN 4   0
EN 5   0
EN 6   -18284
EN 7   0
EN 8   0
EN 9   0
FHU-RIB:PRO-S1
EN 3   0
EN 4   0
EN 5   0
EN 6   0
EN 7   0
EN 8   -12786
EN 9   0
A-RIB:HIS-S1
EN 3   0
EN 4   0
EN 5   -5588
EN 6   -6221
EN 7   -5574
EN 8   -5517
EN 9   -3761
U31-RIB:ASN-CA
EN 3   0
EN 4   0
EN 5   0
EN 6   0
EN 7   0
EN 8   -14883
EN 9   0
C-RIB:LEU-S1
EN 3   0
EN 4   0
EN 5   25
EN 6   -2380
EN 7   -2413
EN 8   -3312
EN 9   -3083
H2U-RIB:GLU-S2
EN 3   0
EN 4   0
EN 5   0

EN 6   0
EN 7   0
EN 8   -9161
EN 9   0
IU-MY:LEU-S2
EN 3   0
EN 4   0
EN 5   0
EN 6   0
EN 7   -9815
EN 8   0
EN 9   0
U34-MY:ASP-CA
EN 3   0
EN 4   0
EN 5   0
EN 6   0
EN 7   0
EN 8   0
EN 9   -12757
QUO-M6:GLU-S1
EN 3   0
EN 4   0
EN 5   0
EN 6   0
EN 7   -14240
EN 8   0
EN 9   0
U-RIB:ASN-CA
EN 3   0
EN 4   0
EN 5   -1152
EN 6   -4653
EN 7   -5309
EN 8   -4516
EN 9   -4859
G-P:TRP-S2
EN 3   0
EN 4   0
EN 5   0
EN 6   -3934
EN 7   -4590
EN 8   -4732
EN 9   -5004
U31-P:ARG-CA
EN 3   0
EN 4   0
EN 5   0
EN 6   0
EN 7   0
EN 8   0
EN 9   -12509
A-R6:TYR-S1
EN 3   0
EN 4   -9012
EN 5   -868
EN 6   -2982
EN 7   -3307

EN 8 -3856
EN 9 -2476
DA-M6:TYR-S2
EN 3 0
EN 4 0
EN 5 0
EN 6 0
EN 7 0
EN 8 0
EN 9 -14327
FHU-RIB:TYR-S2
EN 3 0
EN 4 0
EN 5 -18877
EN 6 0
EN 7 -14777
EN 8 -13653
EN 9 0
G-R5:ASN-S2
EN 3 0
EN 4 0
EN 5 -4180
EN 6 -4103
EN 7 -4333
EN 8 -4109
EN 9 -4106
IU-P:HIS-S2
EN 3 0
EN 4 0
EN 5 0
EN 6 -15579
EN 7 0
EN 8 0
EN 9 0
DA-M5:SER-CA
EN 3 0
EN 4 0
EN 5 0
EN 6 -16249
EN 7 0
EN 8 0
EN 9 -13021
QUO-M5:ASP-S1
EN 3 0
EN 4 0
EN 5 0
EN 6 0
EN 7 0
EN 8 -13803
EN 9 -12759
G-R6:VAL-CA
EN 3 0
EN 4 0
EN 5 0
EN 6 -1409
EN 7 -348
EN 8 775
EN 9 -1004

GTP-M5:SER-CA
EN 3 0
EN 4 0
EN 5 0
EN 6 0
EN 7 0
EN 8 -14064
EN 9 0
FMU-MY:ALA-CA
EN 3 0
EN 4 0
EN 5 0
EN 6 -15244
EN 7 0
EN 8 0
EN 9 0
U31-P:ALA-S1
EN 3 0
EN 4 0
EN 5 0
EN 6 0
EN 7 0
EN 8 0
EN 9 -12016
U34-MY:SER-S1
EN 3 0
EN 4 0
EN 5 -17570
EN 6 0
EN 7 -15188
EN 8 0
EN 9 0
U31-MY:GLN-S2
EN 3 0
EN 4 0
EN 5 0
EN 6 0
EN 7 0
EN 8 -15157
EN 9 0
A-P:GLY-CA
EN 3 0
EN 4 -8361
EN 5 -7899
EN 6 -4792
EN 7 -4736
EN 8 -6042
EN 9 -4857
GTP-M6:GLY-CA
EN 3 0
EN 4 0
EN 5 0
EN 6 0
EN 7 0
EN 8 -13448
EN 9 0
G-R6:ALA-CA
EN 3 0

EN 4 0
EN 5 -723
EN 6 -668
EN 7 -440
EN 8 -990
EN 9 -2261
A-RIB:ALA-CA
EN 3 0
EN 4 0
EN 5 1438
EN 6 -3397
EN 7 -3838
EN 8 -1925
EN 9 -2520
C31-P:GLU-S1
EN 3 0
EN 4 0
EN 5 0
EN 6 0
EN 7 -14240
EN 8 0
EN 9 0
H2U-MY:ILE-CA
EN 3 0
EN 4 0
EN 5 0
EN 6 0
EN 7 0
EN 8 0
EN 9 -9016
G-R5:GLN-CA
EN 3 0
EN 4 0
EN 5 0
EN 6 0
EN 7 596
EN 8 -1384
EN 9 -4106
H2U-MY:TRP-S1
EN 3 0
EN 4 0
EN 5 0
EN 6 0
EN 7 -14700
EN 8 0
EN 9 0
C-RIB:TYR-CA
EN 3 0
EN 4 0
EN 5 0
EN 6 -2825
EN 7 -3773
EN 8 -4225
EN 9 -3277
DA-RIB:ARG-S2
EN 3 0
EN 4 0
EN 5 0

EN 6   0
EN 7   0
EN 8   0
EN 9   -12521
C31-P:GLN-S1
EN 3   0
EN 4   0
EN 5   0
EN 6   0
EN 7   0
EN 8   -15127
EN 9   0
G-R5:GLN-S2
EN 3   0
EN 4   -5520
EN 5   -2821
EN 6   -4935
EN 7   -4736
EN 8   -4252
EN 9   -3693
C-Y:ASN-CA
EN 3   0
EN 4   0
EN 5   -228
EN 6   1092
EN 7   -2285
EN 8   -1875
EN 9   -3845
U-P:LEU-S2
EN 3   0
EN 4   0
EN 5   0
EN 6   -589
EN 7   -2250
EN 8   -3105
EN 9   -2614
U31-MY:ILE-S1
EN 3   0
EN 4   0
EN 5   0
EN 6   0
EN 7   0
EN 8   -14047
EN 9   0
C-RIB:ASP-S1
EN 3   0
EN 4   0
EN 5   -3969
EN 6   -5486
EN 7   -4424
EN 8   -2803
EN 9   -3738
A-R6:VAL-S1
EN 3   0
EN 4   0
EN 5   -3292
EN 6   -3887
EN 7   94

EN 8   -2056
EN 9   -2177
U34-MY:ASN-CA
EN 3   0
EN 4   0
EN 5   0
EN 6   0
EN 7   0
EN 8   0
EN 9   -13840
U-Y:TYR-S1
EN 3   0
EN 4   0
EN 5   -5070
EN 6   0
EN 7   -3692
EN 8   -2951
EN 9   -1786
C-Y:HIS-S2
EN 3   0
EN 4   -6882
EN 5   0
EN 6   -4579
EN 7   -5788
EN 8   -5250
EN 9   -3450
IU-MY:ALA-S1
EN 3   0
EN 4   0
EN 5   -12342
EN 6   0
EN 7   0
EN 8   -9840
EN 9   0
C31-MY:ALA-CA
EN 3   0
EN 4   0
EN 5   0
EN 6   0
EN 7   0
EN 8   0
EN 9   -12016
5BU-P:ARG-S1
EN 3   0
EN 4   0
EN 5   0
EN 6   0
EN 7   -12958
EN 8   0
EN 9   0
QUO-RIB:LYS-S1
EN 3   0
EN 4   0
EN 5   0
EN 6   0
EN 7   0
EN 8   -13479
EN 9   0

QUO-M5:ASN-S2
EN 3 0
EN 4 0
EN 5 -18410
EN 6 0
EN 7 0
EN 8 0
EN 9 0
U-Y:GLU-S2
EN 3 0
EN 4 0
EN 5 -2140
EN 6 -1532
EN 7 242
EN 8 -733
EN 9 -2164
A-RIB:LEU-CA
EN 3 0
EN 4 0
EN 5 0
EN 6 -1532
EN 7 -2386
EN 8 -1617
EN 9 -1937
IU-P:LEU-S1
EN 3 0
EN 4 0
EN 5 0
EN 6 0
EN 7 0
EN 8 -10190
EN 9 0
C-Y:LEU-S2
EN 3 0
EN 4 0
EN 5 -1695
EN 6 338
EN 7 -1030
EN 8 -198
EN 9 -1369
G-R6:PHE-S1
EN 3 0
EN 4 0
EN 5 54
EN 6 -342
EN 7 -70
EN 8 125
EN 9 -1337
H2U-MY:GLU-S1
EN 3 0
EN 4 0
EN 5 -12635
EN 6 0
EN 7 0
EN 8 0
EN 9 0
A-P:MET-S2
EN 3 0

EN 4 0
EN 5 -1547
EN 6 -4214
EN 7 -3605
EN 8 -2481
EN 9 -2703
U34-P:GLU-S2
EN 3 0
EN 4 0
EN 5 0
EN 6 -15334
EN 7 0
EN 8 0
EN 9 0
U34-P:PRO-S1
EN 3 0
EN 4 0
EN 5 0
EN 6 0
EN 7 0
EN 8 -14503
EN 9 0
A-R5:ALA-S1
EN 3 0
EN 4 -6705
EN 5 0
EN 6 37
EN 7 -2718
EN 8 -1411
EN 9 -2520
IU-RIB:ALA-CA
EN 3 0
EN 4 0
EN 5 0
EN 6 0
EN 7 -9959
EN 8 -9840
EN 9 0
C-Y:ASP-CA
EN 3 0
EN 4 0
EN 5 0
EN 6 -2977
EN 7 -1585
EN 8 -2509
EN 9 -3543
FMU-MY:ALA-S1
EN 3 0
EN 4 0
EN 5 0
EN 6 0
EN 7 -14183
EN 8 0
EN 9 0
FHU-MY:CYS-S1
EN 3 0
EN 4 0
EN 5 0

EN 6   0
EN 7   0
EN 8   0
EN 9   -15298
C31-RIB:ASP-CA
EN 3   0
EN 4   0
EN 5   0
EN 6   0
EN 7   -14924
EN 8   0
EN 9   0
C-Y:GLN-S1
EN 3   0
EN 4   -5893
EN 5   0
EN 6   849
EN 7   -4030
EN 8   -3504
EN 9   -4265
H2U-P:TRP-S2
EN 3   0
EN 4   0
EN 5   0
EN 6   -15762
EN 7   0
EN 8   0
EN 9   0
A-R6:LYS-S1
EN 3   0
EN 4   -7124
EN 5   -2415
EN 6   -2481
EN 7   -2755
EN 8   -3018
EN 9   -3863
U31-MY:MET-S1
EN 3   0
EN 4   0
EN 5   -19538
EN 6   0
EN 7   0
EN 8   0
EN 9   0
FHU-MY:PRO-S1
EN 3   0
EN 4   0
EN 5   0
EN 6   0
EN 7   0
EN 8   -12786
EN 9   0
IU-MY:ARG-CA
EN 3   0
EN 4   0
EN 5   0
EN 6   0
EN 7   0

EN 8   -10333
EN 9   -8285
A-P:TYR-CA
EN 3   0
EN 4   0
EN 5   -4306
EN 6   449
EN 7   -2928
EN 8   -2186
EN 9   -4297
IU-P:ARG-S1
EN 3   0
EN 4   0
EN 5   0
EN 6   0
EN 7   0
EN 8   -12548
EN 9   0
5BU-RIB:PRO-CA
EN 3   0
EN 4   0
EN 5   0
EN 6   0
EN 7   -13909
EN 8   0
EN 9   0
U31-MY:TYR-S1
EN 3   0
EN 4   0
EN 5   0
EN 6   0
EN 7   0
EN 8   -15367
EN 9   0
U-RIB:ARG-CA
EN 3   0
EN 4   0
EN 5   -2543
EN 6   -3944
EN 7   -3794
EN 8   -3974
EN 9   -4811
DA-M6:ASN-S2
EN 3   0
EN 4   0
EN 5   0
EN 6   -17089
EN 7   0
EN 8   0
EN 9   0
QUO-M6:ASP-S1
EN 3   0
EN 4   0
EN 5   0
EN 6   0
EN 7   -14926
EN 8   0
EN 9   0

C31-MY:PHE-S2
EN 3 0
EN 4 0
EN 5 -18513
EN 6 0
EN 7 0
EN 8 0
EN 9 0
I-RIB:ALA-CA
EN 3 0
EN 4 0
EN 5 0
EN 6 0
EN 7 0
EN 8 0
EN 9 -12016
GTP-RIB:ARG-S2
EN 3 0
EN 4 0
EN 5 0
EN 6 0
EN 7 0
EN 8 0
EN 9 -12521
A-R6:PHE-CA
EN 3 0
EN 4 0
EN 5 -505
EN 6 -902
EN 7 1876
EN 8 -2152
EN 9 -1400
U-P:ASN-S2
EN 3 0
EN 4 -6599
EN 5 -7119
EN 6 -6014
EN 7 -4952
EN 8 -4382
EN 9 -5292
G-R5:TRP-S1
EN 3 0
EN 4 -7927
EN 5 0
EN 6 -3907
EN 7 -2846
EN 8 -2435
EN 9 -3898
FHU-P:TYR-S1
EN 3 0
EN 4 0
EN 5 0
EN 6 0
EN 7 0
EN 8 -13649
EN 9 0
QUO-M5:LEU-S2
EN 3 0

EN 4   0
EN 5   -16421
EN 6   0
EN 7   0
EN 8   0
EN 9   -11871
H2U-MY:GLN-S1
EN 3   0
EN 4   0
EN 5   0
EN 6   0
EN 7   -12262
EN 8   0
EN 9   0
A-R5:LEU-S1
EN 3   0
EN 4   0
EN 5   0
EN 6   -2537
EN 7   -471
EN 8   -1777
EN 9   -1738
QUO-RIB:LEU-CA
EN 3   0
EN 4   0
EN 5   0
EN 6   0
EN 7   0
EN 8   0
EN 9   -13586
A-R5:MET-S2
EN 3   0
EN 4   0
EN 5   -6366
EN 6   -224
EN 7   -3984
EN 8   -3483
EN 9   -2700
C31-P:GLN-S2
EN 3   0
EN 4   0
EN 5   0
EN 6   0
EN 7   0
EN 8   0
EN 9   -14114
M2G-P:GLU-S1
EN 3   0
EN 4   0
EN 5   0
EN 6   0
EN 7   0
EN 8   -13116
EN 9   0
FHU-P:ASP-CA
EN 3   0
EN 4   0
EN 5   0

EN 6 0
EN 7 0
EN 8 -13801
EN 9 -11040
OMC-P:LYS-S2
EN 3 0
EN 4 0
EN 5 -13597
EN 6 0
EN 7 0
EN 8 0
EN 9 -9048
IU-RIB:ARG-CA
EN 3 0
EN 4 0
EN 5 0
EN 6 -11513
EN 7 0
EN 8 -10333
EN 9 0
U-RIB:PHE-S2
EN 3 0
EN 4 0
EN 5 -1276
EN 6 -6311
EN 7 -4835
EN 8 -990
EN 9 -3266
G-R6:GLY-CA
EN 3 0
EN 4 0
EN 5 -4094
EN 6 -4088
EN 7 -3776
EN 8 -4085
EN 9 -3700
QUO-M5:ARG-S1
EN 3 0
EN 4 0
EN 5 0
EN 6 0
EN 7 0
EN 8 0
EN 9 -12509
U31-RIB:PHE-S1
EN 3 0
EN 4 0
EN 5 0
EN 6 0
EN 7 0
EN 8 -15004
EN 9 0
FHU-RIB:LEU-CA
EN 3 0
EN 4 0
EN 5 0
EN 6 -15097
EN 7 0

EN 8 0
EN 9 0
GTP-M5:ASP-S2
EN 3 0
EN 4 0
EN 5 0
EN 6 0
EN 7 0
EN 8 0
EN 9 -12804
U34-MY:PRO-CA
EN 3 0
EN 4 0
EN 5 0
EN 6 0
EN 7 0
EN 8 0
EN 9 -13459
DA-RIB:GLN-S1
EN 3 0
EN 4 0
EN 5 0
EN 6 0
EN 7 0
EN 8 0
EN 9 -15800
IU-MY:VAL-CA
EN 3 0
EN 4 0
EN 5 0
EN 6 0
EN 7 0
EN 8 0
EN 9 -9799
G-P:THR-CA
EN 3 0
EN 4 -4694
EN 5 -4425
EN 6 -4108
EN 7 -4313
EN 8 -3425
EN 9 -3150
C31-RIB:GLU-CA
EN 3 0
EN 4 0
EN 5 0
EN 6 -15295
EN 7 0
EN 8 0
EN 9 0
GTP-RIB:SER-CA
EN 3 0
EN 4 0
EN 5 0
EN 6 0
EN 7 0
EN 8 0
EN 9 -14738

GTP-M5:ALA-CA
EN 3 0
EN 4 0
EN 5 0
EN 6 0
EN 7 0
EN 8 0
EN 9 -12016
H2U-MY:PHE-S2
EN 3 0
EN 4 -19947
EN 5 0
EN 6 0
EN 7 0
EN 8 0
EN 9 0
DA-RIB:SER-S1
EN 3 0
EN 4 0
EN 5 0
EN 6 0
EN 7 0
EN 8 0
EN 9 -13021
QUO-M5:PHE-S1
EN 3 0
EN 4 0
EN 5 0
EN 6 0
EN 7 0
EN 8 -15004
EN 9 -13960
DA-RIB:ALA-CA
EN 3 0
EN 4 0
EN 5 0
EN 6 0
EN 7 -14183
EN 8 0
EN 9 0
FHU-P:THR-S1
EN 3 0
EN 4 0
EN 5 0
EN 6 0
EN 7 -13710
EN 8 -14304
EN 9 0
FMU-MY:ASP-S1
EN 3 0
EN 4 0
EN 5 0
EN 6 0
EN 7 -14926
EN 8 0
EN 9 0
U-P:VAL-CA
EN 3 0

EN 4   0
EN 5   0
EN 6   -1023
EN 7   -2061
EN 8   -3775
EN 9   -3048
FMU-RIB:VAL-S1
EN 3   0
EN 4   0
EN 5   0
EN 6   0
EN 7   -14474
EN 8   0
EN 9   0
5BU-RIB:SER-S1
EN 3   0
EN 4   0
EN 5   0
EN 6   0
EN 7   0
EN 8   0
EN 9   -11303
C31-RIB:LEU-CA
EN 3   0
EN 4   0
EN 5   0
EN 6   -15097
EN 7   0
EN 8   0
EN 9   0
DA-RIB:HIS-S2
EN 3   0
EN 4   0
EN 5   0
EN 6   -18301
EN 7   0
EN 8   0
EN 9   0
U34-P:ASN-CA
EN 3   0
EN 4   0
EN 5   0
EN 6   -17068
EN 7   0
EN 8   0
EN 9   -16562
G-RIB:PRO-CA
EN 3   0
EN 4   -4866
EN 5   554
EN 6   -5285
EN 7   -4693
EN 8   -4173
EN 9   -3842
G-RIB:CYS-S1
EN 3   0
EN 4   0
EN 5   0

EN 6 0
EN 7 -2336
EN 8 0
EN 9 -1886
H2U-P:ARG-S1
EN 3 0
EN 4 0
EN 5 0
EN 6 0
EN 7 0
EN 8 -9565
EN 9 0
DA-M6:HIS-CA
EN 3 0
EN 4 0
EN 5 0
EN 6 -18284
EN 7 0
EN 8 0
EN 9 0
G-RIB:ASP-CA
EN 3 0
EN 4 0
EN 5 -460
EN 6 -2243
EN 7 -2899
EN 8 -2398
EN 9 -3772
U-Y:PHE-S1
EN 3 0
EN 4 0
EN 5 -2990
EN 6 -2382
EN 7 -3712
EN 8 -2206
EN 9 -2428
FHU-MY:ARG-S2
EN 3 0
EN 4 0
EN 5 0
EN 6 -15749
EN 7 0
EN 8 -13564
EN 9 0
C-Y:TYR-S2
EN 3 0
EN 4 -6137
EN 5 -2433
EN 6 -1112
EN 7 -4038
EN 8 -2362
EN 9 -2323
U31-MY:ALA-CA
EN 3 0
EN 4 0
EN 5 0
EN 6 0
EN 7 0

EN 8   -13060
EN 9   0
H2U-P:PHE-S2
EN 3   0
EN 4   0
EN 5   0
EN 6   -13205
EN 7   0
EN 8   0
EN 9   0
G-R6:THR-S1
EN 3   0
EN 4   0
EN 5   0
EN 6   -2364
EN 7   -3218
EN 8   -2759
EN 9   -2465
U31-MY:ASP-S1
EN 3   0
EN 4   0
EN 5   0
EN 6   -17705
EN 7   0
EN 8   -13803
EN 9   -12759
C-RIB:SER-CA
EN 3   0
EN 4   0
EN 5   -4853
EN 6   -5511
EN 7   -5639
EN 8   -5043
EN 9   -4000
FHU-P:ASP-S2
EN 3   0
EN 4   0
EN 5   0
EN 6   0
EN 7   0
EN 8   0
EN 9   -11087
H2U-MY:LEU-S1
EN 3   0
EN 4   0
EN 5   0
EN 6   0
EN 7   0
EN 8   -8924
EN 9   -7880
FMU-RIB:ALA-CA
EN 3   0
EN 4   0
EN 5   0
EN 6   0
EN 7   0
EN 8   -13060
EN 9   0

H2U-P:ASN-CA
EN 3 0
EN 4 0
EN 5 0
EN 6 0
EN 7 0
EN 8 -12613
EN 9 0
U34-MY:SER-CA
EN 3 0
EN 4 0
EN 5 0
EN 6 -17967
EN 7 0
EN 8 0
EN 9 -13021
C-Y:LEU-CA
EN 3 0
EN 4 0
EN 5 25
EN 6 1346
EN 7 690
EN 8 -1106
EN 9 -1780
G-RIB:SER-S1
EN 3 0
EN 4 -4427
EN 5 -5716
EN 6 -3790
EN 7 -4790
EN 8 -4087
EN 9 -4430
FHU-RIB:LYS-CA
EN 3 0
EN 4 0
EN 5 0
EN 6 0
EN 7 0
EN 8 0
EN 9 -10712
U31-RIB:ASN-S1
EN 3 0
EN 4 0
EN 5 0
EN 6 0
EN 7 -16007
EN 8 0
EN 9 0
C-RIB:ALA-CA
EN 3 0
EN 4 0
EN 5 -4110
EN 6 -4103
EN 7 -4609
EN 8 -2557
EN 9 -3645
DA-RIB:GLN-S2
EN 3 0

EN 4 0
EN 5 0
EN 6 0
EN 7 0
EN 8 -15157
EN 9 0
C31-P:GLU-CA
EN 3 0
EN 4 0
EN 5 0
EN 6 -15295
EN 7 0
EN 8 0
EN 9 0
U31-P:HIS-S1
EN 3 0
EN 4 0
EN 5 0
EN 6 0
EN 7 0
EN 8 -16099
EN 9 0
DA-RIB:VAL-CA
EN 3 0
EN 4 0
EN 5 0
EN 6 0
EN 7 0
EN 8 -13349
EN 9 0
C31-P:ASN-CA
EN 3 0
EN 4 0
EN 5 0
EN 6 0
EN 7 0
EN 8 0
EN 9 -13840
H2U-RIB:PHE-CA
EN 3 0
EN 4 0
EN 5 0
EN 6 0
EN 7 0
EN 8 0
EN 9 -9973
QUO-P:SER-S1
EN 3 0
EN 4 0
EN 5 0
EN 6 0
EN 7 0
EN 8 0
EN 9 -13021
H2U-P:THR-CA
EN 3 0
EN 4 0
EN 5 0

EN 6 0
EN 7 0
EN 8 0
EN 9 -10990
DA-M5:ASN-CA
EN 3 0
EN 4 0
EN 5 0
EN 6 0
EN 7 0
EN 8 0
EN 9 -13840
FHU-P:VAL-S1
EN 3 0
EN 4 0
EN 5 0
EN 6 0
EN 7 0
EN 8 -13351
EN 9 0
U-RIB:ASP-S2
EN 3 0
EN 4 0
EN 5 -4556
EN 6 -2784
EN 7 -2556
EN 8 -3150
EN 9 -3111
DA-RIB:VAL-S1
EN 3 0
EN 4 0
EN 5 0
EN 6 0
EN 7 0
EN 8 -13351
EN 9 0
C-P:PRO-CA
EN 3 0
EN 4 0
EN 5 -5563
EN 6 -5076
EN 7 -3632
EN 8 -4860
EN 9 -4255
H2U-MY:ASN-S2
EN 3 0
EN 4 0
EN 5 -14422
EN 6 0
EN 7 -12040
EN 8 0
EN 9 -11590
QUO-M6:PHE-CA
EN 3 0
EN 4 0
EN 5 0
EN 6 0
EN 7 0

EN 8   -15004
EN 9   -13960
DA-M6:THR-CA
EN 3   0
EN 4   0
EN 5   0
EN 6   0
EN 7   0
EN 8   0
EN 9   -14978
A-R6:GLN-CA
EN 3   0
EN 4   0
EN 5   0
EN 6   -3747
EN 7   -3067
EN 8   -1562
EN 9   -3374
U31-MY:TYR-CA
EN 3   0
EN 4   0
EN 5   0
EN 6   0
EN 7   0
EN 8   -15367
EN 9   0
G-R5:GLU-S2
EN 3   0
EN 4   -3512
EN 5   -1525
EN 6   -630
EN 7   -2971
EN 8   -1746
EN 9   -1947
C-RIB:CYS-S1
EN 3   0
EN 4   0
EN 5   0
EN 6   0
EN 7   -4457
EN 8   -2620
EN 9   -2290
IU-P:ALA-CA
EN 3   0
EN 4   0
EN 5   0
EN 6   0
EN 7   0
EN 8   0
EN 9   -9294
QUO-M5:GLU-S2
EN 3   0
EN 4   0
EN 5   0
EN 6   0
EN 7   -14272
EN 8   0
EN 9   0

A-R5:VAL-CA
EN 3 0
EN 4 0
EN 5 1148
EN 6 -964
EN 7 -2409
EN 8 -166
EN 9 -1597
FMU-RIB:MET-S2
EN 3 0
EN 4 0
EN 5 0
EN 6 0
EN 7 0
EN 8 0
EN 9 -15000
DA-M5:THR-CA
EN 3 0
EN 4 0
EN 5 0
EN 6 0
EN 7 0
EN 8 0
EN 9 -13260
FMU-MY:GLN-S1
EN 3 0
EN 4 0
EN 5 0
EN 6 0
EN 7 -16250
EN 8 0
EN 9 0
IU-RIB:LEU-S2
EN 3 0
EN 4 0
EN 5 0
EN 6 -10876
EN 7 0
EN 8 0
EN 9 0
G-P:CYS-S1
EN 3 0
EN 4 0
EN 5 0
EN 6 -1707
EN 7 -646
EN 8 0
EN 9 -1201
H2U-MY:LYS-S2
EN 3 0
EN 4 0
EN 5 0
EN 6 0
EN 7 -10662
EN 8 0
EN 9 0
GTP-M5:THR-S1
EN 3 0

EN 4 0
EN 5 0
EN 6 0
EN 7 0
EN 8 0
EN 9 -13260
U31-P:MET-CA
EN 3 0
EN 4 0
EN 5 0
EN 6 -18217
EN 7 0
EN 8 -16032
EN 9 0
IU-P:HIS-S1
EN 3 0
EN 4 0
EN 5 -16883
EN 6 0
EN 7 0
EN 8 0
EN 9 0
G-RIB:GLU-S1
EN 3 0
EN 4 0
EN 5 -1493
EN 6 -1559
EN 7 -2545
EN 8 -437
EN 9 -2570
C-P:ARG-CA
EN 3 0
EN 4 0
EN 5 -5446
EN 6 -5561
EN 7 -6498
EN 8 -5963
EN 9 -5768
5BU-MY:ILE-S1
EN 3 0
EN 4 0
EN 5 0
EN 6 0
EN 7 -13453
EN 8 0
EN 9 0
H2U-P:LEU-S1
EN 3 0
EN 4 0
EN 5 0
EN 6 0
EN 7 0
EN 8 0
EN 9 -7880
U-Y:MET-S1
EN 3 0
EN 4 0
EN 5 -2301

EN 6   -2697
EN 7   -3353
EN 8   -512
EN 9   -1186
A-R5:PRO-CA
EN 3   0
EN 4   0
EN 5   -5
EN 6   -2119
EN 7   -3978
EN 8   -3209
EN 9   -3224
U-RIB:TRP-S2
EN 3   0
EN 4   0
EN 5   -3833
EN 6   -5947
EN 7   -4173
EN 8   -4767
EN 9   -2719
G-RIB:ALA-CA
EN 3   0
EN 4   0
EN 5   280
EN 6   -3219
EN 7   -2639
EN 8   -2264
EN 9   -3181
FHU-MY:ILE-S1
EN 3   0
EN 4   0
EN 5   0
EN 6   -16232
EN 7   0
EN 8   0
EN 9   0
FHU-MY:ASP-CA
EN 3   0
EN 4   0
EN 5   0
EN 6   0
EN 7   -14924
EN 8   0
EN 9   0
A-RIB:CYS-CA
EN 3   0
EN 4   0
EN 5   0
EN 6   -2240
EN 7   -4613
EN 8   -3490
EN 9   -1734
U34-P:HIS-S1
EN 3   0
EN 4   0
EN 5   0
EN 6   0
EN 7   0

EN 8   -16099
EN 9   0
G-R6:CYS-CA
EN 3   0
EN 4   0
EN 5   0
EN 6   0
EN 7   -3341
EN 8   504
EN 9   -169
FHU-RIB:LYS-S1
EN 3   0
EN 4   0
EN 5   0
EN 6   0
EN 7   0
EN 8   0
EN 9   -12435
GTP-M6:SER-CA
EN 3   0
EN 4   0
EN 5   0
EN 6   0
EN 7   0
EN 8   0
EN 9   -13021
C31-P:THR-S1
EN 3   0
EN 4   0
EN 5   0
EN 6   0
EN 7   0
EN 8   -16021
EN 9   0
H2U-P:PRO-CA
EN 3   0
EN 4   0
EN 5   0
EN 6   0
EN 7   0
EN 8   0
EN 9   -9472
C-P:ASN-S2
EN 3   0
EN 4   0
EN 5   -7420
EN 6   -6361
EN 7   -5420
EN 8   -5009
EN 9   -5086
FMU-P:ASP-S1
EN 3   0
EN 4   0
EN 5   0
EN 6   0
EN 7   0
EN 8   -13803
EN 9   0

A-RIB:PHE-S2
EN 3   0
EN 4   0
EN 5   -4949
EN 6   -4340
EN 7   -3571
EN 8   -1825
EN 9   -2315
QUO-M6:ASN-S2
EN 3   0
EN 4   0
EN 5   -18410
EN 6   0
EN 7   0
EN 8   0
EN 9   0
H2U-P:LEU-S2
EN 3   0
EN 4   0
EN 5   0
EN 6   0
EN 7   -10051
EN 8   0
EN 9   0
QUO-M6:ARG-CA
EN 3   0
EN 4   0
EN 5   0
EN 6   0
EN 7   -14676
EN 8   0
EN 9   0
G-R6:PHE-S2
EN 3   0
EN 4   -7088
EN 5   0
EN 6   -2489
EN 7   2432
EN 8   121
EN 9   -1894
A-P:THR-CA
EN 20   0
EN 40   -1478
EN 60   -2559
EN 80   -3944
EN 100   -5638
EN 120   -5143
EN 140   -5867
EN 160   -4393
EN 180   -2721
EN 200   0
EN 220   -1333
EN 240   -5301
EN 260   -3926
EN 280   -4081
EN 300   -4291
EN 320   -3974
EN 340   -311

EN 360 -7042
U-Y:ASN-S2
EN 20 -5970
EN 40 -4560
EN 60 -5189
EN 80 -5640
EN 100 -5446
EN 120 -5719
EN 140 -3797
EN 160 -3488
EN 180 0
EN 200 -7942
EN 220 -5420
EN 240 -6666
EN 260 -5622
EN 280 -4591
EN 300 -4943
EN 320 -4334
EN 340 -4399
EN 360 -3968
C31-RIB:LEU-CA
EN 20 0
EN 40 0
EN 60 0
EN 80 0
EN 100 0
EN 120 0
EN 140 0
EN 160 0
EN 180 0
EN 200 0
EN 220 0
EN 240 0
EN 260 0
EN 280 0
EN 300 -15748
EN 320 0
EN 340 0
EN 360 0
C-Y:ASP-S1
EN 20 -6666
EN 40 -5257
EN 60 -3616
EN 80 -3615
EN 100 -3421
EN 120 -259
EN 140 -2484
EN 160 -3180
EN 180 0
EN 200 -5917
EN 220 -4660
EN 240 -3636
EN 260 -3597
EN 280 -990
EN 300 -196
EN 320 -4026
EN 340 -2373
EN 360 -1943

C-RIB:CYS-S1
EN 20   0
EN 40   0
EN 60   -3433
EN 80   0
EN 100   -4242
EN 120   0
EN 140   -3306
EN 160   0
EN 180   0
EN 200   0
EN 220   0
EN 240   0
EN 260   -4419
EN 280   -5960
EN 300   0
EN 320   -3130
EN 340   0
EN 360   0
U-Y:HIS-S2
EN 20   -8899
EN 40   0
EN 60   -6401
EN 80   -5135
EN 100   -5945
EN 120   -5766
EN 140   -5009
EN 160   0
EN 180   0
EN 200   0
EN 220   -5627
EN 240   -6873
EN 260   -4404
EN 280   -3223
EN 300   -1715
EN 320   -2111
EN 340   0
EN 360   -6897
G-RIB:LYS-S1
EN 20   -3217
EN 40   -4530
EN 60   -5611
EN 80   -5610
EN 100   -5415
EN 120   -4684
EN 140   -4677
EN 160   -3005
EN 180   -1334
EN 200   -6907
EN 220   -4385
EN 240   -6445
EN 260   -5376
EN 280   -3698
EN 300   -4912
EN 320   -4088
EN 340   -3363
EN 360   -4650
G-R5:GLU-S1

EN 20   0
EN 40   -4168
EN 60   -2075
EN 80   -808
EN 100   1103
EN 120   0
EN 140   -1235
EN 160   -373
EN 180   -971
EN 200   -3110
EN 220   -3018
EN 240   -1542
EN 260   926
EN 280   -454
EN 300   -1249
EN 320   -1772
EN 340   -1284
EN 360   -853
OMC-RIB:LYS-S2
EN 20   0
EN 40   0
EN 60   0
EN 80   0
EN 100   0
EN 120   0
EN 140   0
EN 160   0
EN 180   0
EN 200   0
EN 220   0
EN 240   -13264
EN 260   0
EN 280   -12336
EN 300   0
EN 320   0
EN 340   0
EN 360   0
G-RIB:ALA-S1
EN 20   -6233
EN 40   328
EN 60   -2018
EN 80   -3257
EN 100   -2827
EN 120   -3552
EN 140   -3608
EN 160   -3038
EN 180   -4349
EN 200   -4770
EN 220   -4347
EN 240   -2872
EN 260   -2120
EN 280   -1561
EN 300   -3037
EN 320   -1162
EN 340   -3326
EN 360   -796
U-RIB:ARG-S1
EN 20   -6335

EN 40   -4926
EN 60   -6007
EN 80   -5846
EN 100   -4883
EN 120   -5570
EN 140   -4875
EN 160   -6575
EN 180   -6722
EN 200   0
EN 220   -5334
EN 240   -5811
EN 260   -5657
EN 280   -4094
EN 300   -5308
EN 320   -5252
EN 340   -5146
EN 360   -2616
C-Y:ASN-S2
EN 20   -8481
EN 40   -6359
EN 60   -5099
EN 80   -4386
EN 100   -4191
EN 120   -3078
EN 140   -1868
EN 160   -4281
EN 180   0
EN 200   -7018
EN 220   -6213
EN 240   -4738
EN 260   -3986
EN 280   -4070
EN 300   -4401
EN 320   -1693
EN 340   -3475
EN 360   0
C-RIB:GLU-S1
EN 20   -3258
EN 40   -131
EN 60   -3311
EN 80   -2929
EN 100   2417
EN 120   -1843
EN 140   -3185
EN 160   -3046
EN 180   -4097
EN 200   -3513
EN 220   0
EN 240   -1232
EN 260   -2580
EN 280   -2734
EN 300   -2613
EN 320   -1622
EN 340   -3404
EN 360   -4691
IU-MY:TYR-CA
EN 20   0
EN 40   0

EN 60 0
EN 80 0
EN 100 0
EN 120 -14042
EN 140 0
EN 160 0
EN 180 0
EN 200 0
EN 220 0
EN 240 0
EN 260 0
EN 280 0
EN 300 0
EN 320 0
EN 340 0
EN 360 0
G-RIB:ASP-S1
EN 20 -5259
EN 40 -414
EN 60 -3213
EN 80 -1494
EN 100 -3309
EN 120 -3844
EN 140 -2634
EN 160 -3781
EN 180 -5645
EN 200 -3796
EN 220 -269
EN 240 -1516
EN 260 -3194
EN 280 -3017
EN 300 -3227
EN 320 -3915
EN 340 -4069
EN 360 -5979
G-R6:VAL-S1
EN 20 0
EN 40 37
EN 60 0
EN 80 2392
EN 100 -1401
EN 120 595
EN 140 0
EN 160 0
EN 180 -3928
EN 200 0
EN 220 182
EN 240 -2329
EN 260 -1923
EN 280 1199
EN 300 -1611
EN 320 -1453
EN 340 1203
EN 360 -2805
U-RIB:GLU-S2
EN 20 0
EN 40 0
EN 60 -1164

EN 80   -450
EN 100   -1260
EN 120   -2246
EN 140   -2042
EN 160   -1732
EN 180   -4048
EN 200   0
EN 220   -942
EN 240   -2901
EN 260   -432
EN 280   -4243
EN 300   -2183
EN 320   -861
EN 340   -3909
EN 360   -3930
A-P:LEU-CA
EN 20   0
EN 40   -86
EN 60   -162
EN 80   -1166
EN 100   -259
EN 120   -1798
EN 140   -35
EN 160   -2448
EN 180   0
EN 200   0
EN 220   -1658
EN 240   -182
EN 260   -1148
EN 280   -971
EN 300   -3190
EN 320   139
EN 340   -3359
EN 360   -1211
G-R5:CYS-S1
EN 20   0
EN 40   0
EN 60   0
EN 80   0
EN 100   0
EN 120   -2395
EN 140   0
EN 160   -3598
EN 180   0
EN 200   0
EN 220   -4526
EN 240   0
EN 260   0
EN 280   0
EN 300   0
EN 320   0
EN 340   0
EN 360   0
A-P:ASN-S2
EN 20   -5205
EN 40   -2078
EN 60   -3159
EN 80   -5665

EN 100 -5471
EN 120 -5744
EN 140 -4298
EN 160 -5827
EN 180 -3322
EN 200 0
EN 220 0
EN 240 -5902
EN 260 -4145
EN 280 -4350
EN 300 -4178
EN 320 -4122
EN 340 -5351
EN 360 0
A-RIB:ILE-S1
EN 20 0
EN 40 0
EN 60 422
EN 80 -2851
EN 100 -2104
EN 120 -2930
EN 140 -2885
EN 160 -1863
EN 180 -4179
EN 200 0
EN 220 -2791
EN 240 0
EN 260 -1567
EN 280 -386
EN 300 1120
EN 320 -2709
EN 340 -52
EN 360 -2343
G-RIB:HIS-CA
EN 20 0
EN 40 -2711
EN 60 -1069
EN 80 -3790
EN 100 -3596
EN 120 -434
EN 140 -6648
EN 160 0
EN 180 -6676
EN 200 0
EN 220 -4283
EN 240 -1090
EN 260 -6495
EN 280 -5313
EN 300 -2088
EN 320 -4202
EN 340 -5532
EN 360 -5553
A-P:VAL-CA
EN 20 -3650
EN 40 0
EN 60 -2317
EN 80 -2608
EN 100 -2795

EN 120   -3691
EN 140   -1477
EN 160   -2885
EN 180   -1766
EN 200   0
EN 220   0
EN 240   -620
EN 260   -872
EN 280   -3915
EN 300   -2623
EN 320   -2567
EN 340   -2791
EN 360   -4370
C31-P:THR-CA
EN 20   0
EN 40   0
EN 60   -17838
EN 80   0
EN 100   -16930
EN 120   0
EN 140   0
EN 160   0
EN 180   0
EN 200   0
EN 220   0
EN 240   0
EN 260   0
EN 280   0
EN 300   0
EN 320   0
EN 340   0
EN 360   0
A-RIB:ASN-S1
EN 20   0
EN 40   -2055
EN 60   -2131
EN 80   -5144
EN 100   -4950
EN 120   -3767
EN 140   -5439
EN 160   -4970
EN 180   -3298
EN 200   0
EN 220   -5898
EN 240   -5586
EN 260   -3117
EN 280   -4658
EN 300   -3703
EN 320   -5816
EN 340   -5328
EN 360   -6615
C-P:TRP-S2
EN 20   0
EN 40   0
EN 60   -4665
EN 80   -3951
EN 100   -5475
EN 120   -5035

EN 140   -4538
EN 160   -5234
EN 180   0
EN 200   0
EN 220   0
EN 240   -4685
EN 260   -6204
EN 280   0
EN 300   -5684
EN 320   0
EN 340   -3422
EN 360   0
C-Y:LYS-CA
EN 20   -3614
EN 40   -3922
EN 60   -3999
EN 80   -3577
EN 100   -3880
EN 120   -2651
EN 140   -2154
EN 160   -3403
EN 180   -3448
EN 200   -3869
EN 220   -3064
EN 240   -3688
EN 260   -2936
EN 280   2061
EN 300   -2136
EN 320   -261
EN 340   -1039
EN 360   -3330
A-R6:ILE-S1
EN 20   -4345
EN 40   -2936
EN 60   -1295
EN 80   -581
EN 100   0
EN 120   1057
EN 140   -2172
EN 160   0
EN 180   -2462
EN 200   0
EN 220   0
EN 240   402
EN 260   -1567
EN 280   1330
EN 300   -2867
EN 320   -2709
EN 340   -3487
EN 360   0
U-RIB:ARG-S2
EN 20   -9069
EN 40   -6947
EN 60   -6882
EN 80   -7404
EN 100   -5309
EN 120   -5766
EN 140   -6090

EN 160   -5583
EN 180   -7186
EN 200   -6602
EN 220   -7515
EN 240   -7426
EN 260   -6000
EN 280   -5974
EN 300   -4608
EN 320   -5265
EN 340   -4776
EN 360   -7068
C-RIB:TRP-S1
EN 20   0
EN 40   0
EN 60   -2938
EN 80   -3942
EN 100   -6018
EN 120   -5025
EN 140   0
EN 160   0
EN 180   -5823
EN 200   0
EN 220   0
EN 240   0
EN 260   -3924
EN 280   -4752
EN 300   0
EN 320   0
EN 340   -6135
EN 360   -5704
G-RIB:ASN-CA
EN 20   -4622
EN 40   -5483
EN 60   -5006
EN 80   -3962
EN 100   -4098
EN 120   -4040
EN 140   -3715
EN 160   -6127
EN 180   -4456
EN 200   0
EN 220   -4072
EN 240   -5318
EN 260   -3110
EN 280   -4390
EN 300   -4600
EN 320   -3991
EN 340   -5481
EN 360   -4337
G-P:LEU-S1
EN 20   0
EN 40   449
EN 60   -1897
EN 80   -2017
EN 100   -2153
EN 120   -2096
EN 140   -1770
EN 160   -195

EN 180   -3516
EN 200   -2932
EN 220   594
EN 240   -3374
EN 260   -1165
EN 280   -1822
EN 300   -2032
EN 320   -2428
EN 340   -101
EN 360   -675
C-RIB:ASP-S1
EN 20   0
EN 40   0
EN 60   -3616
EN 80   -2450
EN 100   -4624
EN 120   -4247
EN 140   -4494
EN 160   -2467
EN 180   -6049
EN 200   0
EN 220   -672
EN 240   -1919
EN 260   -4984
EN 280   -3712
EN 300   -1200
EN 320   -5413
EN 340   -4803
EN 360   -6382
G-RIB:LEU-S1
EN 20   0
EN 40   476
EN 60   -1870
EN 80   -1608
EN 100   -2418
EN 120   -2069
EN 140   -477
EN 160   -2438
EN 180   -767
EN 200   0
EN 220   -3366
EN 240   379
EN 260   -1138
EN 280   -2679
EN 300   -1623
EN 320   -1567
EN 340   -74
EN 360   -2366
G-P:LYS-S1
EN 20   0
EN 40   0
EN 60   -3920
EN 80   -5738
EN 100   -5734
EN 120   -6098
EN 140   -6422
EN 160   -6340
EN 180   -7066

EN 200 0
EN 220 -2694
EN 240 -5035
EN 260 -5288
EN 280 -5543
EN 300 -5073
EN 320 -5942
EN 340 -6373
EN 360 -6947
FHU-RIB:ALA-S1
EN 20 0
EN 40 0
EN 60 0
EN 80 0
EN 100 -15686
EN 120 -14242
EN 140 0
EN 160 0
EN 180 0
EN 200 0
EN 220 0
EN 240 0
EN 260 0
EN 280 -13969
EN 300 -14178
EN 320 0
EN 340 0
EN 360 0
IU-MY:ARG-S2
EN 20 0
EN 40 0
EN 60 0
EN 80 0
EN 100 0
EN 120 -13245
EN 140 -15470
EN 160 -14448
EN 180 0
EN 200 0
EN 220 0
EN 240 0
EN 260 0
EN 280 0
EN 300 0
EN 320 0
EN 340 0
EN 360 0
A-RIB:HIS-S1
EN 20 0
EN 40 -4988
EN 60 -4351
EN 80 -2633
EN 100 -5543
EN 120 -4429
EN 140 -4937
EN 160 -3915
EN 180 -6231
EN 200 0

EN 220 0
EN 240 -5637
EN 260 -4885
EN 280 -5543
EN 300 -6375
EN 320 -6479
EN 340 -4826
EN 360 -4396
U-P:GLU-S2
EN 20 0
EN 40 -1092
EN 60 -2173
EN 80 -2725
EN 100 -3364
EN 120 -4521
EN 140 -2046
EN 160 -3454
EN 180 -6323
EN 200 0
EN 220 -2664
EN 240 -2193
EN 260 -437
EN 280 -2531
EN 300 -3192
EN 320 -4854
EN 340 -3361
EN 360 -4939
A-R6:GLU-S2
EN 20 0
EN 40 -4308
EN 60 -3119
EN 80 -2405
EN 100 -1206
EN 120 -2866
EN 140 -1987
EN 160 -965
EN 180 -4286
EN 200 0
EN 220 -175
EN 240 -1422
EN 260 -1935
EN 280 -3476
EN 300 -1969
EN 320 -94
EN 340 845
EN 360 -4167
C-P:PRO-CA
EN 20 0
EN 40 -5967
EN 60 -3321
EN 80 -3612
EN 100 -3800
EN 120 -5607
EN 140 -5465
EN 160 -4442
EN 180 -4488
EN 200 0
EN 220 0

EN 240   -3341
EN 260   -4307
EN 280   -5688
EN 300   -3628
EN 320   -4023
EN 340   -5513
EN 360   -6087
C-P:ARG-S1
EN 20   -3704
EN 40   -5729
EN 60   -5093
EN 80   -5383
EN 100   -6837
EN 120   -6809
EN 140   -6578
EN 160   -6374
EN 180   -8359
EN 200   0
EN 220   -4419
EN 240   -4897
EN 260   -5747
EN 280   -7347
EN 300   -7439
EN 320   -6292
EN 340   -7069
EN 360   -6141
C-RIB:PRO-S1
EN 20   -8632
EN 40   -3235
EN 60   -5321
EN 80   -4868
EN 100   -3790
EN 120   -4394
EN 140   -4189
EN 160   -3880
EN 180   -5483
EN 200   0
EN 220   -4807
EN 240   -3885
EN 260   -3966
EN 280   -4674
EN 300   -4622
EN 320   -6284
EN 340   -351
EN 360   -2643
A-R6:GLN-CA
EN 20   0
EN 40   0
EN 60   -2374
EN 80   56
EN 100   -5193
EN 120   -1739
EN 140   -3965
EN 160   -2943
EN 180   -5259
EN 200   -5680
EN 220   0
EN 240   -677

EN 260   -3360
EN 280   0
EN 300   -2681
EN 320   -4342
EN 340   -2849
EN 360   0
G-P:ASN-S2
EN 20   0
EN 40   -4978
EN 60   -5607
EN 80   -4632
EN 100   -4935
EN 120   -4972
EN 140   -4927
EN 160   -6411
EN 180   -6774
EN 200   0
EN 220   -4120
EN 240   -4743
EN 260   -5111
EN 280   -6537
EN 300   -5559
EN 320   -4421
EN 340   -5198
EN 360   -2668
A-R6:LYS-S2
EN 20   0
EN 40   -698
EN 60   -4762
EN 80   -4698
EN 100   -4504
EN 120   -3574
EN 140   -3369
EN 160   -3613
EN 180   -1941
EN 200   -4080
EN 220   -3987
EN 240   -4521
EN 260   -4680
EN 280   -4305
EN 300   -4713
EN 320   -3906
EN 340   -2966
EN 360   0
U-P:MET-S2
EN 20   0
EN 40   0
EN 60   -4063
EN 80   -5067
EN 100   -1437
EN 120   0
EN 140   -4941
EN 160   -4631
EN 180   0
EN 200   0
EN 220   -5559
EN 240   -2366
EN 260   -5048

EN 280 -4159
EN 300 -1647
EN 320 -3760
EN 340 -4538
EN 360 0
C-P:ALA-S1
EN 20 0
EN 40 -5528
EN 60 -2882
EN 80 -3886
EN 100 -4245
EN 120 -2247
EN 140 -4855
EN 160 -3833
EN 180 -4049
EN 200 0
EN 220 -4760
EN 240 -4404
EN 260 -2533
EN 280 -3476
EN 300 -4614
EN 320 -4495
EN 340 -2905
EN 360 -4644
C-P:VAL-S1
EN 20 0
EN 40 -2093
EN 60 -3555
EN 80 -3726
EN 100 -4536
EN 120 -2921
EN 140 -4312
EN 160 -3742
EN 180 -3336
EN 200 0
EN 220 -3665
EN 240 -4198
EN 260 -3707
EN 280 -2647
EN 300 -2857
EN 320 -4970
EN 340 -1931
EN 360 -1500
C-Y:GLY-CA
EN 20 -8029
EN 40 -5906
EN 60 -5983
EN 80 -3813
EN 100 -4070
EN 120 -2174
EN 140 -2682
EN 160 -4211
EN 180 -3423
EN 200 -8996
EN 220 -5761
EN 240 -5197
EN 260 -4959
EN 280 -4452

EN 300   -2944
EN 320   -3340
EN 340   0
EN 360   -1587
G-R5:ASN-S1
EN 20   -6339
EN 40   -5935
EN 60   -4585
EN 80   -4783
EN 100   -2934
EN 120   -3207
EN 140   -3715
EN 160   -3697
EN 180   -2738
EN 200   -7599
EN 220   -6171
EN 240   -3735
EN 260   -4566
EN 280   -1668
EN 300   -3595
EN 320   -2273
EN 340   -2046
EN 360   -1907
QUO-M5:LYS-S1
EN 20   0
EN 40   0
EN 60   0
EN 80   -16300
EN 100   0
EN 120   0
EN 140   0
EN 160   0
EN 180   0
EN 200   0
EN 220   0
EN 240   0
EN 260   0
EN 280   0
EN 300   0
EN 320   0
EN 340   0
EN 360   0
U-Y:ARG-S1
EN 20   -6335
EN 40   -3208
EN 60   -3837
EN 80   -5491
EN 100   -4807
EN 120   -5156
EN 140   -4162
EN 160   -3140
EN 180   -4452
EN 200   0
EN 220   -4068
EN 240   -1588
EN 260   -4270
EN 280   -2929
EN 300   -1874

EN 320   -2982
EN 340   -5146
EN 360   -5338
G-RIB:GLN-S2
EN 20   0
EN 40   -4491
EN 60   -5572
EN 80   -3402
EN 100   -4925
EN 120   -3480
EN 140   -5445
EN 160   -5517
EN 180   -7452
EN 200   -6868
EN 220   -5058
EN 240   -3583
EN 260   -3384
EN 280   -5575
EN 300   -4582
EN 320   -3260
EN 340   -5042
EN 360   -8338
G-R5:ASP-S2
EN 20   0
EN 40   -3182
EN 60   -2806
EN 80   -3810
EN 100   -1345
EN 120   -1618
EN 140   -3513
EN 160   -2109
EN 180   -1703
EN 200   0
EN 220   -6256
EN 240   -2826
EN 260   -4956
EN 280   -4067
EN 300   -842
EN 320   -3668
EN 340   -2728
EN 360   -1584
G-RIB:ARG-S2
EN 20   -9244
EN 40   -7338
EN 60   -7548
EN 80   -6077
EN 100   -5602
EN 120   -6066
EN 140   -5428
EN 160   -821
EN 180   -4854
EN 200   -8379
EN 220   -7800
EN 240   -6898
EN 260   -6682
EN 280   -6214
EN 300   -6424
EN 320   -4389

EN 340 -5365
EN 360 -6122
C-P:LYS-CA
EN 20 0
EN 40 -2215
EN 60 -3296
EN 80 -4852
EN 100 -4968
EN 120 -5517
EN 140 -6388
EN 160 -5963
EN 180 -5176
EN 200 0
EN 220 -3074
EN 240 -2311
EN 260 -4281
EN 280 -4487
EN 300 -5927
EN 320 -6529
EN 340 -6201
EN 360 -5610
U-P:ASN-S1
EN 20 -5954
EN 40 0
EN 60 -4620
EN 80 -472
EN 100 -5983
EN 120 -5703
EN 140 -6503
EN 160 -5742
EN 180 -4070
EN 200 0
EN 220 -4399
EN 240 -3928
EN 260 -4893
EN 280 -4717
EN 300 -3922
EN 320 -4318
EN 340 -6100
EN 360 -3952
G-R6:LEU-S1
EN 20 -2650
EN 40 -1241
EN 60 -604
EN 80 -603
EN 100 0
EN 120 -1235
EN 140 -477
EN 160 -168
EN 180 0
EN 200 0
EN 220 -1096
EN 240 2097
EN 260 -1590
EN 280 -1413
EN 300 93
EN 320 2419
EN 340 0

EN 360   0
A-P:GLU-S2
EN 20   -3450
EN 40   -3045
EN 60   -3834
EN 80   -1403
EN 100   -496
EN 120   -2486
EN 140   -3999
EN 160   749
EN 180   -3284
EN 200   0
EN 220   -4166
EN 240   -2137
EN 260   -2390
EN 280   -1761
EN 300   -4140
EN 320   -3201
EN 340   -3144
EN 360   -1448
U-P:SER-S1
EN 20   -5134
EN 40   -3725
EN 60   -2084
EN 80   -5594
EN 100   -3446
EN 120   -5673
EN 140   -4679
EN 160   -4922
EN 180   -3251
EN 200   0
EN 220   -3580
EN 240   -4374
EN 260   -5079
EN 280   -5163
EN 300   -5112
EN 320   -6933
EN 340   -5662
EN 360   -5855
C-RIB:ASN-S1
EN 20   0
EN 40   -5886
EN 60   -5701
EN 80   -5485
EN 100   -5704
EN 120   -4774
EN 140   -4952
EN 160   -3547
EN 180   -4859
EN 200   -5280
EN 220   -5188
EN 240   -6633
EN 260   -3965
EN 280   -4501
EN 300   -4711
EN 320   -4776
EN 340   -2449
EN 360   -6458

A-R6:GLN-S2
EN 20 0
EN 40 -4046
EN 60 -4122
EN 80 -4795
EN 100 -4601
EN 120 -5205
EN 140 -4548
EN 160 -4691
EN 180 -3572
EN 200 -5711
EN 220 -3901
EN 240 -4696
EN 260 -3944
EN 280 -1497
EN 300 -4811
EN 320 -5537
EN 340 -2880
EN 360 -3454
C-RIB:MET-S2
EN 20 0
EN 40 0
EN 60 -4139
EN 80 -4691
EN 100 -3944
EN 120 -4770
EN 140 -4012
EN 160 -6425
EN 180 -6019
EN 200 0
EN 220 -2913
EN 240 -3154
EN 260 -4120
EN 280 -4496
EN 300 -3440
EN 320 -5102
EN 340 -4614
EN 360 0
A-P:SER-CA
EN 20 0
EN 40 -1238
EN 60 -5302
EN 80 -3705
EN 100 -4846
EN 120 -4406
EN 140 -5825
EN 160 -5318
EN 180 -2482
EN 200 0
EN 220 -2811
EN 240 -3052
EN 260 -3305
EN 280 -4394
EN 300 -5056
EN 320 -4447
EN 340 -6229
EN 360 -6803
U-RIB:LEU-CA

```
EN 20   0
EN 40   0
EN 60   -2644
EN 80   0
EN 100   -1736
EN 120   -2562
EN 140   -3070
EN 160   -2500
EN 180   -5529
EN 200   0
EN 220   0
EN 240   -947
EN 260   -195
EN 280   -1023
EN 300   -1946
EN 320   -2341
EN 340   -3119
EN 360   0
U-Y:LYS-S1
EN 20   -6262
EN 40   -1417
EN 60   -3211
EN 80   -3502
EN 100   -2303
EN 120   -2576
EN 140   -2371
EN 160   -3067
EN 180   -2661
EN 200   -4799
EN 220   -5712
EN 240   -4618
EN 260   -4489
EN 280   -4573
EN 300   -3376
EN 320   -3913
EN 340   -5601
EN 360   -4260
U-RIB:TYR-S1
EN 20   0
EN 40   0
EN 60   0
EN 80   -4385
EN 100   -756
EN 120   -1029
EN 140   -5525
EN 160   -3950
EN 180   0
EN 200   0
EN 220   0
EN 240   0
EN 260   0
EN 280   -6200
EN 300   -966
EN 320   -1361
EN 340   -6126
EN 360   -4430
G-P:TYR-CA
EN 20   0
```

EN 40 0
EN 60 -3086
EN 80 -3638
EN 100 -3444
EN 120 -2451
EN 140 -2959
EN 160 -2650
EN 180 -4966
EN 200 0
EN 220 0
EN 240 -384
EN 260 -2354
EN 280 -2891
EN 300 -2388
EN 320 -4883
EN 340 -4274
EN 360 0
A-R6:THR-CA
EN 20 0
EN 40 -1475
EN 60 -3269
EN 80 -838
EN 100 -3748
EN 120 -3187
EN 140 -3142
EN 160 -2120
EN 180 -4436
EN 200 0
EN 220 -3048
EN 240 -4294
EN 260 -3542
EN 280 -1648
EN 300 -1858
EN 320 -2254
EN 340 -2026
EN 360 0
A-R6:ALA-S1
EN 20 0
EN 40 -1949
EN 60 -3029
EN 80 -1864
EN 100 -1117
EN 120 -1390
EN 140 -1185
EN 160 -876
EN 180 0
EN 200 0
EN 220 -2808
EN 240 -2045
EN 260 423
EN 280 -1117
EN 300 -3044
EN 320 -1009
EN 340 -1787
EN 360 -1356
U-RIB:ASP-CA
EN 20 0
EN 40 -1739

EN 60 -1815
EN 80 -2106
EN 100 809
EN 120 -4616
EN 140 -1689
EN 160 -3389
EN 180 -4700
EN 200 -5121
EN 220 -1594
EN 240 -118
EN 260 633
EN 280 -3629
EN 300 -4221
EN 320 -3783
EN 340 -4561
EN 360 -4582
A-P:ARG-S1
EN 20 0
EN 40 -4161
EN 60 -5242
EN 80 -5533
EN 100 -6343
EN 120 -6878
EN 140 -6254
EN 160 -6815
EN 180 -7414
EN 200 -4108
EN 220 -2299
EN 240 -5047
EN 260 -6426
EN 280 -6250
EN 300 -6730
EN 320 -6078
EN 340 -6580
EN 360 -6673
C-RIB:LEU-S1
EN 20 0
EN 40 -1644
EN 60 -2273
EN 80 -3277
EN 100 -1365
EN 120 -2090
EN 140 -3864
EN 160 -3293
EN 180 -2887
EN 200 0
EN 220 0
EN 240 -1028
EN 260 -3909
EN 280 -2821
EN 300 -4297
EN 320 -3135
EN 340 -2195
EN 360 -2769
A-RIB:GLU-CA
EN 20 0
EN 40 -1999
EN 60 0

EN 80 -3370
EN 100 -3176
EN 120 -3157
EN 140 -2501
EN 160 -926
EN 180 0
EN 200 0
EN 220 0
EN 240 0
EN 260 -1343
EN 280 -1167
EN 300 -2763
EN 320 -1059
EN 340 -2550
EN 360 -1406
A-R6:HIS-S1
EN 20 -6397
EN 40 0
EN 60 -4351
EN 80 -4350
EN 100 -2439
EN 120 -4982
EN 140 -1502
EN 160 -4920
EN 180 0
EN 200 0
EN 220 -3125
EN 240 -6471
EN 260 -4332
EN 280 -5543
EN 300 -931
EN 320 -5314
EN 340 -2104
EN 360 -6113
G-RIB:SER-S1
EN 20 -6525
EN 40 -4111
EN 60 -4740
EN 80 -5190
EN 100 -4482
EN 120 -2387
EN 140 -5065
EN 160 -3591
EN 180 -4641
EN 200 -6780
EN 220 -5683
EN 240 -3043
EN 260 -3455
EN 280 -4283
EN 300 -3780
EN 320 -4437
EN 340 -5214
EN 360 -6240
A-R5:LYS-S1
EN 20 -6499
EN 40 -2368
EN 60 -3830
EN 80 -4000

EN 100   -4258
EN 120   -2362
EN 140   -4034
EN 160   422
EN 180   0
EN 200   -7467
EN 220   -5949
EN 240   -4971
EN 260   -4219
EN 280   -2540
EN 300   -4850
EN 320   -3146
EN 340   -1201
EN 360   -1775
U-P:ARG-CA
EN 20   0
EN 40   -1496
EN 60   -2577
EN 80   -3580
EN 100   -5816
EN 120   -5929
EN 140   -7386
EN 160   -5575
EN 180   -6174
EN 200   0
EN 220   -4073
EN 240   -3310
EN 260   -2558
EN 280   -5816
EN 300   -6452
EN 320   -4996
EN 340   -5151
EN 360   -6056
G-R6:ASP-CA
EN 20   0
EN 40   -412
EN 60   -2759
EN 80   -1918
EN 100   -1298
EN 120   145
EN 140   1355
EN 160   -2774
EN 180   -1656
EN 200   0
EN 220   -2989
EN 240   -509
EN 260   -2027
EN 280   -3568
EN 300   -1508
EN 320   -186
EN 340   -2681
EN 360   -3255
C-Y:LYS-S2
EN 20   -8107
EN 40   -6246
EN 60   -5439
EN 80   -6259
EN 100   -4149

EN 120 -3086
EN 140 -3212
EN 160 -3908
EN 180 -3502
EN 200 -7911
EN 220 -5548
EN 240 -5459
EN 260 -4878
EN 280 -4347
EN 300 -3646
EN 320 -4302
EN 340 -4819
EN 360 -3383
U-RIB:ASN-CA
EN 20 -7666
EN 40 -2822
EN 60 -2898
EN 80 -4455
EN 100 -2995
EN 120 -4534
EN 140 -4489
EN 160 -5184
EN 180 -6788
EN 200 -6204
EN 220 -5399
EN 240 -5189
EN 260 -3884
EN 280 -4261
EN 300 -4470
EN 320 -5318
EN 340 -4378
EN 360 -7935
C-RIB:TRP-CA
EN 20 0
EN 40 0
EN 60 -4655
EN 80 -2224
EN 100 -4752
EN 120 -6291
EN 140 0
EN 160 0
EN 180 -5823
EN 200 0
EN 220 0
EN 240 0
EN 260 0
EN 280 -4752
EN 300 0
EN 320 0
EN 340 -5130
EN 360 -5704
A-P:TYR-S2
EN 20 0
EN 40 -6533
EN 60 -4338
EN 80 -4629
EN 100 -2718
EN 120 -1986

EN 140   -5216
EN 160   -1472
EN 180   -3788
EN 200   0
EN 220   -5122
EN 240   -5745
EN 260   -4160
EN 280   -1713
EN 300   -4193
EN 320   -4036
EN 340   -3096
EN 360   -3670
C-RIB:THR-S1
EN 20   -4445
EN 40   -5306
EN 60   -3665
EN 80   -4408
EN 100   -3922
EN 120   -4984
EN 140   -4703
EN 160   -2968
EN 180   -5284
EN 200   -4700
EN 220   -3895
EN 240   -5855
EN 260   -4098
EN 280   -3921
EN 300   -5136
EN 320   -5914
EN 340   -5596
EN 360   -5166
C-Y:ALA-S1
EN 20   -4918
EN 40   -4514
EN 60   -3254
EN 80   -2872
EN 100   -1965
EN 120   -520
EN 140   -24
EN 160   -2436
EN 180   0
EN 200   -8278
EN 220   -5081
EN 240   -4395
EN 260   -3145
EN 280   -247
EN 300   -2887
EN 320   -2118
EN 340   -625
EN 360   0
G-R5:VAL-CA
EN 20   0
EN 40   39
EN 60   1680
EN 80   2393
EN 100   -1399
EN 120   597
EN 140   89

EN 160   1111
EN 180   -1204
EN 200   -5060
EN 220   -1533
EN 240   -2327
EN 260   694
EN 280   -1399
EN 300   2378
EN 320   1982
EN 340   -511
EN 360   0
G-RIB:HIS-S1
EN 20   -5837
EN 40   -4428
EN 60   -1069
EN 80   -4343
EN 100   -2883
EN 120   -3157
EN 140   -6387
EN 160   -3355
EN 180   -7389
EN 200   0
EN 220   -2565
EN 240   -4524
EN 260   -5490
EN 280   -4601
EN 300   -3806
EN 320   -5588
EN 340   -6696
EN 360   -3836
G-R6:VAL-CA
EN 20   0
EN 40   0
EN 60   1680
EN 80   2393
EN 100   -1851
EN 120   -406
EN 140   1807
EN 160   0
EN 180   -1204
EN 200   0
EN 220   184
EN 240   -1062
EN 260   -1022
EN 280   -1272
EN 300   -343
EN 320   -739
EN 340   -2782
EN 360   0
A-R6:ASP-S1
EN 20   0
EN 40   -2692
EN 60   -1051
EN 80   -337
EN 100   -1860
EN 120   -2686
EN 140   -2641
EN 160   -2624

EN 180   0
EN 200   0
EN 220   -2547
EN 240   -2076
EN 260   -2036
EN 280   -2864
EN 300   -3456
EN 320   -2465
EN 340   191
EN 360   -2099
U31-RIB:TYR-S1
EN 20   0
EN 40   0
EN 60   0
EN 80   0
EN 100   0
EN 120   0
EN 140   0
EN 160   0
EN 180   0
EN 200   0
EN 220   0
EN 240   0
EN 260   0
EN 280   -17993
EN 300   0
EN 320   0
EN 340   0
EN 360   0
C-P:ILE-S1
EN 20   0
EN 40   -2789
EN 60   -2865
EN 80   -3538
EN 100   -3344
EN 120   -1517
EN 140   -3291
EN 160   -1716
EN 180   -2315
EN 200   0
EN 220   -926
EN 240   -2885
EN 260   -2133
EN 280   -2961
EN 300   -2167
EN 320   -4280
EN 340   0
EN 360   -2196
A-P:ASP-S1
EN 20   0
EN 40   -977
EN 60   -1053
EN 80   -3443
EN 100   -3580
EN 120   -3140
EN 140   -3648
EN 160   -2626
EN 180   -2220

EN 200   0
EN 220   -2549
EN 240   -3344
EN 260   -3425
EN 280   -2415
EN 300   -3459
EN 320   -3855
EN 340   -4250
EN 360   -2102
G-P:ARG-S2
EN 20   0
EN 40   -5647
EN 60   -5101
EN 80   -6925
EN 100   -6162
EN 120   -6874
EN 140   -6943
EN 160   -7473
EN 180   -5434
EN 200   0
EN 220   -3493
EN 240   -4739
EN 260   -5704
EN 280   -7030
EN 300   -6527
EN 320   -6425
EN 340   -7624
EN 360   -7033
C-RIB:ILE-CA
EN 20   0
EN 40   -2779
EN 60   -1138
EN 80   -3528
EN 100   -2952
EN 120   -3225
EN 140   705
EN 160   -1706
EN 180   -2305
EN 200   0
EN 220   -916
EN 240   0
EN 260   -2676
EN 280   -1234
EN 300   -3874
EN 320   -4270
EN 340   -3330
EN 360   -3904
U-RIB:ASP-S2
EN 20   0
EN 40   -1787
EN 60   -1863
EN 80   -3419
EN 100   -1959
EN 120   -2233
EN 140   -4458
EN 160   -4702
EN 180   -4747
EN 200   0

EN 220   -3359
EN 240   -2888
EN 260   -2136
EN 280   -2672
EN 300   -3435
EN 320   -3278
EN 340   -3342
EN 360   -4629
A-RIB:VAL-CA
EN 20   0
EN 40   -3243
EN 60   -2314
EN 80   -2153
EN 100   -1406
EN 120   -3066
EN 140   -2740
EN 160   -4600
EN 180   -1764
EN 200   0
EN 220   0
EN 240   1099
EN 260   -2587
EN 280   -3415
EN 300   -2620
EN 320   -3016
EN 340   -2789
EN 360   -4367
C-Y:HIS-S1
EN 20   0
EN 40   -5836
EN 60   -5912
EN 80   -4194
EN 100   -5386
EN 120   -2555
EN 140   -3063
EN 160   -2041
EN 180   -4357
EN 200   0
EN 220   -6403
EN 240   -5481
EN 260   -6185
EN 280   -3286
EN 300   -774
EN 320   -2887
EN 340   -1947
EN 360   0
A-R5:GLU-S1
EN 20   0
EN 40   -4727
EN 60   -1369
EN 80   -1921
EN 100   -2178
EN 120   -1447
EN 140   -2959
EN 160   784
EN 180   0
EN 200   -3670
EN 220   -3578

EN 240   -385
EN 260   -637
EN 280   -461
EN 300   -1936
EN 320   -1066
EN 340   877
EN 360   -1413
U-Y:CYS-S1
EN 20   0
EN 40   0
EN 60   0
EN 80   -3643
EN 100   0
EN 120   0
EN 140   -4230
EN 160   0
EN 180   0
EN 200   0
EN 220   -5853
EN 240   -3372
EN 260   0
EN 280   -5166
EN 300   -3658
EN 320   -4054
EN 340   0
EN 360   0
G-RIB:GLY-CA
EN 20   -3186
EN 40   -4499
EN 60   -5713
EN 80   -3861
EN 100   -5583
EN 120   -5079
EN 140   -5311
EN 160   -5696
EN 180   -4737
EN 200   -6163
EN 220   -4735
EN 240   -4380
EN 260   -4848
EN 280   -5168
EN 300   -4429
EN 320   -4985
EN 340   -5248
EN 360   -6336
C-P:HIS-S2
EN 20   0
EN 40   -6575
EN 60   -4221
EN 80   -4773
EN 100   -5413
EN 120   -4299
EN 140   -5812
EN 160   -5503
EN 180   0
EN 200   0
EN 220   -6983
EN 240   -3237

```
EN 260   -5589
EN 280   -5031
EN 300   -5240
EN 320   -5636
EN 340   -3691
EN 360   -7700
G-R6:ASN-S2
EN 20   0
EN 40   0
EN 60   -4867
EN 80   -4313
EN 100   -3698
EN 120   -3679
EN 140   -4569
EN 160   -1830
EN 180   -4477
EN 200   -4898
EN 220   -4806
EN 240   -5537
EN 260   -3964
EN 280   -4411
EN 300   -4551
EN 320   -3560
EN 340   -2067
EN 360   0
FHU-MY:LEU-S2
EN 20   0
EN 40   0
EN 60   0
EN 80   -14018
EN 100   0
EN 120   0
EN 140   0
EN 160   -15300
EN 180   0
EN 200   0
EN 220   0
EN 240   0
EN 260   0
EN 280   -13824
EN 300   0
EN 320   0
EN 340   -15207
EN 360   0
U-Y:ALA-CA
EN 20   -4125
EN 40   -2716
EN 60   -3796
EN 80   -2631
EN 100   -1171
EN 120   -1152
EN 140   -496
EN 160   -2647
EN 180   0
EN 200   0
EN 220   -853
EN 240   -3365
EN 260   -3778
```

EN 280   -1171
EN 300   -1381
EN 320   -1776
EN 340   -2554
EN 360   0
U-Y:TRP-S2
EN 20   -8630
EN 40   -5503
EN 60   -5579
EN 80   -3148
EN 100   -2954
EN 120   0
EN 140   0
EN 160   0
EN 180   0
EN 200   -8885
EN 220   0
EN 240   0
EN 260   -4848
EN 280   0
EN 300   -3164
EN 320   -3559
EN 340   0
EN 360   0
A-P:ASN-S1
EN 20   -5184
EN 40   -2058
EN 60   -2134
EN 80   -4524
EN 100   -6218
EN 120   -4603
EN 140   -3012
EN 160   -6137
EN 180   -3301
EN 200   0
EN 220   -3630
EN 240   -4876
EN 260   -4506
EN 280   -4661
EN 300   -3706
EN 320   -4553
EN 340   -6043
EN 360   -3183
A-R5:TYR-S1
EN 20   0
EN 40   -2538
EN 60   -5718
EN 80   -4623
EN 100   -1706
EN 120   -4701
EN 140   -4758
EN 160   0
EN 180   -3781
EN 200   0
EN 220   -4110
EN 240   -917
EN 260   -3600
EN 280   -1706

EN 300 -199
EN 320 -3101
EN 340 -1372
EN 360 -3663
C-Y:LEU-S1
EN 20 -3053
EN 40 72
EN 60 -1007
EN 80 710
EN 100 -1817
EN 120 -2472
EN 140 1841
EN 160 -571
EN 180 0
EN 200 -3308
EN 220 -3216
EN 240 -3458
EN 260 -276
EN 280 -812
EN 300 695
EN 320 2016
EN 340 1239
EN 360 -1051
U-RIB:VAL-S1
EN 20 0
EN 40 -1289
EN 60 -2370
EN 80 -2922
EN 100 -3180
EN 120 -3453
EN 140 -2243
EN 160 -1934
EN 180 -2533
EN 200 0
EN 220 0
EN 240 330
EN 260 -2904
EN 280 -2175
EN 300 1049
EN 320 -4167
EN 340 -1840
EN 360 0
A-RIB:PRO-CA
EN 20 0
EN 40 -1675
EN 60 -3468
EN 80 -3307
EN 100 -4278
EN 120 -5340
EN 140 -2629
EN 160 -4589
EN 180 -6353
EN 200 0
EN 220 0
EN 240 -3488
EN 260 -4746
EN 280 -2560
EN 300 -3775

EN 320   -5436
EN 340   -3230
EN 360   0
G-R6:PRO-CA
EN 20   0
EN 40   -4549
EN 60   -3913
EN 80   -2747
EN 100   -283
EN 120   -3278
EN 140   -1064
EN 160   -42
EN 180   0
EN 200   0
EN 220   -2687
EN 240   -2216
EN 260   -3473
EN 280   -3387
EN 300   0
EN 320   -1893
EN 340   -2670
EN 360   -2240
QUO-M5:ARG-S2
EN 20   0
EN 40   0
EN 60   0
EN 80   0
EN 100   -16191
EN 120   0
EN 140   0
EN 160   0
EN 180   -19984
EN 200   0
EN 220   0
EN 240   0
EN 260   0
EN 280   0
EN 300   0
EN 320   0
EN 340   0
EN 360   0
C-Y:THR-CA
EN 20   -4445
EN 40   -3036
EN 60   -2399
EN 80   -3403
EN 100   -1989
EN 120   956
EN 140   -2985
EN 160   -246
EN 180   -2562
EN 200   -7422
EN 220   -1173
EN 240   -5403
EN 260   -2380
EN 280   -487
EN 300   -696
EN 320   -2097

EN 340   -2874
EN 360   0
A-P:GLU-S1
EN 20   0
EN 40   -2008
EN 60   -3471
EN 80   -2757
EN 100   540
EN 120   -3459
EN 140   -3344
EN 160   781
EN 180   0
EN 200   0
EN 220   -145
EN 240   -1392
EN 260   -2357
EN 280   -2181
EN 300   -2772
EN 320   -2335
EN 340   -2559
EN 360   -1416
A-RIB:THR-S1
EN 20   0
EN 40   -5463
EN 60   -4655
EN 80   -3108
EN 100   -4078
EN 120   -4643
EN 140   -4529
EN 160   -4390
EN 180   -4436
EN 200   0
EN 220   -5770
EN 240   -4676
EN 260   -5259
EN 280   -3747
EN 300   -4841
EN 320   -3971
EN 340   -5461
EN 360   -2600
A-R6:THR-S1
EN 20   0
EN 40   -3193
EN 60   -2556
EN 80   -4565
EN 100   -4078
EN 120   -3639
EN 140   -1425
EN 160   -3837
EN 180   -2719
EN 200   0
EN 220   -5318
EN 240   -3289
EN 260   -4546
EN 280   -2361
EN 300   -2411
EN 320   -1249
EN 340   -4297

EN 360 -2600
G-RIB:THR-CA
EN 20 -5760
EN 40 -3637
EN 60 -992
EN 80 -3713
EN 100 -3188
EN 120 -4083
EN 140 -3969
EN 160 -3277
EN 180 -2159
EN 200 -7019
EN 220 -4205
EN 240 -2729
EN 260 -1977
EN 280 -1088
EN 300 -3015
EN 320 -2959
EN 340 -3184
EN 360 -5475
C-Y:HIS-S2
EN 20 -6257
EN 40 -6566
EN 60 -4212
EN 80 -5215
EN 100 -5734
EN 120 -2572
EN 140 -3080
EN 160 -2058
EN 180 0
EN 200 0
EN 220 -5708
EN 240 -7215
EN 260 -5197
EN 280 -4569
EN 300 -2509
EN 320 0
EN 340 -1964
EN 360 -4256
G-P:TRP-S2
EN 20 0
EN 40 -5920
EN 60 -4279
EN 80 -4570
EN 100 -4376
EN 120 -3644
EN 140 -5157
EN 160 -6565
EN 180 0
EN 200 0
EN 220 -4058
EN 240 -2582
EN 260 -3548
EN 280 -5089
EN 300 -4586
EN 320 -5694
EN 340 -3037
EN 360 -5328

G-R5:ILE-CA
EN 20 -3785
EN 40 0
EN 60 982
EN 80 2408
EN 100 -831
EN 120 0
EN 140 0
EN 160 0
EN 180 0
EN 200 -4040
EN 220 -2231
EN 240 -755
EN 260 -3
EN 280 -831
EN 300 -36
EN 320 0
EN 340 -1209
EN 360 0
A-P:MET-S2
EN 20 -6344
EN 40 -3217
EN 60 -5011
EN 80 -2580
EN 100 -668
EN 120 -2659
EN 140 -3167
EN 160 -2145
EN 180 0
EN 200 0
EN 220 0
EN 240 -4318
EN 260 -2562
EN 280 -4656
EN 300 -878
EN 320 -2991
EN 340 -2051
EN 360 0
G-RIB:LEU-CA
EN 20 0
EN 40 476
EN 60 -604
EN 80 -603
EN 100 -3131
EN 120 -2069
EN 140 -2577
EN 160 -1173
EN 180 -4201
EN 200 0
EN 220 621
EN 240 -2342
EN 260 1131
EN 280 -3329
EN 300 -1172
EN 320 -2019
EN 340 -1079
EN 360 -3370
G-P:GLU-S1

EN 20 0
EN 40 244
EN 60 1885
EN 80 -1840
EN 100 -2358
EN 120 -3184
EN 140 -3692
EN 160 -4387
EN 180 -998
EN 200 0
EN 220 -2332
EN 240 147
EN 260 -2534
EN 280 2793
EN 300 -1855
EN 320 -2251
EN 340 -3028
EN 360 -2597
C-P:PHE-S2
EN 20 0
EN 40 0
EN 60 -4379
EN 80 -1395
EN 100 -1200
EN 120 -3191
EN 140 -1981
EN 160 -2677
EN 180 0
EN 200 0
EN 220 -1887
EN 240 -4399
EN 260 -2381
EN 280 -2205
EN 300 -3127
EN 320 -4076
EN 340 -2583
EN 360 0
C-Y:GLN-S1
EN 20 0
EN 40 -4863
EN 60 -5321
EN 80 -4608
EN 100 407
EN 120 -3300
EN 140 -373
EN 160 -1069
EN 180 0
EN 200 -7241
EN 220 -5431
EN 240 -6876
EN 260 -3203
EN 280 -3027
EN 300 0
EN 320 -3632
EN 340 -975
EN 360 0
U-P:HIS-S2
EN 20 0

EN 40   -5777
EN 60   -4136
EN 80   -6526
EN 100   -4945
EN 120   -3501
EN 140   -5726
EN 160   -5709
EN 180   -8738
EN 200   0
EN 220   -7349
EN 240   -6878
EN 260   -6126
EN 280   -6662
EN 300   -5708
EN 320   -6103
EN 340   -2893
EN 360   -5184
IU-P:ALA-CA
EN 20   0
EN 40   0
EN 60   -13872
EN 80   0
EN 100   0
EN 120   0
EN 140   0
EN 160   0
EN 180   0
EN 200   0
EN 220   0
EN 240   0
EN 260   0
EN 280   0
EN 300   0
EN 320   0
EN 340   0
EN 360   0
G-P:TYR-S1
EN 20   0
EN 40   0
EN 60   -2081
EN 80   -3085
EN 100   -3895
EN 120   -4169
EN 140   -2959
EN 160   -6085
EN 180   0
EN 200   0
EN 220   0
EN 240   -384
EN 260   -1350
EN 280   -2891
EN 300   -3100
EN 320   -4049
EN 340   -3561
EN 360   -3130
G-RIB:LYS-CA
EN 20   -5933
EN 40   -4072

EN 60   -4798
EN 80   -4891
EN 100   -5194
EN 120   -4164
EN 140   -1751
EN 160   -3833
EN 180   -4763
EN 200   -5184
EN 220   -5644
EN 240   -2451
EN 260   -5255
EN 280   -2687
EN 300   -4615
EN 320   -4496
EN 340   -3357
EN 360   -3931
C-P:TRP-CA
EN 20   0
EN 40   0
EN 60   -2948
EN 80   -2234
EN 100   0
EN 120   -5035
EN 140   -4538
EN 160   -3516
EN 180   0
EN 200   0
EN 220   0
EN 240   -5690
EN 260   -3933
EN 280   -4762
EN 300   0
EN 320   -4363
EN 340   -5140
EN 360   0
C-RIB:GLN-CA
EN 20   0
EN 40   -3859
EN 60   -3935
EN 80   -4608
EN 100   -3027
EN 120   -4687
EN 140   -4813
EN 160   -3791
EN 180   -6107
EN 200   0
EN 220   -1996
EN 240   -2238
EN 260   -4590
EN 280   -4744
EN 300   -6157
EN 320   -5350
EN 340   -6680
EN 360   -3266
U-Y:MET-CA
EN 20   0
EN 40   -3971
EN 60   -5052

EN 80 -3333
EN 100 0
EN 120 0
EN 140 0
EN 160 0
EN 180 0
EN 200 0
EN 220 -6548
EN 240 -2350
EN 260 -3315
EN 280 0
EN 300 -1631
EN 320 -2027
EN 340 0
EN 360 0
G-R5:GLU-CA
EN 20 -2848
EN 40 -1439
EN 60 202
EN 80 2633
EN 100 2827
EN 120 836
EN 140 2759
EN 160 -366
EN 180 -965
EN 200 0
EN 220 423
EN 240 -1535
EN 260 -70
EN 280 -447
EN 300 2617
EN 320 -1052
EN 340 -1277
EN 360 -2564
U34-P:HIS-CA
EN 20 0
EN 40 0
EN 60 0
EN 80 0
EN 100 0
EN 120 0
EN 140 0
EN 160 -20202
EN 180 0
EN 200 0
EN 220 0
EN 240 0
EN 260 0
EN 280 0
EN 300 0
EN 320 0
EN 340 0
EN 360 0
A-R6:VAL-CA
EN 20 0
EN 40 -520
EN 60 1120
EN 80 -3610

EN 100 -1959
EN 120 -1679
EN 140 -2187
EN 160 551
EN 180 -1764
EN 200 0
EN 220 0
EN 240 1099
EN 260 -2135
EN 280 -1959
EN 300 -2169
EN 320 -2564
EN 340 -1071
EN 360 0
U-Y:THR-S1
EN 20 0
EN 40 -4965
EN 60 -5041
EN 80 -1605
EN 100 -4845
EN 120 -3954
EN 140 -2192
EN 160 -3892
EN 180 0
EN 200 -5624
EN 220 -4819
EN 240 -4056
EN 260 -2592
EN 280 306
EN 300 -2625
EN 320 -299
EN 340 0
EN 360 0
U-RIB:HIS-S2
EN 20 0
EN 40 -4055
EN 60 -2413
EN 80 -7144
EN 100 -5945
EN 120 -4501
EN 140 -6726
EN 160 -7421
EN 180 -8733
EN 200 0
EN 220 -3909
EN 240 -2434
EN 260 -5669
EN 280 -5493
EN 300 -5150
EN 320 -5546
EN 340 -2888
EN 360 -6897
A-R5:ARG-S2
EN 20 -8302
EN 40 -6677
EN 60 -7167
EN 80 -5543
EN 100 -5482

EN 120   -5480
EN 140   -4852
EN 160   -2385
EN 180   -3697
EN 200   -9270
EN 220   -5412
EN 240   -5654
EN 260   -5383
EN 280   -5730
EN 300   -5106
EN 320   -4497
EN 340   -3004
EN 360   -1861
G-R5:LYS-CA
EN 20   -5933
EN 40   -2806
EN 60   -2431
EN 80   -1164
EN 100   -2357
EN 120   -2882
EN 140   -2304
EN 160   -2446
EN 180   -3045
EN 200   -7454
EN 220   -3927
EN 240   -1898
EN 260   -2533
EN 280   -3476
EN 300   -4455
EN 320   -3585
EN 340   -3357
EN 360   -1209
C-P:LEU-S1
EN 20   0
EN 40   63
EN 60   -3116
EN 80   -2403
EN 100   -1375
EN 120   -3817
EN 140   -2156
EN 160   -581
EN 180   -1180
EN 200   0
EN 220   208
EN 240   1684
EN 260   -3505
EN 280   -2208
EN 300   -2418
EN 320   -1427
EN 340   -1492
EN 360   -1061
G-R6:SER-CA
EN 20   0
EN 40   -3398
EN 60   -4187
EN 80   -3473
EN 100   -2114
EN 120   -1835

EN 140   -625
EN 160   -1320
EN 180   0
EN 200   0
EN 220   -2248
EN 240   -772
EN 260   -3376
EN 280   -2948
EN 300   -4041
EN 320   -2167
EN 340   -1227
EN 360   -1801
FHU-P:LEU-S2
EN 20   0
EN 40   0
EN 60   0
EN 80   -14018
EN 100   0
EN 120   0
EN 140   0
EN 160   0
EN 180   0
EN 200   0
EN 220   0
EN 240   -14752
EN 260   0
EN 280   0
EN 300   0
EN 320   -14429
EN 340   0
EN 360   0
U-Y:SER-CA
EN 20   0
EN 40   -4725
EN 60   -4801
EN 80   -4348
EN 100   -2176
EN 120   -3162
EN 140   -235
EN 160   -930
EN 180   0
EN 200   -5385
EN 220   -3575
EN 240   -3104
EN 260   -2613
EN 280   -2888
EN 300   -929
EN 320   -4047
EN 340   -4824
EN 360   -3128
C-Y:ARG-S1
EN 20   -6416
EN 40   -5007
EN 60   -5465
EN 80   -5508
EN 100   -5677
EN 120   -3443
EN 140   -5278

EN 160   -2929
EN 180   -6250
EN 200   -5666
EN 220   -6579
EN 240   -5301
EN 260   -5214
EN 280   -4887
EN 300   -3672
EN 320   -3445
EN 340   -2835
EN 360   -3409
G-R5:LYS-S1
EN 20   -5939
EN 40   -5975
EN 60   -4154
EN 80   -2557
EN 100   -2985
EN 120   -2888
EN 140   -1757
EN 160   -2452
EN 180   -5321
EN 200   -7912
EN 220   -1663
EN 240   -3291
EN 260   -2539
EN 280   -3482
EN 300   -4771
EN 320   -4303
EN 340   -5081
EN 360   0
FHU-RIB:ARG-S1
EN 20   0
EN 40   0
EN 60   0
EN 80   -14656
EN 100   0
EN 120   0
EN 140   0
EN 160   0
EN 180   0
EN 200   0
EN 220   0
EN 240   0
EN 260   -14638
EN 280   0
EN 300   0
EN 320   0
EN 340   -15844
EN 360   -18136
C-Y:ILE-S1
EN 20   -4188
EN 40   -2779
EN 60   -1138
EN 80   -1429
EN 100   -1234
EN 120   -503
EN 140   -1011
EN 160   0

EN 180 0
EN 200 0
EN 220 -3638
EN 240 -2876
EN 260 -406
EN 280 -1947
EN 300 -1444
EN 320 881
EN 340 0
EN 360 0
A-R6:HIS-S2
EN 20 0
EN 40 -5005
EN 60 -4368
EN 80 -933
EN 100 -5178
EN 120 -5833
EN 140 -5507
EN 160 -3932
EN 180 -6248
EN 200 0
EN 220 -4860
EN 240 -4389
EN 260 -6856
EN 280 -6182
EN 300 -3670
EN 320 -1344
EN 340 -4843
EN 360 -4412
A-RIB:SER-CA
EN 20 -7084
EN 40 -5223
EN 60 -3029
EN 80 -3320
EN 100 -4131
EN 120 -1682
EN 140 -2902
EN 160 -2885
EN 180 -2479
EN 200 -4618
EN 220 -5530
EN 240 -4436
EN 260 -3684
EN 280 -4130
EN 300 -5435
EN 320 -3731
EN 340 -4057
EN 360 -4078
U31-MY:ASP-S2
EN 20 0
EN 40 0
EN 60 0
EN 80 -16669
EN 100 0
EN 120 -16748
EN 140 0
EN 160 0
EN 180 0

EN 200 0
EN 220 0
EN 240 -17403
EN 260 0
EN 280 -16474
EN 300 0
EN 320 0
EN 340 0
EN 360 0
G-R6:ARG-S1
EN 20 0
EN 40 -2886
EN 60 -3344
EN 80 -4679
EN 100 -3970
EN 120 -3530
EN 140 -3217
EN 160 -3912
EN 180 0
EN 200 0
EN 220 -2741
EN 240 -3364
EN 260 -3496
EN 280 -3556
EN 300 -4534
EN 320 -2660
EN 340 -2985
EN 360 -4724
G-R5:THR-CA
EN 20 -4042
EN 40 -2633
EN 60 725
EN 80 -2548
EN 100 -84
EN 120 1360
EN 140 852
EN 160 -2565
EN 180 0
EN 200 -4297
EN 220 -4758
EN 240 -4116
EN 260 -2982
EN 280 -2354
EN 300 -1298
EN 320 -2406
EN 340 0
EN 360 0
A-R5:ASN-CA
EN 20 -7904
EN 40 -3772
EN 60 -3136
EN 80 -3688
EN 100 -3494
EN 120 -2501
EN 140 -3009
EN 160 0
EN 180 0
EN 200 -5437

EN 220 -1910
EN 240 -2152
EN 260 -4503
EN 280 -1223
EN 300 283
EN 320 -2833
EN 340 -889
EN 360 -4567
C-P:CYS-CA
EN 20 0
EN 40 0
EN 60 0
EN 80 -4446
EN 100 0
EN 120 0
EN 140 -3316
EN 160 0
EN 180 -6327
EN 200 0
EN 220 0
EN 240 -3463
EN 260 -2711
EN 280 -2535
EN 300 0
EN 320 -5862
EN 340 0
EN 360 0
A-RIB:HIS-S2
EN 20 0
EN 40 0
EN 60 -6086
EN 80 -4920
EN 100 -5560
EN 120 -4446
EN 140 -5959
EN 160 -4937
EN 180 -7253
EN 200 0
EN 220 -4860
EN 240 -6488
EN 260 -6620
EN 280 -6443
EN 300 -5769
EN 320 -6165
EN 340 -5556
EN 360 -4412
C-P:TYR-S1
EN 20 0
EN 40 -5826
EN 60 -4738
EN 80 -4858
EN 100 -1559
EN 120 -4103
EN 140 -3345
EN 160 -1318
EN 180 -3634
EN 200 0
EN 220 0

EN 240   -770
EN 260   -1736
EN 280   -3829
EN 300   -4491
EN 320   -2165
EN 340   -3947
EN 360   0
U31-MY:ILE-S1
EN 20   0
EN 40   0
EN 60   0
EN 80   0
EN 100   0
EN 120   0
EN 140   0
EN 160   0
EN 180   0
EN 200   0
EN 220   0
EN 240   0
EN 260   0
EN 280   0
EN 300   0
EN 320   0
EN 340   -18056
EN 360   0
G-RIB:ASN-S1
EN 20   -6339
EN 40   -3213
EN 60   -5795
EN 80   -5297
EN 100   -4390
EN 120   -3658
EN 140   -3715
EN 160   -5574
EN 180   0
EN 200   -4877
EN 220   -3067
EN 240   -6031
EN 260   -3944
EN 280   -5301
EN 300   -4600
EN 320   -3539
EN 340   -4316
EN 360   -4337
C-RIB:ASP-S2
EN 20   0
EN 40   -2580
EN 60   -2656
EN 80   -4449
EN 100   -3758
EN 120   -4031
EN 140   -5036
EN 160   -6145
EN 180   -5541
EN 200   0
EN 220   -2435
EN 240   -3681

EN 260   -4845
EN 280   -3135
EN 300   -4464
EN 320   -5076
EN 340   -4518
EN 360   -6427
FHU-MY:PRO-S1
EN 20   0
EN 40   0
EN 60   0
EN 80   0
EN 100   -15412
EN 120   0
EN 140   0
EN 160   0
EN 180   0
EN 200   0
EN 220   0
EN 240   0
EN 260   0
EN 280   0
EN 300   0
EN 320   0
EN 340   0
EN 360   0
C-RIB:ALA-S1
EN 20   -4918
EN 40   -4062
EN 60   -3254
EN 80   -3163
EN 100   -3682
EN 120   -2951
EN 140   -4011
EN 160   -3441
EN 180   -5305
EN 200   -5174
EN 220   -2651
EN 240   -4159
EN 260   -3858
EN 280   -3466
EN 300   -4445
EN 320   -3836
EN 340   -3729
EN 360   -1199
A-R6:GLN-S1
EN 20   0
EN 40   -2298
EN 60   -3379
EN 80   -2665
EN 100   -3737
EN 120   -1739
EN 140   -3252
EN 160   -4660
EN 180   -5259
EN 200   0
EN 220   -2153
EN 240   -2395
EN 260   -3913

EN 280   -1466
EN 300   -3946
EN 320   -5798
EN 340   -2849
EN 360   -3423
A-R6:ASN-S2
EN 20   0
EN 40   -6064
EN 60   -2152
EN 80   -4160
EN 100   -4348
EN 120   -3235
EN 140   -4642
EN 160   -2720
EN 180   -3319
EN 200   0
EN 220   -5365
EN 240   -5607
EN 260   -5644
EN 280   -4679
EN 300   -2458
EN 320   -4572
EN 340   -4344
EN 360   -4918
A-R5:THR-CA
EN 20   -6319
EN 40   -1475
EN 60   -3822
EN 80   -3108
EN 100   -4078
EN 120   -3187
EN 140   292
EN 160   -3837
EN 180   0
EN 200   0
EN 220   -4765
EN 240   -4676
EN 260   -2537
EN 280   -3365
EN 300   -2571
EN 320   -1249
EN 340   -4297
EN 360   0
U-RIB:TRP-S2
EN 20   0
EN 40   0
EN 60   -3862
EN 80   -5870
EN 100   0
EN 120   -5949
EN 140   -3735
EN 160   -4430
EN 180   0
EN 200   0
EN 220   0
EN 240   -3882
EN 260   -6565
EN 280   -4671

EN 300 0
EN 320 0
EN 340 -4337
EN 360 -6628
U-Y:LYS-CA
EN 20 -4538
EN 40 -1412
EN 60 -2492
EN 80 -1779
EN 100 -1584
EN 120 -3123
EN 140 356
EN 160 -339
EN 180 -3921
EN 200 -7515
EN 220 -3988
EN 240 -1508
EN 260 -3860
EN 280 -2297
EN 300 -2507
EN 320 -3455
EN 340 -3680
EN 360 0
C-Y:LYS-S1
EN 20 -8442
EN 40 -4933
EN 60 -4794
EN 80 -2960
EN 100 -3388
EN 120 -3662
EN 140 -443
EN 160 -2856
EN 180 -3454
EN 200 -6597
EN 220 -3783
EN 240 -3312
EN 260 -4277
EN 280 -3388
EN 300 -1589
EN 320 -2537
EN 340 -2049
EN 360 -4341
G-R6:GLU-S2
EN 20 -2887
EN 40 -4200
EN 60 -3271
EN 80 -1845
EN 100 -2585
EN 120 -1924
EN 140 -499
EN 160 -3509
EN 180 -2721
EN 200 0
EN 220 384
EN 240 -4024
EN 260 -2540
EN 280 -1199
EN 300 -143

EN 320   -1804
EN 340   -1100
EN 360   -2603
A-RIB:LEU-S1
EN 20   0
EN 40   -3518
EN 60   -159
EN 80   553
EN 100   -2686
EN 120   -529
EN 140   -2303
EN 160   -3450
EN 180   -3044
EN 200   0
EN 220   61
EN 240   0
EN 260   -1145
EN 280   -1973
EN 300   -466
EN 320   -2961
EN 340   -2905
EN 360   -2926
U31-MY:ASP-S1
EN 20   0
EN 40   0
EN 60   -17337
EN 80   -16624
EN 100   -16429
EN 120   -16703
EN 140   0
EN 160   0
EN 180   0
EN 200   0
EN 220   0
EN 240   0
EN 260   0
EN 280   0
EN 300   0
EN 320   0
EN 340   0
EN 360   0
C-P:TRP-S1
EN 20   0
EN 40   0
EN 60   -2948
EN 80   -3951
EN 100   -2040
EN 120   -2313
EN 140   -4538
EN 160   -3516
EN 180   0
EN 200   0
EN 220   -6161
EN 240   -4685
EN 260   -3933
EN 280   -3757
EN 300   0
EN 320   -6633

EN 340   0
EN 360   0
G-P:HIS-S2
EN 20   0
EN 40   -4472
EN 60   -5935
EN 80   -4839
EN 100   -5911
EN 120   -5300
EN 140   -4974
EN 160   -4404
EN 180   0
EN 200   0
EN 220   -5331
EN 240   -3856
EN 260   -4821
EN 280   -6915
EN 300   -5237
EN 320   -5963
EN 340   -5576
EN 360   -5597
G-R6:MET-S1
EN 20   0
EN 40   -2644
EN 60   0
EN 80   -5110
EN 100   -1812
EN 120   -368
EN 140   0
EN 160   0
EN 180   0
EN 200   0
EN 220   -2499
EN 240   -1023
EN 260   -3706
EN 280   -2816
EN 300   -3026
EN 320   -700
EN 340   -4199
EN 360   0
U-Y:GLY-CA
EN 20   -8952
EN 40   -4821
EN 60   -6100
EN 80   968
EN 100   -3989
EN 120   -3098
EN 140   -1336
EN 160   -2031
EN 180   -4347
EN 200   -7490
EN 220   -6393
EN 240   -5470
EN 260   -5368
EN 280   -3658
EN 300   -1769
EN 320   557
EN 340   -3655

EN 360   -4229
A-R5:TYR-S2
EN 20   -7386
EN 40   -2542
EN 60   -4336
EN 80   -4627
EN 100   -2715
EN 120   -3701
EN 140   -3496
EN 160   -3187
EN 180   0
EN 200   -8646
EN 220   -2397
EN 240   -3643
EN 260   -4991
EN 280   6
EN 300   -1920
EN 320   0
EN 340   -1376
EN 360   -5385
G-P:SER-CA
EN 20   0
EN 40   -3425
EN 60   -3501
EN 80   -3792
EN 100   -2975
EN 120   -4966
EN 140   -6357
EN 160   -5335
EN 180   -1946
EN 200   0
EN 220   -2275
EN 240   -4234
EN 260   -4271
EN 280   -5449
EN 300   -4520
EN 320   -4464
EN 340   -3524
EN 360   -5815
C-RIB:SER-CA
EN 20   -7641
EN 40   -5518
EN 60   -5379
EN 80   -3876
EN 100   -4885
EN 120   -5158
EN 140   -4755
EN 160   -3441
EN 180   -5044
EN 200   0
EN 220   -4921
EN 240   -4279
EN 260   -5061
EN 280   -2517
EN 300   -4183
EN 320   -5490
EN 340   -5065
EN 360   -7648

C-P:CYS-S1
EN 20 0
EN 40 0
EN 60 -3443
EN 80 -5451
EN 100 0
EN 120 -2808
EN 140 0
EN 160 0
EN 180 0
EN 200 0
EN 220 0
EN 240 -3463
EN 260 0
EN 280 -2535
EN 300 -4462
EN 320 0
EN 340 0
EN 360 -6209
A-P:GLN-S1
EN 20 0
EN 40 -2301
EN 60 -3381
EN 80 -5651
EN 100 -4904
EN 120 -2747
EN 140 -5354
EN 160 -3950
EN 180 0
EN 200 0
EN 220 -4878
EN 240 -3402
EN 260 -3363
EN 280 -3186
EN 300 -4783
EN 320 -5509
EN 340 -5574
EN 360 0
G-RIB:ASN-S2
EN 20 -6360
EN 40 -5955
EN 60 -6032
EN 80 -5102
EN 100 -5124
EN 120 -2675
EN 140 -5689
EN 160 -5264
EN 180 -5481
EN 200 -6615
EN 220 -4806
EN 240 -5339
EN 260 -4848
EN 280 -4119
EN 300 -5532
EN 320 -1290
EN 340 -4789
EN 360 -2641
U-RIB:LEU-S2

EN 20   0
EN 40   0
EN 60   0
EN 80   -4656
EN 100   -1026
EN 120   -3399
EN 140   -2520
EN 160   218
EN 180   -2097
EN 200   0
EN 220   -708
EN 240   766
EN 260   -198
EN 280   -1739
EN 300   -1236
EN 320   -2345
EN 340   -1404
EN 360   -1979
U31-MY:PHE-CA
EN 20   0
EN 40   0
EN 60   0
EN 80   0
EN 100   0
EN 120   0
EN 140   0
EN 160   0
EN 180   0
EN 200   0
EN 220   0
EN 240   0
EN 260   0
EN 280   0
EN 300   -17840
EN 320   0
EN 340   0
EN 360   0
C-Y:THR-S1
EN 20   -4445
EN 40   -5758
EN 60   -4117
EN 80   -4116
EN 100   -2757
EN 120   -760
EN 140   -2985
EN 160   -1963
EN 180   -4279
EN 200   -7422
EN 220   -5161
EN 240   -5403
EN 260   -2933
EN 280   -2204
EN 300   1020
EN 320   -2097
EN 340   -4140
EN 360   -4161
G-RIB:MET-S1
EN 20   0

EN 40   -2644
EN 60   -1002
EN 80   -2006
EN 100   -1812
EN 120   -368
EN 140   -3598
EN 160   -3288
EN 180   -6609
EN 200   -6026
EN 220   -2499
EN 240   -2740
EN 260   -2993
EN 280   -2816
EN 300   -3026
EN 320   -5139
EN 340   -1477
EN 360   -7756
A-R5:GLU-CA
EN 20   -5125
EN 40   -3003
EN 60   0
EN 80   -2366
EN 100   550
EN 120   -3157
EN 140   -2501
EN 160   791
EN 180   -1525
EN 200   0
EN 220   -3571
EN 240   1339
EN 260   2091
EN 280   -1167
EN 300   -664
EN 320   -1772
EN 340   0
EN 360   -1406
U-P:TYR-S2
EN 20   0
EN 40   0
EN 60   -4395
EN 80   -4394
EN 100   -4200
EN 120   -2755
EN 140   -3263
EN 160   -3959
EN 180   0
EN 200   0
EN 220   -3169
EN 240   -1693
EN 260   -4929
EN 280   -765
EN 300   -4962
EN 320   -1370
EN 340   -3865
EN 360   0
A-R5:GLN-S2
EN 20   -5456
EN 40   -6317

EN 60   -5509
EN 80   -4795
EN 100   -4601
EN 120   -4492
EN 140   -2278
EN 160   -3978
EN 180   0
EN 200   -7428
EN 220   -5618
EN 240   -4696
EN 260   -5108
EN 280   -3214
EN 300   -2711
EN 320   -4824
EN 340   -2880
EN 360   -5171
C-P:VAL-CA
EN 20   0
EN 40   -373
EN 60   -2167
EN 80   -4374
EN 100   -4179
EN 120   -4254
EN 140   -3758
EN 160   -1018
EN 180   -3334
EN 200   0
EN 220   -228
EN 240   -4197
EN 260   -3153
EN 280   -3529
EN 300   -3186
EN 320   -4135
EN 340   -4651
EN 360   -1498
A-P:ARG-CA
EN 20   0
EN 40   -2444
EN 60   -3525
EN 80   -4727
EN 100   -5197
EN 120   -6426
EN 140   -5828
EN 160   -5098
EN 180   -5958
EN 200   0
EN 220   -3303
EN 240   -4258
EN 260   -4295
EN 280   -5197
EN 300   -6553
EN 320   -6078
EN 340   -6270
EN 360   -6291
U-RIB:LEU-S1
EN 20   0
EN 40   0
EN 60   -3197

EN 80   -1218
EN 100   1698
EN 120   -2562
EN 140   -800
EN 160   -1495
EN 180   -2094
EN 200   0
EN 220   0
EN 240   0
EN 260   -1912
EN 280   -1023
EN 300   -2950
EN 320   -2341
EN 340   -2406
EN 360   -1975
A-P:ASP-S2
EN 20   0
EN 40   -4457
EN 60   -2816
EN 80   -2655
EN 100   -3625
EN 120   -4190
EN 140   -4406
EN 160   -4771
EN 180   0
EN 200   0
EN 220   0
EN 240   -3389
EN 260   -3471
EN 280   -3294
EN 300   -3122
EN 320   -3900
EN 340   -2578
EN 360   -3864
G-P:LYS-S2
EN 20   -3292
EN 40   -165
EN 60   -5819
EN 80   -6757
EN 100   -7102
EN 120   -6698
EN 140   -6271
EN 160   -7165
EN 180   -7113
EN 200   0
EN 220   -3454
EN 240   -5967
EN 260   -5666
EN 280   -6562
EN 300   -6334
EN 320   -6477
EN 340   -6767
EN 360   -6442
G-P:ILE-S1
EN 20   0
EN 40   -686
EN 60   -2479
EN 80   -1053

EN 100   -2124
EN 120   -4115
EN 140   -2905
EN 160   -1330
EN 180   0
EN 200   0
EN 220   -2258
EN 240   -2499
EN 260   -1747
EN 280   -1571
EN 300   -1781
EN 320   -2177
EN 340   -1236
EN 360   -1810
G-R6:PHE-CA
EN 20   0
EN 40   -1615
EN 60   -1692
EN 80   -1983
EN 100   0
EN 120   660
EN 140   -1565
EN 160   0
EN 180   0
EN 200   0
EN 220   0
EN 240   -1712
EN 260   -960
EN 280   -1788
EN 300   -662
EN 320   0
EN 340   0
EN 360   0
C-Y:ASN-CA
EN 20   0
EN 40   -1898
EN 60   -5409
EN 80   -2978
EN 100   -2071
EN 120   -1340
EN 140   -2852
EN 160   0
EN 180   -3142
EN 200   -6998
EN 220   0
EN 240   -2999
EN 260   -2960
EN 280   -2784
EN 300   -1276
EN 320   -2677
EN 340   -732
EN 360   -4741
U-P:ASP-CA
EN 20   0
EN 40   -1744
EN 60   -4091
EN 80   610
EN 100   -1917

EN 120   -4912
EN 140   -4415
EN 160   -4106
EN 180   -4705
EN 200   0
EN 220   0
EN 240   -123
EN 260   -2093
EN 280   -912
EN 300   -2127
EN 320   -2522
EN 340   -5399
EN 360   -2869
G-P:MET-S2
EN 20   -5809
EN 40   0
EN 60   -3763
EN 80   -4315
EN 100   -1850
EN 120   -406
EN 140   -914
EN 160   -3326
EN 180   0
EN 200   0
EN 220   -4254
EN 240   -2778
EN 260   -2026
EN 280   -132
EN 300   -3064
EN 320   -2455
EN 340   0
EN 360   0
C-P:LEU-S2
EN 20   0
EN 40   60
EN 60   -3450
EN 80   -2406
EN 100   -1830
EN 120   -2485
EN 140   -2993
EN 160   -2301
EN 180   0
EN 200   0
EN 220   -1512
EN 240   -1753
EN 260   -1554
EN 280   -2211
EN 300   -4310
EN 320   -718
EN 340   -1495
EN 360   -2782
U-P:PHE-S2
EN 20   0
EN 40   -2951
EN 60   -3027
EN 80   0
EN 100   -3124
EN 120   -4663

EN 140   -1183
EN 160   -1878
EN 180   0
EN 200   0
EN 220   -5528
EN 240   -7035
EN 260   -2295
EN 280   -402
EN 300   -4046
EN 320   0
EN 340   0
EN 360   -4076
A-RIB:TRP-S1
EN 20   0
EN 40   -4736
EN 60   0
EN 80   0
EN 100   -3904
EN 120   -4177
EN 140   -6403
EN 160   -3663
EN 180   -5979
EN 200   0
EN 220   0
EN 240   0
EN 260   -4080
EN 280   -3904
EN 300   -4114
EN 320   -4510
EN 340   -6292
EN 360   0
A-R6:TYR-S1
EN 20   0
EN 40   0
EN 60   -5336
EN 80   -5005
EN 100   10
EN 120   -3697
EN 140   -770
EN 160   -4187
EN 180   -3781
EN 200   0
EN 220   0
EN 240   -4905
EN 260   -2887
EN 280   10
EN 300   -2921
EN 320   -4029
EN 340   -659
EN 360   -3663
U-RIB:ASN-S1
EN 20   -7666
EN 40   0
EN 60   -6333
EN 80   -3902
EN 100   -4261
EN 120   -4986
EN 140   -3776

EN 160   -5184
EN 180   -8053
EN 200   0
EN 220   -5399
EN 240   -5641
EN 260   -4437
EN 280   -5094
EN 300   -4922
EN 320   -4313
EN 340   -7100
EN 360   -7382
U-P:ALA-CA
EN 20   0
EN 40   -1003
EN 60   -3801
EN 80   -3800
EN 100   -4159
EN 120   -2715
EN 140   -5176
EN 160   -5374
EN 180   -5681
EN 200   0
EN 220   -3580
EN 240   -1099
EN 260   -3782
EN 280   -3606
EN 300   -4605
EN 320   -4211
EN 340   -4276
EN 360   -3845
U-P:PRO-CA
EN 20   0
EN 40   -2446
EN 60   -805
EN 80   -4079
EN 100   -5602
EN 120   -4610
EN 140   -3400
EN 160   -6195
EN 180   -7125
EN 200   0
EN 220   -2301
EN 240   -4813
EN 260   -73
EN 280   -4337
EN 300   -3542
EN 320   -2220
EN 340   -5719
EN 360   0
C-RIB:THR-CA
EN 20   -4445
EN 40   -1319
EN 60   -4830
EN 80   -3785
EN 100   -2757
EN 120   -5752
EN 140   -2985
EN 160   -3681

EN 180   -6550
EN 200   -4700
EN 220   -3895
EN 240   -4137
EN 260   -4651
EN 280   -4213
EN 300   -4684
EN 320   -4527
EN 340   -5304
EN 360   -5878
G-R6:MET-S2
EN 20   0
EN 40   -4372
EN 60   -3736
EN 80   -3022
EN 100   -1823
EN 120   -3101
EN 140   0
EN 160   0
EN 180   -3898
EN 200   0
EN 220   0
EN 240   -5022
EN 260   -4270
EN 280   -2827
EN 300   -315
EN 320   -3433
EN 340   -1488
EN 360   0
C-P:LEU-CA
EN 20   0
EN 40   -1654
EN 60   -2283
EN 80   -3025
EN 100   -1827
EN 120   -3817
EN 140   -2156
EN 160   -581
EN 180   0
EN 200   0
EN 220   -1509
EN 240   -1037
EN 260   -2716
EN 280   -2831
EN 300   -2036
EN 320   -1427
EN 340   1229
EN 360   -2779
C-Y:TRP-S1
EN 20   0
EN 40   -6297
EN 60   -2938
EN 80   -1220
EN 100   -3747
EN 120   -3569
EN 140   0
EN 160   -3506
EN 180   0

EN 200   -9678
EN 220   0
EN 240   0
EN 260   -2206
EN 280   -2030
EN 300   0
EN 320   -2635
EN 340   -3413
EN 360   0
G-P:THR-S1
EN 20   -4069
EN 40   0
EN 60   -2736
EN 80   -3027
EN 100   -3545
EN 120   -3106
EN 140   -4327
EN 160   -4691
EN 180   -5621
EN 200   -4324
EN 220   0
EN 240   -3761
EN 260   -4511
EN 280   -2381
EN 300   -4047
EN 320   -3820
EN 340   -5481
EN 360   -4789
U-P:TRP-CA
EN 20   0
EN 40   0
EN 60   0
EN 80   -5875
EN 100   -4676
EN 120   -5954
EN 140   -6462
EN 160   -4435
EN 180   -8469
EN 200   0
EN 220   0
EN 240   0
EN 260   -3135
EN 280   -5680
EN 300   -6603
EN 320   0
EN 340   -4341
EN 360   0
G-R6:CYS-S1
EN 20   0
EN 40   0
EN 60   0
EN 80   0
EN 100   0
EN 120   0
EN 140   -4620
EN 160   -3598
EN 180   0
EN 200   0

EN 220   0
EN 240   -3050
EN 260   0
EN 280   0
EN 300   -2331
EN 320   0
EN 340   0
EN 360   0
C-P:SER-S1
EN 20   0
EN 40   -5910
EN 60   -2169
EN 80   -5603
EN 100   -3692
EN 120   -5522
EN 140   -5262
EN 160   -4837
EN 180   -5767
EN 200   0
EN 220   -4931
EN 240   -4620
EN 260   -5585
EN 280   -5962
EN 300   -4906
EN 320   -4589
EN 340   -6792
EN 360   -6653
U-P:PHE-CA
EN 20   0
EN 40   0
EN 60   -3023
EN 80   -2310
EN 100   0
EN 120   -3393
EN 140   -3901
EN 160   -1874
EN 180   -4191
EN 200   0
EN 220   -2802
EN 240   -3044
EN 260   -3296
EN 280   -3833
EN 300   0
EN 320   -2721
EN 340   0
EN 360   -4072
G-R5:PHE-S1
EN 20   -4742
EN 40   0
EN 60   25
EN 80   738
EN 100   -784
EN 120   0
EN 140   152
EN 160   -543
EN 180   0
EN 200   0
EN 220   -1470

EN 240   -4434
EN 260   -2677
EN 280   1646
EN 300   -993
EN 320   327
EN 340   0
EN 360   0
QUO-M6:ARG-S1
EN 20   0
EN 40   0
EN 60   0
EN 80   0
EN 100   0
EN 120   -16452
EN 140   0
EN 160   0
EN 180   -19971
EN 200   0
EN 220   0
EN 240   0
EN 260   0
EN 280   0
EN 300   0
EN 320   0
EN 340   0
EN 360   0
A-R5:GLU-S2
EN 20   0
EN 40   -5142
EN 60   -3831
EN 80   -1953
EN 100   -1206
EN 120   237
EN 140   -2992
EN 160   -965
EN 180   -1564
EN 200   -3702
EN 220   -2897
EN 240   -2687
EN 260   -3392
EN 280   -2210
EN 300   -3133
EN 320   -1099
EN 340   -871
EN 360   -1445
C-RIB:TRP-S2
EN 20   0
EN 40   -4579
EN 60   0
EN 80   -6212
EN 100   -4752
EN 120   -2303
EN 140   0
EN 160   -5224
EN 180   0
EN 200   0
EN 220   0
EN 240   -5680

EN 260   -2206
EN 280   -4752
EN 300   -2240
EN 320   -4353
EN 340   -3413
EN 360   -7422
G-RIB:VAL-CA
EN 20   0
EN 40   0
EN 60   -37
EN 80   -1040
EN 100   -133
EN 120   -2506
EN 140   -2180
EN 160   -2322
EN 180   0
EN 200   0
EN 220   -1533
EN 240   -57
EN 260   -1022
EN 280   -846
EN 300   -2060
EN 320   -2456
EN 340   -1516
EN 360   -2803
GTP-M6:ASN-S1
EN 20   0
EN 40   -20059
EN 60   0
EN 80   0
EN 100   0
EN 120   0
EN 140   0
EN 160   0
EN 180   0
EN 200   0
EN 220   0
EN 240   0
EN 260   0
EN 280   0
EN 300   0
EN 320   0
EN 340   0
EN 360   0
G-R6:GLN-S1
EN 20   0
EN 40   -5726
EN 60   -4085
EN 80   -5088
EN 100   -1911
EN 120   -4283
EN 140   0
EN 160   -665
EN 180   0
EN 200   0
EN 220   -6414
EN 240   -2839
EN 260   -5306

EN 280 -3628
EN 300 -2121
EN 320 -1512
EN 340 0
EN 360 0
G-RIB:ILE-CA
EN 20 0
EN 40 -658
EN 60 0
EN 80 -2291
EN 100 -2931
EN 120 -3535
EN 140 -3330
EN 160 -1303
EN 180 0
EN 200 0
EN 220 -513
EN 240 0
EN 260 -1720
EN 280 -1544
EN 300 -36
EN 320 -2702
EN 340 507
EN 360 0
U-Y:ASN-CA
EN 20 0
EN 40 -2822
EN 60 -5169
EN 80 -2185
EN 100 -5425
EN 120 -3268
EN 140 -4489
EN 160 -1749
EN 180 0
EN 200 0
EN 220 -4394
EN 240 -3923
EN 260 -5271
EN 280 -4090
EN 300 -2200
EN 320 -4866
EN 340 -4378
EN 360 0
A-R5:SER-CA
EN 20 -4362
EN 40 -4671
EN 60 -4416
EN 80 -2869
EN 100 1313
EN 120 -2394
EN 140 -4289
EN 160 -2885
EN 180 -5914
EN 200 0
EN 220 -3813
EN 240 -4054
EN 260 -580
EN 280 -3508

EN 300   -1618
EN 320   -2727
EN 340   -2791
EN 360   -4078
G-RIB:PHE-CA
EN 20   0
EN 40   -3333
EN 60   -1692
EN 80   -2695
EN 100   -3506
EN 120   -3779
EN 140   -1565
EN 160   -2260
EN 180   0
EN 200   0
EN 220   0
EN 240   -2717
EN 260   -960
EN 280   -3054
EN 300   723
EN 320   -2394
EN 340   -3171
EN 360   -5462
G-R5:GLU-S2
EN 20   -4605
EN 40   -1478
EN 60   -3271
EN 80   -3111
EN 100   66
EN 120   -1472
EN 140   -3145
EN 160   -405
EN 180   -1004
EN 200   -3142
EN 220   -2337
EN 240   -3292
EN 260   -2461
EN 280   -486
EN 300   -1282
EN 320   -1251
EN 340   -1316
EN 360   0
C-Y:PRO-CA
EN 20   0
EN 40   -6218
EN 60   -4698
EN 80   -3151
EN 100   -3408
EN 120   -2677
EN 140   -1467
EN 160   -3880
EN 180   0
EN 200   -8335
EN 220   -3090
EN 240   -3885
EN 260   854
EN 280   -2956
EN 300   -3166

EN 320   -1291
EN 340   -351
EN 360   0
G-R5:HIS-S2
EN 20   -8576
EN 40   -4445
EN 60   -7191
EN 80   -6314
EN 100   -5000
EN 120   -5273
EN 140   -960
EN 160   -3372
EN 180   0
EN 200   0
EN 220   -7022
EN 240   -5838
EN 260   -3789
EN 280   -5883
EN 300   -3110
EN 320   0
EN 340   -4283
EN 360   0
U-P:GLY-CA
EN 20   -4518
EN 40   -5830
EN 60   -5455
EN 80   -4188
EN 100   -2829
EN 120   -4820
EN 140   -7282
EN 160   -6673
EN 180   -5356
EN 200   -6490
EN 220   -6067
EN 240   -3205
EN 260   -5728
EN 280   -5861
EN 300   -5761
EN 320   -2882
EN 340   -6381
EN 360   -2516
U-Y:ASP-S1
EN 20   -4868
EN 40   -3459
EN 60   -2822
EN 80   -2821
EN 100   807
EN 120   -1183
EN 140   -3961
EN 160   -669
EN 180   0
EN 200   0
EN 220   -3314
EN 240   -4108
EN 260   -2091
EN 280   0
EN 300   -1119
EN 320   0

EN 340   0
EN 360   -2867
U-P:MET-S1
EN 20   0
EN 40   -5693
EN 60   -2334
EN 80   -3338
EN 100   -4861
EN 120   -3417
EN 140   0
EN 160   -2903
EN 180   -5219
EN 200   0
EN 220   -5548
EN 240   -5077
EN 260   -1603
EN 280   -3144
EN 300   -3353
EN 320   -4754
EN 340   -4527
EN 360   0
U-P:ASP-S2
EN 20   0
EN 40   0
EN 60   -150
EN 80   -2158
EN 100   -3230
EN 120   -5457
EN 140   -3458
EN 160   -3441
EN 180   -4752
EN 200   0
EN 220   -5081
EN 240   -1888
EN 260   -3406
EN 280   -3230
EN 300   -2887
EN 320   -3835
EN 340   -5777
EN 360   -5638
G-RIB:TRP-CA
EN 20   0
EN 40   -5893
EN 60   0
EN 80   -3538
EN 100   -3344
EN 120   0
EN 140   -4125
EN 160   -5825
EN 180   0
EN 200   0
EN 220   0
EN 240   -5277
EN 260   -3520
EN 280   -5614
EN 300   -4558
EN 320   -3950
EN 340   0

EN 360 0
U-RIB:THR-CA
EN 20 0
EN 40 -3960
EN 60 -4036
EN 80 -5040
EN 100 -5137
EN 120 -3954
EN 140 -5919
EN 160 -4604
EN 180 0
EN 200 -5624
EN 220 -4819
EN 240 -2339
EN 260 -3304
EN 280 -2415
EN 300 -5055
EN 320 -3734
EN 340 -1076
EN 360 -3367
U-Y:MET-S2
EN 20 0
EN 40 -5699
EN 60 -2341
EN 80 -1627
EN 100 -1433
EN 120 -4428
EN 140 -2214
EN 160 0
EN 180 0
EN 200 0
EN 220 -3837
EN 240 -4078
EN 260 -1609
EN 280 -3150
EN 300 0
EN 320 -2038
EN 340 -2815
EN 360 0
U-RIB:ASP-S1
EN 20 0
EN 40 -1742
EN 60 -2822
EN 80 -1104
EN 100 -4345
EN 120 -3905
EN 140 -3408
EN 160 -3391
EN 180 -4702
EN 200 0
EN 220 -3314
EN 240 -1838
EN 260 630
EN 280 -910
EN 300 -2124
EN 320 -3785
EN 340 -4563
EN 360 -2867

G-R5:PHE-CA
EN 20   -7464
EN 40   -1615
EN 60   -1692
EN 80   -1983
EN 100   -784
EN 120   0
EN 140   0
EN 160   -543
EN 180   0
EN 200   -4997
EN 220   -1470
EN 240   -2717
EN 260   -1965
EN 280   933
EN 300   0
EN 320   327
EN 340   0
EN 360   0
C-RIB:VAL-CA
EN 20   0
EN 40   -2081
EN 60   -1444
EN 80   -3714
EN 100   -2636
EN 120   -4244
EN 140   -4752
EN 160   -3730
EN 180   -1607
EN 200   0
EN 220   -1936
EN 240   -1465
EN 260   -4700
EN 280   -3258
EN 300   -2464
EN 320   -4125
EN 340   -4019
EN 360   0
A-P:HIS-S1
EN 20   0
EN 40   -3273
EN 60   -6453
EN 80   -4906
EN 100   -5545
EN 120   -3719
EN 140   -5945
EN 160   -4922
EN 180   -6234
EN 200   0
EN 220   -4845
EN 240   -4374
EN 260   -2618
EN 280   -5876
EN 300   -2651
EN 320   -4051
EN 340   -6094
EN 360   -6116
U-RIB:ALA-CA

EN 20 -6847
EN 40 -2716
EN 60 -4178
EN 80 -4087
EN 100 -3601
EN 120 -2710
EN 140 -4052
EN 160 -3360
EN 180 -2242
EN 200 0
EN 220 -2570
EN 240 -2812
EN 260 -3778
EN 280 -3893
EN 300 -3480
EN 320 -3876
EN 340 -3267
EN 360 -3841
G-R5:ILE-S1
EN 20 0
EN 40 -2376
EN 60 982
EN 80 2408
EN 100 172
EN 120 -100
EN 140 1109
EN 160 0
EN 180 0
EN 200 -4040
EN 220 -513
EN 240 961
EN 260 -3
EN 280 1890
EN 300 -36
EN 320 0
EN 340 0
EN 360 0
U-RIB:SER-S1
EN 20 -5130
EN 40 -3720
EN 60 -5183
EN 80 -5353
EN 100 -4606
EN 120 -4166
EN 140 -4223
EN 160 -2647
EN 180 -4964
EN 200 -7102
EN 220 -1858
EN 240 -4821
EN 260 -4451
EN 280 -4898
EN 300 -2385
EN 320 -4047
EN 340 -5276
EN 360 -3128
C-RIB:LYS-S2
EN 20 -9609

EN 40   -8884
EN 60   -5920
EN 80   -5482
EN 100   -5012
EN 120   -5924
EN 140   -4478
EN 160   -5173
EN 180   -6606
EN 200   -8362
EN 220   -8270
EN 240   -4861
EN 260   -5330
EN 280   -5153
EN 300   -5860
EN 320   -4538
EN 340   -4196
EN 360   -4388
G-R6:PRO-S1
EN 20   0
EN 40   -2832
EN 60   -4295
EN 80   -2195
EN 100   -1287
EN 120   -2826
EN 140   -1064
EN 160   -1759
EN 180   0
EN 200   0
EN 220   -4957
EN 240   -2929
EN 260   -4116
EN 280   -2553
EN 300   -492
EN 320   -2606
EN 340   -2670
EN 360   0
G-R5:ARG-CA
EN 20   -3291
EN 40   -4985
EN 60   -2510
EN 80   -1244
EN 100   -2054
EN 120   -1323
EN 140   -2835
EN 160   908
EN 180   -4842
EN 200   -6268
EN 220   -5463
EN 240   -2531
EN 260   -3496
EN 280   -2767
EN 300   -2646
EN 320   -3925
EN 340   -3437
EN 360   -1289
A-RIB:MET-S2
EN 20   0
EN 40   -3215

EN 60   -1573
EN 80   -860
EN 100   -4653
EN 120   -2656
EN 140   -3164
EN 160   -3859
EN 180   0
EN 200   0
EN 220   -3070
EN 240   -1594
EN 260   -3564
EN 280   -2383
EN 300   -3597
EN 320   -1271
EN 340   -3766
EN 360   0
C-RIB:VAL-S1
EN 20   0
EN 40   -2083
EN 60   -2159
EN 80   -3455
EN 100   -3521
EN 120   -3795
EN 140   -4042
EN 160   -4445
EN 180   0
EN 200   0
EN 220   0
EN 240   -3184
EN 260   -4531
EN 280   -2638
EN 300   -3178
EN 320   -3866
EN 340   -2634
EN 360   -1490
A-R6:MET-CA
EN 20   0
EN 40   0
EN 60   -3280
EN 80   -2566
EN 100   -4642
EN 120   -3650
EN 140   -4158
EN 160   0
EN 180   -4447
EN 200   0
EN 220   0
EN 240   -1583
EN 260   -831
EN 280   -654
EN 300   -4299
EN 320   -3982
EN 340   -2037
EN 360   -4329
U-RIB:GLN-CA
EN 20   0
EN 40   -4783
EN 60   -3141

EN 80 -710
EN 100 -4504
EN 120 -2507
EN 140 -3015
EN 160 -5427
EN 180 -4309
EN 200 0
EN 220 -4637
EN 240 -4166
EN 260 -692
EN 280 -5337
EN 300 -5165
EN 320 -2839
EN 340 -4621
EN 360 -5908
U-Y:GLU-S1
EN 20 -4182
EN 40 -5043
EN 60 -1131
EN 80 -418
EN 100 -223
EN 120 0
EN 140 712
EN 160 -1700
EN 180 -2298
EN 200 0
EN 220 -910
EN 240 -1152
EN 260 1317
EN 280 -223
EN 300 -433
EN 320 0
EN 340 110
EN 360 0
C-RIB:ASP-CA
EN 20 0
EN 40 -815
EN 60 -3614
EN 80 -1183
EN 100 -4621
EN 120 -4696
EN 140 -3035
EN 160 -1460
EN 180 -5494
EN 200 0
EN 220 -670
EN 240 -912
EN 260 -3887
EN 280 -2705
EN 300 -4181
EN 320 -3693
EN 340 -5093
EN 360 -5928
C-RIB:CYS-CA
EN 20 0
EN 40 -5074
EN 60 0
EN 80 -2719

EN 100   -2525
EN 120   0
EN 140   -3306
EN 160   -4001
EN 180   0
EN 200   0
EN 220   0
EN 240   0
EN 260   -2701
EN 280   -5247
EN 300   -2735
EN 320   -3130
EN 340   -3908
EN 360   0
G-R5:MET-S1
EN 20   -5770
EN 40   0
EN 60   -3725
EN 80   -4728
EN 100   0
EN 120   -368
EN 140   0
EN 160   0
EN 180   0
EN 200   0
EN 220   -4216
EN 240   0
EN 260   -4259
EN 280   -94
EN 300   -2022
EN 320   -3422
EN 340   -1477
EN 360   0
G-P:THR-CA
EN 20   0
EN 40   -4377
EN 60   698
EN 80   -3027
EN 100   -3215
EN 120   -2101
EN 140   -5331
EN 160   -5314
EN 180   -6173
EN 200   0
EN 220   -3519
EN 240   -1039
EN 260   -3722
EN 280   -3214
EN 300   -3042
EN 320   -4443
EN 340   -4928
EN 360   -3785
U-Y:SER-S1
EN 20   -6847
EN 40   -6442
EN 60   -3796
EN 80   -4348
EN 100   -3441

EN 120 -2449
EN 140 -1952
EN 160 -930
EN 180 -3246
EN 200 -5385
EN 220 -7010
EN 240 -3104
EN 260 -2849
EN 280 546
EN 300 -3651
EN 320 -3494
EN 340 -4824
EN 360 0
H2U-RIB:GLU-S2
EN 20 0
EN 40 0
EN 60 0
EN 80 0
EN 100 0
EN 120 0
EN 140 0
EN 160 0
EN 180 0
EN 200 0
EN 220 0
EN 240 0
EN 260 0
EN 280 -11788
EN 300 0
EN 320 0
EN 340 0
EN 360 0
U-Y:HIS-CA
EN 20 -7164
EN 40 -6760
EN 60 -4114
EN 80 0
EN 100 -1488
EN 120 -1762
EN 140 -3987
EN 160 -4682
EN 180 0
EN 200 0
EN 220 -5610
EN 240 -2417
EN 260 0
EN 280 -3206
EN 300 -3416
EN 320 -3811
EN 340 0
EN 360 0
U31-P:MET-S2
EN 20 0
EN 40 0
EN 60 0
EN 80 0
EN 100 0
EN 120 0

EN 140   -19451
EN 160   0
EN 180   0
EN 200   0
EN 220   0
EN 240   -19598
EN 260   0
EN 280   0
EN 300   0
EN 320   0
EN 340   0
EN 360   0
G-P:TRP-CA
EN 20   0
EN 40   -5920
EN 60   -4279
EN 80   -3566
EN 100   -4376
EN 120   -1927
EN 140   0
EN 160   -5852
EN 180   0
EN 200   0
EN 220   0
EN 240   -2582
EN 260   -1830
EN 280   -1654
EN 300   -4586
EN 320   -5694
EN 340   0
EN 360   -7045
U-RIB:GLU-S1
EN 20   0
EN 40   -1055
EN 60   585
EN 80   -3140
EN 100   -1228
EN 120   -496
EN 140   712
EN 160   -1700
EN 180   0
EN 200   -4437
EN 220   0
EN 240   -1152
EN 260   -400
EN 280   -223
EN 300   -2150
EN 320   888
EN 340   -2611
EN 360   -2180
C-RIB:TYR-S1
EN 20   0
EN 40   0
EN 60   -740
EN 80   -1744
EN 100   -1549
EN 120   -4093
EN 140   -2331

EN 160   -1308
EN 180   0
EN 200   0
EN 220   -2236
EN 240   0
EN 260   -3996
EN 280   -4653
EN 300   -5486
EN 320   -2155
EN 340   -5203
EN 360   -6941
A-RIB:LEU-CA
EN 20   0
EN 40   -3518
EN 60   1557
EN 80   2271
EN 100   -1522
EN 120   -2959
EN 140   -2303
EN 160   -2998
EN 180   -4049
EN 200   0
EN 220   61
EN 240   0
EN 260   -1698
EN 280   -256
EN 300   -2565
EN 320   -2961
EN 340   -1639
EN 360   -2926
C-P:TYR-S2
EN 20   0
EN 40   0
EN 60   -4742
EN 80   -4480
EN 100   -3834
EN 120   -5271
EN 140   -627
EN 160   -3040
EN 180   -3639
EN 200   0
EN 220   -2250
EN 240   -2492
EN 260   -3457
EN 280   -3834
EN 300   -2778
EN 320   -4891
EN 340   -2946
EN 360   0
A-RIB:ALA-S1
EN 20   0
EN 40   -2953
EN 60   -4531
EN 80   -2698
EN 100   -3839
EN 120   -1390
EN 140   -2902
EN 160   -2593

EN 180 0
EN 200 -3613
EN 220 -4525
EN 240 -3050
EN 260 -3563
EN 280 -2834
EN 300 -2713
EN 320 -4229
EN 340 -782
EN 360 -3073
A-P:LYS-S2
EN 20 -5545
EN 40 -4135
EN 60 -4211
EN 80 -6220
EN 100 -6127
EN 120 -5968
EN 140 -5952
EN 160 -7286
EN 180 -7096
EN 200 0
EN 220 -2273
EN 240 -5618
EN 260 -4683
EN 280 -6224
EN 300 -5656
EN 320 -6829
EN 340 -5691
EN 360 -5813
A-RIB:GLN-S1
EN 20 0
EN 40 -5020
EN 60 -4092
EN 80 -3378
EN 100 -4570
EN 120 -3457
EN 140 -5351
EN 160 -1225
EN 180 -6263
EN 200 -5680
EN 220 0
EN 240 -5117
EN 260 -4365
EN 280 -5690
EN 300 -4398
EN 320 -2072
EN 340 -2849
EN 360 -5140
A-P:PHE-S2
EN 20 0
EN 40 -3899
EN 60 -3262
EN 80 -3262
EN 100 -2354
EN 120 -1623
EN 140 -2131
EN 160 -1109
EN 180 -3425

EN 200   0
EN 220   -3754
EN 240   -5000
EN 260   -3244
EN 280   -3067
EN 300   157
EN 320   -4226
EN 340   -2733
EN 360   0
C-P:MET-S2
EN 20   0
EN 40   -4785
EN 60   -5414
EN 80   -4701
EN 100   -2236
EN 120   -2509
EN 140   -4735
EN 160   0
EN 180   -4311
EN 200   0
EN 220   -2923
EN 240   -4882
EN 260   -4130
EN 280   -3953
EN 300   -728
EN 320   -1124
EN 340   -3619
EN 360   -5910
G-RIB:ILE-S1
EN 20   0
EN 40   -658
EN 60   0
EN 80   -2743
EN 100   -1544
EN 120   -1104
EN 140   -1612
EN 160   0
EN 180   0
EN 200   0
EN 220   -2231
EN 240   0
EN 260   -1720
EN 280   -2930
EN 300   1680
EN 320   -2702
EN 340   -1209
EN 360   -1783
G-RIB:ALA-CA
EN 20   -5520
EN 40   -2393
EN 60   -1465
EN 80   -3021
EN 100   -3063
EN 120   -3750
EN 140   -2724
EN 160   -3751
EN 180   -2632
EN 200   -3053

EN 220 -2248
EN 240 -2490
EN 260 -1738
EN 280 -1561
EN 300 -2776
EN 320 -2167
EN 340 -1227
EN 360 -4784
A-P:THR-S1
EN 20 -4605
EN 40 -4200
EN 60 -4989
EN 80 -3944
EN 100 -5468
EN 120 -5741
EN 140 -5154
EN 160 -4845
EN 180 -6156
EN 200 0
EN 220 -1333
EN 240 -5301
EN 260 -3544
EN 280 -4634
EN 300 -5295
EN 320 -3974
EN 340 -3033
EN 360 -2603
U-P:ILE-S1
EN 20 0
EN 40 0
EN 60 -2066
EN 80 364
EN 100 -1158
EN 120 -1432
EN 140 -222
EN 160 -3639
EN 180 0
EN 200 0
EN 220 -3562
EN 240 0
EN 260 -3052
EN 280 558
EN 300 -3638
EN 320 0
EN 340 -2541
EN 360 -3115
FHU-MY:TYR-S1
EN 20 0
EN 40 0
EN 60 0
EN 80 0
EN 100 -16276
EN 120 0
EN 140 -17057
EN 160 0
EN 180 0
EN 200 0
EN 220 0

EN 240   0
EN 260   0
EN 280   -16276
EN 300   0
EN 320   -16881
EN 340   0
EN 360   0
G-RIB:LYS-S2
EN 20   -8086
EN 40   -7007
EN 60   -7005
EN 80   -5326
EN 100   -6016
EN 120   -4401
EN 140   -5239
EN 160   -4217
EN 180   -1381
EN 200   -6242
EN 220   -7536
EN 240   -7464
EN 260   -6428
EN 280   -5845
EN 300   -4818
EN 320   -3346
EN 340   -4676
EN 360   -3985
C-Y:GLU-S2
EN 20   -3290
EN 40   -4603
EN 60   -4228
EN 80   -2248
EN 100   -2054
EN 120   -610
EN 140   -113
EN 160   -2526
EN 180   -3124
EN 200   -6268
EN 220   -2740
EN 240   -2982
EN 260   -2612
EN 280   -2054
EN 300   2175
EN 320   -2208
EN 340   -715
EN 360   -4011
C-P:GLN-CA
EN 20   0
EN 40   -2151
EN 60   -4498
EN 80   -4948
EN 100   -4041
EN 120   -4696
EN 140   -3105
EN 160   -4513
EN 180   -6116
EN 200   0
EN 220   -3723
EN 240   -3252

EN 260   -4218
EN 280   -5759
EN 300   -5968
EN 320   -5912
EN 340   -4972
EN 360   -4993
U-P:SER-CA
EN 20   0
EN 40   -2008
EN 60   -4354
EN 80   -4092
EN 100   -2893
EN 120   -1449
EN 140   -5945
EN 160   -5374
EN 180   -3251
EN 200   0
EN 220   -4584
EN 240   -5208
EN 260   -3069
EN 280   -5997
EN 300   -5609
EN 320   -6603
EN 340   -5281
EN 360   -3133
A-R5:THR-S1
EN 20   -7324
EN 40   -4197
EN 60   -4655
EN 80   -3942
EN 100   -4078
EN 120   -1921
EN 140   -3142
EN 160   -403
EN 180   -2719
EN 200   -6575
EN 220   -5770
EN 240   -3842
EN 260   -4255
EN 280   -643
EN 300   -1858
EN 320   -1249
EN 340   -3031
EN 360   0
DA-M6:LEU-S2
EN 20   0
EN 40   0
EN 60   0
EN 80   0
EN 100   -15541
EN 120   0
EN 140   0
EN 160   0
EN 180   0
EN 200   0
EN 220   0
EN 240   0
EN 260   0

EN 280 0
EN 300 -15751
EN 320 0
EN 340 0
EN 360 0
C-Y:TYR-S2
EN 20 0
EN 40 -4103
EN 60 -2462
EN 80 -3465
EN 100 -3824
EN 120 -1827
EN 140 -617
EN 160 -1313
EN 180 0
EN 200 0
EN 220 -3958
EN 240 -3487
EN 260 -2735
EN 280 -3271
EN 300 -2768
EN 320 -442
EN 340 -2936
EN 360 0
G-RIB:TYR-S2
EN 20 0
EN 40 -1982
EN 60 -3063
EN 80 -3615
EN 100 -3873
EN 120 -2428
EN 140 -3649
EN 160 -3632
EN 180 -4943
EN 200 0
EN 220 -1837
EN 240 -5805
EN 260 -4049
EN 280 -2868
EN 300 356
EN 320 -4026
EN 340 -816
EN 360 0
G-R6:ASP-S2
EN 20 -3586
EN 40 -3894
EN 60 -1540
EN 80 -2926
EN 100 -3063
EN 120 -3005
EN 140 -2552
EN 160 -3374
EN 180 -4425
EN 200 -5559
EN 220 -3749
EN 240 -3660
EN 260 -4028
EN 280 -4067

EN 300 -3825
EN 320 -2850
EN 340 -4114
EN 360 -3302
U-Y:ASN-S1
EN 20 -8671
EN 40 -2822
EN 60 -5169
EN 80 -3902
EN 100 -4712
EN 120 -4534
EN 140 -4489
EN 160 -3467
EN 180 0
EN 200 0
EN 220 -4394
EN 240 -6023
EN 260 -5601
EN 280 -4571
EN 300 -2200
EN 320 -4866
EN 340 -3373
EN 360 -5665
A-RIB:LEU-S2
EN 20 0
EN 40 -86
EN 60 -1880
EN 80 550
EN 100 -972
EN 120 -1245
EN 140 -1753
EN 160 -3453
EN 180 -1330
EN 200 0
EN 220 0
EN 240 1533
EN 260 -436
EN 280 -3478
EN 300 -469
EN 320 -3295
EN 340 -3359
EN 360 -3933
G-RIB:HIS-S2
EN 20 0
EN 40 -4445
EN 60 -6791
EN 80 -3807
EN 100 -4166
EN 120 -3886
EN 140 -5781
EN 160 -7360
EN 180 -3971
EN 200 -6109
EN 220 -5304
EN 240 -5094
EN 260 -4342
EN 280 -5622
EN 300 -5832

EN 320   -5936
EN 340   -4996
EN 360   -8292
G-P:PHE-S1
EN 20   0
EN 40   0
EN 60   -1719
EN 80   -3727
EN 100   -3081
EN 120   -1084
EN 140   -3309
EN 160   -4557
EN 180   0
EN 200   0
EN 220   0
EN 240   -4843
EN 260   -987
EN 280   -811
EN 300   -3291
EN 320   -2421
EN 340   -2193
EN 360   -2767
A-RIB:SER-S1
EN 20   -7084
EN 40   -5223
EN 60   -3029
EN 80   -5038
EN 100   -4628
EN 120   -2394
EN 140   -2902
EN 160   -3598
EN 180   -4196
EN 200   -4618
EN 220   -6535
EN 240   -4436
EN 260   -4307
EN 280   -4843
EN 300   -5766
EN 320   -4444
EN 340   -4057
EN 360   -5796
A-RIB:MET-S1
EN 20   0
EN 40   0
EN 60   0
EN 80   -4837
EN 100   -4642
EN 120   -3650
EN 140   -4158
EN 160   -2131
EN 180   0
EN 200   0
EN 220   -3059
EN 240   -3300
EN 260   -2548
EN 280   -654
EN 300   -4852
EN 320   -1260

EN 340 -4759
EN 360 -4329
A-P:PRO-CA
EN 20 0
EN 40 0
EN 60 -2758
EN 80 -3762
EN 100 -5285
EN 120 -3389
EN 140 -3897
EN 160 -4039
EN 180 -5643
EN 200 0
EN 220 -3249
EN 240 -4044
EN 260 -3744
EN 280 -4572
EN 300 -3777
EN 320 -5890
EN 340 -3946
EN 360 -2802
G-P:ARG-CA
EN 20 0
EN 40 -3626
EN 60 -3702
EN 80 -4856
EN 100 -5300
EN 120 -6258
EN 140 -5966
EN 160 -6414
EN 180 -4869
EN 200 0
EN 220 -46
EN 240 -4014
EN 260 -4357
EN 280 -5985
EN 300 -5013
EN 320 -6413
EN 340 -6683
EN 360 -6137
U-Y:MET-S1
EN 20 0
EN 40 -3971
EN 60 -5052
EN 80 0
EN 100 -1422
EN 120 0
EN 140 0
EN 160 0
EN 180 0
EN 200 -7353
EN 220 0
EN 240 -4067
EN 260 -1598
EN 280 -3139
EN 300 0
EN 320 -2027
EN 340 -2804

EN 360   0
A-RIB:VAL-S1
EN 20   0
EN 40   -2240
EN 60   -4033
EN 80   -889
EN 100   -2413
EN 120   -3068
EN 140   -3906
EN 160   -3437
EN 180   -3483
EN 200   0
EN 220   0
EN 240   -1623
EN 260   -1584
EN 280   -3914
EN 300   -1618
EN 320   -2566
EN 340   -3344
EN 360   -5082
G-P:ASN-S1
EN 20   0
EN 40   -3240
EN 60   -5822
EN 80   -5324
EN 100   -4125
EN 120   -4951
EN 140   -4575
EN 160   -6805
EN 180   -6753
EN 200   0
EN 220   -1377
EN 240   -3889
EN 260   -5504
EN 280   -4914
EN 300   -4888
EN 320   -5022
EN 340   -4795
EN 360   -5369
U-RIB:PHE-S2
EN 20   0
EN 40   -2946
EN 60   -1305
EN 80   -2309
EN 100   -5218
EN 120   -5492
EN 140   -4613
EN 160   -1873
EN 180   0
EN 200   0
EN 220   0
EN 240   -4047
EN 260   0
EN 280   -397
EN 300   -5046
EN 320   -4990
EN 340   -3497
EN 360   -5789

C-Y:GLN-S2
EN 20 -8021
EN 40 -7324
EN 60 -5975
EN 80 -5522
EN 100 -4444
EN 120 -1613
EN 140 -3126
EN 160 0
EN 180 0
EN 200 -5554
EN 220 -5462
EN 240 -7090
EN 260 -5243
EN 280 -4775
EN 300 -2555
EN 320 0
EN 340 -4440
EN 360 0
FHU-P:THR-CA
EN 20 0
EN 40 0
EN 60 -16121
EN 80 -15407
EN 100 0
EN 120 0
EN 140 0
EN 160 0
EN 180 0
EN 200 0
EN 220 0
EN 240 0
EN 260 -15389
EN 280 0
EN 300 0
EN 320 -15818
EN 340 0
EN 360 0
A-P:HIS-CA
EN 20 0
EN 40 -4991
EN 60 -6071
EN 80 -4906
EN 100 -4712
EN 120 -4432
EN 140 -5945
EN 160 -3918
EN 180 -6234
EN 200 0
EN 220 -3128
EN 240 -4374
EN 260 -2618
EN 280 -3446
EN 300 -4921
EN 320 -4051
EN 340 -3824
EN 360 -6116
C-Y:SER-S1

EN 20 -4206
EN 40 -5900
EN 60 -4882
EN 80 -5203
EN 100 -4235
EN 120 -2238
EN 140 -1028
EN 160 -6
EN 180 -2322
EN 200 -7896
EN 220 -6086
EN 240 -5399
EN 260 -4411
EN 280 -4686
EN 300 -3561
EN 320 -853
EN 340 -3347
EN 360 0
G-RIB:PRO-S1
EN 20 -4241
EN 40 -2832
EN 60 -5415
EN 80 -4701
EN 100 -5104
EN 120 -3991
EN 140 -5052
EN 160 -4863
EN 180 -2358
EN 200 -4496
EN 220 -969
EN 240 -4315
EN 260 -3563
EN 280 -3387
EN 300 -4219
EN 320 -3610
EN 340 -4388
EN 360 -4962
C-P:GLY-CA
EN 20 0
EN 40 -5916
EN 60 -3652
EN 80 -4472
EN 100 -6179
EN 120 -4906
EN 140 -6127
EN 160 -6269
EN 180 -5703
EN 200 0
EN 220 -5771
EN 240 -5871
EN 260 -4638
EN 280 -5581
EN 300 -5294
EN 320 -6504
EN 340 -6015
EN 360 -5032
U-RIB:ILE-CA
EN 20 0

EN 40 0
EN 60 0
EN 80 -2353
EN 100 -1154
EN 120 -3697
EN 140 -3652
EN 160 -913
EN 180 0
EN 200 0
EN 220 -1840
EN 240 0
EN 260 386
EN 280 -2871
EN 300 353
EN 320 -2764
EN 340 -819
EN 360 -3110
U-P:ALA-S1
EN 20 0
EN 40 -1003
EN 60 -2797
EN 80 -4805
EN 100 -3275
EN 120 -2162
EN 140 -4387
EN 160 -3918
EN 180 -5681
EN 200 -4385
EN 220 -3580
EN 240 -2104
EN 260 -4571
EN 280 -4610
EN 300 -3816
EN 320 -5414
EN 340 -3271
EN 360 -6116
G-R5:LEU-S1
EN 20 0
EN 40 476
EN 60 399
EN 80 -603
EN 100 -409
EN 120 1034
EN 140 -477
EN 160 -168
EN 180 0
EN 200 -2905
EN 220 -2100
EN 240 2097
EN 260 -2303
EN 280 -409
EN 300 1098
EN 320 -302
EN 340 1642
EN 360 0
C-P:LYS-S2
EN 20 0
EN 40 -4990

EN 60   -5265
EN 80   -5216
EN 100   -5163
EN 120   -6862
EN 140   -6552
EN 160   -6017
EN 180   -7238
EN 200   -5650
EN 220   -4845
EN 240   -5469
EN 260   -5721
EN 280   -5424
EN 300   -6875
EN 320   -7270
EN 340   -7811
EN 360   -7120
G-P:MET-CA
EN 20   0
EN 40   0
EN 60   -2747
EN 80   -3751
EN 100   -4109
EN 120   -395
EN 140   -903
EN 160   0
EN 180   0
EN 200   0
EN 220   -2526
EN 240   -1050
EN 260   -298
EN 280   -4109
EN 300   -331
EN 320   -4162
EN 340   -4939
EN 360   -3796
G-RIB:ARG-S1
EN 20   -6013
EN 40   -5316
EN 60   -5945
EN 80   -6152
EN 100   -3970
EN 120   -5049
EN 140   -3840
EN 160   -5446
EN 180   -3125
EN 200   -6268
EN 220   -3454
EN 240   -6518
EN 260   -5560
EN 280   -5383
EN 300   -5483
EN 320   -5090
EN 340   -2432
EN 360   -4724
C-P:HIS-S1
EN 20   0
EN 40   -7111
EN 60   -3200

EN 80   -3491
EN 100   -6516
EN 120   -3570
EN 140   -4078
EN 160   -5486
EN 180   0
EN 200   0
EN 220   0
EN 240   -3220
EN 260   -4738
EN 280   -4562
EN 300   -2501
EN 320   -6332
EN 340   -4679
EN 360   -4248
G-P:HIS-CA
EN 20   0
EN 40   -2738
EN 60   -4531
EN 80   -6738
EN 100   -4628
EN 120   -4901
EN 140   -5409
EN 160   -5100
EN 180   -5698
EN 200   0
EN 220   0
EN 240   -1117
EN 260   -2082
EN 280   -5010
EN 300   -5842
EN 320   -5233
EN 340   -3289
EN 360   0
A-R6:TYR-S2
EN 20   0
EN 40   -5264
EN 60   -3623
EN 80   -4627
EN 100   -4433
EN 120   -2988
EN 140   -2492
EN 160   -1469
EN 180   0
EN 200   0
EN 220   -4114
EN 240   -3643
EN 260   -2891
EN 280   -1710
EN 300   -203
EN 320   -598
EN 340   -4098
EN 360   0
G-R6:LYS-S2
EN 20   0
EN 40   -4577
EN 60   -5206
EN 80   -4493

EN 100 -3530
EN 120 -4019
EN 140 -3191
EN 160 -2500
EN 180 -1381
EN 200 0
EN 220 -2715
EN 240 -5056
EN 260 -4304
EN 280 -5132
EN 300 -4818
EN 320 -3899
EN 340 -3793
EN 360 0
C-Y:LEU-S2
EN 20 -3056
EN 40 -2652
EN 60 -1723
EN 80 -297
EN 100 -815
EN 120 -376
EN 140 1837
EN 160 0
EN 180 -1173
EN 200 -3312
EN 220 -3219
EN 240 -1744
EN 260 -992
EN 280 -2824
EN 300 -1025
EN 320 0
EN 340 0
EN 360 0
A-R6:SER-S1
EN 20 0
EN 40 -3958
EN 60 -4034
EN 80 -1603
EN 100 -3126
EN 120 -677
EN 140 -5173
EN 160 -2885
EN 180 -4196
EN 200 -4618
EN 220 -4525
EN 240 -4054
EN 260 -2298
EN 280 -4130
EN 300 -2331
EN 320 -2727
EN 340 -4891
EN 360 -2361
C-RIB:HIS-S2
EN 20 -6257
EN 40 -4848
EN 60 -4924
EN 80 -4764
EN 100 -5021

EN 120   -6007
EN 140   -4798
EN 160   -6497
EN 180   -7809
EN 200   0
EN 220   -2986
EN 240   -4945
EN 260   -4193
EN 280   -5734
EN 300   -5231
EN 320   -6892
EN 340   -3682
EN 360   -7691
C-RIB:GLU-CA
EN 20   -3251
EN 40   -1842
EN 60   -1918
EN 80   -2209
EN 100   -1563
EN 120   433
EN 140   -3801
EN 160   -3039
EN 180   -3085
EN 200   -3506
EN 220   -1697
EN 240   1495
EN 260   -1186
EN 280   -2015
EN 300   -3726
EN 320   -2168
EN 340   -3397
EN 360   -3971
C-Y:PHE-S2
EN 20   -6866
EN 40   0
EN 60   -2098
EN 80   -1385
EN 100   526
EN 120   -2468
EN 140   -254
EN 160   0
EN 180   0
EN 200   0
EN 220   -1877
EN 240   -3836
EN 260   -2371
EN 280   -2195
EN 300   -1400
EN 320   0
EN 340   0
EN 360   0
G-R5:PRO-CA
EN 20   0
EN 40   -4549
EN 60   -3807
EN 80   -1482
EN 100   -1287
EN 120   -556

EN 140   -2069
EN 160   0
EN 180   0
EN 200   0
EN 220   -6122
EN 240   -3481
EN 260   -2176
EN 280   -2000
EN 300   1224
EN 320   -888
EN 340   51
EN 360   -4962
C-RIB:ARG-S1
EN 20   0
EN 40   -7437
EN 60   -6695
EN 80   -6285
EN 100   -5993
EN 120   -5311
EN 140   -5819
EN 160   -5436
EN 180   -7255
EN 200   -7384
EN 220   -7132
EN 240   -6490
EN 260   -5617
EN 280   -5561
EN 300   -6102
EN 320   -5919
EN 340   -5557
EN 360   -5680
A-R6:ASN-S1
EN 20   -5182
EN 40   0
EN 60   -2131
EN 80   -3688
EN 100   -3494
EN 120   -4931
EN 140   -2004
EN 160   -2369
EN 180   0
EN 200   0
EN 220   -3627
EN 240   -3869
EN 260   -4503
EN 280   -2940
EN 300   -3703
EN 320   -2833
EN 340   -4323
EN 360   -3180
U-RIB:TYR-S2
EN 20   0
EN 40   -3309
EN 60   0
EN 80   -4389
EN 100   -4748
EN 120   -2751
EN 140   -1541

EN 160   -4959
EN 180   -6270
EN 200   0
EN 220   -3164
EN 240   -1688
EN 260   -5758
EN 280   -3482
EN 300   -970
EN 320   -4800
EN 340   -4865
EN 360   -4434
C-P:ASN-CA
EN 20   0
EN 40   -1908
EN 60   -3702
EN 80   -4375
EN 100   -5064
EN 120   -5573
EN 140   -3575
EN 160   -4270
EN 180   -5873
EN 200   0
EN 220   -4485
EN 240   -4275
EN 260   -5476
EN 280   -5714
EN 300   -5924
EN 320   -5116
EN 340   -5181
EN 360   -6468
C-RIB:TYR-CA
EN 20   0
EN 40   0
EN 60   -740
EN 80   -1744
EN 100   -4272
EN 120   -4545
EN 140   0
EN 160   0
EN 180   0
EN 200   0
EN 220   -2236
EN 240   -760
EN 260   -3996
EN 280   -5537
EN 300   -5194
EN 320   -2155
EN 340   -3937
EN 360   -5224
U-Y:TYR-S1
EN 20   0
EN 40   0
EN 60   -1664
EN 80   -2216
EN 100   -756
EN 120   -2747
EN 140   -1537
EN 160   0

EN 180   0
EN 200   0
EN 220   0
EN 240   -3402
EN 260   -932
EN 280   -4191
EN 300   -5787
EN 320   -4083
EN 340   0
EN 360   0
U-P:MET-CA
EN 20   0
EN 40   -3976
EN 60   -5056
EN 80   0
EN 100   -3144
EN 120   -5134
EN 140   -3925
EN 160   -2903
EN 180   0
EN 200   -7357
EN 220   -3830
EN 240   -4072
EN 260   0
EN 280   -1426
EN 300   -4358
EN 320   -2032
EN 340   0
EN 360   0
A-P:TRP-S1
EN 20   0
EN 40   -4739
EN 60   0
EN 80   -4101
EN 100   -2189
EN 120   -5185
EN 140   0
EN 160   -6388
EN 180   0
EN 200   0
EN 220   0
EN 240   0
EN 260   0
EN 280   -2189
EN 300   -2399
EN 320   -2795
EN 340   -3572
EN 360   -5864
G-RIB:LEU-S2
EN 20   0
EN 40   0
EN 60   -2707
EN 80   -3113
EN 100   -3134
EN 120   1031
EN 140   -2580
EN 160   -171
EN 180   -2487

EN 200   -2908
EN 220   -1099
EN 240   376
EN 260   -1975
EN 280   -2421
EN 300   -2008
EN 320   -2404
EN 340   -1082
EN 360   -2369
C-P:ALA-CA
EN 20   0
EN 40   -3519
EN 60   -3595
EN 80   -3434
EN 100   -4245
EN 120   -3513
EN 140   -3760
EN 160   -5168
EN 180   -3045
EN 200   -3466
EN 220   -2661
EN 240   -3907
EN 260   -3416
EN 280   -4244
EN 300   -4906
EN 320   -3845
EN 340   -4070
EN 360   0
C-P:ASN-S1
EN 20   0
EN 40   -5343
EN 60   -3702
EN 80   -3993
EN 100   -5898
EN 120   -5987
EN 140   -4128
EN 160   -5275
EN 180   -5873
EN 200   0
EN 220   -5751
EN 240   -3722
EN 260   -6074
EN 280   -5300
EN 300   -5924
EN 320   -5906
EN 340   -6186
EN 360   -5755
G-R6:GLU-S1
EN 20   0
EN 40   -1445
EN 60   -2075
EN 80   -808
EN 100   -1167
EN 120   -1179
EN 140   322
EN 160   -1377
EN 180   -3693
EN 200   0

EN 220 -1300
EN 240 -2547
EN 260 -1689
EN 280 -613
EN 300 -1376
EN 320 -1772
EN 340 -2896
EN 360 -853
U-Y:LEU-CA
EN 20 -3977
EN 40 -2568
EN 60 -2644
EN 80 -213
EN 100 -19
EN 120 -2562
EN 140 917
EN 160 0
EN 180 -2094
EN 200 0
EN 220 0
EN 240 -947
EN 260 1522
EN 280 1698
EN 300 -3332
EN 320 -2341
EN 340 -2406
EN 360 0
C31-P:ASN-CA
EN 20 0
EN 40 0
EN 60 0
EN 80 0
EN 100 0
EN 120 0
EN 140 -18291
EN 160 0
EN 180 0
EN 200 0
EN 220 0
EN 240 0
EN 260 0
EN 280 0
EN 300 0
EN 320 0
EN 340 0
EN 360 0
C-Y:ASN-S1
EN 20 -8460
EN 40 -6338
EN 60 -5078
EN 80 -4987
EN 100 0
EN 120 -3610
EN 140 -1848
EN 160 -825
EN 180 0
EN 200 -8715
EN 220 -1753

EN 240   -5099
EN 260   -3513
EN 280   -3647
EN 300   -3546
EN 320   0
EN 340   -2449
EN 360   -3023
IU-MY:ARG-CA
EN 20   -17631
EN 40   0
EN 60   0
EN 80   -13154
EN 100   0
EN 120   0
EN 140   0
EN 160   0
EN 180   0
EN 200   0
EN 220   0
EN 240   0
EN 260   0
EN 280   0
EN 300   0
EN 320   0
EN 340   0
EN 360   0
U-Y:PHE-CA
EN 20   0
EN 40   0
EN 60   -3019
EN 80   -588
EN 100   0
EN 120   -2384
EN 140   -1174
EN 160   0
EN 180   0
EN 200   0
EN 220   -5519
EN 240   -1322
EN 260   -2287
EN 280   -5215
EN 300   -4420
EN 320   -999
EN 340   0
EN 360   0
U-RIB:PRO-CA
EN 20   -5568
EN 40   -5164
EN 60   -2518
EN 80   -2809
EN 100   -3880
EN 120   -4987
EN 140   -674
EN 160   -5808
EN 180   -5402
EN 200   0
EN 220   0
EN 240   -4256

EN 260 -5513
EN 280 -1610
EN 300 -5546
EN 320 -3933
EN 340 0
EN 360 -5284
C-P:PHE-CA
EN 20 0
EN 40 0
EN 60 -2105
EN 80 -1391
EN 100 -2201
EN 120 247
EN 140 -260
EN 160 -4943
EN 180 0
EN 200 0
EN 220 0
EN 240 -2125
EN 260 -3090
EN 280 -3467
EN 300 -2411
EN 320 0
EN 340 -2579
EN 360 0
C-Y:ASP-S2
EN 20 -3989
EN 40 -6568
EN 60 -1944
EN 80 -5528
EN 100 -2301
EN 120 -1309
EN 140 -2530
EN 160 -3778
EN 180 -4828
EN 200 -4245
EN 220 -4152
EN 240 -4947
EN 260 -2478
EN 280 -2753
EN 300 -241
EN 320 -2907
EN 340 -2418
EN 360 0
A-P:TRP-S2
EN 20 -7865
EN 40 0
EN 60 -5819
EN 80 -2384
EN 100 -2189
EN 120 0
EN 140 -2970
EN 160 -3666
EN 180 -5982
EN 200 0
EN 220 0
EN 240 -4835
EN 260 0

EN 280   -2189
EN 300   -2399
EN 320   -5517
EN 340   0
EN 360   -5864
U-P:ILE-CA
EN 20   0
EN 40   0
EN 60   -2066
EN 80   0
EN 100   -3429
EN 120   -1432
EN 140   -3657
EN 160   -917
EN 180   0
EN 200   0
EN 220   0
EN 240   -369
EN 260   -1335
EN 280   558
EN 300   348
EN 320   -4034
EN 340   -3546
EN 360   0
FHU-P:LYS-CA
EN 20   0
EN 40   0
EN 60   0
EN 80   0
EN 100   -14382
EN 120   -14655
EN 140   0
EN 160   0
EN 180   0
EN 200   0
EN 220   0
EN 240   0
EN 260   0
EN 280   0
EN 300   -14592
EN 320   0
EN 340   0
EN 360   -18056
C-Y:ARG-CA
EN 20   0
EN 40   -4555
EN 60   -2914
EN 80   -3656
EN 100   -4557
EN 120   -2589
EN 140   -3239
EN 160   505
EN 180   -1810
EN 200   -6671
EN 220   -422
EN 240   -3768
EN 260   -3638
EN 280   -3723

EN 300   -1662
EN 320   -3776
EN 340   -2123
EN 360   0
C-P:GLN-S2
EN 20   -5309
EN 40   -5617
EN 60   -6246
EN 80   -4979
EN 100   -4072
EN 120   -5058
EN 140   -3849
EN 160   -5548
EN 180   -5143
EN 200   0
EN 220   -5471
EN 240   -5713
EN 260   -5514
EN 280   -6342
EN 300   -5784
EN 320   -5390
EN 340   -4450
EN 360   -7294
A-RIB:ARG-S2
EN 20   -8302
EN 40   -7091
EN 60   -7522
EN 80   -6547
EN 100   -6353
EN 120   -6229
EN 140   -5988
EN 160   -4103
EN 180   -5967
EN 200   -9270
EN 220   -7301
EN 240   -6411
EN 260   -7242
EN 280   -5845
EN 300   -5940
EN 320   -4949
EN 340   -6279
EN 360   -6300
C-Y:SER-CA
EN 20   -6928
EN 40   -5900
EN 60   -4882
EN 80   -3771
EN 100   -2969
EN 120   -1525
EN 140   688
EN 160   -2728
EN 180   -2322
EN 200   0
EN 220   -4921
EN 240   -4902
EN 260   -4411
EN 280   -3351
EN 300   -2174

EN 320   -1857
EN 340   -2634
EN 360   0
A-R6:CYS-CA
EN 20   0
EN 40   0
EN 60   -3590
EN 80   -2876
EN 100   0
EN 120   0
EN 140   -3463
EN 160   0
EN 180   0
EN 200   0
EN 220   0
EN 240   -3610
EN 260   0
EN 280   -2682
EN 300   -2891
EN 320   0
EN 340   0
EN 360   0
U-RIB:TRP-S1
EN 20   0
EN 40   -7221
EN 60   -3862
EN 80   -5870
EN 100   0
EN 120   0
EN 140   -5452
EN 160   -4430
EN 180   0
EN 200   0
EN 220   0
EN 240   -7317
EN 260   -4848
EN 280   -2954
EN 300   0
EN 320   0
EN 340   -4337
EN 360   0
G-P:TRP-S1
EN 20   0
EN 40   -4203
EN 60   -5284
EN 80   0
EN 100   -5642
EN 120   0
EN 140   -2435
EN 160   -4848
EN 180   -5446
EN 200   0
EN 220   0
EN 240   -2582
EN 260   -3548
EN 280   -3371
EN 300   -3581
EN 320   -3977

EN 340 0
EN 360 -5328
A-R5:TYR-CA
EN 20 0
EN 40 -2538
EN 60 -4332
EN 80 -4623
EN 100 -2711
EN 120 -3697
EN 140 -2487
EN 160 0
EN 180 -5499
EN 200 0
EN 220 -4110
EN 240 -3639
EN 260 -4153
EN 280 -2711
EN 300 -1916
EN 320 -3316
EN 340 -659
EN 360 0
C-P:ASP-CA
EN 20 -5669
EN 40 0
EN 60 -3172
EN 80 -3914
EN 100 -3720
EN 120 -4706
EN 140 -3879
EN 160 -5196
EN 180 0
EN 200 0
EN 220 -3402
EN 240 -4357
EN 260 -1174
EN 280 -3981
EN 300 -3930
EN 320 -2869
EN 340 -3094
EN 360 0
G-R5:GLN-S2
EN 20 -4896
EN 40 -5204
EN 60 -6666
EN 80 -4858
EN 100 -4925
EN 120 -2928
EN 140 -1
EN 160 0
EN 180 0
EN 200 -7657
EN 220 -1624
EN 240 -4969
EN 260 -5751
EN 280 -4372
EN 300 -2864
EN 320 174
EN 340 -602

EN 360   -2894
C-P:GLU-S1
EN 20   0
EN 40   -2863
EN 60   -2487
EN 80   -2226
EN 100   -2744
EN 120   -3309
EN 140   -2361
EN 160   -1790
EN 180   -1384
EN 200   0
EN 220   -3431
EN 240   1479
EN 260   -1756
EN 280   -3947
EN 300   -2954
EN 320   -3349
EN 340   -3414
EN 360   -2983
G-R5:ARG-S2
EN 20   -7742
EN 40   -5881
EN 60   -4692
EN 80   -5480
EN 100   -4457
EN 120   -4255
EN 140   -5951
EN 160   -3925
EN 180   -4141
EN 200   -9865
EN 220   -6570
EN 240   -4712
EN 260   -4342
EN 280   -5475
EN 300   -3542
EN 320   -3676
EN 340   -4951
EN 360   -5289
A-R6:CYS-S1
EN 20   0
EN 40   0
EN 60   -5307
EN 80   -2876
EN 100   0
EN 120   0
EN 140   0
EN 160   0
EN 180   0
EN 200   0
EN 220   0
EN 240   0
EN 260   -2858
EN 280   -2682
EN 300   0
EN 320   -3287
EN 340   0
EN 360   0

C-Y:LEU-CA
EN 20 -4771
EN 40 -1644
EN 60 -1720
EN 80 -1006
EN 100 -99
EN 120 -3095
EN 140 -881
EN 160 -571
EN 180 0
EN 200 0
EN 220 218
EN 240 -1740
EN 260 -276
EN 280 -1365
EN 300 2412
EN 320 2016
EN 340 -477
EN 360 -1051
A-RIB:HIS-CA
EN 20 0
EN 40 -5993
EN 60 -1629
EN 80 -4350
EN 100 -4709
EN 120 -3717
EN 140 -1502
EN 160 -5633
EN 180 -6231
EN 200 0
EN 220 0
EN 240 -3367
EN 260 -6050
EN 280 -4156
EN 300 -7286
EN 320 -5766
EN 340 -6925
EN 360 -4396
C-RIB:LEU-S2
EN 20 0
EN 40 69
EN 60 -1723
EN 80 -2014
EN 100 -1820
EN 120 -1641
EN 140 -3606
EN 160 -2845
EN 180 -1173
EN 200 -3312
EN 220 0
EN 240 -1744
EN 260 -1996
EN 280 -3322
EN 300 -2742
EN 320 -3138
EN 340 -2198
EN 360 -2772
H2U-P:GLU-S1

EN 20   0
EN 40   0
EN 60   0
EN 80   -11950
EN 100   0
EN 120   0
EN 140   -12536
EN 160   0
EN 180   0
EN 200   0
EN 220   0
EN 240   0
EN 260   0
EN 280   0
EN 300   0
EN 320   0
EN 340   0
EN 360   0
A-R5:HIS-S2
EN 20   -6414
EN 40   -6722
EN 60   -5081
EN 80   -2650
EN 100   -5178
EN 120   -5451
EN 140   -4242
EN 160   -2215
EN 180   0
EN 200   -8387
EN 220   -5864
EN 240   -6106
EN 260   -6856
EN 280   -4726
EN 300   -5387
EN 320   -5331
EN 340   -2121
EN 360   0
A-RIB:LYS-CA
EN 20   -5489
EN 40   -4632
EN 60   -2991
EN 80   -3442
EN 100   -4634
EN 120   -4073
EN 140   -3698
EN 160   -4011
EN 180   -3605
EN 200   0
EN 220   -3934
EN 240   -4176
EN 260   -4213
EN 280   -4965
EN 300   -4462
EN 320   -4406
EN 340   -4630
EN 360   -3487
A-R5:TRP-S1
EN 20   -7863

EN 40   -4736
EN 60   0
EN 80   -2381
EN 100   -2187
EN 120   -2460
EN 140   -2968
EN 160   0
EN 180   0
EN 200   0
EN 220   0
EN 240   -5837
EN 260   -4080
EN 280   -4909
EN 300   -2396
EN 320   -2792
EN 340   0
EN 360   0
A-R5:PHE-S2
EN 20   -5306
EN 40   -4901
EN 60   0
EN 80   -2546
EN 100   -1347
EN 120   -3338
EN 140   0
EN 160   0
EN 180   -3422
EN 200   -5561
EN 220   -6022
EN 240   0
EN 260   -3241
EN 280   -3617
EN 300   -1557
EN 320   0
EN 340   0
EN 360   -3304
A-RIB:THR-CA
EN 20   0
EN 40   -4910
EN 60   -3822
EN 80   -3942
EN 100   -4631
EN 120   -4643
EN 140   -4147
EN 160   -2120
EN 180   -4436
EN 200   0
EN 220   -1330
EN 240   -5007
EN 260   -3924
EN 280   -2914
EN 300   -3957
EN 320   -5473
EN 340   -4748
EN 360   -2600
A-P:TRP-CA
EN 20   0
EN 40   0

EN 60   -3097
EN 80   -4101
EN 100   -3907
EN 120   -5185
EN 140   -2970
EN 160   -3666
EN 180   0
EN 200   0
EN 220   0
EN 240   0
EN 260   -2366
EN 280   -2189
EN 300   -2399
EN 320   -2795
EN 340   -3572
EN 360   -5864
A-RIB:GLN-S2
EN 20   -5456
EN 40   -4046
EN 60   -5127
EN 80   -5418
EN 100   -4601
EN 120   -4492
EN 140   -3283
EN 160   -3978
EN 180   -3572
EN 200   0
EN 220   -4906
EN 240   -4143
EN 260   -5400
EN 280   -5224
EN 300   -5694
EN 320   -3107
EN 340   -5602
EN 360   -5171
A-R5:MET-S1
EN 20   0
EN 40   0
EN 60   -3280
EN 80   -2566
EN 100   -3377
EN 120   -2645
EN 140   -3153
EN 160   0
EN 180   0
EN 200   0
EN 220   -3059
EN 240   -1583
EN 260   -2548
EN 280   0
EN 300   -4299
EN 320   -1260
EN 340   -4759
EN 360   -4329
C-RIB:ALA-CA
EN 20   -3201
EN 40   -1792
EN 60   -3585

EN 80   -3425
EN 100   -4235
EN 120   -2238
EN 140   -4845
EN 160   -3823
EN 180   -3035
EN 200   -3456
EN 220   -3364
EN 240   -3606
EN 260   -3407
EN 280   -2969
EN 300   -3676
EN 320   -4287
EN 340   -4613
EN 360   -3921
G-R5:HIS-CA
EN 20   -5837
EN 40   -5433
EN 60   -3791
EN 80   -3631
EN 100   -1879
EN 120   -3157
EN 140   -943
EN 160   0
EN 180   0
EN 200   -6092
EN 220   -6000
EN 240   -2807
EN 260   -4198
EN 280   -1879
EN 300   -2088
EN 320   -2484
EN 340   -3261
EN 360   0
G-RIB:MET-S2
EN 20   0
EN 40   -4372
EN 60   -1014
EN 80   -300
EN 100   -2828
EN 120   -2096
EN 140   -2604
EN 160   -5570
EN 180   0
EN 200   0
EN 220   0
EN 240   -3756
EN 260   -4270
EN 280   -2827
EN 300   -2033
EN 320   -4146
EN 340   -6932
EN 360   -3780
A-P:GLN-CA
EN 20   -5427
EN 40   0
EN 60   -3381
EN 80   -4385

EN 100 -4191
EN 120 -4464
EN 140 -4972
EN 160 -4663
EN 180 0
EN 200 0
EN 220 -4878
EN 240 -680
EN 260 -1645
EN 280 -4573
EN 300 -5902
EN 320 -4345
EN 340 -5574
EN 360 -5143
C-P:PRO-S1
EN 20 0
EN 40 -6349
EN 60 -3874
EN 80 -4617
EN 100 -4131
EN 120 -5193
EN 140 -3747
EN 160 -4894
EN 180 0
EN 200 0
EN 220 -3100
EN 240 -4728
EN 260 -3594
EN 280 -5135
EN 300 -4009
EN 320 -3019
EN 340 -5805
EN 360 -6087
A-RIB:ASP-CA
EN 20 0
EN 40 0
EN 60 0
EN 80 -2605
EN 100 -2862
EN 120 -1418
EN 140 -922
EN 160 -1617
EN 180 -2215
EN 200 0
EN 220 -827
EN 240 648
EN 260 -3039
EN 280 -3575
EN 300 -4338
EN 320 -3468
EN 340 -5250
EN 360 0
G-R5:GLY-CA
EN 20 -8007
EN 40 -5764
EN 60 -5438
EN 80 -4866
EN 100 -2331

EN 120   -3227
EN 140   -8
EN 160   -2974
EN 180   -1302
EN 200   -8262
EN 220   -6624
EN 240   -5148
EN 260   -4041
EN 280   -4379
EN 300   -2541
EN 320   -2555
EN 340   -3332
EN 360   -1184
G-P:ALA-S1
EN 20   0
EN 40   -1416
EN 60   -2045
EN 80   -1783
EN 100   -3688
EN 120   -3127
EN 140   -4285
EN 160   -2060
EN 180   -2659
EN 200   -3080
EN 220   -1270
EN 240   -2899
EN 260   -3030
EN 280   -3090
EN 300   -3064
EN 320   -3198
EN 340   -3976
EN 360   -823
A-P:MET-CA
EN 20   0
EN 40   0
EN 60   -3282
EN 80   -3573
EN 100   -3379
EN 120   -3652
EN 140   -3156
EN 160   -2133
EN 180   0
EN 200   0
EN 220   0
EN 240   -3303
EN 260   -4821
EN 280   -3379
EN 300   -3589
EN 320   -3985
EN 340   0
EN 360   0
U-RIB:PHE-S1
EN 20   0
EN 40   0
EN 60   -3019
EN 80   -588
EN 100   -3828
EN 120   -5488

EN 140   -5614
EN 160   -1870
EN 180   0
EN 200   0
EN 220   0
EN 240   -1322
EN 260   -570
EN 280   -2111
EN 300   -5042
EN 320   -3721
EN 340   -3493
EN 360   -4068
A-RIB:GLN-CA
EN 20   0
EN 40   0
EN 60   -3379
EN 80   -2665
EN 100   -2471
EN 120   -4843
EN 140   -530
EN 160   -1225
EN 180   -3541
EN 200   0
EN 220   -3870
EN 240   -4112
EN 260   -4747
EN 280   -3736
EN 300   -2681
EN 320   -2072
EN 340   -2849
EN 360   -6145
A-R6:GLY-CA
EN 20   -3746
EN 40   -6560
EN 60   -4130
EN 80   -4421
EN 100   -3514
EN 120   -4500
EN 140   -3582
EN 160   -3986
EN 180   0
EN 200   -4001
EN 220   -5918
EN 240   -4939
EN 260   -4187
EN 280   -4779
EN 300   -4221
EN 320   -3115
EN 340   -4605
EN 360   -3461
U-P:GLU-S1
EN 20   0
EN 40   -1060
EN 60   580
EN 80   -1427
EN 100   -228
EN 120   -4228
EN 140   -3731

EN 160   -2709
EN 180   -2303
EN 200   0
EN 220   -915
EN 240   -1156
EN 260   -2675
EN 280   -1233
EN 300   -2708
EN 320   -3556
EN 340   -4333
EN 360   0
G-R6:ILE-CA
EN 20   0
EN 40   0
EN 60   982
EN 80   0
EN 100   -831
EN 120   1617
EN 140   0
EN 160   413
EN 180   -1902
EN 200   0
EN 220   0
EN 240   -1760
EN 260   -792
EN 280   173
EN 300   -1754
EN 320   1284
EN 340   0
EN 360   0
C-RIB:MET-CA
EN 20   0
EN 40   -3047
EN 60   0
EN 80   -3414
EN 100   -4937
EN 120   -771
EN 140   -2996
EN 160   0
EN 180   -6008
EN 200   0
EN 220   0
EN 240   -4148
EN 260   -674
EN 280   -3220
EN 300   -5147
EN 320   -3825
EN 340   0
EN 360   -6894
G-P:ALA-CA
EN 20   0
EN 40   0
EN 60   -2045
EN 80   -1331
EN 100   -2854
EN 120   -4132
EN 140   -2752
EN 160   -3065

EN 180   0
EN 200   0
EN 220   -3541
EN 240   -2517
EN 260   -2769
EN 280   -3306
EN 300   -3516
EN 320   -3696
EN 340   -4237
EN 360   -2541
G-R6:CYS-CA
EN 20   0
EN 40   -4671
EN 60   0
EN 80   -2316
EN 100   0
EN 120   -2395
EN 140   -2903
EN 160   0
EN 180   -5914
EN 200   0
EN 220   0
EN 240   0
EN 260   0
EN 280   0
EN 300   -2331
EN 320   0
EN 340   0
EN 360   0
C-RIB:ASN-CA
EN 20   -5025
EN 40   -1898
EN 60   -3692
EN 80   -5899
EN 100   -5054
EN 120   -4774
EN 140   -5282
EN 160   -3547
EN 180   -5864
EN 200   0
EN 220   -4475
EN 240   -4717
EN 260   -4678
EN 280   -2784
EN 300   -5003
EN 320   -4776
EN 340   -4167
EN 360   -4741
U-RIB:PHE-CA
EN 20   0
EN 40   0
EN 60   -1301
EN 80   -4022
EN 100   -3115
EN 120   -4101
EN 140   -4609
EN 160   -1870
EN 180   0

EN 200   0
EN 220   0
EN 240   -3039
EN 260   0
EN 280   -2111
EN 300   -5424
EN 320   -4434
EN 340   -4498
EN 360   0
A-P:ALA-CA
EN 20   0
EN 40   -1951
EN 60   -3032
EN 80   -3323
EN 100   -3626
EN 120   -2779
EN 140   -3910
EN 160   -3149
EN 180   -4912
EN 200   0
EN 220   0
EN 240   -2048
EN 260   -3013
EN 280   -2124
EN 300   -3599
EN 320   -4231
EN 340   -4219
EN 360   -3076
U-RIB:MET-S1
EN 20   0
EN 40   0
EN 60   -2330
EN 80   -1616
EN 100   -5861
EN 120   -4417
EN 140   -2203
EN 160   -2898
EN 180   0
EN 200   0
EN 220   0
EN 240   -4067
EN 260   -3315
EN 280   -3139
EN 300   -1631
EN 320   -3744
EN 340   0
EN 360   -5096
IU-RIB:ILE-S1
EN 20   0
EN 40   0
EN 60   0
EN 80   0
EN 100   -13454
EN 120   0
EN 140   0
EN 160   0
EN 180   0
EN 200   0

EN 220   0
EN 240   0
EN 260   0
EN 280   0
EN 300   0
EN 320   0
EN 340   0
EN 360   0
A-R5:ARG-S1
EN 20   -7285
EN 40   -5876
EN 60   -4235
EN 80   -5664
EN 100   -4116
EN 120   -3892
EN 140   -3777
EN 160   -1368
EN 180   -1967
EN 200   -7541
EN 220   -2296
EN 240   -4547
EN 260   -5221
EN 280   -2996
EN 300   -4089
EN 320   -3932
EN 340   0
EN 360   -1849
C-P:THR-S1
EN 20   -4455
EN 40   -5316
EN 60   -4839
EN 80   -5130
EN 100   -4720
EN 120   -4496
EN 140   -5915
EN 160   -5961
EN 180   -4289
EN 200   0
EN 220   -3905
EN 240   -3695
EN 260   -4661
EN 280   -4484
EN 300   -5344
EN 320   -3824
EN 340   -5606
EN 360   -5175
U31-P:MET-S1
EN 20   0
EN 40   0
EN 60   0
EN 80   0
EN 100   0
EN 120   0
EN 140   0
EN 160   0
EN 180   0
EN 200   0
EN 220   0

EN 240 -19587
EN 260 0
EN 280 0
EN 300 -18868
EN 320 0
EN 340 0
EN 360 0
A-RIB:CYS-S1
EN 20 0
EN 40 0
EN 60 -5307
EN 80 0
EN 100 -5404
EN 120 -5677
EN 140 0
EN 160 0
EN 180 -6474
EN 200 0
EN 220 0
EN 240 -3610
EN 260 0
EN 280 0
EN 300 -2891
EN 320 0
EN 340 -4064
EN 360 0
FHU-P:LEU-S1
EN 20 0
EN 40 0
EN 60 -14729
EN 80 0
EN 100 0
EN 120 0
EN 140 0
EN 160 -15297
EN 180 0
EN 200 0
EN 220 0
EN 240 -14749
EN 260 0
EN 280 0
EN 300 0
EN 320 0
EN 340 -16921
EN 360 0
U-P:TYR-S1
EN 20 0
EN 40 0
EN 60 -5656
EN 80 -955
EN 100 -761
EN 120 -1034
EN 140 -4264
EN 160 0
EN 180 0
EN 200 0
EN 220 0
EN 240 0

EN 260   -4372
EN 280   -2478
EN 300   -3692
EN 320   0
EN 340   0
EN 360   0
A-R6:TYR-CA
EN 20   0
EN 40   -2538
EN 60   -3619
EN 80   -4623
EN 100   -1706
EN 120   -2984
EN 140   0
EN 160   -3183
EN 180   -3781
EN 200   0
EN 220   -2393
EN 240   -4352
EN 260   -4153
EN 280   10
EN 300   -3633
EN 320   -4029
EN 340   -3089
EN 360   -5050
G-R5:LEU-S2
EN 20   -4371
EN 40   -3514
EN 60   2114
EN 80   105
EN 100   300
EN 120   -685
EN 140   -480
EN 160   1545
EN 180   0
EN 200   -4626
EN 220   -2816
EN 240   -1340
EN 260   -1593
EN 280   300
EN 300   -622
EN 320   -1570
EN 340   0
EN 360   0
U-P:PHE-S1
EN 20   0
EN 40   -4665
EN 60   -1306
EN 80   -592
EN 100   -398
EN 120   -2389
EN 140   -2897
EN 160   -1874
EN 180   -4191
EN 200   0
EN 220   -2802
EN 240   -1326
EN 260   -2292

EN 280   -4386
EN 300   -2325
EN 320   -1003
EN 340   0
EN 360   -4072
FMU-MY:PHE-S2
EN 20   0
EN 40   0
EN 60   0
EN 80   0
EN 100   0
EN 120   0
EN 140   0
EN 160   0
EN 180   0
EN 200   0
EN 220   0
EN 240   0
EN 260   -19528
EN 280   0
EN 300   0
EN 320   0
EN 340   0
EN 360   0
U-P:GLU-CA
EN 20   0
EN 40   -1053
EN 60   0
EN 80   -1420
EN 100   -1939
EN 120   -3216
EN 140   -3725
EN 160   -2702
EN 180   -4014
EN 200   0
EN 220   -908
EN 240   -2867
EN 260   -1402
EN 280   -1939
EN 300   -1436
EN 320   -3931
EN 340   -2609
EN 360   0
A-R5:GLN-CA
EN 20   -5425
EN 40   0
EN 60   -4645
EN 80   56
EN 100   -3737
EN 120   -1739
EN 140   -2247
EN 160   -3947
EN 180   0
EN 200   -7397
EN 220   -2153
EN 240   -677
EN 260   -3913
EN 280   -1466

EN 300 -3393
EN 320 -354
EN 340 0
EN 360 0
C31-P:GLU-S1
EN 20 0
EN 40 0
EN 60 0
EN 80 0
EN 100 0
EN 120 0
EN 140 0
EN 160 0
EN 180 0
EN 200 0
EN 220 0
EN 240 0
EN 260 -15919
EN 280 0
EN 300 0
EN 320 0
EN 340 0
EN 360 0
G-RIB:GLU-CA
EN 20 0
EN 40 0
EN 60 1919
EN 80 -1354
EN 100 -607
EN 120 -2597
EN 140 -2393
EN 160 -2083
EN 180 -2682
EN 200 0
EN 220 -1293
EN 240 181
EN 260 933
EN 280 -2324
EN 300 -2826
EN 320 -2217
EN 340 -272
EN 360 -3568
IU-P:ARG-CA
EN 20 0
EN 40 0
EN 60 -14365
EN 80 0
EN 100 0
EN 120 0
EN 140 0
EN 160 0
EN 180 0
EN 200 0
EN 220 0
EN 240 0
EN 260 0
EN 280 0
EN 300 0

EN 320   -14062
EN 340   0
EN 360   0
FMU-MY:SER-CA
EN 20   0
EN 40   0
EN 60   0
EN 80   0
EN 100   0
EN 120   0
EN 140   -17472
EN 160   0
EN 180   0
EN 200   0
EN 220   0
EN 240   0
EN 260   0
EN 280   0
EN 300   0
EN 320   0
EN 340   0
EN 360   0
G-R6:HIS-CA
EN 20   0
EN 40   0
EN 60   -3791
EN 80   -3790
EN 100   -2883
EN 120   -4422
EN 140   -943
EN 160   0
EN 180   0
EN 200   0
EN 220   -5287
EN 240   -4365
EN 260   -5490
EN 280   -1879
EN 300   -4811
EN 320   -2484
EN 340   0
EN 360   0
U-P:LEU-S2
EN 20   -3985
EN 40   -3580
EN 60   -1939
EN 80   -221
EN 100   1690
EN 120   -3404
EN 140   -3078
EN 160   -3773
EN 180   0
EN 200   0
EN 220   -4701
EN 240   -955
EN 260   -2925
EN 280   -2748
EN 300   -1954
EN 320   -2349

EN 340 -2414
EN 360 0
G-R5:ASP-S1
EN 20 0
EN 40 -3137
EN 60 -2761
EN 80 -1494
EN 100 -1726
EN 120 -860
EN 140 -2081
EN 160 -2776
EN 180 0
EN 200 -5514
EN 220 -4709
EN 240 -3233
EN 260 -3194
EN 280 -2686
EN 300 207
EN 320 -2910
EN 340 -965
EN 360 -1539
G-R6:GLY-CA
EN 20 -4903
EN 40 -4881
EN 60 -5128
EN 80 -4414
EN 100 -3451
EN 120 -3488
EN 140 -3112
EN 160 -2421
EN 180 -4024
EN 200 -6163
EN 220 -4735
EN 240 -3260
EN 260 -4517
EN 280 -3451
EN 300 -3425
EN 320 -3758
EN 340 -2327
EN 360 -3906
5BU-P:ILE-CA
EN 20 0
EN 40 0
EN 60 0
EN 80 0
EN 100 0
EN 120 -15229
EN 140 0
EN 160 0
EN 180 0
EN 200 0
EN 220 0
EN 240 0
EN 260 0
EN 280 0
EN 300 -15166
EN 320 -15561
EN 340 0

EN 360   0
A-R5:ARG-CA
EN 20   -3851
EN 40   -4159
EN 60   -4235
EN 80   -4526
EN 100   -4530
EN 120   -165
EN 140   -3777
EN 160   -1368
EN 180   0
EN 200   -5823
EN 220   -4014
EN 240   -3924
EN 260   -5061
EN 280   -1609
EN 300   -4089
EN 320   -4224
EN 340   -2992
EN 360   -1849
C-Y:GLU-S1
EN 20   -3258
EN 40   -2853
EN 60   -207
EN 80   505
EN 100   -2021
EN 120   0
EN 140   -1085
EN 160   -3498
EN 180   -1375
EN 200   0
EN 220   -2708
EN 240   -3663
EN 260   -2198
EN 280   2417
EN 300   0
EN 320   -1622
EN 340   1034
EN 360   0
C-P:MET-S1
EN 20   0
EN 40   -7044
EN 60   -3133
EN 80   -4690
EN 100   -507
EN 120   -3503
EN 140   -3006
EN 160   -1984
EN 180   0
EN 200   0
EN 220   -2912
EN 240   -4871
EN 260   -4119
EN 280   -4495
EN 300   -2435
EN 320   -2830
EN 340   -5878
EN 360   0

U-RIB:MET-CA
EN 20 0
EN 40 -6693
EN 60 -2330
EN 80 -1616
EN 100 -4144
EN 120 -4417
EN 140 0
EN 160 -4615
EN 180 0
EN 200 0
EN 220 -5543
EN 240 0
EN 260 -1598
EN 280 -4144
EN 300 -4353
EN 320 0
EN 340 0
EN 360 0
G-R5:TYR-S1
EN 20 0
EN 40 0
EN 60 -337
EN 80 -2898
EN 100 570
EN 120 297
EN 140 0
EN 160 -905
EN 180 -3221
EN 200 0
EN 220 -3550
EN 240 -357
EN 260 -2327
EN 280 570
EN 300 -1356
EN 320 -2955
EN 340 0
EN 360 0
A-R5:ASN-S2
EN 20 -9190
EN 40 -6064
EN 60 -4422
EN 80 -4160
EN 100 -4348
EN 120 -3788
EN 140 -3030
EN 160 -4991
EN 180 0
EN 200 -7175
EN 220 -5918
EN 240 -5607
EN 260 -4855
EN 280 -4348
EN 300 -4889
EN 320 -132
EN 340 -909
EN 360 -2488
U-P:ASP-S1

EN 20   -4873
EN 40   0
EN 60   -105
EN 80   -2113
EN 100   -2632
EN 120   -3458
EN 140   -5422
EN 160   0
EN 180   -4707
EN 200   0
EN 220   -4323
EN 240   -125
EN 260   -2808
EN 280   -1919
EN 300   -2129
EN 320   -1520
EN 340   -5732
EN 360   -5593
C-P:SER-CA
EN 20   0
EN 40   -3811
EN 60   -4892
EN 80   -5089
EN 100   -4895
EN 120   -3252
EN 140   -4142
EN 160   -6173
EN 180   -5767
EN 200   0
EN 220   -5383
EN 240   -3456
EN 260   -5071
EN 280   -5962
EN 300   -4193
EN 320   -4297
EN 340   -5864
EN 360   -6653
A-P:GLY-CA
EN 20   0
EN 40   -5443
EN 60   -5519
EN 80   -3972
EN 100   -5731
EN 120   -5366
EN 140   -5723
EN 160   -5490
EN 180   -4587
EN 200   -4004
EN 220   -6832
EN 240   -4153
EN 260   -5544
EN 280   -6416
EN 300   -5578
EN 320   -5388
EN 340   -6325
EN 360   -6568
A-P:TYR-CA
EN 20   0

EN 40   0
EN 60   -3621
EN 80   -2908
EN 100   -2713
EN 120   -3699
EN 140   -4207
EN 160   -4190
EN 180   0
EN 200   0
EN 220   -2395
EN 240   -2637
EN 260   -4607
EN 280   -3426
EN 300   -2923
EN 320   -5036
EN 340   -1374
EN 360   0
IU-MY:VAL-CA
EN 20   0
EN 40   0
EN 60   0
EN 80   0
EN 100   -11752
EN 120   -12025
EN 140   0
EN 160   0
EN 180   0
EN 200   0
EN 220   0
EN 240   0
EN 260   0
EN 280   0
EN 300   0
EN 320   0
EN 340   0
EN 360   0
G-R6:ALA-CA
EN 20   0
EN 40   328
EN 60   252
EN 80   -2138
EN 100   -983
EN 120   -1835
EN 140   -1891
EN 160   -2033
EN 180   -914
EN 200   0
EN 220   -3965
EN 240   -2038
EN 260   -2120
EN 280   155
EN 300   -2776
EN 320   554
EN 340   1494
EN 360   0
U-RIB:HIS-S1
EN 20   0
EN 40   0

EN 60   -5118
EN 80   -6835
EN 100   -6310
EN 120   -4484
EN 140   -6257
EN 160   -5687
EN 180   -6998
EN 200   0
EN 220   0
EN 240   0
EN 260   -5652
EN 280   -4923
EN 300   -1698
EN 320   -2094
EN 340   -6859
EN 360   0
A-R6:ARG-CA
EN 20   0
EN 40   0
EN 60   -4788
EN 80   -4074
EN 100   -3619
EN 120   -4153
EN 140   -4400
EN 160   -3086
EN 180   0
EN 200   0
EN 220   -3301
EN 240   -4547
EN 260   -1073
EN 280   -4116
EN 300   -1819
EN 320   -3932
EN 340   -3891
EN 360   0
C-Y:GLU-CA
EN 20   0
EN 40   -2847
EN 60   -1918
EN 80   -989
EN 100   0
EN 120   -2670
EN 140   -74
EN 160   -769
EN 180   -1368
EN 200   0
EN 220   -1697
EN 240   -1938
EN 260   2247
EN 280   2424
EN 300   2214
EN 320   0
EN 340   -1680
EN 360   0
C-Y:VAL-CA
EN 20   0
EN 40   -3086
EN 60   -1444

EN 80 -2448
EN 100 467
EN 120 -2527
EN 140 -313
EN 160 708
EN 180 0
EN 200 0
EN 220 -1936
EN 240 -2730
EN 260 291
EN 280 -2254
EN 300 -2012
EN 320 -137
EN 340 0
EN 360 0
G-RIB:VAL-S1
EN 20 0
EN 40 -2684
EN 60 -38
EN 80 -2047
EN 100 -2234
EN 120 -1121
EN 140 -916
EN 160 -607
EN 180 -1206
EN 200 0
EN 220 -1535
EN 240 -1776
EN 260 692
EN 280 -848
EN 300 -345
EN 320 -3463
EN 340 -1518
EN 360 -3809
A-R6:ASN-CA
EN 20 0
EN 40 0
EN 60 -2131
EN 80 -2422
EN 100 -3945
EN 120 -4931
EN 140 -3009
EN 160 -4257
EN 180 -3298
EN 200 0
EN 220 -4632
EN 240 -434
EN 260 -4122
EN 280 -3945
EN 300 -3703
EN 320 -111
EN 340 -2606
EN 360 0
DA-M5:VAL-S1
EN 20 0
EN 40 0
EN 60 0
EN 80 0

EN 100   0
EN 120   0
EN 140   0
EN 160   0
EN 180   0
EN 200   0
EN 220   0
EN 240   -16906
EN 260   0
EN 280   0
EN 300   0
EN 320   0
EN 340   0
EN 360   0
A-P:LYS-CA
EN 20   0
EN 40   -3369
EN 60   -4450
EN 80   -3997
EN 100   -4967
EN 120   -6245
EN 140   -5234
EN 160   -6594
EN 180   -5325
EN 200   0
EN 220   -502
EN 240   -4470
EN 260   -3095
EN 280   -4254
EN 300   -4464
EN 320   -3525
EN 340   -5836
EN 360   -6593
C-RIB:PHE-S2
EN 20   0
EN 40   0
EN 60   -3103
EN 80   -2389
EN 100   -2908
EN 120   -3181
EN 140   -1972
EN 160   -3671
EN 180   -3266
EN 200   0
EN 220   0
EN 240   0
EN 260   -1367
EN 280   -3912
EN 300   316
EN 320   -2801
EN 340   -3578
EN 360   -3147
G-RIB:TRP-S1
EN 20   0
EN 40   -4176
EN 60   -4252
EN 80   -1821
EN 100   -3344

EN 120   -3617
EN 140   -4125
EN 160   -3103
EN 180   0
EN 200   0
EN 220   0
EN 240   -5990
EN 260   -1803
EN 280   -4349
EN 300   -5824
EN 320   -3950
EN 340   -3009
EN 360   0
A-P:VAL-S1
EN 20   0
EN 40   -2242
EN 60   -4036
EN 80   -892
EN 100   -2797
EN 120   -2237
EN 140   -2192
EN 160   -3892
EN 180   -1768
EN 200   0
EN 220   -380
EN 240   -2339
EN 260   -874
EN 280   -3917
EN 300   -2625
EN 320   -3021
EN 340   -1076
EN 360   -1650
U31-MY:MET-S2
EN 20   0
EN 40   0
EN 60   0
EN 80   0
EN 100   -18670
EN 120   0
EN 140   0
EN 160   0
EN 180   0
EN 200   0
EN 220   0
EN 240   0
EN 260   0
EN 280   0
EN 300   0
EN 320   0
EN 340   0
EN 360   0
G-RIB:SER-CA
EN 20   -3802
EN 40   -5115
EN 60   -5390
EN 80   -2760
EN 100   -4068
EN 120   -2839

EN 140 -4849
EN 160 -3591
EN 180 -5907
EN 200 0
EN 220 -5683
EN 240 -3043
EN 260 -3747
EN 280 -4068
EN 300 -4691
EN 320 -3171
EN 340 -2944
EN 360 -4523
G-P:GLY-CA
EN 20 -3213
EN 40 -4074
EN 60 -4602
EN 80 -5500
EN 100 -4698
EN 120 -5106
EN 140 -5338
EN 160 -4719
EN 180 -6151
EN 200 0
EN 220 -5093
EN 240 -5761
EN 260 -4423
EN 280 -5609
EN 300 -5405
EN 320 -4681
EN 340 -3359
EN 360 -2929
A-R6:ASP-S2
EN 20 0
EN 40 -3742
EN 60 -2813
EN 80 -1387
EN 100 -1905
EN 120 -2178
EN 140 -1973
EN 160 -3382
EN 180 -2263
EN 200 -4401
EN 220 -2592
EN 240 -4220
EN 260 -1369
EN 280 -4175
EN 300 -4124
EN 320 -1798
EN 340 -1570
EN 360 -3862
G-P:ASP-CA
EN 20 0
EN 40 0
EN 60 0
EN 80 -2906
EN 100 -3334
EN 120 -3315
EN 140 -4828

EN 160 -4519
EN 180 -1683
EN 200 0
EN 220 -3016
EN 240 -3258
EN 260 -1501
EN 280 -3595
EN 300 -5261
EN 320 -4201
EN 340 -3712
EN 360 -5552
A-P:LEU-S1
EN 20 0
EN 40 -86
EN 60 -162
EN 80 -1166
EN 100 -259
EN 120 472
EN 140 -1040
EN 160 -731
EN 180 -3047
EN 200 0
EN 220 -1658
EN 240 -1900
EN 260 -1148
EN 280 745
EN 300 -3452
EN 320 -1577
EN 340 -2907
EN 360 0
A-P:HIS-S2
EN 20 0
EN 40 -5008
EN 60 -7093
EN 80 -5375
EN 100 -6185
EN 120 -5835
EN 140 -4957
EN 160 -3935
EN 180 -6251
EN 200 0
EN 220 -4862
EN 240 -4391
EN 260 -5357
EN 280 -5180
EN 300 -4385
EN 320 -4068
EN 340 -6563
EN 360 -4415
A-R5:PHE-S1
EN 20 -5302
EN 40 -2175
EN 60 -2252
EN 80 -1538
EN 100 373
EN 120 100
EN 140 0
EN 160 0

EN 180   0
EN 200   0
EN 220   -4752
EN 240   -554
EN 260   -1520
EN 280   -1343
EN 300   -3271
EN 320   0
EN 340   0
EN 360   0
C-RIB:ARG-CA
EN 20   -5411
EN 40   -6011
EN 60   -5281
EN 80   -5082
EN 100   -4888
EN 120   -5161
EN 140   -4956
EN 160   -5849
EN 180   -5798
EN 200   -3949
EN 220   -3144
EN 240   -5485
EN 260   -5490
EN 280   -5179
EN 300   -5247
EN 320   -5493
EN 340   -4845
EN 360   -5127
U-RIB:LYS-S1
EN 20   0
EN 40   -5405
EN 60   -4598
EN 80   -5219
EN 100   -4809
EN 120   -6161
EN 140   -2371
EN 160   -6050
EN 180   -6096
EN 200   -4799
EN 220   -6094
EN 240   -6152
EN 260   -6206
EN 280   -4809
EN 300   -3517
EN 320   -5179
EN 340   -5072
EN 360   -5264
G-P:ASN-CA
EN 20   0
EN 40   -1522
EN 60   -3869
EN 80   -3989
EN 100   -4914
EN 120   -5187
EN 140   -5198
EN 160   -3171
EN 180   -7205

EN 200   -6621
EN 220   -1377
EN 240   -3889
EN 260   -4593
EN 280   -3794
EN 300   -5339
EN 320   -4731
EN 340   -4795
EN 360   -4365
A-R5:LYS-CA
EN 20   -5489
EN 40   -4079
EN 60   -2438
EN 80   -1724
EN 100   -3247
EN 120   -2356
EN 140   -594
EN 160   -2294
EN 180   -3605
EN 200   -4026
EN 220   -3221
EN 240   -1746
EN 260   -2711
EN 280   -4252
EN 300   -1740
EN 320   -2135
EN 340   -2913
EN 360   0
G-R5:TYR-S2
EN 20   0
EN 40   -1982
EN 60   -3776
EN 80   -1345
EN 100   -5075
EN 120   -2213
EN 140   0
EN 160   0
EN 180   0
EN 200   0
EN 220   0
EN 240   -4349
EN 260   -1327
EN 280   -3294
EN 300   -2365
EN 320   -1425
EN 340   -2533
EN 360   -3107
A-R5:LEU-S2
EN 20   0
EN 40   0
EN 60   1554
EN 80   -2171
EN 100   -2359
EN 120   -1245
EN 140   1681
EN 160   0
EN 180   -3047
EN 200   -3468

EN 220   -3929
EN 240   -1188
EN 260   -436
EN 280   -3478
EN 300   -469
EN 320   -2582
EN 340   1079
EN 360   0
A-RIB:TYR-CA
EN 20   0
EN 40   0
EN 60   -4332
EN 80   -3618
EN 100   -3977
EN 120   -1979
EN 140   -2487
EN 160   -4187
EN 180   -3781
EN 200   0
EN 220   -2393
EN 240   -917
EN 260   -2887
EN 280   -2711
EN 300   -4186
EN 320   -3316
EN 340   -5359
EN 360   0
A-R5:SER-S1
EN 20   -7797
EN 40   -5223
EN 60   -5300
EN 80   -2869
EN 100   -2674
EN 120   -3294
EN 140   -3907
EN 160   -3598
EN 180   -5201
EN 200   -6335
EN 220   -5530
EN 240   -4436
EN 260   -4307
EN 280   -4391
EN 300   -2884
EN 320   -3280
EN 340   -1787
EN 360   0
C-P:THR-CA
EN 20   0
EN 40   -3046
EN 60   -4127
EN 80   -4915
EN 100   -4484
EN 120   -5591
EN 140   -4000
EN 160   -5077
EN 180   -4289
EN 200   0
EN 220   -2901

EN 240   -3142
EN 260   -4108
EN 280   -4720
EN 300   -4141
EN 320   -4537
EN 340   -4150
EN 360   -5175
C-P:MET-CA
EN 20   0
EN 40   -3057
EN 60   -4850
EN 80   -2419
EN 100   -3230
EN 120   -4215
EN 140   -3006
EN 160   0
EN 180   0
EN 200   0
EN 220   -2912
EN 240   -4158
EN 260   -3406
EN 280   -507
EN 300   -4152
EN 320   -5101
EN 340   -1890
EN 360   0
U-Y:VAL-S1
EN 20   -4416
EN 40   -3720
EN 60   -1366
EN 80   -652
EN 100   -1462
EN 120   0
EN 140   -1239
EN 160   0
EN 180   0
EN 200   0
EN 220   -3866
EN 240   -2391
EN 260   -1639
EN 280   -1462
EN 300   0
EN 320   653
EN 340   -123
EN 360   0
A-P:LEU-S2
EN 20   0
EN 40   -89
EN 60   -1883
EN 80   -456
EN 100   -262
EN 120   469
EN 140   -1043
EN 160   -1738
EN 180   0
EN 200   0
EN 220   55
EN 240   -2456

EN 260   566
EN 280   -1979
EN 300   -1737
EN 320   -2967
EN 340   -2357
EN 360   -3936
FHU-P:LYS-S1
EN 20   0
EN 40   0
EN 60   0
EN 80   0
EN 100   0
EN 120   -14661
EN 140   -15169
EN 160   0
EN 180   0
EN 200   0
EN 220   0
EN 240   0
EN 260   0
EN 280   0
EN 300   -14598
EN 320   -14993
EN 340   0
EN 360   0
U-RIB:PRO-S1
EN 20   -5568
EN 40   0
EN 60   -5622
EN 80   -3522
EN 100   -3880
EN 120   -4153
EN 140   -4661
EN 160   -4804
EN 180   -3685
EN 200   0
EN 220   -4014
EN 240   -2538
EN 260   -4890
EN 280   -4714
EN 300   -2824
EN 320   -5650
EN 340   -2993
EN 360   -6289
A-R5:ASP-S1
EN 20   0
EN 40   -5796
EN 60   666
EN 80   -3059
EN 100   0
EN 120   -2133
EN 140   -924
EN 160   -2624
EN 180   0
EN 200   -6074
EN 220   -2547
EN 240   -3341
EN 260   1398

EN 280   -2413
EN 300   1364
EN 320   -2465
EN 340   -2530
EN 360   -2099
U-P:THR-S1
EN 20   0
EN 40   -4969
EN 60   -3328
EN 80   -2614
EN 100   -3133
EN 120   -2693
EN 140   -5301
EN 160   -5162
EN 180   -5208
EN 200   0
EN 220   -4824
EN 240   -4614
EN 260   -4314
EN 280   -4137
EN 300   -2630
EN 320   -5456
EN 340   -6233
EN 360   -6807
A-R5:LYS-S2
EN 20   -3824
EN 40   -4686
EN 60   -6479
EN 80   -4500
EN 100   -4305
EN 120   -4363
EN 140   -4374
EN 160   -2347
EN 180   0
EN 200   -7514
EN 220   -5374
EN 240   -5571
EN 260   -4864
EN 280   -5168
EN 300   -5068
EN 320   -4459
EN 340   -2966
EN 360   0
A-RIB:ARG-CA
EN 20   -6573
EN 40   -4712
EN 60   -3904
EN 80   -4310
EN 100   -5718
EN 120   -4605
EN 140   -4897
EN 160   -6006
EN 180   -3685
EN 200   -4106
EN 220   -5018
EN 240   -3924
EN 260   -5221
EN 280   -4530

EN 300   -4740
EN 320   -4937
EN 340   -4710
EN 360   -5837
U34-P:TYR-CA
EN 20   0
EN 40   0
EN 60   0
EN 80   -18187
EN 100   0
EN 120   0
EN 140   0
EN 160   0
EN 180   0
EN 200   0
EN 220   0
EN 240   0
EN 260   0
EN 280   0
EN 300   0
EN 320   -18599
EN 340   0
EN 360   0
U-Y:ALA-S1
EN 20   -5842
EN 40   -5820
EN 60   -2792
EN 80   -3465
EN 100   1550
EN 120   -1941
EN 140   -2449
EN 160   -2647
EN 180   0
EN 200   -4380
EN 220   0
EN 240   -3365
EN 260   -1347
EN 280   -166
EN 300   -1381
EN 320   -2489
EN 340   -1549
EN 360   0
U-P:VAL-S1
EN 20   0
EN 40   -1294
EN 60   -1370
EN 80   -2374
EN 100   -2733
EN 120   -2453
EN 140   -2961
EN 160   -3656
EN 180   -2537
EN 200   -4676
EN 220   -4584
EN 240   -2395
EN 260   -2356
EN 280   -3566
EN 300   -3394

EN 320 -4172
EN 340 -1845
EN 360 0
FHU-P:TYR-S1
EN 20 0
EN 40 0
EN 60 0
EN 80 0
EN 100 0
EN 120 0
EN 140 0
EN 160 0
EN 180 -20068
EN 200 0
EN 220 0
EN 240 0
EN 260 0
EN 280 0
EN 300 0
EN 320 0
EN 340 0
EN 360 0
C-Y:TRP-S2
EN 20 0
EN 40 -7301
EN 60 -2938
EN 80 -5659
EN 100 -2030
EN 120 -4021
EN 140 -4529
EN 160 -2502
EN 180 0
EN 200 -7961
EN 220 -4434
EN 240 0
EN 260 -2206
EN 280 -5249
EN 300 -4962
EN 320 -2635
EN 340 0
EN 360 0
A-R5:PRO-S1
EN 20 -8236
EN 40 -1675
EN 60 -1751
EN 80 -3759
EN 100 -3947
EN 120 -3386
EN 140 -3894
EN 160 0
EN 180 0
EN 200 0
EN 220 -5517
EN 240 -5206
EN 260 -3289
EN 280 -3947
EN 300 -3196
EN 320 -3166

EN 340   -508
EN 360   -2800
C-P:GLN-S1
EN 20   0
EN 40   -3869
EN 60   -3232
EN 80   -5240
EN 100   -5046
EN 120   -4696
EN 140   -3105
EN 160   -5066
EN 180   -5112
EN 200   0
EN 220   -3723
EN 240   -5352
EN 260   -5222
EN 280   -6471
EN 300   -5256
EN 320   -6149
EN 340   -4419
EN 360   -6711
C-Y:VAL-S1
EN 20   -3492
EN 40   -3088
EN 60   -3546
EN 80   -733
EN 100   -1251
EN 120   -812
EN 140   -1320
EN 160   0
EN 180   0
EN 200   -5465
EN 220   -2942
EN 240   -3184
EN 260   -1980
EN 280   -2256
EN 300   -748
EN 320   -1144
EN 340   0
EN 360   0
C-RIB:LYS-S1
EN 20   -7055
EN 40   -5315
EN 60   -6828
EN 80   -4494
EN 100   -4299
EN 120   -5379
EN 140   -4431
EN 160   -5362
EN 180   -5172
EN 200   -5593
EN 220   -6053
EN 240   -5582
EN 260   -3826
EN 280   -5240
EN 300   -4864
EN 320   -4255
EN 340   -5268

EN 360   -4341
QUO-RIB:LEU-CA
EN 20   0
EN 40   0
EN 60   0
EN 80   0
EN 100   0
EN 120   0
EN 140   0
EN 160   0
EN 180   0
EN 200   0
EN 220   0
EN 240   0
EN 260   -17432
EN 280   0
EN 300   0
EN 320   0
EN 340   0
EN 360   0
A-R5:HIS-S1
EN 20   -6397
EN 40   -4988
EN 60   -3347
EN 80   -5737
EN 100   -3443
EN 120   -4982
EN 140   0
EN 160   -2198
EN 180   0
EN 200   0
EN 220   -4843
EN 240   -6089
EN 260   -3620
EN 280   -5161
EN 300   -2648
EN 320   -5314
EN 340   0
EN 360   0
A-RIB:GLY-CA
EN 20   -8567
EN 40   -6560
EN 60   -5848
EN 80   -5284
EN 100   -5613
EN 120   -4500
EN 140   -6147
EN 160   -5488
EN 180   -1862
EN 200   -4001
EN 220   -6415
EN 240   -5708
EN 260   -3951
EN 280   -4425
EN 300   -6048
EN 320   -5695
EN 340   -5158
EN 360   -5732

FHU-MY:ALA-CA
EN 20 0
EN 40 -16518
EN 60 0
EN 80 0
EN 100 0
EN 120 -14242
EN 140 0
EN 160 0
EN 180 0
EN 200 0
EN 220 0
EN 240 0
EN 260 0
EN 280 0
EN 300 -14178
EN 320 0
EN 340 0
EN 360 0
IU-MY:ALA-CA
EN 20 0
EN 40 0
EN 60 -13375
EN 80 0
EN 100 0
EN 120 -14006
EN 140 0
EN 160 0
EN 180 0
EN 200 0
EN 220 0
EN 240 0
EN 260 0
EN 280 0
EN 300 0
EN 320 0
EN 340 0
EN 360 0
G-P:LEU-S2
EN 20 0
EN 40 445
EN 60 -2734
EN 80 -1186
EN 100 -1826
EN 120 -2099
EN 140 -1773
EN 160 -198
EN 180 -4232
EN 200 0
EN 220 -2130
EN 240 -2372
EN 260 -2886
EN 280 -2156
EN 300 -1202
EN 320 -2049
EN 340 -1822
EN 360 -678
A-RIB:PRO-S1

EN 20 0
EN 40 -1675
EN 60 -5186
EN 80 -2754
EN 100 -4278
EN 120 -5754
EN 140 -4728
EN 160 -5754
EN 180 -2918
EN 200 0
EN 220 0
EN 240 -2776
EN 260 -3289
EN 280 -4830
EN 300 -3323
EN 320 -4552
EN 340 -4496
EN 360 -6787
G-R5:ARG-S1
EN 20 -8112
EN 40 -4985
EN 60 -3967
EN 80 -3514
EN 100 -3059
EN 120 -3040
EN 140 -3548
EN 160 -4535
EN 180 -3125
EN 200 -7534
EN 220 -1736
EN 240 -3695
EN 260 -3732
EN 280 -2436
EN 300 -4180
EN 320 -4377
EN 340 -4442
EN 360 -4011
G-P:ASP-S2
EN 20 0
EN 40 -3209
EN 60 -3667
EN 80 -2953
EN 100 -4957
EN 120 -4749
EN 140 -4163
EN 160 -4235
EN 180 -5718
EN 200 0
EN 220 -3776
EN 240 -2301
EN 260 -3266
EN 280 -5099
EN 300 -4304
EN 320 -3695
EN 340 -6030
EN 360 -4334
A-R5:PHE-CA
EN 20 0

EN 40   0
EN 60   -2252
EN 80   0
EN 100   -2348
EN 120   -1617
EN 140   -407
EN 160   0
EN 180   0
EN 200   -5557
EN 220   -2030
EN 240   -2272
EN 260   -1520
EN 280   -2348
EN 300   -2558
EN 320   0
EN 340   0
EN 360   0
A-R5:VAL-CA
EN 20   0
EN 40   -3243
EN 60   -1601
EN 80   -888
EN 100   -2411
EN 120   -2232
EN 140   1247
EN 160   -2882
EN 180   0
EN 200   0
EN 220   -375
EN 240   -617
EN 260   1852
EN 280   -1959
EN 300   -1616
EN 320   -1299
EN 340   645
EN 360   0
G-R5:SER-CA
EN 20   -6525
EN 40   -4111
EN 60   -4976
EN 80   -2760
EN 100   155
EN 120   -1835
EN 140   -2343
EN 160   396
EN 180   -1919
EN 200   -5775
EN 220   -2248
EN 240   -3494
EN 260   -2742
EN 280   -1987
EN 300   -2324
EN 320   -2167
EN 340   -2231
EN 360   0
C-RIB:PHE-S1
EN 20   0
EN 40   -2019

EN 60 -377
EN 80 -2386
EN 100 -3457
EN 120 -3730
EN 140 -3685
EN 160 -2663
EN 180 -3262
EN 200 0
EN 220 0
EN 240 0
EN 260 -2368
EN 280 -2191
EN 300 -2401
EN 320 -2797
EN 340 -852
EN 360 0
U31-RIB:MET-S1
EN 20 0
EN 40 -21208
EN 60 0
EN 80 -18853
EN 100 0
EN 120 0
EN 140 0
EN 160 0
EN 180 0
EN 200 0
EN 220 0
EN 240 0
EN 260 0
EN 280 0
EN 300 0
EN 320 0
EN 340 0
EN 360 0
A-RIB:ARG-S1
EN 20 -3851
EN 40 -7263
EN 60 -6103
EN 80 -6138
EN 100 -6150
EN 120 -5468
EN 140 -5495
EN 160 -6361
EN 180 -5402
EN 200 0
EN 220 -6023
EN 240 -6123
EN 260 -5512
EN 280 -6247
EN 300 -4923
EN 320 -5490
EN 340 -4710
EN 360 -6670
G-RIB:TYR-CA
EN 20 0
EN 40 0
EN 60 -337

EN 80   -2345
EN 100   570
EN 120   0
EN 140   -210
EN 160   -3627
EN 180   0
EN 200   0
EN 220   -3550
EN 240   0
EN 260   -3593
EN 280   -2864
EN 300   -2361
EN 320   0
EN 340   -3534
EN 360   0
QUO-M5:PHE-S1
EN 20   0
EN 40   0
EN 60   0
EN 80   0
EN 100   0
EN 120   0
EN 140   0
EN 160   0
EN 180   0
EN 200   0
EN 220   0
EN 240   0
EN 260   -17807
EN 280   0
EN 300   0
EN 320   -18236
EN 340   0
EN 360   0
A-R6:PHE-CA
EN 20   0
EN 40   -2175
EN 60   -2252
EN 80   179
EN 100   -3061
EN 120   -1617
EN 140   -407
EN 160   0
EN 180   0
EN 200   0
EN 220   0
EN 240   -2272
EN 260   197
EN 280   -3061
EN 300   -2558
EN 320   0
EN 340   0
EN 360   0
C-RIB:ARG-S2
EN 20   -9150
EN 40   -8348
EN 60   -7747
EN 80   -6481

EN 100   -5799
EN 120   -6072
EN 140   -6787
EN 160   -5212
EN 180   -6975
EN 200   -9113
EN 220   -8507
EN 240   -7971
EN 260   -6633
EN 280   -6457
EN 300   -6496
EN 320   -6611
EN 340   -6433
EN 360   -7148
DA-M6:MET-S2
EN 20   0
EN 40   0
EN 60   0
EN 80   0
EN 100   0
EN 120   0
EN 140   0
EN 160   0
EN 180   0
EN 200   0
EN 220   0
EN 240   -19598
EN 260   0
EN 280   0
EN 300   0
EN 320   0
EN 340   0
EN 360   0
U-Y:GLU-S2
EN 20   -4214
EN 40   -1088
EN 60   -2881
EN 80   -1455
EN 100   -1260
EN 120   1188
EN 140   -2042
EN 160   -2737
EN 180   -5766
EN 200   -4469
EN 220   0
EN 240   -2189
EN 260   -2149
EN 280   -1260
EN 300   1251
EN 320   0
EN 340   78
EN 360   0
G-R5:LEU-CA
EN 20   0
EN 40   476
EN 60   -604
EN 80   1113
EN 100   -409

EN 120 -1235
EN 140 -2195
EN 160 1548
EN 180 0
EN 200 0
EN 220 -2100
EN 240 -1890
EN 260 -2303
EN 280 1308
EN 300 -619
EN 320 702
EN 340 0
EN 360 -648
A-P:ALA-S1
EN 20 0
EN 40 -234
EN 60 -3745
EN 80 -3323
EN 100 -2837
EN 120 -2779
EN 140 -4171
EN 160 -4605
EN 180 -1477
EN 200 0
EN 220 0
EN 240 -3434
EN 260 -2300
EN 280 -3841
EN 300 -3599
EN 320 -3111
EN 340 -4772
EN 360 -1359
G-R6:LEU-S2
EN 20 -2653
EN 40 -1244
EN 60 396
EN 80 105
EN 100 -412
EN 120 -1238
EN 140 523
EN 160 -171
EN 180 0
EN 200 0
EN 220 -1099
EN 240 -1340
EN 260 -588
EN 280 300
EN 300 -1626
EN 320 699
EN 340 0
EN 360 0
FMU-P:ASP-CA
EN 20 0
EN 40 0
EN 60 0
EN 80 0
EN 100 0
EN 120 0

EN 140 -17208
EN 160 0
EN 180 0
EN 200 0
EN 220 0
EN 240 0
EN 260 0
EN 280 0
EN 300 0
EN 320 0
EN 340 0
EN 360 0
A-P:ILE-CA
EN 20 0
EN 40 0
EN 60 -1297
EN 80 -583
EN 100 -1394
EN 120 -662
EN 140 -1170
EN 160 -148
EN 180 -2464
EN 200 0
EN 220 -1076
EN 240 399
EN 260 -3669
EN 280 -2106
EN 300 -599
EN 320 -1999
EN 340 -4042
EN 360 -2346
A-R6:ALA-CA
EN 20 0
EN 40 -1949
EN 60 -2578
EN 80 -598
EN 100 -1670
EN 120 -1390
EN 140 0
EN 160 -876
EN 180 -3192
EN 200 0
EN 220 -1803
EN 240 -2598
EN 260 0
EN 280 -1669
EN 300 -1879
EN 320 -3992
EN 340 -1787
EN 360 -1356
U-P:CYS-S1
EN 20 0
EN 40 0
EN 60 -4361
EN 80 0
EN 100 0
EN 120 0
EN 140 -4235

```
EN 160   -4930
EN 180   0
EN 200   0
EN 220   0
EN 240   0
EN 260   0
EN 280   0
EN 300   -3663
EN 320   0
EN 340   -4836
EN 360   0
A-P:PHE-S1
EN 20   0
EN 40   0
EN 60   -2254
EN 80   -2545
EN 100   -3064
EN 120   -1619
EN 140   -410
EN 160   -1105
EN 180   0
EN 200   0
EN 220   -3750
EN 240   -557
EN 260   -3793
EN 280   -1346
EN 300   -4660
EN 320   -3669
EN 340   -1012
EN 360   -3303
G-RIB:GLU-S1
EN 20   0
EN 40   0
EN 60   1912
EN 80   -1813
EN 100   -1167
EN 120   -2896
EN 140   -1395
EN 160   -2643
EN 180   -3693
EN 200   -3110
EN 220   416
EN 240   174
EN 260   -77
EN 280   -3120
EN 300   -1376
EN 320   -2224
EN 340   -3714
EN 360   -4841
A-R6:PRO-CA
EN 20   0
EN 40   0
EN 60   -1751
EN 80   -2754
EN 100   -4569
EN 120   -2121
EN 140   -3341
EN 160   -4589
```

EN 180 -2918
EN 200 0
EN 220 -4251
EN 240 -2776
EN 260 -3741
EN 280 -3113
EN 300 -4779
EN 320 -2453
EN 340 -508
EN 360 -2800
G-RIB:PHE-S1
EN 20 -4742
EN 40 -3333
EN 60 0
EN 80 -2695
EN 100 -2501
EN 120 -4492
EN 140 -1565
EN 160 -543
EN 180 -2859
EN 200 0
EN 220 0
EN 240 -2717
EN 260 -1965
EN 280 -2501
EN 300 -993
EN 320 327
EN 340 -4888
EN 360 -4458
U-RIB:GLN-S1
EN 20 0
EN 40 -5787
EN 60 -4146
EN 80 -3432
EN 100 -3951
EN 120 -3511
EN 140 -4732
EN 160 -4714
EN 180 -6026
EN 200 0
EN 220 -2920
EN 240 -4166
EN 260 -4127
EN 280 -4955
EN 300 -3448
EN 320 -5943
EN 340 -4621
EN 360 -6912
U31-P:ASP-S1
EN 20 0
EN 40 0
EN 60 0
EN 80 0
EN 100 0
EN 120 0
EN 140 0
EN 160 0
EN 180 0

EN 200   0
EN 220   0
EN 240   0
EN 260   -16606
EN 280   0
EN 300   -16639
EN 320   0
EN 340   0
EN 360   0
C-Y:CYS-S1
EN 20   0
EN 40   0
EN 60   0
EN 80   -2719
EN 100   0
EN 120   -2798
EN 140   0
EN 160   0
EN 180   0
EN 200   0
EN 220   0
EN 240   -3453
EN 260   0
EN 280   0
EN 300   -4452
EN 320   0
EN 340   0
EN 360   0
FMU-P:VAL-S1
EN 20   0
EN 40   0
EN 60   -16885
EN 80   0
EN 100   0
EN 120   0
EN 140   0
EN 160   0
EN 180   0
EN 200   0
EN 220   0
EN 240   0
EN 260   0
EN 280   0
EN 300   0
EN 320   0
EN 340   0
EN 360   0
U-RIB:SER-CA
EN 20   0
EN 40   -4725
EN 60   -5183
EN 80   -4087
EN 100   -5159
EN 120   -4879
EN 140   -1952
EN 160   -4918
EN 180   -4964
EN 200   -5385

EN 220   -3575
EN 240   -4821
EN 260   -3618
EN 280   -4275
EN 300   -3651
EN 320   -4499
EN 340   -2554
EN 360   -4845
U31-P:HIS-CA
EN 20   0
EN 40   0
EN 60   0
EN 80   0
EN 100   0
EN 120   0
EN 140   0
EN 160   0
EN 180   0
EN 200   0
EN 220   0
EN 240   0
EN 260   0
EN 280   0
EN 300   0
EN 320   -19331
EN 340   0
EN 360   0
G-R5:LYS-S2
EN 20   -8086
EN 40   -4959
EN 60   -6265
EN 80   -5442
EN 100   -4458
EN 120   -3504
EN 140   -1092
EN 160   -3886
EN 180   -5369
EN 200   -8341
EN 220   -4814
EN 240   -5812
EN 260   -4785
EN 280   -4298
EN 300   -3441
EN 320   -6068
EN 340   -4415
EN 360   -2980
U-Y:GLU-CA
EN 20   0
EN 40   -2766
EN 60   592
EN 80   -1416
EN 100   1500
EN 120   1227
EN 140   719
EN 160   23
EN 180   -2292
EN 200   -4430
EN 220   -903

EN 240   -1145
EN 260   1324
EN 280   -216
EN 300   1290
EN 320   894
EN 340   0
EN 360   0
IU-P:LYS-S1
EN 20   -19059
EN 40   0
EN 60   0
EN 80   0
EN 100   -13383
EN 120   0
EN 140   0
EN 160   0
EN 180   0
EN 200   0
EN 220   0
EN 240   0
EN 260   0
EN 280   0
EN 300   0
EN 320   0
EN 340   0
EN 360   0
A-P:LYS-S1
EN 20   0
EN 40   -4088
EN 60   -2446
EN 80   -4455
EN 100   -6447
EN 120   -6352
EN 140   -6428
EN 160   -6119
EN 180   -7048
EN 200   0
EN 220   0
EN 240   -3853
EN 260   -5702
EN 280   -4973
EN 300   -4852
EN 320   -5579
EN 340   -3926
EN 360   -6599
C-Y:ILE-CA
EN 20   -4188
EN 40   -2779
EN 60   -3408
EN 80   1292
EN 100   -1234
EN 120   -1508
EN 140   -1011
EN 160   10
EN 180   0
EN 200   0
EN 220   -4351
EN 240   -1158

EN 260   -406
EN 280   1487
EN 300   -2157
EN 320   881
EN 340   104
EN 360   0
IU-MY:LEU-CA
EN 20   0
EN 40   -13864
EN 60   0
EN 80   0
EN 100   0
EN 120   -11588
EN 140   0
EN 160   0
EN 180   0
EN 200   0
EN 220   0
EN 240   0
EN 260   0
EN 280   0
EN 300   0
EN 320   0
EN 340   0
EN 360   0
U-P:LYS-S1
EN 20   0
EN 40   -1422
EN 60   -1498
EN 80   -6363
EN 100   -6416
EN 120   -6569
EN 140   -6009
EN 160   -6888
EN 180   -6100
EN 200   0
EN 220   -2994
EN 240   -1519
EN 260   -5404
EN 280   -4025
EN 300   -5239
EN 320   -5635
EN 340   -5961
EN 360   -6535
U31-MY:ILE-CA
EN 20   0
EN 40   0
EN 60   0
EN 80   0
EN 100   0
EN 120   0
EN 140   0
EN 160   0
EN 180   0
EN 200   0
EN 220   0
EN 240   0
EN 260   0

EN 280   0
EN 300   0
EN 320   0
EN 340   0
EN 360   -20348
5BU-P:ARG-S2
EN 20   0
EN 40   0
EN 60   0
EN 80   0
EN 100   0
EN 120   0
EN 140   0
EN 160   -15950
EN 180   0
EN 200   0
EN 220   0
EN 240   0
EN 260   0
EN 280   0
EN 300   0
EN 320   0
EN 340   0
EN 360   0
G-R6:TRP-S1
EN 20   0
EN 40   -4176
EN 60   -2535
EN 80   -4543
EN 100   -3344
EN 120   -5335
EN 140   -2408
EN 160   0
EN 180   0
EN 200   0
EN 220   -5748
EN 240   -4272
EN 260   -1803
EN 280   -1627
EN 300   -5824
EN 320   -2232
EN 340   -4727
EN 360   0
G-R5:GLN-CA
EN 20   -4865
EN 40   -6178
EN 60   -5249
EN 80   -1101
EN 100   0
EN 120   0
EN 140   29
EN 160   0
EN 180   0
EN 200   -6837
EN 220   -5581
EN 240   -2839
EN 260   -4187
EN 280   -906

EN 300   -1116
EN 320   -1512
EN 340   0
EN 360   0
A-R6:GLU-CA
EN 20   -3408
EN 40   -281
EN 60   0
EN 80   -1914
EN 100   -1167
EN 120   276
EN 140   -2953
EN 160   -2643
EN 180   0
EN 200   0
EN 220   -2858
EN 240   -1382
EN 260   -630
EN 280   550
EN 300   -3094
EN 320   -3782
EN 340   0
EN 360   0
A-R5:LEU-S1
EN 20   0
EN 40   -1801
EN 60   1557
EN 80   -2881
EN 100   -2686
EN 120   -529
EN 140   0
EN 160   989
EN 180   0
EN 200   0
EN 220   -5090
EN 240   -1897
EN 260   -1698
EN 280   -1973
EN 300   -1732
EN 320   1859
EN 340   -1639
EN 360   0
A-R5:MET-CA
EN 20   0
EN 40   -3204
EN 60   -4285
EN 80   -3571
EN 100   -4642
EN 120   -928
EN 140   -1436
EN 160   -2131
EN 180   0
EN 200   0
EN 220   -3059
EN 240   -3300
EN 260   -831
EN 280   0
EN 300   -2582

EN 320   -2977
EN 340   -3755
EN 360   -4329
C-Y:GLN-CA
EN 20   0
EN 40   -4863
EN 60   -500
EN 80   -3221
EN 100   -1310
EN 120   -1583
EN 140   -2091
EN 160   0
EN 180   0
EN 200   -5523
EN 220   -6436
EN 240   -4960
EN 260   -3756
EN 280   407
EN 300   -1519
EN 320   0
EN 340   -975
EN 360   0
A-R6:GLU-S1
EN 20   0
EN 40   -2005
EN 60   -1369
EN 80   -2373
EN 100   -2891
EN 120   -2000
EN 140   -1242
EN 160   -1937
EN 180   -3249
EN 200   0
EN 220   -2865
EN 240   -1389
EN 260   -1903
EN 280   -461
EN 300   -2770
EN 320   -1066
EN 340   -1844
EN 360   0
A-R6:PHE-S2
EN 20   0
EN 40   -3896
EN 60   0
EN 80   -2546
EN 100   -3065
EN 120   -2625
EN 140   -411
EN 160   0
EN 180   0
EN 200   0
EN 220   -3751
EN 240   -558
EN 260   -4246
EN 280   369
EN 300   159
EN 320   0

EN 340   -4447
EN 360   0
H2U-RIB:PRO-S1
EN 20   0
EN 40   0
EN 60   0
EN 80   0
EN 100   0
EN 120   0
EN 140   0
EN 160   0
EN 180   0
EN 200   0
EN 220   0
EN 240   0
EN 260   0
EN 280   0
EN 300   -13352
EN 320   0
EN 340   0
EN 360   0
G-P:PHE-S2
EN 20   0
EN 40   -3364
EN 60   -3993
EN 80   -2726
EN 100   -3085
EN 120   -3810
EN 140   -2600
EN 160   -4561
EN 180   0
EN 200   0
EN 220   -1501
EN 240   -25
EN 260   726
EN 280   -814
EN 300   692
EN 320   -3690
EN 340   -480
EN 360   -2771
A-RIB:ASP-S1
EN 20   0
EN 40   0
EN 60   0
EN 80   -3772
EN 100   -3577
EN 120   -1420
EN 140   -3194
EN 160   -1619
EN 180   0
EN 200   0
EN 220   0
EN 240   -2076
EN 260   -3423
EN 280   -2864
EN 300   -4792
EN 320   -3470
EN 340   -4960

EN 360   -2099
U-P:TRP-S1
EN 20   0
EN 40   0
EN 60   -5584
EN 80   -3153
EN 100   -6946
EN 120   -3232
EN 140   -3740
EN 160   -4435
EN 180   -6751
EN 200   0
EN 220   0
EN 240   0
EN 260   -3135
EN 280   -6393
EN 300   -7156
EN 320   -5281
EN 340   -4341
EN 360   0
G-R6:ALA-S1
EN 20   -2798
EN 40   328
EN 60   252
EN 80   -2138
EN 100   1491
EN 120   -2547
EN 140   -2343
EN 160   -316
EN 180   -914
EN 200   0
EN 220   -3965
EN 240   -2872
EN 260   -1738
EN 280   -1561
EN 300   -54
EN 320   -1715
EN 340   1494
EN 360   0
G-P:GLN-S2
EN 20   0
EN 40   -3513
EN 60   -5860
EN 80   -3429
EN 100   -4068
EN 120   -4964
EN 140   -5180
EN 160   -6664
EN 180   -6474
EN 200   0
EN 220   -5638
EN 240   -4163
EN 260   -3862
EN 280   -5785
EN 300   -4609
EN 320   -4292
EN 340   -6073
EN 360   -6909

G-R6:ARG-S2
EN 20 0
EN 40 -3611
EN 60 -4890
EN 80 -5117
EN 100 -4889
EN 120 -5061
EN 140 -4113
EN 160 -3091
EN 180 -5407
EN 200 -5275
EN 220 -2753
EN 240 -5094
EN 260 -4673
EN 280 -4922
EN 300 -5228
EN 320 -3937
EN 340 -3741
EN 360 -3018
A-R5:ALA-S1
EN 20 -5075
EN 40 -5053
EN 60 -3029
EN 80 -1864
EN 100 -1670
EN 120 -2394
EN 140 -180
EN 160 -1880
EN 180 -1474
EN 200 -7048
EN 220 -4525
EN 240 -1332
EN 260 -1846
EN 280 600
EN 300 -614
EN 320 -5
EN 340 -782
EN 360 0
IU-P:LEU-S1
EN 20 0
EN 40 0
EN 60 0
EN 80 0
EN 100 0
EN 120 0
EN 140 0
EN 160 0
EN 180 0
EN 200 0
EN 220 0
EN 240 0
EN 260 0
EN 280 -12816
EN 300 0
EN 320 0
EN 340 0
EN 360 0
C-RIB:TYR-S2

EN 20   0
EN 40   -2386
EN 60   -2462
EN 80   -3465
EN 100   -3824
EN 120   -2831
EN 140   -4605
EN 160   -1313
EN 180   0
EN 200   0
EN 220   -4962
EN 240   -3487
EN 260   -4834
EN 280   -4658
EN 300   -2768
EN 320   -4429
EN 340   -4654
EN 360   -5228
G-R5:ASN-S2
EN 20   -7365
EN 40   -4951
EN 60   -3735
EN 80   -3495
EN 100   -4119
EN 120   -4061
EN 140   -4479
EN 160   -3165
EN 180   -2759
EN 200   -7620
EN 220   -4093
EN 240   -5836
EN 260   -3582
EN 280   -5322
EN 300   -3616
EN 320   -1290
EN 340   0
EN 360   -4358
C-P:LYS-S1
EN 20   -3630
EN 40   -2221
EN 60   -4803
EN 80   -3853
EN 100   -4974
EN 120   -5247
EN 140   -6808
EN 160   -5136
EN 180   -6899
EN 200   0
EN 220   -3793
EN 240   -4035
EN 260   -4486
EN 280   -6381
EN 300   -5184
EN 320   -7297
EN 340   -6498
EN 360   -7785
A-P:TYR-S1
EN 20   0

EN 40 0
EN 60 -4334
EN 80 -1903
EN 100 -3426
EN 120 -3699
EN 140 -4207
EN 160 -4903
EN 180 0
EN 200 0
EN 220 0
EN 240 -4355
EN 260 -4155
EN 280 -2713
EN 300 -4189
EN 320 -4032
EN 340 -1374
EN 360 0
C-P:HIS-CA
EN 20 0
EN 40 -5846
EN 60 -3200
EN 80 -769
EN 100 -6279
EN 120 -4835
EN 140 -5795
EN 160 -6038
EN 180 -4367
EN 200 0
EN 220 0
EN 240 -1503
EN 260 -5903
EN 280 -3296
EN 300 -4219
EN 320 -6332
EN 340 -1957
EN 360 -4248
H2U-MY:ARG-S2
EN 20 0
EN 40 0
EN 60 0
EN 80 0
EN 100 -12203
EN 120 0
EN 140 0
EN 160 0
EN 180 0
EN 200 0
EN 220 0
EN 240 0
EN 260 -12380
EN 280 0
EN 300 -12413
EN 320 0
EN 340 -13586
EN 360 0
G-R6:GLN-CA
EN 20 0
EN 40 -3456

EN 60   -1814
EN 80   -4205
EN 100   -2624
EN 120   537
EN 140   0
EN 160   -2383
EN 180   0
EN 200   0
EN 220   -4315
EN 240   -1835
EN 260   -4809
EN 280   -1911
EN 300   600
EN 320   0
EN 340   0
EN 360   0
U-P:THR-CA
EN 20   0
EN 40   -3965
EN 60   -2323
EN 80   -2614
EN 100   -2420
EN 120   -5123
EN 140   -4467
EN 160   -2892
EN 180   -3491
EN 200   0
EN 220   -3819
EN 240   -5066
EN 260   -5027
EN 280   -4850
EN 300   -4347
EN 320   -5456
EN 340   -4516
EN 360   -5090
G-P:PRO-CA
EN 20   0
EN 40   -1142
EN 60   -4322
EN 80   -3608
EN 100   -3414
EN 120   -5221
EN 140   -3813
EN 160   -4890
EN 180   -7206
EN 200   0
EN 220   -996
EN 240   -4965
EN 260   -4926
EN 280   -5131
EN 300   -4959
EN 320   -4019
EN 340   -3963
EN 360   -3984
U-RIB:GLY-CA
EN 20   -6230
EN 40   -5374
EN 60   -6906

EN 80   -5188
EN 100   -3658
EN 120   -5051
EN 140   -6157
EN 160   -5466
EN 180   -7451
EN 200   -6486
EN 220   -6685
EN 240   -2487
EN 260   -3453
EN 280   -6132
EN 300   -5402
EN 320   -6462
EN 340   -5925
EN 360   -5233
U-P:GLN-S1
EN 20   0
EN 40   0
EN 60   -4864
EN 80   -5155
EN 100   -3956
EN 120   -5233
EN 140   -1302
EN 160   -4719
EN 180   -4313
EN 200   0
EN 220   -4642
EN 240   -4171
EN 260   -2414
EN 280   -3955
EN 300   -6175
EN 320   -6278
EN 340   -4626
EN 360   -5912
C-P:TYR-CA
EN 20   0
EN 40   0
EN 60   -5189
EN 80   -4476
EN 100   -1559
EN 120   -4554
EN 140   -2340
EN 160   -4753
EN 180   0
EN 200   0
EN 220   0
EN 240   0
EN 260   -4006
EN 280   -2564
EN 300   -3486
EN 320   -2165
EN 340   -1225
EN 360   -5233
A-R6:PHE-S1
EN 20   0
EN 40   -3893
EN 60   -534
EN 80   0

EN 100 -4066
EN 120 -1617
EN 140 0
EN 160 0
EN 180 0
EN 200 0
EN 220 0
EN 240 0
EN 260 -2524
EN 280 -1343
EN 300 -1553
EN 320 0
EN 340 0
EN 360 0
FMU-MY:GLU-CA
EN 20 0
EN 40 0
EN 60 0
EN 80 0
EN 100 0
EN 120 0
EN 140 0
EN 160 0
EN 180 0
EN 200 0
EN 220 0
EN 240 0
EN 260 0
EN 280 0
EN 300 -15946
EN 320 0
EN 340 0
EN 360 0
A-RIB:ALA-CA
EN 20 -3358
EN 40 -2953
EN 60 -2578
EN 80 -3028
EN 100 -2834
EN 120 -3107
EN 140 -2451
EN 160 -1880
EN 180 0
EN 200 0
EN 220 -3521
EN 240 -3050
EN 260 -1293
EN 280 -2503
EN 300 -3336
EN 320 -3109
EN 340 -2499
EN 360 0
G-P:GLU-S2
EN 20 0
EN 40 -1505
EN 60 -1581
EN 80 -1420
EN 100 -3395

EN 120   -3453
EN 140   -3961
EN 160   -4872
EN 180   -1031
EN 200   0
EN 220   -3077
EN 240   -3319
EN 260   867
EN 280   -2682
EN 300   -3389
EN 320   -3288
EN 340   -3060
EN 360   -3635
G-P:PRO-S1
EN 20   0
EN 40   -4577
EN 60   -5206
EN 80   -3226
EN 100   -5131
EN 120   -4571
EN 140   -4526
EN 160   -4890
EN 180   -5107
EN 200   0
EN 220   -3719
EN 240   -5226
EN 260   -4926
EN 280   -5131
EN 300   -4959
EN 320   -4642
EN 340   -2697
EN 360   -6254
U-RIB:ARG-CA
EN 20   -4618
EN 40   -3208
EN 60   -3837
EN 80   -3124
EN 100   -4386
EN 120   -5570
EN 140   -5167
EN 160   -4406
EN 180   -2734
EN 200   -4873
EN 220   -4068
EN 240   -5811
EN 260   -2553
EN 280   -2929
EN 300   -5093
EN 320   -2269
EN 340   -4312
EN 360   -2616
A-R6:LEU-CA
EN 20   0
EN 40   -83
EN 60   -159
EN 80   -2881
EN 100   -2355
EN 120   -2629

EN 140 -1037
EN 160 0
EN 180 -1327
EN 200 0
EN 220 -1656
EN 240 0
EN 260 -1698
EN 280 -1973
EN 300 -2565
EN 320 -2000
EN 340 1082
EN 360 0
A-R6:ASP-CA
EN 20 0
EN 40 -2690
EN 60 668
EN 80 -335
EN 100 -2862
EN 120 -1418
EN 140 -2639
EN 160 -2621
EN 180 0
EN 200 0
EN 220 0
EN 240 648
EN 260 -1321
EN 280 -140
EN 300 -3785
EN 320 -746
EN 340 193
EN 360 -2097
U-Y:PHE-S2
EN 20 0
EN 40 -4664
EN 60 -3022
EN 80 -591
EN 100 -2827
EN 120 -670
EN 140 -2896
EN 160 0
EN 180 0
EN 200 0
EN 220 -2801
EN 240 -4760
EN 260 -5013
EN 280 -3119
EN 300 -2324
EN 320 -2720
EN 340 0
EN 360 0
U-RIB:ASN-S2
EN 20 -5970
EN 40 -5565
EN 60 -4637
EN 80 -5932
EN 100 -5115
EN 120 -5719
EN 140 -5896

EN 160 -6592
EN 180 -4086
EN 200 0
EN 220 -6686
EN 240 -5662
EN 260 -3905
EN 280 -4733
EN 300 -4491
EN 320 -6051
EN 340 -7618
EN 360 -6690
U-P:LYS-S2
EN 20 -6314
EN 40 -3187
EN 60 -4650
EN 80 -6276
EN 100 -7087
EN 120 -6355
EN 140 -6721
EN 160 -7417
EN 180 -2713
EN 200 0
EN 220 -4759
EN 240 -5001
EN 260 -5636
EN 280 -6689
EN 300 -5840
EN 320 -6396
EN 340 -6244
EN 360 -6029
FHU-P:LEU-CA
EN 20 0
EN 40 0
EN 60 -14729
EN 80 0
EN 100 0
EN 120 0
EN 140 0
EN 160 -15297
EN 180 0
EN 200 0
EN 220 0
EN 240 -14749
EN 260 0
EN 280 0
EN 300 0
EN 320 0
EN 340 -15204
EN 360 -17495
A-P:ASN-CA
EN 20 0
EN 40 -3775
EN 60 -3138
EN 80 -5408
EN 100 -5450
EN 120 -3769
EN 140 -5442
EN 160 -4973

EN 180 -3301
EN 200 0
EN 220 -3630
EN 240 -2154
EN 260 -3120
EN 280 -4330
EN 300 -3706
EN 320 -5819
EN 340 -2609
EN 360 -4900
U-RIB:CYS-S1
EN 20 0
EN 40 0
EN 60 0
EN 80 0
EN 100 0
EN 120 -3722
EN 140 -4230
EN 160 -6643
EN 180 0
EN 200 0
EN 220 0
EN 240 0
EN 260 0
EN 280 -3449
EN 300 0
EN 320 0
EN 340 0
EN 360 0
A-RIB:TRP-S2
EN 20 0
EN 40 -4736
EN 60 -3095
EN 80 0
EN 100 -2187
EN 120 -6899
EN 140 -6403
EN 160 -5381
EN 180 0
EN 200 0
EN 220 0
EN 240 -3115
EN 260 -2363
EN 280 -3904
EN 300 -5118
EN 320 -2792
EN 340 -5287
EN 360 -7578
A-R6:TRP-S2
EN 20 0
EN 40 0
EN 60 -4812
EN 80 -2381
EN 100 -4909
EN 120 -2460
EN 140 0
EN 160 0
EN 180 0

EN 200   0
EN 220   -4591
EN 240   -5837
EN 260   -2363
EN 280   0
EN 300   -6384
EN 320   -2792
EN 340   0
EN 360   -7578
C-P:ASP-S1
EN 20   0
EN 40   -4815
EN 60   -4338
EN 80   -3917
EN 100   -5148
EN 120   -4256
EN 140   -4764
EN 160   -4907
EN 180   -3788
EN 200   0
EN 220   -4117
EN 240   -4651
EN 260   -2894
EN 280   -3430
EN 300   -4645
EN 320   -2318
EN 340   -4100
EN 360   -1952
G-R5:SER-S1
EN 20   -5520
EN 40   -5115
EN 60   -5191
EN 80   -2760
EN 100   -3279
EN 120   -2839
EN 140   -2895
EN 160   -1320
EN 180   -3636
EN 200   -7492
EN 220   -3965
EN 240   -4207
EN 260   -3747
EN 280   -1987
EN 300   -2324
EN 320   -1454
EN 340   -2944
EN 360   -1801
G-R6:LYS-CA
EN 20   0
EN 40   -2806
EN 60   -1165
EN 80   -1004
EN 100   -3240
EN 120   -2630
EN 140   -34
EN 160   987
EN 180   -3045
EN 200   0

EN 220 60
EN 240 -3616
EN 260 -3417
EN 280 -2979
EN 300 -467
EN 320 -3293
EN 340 -3739
EN 360 -2927
G-R5:CYS-CA
EN 20 0
EN 40 -4671
EN 60 0
EN 80 -2316
EN 100 0
EN 120 -4112
EN 140 0
EN 160 -3598
EN 180 0
EN 200 0
EN 220 0
EN 240 0
EN 260 -2298
EN 280 0
EN 300 0
EN 320 0
EN 340 0
EN 360 0
A-R5:ILE-CA
EN 20 -4345
EN 40 -1218
EN 60 -2299
EN 80 1136
EN 100 1330
EN 120 -1664
EN 140 0
EN 160 -1863
EN 180 0
EN 200 0
EN 220 -1073
EN 240 -2320
EN 260 1154
EN 280 0
EN 300 -2314
EN 320 724
EN 340 0
EN 360 0
IU-P:ILE-S1
EN 20 0
EN 40 0
EN 60 0
EN 80 0
EN 100 0
EN 120 0
EN 140 0
EN 160 0
EN 180 0
EN 200 0
EN 220 0

EN 240   0
EN 260   -14128
EN 280   0
EN 300   0
EN 320   0
EN 340   0
EN 360   0
U-Y:ASP-CA
EN 20   -4866
EN 40   -1739
EN 60   -1815
EN 80   -1102
EN 100   0
EN 120   -1181
EN 140   28
EN 160   -666
EN 180   0
EN 200   0
EN 220   -3312
EN 240   -118
EN 260   -2801
EN 280   0
EN 300   599
EN 320   203
EN 340   -3295
EN 360   -2864
FHU-MY:ASP-CA
EN 20   0
EN 40   0
EN 60   0
EN 80   0
EN 100   -14710
EN 120   0
EN 140   0
EN 160   0
EN 180   0
EN 200   0
EN 220   0
EN 240   0
EN 260   0
EN 280   -14710
EN 300   0
EN 320   0
EN 340   0
EN 360   0
C-RIB:GLN-S1
EN 20   -7990
EN 40   -5576
EN 60   -3935
EN 80   -5943
EN 100   -5297
EN 120   -3853
EN 140   -4361
EN 160   -5056
EN 180   -6819
EN 200   0
EN 220   -5431
EN 240   -2238

EN 260   -4590
EN 280   -4031
EN 300   -4954
EN 320   -6139
EN 340   -6419
EN 360   -8088
A-P:MET-S1
EN 20   0
EN 40   0
EN 60   -3282
EN 80   -2569
EN 100   -3379
EN 120   -2648
EN 140   -5878
EN 160   -2133
EN 180   0
EN 200   0
EN 220   0
EN 240   -1585
EN 260   -5655
EN 280   -3379
EN 300   -2584
EN 320   -2980
EN 340   0
EN 360   0
IU-RIB:ALA-S1
EN 20   0
EN 40   0
EN 60   0
EN 80   0
EN 100   -12467
EN 120   0
EN 140   0
EN 160   0
EN 180   0
EN 200   0
EN 220   0
EN 240   0
EN 260   0
EN 280   0
EN 300   -12677
EN 320   0
EN 340   0
EN 360   0
FHU-RIB:ASP-CA
EN 20   0
EN 40   0
EN 60   0
EN 80   0
EN 100   0
EN 120   0
EN 140   -15491
EN 160   0
EN 180   0
EN 200   0
EN 220   0
EN 240   0
EN 260   0

EN 280 0
EN 300 0
EN 320 -15315
EN 340 0
EN 360 0
G-R6:LYS-S1
EN 20 -3217
EN 40 -1808
EN 60 -3893
EN 80 -2557
EN 100 -3482
EN 120 -2966
EN 140 -1757
EN 160 -3005
EN 180 0
EN 200 0
EN 220 54
EN 240 -2909
EN 260 -3874
EN 280 -4080
EN 300 -3692
EN 320 -3299
EN 340 -1646
EN 360 -1215
QUO-M6:LEU-CA
EN 20 0
EN 40 0
EN 60 0
EN 80 0
EN 100 0
EN 120 0
EN 140 0
EN 160 0
EN 180 0
EN 200 0
EN 220 0
EN 240 0
EN 260 -15715
EN 280 0
EN 300 0
EN 320 0
EN 340 0
EN 360 0
G-P:PHE-CA
EN 20 0
EN 40 0
EN 60 -1719
EN 80 -2722
EN 100 -3533
EN 120 -1084
EN 140 -3862
EN 160 -4557
EN 180 0
EN 200 0
EN 220 -3215
EN 240 -3456
EN 260 -3257
EN 280 -811

EN 300 -2738
EN 320 -2421
EN 340 -2193
EN 360 0
A-P:SER-S1
EN 20 0
EN 40 -5678
EN 60 -5041
EN 80 -5040
EN 100 -3129
EN 120 -5501
EN 140 -6340
EN 160 -3600
EN 180 -6469
EN 200 0
EN 220 -2811
EN 240 -3052
EN 260 -5221
EN 280 -4133
EN 300 -3720
EN 320 -6004
EN 340 -4893
EN 360 -6351
G-R6:TRP-S2
EN 20 0
EN 40 -4176
EN 60 0
EN 80 -3538
EN 100 -3344
EN 120 -6339
EN 140 -2408
EN 160 0
EN 180 -5419
EN 200 -7558
EN 220 -4031
EN 240 -4272
EN 260 -1803
EN 280 -4349
EN 300 -3554
EN 320 -3950
EN 340 0
EN 360 0
FHU-P:HIS-S2
EN 20 0
EN 40 0
EN 60 0
EN 80 0
EN 100 0
EN 120 0
EN 140 0
EN 160 0
EN 180 0
EN 200 0
EN 220 0
EN 240 0
EN 260 0
EN 280 -18742
EN 300 0

EN 320 0
EN 340 0
EN 360 0
G-P:ARG-S1
EN 20 0
EN 40 -5012
EN 60 -3994
EN 80 -5495
EN 100 -6782
EN 120 -6081
EN 140 -6589
EN 160 -6541
EN 180 -7790
EN 200 0
EN 220 -3481
EN 240 -4014
EN 260 -5361
EN 280 -6379
EN 300 -6661
EN 320 -6223
EN 340 -6568
EN 360 -7257
IU-RIB:PRO-S1
EN 20 0
EN 40 0
EN 60 0
EN 80 0
EN 100 0
EN 120 -14183
EN 140 0
EN 160 0
EN 180 0
EN 200 0
EN 220 0
EN 240 0
EN 260 0
EN 280 0
EN 300 0
EN 320 0
EN 340 0
EN 360 0
U-Y:TYR-CA
EN 20 0
EN 40 0
EN 60 -1664
EN 80 -950
EN 100 -3478
EN 120 0
EN 140 -3255
EN 160 0
EN 180 0
EN 200 0
EN 220 -3160
EN 240 -1684
EN 260 -2650
EN 280 -6461
EN 300 -4401
EN 320 -4083

EN 340   -2139
EN 360   0
G-R5:PHE-S2
EN 20   0
EN 40   -3337
EN 60   21
EN 80   -982
EN 100   -787
EN 120   0
EN 140   -1568
EN 160   0
EN 180   0
EN 200   0
EN 220   -4909
EN 240   -1716
EN 260   -3580
EN 280   929
EN 300   719
EN 320   0
EN 340   0
EN 360   0
DA-M5:THR-CA
EN 20   0
EN 40   0
EN 60   0
EN 80   0
EN 100   0
EN 120   0
EN 140   0
EN 160   0
EN 180   0
EN 200   0
EN 220   0
EN 240   0
EN 260   0
EN 280   0
EN 300   0
EN 320   -17536
EN 340   0
EN 360   0
FHU-RIB:TYR-S1
EN 20   0
EN 40   0
EN 60   0
EN 80   0
EN 100   0
EN 120   0
EN 140   0
EN 160   0
EN 180   0
EN 200   0
EN 220   0
EN 240   0
EN 260   0
EN 280   0
EN 300   -16485
EN 320   -16881
EN 340   0

EN 360 0
G-R6:GLN-S2
EN 20 0
EN 40 -5204
EN 60 -6284
EN 80 -5355
EN 100 -4041
EN 120 -4867
EN 140 -2723
EN 160 0
EN 180 -3012
EN 200 0
EN 220 -4346
EN 240 -3583
EN 260 -5101
EN 280 -4664
EN 300 -3869
EN 320 174
EN 340 -2320
EN 360 0
C-RIB:ILE-S1
EN 20 0
EN 40 -1062
EN 60 -2142
EN 80 -1429
EN 100 -3334
EN 120 -2220
EN 140 -1011
EN 160 -3424
EN 180 0
EN 200 0
EN 220 0
EN 240 -2163
EN 260 -3510
EN 280 -2952
EN 300 -2710
EN 320 -1840
EN 340 -1613
EN 360 0
A-R6:MET-S2
EN 20 0
EN 40 -3215
EN 60 -1573
EN 80 -4848
EN 100 -665
EN 120 -3661
EN 140 -1447
EN 160 -3859
EN 180 0
EN 200 0
EN 220 -4787
EN 240 -1594
EN 260 -842
EN 280 -4100
EN 300 -2593
EN 320 -5259
EN 340 -3766
EN 360 0

QUO-M5:ASN-S2
EN 20 0
EN 40 0
EN 60 0
EN 80 -17725
EN 100 0
EN 120 0
EN 140 0
EN 160 0
EN 180 0
EN 200 0
EN 220 0
EN 240 0
EN 260 0
EN 280 0
EN 300 0
EN 320 0
EN 340 0
EN 360 0
C31-P:GLN-S2
EN 20 0
EN 40 0
EN 60 0
EN 80 0
EN 100 0
EN 120 0
EN 140 0
EN 160 -19260
EN 180 0
EN 200 0
EN 220 0
EN 240 0
EN 260 0
EN 280 0
EN 300 0
EN 320 0
EN 340 0
EN 360 0
U-Y:ILE-S1
EN 20 -5112
EN 40 0
EN 60 -344
EN 80 -2353
EN 100 -2871
EN 120 -3144
EN 140 -217
EN 160 0
EN 180 0
EN 200 0
EN 220 -1840
EN 240 -1630
EN 260 386
EN 280 563
EN 300 353
EN 320 -2764
EN 340 -819
EN 360 0
IU-MY:ILE-S1

EN 20   0
EN 40   0
EN 60   0
EN 80   0
EN 100   0
EN 120   0
EN 140   0
EN 160   0
EN 180   0
EN 200   0
EN 220   0
EN 240   -13378
EN 260   0
EN 280   0
EN 300   0
EN 320   0
EN 340   0
EN 360   0
5BU-MY:ARG-S2
EN 20   0
EN 40   0
EN 60   0
EN 80   0
EN 100   0
EN 120   0
EN 140   0
EN 160   0
EN 180   0
EN 200   0
EN 220   0
EN 240   0
EN 260   -14650
EN 280   0
EN 300   0
EN 320   0
EN 340   0
EN 360   0
U-Y:LEU-S1
EN 20   0
EN 40   -2568
EN 60   -4031
EN 80   -213
EN 100   1698
EN 120   -2009
EN 140   917
EN 160   221
EN 180   -2094
EN 200   0
EN 220   0
EN 240   -2664
EN 260   -1912
EN 280   -1023
EN 300   -1946
EN 320   -624
EN 340   -1401
EN 360   0
A-RIB:TYR-S1
EN 20   0

EN 40   -2538
EN 60   -2614
EN 80   -3618
EN 100   -4428
EN 120   -1979
EN 140   -3492
EN 160   -1465
EN 180   -3781
EN 200   0
EN 220   0
EN 240   0
EN 260   -2887
EN 280   -3976
EN 300   -2921
EN 320   -4029
EN 340   -5359
EN 360   -3663
U-P:ASN-CA
EN 20   0
EN 40   -2827
EN 60   -2903
EN 80   -4460
EN 100   -4265
EN 120   -6256
EN 140   -6211
EN 160   -5189
EN 180   -4070
EN 200   0
EN 220   0
EN 240   -2923
EN 260   -4893
EN 280   -4717
EN 300   -4927
EN 320   -3605
EN 340   -4382
EN 360   0
G-R5:ASN-CA
EN 20   0
EN 40   -5483
EN 60   -4762
EN 80   -1862
EN 100   -2934
EN 120   -2654
EN 140   -3715
EN 160   -2140
EN 180   -4125
EN 200   -4877
EN 220   -4785
EN 240   -4948
EN 260   -4196
EN 280   0
EN 300   -2590
EN 320   -1269
EN 340   -3051
EN 360   -1907
C-P:ASN-S2
EN 20   0
EN 40   -4651

EN 60 -5440
EN 80 -1291
EN 100 -5918
EN 120 -3641
EN 140 -6516
EN 160 -6008
EN 180 -5894
EN 200 0
EN 220 -1784
EN 240 -6013
EN 260 -4708
EN 280 -6675
EN 300 -6128
EN 320 -5137
EN 340 -6919
EN 360 -5776
G-R5:ALA-S1
EN 20 0
EN 40 -4823
EN 60 -752
EN 80 -751
EN 100 -2195
EN 120 2604
EN 140 -1338
EN 160 -316
EN 180 -3636
EN 200 -4770
EN 220 -4678
EN 240 -3494
EN 260 -2742
EN 280 -1109
EN 300 -766
EN 320 -449
EN 340 1494
EN 360 -796
A-R6:ILE-CA
EN 20 0
EN 40 -1218
EN 60 -1295
EN 80 -581
EN 100 0
EN 120 -660
EN 140 -2172
EN 160 -1863
EN 180 -2462
EN 200 0
EN 220 0
EN 240 402
EN 260 -563
EN 280 -1391
EN 300 -2314
EN 320 724
EN 340 -1769
EN 360 -2343
C-Y:TYR-S1
EN 20 0
EN 40 0
EN 60 -2457

EN 80 -3461
EN 100 -1549
EN 120 -3540
EN 140 -613
EN 160 0
EN 180 0
EN 200 0
EN 220 0
EN 240 -2478
EN 260 -3443
EN 280 -2554
EN 300 -4029
EN 320 -2155
EN 340 -1215
EN 360 0
FMU-RIB:ILE-CA
EN 20 0
EN 40 0
EN 60 0
EN 80 0
EN 100 0
EN 120 0
EN 140 0
EN 160 0
EN 180 0
EN 200 0
EN 220 0
EN 240 0
EN 260 -16850
EN 280 0
EN 300 0
EN 320 0
EN 340 0
EN 360 0
G-R6:ASN-CA
EN 20 0
EN 40 0
EN 60 -4394
EN 80 -4292
EN 100 -2381
EN 120 -4293
EN 140 -1444
EN 160 -1809
EN 180 0
EN 200 0
EN 220 -4072
EN 240 -3309
EN 260 -2397
EN 280 -3677
EN 300 -3595
EN 320 -2986
EN 340 -329
EN 360 -2620
U-Y:LEU-S2
EN 20 0
EN 40 -2571
EN 60 -2647
EN 80 -1221

EN 100   -1739
EN 120   -295
EN 140   -1808
EN 160   218
EN 180   -2097
EN 200   0
EN 220   -708
EN 240   -4054
EN 260   -1203
EN 280   -22
EN 300   1485
EN 320   -627
EN 340   312
EN 360   -1979
A-RIB:TYR-S2
EN 20   0
EN 40   -4260
EN 60   -2618
EN 80   -5009
EN 100   -3981
EN 120   -4706
EN 140   -3496
EN 160   -3187
EN 180   -3786
EN 200   0
EN 220   -2397
EN 240   -2639
EN 260   -5322
EN 280   -2715
EN 300   -4642
EN 320   -4033
EN 340   0
EN 360   0
U-RIB:TRP-CA
EN 20   0
EN 40   0
EN 60   -6584
EN 80   -3148
EN 100   0
EN 120   -3227
EN 140   -3735
EN 160   -4430
EN 180   0
EN 200   0
EN 220   -5358
EN 240   -5600
EN 260   -4848
EN 280   -2954
EN 300   -4881
EN 320   0
EN 340   -4337
EN 360   0
A-R6:PRO-S1
EN 20   0
EN 40   -1675
EN 60   -1751
EN 80   -3307
EN 100   -4830

EN 120   -4220
EN 140   -4728
EN 160   -4589
EN 180   -2918
EN 200   0
EN 220   -4251
EN 240   -4493
EN 260   -2024
EN 280   -4569
EN 300   -3669
EN 320   -4170
EN 340   -508
EN 360   -2800
G-P:GLN-CA
EN 20   0
EN 40   -1765
EN 60   -2846
EN 80   -2845
EN 100   -3204
EN 120   -4641
EN 140   -5149
EN 160   -3414
EN 180   -5731
EN 200   0
EN 220   -1620
EN 240   -4132
EN 260   -3832
EN 280   -5157
EN 300   -4578
EN 320   -5266
EN 340   -6043
EN 360   -5612
H2U-RIB:ASN-S2
EN 20   0
EN 40   0
EN 60   0
EN 80   0
EN 100   0
EN 120   0
EN 140   0
EN 160   0
EN 180   0
EN 200   0
EN 220   -15947
EN 240   0
EN 260   -13719
EN 280   0
EN 300   -13753
EN 320   0
EN 340   0
EN 360   0
C31-P:MET-S2
EN 20   0
EN 40   0
EN 60   0
EN 80   0
EN 100   0
EN 120   0

EN 140 0
EN 160 0
EN 180 0
EN 200 0
EN 220 0
EN 240 0
EN 260 0
EN 280 0
EN 300 0
EN 320 -19275
EN 340 0
EN 360 0
5BU-P:SER-CA
EN 20 0
EN 40 0
EN 60 0
EN 80 0
EN 100 0
EN 120 0
EN 140 0
EN 160 0
EN 180 0
EN 200 0
EN 220 0
EN 240 0
EN 260 0
EN 280 0
EN 300 -15183
EN 320 0
EN 340 0
EN 360 0
U34-P:ASN-S2
EN 20 0
EN 40 0
EN 60 0
EN 80 0
EN 100 -17531
EN 120 0
EN 140 0
EN 160 0
EN 180 0
EN 200 0
EN 220 0
EN 240 -18459
EN 260 -17707
EN 280 0
EN 300 0
EN 320 0
EN 340 0
EN 360 0
A-R5:HIS-CA
EN 20 -6397
EN 40 -4988
EN 60 -4351
EN 80 -5355
EN 100 -4156
EN 120 -5434
EN 140 0

EN 160   -2198
EN 180   0
EN 200   0
EN 220   -6560
EN 240   -5637
EN 260   -4332
EN 280   -2439
EN 300   -2648
EN 320   -5314
EN 340   -2104
EN 360   0
G-R5:ASP-CA
EN 20   -3539
EN 40   -2130
EN 60   -2759
EN 80   -1332
EN 100   -585
EN 120   0
EN 140   -2079
EN 160   -2774
EN 180   0
EN 200   -3794
EN 220   -3702
EN 240   -2779
EN 260   -1474
EN 280   -1298
EN 300   -2512
EN 320   -186
EN 340   -1968
EN 360   0
G-RIB:TYR-S1
EN 20   0
EN 40   0
EN 60   -2054
EN 80   -1341
EN 100   -1146
EN 120   -2424
EN 140   -210
EN 160   -3627
EN 180   0
EN 200   0
EN 220   -1833
EN 240   -357
EN 260   -3593
EN 280   -3868
EN 300   -1356
EN 320   0
EN 340   -4246
EN 360   0
G-RIB:ASP-CA
EN 20   -3539
EN 40   -412
EN 60   -2206
EN 80   -1492
EN 100   -3015
EN 120   -4293
EN 140   -3466
EN 160   -4161

EN 180   -5090
EN 200   -3794
EN 220   -1984
EN 240   -509
EN 260   -3192
EN 280   -2302
EN 300   -2512
EN 320   -2908
EN 340   -4067
EN 360   -3255
G-R5:TRP-CA
EN 20   -7303
EN 40   -5893
EN 60   0
EN 80   -1821
EN 100   -5062
EN 120   -3617
EN 140   0
EN 160   0
EN 180   0
EN 200   0
EN 220   -7466
EN 240   0
EN 260   -1803
EN 280   -1627
EN 300   -4558
EN 320   0
EN 340   0
EN 360   0
C-Y:TRP-CA
EN 20   0
EN 40   0
EN 60   -2938
EN 80   -1220
EN 100   -4460
EN 120   0
EN 140   -2811
EN 160   0
EN 180   0
EN 200   0
EN 220   0
EN 240   -2958
EN 260   0
EN 280   -2030
EN 300   0
EN 320   -2635
EN 340   -3413
EN 360   0
A-P:ARG-S2
EN 20   0
EN 40   -7277
EN 60   -6259
EN 80   -6811
EN 100   -6853
EN 120   -6231
EN 140   -6845
EN 160   -7922
EN 180   -6803

EN 200 0
EN 220 -5415
EN 240 -6138
EN 260 -6622
EN 280 -6853
EN 300 -6058
EN 320 -6669
EN 340 -7231
EN 360 -5851
FHU-MY:ASP-S2
EN 20 0
EN 40 0
EN 60 0
EN 80 0
EN 100 -14757
EN 120 0
EN 140 0
EN 160 0
EN 180 0
EN 200 0
EN 220 0
EN 240 0
EN 260 0
EN 280 -14757
EN 300 -14967
EN 320 0
EN 340 0
EN 360 0
IU-P:LYS-CA
EN 20 0
EN 40 0
EN 60 0
EN 80 0
EN 100 -13377
EN 120 0
EN 140 0
EN 160 0
EN 180 0
EN 200 0
EN 220 -15781
EN 240 0
EN 260 0
EN 280 0
EN 300 0
EN 320 0
EN 340 0
EN 360 0
U-Y:VAL-CA
EN 20 0
EN 40 -3005
EN 60 353
EN 80 -650
EN 100 -2173
EN 120 0
EN 140 -1237
EN 160 -215
EN 180 0
EN 200 0

EN 220   0
EN 240   -3101
EN 260   -1637
EN 280   -2726
EN 300   1051
EN 320   -2066
EN 340   -1838
EN 360   0
A-R6:HIS-CA
EN 20   0
EN 40   0
EN 60   -5617
EN 80   -4903
EN 100   -2439
EN 120   -4982
EN 140   -3220
EN 160   -4920
EN 180   0
EN 200   0
EN 220   0
EN 240   -6089
EN 260   -5337
EN 280   -4156
EN 300   -2648
EN 320   -4602
EN 340   -5539
EN 360   0
A-R6:VAL-S1
EN 20   0
EN 40   -2240
EN 60   -1603
EN 80   -3873
EN 100   -2413
EN 120   -2686
EN 140   -2189
EN 160   -2884
EN 180   0
EN 200   0
EN 220   -2095
EN 240   -1623
EN 260   -2137
EN 280   -695
EN 300   -3627
EN 320   -1301
EN 340   -1073
EN 360   0
G-R5:TRP-S2
EN 20   -7303
EN 40   -4176
EN 60   0
EN 80   -1821
EN 100   -5062
EN 120   -5888
EN 140   -2408
EN 160   -3103
EN 180   0
EN 200   0
EN 220   -4031

EN 240 -4272
EN 260 -3520
EN 280 0
EN 300 -4558
EN 320 -2232
EN 340 0
EN 360 0
FMU-RIB:ARG-S1
EN 20 0
EN 40 0
EN 60 0
EN 80 0
EN 100 0
EN 120 -16452
EN 140 0
EN 160 0
EN 180 0
EN 200 0
EN 220 0
EN 240 -17107
EN 260 0
EN 280 0
EN 300 0
EN 320 0
EN 340 0
EN 360 0
FHU-MY:LEU-S1
EN 20 0
EN 40 0
EN 60 0
EN 80 -14015
EN 100 0
EN 120 0
EN 140 -14602
EN 160 -15297
EN 180 0
EN 200 0
EN 220 0
EN 240 0
EN 260 -13997
EN 280 0
EN 300 0
EN 320 0
EN 340 -15204
EN 360 0
G-RIB:ASP-S2
EN 20 0
EN 40 -460
EN 60 -3971
EN 80 -4261
EN 100 -4265
EN 120 -4125
EN 140 -2126
EN 160 -4831
EN 180 -4425
EN 200 -3841
EN 220 0
EN 240 -3278

EN 260 -3239
EN 280 -3615
EN 300 -2559
EN 320 -4673
EN 340 -4998
EN 360 -6024
C-RIB:GLN-S2
EN 20 -5299
EN 40 -7324
EN 60 -6472
EN 80 -5522
EN 100 -5067
EN 120 -5340
EN 140 -3126
EN 160 -7256
EN 180 -3415
EN 200 0
EN 220 -3744
EN 240 -4539
EN 260 -5243
EN 280 -5328
EN 300 -4985
EN 320 -5933
EN 340 -7875
EN 360 -8118
C-P:ARG-S2
EN 20 -3716
EN 40 -7299
EN 60 -6109
EN 80 -6971
EN 100 -7472
EN 120 -7122
EN 140 -7329
EN 160 -7859
EN 180 -7276
EN 200 0
EN 220 -4883
EN 240 -5678
EN 260 -7025
EN 280 -7416
EN 300 -6322
EN 320 -7154
EN 340 -7081
EN 360 -6153
H2U-MY:LEU-S1
EN 20 0
EN 40 0
EN 60 0
EN 80 0
EN 100 0
EN 120 0
EN 140 0
EN 160 -13027
EN 180 0
EN 200 0
EN 220 0
EN 240 0
EN 260 0

EN 280 0
EN 300 0
EN 320 -12156
EN 340 0
EN 360 0
FMU-RIB:CYS-CA
EN 20 0
EN 40 0
EN 60 0
EN 80 0
EN 100 0
EN 120 0
EN 140 0
EN 160 -22162
EN 180 0
EN 200 0
EN 220 0
EN 240 0
EN 260 0
EN 280 0
EN 300 0
EN 320 0
EN 340 0
EN 360 0
DA-M5:GLU-S1
EN 20 0
EN 40 0
EN 60 0
EN 80 0
EN 100 0
EN 120 0
EN 140 0
EN 160 0
EN 180 0
EN 200 0
EN 220 0
EN 240 0
EN 260 0
EN 280 0
EN 300 0
EN 320 -16348
EN 340 0
EN 360 0
FMU-RIB:PHE-CA
EN 20 0
EN 40 0
EN 60 0
EN 80 0
EN 100 0
EN 120 0
EN 140 0
EN 160 0
EN 180 0
EN 200 0
EN 220 -20034
EN 240 0
EN 260 0
EN 280 0

EN 300 0
EN 320 0
EN 340 0
EN 360 0
G-R5:VAL-S1
EN 20 0
EN 40 -1680
EN 60 1678
EN 80 -1042
EN 100 -135
EN 120 -408
EN 140 1805
EN 160 0
EN 180 -2923
EN 200 -5062
EN 220 -3252
EN 240 -1776
EN 260 139
EN 280 -135
EN 300 -345
EN 320 -741
EN 340 0
EN 360 0
QUO-M6:ASN-S1
EN 20 0
EN 40 0
EN 60 0
EN 80 -17704
EN 100 0
EN 120 0
EN 140 0
EN 160 0
EN 180 0
EN 200 0
EN 220 0
EN 240 0
EN 260 0
EN 280 0
EN 300 0
EN 320 0
EN 340 0
EN 360 0
U-P:PRO-S1
EN 20 0
EN 40 -2446
EN 60 -3527
EN 80 -4079
EN 100 -4337
EN 120 -5323
EN 140 -5500
EN 160 -5361
EN 180 -8129
EN 200 0
EN 220 -4019
EN 240 -4813
EN 260 -3508
EN 280 -4718
EN 300 -4095

EN 320 -3937
EN 340 -2997
EN 360 -5289
G-R6:ARG-CA
EN 20 0
EN 40 -1881
EN 60 -1957
EN 80 -2248
EN 100 -2767
EN 120 -1876
EN 140 -2835
EN 160 -2526
EN 180 -1407
EN 200 0
EN 220 -4006
EN 240 -2531
EN 260 -3235
EN 280 -1602
EN 300 -3530
EN 320 -2208
EN 340 -2985
EN 360 0
A-R5:ALA-CA
EN 20 -3358
EN 40 -2953
EN 60 -1312
EN 80 -598
EN 100 -404
EN 120 -1943
EN 140 -1185
EN 160 -2593
EN 180 0
EN 200 -5330
EN 220 -2808
EN 240 -328
EN 260 -1846
EN 280 -404
EN 300 -1879
EN 320 -5
EN 340 934
EN 360 -1356
FMU-P:ALA-CA
EN 20 0
EN 40 0
EN 60 0
EN 80 0
EN 100 0
EN 120 0
EN 140 0
EN 160 0
EN 180 0
EN 200 0
EN 220 0
EN 240 -16614
EN 260 0
EN 280 0
EN 300 0
EN 320 0

EN 340   0
EN 360   0
FHU-RIB:THR-S1
EN 20   0
EN 40   0
EN 60   0
EN 80   -15407
EN 100   0
EN 120   0
EN 140   0
EN 160   0
EN 180   0
EN 200   0
EN 220   0
EN 240   0
EN 260   -15389
EN 280   -15213
EN 300   0
EN 320   0
EN 340   0
EN 360   0
QUO-M6:PHE-S2
EN 20   0
EN 40   0
EN 60   0
EN 80   0
EN 100   0
EN 120   0
EN 140   0
EN 160   0
EN 180   0
EN 200   0
EN 220   0
EN 240   0
EN 260   -17810
EN 280   0
EN 300   -17844
EN 320   0
EN 340   0
EN 360   0
FMU-MY:PRO-CA
EN 20   0
EN 40   0
EN 60   0
EN 80   0
EN 100   0
EN 120   0
EN 140   -17911
EN 160   0
EN 180   0
EN 200   0
EN 220   0
EN 240   0
EN 260   0
EN 280   0
EN 300   0
EN 320   0
EN 340   0

EN 360 0
U-Y:THR-CA
EN 20 0
EN 40 -3960
EN 60 -4036
EN 80 -2610
EN 100 -2415
EN 120 -3954
EN 140 -474
EN 160 0
EN 180 -3486
EN 200 -5624
EN 220 0
EN 240 -622
EN 260 -2853
EN 280 -1411
EN 300 -1620
EN 320 -2016
EN 340 -2793
EN 360 0
A-R5:ASP-CA
EN 20 -5816
EN 40 -4407
EN 60 668
EN 80 -335
EN 100 1576
EN 120 -1418
EN 140 -2639
EN 160 -1617
EN 180 0
EN 200 -4354
EN 220 0
EN 240 -1069
EN 260 -2034
EN 280 -2411
EN 300 1366
EN 320 -2463
EN 340 -1523
EN 360 0
C-Y:TYR-CA
EN 20 0
EN 40 0
EN 60 -740
EN 80 -4014
EN 100 -1549
EN 120 -1823
EN 140 -613
EN 160 -3026
EN 180 0
EN 200 0
EN 220 -2236
EN 240 -2478
EN 260 -4448
EN 280 167
EN 300 -3477
EN 320 -438
EN 340 -1215
EN 360 0

G-R5:MET-S2
EN 20 -8504
EN 40 -4372
EN 60 -3736
EN 80 -4288
EN 100 0
EN 120 -379
EN 140 -887
EN 160 -1582
EN 180 0
EN 200 -6037
EN 220 -2510
EN 240 -2751
EN 260 -4721
EN 280 -4545
EN 300 0
EN 320 -2428
EN 340 -1488
EN 360 0
G-R6:TYR-CA
EN 20 0
EN 40 0
EN 60 0
EN 80 376
EN 100 -3289
EN 120 297
EN 140 -210
EN 160 -192
EN 180 -3221
EN 200 0
EN 220 0
EN 240 0
EN 260 -2112
EN 280 570
EN 300 -2361
EN 320 -2756
EN 340 0
EN 360 -4820
G-R5:GLN-S1
EN 20 0
EN 40 -6560
EN 60 -5802
EN 80 -3823
EN 100 810
EN 120 537
EN 140 0
EN 160 -665
EN 180 0
EN 200 -7842
EN 220 -6414
EN 240 -4105
EN 260 -5522
EN 280 -1911
EN 300 -2121
EN 320 0
EN 340 -572
EN 360 0
G-R6:GLU-CA

EN 20   0
EN 40   -2443
EN 60   202
EN 80   -801
EN 100   105
EN 120   47
EN 140   2046
EN 160   -2083
EN 180   -2682
EN 200   0
EN 220   423
EN 240   -822
EN 260   -1682
EN 280   1110
EN 300   -817
EN 320   -500
EN 340   -2994
EN 360   -2233
IU-RIB:GLN-S2
EN 20   0
EN 40   0
EN 60   0
EN 80   0
EN 100   0
EN 120   0
EN 140   0
EN 160   0
EN 180   0
EN 200   0
EN 220   0
EN 240   0
EN 260   0
EN 280   0
EN 300   -14774
EN 320   0
EN 340   0
EN 360   0
U-P:ARG-S2
EN 20   -6352
EN 40   -5947
EN 60   -5808
EN 80   -6023
EN 100   -6934
EN 120   -5942
EN 140   -7812
EN 160   -7858
EN 180   -8195
EN 200   0
EN 220   -4798
EN 240   -4326
EN 260   -7562
EN 280   -6833
EN 300   -6188
EN 320   -5008
EN 340   -7764
EN 360   -8790
DA-M6:THR-S1
EN 20   0

EN 40 0
EN 60 0
EN 80 0
EN 100 0
EN 120 0
EN 140 0
EN 160 0
EN 180 0
EN 200 0
EN 220 0
EN 240 0
EN 260 0
EN 280 -16930
EN 300 0
EN 320 0
EN 340 0
EN 360 0
U-Y:ARG-CA
EN 20 -4618
EN 40 -1491
EN 60 -3837
EN 80 -5293
EN 100 -4094
EN 120 -3203
EN 140 -4162
EN 160 -418
EN 180 -2734
EN 200 0
EN 220 -3063
EN 240 -1588
EN 260 -835
EN 280 -1664
EN 300 -3139
EN 320 -2269
EN 340 -4312
EN 360 0
H2U-MY:PRO-CA
EN 20 0
EN 40 0
EN 60 0
EN 80 0
EN 100 -14859
EN 120 0
EN 140 -13923
EN 160 0
EN 180 0
EN 200 0
EN 220 0
EN 240 0
EN 260 0
EN 280 0
EN 300 0
EN 320 0
EN 340 0
EN 360 0
G-P:HIS-S1
EN 20 0
EN 40 -4455

EN 60   -3818
EN 80   -5827
EN 100   -5010
EN 120   -4901
EN 140   -4957
EN 160   -1665
EN 180   -3981
EN 200   0
EN 220   -6027
EN 240   -1117
EN 260   -4352
EN 280   -4176
EN 300   -6103
EN 320   -3516
EN 340   -5559
EN 360   -3863
A-R5:LEU-CA
EN 20   -4927
EN 40   -83
EN 60   -1164
EN 80   -2550
EN 100   -1974
EN 120   -1242
EN 140   1684
EN 160   989
EN 180   0
EN 200   0
EN 220   61
EN 240   1537
EN 260   -1145
EN 280   748
EN 300   -1732
EN 320   142
EN 340   -1639
EN 360   0
A-RIB:PHE-CA
EN 20   0
EN 40   0
EN 60   -2252
EN 80   -3808
EN 100   -1344
EN 120   -3334
EN 140   -3129
EN 160   -2820
EN 180   -3419
EN 200   0
EN 220   0
EN 240   -554
EN 260   -2524
EN 280   -3061
EN 300   -3271
EN 320   -4671
EN 340   -2726
EN 360   -3300
H2U-P:ASN-S2
EN 20   0
EN 40   0
EN 60   0

EN 80 0
EN 100 -13543
EN 120 -13816
EN 140 -14324
EN 160 0
EN 180 0
EN 200 0
EN 220 0
EN 240 0
EN 260 0
EN 280 0
EN 300 0
EN 320 0
EN 340 0
EN 360 0
A-RIB:ASN-S2
EN 20 0
EN 40 -2076
EN 60 -2152
EN 80 -5878
EN 100 -6065
EN 120 -3235
EN 140 -5752
EN 160 -6155
EN 180 -7759
EN 200 -5458
EN 220 -4653
EN 240 -5607
EN 260 -5860
EN 280 -3966
EN 300 -4176
EN 320 -4120
EN 340 -6353
EN 360 -5923
DA-M6:TYR-CA
EN 20 0
EN 40 0
EN 60 0
EN 80 0
EN 100 0
EN 120 -18266
EN 140 0
EN 160 0
EN 180 0
EN 200 0
EN 220 0
EN 240 0
EN 260 0
EN 280 0
EN 300 -18203
EN 320 0
EN 340 0
EN 360 0
FHU-RIB:ASP-S2
EN 20 0
EN 40 0
EN 60 0
EN 80 0

EN 100   0
EN 120   0
EN 140   0
EN 160   -16234
EN 180   0
EN 200   0
EN 220   0
EN 240   0
EN 260   0
EN 280   0
EN 300   0
EN 320   0
EN 340   -16140
EN 360   0
U-P:VAL-CA
EN 20   0
EN 40   0
EN 60   -1368
EN 80   -2372
EN 100   -3564
EN 120   -734
EN 140   -3512
EN 160   -1937
EN 180   -2536
EN 200   0
EN 220   -1147
EN 240   -3106
EN 260   -1641
EN 280   -4187
EN 300   -4397
EN 320   -2783
EN 340   -2848
EN 360   -2417
A-RIB:LYS-S2
EN 20   -7259
EN 40   -6855
EN 60   -6600
EN 80   -4048
EN 100   -5444
EN 120   -4127
EN 140   -4635
EN 160   -4064
EN 180   -3659
EN 200   -9524
EN 220   -7262
EN 240   -6951
EN 260   -5345
EN 280   -5571
EN 300   -5068
EN 320   -4198
EN 340   -6551
EN 360   -5258
H2U-MY:LYS-S1
EN 20   0
EN 40   0
EN 60   0
EN 80   0
EN 100   0

EN 120   0
EN 140   0
EN 160   0
EN 180   0
EN 200   0
EN 220   0
EN 240   0
EN 260   -12294
EN 280   0
EN 300   0
EN 320   0
EN 340   0
EN 360   0
U-P:TRP-S2
EN 20   0
EN 40   0
EN 60   0
EN 80   -6588
EN 100   -2959
EN 120   -4949
EN 140   -3740
EN 160   -4435
EN 180   0
EN 200   0
EN 220   -7080
EN 240   -3887
EN 260   -3135
EN 280   -5680
EN 300   0
EN 320   -6286
EN 340   -7063
EN 360   0
H2U-MY:ASN-S2
EN 20   0
EN 40   0
EN 60   0
EN 80   0
EN 100   -13543
EN 120   0
EN 140   -14324
EN 160   0
EN 180   0
EN 200   0
EN 220   0
EN 240   0
EN 260   -15437
EN 280   0
EN 300   0
EN 320   0
EN 340   0
EN 360   0
U-Y:PRO-CA
EN 20   0
EN 40   -4159
EN 60   -5240
EN 80   -4075
EN 100   107
EN 120   -166

EN 140 -3396
EN 160 -1369
EN 180 -3685
EN 200 0
EN 220 0
EN 240 -3543
EN 260 -2791
EN 280 -3327
EN 300 -4542
EN 320 -2215
EN 340 -3997
EN 360 -3567
G-R5:PRO-S1
EN 20 -8229
EN 40 -4549
EN 60 -4917
EN 80 -2747
EN 100 -2000
EN 120 -2273
EN 140 652
EN 160 -1759
EN 180 0
EN 200 -6214
EN 220 -6911
EN 240 -4225
EN 260 -2729
EN 280 -2000
EN 300 -2763
EN 320 828
EN 340 -2670
EN 360 0
G-R6:PHE-S1
EN 20 0
EN 40 0
EN 60 25
EN 80 -978
EN 100 933
EN 120 -2774
EN 140 152
EN 160 -543
EN 180 0
EN 200 0
EN 220 -3188
EN 240 -1712
EN 260 -1965
EN 280 933
EN 300 -1782
EN 320 0
EN 340 0
EN 360 0
U31-P:SER-S1
EN 20 0
EN 40 0
EN 60 0
EN 80 0
EN 100 0
EN 120 0
EN 140 0

EN 160   0
EN 180   0
EN 200   0
EN 220   0
EN 240   0
EN 260   0
EN 280   0
EN 300   -16900
EN 320   0
EN 340   0
EN 360   0
G-R6:PHE-S2
EN 20   -4746
EN 40   0
EN 60   -1695
EN 80   -2699
EN 100   -2505
EN 120   656
EN 140   148
EN 160   -546
EN 180   0
EN 200   0
EN 220   -1474
EN 240   -3433
EN 260   -964
EN 280   -1576
EN 300   719
EN 320   0
EN 340   259
EN 360   0
C-Y:PHE-CA
EN 20   0
EN 40   0
EN 60   -2095
EN 80   335
EN 100   -2904
EN 120   256
EN 140   -251
EN 160   -2663
EN 180   0
EN 200   0
EN 220   -1873
EN 240   0
EN 260   353
EN 280   -1187
EN 300   320
EN 320   -75
EN 340   -852
EN 360   -3144
U-RIB:THR-S1
EN 20   -8804
EN 40   -5677
EN 60   -3323
EN 80   -5040
EN 100   -5137
EN 120   -3401
EN 140   -4914
EN 160   -1170

EN 180   -6208
EN 200   0
EN 220   -3815
EN 240   -3344
EN 260   -2592
EN 280   -3681
EN 300   -3891
EN 320   -5451
EN 340   -2793
EN 360   0
FMU-RIB:ARG-S2
EN 20   0
EN 40   0
EN 60   0
EN 80   0
EN 100   -16191
EN 120   0
EN 140   0
EN 160   0
EN 180   0
EN 200   0
EN 220   -18595
EN 240   0
EN 260   0
EN 280   -16191
EN 300   0
EN 320   0
EN 340   0
EN 360   0
U-Y:GLN-S2
EN 20   -6223
EN 40   -4813
EN 60   -6276
EN 80   -3463
EN 100   -3269
EN 120   -4255
EN 140   -5767
EN 160   -5242
EN 180   -4339
EN 200   -9200
EN 220   -2951
EN 240   -4910
EN 260   -5544
EN 280   -5247
EN 300   -4191
EN 320   -1152
EN 340   -3647
EN 360   -4221
G-RIB:TRP-S2
EN 20   0
EN 40   0
EN 60   -2535
EN 80   -4543
EN 100   -4349
EN 120   -4622
EN 140   -2408
EN 160   -3103
EN 180   -5419

EN 200   0
EN 220   0
EN 240   -2555
EN 260   -4525
EN 280   -1627
EN 300   -5824
EN 320   -4954
EN 340   -3009
EN 360   0
A-R5:ILE-S1
EN 20   0
EN 40   -2936
EN 60   -2299
EN 80   1136
EN 100   0
EN 120   -660
EN 140   549
EN 160   0
EN 180   0
EN 200   -4600
EN 220   0
EN 240   -2320
EN 260   -2280
EN 280   0
EN 300   -1601
EN 320   -992
EN 340   -1769
EN 360   0
A-R6:MET-S1
EN 20   0
EN 40   0
EN 60   -4285
EN 80   -3571
EN 100   -2372
EN 120   -3650
EN 140   -4158
EN 160   -3848
EN 180   0
EN 200   0
EN 220   0
EN 240   -3300
EN 260   0
EN 280   -3376
EN 300   -4852
EN 320   -1260
EN 340   -2037
EN 360   -4329
U-RIB:HIS-CA
EN 20   0
EN 40   -4038
EN 60   -2396
EN 80   -5670
EN 100   -7430
EN 120   -4484
EN 140   -3987
EN 160   -2965
EN 180   0
EN 200   0

EN 220   0
EN 240   0
EN 260   -4387
EN 280   -4210
EN 300   -3416
EN 320   -4816
EN 340   -6306
EN 360   -5163
G-RIB:GLN-CA
EN 20   0
EN 40   -3456
EN 60   -4085
EN 80   -5088
EN 100   -4010
EN 120   -4614
EN 140   -5911
EN 160   -5818
EN 180   -6416
EN 200   -5120
EN 220   -3310
EN 240   -1835
EN 260   -3805
EN 280   -906
EN 300   -2833
EN 320   -4234
EN 340   -5393
EN 360   -2863
IU-MY:PRO-CA
EN 20   0
EN 40   0
EN 60   0
EN 80   -14105
EN 100   0
EN 120   0
EN 140   0
EN 160   0
EN 180   0
EN 200   0
EN 220   0
EN 240   0
EN 260   0
EN 280   -12906
EN 300   0
EN 320   0
EN 340   0
EN 360   0
G-R6:ILE-S1
EN 20   -3785
EN 40   0
EN 60   982
EN 80   0
EN 100   -831
EN 120   -100
EN 140   0
EN 160   413
EN 180   0
EN 200   0
EN 220   0

EN 240   -755
EN 260   327
EN 280   173
EN 300   294
EN 320   0
EN 340   0
EN 360   -1783
GTP-M6:SER-CA
EN 20   0
EN 40   0
EN 60   0
EN 80   0
EN 100   0
EN 120   0
EN 140   0
EN 160   0
EN 180   0
EN 200   0
EN 220   0
EN 240   0
EN 260   -16867
EN 280   0
EN 300   0
EN 320   0
EN 340   0
EN 360   0
FHU-RIB:SER-S1
EN 20   0
EN 40   0
EN 60   0
EN 80   0
EN 100   -16691
EN 120   0
EN 140   -15754
EN 160   0
EN 180   0
EN 200   0
EN 220   0
EN 240   0
EN 260   0
EN 280   0
EN 300   0
EN 320   -15579
EN 340   0
EN 360   0
FMU-MY:HIS-S1
EN 20   0
EN 40   0
EN 60   0
EN 80   0
EN 100   0
EN 120   0
EN 140   0
EN 160   0
EN 180   0
EN 200   0
EN 220   0
EN 240   -19654

EN 260   0
EN 280   0
EN 300   0
EN 320   0
EN 340   0
EN 360   0
A-P:GLU-CA
EN 20   0
EN 40   -3006
EN 60   -360
EN 80   -1364
EN 100   -1722
EN 120   -730
EN 140   -1238
EN 160   -2646
EN 180   0
EN 200   0
EN 220   -1856
EN 240   -2098
EN 260   -2350
EN 280   -1722
EN 300   -2384
EN 320   -2328
EN 340   0
EN 360   0
U31-MY:THR-CA
EN 20   0
EN 40   0
EN 60   0
EN 80   0
EN 100   0
EN 120   0
EN 140   0
EN 160   0
EN 180   -20723
EN 200   0
EN 220   0
EN 240   0
EN 260   0
EN 280   0
EN 300   0
EN 320   -17536
EN 340   0
EN 360   0
U31-RIB:GLN-S2
EN 20   0
EN 40   0
EN 60   -18692
EN 80   0
EN 100   0
EN 120   0
EN 140   0
EN 160   0
EN 180   0
EN 200   0
EN 220   0
EN 240   0
EN 260   0

EN 280   0
EN 300   0
EN 320   0
EN 340   0
EN 360   0
QUO-P:SER-S1
EN 20   0
EN 40   0
EN 60   0
EN 80   0
EN 100   0
EN 120   -16964
EN 140   0
EN 160   0
EN 180   0
EN 200   0
EN 220   0
EN 240   0
EN 260   0
EN 280   0
EN 300   0
EN 320   0
EN 340   0
EN 360   0
A-RIB:GLU-S1
EN 20   0
EN 40   -288
EN 60   1352
EN 80   -2754
EN 100   -3680
EN 120   -2833
EN 140   -3964
EN 160   -933
EN 180   -1531
EN 200   0
EN 220   0
EN 240   1332
EN 260   -2355
EN 280   -1726
EN 300   -2388
EN 320   -2332
EN 340   -3109
EN 360   -3130
A-R5:TRP-S2
EN 20   0
EN 40   0
EN 60   -6530
EN 80   0
EN 100   -4909
EN 120   -2460
EN 140   0
EN 160   -5381
EN 180   0
EN 200   0
EN 220   -4591
EN 240   -4832
EN 260   -2363
EN 280   -4909

EN 300 -2396
EN 320 -2792
EN 340 0
EN 360 0
FHU-MY:THR-S1
EN 20 0
EN 40 0
EN 60 0
EN 80 0
EN 100 -15213
EN 120 0
EN 140 0
EN 160 0
EN 180 0
EN 200 0
EN 220 -17617
EN 240 0
EN 260 0
EN 280 -16930
EN 300 0
EN 320 0
EN 340 0
EN 360 0
IU-MY:LYS-CA
EN 20 0
EN 40 0
EN 60 -13788
EN 80 0
EN 100 -11876
EN 120 0
EN 140 -14927
EN 160 0
EN 180 0
EN 200 0
EN 220 0
EN 240 0
EN 260 -12052
EN 280 0
EN 300 0
EN 320 0
EN 340 0
EN 360 0
FHU-P:VAL-S1
EN 20 0
EN 40 0
EN 60 0
EN 80 0
EN 100 0
EN 120 -14533
EN 140 0
EN 160 0
EN 180 0
EN 200 0
EN 220 0
EN 240 0
EN 260 0
EN 280 0
EN 300 0

EN 320   -14865
EN 340   0
EN 360   0
G-R5:TYR-CA
EN 20   0
EN 40   -3696
EN 60   0
EN 80   -2898
EN 100   0
EN 120   297
EN 140   -210
EN 160   -905
EN 180   -3221
EN 200   -5360
EN 220   0
EN 240   -2075
EN 260   -2327
EN 280   0
EN 300   -1025
EN 320   -3138
EN 340   0
EN 360   0
G-R6:ASP-S1
EN 20   0
EN 40   -3849
EN 60   -491
EN 80   -2499
EN 100   -1140
EN 120   -3291
EN 140   -1368
EN 160   -2064
EN 180   -1658
EN 200   0
EN 220   -2991
EN 240   -2228
EN 260   -2863
EN 280   -4022
EN 300   -2063
EN 320   -188
EN 340   -3687
EN 360   -3257
A-R5:ASP-S2
EN 20   -4146
EN 40   -3742
EN 60   -4531
EN 80   -3104
EN 100   -1905
EN 120   -1465
EN 140   -2686
EN 160   52
EN 180   -2263
EN 200   0
EN 220   -4862
EN 240   -3838
EN 260   -1369
EN 280   -2458
EN 300   -397
EN 320   -3063

EN 340   -1570
EN 360   -2144
A-R5:GLY-CA
EN 20   -8185
EN 40   -6974
EN 60   -4422
EN 80   -4974
EN 100   -3514
EN 120   -1065
EN 140   -2839
EN 160   -3534
EN 180   -1862
EN 200   -8822
EN 220   -5295
EN 240   -5708
EN 260   -5408
EN 280   -2891
EN 300   -4221
EN 320   -3497
EN 340   -2887
EN 360   -5179
FMU-P:ARG-S1
EN 20   0
EN 40   0
EN 60   0
EN 80   0
EN 100   -16179
EN 120   -16452
EN 140   0
EN 160   0
EN 180   0
EN 200   0
EN 220   0
EN 240   -17107
EN 260   0
EN 280   0
EN 300   0
EN 320   0
EN 340   0
EN 360   0
U-Y:ARG-S2
EN 20   -7352
EN 40   -6655
EN 60   -6401
EN 80   -5687
EN 100   -6115
EN 120   -4848
EN 140   -4557
EN 160   -3865
EN 180   -7186
EN 200   -10329
EN 220   -3075
EN 240   -5587
EN 260   -3570
EN 280   -4659
EN 300   -1886
EN 320   -4712
EN 340   -4776

EN 360   -2628
U-P:HIS-S1
EN 20   0
EN 40   -5760
EN 60   -5123
EN 80   -5122
EN 100   -3211
EN 120   -3484
EN 140   -5709
EN 160   -6404
EN 180   -7003
EN 200   0
EN 220   0
EN 240   -6409
EN 260   -6491
EN 280   -5481
EN 300   -4425
EN 320   -6086
EN 340   -5598
EN 360   0
C31-RIB:THR-CA
EN 20   0
EN 40   0
EN 60   0
EN 80   0
EN 100   0
EN 120   0
EN 140   0
EN 160   -18407
EN 180   0
EN 200   0
EN 220   0
EN 240   -17859
EN 260   0
EN 280   -16930
EN 300   0
EN 320   0
EN 340   0
EN 360   0
U-Y:HIS-S1
EN 20   0
EN 40   -5755
EN 60   -5831
EN 80   -1683
EN 100   -4210
EN 120   -1762
EN 140   -3987
EN 160   -2965
EN 180   0
EN 200   0
EN 220   -7327
EN 240   -2417
EN 260   0
EN 280   -3206
EN 300   -4420
EN 320   -2094
EN 340   -2871
EN 360   0

IU-RIB:ARG-S2
EN 20 0
EN 40 0
EN 60 0
EN 80 0
EN 100 -12972
EN 120 0
EN 140 0
EN 160 0
EN 180 0
EN 200 0
EN 220 0
EN 240 0
EN 260 0
EN 280 0
EN 300 0
EN 320 0
EN 340 0
EN 360 0
U-RIB:ILE-S1
EN 20 -5112
EN 40 0
EN 60 -344
EN 80 -1348
EN 100 -1154
EN 120 -2432
EN 140 -3652
EN 160 -2630
EN 180 0
EN 200 0
EN 220 -1840
EN 240 -2082
EN 260 -1330
EN 280 -2871
EN 300 0
EN 320 -1759
EN 340 -4254
EN 360 -3110
A-RIB:ASP-S2
EN 20 0
EN 40 0
EN 60 -1096
EN 80 -3817
EN 100 -2458
EN 120 -3183
EN 140 748
EN 160 -1664
EN 180 -4985
EN 200 0
EN 220 -3596
EN 240 -2833
EN 260 -3799
EN 280 -4175
EN 300 -4385
EN 320 -4781
EN 340 -4292
EN 360 0
A-P:CYS-S1

```
EN 20   0
EN 40   0
EN 60   -3592
EN 80   0
EN 100   -2684
EN 120   0
EN 140   0
EN 160   0
EN 180   -6477
EN 200   0
EN 220   0
EN 240   0
EN 260   -5583
EN 280   -4402
EN 300   -2894
EN 320   0
EN 340   0
EN 360   0
G-R5:HIS-S1
EN 20   -7555
EN 40   -7150
EN 60   -5509
EN 80   -4690
EN 100   -4601
EN 120   -3869
EN 140   -2660
EN 160   0
EN 180   0
EN 200   0
EN 220   -4283
EN 240   -3812
EN 260   -5069
EN 280   -1879
EN 300   -3806
EN 320   -4202
EN 340   0
EN 360   0
FHU-P:LYS-S2
EN 20   0
EN 40   0
EN 60   0
EN 80   0
EN 100   0
EN 120   -14709
EN 140   -15217
EN 160   0
EN 180   0
EN 200   0
EN 220   0
EN 240   0
EN 260   0
EN 280   0
EN 300   -14645
EN 320   0
EN 340   -15818
EN 360   0
U-P:GLN-S2
EN 20   -6227
```

EN 40   -4818
EN 60   -4894
EN 80   -4733
EN 100   -5996
EN 120   -4259
EN 140   -4055
EN 160   -4750
EN 180   -6061
EN 200   0
EN 220   -6390
EN 240   -4202
EN 260   -3450
EN 280   -5704
EN 300   -5913
EN 320   -4592
EN 340   -5369
EN 360   0
QUO-M5:LEU-S1
EN 20   0
EN 40   0
EN 60   0
EN 80   0
EN 100   0
EN 120   0
EN 140   0
EN 160   0
EN 180   0
EN 200   0
EN 220   0
EN 240   -16467
EN 260   0
EN 280   -15538
EN 300   0
EN 320   0
EN 340   0
EN 360   0
FMU-MY:CYS-CA
EN 20   0
EN 40   0
EN 60   0
EN 80   0
EN 100   0
EN 120   -20959
EN 140   0
EN 160   0
EN 180   0
EN 200   0
EN 220   0
EN 240   0
EN 260   0
EN 280   0
EN 300   0
EN 320   0
EN 340   0
EN 360   0
G-RIB:GLU-S2
EN 20   -2887
EN 40   239

EN 60   -841
EN 80   -1845
EN 100   -2916
EN 120   -1924
EN 140   -1980
EN 160   -4844
EN 180   -2721
EN 200   0
EN 220   -1333
EN 240   -2127
EN 260   -1827
EN 280   -2655
EN 300   861
EN 320   -2256
EN 340   -4949
EN 360   -4873
G-P:GLU-CA
EN 20   0
EN 40   251
EN 60   1892
EN 80   888
EN 100   78
EN 120   -4011
EN 140   -3424
EN 160   -2110
EN 180   -992
EN 200   0
EN 220   -2325
EN 240   0
EN 260   -2197
EN 280   -1187
EN 300   -1397
EN 320   -2244
EN 340   -3403
EN 360   0
G-P:CYS-CA
EN 20   0
EN 40   0
EN 60   0
EN 80   0
EN 100   -2149
EN 120   -2422
EN 140   -4647
EN 160   0
EN 180   0
EN 200   0
EN 220   -4553
EN 240   0
EN 260   0
EN 280   0
EN 300   0
EN 320   -2754
EN 340   0
EN 360   -5823
U-P:ASN-S2
EN 20   0
EN 40   -4565
EN 60   -2924

EN 80 -5314
EN 100 -6004
EN 120 -6016
EN 140 -6524
EN 160 -5210
EN 180 -4091
EN 200 -6230
EN 220 -5425
EN 240 -4662
EN 260 -5627
EN 280 -5120
EN 300 -4948
EN 320 -6348
EN 340 -1681
EN 360 -3973
C31-RIB:GLU-S1
EN 20 0
EN 40 0
EN 60 0
EN 80 -15937
EN 100 0
EN 120 0
EN 140 0
EN 160 0
EN 180 0
EN 200 0
EN 220 0
EN 240 0
EN 260 0
EN 280 0
EN 300 0
EN 320 0
EN 340 0
EN 360 0
U34-MY:ASN-S1
EN 20 0
EN 40 0
EN 60 0
EN 80 0
EN 100 0
EN 120 0
EN 140 0
EN 160 0
EN 180 0
EN 200 0
EN 220 0
EN 240 -18438
EN 260 -17686
EN 280 0
EN 300 0
EN 320 0
EN 340 0
EN 360 0
U31-P:LEU-S2
EN 20 0
EN 40 0
EN 60 0
EN 80 0

EN 100   0
EN 120   0
EN 140   0
EN 160   0
EN 180   0
EN 200   0
EN 220   0
EN 240   0
EN 260   0
EN 280   0
EN 300   -15751
EN 320   0
EN 340   0
EN 360   0
C31-MY:GLU-S2
EN 20   0
EN 40   -18325
EN 60   0
EN 80   0
EN 100   0
EN 120   0
EN 140   -16557
EN 160   0
EN 180   0
EN 200   0
EN 220   0
EN 240   0
EN 260   0
EN 280   0
EN 300   0
EN 320   0
EN 340   0
EN 360   0
A-RIB:PHE-S1
EN 20   0
EN 40   0
EN 60   -2252
EN 80   -3255
EN 100   -1344
EN 120   -3334
EN 140   -2125
EN 160   -2820
EN 180   -5136
EN 200   0
EN 220   0
EN 240   -554
EN 260   -2524
EN 280   -1343
EN 300   -3824
EN 320   -3666
EN 340   -3731
EN 360   0
A-R6:LYS-S1
EN 20   0
EN 40   -2368
EN 60   -3448
EN 80   -3448
EN 100   -2922

EN 120   -3818
EN 140   -2317
EN 160   -4017
EN 180   0
EN 200   0
EN 220   -3227
EN 240   -3017
EN 260   -4816
EN 280   -4456
EN 300   -3132
EN 320   -3146
EN 340   -1201
EN 360   0
G-P:VAL-CA
EN 20   0
EN 40   11
EN 60   -64
EN 80   -1067
EN 100   -1878
EN 120   -2533
EN 140   -3925
EN 160   -3354
EN 180   -3953
EN 200   0
EN 220   -1560
EN 240   0
EN 260   -1049
EN 280   -160
EN 300   -1636
EN 320   -2865
EN 340   -3261
EN 360   0
H2U-P:THR-CA
EN 20   0
EN 40   0
EN 60   0
EN 80   0
EN 100   0
EN 120   -14933
EN 140   0
EN 160   0
EN 180   0
EN 200   0
EN 220   0
EN 240   0
EN 260   0
EN 280   0
EN 300   0
EN 320   0
EN 340   0
EN 360   0
A-R6:ARG-S1
EN 20   0
EN 40   -5163
EN 60   -4788
EN 80   -4724
EN 100   -5044
EN 120   -3892

EN 140 -4108
EN 160 -4803
EN 180 -3685
EN 200 0
EN 220 -3301
EN 240 -4255
EN 260 -3795
EN 280 -2996
EN 300 -2372
EN 320 -4154
EN 340 -3545
EN 360 -1849
U-Y:PRO-S1
EN 20 -7286
EN 40 -4159
EN 60 -4235
EN 80 -4075
EN 100 -2615
EN 120 -166
EN 140 -4108
EN 160 -1369
EN 180 -3685
EN 200 0
EN 220 -2296
EN 240 -4809
EN 260 -4056
EN 280 -2614
EN 300 -4542
EN 320 0
EN 340 -2993
EN 360 -3567
A-RIB:ILE-CA
EN 20 0
EN 40 0
EN 60 -2299
EN 80 -3685
EN 100 -2657
EN 120 -1664
EN 140 -2172
EN 160 -1863
EN 180 -2462
EN 200 0
EN 220 -1073
EN 240 0
EN 260 -1567
EN 280 -3108
EN 300 -1601
EN 320 -2709
EN 340 -52
EN 360 -5065
FHU-RIB:THR-CA
EN 20 0
EN 40 0
EN 60 0
EN 80 -15407
EN 100 0
EN 120 0
EN 140 0

EN 160   0
EN 180   0
EN 200   0
EN 220   0
EN 240   0
EN 260   -15389
EN 280   -15213
EN 300   0
EN 320   0
EN 340   0
EN 360   0
C-RIB:ASN-S2
EN 20   -5046
EN 40   -4641
EN 60   -6435
EN 80   -5721
EN 100   -5725
EN 120   -5087
EN 140   -4590
EN 160   -5286
EN 180   -4880
EN 200   -5301
EN 220   -4496
EN 240   -6239
EN 260   -3534
EN 280   -5075
EN 300   -4019
EN 320   -3963
EN 340   -4740
EN 360   -5766
IU-RIB:GLN-S1
EN 20   0
EN 40   0
EN 60   0
EN 80   0
EN 100   0
EN 120   0
EN 140   0
EN 160   0
EN 180   0
EN 200   0
EN 220   0
EN 240   0
EN 260   0
EN 280   0
EN 300   -14744
EN 320   0
EN 340   0
EN 360   0
U-P:GLN-CA
EN 20   0
EN 40   -4787
EN 60   -4864
EN 80   -4150
EN 100   -3956
EN 120   -4229
EN 140   -3019
EN 160   -5432

EN 180 -6031
EN 200 0
EN 220 0
EN 240 -4171
EN 260 0
EN 280 -4960
EN 300 -4718
EN 320 -7067
EN 340 -3621
EN 360 -5912
C-Y:MET-CA
EN 20 0
EN 40 -4764
EN 60 -4841
EN 80 -2409
EN 100 -498
EN 120 -771
EN 140 0
EN 160 0
EN 180 0
EN 200 0
EN 220 -2902
EN 240 0
EN 260 -674
EN 280 -3220
EN 300 -707
EN 320 0
EN 340 0
EN 360 0
U-P:ARG-S1
EN 20 0
EN 40 -1496
EN 60 -2577
EN 80 -5496
EN 100 -5816
EN 120 -5929
EN 140 -7023
EN 160 -6580
EN 180 -7560
EN 200 -4878
EN 220 -4785
EN 240 -4314
EN 260 -5662
EN 280 -6490
EN 300 -6700
EN 320 -6262
EN 340 -7039
EN 360 -6056
QUO-M5:GLU-S2
EN 20 0
EN 40 0
EN 60 0
EN 80 0
EN 100 0
EN 120 0
EN 140 -16557
EN 160 0
EN 180 0

EN 200 0
EN 220 0
EN 240 0
EN 260 0
EN 280 0
EN 300 0
EN 320 0
EN 340 0
EN 360 0
G-P:ILE-CA
EN 20 0
EN 40 0
EN 60 -2479
EN 80 -1053
EN 100 -1571
EN 120 -3231
EN 140 -2905
EN 160 -3600
EN 180 0
EN 200 0
EN 220 0
EN 240 -782
EN 260 -4018
EN 280 1863
EN 300 -1068
EN 320 -2729
EN 340 -1236
EN 360 0
C-P:GLU-S2
EN 20 -3300
EN 40 -1891
EN 60 -3684
EN 80 -1806
EN 100 -4334
EN 120 -4607
EN 140 -3227
EN 160 -4545
EN 180 -3134
EN 200 0
EN 220 -1746
EN 240 -1275
EN 260 -2240
EN 280 -4163
EN 300 -3539
EN 320 -2669
EN 340 -1729
EN 360 -4020
G-R5:THR-S1
EN 20 -4042
EN 40 -3637
EN 60 -3262
EN 80 -278
EN 100 -3188
EN 120 -357
EN 140 852
EN 160 156
EN 180 0
EN 200 -4297

EN 220   -5592
EN 240   -4116
EN 260   -3364
EN 280   -1801
EN 300   -1298
EN 320   -2959
EN 340   250
EN 360   0
DA-M6:HIS-S1
EN 20   0
EN 40   0
EN 60   0
EN 80   0
EN 100   0
EN 120   0
EN 140   0
EN 160   0
EN 180   0
EN 200   0
EN 220   0
EN 240   0
EN 260   -18902
EN 280   0
EN 300   0
EN 320   0
EN 340   0
EN 360   0
FMU-P:PHE-S2
EN 20   0
EN 40   0
EN 60   0
EN 80   -17828
EN 100   0
EN 120   0
EN 140   0
EN 160   0
EN 180   0
EN 200   0
EN 220   0
EN 240   0
EN 260   0
EN 280   0
EN 300   0
EN 320   0
EN 340   0
EN 360   0
U31-P:HIS-S1
EN 20   0
EN 40   0
EN 60   0
EN 80   0
EN 100   0
EN 120   0
EN 140   0
EN 160   0
EN 180   0
EN 200   0
EN 220   0

EN 240   0
EN 260   0
EN 280   0
EN 300   0
EN 320   -19331
EN 340   0
EN 360   0
C-Y:MET-S2
EN 20   0
EN 40   -4775
EN 60   -4139
EN 80   -5143
EN 100   -2226
EN 120   -2499
EN 140   0
EN 160   0
EN 180   0
EN 200   -9162
EN 220   -7352
EN 240   -1437
EN 260   -2402
EN 280   -3943
EN 300   -2436
EN 320   0
EN 340   0
EN 360   0
G-P:ASP-S1
EN 20   0
EN 40   -442
EN 60   -2788
EN 80   -2526
EN 100   -3597
EN 120   -4704
EN 140   -4118
EN 160   -4190
EN 180   -3402
EN 200   0
EN 220   -3018
EN 240   -3260
EN 260   -791
EN 280   -3833
EN 300   -5397
EN 320   -4439
EN 340   -5216
EN 360   -5001
A-R5:VAL-S1
EN 20   -6371
EN 40   -3957
EN 60   -598
EN 80   -2989
EN 100   -3417
EN 120   35
EN 140   -472
EN 160   -1167
EN 180   0
EN 200   0
EN 220   -3812
EN 240   1098

EN 260 -871
EN 280 -1408
EN 300 -2622
EN 320 -1301
EN 340 643
EN 360 0
FHU-MY:ARG-S2
EN 20 0
EN 40 0
EN 60 0
EN 80 -14668
EN 100 -14474
EN 120 0
EN 140 0
EN 160 0
EN 180 0
EN 200 0
EN 220 0
EN 240 0
EN 260 -14650
EN 280 -14473
EN 300 0
EN 320 0
EN 340 0
EN 360 0
U34-P:ASN-CA
EN 20 0
EN 40 0
EN 60 0
EN 80 -19422
EN 100 -17510
EN 120 0
EN 140 0
EN 160 0
EN 180 0
EN 200 0
EN 220 0
EN 240 0
EN 260 -17686
EN 280 0
EN 300 0
EN 320 0
EN 340 0
EN 360 0
FMU-MY:CYS-S1
EN 20 0
EN 40 0
EN 60 0
EN 80 0
EN 100 -20686
EN 120 0
EN 140 0
EN 160 0
EN 180 0
EN 200 0
EN 220 0
EN 240 0
EN 260 0

EN 280   0
EN 300   0
EN 320   0
EN 340   0
EN 360   0
DA-M5:LYS-S2
EN 20   0
EN 40   0
EN 60   0
EN 80   0
EN 100   0
EN 120   0
EN 140   0
EN 160   0
EN 180   0
EN 200   0
EN 220   0
EN 240   0
EN 260   0
EN 280   -16153
EN 300   0
EN 320   0
EN 340   0
EN 360   0
U31-P:MET-CA
EN 20   0
EN 40   0
EN 60   0
EN 80   0
EN 100   0
EN 120   0
EN 140   0
EN 160   0
EN 180   0
EN 200   0
EN 220   0
EN 240   0
EN 260   -18835
EN 280   0
EN 300   -18868
EN 320   0
EN 340   0
EN 360   0
QUO-RIB:LEU-S1
EN 20   0
EN 40   0
EN 60   0
EN 80   0
EN 100   0
EN 120   0
EN 140   0
EN 160   0
EN 180   0
EN 200   0
EN 220   0
EN 240   0
EN 260   -17432
EN 280   0

EN 300   0
EN 320   0
EN 340   0
EN 360   0
IU-MY:LYS-S2
EN 20   0
EN 40   0
EN 60   0
EN 80   0
EN 100   -11929
EN 120   0
EN 140   -13715
EN 160   0
EN 180   -16726
EN 200   0
EN 220   0
EN 240   0
EN 260   -13110
EN 280   0
EN 300   -12139
EN 320   0
EN 340   -13312
EN 360   0
G-R5:TRP-S1
EN 20   -7303
EN 40   -4176
EN 60   0
EN 80   -3538
EN 100   -6066
EN 120   -3617
EN 140   0
EN 160   0
EN 180   0
EN 200   0
EN 220   -5748
EN 240   -4272
EN 260   0
EN 280   -3344
EN 300   -4558
EN 320   0
EN 340   -3009
EN 360   0
A-R6:TRP-S1
EN 20   0
EN 40   0
EN 60   0
EN 80   -4098
EN 100   -2187
EN 120   -2460
EN 140   0
EN 160   0
EN 180   0
EN 200   0
EN 220   0
EN 240   -3115
EN 260   -5085
EN 280   -3904
EN 300   0

EN 320   -2792
EN 340   -3569
EN 360   0
FHU-P:TYR-S2
EN 20   0
EN 40   -18829
EN 60   0
EN 80   -16474
EN 100   0
EN 120   0
EN 140   0
EN 160   0
EN 180   0
EN 200   0
EN 220   -18684
EN 240   0
EN 260   -16456
EN 280   0
EN 300   0
EN 320   0
EN 340   0
EN 360   0
A-P:ASP-CA
EN 20   -4101
EN 40   0
EN 60   -2056
EN 80   -3772
EN 100   -1860
EN 120   -4856
EN 140   -924
EN 160   -3890
EN 180   -3936
EN 200   0
EN 220   -2547
EN 240   -2789
EN 260   -2590
EN 280   -3578
EN 300   -3787
EN 320   -1753
EN 340   -4630
EN 360   -3817
G-RIB:PRO-CA
EN 20   0
EN 40   -1115
EN 60   -4626
EN 80   -5469
EN 100   -4722
EN 120   -3660
EN 140   -4168
EN 160   -5747
EN 180   -5080
EN 200   -4496
EN 220   -3691
EN 240   -2929
EN 260   -3181
EN 280   -3717
EN 300   -4480
EN 320   -3610

EN 340   -3383
EN 360   0
C-Y:HIS-CA
EN 20   0
EN 40   -5836
EN 60   -1473
EN 80   -3481
EN 100   -2282
EN 120   -2555
EN 140   -3063
EN 160   -2041
EN 180   -4357
EN 200   0
EN 220   -6956
EN 240   -5481
EN 260   -4729
EN 280   -2282
EN 300   -2492
EN 320   -2887
EN 340   0
EN 360   0
QUO-M5:ASP-S2
EN 20   0
EN 40   0
EN 60   0
EN 80   0
EN 100   0
EN 120   -16748
EN 140   0
EN 160   0
EN 180   0
EN 200   0
EN 220   0
EN 240   0
EN 260   0
EN 280   0
EN 300   0
EN 320   -17080
EN 340   0
EN 360   0
U31-RIB:ASN-CA
EN 20   0
EN 40   0
EN 60   0
EN 80   0
EN 100   0
EN 120   0
EN 140   0
EN 160   0
EN 180   0
EN 200   0
EN 220   0
EN 240   -18438
EN 260   0
EN 280   0
EN 300   0
EN 320   0
EN 340   0

EN 360   0
A-P:PRO-S1
EN 20   0
EN 40   -1677
EN 60   -4475
EN 80   -3310
EN 100   -5069
EN 120   -3389
EN 140   -2631
EN 160   -5757
EN 180   0
EN 200   0
EN 220   -6353
EN 240   -2778
EN 260   -3744
EN 280   -4572
EN 300   -4159
EN 320   -5177
EN 340   -5663
EN 360   -2802
IU-RIB:ILE-CA
EN 20   0
EN 40   0
EN 60   0
EN 80   0
EN 100   0
EN 120   0
EN 140   0
EN 160   0
EN 180   0
EN 200   0
EN 220   0
EN 240   0
EN 260   0
EN 280   -13454
EN 300   0
EN 320   0
EN 340   0
EN 360   0
G-P:GLN-S1
EN 20   0
EN 40   -6205
EN 60   -3559
EN 80   -3850
EN 100   -3655
EN 120   -4641
EN 140   -4819
EN 160   -4127
EN 180   -7830
EN 200   0
EN 220   -1620
EN 240   -4132
EN 260   -5097
EN 280   -4037
EN 300   -4870
EN 320   -4643
EN 340   -6954
EN 360   -5612

FHU-RIB:LEU-S1
EN 20 0
EN 40 0
EN 60 0
EN 80 0
EN 100 -13821
EN 120 0
EN 140 0
EN 160 0
EN 180 0
EN 200 0
EN 220 0
EN 240 0
EN 260 0
EN 280 -13821
EN 300 0
EN 320 0
EN 340 0
EN 360 0
U-P:LEU-CA
EN 20 0
EN 40 -855
EN 60 785
EN 80 -1222
EN 100 -1741
EN 120 -3019
EN 140 -1809
EN 160 0
EN 180 -3816
EN 200 0
EN 220 -2427
EN 240 765
EN 260 1517
EN 280 -3750
EN 300 -3668
EN 320 -2899
EN 340 310
EN 360 0
IU-MY:GLU-S2
EN 20 0
EN 40 0
EN 60 0
EN 80 0
EN 100 0
EN 120 0
EN 140 -12333
EN 160 0
EN 180 0
EN 200 0
EN 220 0
EN 240 0
EN 260 0
EN 280 0
EN 300 0
EN 320 0
EN 340 0
EN 360 0
H2U-MY:ARG-CA

EN 20   0
EN 40   0
EN 60   0
EN 80   0
EN 100   0
EN 120   0
EN 140   0
EN 160   0
EN 180   0
EN 200   0
EN 220   0
EN 240   0
EN 260   0
EN 280   0
EN 300   0
EN 320   -12797
EN 340   0
EN 360   0
U-P:LEU-S1
EN 20   0
EN 40   -2573
EN 60   -2649
EN 80   1499
EN 100   -2294
EN 120   -3019
EN 140   -805
EN 160   -2504
EN 180   0
EN 200   0
EN 220   -3432
EN 240   -952
EN 260   -1204
EN 280   -3750
EN 300   -1950
EN 320   -2899
EN 340   -2411
EN 360   0
FHU-RIB:SER-CA
EN 20   0
EN 40   0
EN 60   0
EN 80   0
EN 100   0
EN 120   0
EN 140   -15754
EN 160   0
EN 180   0
EN 200   0
EN 220   0
EN 240   0
EN 260   0
EN 280   0
EN 300   0
EN 320   -15579
EN 340   0
EN 360   0
QUO-M6:GLN-S1
EN 20   0

EN 40   0
EN 60   0
EN 80   0
EN 100   0
EN 120   -18026
EN 140   0
EN 160   0
EN 180   0
EN 200   0
EN 220   0
EN 240   0
EN 260   0
EN 280   0
EN 300   0
EN 320   0
EN 340   0
EN 360   0
DA-M6:LYS-S1
EN 20   0
EN 40   0
EN 60   0
EN 80   0
EN 100   0
EN 120   0
EN 140   0
EN 160   0
EN 180   0
EN 200   0
EN 220   0
EN 240   0
EN 260   0
EN 280   0
EN 300   -16315
EN 320   0
EN 340   0
EN 360   0
FMU-MY:ARG-S1
EN 20   0
EN 40   0
EN 60   -17087
EN 80   0
EN 100   0
EN 120   0
EN 140   0
EN 160   0
EN 180   0
EN 200   0
EN 220   0
EN 240   0
EN 260   0
EN 280   0
EN 300   0
EN 320   0
EN 340   0
EN 360   0
C-RIB:GLY-CA
EN 20   -6311
EN 40   -5906

EN 60   -6244
EN 80   -4646
EN 100   -5457
EN 120   -5482
EN 140   -5233
EN 160   -5928
EN 180   -6145
EN 200   -3844
EN 220   -5761
EN 240   -5380
EN 260   -4959
EN 280   -5074
EN 300   -5545
EN 320   -5388
EN 340   -5237
EN 360   -5022
QUO-P:PHE-S2
EN 20   0
EN 40   -20183
EN 60   0
EN 80   0
EN 100   0
EN 120   0
EN 140   0
EN 160   0
EN 180   0
EN 200   0
EN 220   0
EN 240   0
EN 260   0
EN 280   0
EN 300   0
EN 320   0
EN 340   0
EN 360   0
FHU-RIB:LEU-S2
EN 20   0
EN 40   0
EN 60   0
EN 80   0
EN 100   -13824
EN 120   -14097
EN 140   0
EN 160   0
EN 180   0
EN 200   0
EN 220   0
EN 240   0
EN 260   0
EN 280   -15541
EN 300   -14034
EN 320   0
EN 340   0
EN 360   0
FHU-P:VAL-CA
EN 20   0
EN 40   0
EN 60   0

EN 80 0
EN 100 0
EN 120 -14531
EN 140 0
EN 160 0
EN 180 0
EN 200 0
EN 220 0
EN 240 0
EN 260 0
EN 280 0
EN 300 0
EN 320 -14863
EN 340 0
EN 360 0
G-R5:ALA-CA
EN 20 0
EN 40 -2393
EN 60 252
EN 80 -751
EN 100 -1853
EN 120 0
EN 140 -1338
EN 160 -316
EN 180 0
EN 200 0
EN 220 -4347
EN 240 -3494
EN 260 -1738
EN 280 -1561
EN 300 -1771
EN 320 -1162
EN 340 1494
EN 360 -796
DA-M6:TYR-S1
EN 20 0
EN 40 0
EN 60 0
EN 80 0
EN 100 -17993
EN 120 0
EN 140 0
EN 160 0
EN 180 0
EN 200 0
EN 220 0
EN 240 0
EN 260 0
EN 280 0
EN 300 -18203
EN 320 0
EN 340 0
EN 360 0
A-RIB:PHE-S2
EN 20 0
EN 40 -2179
EN 60 -538
EN 80 -4646

```
EN 100   -3618
EN 120   -2625
EN 140   -3846
EN 160   -2824
EN 180   -5140
EN 200   0
EN 220   0
EN 240   0
EN 260   -3241
EN 280   -2352
EN 300   -4279
EN 320   0
EN 340   -2730
EN 360   -6026
G-R5:MET-CA
EN 20   -5770
EN 40   0
EN 60   -2720
EN 80   -4728
EN 100   -95
EN 120   0
EN 140   0
EN 160   0
EN 180   0
EN 200   0
EN 220   0
EN 240   -2740
EN 260   -1988
EN 280   -4082
EN 300   0
EN 320   -3422
EN 340   -1477
EN 360   -3769
U-P:CYS-CA
EN 20   0
EN 40   0
EN 60   0
EN 80   -3648
EN 100   -3454
EN 120   -5444
EN 140   0
EN 160   -4930
EN 180   0
EN 200   0
EN 220   0
EN 240   0
EN 260   0
EN 280   0
EN 300   0
EN 320   0
EN 340   0
EN 360   0
G-R6:HIS-S1
EN 20   -5837
EN 40   -5433
EN 60   -3791
EN 80   -4795
EN 100   -4601
```

EN 120 -5256
EN 140 -943
EN 160 0
EN 180 0
EN 200 0
EN 220 -4283
EN 240 -4950
EN 260 -5490
EN 280 -4149
EN 300 -4359
EN 320 -3489
EN 340 0
EN 360 -3836
C31-P:TYR-CA
EN 20 0
EN 40 0
EN 60 0
EN 80 0
EN 100 -17993
EN 120 0
EN 140 0
EN 160 0
EN 180 0
EN 200 0
EN 220 0
EN 240 0
EN 260 0
EN 280 -17993
EN 300 0
EN 320 0
EN 340 0
EN 360 0
C-P:PHE-S1
EN 20 0
EN 40 -2028
EN 60 0
EN 80 -3108
EN 100 0
EN 120 -1470
EN 140 -2982
EN 160 -3678
EN 180 0
EN 200 0
EN 220 0
EN 240 -2125
EN 260 -3090
EN 280 -1196
EN 300 -3124
EN 320 0
EN 340 -3584
EN 360 0
A-R5:CYS-S1
EN 20 0
EN 40 -5231
EN 60 0
EN 80 -5598
EN 100 0
EN 120 0

EN 140   0
EN 160   0
EN 180   0
EN 200   0
EN 220   0
EN 240   -3610
EN 260   0
EN 280   -4399
EN 300   -2891
EN 320   0
EN 340   0
EN 360   0
C-RIB:GLU-S2
EN 20   0
EN 40   0
EN 60   -2962
EN 80   -2961
EN 100   -2054
EN 120   -3332
EN 140   -1830
EN 160   -5630
EN 180   -4129
EN 200   0
EN 220   0
EN 240   -3364
EN 260   -3948
EN 280   -2436
EN 300   -546
EN 320   -2208
EN 340   -2985
EN 360   -4723
G-RIB:PHE-S2
EN 20   0
EN 40   0
EN 60   0
EN 80   735
EN 100   929
EN 120   -4164
EN 140   -3286
EN 160   -546
EN 180   -4580
EN 200   0
EN 220   0
EN 240   -2720
EN 260   -3234
EN 280   -4222
EN 300   0
EN 320   -2397
EN 340   -4440
EN 360   -2744
C31-P:TYR-S1
EN 20   0
EN 40   0
EN 60   0
EN 80   0
EN 100   -17993
EN 120   0
EN 140   0

EN 160   0
EN 180   0
EN 200   0
EN 220   0
EN 240   0
EN 260   0
EN 280   -17993
EN 300   0
EN 320   0
EN 340   0
EN 360   0
G-R6:MET-CA
EN 20   0
EN 40   -2644
EN 60   -2720
EN 80   -4277
EN 100   -95
EN 120   -2085
EN 140   0
EN 160   0
EN 180   0
EN 200   0
EN 220   0
EN 240   -3745
EN 260   -1988
EN 280   -1812
EN 300   -3026
EN 320   -2417
EN 340   -1477
EN 360   -3769
U31-RIB:ASP-S1
EN 20   0
EN 40   -18979
EN 60   0
EN 80   -16624
EN 100   0
EN 120   0
EN 140   0
EN 160   -17906
EN 180   0
EN 200   0
EN 220   0
EN 240   0
EN 260   0
EN 280   0
EN 300   0
EN 320   -17035
EN 340   0
EN 360   0
QUO-RIB:ASN-S2
EN 20   0
EN 40   0
EN 60   0
EN 80   0
EN 100   0
EN 120   0
EN 140   0
EN 160   -19007

EN 180   0
EN 200   0
EN 220   0
EN 240   0
EN 260   0
EN 280   0
EN 300   0
EN 320   0
EN 340   0
EN 360   0
U31-MY:ASP-CA
EN 20   0
EN 40   0
EN 60   0
EN 80   -16622
EN 100   -16427
EN 120   -16700
EN 140   0
EN 160   0
EN 180   0
EN 200   0
EN 220   0
EN 240   0
EN 260   0
EN 280   0
EN 300   0
EN 320   0
EN 340   0
EN 360   0
H2U-MY:ALA-CA
EN 20   0
EN 40   0
EN 60   0
EN 80   0
EN 100   0
EN 120   0
EN 140   -12480
EN 160   0
EN 180   0
EN 200   0
EN 220   0
EN 240   0
EN 260   0
EN 280   0
EN 300   0
EN 320   0
EN 340   0
EN 360   0
C-RIB:PHE-CA
EN 20   0
EN 40   0
EN 60   -2095
EN 80   -3099
EN 100   -2904
EN 120   -3177
EN 140   -1968
EN 160   -3668
EN 180   0

EN 200 0
EN 220 0
EN 240 0
EN 260 -2368
EN 280 -2191
EN 300 320
EN 320 -2797
EN 340 0
EN 360 -3144
G-RIB:MET-CA
EN 20 -5770
EN 40 0
EN 60 -3725
EN 80 -289
EN 100 -1812
EN 120 -3090
EN 140 -2593
EN 160 -5559
EN 180 -5604
EN 200 0
EN 220 0
EN 240 -1023
EN 260 -3706
EN 280 -2816
EN 300 -2022
EN 320 -3422
EN 340 -4912
EN 360 -3769
G-RIB:ARG-CA
EN 20 0
EN 40 -164
EN 60 -4228
EN 80 -4519
EN 100 -5273
EN 120 -4045
EN 140 -4935
EN 160 -5248
EN 180 -4129
EN 200 -3546
EN 220 -1736
EN 240 -5082
EN 260 -4952
EN 280 -4634
EN 300 -3268
EN 320 -4759
EN 340 -4939
EN 360 -3006
U-Y:CYS-CA
EN 20 0
EN 40 0
EN 60 -4357
EN 80 0
EN 100 0
EN 120 0
EN 140 0
EN 160 0
EN 180 0
EN 200 0

EN 220   0
EN 240   0
EN 260   -2620
EN 280   0
EN 300   -5376
EN 320   -4054
EN 340   -4832
EN 360   0
FMU-MY:PHE-CA
EN 20   0
EN 40   0
EN 60   0
EN 80   0
EN 100   0
EN 120   0
EN 140   0
EN 160   0
EN 180   0
EN 200   0
EN 220   0
EN 240   0
EN 260   -17807
EN 280   0
EN 300   0
EN 320   0
EN 340   0
EN 360   0
A-R6:LEU-S1
EN 20   0
EN 40   -2805
EN 60   -159
EN 80   -2881
EN 100   -2686
EN 120   -1795
EN 140   1684
EN 160   -728
EN 180   0
EN 200   0
EN 220   -3373
EN 240   1537
EN 260   -2150
EN 280   -2686
EN 300   -3188
EN 320   -1574
EN 340   1082
EN 360   -1208
C-Y:PRO-S1
EN 20   -6362
EN 40   -2784
EN 60   -5029
EN 80   -3602
EN 100   -3790
EN 120   -2677
EN 140   -1467
EN 160   -2162
EN 180   0
EN 200   -8335
EN 220   -4095

EN 240   -3885
EN 260   -3584
EN 280   -3790
EN 300   -896
EN 320   -1291
EN 340   -351
EN 360   0
FMU-RIB:PHE-S1
EN 20   0
EN 40   0
EN 60   0
EN 80   0
EN 100   0
EN 120   0
EN 140   0
EN 160   0
EN 180   0
EN 200   0
EN 220   0
EN 240   -18559
EN 260   0
EN 280   0
EN 300   0
EN 320   0
EN 340   -19013
EN 360   0
U31-MY:THR-S1
EN 20   0
EN 40   0
EN 60   0
EN 80   -17125
EN 100   0
EN 120   0
EN 140   0
EN 160   -18407
EN 180   0
EN 200   0
EN 220   0
EN 240   0
EN 260   0
EN 280   0
EN 300   0
EN 320   -17536
EN 340   0
EN 360   0
A-R6:LEU-S2
EN 20   0
EN 40   -86
EN 60   1554
EN 80   -2171
EN 100   -2689
EN 120   -1245
EN 140   -2306
EN 160   985
EN 180   -3047
EN 200   0
EN 220   58
EN 240   -1188

EN 260   -2153
EN 280   -2981
EN 300   -3904
EN 320   -1577
EN 340   -2355
EN 360   0
FMU-RIB:ARG-CA
EN 20   0
EN 40   0
EN 60   0
EN 80   0
EN 100   0
EN 120   -16452
EN 140   0
EN 160   0
EN 180   0
EN 200   0
EN 220   0
EN 240   0
EN 260   0
EN 280   0
EN 300   0
EN 320   0
EN 340   0
EN 360   0
A-R5:MET-S2
EN 20   0
EN 40   -4932
EN 60   -4296
EN 80   -860
EN 100   -4100
EN 120   -4926
EN 140   -4169
EN 160   -4864
EN 180   0
EN 200   0
EN 220   -3070
EN 240   -1594
EN 260   -3564
EN 280   0
EN 300   -3597
EN 320   -3993
EN 340   0
EN 360   -6057
C-RIB:LEU-CA
EN 20   0
EN 40   -1644
EN 60   -2273
EN 80   -2724
EN 100   -2530
EN 120   -2472
EN 140   -2598
EN 160   -2289
EN 180   -4605
EN 200   0
EN 220   0
EN 240   -1740
EN 260   -2375

EN 280   -4247
EN 300   -3744
EN 320   -3427
EN 340   -3912
EN 360   0
G-R6:HIS-S2
EN 20   -5854
EN 40   -6003
EN 60   -5908
EN 80   -6078
EN 100   -4166
EN 120   -6393
EN 140   -4394
EN 160   -1655
EN 180   0
EN 200   -6109
EN 220   -6570
EN 240   -5094
EN 260   -4689
EN 280   -3613
EN 300   -6545
EN 320   -4219
EN 340   0
EN 360   -3853
QUO-P:LEU-CA
EN 20   0
EN 40   0
EN 60   0
EN 80   0
EN 100   -17256
EN 120   0
EN 140   0
EN 160   0
EN 180   0
EN 200   0
EN 220   0
EN 240   0
EN 260   0
EN 280   0
EN 300   0
EN 320   0
EN 340   0
EN 360   0
U-P:LYS-CA
EN 20   0
EN 40   0
EN 60   -4214
EN 80   -5417
EN 100   -5024
EN 120   -4584
EN 140   -6187
EN 160   -6699
EN 180   -6094
EN 200   0
EN 220   -1271
EN 240   -4948
EN 260   -4196
EN 280   -4019

EN 300   -4782
EN 320   -6342
EN 340   -5402
EN 360   -5263
A-P:PHE-CA
EN 20   0
EN 40   0
EN 60   -2254
EN 80   -1541
EN 100   -1346
EN 120   97
EN 140   -410
EN 160   -4540
EN 180   -3421
EN 200   0
EN 220   -2033
EN 240   -2275
EN 260   -1522
EN 280   -2351
EN 300   -4991
EN 320   -3669
EN 340   -3734
EN 360   0
IU-MY:THR-S1
EN 20   0
EN 40   0
EN 60   0
EN 80   0
EN 100   0
EN 120   -13984
EN 140   0
EN 160   0
EN 180   0
EN 200   0
EN 220   0
EN 240   0
EN 260   0
EN 280   0
EN 300   0
EN 320   0
EN 340   -14089
EN 360   0
G-R6:TYR-S1
EN 20   0
EN 40   0
EN 60   -2054
EN 80   376
EN 100   -2704
EN 120   -1419
EN 140   0
EN 160   -2292
EN 180   0
EN 200   0
EN 220   -3550
EN 240   0
EN 260   -1323
EN 280   -2704
EN 300   -1356

EN 320 -2756
EN 340 -812
EN 360 -4820
OMC-RIB:LYS-S1
EN 20 0
EN 40 0
EN 60 0
EN 80 0
EN 100 0
EN 120 0
EN 140 0
EN 160 0
EN 180 0
EN 200 0
EN 220 0
EN 240 -13217
EN 260 0
EN 280 0
EN 300 0
EN 320 0
EN 340 0
EN 360 0
A-RIB:ASN-CA
EN 20 0
EN 40 -2055
EN 60 -3849
EN 80 -3135
EN 100 -4950
EN 120 -3767
EN 140 -5731
EN 160 -2700
EN 180 -5016
EN 200 0
EN 220 -3627
EN 240 -5878
EN 260 -4122
EN 280 -3945
EN 300 -3703
EN 320 -5555
EN 340 -3611
EN 360 -7619
U-P:TYR-CA
EN 20 0
EN 40 -3310
EN 60 -4391
EN 80 -3677
EN 100 -761
EN 120 0
EN 140 -3259
EN 160 -2237
EN 180 0
EN 200 0
EN 220 0
EN 240 0
EN 260 -2654
EN 280 -3483
EN 300 -3692
EN 320 -1366

EN 340 0
EN 360 0
C31-MY:THR-CA
EN 20 0
EN 40 0
EN 60 0
EN 80 0
EN 100 0
EN 120 0
EN 140 0
EN 160 0
EN 180 0
EN 200 0
EN 220 0
EN 240 0
EN 260 -17107
EN 280 0
EN 300 0
EN 320 0
EN 340 0
EN 360 0
QUO-M6:ARG-S2
EN 20 0
EN 40 0
EN 60 0
EN 80 0
EN 100 0
EN 120 -16464
EN 140 0
EN 160 0
EN 180 0
EN 200 0
EN 220 0
EN 240 0
EN 260 0
EN 280 0
EN 300 0
EN 320 0
EN 340 0
EN 360 -19865
U31-RIB:PHE-CA
EN 20 0
EN 40 0
EN 60 0
EN 80 0
EN 100 0
EN 120 -17904
EN 140 0
EN 160 0
EN 180 0
EN 200 0
EN 220 0
EN 240 0
EN 260 0
EN 280 0
EN 300 0
EN 320 0
EN 340 0

EN 360 0
C31-RIB:ALA-S1
EN 20 0
EN 40 0
EN 60 0
EN 80 0
EN 100 0
EN 120 0
EN 140 -16467
EN 160 0
EN 180 0
EN 200 0
EN 220 0
EN 240 0
EN 260 0
EN 280 0
EN 300 -15896
EN 320 0
EN 340 0
EN 360 0
U31-P:ASN-S2
EN 20 0
EN 40 0
EN 60 0
EN 80 0
EN 100 -17531
EN 120 0
EN 140 0
EN 160 0
EN 180 0
EN 200 0
EN 220 0
EN 240 0
EN 260 0
EN 280 0
EN 300 0
EN 320 0
EN 340 0
EN 360 0
C-RIB:HIS-CA
EN 20 0
EN 40 0
EN 60 -1473
EN 80 -5580
EN 100 -4552
EN 120 -2555
EN 140 0
EN 160 -6862
EN 180 -6075
EN 200 0
EN 220 0
EN 240 -4215
EN 260 -741
EN 280 -4552
EN 300 -6479
EN 320 -5158
EN 340 -4669
EN 360 -6961

```
FHU-MY:LEU-CA
EN 20   0
EN 40   0
EN 60   0
EN 80   -14015
EN 100   0
EN 120   0
EN 140   0
EN 160   -15297
EN 180   0
EN 200   0
EN 220   0
EN 240   0
EN 260   -13997
EN 280   0
EN 300   0
EN 320   0
EN 340   -15204
EN 360   0
M2G-P:GLU-CA
EN 20   0
EN 40   0
EN 60   0
EN 80   0
EN 100   0
EN 120   0
EN 140   0
EN 160   0
EN 180   0
EN 200   0
EN 220   0
EN 240   0
EN 260   0
EN 280   0
EN 300   0
EN 320   0
EN 340   0
EN 360   -19410
U-RIB:TYR-CA
EN 20   0
EN 40   -3305
EN 60   0
EN 80   -3672
EN 100   -756
EN 120   -2747
EN 140   0
EN 160   -4954
EN 180   0
EN 200   0
EN 220   0
EN 240   0
EN 260   0
EN 280   -5577
EN 300   -4401
EN 320   -4083
EN 340   -6126
EN 360   -6148
C-RIB:MET-S1
```

EN 20 0
EN 40 0
EN 60 0
EN 80 -5132
EN 100 -4485
EN 120 -2488
EN 140 0
EN 160 -4696
EN 180 -7012
EN 200 0
EN 220 0
EN 240 -4148
EN 260 -2391
EN 280 -3220
EN 300 -6152
EN 320 -2821
EN 340 0
EN 360 -4172
G-R6:TRP-CA
EN 20 0
EN 40 0
EN 60 -2535
EN 80 -6261
EN 100 0
EN 120 -3617
EN 140 -4125
EN 160 0
EN 180 0
EN 200 0
EN 220 -4031
EN 240 0
EN 260 -1803
EN 280 -1627
EN 300 -3554
EN 320 -2232
EN 340 0
EN 360 0
FMU-MY:ASN-CA
EN 20 0
EN 40 0
EN 60 0
EN 80 0
EN 100 -17510
EN 120 0
EN 140 0
EN 160 0
EN 180 0
EN 200 0
EN 220 0
EN 240 0
EN 260 0
EN 280 0
EN 300 0
EN 320 0
EN 340 0
EN 360 0
U31-RIB:GLU-S2
EN 20 0

EN 40   0
EN 60   0
EN 80   0
EN 100   0
EN 120   0
EN 140   0
EN 160   0
EN 180   0
EN 200   0
EN 220   0
EN 240   0
EN 260   0
EN 280   0
EN 300   0
EN 320   0
EN 340   0
EN 360   -19450
FMU-P:ASP-S1
EN 20   0
EN 40   0
EN 60   0
EN 80   0
EN 100   0
EN 120   0
EN 140   -17211
EN 160   0
EN 180   0
EN 200   0
EN 220   0
EN 240   0
EN 260   0
EN 280   0
EN 300   0
EN 320   0
EN 340   0
EN 360   0
U-RIB:ALA-S1
EN 20   -5842
EN 40   -4433
EN 60   -4509
EN 80   -4585
EN 100   -2888
EN 120   -2710
EN 140   -3670
EN 160   -5078
EN 180   0
EN 200   -4380
EN 220   -3575
EN 240   -4199
EN 260   -3447
EN 280   -4154
EN 300   -2094
EN 320   -2489
EN 340   -2554
EN 360   -3841
U-RIB:CYS-CA
EN 20   0
EN 40   0

EN 60   0
EN 80   0
EN 100   0
EN 120   0
EN 140   -5947
EN 160   -4925
EN 180   -7241
EN 200   0
EN 220   -5853
EN 240   0
EN 260   -5343
EN 280   0
EN 300   0
EN 320   0
EN 340   0
EN 360   0
G-R6:ASN-S1
EN 20   0
EN 40   -1495
EN 60   -4078
EN 80   -4845
EN 100   -4098
EN 120   -4293
EN 140   -3715
EN 160   -1809
EN 180   -2738
EN 200   0
EN 220   -4785
EN 240   -2381
EN 260   -3944
EN 280   -3385
EN 300   -4798
EN 320   -1269
EN 340   -329
EN 360   0
U-Y:ASP-S2
EN 20   -4913
EN 40   -3504
EN 60   -3580
EN 80   -3419
EN 100   762
EN 120   -2233
EN 140   -4006
EN 160   -3436
EN 180   0
EN 200   -5169
EN 220   -1641
EN 240   -4605
EN 260   -2136
EN 280   762
EN 300   -1165
EN 320   156
EN 340   -3342
EN 360   0
C-RIB:PRO-CA
EN 20   0
EN 40   -3235
EN 60   -5321

EN 80 -4868
EN 100 -4413
EN 120 -4063
EN 140 -3185
EN 160 -2162
EN 180 -4479
EN 200 0
EN 220 -3090
EN 240 -3885
EN 260 -3584
EN 280 -4910
EN 300 -3618
EN 320 -5515
EN 340 -4339
EN 360 -2643
H2U-P:PRO-S1
EN 20 0
EN 40 0
EN 60 0
EN 80 0
EN 100 -13142
EN 120 0
EN 140 0
EN 160 0
EN 180 0
EN 200 0
EN 220 0
EN 240 0
EN 260 0
EN 280 0
EN 300 0
EN 320 0
EN 340 0
EN 360 0
U34-RIB:ASN-CA
EN 20 0
EN 40 0
EN 60 0
EN 80 -17704
EN 100 0
EN 120 0
EN 140 0
EN 160 0
EN 180 0
EN 200 0
EN 220 0
EN 240 -18438
EN 260 -17686
EN 280 -17510
EN 300 0
EN 320 0
EN 340 0
EN 360 0
A-P:CYS-CA
EN 20 0
EN 40 -5234
EN 60 0
EN 80 -2879

EN 100   -4402
EN 120   -2957
EN 140   -3465
EN 160   0
EN 180   0
EN 200   0
EN 220   0
EN 240   -3613
EN 260   -4578
EN 280   -2684
EN 300   0
EN 320   0
EN 340   0
EN 360   -6358
H2U-RIB:LYS-S1
EN 20   0
EN 40   0
EN 60   0
EN 80   0
EN 100   0
EN 120   0
EN 140   0
EN 160   0
EN 180   0
EN 200   0
EN 220   0
EN 240   0
EN 260   0
EN 280   -12117
EN 300   0
EN 320   0
EN 340   0
EN 360   0
5BU-RIB:PRO-CA
EN 20   0
EN 40   -17961
EN 60   0
EN 80   0
EN 100   0
EN 120   0
EN 140   0
EN 160   0
EN 180   0
EN 200   0
EN 220   0
EN 240   0
EN 260   0
EN 280   0
EN 300   0
EN 320   0
EN 340   0
EN 360   0
G-R6:SER-S1
EN 20   -3802
EN 40   -4663
EN 60   -5191
EN 80   -3142
EN 100   -2114

EN 120   -2839
EN 140   -625
EN 160   396
EN 180   -1919
EN 200   -4058
EN 220   -3965
EN 240   -1561
EN 260   -4460
EN 280   -4068
EN 300   -4041
EN 320   -3553
EN 340   490
EN 360   0
FHU-RIB:ARG-S2
EN 20   0
EN 40   0
EN 60   -15382
EN 80   0
EN 100   0
EN 120   0
EN 140   0
EN 160   0
EN 180   -18266
EN 200   0
EN 220   0
EN 240   -15402
EN 260   -14650
EN 280   0
EN 300   0
EN 320   0
EN 340   0
EN 360   -18148
IU-MY:ILE-CA
EN 20   0
EN 40   0
EN 60   -14362
EN 80   0
EN 100   0
EN 120   0
EN 140   0
EN 160   0
EN 180   0
EN 200   0
EN 220   0
EN 240   -13378
EN 260   0
EN 280   0
EN 300   0
EN 320   0
EN 340   0
EN 360   0
U-RIB:GLN-S2
EN 20   0
EN 40   -6531
EN 60   -4177
EN 80   -4729
EN 100   -5699
EN 120   -5259

EN 140 -4763
EN 160 -5458
EN 180 -6057
EN 200 0
EN 220 -4668
EN 240 -6919
EN 260 -4158
EN 280 -5368
EN 300 -4744
EN 320 -3874
EN 340 -6369
EN 360 0
U-Y:GLN-S1
EN 20 0
EN 40 -3065
EN 60 -6245
EN 80 -2428
EN 100 -516
EN 120 -5229
EN 140 -3015
EN 160 -5809
EN 180 -4309
EN 200 0
EN 220 -4637
EN 240 -4879
EN 260 -5514
EN 280 -1782
EN 300 -4161
EN 320 -4556
EN 340 0
EN 360 -5908
U31-MY:MET-CA
EN 20 0
EN 40 0
EN 60 0
EN 80 -18853
EN 100 0
EN 120 0
EN 140 0
EN 160 0
EN 180 0
EN 200 0
EN 220 0
EN 240 0
EN 260 0
EN 280 0
EN 300 0
EN 320 0
EN 340 0
EN 360 0
FHU-P:THR-S1
EN 20 0
EN 40 0
EN 60 0
EN 80 -15407
EN 100 0
EN 120 0
EN 140 0

EN 160 0
EN 180 0
EN 200 0
EN 220 0
EN 240 -16141
EN 260 0
EN 280 0
EN 300 0
EN 320 -15818
EN 340 0
EN 360 0
U34-RIB:SER-CA
EN 20 0
EN 40 0
EN 60 0
EN 80 0
EN 100 0
EN 120 0
EN 140 0
EN 160 0
EN 180 0
EN 200 0
EN 220 0
EN 240 0
EN 260 0
EN 280 0
EN 300 -16900
EN 320 0
EN 340 0
EN 360 0
G-R6:THR-CA
EN 20 0
EN 40 -4350
EN 60 -992
EN 80 -1282
EN 100 -2354
EN 120 -1361
EN 140 -865
EN 160 156
EN 180 0
EN 200 0
EN 220 -3492
EN 240 -1012
EN 260 -3364
EN 280 -2354
EN 300 -3015
EN 320 1027
EN 340 0
EN 360 0
DA-M5:ASN-S1
EN 20 0
EN 40 0
EN 60 0
EN 80 0
EN 100 0
EN 120 0
EN 140 0
EN 160 0

EN 180   0
EN 200   0
EN 220   0
EN 240   -18438
EN 260   0
EN 280   0
EN 300   0
EN 320   0
EN 340   0
EN 360   0
U-Y:LYS-S2
EN 20   -7314
EN 40   -4187
EN 60   -5981
EN 80   -5051
EN 100   -5785
EN 120   -2624
EN 140   -2419
EN 160   -5213
EN 180   -6143
EN 200   -7569
EN 220   -5307
EN 240   -6864
EN 260   -5447
EN 280   -4621
EN 300   -4278
EN 320   -3819
EN 340   -5742
EN 360   -5312
A-P:ILE-S1
EN 20   0
EN 40   -1221
EN 60   -2302
EN 80   -583
EN 100   -389
EN 120   -2380
EN 140   -1170
EN 160   -2870
EN 180   -2464
EN 200   0
EN 220   -1076
EN 240   -2322
EN 260   -2283
EN 280   -2106
EN 300   0
EN 320   -3265
EN 340   -2777
EN 360   -4063
U34-MY:SER-CA
EN 20   0
EN 40   0
EN 60   0
EN 80   0
EN 100   0
EN 120   0
EN 140   -17472
EN 160   0
EN 180   0

EN 200 0
EN 220 0
EN 240 0
EN 260 0
EN 280 0
EN 300 -16900
EN 320 0
EN 340 -18073
EN 360 0
U31-MY:PHE-S1
EN 20 0
EN 40 0
EN 60 0
EN 80 0
EN 100 0
EN 120 0
EN 140 0
EN 160 0
EN 180 0
EN 200 0
EN 220 0
EN 240 0
EN 260 0
EN 280 -17630
EN 300 0
EN 320 0
EN 340 0
EN 360 0
QUO-M5:LEU-CA
EN 20 0
EN 40 0
EN 60 0
EN 80 0
EN 100 0
EN 120 0
EN 140 0
EN 160 0
EN 180 0
EN 200 0
EN 220 0
EN 240 0
EN 260 0
EN 280 -15538
EN 300 0
EN 320 0
EN 340 0
EN 360 0
C-P:ARG-CA
EN 20 0
EN 40 -5398
EN 60 -2923
EN 80 -5991
EN 100 -5796
EN 120 -6964
EN 140 -6231
EN 160 -6374
EN 180 -7264
EN 200 0

EN 220   -432
EN 240   -3777
EN 260   -5224
EN 280   -6194
EN 300   -6494
EN 320   -7060
EN 340   -5766
EN 360   -6523
C31-MY:TYR-S2
EN 20   0
EN 40   0
EN 60   -20623
EN 80   0
EN 100   0
EN 120   0
EN 140   0
EN 160   0
EN 180   0
EN 200   0
EN 220   0
EN 240   0
EN 260   0
EN 280   0
EN 300   0
EN 320   0
EN 340   0
EN 360   0
A-R6:TRP-CA
EN 20   0
EN 40   0
EN 60   -3095
EN 80   -2381
EN 100   -2187
EN 120   -2460
EN 140   0
EN 160   0
EN 180   0
EN 200   0
EN 220   0
EN 240   -3115
EN 260   -5085
EN 280   -2187
EN 300   -2396
EN 320   0
EN 340   -3569
EN 360   0
H2U-MY:TRP-S2
EN 20   0
EN 40   0
EN 60   0
EN 80   0
EN 100   0
EN 120   0
EN 140   0
EN 160   0
EN 180   0
EN 200   0
EN 220   0

EN 240   0
EN 260   0
EN 280   0
EN 300   -16413
EN 320   0
EN 340   0
EN 360   0
G-RIB:THR-S1
EN 20   -6764
EN 40   -3637
EN 60   -1996
EN 80   -4005
EN 100   -4523
EN 120   -3079
EN 140   -4591
EN 160   -3830
EN 180   -3876
EN 200   -7019
EN 220   -4758
EN 240   -2016
EN 260   -3987
EN 280   -3187
EN 300   -3015
EN 320   -1694
EN 340   -3184
EN 360   -4762
C31-P:SER-S1
EN 20   0
EN 40   0
EN 60   0
EN 80   0
EN 100   0
EN 120   0
EN 140   0
EN 160   0
EN 180   0
EN 200   0
EN 220   0
EN 240   0
EN 260   0
EN 280   0
EN 300   0
EN 320   -17296
EN 340   0
EN 360   0
IU-MY:SER-CA
EN 20   0
EN 40   0
EN 60   0
EN 80   -12661
EN 100   0
EN 120   0
EN 140   0
EN 160   0
EN 180   0
EN 200   0
EN 220   0
EN 240   0

EN 260   0
EN 280   0
EN 300   0
EN 320   0
EN 340   0
EN 360   0
H2U-MY:PHE-S2
EN 20   0
EN 40   0
EN 60   0
EN 80   0
EN 100   -13646
EN 120   0
EN 140   0
EN 160   0
EN 180   0
EN 200   0
EN 220   0
EN 240   0
EN 260   0
EN 280   0
EN 300   0
EN 320   0
EN 340   0
EN 360   0
A-R5:ASN-S1
EN 20   -9169
EN 40   -2055
EN 60   -3849
EN 80   -4140
EN 100   -4327
EN 120   -1496
EN 140   -5108
EN 160   -3704
EN 180   0
EN 200   0
EN 220   -4632
EN 240   -5586
EN 260   -3117
EN 280   -2228
EN 300   -2438
EN 320   -1829
EN 340   -889
EN 360   -4567
C-RIB:SER-S1
EN 20   -5923
EN 40   -5067
EN 60   -5595
EN 80   -5434
EN 100   -4885
EN 120   -5158
EN 140   -6021
EN 160   -4446
EN 180   -2322
EN 200   -4461
EN 220   -6086
EN 240   -5813
EN 260   -3858

EN 280   -4235
EN 300   -5095
EN 320   -5490
EN 340   -5065
EN 360   -6643
U34-P:PHE-S2
EN 20   0
EN 40   0
EN 60   0
EN 80   0
EN 100   0
EN 120   0
EN 140   0
EN 160   0
EN 180   0
EN 200   0
EN 220   0
EN 240   0
EN 260   0
EN 280   0
EN 300   -17844
EN 320   0
EN 340   0
EN 360   0
C-Y:ASP-CA
EN 20   -5660
EN 40   -2533
EN 60   -3162
EN 80   -2900
EN 100   -3088
EN 120   -1261
EN 140   -3035
EN 160   -2465
EN 180   0
EN 200   0
EN 220   -4658
EN 240   -3182
EN 260   -3264
EN 280   1733
EN 300   -2464
EN 320   -3693
EN 340   -3084
EN 360   -1940
FHU-MY:TYR-S2
EN 20   0
EN 40   0
EN 60   -17188
EN 80   0
EN 100   -16280
EN 120   0
EN 140   0
EN 160   0
EN 180   0
EN 200   0
EN 220   0
EN 240   -17208
EN 260   0
EN 280   0

EN 300   -16490
EN 320   0
EN 340   0
EN 360   0
U31-RIB:ASP-CA
EN 20   0
EN 40   -18976
EN 60   0
EN 80   -16622
EN 100   0
EN 120   0
EN 140   -17208
EN 160   0
EN 180   0
EN 200   0
EN 220   0
EN 240   0
EN 260   0
EN 280   0
EN 300   0
EN 320   -17033
EN 340   0
EN 360   0
FMU-MY:ASP-CA
EN 20   0
EN 40   0
EN 60   0
EN 80   0
EN 100   0
EN 120   0
EN 140   0
EN 160   -17904
EN 180   0
EN 200   0
EN 220   0
EN 240   0
EN 260   0
EN 280   0
EN 300   0
EN 320   0
EN 340   0
EN 360   0
G-P:LYS-CA
EN 20   0
EN 40   -1829
EN 60   -2910
EN 80   -4626
EN 100   -4724
EN 120   -6423
EN 140   -5363
EN 160   -5196
EN 180   -6507
EN 200   0
EN 220   -1684
EN 240   -5200
EN 260   -4277
EN 280   -5221
EN 300   -5195

EN 320 -5937
EN 340 -6819
EN 360 -6389
A-RIB:GLU-S2
EN 20 0
EN 40 -320
EN 60 -2114
EN 80 -2787
EN 100 510
EN 120 -4400
EN 140 -2992
EN 160 -4692
EN 180 -4286
EN 200 0
EN 220 0
EN 240 -417
EN 260 -2769
EN 280 -2592
EN 300 -2802
EN 320 -3821
EN 340 -3142
EN 360 -1445
U34-RIB:ASN-S1
EN 20 0
EN 40 0
EN 60 -18418
EN 80 -17704
EN 100 0
EN 120 0
EN 140 0
EN 160 0
EN 180 0
EN 200 0
EN 220 0
EN 240 0
EN 260 0
EN 280 -17510
EN 300 0
EN 320 0
EN 340 0
EN 360 0
C31-P:ASP-S2
EN 20 0
EN 40 0
EN 60 0
EN 80 0
EN 100 0
EN 120 -16748
EN 140 0
EN 160 0
EN 180 0
EN 200 0
EN 220 0
EN 240 0
EN 260 0
EN 280 0
EN 300 0
EN 320 -18797

EN 340   0
EN 360   0
A-R5:TRP-CA
EN 20   0
EN 40   -4736
EN 60   0
EN 80   -2381
EN 100   -2187
EN 120   -2460
EN 140   -2968
EN 160   0
EN 180   0
EN 200   0
EN 220   -4591
EN 240   -4832
EN 260   -5798
EN 280   -2187
EN 300   0
EN 320   -2792
EN 340   0
EN 360   0
U31-RIB:MET-S2
EN 20   0
EN 40   0
EN 60   0
EN 80   0
EN 100   0
EN 120   -18943
EN 140   0
EN 160   0
EN 180   0
EN 200   0
EN 220   0
EN 240   0
EN 260   0
EN 280   0
EN 300   0
EN 320   0
EN 340   0
EN 360   0
C-Y:ALA-CA
EN 20   -4918
EN 40   -4514
EN 60   -2421
EN 80   -2872
EN 100   -1965
EN 120   -1233
EN 140   -24
EN 160   -2436
EN 180   0
EN 200   -3456
EN 220   -5373
EN 240   -3606
EN 260   -1689
EN 280   -960
EN 300   -1723
EN 320   151
EN 340   -1630

EN 360 0
U31-P:ARG-S1
EN 20 0
EN 40 0
EN 60 0
EN 80 0
EN 100 0
EN 120 0
EN 140 0
EN 160 0
EN 180 0
EN 200 0
EN 220 0
EN 240 0
EN 260 0
EN 280 0
EN 300 0
EN 320 -16784
EN 340 0
EN 360 0
DA-M5:HIS-CA
EN 20 0
EN 40 0
EN 60 0
EN 80 0
EN 100 0
EN 120 0
EN 140 0
EN 160 0
EN 180 0
EN 200 0
EN 220 0
EN 240 0
EN 260 -18902
EN 280 0
EN 300 0
EN 320 0
EN 340 0
EN 360 0
DA-M5:HIS-S2
EN 20 0
EN 40 0
EN 60 0
EN 80 0
EN 100 0
EN 120 0
EN 140 0
EN 160 0
EN 180 0
EN 200 0
EN 220 0
EN 240 0
EN 260 0
EN 280 -18742
EN 300 0
EN 320 0
EN 340 0
EN 360 0

C-Y:PHE-S1
EN 20 0
EN 40 0
EN 60 0
EN 80 -1381
EN 100 -1187
EN 120 -1460
EN 140 -251
EN 160 -2663
EN 180 0
EN 200 -5401
EN 220 -1873
EN 240 0
EN 260 -2368
EN 280 -2191
EN 300 320
EN 320 -75
EN 340 0
EN 360 -3144
IU-RIB:ARG-CA
EN 20 0
EN 40 0
EN 60 0
EN 80 0
EN 100 0
EN 120 0
EN 140 0
EN 160 0
EN 180 0
EN 200 0
EN 220 0
EN 240 0
EN 260 0
EN 280 -12959
EN 300 0
EN 320 0
EN 340 0
EN 360 0
U31-RIB:ASP-S2
EN 20 0
EN 40 -19024
EN 60 0
EN 80 0
EN 100 -16475
EN 120 0
EN 140 0
EN 160 -17951
EN 180 0
EN 200 0
EN 220 0
EN 240 0
EN 260 0
EN 280 0
EN 300 -16684
EN 320 0
EN 340 -17857
EN 360 0
U31-RIB:THR-S1

EN 20 0
EN 40 0
EN 60 0
EN 80 0
EN 100 -16930
EN 120 -17204
EN 140 0
EN 160 0
EN 180 0
EN 200 0
EN 220 0
EN 240 0
EN 260 0
EN 280 0
EN 300 0
EN 320 0
EN 340 0
EN 360 0
C31-MY:LEU-S2
EN 20 0
EN 40 0
EN 60 0
EN 80 0
EN 100 0
EN 120 0
EN 140 0
EN 160 0
EN 180 0
EN 200 0
EN 220 0
EN 240 0
EN 260 0
EN 280 -15541
EN 300 0
EN 320 0
EN 340 0
EN 360 0
G-R6:TYR-S2
EN 20 0
EN 40 0
EN 60 -3776
EN 80 -3615
EN 100 -2708
EN 120 -4146
EN 140 -1601
EN 160 -909
EN 180 0
EN 200 0
EN 220 -1837
EN 240 -3796
EN 260 -1327
EN 280 -2868
EN 300 -3503
EN 320 -3899
EN 340 -2533
EN 360 -3107
FMU-MY:ASP-S2
EN 20 0

EN 40   0
EN 60   0
EN 80   0
EN 100   0
EN 120   0
EN 140   0
EN 160   -17951
EN 180   0
EN 200   0
EN 220   0
EN 240   0
EN 260   0
EN 280   0
EN 300   0
EN 320   0
EN 340   0
EN 360   0
H2U-RIB:TRP-S2
EN 20   0
EN 40   0
EN 60   0
EN 80   0
EN 100   0
EN 120   0
EN 140   0
EN 160   0
EN 180   0
EN 200   0
EN 220   0
EN 240   -17132
EN 260   0
EN 280   0
EN 300   0
EN 320   0
EN 340   0
EN 360   0
QUO-RIB:LYS-S2
EN 20   0
EN 40   0
EN 60   0
EN 80   0
EN 100   0
EN 120   -16426
EN 140   0
EN 160   0
EN 180   0
EN 200   0
EN 220   0
EN 240   0
EN 260   0
EN 280   0
EN 300   0
EN 320   0
EN 340   0
EN 360   0
FHU-MY:ALA-S1
EN 20   0
EN 40   -16518

```
EN 60   0
EN 80   0
EN 100   0
EN 120   -14242
EN 140   0
EN 160   -15445
EN 180   0
EN 200   0
EN 220   0
EN 240   0
EN 260   0
EN 280   0
EN 300   -14178
EN 320   0
EN 340   0
EN 360   0
A-R6:ARG-S2
EN 20   0
EN 40   -5888
EN 60   -5036
EN 80   -5925
EN 100   -5056
EN 120   -5480
EN 140   -5678
EN 160   -5107
EN 180   -3697
EN 200   0
EN 220   -4579
EN 240   -5985
EN 260   -5525
EN 280   -3008
EN 300   -4752
EN 320   -5502
EN 340   -5014
EN 360   -3578
U34-P:SER-S1
EN 20   0
EN 40   0
EN 60   -17599
EN 80   0
EN 100   0
EN 120   -16964
EN 140   0
EN 160   0
EN 180   0
EN 200   0
EN 220   0
EN 240   0
EN 260   -16867
EN 280   0
EN 300   0
EN 320   0
EN 340   0
EN 360   0
FMU-MY:ASP-S1
EN 20   0
EN 40   0
EN 60   0
```

EN 80   0
EN 100   0
EN 120   0
EN 140   0
EN 160   -17906
EN 180   0
EN 200   0
EN 220   0
EN 240   0
EN 260   0
EN 280   0
EN 300   0
EN 320   0
EN 340   0
EN 360   0
FHU-MY:SER-S1
EN 20   0
EN 40   0
EN 60   0
EN 80   0
EN 100   -14973
EN 120   0
EN 140   0
EN 160   0
EN 180   0
EN 200   0
EN 220   0
EN 240   0
EN 260   0
EN 280   0
EN 300   0
EN 320   0
EN 340   0
EN 360   0
DA-M6:ASN-S1
EN 20   0
EN 40   0
EN 60   0
EN 80   0
EN 100   0
EN 120   0
EN 140   -18291
EN 160   0
EN 180   0
EN 200   0
EN 220   0
EN 240   -18438
EN 260   0
EN 280   0
EN 300   0
EN 320   0
EN 340   0
EN 360   0
C-RIB:LYS-CA
EN 20   -3614
EN 40   -5932
EN 60   -3999
EN 80   -4488

EN 100   -3880
EN 120   -4153
EN 140   -3872
EN 160   -4567
EN 180   -4453
EN 200   -3869
EN 220   -4782
EN 240   -4019
EN 260   -5537
EN 280   -4808
EN 300   -3853
EN 320   -5254
EN 340   -5478
EN 360   -4335
U-RIB:MET-S2
EN 20   0
EN 40   0
EN 60   0
EN 80   -5615
EN 100   -3150
EN 120   -5141
EN 140   0
EN 160   -2909
EN 180   -6943
EN 200   0
EN 220   0
EN 240   -2361
EN 260   -5044
EN 280   -1432
EN 300   -1642
EN 320   -2038
EN 340   -2815
EN 360   0
G-P:MET-S1
EN 20   0
EN 40   0
EN 60   -2747
EN 80   -3038
EN 100   -2844
EN 120   -2112
EN 140   -3625
EN 160   -1598
EN 180   -3914
EN 200   0
EN 220   0
EN 240   -2767
EN 260   -298
EN 280   -1839
EN 300   -331
EN 320   -3449
EN 340   -3222
EN 360   0
A-RIB:TRP-CA
EN 20   0
EN 40   0
EN 60   -3095
EN 80   0
EN 100   -3904

EN 120   -5895
EN 140   -4685
EN 160   -3663
EN 180   -5979
EN 200   0
EN 220   0
EN 240   -3115
EN 260   -4080
EN 280   -3904
EN 300   -2396
EN 320   -4510
EN 340   -5287
EN 360   0
FHU-P:GLY-CA
EN 20   0
EN 40   0
EN 60   0
EN 80   -14551
EN 100   0
EN 120   0
EN 140   0
EN 160   0
EN 180   0
EN 200   0
EN 220   0
EN 240   0
EN 260   -14533
EN 280   -14357
EN 300   -14566
EN 320   -14962
EN 340   0
EN 360   0
FHU-P:ARG-S2
EN 20   0
EN 40   0
EN 60   0
EN 80   -14668
EN 100   -14474
EN 120   0
EN 140   0
EN 160   0
EN 180   0
EN 200   0
EN 220   0
EN 240   0
EN 260   0
EN 280   -14473
EN 300   0
EN 320   0
EN 340   0
EN 360   0
FHU-RIB:TYR-S2
EN 20   0
EN 40   0
EN 60   0
EN 80   -16474
EN 100   0
EN 120   -16553

EN 140   0
EN 160   0
EN 180   0
EN 200   0
EN 220   0
EN 240   0
EN 260   -16456
EN 280   0
EN 300   -16490
EN 320   0
EN 340   0
EN 360   0
FMU-P:GLN-CA
EN 20   0
EN 40   0
EN 60   -18661
EN 80   0
EN 100   0
EN 120   0
EN 140   0
EN 160   0
EN 180   0
EN 200   0
EN 220   0
EN 240   0
EN 260   0
EN 280   0
EN 300   0
EN 320   0
EN 340   0
EN 360   0
C-P:GLU-CA
EN 20   0
EN 40   -134
EN 60   -1928
EN 80   -501
EN 100   -2024
EN 120   -2680
EN 140   -2354
EN 160   -779
EN 180   -4812
EN 200   0
EN 220   -2711
EN 240   1486
EN 260   -483
EN 280   -3029
EN 300   -3239
EN 320   -3343
EN 340   -2955
EN 360   -1259
C31-RIB:GLN-S2
EN 20   0
EN 40   0
EN 60   0
EN 80   0
EN 100   0
EN 120   0
EN 140   -18565

EN 160 0
EN 180 0
EN 200 0
EN 220 0
EN 240 0
EN 260 0
EN 280 0
EN 300 0
EN 320 0
EN 340 0
EN 360 0
FMU-MY:ARG-S2
EN 20 0
EN 40 -18740
EN 60 0
EN 80 0
EN 100 0
EN 120 0
EN 140 0
EN 160 0
EN 180 0
EN 200 0
EN 220 0
EN 240 0
EN 260 0
EN 280 0
EN 300 0
EN 320 0
EN 340 0
EN 360 0
GTP-M5:ALA-S1
EN 20 0
EN 40 0
EN 60 0
EN 80 0
EN 100 0
EN 120 0
EN 140 0
EN 160 0
EN 180 0
EN 200 0
EN 220 0
EN 240 0
EN 260 0
EN 280 -15686
EN 300 0
EN 320 0
EN 340 0
EN 360 0
FMU-P:PHE-S1
EN 20 0
EN 40 0
EN 60 0
EN 80 0
EN 100 -17630
EN 120 0
EN 140 0
EN 160 0

EN 180   0
EN 200   0
EN 220   0
EN 240   0
EN 260   0
EN 280   0
EN 300   0
EN 320   0
EN 340   0
EN 360   0
C-Y:ARG-S2
EN 20   -8145
EN 40   -5401
EN 60   -6361
EN 80   -6733
EN 100   -5904
EN 120   -5726
EN 140   -4162
EN 160   -4328
EN 180   -5257
EN 200   -9113
EN 220   -6591
EN 240   -5978
EN 260   -6279
EN 280   -5799
EN 300   -4595
EN 320   -3457
EN 340   -2135
EN 360   -7646
QUO-M5:PHE-S2
EN 20   0
EN 40   0
EN 60   0
EN 80   0
EN 100   0
EN 120   0
EN 140   0
EN 160   0
EN 180   0
EN 200   0
EN 220   0
EN 240   0
EN 260   -17810
EN 280   0
EN 300   0
EN 320   -18239
EN 340   0
EN 360   0
C31-P:ASP-CA
EN 20   0
EN 40   0
EN 60   0
EN 80   0
EN 100   0
EN 120   -16700
EN 140   -17208
EN 160   0
EN 180   0

EN 200   0
EN 220   0
EN 240   0
EN 260   0
EN 280   0
EN 300   0
EN 320   -17033
EN 340   0
EN 360   0
U34-P:SER-CA
EN 20   0
EN 40   0
EN 60   0
EN 80   -16885
EN 100   0
EN 120   -16964
EN 140   0
EN 160   0
EN 180   0
EN 200   0
EN 220   0
EN 240   0
EN 260   0
EN 280   0
EN 300   0
EN 320   0
EN 340   0
EN 360   0
H2U-P:ASN-S1
EN 20   0
EN 40   0
EN 60   0
EN 80   0
EN 100   -13522
EN 120   -13795
EN 140   -14303
EN 160   0
EN 180   0
EN 200   0
EN 220   0
EN 240   0
EN 260   0
EN 280   0
EN 300   0
EN 320   0
EN 340   0
EN 360   0
G-RIB:GLN-S1
EN 20   -4865
EN 40   -3456
EN 60   -4085
EN 80   -4827
EN 100   -3628
EN 120   -4283
EN 140   -4791
EN 160   -6370
EN 180   -7421
EN 200   0

EN 220   -4315
EN 240   -2839
EN 260   -3805
EN 280   -2624
EN 300   -4220
EN 320   -3782
EN 340   -4006
EN 360   -8015
C31-P:GLN-CA
EN 20   0
EN 40   0
EN 60   0
EN 80   0
EN 100   0
EN 120   0
EN 140   0
EN 160   -19230
EN 180   0
EN 200   0
EN 220   0
EN 240   0
EN 260   0
EN 280   0
EN 300   0
EN 320   0
EN 340   0
EN 360   0
A-RIB:LYS-S1
EN 20   -3777
EN 40   -3372
EN 60   -4950
EN 80   -5005
EN 100   -4811
EN 120   -3196
EN 140   -5039
EN 160   -3012
EN 180   -4616
EN 200   -6754
EN 220   -4493
EN 240   -5568
EN 260   -5439
EN 280   -2922
EN 300   -4252
EN 320   -5868
EN 340   -5189
EN 360   -5210
QUO-P:LEU-S1
EN 20   0
EN 40   0
EN 60   0
EN 80   -15733
EN 100   -15538
EN 120   0
EN 140   0
EN 160   0
EN 180   0
EN 200   0
EN 220   0

EN 240 0
EN 260 0
EN 280 0
EN 300 0
EN 320 0
EN 340 0
EN 360 0
I-RIB:ALA-S1
EN 20 0
EN 40 0
EN 60 0
EN 80 0
EN 100 0
EN 120 0
EN 140 0
EN 160 -17163
EN 180 0
EN 200 0
EN 220 0
EN 240 0
EN 260 0
EN 280 0
EN 300 0
EN 320 0
EN 340 0
EN 360 0
H2U-RIB:GLY-CA
EN 20 0
EN 40 0
EN 60 0
EN 80 0
EN 100 0
EN 120 0
EN 140 0
EN 160 0
EN 180 0
EN 200 0
EN 220 0
EN 240 0
EN 260 0
EN 280 0
EN 300 0
EN 320 -12692
EN 340 0
EN 360 0
H2U-P:LYS-S2
EN 20 0
EN 40 -14714
EN 60 0
EN 80 0
EN 100 0
EN 120 0
EN 140 0
EN 160 0
EN 180 -15958
EN 200 0
EN 220 0
EN 240 0

EN 260   0
EN 280   0
EN 300   0
EN 320   0
EN 340   0
EN 360   0
QUO-M6:LEU-S1
EN 20   0
EN 40   0
EN 60   0
EN 80   0
EN 100   0
EN 120   0
EN 140   0
EN 160   0
EN 180   0
EN 200   0
EN 220   0
EN 240   0
EN 260   -15715
EN 280   0
EN 300   0
EN 320   0
EN 340   0
EN 360   0
QUO-RIB:ASN-S1
EN 20   0
EN 40   0
EN 60   0
EN 80   0
EN 100   0
EN 120   0
EN 140   0
EN 160   -18986
EN 180   0
EN 200   0
EN 220   0
EN 240   0
EN 260   0
EN 280   0
EN 300   0
EN 320   0
EN 340   0
EN 360   0
FHU-MY:CYS-S1
EN 20   0
EN 40   0
EN 60   0
EN 80   0
EN 100   0
EN 120   0
EN 140   0
EN 160   0
EN 180   0
EN 200   0
EN 220   0
EN 240   0
EN 260   0

EN 280   -18968
EN 300   0
EN 320   0
EN 340   0
EN 360   0
FMU-P:ARG-S2
EN 20   0
EN 40   0
EN 60   0
EN 80   0
EN 100   0
EN 120   -18182
EN 140   0
EN 160   0
EN 180   0
EN 200   0
EN 220   0
EN 240   -17119
EN 260   0
EN 280   0
EN 300   0
EN 320   0
EN 340   0
EN 360   0
OMC-MY:LYS-S2
EN 20   0
EN 40   0
EN 60   -13244
EN 80   0
EN 100   0
EN 120   0
EN 140   0
EN 160   0
EN 180   0
EN 200   0
EN 220   0
EN 240   0
EN 260   0
EN 280   0
EN 300   0
EN 320   0
EN 340   0
EN 360   0
A-R5:GLN-S1
EN 20   0
EN 40   -5020
EN 60   -3379
EN 80   -3378
EN 100   -2471
EN 120   -4462
EN 140   -3252
EN 160   -2943
EN 180   -5259
EN 200   -5680
EN 220   -4875
EN 240   -2395
EN 260   -4365
EN 280   250

EN 300 -3393
EN 320 -3076
EN 340 0
EN 360 0
U-RIB:LYS-CA
EN 20 0
EN 40 -3129
EN 60 229
EN 80 -4501
EN 100 -5732
EN 120 -5077
EN 140 -3078
EN 160 -5783
EN 180 -4372
EN 200 -4793
EN 220 -4701
EN 240 -3778
EN 260 -5195
EN 280 -5019
EN 300 -1794
EN 320 -4620
EN 340 -5397
EN 360 -2536
FHU-RIB:LYS-S1
EN 20 0
EN 40 -16937
EN 60 0
EN 80 0
EN 100 0
EN 120 0
EN 140 0
EN 160 0
EN 180 0
EN 200 0
EN 220 0
EN 240 -15316
EN 260 0
EN 280 0
EN 300 0
EN 320 0
EN 340 0
EN 360 0
U-RIB:LYS-S2
EN 20 -8026
EN 40 -7170
EN 60 -5981
EN 80 -5649
EN 100 -5785
EN 120 -5346
EN 140 -3685
EN 160 -3114
EN 180 -5430
EN 200 -9286
EN 220 -8481
EN 240 -6714
EN 260 -6387
EN 280 -6338
EN 300 -5067

EN 320   -3961
EN 340   -5451
EN 360   -6025
C-Y:CYS-CA
EN 20   0
EN 40   0
EN 60   -3433
EN 80   0
EN 100   0
EN 120   -2798
EN 140   0
EN 160   0
EN 180   0
EN 200   0
EN 220   0
EN 240   0
EN 260   0
EN 280   0
EN 300   -2735
EN 320   0
EN 340   0
EN 360   0
FMU-MY:MET-S1
EN 20   0
EN 40   0
EN 60   0
EN 80   0
EN 100   0
EN 120   0
EN 140   0
EN 160   0
EN 180   0
EN 200   0
EN 220   0
EN 240   0
EN 260   0
EN 280   -18659
EN 300   0
EN 320   0
EN 340   0
EN 360   0
H2U-P:PRO-CA
EN 20   0
EN 40   0
EN 60   0
EN 80   0
EN 100   -13142
EN 120   0
EN 140   0
EN 160   0
EN 180   0
EN 200   0
EN 220   0
EN 240   0
EN 260   0
EN 280   0
EN 300   0
EN 320   0

EN 340 0
EN 360 0
G-P:SER-S1
EN 20 0
EN 40 -5142
EN 60 -3501
EN 80 -3500
EN 100 -4310
EN 120 -5137
EN 140 -6478
EN 160 -5787
EN 180 -3664
EN 200 0
EN 220 -3992
EN 240 -2517
EN 260 -5200
EN 280 -5173
EN 300 -4520
EN 320 -4464
EN 340 -5478
EN 360 -3545
H2U-RIB:LEU-S2
EN 20 0
EN 40 0
EN 60 0
EN 80 0
EN 100 0
EN 120 0
EN 140 0
EN 160 0
EN 180 0
EN 200 0
EN 220 0
EN 240 -12482
EN 260 0
EN 280 0
EN 300 0
EN 320 0
EN 340 0
EN 360 0
U-RIB:VAL-CA
EN 20 0
EN 40 0
EN 60 -1364
EN 80 -650
EN 100 -3178
EN 120 -2999
EN 140 -1237
EN 160 0
EN 180 -4248
EN 200 0
EN 220 0
EN 240 0
EN 260 -3354
EN 280 -456
EN 300 -4100
EN 320 -1061
EN 340 -121

EN 360   -2413
H2U-P:GLU-S2
EN 20   0
EN 40   0
EN 60   -12696
EN 80   0
EN 100   0
EN 120   0
EN 140   -12569
EN 160   0
EN 180   0
EN 200   0
EN 220   0
EN 240   0
EN 260   0
EN 280   0
EN 300   0
EN 320   0
EN 340   0
EN 360   0
C31-P:LEU-S2
EN 20   0
EN 40   -18091
EN 60   0
EN 80   0
EN 100   0
EN 120   0
EN 140   0
EN 160   0
EN 180   0
EN 200   0
EN 220   0
EN 240   0
EN 260   0
EN 280   0
EN 300   0
EN 320   0
EN 340   0
EN 360   0
QUO-M5:GLU-S1
EN 20   0
EN 40   0
EN 60   0
EN 80   0
EN 100   0
EN 120   0
EN 140   -16524
EN 160   0
EN 180   0
EN 200   0
EN 220   0
EN 240   0
EN 260   0
EN 280   0
EN 300   0
EN 320   0
EN 340   0
EN 360   0

QUO-M5:ASP-S1
EN 20   0
EN 40   0
EN 60   0
EN 80   0
EN 100   0
EN 120   -16703
EN 140   0
EN 160   0
EN 180   0
EN 200   0
EN 220   0
EN 240   0
EN 260   0
EN 280   0
EN 300   0
EN 320   -17035
EN 340   0
EN 360   0
FMU-RIB:ALA-CA
EN 20   0
EN 40   0
EN 60   0
EN 80   0
EN 100   -15686
EN 120   0
EN 140   0
EN 160   0
EN 180   0
EN 200   0
EN 220   0
EN 240   0
EN 260   0
EN 280   0
EN 300   0
EN 320   0
EN 340   0
EN 360   0
U-Y:TRP-S1
EN 20   0
EN 40   0
EN 60   -5579
EN 80   -3148
EN 100   -5676
EN 120   0
EN 140   0
EN 160   0
EN 180   0
EN 200   0
EN 220   -5358
EN 240   0
EN 260   -3130
EN 280   -2954
EN 300   0
EN 320   0
EN 340   -4337
EN 360   0
DA-M6:LYS-CA

EN 20   0
EN 40   0
EN 60   0
EN 80   0
EN 100   0
EN 120   0
EN 140   0
EN 160   0
EN 180   0
EN 200   0
EN 220   0
EN 240   0
EN 260   0
EN 280   -16099
EN 300   0
EN 320   0
EN 340   0
EN 360   0
DA-M5:ASN-CA
EN 20   0
EN 40   0
EN 60   0
EN 80   0
EN 100   0
EN 120   0
EN 140   0
EN 160   0
EN 180   0
EN 200   0
EN 220   0
EN 240   -18438
EN 260   0
EN 280   0
EN 300   0
EN 320   0
EN 340   0
EN 360   0
A-R6:LYS-CA
EN 20   0
EN 40   -3366
EN 60   -2438
EN 80   -1724
EN 100   -2917
EN 120   -2808
EN 140   -3316
EN 160   -2294
EN 180   0
EN 200   0
EN 220   -499
EN 240   -3011
EN 260   -3424
EN 280   -4036
EN 300   -2293
EN 320   -2688
EN 340   521
EN 360   0
QUO-M6:ARG-CA
EN 20   0

EN 40 0
EN 60 0
EN 80 0
EN 100 0
EN 120 0
EN 140 -16960
EN 160 0
EN 180 0
EN 200 0
EN 220 0
EN 240 0
EN 260 0
EN 280 0
EN 300 0
EN 320 0
EN 340 0
EN 360 0
U31-P:TYR-S1
EN 20 0
EN 40 0
EN 60 0
EN 80 -18187
EN 100 0
EN 120 0
EN 140 0
EN 160 0
EN 180 0
EN 200 0
EN 220 0
EN 240 0
EN 260 0
EN 280 0
EN 300 0
EN 320 0
EN 340 0
EN 360 0
5BU-MY:PRO-CA
EN 20 0
EN 40 0
EN 60 -16320
EN 80 0
EN 100 0
EN 120 0
EN 140 0
EN 160 0
EN 180 0
EN 200 0
EN 220 0
EN 240 0
EN 260 0
EN 280 0
EN 300 0
EN 320 0
EN 340 0
EN 360 0
FMU-P:CYS-S1
EN 20 0
EN 40 0

EN 60   0
EN 80   0
EN 100   0
EN 120   0
EN 140   0
EN 160   0
EN 180   0
EN 200   -26617
EN 220   0
EN 240   0
EN 260   0
EN 280   0
EN 300   0
EN 320   0
EN 340   0
EN 360   0
H2U-RIB:PRO-CA
EN 20   0
EN 40   0
EN 60   0
EN 80   0
EN 100   0
EN 120   0
EN 140   0
EN 160   0
EN 180   0
EN 200   0
EN 220   0
EN 240   0
EN 260   0
EN 280   0
EN 300   -13352
EN 320   0
EN 340   0
EN 360   0
IU-P:ARG-S1
EN 20   0
EN 40   0
EN 60   -14365
EN 80   0
EN 100   0
EN 120   0
EN 140   0
EN 160   0
EN 180   0
EN 200   0
EN 220   0
EN 240   0
EN 260   0
EN 280   0
EN 300   0
EN 320   0
EN 340   -14840
EN 360   0
IU-RIB:PRO-CA
EN 20   0
EN 40   0
EN 60   0

EN 80   0
EN 100   0
EN 120   0
EN 140   -14692
EN 160   0
EN 180   0
EN 200   0
EN 220   0
EN 240   0
EN 260   0
EN 280   0
EN 300   0
EN 320   0
EN 340   0
EN 360   0
U34-MY:VAL-CA
EN 20   0
EN 40   0
EN 60   0
EN 80   0
EN 100   0
EN 120   0
EN 140   0
EN 160   0
EN 180   0
EN 200   0
EN 220   0
EN 240   0
EN 260   0
EN 280   0
EN 300   0
EN 320   0
EN 340   -17358
EN 360   0
C-P:ILE-CA
EN 20   0
EN 40   0
EN 60   -3870
EN 80   -2151
EN 100   -240
EN 120   -4952
EN 140   -2738
EN 160   -1716
EN 180   -2315
EN 200   0
EN 220   0
EN 240   0
EN 260   -3851
EN 280   -1244
EN 300   -3553
EN 320   -3567
EN 340   -3340
EN 360   -2196
U-Y:TYR-S2
EN 20   0
EN 40   -3309
EN 60   0
EN 80   -4174

EN 100 -2478
EN 120 -2751
EN 140 -3259
EN 160 -2237
EN 180 0
EN 200 0
EN 220 -3164
EN 240 -4411
EN 260 -4371
EN 280 -4748
EN 300 -5791
EN 320 -4800
EN 340 0
EN 360 0
G-R6:THR-S1
EN 20 0
EN 40 -915
EN 60 -1996
EN 80 -2548
EN 100 -3518
EN 120 -3079
EN 140 -1869
EN 160 156
EN 180 0
EN 200 0
EN 220 -3492
EN 240 -4739
EN 260 -1264
EN 280 -2354
EN 300 -3397
EN 320 -2406
EN 340 250
EN 360 0
QUO-P:LEU-S2
EN 20 0
EN 40 0
EN 60 0
EN 80 -17453
EN 100 0
EN 120 0
EN 140 0
EN 160 0
EN 180 0
EN 200 0
EN 220 0
EN 240 0
EN 260 0
EN 280 0
EN 300 0
EN 320 0
EN 340 0
EN 360 0
C31-P:MET-S1
EN 20 0
EN 40 0
EN 60 0
EN 80 0
EN 100 0

EN 120   0
EN 140   0
EN 160   0
EN 180   0
EN 200   0
EN 220   0
EN 240   0
EN 260   0
EN 280   0
EN 300   0
EN 320   -19264
EN 340   0
EN 360   0
H2U-P:ARG-S2
EN 20   0
EN 40   0
EN 60   0
EN 80   -12398
EN 100   0
EN 120   0
EN 140   0
EN 160   0
EN 180   0
EN 200   0
EN 220   0
EN 240   0
EN 260   0
EN 280   0
EN 300   0
EN 320   0
EN 340   0
EN 360   0
QUO-RIB:PHE-S1
EN 20   0
EN 40   0
EN 60   0
EN 80   0
EN 100   0
EN 120   0
EN 140   0
EN 160   0
EN 180   0
EN 200   0
EN 220   0
EN 240   0
EN 260   0
EN 280   0
EN 300   -17840
EN 320   0
EN 340   0
EN 360   0
U34-P:TYR-S1
EN 20   0
EN 40   0
EN 60   0
EN 80   0
EN 100   0
EN 120   0

EN 140 0
EN 160 0
EN 180 0
EN 200 0
EN 220 0
EN 240 0
EN 260 0
EN 280 0
EN 300 0
EN 320 -18599
EN 340 0
EN 360 0
FMU-RIB:GLU-S2
EN 20 0
EN 40 0
EN 60 0
EN 80 0
EN 100 0
EN 120 0
EN 140 0
EN 160 0
EN 180 0
EN 200 0
EN 220 0
EN 240 0
EN 260 0
EN 280 0
EN 300 0
EN 320 0
EN 340 -17158
EN 360 0
QUO-M5:ASN-S1
EN 20 0
EN 40 0
EN 60 0
EN 80 -17704
EN 100 0
EN 120 0
EN 140 0
EN 160 0
EN 180 0
EN 200 0
EN 220 0
EN 240 0
EN 260 0
EN 280 0
EN 300 0
EN 320 0
EN 340 0
EN 360 0
G-P:CYS-S1
EN 20 0
EN 40 0
EN 60 0
EN 80 -2343
EN 100 0
EN 120 -2422
EN 140 -2930

EN 160 0
EN 180 0
EN 200 0
EN 220 0
EN 240 0
EN 260 0
EN 280 0
EN 300 0
EN 320 -2754
EN 340 0
EN 360 -5823
A-R5:PRO-CA
EN 20 -4801
EN 40 -3392
EN 60 -3468
EN 80 -3307
EN 100 -2560
EN 120 -3386
EN 140 -3894
EN 160 0
EN 180 0
EN 200 -5056
EN 220 -4251
EN 240 -3488
EN 260 -2024
EN 280 -3565
EN 300 -4487
EN 320 -1448
EN 340 -3230
EN 360 0
H2U-RIB:PHE-S1
EN 20 0
EN 40 0
EN 60 0
EN 80 0
EN 100 0
EN 120 0
EN 140 0
EN 160 0
EN 180 0
EN 200 0
EN 220 0
EN 240 0
EN 260 0
EN 280 -13643
EN 300 0
EN 320 0
EN 340 0
EN 360 0
C31-RIB:GLU-S2
EN 20 0
EN 40 0
EN 60 -16683
EN 80 0
EN 100 0
EN 120 -16049
EN 140 0
EN 160 0

EN 180   0
EN 200   0
EN 220   0
EN 240   0
EN 260   0
EN 280   0
EN 300   0
EN 320   0
EN 340   0
EN 360   0
U-RIB:GLU-CA
EN 20   -4175
EN 40   -2766
EN 60   -1125
EN 80   -1416
EN 100   -2487
EN 120   -1494
EN 140   -998
EN 160   23
EN 180   0
EN 200   0
EN 220   -903
EN 240   572
EN 260   -2110
EN 280   -216
EN 300   -426
EN 320   -2539
EN 340   -3317
EN 360   -2173
IU-MY:PRO-S1
EN 20   0
EN 40   0
EN 60   0
EN 80   -14105
EN 100   0
EN 120   0
EN 140   0
EN 160   0
EN 180   0
EN 200   0
EN 220   0
EN 240   0
EN 260   0
EN 280   0
EN 300   -13115
EN 320   0
EN 340   0
EN 360   0
G-P:TYR-S2
EN 20   0
EN 40   0
EN 60   -2086
EN 80   -4476
EN 100   -3900
EN 120   -4886
EN 140   -4681
EN 160   -5376
EN 180   -3253

EN 200 0
EN 220 0
EN 240 -2106
EN 260 -4076
EN 280 -1177
EN 300 -2392
EN 320 -3500
EN 340 -3565
EN 360 0
GTP-M6:SER-S1
EN 20 0
EN 40 0
EN 60 -17599
EN 80 0
EN 100 0
EN 120 0
EN 140 0
EN 160 0
EN 180 0
EN 200 0
EN 220 0
EN 240 0
EN 260 0
EN 280 -16691
EN 300 0
EN 320 0
EN 340 0
EN 360 0
FHU-MY:ARG-S1
EN 20 0
EN 40 0
EN 60 0
EN 80 0
EN 100 -14461
EN 120 0
EN 140 -15243
EN 160 0
EN 180 0
EN 200 0
EN 220 0
EN 240 0
EN 260 0
EN 280 -14461
EN 300 0
EN 320 -15067
EN 340 0
EN 360 0
FMU-RIB:CYS-S1
EN 20 0
EN 40 0
EN 60 0
EN 80 0
EN 100 0
EN 120 0
EN 140 0
EN 160 -22162
EN 180 0
EN 200 0

EN 220   0
EN 240   0
EN 260   0
EN 280   0
EN 300   0
EN 320   0
EN 340   0
EN 360   0
U-P:HIS-CA
EN 20   0
EN 40   -6764
EN 60   -5123
EN 80   -3405
EN 100   -3211
EN 120   -5201
EN 140   -3992
EN 160   -6404
EN 180   0
EN 200   0
EN 220   0
EN 240   -5856
EN 260   -6109
EN 280   -5932
EN 300   -4425
EN 320   -3816
EN 340   -6864
EN 360   0
C31-P:TYR-S2
EN 20   0
EN 40   0
EN 60   0
EN 80   -18192
EN 100   0
EN 120   0
EN 140   0
EN 160   0
EN 180   0
EN 200   0
EN 220   0
EN 240   0
EN 260   -18174
EN 280   0
EN 300   0
EN 320   0
EN 340   0
EN 360   0
FHU-P:ALA-CA
EN 20   0
EN 40   0
EN 60   0
EN 80   0
EN 100   0
EN 120   0
EN 140   0
EN 160   0
EN 180   0
EN 200   0
EN 220   0

EN 240 -14897
EN 260 0
EN 280 0
EN 300 0
EN 320 0
EN 340 0
EN 360 0
FHU-MY:ARG-CA
EN 20 0
EN 40 0
EN 60 0
EN 80 0
EN 100 0
EN 120 -14735
EN 140 -16960
EN 160 0
EN 180 0
EN 200 0
EN 220 0
EN 240 0
EN 260 0
EN 280 0
EN 300 -14671
EN 320 -15067
EN 340 0
EN 360 0
FHU-P:ALA-S1
EN 20 0
EN 40 0
EN 60 0
EN 80 0
EN 100 0
EN 120 0
EN 140 0
EN 160 0
EN 180 0
EN 200 0
EN 220 0
EN 240 -14897
EN 260 0
EN 280 0
EN 300 0
EN 320 0
EN 340 0
EN 360 0
G-R6:LEU-CA
EN 20 0
EN 40 -2958
EN 60 2117
EN 80 -603
EN 100 303
EN 120 2752
EN 140 -477
EN 160 -1885
EN 180 0
EN 200 0
EN 220 -1096
EN 240 -624

EN 260   -3092
EN 280   -962
EN 300   -619
EN 320   -1567
EN 340   0
EN 360   0
FHU-RIB:GLY-CA
EN 20   0
EN 40   -18623
EN 60   -15265
EN 80   0
EN 100   0
EN 120   0
EN 140   0
EN 160   0
EN 180   0
EN 200   0
EN 220   0
EN 240   -15285
EN 260   0
EN 280   0
EN 300   0
EN 320   0
EN 340   0
EN 360   0
C31-RIB:PHE-CA
EN 20   0
EN 40   0
EN 60   0
EN 80   0
EN 100   -17630
EN 120   -17904
EN 140   0
EN 160   0
EN 180   0
EN 200   0
EN 220   0
EN 240   0
EN 260   0
EN 280   0
EN 300   0
EN 320   0
EN 340   0
EN 360   0
U31-MY:GLN-S1
EN 20   0
EN 40   0
EN 60   -18661
EN 80   0
EN 100   0
EN 120   0
EN 140   0
EN 160   0
EN 180   0
EN 200   0
EN 220   0
EN 240   0
EN 260   0

EN 280   0
EN 300   0
EN 320   0
EN 340   0
EN 360   0
C31-P:GLU-CA
EN 20   0
EN 40   0
EN 60   0
EN 80   -15931
EN 100   0
EN 120   0
EN 140   0
EN 160   0
EN 180   0
EN 200   0
EN 220   0
EN 240   0
EN 260   0
EN 280   0
EN 300   0
EN 320   0
EN 340   0
EN 360   0
U-Y:TRP-CA
EN 20   0
EN 40   -5503
EN 60   0
EN 80   0
EN 100   -5676
EN 120   0
EN 140   0
EN 160   0
EN 180   0
EN 200   0
EN 220   -5358
EN 240   0
EN 260   -3130
EN 280   0
EN 300   -3164
EN 320   0
EN 340   -4337
EN 360   0
FHU-RIB:PRO-S1
EN 20   0
EN 40   0
EN 60   0
EN 80   0
EN 100   0
EN 120   0
EN 140   0
EN 160   -16889
EN 180   0
EN 200   0
EN 220   0
EN 240   0
EN 260   0
EN 280   0

EN 300   0
EN 320   0
EN 340   0
EN 360   0
DA-M5:SER-S1
EN 20   0
EN 40   0
EN 60   0
EN 80   0
EN 100   0
EN 120   0
EN 140   0
EN 160   0
EN 180   0
EN 200   0
EN 220   0
EN 240   0
EN 260   0
EN 280   -16691
EN 300   0
EN 320   0
EN 340   0
EN 360   -20365
DA-M6:GLN-S1
EN 20   0
EN 40   0
EN 60   0
EN 80   0
EN 100   0
EN 120   0
EN 140   0
EN 160   0
EN 180   0
EN 200   0
EN 220   0
EN 240   0
EN 260   -17929
EN 280   0
EN 300   0
EN 320   0
EN 340   0
EN 360   0
IU-P:ILE-CA
EN 20   0
EN 40   0
EN 60   0
EN 80   -14146
EN 100   0
EN 120   0
EN 140   0
EN 160   0
EN 180   0
EN 200   0
EN 220   0
EN 240   0
EN 260   0
EN 280   0
EN 300   0

EN 320   0
EN 340   0
EN 360   0
QUO-M6:LYS-S1
EN 20   0
EN 40   0
EN 60   0
EN 80   -16300
EN 100   0
EN 120   0
EN 140   0
EN 160   0
EN 180   0
EN 200   0
EN 220   0
EN 240   0
EN 260   0
EN 280   0
EN 300   0
EN 320   0
EN 340   0
EN 360   0
H2U-MY:TRP-S1
EN 20   0
EN 40   0
EN 60   0
EN 80   0
EN 100   0
EN 120   0
EN 140   0
EN 160   0
EN 180   0
EN 200   0
EN 220   0
EN 240   0
EN 260   0
EN 280   0
EN 300   0
EN 320   -16809
EN 340   0
EN 360   0
C-RIB:HIS-S1
EN 20   0
EN 40   -4831
EN 60   -4907
EN 80   -2476
EN 100   -5386
EN 120   -3560
EN 140   -1346
EN 160   -6480
EN 180   -7792
EN 200   -6496
EN 220   0
EN 240   -5481
EN 260   -4176
EN 280   -5004
EN 300   -6479
EN 320   -5991

EN 340   -3665
EN 360   -6961
QUO-M5:ARG-CA
EN 20   0
EN 40   0
EN 60   0
EN 80   0
EN 100   0
EN 120   -16452
EN 140   0
EN 160   0
EN 180   0
EN 200   0
EN 220   0
EN 240   0
EN 260   0
EN 280   0
EN 300   0
EN 320   0
EN 340   0
EN 360   0
C31-MY:THR-S1
EN 20   0
EN 40   0
EN 60   0
EN 80   0
EN 100   0
EN 120   0
EN 140   0
EN 160   0
EN 180   0
EN 200   0
EN 220   -19334
EN 240   0
EN 260   0
EN 280   0
EN 300   0
EN 320   0
EN 340   0
EN 360   0
QUO-M6:PHE-S1
EN 20   0
EN 40   0
EN 60   0
EN 80   0
EN 100   0
EN 120   0
EN 140   0
EN 160   0
EN 180   0
EN 200   0
EN 220   0
EN 240   0
EN 260   -17807
EN 280   0
EN 300   0
EN 320   -18236
EN 340   0

EN 360   0
U34-RIB:ASN-S2
EN 20   0
EN 40   0
EN 60   -18439
EN 80   -17725
EN 100   0
EN 120   0
EN 140   0
EN 160   0
EN 180   0
EN 200   0
EN 220   0
EN 240   0
EN 260   0
EN 280   0
EN 300   -17741
EN 320   0
EN 340   0
EN 360   0
C31-MY:GLU-CA
EN 20   0
EN 40   0
EN 60   0
EN 80   0
EN 100   0
EN 120   -16009
EN 140   0
EN 160   0
EN 180   0
EN 200   0
EN 220   0
EN 240   0
EN 260   0
EN 280   0
EN 300   0
EN 320   0
EN 340   0
EN 360   0
U31-P:ASP-S2
EN 20   0
EN 40   0
EN 60   0
EN 80   0
EN 100   -16475
EN 120   0
EN 140   0
EN 160   0
EN 180   0
EN 200   0
EN 220   0
EN 240   0
EN 260   -16651
EN 280   0
EN 300   -16684
EN 320   0
EN 340   0
EN 360   0

H2U-P:ASN-CA
EN 20 0
EN 40 0
EN 60 0
EN 80 0
EN 100 0
EN 120 -13795
EN 140 -14303
EN 160 0
EN 180 0
EN 200 0
EN 220 0
EN 240 0
EN 260 0
EN 280 0
EN 300 0
EN 320 0
EN 340 0
EN 360 0
H2U-RIB:ASN-S1
EN 20 0
EN 40 0
EN 60 0
EN 80 0
EN 100 0
EN 120 0
EN 140 0
EN 160 0
EN 180 0
EN 200 0
EN 220 0
EN 240 0
EN 260 -13699
EN 280 0
EN 300 -13732
EN 320 0
EN 340 0
EN 360 0
C-Y:MET-S1
EN 20 0
EN 40 -5769
EN 60 -3123
EN 80 -3414
EN 100 -3220
EN 120 0
EN 140 0
EN 160 0
EN 180 0
EN 200 -6429
EN 220 -4619
EN 240 -3143
EN 260 -674
EN 280 -3220
EN 300 -707
EN 320 0
EN 340 0
EN 360 0
FMU-RIB:ASN-S1

EN 20   0
EN 40   0
EN 60   0
EN 80   0
EN 100   0
EN 120   0
EN 140   -18291
EN 160   0
EN 180   0
EN 200   0
EN 220   0
EN 240   0
EN 260   0
EN 280   0
EN 300   0
EN 320   0
EN 340   0
EN 360   0
A-R6:SER-CA
EN 20   -4362
EN 40   -2953
EN 60   -2317
EN 80   -1603
EN 100   -404
EN 120   -3399
EN 140   -4620
EN 160   -2885
EN 180   0
EN 200   0
EN 220   -3813
EN 240   -3603
EN 260   -2850
EN 280   -2121
EN 300   -2884
EN 320   -2727
EN 340   -4057
EN 360   -4078
DA-M6:LEU-S1
EN 20   0
EN 40   0
EN 60   0
EN 80   0
EN 100   0
EN 120   -15811
EN 140   0
EN 160   0
EN 180   0
EN 200   0
EN 220   0
EN 240   0
EN 260   0
EN 280   0
EN 300   -15748
EN 320   0
EN 340   0
EN 360   0
H2U-P:PHE-S2
EN 20   0

EN 40   0
EN 60   0
EN 80   -13841
EN 100   0
EN 120   0
EN 140   0
EN 160   0
EN 180   0
EN 200   0
EN 220   0
EN 240   0
EN 260   0
EN 280   0
EN 300   0
EN 320   0
EN 340   0
EN 360   0
I-RIB:ALA-CA
EN 20   0
EN 40   0
EN 60   0
EN 80   0
EN 100   0
EN 120   0
EN 140   0
EN 160   -17163
EN 180   0
EN 200   0
EN 220   0
EN 240   0
EN 260   0
EN 280   0
EN 300   0
EN 320   0
EN 340   0
EN 360   0
H2U-MY:LEU-CA
EN 20   0
EN 40   0
EN 60   0
EN 80   0
EN 100   0
EN 120   0
EN 140   0
EN 160   -13027
EN 180   0
EN 200   0
EN 220   0
EN 240   0
EN 260   0
EN 280   0
EN 300   0
EN 320   -12156
EN 340   0
EN 360   0
U34-P:ASN-S1
EN 20   0
EN 40   0

EN 60   -18418
EN 80   0
EN 100   -17510
EN 120   0
EN 140   0
EN 160   0
EN 180   0
EN 200   0
EN 220   0
EN 240   0
EN 260   -17686
EN 280   -17510
EN 300   0
EN 320   0
EN 340   0
EN 360   0
A-RIB:MET-CA
EN 20   0
EN 40   -3204
EN 60   -1562
EN 80   -4837
EN 100   -654
EN 120   -4362
EN 140   -3153
EN 160   -2131
EN 180   0
EN 200   0
EN 220   -4776
EN 240   -1583
EN 260   -3553
EN 280   -4089
EN 300   -3586
EN 320   -1260
EN 340   -4759
EN 360   0
FMU-MY:VAL-S1
EN 20   0
EN 40   0
EN 60   0
EN 80   0
EN 100   0
EN 120   0
EN 140   0
EN 160   0
EN 180   0
EN 200   0
EN 220   0
EN 240   -16906
EN 260   0
EN 280   0
EN 300   0
EN 320   0
EN 340   0
EN 360   -19652
U34-P:HIS-S1
EN 20   0
EN 40   0
EN 60   0

EN 80 0
EN 100 0
EN 120 0
EN 140 0
EN 160 -20202
EN 180 0
EN 200 0
EN 220 0
EN 240 0
EN 260 0
EN 280 0
EN 300 0
EN 320 0
EN 340 0
EN 360 0
C31-MY:ALA-CA
EN 20 0
EN 40 0
EN 60 0
EN 80 0
EN 100 0
EN 120 0
EN 140 0
EN 160 0
EN 180 0
EN 200 0
EN 220 0
EN 240 0
EN 260 0
EN 280 -15686
EN 300 0
EN 320 0
EN 340 0
EN 360 0
FMU-P:ILE-CA
EN 20 0
EN 40 0
EN 60 0
EN 80 0
EN 100 -16673
EN 120 0
EN 140 0
EN 160 0
EN 180 0
EN 200 0
EN 220 0
EN 240 0
EN 260 0
EN 280 0
EN 300 0
EN 320 0
EN 340 0
EN 360 0
FMU-RIB:VAL-S1
EN 20 0
EN 40 0
EN 60 0
EN 80 0

EN 100   0
EN 120   0
EN 140   0
EN 160   0
EN 180   0
EN 200   0
EN 220   0
EN 240   0
EN 260   0
EN 280   -15977
EN 300   0
EN 320   0
EN 340   0
EN 360   0
FHU-MY:TYR-CA
EN 20   0
EN 40   0
EN 60   0
EN 80   0
EN 100   -16276
EN 120   0
EN 140   -17057
EN 160   0
EN 180   0
EN 200   0
EN 220   0
EN 240   0
EN 260   0
EN 280   -16276
EN 300   0
EN 320   -16881
EN 340   0
EN 360   0
FHU-MY:LYS-S1
EN 20   0
EN 40   0
EN 60   0
EN 80   -14582
EN 100   0
EN 120   0
EN 140   0
EN 160   0
EN 180   0
EN 200   0
EN 220   0
EN 240   0
EN 260   -14564
EN 280   0
EN 300   0
EN 320   0
EN 340   0
EN 360   0
IU-RIB:HIS-CA
EN 20   0
EN 40   0
EN 60   0
EN 80   0
EN 100   0

EN 120 0
EN 140 0
EN 160 0
EN 180 0
EN 200 0
EN 220 0
EN 240 -16435
EN 260 0
EN 280 0
EN 300 0
EN 320 0
EN 340 0
EN 360 0
QUO-RIB:PHE-S2
EN 20 0
EN 40 0
EN 60 0
EN 80 0
EN 100 0
EN 120 0
EN 140 0
EN 160 0
EN 180 0
EN 200 0
EN 220 0
EN 240 0
EN 260 0
EN 280 0
EN 300 -19561
EN 320 0
EN 340 0
EN 360 0
GTP-M5:ASN-S1
EN 20 -23186
EN 40 0
EN 60 0
EN 80 0
EN 100 0
EN 120 0
EN 140 0
EN 160 0
EN 180 0
EN 200 0
EN 220 0
EN 240 0
EN 260 0
EN 280 0
EN 300 0
EN 320 0
EN 340 0
EN 360 0
U31-P:LEU-S1
EN 20 0
EN 40 0
EN 60 0
EN 80 0
EN 100 0
EN 120 0

EN 140 0
EN 160 0
EN 180 0
EN 200 0
EN 220 0
EN 240 0
EN 260 0
EN 280 -15538
EN 300 0
EN 320 0
EN 340 0
EN 360 0
FHU-RIB:ILE-S1
EN 20 0
EN 40 0
EN 60 0
EN 80 0
EN 100 0
EN 120 0
EN 140 0
EN 160 0
EN 180 0
EN 200 0
EN 220 0
EN 240 0
EN 260 0
EN 280 0
EN 300 -16883
EN 320 0
EN 340 0
EN 360 0
DA-M5:GLN-CA
EN 20 0
EN 40 0
EN 60 0
EN 80 0
EN 100 0
EN 120 0
EN 140 0
EN 160 0
EN 180 0
EN 200 0
EN 220 0
EN 240 0
EN 260 -17929
EN 280 0
EN 300 0
EN 320 0
EN 340 0
EN 360 0
C-P:ASP-S2
EN 20 0
EN 40 -5694
EN 60 -2666
EN 80 -4873
EN 100 -5193
EN 120 -4753
EN 140 -5261

EN 160   -6155
EN 180   -2116
EN 200   0
EN 220   -5549
EN 240   -4404
EN 260   -4205
EN 280   -3475
EN 300   -4474
EN 320   -2364
EN 340   -3141
EN 360   -3715
G-RIB:CYS-CA
EN 20   0
EN 40   0
EN 60   0
EN 80   0
EN 100   0
EN 120   -4112
EN 140   0
EN 160   0
EN 180   -5914
EN 200   0
EN 220   0
EN 240   0
EN 260   0
EN 280   -4844
EN 300   -2331
EN 320   0
EN 340   0
EN 360   0
FHU-RIB:LYS-S2
EN 20   -20111
EN 40   0
EN 60   0
EN 80   0
EN 100   -14435
EN 120   0
EN 140   0
EN 160   0
EN 180   0
EN 200   0
EN 220   -16839
EN 240   0
EN 260   0
EN 280   0
EN 300   0
EN 320   0
EN 340   0
EN 360   0
QUO-M5:ASP-CA
EN 20   0
EN 40   0
EN 60   0
EN 80   0
EN 100   0
EN 120   -16700
EN 140   0
EN 160   0

EN 180   0
EN 200   0
EN 220   0
EN 240   0
EN 260   0
EN 280   0
EN 300   0
EN 320   -17033
EN 340   0
EN 360   0
DA-M5:LYS-S1
EN 20   0
EN 40   0
EN 60   0
EN 80   0
EN 100   0
EN 120   0
EN 140   0
EN 160   0
EN 180   0
EN 200   0
EN 220   0
EN 240   0
EN 260   0
EN 280   -16105
EN 300   0
EN 320   0
EN 340   0
EN 360   0
DA-M5:MET-S2
EN 20   0
EN 40   0
EN 60   0
EN 80   0
EN 100   0
EN 120   0
EN 140   0
EN 160   0
EN 180   0
EN 200   0
EN 220   -21074
EN 240   0
EN 260   0
EN 280   0
EN 300   0
EN 320   0
EN 340   0
EN 360   0
U31-MY:ALA-CA
EN 20   0
EN 40   0
EN 60   0
EN 80   0
EN 100   0
EN 120   0
EN 140   0
EN 160   0
EN 180   0

EN 200   0
EN 220   0
EN 240   0
EN 260   0
EN 280   -15686
EN 300   0
EN 320   0
EN 340   0
EN 360   0
FHU-P:SER-CA
EN 20   0
EN 40   0
EN 60   0
EN 80   0
EN 100   0
EN 120   -15246
EN 140   0
EN 160   -16450
EN 180   0
EN 200   0
EN 220   0
EN 240   0
EN 260   0
EN 280   0
EN 300   0
EN 320   0
EN 340   0
EN 360   0
FMU-MY:VAL-CA
EN 20   0
EN 40   0
EN 60   0
EN 80   0
EN 100   0
EN 120   0
EN 140   0
EN 160   0
EN 180   -19768
EN 200   0
EN 220   0
EN 240   0
EN 260   -16152
EN 280   0
EN 300   0
EN 320   0
EN 340   0
EN 360   0
U-Y:ILE-CA
EN 20   0
EN 40   0
EN 60   -2062
EN 80   -2353
EN 100   563
EN 120   -1427
EN 140   -217
EN 160   -913
EN 180   0
EN 200   0

EN 220   0
EN 240   -365
EN 260   386
EN 280   -2158
EN 300   -3081
EN 320   -1759
EN 340   -819
EN 360   0
C31-RIB:SER-S1
EN 20   0
EN 40   0
EN 60   0
EN 80   0
EN 100   0
EN 120   0
EN 140   0
EN 160   0
EN 180   0
EN 200   0
EN 220   0
EN 240   0
EN 260   0
EN 280   0
EN 300   0
EN 320   -17296
EN 340   0
EN 360   0
QUO-M6:ASP-S2
EN 20   0
EN 40   0
EN 60   0
EN 80   0
EN 100   0
EN 120   -16748
EN 140   0
EN 160   0
EN 180   0
EN 200   0
EN 220   0
EN 240   0
EN 260   0
EN 280   0
EN 300   -16684
EN 320   0
EN 340   0
EN 360   0
IU-MY:VAL-S1
EN 20   0
EN 40   0
EN 60   0
EN 80   0
EN 100   -11753
EN 120   0
EN 140   0
EN 160   0
EN 180   0
EN 200   0
EN 220   0

EN 240   0
EN 260   0
EN 280   0
EN 300   0
EN 320   0
EN 340   0
EN 360   0
U34-MY:PHE-CA
EN 20   0
EN 40   0
EN 60   0
EN 80   0
EN 100   0
EN 120   -17904
EN 140   0
EN 160   0
EN 180   0
EN 200   0
EN 220   0
EN 240   0
EN 260   0
EN 280   0
EN 300   0
EN 320   0
EN 340   0
EN 360   0
U31-P:ASP-CA
EN 20   0
EN 40   0
EN 60   0
EN 80   0
EN 100   0
EN 120   0
EN 140   0
EN 160   0
EN 180   0
EN 200   0
EN 220   0
EN 240   0
EN 260   -16603
EN 280   0
EN 300   -16637
EN 320   0
EN 340   0
EN 360   0
H2U-MY:GLU-S1
EN 20   0
EN 40   0
EN 60   0
EN 80   0
EN 100   0
EN 120   0
EN 140   -12536
EN 160   0
EN 180   0
EN 200   0
EN 220   0
EN 240   0

EN 260   0
EN 280   0
EN 300   0
EN 320   0
EN 340   0
EN 360   0
U34-P:ARG-S2
EN 20   0
EN 40   0
EN 60   0
EN 80   0
EN 100   0
EN 120   0
EN 140   0
EN 160   0
EN 180   0
EN 200   0
EN 220   0
EN 240   0
EN 260   0
EN 280   0
EN 300   -16401
EN 320   0
EN 340   0
EN 360   0
FHU-MY:SER-CA
EN 20   0
EN 40   0
EN 60   0
EN 80   0
EN 100   0
EN 120   -15246
EN 140   0
EN 160   0
EN 180   0
EN 200   0
EN 220   0
EN 240   -15902
EN 260   0
EN 280   0
EN 300   0
EN 320   0
EN 340   0
EN 360   0
IU-MY:LEU-S2
EN 20   0
EN 40   0
EN 60   -12226
EN 80   0
EN 100   0
EN 120   0
EN 140   0
EN 160   0
EN 180   0
EN 200   0
EN 220   0
EN 240   0
EN 260   0

EN 280   0
EN 300   0
EN 320   0
EN 340   0
EN 360   0
C31-P:THR-S1
EN 20   0
EN 40   0
EN 60   -17838
EN 80   0
EN 100   -16930
EN 120   0
EN 140   0
EN 160   0
EN 180   0
EN 200   0
EN 220   0
EN 240   0
EN 260   0
EN 280   0
EN 300   0
EN 320   0
EN 340   0
EN 360   0
H2U-MY:GLU-CA
EN 20   0
EN 40   0
EN 60   0
EN 80   0
EN 100   0
EN 120   0
EN 140   0
EN 160   -13225
EN 180   0
EN 200   0
EN 220   0
EN 240   0
EN 260   0
EN 280   0
EN 300   0
EN 320   0
EN 340   0
EN 360   0
C31-P:PHE-CA
EN 20   0
EN 40   0
EN 60   0
EN 80   0
EN 100   0
EN 120   0
EN 140   0
EN 160   0
EN 180   0
EN 200   0
EN 220   0
EN 240   -18559
EN 260   0
EN 280   0

EN 300 0
EN 320 0
EN 340 0
EN 360 0
A-P:GLN-S2
EN 20 0
EN 40 -6319
EN 60 -4125
EN 80 -6515
EN 100 -3770
EN 120 -4043
EN 140 -5385
EN 160 -5246
EN 180 -6297
EN 200 0
EN 220 -3904
EN 240 -3433
EN 260 -5403
EN 280 -5487
EN 300 -1709
EN 320 -5540
EN 340 -4600
EN 360 -5174
U31-P:ASN-S1
EN 20 0
EN 40 0
EN 60 0
EN 80 0
EN 100 -17510
EN 120 0
EN 140 0
EN 160 0
EN 180 0
EN 200 0
EN 220 0
EN 240 0
EN 260 0
EN 280 0
EN 300 0
EN 320 0
EN 340 0
EN 360 0
IU-RIB:HIS-S1
EN 20 0
EN 40 0
EN 60 0
EN 80 0
EN 100 0
EN 120 0
EN 140 0
EN 160 0
EN 180 0
EN 200 0
EN 220 0
EN 240 -16435
EN 260 0
EN 280 0
EN 300 0

EN 320   0
EN 340   0
EN 360   0
FMU-MY:MET-CA
EN 20   0
EN 40   0
EN 60   0
EN 80   0
EN 100   0
EN 120   0
EN 140   0
EN 160   0
EN 180   0
EN 200   0
EN 220   0
EN 240   0
EN 260   0
EN 280   -18659
EN 300   0
EN 320   0
EN 340   0
EN 360   0
U31-MY:GLU-S2
EN 20   0
EN 40   0
EN 60   0
EN 80   0
EN 100   0
EN 120   0
EN 140   0
EN 160   -17252
EN 180   0
EN 200   0
EN 220   0
EN 240   0
EN 260   -15952
EN 280   0
EN 300   0
EN 320   0
EN 340   0
EN 360   0
FMU-RIB:MET-S2
EN 20   0
EN 40   0
EN 60   0
EN 80   0
EN 100   0
EN 120   0
EN 140   0
EN 160   0
EN 180   0
EN 200   0
EN 220   0
EN 240   0
EN 260   0
EN 280   0
EN 300   0
EN 320   -19275

EN 340 0
EN 360 0
FHU-MY:ASP-S1
EN 20 0
EN 40 0
EN 60 0
EN 80 0
EN 100 -14712
EN 120 0
EN 140 -15493
EN 160 0
EN 180 0
EN 200 0
EN 220 0
EN 240 0
EN 260 0
EN 280 -14712
EN 300 0
EN 320 -15317
EN 340 0
EN 360 0
G-P:VAL-S1
EN 20 0
EN 40 10
EN 60 -66
EN 80 2365
EN 100 -2262
EN 120 -3157
EN 140 -4577
EN 160 -2351
EN 180 -1233
EN 200 0
EN 220 -2566
EN 240 -1091
EN 260 -1051
EN 280 -1428
EN 300 -372
EN 320 -2485
EN 340 -2811
EN 360 -3836
GTP-M5:ALA-CA
EN 20 0
EN 40 0
EN 60 0
EN 80 0
EN 100 0
EN 120 0
EN 140 0
EN 160 0
EN 180 0
EN 200 0
EN 220 0
EN 240 0
EN 260 0
EN 280 -15686
EN 300 0
EN 320 0
EN 340 0

EN 360   0
I-RIB:TRP-S1
EN 20   0
EN 40   0
EN 60   0
EN 80   0
EN 100   0
EN 120   0
EN 140   -20972
EN 160   0
EN 180   0
EN 200   0
EN 220   0
EN 240   0
EN 260   0
EN 280   0
EN 300   0
EN 320   0
EN 340   0
EN 360   0
5BU-P:ARG-CA
EN 20   0
EN 40   0
EN 60   0
EN 80   0
EN 100   0
EN 120   -14735
EN 140   -15243
EN 160   0
EN 180   0
EN 200   0
EN 220   0
EN 240   0
EN 260   0
EN 280   0
EN 300   0
EN 320   0
EN 340   0
EN 360   0
U34-MY:ASP-CA
EN 20   0
EN 40   0
EN 60   0
EN 80   0
EN 100   -16427
EN 120   0
EN 140   0
EN 160   0
EN 180   0
EN 200   0
EN 220   0
EN 240   0
EN 260   0
EN 280   0
EN 300   0
EN 320   0
EN 340   0
EN 360   0

U34-MY:SER-S1
EN 20 0
EN 40 0
EN 60 0
EN 80 0
EN 100 0
EN 120 0
EN 140 0
EN 160 0
EN 180 0
EN 200 0
EN 220 0
EN 240 0
EN 260 0
EN 280 0
EN 300 -16900
EN 320 0
EN 340 0
EN 360 -20365
G-P:LEU-CA
EN 20 0
EN 40 0
EN 60 -1344
EN 80 1086
EN 100 -3356
EN 120 -2979
EN 140 -1770
EN 160 -1912
EN 180 -2511
EN 200 0
EN 220 0
EN 240 -3082
EN 260 -612
EN 280 -2153
EN 300 -2363
EN 320 -2759
EN 340 -1819
EN 360 -675
4SU-P:GLU-S2
EN 20 0
EN 40 0
EN 60 0
EN 80 0
EN 100 0
EN 120 0
EN 140 0
EN 160 0
EN 180 0
EN 200 0
EN 220 0
EN 240 0
EN 260 0
EN 280 0
EN 300 0
EN 320 0
EN 340 -17158
EN 360 0
FHU-RIB:ARG-CA

EN 20 0
EN 40 0
EN 60 0
EN 80 0
EN 100 0
EN 120 0
EN 140 0
EN 160 0
EN 180 0
EN 200 0
EN 220 0
EN 240 0
EN 260 0
EN 280 0
EN 300 0
EN 320 -15067
EN 340 -15844
EN 360 0
FMU-MY:PHE-S1
EN 20 0
EN 40 0
EN 60 0
EN 80 0
EN 100 0
EN 120 0
EN 140 0
EN 160 0
EN 180 0
EN 200 0
EN 220 0
EN 240 0
EN 260 -17807
EN 280 0
EN 300 0
EN 320 0
EN 340 0
EN 360 0
C31-P:PHE-S1
EN 20 0
EN 40 0
EN 60 0
EN 80 0
EN 100 0
EN 120 0
EN 140 0
EN 160 0
EN 180 0
EN 200 0
EN 220 0
EN 240 -18559
EN 260 0
EN 280 0
EN 300 0
EN 320 0
EN 340 0
EN 360 0
U34-RIB:SER-S1
EN 20 0

```
EN 40   0
EN 60   0
EN 80   -16885
EN 100   0
EN 120   0
EN 140   0
EN 160   0
EN 180   0
EN 200   0
EN 220   0
EN 240   0
EN 260   0
EN 280   -16691
EN 300   0
EN 320   0
EN 340   0
EN 360   0
M2G-P:GLY-CA
EN 20   0
EN 40   0
EN 60   0
EN 80   0
EN 100   0
EN 120   0
EN 140   0
EN 160   -17551
EN 180   0
EN 200   0
EN 220   0
EN 240   0
EN 260   0
EN 280   0
EN 300   0
EN 320   0
EN 340   0
EN 360   0
I-RIB:GLY-CA
EN 20   0
EN 40   0
EN 60   0
EN 80   0
EN 100   0
EN 120   0
EN 140   0
EN 160   -17551
EN 180   0
EN 200   0
EN 220   0
EN 240   0
EN 260   0
EN 280   0
EN 300   0
EN 320   0
EN 340   0
EN 360   0
IU-RIB:LYS-S2
EN 20   0
EN 40   0
```

EN 60   0
EN 80   0
EN 100   0
EN 120   0
EN 140   0
EN 160   -14410
EN 180   0
EN 200   0
EN 220   -15338
EN 240   0
EN 260   0
EN 280   0
EN 300   0
EN 320   0
EN 340   0
EN 360   0
FHU-MY:GLY-CA
EN 20   0
EN 40   0
EN 60   -15265
EN 80   0
EN 100   0
EN 120   0
EN 140   0
EN 160   0
EN 180   0
EN 200   0
EN 220   0
EN 240   -15285
EN 260   0
EN 280   0
EN 300   0
EN 320   0
EN 340   0
EN 360   0
H2U-MY:ASN-S1
EN 20   0
EN 40   0
EN 60   0
EN 80   0
EN 100   0
EN 120   0
EN 140   0
EN 160   -14999
EN 180   0
EN 200   0
EN 220   0
EN 240   0
EN 260   0
EN 280   -15240
EN 300   0
EN 320   0
EN 340   0
EN 360   0
H2U-P:TRP-S1
EN 20   0
EN 40   0
EN 60   0

EN 80 0
EN 100 -16203
EN 120 0
EN 140 0
EN 160 0
EN 180 0
EN 200 0
EN 220 0
EN 240 0
EN 260 0
EN 280 0
EN 300 0
EN 320 0
EN 340 0
EN 360 0
DA-M5:TYR-S1
EN 20 0
EN 40 0
EN 60 0
EN 80 0
EN 100 -17993
EN 120 0
EN 140 0
EN 160 0
EN 180 0
EN 200 0
EN 220 0
EN 240 0
EN 260 0
EN 280 0
EN 300 -18203
EN 320 0
EN 340 0
EN 360 0
DA-M6:SER-CA
EN 20 0
EN 40 0
EN 60 0
EN 80 0
EN 100 0
EN 120 0
EN 140 0
EN 160 0
EN 180 0
EN 200 0
EN 220 0
EN 240 0
EN 260 0
EN 280 0
EN 300 -16900
EN 320 -17296
EN 340 0
EN 360 0
DA-M5:ASN-S2
EN 20 0
EN 40 0
EN 60 0
EN 80 0

EN 100   0
EN 120   0
EN 140   0
EN 160   0
EN 180   0
EN 200   0
EN 220   0
EN 240   -18459
EN 260   0
EN 280   0
EN 300   0
EN 320   0
EN 340   0
EN 360   0
IU-RIB:LYS-CA
EN 20   0
EN 40   0
EN 60   0
EN 80   0
EN 100   0
EN 120   -13153
EN 140   0
EN 160   0
EN 180   0
EN 200   0
EN 220   0
EN 240   -13808
EN 260   0
EN 280   0
EN 300   0
EN 320   0
EN 340   0
EN 360   0
FMU-RIB:HIS-S1
EN 20   0
EN 40   0
EN 60   0
EN 80   0
EN 100   0
EN 120   0
EN 140   0
EN 160   -20202
EN 180   0
EN 200   0
EN 220   0
EN 240   0
EN 260   0
EN 280   0
EN 300   0
EN 320   0
EN 340   0
EN 360   0
H2U-P:LEU-S1
EN 20   0
EN 40   0
EN 60   0
EN 80   0
EN 100   -11551

EN 120 0
EN 140 0
EN 160 0
EN 180 0
EN 200 0
EN 220 0
EN 240 0
EN 260 0
EN 280 0
EN 300 0
EN 320 0
EN 340 0
EN 360 0
H2U-MY:GLN-CA
EN 20 0
EN 40 0
EN 60 0
EN 80 0
EN 100 0
EN 120 0
EN 140 0
EN 160 0
EN 180 0
EN 200 0
EN 220 0
EN 240 0
EN 260 0
EN 280 0
EN 300 0
EN 320 0
EN 340 0
EN 360 -17440
C31-MY:SER-S1
EN 20 0
EN 40 0
EN 60 0
EN 80 0
EN 100 0
EN 120 0
EN 140 0
EN 160 0
EN 180 0
EN 200 0
EN 220 0
EN 240 0
EN 260 -16867
EN 280 0
EN 300 0
EN 320 0
EN 340 0
EN 360 0
C31-RIB:PHE-S2
EN 20 0
EN 40 0
EN 60 0
EN 80 0
EN 100 -17634
EN 120 -17907

EN 140   0
EN 160   0
EN 180   0
EN 200   0
EN 220   0
EN 240   0
EN 260   0
EN 280   0
EN 300   0
EN 320   0
EN 340   0
EN 360   0
H2U-P:THR-S1
EN 20   0
EN 40   0
EN 60   0
EN 80   0
EN 100   0
EN 120   0
EN 140   0
EN 160   0
EN 180   0
EN 200   0
EN 220   0
EN 240   0
EN 260   0
EN 280   -12942
EN 300   -13152
EN 320   0
EN 340   0
EN 360   0
GTP-M5:ASP-S2
EN 20   0
EN 40   0
EN 60   0
EN 80   0
EN 100   0
EN 120   0
EN 140   0
EN 160   0
EN 180   0
EN 200   0
EN 220   0
EN 240   0
EN 260   0
EN 280   0
EN 300   -16684
EN 320   0
EN 340   0
EN 360   0
C31-P:PHE-S2
EN 20   0
EN 40   0
EN 60   0
EN 80   0
EN 100   0
EN 120   0
EN 140   0

EN 160 0
EN 180 0
EN 200 0
EN 220 -20038
EN 240 -18562
EN 260 0
EN 280 0
EN 300 0
EN 320 0
EN 340 0
EN 360 0
C31-MY:PHE-CA
EN 20 0
EN 40 0
EN 60 0
EN 80 0
EN 100 -17630
EN 120 0
EN 140 0
EN 160 0
EN 180 0
EN 200 0
EN 220 0
EN 240 0
EN 260 0
EN 280 0
EN 300 0
EN 320 0
EN 340 0
EN 360 0
FMU-RIB:VAL-CA
EN 20 0
EN 40 0
EN 60 0
EN 80 0
EN 100 0
EN 120 0
EN 140 0
EN 160 0
EN 180 0
EN 200 0
EN 220 0
EN 240 0
EN 260 0
EN 280 -15975
EN 300 0
EN 320 0
EN 340 0
EN 360 0
U31-MY:PHE-S2
EN 20 0
EN 40 0
EN 60 0
EN 80 0
EN 100 0
EN 120 0
EN 140 0
EN 160 0

EN 180 0
EN 200 0
EN 220 0
EN 240 0
EN 260 0
EN 280 -17634
EN 300 0
EN 320 0
EN 340 0
EN 360 0
C31-RIB:THR-S1
EN 20 0
EN 40 0
EN 60 0
EN 80 0
EN 100 0
EN 120 0
EN 140 0
EN 160 0
EN 180 0
EN 200 0
EN 220 0
EN 240 0
EN 260 0
EN 280 -16930
EN 300 0
EN 320 0
EN 340 0
EN 360 0
U34-MY:ASN-S2
EN 20 0
EN 40 0
EN 60 0
EN 80 0
EN 100 0
EN 120 0
EN 140 0
EN 160 0
EN 180 0
EN 200 0
EN 220 -19935
EN 240 0
EN 260 -17707
EN 280 0
EN 300 0
EN 320 0
EN 340 0
EN 360 0
GTP-M6:THR-S1
EN 20 0
EN 40 0
EN 60 0
EN 80 -17125
EN 100 0
EN 120 0
EN 140 0
EN 160 0
EN 180 0

EN 200 0
EN 220 0
EN 240 0
EN 260 0
EN 280 0
EN 300 0
EN 320 0
EN 340 0
EN 360 0
FHU-RIB:ILE-CA
EN 20 0
EN 40 0
EN 60 0
EN 80 0
EN 100 0
EN 120 0
EN 140 0
EN 160 0
EN 180 0
EN 200 0
EN 220 0
EN 240 0
EN 260 0
EN 280 0
EN 300 -15166
EN 320 -15561
EN 340 0
EN 360 0
5BU-P:ARG-S1
EN 20 0
EN 40 0
EN 60 0
EN 80 0
EN 100 0
EN 120 0
EN 140 -15243
EN 160 0
EN 180 0
EN 200 0
EN 220 0
EN 240 0
EN 260 0
EN 280 0
EN 300 0
EN 320 0
EN 340 0
EN 360 0
FMU-RIB:HIS-S2
EN 20 0
EN 40 0
EN 60 0
EN 80 0
EN 100 0
EN 120 0
EN 140 -19524
EN 160 0
EN 180 0
EN 200 0

EN 220 0
EN 240 0
EN 260 0
EN 280 0
EN 300 0
EN 320 0
EN 340 0
EN 360 0
A-RIB:CYS-CA
EN 20 0
EN 40 0
EN 60 0
EN 80 -5598
EN 100 -2682
EN 120 -5677
EN 140 -3463
EN 160 0
EN 180 0
EN 200 0
EN 220 0
EN 240 -3610
EN 260 0
EN 280 -2682
EN 300 0
EN 320 -5005
EN 340 0
EN 360 0
H2U-RIB:ARG-S2
EN 20 0
EN 40 0
EN 60 0
EN 80 0
EN 100 0
EN 120 0
EN 140 0
EN 160 0
EN 180 0
EN 200 0
EN 220 0
EN 240 0
EN 260 -12380
EN 280 0
EN 300 0
EN 320 -12809
EN 340 0
EN 360 0
C31-RIB:LEU-S2
EN 20 0
EN 40 0
EN 60 0
EN 80 0
EN 100 0
EN 120 0
EN 140 0
EN 160 0
EN 180 0
EN 200 0
EN 220 0

EN 240   0
EN 260   0
EN 280   0
EN 300   -15751
EN 320   0
EN 340   0
EN 360   0
I-RIB:TRP-S2
EN 20   0
EN 40   0
EN 60   0
EN 80   0
EN 100   0
EN 120   -20464
EN 140   0
EN 160   0
EN 180   0
EN 200   0
EN 220   0
EN 240   0
EN 260   0
EN 280   0
EN 300   0
EN 320   0
EN 340   0
EN 360   0
U-Y:GLN-CA
EN 20   0
EN 40   -3065
EN 60   -5412
EN 80   -710
EN 100   0
EN 120   -789
EN 140   -3015
EN 160   -4423
EN 180   0
EN 200   -8165
EN 220   0
EN 240   -3162
EN 260   -2410
EN 280   -2233
EN 300   -2443
EN 320   -4556
EN 340   0
EN 360   0
G-RIB:CYS-S1
EN 20   0
EN 40   0
EN 60   0
EN 80   0
EN 100   0
EN 120   -2395
EN 140   -2903
EN 160   0
EN 180   -5914
EN 200   0
EN 220   0
EN 240   0

EN 260   -2298
EN 280   0
EN 300   -2331
EN 320   -2727
EN 340   0
EN 360   0
U31-MY:ALA-S1
EN 20   0
EN 40   0
EN 60   0
EN 80   0
EN 100   0
EN 120   -15959
EN 140   0
EN 160   0
EN 180   0
EN 200   0
EN 220   0
EN 240   0
EN 260   0
EN 280   -15686
EN 300   0
EN 320   0
EN 340   0
EN 360   0
IU-P:LEU-CA
EN 20   0
EN 40   0
EN 60   0
EN 80   0
EN 100   0
EN 120   0
EN 140   -13597
EN 160   0
EN 180   0
EN 200   0
EN 220   0
EN 240   0
EN 260   0
EN 280   0
EN 300   -13026
EN 320   0
EN 340   0
EN 360   0
C31-MY:LEU-CA
EN 20   0
EN 40   0
EN 60   0
EN 80   0
EN 100   0
EN 120   0
EN 140   0
EN 160   0
EN 180   0
EN 200   0
EN 220   0
EN 240   0
EN 260   -15715

EN 280 0
EN 300 0
EN 320 0
EN 340 0
EN 360 0
H2U-MY:PRO-S1
EN 20 0
EN 40 0
EN 60 0
EN 80 0
EN 100 -14859
EN 120 0
EN 140 0
EN 160 0
EN 180 0
EN 200 0
EN 220 0
EN 240 0
EN 260 0
EN 280 0
EN 300 0
EN 320 0
EN 340 0
EN 360 0
FMU-MY:GLU-S2
EN 20 0
EN 40 0
EN 60 0
EN 80 0
EN 100 0
EN 120 0
EN 140 0
EN 160 0
EN 180 0
EN 200 0
EN 220 0
EN 240 0
EN 260 0
EN 280 0
EN 300 0
EN 320 -16381
EN 340 0
EN 360 0
U31-RIB:MET-CA
EN 20 0
EN 40 0
EN 60 -19567
EN 80 0
EN 100 0
EN 120 0
EN 140 0
EN 160 0
EN 180 0
EN 200 0
EN 220 0
EN 240 0
EN 260 0
EN 280 0

EN 300 0
EN 320 0
EN 340 0
EN 360 0
C31-P:ASN-S2
EN 20 0
EN 40 0
EN 60 0
EN 80 0
EN 100 0
EN 120 -17804
EN 140 0
EN 160 0
EN 180 0
EN 200 0
EN 220 0
EN 240 0
EN 260 0
EN 280 0
EN 300 0
EN 320 0
EN 340 0
EN 360 0
U-Y:PHE-S1
EN 20 0
EN 40 -5665
EN 60 -3019
EN 80 -588
EN 100 0
EN 120 -2384
EN 140 0
EN 160 0
EN 180 0
EN 200 -6324
EN 220 -5519
EN 240 -1322
EN 260 -3292
EN 280 -4833
EN 300 -4420
EN 320 -999
EN 340 0
EN 360 0
QUO-M6:ASP-S1
EN 20 0
EN 40 0
EN 60 0
EN 80 0
EN 100 0
EN 120 -16703
EN 140 0
EN 160 0
EN 180 0
EN 200 0
EN 220 0
EN 240 0
EN 260 0
EN 280 0
EN 300 0

EN 320 0
EN 340 0
EN 360 0
C31-P:GLN-S1
EN 20 0
EN 40 0
EN 60 0
EN 80 0
EN 100 0
EN 120 0
EN 140 0
EN 160 -19230
EN 180 0
EN 200 0
EN 220 0
EN 240 0
EN 260 0
EN 280 0
EN 300 0
EN 320 0
EN 340 0
EN 360 0
DA-M6:TYR-S2
EN 20 0
EN 40 0
EN 60 0
EN 80 0
EN 100 0
EN 120 -18270
EN 140 0
EN 160 0
EN 180 0
EN 200 0
EN 220 0
EN 240 0
EN 260 0
EN 280 0
EN 300 0
EN 320 0
EN 340 0
EN 360 0
U34-P:GLU-S2
EN 20 0
EN 40 0
EN 60 0
EN 80 0
EN 100 0
EN 120 0
EN 140 0
EN 160 0
EN 180 0
EN 200 0
EN 220 0
EN 240 0
EN 260 0
EN 280 0
EN 300 0
EN 320 0

EN 340   -17158
EN 360   0
FHU-P:ARG-S1
EN 20   0
EN 40   0
EN 60   0
EN 80   -14656
EN 100   0
EN 120   0
EN 140   0
EN 160   0
EN 180   0
EN 200   0
EN 220   0
EN 240   0
EN 260   0
EN 280   -14461
EN 300   0
EN 320   0
EN 340   0
EN 360   0
FHU-P:ASP-CA
EN 20   0
EN 40   0
EN 60   0
EN 80   0
EN 100   0
EN 120   0
EN 140   -15491
EN 160   -17904
EN 180   0
EN 200   0
EN 220   0
EN 240   0
EN 260   0
EN 280   0
EN 300   0
EN 320   0
EN 340   0
EN 360   0
C31-P:ALA-CA
EN 20   0
EN 40   0
EN 60   0
EN 80   0
EN 100   0
EN 120   0
EN 140   0
EN 160   0
EN 180   0
EN 200   0
EN 220   0
EN 240   0
EN 260   0
EN 280   0
EN 300   -15896
EN 320   0
EN 340   0

EN 360   0
FMU-RIB:PHE-S2
EN 20   0
EN 40   0
EN 60   0
EN 80   0
EN 100   0
EN 120   0
EN 140   0
EN 160   0
EN 180   0
EN 200   0
EN 220   0
EN 240   0
EN 260   -17810
EN 280   0
EN 300   0
EN 320   0
EN 340   -19017
EN 360   0
IU-MY:THR-CA
EN 20   0
EN 40   0
EN 60   0
EN 80   0
EN 100   0
EN 120   -13984
EN 140   -13488
EN 160   0
EN 180   0
EN 200   0
EN 220   0
EN 240   0
EN 260   0
EN 280   0
EN 300   0
EN 320   0
EN 340   0
EN 360   0
U31-P:SER-CA
EN 20   0
EN 40   0
EN 60   0
EN 80   0
EN 100   0
EN 120   0
EN 140   0
EN 160   0
EN 180   0
EN 200   0
EN 220   0
EN 240   0
EN 260   0
EN 280   0
EN 300   -16900
EN 320   0
EN 340   0
EN 360   0

FMU-MY:GLN-S2
EN 20 0
EN 40 0
EN 60 0
EN 80 0
EN 100 0
EN 120 0
EN 140 0
EN 160 0
EN 180 0
EN 200 0
EN 220 0
EN 240 0
EN 260 0
EN 280 0
EN 300 -17994
EN 320 0
EN 340 0
EN 360 0
DA-M5:SER-CA
EN 20 0
EN 40 0
EN 60 0
EN 80 0
EN 100 0
EN 120 0
EN 140 0
EN 160 0
EN 180 0
EN 200 0
EN 220 0
EN 240 0
EN 260 0
EN 280 0
EN 300 -16900
EN 320 0
EN 340 -18073
EN 360 0
FMU-MY:GLU-S1
EN 20 0
EN 40 0
EN 60 0
EN 80 0
EN 100 0
EN 120 0
EN 140 0
EN 160 0
EN 180 0
EN 200 0
EN 220 0
EN 240 0
EN 260 0
EN 280 0
EN 300 -15953
EN 320 0
EN 340 0
EN 360 0
5BU-P:SER-S1

EN 20 0
EN 40 0
EN 60 0
EN 80 0
EN 100 0
EN 120 0
EN 140 0
EN 160 0
EN 180 0
EN 200 0
EN 220 0
EN 240 0
EN 260 0
EN 280 -14973
EN 300 0
EN 320 0
EN 340 0
EN 360 0
U31-RIB:TYR-S2
EN 20 0
EN 40 0
EN 60 0
EN 80 0
EN 100 0
EN 120 0
EN 140 0
EN 160 -19474
EN 180 0
EN 200 0
EN 220 0
EN 240 0
EN 260 0
EN 280 -17997
EN 300 0
EN 320 0
EN 340 0
EN 360 0
FMU-MY:ASN-S1
EN 20 0
EN 40 0
EN 60 0
EN 80 0
EN 100 -17510
EN 120 0
EN 140 0
EN 160 0
EN 180 0
EN 200 0
EN 220 0
EN 240 0
EN 260 0
EN 280 0
EN 300 0
EN 320 0
EN 340 0
EN 360 0
U34-P:PRO-S1
EN 20 0

EN 40 0
EN 60 0
EN 80 0
EN 100 0
EN 120 0
EN 140 0
EN 160 0
EN 180 0
EN 200 0
EN 220 0
EN 240 0
EN 260 0
EN 280 -17129
EN 300 0
EN 320 0
EN 340 0
EN 360 0
QUO-M5:LEU-S2
EN 20 0
EN 40 0
EN 60 0
EN 80 0
EN 100 0
EN 120 0
EN 140 0
EN 160 0
EN 180 0
EN 200 0
EN 220 0
EN 240 -16470
EN 260 0
EN 280 -15541
EN 300 0
EN 320 0
EN 340 0
EN 360 0
I-P:TRP-S2
EN 20 0
EN 40 0
EN 60 0
EN 80 0
EN 100 0
EN 120 0
EN 140 0
EN 160 0
EN 180 0
EN 200 0
EN 220 0
EN 240 -21119
EN 260 0
EN 280 0
EN 300 0
EN 320 0
EN 340 0
EN 360 0
C31-RIB:TYR-S1
EN 20 0
EN 40 0

EN 60   0
EN 80   0
EN 100   0
EN 120   0
EN 140   0
EN 160   0
EN 180   0
EN 200   0
EN 220   0
EN 240   -18921
EN 260   0
EN 280   0
EN 300   0
EN 320   0
EN 340   0
EN 360   0
5BU-RIB:SER-S1
EN 20   0
EN 40   0
EN 60   -15881
EN 80   0
EN 100   0
EN 120   0
EN 140   0
EN 160   0
EN 180   0
EN 200   0
EN 220   0
EN 240   0
EN 260   0
EN 280   0
EN 300   0
EN 320   0
EN 340   0
EN 360   0
IU-P:LYS-S2
EN 20   0
EN 40   0
EN 60   0
EN 80   0
EN 100   0
EN 120   0
EN 140   -14212
EN 160   0
EN 180   0
EN 200   0
EN 220   0
EN 240   0
EN 260   0
EN 280   0
EN 300   -13640
EN 320   0
EN 340   0
EN 360   0
FMU-MY:GLN-S1
EN 20   0
EN 40   0
EN 60   0

EN 80 0
EN 100 0
EN 120 0
EN 140 0
EN 160 0
EN 180 0
EN 200 0
EN 220 0
EN 240 0
EN 260 0
EN 280 0
EN 300 -17963
EN 320 0
EN 340 0
EN 360 0
IU-P:SER-S1
EN 20 0
EN 40 0
EN 60 0
EN 80 0
EN 100 -13969
EN 120 0
EN 140 0
EN 160 0
EN 180 0
EN 200 0
EN 220 0
EN 240 0
EN 260 0
EN 280 -13969
EN 300 0
EN 320 0
EN 340 0
EN 360 0
FHU-MY:VAL-S1
EN 20 0
EN 40 0
EN 60 0
EN 80 0
EN 100 -14260
EN 120 0
EN 140 0
EN 160 0
EN 180 -18052
EN 200 0
EN 220 0
EN 240 0
EN 260 0
EN 280 0
EN 300 0
EN 320 0
EN 340 -15643
EN 360 0
H2U-P:TRP-S2
EN 20 0
EN 40 0
EN 60 0
EN 80 0

EN 100 -16203
EN 120 0
EN 140 0
EN 160 0
EN 180 0
EN 200 0
EN 220 0
EN 240 0
EN 260 0
EN 280 0
EN 300 0
EN 320 0
EN 340 0
EN 360 0
FMU-MY:SER-S1
EN 20 0
EN 40 0
EN 60 0
EN 80 0
EN 100 0
EN 120 0
EN 140 -17472
EN 160 0
EN 180 0
EN 200 0
EN 220 0
EN 240 0
EN 260 0
EN 280 0
EN 300 0
EN 320 0
EN 340 0
EN 360 0
FHU-P:CYS-S1
EN 20 0
EN 40 0
EN 60 0
EN 80 0
EN 100 -18968
EN 120 0
EN 140 0
EN 160 0
EN 180 0
EN 200 0
EN 220 0
EN 240 0
EN 260 0
EN 280 0
EN 300 0
EN 320 0
EN 340 0
EN 360 0
C31-MY:PHE-S1
EN 20 0
EN 40 0
EN 60 0
EN 80 0
EN 100 -17630

EN 120   0
EN 140   0
EN 160   0
EN 180   0
EN 200   0
EN 220   -20034
EN 240   0
EN 260   0
EN 280   0
EN 300   0
EN 320   0
EN 340   0
EN 360   0
C31-MY:ALA-S1
EN 20   0
EN 40   0
EN 60   0
EN 80   0
EN 100   0
EN 120   0
EN 140   0
EN 160   0
EN 180   0
EN 200   0
EN 220   0
EN 240   0
EN 260   0
EN 280   -15686
EN 300   0
EN 320   0
EN 340   0
EN 360   0
5BU-P:ILE-S1
EN 20   0
EN 40   0
EN 60   0
EN 80   0
EN 100   0
EN 120   -15229
EN 140   0
EN 160   0
EN 180   0
EN 200   0
EN 220   0
EN 240   0
EN 260   0
EN 280   0
EN 300   0
EN 320   0
EN 340   -16339
EN 360   0
FMU-RIB:GLN-S1
EN 20   0
EN 40   0
EN 60   0
EN 80   0
EN 100   0
EN 120   0

EN 140   0
EN 160   0
EN 180   0
EN 200   0
EN 220   0
EN 240   0
EN 260   0
EN 280   -17753
EN 300   0
EN 320   0
EN 340   0
EN 360   0
C31-P:MET-CA
EN 20   0
EN 40   0
EN 60   0
EN 80   0
EN 100   0
EN 120   0
EN 140   0
EN 160   0
EN 180   0
EN 200   0
EN 220   0
EN 240   0
EN 260   0
EN 280   0
EN 300   0
EN 320   -19264
EN 340   0
EN 360   0
FHU-RIB:LEU-CA
EN 20   0
EN 40   0
EN 60   0
EN 80   0
EN 100   -13821
EN 120   0
EN 140   0
EN 160   0
EN 180   0
EN 200   0
EN 220   0
EN 240   0
EN 260   0
EN 280   -13821
EN 300   0
EN 320   0
EN 340   0
EN 360   0
C31-MY:PHE-S2
EN 20   0
EN 40   0
EN 60   0
EN 80   0
EN 100   0
EN 120   0
EN 140   0

EN 160   0
EN 180   0
EN 200   0
EN 220   0
EN 240   0
EN 260   -17810
EN 280   0
EN 300   0
EN 320   0
EN 340   0
EN 360   0
C31-RIB:GLU-CA
EN 20   0
EN 40   0
EN 60   0
EN 80   0
EN 100   0
EN 120   0
EN 140   0
EN 160   0
EN 180   0
EN 200   0
EN 220   0
EN 240   0
EN 260   -15913
EN 280   0
EN 300   0
EN 320   0
EN 340   0
EN 360   0
U31-RIB:GLU-CA
EN 20   0
EN 40   0
EN 60   0
EN 80   0
EN 100   0
EN 120   0
EN 140   0
EN 160   0
EN 180   0
EN 200   0
EN 220   0
EN 240   0
EN 260   0
EN 280   0
EN 300   0
EN 320   0
EN 340   -17119
EN 360   0
U34-P:GLU-S1
EN 20   0
EN 40   0
EN 60   0
EN 80   0
EN 100   0
EN 120   0
EN 140   0
EN 160   0

EN 180   0
EN 200   0
EN 220   0
EN 240   0
EN 260   0
EN 280   0
EN 300   0
EN 320   0
EN 340   0
EN 360   -19417
IU-RIB:HIS-S2
EN 20   0
EN 40   0
EN 60   0
EN 80   0
EN 100   0
EN 120   0
EN 140   0
EN 160   0
EN 180   0
EN 200   0
EN 220   -17927
EN 240   0
EN 260   0
EN 280   0
EN 300   0
EN 320   0
EN 340   0
EN 360   0
FHU-RIB:ASP-S1
EN 20   0
EN 40   0
EN 60   0
EN 80   0
EN 100   0
EN 120   0
EN 140   0
EN 160   -16188
EN 180   0
EN 200   0
EN 220   0
EN 240   0
EN 260   0
EN 280   0
EN 300   0
EN 320   -15317
EN 340   0
EN 360   0
U31-P:ALA-S1
EN 20   0
EN 40   0
EN 60   0
EN 80   0
EN 100   0
EN 120   0
EN 140   0
EN 160   0
EN 180   0

EN 200   0
EN 220   0
EN 240   0
EN 260   0
EN 280   0
EN 300   0
EN 320   0
EN 340   -17069
EN 360   0
U31-P:GLN-CA
EN 20   0
EN 40   0
EN 60   0
EN 80   0
EN 100   0
EN 120   0
EN 140   0
EN 160   0
EN 180   0
EN 200   0
EN 220   0
EN 240   0
EN 260   -17929
EN 280   0
EN 300   0
EN 320   0
EN 340   0
EN 360   0
5BU-P:PRO-S1
EN 20   0
EN 40   0
EN 60   0
EN 80   0
EN 100   0
EN 120   0
EN 140   0
EN 160   0
EN 180   0
EN 200   0
EN 220   0
EN 240   0
EN 260   0
EN 280   -15412
EN 300   0
EN 320   0
EN 340   0
EN 360   0
H2U-MY:THR-CA
EN 20   0
EN 40   0
EN 60   0
EN 80   0
EN 100   0
EN 120   -13216
EN 140   0
EN 160   0
EN 180   0
EN 200   0

EN 220   0
EN 240   0
EN 260   0
EN 280   0
EN 300   -13152
EN 320   0
EN 340   0
EN 360   0
QUO-M6:ASN-CA
EN 20   0
EN 40   0
EN 60   0
EN 80   -17704
EN 100   0
EN 120   0
EN 140   0
EN 160   0
EN 180   0
EN 200   0
EN 220   0
EN 240   0
EN 260   0
EN 280   0
EN 300   0
EN 320   0
EN 340   0
EN 360   0
IU-MY:ALA-S1
EN 20   0
EN 40   0
EN 60   0
EN 80   0
EN 100   -12467
EN 120   -11735
EN 140   0
EN 160   0
EN 180   0
EN 200   0
EN 220   0
EN 240   0
EN 260   0
EN 280   0
EN 300   0
EN 320   0
EN 340   0
EN 360   0
DA-M5:LEU-S1
EN 20   0
EN 40   0
EN 60   0
EN 80   0
EN 100   0
EN 120   -15811
EN 140   0
EN 160   0
EN 180   0
EN 200   0
EN 220   0

EN 240   0
EN 260   0
EN 280   0
EN 300   0
EN 320   0
EN 340   0
EN 360   0
H2U-MY:LEU-S2
EN 20   0
EN 40   0
EN 60   0
EN 80   0
EN 100   0
EN 120   0
EN 140   0
EN 160   0
EN 180   0
EN 200   0
EN 220   0
EN 240   0
EN 260   0
EN 280   0
EN 300   -11763
EN 320   0
EN 340   0
EN 360   0
DA-M5:TYR-CA
EN 20   0
EN 40   0
EN 60   0
EN 80   0
EN 100   0
EN 120   0
EN 140   0
EN 160   0
EN 180   0
EN 200   0
EN 220   0
EN 240   0
EN 260   0
EN 280   -17993
EN 300   -18203
EN 320   0
EN 340   0
EN 360   0
IU-RIB:ARG-S1
EN 20   0
EN 40   0
EN 60   0
EN 80   0
EN 100   0
EN 120   0
EN 140   0
EN 160   0
EN 180   0
EN 200   0
EN 220   0
EN 240   0

EN 260 0
EN 280 -12959
EN 300 0
EN 320 0
EN 340 0
EN 360 0
QUO-M6:GLU-S1
EN 20 0
EN 40 0
EN 60 0
EN 80 0
EN 100 0
EN 120 0
EN 140 0
EN 160 -17219
EN 180 0
EN 200 0
EN 220 0
EN 240 0
EN 260 0
EN 280 0
EN 300 0
EN 320 0
EN 340 0
EN 360 0
U31-MY:TYR-S2
EN 20 0
EN 40 0
EN 60 0
EN 80 0
EN 100 0
EN 120 0
EN 140 0
EN 160 0
EN 180 0
EN 200 0
EN 220 0
EN 240 0
EN 260 0
EN 280 -17997
EN 300 0
EN 320 0
EN 340 0
EN 360 0
FMU-MY:GLN-CA
EN 20 0
EN 40 0
EN 60 0
EN 80 0
EN 100 0
EN 120 0
EN 140 0
EN 160 0
EN 180 0
EN 200 0
EN 220 0
EN 240 0
EN 260 0

EN 280   -17753
EN 300   0
EN 320   0
EN 340   0
EN 360   0
H2U-P:PHE-S1
EN 20   0
EN 40   0
EN 60   0
EN 80   -13837
EN 100   0
EN 120   0
EN 140   0
EN 160   0
EN 180   0
EN 200   0
EN 220   0
EN 240   0
EN 260   0
EN 280   0
EN 300   0
EN 320   0
EN 340   0
EN 360   0
DA-M6:ASN-S2
EN 20   0
EN 40   0
EN 60   0
EN 80   0
EN 100   0
EN 120   0
EN 140   0
EN 160   0
EN 180   0
EN 200   0
EN 220   0
EN 240   0
EN 260   -17707
EN 280   0
EN 300   0
EN 320   0
EN 340   0
EN 360   0
FHU-MY:ILE-S1
EN 20   0
EN 40   0
EN 60   0
EN 80   0
EN 100   0
EN 120   0
EN 140   -15737
EN 160   0
EN 180   0
EN 200   0
EN 220   0
EN 240   0
EN 260   0
EN 280   0

EN 300 0
EN 320 -15561
EN 340 0
EN 360 0
H2U-RIB:PHE-S2
EN 20 0
EN 40 0
EN 60 0
EN 80 0
EN 100 0
EN 120 0
EN 140 0
EN 160 0
EN 180 0
EN 200 0
EN 220 0
EN 240 0
EN 260 -13823
EN 280 0
EN 300 0
EN 320 0
EN 340 0
EN 360 0
C31-RIB:PHE-S1
EN 20 0
EN 40 0
EN 60 0
EN 80 0
EN 100 -17630
EN 120 -17904
EN 140 0
EN 160 0
EN 180 0
EN 200 0
EN 220 0
EN 240 0
EN 260 0
EN 280 0
EN 300 0
EN 320 0
EN 340 0
EN 360 0
U34-RIB:GLY-CA
EN 20 0
EN 40 0
EN 60 0
EN 80 0
EN 100 0
EN 120 -16347
EN 140 0
EN 160 0
EN 180 0
EN 200 0
EN 220 0
EN 240 0
EN 260 0
EN 280 0
EN 300 0

EN 320   0
EN 340   0
EN 360   0
A-R5:CYS-CA
EN 20   0
EN 40   0
EN 60   -3590
EN 80   -2876
EN 100   -2682
EN 120   0
EN 140   0
EN 160   0
EN 180   0
EN 200   0
EN 220   0
EN 240   0
EN 260   0
EN 280   -4399
EN 300   0
EN 320   0
EN 340   0
EN 360   0
OMC-P:LYS-S2
EN 20   0
EN 40   0
EN 60   0
EN 80   -12912
EN 100   -12718
EN 120   0
EN 140   0
EN 160   0
EN 180   0
EN 200   0
EN 220   0
EN 240   0
EN 260   0
EN 280   0
EN 300   0
EN 320   0
EN 340   0
EN 360   0
IU-MY:LYS-S1
EN 20   0
EN 40   0
EN 60   -13794
EN 80   0
EN 100   -11881
EN 120   0
EN 140   0
EN 160   -15628
EN 180   0
EN 200   0
EN 220   0
EN 240   -13814
EN 260   0
EN 280   -11881
EN 300   0
EN 320   0

EN 340 0
EN 360 0
H2U-MY:TRP-CA
EN 20 0
EN 40 0
EN 60 0
EN 80 0
EN 100 0
EN 120 0
EN 140 0
EN 160 0
EN 180 0
EN 200 0
EN 220 0
EN 240 0
EN 260 0
EN 280 0
EN 300 0
EN 320 -16809
EN 340 0
EN 360 0
C31-MY:GLN-S1
EN 20 0
EN 40 0
EN 60 0
EN 80 0
EN 100 0
EN 120 0
EN 140 0
EN 160 0
EN 180 0
EN 200 0
EN 220 0
EN 240 0
EN 260 0
EN 280 0
EN 300 -17963
EN 320 0
EN 340 0
EN 360 0
QUO-M6:PHE-CA
EN 20 0
EN 40 0
EN 60 0
EN 80 0
EN 100 0
EN 120 0
EN 140 0
EN 160 0
EN 180 0
EN 200 0
EN 220 0
EN 240 0
EN 260 0
EN 280 -17630
EN 300 0
EN 320 -18236
EN 340 0

EN 360 0
U31-MY:GLU-S1
EN 20 0
EN 40 0
EN 60 0
EN 80 0
EN 100 0
EN 120 0
EN 140 -16524
EN 160 0
EN 180 0
EN 200 0
EN 220 0
EN 240 0
EN 260 0
EN 280 0
EN 300 0
EN 320 0
EN 340 0
EN 360 0
U34-MY:ASN-CA
EN 20 0
EN 40 0
EN 60 0
EN 80 0
EN 100 0
EN 120 0
EN 140 0
EN 160 0
EN 180 0
EN 200 0
EN 220 0
EN 240 -18438
EN 260 0
EN 280 0
EN 300 0
EN 320 0
EN 340 0
EN 360 0
QUO-M5:GLN-S2
EN 20 0
EN 40 0
EN 60 0
EN 80 0
EN 100 -17784
EN 120 0
EN 140 0
EN 160 0
EN 180 0
EN 200 0
EN 220 0
EN 240 0
EN 260 0
EN 280 0
EN 300 0
EN 320 0
EN 340 0
EN 360 0

U31-MY:VAL-CA
EN 20 0
EN 40 0
EN 60 0
EN 80 0
EN 100 0
EN 120 -16249
EN 140 0
EN 160 0
EN 180 0
EN 200 0
EN 220 0
EN 240 0
EN 260 0
EN 280 0
EN 300 0
EN 320 0
EN 340 0
EN 360 0
U34-P:TYR-S2
EN 20 0
EN 40 0
EN 60 0
EN 80 0
EN 100 0
EN 120 0
EN 140 0
EN 160 0
EN 180 0
EN 200 0
EN 220 0
EN 240 0
EN 260 0
EN 280 0
EN 300 0
EN 320 -18603
EN 340 0
EN 360 0
5BU-RIB:ARG-S2
EN 20 0
EN 40 0
EN 60 0
EN 80 0
EN 100 0
EN 120 0
EN 140 0
EN 160 0
EN 180 0
EN 200 -20405
EN 220 0
EN 240 0
EN 260 0
EN 280 0
EN 300 0
EN 320 0
EN 340 0
EN 360 0
U31-MY:GLU-CA

EN 20   0
EN 40   0
EN 60   0
EN 80   0
EN 100   0
EN 120   -16009
EN 140   0
EN 160   0
EN 180   0
EN 200   0
EN 220   0
EN 240   0
EN 260   0
EN 280   0
EN 300   0
EN 320   0
EN 340   0
EN 360   0
IU-MY:HIS-CA
EN 20   0
EN 40   -18056
EN 60   0
EN 80   0
EN 100   0
EN 120   0
EN 140   0
EN 160   0
EN 180   0
EN 200   0
EN 220   -16906
EN 240   0
EN 260   0
EN 280   0
EN 300   0
EN 320   0
EN 340   0
EN 360   0
FHU-MY:THR-CA
EN 20   0
EN 40   0
EN 60   0
EN 80   -15407
EN 100   0
EN 120   0
EN 140   0
EN 160   0
EN 180   0
EN 200   0
EN 220   -17617
EN 240   0
EN 260   -15389
EN 280   0
EN 300   -15423
EN 320   0
EN 340   0
EN 360   0
GTP-M5:SER-S1
EN 20   0

EN 40   0
EN 60   -17599
EN 80   0
EN 100   0
EN 120   0
EN 140   0
EN 160   0
EN 180   0
EN 200   0
EN 220   0
EN 240   0
EN 260   0
EN 280   0
EN 300   -16900
EN 320   0
EN 340   0
EN 360   0
DA-M6:GLN-CA
EN 20   0
EN 40   0
EN 60   0
EN 80   0
EN 100   0
EN 120   0
EN 140   0
EN 160   0
EN 180   0
EN 200   0
EN 220   0
EN 240   0
EN 260   -17929
EN 280   0
EN 300   0
EN 320   0
EN 340   0
EN 360   0
5BU-RIB:PRO-S1
EN 20   0
EN 40   0
EN 60   -16320
EN 80   0
EN 100   0
EN 120   0
EN 140   0
EN 160   0
EN 180   0
EN 200   0
EN 220   0
EN 240   0
EN 260   0
EN 280   0
EN 300   0
EN 320   0
EN 340   0
EN 360   0
C31-MY:SER-CA
EN 20   0
EN 40   0

EN 60 0
EN 80 0
EN 100 0
EN 120 0
EN 140 0
EN 160 0
EN 180 0
EN 200 0
EN 220 0
EN 240 -17619
EN 260 0
EN 280 0
EN 300 0
EN 320 0
EN 340 0
EN 360 0
H2U-MY:ILE-CA
EN 20 0
EN 40 0
EN 60 0
EN 80 0
EN 100 0
EN 120 0
EN 140 0
EN 160 -14162
EN 180 0
EN 200 0
EN 220 0
EN 240 0
EN 260 0
EN 280 0
EN 300 0
EN 320 0
EN 340 0
EN 360 0
H2U-MY:GLU-S2
EN 20 0
EN 40 0
EN 60 0
EN 80 0
EN 100 -11788
EN 120 0
EN 140 0
EN 160 0
EN 180 0
EN 200 0
EN 220 0
EN 240 0
EN 260 0
EN 280 0
EN 300 0
EN 320 0
EN 340 0
EN 360 0
H2U-MY:THR-S1
EN 20 0
EN 40 0
EN 60 0

EN 80   0
EN 100   0
EN 120   0
EN 140   0
EN 160   0
EN 180   0
EN 200   0
EN 220   0
EN 240   0
EN 260   0
EN 280   0
EN 300   0
EN 320   -13548
EN 340   0
EN 360   0
U31-MY:VAL-S1
EN 20   0
EN 40   0
EN 60   0
EN 80   0
EN 100   0
EN 120   -16250
EN 140   0
EN 160   0
EN 180   0
EN 200   0
EN 220   0
EN 240   0
EN 260   0
EN 280   0
EN 300   0
EN 320   0
EN 340   0
EN 360   0
QUO-M6:LYS-CA
EN 20   0
EN 40   0
EN 60   0
EN 80   -16294
EN 100   0
EN 120   0
EN 140   0
EN 160   0
EN 180   0
EN 200   0
EN 220   0
EN 240   0
EN 260   0
EN 280   0
EN 300   0
EN 320   0
EN 340   0
EN 360   0
DA-M6:LYS-S2
EN 20   0
EN 40   0
EN 60   0
EN 80   0

EN 100   0
EN 120   0
EN 140   0
EN 160   0
EN 180   0
EN 200   0
EN 220   0
EN 240   0
EN 260   0
EN 280   0
EN 300   -16362
EN 320   0
EN 340   0
EN 360   0
C31-RIB:TYR-S2
EN 20   0
EN 40   0
EN 60   -18905
EN 80   0
EN 100   0
EN 120   0
EN 140   0
EN 160   0
EN 180   0
EN 200   0
EN 220   0
EN 240   0
EN 260   -18174
EN 280   0
EN 300   0
EN 320   0
EN 340   0
EN 360   0
C31-P:GLU-S2
EN 20   0
EN 40   0
EN 60   0
EN 80   0
EN 100   0
EN 120   0
EN 140   0
EN 160   0
EN 180   0
EN 200   0
EN 220   0
EN 240   0
EN 260   -15952
EN 280   0
EN 300   0
EN 320   0
EN 340   0
EN 360   0
FMU-RIB:GLN-S2
EN 20   0
EN 40   0
EN 60   0
EN 80   0
EN 100   0

EN 120 0
EN 140 0
EN 160 0
EN 180 0
EN 200 0
EN 220 0
EN 240 0
EN 260 0
EN 280 0
EN 300 -17994
EN 320 0
EN 340 0
EN 360 0
DA-M5:HIS-S1
EN 20 0
EN 40 0
EN 60 0
EN 80 0
EN 100 0
EN 120 0
EN 140 0
EN 160 0
EN 180 0
EN 200 0
EN 220 0
EN 240 0
EN 260 -18902
EN 280 0
EN 300 0
EN 320 0
EN 340 0
EN 360 0
C31-RIB:ASP-CA
EN 20 0
EN 40 0
EN 60 0
EN 80 0
EN 100 0
EN 120 0
EN 140 0
EN 160 0
EN 180 0
EN 200 0
EN 220 0
EN 240 0
EN 260 0
EN 280 0
EN 300 0
EN 320 -17033
EN 340 0
EN 360 0
FHU-RIB:ALA-CA
EN 20 0
EN 40 0
EN 60 0
EN 80 -14163
EN 100 0
EN 120 -14242

EN 140 0
EN 160 0
EN 180 0
EN 200 0
EN 220 0
EN 240 0
EN 260 0
EN 280 0
EN 300 -14178
EN 320 0
EN 340 0
EN 360 0
FHU-MY:PHE-S2
EN 20 0
EN 40 0
EN 60 0
EN 80 0
EN 100 0
EN 120 0
EN 140 0
EN 160 0
EN 180 0
EN 200 0
EN 220 0
EN 240 0
EN 260 0
EN 280 0
EN 300 0
EN 320 -16522
EN 340 0
EN 360 0
IU-RIB:ALA-CA
EN 20 0
EN 40 0
EN 60 0
EN 80 0
EN 100 0
EN 120 0
EN 140 0
EN 160 0
EN 180 0
EN 200 0
EN 220 0
EN 240 0
EN 260 0
EN 280 -12467
EN 300 0
EN 320 0
EN 340 0
EN 360 0
C31-P:ASP-S1
EN 20 0
EN 40 0
EN 60 0
EN 80 0
EN 100 0
EN 120 -16703
EN 140 -17211

EN 160   0
EN 180   0
EN 200   0
EN 220   0
EN 240   0
EN 260   0
EN 280   0
EN 300   0
EN 320   -17035
EN 340   0
EN 360   0
QUO-RIB:GLN-S2
EN 20   0
EN 40   0
EN 60   0
EN 80   0
EN 100   0
EN 120   0
EN 140   0
EN 160   0
EN 180   0
EN 200   0
EN 220   0
EN 240   0
EN 260   0
EN 280   0
EN 300   0
EN 320   0
EN 340   -19167
EN 360   0
M2G-P:GLU-S2
EN 20   0
EN 40   0
EN 60   0
EN 80   0
EN 100   0
EN 120   0
EN 140   0
EN 160   0
EN 180   0
EN 200   0
EN 220   0
EN 240   0
EN 260   0
EN 280   0
EN 300   0
EN 320   0
EN 340   -17158
EN 360   0
GTP-M5:SER-CA
EN 20   0
EN 40   0
EN 60   0
EN 80   0
EN 100   -16691
EN 120   0
EN 140   0
EN 160   0

EN 180   0
EN 200   0
EN 220   0
EN 240   0
EN 260   0
EN 280   0
EN 300   0
EN 320   0
EN 340   0
EN 360   0
H2U-RIB:GLU-CA
EN 20   0
EN 40   0
EN 60   0
EN 80   0
EN 100   0
EN 120   0
EN 140   0
EN 160   0
EN 180   0
EN 200   0
EN 220   0
EN 240   0
EN 260   -11925
EN 280   0
EN 300   0
EN 320   0
EN 340   0
EN 360   0
OMC-P:LYS-CA
EN 20   0
EN 40   0
EN 60   0
EN 80   0
EN 100   -12665
EN 120   0
EN 140   0
EN 160   0
EN 180   0
EN 200   0
EN 220   0
EN 240   0
EN 260   0
EN 280   0
EN 300   0
EN 320   0
EN 340   0
EN 360   0
H2U-MY:PHE-CA
EN 20   0
EN 40   0
EN 60   0
EN 80   0
EN 100   0
EN 120   0
EN 140   -14424
EN 160   0
EN 180   0

EN 200 0
EN 220 0
EN 240 0
EN 260 0
EN 280 0
EN 300 0
EN 320 0
EN 340 0
EN 360 0
GTP-M6:ASN-CA
EN 20 0
EN 40 -20059
EN 60 0
EN 80 0
EN 100 0
EN 120 0
EN 140 0
EN 160 0
EN 180 0
EN 200 0
EN 220 0
EN 240 0
EN 260 0
EN 280 0
EN 300 0
EN 320 0
EN 340 0
EN 360 0
U31-P:ASN-CA
EN 20 0
EN 40 0
EN 60 0
EN 80 0
EN 100 -17510
EN 120 0
EN 140 0
EN 160 0
EN 180 0
EN 200 0
EN 220 0
EN 240 0
EN 260 0
EN 280 0
EN 300 0
EN 320 0
EN 340 0
EN 360 0
C31-RIB:ASP-S1
EN 20 0
EN 40 0
EN 60 0
EN 80 0
EN 100 0
EN 120 0
EN 140 0
EN 160 0
EN 180 0
EN 200 0

EN 220 0
EN 240 0
EN 260 0
EN 280 0
EN 300 0
EN 320 -17035
EN 340 0
EN 360 0
U31-MY:GLN-S2
EN 20 0
EN 40 0
EN 60 -18692
EN 80 0
EN 100 0
EN 120 0
EN 140 0
EN 160 0
EN 180 0
EN 200 0
EN 220 0
EN 240 0
EN 260 0
EN 280 0
EN 300 0
EN 320 0
EN 340 0
EN 360 0
FMU-MY:MET-S2
EN 20 0
EN 40 0
EN 60 0
EN 80 0
EN 100 0
EN 120 0
EN 140 0
EN 160 0
EN 180 0
EN 200 0
EN 220 0
EN 240 0
EN 260 0
EN 280 0
EN 300 -18879
EN 320 -19275
EN 340 0
EN 360 0
5BU-P:ALA-S1
EN 20 0
EN 40 0
EN 60 0
EN 80 0
EN 100 0
EN 120 0
EN 140 0
EN 160 0
EN 180 0
EN 200 0
EN 220 0

EN 240   0
EN 260   0
EN 280   0
EN 300   0
EN 320   -14574
EN 340   0
EN 360   0
QUO-RIB:ASN-CA
EN 20   0
EN 40   0
EN 60   0
EN 80   0
EN 100   0
EN 120   0
EN 140   0
EN 160   -18986
EN 180   0
EN 200   0
EN 220   0
EN 240   0
EN 260   0
EN 280   0
EN 300   0
EN 320   0
EN 340   0
EN 360   0
IU-MY:LEU-S1
EN 20   0
EN 40   0
EN 60   -12222
EN 80   0
EN 100   0
EN 120   0
EN 140   0
EN 160   0
EN 180   0
EN 200   0
EN 220   0
EN 240   0
EN 260   0
EN 280   0
EN 300   0
EN 320   0
EN 340   0
EN 360   0
IU-RIB:LYS-S1
EN 20   0
EN 40   0
EN 60   0
EN 80   0
EN 100   0
EN 120   0
EN 140   0
EN 160   0
EN 180   0
EN 200   0
EN 220   0
EN 240   -13814

EN 260   0
EN 280   0
EN 300   0
EN 320   0
EN 340   -14269
EN 360   0
C31-RIB:LEU-S1
EN 20   0
EN 40   0
EN 60   0
EN 80   0
EN 100   0
EN 120   0
EN 140   0
EN 160   0
EN 180   0
EN 200   0
EN 220   0
EN 240   0
EN 260   0
EN 280   0
EN 300   0
EN 320   -16144
EN 340   0
EN 360   0
C31-P:ALA-S1
EN 20   0
EN 40   0
EN 60   0
EN 80   0
EN 100   0
EN 120   0
EN 140   0
EN 160   0
EN 180   0
EN 200   0
EN 220   0
EN 240   0
EN 260   0
EN 280   -15686
EN 300   0
EN 320   0
EN 340   0
EN 360   0
DA-M5:LEU-S2
EN 20   0
EN 40   0
EN 60   0
EN 80   0
EN 100   0
EN 120   -15815
EN 140   0
EN 160   0
EN 180   0
EN 200   0
EN 220   0
EN 240   0
EN 260   0

EN 280   0
EN 300   0
EN 320   0
EN 340   0
EN 360   0
H2U-RIB:ARG-S1
EN 20   0
EN 40   0
EN 60   0
EN 80   0
EN 100   0
EN 120   0
EN 140   0
EN 160   0
EN 180   0
EN 200   0
EN 220   0
EN 240   0
EN 260   -12367
EN 280   0
EN 300   0
EN 320   0
EN 340   0
EN 360   0
GTP-M5:ASP-S1
EN 20   0
EN 40   0
EN 60   0
EN 80   0
EN 100   0
EN 120   0
EN 140   0
EN 160   0
EN 180   0
EN 200   0
EN 220   0
EN 240   0
EN 260   0
EN 280   0
EN 300   -16639
EN 320   0
EN 340   0
EN 360   0
H2U-P:TRP-CA
EN 20   0
EN 40   0
EN 60   0
EN 80   0
EN 100   -16203
EN 120   0
EN 140   0
EN 160   0
EN 180   0
EN 200   0
EN 220   0
EN 240   0
EN 260   0
EN 280   0

EN 300   0
EN 320   0
EN 340   0
EN 360   0
U34-MY:PRO-CA
EN 20   0
EN 40   0
EN 60   0
EN 80   0
EN 100   0
EN 120   0
EN 140   0
EN 160   0
EN 180   0
EN 200   0
EN 220   0
EN 240   0
EN 260   0
EN 280   0
EN 300   -17339
EN 320   0
EN 340   0
EN 360   0
IU-P:HIS-CA
EN 20   0
EN 40   0
EN 60   0
EN 80   0
EN 100   -16004
EN 120   0
EN 140   0
EN 160   0
EN 180   0
EN 200   0
EN 220   0
EN 240   0
EN 260   0
EN 280   0
EN 300   0
EN 320   0
EN 340   0
EN 360   0
U31-P:GLN-S2
EN 20   0
EN 40   0
EN 60   0
EN 80   0
EN 100   0
EN 120   0
EN 140   0
EN 160   0
EN 180   0
EN 200   0
EN 220   0
EN 240   0
EN 260   0
EN 280   -17784
EN 300   0

EN 320 0
EN 340 0
EN 360 0
IU-MY:SER-S1
EN 20 0
EN 40 0
EN 60 0
EN 80 -12661
EN 100 0
EN 120 -13745
EN 140 0
EN 160 0
EN 180 0
EN 200 0
EN 220 0
EN 240 0
EN 260 0
EN 280 0
EN 300 0
EN 320 0
EN 340 0
EN 360 0
IU-P:HIS-S2
EN 20 0
EN 40 0
EN 60 0
EN 80 0
EN 100 0
EN 120 -16294
EN 140 0
EN 160 0
EN 180 0
EN 200 0
EN 220 0
EN 240 0
EN 260 0
EN 280 0
EN 300 0
EN 320 0
EN 340 0
EN 360 0
U31-P:GLN-S1
EN 20 0
EN 40 0
EN 60 0
EN 80 0
EN 100 0
EN 120 0
EN 140 0
EN 160 0
EN 180 0
EN 200 0
EN 220 0
EN 240 -18681
EN 260 0
EN 280 0
EN 300 0
EN 320 0

EN 340 0
EN 360 0
C31-RIB:SER-CA
EN 20 0
EN 40 0
EN 60 0
EN 80 0
EN 100 0
EN 120 0
EN 140 0
EN 160 0
EN 180 0
EN 200 0
EN 220 0
EN 240 0
EN 260 0
EN 280 0
EN 300 0
EN 320 0
EN 340 -18073
EN 360 0
FHU-MY:GLN-S2
EN 20 0
EN 40 0
EN 60 0
EN 80 0
EN 100 -16066
EN 120 0
EN 140 0
EN 160 0
EN 180 0
EN 200 0
EN 220 0
EN 240 0
EN 260 0
EN 280 -16066
EN 300 0
EN 320 0
EN 340 0
EN 360 0
FHU-P:PRO-CA
EN 20 0
EN 40 0
EN 60 0
EN 80 0
EN 100 0
EN 120 -15685
EN 140 0
EN 160 0
EN 180 0
EN 200 0
EN 220 0
EN 240 0
EN 260 0
EN 280 0
EN 300 0
EN 320 0
EN 340 0

EN 360   0
QUO-RIB:LYS-S1
EN 20   0
EN 40   0
EN 60   0
EN 80   0
EN 100   0
EN 120   0
EN 140   -16886
EN 160   0
EN 180   0
EN 200   0
EN 220   0
EN 240   0
EN 260   0
EN 280   0
EN 300   0
EN 320   0
EN 340   0
EN 360   0
IU-P:SER-CA
EN 20   0
EN 40   0
EN 60   0
EN 80   0
EN 100   -13969
EN 120   0
EN 140   0
EN 160   0
EN 180   0
EN 200   0
EN 220   0
EN 240   0
EN 260   0
EN 280   0
EN 300   0
EN 320   0
EN 340   0
EN 360   0
U31-RIB:ASN-S1
EN 20   0
EN 40   0
EN 60   0
EN 80   0
EN 100   0
EN 120   0
EN 140   0
EN 160   0
EN 180   0
EN 200   0
EN 220   0
EN 240   -18438
EN 260   0
EN 280   0
EN 300   0
EN 320   0
EN 340   0
EN 360   0

FHU-P:ASP-S2
EN 20 0
EN 40 0
EN 60 0
EN 80 0
EN 100 0
EN 120 0
EN 140 0
EN 160 -16234
EN 180 0
EN 200 0
EN 220 0
EN 240 0
EN 260 0
EN 280 0
EN 300 0
EN 320 0
EN 340 0
EN 360 0
5BU-RIB:ILE-S1
EN 20 0
EN 40 0
EN 60 0
EN 80 0
EN 100 0
EN 120 0
EN 140 0
EN 160 0
EN 180 0
EN 200 0
EN 220 0
EN 240 -15884
EN 260 0
EN 280 0
EN 300 0
EN 320 0
EN 340 0
EN 360 0
FHU-RIB:LYS-CA
EN 20 0
EN 40 0
EN 60 0
EN 80 0
EN 100 0
EN 120 0
EN 140 0
EN 160 0
EN 180 0
EN 200 0
EN 220 -16786
EN 240 0
EN 260 0
EN 280 0
EN 300 0
EN 320 0
EN 340 0
EN 360 0
DA-M6:HIS-S2

EN 20   0
EN 40   0
EN 60   0
EN 80   0
EN 100   0
EN 120   0
EN 140   0
EN 160   0
EN 180   0
EN 200   0
EN 220   0
EN 240   0
EN 260   -18919
EN 280   0
EN 300   0
EN 320   0
EN 340   0
EN 360   0
QUO-RIB:LEU-S2
EN 20   0
EN 40   0
EN 60   0
EN 80   0
EN 100   0
EN 120   0
EN 140   0
EN 160   0
EN 180   0
EN 200   0
EN 220   0
EN 240   0
EN 260   -17435
EN 280   0
EN 300   0
EN 320   0
EN 340   0
EN 360   0
IU-MY:MET-CA
EN 20   0
EN 40   0
EN 60   0
EN 80   0
EN 100   0
EN 120   0
EN 140   0
EN 160   -15911
EN 180   0
EN 200   0
EN 220   0
EN 240   0
EN 260   0
EN 280   0
EN 300   0
EN 320   0
EN 340   0
EN 360   0
IU-MY:ARG-S1
EN 20   0

EN 40 0
EN 60 0
EN 80 0
EN 100 -12960
EN 120 0
EN 140 -13741
EN 160 0
EN 180 0
EN 200 0
EN 220 0
EN 240 0
EN 260 0
EN 280 0
EN 300 0
EN 320 0
EN 340 0
EN 360 0
DA-M6:GLU-S1
EN 20 0
EN 40 0
EN 60 0
EN 80 0
EN 100 0
EN 120 0
EN 140 0
EN 160 0
EN 180 0
EN 200 0
EN 220 0
EN 240 0
EN 260 0
EN 280 0
EN 300 0
EN 320 0
EN 340 -17126
EN 360 0
GTP-M5:GLY-CA
EN 20 0
EN 40 0
EN 60 -16982
EN 80 0
EN 100 0
EN 120 0
EN 140 0
EN 160 0
EN 180 0
EN 200 0
EN 220 0
EN 240 0
EN 260 0
EN 280 0
EN 300 0
EN 320 0
EN 340 0
EN 360 0
H2U-MY:ASN-CA
EN 20 0
EN 40 0

EN 60 0
EN 80 0
EN 100 0
EN 120 0
EN 140 -14303
EN 160 0
EN 180 0
EN 200 0
EN 220 0
EN 240 0
EN 260 0
EN 280 -13522
EN 300 0
EN 320 0
EN 340 0
EN 360 0
U31-MY:TYR-CA
EN 20 0
EN 40 0
EN 60 0
EN 80 0
EN 100 0
EN 120 0
EN 140 0
EN 160 0
EN 180 0
EN 200 0
EN 220 0
EN 240 0
EN 260 0
EN 280 0
EN 300 0
EN 320 -18599
EN 340 0
EN 360 0
FMU-MY:ALA-CA
EN 20 0
EN 40 0
EN 60 0
EN 80 0
EN 100 -15686
EN 120 0
EN 140 0
EN 160 0
EN 180 0
EN 200 0
EN 220 0
EN 240 0
EN 260 0
EN 280 0
EN 300 0
EN 320 0
EN 340 0
EN 360 0
U31-MY:GLN-CA
EN 20 0
EN 40 0
EN 60 -18661

EN 80   0
EN 100   0
EN 120   0
EN 140   0
EN 160   0
EN 180   0
EN 200   0
EN 220   0
EN 240   0
EN 260   0
EN 280   0
EN 300   0
EN 320   0
EN 340   0
EN 360   0
QUO-M5:LYS-S2
EN 20   0
EN 40   0
EN 60   0
EN 80   -16347
EN 100   0
EN 120   0
EN 140   0
EN 160   0
EN 180   0
EN 200   0
EN 220   0
EN 240   0
EN 260   0
EN 280   0
EN 300   0
EN 320   0
EN 340   0
EN 360   0
FMU-P:ASP-S2
EN 20   0
EN 40   0
EN 60   0
EN 80   0
EN 100   0
EN 120   0
EN 140   0
EN 160   -17951
EN 180   0
EN 200   0
EN 220   0
EN 240   0
EN 260   0
EN 280   0
EN 300   0
EN 320   0
EN 340   0
EN 360   0
QUO-M5:ARG-S1
EN 20   0
EN 40   0
EN 60   0
EN 80   0

EN 100   0
EN 120   -16452
EN 140   0
EN 160   0
EN 180   0
EN 200   0
EN 220   0
EN 240   0
EN 260   0
EN 280   0
EN 300   0
EN 320   0
EN 340   0
EN 360   0
PSU-RIB:ARG-S2
EN 20   0
EN 40   0
EN 60   0
EN 80   0
EN 100   0
EN 120   -12476
EN 140   0
EN 160   0
EN 180   0
EN 200   0
EN 220   0
EN 240   0
EN 260   0
EN 280   0
EN 300   0
EN 320   0
EN 340   0
EN 360   0
U34-P:HIS-S2
EN 20   0
EN 40   0
EN 60   0
EN 80   0
EN 100   0
EN 120   0
EN 140   0
EN 160   -20219
EN 180   0
EN 200   0
EN 220   0
EN 240   0
EN 260   0
EN 280   -18742
EN 300   0
EN 320   0
EN 340   0
EN 360   0
M2G-P:GLU-S1
EN 20   0
EN 40   0
EN 60   0
EN 80   0
EN 100   0

EN 120 0
EN 140 0
EN 160 0
EN 180 0
EN 200 0
EN 220 0
EN 240 0
EN 260 0
EN 280 0
EN 300 0
EN 320 0
EN 340 0
EN 360 -19417
QUO-M6:GLN-S2
EN 20 0
EN 40 0
EN 60 0
EN 80 0
EN 100 -17784
EN 120 0
EN 140 0
EN 160 0
EN 180 0
EN 200 0
EN 220 0
EN 240 0
EN 260 0
EN 280 0
EN 300 0
EN 320 0
EN 340 0
EN 360 0
FMU-MY:ILE-CA
EN 20 0
EN 40 0
EN 60 0
EN 80 0
EN 100 0
EN 120 0
EN 140 0
EN 160 0
EN 180 0
EN 200 0
EN 220 0
EN 240 0
EN 260 0
EN 280 -16673
EN 300 0
EN 320 0
EN 340 0
EN 360 0
U31-RIB:ASN-S2
EN 20 0
EN 40 0
EN 60 0
EN 80 0
EN 100 0
EN 120 0

```
EN 140   0
EN 160   0
EN 180   0
EN 200   0
EN 220   0
EN 240   -18459
EN 260   0
EN 280   0
EN 300   0
EN 320   0
EN 340   0
EN 360   0
5BU-MY:PRO-S1
EN 20   0
EN 40   0
EN 60   -16320
EN 80   0
EN 100   0
EN 120   0
EN 140   0
EN 160   0
EN 180   0
EN 200   0
EN 220   0
EN 240   0
EN 260   0
EN 280   0
EN 300   0
EN 320   0
EN 340   0
EN 360   0
H2U-P:ARG-S1
EN 20   0
EN 40   0
EN 60   0
EN 80   -12385
EN 100   0
EN 120   0
EN 140   0
EN 160   0
EN 180   0
EN 200   0
EN 220   0
EN 240   0
EN 260   0
EN 280   0
EN 300   0
EN 320   0
EN 340   0
EN 360   0
H2U-MY:GLY-CA
EN 20   0
EN 40   0
EN 60   0
EN 80   0
EN 100   0
EN 120   -12360
EN 140   0
```

EN 160 0
EN 180 0
EN 200 0
EN 220 0
EN 240 0
EN 260 0
EN 280 0
EN 300 0
EN 320 0
EN 340 0
EN 360 0
U34-RIB:TYR-S2
EN 20 0
EN 40 0
EN 60 0
EN 80 0
EN 100 0
EN 120 0
EN 140 0
EN 160 0
EN 180 0
EN 200 0
EN 220 0
EN 240 0
EN 260 0
EN 280 0
EN 300 -18207
EN 320 0
EN 340 0
EN 360 0
H2U-MY:LYS-S2
EN 20 0
EN 40 0
EN 60 0
EN 80 0
EN 100 0
EN 120 0
EN 140 0
EN 160 0
EN 180 0
EN 200 0
EN 220 0
EN 240 -13093
EN 260 0
EN 280 0
EN 300 0
EN 320 0
EN 340 0
EN 360 0
H2U-MY:PHE-S1
EN 20 0
EN 40 0
EN 60 0
EN 80 0
EN 100 0
EN 120 0
EN 140 -14424
EN 160 0

EN 180   0
EN 200   0
EN 220   0
EN 240   0
EN 260   0
EN 280   0
EN 300   0
EN 320   0
EN 340   0
EN 360   0
FMU-MY:PRO-S1
EN 20   0
EN 40   0
EN 60   0
EN 80   0
EN 100   0
EN 120   0
EN 140   -17911
EN 160   0
EN 180   0
EN 200   0
EN 220   0
EN 240   0
EN 260   0
EN 280   0
EN 300   0
EN 320   0
EN 340   0
EN 360   0
QUO-M6:LYS-S2
EN 20   0
EN 40   0
EN 60   -17061
EN 80   0
EN 100   0
EN 120   0
EN 140   0
EN 160   0
EN 180   0
EN 200   0
EN 220   0
EN 240   0
EN 260   0
EN 280   0
EN 300   0
EN 320   0
EN 340   0
EN 360   0
FMU-RIB:MET-S1
EN 20   0
EN 40   0
EN 60   0
EN 80   0
EN 100   0
EN 120   0
EN 140   0
EN 160   0
EN 180   0

EN 200   0
EN 220   0
EN 240   0
EN 260   0
EN 280   0
EN 300   0
EN 320   -19264
EN 340   0
EN 360   0
H2U-RIB:GLU-S1
EN 20   0
EN 40   0
EN 60   0
EN 80   0
EN 100   0
EN 120   0
EN 140   0
EN 160   0
EN 180   0
EN 200   0
EN 220   0
EN 240   0
EN 260   0
EN 280   -11755
EN 300   0
EN 320   0
EN 340   0
EN 360   0
FHU-RIB:TYR-CA
EN 20   0
EN 40   0
EN 60   0
EN 80   0
EN 100   0
EN 120   0
EN 140   0
EN 160   0
EN 180   0
EN 200   0
EN 220   0
EN 240   0
EN 260   0
EN 280   0
EN 300   -18203
EN 320   0
EN 340   0
EN 360   0
FHU-P:TYR-CA
EN 20   0
EN 40   -20542
EN 60   0
EN 80   0
EN 100   0
EN 120   0
EN 140   0
EN 160   0
EN 180   -20068
EN 200   0

EN 220 0
EN 240 0
EN 260 0
EN 280 0
EN 300 0
EN 320 0
EN 340 0
EN 360 0
GTP-M5:THR-CA
EN 20 0
EN 40 0
EN 60 0
EN 80 0
EN 100 -16930
EN 120 0
EN 140 0
EN 160 0
EN 180 0
EN 200 0
EN 220 0
EN 240 0
EN 260 0
EN 280 0
EN 300 0
EN 320 0
EN 340 0
EN 360 0
U31-MY:MET-S1
EN 20 0
EN 40 0
EN 60 0
EN 80 -18853
EN 100 0
EN 120 0
EN 140 0
EN 160 0
EN 180 0
EN 200 0
EN 220 0
EN 240 0
EN 260 0
EN 280 0
EN 300 0
EN 320 0
EN 340 0
EN 360 0
FMU-RIB:ASP-S2
EN 20 0
EN 40 0
EN 60 0
EN 80 0
EN 100 0
EN 120 -16748
EN 140 0
EN 160 0
EN 180 0
EN 200 0
EN 220 0

EN 240   0
EN 260   0
EN 280   0
EN 300   0
EN 320   0
EN 340   0
EN 360   0
H2U-RIB:LYS-S2
EN 20   0
EN 40   0
EN 60   0
EN 80   0
EN 100   0
EN 120   0
EN 140   0
EN 160   0
EN 180   0
EN 200   0
EN 220   0
EN 240   0
EN 260   0
EN 280   0
EN 300   -12375
EN 320   0
EN 340   0
EN 360   0
DA-M5:THR-S1
EN 20   0
EN 40   0
EN 60   0
EN 80   0
EN 100   0
EN 120   0
EN 140   0
EN 160   0
EN 180   0
EN 200   0
EN 220   0
EN 240   0
EN 260   0
EN 280   0
EN 300   0
EN 320   -17536
EN 340   0
EN 360   0
U31-MY:TYR-S1
EN 20   0
EN 40   0
EN 60   0
EN 80   0
EN 100   0
EN 120   0
EN 140   0
EN 160   0
EN 180   0
EN 200   0
EN 220   0
EN 240   0

EN 260 0
EN 280 0
EN 300 -18203
EN 320 0
EN 340 0
EN 360 0
U34-P:GLU-CA
EN 20 0
EN 40 0
EN 60 0
EN 80 0
EN 100 0
EN 120 0
EN 140 0
EN 160 0
EN 180 0
EN 200 0
EN 220 0
EN 240 0
EN 260 0
EN 280 0
EN 300 0
EN 320 0
EN 340 0
EN 360 -19410
FMU-MY:ARG-CA
EN 20 0
EN 40 0
EN 60 0
EN 80 -16373
EN 100 0
EN 120 0
EN 140 0
EN 160 0
EN 180 0
EN 200 0
EN 220 0
EN 240 0
EN 260 0
EN 280 0
EN 300 0
EN 320 0
EN 340 0
EN 360 0
H2U-MY:GLN-S2
EN 20 0
EN 40 0
EN 60 0
EN 80 0
EN 100 0
EN 120 0
EN 140 0
EN 160 0
EN 180 0
EN 200 0
EN 220 0
EN 240 0
EN 260 0

EN 280   0
EN 300   0
EN 320   0
EN 340   0
EN 360   -17470
U34-MY:PHE-S1
EN 20   0
EN 40   0
EN 60   0
EN 80   0
EN 100   0
EN 120   0
EN 140   0
EN 160   0
EN 180   0
EN 200   0
EN 220   0
EN 240   0
EN 260   0
EN 280   0
EN 300   -17840
EN 320   0
EN 340   0
EN 360   0
QUO-M6:LEU-S2
EN 20   0
EN 40   0
EN 60   0
EN 80   0
EN 100   0
EN 120   0
EN 140   0
EN 160   0
EN 180   0
EN 200   0
EN 220   0
EN 240   -16470
EN 260   -15718
EN 280   0
EN 300   0
EN 320   0
EN 340   0
EN 360   0
FHU-P:PRO-S1
EN 20   0
EN 40   0
EN 60   0
EN 80   0
EN 100   0
EN 120   -15685
EN 140   0
EN 160   0
EN 180   0
EN 200   0
EN 220   0
EN 240   0
EN 260   0
EN 280   0

EN 300   0
EN 320   0
EN 340   0
EN 360   0
FMU-P:ALA-S1
EN 20   0
EN 40   0
EN 60   0
EN 80   0
EN 100   0
EN 120   0
EN 140   0
EN 160   0
EN 180   0
EN 200   0
EN 220   0
EN 240   0
EN 260   -15862
EN 280   0
EN 300   0
EN 320   0
EN 340   0
EN 360   0
5BU-P:THR-CA
EN 20   0
EN 40   0
EN 60   0
EN 80   0
EN 100   0
EN 120   0
EN 140   0
EN 160   -16689
EN 180   0
EN 200   0
EN 220   0
EN 240   0
EN 260   0
EN 280   0
EN 300   0
EN 320   0
EN 340   0
EN 360   0
FHU-MY:ILE-CA
EN 20   0
EN 40   0
EN 60   0
EN 80   0
EN 100   0
EN 120   0
EN 140   -15737
EN 160   0
EN 180   0
EN 200   0
EN 220   0
EN 240   0
EN 260   0
EN 280   0
EN 300   0

EN 320 -15561
EN 340 0
EN 360 0
DA-M6:THR-CA
EN 20 0
EN 40 0
EN 60 0
EN 80 0
EN 100 0
EN 120 0
EN 140 0
EN 160 0
EN 180 0
EN 200 0
EN 220 0
EN 240 0
EN 260 0
EN 280 0
EN 300 -17140
EN 320 0
EN 340 -18313
EN 360 0
C31-MY:GLU-S1
EN 20 0
EN 40 0
EN 60 0
EN 80 0
EN 100 0
EN 120 0
EN 140 -16524
EN 160 0
EN 180 0
EN 200 0
EN 220 0
EN 240 0
EN 260 0
EN 280 0
EN 300 0
EN 320 0
EN 340 0
EN 360 0
DA-M6:ASN-CA
EN 20 0
EN 40 0
EN 60 0
EN 80 0
EN 100 0
EN 120 0
EN 140 0
EN 160 0
EN 180 0
EN 200 0
EN 220 0
EN 240 -18438
EN 260 0
EN 280 0
EN 300 0
EN 320 0

EN 340 0
EN 360 0
IU-P:ARG-S2
EN 20 0
EN 40 0
EN 60 0
EN 80 0
EN 100 0
EN 120 0
EN 140 0
EN 160 0
EN 180 0
EN 200 0
EN 220 0
EN 240 -14397
EN 260 0
EN 280 0
EN 300 0
EN 320 0
EN 340 0
EN 360 0
C31-RIB:ASP-S2
EN 20 0
EN 40 -20741
EN 60 0
EN 80 0
EN 100 0
EN 120 0
EN 140 0
EN 160 0
EN 180 0
EN 200 0
EN 220 0
EN 240 0
EN 260 0
EN 280 0
EN 300 0
EN 320 0
EN 340 0
EN 360 0
DA-M5:GLU-S2
EN 20 0
EN 40 0
EN 60 0
EN 80 0
EN 100 0
EN 120 0
EN 140 0
EN 160 0
EN 180 0
EN 200 0
EN 220 0
EN 240 0
EN 260 0
EN 280 0
EN 300 0
EN 320 -16381
EN 340 0

EN 360   0
C31-MY:ASP-CA
EN 20   0
EN 40   0
EN 60   0
EN 80   0
EN 100   0
EN 120   0
EN 140   0
EN 160   0
EN 180   0
EN 200   0
EN 220   0
EN 240   -17356
EN 260   0
EN 280   0
EN 300   0
EN 320   0
EN 340   0
EN 360   0
U34-MY:TYR-S2
EN 20   0
EN 40   0
EN 60   0
EN 80   0
EN 100   0
EN 120   0
EN 140   0
EN 160   0
EN 180   0
EN 200   0
EN 220   0
EN 240   0
EN 260   0
EN 280   0
EN 300   -18207
EN 320   0
EN 340   0
EN 360   0
H2U-MY:GLN-S1
EN 20   0
EN 40   0
EN 60   0
EN 80   0
EN 100   0
EN 120   0
EN 140   0
EN 160   0
EN 180   0
EN 200   0
EN 220   0
EN 240   0
EN 260   0
EN 280   0
EN 300   0
EN 320   0
EN 340   0
EN 360   -17440

5BU-MY:ILE-S1
EN 20 0
EN 40 0
EN 60 0
EN 80 0
EN 100 0
EN 120 0
EN 140 0
EN 160 0
EN 180 0
EN 200 0
EN 220 0
EN 240 0
EN 260 0
EN 280 0
EN 300 -15166
EN 320 0
EN 340 0
EN 360 0
QUO-M6:GLU-S2
EN 20 0
EN 40 0
EN 60 0
EN 80 0
EN 100 0
EN 120 0
EN 140 0
EN 160 -17252
EN 180 0
EN 200 0
EN 220 0
EN 240 0
EN 260 0
EN 280 0
EN 300 0
EN 320 0
EN 340 0
EN 360 0
U31-P:ARG-CA
EN 20 0
EN 40 0
EN 60 0
EN 80 0
EN 100 0
EN 120 0
EN 140 0
EN 160 0
EN 180 0
EN 200 0
EN 220 0
EN 240 0
EN 260 0
EN 280 0
EN 300 0
EN 320 0
EN 340 -17562
EN 360 0
5BU-P:PRO-CA

EN 20 0
EN 40 0
EN 60 0
EN 80 0
EN 100 0
EN 120 0
EN 140 0
EN 160 0
EN 180 0
EN 200 0
EN 220 0
EN 240 0
EN 260 0
EN 280 0
EN 300 -15622
EN 320 0
EN 340 0
EN 360 0
DA-M5:ASP-S1
EN 20 0
EN 40 0
EN 60 0
EN 80 0
EN 100 0
EN 120 0
EN 140 0
EN 160 -17906
EN 180 0
EN 200 0
EN 220 0
EN 240 0
EN 260 0
EN 280 0
EN 300 0
EN 320 0
EN 340 0
EN 360 0
GTP-M6:GLY-CA
EN 20 0
EN 40 0
EN 60 0
EN 80 -16268
EN 100 0
EN 120 0
EN 140 0
EN 160 0
EN 180 0
EN 200 0
EN 220 0
EN 240 0
EN 260 0
EN 280 0
EN 300 0
EN 320 0
EN 340 0
EN 360 0
H2U-P:LEU-S2
EN 20 0

EN 40 0
EN 60 0
EN 80 0
EN 100 -11554
EN 120 0
EN 140 0
EN 160 0
EN 180 0
EN 200 0
EN 220 0
EN 240 0
EN 260 0
EN 280 0
EN 300 0
EN 320 0
EN 340 0
EN 360 0
C31-MY:GLN-S2
EN 20 0
EN 40 0
EN 60 0
EN 80 0
EN 100 0
EN 120 0
EN 140 0
EN 160 0
EN 180 0
EN 200 0
EN 220 0
EN 240 0
EN 260 0
EN 280 0
EN 300 -17994
EN 320 0
EN 340 0
EN 360 0
QUO-M5:ASN-CA
EN 20 0
EN 40 0
EN 60 0
EN 80 -17704
EN 100 0
EN 120 0
EN 140 0
EN 160 0
EN 180 0
EN 200 0
EN 220 0
EN 240 0
EN 260 0
EN 280 0
EN 300 0
EN 320 0
EN 340 0
EN 360 0
IU-P:ASP-S2
EN 20 0
EN 40 0

EN 60   0
EN 80   0
EN 100   0
EN 120   0
EN 140   -14534
EN 160   0
EN 180   0
EN 200   0
EN 220   0
EN 240   0
EN 260   0
EN 280   0
EN 300   0
EN 320   0
EN 340   0
EN 360   0
QUO-M6:GLU-CA
EN 20   0
EN 40   0
EN 60   0
EN 80   0
EN 100   0
EN 120   0
EN 140   -16517
EN 160   0
EN 180   0
EN 200   0
EN 220   0
EN 240   0
EN 260   0
EN 280   0
EN 300   0
EN 320   0
EN 340   0
EN 360   0
FHU-P:ASP-S1
EN 20   0
EN 40   0
EN 60   0
EN 80   0
EN 100   0
EN 120   0
EN 140   0
EN 160   -16188
EN 180   0
EN 200   0
EN 220   0
EN 240   0
EN 260   0
EN 280   0
EN 300   0
EN 320   0
EN 340   0
EN 360   0
U31-P:ARG-S2
EN 20   0
EN 40   0
EN 60   0

EN 80   0
EN 100   0
EN 120   0
EN 140   0
EN 160   0
EN 180   0
EN 200   0
EN 220   0
EN 240   0
EN 260   0
EN 280   0
EN 300   -16401
EN 320   0
EN 340   0
EN 360   0
QUO-P:ASN-S2
EN 20   0
EN 40   0
EN 60   0
EN 80   0
EN 100   0
EN 120   0
EN 140   0
EN 160   0
EN 180   0
EN 200   -23462
EN 220   0
EN 240   0
EN 260   0
EN 280   0
EN 300   0
EN 320   0
EN 340   0
EN 360   0
U31-RIB:GLN-S1
EN 20   0
EN 40   0
EN 60   0
EN 80   -17947
EN 100   0
EN 120   0
EN 140   0
EN 160   0
EN 180   0
EN 200   0
EN 220   0
EN 240   0
EN 260   0
EN 280   0
EN 300   0
EN 320   0
EN 340   0
EN 360   0
FHU-P:SER-S1
EN 20   0
EN 40   0
EN 60   0
EN 80   0

EN 100 0
EN 120 0
EN 140 0
EN 160 0
EN 180 0
EN 200 0
EN 220 0
EN 240 0
EN 260 0
EN 280 0
EN 300 0
EN 320 0
EN 340 -16356
EN 360 0
M2G-P:SER-S1
EN 20 0
EN 40 0
EN 60 0
EN 80 0
EN 100 0
EN 120 0
EN 140 0
EN 160 0
EN 180 0
EN 200 0
EN 220 0
EN 240 0
EN 260 0
EN 280 0
EN 300 0
EN 320 -17296
EN 340 0
EN 360 0
4SU-P:THR-S1
EN 20 0
EN 40 0
EN 60 0
EN 80 0
EN 100 0
EN 120 0
EN 140 0
EN 160 -18407
EN 180 0
EN 200 0
EN 220 0
EN 240 0
EN 260 0
EN 280 0
EN 300 0
EN 320 0
EN 340 0
EN 360 0
QUO-M6:ASN-S2
EN 20 0
EN 40 0
EN 60 0
EN 80 -17725
EN 100 0

EN 120   0
EN 140   0
EN 160   0
EN 180   0
EN 200   0
EN 220   0
EN 240   0
EN 260   0
EN 280   0
EN 300   0
EN 320   0
EN 340   0
EN 360   0
GTP-M5:THR-S1
EN 20   0
EN 40   0
EN 60   0
EN 80   0
EN 100   -16930
EN 120   0
EN 140   0
EN 160   0
EN 180   0
EN 200   0
EN 220   0
EN 240   0
EN 260   0
EN 280   0
EN 300   0
EN 320   0
EN 340   0
EN 360   0
FMU-RIB:ALA-S1
EN 20   0
EN 40   0
EN 60   0
EN 80   -15880
EN 100   0
EN 120   0
EN 140   0
EN 160   0
EN 180   0
EN 200   0
EN 220   0
EN 240   0
EN 260   0
EN 280   0
EN 300   0
EN 320   0
EN 340   0
EN 360   0
H2U-MY:ARG-S1
EN 20   0
EN 40   0
EN 60   0
EN 80   0
EN 100   0
EN 120   0

```
EN 140   0
EN 160   0
EN 180   0
EN 200   0
EN 220   0
EN 240   0
EN 260   0
EN 280   0
EN 300   -12401
EN 320   0
EN 340   0
EN 360   0
C31-P:ASN-S1
EN 20   0
EN 40   0
EN 60   0
EN 80   0
EN 100   0
EN 120   0
EN 140   -18291
EN 160   0
EN 180   0
EN 200   0
EN 220   0
EN 240   0
EN 260   0
EN 280   0
EN 300   0
EN 320   0
EN 340   0
EN 360   0
OMC-P:LYS-S1
EN 20   0
EN 40   0
EN 60   0
EN 80   0
EN 100   -12670
EN 120   0
EN 140   0
EN 160   0
EN 180   0
EN 200   0
EN 220   0
EN 240   0
EN 260   0
EN 280   0
EN 300   0
EN 320   0
EN 340   0
EN 360   0
U34-MY:ASP-S2
EN 20   0
EN 40   0
EN 60   0
EN 80   0
EN 100   -16475
EN 120   0
EN 140   0
```

EN 160   0
EN 180   0
EN 200   0
EN 220   0
EN 240   0
EN 260   0
EN 280   0
EN 300   0
EN 320   0
EN 340   0
EN 360   0
U31-RIB:GLN-CA
EN 20   0
EN 40   0
EN 60   0
EN 80   -17947
EN 100   0
EN 120   0
EN 140   0
EN 160   0
EN 180   0
EN 200   0
EN 220   0
EN 240   0
EN 260   0
EN 280   0
EN 300   0
EN 320   0
EN 340   0
EN 360   0
U31-RIB:PHE-S1
EN 20   0
EN 40   0
EN 60   0
EN 80   0
EN 100   0
EN 120   0
EN 140   0
EN 160   0
EN 180   0
EN 200   0
EN 220   0
EN 240   0
EN 260   0
EN 280   0
EN 300   -17840
EN 320   0
EN 340   0
EN 360   0
U34-P:ARG-S1
EN 20   0
EN 40   0
EN 60   0
EN 80   0
EN 100   0
EN 120   0
EN 140   0
EN 160   0

EN 180 0
EN 200 0
EN 220 0
EN 240 0
EN 260 0
EN 280 0
EN 300 0
EN 320 -16784
EN 340 0
EN 360 0
DA-M6:HIS-CA
EN 20 0
EN 40 0
EN 60 0
EN 80 0
EN 100 0
EN 120 0
EN 140 0
EN 160 0
EN 180 0
EN 200 0
EN 220 0
EN 240 0
EN 260 -18902
EN 280 0
EN 300 0
EN 320 0
EN 340 0
EN 360 0
FMU-MY:ALA-S1
EN 20 0
EN 40 0
EN 60 0
EN 80 0
EN 100 -15686
EN 120 0
EN 140 0
EN 160 0
EN 180 0
EN 200 0
EN 220 0
EN 240 0
EN 260 0
EN 280 0
EN 300 0
EN 320 0
EN 340 0
EN 360 0
H2U-P:GLU-CA
EN 20 0
EN 40 0
EN 60 0
EN 80 -11943
EN 100 0
EN 120 0
EN 140 0
EN 160 0
EN 180 0

EN 200   0
EN 220   0
EN 240   0
EN 260   0
EN 280   0
EN 300   0
EN 320   0
EN 340   0
EN 360   0
IU-P:HIS-S1
EN 20   0
EN 40   0
EN 60   0
EN 80   0
EN 100   -16004
EN 120   0
EN 140   0
EN 160   0
EN 180   0
EN 200   0
EN 220   0
EN 240   0
EN 260   0
EN 280   0
EN 300   0
EN 320   0
EN 340   0
EN 360   0
DA-M5:ASP-S2
EN 20   0
EN 40   0
EN 60   0
EN 80   0
EN 100   0
EN 120   0
EN 140   0
EN 160   -17951
EN 180   0
EN 200   0
EN 220   0
EN 240   0
EN 260   0
EN 280   0
EN 300   0
EN 320   0
EN 340   0
EN 360   0
DA-M6:GLU-S2
EN 20   0
EN 40   0
EN 60   0
EN 80   0
EN 100   0
EN 120   0
EN 140   0
EN 160   0
EN 180   0
EN 200   0

EN 220   0
EN 240   0
EN 260   0
EN 280   0
EN 300   0
EN 320   0
EN 340   -17158
EN 360   0
FHU-RIB:PRO-CA
EN 20   0
EN 40   0
EN 60   0
EN 80   0
EN 100   0
EN 120   0
EN 140   0
EN 160   0
EN 180   0
EN 200   0
EN 220   0
EN 240   -16340
EN 260   0
EN 280   0
EN 300   0
EN 320   0
EN 340   0
EN 360   0
QUO-M6:ASP-CA
EN 20   0
EN 40   0
EN 60   0
EN 80   0
EN 100   0
EN 120   -16700
EN 140   0
EN 160   0
EN 180   0
EN 200   0
EN 220   0
EN 240   0
EN 260   0
EN 280   0
EN 300   0
EN 320   0
EN 340   0
EN 360   0
DA-M6:SER-S1
EN 20   0
EN 40   0
EN 60   0
EN 80   0
EN 100   0
EN 120   0
EN 140   -17472
EN 160   0
EN 180   0
EN 200   0
EN 220   0

EN 240   0
EN 260   0
EN 280   0
EN 300   0
EN 320   0
EN 340   0
EN 360   0
H2U-RIB:PHE-CA
EN 20   0
EN 40   0
EN 60   0
EN 80   0
EN 100   0
EN 120   0
EN 140   0
EN 160   0
EN 180   0
EN 200   0
EN 220   0
EN 240   0
EN 260   0
EN 280   -13643
EN 300   0
EN 320   0
EN 340   0
EN 360   0
FMU-P:PHE-CA
EN 20   0
EN 40   0
EN 60   0
EN 80   0
EN 100   0
EN 120   -17904
EN 140   0
EN 160   0
EN 180   0
EN 200   0
EN 220   0
EN 240   0
EN 260   0
EN 280   0
EN 300   0
EN 320   0
EN 340   0
EN 360   0
QUO-M5:PHE-CA
EN 20   0
EN 40   0
EN 60   0
EN 80   0
EN 100   0
EN 120   0
EN 140   0
EN 160   0
EN 180   0
EN 200   0
EN 220   0
EN 240   0

EN 260   0
EN 280   0
EN 300   -17840
EN 320   0
EN 340   0
EN 360   0
FMU-RIB:GLN-CA
EN 20   0
EN 40   0
EN 60   0
EN 80   0
EN 100   0
EN 120   0
EN 140   0
EN 160   0
EN 180   0
EN 200   0
EN 220   0
EN 240   0
EN 260   0
EN 280   -17753
EN 300   0
EN 320   0
EN 340   0
EN 360   0
C31-MY:LEU-S1
EN 20   0
EN 40   0
EN 60   0
EN 80   0
EN 100   0
EN 120   0
EN 140   0
EN 160   0
EN 180   0
EN 200   0
EN 220   0
EN 240   0
EN 260   0
EN 280   -15538
EN 300   0
EN 320   0
EN 340   0
EN 360   0
QUO-RIB:ASP-S1
EN 20   0
EN 40   0
EN 60   0
EN 80   0
EN 100   0
EN 120   0
EN 140   -17211
EN 160   0
EN 180   0
EN 200   0
EN 220   0
EN 240   0
EN 260   0

EN 280   0
EN 300   0
EN 320   0
EN 340   0
EN 360   0
FMU-RIB:GLU-S1
EN 20   0
EN 40   0
EN 60   0
EN 80   0
EN 100   0
EN 120   0
EN 140   0
EN 160   0
EN 180   0
EN 200   0
EN 220   0
EN 240   0
EN 260   0
EN 280   0
EN 300   0
EN 320   0
EN 340   -17126
EN 360   0
G:HIS-S2
EN WoCr   -7410
EN Sug   -7078
EN Hoo   -7016
C:GLU-CA
EN WoCr   581
EN Sug   2681
EN Hoo   2594
A:ASN-CA
EN WoCr   -4071
EN Sug   -1539
EN Hoo   -1465
C:LEU-CA
EN WoCr   -914
EN Sug   837
EN Hoo   2294
U:ASN-S2
EN WoCr   -3091
EN Sug   -6897
EN Hoo   -7215
G:ARG-CA
EN WoCr   -3746
EN Sug   -4283
EN Hoo   -1838
C:SER-CA
EN WoCr   -2967
EN Sug   -3604
EN Hoo   -3128
A:GLU-CA
EN WoCr   -2103
EN Sug   -364
EN Hoo   458
G:GLN-CA
EN WoCr   2095

EN Sug   -3807
EN Hoo   -1449
C:ASP-S2
EN WoCr   -3836
EN Sug   -5674
EN Hoo   -3473
C:VAL-S1
EN WoCr   -915
EN Sug   -906
EN Hoo   -3080
G:ALA-S1
EN WoCr   -2915
EN Sug   -3357
EN Hoo   -2824
C:GLN-S1
EN WoCr   -4173
EN Sug   -5589
EN Hoo   -1887
G:GLN-S1
EN WoCr   -3010
EN Sug   -5440
EN Hoo   -3317
U:LYS-S2
EN WoCr   -4143
EN Sug   -5895
EN Hoo   -8467
C:ASN-S2
EN WoCr   -1601
EN Sug   -5789
EN Hoo   -5349
A:LYS-S2
EN WoCr   -6013
EN Sug   -5197
EN Hoo   -8348
G:LYS-S1
EN WoCr   -3616
EN Sug   -3708
EN Hoo   -4431
C:GLU-S1
EN WoCr   1150
EN Sug   -833
EN Hoo   -937
G:MET-CA
EN WoCr   -1984
EN Sug   -2668
EN Hoo   2796
C:TRP-S1
EN WoCr   811
EN Sug   -3363
EN Hoo   861
G:TRP-CA
EN WoCr   -2710
EN Sug   -3732
EN Hoo   -1
U:GLU-S1
EN WoCr   -1818
EN Sug   -1592
EN Hoo   -1203

G:GLU-S2
EN WoCr -3122
EN Sug -4232
EN Hoo -2034
A:PHE-S1
EN WoCr -295
EN Sug -2907
EN Hoo 1547
G:CYS-CA
EN WoCr -1671
EN Sug 1118
EN Hoo 0
A:ASN-S2
EN WoCr -7284
EN Sug -6667
EN Hoo -4992
G:ALA-CA
EN WoCr -1492
EN Sug -681
EN Hoo -1870
A:ASN-S1
EN WoCr -4898
EN Sug -5096
EN Hoo -3240
G:LEU-CA
EN WoCr -963
EN Sug -255
EN Hoo -3657
G:ASN-CA
EN WoCr -3647
EN Sug -4504
EN Hoo -3238
A:TYR-S1
EN WoCr -3361
EN Sug -4549
EN Hoo -1918
C:LEU-S2
EN WoCr -2438
EN Sug 748
EN Hoo 2527
G:LYS-CA
EN WoCr -3692
EN Sug -3039
EN Hoo -4397
C:TYR-S1
EN WoCr -915
EN Sug -579
EN Hoo 959
G:TRP-S1
EN WoCr -3777
EN Sug -4491
EN Hoo -4657
U:THR-S1
EN WoCr -3031
EN Sug -4616
EN Hoo -233
C:MET-CA
EN WoCr 3056

EN Sug   -1857
EN Hoo   -1175
U:ASN-S1
EN WoCr   -3807
EN Sug   -4020
EN Hoo   -4013
C:LYS-S1
EN WoCr   -3680
EN Sug   -4337
EN Hoo   -2950
A:ILE-S1
EN WoCr   -4338
EN Sug   -2718
EN Hoo   2956
G:HIS-CA
EN WoCr   -3145
EN Sug   -4034
EN Hoo   -3609
G:GLN-S2
EN WoCr   -7202
EN Sug   -5532
EN Hoo   -6417
C:LEU-S1
EN WoCr   -890
EN Sug   -2874
EN Hoo   -2876
U:LYS-CA
EN WoCr   -2041
EN Sug   -2295
EN Hoo   -3019
A:MET-CA
EN WoCr   -5124
EN Sug   -446
EN Hoo   -746
G:ASP-CA
EN WoCr   -3854
EN Sug   -1205
EN Hoo   1809
C:ALA-S1
EN WoCr   -2452
EN Sug   -3561
EN Hoo   -2769
U:PRO-S1
EN WoCr   -1779
EN Sug   -5573
EN Hoo   -1093
C:PHE-CA
EN WoCr   650
EN Sug   -1143
EN Hoo   -1831
G:VAL-S1
EN WoCr   -3488
EN Sug   -1481
EN Hoo   2043
A:TRP-S1
EN WoCr   -3784
EN Sug   -3125
EN Hoo   -1012

U:GLU-S2
EN WoCr   -80
EN Sug   -1033
EN Hoo   -3809
U:GLN-S1
EN WoCr   -4482
EN Sug   -4058
EN Hoo   -3636
G:ARG-S2
EN WoCr   -6654
EN Sug   -5638
EN Hoo   -7556
G:TYR-S2
EN WoCr   -3963
EN Sug   -3086
EN Hoo   -3223
G:ASN-S2
EN WoCr   -6753
EN Sug   -4706
EN Hoo   -6645
A:HIS-CA
EN WoCr   -4975
EN Sug   -4750
EN Hoo   -2530
U:SER-S1
EN WoCr   -533
EN Sug   -5952
EN Hoo   -1259
C:VAL-CA
EN WoCr   918
EN Sug   -178
EN Hoo   2354
G:GLY-CA
EN WoCr   -3989
EN Sug   -5303
EN Hoo   -6061
C:ASP-S1
EN WoCr   -2073
EN Sug   -3433
EN Hoo   -198
A:HIS-S2
EN WoCr   -6794
EN Sug   -6834
EN Hoo   -4878
A:LEU-S2
EN WoCr   -3906
EN Sug   -614
EN Hoo   -2485
U:TRP-CA
EN WoCr   2321
EN Sug   2559
EN Hoo   -1325
U:ASP-S2
EN WoCr   -4107
EN Sug   -5630
EN Hoo   -3229
A:TRP-CA
EN WoCr   -1354

EN Sug   -1314
EN Hoo   -1012
A:ALA-S1
EN WoCr   -4436
EN Sug   -3029
EN Hoo   -4749
G:HIS-S1
EN WoCr   -4885
EN Sug   -5064
EN Hoo   -2343
A:SER-S1
EN WoCr   -5111
EN Sug   -5009
EN Hoo   -6122
G:GLU-CA
EN WoCr   -2085
EN Sug   -353
EN Hoo   1421
U:VAL-CA
EN WoCr   -1748
EN Sug   -688
EN Hoo   -3527
G:ARG-S1
EN WoCr   -5323
EN Sug   -4632
EN Hoo   -6367
C:ASP-CA
EN WoCr   -2586
EN Sug   -3346
EN Hoo   -2741
U:PHE-S2
EN WoCr   -827
EN Sug   -5220
EN Hoo   6224
U:TYR-S2
EN WoCr   -1426
EN Sug   -8
EN Hoo   -1498
U:TYR-CA
EN WoCr   -2776
EN Sug   -2538
EN Hoo   2138
U:PHE-CA
EN WoCr   -1059
EN Sug   -2423
EN Hoo   3957
C:LYS-CA
EN WoCr   -2905
EN Sug   -4283
EN Hoo   -3283
U:HIS-CA
EN WoCr   -201
EN Sug   -4496
EN Hoo   -693
C:GLU-S2
EN WoCr   -3281
EN Sug   -3882
EN Hoo   -2585

U:CYS-S1
EN WoCr -1608
EN Sug 2064
EN Hoo 902
A:ALA-CA
EN WoCr -2502
EN Sug -1288
EN Hoo -324
U:CYS-CA
EN WoCr -6116
EN Sug 3781
EN Hoo 902
U:LEU-S2
EN WoCr 206
EN Sug -2012
EN Hoo -527
A:ASP-S2
EN WoCr -4094
EN Sug -3521
EN Hoo -5344
G:MET-S2
EN WoCr -3878
EN Sug -5090
EN Hoo -2681
A:GLN-CA
EN WoCr -3853
EN Sug -1108
EN Hoo -3828
U:ARG-CA
EN WoCr -3797
EN Sug -4053
EN Hoo -5255
U:VAL-S1
EN WoCr 3099
EN Sug -789
EN Hoo -2304
A:PRO-CA
EN WoCr -8130
EN Sug -2173
EN Hoo -3395
U:HIS-S1
EN WoCr -1034
EN Sug -4899
EN Hoo -693
U:MET-S2
EN WoCr -858
EN Sug -1897
EN Hoo -4428
A:PRO-S1
EN WoCr -5918
EN Sug -5104
EN Hoo -2728
G:ASP-S1
EN WoCr -2819
EN Sug -3469
EN Hoo -970
A:GLN-S2
EN WoCr -6091

EN Sug   -5024
EN Hoo   -5601
U:ALA-CA
EN WoCr   228
EN Sug   -3543
EN Hoo   -3573
A:ARG-S1
EN WoCr   -4186
EN Sug   -3908
EN Hoo   -6453
U:PHE-S1
EN WoCr   -823
EN Sug   -1505
EN Hoo   3957
U:ASN-CA
EN WoCr   -3661
EN Sug   -3783
EN Hoo   -2352
C:GLN-CA
EN WoCr   -3770
EN Sug   -1868
EN Hoo   2586
G:SER-S1
EN WoCr   -4478
EN Sug   -5368
EN Hoo   -4685
G:PHE-S1
EN WoCr   -3596
EN Sug   -3291
EN Hoo   3112
G:SER-CA
EN WoCr   -4638
EN Sug   -4296
EN Hoo   -3783
U:MET-CA
EN WoCr   3853
EN Sug   103
EN Hoo   1472
C:ILE-S1
EN WoCr   2771
EN Sug   -2321
EN Hoo   -841
A:GLU-S2
EN WoCr   -4048
EN Sug   -2651
EN Hoo   -3466
U:HIS-S2
EN WoCr   -9514
EN Sug   -6446
EN Hoo   -3471
A:ARG-S2
EN WoCr   -7601
EN Sug   -6685
EN Hoo   -8204
A:LEU-CA
EN WoCr   -1610
EN Sug   -246
EN Hoo   1540

G:PHE-S2
EN WoCr 435
EN Sug -3770
EN Hoo -476
G:CYS-S1
EN WoCr -1289
EN Sug 3388
EN Hoo 0
G:ASP-S2
EN WoCr -6030
EN Sug -5125
EN Hoo -5365
A:PHE-S2
EN WoCr -4025
EN Sug -3481
EN Hoo 42
C:GLY-CA
EN WoCr -6412
EN Sug -6691
EN Hoo -4980
G:TRP-S2
EN WoCr -7362
EN Sug -5414
EN Hoo -834
U:GLU-CA
EN WoCr -41
EN Sug 504
EN Hoo 3129
G:TYR-S1
EN WoCr -2457
EN Sug -2407
EN Hoo -3016
U:GLN-S2
EN WoCr -6129
EN Sug -5741
EN Hoo -3667
A:HIS-S1
EN WoCr -5355
EN Sug -5746
EN Hoo -2269
U:ILE-CA
EN WoCr -102
EN Sug 3806
EN Hoo -174
A:MET-S1
EN WoCr -4278
EN Sug -1352
EN Hoo -3384
C:TRP-CA
EN WoCr 22
EN Sug 1978
EN Hoo 1866
G:ASN-S1
EN WoCr -5370
EN Sug -3839
EN Hoo -5895
G:PRO-CA
EN WoCr -2417

EN Sug   -4698
EN Hoo   -2491
A:THR-CA
EN WoCr   -2731
EN Sug   -1661
EN Hoo   -2865
G:LEU-S1
EN WoCr   225
EN Sug   905
EN Hoo   870
A:ILE-CA
EN WoCr   -1675
EN Sug   -1617
EN Hoo   -147
G:ILE-CA
EN WoCr   -762
EN Sug   650
EN Hoo   2060
A:ARG-CA
EN WoCr   -4582
EN Sug   -3950
EN Hoo   -2315
C:HIS-S2
EN WoCr   -3624
EN Sug   -5902
EN Hoo   -6386
C:ASN-CA
EN WoCr   -616
EN Sug   -4156
EN Hoo   -1431
U:GLY-CA
EN WoCr   -3506
EN Sug   -6974
EN Hoo   -1755
A:GLY-CA
EN WoCr   -5154
EN Sug   -6225
EN Hoo   -6504
G:PRO-S1
EN WoCr   -3568
EN Sug   -6366
EN Hoo   -2980
C:HIS-CA
EN WoCr   -3132
EN Sug   -5005
EN Hoo   -4849
C:ARG-S1
EN WoCr   -6943
EN Sug   -4936
EN Hoo   -6772
C:THR-S1
EN WoCr   -3600
EN Sug   -5821
EN Hoo   -3300
C:CYS-S1
EN WoCr   2746
EN Sug   2985
EN Hoo   1371

C:HIS-S1
EN WoCr -1889
EN Sug -5551
EN Hoo -656
A:TYR-CA
EN WoCr -4565
EN Sug -1498
EN Hoo -1918
A:LEU-S1
EN WoCr -2235
EN Sug -2733
EN Hoo 6
A:CYS-S1
EN WoCr -844
EN Sug 1111
EN Hoo -1507
A:CYS-CA
EN WoCr -131
EN Sug 1111
EN Hoo -503
C:PHE-S2
EN WoCr 264
EN Sug 459
EN Hoo -729
C:CYS-CA
EN WoCr 2746
EN Sug 4702
EN Hoo 3088
G:LEU-S2
EN WoCr -2460
EN Sug -2485
EN Hoo -2871
A:ASP-CA
EN WoCr -4136
EN Sug -1730
EN Hoo -1636
U:LEU-CA
EN WoCr 263
EN Sug 2772
EN Hoo -692
G:TYR-CA
EN WoCr -1640
EN Sug 852
EN Hoo 2075
U:MET-S1
EN WoCr -444
EN Sug -2263
EN Hoo 2477
U:LYS-S1
EN WoCr -2606
EN Sug -3881
EN Hoo -2492
U:ARG-S2
EN WoCr -2670
EN Sug -7150
EN Hoo -5509
C:ILE-CA
EN WoCr 1938

EN Sug   -1483
EN Hoo   -2782
G:GLU-S1
EN WoCr   -1406
EN Sug   -2006
EN Hoo   950
G:ILE-S1
EN WoCr   -1592
EN Sug   -586
EN Hoo   4069
A:ASP-S1
EN WoCr   -2458
EN Sug   -3195
EN Hoo   -3164
U:SER-CA
EN WoCr   -335
EN Sug   -2689
EN Hoo   -2828
U:PRO-CA
EN WoCr   -5292
EN Sug   -3223
EN Hoo   -2863
U:ILE-S1
EN WoCr   -1276
EN Sug   -3289
EN Hoo   -4137
U:THR-CA
EN WoCr   429
EN Sug   1450
EN Hoo   -786
C:LYS-S2
EN WoCr   -4433
EN Sug   -6177
EN Hoo   -7638
C:ARG-S2
EN WoCr   -4374
EN Sug   -5790
EN Hoo   -8186
U:ARG-S1
EN WoCr   -3838
EN Sug   -2795
EN Hoo   -6530
G:THR-S1
EN WoCr   -4429
EN Sug   -4066
EN Hoo   -2681
C:PHE-S1
EN WoCr   -2332
EN Sug   3318
EN Hoo   1704
C:TYR-CA
EN WoCr   -4
EN Sug   525
EN Hoo   2346
A:LYS-S1
EN WoCr   -4401
EN Sug   -4959
EN Hoo   -4659

A:VAL-S1
EN WoCr -3146
EN Sug -3656
EN Hoo -1832
C:THR-CA
EN WoCr -1538
EN Sug -4507
EN Hoo 1399
A:TRP-S2
EN WoCr -5148
EN Sug -7085
EN Hoo -561
C:PRO-CA
EN WoCr -5633
EN Sug -5040
EN Hoo 106
C:GLN-S2
EN WoCr -7551
EN Sug -6723
EN Hoo -4559
A:MET-S2
EN WoCr -4390
EN Sug -3519
EN Hoo -496
C:SER-S1
EN WoCr -2917
EN Sug -4515
EN Hoo -4463
C:ALA-CA
EN WoCr -195
EN Sug -3702
EN Hoo -2449
G:PHE-CA
EN WoCr 1474
EN Sug -1931
EN Hoo 2107
C:ARG-CA
EN WoCr -620
EN Sug -1232
EN Hoo -5597
C:MET-S2
EN WoCr -1252
EN Sug -6054
EN Hoo 1116
C:MET-S1
EN WoCr 1339
EN Sug -2171
EN Hoo 2393
A:GLN-S1
EN WoCr -7448
EN Sug -3151
EN Hoo -674
A:GLU-S1
EN WoCr -3452
EN Sug -2660
EN Hoo 0
U:ASP-S1
EN WoCr -6548

EN Sug   -3547
EN Hoo   -2288
A:LYS-CA
EN WoCr   -3874
EN Sug   -3751
EN Hoo   -2399
U:ASP-CA
EN WoCr   1645
EN Sug   132
EN Hoo   2438
U:TRP-S1
EN WoCr   0
EN Sug   -3665
EN Hoo   -1776
C:TRP-S2
EN WoCr   -5959
EN Sug   -6664
EN Hoo   -1463
G:MET-S1
EN WoCr   -1177
EN Sug   -1676
EN Hoo   2084
U:TRP-S2
EN WoCr   -2615
EN Sug   -6953
EN Hoo   -6306
C:ASN-S1
EN WoCr   -5783
EN Sug   -5566
EN Hoo   -5027
A:TYR-S2
EN WoCr   -3668
EN Sug   -4758
EN Hoo   -4084
C:TYR-S2
EN WoCr   -919
EN Sug   -2268
EN Hoo   -3169
U:TYR-S1
EN WoCr   -2190
EN Sug   3039
EN Hoo   -4832
A:PHE-CA
EN WoCr   -1374
EN Sug   -2003
EN Hoo   1165
U:GLN-CA
EN WoCr   -2005
EN Sug   -1570
EN Hoo   -5607
U:ALA-S1
EN WoCr   1673
EN Sug   -3444
EN Hoo   -3110
A:SER-CA
EN WoCr   -4697
EN Sug   -3417
EN Hoo   -841

G:LYS-S2
EN WoCr   -5397
EN Sug   -5966
EN Hoo   -8180
A:THR-S1
EN WoCr   -4726
EN Sug   -4466
EN Hoo   -4778
G:THR-CA
EN WoCr   -255
EN Sug   -2555
EN Hoo   -627
A:VAL-CA
EN WoCr   -3982
EN Sug   -857
EN Hoo   -1131
U:LEU-S1
EN WoCr   -2082
EN Sug   -408
EN Hoo   1871
C:PRO-S1
EN WoCr   -2151
EN Sug   -5131
EN Hoo   -5031
G:VAL-CA
EN WoCr   -1052
EN Sug   1941
EN Hoo   1332
