# Supplementary material for: DARS-RNP and QUASI-RNP: New statistical potentials for protein-RNA docking
Source: BMC Bioinformatics. 2011 Aug 18;12:348. doi: 10.1186/1471-2105-12-348 (PMC3179970; doi:10.1186/1471-2105-12-348)
Supplement: Additional file 4 — Observed.pdf. Observed number of contacts in each distance, angle, and site bin, for each pair wise interaction. [file 1471-2105-12-348-S4.PDF]

U-P:CYS-S1
3 0
4 0
5 0
6 0
7 6
8 3
9 6
A-RIB:GLN-S2
3 0
4 4
5 32
6 44
7 44
8 104
9 108
QUO-M6:GLU-S2
3 0
4 0
5 4
6 0
7 0
8 0
9 0
G-P:ASN-CA
3 0
4 4
5 40
6 56
7 68
8 104
9 148
DA-RIB:ALA-S1
3 0
4 0
5 0
6 0
7 3
8 0
9 0
FMU-RIB:PHE-S2
3 0
4 0
5 0
6 6
7 0
8 0
9 0
A-R5:TYR-CA
3 0
4 0
5 0
6 28
7 24
8 40
9 71
A-R6:GLY-CA
3 0

4 4
5 20
6 64
7 88
8 164
9 219
U31-RIB:PHE-CA
3 0
4 0
5 0
6 0
7 0
8 0
9 3
U-Y:GLU-S1
3 0
4 0
5 6
6 0
7 6
8 21
9 42
IU-RIB:VAL-S1
3 0
4 0
5 0
6 0
7 0
8 0
9 2
C31-MY:LEU-S1
3 0
4 0
5 0
6 3
7 0
8 0
9 0
G-RIB:ILE-S1
3 0
4 0
5 12
6 20
7 31
8 39
9 66
OMC-P:LYS-S1
3 0
4 0
5 0
6 0
7 3
8 0
9 0
M2G-P:SER-S1
3 0
4 0
5 0

6 0
7 0
8 0
9 4
U-P:MET-CA
3 0
4 0
5 12
6 6
7 6
8 15
9 27
A-RIB:GLY-CA
3 0
4 12
5 96
6 76
7 152
8 196
9 348
C-Y:HIS-CA
3 0
4 0
5 0
6 6
7 24
8 30
9 48
C-RIB:MET-S2
3 0
4 3
5 12
6 30
7 36
8 27
9 45
C-P:GLU-S1
3 0
4 3
5 12
6 12
7 30
8 78
9 126
H2U-MY:GLY-CA
3 0
4 0
5 0
6 0
7 3
8 0
9 0
G-R5:TRP-CA
3 0
4 0
5 0
6 8
7 8

8 20
9 40
A-R6:THR-CA
3 0
4 0
5 4
6 24
7 60
8 28
9 92
A-P:PHE-S1
3 0
4 0
5 8
6 4
7 24
8 32
9 76
G-RIB:GLU-S2
3 0
4 0
5 48
6 47
7 52
8 99
9 139
A-R5:LEU-S2
3 0
4 8
5 16
6 20
7 20
8 68
9 84
A-RIB:ASP-S2
3 0
4 4
5 20
6 48
7 48
8 84
9 108
C31-RIB:GLN-S2
3 0
4 0
5 0
6 0
7 0
8 0
9 3
QUO-M6:ARG-S2
3 0
4 0
5 0
6 4
7 0
8 4
9 0

U-P:ALA-S1
3 0
4 12
5 45
6 21
7 54
8 69
9 114
A-R5:ARG-S2
3 0
4 44
5 40
6 92
7 76
8 180
9 407
U-RIB:CYS-S1
3 0
4 0
5 0
6 3
7 2
8 6
9 6
C-RIB:GLU-S2
3 0
4 0
5 30
6 36
7 33
8 69
9 113
U-RIB:HIS-S2
3 0
4 3
5 15
6 39
7 30
8 42
9 48
C-P:GLU-CA
3 0
4 0
5 18
6 6
7 9
8 51
9 129
H2U-RIB:PHE-S2
3 0
4 0
5 0
6 3
7 0
8 0
9 0
C-Y:PHE-CA
3 0

4 0
5 0
6 6
7 6
8 9
9 36
G-R5:ASP-S2
3 0
4 0
5 4
6 16
7 96
8 108
9 164
G-P:HIS-S2
3 0
4 0
5 28
6 68
7 64
8 76
9 120
C31-P:ASN-S1
3 0
4 0
5 0
6 0
7 0
8 3
9 0
U-RIB:ALA-CA
3 0
4 0
5 12
6 18
7 54
8 59
9 126
C-P:LYS-S2
3 0
4 48
5 183
6 75
7 153
8 192
9 249
C-RIB:PRO-S1
3 0
4 0
5 23
6 48
7 54
8 90
9 105
QUO-M5:PHE-CA
3 0
4 0
5 0

6 0
7 0
8 0
9 4
C31-RIB:PHE-CA
3 0
4 0
5 0
6 0
7 0
8 6
9 0
C-P:GLN-S1
3 0
4 0
5 27
6 18
7 48
8 99
9 114
U-RIB:PHE-CA
3 0
4 0
5 6
6 6
7 18
8 27
9 50
QUO-RIB:ASN-S2
3 0
4 0
5 4
6 0
7 0
8 0
9 0
G-P:VAL-CA
3 0
4 0
5 0
6 24
7 44
8 88
9 108
U31-P:ASP-S2
3 0
4 0
5 0
6 3
7 0
8 3
9 3
FHU-RIB:THR-S1
3 0
4 0
5 0
6 0
7 3

8 3
9 3
U-P:GLU-CA
3 0
4 0
5 12
6 6
7 21
8 42
9 69
FMU-MY:GLU-S2
3 0
4 0
5 3
6 0
7 0
8 0
9 0
G-RIB:ARG-S2
3 0
4 4
5 68
6 171
7 272
8 416
9 537
A-RIB:GLN-S1
3 0
4 0
5 0
6 48
7 48
8 52
9 128
M2G-P:GLU-S2
3 0
4 0
5 0
6 0
7 4
8 0
9 0
QUO-RIB:LEU-S1
3 0
4 0
5 0
6 0
7 0
8 8
9 0
A-R6:VAL-CA
3 0
4 0
5 4
6 20
7 56
8 44
9 72

C-P:TRP-S1
3   0
4   0
5   0
6   3
7   18
8   30
9   12
G-RIB:LEU-CA
3   0
4   0
5   4
6   28
7   48
8   83
9   160
G-P:TRP-CA
3   0
4   0
5   12
6   12
7   8
8   36
9   32
A-R6:HIS-S1
3   0
4   8
5   16
6   20
7   28
8   56
9   60
U-Y:ASP-S1
3   0
4   0
5   3
6   3
7   12
8   27
9   51
A-P:THR-CA
3   0
4   0
5   32
6   44
7   52
8   104
9   172
U-RIB:LEU-S2
3   0
4   0
5   21
6   21
7   33
8   21
9   42
C31-RIB:PHE-S1
3   0

4 0
5 0
6 3
7 3
8 0
9 0
A-RIB:ASN-S2
3 0
4 0
5 56
6 52
7 48
8 84
9 196
U31-P:ARG-S2
3 0
4 0
5 0
6 0
7 0
8 3
9 0
DA-RIB:MET-S2
3 0
4 0
5 3
6 0
7 0
8 0
9 0
U-P:ARG-CA
3 0
4 3
5 21
6 54
7 54
8 120
9 171
U-RIB:TYR-CA
3 0
4 0
5 3
6 3
7 15
8 30
9 44
DA-M6:SER-CA
3 0
4 0
5 3
6 0
7 0
8 0
9 3
FHU-P:LEU-CA
3 0
4 0
5 0

6 3
7 0
8 6
9 6
A-RIB:SER-S1
3 0
4 0
5 36
6 76
7 64
8 120
9 184
C-P:TRP-CA
3 0
4 0
5 0
6 3
7 6
8 18
9 33
U-Y:TRP-CA
3 0
4 0
5 0
6 3
7 9
8 3
9 9
5BU-P:ILE-CA
3 0
4 0
5 0
6 3
7 0
8 6
9 0
C31-P:ASP-CA
3 0
4 0
5 0
6 0
7 3
8 3
9 3
U-P:PHE-S2
3 0
4 0
5 0
6 21
7 9
8 27
9 45
G-RIB:ASP-S1
3 0
4 0
5 28
6 44
7 48

8   68
9   211
A-P:MET-CA
3   0
4   0
5   16
6   4
7   16
8   28
9   56
G-R6:TYR-S2
3   0
4   4
5   10
6   24
7   8
8   46
9   88
G-RIB:GLN-S1
3   0
4   0
5   28
6   59
7   76
8   88
9   124
G-R5:ARG-CA
3   0
4   0
5   8
6   32
7   32
8   88
9   228
H2U-MY:PRO-S1
3   0
4   0
5   0
6   0
7   3
8   0
9   3
A-P:PHE-S2
3   0
4   0
5   0
6   16
7   28
8   60
9   64
C-P:LEU-CA
3   0
4   0
5   9
6   6
7   36
8   78
9   99

A-RIB:LYS-S2
3 0
4 0
5 48
6 95
7 216
8 228
9 312
C31-P:ALA-CA
3 0
4 0
5 0
6 0
7 0
8 3
9 0
C-Y:ILE-S1
3 0
4 0
5 3
6 6
7 15
8 27
9 45
U-Y:ARG-CA
3 0
4 3
5 3
6 9
7 36
8 42
9 93
A-R6:TYR-S2
3 0
4 8
5 12
6 24
7 24
8 28
9 60
C31-MY:PHE-CA
3 0
4 0
5 0
6 0
7 3
8 0
9 0
U31-MY:PHE-S1
3 0
4 0
5 0
6 0
7 3
8 0
9 0
C-Y:TYR-CA
3 0

4 0
5 3
6 18
7 12
8 18
9 36
U-P:LYS-CA
3 0
4 0
5 18
6 30
7 66
8 120
9 165
U-Y:ILE-S1
3 0
4 0
5 6
6 9
7 9
8 17
9 30
A-R6:THR-S1
3 0
4 4
5 20
6 44
7 36
8 48
9 123
A-P:TRP-S1
3 0
4 0
5 4
6 4
7 4
8 36
9 12
C-P:GLN-S2
3 0
4 0
5 42
6 57
7 42
8 99
9 102
U-RIB:GLU-S1
3 0
4 0
5 9
6 15
7 24
8 18
9 30
U-P:HIS-S1
3 0
4 0
5 0

6   15
7   18
8   54
9   66
QUO-RIB:PHE-S2
3   0
4   0
5   0
6   4
7   0
8   0
9   4
C31-P:ALA-S1
3   0
4   0
5   0
6   0
7   0
8   3
9   0
U-Y:PHE-CA
3   0
4   0
5   0
6   6
7   20
8   15
9   33
QUO-M6:GLN-S1
3   0
4   0
5   0
6   0
7   0
8   0
9   4
C-P:VAL-S1
3   0
4   0
5   9
6   48
7   39
8   90
9   156
C-Y:ASP-S1
3   0
4   0
5   12
6   15
7   45
8   63
9   102
A-P:PRO-S1
3   0
4   0
5   32
6   36
7   36

8 88
9 164
G-R6:GLU-S1
3 0
4 0
5 0
6 24
7 47
8 74
9 107
U-Y:GLN-CA
3 0
4 0
5 3
6 3
7 9
8 23
9 42
C-P:ILE-CA
3 0
4 0
5 0
6 9
7 30
8 57
9 84
U-Y:MET-S2
3 0
4 0
5 3
6 6
7 12
8 6
9 24
C-P:THR-S1
3 0
4 0
5 51
6 33
7 51
8 111
9 144
A-RIB:TRP-CA
3 0
4 0
5 0
6 12
7 8
8 36
9 28
G-RIB:LEU-S2
3 0
4 0
5 28
6 44
7 56
8 56
9 148

U34-P:TYR-CA
3 0
4 0
5 0
6 0
7 0
8 0
9 6
U-P:SER-S1
3 0
4 15
5 33
6 21
7 69
8 81
9 96
U31-MY:VAL-S1
3 0
4 0
5 0
6 0
7 0
8 3
9 0
G-R5:LYS-CA
3 0
4 4
5 4
6 16
7 43
8 100
9 248
U31-MY:ASP-CA
3 0
4 0
5 0
6 6
7 0
8 3
9 0
GTP-RIB:ARG-CA
3 0
4 0
5 0
6 0
7 0
8 3
9 0
GTP-M5:ASN-S1
3 0
4 0
5 0
6 0
7 0
8 0
9 3
G-R6:ILE-CA
3 0

4 0
5 0
6 4
7 4
8 16
9 55
A-R5:VAL-CA
3 0
4 0
5 4
6 16
7 44
8 28
9 76
A-RIB:TRP-S2
3 0
4 0
5 8
6 12
7 32
8 12
9 44
G-R6:PRO-S1
3 0
4 0
5 16
6 40
7 40
8 59
9 72
A-R5:VAL-S1
3 0
4 4
5 16
6 24
7 36
8 48
9 84
H2U-P:PRO-S1
3 0
4 0
5 0
6 0
7 0
8 0
9 3
QUO-M5:ASP-CA
3 0
4 0
5 0
6 0
7 0
8 0
9 8
U31-P:MET-CA
3 0
4 0
5 0

6 3
7 0
8 3
9 0
QUO-M6:LYS-S2
3 0
4 0
5 0
6 0
7 0
8 0
9 4
G-RIB:GLN-S2
3 0
4 4
5 64
6 59
7 84
8 104
9 124
U-RIB:ASN-S2
3 0
4 3
5 21
6 39
7 45
8 92
9 87
G-R6:SER-CA
3 0
4 0
5 4
6 24
7 48
8 111
9 84
H2U-MY:LYS-S1
3 0
4 0
5 0
6 0
7 0
8 0
9 3
G-RIB:GLU-CA
3 0
4 0
5 12
6 8
7 28
8 68
9 143
QUO-M5:ARG-CA
3 0
4 0
5 0
6 0
7 0

8   0
9   4
C-RIB:HIS-S1
3   0
4   6
5   12
6   30
7   21
8   57
9   75
DA-M6:GLN-S1
3   0
4   0
5   0
6   0
7   0
8   0
9   3
A-R5:TYR-S1
3   0
4   0
5   24
6   8
7   24
8   60
9   55
QUO-M6:LEU-S2
3   0
4   0
5   0
6   4
7   0
8   0
9   4
G-R6:ARG-S1
3   0
4   8
5   4
6   28
7   100
8   124
9   231
G-R6:PRO-CA
3   0
4   0
5   4
6   32
7   24
8   76
9   71
C-RIB:PHE-S2
3   0
4   0
5   9
6   18
7   33
8   24
9   24

C31-MY:GLN-S2
3   0
4   0
5   0
6   0
7   3
8   0
9   0
G-R6:MET-CA
3   0
4   0
5   4
6   8
7   20
8   40
9   28
FMU-RIB:ARG-S1
3   0
4   0
5   0
6   3
7   0
8   0
9   3
U31-RIB:ASP-S2
3   0
4   0
5   0
6   6
7   3
8   3
9   3
C-Y:MET-CA
3   0
4   0
5   3
6   0
7   12
8   9
9   24
QUO-M6:LYS-CA
3   0
4   0
5   0
6   0
7   0
8   0
9   4
A-R6:LEU-CA
3   0
4   0
5   4
6   16
7   36
8   84
9   79
A-R5:PHE-S1
3   0

4 0
5 8
6 8
7 8
8 8
9 48
C-Y:ASN-S1
3 0
4 0
5 3
6 12
7 30
8 45
9 104
U31-MY:ILE-CA
3 0
4 0
5 0
6 0
7 3
8 0
9 0
U-Y:GLN-S2
3 0
4 0
5 12
6 44
7 30
8 36
9 59
U-Y:ASN-CA
3 0
4 0
5 3
6 3
7 18
8 45
9 83
A-P:HIS-S2
3 0
4 0
5 28
6 44
7 60
8 76
9 80
U31-P:GLN-S2
3 0
4 0
5 3
6 0
7 0
8 0
9 0
DA-M5:ASN-S2
3 0
4 0
5 0

6 0
7 3
8 0
9 0
A-RIB:HIS-S2
3 0
4 0
5 20
6 64
7 76
8 64
9 104
C-Y:LYS-S2
3 0
4 3
5 33
6 48
7 66
8 96
9 276
A-R6:HIS-CA
3 0
4 0
5 4
6 36
7 16
8 32
9 103
DA-RIB:LYS-S2
3 0
4 0
5 0
6 0
7 0
8 3
9 0
A-R6:ASP-CA
3 0
4 4
5 4
6 12
7 24
8 48
9 64
C-Y:ASP-CA
3 0
4 0
5 0
6 24
7 21
8 48
9 111
C-P:ASP-CA
3 0
4 0
5 12
6 12
7 30

8   96
9   135
C-P:ARG-S2
3   0
4   9
5   261
6   168
7   153
8   258
9   357
C-Y:GLN-CA
3   0
4   0
5   0
6   9
7   9
8   21
9   69
A-RIB:HIS-CA
3   0
4   0
5   4
6   40
7   52
8   68
9   92
DA-M6:TYR-CA
3   0
4   0
5   0
6   3
7   0
8   3
9   0
FMU-MY:GLN-CA
3   0
4   0
5   0
6   0
7   3
8   0
9   0
C-RIB:ALA-S1
3   0
4   6
5   54
6   75
7   51
8   72
9   147
IU-MY:LYS-S1
3   0
4   0
5   0
6   2
7   3
8   5
9   5

QUO-P:LEU-CA
3 0
4 0
5 0
6 0
7 0
8 0
9 8
C-RIB:LEU-S2
3 0
4 0
5 9
6 36
7 66
8 56
9 78
FHU-MY:TYR-CA
3 0
4 0
5 6
6 0
7 0
8 0
9 6
C-RIB:ARG-S2
3 0
4 6
5 54
6 180
7 204
8 297
9 448
DA-M6:LYS-S1
3 0
4 0
5 0
6 0
7 3
8 0
9 0
A-P:LEU-S1
3 0
4 0
5 12
6 24
7 24
8 52
9 88
A-RIB:LEU-S1
3 0
4 0
5 12
6 32
7 48
8 64
9 84
QUO-M6:GLU-CA
3 0

4 0
5 0
6 0
7 0
8 0
9 4
G-R5:HIS-S2
3 0
4 8
5 0
6 36
7 43
8 92
9 131
G-P:GLU-CA
3 0
4 0
5 4
6 12
7 12
8 76
9 168
IU-P:SER-CA
3 0
4 0
5 0
6 0
7 0
8 0
9 3
C-RIB:LYS-S2
3 0
4 0
5 30
6 117
7 168
8 206
9 267
U34-RIB:GLY-CA
3 0
4 0
5 0
6 0
7 0
8 3
9 0
A-R5:GLN-S1
3 0
4 0
5 4
6 24
7 20
8 44
9 92
GTP-RIB:THR-S1
3 0
4 0
5 0

6 0
7 0
8 0
9 6
C-Y:GLU-S1
3 0
4 0
5 6
6 3
7 24
8 33
9 75
C-Y:ALA-CA
3 0
4 0
5 6
6 24
7 39
8 57
9 96
A-RIB:THR-CA
3 0
4 4
5 8
6 36
7 112
8 84
9 120
U-Y:SER-CA
3 0
4 0
5 3
6 15
7 26
8 35
9 73
G-R5:THR-CA
3 0
4 0
5 0
6 12
7 32
8 28
9 116
C-Y:ARG-CA
3 0
4 0
5 0
6 24
7 57
8 98
9 96
U-RIB:GLN-CA
3 0
4 0
5 0
6 27
7 27

8   18
9   63
C31-MY:GLU-S2
3   0
4   0
5   0
6   0
7   3
8   0
9   3
QUO-M5:ASN-CA
3   0
4   0
5   0
6   0
7   0
8   4
9   0
IU-MY:THR-CA
3   0
4   0
5   0
6   0
7   2
8   3
9   0
G-P:ALA-CA
3   0
4   4
5   32
6   56
7   60
8   128
9   180
G-RIB:ASN-S1
3   0
4   0
5   22
6   72
7   107
8   120
9   144
U-P:HIS-CA
3   0
4   0
5   3
6   0
7   24
8   27
9   84
C31-P:ASN-S2
3   0
4   0
5   0
6   0
7   0
8   0
9   3

U34-P:PHE-S2
3 0
4 0
5 0
6 0
7 0
8 0
9 3
G-P:MET-S1
3 0
4 0
5 8
6 4
7 24
8 36
9 32
C31-RIB:SER-CA
3 0
4 0
5 0
6 0
7 3
8 0
9 0
U34-MY:TYR-S2
3 0
4 0
5 0
6 0
7 3
8 0
9 0
U-P:GLN-S2
3 0
4 0
5 30
6 24
7 30
8 48
9 69
U31-MY:GLU-S2
3 0
4 0
5 0
6 0
7 3
8 3
9 0
A-R5:ARG-S1
3 0
4 0
5 24
6 48
7 88
8 116
9 228
U-RIB:THR-S1
3 0

4   0
5   21
6   24
7   36
8   48
9   83
G-R5:TYR-CA
3   0
4   0
5   3
6   0
7   22
8   12
9   47
G-R6:ASP-S2
3   0
4   0
5   40
6   40
7   48
8   120
9   166
U-RIB:PRO-S1
3   0
4   0
5   9
6   27
7   36
8   39
9   87
FHU-P:TYR-S1
3   0
4   0
5   0
6   0
7   0
8   3
9   0
OMC-RIB:LYS-S2
3   0
4   0
5   0
6   0
7   3
8   3
9   0
U-Y:LYS-S1
3   0
4   0
5   12
6   12
7   35
8   60
9   131
G-R6:GLN-CA
3   0
4   0
5   0

6 4
7 8
8 32
9 100
C-P:PHE-S1
3 0
4 0
5 0
6 9
7 12
8 33
9 30
H2U-RIB:GLU-S1
3 0
4 0
5 0
6 0
7 0
8 3
9 0
A-RIB:ILE-S1
3 0
4 0
5 12
6 8
7 36
8 40
9 52
C31-MY:THR-S1
3 0
4 0
5 0
6 0
7 0
8 0
9 3
U31-MY:TYR-S1
3 0
4 0
5 0
6 0
7 0
8 3
9 0
G-R5:ILE-CA
3 0
4 0
5 0
6 8
7 0
8 32
9 39
A-R6:ALA-S1
3 0
4 8
5 12
6 32
7 8

8   52
9   104
FHU-MY:GLN-S2
3   0
4   0
5   0
6   0
7   0
8   0
9   6
A-P:ARG-S1
3   0
4   0
5   108
6   172
7   124
8   328
9   368
U-P:THR-S1
3   0
4   3
5   33
6   18
7   33
8   69
9   63
C-Y:ASN-S2
3   0
4   0
5   6
6   39
7   24
8   26
9   144
U-P:ASP-S1
3   0
4   3
5   9
6   18
7   21
8   40
9   66
A-R6:ALA-CA
3   0
4   4
5   16
6   8
7   32
8   32
9   124
U-Y:HIS-S1
3   0
4   6
5   3
6   6
7   12
8   24
9   27

C-RIB:GLU-CA
3 0
4 0
5 6
6 12
7 30
8 66
9 113
U31-RIB:MET-S1
3 0
4 0
5 0
6 3
7 0
8 0
9 3
H2U-MY:LEU-S2
3 0
4 0
5 0
6 0
7 3
8 0
9 0
U-RIB:HIS-CA
3 0
4 0
5 6
6 12
7 27
8 27
9 48
DA-M6:ASN-S2
3 0
4 0
5 0
6 3
7 0
8 0
9 0
OMC-P:LYS-CA
3 0
4 0
5 0
6 0
7 0
8 0
9 3
H2U-RIB:LYS-S2
3 0
4 0
5 0
6 3
7 0
8 0
9 0
U31-P:ARG-CA
3 0

4   0
5   0
6   0
7   0
8   0
9   3
G-RIB:LYS-S1
3   0
4   0
5   24
6   88
7   195
8   231
9   332
G-P:THR-S1
3   0
4   16
5   64
6   44
7   68
8   56
9   136
C-P:HIS-S1
3   0
4   0
5   9
6   15
7   30
8   72
9   45
5BU-P:PRO-CA
3   0
4   0
5   3
6   0
7   0
8   0
9   0
G-RIB:SER-CA
3   0
4   0
5   12
6   76
7   96
8   127
9   184
G-RIB:VAL-CA
3   0
4   0
5   4
6   16
7   44
8   68
9   87
U-P:ARG-S1
3   0
4   0
5   42

6 72
7 78
8 201
9 168
A-R5:ILE-CA
3 0
4 0
5 0
6 4
7 8
8 28
9 48
U-Y:ARG-S2
3 0
4 21
5 24
6 77
7 78
8 60
9 156
G-P:MET-S2
3 0
4 0
5 16
6 16
7 24
8 32
9 20
DA-M6:SER-S1
3 0
4 0
5 3
6 0
7 0
8 0
9 0
DA-RIB:ASN-S1
3 0
4 0
5 0
6 3
7 0
8 0
9 0
C-P:GLY-CA
3 0
4 15
5 99
6 39
7 111
8 228
9 219
A-P:GLN-S1
3 0
4 0
5 20
6 32
7 28

8 76
9 124
FHU-MY:LEU-S2
3 0
4 0
5 6
6 0
7 6
8 0
9 0
C-RIB:ASN-S2
3 0
4 3
5 39
6 24
7 63
8 87
9 125
C31-P:PHE-S2
3 0
4 0
5 0
6 0
7 0
8 6
9 0
A-RIB:CYS-S1
3 0
4 0
5 8
6 4
7 4
8 20
9 12
H2U-MY:PRO-CA
3 0
4 0
5 0
6 0
7 0
8 3
9 6
C31-P:GLN-CA
3 0
4 0
5 0
6 0
7 0
8 3
9 0
G-RIB:PHE-CA
3 0
4 0
5 4
6 28
7 20
8 68
9 59

H2U-RIB:GLU-CA
3 0
4 0
5 0
6 0
7 0
8 0
9 3
IU-MY:VAL-S1
3 0
4 0
5 0
6 0
7 0
8 0
9 2
U31-MY:GLN-CA
3 0
4 0
5 0
6 0
7 0
8 0
9 3
U34-MY:ASN-S1
3 0
4 0
5 0
6 0
7 0
8 3
9 3
A-RIB:MET-S1
3 0
4 0
5 0
6 24
7 24
8 24
9 60
G-RIB:HIS-S1
3 0
4 4
5 24
6 43
7 44
8 87
9 83
G-P:VAL-S1
3 0
4 0
5 8
6 28
7 44
8 96
9 104
A-P:GLU-S2
3 0

4 0
5 0
6 24
7 80
8 92
9 132
U-RIB:ASN-S1
3 0
4 3
5 9
6 33
7 45
8 63
9 92
DA-RIB:GLN-S1
3 0
4 0
5 0
6 0
7 0
8 0
9 6
G-RIB:GLY-CA
3 0
4 8
5 104
6 124
7 168
8 215
9 315
A-RIB:PHE-CA
3 0
4 0
5 4
6 12
7 20
8 64
9 60
DA-RIB:GLN-S2
3 0
4 0
5 0
6 0
7 0
8 3
9 0
G-R5:PHE-S2
3 0
4 8
5 0
6 8
7 11
8 24
9 40
G-R5:MET-CA
3 0
4 0
5 4

6 4
7 12
8 28
9 48
DA-RIB:ASP-CA
3 0
4 0
5 0
6 3
7 0
8 0
9 0
C-RIB:GLN-CA
3 0
4 0
5 3
6 21
7 72
8 75
9 87
U-RIB:MET-S1
3 0
4 0
5 12
6 3
7 15
8 27
9 12
A-R6:CYS-CA
3 0
4 0
5 4
6 0
7 4
8 8
9 8
A-P:PRO-CA
3 0
4 0
5 8
6 20
7 52
8 132
9 120
U31-RIB:ASP-S1
3 0
4 0
5 0
6 3
7 3
8 6
9 0
G-R5:SER-S1
3 0
4 0
5 27
6 28
7 52

8 108
9 152
U-RIB:ARG-S2
3 0
4 0
5 42
6 123
7 111
8 149
9 216
U-RIB:LEU-CA
3 0
4 0
5 6
6 21
7 12
8 24
9 69
C-RIB:MET-CA
3 0
4 0
5 6
6 27
7 9
8 24
9 36
DA-M5:LEU-S1
3 0
4 0
5 0
6 0
7 0
8 0
9 3
FHU-P:TYR-S2
3 0
4 0
5 0
6 0
7 0
8 9
9 3
A-R6:HIS-S2
3 0
4 24
5 12
6 36
7 48
8 56
9 68
C-RIB:THR-CA
3 0
4 0
5 9
6 36
7 84
8 87
9 111

H2U-RIB:ARG-S1
3 0
4 0
5 0
6 0
7 0
8 0
9 3
FHU-P:VAL-CA
3 0
4 0
5 0
6 0
7 0
8 0
9 6
A-R6:PHE-CA
3 0
4 0
5 4
6 8
7 4
8 32
9 36
C-P:MET-S2
3 0
4 0
5 6
6 15
7 30
8 42
9 27
G-RIB:GLN-CA
3 0
4 0
5 8
6 48
7 32
8 115
9 144
5BU-MY:PRO-S1
3 0
4 0
5 0
6 0
7 0
8 0
9 3
U-P:ASN-S2
3 0
4 3
5 33
6 36
7 36
8 45
9 99
U34-RIB:ASN-S2
3 0

4 0
5 0
6 6
7 0
8 3
9 0
A-P:ASN-S1
3 0
4 8
5 32
6 16
7 56
8 96
9 144
G-R5:GLU-CA
3 0
4 0
5 4
6 0
7 20
8 54
9 59
G-R5:LEU-S2
3 0
4 0
5 28
6 24
7 4
8 48
9 96
U-P:MET-S2
3 0
4 0
5 0
6 12
7 18
8 30
9 21
G-RIB:LYS-S2
3 0
4 0
5 56
6 144
7 280
8 327
9 415
C31-P:ASP-S1
3 0
4 0
5 0
6 3
7 3
8 0
9 3
A-R5:ARG-CA
3 0
4 0
5 0

6 28
7 60
8 88
9 244
FHU-MY:ASP-S2
3 0
4 0
5 6
6 0
7 0
8 0
9 3
C-P:TYR-S2
3 0
4 0
5 9
6 30
7 30
8 24
9 60
H2U-P:GLU-S1
3 0
4 0
5 0
6 0
7 0
8 0
9 6
A-R5:MET-CA
3 0
4 0
5 4
6 8
7 20
8 16
9 56
U34-P:GLU-CA
3 0
4 0
5 0
6 0
7 0
8 0
9 3
DA-RIB:HIS-S1
3 0
4 0
5 0
6 3
7 0
8 3
9 0
A-R6:TYR-CA
3 0
4 0
5 4
6 20
7 24

8   47
9   68
A-P:HIS-CA
3   0
4   0
5   4
6   20
7   20
8   76
9   92
H2U-MY:ARG-CA
3   0
4   0
5   0
6   0
7   0
8   3
9   0
U31-MY:ALA-S1
3   0
4   0
5   0
6   0
7   0
8   3
9   3
U-Y:ASP-CA
3   0
4   0
5   0
6   3
7   9
8   21
9   36
G-P:ARG-S1
3   0
4   0
5   100
6   156
7   184
8   428
9   432
G-R5:ASN-CA
3   0
4   0
5   7
6   16
7   49
8   68
9   113
DA-RIB:TYR-S1
3   0
4   0
5   0
6   0
7   0
8   0
9   3

A-R5:GLN-CA
3 0
4 0
5 4
6 4
7 12
8 48
9 72
C-Y:PHE-S2
3 0
4 6
5 3
6 6
7 15
8 15
9 27
U-P:PRO-S1
3 0
4 0
5 21
6 27
7 30
8 72
9 66
A-R5:PRO-S1
3 0
4 4
5 16
6 16
7 44
8 68
9 115
FMU-MY:MET-S1
3 0
4 0
5 0
6 0
7 0
8 3
9 0
5BU-P:THR-CA
3 0
4 0
5 0
6 0
7 0
8 0
9 3
U-Y:GLU-CA
3 0
4 0
5 0
6 6
7 6
8 6
9 42
U-Y:PRO-S1
3 0

4 6
5 6
6 9
7 30
8 33
9 54
U-Y:VAL-CA
3 0
4 0
5 0
6 6
7 9
8 33
9 42
C-RIB:ASP-S2
3 0
4 3
5 45
6 60
7 42
8 96
9 135
A-R5:HIS-S1
3 0
4 4
5 8
6 28
7 32
8 56
9 52
C31-MY:GLN-S1
3 0
4 0
5 0
6 0
7 0
8 0
9 3
DA-RIB:MET-S1
3 0
4 0
5 0
6 0
7 3
8 0
9 0
G-P:LEU-CA
3 0
4 0
5 20
6 16
7 32
8 84
9 172
A-P:MET-S2
3 0
4 0
5 4

6   20
7   24
8   24
9   40
U-Y:PRO-CA
3   0
4   0
5   6
6   9
7   21
8   39
9   51
U-RIB:GLY-CA
3   0
4   6
5   45
6   63
7   86
8   121
9   183
H2U-RIB:ASN-S2
3   0
4   0
5   0
6   0
7   0
8   6
9   3
U-RIB:GLU-CA
3   0
4   3
5   3
6   6
7   21
8   18
9   63
G-P:GLN-S2
3   0
4   0
5   36
6   60
7   80
8   124
9   156
A-R5:TRP-CA
3   0
4   0
5   0
6   4
7   4
8   8
9   40
I-RIB:TRP-S1
3   0
4   0
5   0
6   0
7   0

8 0
9 2
IU-P:LEU-CA
3 0
4 0
5 0
6 0
7 0
8 3
9 3
DA-M5:VAL-S1
3 0
4 0
5 0
6 0
7 0
8 0
9 3
A-P:GLU-S1
3 0
4 0
5 4
6 8
7 28
8 76
9 156
IU-P:ALA-CA
3 0
4 0
5 0
6 0
7 0
8 0
9 3
U31-P:ASN-S1
3 0
4 0
5 0
6 3
7 0
8 0
9 0
H2U-MY:PHE-CA
3 0
4 0
5 0
6 0
7 3
8 0
9 0
U-P:GLU-S2
3 0
4 0
5 0
6 15
7 54
8 54
9 96

G-R5:ARG-S1
3   0
4   4
5   12
6   44
7   72
8   136
9   288
U-RIB:VAL-CA
3   0
4   0
5   0
6   9
7   44
8   24
9   44
U-RIB:ILE-CA
3   0
4   0
5   0
6   0
7   18
8   17
9   48
C-P:ALA-S1
3   0
4   9
5   45
6   33
7   81
8   123
9   147
C-RIB:TRP-S1
3   0
4   0
5   8
6   6
7   12
8   23
9   18
G-R5:LEU-CA
3   0
4   0
5   4
6   12
7   16
8   72
9   84
C-Y:TRP-S1
3   0
4   0
5   5
6   8
7   6
8   9
9   15
G-R5:LYS-S1
3   0

```
4   8
5   32
6   24
7   80
8   119
9   275
A-R6:ASP-S1
3   0
4   4
5   8
6   24
7   12
8   56
9   96
U31-P:ASN-CA
3   0
4   0
5   0
6   0
7   3
8   0
9   0
H2U-P:ASN-S2
3   0
4   0
5   0
6   3
7   6
8   0
9   0
C-P:ASP-S1
3   0
4   0
5   21
6   15
7   39
8   126
9   153
DA-M5:ASP-S2
3   0
4   0
5   0
6   0
7   0
8   0
9   3
A-P:ILE-S1
3   0
4   0
5   4
6   20
7   32
8   40
9   64
G-R5:VAL-S1
3   0
4   0
5   8
```

6 12
7 16
8 36
9 78
U31-MY:ASP-S2
3 0
4 6
5 0
6 0
7 0
8 3
9 3
A-RIB:ARG-S2
3 0
4 0
5 68
6 220
7 256
8 324
9 364
U-P:ILE-CA
3 0
4 0
5 0
6 3
7 15
8 12
9 51
U-P:LYS-S2
3 0
4 48
5 117
6 51
7 72
8 123
9 201
DA-M6:THR-S1
3 0
4 0
5 0
6 0
7 0
8 3
9 0
IU-MY:LEU-S1
3 0
4 0
5 0
6 0
7 2
8 0
9 0
H2U-P:ARG-S1
3 0
4 0
5 0
6 0
7 0

8 3
9 0
DA-M6:LYS-CA
3 0
4 0
5 0
6 0
7 0
8 3
9 0
U31-P:GLN-CA
3 0
4 0
5 0
6 0
7 3
8 0
9 0
G-P:TYR-S1
3 0
4 0
5 4
6 8
7 32
8 84
9 68
U31-RIB:GLN-CA
3 0
4 0
5 0
6 0
7 3
8 0
9 0
A-R5:SER-S1
3 0
4 0
5 8
6 36
7 76
8 87
9 168
A-R6:GLN-S1
3 0
4 0
5 8
6 32
7 32
8 56
9 72
U-P:TYR-S2
3 0
4 0
5 0
6 21
7 15
8 27
9 36

IU-P:LYS-S1
3 0
4 0
5 0
6 3
7 0
8 0
9 3
A-RIB:GLU-S2
3 0
4 12
5 24
6 44
7 64
8 64
9 124
A-P:TRP-CA
3 0
4 0
5 8
6 4
7 4
8 32
9 16
G-RIB:PRO-CA
3 0
4 4
5 4
6 72
7 87
8 111
9 148
FMU-MY:ARG-S2
3 0
4 0
5 0
6 0
7 0
8 3
9 0
U31-RIB:THR-S1
3 0
4 0
5 0
6 0
7 0
8 0
9 6
QUO-M6:LEU-S1
3 0
4 0
5 0
6 0
7 0
8 4
9 0
C-RIB:LEU-CA
3 0

4 0
5 6
6 24
7 30
8 102
9 140
FMU-P:ILE-CA
3 0
4 0
5 0
6 0
7 0
8 3
9 0
G-R5:MET-S1
3 0
4 4
5 0
6 8
7 28
8 20
9 40
G-R5:THR-S1
3 0
4 4
5 8
6 8
7 44
8 68
9 96
U31-RIB:ASN-S1
3 0
4 0
5 0
6 0
7 3
8 0
9 0
G-R6:HIS-S2
3 0
4 12
5 0
6 20
7 80
8 92
9 118
G-P:GLU-S1
3 0
4 0
5 8
6 8
7 36
8 128
9 144
H2U-RIB:GLY-CA
3 0
4 0
5 0

6 0
7 0
8 3
9 0
U-P:VAL-CA
3 0
4 0
5 0
6 9
7 21
8 66
9 75
C-P:LYS-CA
3 0
4 0
5 39
6 69
7 96
8 186
9 213
H2U-MY:LEU-CA
3 0
4 0
5 0
6 0
7 0
8 0
9 6
I-P:TRP-S2
3 0
4 0
5 0
6 0
7 0
8 0
9 2
C-Y:THR-S1
3 0
4 0
5 9
6 24
7 27
8 54
9 108
U-P:MET-S1
3 0
4 0
5 3
6 6
7 9
8 30
9 36
G-RIB:THR-S1
3 0
4 0
5 16
6 64
7 68

8   72
9   163
C-RIB:TYR-S1
3   0
4   0
5   6
6   15
7   23
8   51
9   45
IU-P:HIS-CA
3   0
4   0
5   3
6   0
7   0
8   0
9   0
G-R6:TRP-S2
3   0
4   8
5   12
6   8
7   20
8   16
9   36
G-RIB:LYS-CA
3   0
4   0
5   20
6   64
7   115
8   199
9   306
DA-M5:LYS-S2
3   0
4   0
5   0
6   3
7   0
8   0
9   0
G-P:LYS-CA
3   0
4   0
5   84
6   92
7   124
8   324
9   332
FMU-RIB:CYS-CA
3   0
4   0
5   0
6   0
7   3
8   0
9   0

U-RIB:ARG-S1
3 0
4 0
5 21
6 36
7 69
8 129
9 199
FMU-P:ARG-S2
3 0
4 0
5 3
6 0
7 0
8 6
9 0
G-RIB:MET-S1
3 0
4 0
5 0
6 24
7 44
8 44
9 48
U-P:ASP-S2
3 0
4 0
5 9
6 27
7 40
8 45
9 75
C-Y:TRP-S2
3 0
4 5
5 3
6 2
7 15
8 18
9 30
C31-RIB:SER-S1
3 0
4 0
5 0
6 0
7 0
8 3
9 0
C31-P:MET-S2
3 0
4 0
5 0
6 0
7 0
8 3
9 0
C-RIB:ASN-CA
3 0

4 0
5 3
6 30
7 57
8 78
9 114
U-RIB:LYS-S1
3 0
4 0
5 0
6 60
7 72
8 90
9 215
C31-RIB:TYR-S1
3 0
4 0
5 0
6 0
7 0
8 0
9 3
DA-RIB:GLN-CA
3 0
4 0
5 0
6 0
7 0
8 3
9 0
DA-M5:ASP-S1
3 0
4 0
5 0
6 0
7 0
8 0
9 3
G-P:LEU-S1
3 0
4 0
5 24
6 32
7 20
8 88
9 148
FHU-MY:TYR-S1
3 0
4 0
5 0
6 6
7 0
8 6
9 0
A-P:LYS-S1
3 0
4 8
5 72

6 136
7 156
8 176
9 264
C31-P:GLN-S1
3 0
4 0
5 0
6 0
7 0
8 3
9 0
C-P:THR-CA
3 0
4 3
5 18
6 21
7 57
8 96
9 126
G-P:ASN-S2
3 0
4 0
5 64
6 56
7 100
8 148
9 184
A-P:ASP-S1
3 0
4 0
5 4
6 20
7 36
8 96
9 140
G-RIB:ALA-S1
3 0
4 8
5 36
6 56
7 44
8 100
9 215
A-R6:GLU-S2
3 0
4 0
5 20
6 20
7 88
8 60
9 80
G-P:TRP-S1
3 0
4 0
5 8
6 16
7 16

8   32
9   20
A-P:VAL-S1
3   0
4   0
5   36
6   28
7   40
8   44
9   132
C-P:LEU-S2
3   0
4   0
5   6
6   24
7   63
8   33
9   117
A-RIB:GLU-S1
3   0
4   0
5   16
6   44
7   36
8   64
9   116
G-P:TYR-S2
3   0
4   0
5   12
6   56
7   48
8   28
9   80
FHU-RIB:ASP-CA
3   0
4   0
5   0
6   0
7   6
8   0
9   0
C31-MY:LEU-CA
3   0
4   0
5   0
6   3
7   0
8   0
9   0
C-Y:ALA-S1
3   0
4   3
5   18
6   18
7   45
8   84
9   105

U-RIB:PRO-CA
3 0
4 0
5 0
6 18
7 27
8 81
9 54
FMU-MY:ASP-CA
3 0
4 0
5 0
6 0
7 0
8 3
9 0
U-Y:LEU-S1
3 0
4 0
5 0
6 9
7 27
8 27
9 51
C-Y:ARG-S2
3 0
4 9
5 75
6 84
7 93
8 177
9 305
U-Y:THR-CA
3 0
4 0
5 6
6 18
7 18
8 12
9 43
A-R5:CYS-S1
3 0
4 0
5 4
6 4
7 8
8 12
9 4
FHU-RIB:TYR-S1
3 0
4 0
5 0
6 3
7 3
8 0
9 0
C-P:TYR-S1
3 0

4 0
5 3
6 6
7 21
8 45
9 66
4SU-P:GLU-S2
3 0
4 0
5 0
6 0
7 0
8 0
9 3
GTP-M6:GLY-CA
3 0
4 0
5 0
6 0
7 0
8 3
9 0
H2U-MY:GLU-S2
3 0
4 0
5 0
6 3
7 0
8 0
9 0
FHU-RIB:ARG-S1
3 0
4 0
5 0
6 0
7 0
8 3
9 9
DA-M6:HIS-S2
3 0
4 0
5 3
6 0
7 0
8 0
9 0
FMU-MY:VAL-CA
3 0
4 0
5 0
6 0
7 0
8 3
9 3
C31-P:MET-CA
3 0
4 0
5 0

6 0
7 0
8 0
9 3
DA-RIB:ARG-S1
3 0
4 0
5 0
6 0
7 0
8 0
9 3
C31-MY:GLU-CA
3 0
4 0
5 0
6 0
7 0
8 3
9 0
IU-RIB:ARG-S2
3 0
4 0
5 0
6 0
7 0
8 3
9 0
A-R6:SER-CA
3 0
4 0
5 4
6 40
7 36
8 76
9 88
H2U-MY:TRP-S2
3 0
4 0
5 3
6 0
7 0
8 0
9 0
A-R6:LEU-S2
3 0
4 8
5 12
6 56
7 28
8 72
9 92
G-P:ILE-CA
3 0
4 0
5 12
6 0
7 20

8 76
9 96
C-Y:PRO-CA
3 0
4 0
5 6
6 12
7 30
8 41
9 90
FHU-P:SER-S1
3 0
4 0
5 0
6 0
7 0
8 3
9 0
FMU-P:PHE-CA
3 0
4 0
5 0
6 3
7 0
8 0
9 0
C31-P:GLU-S2
3 0
4 0
5 3
6 0
7 0
8 0
9 0
G-R5:ASN-S1
3 0
4 0
5 27
6 23
7 47
8 75
9 159
G-RIB:MET-S2
3 0
4 0
5 32
6 20
7 24
8 52
9 44
QUO-M5:LYS-S2
3 0
4 0
5 0
6 0
7 0
8 0
9 4

C-P:MET-S1
3 0
4 0
5 6
6 9
7 15
8 51
9 45
U31-RIB:ASP-CA
3 0
4 0
5 0
6 0
7 6
8 3
9 3
GTP-RIB:ALA-S1
3 0
4 0
5 0
6 0
7 0
8 0
9 3
FHU-RIB:ALA-CA
3 0
4 0
5 0
6 3
7 0
8 0
9 6
G-R6:ARG-S2
3 0
4 4
5 16
6 136
7 166
8 188
9 231
A-P:TYR-S2
3 0
4 0
5 8
6 52
7 44
8 56
9 68
A-P:ALA-S1
3 0
4 4
5 56
6 68
7 80
8 72
9 144
U31-RIB:TYR-S2
3 0

4 0
5 0
6 0
7 3
8 0
9 3
U-Y:LYS-CA
3 0
4 0
5 9
6 12
7 11
8 54
9 69
G-P:MET-CA
3 0
4 0
5 4
6 8
7 12
8 44
9 56
QUO-RIB:LYS-S2
3 0
4 0
5 0
6 0
7 0
8 4
9 0
C-P:PRO-S1
3 0
4 3
5 36
6 36
7 42
8 84
9 105
G-R6:ARG-CA
3 0
4 4
5 12
6 28
7 28
8 96
9 152
U-P:LEU-S1
3 0
4 0
5 0
6 6
7 27
8 45
9 78
A-R5:PHE-CA
3 0
4 0
5 0

6	12
7	8
8	8
9	52
C-Y:PRO-S1	
3	0
4	3
5	6
6	24
7	27
8	41
9	87
U-RIB:TRP-S2	
3	0
4	0
5	3
6	12
7	9
8	18
9	12
U-Y:ASN-S2	
3	0
4	6
5	6
6	32
7	45
8	51
9	96
G-RIB:ILE-CA	
3	0
4	0
5	8
6	16
7	24
8	72
9	67
U31-P:MET-S2	
3	0
4	0
5	0
6	3
7	0
8	0
9	3
C-RIB:ARG-S1	
3	0
4	0
5	36
6	90
7	141
8	246
9	342
C-Y:HIS-S1	
3	0
4	0
5	3
6	21
7	21

8 36
9 72
G-P:SER-S1
3 0
4 16
5 68
6 56
7 92
8 152
9 248
DA-M5:MET-S2
3 0
4 0
5 0
6 0
7 0
8 3
9 0
G-R5:PHE-S1
3 0
4 0
5 0
6 8
7 16
8 12
9 51
C31-RIB:LEU-S1
3 0
4 0
5 0
6 0
7 3
8 0
9 0
U-Y:CYS-S1
3 0
4 0
5 0
6 3
7 0
8 5
9 15
U-Y:LEU-S2
3 0
4 0
5 3
6 15
7 42
8 18
9 36
U-RIB:HIS-S1
3 0
4 0
5 9
6 18
7 27
8 42
9 45

U-RIB:TYR-S1
3 0
4 0
5 0
6 6
7 18
8 32
9 36
G-R6:GLN-S2
3 0
4 0
5 28
6 44
7 88
8 84
9 83
C-RIB:GLU-S1
3 0
4 3
5 12
6 24
7 42
8 54
9 101
U-Y:HIS-CA
3 0
4 0
5 0
6 9
7 15
8 15
9 24
U-P:ALA-CA
3 0
4 0
5 27
6 36
7 30
8 84
9 129
A-RIB:ASN-S1
3 0
4 0
5 28
6 40
7 72
8 72
9 148
U-RIB:MET-CA
3 0
4 0
5 9
6 12
7 15
8 15
9 17
IU-MY:GLU-S2
3 0

4 0
5 0
6 0
7 0
8 2
9 0
C-P:HIS-S2
3 0
4 0
5 12
6 45
7 42
8 45
9 69
G-R5:PHE-CA
3 0
4 0
5 0
6 0
7 8
8 20
9 60
C-Y:LEU-S1
3 0
4 0
5 3
6 15
7 18
8 27
9 81
FMU-RIB:GLN-CA
3 0
4 0
5 0
6 0
7 0
8 3
9 0
IU-MY:ARG-S2
3 0
4 0
5 3
6 0
7 3
8 0
9 6
C-Y:ARG-S1
3 0
4 0
5 12
6 42
7 90
8 131
9 258
IU-MY:HIS-CA
3 0
4 0
5 0

6 0
7 0
8 0
9 5
A-R6:ASN-S1
3 0
4 0
5 16
6 28
7 55
8 56
9 64
C-Y:GLN-S2
3 0
4 0
5 9
6 36
7 57
8 69
9 84
H2U-MY:ASN-CA
3 0
4 0
5 0
6 3
7 0
8 0
9 3
G-P:SER-CA
3 0
4 4
5 44
6 60
7 108
8 148
9 204
G-R5:MET-S2
3 0
4 4
5 4
6 20
7 24
8 48
9 40
A-R6:ARG-S2
3 0
4 36
5 60
6 80
7 108
8 156
9 296
G-P:HIS-CA
3 0
4 4
5 0
6 16
7 48

8 80
9 128
IU-RIB:PRO-CA
3 0
4 0
5 0
6 0
7 0
8 3
9 2
QUO-M5:GLN-S2
3 0
4 0
5 0
6 0
7 0
8 4
9 0
IU-RIB:SER-CA
3 0
4 0
5 0
6 0
7 2
8 2
9 0
U-Y:GLU-S2
3 0
4 0
5 9
6 12
7 9
8 21
9 57
A-R6:PRO-S1
3 0
4 0
5 24
6 56
7 24
8 95
9 92
FMU-RIB:ALA-S1
3 0
4 0
5 0
6 0
7 0
8 3
9 0
C-Y:GLU-S2
3 0
4 0
5 3
6 18
7 33
8 63
9 111

A-P:LYS-S2
3   0
4   60
5   176
6   112
7   132
8   220
9   292
A-P:THR-S1
3   0
4   4
5   60
6   48
7   68
8   144
9   132
QUO-M6:ASN-CA
3   0
4   0
5   0
6   0
7   4
8   0
9   0
DA-M6:LEU-S2
3   0
4   0
5   0
6   0
7   0
8   0
9   6
FMU-MY:GLU-CA
3   0
4   0
5   0
6   0
7   0
8   3
9   0
U34-P:TYR-S1
3   0
4   0
5   0
6   0
7   0
8   0
9   3
U34-P:GLU-S1
3   0
4   0
5   0
6   0
7   0
8   3
9   0
U-RIB:ASP-CA
3   0

4 0
5 6
6 12
7 24
8 48
9 63
A-R5:LEU-CA
3 0
4 0
5 0
6 12
7 28
8 56
9 76
A-R6:LYS-S2
3 0
4 0
5 36
6 80
7 88
8 112
9 168
H2U-MY:ARG-S1
3 0
4 0
5 0
6 3
7 0
8 0
9 0
A-RIB:SER-CA
3 0
4 0
5 20
6 32
7 80
8 80
9 168
DA-M5:TYR-S1
3 0
4 0
5 0
6 0
7 0
8 6
9 0
C31-RIB:LEU-S2
3 0
4 0
5 0
6 3
7 0
8 0
9 0
A-R5:THR-CA
3 0
4 0
5 0

6 28
7 36
8 68
9 120
DA-M5:GLU-S1
3 0
4 0
5 0
6 0
7 0
8 0
9 3
GTP-RIB:ASN-S2
3 0
4 0
5 0
6 3
7 0
8 0
9 0
C31-P:THR-S1
3 0
4 0
5 0
6 0
7 0
8 6
9 0
G-RIB:HIS-S2
3 0
4 0
5 32
6 71
7 67
8 110
9 104
FMU-P:ASP-S2
3 0
4 0
5 0
6 0
7 0
8 0
9 3
C31-MY:PHE-S2
3 0
4 0
5 3
6 0
7 0
8 0
9 0
U31-P:LEU-S1
3 0
4 0
5 0
6 0
7 0

8   0
9   3
C-Y:ILE-CA
3   0
4   0
5   0
6   3
7   6
8   21
9   69
U-Y:ARG-S1
3   0
4   0
5   21
6   36
7   36
8   71
9   105
G-R6:LYS-S1
3   0
4   8
5   24
6   16
7   68
8   128
9   168
FMU-P:CYS-S1
3   0
4   0
5   0
6   0
7   0
8   0
9   3
C31-P:THR-CA
3   0
4   0
5   0
6   0
7   3
8   0
9   3
C31-MY:PHE-S1
3   0
4   0
5   3
6   0
7   0
8   0
9   3
U-Y:VAL-S1
3   0
4   0
5   6
6   9
7   21
8   20
9   24

C-Y:TYR-S2
3 0
4 3
5 6
6 6
7 30
8 24
9 36
U31-RIB:GLN-S2
3 0
4 0
5 0
6 0
7 3
8 0
9 0
G-R5:TYR-S1
3 0
4 0
5 0
6 14
7 11
8 32
9 27
DA-M6:GLN-CA
3 0
4 0
5 0
6 0
7 0
8 3
9 0
C-RIB:VAL-CA
3 0
4 0
5 9
6 24
7 69
8 105
9 108
U31-RIB:GLU-CA
3 0
4 0
5 0
6 0
7 0
8 0
9 3
C-Y:LEU-S2
3 0
4 0
5 12
6 9
7 24
8 27
9 66
C-RIB:ILE-S1
3 0

4 0
5 9
6 30
7 30
8 24
9 57
G-P:ARG-S2
3 0
4 4
5 372
6 172
7 232
8 308
9 436
C-P:ASN-CA
3 0
4 3
5 9
6 36
7 60
8 84
9 129
G-R5:TRP-S2
3 0
4 0
5 12
6 12
7 4
8 48
9 16
A-R6:CYS-S1
3 0
4 0
5 0
6 12
7 4
8 8
9 0
QUO-M5:LEU-S1
3 0
4 0
5 0
6 0
7 4
8 0
9 4
U-P:ASN-S1
3 0
4 6
5 24
6 27
7 30
8 75
9 51
H2U-MY:GLN-S2
3 0
4 0
5 0

6   3
7   0
8   0
9   0
A-P:PHE-CA
3   0
4   0
5   0
6   16
7   24
8   36
9   68
C31-P:TYR-S1
3   0
4   0
5   0
6   0
7   0
8   3
9   3
A-R6:LEU-S1
3   0
4   0
5   8
6   40
7   40
8   52
9   112
G-RIB:HIS-CA
3   0
4   0
5   4
6   28
7   63
8   60
9   91
G-R6:LEU-S1
3   0
4   4
5   4
6   20
7   24
8   48
9   56
FMU-MY:CYS-CA
3   0
4   0
5   3
6   0
7   0
8   0
9   0
C31-MY:TYR-S2
3   0
4   0
5   0
6   0
7   0

8   3
9   3
QUO-M5:LYS-S1
3   0
4   0
5   0
6   0
7   0
8   0
9   4
C-Y:MET-S2
3   0
4   0
5   3
6   6
7   12
8   30
9   48
U-RIB:LEU-S1
3   0
4   0
5   12
6   15
7   21
8   33
9   39
A-R5:GLN-S2
3   0
4   0
5   16
6   24
7   44
8   76
9   136
A-R6:GLU-CA
3   0
4   0
5   16
6   20
7   20
8   44
9   104
A-R5:MET-S1
3   0
4   4
5   4
6   12
7   16
8   36
9   24
G-R5:ASP-CA
3   0
4   0
5   0
6   8
7   8
8   44
9   147

G-RIB:TYR-S1
3 0
4 0
5 4
6 8
7 19
8 55
9 54
IU-RIB:LYS-CA
3 0
4 0
5 3
6 0
7 0
8 4
9 5
C-RIB:HIS-CA
3 0
4 0
5 12
6 24
7 30
8 39
9 57
C-RIB:GLN-S2
3 0
4 0
5 45
6 63
7 75
8 69
9 141
G-R5:ILE-S1
3 0
4 4
5 4
6 4
7 0
8 19
9 36
U-RIB:ILE-S1
3 0
4 0
5 3
6 17
7 15
8 27
9 33
FHU-P:TYR-CA
3 0
4 0
5 0
6 0
7 0
8 3
9 6
U31-P:ASP-CA
3 0

4 3
5 0
6 0
7 0
8 3
9 0
QUO-P:LEU-S2
3 0
4 0
5 0
6 0
7 8
8 0
9 0
G-RIB:ASP-CA
3 0
4 0
5 8
6 28
7 56
8 72
9 191
G-R6:LEU-CA
3 0
4 0
5 4
6 4
7 36
8 60
9 96
A-RIB:PRO-S1
3 0
4 0
5 8
6 64
7 40
8 92
9 156
A-R5:ASP-S1
3 0
4 0
5 4
6 20
7 32
8 40
9 92
A-P:TYR-S1
3 0
4 0
5 12
6 8
7 28
8 60
9 68
C-Y:THR-CA
3 0
4 0
5 0

6 18
7 24
8 42
9 68
DA-M6:LEU-S1
3 0
4 0
5 0
6 0
7 0
8 0
9 6
GTP-M5:SER-S1
3 0
4 0
5 0
6 0
7 0
8 3
9 3
C31-MY:SER-CA
3 0
4 0
5 0
6 0
7 0
8 3
9 0
M2G-P:GLU-CA
3 0
4 0
5 0
6 0
7 0
8 4
9 0
C-RIB:GLN-S1
3 0
4 0
5 6
6 51
7 66
8 87
9 108
QUO-RIB:ASN-CA
3 0
4 0
5 0
6 0
7 0
8 4
9 0
PSU-RIB:ARG-S2
3 0
4 0
5 0
6 0
7 0

8 0
9 3
U31-P:ARG-S1
3 0
4 0
5 0
6 0
7 0
8 0
9 3
IU-MY:VAL-CA
3 0
4 0
5 0
6 0
7 0
8 0
9 4
QUO-M5:PHE-S2
3 0
4 0
5 4
6 4
7 0
8 0
9 0
A-R5:HIS-CA
3 0
4 0
5 0
6 20
7 28
8 52
9 84
U-RIB:GLN-S2
3 0
4 3
5 24
6 24
7 41
8 54
9 72
C-Y:SER-S1
3 0
4 0
5 15
6 39
7 42
8 84
9 140
U-RIB:GLN-S1
3 0
4 0
5 6
6 27
7 33
8 36
9 65

C-Y:MET-S1
3 0
4 0
5 3
6 6
7 6
8 21
9 27
DA-M6:TYR-S1
3 0
4 0
5 0
6 0
7 6
8 0
9 0
IU-MY:LEU-CA
3 0
4 0
5 0
6 0
7 0
8 2
9 2
G-RIB:ASN-CA
3 0
4 0
5 16
6 46
7 79
8 142
9 157
G-P:CYS-CA
3 0
4 0
5 0
6 0
7 0
8 12
9 16
A-RIB:PRO-CA
3 0
4 0
5 12
6 32
7 68
8 64
9 132
G-R6:ASN-S2
3 0
4 0
5 8
6 64
7 89
8 67
9 114
M2G-P:GLY-CA
3 0

4 0
5 0
6 0
7 0
8 4
9 0
H2U-MY:PHE-S1
3 0
4 0
5 3
6 0
7 0
8 0
9 0
A-P:SER-CA
3 0
4 8
5 40
6 76
7 68
8 152
9 140
A-R5:LYS-CA
3 0
4 4
5 0
6 16
7 40
8 68
9 156
C-RIB:TRP-CA
3 0
4 0
5 0
6 5
7 17
8 24
9 12
C31-RIB:THR-CA
3 0
4 0
5 0
6 3
7 0
8 0
9 6
C-Y:TRP-CA
3 0
4 0
5 5
6 2
7 9
8 3
9 9
QUO-P:LEU-S1
3 0
4 0
5 0

6 0
7 8
8 0
9 0
QUO-M5:ASP-S2
3 0
4 0
5 0
6 0
7 4
8 4
9 0
A-R5:TRP-S1
3 0
4 0
5 0
6 4
7 4
8 8
9 48
IU-P:ASP-S2
3 0
4 0
5 0
6 0
7 0
8 3
9 0
U-RIB:TYR-S2
3 0
4 0
5 6
6 17
7 33
8 21
9 42
FHU-P:HIS-S2
3 0
4 0
5 0
6 0
7 0
8 0
9 6
U34-P:HIS-CA
3 0
4 0
5 0
6 0
7 0
8 0
9 3
C31-MY:ALA-S1
3 0
4 0
5 0
6 0
7 0

8 3
9 0
C31-P:TYR-S2
3 0
4 0
5 0
6 3
7 3
8 0
9 0
G-RIB:CYS-CA
3 0
4 0
5 4
6 0
7 4
8 8
9 12
A-R6:TRP-CA
3 0
4 0
5 0
6 0
7 12
8 12
9 20
C31-RIB:GLU-S1
3 0
4 0
5 0
6 0
7 3
8 0
9 0
G-R6:ASN-S1
3 0
4 0
5 8
6 43
7 54
8 101
9 84
C-RIB:ASN-S1
3 0
4 6
5 9
6 39
7 66
8 81
9 132
5BU-P:ARG-S2
3 0
4 0
5 3
6 0
7 0
8 0
9 0

A-RIB:LEU-S2
3 0
4 0
5 8
6 36
7 52
8 40
9 108
U-P:ASN-CA
3 0
4 3
5 21
6 15
7 27
8 51
9 69
U-RIB:SER-CA
3 0
4 0
5 12
6 21
7 74
8 50
9 84
C31-MY:GLU-S1
3 0
4 0
5 0
6 0
7 0
8 0
9 3
FHU-MY:ARG-S1
3 0
4 0
5 0
6 6
7 0
8 6
9 0
C31-P:SER-S1
3 0
4 0
5 0
6 0
7 0
8 0
9 3
U-Y:SER-S1
3 0
4 3
5 14
6 27
7 24
8 35
9 81
G-P:THR-CA
3 0

4   4
5   32
6   48
7   80
8   88
9   120
FHU-MY:LYS-S1
3   0
4   0
5   0
6   0
7   0
8   0
9   6
U-Y:TYR-S2
3   0
4   9
5   12
6   3
7   23
8   18
9   42
U-Y:PHE-S2
3   0
4   9
5   6
6   17
7   6
8   21
9   27
QUO-M5:ASN-S1
3   0
4   0
5   0
6   0
7   4
8   0
9   0
IU-MY:THR-S1
3   0
4   0
5   0
6   0
7   2
8   3
9   0
G-R6:GLY-CA
3   0
4   0
5   40
6   68
7   92
8   164
9   214
IU-MY:LYS-S2
3   0
4   0
5   0

6 5
7 5
8 5
9 0
H2U-P:THR-CA
3 0
4 0
5 0
6 0
7 0
8 0
9 6
A-RIB:VAL-CA
3 0
4 0
5 4
6 8
7 60
8 104
9 112
G-R5:PRO-CA
3 0
4 0
5 8
6 8
7 52
8 48
9 79
A-P:ARG-S2
3 0
4 0
5 300
6 120
7 220
8 340
9 360
G-R5:GLU-S2
3 0
4 4
5 16
6 19
7 75
8 72
9 119
FMU-P:PHE-S2
3 0
4 0
5 0
6 3
7 0
8 0
9 0
C-RIB:ASP-S1
3 0
4 0
5 21
6 66
7 66

8 54
9 120
U34-MY:PHE-S1
3 0
4 0
5 0
6 0
7 0
8 3
9 0
IU-P:ILE-CA
3 0
4 0
5 0
6 0
7 0
8 3
9 0
U-Y:ASN-S1
3 0
4 0
5 6
6 12
7 39
8 65
9 69
C-RIB:ARG-CA
3 0
4 0
5 21
6 42
7 99
8 207
9 242
G-R6:THR-S1
3 0
4 0
5 0
6 24
7 52
8 68
9 92
DA-M6:MET-S2
3 0
4 0
5 0
6 0
7 0
8 3
9 0
FMU-RIB:ILE-CA
3 0
4 0
5 0
6 0
7 0
8 0
9 3

FMU-P:ALA-S1
3 0
4 0
5 0
6 0
7 0
8 3
9 0
FMU-RIB:VAL-CA
3 0
4 0
5 0
6 0
7 0
8 3
9 0
FHU-P:SER-CA
3 0
4 0
5 0
6 0
7 0
8 3
9 3
U-P:LYS-S1
3 0
4 3
5 36
6 96
7 78
8 108
9 153
G-R6:PHE-CA
3 0
4 0
5 0
6 8
7 8
8 27
9 28
G-RIB:VAL-S1
3 0
4 0
5 8
6 36
7 36
8 51
9 107
U-P:SER-CA
3 0
4 0
5 27
6 24
7 72
8 66
9 96
H2U-P:TRP-S1
3 0

4 0
5 0
6 0
7 0
8 3
9 0
QUO-M6:PHE-S2
3 0
4 0
5 4
6 4
7 0
8 0
9 0
GTP-M6:ASN-S1
3 0
4 0
5 0
6 0
7 0
8 0
9 3
QUO-M6:ARG-S1
3 0
4 0
5 0
6 0
7 4
8 0
9 4
U-Y:THR-S1
3 0
4 0
5 9
6 21
7 15
8 39
9 51
C-Y:TYR-S1
3 0
4 3
5 15
6 9
7 9
8 21
9 33
A-R5:LYS-S2
3 0
4 0
5 40
6 36
7 132
8 160
9 263
U31-RIB:MET-CA
3 0
4 0
5 0

6 3
7 0
8 0
9 0
C-Y:PHE-S1
3 0
4 0
5 3
6 3
7 6
8 15
9 33
G-R5:CYS-CA
3 0
4 0
5 0
6 0
7 0
8 8
9 16
U31-MY:MET-S2
3 0
4 3
5 0
6 0
7 0
8 0
9 0
QUO-M6:ASP-S2
3 0
4 0
5 0
6 0
7 4
8 4
9 0
H2U-P:TRP-CA
3 0
4 0
5 0
6 0
7 0
8 0
9 3
FMU-RIB:PHE-CA
3 0
4 0
5 0
6 0
7 0
8 0
9 3
A-R5:TRP-S2
3 0
4 0
5 0
6 8
7 16

8   28
9   24
U-P:GLU-S1
3   0
4   0
5   9
6   12
7   21
8   60
9   60
G-RIB:ASN-S2
3   0
4   4
5   27
6   99
7   94
8   119
9   170
U31-MY:TYR-S2
3   0
4   0
5   3
6   0
7   0
8   0
9   0
C-RIB:LYS-CA
3   0
4   0
5   9
6   42
7   78
8   147
9   213
FHU-RIB:THR-CA
3   0
4   0
5   0
6   3
7   6
8   0
9   0
A-P:MET-S1
3   0
4   0
5   4
6   8
7   20
8   44
9   48
G-R6:GLN-S1
3   0
4   0
5   0
6   8
7   44
8   100
9   100

IU-P:ARG-S2
3 0
4 0
5 0
6 0
7 0
8 0
9 3
A-P:GLN-CA
3 0
4 0
5 8
6 28
7 36
8 72
9 132
A-R5:THR-S1
3 0
4 4
5 16
6 28
7 44
8 76
9 104
QUO-M5:GLU-S1
3 0
4 0
5 0
6 0
7 0
8 0
9 4
C-P:HIS-CA
3 0
4 0
5 3
6 21
7 12
8 57
9 84
U34-P:ARG-S1
3 0
4 0
5 0
6 0
7 0
8 0
9 3
FHU-P:LYS-S2
3 0
4 3
5 3
6 0
7 0
8 6
9 0
U-P:GLN-CA
3 0

4 0
5 6
6 12
7 21
8 45
9 81
IU-MY:TYR-CA
3 0
4 0
5 0
6 0
7 0
8 0
9 2
DA-M6:ASN-S1
3 0
4 0
5 0
6 0
7 3
8 0
9 3
C-RIB:THR-S1
3 0
4 0
5 24
6 63
7 60
8 102
9 120
C-RIB:MET-S1
3 0
4 3
5 12
6 24
7 21
8 30
9 27
U34-MY:VAL-CA
3 0
4 0
5 0
6 0
7 0
8 0
9 3
A-R5:MET-S2
3 0
4 0
5 28
6 4
7 28
8 36
9 40
FHU-P:LEU-S1
3 0
4 0
5 0

6 0
7 3
8 0
9 12
G-P:LYS-S2
3 0
4 60
5 228
6 164
7 304
8 304
9 436
G-R6:LYS-CA
3 0
4 0
5 8
6 20
7 28
8 100
9 163
FMU-MY:ILE-CA
3 0
4 0
5 0
6 0
7 0
8 0
9 3
FMU-MY:GLN-S2
3 0
4 0
5 3
6 0
7 0
8 0
9 0
IU-RIB:LEU-S1
3 0
4 0
5 2
6 0
7 0
8 0
9 0
5BU-MY:ARG-S2
3 0
4 0
5 0
6 0
7 0
8 0
9 3
M2G-P:GLU-S1
3 0
4 0
5 0
6 0
7 0

8 4
9 0
G-P:PRO-CA
3 0
4 0
5 24
6 36
7 88
8 104
9 216
G-R5:TYR-S2
3 0
4 4
5 10
6 8
7 20
8 40
9 74
G-R6:HIS-CA
3 0
4 0
5 4
6 16
7 24
8 20
9 99
QUO-M5:ARG-S2
3 0
4 0
5 0
6 0
7 4
8 0
9 4
G-RIB:PHE-S1
3 0
4 0
5 8
6 16
7 48
8 40
9 67
A-R5:SER-CA
3 0
4 0
5 0
6 12
7 52
8 72
9 128
U-P:PHE-CA
3 0
4 0
5 3
6 3
7 12
8 18
9 39

5BU-RIB:ARG-S2
3 0
4 0
5 0
6 0
7 0
8 0
9 3
FHU-RIB:ARG-CA
3 0
4 0
5 0
6 0
7 0
8 0
9 6
C-P:ASP-S2
3 0
4 0
5 12
6 39
7 66
8 90
9 189
G-R6:MET-S1
3 0
4 0
5 4
6 28
7 16
8 20
9 40
C-Y:VAL-CA
3 0
4 0
5 3
6 24
7 12
8 24
9 72
U-RIB:TRP-S1
3 0
4 0
5 0
6 6
7 18
8 6
9 21
GTP-M5:ASP-S1
3 0
4 0
5 0
6 0
7 0
8 0
9 3
A-R6:MET-S2
3 0

4   4
5   28
6   12
7   16
8   44
9   20
U-RIB:LYS-S2
3   0
4   3
5   24
6   39
7   137
8   182
9   180
FMU-RIB:GLU-S2
3   0
4   0
5   0
6   0
7   0
8   3
9   0
FMU-MY:PHE-CA
3   0
4   0
5   0
6   0
7   0
8   0
9   3
A-P:CYS-S1
3   0
4   0
5   12
6   0
7   0
8   0
9   24
DA-RIB:TYR-CA
3   0
4   0
5   0
6   0
7   0
8   0
9   3
G-RIB:GLU-S1
3   0
4   0
5   16
6   28
7   64
8   43
9   155
C-RIB:HIS-S2
3   0
4   6
5   15

6 42
7 36
8 75
9 63
C31-RIB:ALA-S1
3 0
4 0
5 0
6 0
7 0
8 0
9 6
U-RIB:PHE-S1
3 0
4 0
5 6
6 18
7 12
8 29
9 48
A-P:ILE-CA
3 0
4 0
5 4
6 16
7 12
8 56
9 60
C-P:ASN-S1
3 0
4 3
5 36
6 30
7 87
8 102
9 117
C-P:VAL-CA
3 0
4 0
5 6
6 21
7 60
8 108
9 138
G-R6:ASN-CA
3 0
4 0
5 0
6 26
7 58
8 54
9 99
C31-P:TYR-CA
3 0
4 0
5 0
6 0
7 0

8 0
9 6
C-P:MET-CA
3 0
4 3
5 3
6 3
7 18
8 33
9 42
A-P:TYR-CA
3 0
4 0
5 16
6 4
7 24
8 28
9 100
QUO-M6:ASN-S1
3 0
4 0
5 0
6 4
7 0
8 0
9 0
G-RIB:ARG-S1
3 0
4 0
5 20
6 116
7 147
8 260
9 416
IU-RIB:ILE-CA
3 0
4 0
5 0
6 2
7 3
8 0
9 0
DA-M6:GLU-S1
3 0
4 0
5 0
6 0
7 0
8 3
9 0
G-RIB:PHE-S2
3 0
4 0
5 8
6 32
7 47
8 36
9 44

C-P:PHE-S2
3 0
4 0
5 0
6 18
7 12
8 30
9 60
A-RIB:PHE-S1
3 0
4 0
5 8
6 12
7 32
8 40
9 52
C-P:PHE-CA
3 0
4 0
5 0
6 0
7 9
8 39
9 42
OMC-MY:LYS-S2
3 0
4 0
5 0
6 0
7 0
8 3
9 0
C31-MY:THR-CA
3 0
4 0
5 0
6 0
7 0
8 0
9 3
FHU-P:ASP-S1
3 0
4 0
5 0
6 0
7 0
8 0
9 3
QUO-M6:PHE-S1
3 0
4 0
5 0
6 0
7 4
8 0
9 4
FHU-MY:THR-S1
3 0

4 0
5 0
6 0
7 0
8 3
9 9
A-R6:TRP-S2
3 0
4 0
5 4
6 16
7 20
8 20
9 20
C31-RIB:THR-S1
3 0
4 0
5 0
6 3
7 0
8 0
9 0
H2U-P:ARG-S2
3 0
4 0
5 0
6 3
7 0
8 0
9 0
FMU-P:ASP-CA
3 0
4 0
5 0
6 0
7 0
8 3
9 0
A-P:GLU-CA
3 0
4 0
5 8
6 0
7 20
8 60
9 120
C31-MY:SER-S1
3 0
4 0
5 0
6 0
7 0
8 0
9 3
U34-P:ASN-S1
3 0
4 0
5 3

6   0
7   0
8   6
9   3
G-P:ASP-CA
3   0
4   0
5   20
6   8
7   56
8   136
9   208
A-R6:MET-S1
3   0
4   4
5   8
6   8
7   32
8   28
9   36
U-P:LEU-CA
3   0
4   0
5   0
6   3
7   27
8   48
9   63
U34-MY:PRO-CA
3   0
4   0
5   0
6   0
7   0
8   0
9   3
A-RIB:ASP-S1
3   0
4   4
5   16
6   24
7   64
8   52
9   116
C-P:GLU-S2
3   0
4   0
5   0
6   33
7   66
8   102
9   132
C-RIB:ILE-CA
3   0
4   0
5   0
6   21
7   24

8 51
9 78
FHU-MY:GLY-CA
3 0
4 0
5 0
6 0
7 0
8 0
9 6
DA-M5:TYR-CA
3 0
4 0
5 0
6 0
7 3
8 0
9 3
C-P:TYR-CA
3 0
4 0
5 3
6 0
7 33
8 18
9 75
U31-MY:THR-S1
3 0
4 0
5 0
6 3
7 0
8 3
9 3
A-R5:ALA-CA
3 0
4 0
5 8
6 8
7 36
8 44
9 100
C-Y:CYS-CA
3 0
4 0
5 0
6 3
7 0
8 0
9 6
C-P:ALA-CA
3 0
4 0
5 48
6 33
7 48
8 126
9 177

H2U-RIB:PHE-S1
3 0
4 0
5 0
6 0
7 0
8 3
9 0
H2U-MY:GLN-CA
3 0
4 0
5 0
6 0
7 0
8 0
9 3
U-Y:TRP-S2
3 0
4 3
5 0
6 3
7 3
8 0
9 24
U31-P:SER-S1
3 0
4 0
5 0
6 0
7 0
8 0
9 3
A-P:ASP-CA
3 0
4 0
5 8
6 0
7 24
8 100
9 172
A-R6:TRP-S1
3 0
4 0
5 4
6 4
7 12
8 0
9 28
G-R5:ASP-S1
3 0
4 0
5 0
6 12
7 20
8 108
9 131
U-P:CYS-CA
3 0

4 0
5 0
6 3
7 0
8 6
9 6
DA-M6:GLU-S2
3 0
4 0
5 3
6 0
7 0
8 0
9 0
U31-MY:THR-CA
3 0
4 0
5 0
6 3
7 3
8 0
9 0
FHU-RIB:ILE-S1
3 0
4 0
5 0
6 0
7 0
8 3
9 3
G-P:LYS-S1
3 0
4 8
5 80
6 248
7 204
8 272
9 304
IU-RIB:GLN-S2
3 0
4 0
5 0
6 0
7 0
8 3
9 0
U34-RIB:SER-S1
3 0
4 0
5 0
6 0
7 3
8 0
9 3
IU-MY:MET-CA
3 0
4 0
5 0

6 0
7 0
8 0
9 2
A-R6:ASN-CA
3 0
4 0
5 4
6 16
7 43
8 52
9 88
A-P:ARG-CA
3 0
4 0
5 32
6 72
7 156
8 256
9 280
U31-RIB:ASN-S2
3 0
4 0
5 0
6 0
7 0
8 3
9 0
DA-M5:HIS-CA
3 0
4 0
5 0
6 3
7 0
8 0
9 0
U34-MY:ASP-S2
3 0
4 0
5 0
6 0
7 0
8 0
9 3
U-P:ILE-S1
3 0
4 0
5 6
6 12
7 9
8 15
9 36
DA-M5:LEU-S2
3 0
4 0
5 0
6 0
7 0

8 0
9 3
U-RIB:CYS-CA
3 0
4 0
5 0
6 0
7 0
8 14
9 9
H2U-RIB:ARG-S2
3 0
4 0
5 0
6 3
7 0
8 0
9 3
A-P:LYS-CA
3 0
4 0
5 52
6 44
7 112
8 180
9 268
QUO-RIB:ASN-S1
3 0
4 0
5 0
6 0
7 4
8 0
9 0
DA-M5:GLN-CA
3 0
4 0
5 0
6 0
7 0
8 0
9 3
FMU-RIB:ARG-S2
3 0
4 0
5 3
6 0
7 0
8 6
9 0
G-R6:MET-S2
3 0
4 0
5 8
6 32
7 24
8 28
9 36

C31-RIB:TYR-S2
3 0
4 0
5 0
6 0
7 6
8 0
9 0
U-RIB:ASP-S1
3 0
4 3
5 9
6 9
7 39
8 36
9 54
U34-RIB:SER-CA
3 0
4 0
5 0
6 0
7 0
8 0
9 3
H2U-P:TRP-S2
3 0
4 0
5 0
6 3
7 0
8 0
9 0
DA-M6:HIS-CA
3 0
4 0
5 0
6 3
7 0
8 0
9 0
IU-P:LYS-CA
3 0
4 0
5 0
6 0
7 0
8 6
9 0
FMU-MY:CYS-S1
3 0
4 3
5 0
6 0
7 0
8 0
9 0
DA-RIB:ARG-CA
3 0

4 0
5 0
6 0
7 0
8 0
9 3
DA-RIB:MET-CA
3 0
4 0
5 0
6 0
7 0
8 0
9 3
FHU-P:VAL-S1
3 0
4 0
5 0
6 0
7 0
8 6
9 0
A-R5:ASN-S2
3 0
4 0
5 8
6 8
7 79
8 100
9 132
IU-P:SER-S1
3 0
4 0
5 0
6 0
7 0
8 0
9 6
G-R5:ARG-S2
3 0
4 12
5 52
6 96
7 144
8 212
9 369
QUO-M6:GLN-S2
3 0
4 0
5 0
6 0
7 4
8 0
9 0
U31-MY:MET-S1
3 0
4 0
5 3

6 0
7 0
8 0
9 0
C31-MY:ASP-CA
3 0
4 0
5 0
6 0
7 0
8 0
9 3
QUO-RIB:PHE-S1
3 0
4 0
5 0
6 0
7 0
8 0
9 4
A-RIB:LYS-S1
3 0
4 0
5 12
6 92
7 120
8 152
9 271
GTP-M6:ASN-CA
3 0
4 0
5 0
6 0
7 0
8 0
9 3
GTP-RIB:ASN-CA
3 0
4 0
5 0
6 3
7 0
8 0
9 0
H2U-MY:ASN-S1
3 0
4 0
5 0
6 3
7 0
8 3
9 3
C31-P:MET-S1
3 0
4 0
5 0
6 0
7 0

8   3
9   0
U-Y:ILE-CA
3   0
4   0
5   0
6   0
7   12
8   18
9   36
U-P:GLY-CA
3   0
4   15
5   57
6   57
7   75
8   105
9   156
5BU-P:SER-CA
3   0
4   0
5   0
6   0
7   0
8   3
9   0
C-RIB:SER-S1
3   0
4   0
5   45
6   78
7   77
8   129
9   161
G-R6:CYS-CA
3   0
4   0
5   0
6   0
7   12
8   4
9   8
C31-RIB:ASP-S2
3   0
4   0
5   0
6   0
7   0
8   0
9   6
U-RIB:GLU-S2
3   0
4   0
5   15
6   12
7   24
8   36
9   57

U31-MY:PHE-S2
3 0
4 0
5 0
6 0
7 0
8 0
9 3
U-RIB:THR-CA
3 0
4 0
5 0
6 30
7 33
8 68
9 77
C31-P:GLN-S2
3 0
4 0
5 0
6 0
7 0
8 0
9 3
A-R6:MET-CA
3 0
4 0
5 8
6 8
7 24
8 16
9 56
A-R5:GLU-S2
3 0
4 0
5 8
6 24
7 56
8 100
9 104
G-R5:GLY-CA
3 0
4 4
5 44
6 56
7 88
8 208
9 248
C-P:LEU-S1
3 0
4 0
5 6
6 15
7 57
8 48
9 99
G-RIB:TRP-S1
3 0

4 4
5 8
6 8
7 16
8 52
9 20
FMU-RIB:MET-S1
3 0
4 0
5 0
6 0
7 0
8 0
9 3
C31-RIB:PHE-S2
3 0
4 0
5 3
6 0
7 3
8 0
9 0
A-RIB:GLU-CA
3 0
4 0
5 12
6 20
7 36
8 44
9 120
H2U-P:PHE-S2
3 0
4 0
5 0
6 3
7 0
8 0
9 0
A-R5:GLU-CA
3 0
4 0
5 8
6 4
7 32
8 60
9 80
C-P:LYS-S1
3 0
4 3
5 66
6 141
7 150
8 141
9 213
FMU-MY:SER-CA
3 0
4 0
5 0

6   0
7   3
8   0
9   0
A-RIB:ARG-CA
3   0
4   0
5   16
6   64
7   96
8   160
9   328
A-RIB:MET-CA
3   0
4   0
5   8
6   8
7   20
8   40
9   52
G-R6:SER-S1
3   0
4   0
5   12
6   56
7   59
8   84
9   144
C-RIB:SER-CA
3   0
4   0
5   27
6   60
7   97
8   120
9   120
G-P:ASN-S1
3   0
4   4
5   56
6   48
7   104
8   120
9   172
FMU-MY:VAL-S1
3   0
4   0
5   0
6   0
7   0
8   6
9   0
C-Y:LYS-CA
3   0
4   0
5   0
6   24
7   27

8 54
9 135
G-R6:TRP-CA
3 0
4 0
5 4
6 12
7 4
8 16
9 32
FHU-P:ASP-S2
3 0
4 0
5 0
6 0
7 0
8 0
9 3
G-P:ILE-S1
3 0
4 0
5 8
6 16
7 24
8 64
9 108
FHU-RIB:LYS-S2
3 0
4 0
5 0
6 0
7 0
8 0
9 9
U34-P:HIS-S2
3 0
4 0
5 3
6 0
7 0
8 0
9 3
FMU-P:PHE-S1
3 0
4 0
5 3
6 0
7 0
8 0
9 0
A-R5:CYS-CA
3 0
4 0
5 0
6 4
7 8
8 4
9 4

IU-RIB:LYS-S2
3 0
4 0
5 0
6 3
7 2
8 3
9 0
H2U-MY:THR-CA
3 0
4 0
5 0
6 0
7 0
8 0
9 6
I-RIB:ALA-S1
3 0
4 0
5 0
6 0
7 0
8 2
9 0
DA-M5:SER-S1
3 0
4 0
5 3
6 0
7 0
8 0
9 3
IU-RIB:ALA-S1
3 0
4 0
5 0
6 0
7 2
8 3
9 3
G-RIB:TYR-S2
3 0
4 0
5 0
6 39
7 28
8 62
9 99
IU-MY:SER-CA
3 0
4 0
5 0
6 0
7 2
8 0
9 0
IU-P:ILE-S1
3 0

4 0
5 0
6 0
7 0
8 0
9 3
U-Y:CYS-CA
3 0
4 0
5 0
6 6
7 0
8 5
9 6
U-P:GLN-S1
3 0
4 3
5 9
6 9
7 39
8 48
9 66
G-RIB:TRP-S2
3 0
4 0
5 4
6 16
7 28
8 28
9 32
GTP-M5:GLY-CA
3 0
4 0
5 0
6 0
7 0
8 0
9 3
IU-RIB:LYS-S1
3 0
4 0
5 3
6 0
7 0
8 3
9 6
FHU-RIB:TYR-CA
3 0
4 0
5 0
6 6
7 0
8 0
9 0
FHU-P:ALA-S1
3 0
4 0
5 0

6 0
7 3
8 0
9 0
G-P:GLN-S1
3 0
4 4
5 36
6 24
7 76
8 112
9 160
IU-MY:PRO-S1
3 0
4 0
5 0
6 2
7 3
8 0
9 0
G-R5:HIS-CA
3 0
4 0
5 4
6 8
7 27
8 32
9 75
G-R5:GLN-S1
3 0
4 0
5 4
6 4
7 40
8 60
9 132
FMU-MY:PRO-CA
3 0
4 0
5 0
6 0
7 0
8 3
9 0
A-R5:ASN-S1
3 0
4 0
5 4
6 16
7 32
8 87
9 104
QUO-RIB:LEU-CA
3 0
4 0
5 0
6 0
7 0

8 0
9 8
C31-MY:LEU-S2
3 0
4 3
5 0
6 0
7 0
8 0
9 0
U-RIB:VAL-S1
3 0
4 0
5 23
6 15
7 12
8 36
9 57
U34-RIB:TYR-S2
3 0
4 0
5 0
6 0
7 0
8 0
9 3
G-RIB:TYR-CA
3 0
4 0
5 0
6 8
7 35
8 23
9 47
C-RIB:LYS-S1
3 0
4 0
5 12
6 72
7 96
8 164
9 258
QUO-P:ASN-S2
3 0
4 0
5 0
6 0
7 0
8 0
9 4
G-P:HIS-S1
3 0
4 4
5 8
6 20
7 64
8 120
9 56

FHU-RIB:ASP-S1
3 0
4 0
5 0
6 0
7 6
8 0
9 0
C-RIB:VAL-S1
3 0
4 0
5 24
6 42
7 78
8 60
9 99
U-P:TRP-S2
3 0
4 0
5 0
6 9
7 25
8 12
9 21
G-R6:GLU-CA
3 0
4 0
5 0
6 4
7 28
8 62
9 99
A-P:ASN-CA
3 0
4 0
5 20
6 32
7 40
8 104
9 120
A-P:SER-S1
3 0
4 16
5 92
6 64
7 48
8 124
9 192
FHU-P:ALA-CA
3 0
4 0
5 0
6 0
7 3
8 0
9 0
G-P:PHE-S1
3 0

4 0
5 12
6 8
7 24
8 56
9 84
DA-M6:ASN-CA
3 0
4 0
5 0
6 0
7 0
8 0
9 3
QUO-M6:ASP-S1
3 0
4 0
5 0
6 0
7 4
8 0
9 0
G-RIB:THR-CA
3 0
4 0
5 16
6 40
7 48
8 80
9 136
A-RIB:ASP-CA
3 0
4 0
5 8
6 20
7 40
8 64
9 108
A-P:ALA-CA
3 0
4 4
5 36
6 44
7 88
8 104
9 132
U31-MY:GLU-S1
3 0
4 0
5 0
6 0
7 0
8 3
9 0
FHU-MY:PHE-S2
3 0
4 0
5 0

6 0
7 0
8 3
9 0
IU-MY:ILE-S1
3 0
4 0
5 0
6 0
7 0
8 2
9 0
U-Y:LYS-S2
3 0
4 0
5 0
6 54
7 71
8 102
9 183
OMC-RIB:LYS-S1
3 0
4 0
5 0
6 0
7 0
8 3
9 0
C-Y:LEU-CA
3 0
4 0
5 6
6 6
7 12
8 39
9 78
U31-P:ASP-S1
3 0
4 3
5 0
6 0
7 0
8 3
9 0
G-R6:GLU-S2
3 0
4 0
5 39
6 44
7 47
8 95
9 115
C-Y:GLU-CA
3 0
4 0
5 6
6 12
7 3

8   15
9   68
A-RIB:GLN-CA
3   0
4   0
5   12
6   12
7   24
8   64
9   76
U-Y:MET-S1
3   0
4   0
5   3
6   6
7   12
8   6
9   12
C31-P:LEU-S2
3   0
4   0
5   0
6   0
7   0
8   0
9   3
FHU-RIB:SER-S1
3   0
4   0
5   0
6   0
7   0
8   6
9   6
QUO-RIB:LEU-S2
3   0
4   0
5   0
6   0
7   8
8   0
9   0
G-R5:SER-CA
3   0
4   0
5   0
6   31
7   36
8   92
9   116
A-R5:ASP-CA
3   0
4   0
5   8
6   4
7   28
8   44
9   68

FHU-P:ARG-S2
3 0
4 0
5 0
6 6
7 0
8 0
9 3
U-Y:HIS-S2
3 0
4 9
5 3
6 15
7 39
8 18
9 42
H2U-MY:ALA-CA
3 0
4 0
5 0
6 0
7 0
8 0
9 3
C31-P:PHE-S1
3 0
4 0
5 0
6 0
7 0
8 3
9 0
QUO-M5:LEU-CA
3 0
4 0
5 0
6 0
7 0
8 4
9 0
U-P:TRP-S1
3 0
4 3
5 6
6 9
7 9
8 22
9 27
U31-RIB:MET-S2
3 0
4 0
5 0
6 3
7 0
8 0
9 0
U34-MY:ASN-S2
3 0

4 0
5 0
6 0
7 0
8 6
9 0
DA-RIB:ASN-CA
3 0
4 0
5 0
6 0
7 0
8 3
9 0
U-P:PHE-S1
3 0
4 0
5 3
6 0
7 12
8 27
9 30
IU-RIB:ARG-S1
3 0
4 0
5 0
6 3
7 2
8 0
9 0
FHU-MY:LEU-CA
3 0
4 0
5 0
6 3
7 3
8 6
9 0
G-R6:ALA-S1
3 0
4 4
5 8
6 32
7 7
8 96
9 108
C-Y:CYS-S1
3 0
4 0
5 3
6 0
7 0
8 3
9 9
H2U-P:LYS-S2
3 0
4 0
5 0

6   0
7   0
8   0
9   6
A-P:TRP-S2
3   0
4   0
5   0
6   16
7   12
8   12
9   28
A-RIB:ASN-CA
3   0
4   0
5   8
6   40
7   64
8   68
9   148
A-RIB:LYS-CA
3   0
4   0
5   16
6   40
7   92
8   156
9   216
G-R5:ALA-CA
3   0
4   4
5   8
6   16
7   28
8   79
9   112
FHU-MY:LEU-S1
3   0
4   0
5   0
6   0
7   9
8   3
9   3
G-RIB:ASP-S2
3   0
4   0
5   52
6   36
7   60
8   120
9   212
U-P:HIS-S2
3   0
4   0
5   6
6   21
7   36

8 69
9 60
A-RIB:MET-S2
3 0
4 0
5 16
6 12
7 20
8 28
9 32
C31-P:GLU-CA
3 0
4 0
5 0
6 3
7 0
8 0
9 0
U-RIB:ALA-S1
3 0
4 3
5 15
6 33
7 51
8 65
9 110
C-P:SER-CA
3 0
4 6
5 57
6 45
7 81
8 93
9 153
G-P:ASP-S2
3 0
4 0
5 12
6 48
7 112
8 140
9 252
U-P:THR-CA
3 0
4 3
5 18
6 18
7 21
8 60
9 90
G-R6:ASP-S1
3 0
4 0
5 8
6 28
7 68
8 63
9 120

A-R6:LYS-CA
3 0
4 0
5 12
6 16
7 36
8 100
9 112
C-Y:ASP-S2
3 0
4 3
5 18
6 18
7 57
8 75
9 90
G-R6:TYR-CA
3 0
4 0
5 0
6 21
7 16
8 12
9 36
C-Y:SER-CA
3 0
4 0
5 3
6 21
7 45
8 71
9 105
U-Y:TYR-CA
3 0
4 0
5 3
6 15
7 9
8 18
9 42
G-RIB:ARG-CA
3 0
4 0
5 12
6 88
7 92
8 168
9 348
C-P:CYS-S1
3 0
4 0
5 0
6 3
7 3
8 9
9 15
FHU-MY:ARG-CA
3 0

4 0
5 0
6 3
7 3
8 0
9 9
C-P:SER-S1
3 0
4 27
5 60
6 57
7 63
8 105
9 177
G-R6:CYS-S1
3 0
4 0
5 0
6 4
7 4
8 0
9 12
A-R6:ASN-S2
3 0
4 0
5 12
6 63
7 64
8 72
9 104
A-RIB:TRP-S1
3 0
4 0
5 4
6 4
7 16
8 28
9 36
G-R6:ASP-CA
3 0
4 0
5 0
6 12
7 52
8 55
9 100
G-R5:CYS-S1
3 0
4 0
5 0
6 0
7 4
8 4
9 4
QUO-M6:LYS-S1
3 0
4 0
5 0

6 0
7 0
8 0
9 4
A-P:ASP-S2
3 0
4 0
5 8
6 32
7 88
8 92
9 108
IU-MY:LYS-CA
3 0
4 0
5 2
6 0
7 3
8 2
9 5
U34-RIB:ASN-CA
3 0
4 0
5 0
6 0
7 0
8 3
9 9
H2U-RIB:PHE-CA
3 0
4 0
5 0
6 0
7 0
8 0
9 3
A-P:ASN-S2
3 0
4 0
5 48
6 32
7 60
8 96
9 124
IU-RIB:HIS-S2
3 0
4 0
5 0
6 0
7 0
8 2
9 3
U-Y:GLY-CA
3 0
4 0
5 9
6 42
7 15

8 75
9 141
C31-RIB:GLU-S2
3 0
4 0
5 0
6 3
7 0
8 0
9 3
A-R6:ILE-S1
3 0
4 0
5 8
6 4
7 4
8 48
9 56
A-P:VAL-CA
3 0
4 0
5 24
6 12
7 44
8 80
9 116
A-R5:PRO-CA
3 0
4 0
5 4
6 16
7 52
8 60
9 92
C-RIB:PHE-S1
3 0
4 0
5 6
6 3
7 12
8 24
9 60
G-R5:LEU-S1
3 0
4 0
5 16
6 20
7 16
8 44
9 68
FMU-RIB:GLU-S1
3 0
4 0
5 0
6 0
7 0
8 0
9 3

FHU-RIB:ILE-CA
3 0
4 0
5 0
6 0
7 0
8 6
9 0
5BU-P:PRO-S1
3 0
4 0
5 0
6 3
7 0
8 0
9 0
C-P:ASN-S2
3 0
4 0
5 54
6 60
7 63
8 84
9 132
QUO-P:SER-S1
3 0
4 0
5 0
6 0
7 0
8 0
9 4
U34-RIB:ASN-S1
3 0
4 0
5 0
6 0
7 6
8 3
9 0
IU-RIB:GLN-S1
3 0
4 0
5 0
6 0
7 0
8 0
9 3
U34-MY:PHE-CA
3 0
4 0
5 0
6 0
7 0
8 0
9 3
U31-MY:GLN-S1
3 0

4 0
5 0
6 0
7 3
8 0
9 0
FHU-MY:VAL-S1
3 0
4 0
5 0
6 0
7 0
8 3
9 6
FMU-MY:MET-S2
3 0
4 0
5 0
6 0
7 3
8 3
9 0
FHU-MY:ASP-S1
3 0
4 0
5 0
6 6
7 0
8 0
9 6
C-Y:LYS-S1
3 0
4 0
5 6
6 24
7 60
8 54
9 174
C-RIB:PHE-CA
3 0
4 0
5 0
6 3
7 15
8 33
9 39
H2U-P:GLU-S2
3 0
4 0
5 0
6 0
7 3
8 0
9 3
A-P:HIS-S1
3 0
4 0
5 12

6 12
7 48
8 88
9 84
FMU-RIB:CYS-S1
3 0
4 0
5 3
6 0
7 0
8 0
9 0
A-RIB:VAL-S1
3 0
4 0
5 16
6 48
7 80
8 64
9 100
G-P:ASP-S1
3 0
4 4
5 12
6 28
7 80
8 148
9 216
FMU-RIB:ALA-CA
3 0
4 0
5 0
6 0
7 0
8 3
9 0
G-P:GLY-CA
3 0
4 16
5 120
6 44
7 152
8 244
9 320
G-P:ARG-CA
3 0
4 0
5 28
6 68
7 156
8 324
9 428
U-Y:ALA-S1
3 0
4 0
5 17
6 24
7 27

8 21
9 65
A-RIB:TYR-CA
3 0
4 0
5 4
6 16
7 60
8 24
9 64
H2U-RIB:PRO-S1
3 0
4 0
5 0
6 0
7 0
8 3
9 0
A-R5:GLY-CA
3 0
4 4
5 44
6 36
7 96
8 136
9 268
U34-P:ASN-S2
3 0
4 0
5 3
6 0
7 3
8 0
9 3
DA-M6:LYS-S2
3 0
4 3
5 0
6 0
7 0
8 0
9 0
U-P:VAL-S1
3 0
4 0
5 0
6 21
7 36
8 45
9 75
C-P:ARG-S1
3 0
4 3
5 126
6 162
7 108
8 261
9 327

FHU-P:PRO-S1
3 0
4 0
5 0
6 0
7 0
8 3
9 0
C-Y:VAL-S1
3 0
4 0
5 15
6 27
7 6
8 39
9 69
DA-RIB:HIS-CA
3 0
4 0
5 0
6 0
7 3
8 3
9 0
G-P:PHE-S2
3 0
4 0
5 0
6 28
7 52
8 32
9 60
FMU-MY:SER-S1
3 0
4 0
5 0
6 0
7 0
8 3
9 0
H2U-RIB:PRO-CA
3 0
4 0
5 0
6 0
7 0
8 0
9 3
G-P:LEU-S2
3 0
4 4
5 16
6 48
7 36
8 92
9 128
G-R5:HIS-S1
3 0

4 0
5 12
6 20
7 35
8 44
9 103
U-Y:LEU-CA
3 0
4 0
5 0
6 0
7 21
8 39
9 48
A-R5:HIS-S2
3 0
4 12
5 16
6 20
7 56
8 60
9 100
5BU-P:ILE-S1
3 0
4 0
5 0
6 3
7 3
8 0
9 0
4SU-P:THR-S1
3 0
4 0
5 0
6 0
7 0
8 3
9 0
FMU-MY:PHE-S1
3 0
4 0
5 0
6 0
7 0
8 3
9 0
I-RIB:TRP-S2
3 0
4 0
5 0
6 0
7 2
8 0
9 0
A-R6:ASP-S2
3 0
4 0
5 4

6   32
7   36
8   64
9   124
C31-RIB:ASP-S1
3   0
4   0
5   0
6   0
7   0
8   0
9   3
A-R6:ILE-CA
3   0
4   0
5   4
6   12
7   8
8   28
9   56
U31-P:ASN-S2
3   0
4   0
5   0
6   3
7   0
8   0
9   0
G-R6:THR-CA
3   0
4   0
5   0
6   8
7   24
8   60
9   84
A-P:LEU-S2
3   0
4   0
5   12
6   36
7   20
8   60
9   80
U31-MY:GLU-CA
3   0
4   0
5   0
6   0
7   0
8   3
9   0
FMU-MY:HIS-S1
3   0
4   0
5   0
6   0
7   0

8 0
9 3
G-RIB:MET-CA
3 0
4 4
5 4
6 0
7 32
8 56
9 52
IU-RIB:HIS-S1
3 0
4 0
5 0
6 0
7 5
8 0
9 0
A-RIB:TYR-S1
3 0
4 0
5 12
6 32
7 44
8 32
9 44
G-R6:LYS-S2
3 0
4 4
5 36
6 124
7 72
8 168
9 224
FHU-RIB:LEU-S2
3 0
4 0
5 0
6 0
7 3
8 6
9 6
A-RIB:ALA-S1
3 0
4 4
5 24
6 48
7 88
8 84
9 152
H2U-P:ASN-S1
3 0
4 0
5 0
6 0
7 3
8 6
9 0

C-RIB:TRP-S2
3 0
4 0
5 3
6 14
7 18
8 15
9 27
C-Y:HIS-S2
3 0
4 3
5 0
6 18
7 45
8 57
9 42
H2U-RIB:ASN-S1
3 0
4 0
5 0
6 0
7 0
8 0
9 6
GTP-M5:ALA-S1
3 0
4 0
5 0
6 0
7 0
8 3
9 0
IU-RIB:HIS-CA
3 0
4 0
5 0
6 2
7 3
8 0
9 0
U31-P:SER-CA
3 0
4 0
5 0
6 0
7 0
8 0
9 3
A-RIB:ARG-S1
3 0
4 0
5 24
6 100
7 148
8 288
9 416
G-R6:HIS-S1
3 0

4   0
5   8
6   32
7   28
8   68
9   87
FHU-MY:ALA-S1
3   0
4   0
5   0
6   0
7   6
8   3
9   3
GTP-RIB:SER-S1
3   0
4   0
5   0
6   0
7   3
8   0
9   3
U34-P:SER-CA
3   0
4   0
5   0
6   0
7   0
8   3
9   3
U-P:TYR-CA
3   0
4   0
5   0
6   3
7   6
8   6
9   45
U31-P:HIS-CA
3   0
4   0
5   0
6   0
7   3
8   0
9   0
I-RIB:GLY-CA
3   0
4   0
5   0
6   0
7   0
8   0
9   2
C-RIB:ASP-CA
3   0
4   0
5   15

6   33
7   54
8   84
9   99
U31-MY:VAL-CA
3   0
4   0
5   0
6   0
7   0
8   0
9   3
FMU-RIB:ASN-S1
3   0
4   0
5   0
6   0
7   0
8   0
9   3
C-RIB:LEU-S1
3   0
4   0
5   6
6   27
7   42
8   95
9   132
IU-MY:PRO-CA
3   0
4   0
5   0
6   0
7   0
8   2
9   3
A-P:CYS-CA
3   0
4   0
5   4
6   4
7   4
8   4
9   28
DA-M5:ASN-S1
3   0
4   0
5   0
6   0
7   0
8   3
9   0
QUO-M6:ASP-CA
3   0
4   0
5   0
6   0
7   0

8   4
9   0
FHU-RIB:SER-CA
3   0
4   0
5   0
6   0
7   0
8   6
9   0
FMU-MY:ASP-S2
3   0
4   0
5   0
6   3
7   0
8   0
9   0
IU-P:LYS-S2
3   0
4   0
5   3
6   0
7   0
8   0
9   3
G-R5:PRO-S1
3   0
4   0
5   20
6   8
7   48
8   56
9   155
H2U-RIB:LYS-S1
3   0
4   0
5   0
6   0
7   0
8   0
9   3
H2U-RIB:TRP-S2
3   0
4   0
5   0
6   0
7   3
8   0
9   0
U-P:ASP-CA
3   0
4   0
5   12
6   12
7   15
8   48
9   67

C-P:PRO-CA
3 0
4 0
5 30
6 42
7 36
8 93
9 111
QUO-M6:PHE-CA
3 0
4 0
5 0
6 0
7 0
8 4
9 4
U-P:ARG-S2
3 0
4 0
5 144
6 111
7 78
8 172
9 252
G-P:GLN-CA
3 0
4 0
5 28
6 16
7 56
8 144
9 104
DA-M5:HIS-S1
3 0
4 0
5 0
6 3
7 0
8 0
9 0
FHU-P:LYS-CA
3 0
4 0
5 3
6 3
7 0
8 3
9 3
A-R6:GLN-S2
3 0
4 4
5 24
6 44
7 68
8 60
9 84
FHU-P:THR-CA
3 0

4 0
5 0
6 3
7 6
8 3
9 0
5BU-RIB:ILE-S1
3 0
4 0
5 0
6 0
7 0
8 0
9 3
G-P:PRO-S1
3 0
4 0
5 48
6 48
7 72
8 140
9 212
IU-MY:SER-S1
3 0
4 0
5 2
6 0
7 0
8 0
9 3
GTP-RIB:ARG-S1
3 0
4 0
5 0
6 0
7 0
8 3
9 0
FHU-RIB:LEU-S1
3 0
4 0
5 0
6 0
7 3
8 3
9 0
A-P:LEU-CA
3 0
4 0
5 8
6 16
7 28
8 44
9 108
DA-M5:LYS-S1
3 0
4 0
5 0

6 0
7 0
8 3
9 0
QUO-RIB:ASP-S1
3 0
4 0
5 0
6 0
7 0
8 0
9 4
A-R5:ASN-CA
3 0
4 0
5 0
6 4
7 28
8 59
9 84
A-R6:SER-S1
3 0
4 0
5 44
6 32
7 44
8 80
9 100
U-RIB:MET-S2
3 0
4 0
5 6
6 6
7 15
8 12
9 30
GTP-M5:THR-CA
3 0
4 0
5 0
6 0
7 0
8 0
9 3
A-P:GLN-S2
3 0
4 4
5 36
6 40
7 36
8 116
9 120
FHU-RIB:ARG-S2
3 0
4 0
5 0
6 0
7 12

8 0
9 3
A-RIB:HIS-S1
3 0
4 0
5 20
6 44
7 52
8 80
9 60
G-R6:VAL-S1
3 0
4 0
5 12
6 16
7 20
8 42
9 60
U-RIB:LYS-CA
3 0
4 0
5 6
6 30
7 60
8 114
9 116
GTP-M6:THR-S1
3 0
4 0
5 0
6 0
7 0
8 0
9 3
G-R6:TYR-S1
3 0
4 0
5 7
6 10
7 36
8 4
9 48
IU-RIB:ALA-CA
3 0
4 0
5 0
6 0
7 2
8 3
9 0
U-RIB:SER-S1
3 0
4 6
5 20
6 39
7 51
8 47
9 90

U-P:TRP-CA
3 0
4 0
5 6
6 6
7 21
8 12
9 24
C31-P:PHE-CA
3 0
4 0
5 0
6 0
7 0
8 0
9 3
FHU-MY:SER-S1
3 0
4 0
5 0
6 0
7 0
8 0
9 3
U31-P:MET-S1
3 0
4 0
5 0
6 3
7 3
8 0
9 0
C-P:ILE-S1
3 0
4 0
5 6
6 12
7 30
8 54
9 75
U31-P:TYR-S1
3 0
4 0
5 0
6 0
7 0
8 0
9 3
C-Y:GLY-CA
3 0
4 3
5 15
6 36
7 57
8 105
9 231
G-P:TYR-CA
3 0

4 0
5 8
6 8
7 28
8 52
9 84
U31-MY:ASP-S1
3 0
4 0
5 0
6 6
7 0
8 3
9 3
C-RIB:PRO-CA
3 0
4 3
5 12
6 38
7 66
8 66
9 93
FHU-MY:SER-CA
3 0
4 0
5 0
6 0
7 0
8 0
9 6
U31-MY:MET-CA
3 0
4 0
5 0
6 3
7 0
8 0
9 0
FMU-RIB:PHE-S1
3 0
4 0
5 0
6 0
7 0
8 3
9 3
U31-RIB:TYR-S1
3 0
4 0
5 0
6 0
7 0
8 3
9 0
G-R6:PHE-S1
3 0
4 0
5 4

6 8
7 11
8 16
9 44
FMU-RIB:VAL-S1
3 0
4 0
5 0
6 0
7 3
8 0
9 0
A-R5:PHE-S2
3 0
4 4
5 8
6 12
7 16
8 44
9 44
U31-P:GLN-S1
3 0
4 0
5 0
6 3
7 0
8 0
9 0
FHU-RIB:ASP-S2
3 0
4 0
5 0
6 6
7 0
8 0
9 0
C31-P:ASP-S2
3 0
4 0
5 3
6 3
7 3
8 0
9 0
A-R6:PRO-CA
3 0
4 0
5 16
6 24
7 48
8 76
9 72
A-R5:ASP-S2
3 0
4 0
5 16
6 16
7 36

8   68
9   104
G-R6:ILE-S1
3   0
4   4
5   4
6   0
7   4
8   15
9   39
H2U-P:GLU-CA
3   0
4   0
5   0
6   0
7   0
8   0
9   3
QUO-P:PHE-S2
3   0
4   0
5   0
6   0
7   0
8   0
9   4
DA-RIB:ASP-S1
3   0
4   0
5   0
6   3
7   0
8   0
9   0
DA-M6:HIS-S1
3   0
4   0
5   0
6   3
7   0
8   0
9   0
DA-M5:THR-S1
3   0
4   0
5   0
6   0
7   3
8   0
9   0
FMU-RIB:ASP-S2
3   0
4   0
5   0
6   0
7   0
8   0
9   3

IU-MY:ALA-CA
3 0
4 0
5 2
6 0
7 0
8 0
9 6
C-RIB:CYS-CA
3 0
4 0
5 0
6 3
7 0
8 15
9 18
G-RIB:PRO-S1
3 0
4 0
5 28
6 72
7 116
8 106
9 132
FMU-RIB:GLN-S1
3 0
4 0
5 0
6 0
7 0
8 0
9 3
H2U-MY:ARG-S2
3 0
4 0
5 3
6 0
7 0
8 3
9 6
5BU-RIB:PRO-S1
3 0
4 0
5 0
6 0
7 3
8 0
9 0
IU-RIB:SER-S1
3 0
4 0
5 2
6 0
7 0
8 2
9 0
G-R5:ALA-S1
3 0

4   4
5   12
6   24
7   43
8   68
9   148
IU-MY:ARG-S1
3   0
4   0
5   3
6   0
7   0
8   0
9   3
GTP-M6:SER-S1
3   0
4   0
5   0
6   0
7   0
8   0
9   6
A-RIB:ILE-CA
3   0
4   0
5   8
6   12
7   28
8   40
9   92
G-P:TRP-S2
3   0
4   0
5   0
6   12
7   24
8   40
9   68
FHU-MY:ILE-CA
3   0
4   0
5   6
6   0
7   0
8   0
9   0
DA-RIB:ALA-CA
3   0
4   0
5   0
6   0
7   3
8   0
9   0
G-R5:LYS-S2
3   0
4   8
5   84

6   80
7   124
8   160
9   382
U-RIB:TRP-CA
3   0
4   0
5   0
6   3
7   6
8   18
9   21
FHU-MY:THR-CA
3   0
4   0
5   0
6   0
7   3
8   6
9   3
U34-P:TYR-S2
3   0
4   0
5   0
6   3
7   0
8   0
9   0
DA-RIB:VAL-S1
3   0
4   0
5   0
6   0
7   0
8   3
9   0
A-R6:PHE-S1
3   0
4   8
5   8
6   0
7   4
8   24
9   28
H2U-P:THR-S1
3   0
4   0
5   0
6   0
7   0
8   0
9   6
DA-M5:GLU-S2
3   0
4   0
5   0
6   0
7   3

8 0
9 0
GTP-RIB:ASN-S1
3 0
4 0
5 3
6 0
7 0
8 0
9 0
U31-P:LEU-S2
3 0
4 0
5 0
6 0
7 0
8 0
9 3
FMU-P:GLN-CA
3 0
4 0
5 0
6 0
7 0
8 0
9 3
IU-MY:ILE-CA
3 0
4 0
5 0
6 0
7 0
8 2
9 3
C31-P:ASN-CA
3 0
4 0
5 0
6 0
7 0
8 0
9 3
G-RIB:LEU-S1
3 0
4 0
5 20
6 20
7 56
8 92
9 108
DA-RIB:ASP-S2
3 0
4 0
5 0
6 0
7 0
8 3
9 0

5BU-MY:PRO-CA
3 0
4 0
5 0
6 0
7 0
8 0
9 3
FMU-P:ALA-CA
3 0
4 0
5 0
6 0
7 0
8 0
9 3
C-RIB:TYR-S2
3 0
4 0
5 3
6 26
7 42
8 30
9 66
5BU-P:SER-S1
3 0
4 0
5 0
6 0
7 0
8 3
9 0
H2U-MY:THR-S1
3 0
4 0
5 0
6 0
7 0
8 0
9 3
U34-P:SER-S1
3 0
4 0
5 0
6 0
7 0
8 0
9 9
U31-RIB:GLN-S1
3 0
4 0
5 3
6 0
7 0
8 0
9 0
U-P:TYR-S1
3 0

4 0
5 0
6 3
7 3
8 15
9 39
A-R6:TYR-S1
3 0
4 12
5 4
6 16
7 28
8 55
9 48
FMU-MY:ARG-S1
3 0
4 0
5 0
6 0
7 0
8 3
9 0
DA-M6:TYR-S2
3 0
4 0
5 0
6 0
7 0
8 0
9 3
FHU-RIB:PRO-CA
3 0
4 0
5 0
6 0
7 0
8 0
9 3
FMU-MY:ASN-S1
3 0
4 0
5 0
6 0
7 0
8 0
9 3
G-R6:LEU-S2
3 0
4 4
5 28
6 16
7 8
8 36
9 76
FMU-RIB:ARG-CA
3 0
4 0
5 0

6 0
7 0
8 3
9 0
DA-M5:THR-CA
3 0
4 0
5 0
6 0
7 0
8 0
9 3
G-R6:TRP-S1
3 0
4 4
5 12
6 0
7 24
8 12
9 52
FHU-RIB:PRO-S1
3 0
4 0
5 0
6 0
7 0
8 3
9 0
A-R5:ILE-S1
3 0
4 0
5 4
6 4
7 12
8 20
9 56
FMU-P:ARG-S1
3 0
4 0
5 0
6 0
7 0
8 3
9 6
DA-RIB:ASN-S2
3 0
4 0
5 0
6 3
7 0
8 0
9 0
H2U-RIB:GLU-S2
3 0
4 0
5 0
6 0
7 0

8 3
9 0
IU-MY:LEU-S2
3 0
4 0
5 0
6 0
7 2
8 0
9 0
U34-MY:ASP-CA
3 0
4 0
5 0
6 0
7 0
8 0
9 3
FHU-MY:TYR-S2
3 0
4 0
5 0
6 9
7 3
8 0
9 0
G-RIB:TRP-CA
3 0
4 0
5 4
6 20
7 16
8 28
9 36
QUO-M6:GLU-S1
3 0
4 0
5 0
6 0
7 4
8 0
9 0
H2U-RIB:LEU-S2
3 0
4 0
5 0
6 0
7 0
8 0
9 3
U-RIB:ASN-CA
3 0
4 0
5 3
6 21
7 42
8 48
9 84

FHU-MY:ALA-CA
3 0
4 0
5 0
6 0
7 0
8 9
9 0
A-R6:PHE-S2
3 0
4 4
5 12
6 24
7 12
8 20
9 40
FHU-RIB:TYR-S2
3 0
4 0
5 6
6 0
7 3
8 3
9 0
G-R5:ASN-S2
3 0
4 0
5 23
6 38
7 64
8 92
9 140
IU-P:HIS-S2
3 0
4 0
5 0
6 3
7 0
8 0
9 0
A-RIB:TYR-S2
3 0
4 0
5 36
6 52
7 12
8 32
9 76
DA-M5:SER-CA
3 0
4 0
5 0
6 3
7 0
8 0
9 3
QUO-M5:ASP-S1
3 0

4 0
5 0
6 0
7 0
8 4
9 4
G-R6:VAL-CA
3 0
4 0
5 0
6 24
7 24
8 24
9 75
GTP-M5:SER-CA
3 0
4 0
5 0
6 0
7 0
8 3
9 0
FMU-MY:ALA-CA
3 0
4 0
5 0
6 3
7 0
8 0
9 0
FHU-RIB:GLY-CA
3 0
4 0
5 0
6 3
7 6
8 3
9 0
U31-P:ALA-S1
3 0
4 0
5 0
6 0
7 0
8 0
9 3
U34-MY:SER-S1
3 0
4 0
5 3
6 0
7 3
8 0
9 0
U31-MY:GLN-S2
3 0
4 0
5 0

6 0
7 0
8 3
9 0
G-R6:ALA-CA
3 0
4 0
5 12
6 20
7 28
8 55
9 140
FMU-P:VAL-S1
3 0
4 0
5 0
6 0
7 0
8 0
9 3
A-RIB:ALA-CA
3 0
4 0
5 4
6 48
7 88
8 64
9 124
U34-P:PRO-S1
3 0
4 0
5 0
6 0
7 0
8 3
9 0
C31-P:GLU-S1
3 0
4 0
5 0
6 0
7 3
8 0
9 0
A-P:GLY-CA
3 0
4 20
5 148
6 72
7 108
8 288
9 272
H2U-MY:ILE-CA
3 0
4 0
5 0
6 0
7 0

8 0
9 3
G-R5:GLN-CA
3 0
4 0
5 0
6 0
7 8
8 28
9 128
H2U-MY:TRP-S1
3 0
4 0
5 0
6 0
7 3
8 0
9 0
C-RIB:TYR-CA
3 0
4 0
5 0
6 12
7 27
8 51
9 53
DA-RIB:ARG-S2
3 0
4 0
5 0
6 0
7 0
8 0
9 3
FHU-RIB:ALA-S1
3 0
4 0
5 0
6 3
7 0
8 6
9 6
C-Y:ASN-CA
3 0
4 0
5 3
6 3
7 18
8 24
9 81
U-P:LEU-S2
3 0
4 0
5 0
6 9
7 27
8 60
9 75

U31-MY:ILE-S1
3 0
4 0
5 0
6 0
7 0
8 3
9 0
G-R5:GLN-S2
3 0
4 4
5 12
6 48
7 68
8 88
9 107
H2U-MY:TRP-CA
3 0
4 0
5 0
6 0
7 3
8 0
9 0
A-R5:GLU-S1
3 0
4 0
5 4
6 12
7 40
8 56
9 104
A-R6:VAL-S1
3 0
4 0
5 24
6 52
7 16
8 60
9 96
FMU-MY:PRO-S1
3 0
4 0
5 0
6 0
7 0
8 0
9 3
U34-MY:ASN-CA
3 0
4 0
5 0
6 0
7 0
8 0
9 3
U-Y:TYR-S1
3 0

4 0
5 12
6 0
7 18
8 21
9 20
C31-MY:ALA-CA
3 0
4 0
5 0
6 0
7 0
8 0
9 3
5BU-P:ARG-S1
3 0
4 0
5 0
6 0
7 3
8 0
9 0
QUO-M5:ASN-S2
3 0
4 0
5 4
6 0
7 0
8 0
9 0
5BU-MY:ILE-S1
3 0
4 0
5 0
6 0
7 3
8 0
9 0
A-RIB:LEU-CA
3 0
4 0
5 0
6 24
7 52
8 60
9 104
H2U-P:LEU-S1
3 0
4 0
5 0
6 0
7 0
8 0
9 3
IU-P:LEU-S1
3 0
4 0
5 0

6 0
7 0
8 3
9 0
U31-RIB:ASN-CA
3 0
4 0
5 0
6 0
7 0
8 3
9 0
G-R5:VAL-CA
3 0
4 0
5 0
6 12
7 20
8 28
9 76
H2U-MY:GLU-S1
3 0
4 0
5 3
6 0
7 0
8 0
9 0
U34-P:GLU-S2
3 0
4 0
5 0
6 3
7 0
8 0
9 0
A-R6:ARG-S1
3 0
4 0
5 32
6 72
7 52
8 88
9 231
A-R5:ALA-S1
3 0
4 12
5 0
6 12
7 56
8 52
9 124
IU-MY:ALA-S1
3 0
4 0
5 2
6 0
7 0

8 3
9 0
FHU-P:PRO-CA
3 0
4 0
5 0
6 0
7 3
8 0
9 0
QUO-RIB:LYS-S1
3 0
4 0
5 0
6 0
7 0
8 4
9 0
FMU-MY:ALA-S1
3 0
4 0
5 0
6 0
7 3
8 0
9 0
FHU-MY:CYS-S1
3 0
4 0
5 0
6 0
7 0
8 0
9 3
A-R6:ARG-CA
3 0
4 0
5 12
6 28
7 68
8 100
9 175
C-Y:GLN-S1
3 0
4 3
5 0
6 3
7 33
8 42
9 87
A-R6:LYS-S1
3 0
4 12
5 16
6 28
7 48
8 84
9 180

U-Y:MET-CA
3 0
4 0
5 3
6 6
7 9
8 6
9 18
FHU-MY:PRO-S1
3 0
4 0
5 0
6 0
7 0
8 3
9 0
IU-MY:ARG-CA
3 0
4 0
5 0
6 0
7 0
8 3
9 2
G-RIB:ALA-CA
3 0
4 0
5 8
6 56
7 68
8 92
9 203
C-RIB:GLY-CA
3 0
4 9
5 57
6 81
7 129
8 168
9 243
IU-P:ARG-S1
3 0
4 0
5 0
6 0
7 0
8 6
9 0
5BU-RIB:PRO-CA
3 0
4 0
5 0
6 0
7 3
8 0
9 0
G-P:ALA-S1
3 0

4 12
5 60
6 60
7 56
8 120
9 156
U-RIB:ARG-CA
3 0
4 0
5 9
6 27
7 39
8 66
9 141
G-P:GLU-S2
3 0
4 0
5 16
6 36
7 88
8 96
9 204
I-RIB:ALA-CA
3 0
4 0
5 0
6 0
7 0
8 0
9 2
GTP-RIB:ARG-S2
3 0
4 0
5 0
6 0
7 0
8 0
9 3
FHU-P:CYS-S1
3 0
4 0
5 0
6 0
7 0
8 0
9 3
U34-P:ARG-S2
3 0
4 0
5 0
6 0
7 3
8 0
9 0
G-R5:TRP-S1
3 0
4 4
5 0

6 12
7 12
8 16
9 44
U-RIB:ASP-S2
3 0
4 0
5 18
6 15
7 21
8 42
9 63
G-R6:PHE-S2
3 0
4 8
5 0
6 19
7 4
8 16
9 55
QUO-M5:LEU-S2
3 0
4 0
5 4
6 0
7 0
8 0
9 4
H2U-MY:GLN-S1
3 0
4 0
5 0
6 0
7 3
8 0
9 0
A-R5:LEU-S1
3 0
4 0
5 0
6 36
7 24
8 64
9 96
FHU-P:LEU-S2
3 0
4 0
5 0
6 0
7 3
8 3
9 3
C-P:CYS-CA
3 0
4 0
5 3
6 0
7 3

8 9
9 15
FHU-P:ASP-CA
3 0
4 0
5 0
6 0
7 0
8 6
9 3
U-P:PRO-CA
3 0
4 6
5 21
6 9
7 39
8 54
9 57
IU-RIB:ARG-CA
3 0
4 0
5 0
6 2
7 0
8 3
9 0
U-RIB:PHE-S2
3 0
4 0
5 3
6 39
7 33
8 11
9 42
FMU-MY:ARG-CA
3 0
4 0
5 0
6 0
7 0
8 3
9 0
QUO-M5:ARG-S1
3 0
4 0
5 0
6 0
7 0
8 0
9 4
U31-RIB:PHE-S1
3 0
4 0
5 0
6 0
7 0
8 3
9 0

FHU-RIB:LEU-CA
3 0
4 0
5 0
6 6
7 0
8 0
9 0
GTP-M5:ASP-S2
3 0
4 0
5 0
6 0
7 0
8 0
9 3
H2U-P:PHE-S1
3 0
4 0
5 0
6 0
7 0
8 0
9 3
C31-RIB:GLU-CA
3 0
4 0
5 0
6 3
7 0
8 0
9 0
G-P:PHE-CA
3 0
4 0
5 12
6 12
7 24
8 40
9 96
GTP-RIB:SER-CA
3 0
4 0
5 0
6 0
7 0
8 0
9 6
GTP-M5:ALA-CA
3 0
4 0
5 0
6 0
7 0
8 0
9 3
H2U-MY:PHE-S2
3 0

4 3
5 0
6 0
7 0
8 0
9 0
A-RIB:PHE-S2
3 0
4 0
5 24
6 32
7 36
8 28
9 52
DA-RIB:SER-S1
3 0
4 0
5 0
6 0
7 0
8 0
9 3
QUO-M5:PHE-S1
3 0
4 0
5 0
6 0
7 0
8 4
9 4
C31-RIB:ASP-CA
3 0
4 0
5 0
6 0
7 3
8 0
9 0
C-P:GLN-CA
3 0
4 0
5 15
6 21
7 36
8 78
9 117
FHU-P:THR-S1
3 0
4 0
5 0
6 0
7 3
8 6
9 0
FMU-MY:ASN-CA
3 0
4 0
5 0

6 0
7 0
8 0
9 3
FMU-MY:ASP-S1
3 0
4 0
5 0
6 0
7 3
8 0
9 0
U31-RIB:GLU-S2
3 0
4 0
5 0
6 0
7 3
8 0
9 0
5BU-RIB:SER-S1
3 0
4 0
5 0
6 0
7 0
8 0
9 3
C31-RIB:LEU-CA
3 0
4 0
5 0
6 3
7 0
8 0
9 0
A-R6:GLU-S1
3 0
4 0
5 12
6 24
7 40
8 60
9 100
DA-RIB:HIS-S2
3 0
4 0
5 0
6 3
7 0
8 0
9 0
U31-MY:PHE-CA
3 0
4 0
5 0
6 0
7 3

8 0
9 0
U34-P:ASN-CA
3 0
4 0
5 0
6 3
7 0
8 0
9 9
U-Y:GLN-S1
3 0
4 0
5 0
6 0
7 27
8 65
9 56
G-RIB:CYS-S1
3 0
4 0
5 0
6 0
7 8
8 0
9 16
GTP-RIB:GLY-CA
3 0
4 0
5 0
6 0
7 3
8 0
9 0
U31-MY:ALA-CA
3 0
4 0
5 0
6 0
7 0
8 3
9 0
U-Y:PHE-S1
3 0
4 0
5 6
6 8
7 21
8 18
9 30
FHU-MY:ARG-S2
3 0
4 0
5 0
6 6
7 0
8 6
9 0

H2U-MY:GLU-CA
3 0
4 0
5 0
6 3
7 0
8 0
9 0
A-RIB:THR-S1
3 0
4 0
5 32
6 72
7 60
8 116
9 140
FMU-MY:MET-CA
3 0
4 0
5 0
6 0
7 0
8 0
9 3
5BU-P:ALA-S1
3 0
4 0
5 0
6 0
7 0
8 0
9 3
IU-RIB:PRO-S1
3 0
4 0
5 0
6 0
7 3
8 2
9 0
H2U-MY:LEU-S1
3 0
4 0
5 0
6 0
7 0
8 3
9 3
IU-RIB:LEU-CA
3 0
4 0
5 0
6 2
7 0
8 0
9 4
H2U-P:ASN-CA
3 0

4 0
5 0
6 0
7 0
8 6
9 0
G-RIB:SER-S1
3 0
4 4
5 60
6 47
7 108
8 128
9 224
U34-MY:SER-CA
3 0
4 0
5 0
6 6
7 0
8 0
9 3
5BU-P:ARG-CA
3 0
4 0
5 0
6 0
7 0
8 0
9 6
FHU-RIB:LYS-CA
3 0
4 0
5 0
6 0
7 0
8 0
9 3
FHU-P:GLY-CA
3 0
4 3
5 6
6 0
7 3
8 3
9 0
QUO-RIB:GLN-S2
3 0
4 0
5 0
6 0
7 0
8 0
9 4
G-R5:GLU-S1
3 0
4 0
5 8

6 8
7 35
8 35
9 123
U31-P:HIS-S1
3 0
4 0
5 0
6 0
7 0
8 3
9 0
DA-RIB:VAL-CA
3 0
4 0
5 0
6 0
7 0
8 3
9 0
A-R5:TYR-S2
3 0
4 8
5 12
6 16
7 40
8 60
9 44
U-Y:TRP-S1
3 0
4 0
5 0
6 9
7 3
8 6
9 12
A-R5:LYS-S1
3 0
4 4
5 20
6 20
7 28
8 120
9 256
FMU-RIB:HIS-S2
3 0
4 0
5 0
6 0
7 0
8 3
9 0
IU-RIB:ILE-S1
3 0
4 0
5 0
6 0
7 2

8   3
9   0
DA-M5:ASN-CA
3   0
4   0
5   0
6   0
7   0
8   0
9   3
C-RIB:ALA-CA
3   0
4   0
5   30
6   51
7   96
8   66
9   156
H2U-MY:ASN-S2
3   0
4   0
5   3
6   0
7   3
8   0
9   6
FHU-P:LYS-S1
3   0
4   0
5   3
6   6
7   3
8   0
9   0
DA-M6:THR-CA
3   0
4   0
5   0
6   0
7   0
8   0
9   6
DA-M5:HIS-S2
3   0
4   3
5   0
6   0
7   0
8   0
9   0
GTP-RIB:THR-CA
3   0
4   0
5   0
6   0
7   0
8   0
9   3

A-R6:GLN-CA
3 0
4 0
5 0
6 24
7 28
8 24
9 76
IU-P:ARG-CA
3 0
4 0
5 0
6 0
7 3
8 0
9 3
U31-MY:TYR-CA
3 0
4 0
5 0
6 0
7 0
8 3
9 0
C-P:TRP-S2
3 0
4 0
5 6
6 9
7 18
8 15
9 33
C-RIB:CYS-S1
3 0
4 0
5 0
6 0
7 12
8 9
9 12
QUO-M5:GLU-S2
3 0
4 0
5 0
6 0
7 4
8 0
9 0
U-Y:ALA-CA
3 0
4 0
5 9
6 20
7 18
8 32
9 72
FHU-P:ARG-S1
3 0

4 0
5 0
6 0
7 0
8 3
9 3
FMU-RIB:MET-S2
3 0
4 0
5 0
6 0
7 0
8 0
9 3
FMU-MY:GLN-S1
3 0
4 0
5 0
6 0
7 3
8 0
9 0
IU-RIB:LEU-S2
3 0
4 0
5 0
6 2
7 0
8 0
9 0
G-P:CYS-S1
3 0
4 0
5 0
6 4
7 4
8 0
9 12
H2U-MY:LYS-S2
3 0
4 0
5 0
6 0
7 3
8 0
9 0
GTP-M5:THR-S1
3 0
4 0
5 0
6 0
7 0
8 0
9 3
H2U-P:PRO-CA
3 0
4 0
5 0

6 0
7 0
8 0
9 3
FMU-MY:GLU-S1
3 0
4 0
5 0
6 0
7 3
8 0
9 0
IU-P:HIS-S1
3 0
4 0
5 3
6 0
7 0
8 0
9 0
C-P:ARG-CA
3 0
4 0
5 42
6 75
7 168
8 213
9 300
QUO-M6:LEU-CA
3 0
4 0
5 0
6 0
7 0
8 0
9 4
U-Y:ASP-S2
3 0
4 0
5 3
6 3
7 24
8 48
9 48
OMC-P:LYS-S2
3 0
4 0
5 3
6 0
7 0
8 0
9 3
FHU-MY:ILE-S1
3 0
4 0
5 0
6 6
7 0

8 0
9 0
FHU-MY:ASP-CA
3 0
4 0
5 0
6 0
7 6
8 0
9 0
A-RIB:CYS-CA
3 0
4 0
5 0
6 4
7 16
8 16
9 12
U34-P:HIS-S1
3 0
4 0
5 0
6 0
7 0
8 3
9 0
FHU-RIB:LYS-S1
3 0
4 0
5 0
6 0
7 0
8 0
9 6
GTP-M6:SER-CA
3 0
4 0
5 0
6 0
7 0
8 0
9 3
FMU-RIB:GLN-S2
3 0
4 0
5 0
6 0
7 0
8 3
9 0
FMU-MY:PHE-S2
3 0
4 0
5 0
6 3
7 3
8 0
9 0

FMU-P:ASP-S1
3 0
4 0
5 0
6 0
7 0
8 3
9 0
FMU-RIB:HIS-S1
3 0
4 0
5 0
6 0
7 0
8 3
9 0
QUO-M6:ASN-S2
3 0
4 0
5 4
6 0
7 0
8 0
9 0
H2U-P:LEU-S2
3 0
4 0
5 0
6 0
7 3
8 0
9 0
QUO-M6:ARG-CA
3 0
4 0
5 0
6 0
7 4
8 0
9 0
A-P:THR-CA
20 0
40 4
60 12
80 28
100 60
120 44
140 48
160 20
180 4
200 0
220 4
240 36
260 28
280 32
300 32
320 24
340 4

360   24
U-RIB:SER-CA
20   0
40   9
60   21
80   18
100   30
120   24
140   6
160   15
180   6
200   3
220   6
240   18
260   15
280   21
300   15
320   18
340   6
360   6
C31-RIB:LEU-CA
20   0
40   0
60   0
80   0
100   0
120   0
140   0
160   0
180   0
200   0
220   0
240   0
260   0
280   0
300   3
320   0
340   0
360   0
C-Y:ASP-S1
20   9
40   18
60   18
80   24
100   24
120   6
140   12
160   12
180   0
200   6
220   15
240   18
260   24
280   9
300   6
320   24
340   9
360   3

C-RIB:CYS-S1
20   0
40   0
60   3
80   0
100   6
120   0
140   3
160   0
180   0
200   0
220   0
240   0
260   6
280   12
300   0
320   3
340   0
360   0
U-Y:HIS-S2
20   6
40   0
60   15
80   12
100   18
120   15
140   9
160   0
180   0
200   0
220   6
240   18
260   9
280   6
300   3
320   3
340   0
360   6
G-RIB:LYS-S1
20   4
40   24
60   72
80   96
100   96
120   64
140   52
160   20
180   4
200   16
220   24
240   100
260   88
280   48
300   72
320   44
340   24
360   16
C-RIB:TRP-S1

20 0
40 0
60 3
80 6
100 15
120 9
140 0
160 0
180 3
200 0
220 0
240 0
260 6
280 9
300 0
320 0
340 9
360 3
OMC-RIB:LYS-S2
20 0
40 0
60 0
80 0
100 0
120 0
140 0
160 0
180 0
200 0
220 0
240 3
260 0
280 3
300 0
320 0
340 0
360 0
A-RIB:PRO-S1
20 0
40 4
60 32
80 16
100 32
120 52
140 28
160 32
180 4
200 0
220 0
240 12
260 20
280 40
300 20
320 28
340 20
360 20
G-RIB:ALA-S1
20 16

40   4
60   20
80   44
100   40
120   48
140   40
160   24
180   16
200   8
220   28
240   28
260   28
280   24
300   40
320   16
340   28
360   4
U-RIB:ARG-S1
20   6
40   12
60   36
80   45
100   33
120   39
140   24
160   36
180   15
200   0
220   15
240   33
260   42
280   24
300   36
320   30
340   21
360   3
C-P:ARG-CA
20   0
40   21
60   15
80   69
100   69
120   99
140   60
160   48
180   27
200   0
220   3
240   21
260   51
280   81
300   84
320   90
340   39
360   21
C-RIB:GLU-S1
20   3
40   3

60 21
80 24
100 3
120 15
140 21
160 15
180 9
200 3
220 0
240 9
260 21
280 24
300 21
320 12
340 18
360 12
IU-MY:TYR-CA
20 0
40 0
60 0
80 0
100 0
120 2
140 0
160 0
180 0
200 0
220 0
240 0
260 0
280 0
300 0
320 0
340 0
360 0
G-RIB:ASP-S1
20 8
40 4
60 24
80 16
100 36
120 40
140 20
160 24
180 20
200 4
220 4
240 12
260 32
280 32
300 32
320 36
340 28
360 24
G-R6:VAL-S1
20 0
40 4
60 0

80 4
100 20
120 8
140 0
160 0
180 12
200 0
220 4
240 20
260 23
280 7
300 20
320 16
340 4
360 8
U-RIB:GLU-S2
20 0
40 0
60 6
80 6
100 9
120 12
140 9
160 6
180 6
200 0
220 3
240 12
260 6
280 30
300 12
320 6
340 15
360 6
A-P:LEU-CA
20 0
40 4
60 8
80 16
100 12
120 20
140 8
160 16
180 0
200 0
220 8
240 8
260 16
280 16
300 36
320 8
340 24
360 4
G-R5:CYS-S1
20 0
40 0
60 0
80 0

100 0
120 4
140 0
160 4
180 0
200 0
220 4
240 0
260 0
280 0
300 0
320 0
340 0
360 0
A-P:ASN-S2
20 4
40 4
60 12
80 44
100 44
120 44
140 20
160 28
180 4
200 0
220 0
240 36
260 24
280 28
300 24
320 20
340 24
360 0
U-Y:GLU-CA
20 0
40 6
60 3
80 9
100 3
120 3
140 3
160 3
180 3
200 3
220 3
240 6
260 3
280 6
300 3
320 3
340 0
360 0
G-RIB:HIS-CA
20 0
40 4
60 4
80 16
100 16

120   4
140   40
160   0
180   12
200   0
220   8
240   4
260   48
280   32
300   8
320   16
340   20
360   8
A-P:VAL-CA
20   4
40   0
60   16
80   24
100   28
120   36
140   12
160   16
180   4
200   0
220   0
240   8
260   12
280   44
300   24
320   20
340   16
360   12
C31-P:THR-CA
20   0
40   0
60   3
80   0
100   3
120   0
140   0
160   0
180   0
200   0
220   0
240   0
260   0
280   0
300   0
320   0
340   0
360   0
A-RIB:ASN-S1
20   0
40   4
60   8
80   36
100   36
120   20

140 32
160 20
180 4
200 0
220 20
240 32
260 16
280 32
300 20
320 40
340 24
360 16
C-P:TRP-S2
20 0
40 0
60 6
80 6
100 12
120 9
140 6
160 6
180 0
200 0
220 0
240 6
260 15
280 0
300 12
320 0
340 3
360 0
C-Y:LYS-CA
20 3
40 12
60 24
80 27
100 33
120 18
140 12
160 15
180 6
200 3
220 9
240 21
260 21
280 3
300 15
320 6
340 6
360 6
A-R6:ILE-S1
20 4
40 8
60 8
80 8
100 0
120 4
140 12

160   0
180   4
200   0
220   0
240   4
260   12
280   4
300   20
320   16
340   16
360   0
U-RIB:ARG-S2
20   18
40   27
60   51
80   84
100   39
120   42
140   39
160   24
180   18
200   6
220   36
240   63
260   48
280   51
300   27
320   30
340   18
360   18
G-R6:MET-S1
20   0
40   4
60   0
80   28
100   8
120   4
140   0
160   0
180   0
200   0
220   4
240   4
260   16
280   12
300   12
320   4
340   12
360   0
U-RIB:THR-S1
20   12
40   12
60   9
80   24
100   27
120   12
140   18
160   3

180 9
200 0
220 6
240 9
260 9
280 15
300 15
320 24
340 6
360 0
A-R5:MET-CA
20 0
40 4
60 12
80 12
100 20
120 4
140 4
160 4
180 0
200 0
220 4
240 8
260 4
280 0
300 8
320 8
340 8
360 4
C-RIB:ASP-S1
20 0
40 0
60 18
80 15
100 39
120 30
140 27
160 9
180 15
200 0
220 3
240 9
260 42
280 27
300 9
320 42
340 24
360 18
G-RIB:LEU-S1
20 0
40 4
60 20
80 24
100 36
120 28
140 12
160 20
180 4

200 0
220 20
240 8
260 20
280 40
300 24
320 20
340 8
360 8
G-P:LYS-S1
20 0
40 0
60 36
80 100
100 108
120 112
140 104
160 76
180 40
200 0
220 12
240 56
260 84
280 100
300 76
320 92
340 80
360 40
FHU-RIB:ALA-S1
20 0
40 0
60 0
80 0
100 6
120 3
140 0
160 0
180 0
200 0
220 0
240 0
260 0
280 3
300 3
320 0
340 0
360 0
IU-MY:ARG-S2
20 0
40 0
60 0
80 0
100 0
120 3
140 6
160 3
180 0
200 0

220 0
240 0
260 0
280 0
300 0
320 0
340 0
360 0
G-RIB:TRP-CA
20 0
40 8
60 0
80 8
100 8
120 0
140 8
160 12
180 0
200 0
220 0
240 12
260 8
280 20
300 12
320 8
340 0
360 0
U-P:GLU-S2
20 0
40 3
60 9
80 15
100 21
120 30
140 9
160 12
180 15
200 0
220 6
240 9
260 6
280 15
300 18
320 30
340 12
360 9
A-R6:GLU-S2
20 0
40 20
60 24
80 24
100 16
120 28
140 16
160 8
180 12
200 0
220 4

240 12
260 20
280 40
300 20
320 8
340 4
360 12
U-Y:ASN-S2
20 3
40 6
60 15
80 24
100 24
120 24
140 9
160 6
180 0
200 6
220 9
240 27
260 24
280 17
300 18
320 12
340 9
360 3
G-R5:LYS-S1
20 12
40 43
60 40
80 28
100 36
120 31
140 16
160 16
180 20
200 24
220 8
240 28
260 28
280 44
300 68
320 48
340 48
360 0
C-P:PRO-CA
20 0
40 18
60 12
80 18
100 21
120 39
140 30
160 15
180 6
200 0
220 0
240 12

260 24
280 45
300 18
320 18
340 24
360 12
C-P:ARG-S1
20 3
40 24
60 36
80 54
100 105
120 93
140 69
160 48
180 42
200 0
220 15
240 33
260 63
280 129
300 123
320 66
340 66
360 18
U-Y:LYS-S2
20 9
40 9
60 36
80 33
100 48
120 12
140 9
160 21
180 12
200 9
220 15
240 51
260 39
280 30
300 24
320 17
340 27
360 9
A-R6:GLN-CA
20 0
40 0
60 8
80 4
100 36
120 8
140 16
160 8
180 8
200 4
220 0
240 4
260 16

280 0
300 12
320 20
340 8
360 0
G-P:ASN-S2
20 0
40 16
60 40
80 36
100 44
120 40
140 32
160 44
180 20
200 0
220 12
240 28
260 44
280 84
300 52
320 28
340 28
360 4
U-RIB:LYS-S2
20 12
40 30
60 36
80 42
100 48
120 36
140 15
160 9
180 9
200 18
220 54
240 48
260 57
280 60
300 33
320 18
340 24
360 12
U-P:MET-S2
20 0
40 0
60 6
80 12
100 3
120 0
140 9
160 6
180 0
200 0
220 6
240 3
260 12
280 9

300 3
320 6
340 6
360 0
C-P:ALA-S1
20 0
40 27
60 18
80 36
100 45
120 18
140 42
160 21
180 9
200 0
220 21
240 33
260 21
280 33
300 48
320 39
340 15
360 12
C-P:VAL-S1
20 0
40 6
60 21
80 30
100 45
120 21
140 30
160 18
180 6
200 0
220 12
240 27
260 30
280 21
300 21
320 42
340 9
360 3
C-Y:GLY-CA
20 18
40 27
60 54
80 30
100 36
120 15
140 15
160 21
180 6
200 24
220 27
240 39
260 48
280 42
300 21

320 21
340 0
360 3
G-R5:ASN-S1
20 8
40 24
60 27
80 39
100 20
120 20
140 20
160 15
180 4
200 12
220 28
240 19
260 36
280 12
300 24
320 12
340 8
360 3
QUO-M5:LYS-S1
20 0
40 0
60 0
80 4
100 0
120 0
140 0
160 0
180 0
200 0
220 0
240 0
260 0
280 0
300 0
320 0
340 0
360 0
U-Y:ARG-S1
20 6
40 6
60 15
80 39
100 32
120 33
140 18
160 9
180 6
200 0
220 9
240 6
260 24
280 15
300 9
320 12

340   21
360   9
G-RIB:GLN-S2
20   0
40   12
60   36
80   20
100   40
120   20
140   36
160   28
180   24
200   8
220   16
240   16
260   20
280   52
300   32
320   16
340   24
360   36
G-R5:ASP-S2
20   0
40   12
60   20
80   40
100   16
120   16
140   28
160   12
180   4
200   0
220   44
240   20
260   64
280   48
300   12
320   32
340   16
360   4
G-RIB:ARG-S2
20   44
40   72
60   152
80   112
100   100
120   108
140   68
160   8
180   16
200   28
220   92
240   116
260   144
280   128
300   128
320   48
340   52

360 28
C-P:LYS-CA
20 0
40 6
60 18
80 45
100 51
120 57
140 66
160 42
180 12
200 0
220 9
240 12
260 36
280 42
300 69
320 75
340 48
360 15
U-P:ASN-S1
20 3
40 0
60 12
80 3
100 30
120 24
140 27
160 15
180 3
200 0
220 6
240 9
260 18
280 18
300 12
320 12
340 18
360 3
G-P:LEU-S1
20 0
40 4
60 20
80 28
100 32
120 28
140 20
160 8
180 12
200 4
220 4
240 36
260 20
280 28
300 28
320 28
340 8
360 4

G-R6:LEU-S1
20 4
40 8
60 12
80 16
100 0
120 20
140 12
160 8
180 0
200 0
220 8
240 4
260 24
280 24
300 12
320 4
340 0
360 0
A-P:GLU-S2
20 4
40 12
60 32
80 16
100 12
120 24
140 36
160 4
180 8
200 0
220 20
240 16
260 24
280 20
300 48
320 28
340 20
360 4
U-RIB:VAL-S1
20 0
40 3
60 9
80 15
100 18
120 18
140 9
160 6
180 3
200 0
220 0
240 3
260 15
280 12
300 3
320 21
340 6
360 0
C-RIB:ASN-S1

20   0
40   15
60   27
80   33
100   39
120   24
140   21
160   9
180   6
200   3
220   12
240   39
260   18
280   24
300   24
320   21
340   6
360   12
A-R6:GLN-S2
20   0
40   8
60   16
80   28
100   28
120   32
140   20
160   16
180   4
200   4
220   8
240   20
260   20
280   8
300   28
320   32
340   8
360   4
C-RIB:MET-S2
20   0
40   0
60   9
80   15
100   12
120   15
140   9
160   18
180   6
200   0
220   3
240   6
260   12
280   15
300   9
320   15
340   9
360   0
A-P:SER-CA
20   0

40   4
60   40
80   28
100   48
120   36
140   52
160   32
180   4
200   0
220   8
240   16
260   24
280   40
300   48
320   32
340   48
360   24
U-RIB:LEU-CA
20   0
40   0
60   12
80   0
100   12
120   15
140   15
160   9
180   12
200   0
220   0
240   6
260   6
280   9
300   12
320   12
340   12
360   0
U-Y:LYS-S1
20   6
40   3
60   12
80   18
100   12
120   12
140   9
160   9
180   3
200   3
220   18
240   21
260   27
280   30
300   17
320   18
340   26
360   6
U-RIB:TYR-S1
20   0
40   0

60 0
80 12
100 3
120 3
140 15
160 6
180 0
200 0
220 0
240 0
260 0
280 27
300 3
320 3
340 15
360 3
A-R6:ASP-S2
20 0
40 12
60 16
80 12
100 16
120 16
140 12
160 16
180 4
200 4
220 8
240 28
260 12
280 40
300 36
320 12
340 8
360 8
G-P:TYR-CA
20 0
40 0
60 12
80 20
100 20
120 12
140 12
160 8
180 8
200 0
220 0
240 4
260 12
280 16
300 12
320 28
340 16
360 0
A-R6:THR-CA
20 0
40 4
60 16

80 8
100 28
120 20
140 16
160 8
180 8
200 0
220 8
240 24
260 24
280 12
300 12
320 12
340 8
360 0
A-R6:ALA-S1
20 0
40 8
60 24
80 20
100 16
120 16
140 12
160 8
180 0
200 0
220 12
240 16
260 8
280 16
300 32
320 12
340 12
360 4
U-RIB:ASP-CA
20 0
40 3
60 6
80 9
100 3
120 24
140 6
160 9
180 6
200 3
220 3
240 3
260 3
280 18
300 21
320 15
340 15
360 6
A-P:ARG-S1
20 0
40 16
60 48
80 72

100 108
120 120
140 76
160 72
180 36
200 4
220 8
240 44
260 104
280 104
300 116
320 76
340 68
360 28
C-RIB:LEU-S1
20 0
40 6
60 15
80 30
100 15
120 18
140 30
160 18
180 6
200 0
220 0
240 9
260 39
280 27
300 45
320 24
340 12
360 6
U-RIB:LYS-S1
20 0
40 15
60 21
80 36
100 33
120 51
140 9
160 30
180 12
200 3
220 21
240 39
260 54
280 33
300 18
320 30
340 21
360 9
A-RIB:GLU-CA
20 0
40 8
60 0
80 36
100 36

120   32
140   20
160   8
180   0
200   0
220   0
240   0
260   16
280   16
300   28
320   12
340   16
360   4
A-R6:ASP-S1
20   0
40   8
60   8
80   8
100   16
120   20
140   16
160   12
180   0
200   0
220   8
240   12
260   16
280   24
300   28
320   16
340   4
360   4
G-RIB:SER-S1
20   12
40   16
60   40
80   64
100   52
120   20
140   48
160   20
180   12
200   12
220   32
240   20
260   32
280   48
300   36
320   40
340   40
360   24
A-R5:LYS-S1
20   12
40   8
60   28
80   40
100   48
120   20

140   32
160   4
180   0
200   16
220   36
240   44
260   44
280   24
300   56
320   24
340   8
360   4
U-P:ARG-CA
20   0
40   3
60   9
80   18
100   48
120   45
140   66
160   24
180   12
200   0
220   9
240   12
260   12
280   48
300   57
320   27
340   21
360   12
G-R6:ASP-CA
20   0
40   4
60   20
80   19
100   16
120   8
140   4
160   16
180   4
200   0
220   12
240   8
260   20
280   40
300   16
320   8
340   16
360   8
C-Y:LYS-S2
20   18
40   30
60   42
80   78
100   36
120   21
140   18

160 18
180 6
200 15
220 24
240 42
260 45
280 39
300 27
320 30
340 27
360 6
U-RIB:ASN-CA
20 6
40 3
60 6
80 15
100 9
120 15
140 12
160 12
180 9
200 3
220 9
240 15
260 12
280 15
300 15
320 18
340 9
360 15
C-RIB:TRP-CA
20 0
40 0
60 6
80 3
100 9
120 15
140 0
160 0
180 3
200 0
220 0
240 0
260 0
280 9
300 0
320 0
340 6
360 3
A-P:TYR-S2
20 0
40 20
60 16
80 24
100 12
120 8
140 24
160 4

180 4
200 0
220 12
240 28
260 20
280 8
300 20
320 16
340 8
360 4
C-RIB:THR-S1
20 3
40 15
60 15
80 27
100 24
120 33
140 24
160 9
180 9
200 3
220 9
240 36
260 24
280 24
300 36
320 42
340 27
360 9
U-P:SER-S1
20 3
40 6
60 6
80 33
100 15
120 33
140 18
160 15
180 3
200 0
220 6
240 15
260 27
280 30
300 27
320 48
340 21
360 9
C-Y:ALA-S1
20 6
40 18
60 21
80 24
100 18
120 9
140 6
160 12
180 0

200 21
220 24
240 33
260 27
280 9
300 24
320 15
340 6
360 0
C-RIB:PRO-S1
20 15
40 6
60 27
80 30
100 21
120 24
140 18
160 12
180 9
200 0
220 12
240 15
260 21
280 30
300 27
320 45
340 3
360 3
G-R5:VAL-CA
20 0
40 4
60 4
80 4
100 20
120 8
140 8
160 4
180 4
200 8
220 8
240 20
260 8
280 20
300 4
320 4
340 8
360 0
G-R6:VAL-CA
20 0
40 0
60 4
80 4
100 24
120 12
140 4
160 0
180 4
200 0

220 4
240 12
260 16
280 19
300 12
320 12
340 20
360 0
A-R6:HIS-S1
20 4
40 0
60 12
80 16
100 8
120 20
140 4
160 12
180 0
200 0
220 4
240 28
260 16
280 28
300 4
320 20
340 4
360 8
U31-RIB:TYR-S1
20 0
40 0
60 0
80 0
100 0
120 0
140 0
160 0
180 0
200 0
220 0
240 0
260 0
280 3
300 0
320 0
340 0
360 0
C-P:ILE-S1
20 0
40 6
60 12
80 21
100 21
120 9
140 15
160 6
180 3
200 0
220 3

240   12
260   12
280   18
300   12
320   24
340   0
360   3
A-P:ASP-S1
20   0
40   4
60   8
80   28
100   32
120   24
140   24
160   12
180   4
200   0
220   8
240   20
260   28
280   20
300   28
320   28
340   24
360   4
G-P:ARG-S2
20   0
40   36
60   56
80   156
100   124
120   148
140   124
160   116
180   20
200   0
220   16
240   48
260   96
280   176
300   132
320   108
340   128
360   40
C-RIB:ILE-CA
20   0
40   6
60   6
80   21
100   18
120   18
140   3
160   6
180   3
200   0
220   3
240   0

260 15
280 9
300 24
320 24
340 12
360 6
U-RIB:ASP-S2
20 0
40 3
60 6
80 15
100 9
120 9
140 18
160 15
180 6
200 0
220 6
240 9
260 9
280 12
300 15
320 12
340 9
360 6
A-RIB:VAL-CA
20 0
40 12
60 16
80 20
100 16
120 28
140 20
160 32
180 4
200 0
220 0
240 4
260 24
280 36
300 24
320 24
340 16
360 12
C-Y:HIS-S1
20 0
40 9
60 18
80 12
100 21
120 6
140 6
160 3
180 3
200 0
220 12
240 15
260 27

280 9
300 3
320 6
340 3
360 0
A-R5:GLU-S1
20 0
40 24
60 12
80 20
100 24
120 16
140 24
160 4
180 0
200 4
220 16
240 8
260 12
280 12
300 20
320 12
340 4
360 4
U-Y:VAL-S1
20 3
40 8
60 6
80 6
100 9
120 0
140 6
160 0
180 0
200 0
220 9
240 9
260 9
280 9
300 0
320 3
340 3
360 0
G-RIB:GLY-CA
20 4
40 24
60 76
80 48
100 104
120 76
140 68
160 60
180 16
200 12
220 28
240 44
260 72
280 88

300   60
320   64
340   52
360   32
C-P:HIS-S2
20   0
40   12
60   9
80   15
100   21
120   12
140   18
160   12
180   0
200   0
220   15
240   6
260   21
280   18
300   18
320   18
340   6
360   12
G-R6:ASN-S2
20   0
40   0
60   30
80   32
100   27
120   24
140   28
160   7
180   8
200   4
220   16
240   39
260   28
280   36
300   35
320   20
340   8
360   0
C-RIB:ASP-S2
20   0
40   6
60   12
80   33
100   27
120   27
140   33
160   39
180   12
200   0
220   6
240   18
260   39
280   21
300   33

320	36
340	21
360	18

G-RIB:ARG-CA

20	0
40	4
60	40
80	60
100	88
120	48
140	56
160	48
180	12
200	4
220	8
240	56
260	72
280	68
300	36
320	56
340	44
360	8

U-Y:ALA-CA

20	3
40	6
60	18
80	15
100	9
120	8
140	5
160	9
180	0
200	0
220	3
240	15
260	24
280	9
300	9
320	9
340	9
360	0

C-P:ALA-CA

20	0
40	12
60	24
80	30
100	45
120	30
140	27
160	36
180	6
200	3
220	9
240	27
260	30
280	45
300	54
320	30

340   24
360   0
A-P:ASN-S1
20   4
40   4
60   8
80   28
100   60
120   28
140   12
160   32
180   4
200   0
220   8
240   24
260   28
280   32
300   20
320   24
340   32
360   4
A-R5:TYR-S1
20   0
40   4
60   28
80   24
100   8
120   24
140   20
160   0
180   4
200   0
220   8
240   4
260   16
280   8
300   4
320   11
340   4
360   4
C-Y:LEU-S1
20   3
40   3
60   9
80   6
100   18
120   21
140   3
160   6
180   0
200   3
220   12
240   24
260   9
280   12
300   6
320   3
340   3

360 3
U-Y:ILE-CA
20 0
40 0
60 6
80 9
100 3
120 6
140 3
160 3
180 0
200 0
220 0
240 3
260 3
280 9
300 12
320 6
340 3
360 0
G-R6:PRO-CA
20 0
40 16
60 24
80 20
100 8
120 24
140 8
160 4
180 0
200 0
220 8
240 12
260 27
280 28
300 0
320 12
340 12
360 4
G-R5:TYR-S2
20 0
40 4
60 16
80 8
100 39
120 11
140 0
160 0
180 0
200 0
220 0
240 20
260 8
280 19
300 12
320 7
340 8
360 4

C-Y:THR-CA
20 3
40 6
60 9
80 18
100 11
120 3
140 12
160 3
180 3
200 9
220 3
240 30
260 12
280 6
300 6
320 9
340 9
360 0
A-P:GLU-S1
20 0
40 8
60 28
80 28
100 8
120 36
140 28
160 4
180 0
200 0
220 4
240 12
260 24
280 24
300 28
320 20
340 16
360 4
A-RIB:THR-S1
20 0
40 20
60 28
80 20
100 32
120 36
140 28
160 20
180 8
200 0
220 24
240 28
260 48
280 28
300 40
320 24
340 32
360 4
U-Y:SER-CA

20   0
40   9
60   18
80   20
100   9
120   12
140   3
160   3
180   0
200   3
220   6
240   9
260   10
280   12
300   5
320   15
340   15
360   3
G-RIB:THR-CA
20   8
40   12
60   8
80   32
100   28
120   36
140   28
160   16
180   4
200   12
220   16
240   16
260   16
280   12
300   24
320   20
340   16
360   16
A-R6:GLU-CA
20   4
40   4
60   0
80   20
100   16
120   8
140   24
160   16
180   0
200   0
220   12
240   12
260   12
280   8
300   32
320   36
340   0
360   0
FMU-P:ASP-CA
20   0

40   0
60   0
80   0
100   0
120   0
140   3
160   0
180   0
200   0
220   0
240   0
260   0
280   0
300   0
320   0
340   0
360   0
G-P:TRP-S2
20   0
40   8
60   8
80   12
100   12
120   8
140   12
160   16
180   0
200   0
220   4
240   4
260   8
280   16
300   12
320   16
340   4
360   4
A-P:MET-S2
20   4
40   4
60   16
80   8
100   4
120   8
140   8
160   4
180   0
200   0
220   0
240   12
260   8
280   20
300   4
320   8
340   4
360   0
G-RIB:LEU-CA
20   0
40   4

60 12
80 16
100 48
120 28
140 28
160 12
180 16
200 0
220 4
240 24
260 8
280 52
300 20
320 24
340 12
360 12
G-P:GLU-S1
20 0
40 4
60 4
80 24
100 32
120 40
140 40
160 40
180 4
200 0
220 12
240 8
260 32
280 4
300 24
320 24
340 24
360 8
C-P:PHE-S2
20 0
40 0
60 15
80 6
100 6
120 12
140 6
160 6
180 0
200 0
220 3
240 15
260 9
280 9
300 12
320 15
340 6
360 0
C-Y:GLN-S1
20 0
40 9
60 21

80   21
100   3
120   12
140   3
160   3
180   0
200   6
220   12
240   39
260   12
280   12
300   0
320   12
340   3
360   0
U-P:HIS-S2
20   0
40   6
60   6
80   21
100   12
120   6
140   12
160   9
180   12
200   0
220   12
240   18
260   18
280   24
300   15
320   15
340   3
360   3
G-P:TYR-S1
20   0
40   0
60   8
80   16
100   24
120   24
140   12
160   32
180   0
200   0
220   0
240   4
260   8
280   16
300   16
320   20
340   12
360   4
G-RIB:LYS-CA
20   12
40   20
60   52
80   72

100   88
120   52
140   16
160   28
180   16
200   8
220   40
240   20
260   84
280   32
300   64
320   52
340   24
360   12
C-P:TRP-CA
20   0
40   0
60   3
80   3
100   0
120   9
140   6
160   3
180   0
200   0
220   0
240   9
260   6
280   9
300   0
320   6
340   6
360   0
C-RIB:GLN-CA
20   0
40   6
60   12
80   21
100   12
120   21
140   18
160   9
180   9
200   0
220   3
240   6
260   21
280   24
300   39
320   24
340   30
360   3
U-Y:MET-CA
20   0
40   3
60   9
80   6
100   0

120 0
140 0
160 0
180 0
200 0
220 9
240 3
260 6
280 0
300 3
320 3
340 0
360 0
A-R5:GLY-CA
20 24
40 52
60 36
80 60
100 36
120 12
140 20
160 20
180 4
200 28
220 28
240 60
260 72
280 28
300 44
320 28
340 16
360 16
U34-P:HIS-CA
20 0
40 0
60 0
80 0
100 0
120 0
140 0
160 3
180 0
200 0
220 0
240 0
260 0
280 0
300 0
320 0
340 0
360 0
A-R6:VAL-CA
20 0
40 4
60 4
80 36
100 20
120 16

140 16
160 4
180 4
200 0
220 0
240 4
260 20
280 20
300 20
320 20
340 8
360 0
U-Y:THR-S1
20 0
40 9
60 18
80 6
100 24
120 15
140 6
160 9
180 0
200 3
220 9
240 12
260 9
280 3
300 9
320 3
340 0
360 0
U-RIB:HIS-S2
20 0
40 3
60 3
80 27
100 18
120 9
140 18
160 18
180 12
200 0
220 3
240 3
260 15
280 15
300 12
320 12
340 3
360 6
A-R5:ARG-S2
20 24
40 44
60 104
80 72
100 76
120 68
140 43

160   12
180   8
200   32
220   28
240   56
260   68
280   84
300   60
320   40
340   16
360   4
G-R5:LYS-CA
20   12
40   12
60   20
80   16
100   28
120   31
140   20
160   16
180   8
200   20
220   20
240   16
260   28
280   44
300   60
320   36
340   24
360   4
C-P:LEU-S1
20   0
40   3
60   21
80   21
100   15
120   36
140   15
160   6
180   3
200   0
220   3
240   3
260   33
280   21
300   21
320   12
340   9
360   3
G-R6:SER-CA
20   0
40   12
60   32
80   32
100   20
120   16
140   8
160   8

180   0
200   0
220   8
240   8
260   31
280   28
300   40
320   16
340   8
360   4
FHU-P:LEU-S2
20   0
40   0
60   0
80   3
100   0
120   0
140   0
160   0
180   0
200   0
220   0
240   3
260   0
280   0
300   0
320   3
340   0
360   0
C-Y:ARG-S1
20   9
40   18
60   42
80   57
100   66
120   24
140   41
160   12
180   18
200   6
220   36
240   39
260   51
280   48
300   27
320   21
340   12
360   6
FHU-RIB:ARG-S1
20   0
40   0
60   0
80   3
100   0
120   0
140   0
160   0
180   0

200   0
220   0
240   0
260   3
280   0
300   0
320   0
340   3
360   3
C-Y:ILE-S1
20   3
40   6
60   6
80   9
100   9
120   6
140   6
160   0
180   0
200   0
220   9
240   12
260   6
280   12
300   9
320   3
340   0
360   0
A-R6:HIS-S2
20   0
40   8
60   12
80   4
100   24
120   28
140   20
160   8
180   8
200   0
220   8
240   12
260   44
280   36
300   12
320   4
340   12
360   4
A-RIB:SER-CA
20   12
40   20
60   16
80   24
100   36
120   12
140   16
160   12
180   4
200   4

220   24
240   28
260   28
280   36
300   56
320   24
340   20
360   8
U31-MY:ASP-S2
20   0
40   0
60   0
80   3
100   0
120   3
140   0
160   0
180   0
200   0
220   0
240   3
260   0
280   3
300   0
320   0
340   0
360   0
G-R6:ARG-S1
20   0
40   12
60   28
80   64
100   52
120   39
140   28
160   28
180   0
200   0
220   12
240   28
260   40
280   44
300   60
320   24
340   20
360   16
G-R5:THR-CA
20   4
40   8
60   4
80   20
100   8
120   4
140   4
160   12
180   0
200   4
220   20

240   28
260   24
280   20
300   12
320   16
340   0
360   0
A-R5:ASN-CA
20   12
40   8
60   12
80   20
100   20
120   12
140   12
160   0
180   0
200   4
220   4
240   8
260   28
280   8
300   4
320   12
340   4
360   7
C-P:CYS-CA
20   0
40   0
60   0
80   6
100   0
120   0
140   3
160   0
180   3
200   0
220   0
240   3
260   3
280   3
300   0
320   9
340   0
360   0
A-RIB:HIS-S2
20   0
40   0
60   24
80   20
100   28
120   16
140   24
160   12
180   12
200   0
220   8
240   28

260 40
280 40
300 28
320 28
340 16
360 4
C-P:TYR-S1
20 0
40 12
60 15
80 21
100 6
120 15
140 9
160 3
180 3
200 0
220 0
240 3
260 6
280 15
300 18
320 6
340 9
360 0
G-R6:ASN-S1
20 0
40 4
60 22
80 40
100 32
120 31
140 20
160 7
180 4
200 0
220 16
240 11
260 28
280 24
300 39
320 8
340 4
360 0
U31-MY:ILE-S1
20 0
40 0
60 0
80 0
100 0
120 0
140 0
160 0
180 0
200 0
220 0
240 0
260 0

280 0
300 0
320 0
340 3
360 0
G-RIB:ASN-S1
20 8
40 8
60 44
80 48
100 36
120 24
140 20
160 32
180 0
200 4
220 8
240 48
260 28
280 52
300 36
320 20
340 20
360 8
FHU-MY:PRO-S1
20 0
40 0
60 0
80 0
100 3
120 0
140 0
160 0
180 0
200 0
220 0
240 0
260 0
280 0
300 0
320 0
340 0
360 0
C-RIB:ALA-S1
20 6
40 15
60 21
80 27
100 36
120 24
140 30
160 18
180 15
200 6
220 9
240 30
260 36
280 33

300 45
320 30
340 21
360 3
A-R6:GLN-S1
20 0
40 4
60 12
80 12
100 20
120 8
140 12
160 16
180 8
200 0
220 4
240 8
260 20
280 8
300 20
320 36
340 8
360 4
A-R6:ASN-S2
20 0
40 20
60 8
80 24
100 28
120 16
140 23
160 8
180 4
200 0
220 16
240 32
260 44
280 32
300 12
320 24
340 16
360 8
A-R5:THR-CA
20 8
40 4
60 20
80 20
100 32
120 20
140 4
160 16
180 0
200 0
220 16
240 28
260 16
280 24
300 16

320   8
340   20
360   0
C-P:GLN-S1
20   0
40   6
60   9
80   27
100   27
120   21
140   9
160   15
180   6
200   0
220   6
240   21
260   27
280   48
300   27
320   33
340   12
360   12
U-Y:LYS-CA
20   3
40   3
60   9
80   9
100   9
120   15
140   3
160   3
180   5
200   9
220   9
240   6
260   21
280   12
300   12
320   15
340   12
360   0
C-Y:LYS-S1
20   21
40   18
60   33
80   21
100   27
120   27
140   6
160   12
180   6
200   9
220   12
240   18
260   36
280   27
300   12
320   15

340 9
360 9
G-R6:GLU-S2
20 4
40 24
60 32
80 24
100 35
120 24
140 11
160 28
180 8
200 0
220 4
240 43
260 32
280 20
300 12
320 20
340 11
360 8
A-RIB:LEU-S1
20 0
40 16
60 8
80 8
100 32
120 12
140 20
160 24
180 8
200 0
220 4
240 0
260 16
280 24
300 12
320 28
340 20
360 8
U31-MY:ASP-S1
20 0
40 0
60 3
80 3
100 3
120 3
140 0
160 0
180 0
200 0
220 0
240 0
260 0
280 0
300 0
320 0
340 0

360 0
U-Y:TRP-S2
20 3
40 3
60 6
80 3
100 3
120 0
140 0
160 0
180 0
200 3
220 0
240 0
260 6
280 0
300 3
320 3
340 0
360 0
C-P:TRP-S1
20 0
40 0
60 3
80 6
100 3
120 3
140 6
160 3
180 0
200 0
220 6
240 6
260 6
280 6
300 0
320 15
340 0
360 0
G-RIB:ASN-CA
20 4
40 20
60 32
80 28
100 32
120 28
140 20
160 40
180 8
200 0
220 12
240 36
260 20
280 36
300 36
320 24
340 32
360 8

U-Y:GLY-CA
20   18
40   12
60   39
80   3
100   24
120   15
140   6
160   6
180   6
200   9
220   24
240   30
260   39
280   21
300   9
320   3
340   12
360   6
A-R5:TYR-S2
20   8
40   4
60   16
80   24
100   12
120   16
140   12
160   8
180   0
200   12
220   4
240   12
260   28
280   4
300   8
320   0
340   4
360   8
G-P:SER-CA
20   0
40   12
60   24
80   36
100   28
120   56
140   80
160   40
180   4
200   0
220   8
240   32
260   44
280   76
300   48
320   40
340   20
360   20
C-RIB:SER-CA

20 12
40 18
60 33
80 24
100 39
120 39
140 27
160 12
180 9
200 0
220 15
240 21
260 39
280 15
300 27
320 39
340 24
360 27
C-P:CYS-S1
20 0
40 0
60 3
80 9
100 0
120 3
140 0
160 0
180 0
200 0
220 0
240 3
260 0
280 3
300 6
320 0
340 0
360 3
A-P:GLN-S1
20 0
40 4
60 12
80 40
100 32
120 12
140 28
160 12
180 0
200 0
220 12
240 12
260 16
280 16
300 28
320 32
340 24
360 0
G-RIB:ASN-S2
20 8

40   24
60   48
80   44
100   48
120   16
140   44
160   28
180   12
200   8
220   16
240   36
260   40
280   32
300   52
320   8
340   24
360   4
U-RIB:LEU-S2
20   0
40   0
60   0
80   36
100   9
120   21
140   12
160   3
180   3
200   0
220   3
240   3
260   6
280   12
300   9
320   12
340   6
360   3
C-Y:LEU-S2
20   3
40   9
60   12
80   9
100   12
120   9
140   3
160   0
180   3
200   3
220   12
240   12
260   12
280   27
300   12
320   0
340   0
360   0
C-Y:THR-S1
20   3
40   18

60   18
80   24
100   15
120   6
140   12
160   6
180   6
200   9
220   15
240   30
260   15
280   12
300   3
320   9
340   15
360   6
A-RIB:HIS-S1
20   0
40   8
60   12
80   8
100   28
120   16
140   16
160   8
180   8
200   0
220   0
240   20
260   20
280   28
300   36
320   32
340   12
360   4
G-RIB:MET-S1
20   0
40   4
60   4
80   8
100   8
120   4
140   12
160   8
180   12
200   4
220   4
240   8
260   12
280   12
300   12
320   24
340   4
360   20
A-R5:GLU-CA
20   8
40   12
60   0

80   24
100   8
120   32
140   20
160   4
180   4
200   0
220   16
240   4
260   4
280   16
300   12
320   16
340   0
360   4
U-P:TYR-S2
20   0
40   0
60   9
80   12
100   12
120   6
140   6
160   6
180   0
200   0
220   3
240   3
260   15
280   3
300   15
320   3
340   6
360   0
A-R5:GLN-S2
20   4
40   20
60   28
80   28
100   28
120   24
140   8
160   12
180   0
200   8
220   16
240   20
260   32
280   16
300   12
320   24
340   8
360   8
C-P:VAL-CA
20   0
40   3
60   12
80   39

100   39
120   36
140   24
160   6
180   6
200   0
220   3
240   27
260   24
280   30
300   24
320   30
340   27
360   3
A-P:ARG-CA
20   0
40   8
60   24
80   52
100   68
120   100
140   64
160   36
180   20
200   0
220   12
240   32
260   44
280   68
300   108
320   76
340   60
360   24
U-RIB:LEU-S1
20   0
40   0
60   15
80   9
100   3
120   15
140   6
160   6
180   3
200   0
220   0
240   0
260   12
280   9
300   18
320   12
340   9
360   3
A-P:ASP-S2
20   0
40   16
60   16
80   20
100   32

120   36
140   32
160   28
180   0
200   0
220   0
240   20
260   28
280   28
300   24
320   28
340   12
360   8
G-P:LYS-S2
20   4
40   4
60   76
80   148
100   184
120   140
140   96
160   104
180   40
200   0
220   16
240   80
260   96
280   148
300   124
320   112
340   92
360   32
G-P:ILE-S1
20   0
40   4
60   16
80   12
100   20
120   40
140   20
160   8
180   0
200   0
220   8
240   16
260   16
280   16
300   16
320   16
340   8
360   4
G-R6:PHE-CA
20   0
40   4
60   8
80   12
100   0
120   4

140   8
160   0
180   0
200   0
220   0
240   8
260   8
280   12
300   7
320   0
340   0
360   0
A-R6:LYS-S2
20   0
40   4
60   40
80   52
100   52
120   32
140   24
160   20
180   4
200   4
220   16
240   36
260   52
280   48
300   52
320   32
340   16
360   0
C-Y:ASN-CA
20   0
40   3
60   24
80   12
100   9
120   6
140   9
160   0
180   3
200   6
220   0
240   9
260   12
280   12
300   6
320   9
340   3
360   6
U-P:ASP-CA
20   0
40   3
60   15
80   3
100   9
120   27
140   18

160 12
180 6
200 0
220 0
240 3
260 9
280 6
300 9
320 9
340 21
360 3
G-P:MET-S2
20 4
40 0
60 12
80 20
100 8
120 4
140 4
160 8
180 0
200 0
220 8
240 8
260 8
280 4
300 12
320 8
340 0
360 0
C-P:LEU-S2
20 0
40 3
60 24
80 21
100 18
120 21
140 21
160 12
180 0
200 0
220 6
240 12
260 15
280 21
300 45
320 9
340 9
360 6
U-P:PHE-S2
20 0
40 3
60 6
80 0
100 9
120 15
140 3
160 3

180 0
200 0
220 9
240 30
260 6
280 3
300 12
320 0
340 0
360 3
A-R6:TYR-S1
20 0
40 0
60 24
80 28
100 4
120 16
140 4
160 12
180 4
200 0
220 0
240 20
260 12
280 4
300 12
320 16
340 3
360 4
U-RIB:ASN-S1
20 6
40 0
60 24
80 12
100 15
120 18
140 9
160 12
180 15
200 0
220 9
240 18
260 15
280 21
300 18
320 12
340 27
360 12
U-P:ALA-CA
20 0
40 3
60 18
80 24
100 30
120 15
140 33
160 27
180 12

200 0
220 9
240 6
260 24
280 24
300 33
320 24
340 18
360 6
U-P:PRO-CA
20 0
40 3
60 3
80 15
100 30
120 18
140 9
160 21
180 12
200 0
220 3
240 15
260 3
280 18
300 12
320 6
340 18
360 0
C-RIB:THR-CA
20 3
40 3
60 24
80 21
100 15
120 45
140 12
160 12
180 15
200 3
220 9
240 18
260 30
280 27
300 30
320 24
340 24
360 12
G-R6:MET-S2
20 0
40 8
60 12
80 12
100 8
120 12
140 0
160 0
180 4
200 0

220 0
240 20
260 20
280 12
300 4
320 12
340 4
360 0
C-P:LEU-CA
20 0
40 6
60 15
80 27
100 18
120 36
140 15
160 6
180 0
200 0
220 6
240 9
260 24
280 27
300 18
320 12
340 3
360 6
C-Y:TRP-S1
20 0
40 6
60 3
80 2
100 6
120 5
140 0
160 3
180 0
200 6
220 0
240 0
260 3
280 3
300 0
320 3
340 3
360 0
G-P:THR-S1
20 4
40 0
60 16
80 24
100 32
120 24
140 32
160 28
180 16
200 4
220 0

240 24
260 44
280 20
300 36
320 28
340 40
360 12
U-P:TRP-CA
20 0
40 0
60 0
80 9
100 6
120 9
140 9
160 3
180 6
200 0
220 0
240 0
260 3
280 9
300 12
320 0
340 3
360 0
G-R6:CYS-S1
20 0
40 0
60 0
80 0
100 0
120 0
140 8
160 4
180 0
200 0
220 0
240 4
260 0
280 0
300 4
320 0
340 0
360 0
C-P:SER-S1
20 0
40 21
60 9
80 48
100 24
120 45
140 33
160 21
180 12
200 0
220 15
240 24

260 48
280 60
300 36
320 27
340 48
360 18
U-P:PHE-CA
20 0
40 0
60 6
80 6
100 0
120 9
140 9
160 3
180 3
200 0
220 3
240 6
260 9
280 12
300 0
320 6
340 0
360 3
G-R5:PHE-S1
20 4
40 0
60 4
80 4
100 8
120 0
140 4
160 4
180 0
200 0
220 4
240 24
260 16
280 3
300 8
320 4
340 0
360 0
QUO-M6:ARG-S1
20 0
40 0
60 0
80 0
100 0
120 4
140 0
160 0
180 4
200 0
220 0
240 0
260 0

280 0
300 0
320 0
340 0
360 0
U-RIB:HIS-CA
20 0
40 3
60 3
80 15
100 33
120 9
140 6
160 3
180 0
200 0
220 0
240 0
260 9
280 9
300 6
320 9
340 12
360 3
A-R5:GLU-S2
20 0
40 28
60 32
80 20
100 16
120 8
140 24
160 8
180 4
200 4
220 12
240 20
260 36
280 24
300 32
320 12
340 8
360 4
C-RIB:TRP-S2
20 0
40 3
60 0
80 15
100 9
120 3
140 0
160 6
180 0
200 0
220 0
240 9
260 3
280 9

300 3
320 6
340 3
360 6
G-RIB:VAL-CA
20 0
40 0
60 8
80 16
100 12
120 28
140 20
160 16
180 0
200 0
220 8
240 8
260 16
280 16
300 24
320 24
340 12
360 8
C-RIB:HIS-S1
20 0
40 6
60 12
80 6
100 21
120 9
140 3
160 18
180 12
200 3
220 0
240 15
260 12
280 18
300 30
320 21
340 6
360 9
GTP-M6:ASN-S1
20 0
40 3
60 0
80 0
100 0
120 0
140 0
160 0
180 0
200 0
220 0
240 0
260 0
280 0
300 0

320 0
340 0
360 0
G-RIB:ILE-CA
20 0
40 4
60 0
80 20
100 28
120 32
140 24
160 8
180 0
200 0
220 4
240 0
260 16
280 16
300 8
320 20
340 4
360 0
U-Y:ASN-CA
20 0
40 3
60 15
80 6
100 24
120 9
140 12
160 3
180 0
200 0
220 6
240 9
260 21
280 14
300 6
320 15
340 9
360 0
A-R5:SER-CA
20 4
40 16
60 28
80 20
100 4
120 16
140 28
160 12
180 16
200 0
220 12
240 24
260 8
280 28
300 12
320 16

340 12
360 8
G-R5:GLU-S2
20 8
40 8
60 32
80 40
100 12
120 20
140 32
160 8
180 4
200 4
220 12
240 32
260 31
280 15
300 19
320 16
340 12
360 0
C-Y:PRO-CA
20 0
40 20
60 21
80 15
100 18
120 12
140 6
160 12
180 0
200 12
220 6
240 15
260 3
280 15
300 15
320 6
340 3
360 0
G-R5:HIS-S2
20 12
40 8
60 47
80 44
100 28
120 28
140 4
160 8
180 0
200 0
220 24
240 27
260 16
280 40
300 12
320 0
340 12

360 0
U-P:GLY-CA
20 3
40 18
60 30
80 24
100 15
120 30
140 66
160 39
180 9
200 6
220 21
240 12
260 45
280 51
300 45
320 12
340 36
360 3
U-Y:ASP-S1
20 3
40 6
60 9
80 12
100 3
120 6
140 15
160 3
180 0
200 0
220 6
240 15
260 9
280 0
300 6
320 0
340 0
360 3
U-P:MET-S1
20 0
40 6
60 3
80 6
100 12
120 6
140 0
160 3
180 3
200 0
220 6
240 9
260 3
280 6
300 6
320 9
340 6
360 0

U-P:ASP-S2
20 0
40 0
60 3
80 9
100 15
120 33
140 12
160 9
180 6
200 0
220 12
240 6
260 15
280 15
300 12
320 15
340 24
360 9
C-RIB:LEU-S2
20 0
40 3
60 12
80 18
100 18
120 15
140 27
160 15
180 3
200 3
220 0
240 12
260 18
280 33
300 24
320 24
340 12
360 6
U-RIB:THR-CA
20 0
40 6
60 12
80 24
100 27
120 15
140 27
160 12
180 0
200 3
220 9
240 6
260 12
280 9
300 24
320 12
340 3
360 3
U-Y:MET-S2

20 0
40 6
60 3
80 3
100 3
120 9
140 3
160 0
180 0
200 0
220 3
240 6
260 3
280 6
300 0
320 3
340 3
360 0
U-RIB:ASP-S1
20 0
40 3
60 9
80 6
100 24
120 18
140 12
160 9
180 6
200 0
220 6
240 6
260 3
280 6
300 9
320 15
340 15
360 3
G-R5:PHE-CA
20 12
40 4
60 8
80 12
100 8
120 0
140 0
160 4
180 0
200 4
220 4
240 12
260 12
280 4
300 0
320 4
340 0
360 0
A-P:HIS-S1
20 0

40	4
60	28
80	20
100	28
120	12
140	24
160	12
180	8
200	0
220	8
240	12
260	8
280	32
300	8
320	12
340	20
360	8

G-R5:ILE-S1

20	0
40	8
60	4
80	3
100	8
120	8
140	4
160	0
180	0
200	4
220	4
240	4
260	8
280	4
300	8
320	0
340	0
360	0

U-RIB:SER-S1

20	3
40	6
60	21
80	30
100	24
120	18
140	15
160	6
180	6
200	6
220	3
240	18
260	21
280	27
300	9
320	15
340	18
360	3

C-RIB:LYS-S2

20	33
40	87

60   51
80   57
100   51
120   66
140   30
160   30
180   21
200   18
220   72
240   33
260   54
280   54
300   66
320   33
340   21
360   9
G-R6:PRO-S1
20   0
40   8
60   28
80   16
100   12
120   20
140   8
160   8
180   0
200   0
220   20
240   16
260   35
280   20
300   8
320   16
340   12
360   0
G-R5:ARG-CA
20   4
40   28
60   20
80   16
100   24
120   16
140   24
160   4
180   16
200   12
220   36
240   20
260   40
280   32
300   28
320   40
340   24
360   4
A-RIB:MET-S2
20   0
40   4
60   4

80 4
100 20
120 8
140 8
160 8
180 0
200 0
220 4
240 4
260 12
280 8
300 12
320 4
340 8
360 0
C-RIB:VAL-S1
20 0
40 6
60 12
80 27
100 30
120 30
140 27
160 24
180 0
200 0
220 0
240 18
260 42
280 21
300 24
320 27
340 12
360 3
A-R6:MET-CA
20 0
40 0
60 8
80 8
100 20
120 12
140 12
160 0
180 4
200 0
220 0
240 4
260 4
280 4
300 16
320 12
340 4
360 4
U-RIB:GLN-CA
20 0
40 6
60 6
80 3

100 15
120 6
140 6
160 12
180 3
200 0
220 6
240 9
260 3
280 21
300 18
320 6
340 9
360 6
G-R5:ASN-CA
20 0
40 20
60 29
80 12
100 20
120 16
140 20
160 8
180 7
200 4
220 16
240 31
260 31
280 0
300 16
320 8
340 12
360 3
G-RIB:ASP-CA
20 4
40 4
60 16
80 16
100 32
120 48
140 28
160 28
180 16
200 4
220 8
240 8
260 32
280 24
300 24
320 24
340 28
360 8
C-RIB:ASP-CA
20 0
40 3
60 18
80 9
100 39

120 36
140 15
160 6
180 12
200 0
220 3
240 6
260 27
280 18
300 30
320 21
340 27
360 15
C-RIB:CYS-CA
20 0
40 3
60 0
80 3
100 3
120 0
140 3
160 3
180 0
200 0
220 0
240 0
260 3
280 9
300 3
320 3
340 3
360 0
G-R5:MET-S1
20 4
40 0
60 12
80 24
100 0
120 4
140 0
160 0
180 0
200 0
220 8
240 0
260 20
280 4
300 8
320 12
340 4
360 0
G-P:THR-CA
20 0
40 16
60 4
80 24
100 28
120 16

140 48
160 36
180 20
200 0
220 12
240 8
260 32
280 28
300 24
320 36
340 32
360 8
U-Y:SER-S1
20 6
40 18
60 12
80 20
100 15
120 9
140 6
160 3
180 3
200 3
220 24
240 9
260 11
280 3
300 15
320 12
340 15
360 0
U-Y:CYS-S1
20 0
40 0
60 0
80 3
100 0
120 0
140 3
160 0
180 0
200 0
220 3
240 2
260 0
280 6
300 3
320 3
340 0
360 0
H2U-RIB:GLU-S2
20 0
40 0
60 0
80 0
100 0
120 0
140 0

160 0
180 0
200 0
220 0
240 0
260 0
280 3
300 0
320 0
340 0
360 0
C-P:LYS-S2
20 0
40 18
60 39
80 51
100 54
120 96
140 69
160 42
180 27
200 6
220 18
240 42
260 63
280 60
300 99
320 99
340 90
360 27
U31-P:MET-S2
20 0
40 0
60 0
80 0
100 0
120 0
140 3
160 0
180 0
200 0
220 0
240 3
260 0
280 0
300 0
320 0
340 0
360 0
G-P:TRP-CA
20 0
40 8
60 8
80 8
100 12
120 4
140 0
160 12

180 0
200 0
220 0
240 4
260 4
280 4
300 12
320 16
340 0
360 8
A-R5:LYS-CA
20 8
40 16
60 16
80 16
100 32
120 20
140 8
160 12
180 8
200 4
220 12
240 12
260 24
280 48
300 16
320 16
340 16
360 0
U-RIB:GLU-S1
20 0
40 3
60 3
80 18
100 9
120 6
140 3
160 6
180 0
200 3
220 0
240 6
260 6
280 6
300 12
320 3
340 9
360 3
C-RIB:TYR-S1
20 0
40 0
60 3
80 6
100 6
120 15
140 6
160 3
180 0

200 0
220 3
240 0
260 15
280 21
300 27
320 6
340 15
360 12
A-RIB:LEU-CA
20 0
40 16
60 4
80 4
100 20
120 32
140 20
160 20
180 12
200 0
220 4
240 0
260 20
280 12
300 28
320 28
340 12
360 8
G-RIB:LEU-S2
20 0
40 0
60 28
80 44
100 48
120 8
140 28
160 8
180 8
200 4
220 8
240 8
260 28
280 36
300 28
320 28
340 12
360 8
A-RIB:ALA-S1
20 0
40 12
60 44
80 28
100 48
120 16
140 24
160 16
180 0
200 4

220 24
240 24
260 40
280 32
300 28
320 44
340 8
360 8
A-P:LYS-S2
20 8
40 16
60 32
80 96
100 100
120 84
140 68
160 88
180 32
200 0
220 8
240 56
260 52
280 104
300 76
320 104
340 48
360 20
A-P:PHE-S2
20 0
40 8
60 12
80 16
100 12
120 8
140 8
160 4
180 4
200 0
220 8
240 24
260 16
280 16
300 4
320 20
340 8
360 0
C-P:MET-S2
20 0
40 6
60 15
80 15
100 6
120 6
140 12
160 0
180 3
200 0
220 3

240 12
260 12
280 12
300 3
320 3
340 6
360 6
G-RIB:ILE-S1
20 0
40 4
60 0
80 24
100 16
120 12
140 12
160 0
180 0
200 0
220 8
240 0
260 16
280 28
300 4
320 20
340 8
360 4
G-RIB:ALA-CA
20 12
40 12
60 16
80 40
100 44
120 52
140 28
160 32
180 8
200 4
220 12
240 24
260 24
280 24
300 36
320 24
340 12
360 20
A-P:THR-S1
20 4
40 12
60 32
80 28
100 56
120 56
140 36
160 24
180 16
200 0
220 4
240 36

260 24
280 40
300 48
320 24
340 12
360 4
U-P:ILE-S1
20 0
40 0
60 6
80 3
100 6
120 6
140 3
160 9
180 0
200 0
220 6
240 0
260 12
280 3
300 15
320 0
340 6
360 3
FHU-MY:TYR-S1
20 0
40 0
60 0
80 0
100 3
120 0
140 3
160 0
180 0
200 0
220 0
240 0
260 0
280 3
300 0
320 3
340 0
360 0
G-RIB:LYS-S2
20 28
40 64
60 124
80 84
100 120
120 56
140 64
160 32
180 4
200 12
220 84
240 148
260 132

280   112
300   68
320   32
340   40
360   12
C-Y:GLU-S2
20   3
40   18
60   30
80   18
100   18
120   9
140   6
160   12
180   6
200   9
220   9
240   18
260   21
280   18
300   3
320   15
340   6
360   9
C-P:GLN-CA
20   0
40   3
60   15
80   24
100   18
120   21
140   9
160   12
180   9
200   0
220   6
240   9
260   18
280   36
300   36
320   30
340   15
360   6
U-P:SER-CA
20   0
40   3
60   15
80   18
100   12
120   6
140   30
160   18
180   3
200   0
220   9
240   21
260   12
280   42

300 33
320 42
340 18
360 3
A-R5:THR-S1
20 12
40 12
60 28
80 28
100 32
120 12
140 16
160 4
180 4
200 8
220 24
240 20
260 32
280 8
300 12
320 8
340 12
360 0
DA-M6:LEU-S2
20 0
40 0
60 0
80 0
100 3
120 0
140 0
160 0
180 0
200 0
220 0
240 0
260 0
280 0
300 3
320 0
340 0
360 0
C-Y:TYR-S2
20 0
40 6
60 6
80 12
100 15
120 6
140 3
160 3
180 0
200 0
220 6
240 9
260 9
280 12
300 9

320   3
340   6
360   0
G-RIB:TYR-S2
20   0
40   4
60   12
80   20
100   24
120   12
140   16
160   12
180   8
200   0
220   4
240   36
260   24
280   16
300   4
320   20
340   4
360   0
G-R6:ASP-S2
20   4
40   16
60   12
80   28
100   32
120   28
140   19
160   20
180   12
200   8
220   16
240   28
260   44
280   48
300   40
320   23
340   28
360   8
U-Y:ASN-S1
20   9
40   3
60   15
80   12
100   18
120   15
140   12
160   6
180   0
200   0
220   6
240   21
260   24
280   17
300   6
320   15

340   6
360   6
A-RIB:LEU-S2
20   0
40   4
60   16
80   8
100   16
120   16
140   16
160   24
180   4
200   0
220   0
240   4
260   12
280   44
300   12
320   32
340   24
360   12
G-RIB:HIS-S2
20   0
40   8
60   40
80   16
100   20
120   16
140   28
160   40
180   4
200   4
220   12
240   20
260   20
280   36
300   36
320   32
340   16
360   24
G-P:PHE-S1
20   0
40   0
60   8
80   24
100   20
120   8
140   16
160   20
180   0
200   0
220   0
240   28
260   8
280   8
300   20
320   12
340   8

360   4
A-RIB:SER-S1
20   12
40   20
60   16
80   48
100   44
120   16
140   16
160   16
180   8
200   4
220   36
240   28
260   36
280   48
300   64
320   32
340   20
360   16
A-RIB:MET-S1
20   0
40   0
60   0
80   20
100   20
120   12
140   12
160   4
180   0
200   0
220   4
240   8
260   8
280   4
300   20
320   4
340   12
360   4
U-P:LEU-S1
20   0
40   6
60   12
80   3
100   15
120   18
140   6
160   9
180   0
200   0
220   9
240   6
260   9
280   27
300   12
320   15
340   9
360   0

A-RIB:ASN-S2
20 0
40 4
60 8
80 48
100 56
120 16
140 36
160 32
180 24
200 4
220 12
240 32
260 48
280 24
300 24
320 20
340 36
360 12
U-Y:MET-S1
20 0
40 3
60 9
80 0
100 3
120 0
140 0
160 0
180 0
200 3
220 0
240 6
260 3
280 6
300 0
320 3
340 3
360 0
A-RIB:VAL-S1
20 0
40 8
60 32
80 12
100 24
120 28
140 32
160 20
180 8
200 0
220 0
240 12
260 16
280 44
300 16
320 20
340 20
360 16
G-P:ASN-S1

20 0
40 8
60 44
80 48
100 32
120 40
140 28
160 52
180 20
200 0
220 4
240 20
260 52
280 44
300 40
320 36
340 24
360 12
U-RIB:PHE-S2
20 0
40 3
60 3
80 6
100 21
120 21
140 12
160 3
180 0
200 0
220 0
240 9
260 0
280 3
300 18
320 15
340 6
360 6
A-P:HIS-CA
20 0
40 8
60 24
80 20
100 20
120 16
140 24
160 8
180 8
200 0
220 4
240 12
260 8
280 12
300 20
320 12
340 8
360 8
G-RIB:PRO-S1
20 4

40   8
60   44
80   44
100   56
120   32
140   40
160   28
180   4
200   4
220   4
240   28
260   28
280   28
300   36
320   24
340   24
360   12
A-R5:PHE-S1
20   4
40   4
60   8
80   8
100   4
120   4
140   0
160   0
180   0
200   0
220   12
240   4
260   8
280   8
300   16
320   0
340   0
360   0
U-RIB:ALA-CA
20   9
40   6
60   21
80   27
100   24
120   15
140   21
160   12
180   3
200   0
220   6
240   12
260   24
280   27
300   21
320   21
340   12
360   6
C-P:GLY-CA
20   0
40   27

60 21
80 39
100 84
120 45
140 60
160 48
180 15
200 0
220 27
240 51
260 42
280 66
300 54
320 75
340 45
360 12
C-RIB:LYS-S1
20 12
40 21
60 75
80 39
100 39
120 54
140 30
160 33
180 12
200 6
220 30
240 45
260 30
280 57
300 45
320 30
340 33
360 9
U-Y:GLU-S1
20 3
40 15
60 6
80 6
100 6
120 0
140 3
160 6
180 3
200 0
220 3
240 6
260 3
280 6
300 6
320 0
340 3
360 0
U-P:ALA-S1
20 0
40 3
60 12

80 36
100 21
120 12
140 24
160 15
180 12
200 3
220 9
240 9
260 33
280 36
300 24
320 39
340 12
360 15
G-R5:LEU-S1
20 0
40 4
60 8
80 16
100 16
120 8
140 12
160 8
180 0
200 4
220 12
240 4
260 32
280 16
300 8
320 12
340 4
360 0
G-P:MET-CA
20 0
40 0
60 8
80 16
100 20
120 4
140 4
160 0
180 0
200 0
220 4
240 4
260 4
280 20
300 4
320 16
340 16
360 4
G-RIB:ARG-S1
20 12
40 32
60 80
80 116

100 52
120 72
140 36
160 52
180 8
200 12
220 16
240 100
260 92
280 92
300 88
320 64
340 16
360 16
C-P:HIS-S1
20 0
40 15
60 6
80 9
100 33
120 9
140 9
160 12
180 0
200 0
220 0
240 6
260 15
280 15
300 6
320 24
340 9
360 3
G-P:HIS-CA
20 0
40 4
60 16
80 52
100 24
120 24
140 24
160 16
180 8
200 0
220 0
240 4
260 8
280 28
300 36
320 24
340 8
360 0
A-R6:TYR-S2
20 0
40 12
60 12
80 24
100 24

120 12
140 8
160 4
180 0
200 0
220 8
240 12
260 12
280 8
300 4
320 4
340 12
360 0
G-R6:LYS-S2
20 0
40 24
60 60
80 60
100 44
120 48
140 28
160 16
180 4
200 0
220 12
240 56
260 56
280 84
300 68
320 40
340 28
360 0
A-R6:SER-S1
20 0
40 12
60 24
80 12
100 24
120 8
140 40
160 12
180 8
200 4
220 16
240 24
260 16
280 36
300 16
320 16
340 28
360 4
C-RIB:HIS-S2
20 3
40 6
60 12
80 15
100 18
120 24

140 12
160 18
180 12
200 0
220 3
240 12
260 12
280 24
300 18
320 30
340 6
360 12
C-RIB:GLU-CA
20 3
40 6
60 12
80 18
100 15
120 6
140 27
160 15
180 6
200 3
220 6
240 3
260 12
280 18
300 33
320 15
340 18
360 9
G-R5:PRO-CA
20 0
40 16
60 23
80 12
100 12
120 8
140 12
160 0
180 0
200 0
220 32
240 20
260 16
280 16
300 4
320 8
340 4
360 12
C-RIB:ARG-S1
20 0
40 48
60 69
80 78
100 75
120 51
140 51

160 33
180 27
200 12
220 45
240 63
260 60
280 63
300 72
320 57
340 36
360 15
A-R6:ASN-S1
20 4
40 0
60 8
80 20
100 20
120 32
140 8
160 7
180 0
200 0
220 8
240 16
260 28
280 16
300 20
320 12
340 16
360 4
U-P:GLN-S2
20 3
40 6
60 12
80 15
100 27
120 12
140 9
160 9
180 6
200 0
220 12
240 9
260 9
280 24
300 24
320 12
340 12
360 0
U-RIB:TYR-S2
20 0
40 3
60 0
80 12
100 15
120 6
140 3
160 9

180   6
200   0
220   3
240   3
260   21
280   9
300   3
320   12
340   9
360   3
C-P:ASN-CA
20   0
40   3
60   12
80   21
100   30
120   33
140   12
160   12
180   9
200   0
220   9
240   15
260   33
280   39
300   39
320   24
340   18
360   12
C-RIB:TYR-CA
20   0
40   0
60   3
80   6
100   18
120   18
140   0
160   0
180   0
200   0
220   3
240   3
260   15
280   30
300   24
320   6
340   9
360   6
U-Y:TYR-S1
20   0
40   0
60   3
80   5
100   3
120   6
140   3
160   0
180   0

200 0
220 0
240 6
260 3
280 12
300 21
320 9
340 0
360 0
U-P:MET-CA
20 0
40 3
60 9
80 0
100 6
120 12
140 6
160 3
180 0
200 3
220 3
240 6
260 0
280 3
300 9
320 3
340 0
360 0
C-P:TYR-S2
20 0
40 0
60 15
80 18
100 15
120 24
140 3
160 6
180 3
200 0
220 3
240 6
260 12
280 15
300 9
320 18
340 6
360 0
A-P:TRP-S1
20 0
40 4
60 0
80 8
100 4
120 12
140 0
160 12
180 0
200 0

220 0
240 0
260 0
280 4
300 4
320 4
340 4
360 4
A-R6:CYS-S1
20 0
40 0
60 8
80 4
100 0
120 0
140 0
160 0
180 0
200 0
220 0
240 0
260 4
280 4
300 0
320 4
340 0
360 0
C-P:ASN-S1
20 0
40 12
60 12
80 18
100 42
120 39
140 15
160 18
180 9
200 0
220 15
240 12
260 42
280 33
300 39
320 33
340 27
360 9
G-R6:GLU-S1
20 0
40 8
60 20
80 16
100 20
120 18
140 8
160 12
180 12
200 0
220 8

240 24
260 23
280 16
300 20
320 20
340 23
360 4
G-RIB:THR-S1
20 12
40 12
60 12
80 36
100 48
120 24
140 36
160 20
180 8
200 12
220 20
240 12
260 36
280 28
300 24
320 12
340 16
360 12
C31-P:ASN-CA
20 0
40 0
60 0
80 0
100 0
120 0
140 3
160 0
180 0
200 0
220 0
240 0
260 0
280 0
300 0
320 0
340 0
360 0
C-Y:ASN-S1
20 12
40 18
60 21
80 27
100 0
120 15
140 6
160 3
180 0
200 12
220 3
240 21

260 15
280 17
300 15
320 0
340 6
360 3
IU-MY:ARG-CA
20 2
40 0
60 0
80 3
100 0
120 0
140 0
160 0
180 0
200 0
220 0
240 0
260 0
280 0
300 0
320 0
340 0
360 0
U-Y:PHE-CA
20 0
40 0
60 6
80 3
100 0
120 6
140 3
160 0
180 0
200 0
220 9
240 3
260 6
280 21
300 14
320 3
340 0
360 0
U-RIB:PRO-CA
20 3
40 9
60 6
80 9
100 15
120 21
140 3
160 18
180 6
200 0
220 0
240 12
260 27

280 6
300 27
320 12
340 0
360 6
C-P:PHE-CA
20 0
40 0
60 6
80 6
100 9
120 3
140 3
160 15
180 0
200 0
220 0
240 6
260 12
280 15
300 9
320 0
340 6
360 0
C-Y:ASP-S2
20 3
40 30
60 9
80 51
100 15
120 9
140 12
160 15
180 9
200 3
220 12
240 30
260 15
280 18
300 6
320 15
340 9
360 0
A-P:TRP-S2
20 4
40 0
60 12
80 4
100 4
120 0
140 4
160 4
180 4
200 0
220 0
240 8
260 0
280 4

300 4
320 12
340 0
360 4
U34-P:SER-S1
20 0
40 0
60 3
80 0
100 0
120 3
140 0
160 0
180 0
200 0
220 0
240 0
260 3
280 0
300 0
320 0
340 0
360 0
FHU-P:LYS-CA
20 0
40 0
60 0
80 0
100 3
120 3
140 0
160 0
180 0
200 0
220 0
240 0
260 0
280 0
300 3
320 0
340 0
360 3
C-Y:ARG-CA
20 0
40 15
60 15
80 27
100 42
120 17
140 18
160 3
180 3
200 9
220 3
240 21
260 27
280 30
300 12

320 24
340 9
360 0
U-RIB:VAL-CA
20 0
40 0
60 6
80 6
100 18
120 15
140 6
160 0
180 6
200 0
220 0
240 0
260 18
280 6
300 24
320 6
340 3
360 3
C-RIB:PRO-CA
20 0
40 6
60 27
80 30
100 27
120 21
140 12
160 6
180 6
200 0
220 6
240 15
260 18
280 33
300 18
320 33
340 15
360 3
C-RIB:PHE-S2
20 0
40 0
60 9
80 9
100 12
120 12
140 6
160 9
180 3
200 0
220 0
240 0
260 6
280 18
300 3
320 9

340   9
360   3
C-Y:SER-CA
20   9
40   21
60   27
80   23
100   18
120   9
140   3
160   9
180   3
200   0
220   15
240   27
260   30
280   21
300   12
320   9
340   9
360   0
A-R6:CYS-CA
20   0
40   0
60   4
80   4
100   0
120   0
140   4
160   0
180   0
200   0
220   0
240   4
260   0
280   4
300   4
320   0
340   0
360   0
U-RIB:TRP-S1
20   0
40   6
60   3
80   9
100   0
120   0
140   6
160   3
180   0
200   0
220   0
240   12
260   6
280   3
300   0
320   0
340   3

360 0
G-P:TRP-S1
20 0
40 4
60 12
80 0
100 20
120 0
140 4
160 8
180 4
200 0
220 0
240 4
260 8
280 8
300 8
320 8
340 0
360 4
A-R5:TYR-CA
20 0
40 4
60 16
80 24
100 12
120 16
140 8
160 0
180 8
200 0
220 8
240 12
260 20
280 12
300 8
320 12
340 3
360 0
C31-MY:SER-S1
20 0
40 0
60 0
80 0
100 0
120 0
140 0
160 0
180 0
200 0
220 0
240 0
260 3
280 0
300 0
320 0
340 0
360 0

G-R5:GLN-S2
20   4
40   16
60   56
80   36
100   40
120   16
140   4
160   0
180   0
200   11
220   4
240   28
260   52
280   32
300   16
320   4
340   4
360   4
A-R6:ARG-S2
20   0
40   32
60   44
80   84
100   64
120   68
140   60
160   36
180   8
200   0
220   20
240   64
260   72
280   28
300   52
320   60
340   36
360   8
G-R5:ARG-S2
20   24
40   40
60   48
80   88
100   63
120   52
140   84
160   28
180   12
200   51
220   56
240   48
260   56
280   95
300   40
320   36
340   44
360   20
C-RIB:GLY-CA

20 9
40 27
60 60
80 42
100 63
120 57
140 42
160 42
180 18
200 3
220 27
240 42
260 48
280 54
300 60
320 48
340 33
360 12
C-Y:LEU-CA
20 6
40 6
60 12
80 12
100 9
120 27
140 9
160 6
180 0
200 0
220 3
240 12
260 9
280 15
300 3
320 3
340 6
360 3
A-RIB:HIS-CA
20 0
40 12
60 4
80 16
100 20
120 12
140 4
160 16
180 8
200 0
220 0
240 8
260 32
280 16
300 52
320 24
340 28
360 4
A-R5:SER-S1
20 16

40   20
60   40
80   20
100   20
120   23
140   24
160   16
180   12
200   8
220   24
240   28
260   36
280   40
300   20
320   20
340   8
360   0
G-R6:GLN-S1
20   0
40   20
60   20
80   40
100   12
120   28
140   0
160   4
180   0
200   0
220   28
240   12
260   44
280   24
300   12
320   8
340   0
360   0
H2U-P:GLU-S1
20   0
40   0
60   0
80   3
100   0
120   0
140   3
160   0
180   0
200   0
220   0
240   0
260   0
280   0
300   0
320   0
340   0
360   0
A-R5:HIS-S2
20   4
40   16

60 16
80 8
100 24
120 24
140 12
160 4
180 0
200 8
220 12
240 24
260 44
280 20
300 24
320 20
340 4
360 0
A-RIB:LYS-CA
20 8
40 20
60 20
80 32
100 56
120 40
140 28
160 24
180 8
200 0
220 16
240 32
260 44
280 64
300 48
320 40
340 32
360 8
A-R5:TRP-S1
20 4
40 4
60 0
80 4
100 4
120 4
140 4
160 0
180 0
200 0
220 0
240 12
260 8
280 12
300 4
320 4
340 0
360 0
A-R5:PHE-S2
20 4
40 12
60 0

80 12
100 8
120 16
140 0
160 0
180 4
200 4
220 20
240 0
260 16
280 20
300 8
320 0
340 0
360 4
A-RIB:THR-CA
20 0
40 16
60 20
80 28
100 40
120 36
140 24
160 8
180 8
200 0
220 4
240 32
260 28
280 20
300 28
320 44
340 24
360 4
A-P:TRP-CA
20 0
40 0
60 4
80 8
100 8
120 12
140 4
160 4
180 0
200 0
220 0
240 0
260 4
280 4
300 4
320 4
340 4
360 4
U34-MY:PHE-S1
20 0
40 0
60 0
80 0

100 0
120 0
140 0
160 0
180 0
200 0
220 0
240 0
260 0
280 0
300 3
320 0
340 0
360 0
A-RIB:GLN-S2
20 4
40 8
60 24
80 36
100 28
120 24
140 12
160 12
180 4
200 0
220 12
240 16
260 36
280 36
300 40
320 12
340 24
360 8
A-R5:MET-S1
20 0
40 0
60 8
80 8
100 12
120 8
140 8
160 0
180 0
200 0
220 4
240 4
260 8
280 0
300 16
320 4
340 12
360 4
C-RIB:ALA-CA
20 3
40 6
60 24
80 30
100 45

120   18
140   42
160   21
180   6
200   3
220   12
240   24
260   30
280   27
300   33
320   36
340   30
360   9
G-R5:HIS-CA
20   4
40   12
60   12
80   15
100   8
120   12
140   4
160   0
180   0
200   4
220   16
240   8
260   19
280   8
300   8
320   8
340   8
360   0
G-RIB:MET-S2
20   0
40   8
60   4
80   4
100   12
120   8
140   8
160   20
180   0
200   0
220   0
240   12
260   20
280   12
300   8
320   16
340   36
360   4
A-P:GLN-CA
20   4
40   0
60   12
80   24
100   24
120   24

140 24
160 16
180 0
200 0
220 12
240 4
260 8
280 28
300 44
320 20
340 24
360 8
A-RIB:ASP-CA
20 0
40 0
60 0
80 20
100 24
120 12
140 8
160 8
180 4
200 0
220 4
240 4
260 24
280 32
300 40
320 24
340 36
360 0
G-R5:GLY-CA
20 28
40 40
60 68
80 72
100 28
120 36
140 8
160 20
180 4
200 28
220 60
240 60
260 52
280 64
300 28
320 24
340 24
360 4
G-P:ALA-S1
20 0
40 8
60 20
80 24
100 56
120 40
140 52

160   16
180   8
200   4
220   8
240   28
260   40
280   44
300   40
320   36
340   36
360   4
A-P:MET-CA
20   0
40   0
60   8
80   12
100   12
120   12
140   8
160   4
180   0
200   0
220   0
240   8
260   20
280   12
300   12
320   12
340   0
360   0
A-RIB:GLN-CA
20   0
40   0
60   12
80   12
100   12
120   28
140   4
160   4
180   4
200   0
220   8
240   16
260   28
280   20
300   12
320   8
340   8
360   12
A-R6:GLY-CA
20   4
40   44
60   32
80   48
100   36
120   48
140   27
160   24

180 0
200 4
220 36
240 44
260 44
280 60
300 44
320 24
340 32
360 8
A-RIB:GLN-S1
20 0
40 12
60 16
80 16
100 28
120 16
140 28
160 4
180 12
200 4
220 0
240 24
260 24
280 44
300 24
320 8
340 8
360 8
G-R6:ILE-CA
20 0
40 0
60 4
80 0
100 12
120 4
140 0
160 4
180 4
200 0
220 0
240 12
260 11
280 8
300 16
320 4
340 0
360 0
C-RIB:MET-CA
20 0
40 3
60 0
80 9
100 18
120 3
140 6
160 0
180 6

200 0
220 0
240 9
260 3
280 9
300 18
320 9
340 0
360 9
G-P:ALA-CA
20 0
40 0
60 20
80 20
100 40
120 60
140 28
160 24
180 0
200 0
220 20
240 24
260 36
280 48
300 48
320 44
340 40
360 8
G-R6:CYS-CA
20 0
40 4
60 0
80 4
100 0
120 4
140 4
160 0
180 4
200 0
220 0
240 0
260 0
280 0
300 4
320 0
340 0
360 0
C-RIB:ASN-CA
20 3
40 3
60 12
80 39
100 30
120 24
140 24
160 9
180 9
200 0

220   9
240   18
260   24
280   12
300   27
320   21
340   12
360   6
U-RIB:PHE-CA
20   0
40   0
60   3
80   12
100   9
120   12
140   12
160   3
180   0
200   0
220   0
240   6
260   0
280   6
300   21
320   12
340   9
360   0
A-R5:LEU-S1
20   0
40   8
60   4
80   32
100   32
120   12
140   0
160   4
180   0
200   0
220   32
240   16
260   20
280   24
300   20
320   4
340   12
360   0
U-RIB:MET-S1
20   0
40   0
60   3
80   3
100   18
120   9
140   3
160   3
180   0
200   0
220   0

240   6
260   6
280   6
300   3
320   6
340   0
360   3
IU-RIB:ILE-S1
20   0
40   0
60   0
80   0
100   3
120   0
140   0
160   0
180   0
200   0
220   0
240   0
260   0
280   0
300   0
320   0
340   0
360   0
A-R5:ARG-S1
20   16
40   32
60   32
80   76
100   44
120   36
140   28
160   8
180   4
200   16
220   8
240   36
260   64
280   28
300   40
320   32
340   0
360   4
C-Y:HIS-S2
20   3
40   12
60   9
80   18
100   24
120   6
140   6
160   3
180   0
200   0
220   9
240   30

260 18
280 15
300 6
320 0
340 3
360 3
U31-P:MET-S1
20 0
40 0
60 0
80 0
100 0
120 0
140 0
160 0
180 0
200 0
220 0
240 3
260 0
280 0
300 3
320 0
340 0
360 0
A-RIB:CYS-S1
20 0
40 0
60 8
80 0
100 12
120 12
140 0
160 0
180 4
200 0
220 0
240 4
260 0
280 0
300 4
320 0
340 4
360 0
FHU-P:LEU-S1
20 0
40 0
60 3
80 0
100 0
120 0
140 0
160 3
180 0
200 0
220 0
240 3
260 0

280   0
300   0
320   0
340   6
360   0
U-P:TYR-S1
20   0
40   0
60   15
80   3
100   3
120   3
140   9
160   0
180   0
200   0
220   0
240   0
260   12
280   6
300   9
320   0
340   0
360   0
A-R5:HIS-CA
20   4
40   8
60   12
80   24
100   16
120   24
140   0
160   4
180   0
200   0
220   16
240   20
260   16
280   8
300   8
320   20
340   4
360   0
A-R6:TYR-CA
20   0
40   4
60   12
80   24
100   8
120   12
140   0
160   8
180   4
200   0
220   4
240   16
260   20
280   4

300 16
320 16
340 8
360 7
U-Y:HIS-CA
20 3
40 9
60 6
80 0
100 3
120 3
140 6
160 6
180 0
200 0
220 6
240 3
260 0
280 6
300 6
320 6
340 0
360 0
U-P:GLU-CA
20 0
40 3
60 0
80 9
100 12
120 18
140 18
160 9
180 6
200 0
220 3
240 12
260 9
280 12
300 9
320 21
340 9
360 0
A-R5:GLN-CA
20 4
40 0
60 20
80 4
100 20
120 8
140 8
160 12
180 0
200 8
220 4
240 4
260 20
280 8
300 16

320 4
340 0
360 0
C31-P:GLU-S1
20 0
40 0
60 0
80 0
100 0
120 0
140 0
160 0
180 0
200 0
220 0
240 0
260 3
280 0
300 0
320 0
340 0
360 0
C-RIB:VAL-CA
20 0
40 6
60 9
80 30
100 21
120 36
140 36
160 18
180 3
200 0
220 6
240 9
260 45
280 27
300 18
320 30
340 21
360 0
G-RIB:GLU-CA
20 0
40 0
60 4
80 20
100 16
120 32
140 24
160 16
180 8
200 0
220 8
240 8
260 8
280 32
300 36
320 24

340   8
360   12
IU-P:ARG-CA
20   0
40   0
60   3
80   0
100   0
120   0
140   0
160   0
180   0
200   0
220   0
240   0
260   0
280   0
300   0
320   3
340   0
360   0
U-P:LEU-S2
20   3
40   9
60   9
80   6
100   3
120   21
140   15
160   15
180   0
200   0
220   15
240   6
260   18
280   18
300   12
320   12
340   9
360   0
G-R5:ASP-S1
20   0
40   12
60   20
80   16
100   19
120   12
140   16
160   16
180   0
200   8
220   24
240   24
260   32
280   28
300   8
320   24
340   8

360   4
G-R6:GLY-CA
20   8
40   28
60   60
80   60
100   44
120   40
140   28
160   16
180   12
200   12
220   28
240   28
260   63
280   44
300   40
320   39
340   16
360   12
5BU-P:ILE-CA
20   0
40   0
60   0
80   0
100   0
120   3
140   0
160   0
180   0
200   0
220   0
240   0
260   0
280   0
300   3
320   3
340   0
360   0
A-R5:ARG-CA
20   4
40   16
60   32
80   48
100   52
120   8
140   28
160   8
180   0
200   8
220   16
240   28
260   60
280   16
300   40
320   36
340   16
360   4

C-Y:GLU-S1
20   3
40   9
60   6
80   6
100   18
120   0
140   9
160   18
180   3
200   0
220   9
240   24
260   18
280   3
300   0
320   12
340   3
360   0
C-P:MET-S1
20   0
40   15
60   6
80   15
100   3
120   9
140   6
160   3
180   0
200   0
220   3
240   12
260   12
280   15
300   6
320   6
340   15
360   0
U-RIB:MET-CA
20   0
40   9
60   3
80   3
100   9
120   9
140   0
160   6
180   0
200   0
220   6
240   0
260   3
280   9
300   9
320   0
340   0
360   0
A-R5:ASN-S2

20 20
40 20
60 20
80 24
100 28
120 20
140 12
160 20
180 0
200 8
220 20
240 32
260 32
280 28
300 32
320 4
340 4
360 3
U-P:ASP-S1
20 3
40 0
60 3
80 9
100 12
120 15
140 27
160 0
180 6
200 0
220 9
240 3
260 12
280 9
300 9
320 6
340 24
360 9
G-P:LYS-CA
20 0
40 8
60 24
80 64
100 72
120 128
140 68
160 48
180 32
200 0
220 8
240 60
260 56
280 88
300 80
320 92
340 96
360 32
C-P:SER-CA
20 0

40   9
60   27
80   39
100   39
120   18
140   21
160   36
180   12
200   0
220   18
240   15
260   39
280   60
300   27
320   24
340   33
360   18
A-P:GLY-CA
20   0
40   28
60   56
80   40
100   88
120   68
140   64
160   44
180   12
200   4
220   52
240   32
260   76
280   116
300   76
320   60
340   64
360   28
A-P:TYR-CA
20   0
40   0
60   12
80   12
100   12
120   16
140   16
160   12
180   0
200   0
220   4
240   8
260   24
280   16
300   12
320   24
340   4
360   0
C-Y:ASN-S2
20   12
40   18

60   21
80   21
100   21
120   12
140   6
160   12
180   0
200   6
220   18
240   18
260   18
280   20
300   21
320   6
340   9
360   0
G-R6:LYS-CA
20   0
40   12
60   12
80   15
100   40
120   28
140   8
160   4
180   8
200   0
220   4
240   32
260   40
280   36
300   12
320   32
340   28
360   8
U-RIB:HIS-S1
20   0
40   0
60   9
80   24
100   21
120   9
140   15
160   9
180   6
200   0
220   0
240   0
260   15
280   12
300   3
320   3
340   15
360   0
A-R6:ARG-CA
20   0
40   0
60   40

80   40
100   36
120   40
140   36
160   16
180   0
200   0
220   12
240   36
260   12
280   44
300   16
320   32
340   23
360   0
C-Y:GLU-CA
20   0
40   9
60   12
80   11
100   0
120   21
140   6
160   6
180   3
200   0
220   6
240   12
260   3
280   3
300   3
320   0
340   9
360   0
C-Y:VAL-CA
20   0
40   9
60   9
80   18
100   6
120   18
140   6
160   3
180   0
200   0
220   6
240   15
260   6
280   18
300   15
320   6
340   0
360   0
G-RIB:VAL-S1
20   0
40   12
60   8
80   24

100 28
120 16
140 12
160 8
180 4
200 0
220 8
240 16
260 8
280 16
300 12
320 36
340 12
360 12
A-R6:ASN-CA
20 0
40 0
60 8
80 12
100 24
120 32
140 12
160 15
180 4
200 0
220 12
240 4
260 24
280 24
300 20
320 4
340 8
360 0
DA-M5:VAL-S1
20 0
40 0
60 0
80 0
100 0
120 0
140 0
160 0
180 0
200 0
220 0
240 3
260 0
280 0
300 0
320 0
340 0
360 0
A-P:LYS-CA
20 0
40 12
60 36
80 40
100 64

120 96
140 52
160 68
180 16
200 0
220 4
240 36
260 28
280 48
300 48
320 28
340 52
360 28
G-RIB:TRP-S1
20 0
40 4
60 8
80 4
100 8
120 8
140 8
160 4
180 0
200 0
220 0
240 16
260 4
280 12
300 20
320 8
340 4
360 0
A-P:VAL-S1
20 0
40 8
60 32
80 12
100 28
120 20
140 16
160 24
180 4
200 0
220 4
240 16
260 12
280 44
300 24
320 24
340 8
360 4
U31-MY:MET-S2
20 0
40 0
60 0
80 0
100 3
120 0

140 0
160 0
180 0
200 0
220 0
240 0
260 0
280 0
300 0
320 0
340 0
360 0
G-P:ASP-CA
20 0
40 0
60 0
80 28
100 36
120 32
140 48
160 32
180 4
200 0
220 12
240 24
260 16
280 40
300 72
320 40
340 24
360 20
A-R6:THR-S1
20 0
40 8
60 12
80 36
100 32
120 24
140 8
160 16
180 4
200 0
220 20
240 16
260 36
280 16
300 15
320 8
340 20
360 4
A-P:LEU-S2
20 0
40 4
60 16
80 12
100 12
120 8
140 12

160   12
180   0
200   0
220   4
240   20
260   8
280   24
300   20
320   28
340   16
360   12
A-P:ILE-CA
20   0
40   0
60   8
80   8
100   12
120   8
140   8
160   4
180   4
200   0
220   4
240   4
260   28
280   16
300   8
320   12
340   20
360   4
A-P:HIS-S2
20   0
40   8
60   36
80   24
100   36
120   28
140   16
160   8
180   8
200   0
220   8
240   12
260   24
280   24
300   16
320   12
340   24
360   4
C-RIB:ARG-CA
20   6
40   27
60   39
80   48
100   48
120   48
140   36
160   39

180   15
200   3
220   9
240   42
260   57
280   54
300   51
320   48
340   27
360   12
G-P:ASN-CA
20   0
40   4
60   20
80   28
100   44
120   44
140   36
160   12
180   24
200   8
220   4
240   20
260   36
280   28
300   48
320   32
340   24
360   8
C-P:GLN-S2
20   3
40   12
60   30
80   24
100   18
120   24
140   12
160   18
180   6
200   0
220   12
240   24
260   30
280   45
300   33
320   24
340   12
360   15
A-R5:LEU-S2
20   0
40   0
60   4
80   24
100   28
120   16
140   4
160   0
180   8

200 4
220 20
240 12
260 12
280 44
300 12
320 24
340 4
360 0
A-RIB:TYR-CA
20 0
40 0
60 16
80 16
100 20
120 8
140 8
160 12
180 4
200 0
220 4
240 4
260 12
280 12
300 20
320 12
340 20
360 0
C-P:THR-CA
20 0
40 6
60 18
80 33
100 30
120 42
140 18
160 21
180 6
200 0
220 6
240 12
260 24
280 33
300 24
320 24
340 15
360 9
C-P:MET-CA
20 0
40 3
60 12
80 6
100 9
120 12
140 6
160 0
180 0
200 0

220 3
240 9
260 9
280 3
300 12
320 15
340 3
360 0
G-P:VAL-CA
20 0
40 4
60 8
80 16
100 24
120 28
140 40
160 24
180 12
200 0
220 8
240 0
260 16
280 12
300 20
320 28
340 24
360 0
FHU-P:LYS-S1
20 0
40 0
60 0
80 0
100 0
120 3
140 3
160 0
180 0
200 0
220 0
240 0
260 0
280 0
300 3
320 3
340 0
360 0
U-RIB:PRO-S1
20 3
40 0
60 21
80 12
100 15
120 15
140 15
160 12
180 3
200 0
220 6

240 6
260 21
280 21
300 9
320 24
340 6
360 9
A-R5:ASP-S1
20 0
40 28
60 4
80 24
100 0
120 16
140 8
160 12
180 0
200 8
220 8
240 20
260 4
280 20
300 4
320 16
340 12
360 4
G-R5:TRP-CA
20 4
40 8
60 0
80 4
100 16
120 8
140 0
160 0
180 0
200 0
220 16
240 0
260 4
280 4
300 12
320 0
340 0
360 0
U-P:THR-S1
20 0
40 9
60 9
80 9
100 12
120 9
140 21
160 15
180 6
200 0
220 9
240 15

260 18
280 18
300 9
320 24
340 24
360 12
A-R5:LYS-S2
20 4
40 20
60 80
80 48
100 48
120 44
140 36
160 12
180 0
200 16
220 28
240 55
260 56
280 68
300 60
320 40
340 16
360 0
A-RIB:ARG-CA
20 12
40 20
60 28
80 44
100 84
120 48
140 44
160 52
180 8
200 4
220 24
240 28
260 64
280 52
300 52
320 48
340 32
360 20
U34-P:TYR-CA
20 0
40 0
60 0
80 3
100 0
120 0
140 0
160 0
180 0
200 0
220 0
240 0
260 0

280 0
300 0
320 3
340 0
360 0
U-P:VAL-S1
20 0
40 3
60 6
80 12
100 15
120 12
140 12
160 12
180 3
200 3
220 12
240 9
260 12
280 21
300 18
320 21
340 6
360 0
FHU-P:TYR-S1
20 0
40 0
60 0
80 0
100 0
120 0
140 0
160 0
180 3
200 0
220 0
240 0
260 0
280 0
300 0
320 0
340 0
360 0
C-Y:TRP-S2
20 0
40 9
60 3
80 12
100 3
120 6
140 6
160 2
180 0
200 3
220 3
240 0
260 3
280 11

300   9
320   3
340   0
360   0
IU-P:LYS-S1
20   3
40   0
60   0
80   0
100   3
120   0
140   0
160   0
180   0
200   0
220   0
240   0
260   0
280   0
300   0
320   0
340   0
360   0
C-Y:VAL-S1
20   3
40   9
60   21
80   9
100   12
120   9
140   9
160   0
180   0
200   6
220   9
240   18
260   15
280   18
300   9
320   9
340   0
360   0
QUO-RIB:LEU-CA
20   0
40   0
60   0
80   0
100   0
120   0
140   0
160   0
180   0
200   0
220   0
240   0
260   8
280   0
300   0

320   0
340   0
360   0
A-R5:HIS-S1
20   4
40   8
60   8
80   28
100   12
120   20
140   0
160   4
180   0
200   0
220   8
240   24
260   12
280   24
300   8
320   20
340   0
360   0
A-RIB:GLY-CA
20   28
40   44
60   64
80   68
100   84
120   48
140   76
160   44
180   4
200   4
220   44
240   60
260   40
280   52
300   92
320   68
340   40
360   20
FHU-MY:ALA-CA
20   0
40   3
60   0
80   0
100   0
120   3
140   0
160   0
180   0
200   0
220   0
240   0
260   0
280   0
300   3
320   0

340 0
360 0
IU-MY:ALA-CA
20 0
40 0
60 3
80 0
100 0
120 5
140 0
160 0
180 0
200 0
220 0
240 0
260 0
280 0
300 0
320 0
340 0
360 0
G-P:LEU-S2
20 0
40 4
60 28
80 20
100 28
120 28
140 20
160 8
180 16
200 0
220 12
240 24
260 40
280 32
300 20
320 24
340 16
360 4
G-R5:ARG-S1
20 28
40 28
60 36
80 40
100 36
120 32
140 32
160 36
180 8
200 20
220 8
240 32
260 44
280 28
300 52
320 48
340 36

360 12
G-P:ASP-S2
20 0
40 12
60 28
80 28
100 68
120 56
140 36
160 28
180 20
200 0
220 16
240 16
260 32
280 72
300 48
320 32
340 60
360 12
A-R5:PHE-CA
20 0
40 0
60 8
80 0
100 12
120 8
140 4
160 0
180 0
200 4
220 4
240 8
260 8
280 12
300 12
320 0
340 0
360 0
A-RIB:ASP-S1
20 0
40 0
60 0
80 32
100 32
120 12
140 20
160 8
180 0
200 0
220 0
240 12
260 28
280 24
300 48
320 24
340 32
360 4

A-R5:VAL-CA
20   0
40   12
60   12
80   12
100   24
120   20
140   4
160   16
180   0
200   0
220   4
240   8
260   4
280   20
300   16
320   12
340   4
360   0
G-R5:SER-CA
20   12
40   16
60   44
80   24
100   8
120   16
140   16
160   4
180   4
200   8
220   8
240   24
260   24
280   19
300   20
320   16
340   12
360   0
C-RIB:PHE-S1
20   0
40   3
60   3
80   9
100   15
120   15
140   12
160   6
180   3
200   0
220   0
240   0
260   9
280   9
300   9
320   9
340   3
360   0
U31-RIB:MET-S1

20 0
40 3
60 0
80 3
100 0
120 0
140 0
160 0
180 0
200 0
220 0
240 0
260 0
280 0
300 0
320 0
340 0
360 0
A-R6:MET-S1
20 0
40 0
60 12
80 12
100 8
120 12
140 12
160 8
180 0
200 0
220 0
240 8
260 0
280 12
300 20
320 4
340 4
360 4
G-R6:PHE-S1
20 0
40 0
60 4
80 8
100 4
120 16
140 4
160 4
180 0
200 0
220 8
240 8
260 12
280 4
300 11
320 0
340 0
360 0
A-R6:PHE-CA
20 0

40 4
60 8
80 4
100 16
120 8
140 4
160 0
180 0
200 0
220 0
240 8
260 4
280 16
300 12
320 0
340 0
360 0
C-RIB:ARG-S2
20 27
40 69
60 105
80 84
100 69
120 69
140 75
160 30
180 24
200 24
220 78
240 114
260 90
280 90
300 84
320 75
340 51
360 27
DA-M6:MET-S2
20 0
40 0
60 0
80 0
100 0
120 0
140 0
160 0
180 0
200 0
220 0
240 3
260 0
280 0
300 0
320 0
340 0
360 0
U-Y:GLU-S2
20 3
40 3

60 12
80 9
100 9
120 3
140 9
160 9
180 12
200 3
220 0
240 9
260 12
280 9
300 3
320 0
340 3
360 0
G-R5:LEU-CA
20 0
40 4
60 12
80 8
100 16
120 20
140 24
160 4
180 0
200 0
220 12
240 20
260 32
280 8
300 16
320 8
340 0
360 4
A-P:ALA-S1
20 0
40 4
60 32
80 36
100 32
120 28
140 40
160 36
180 4
200 0
220 0
240 28
260 24
280 48
300 40
320 28
340 40
360 4
G-R6:LEU-S2
20 4
40 8
60 8

80   12
100   16
120   20
140   8
160   8
180   0
200   0
220   8
240   16
260   16
280   12
300   24
320   8
340   0
360   0
C-Y:ASP-CA
20   6
40   6
60   15
80   18
100   21
120   9
140   15
160   9
180   0
200   0
220   15
240   15
260   21
280   3
300   15
320   21
340   12
360   3
A-R6:ALA-CA
20   0
40   8
60   20
80   12
100   20
120   16
140   0
160   8
180   8
200   0
220   8
240   20
260   0
280   20
300   20
320   40
340   12
360   4
U-P:CYS-S1
20   0
40   0
60   3
80   0

100 0
120 0
140 3
160 3
180 0
200 0
220 0
240 0
260 0
280 0
300 3
320 0
340 3
360 0
A-P:PHE-S1
20 0
40 0
60 8
80 12
100 16
120 8
140 4
160 4
180 0
200 0
220 8
240 4
260 20
280 8
300 28
320 16
340 4
360 4
G-RIB:GLU-S1
20 0
40 0
60 4
80 24
100 20
120 36
140 16
160 20
180 12
200 4
220 4
240 8
260 12
280 44
300 20
320 24
340 32
360 20
A-R6:HIS-CA
20 0
40 0
60 20
80 20
100 8

120   20
140   8
160   12
180   0
200   0
220   0
240   24
260   24
280   16
300   8
320   15
340   16
360   0
A-R6:PRO-CA
20   0
40   0
60   8
80   16
100   36
120   12
140   16
160   20
180   4
200   0
220   12
240   12
260   24
280   20
300   36
320   12
340   4
360   4
A-P:LEU-S1
20   0
40   4
60   8
80   16
100   12
120   8
140   12
160   8
180   8
200   0
220   8
240   16
260   16
280   8
300   40
320   16
340   20
360   0
U31-P:ASP-S1
20   0
40   0
60   0
80   0
100   0
120   0

140 0
160 0
180 0
200 0
220 0
240 0
260 3
280 0
300 3
320 0
340 0
360 0
C-Y:CYS-S1
20 0
40 0
60 0
80 3
100 0
120 3
140 0
160 0
180 0
200 0
220 0
240 3
260 0
280 0
300 6
320 0
340 0
360 0
FMU-P:VAL-S1
20 0
40 0
60 3
80 0
100 0
120 0
140 0
160 0
180 0
200 0
220 0
240 0
260 0
280 0
300 0
320 0
340 0
360 0
U31-P:HIS-CA
20 0
40 0
60 0
80 0
100 0
120 0
140 0

160 0
180 0
200 0
220 0
240 0
260 0
280 0
300 0
320 3
340 0
360 0
A-R5:PRO-S1
20 16
40 4
60 8
80 24
100 28
120 20
140 20
160 0
180 0
200 0
220 20
240 32
260 20
280 28
300 19
320 16
340 4
360 4
C-Y:GLN-S2
20 9
40 24
60 27
80 30
100 21
120 6
140 9
160 0
180 0
200 3
220 12
240 42
260 27
280 24
300 9
320 0
340 12
360 0
FHU-P:TYR-S2
20 0
40 3
60 0
80 3
100 0
120 0
140 0
160 0

180 0
200 0
220 3
240 0
260 3
280 0
300 0
320 0
340 0
360 0
G-R5:LYS-S2
20 28
40 28
60 92
80 88
100 64
120 39
140 12
160 28
180 20
200 28
220 28
240 76
260 68
280 60
300 39
320 96
340 36
360 8
A-P:LYS-S1
20 0
40 16
60 16
80 48
100 116
120 100
140 84
160 56
180 32
200 0
220 0
240 28
260 80
280 64
300 56
320 64
340 24
360 28
C-Y:ILE-CA
20 3
40 6
60 15
80 3
100 9
120 9
140 6
160 3
180 0

200 0
220 12
240 6
260 6
280 3
300 12
320 3
340 3
360 0
IU-MY:LEU-CA
20 0
40 2
60 0
80 0
100 0
120 2
140 0
160 0
180 0
200 0
220 0
240 0
260 0
280 0
300 0
320 0
340 0
360 0
U-P:LYS-S1
20 0
40 3
60 6
80 57
100 63
120 60
140 39
160 42
180 12
200 0
220 6
240 6
260 39
280 24
300 36
320 36
340 30
360 15
U31-MY:ILE-CA
20 0
40 0
60 0
80 0
100 0
120 0
140 0
160 0
180 0
200 0

220 0
240 0
260 0
280 0
300 0
320 0
340 0
360 3
G-R6:TRP-S1
20 0
40 4
60 4
80 12
100 8
120 16
140 4
160 0
180 0
200 0
220 8
240 8
260 4
280 4
300 20
320 4
340 8
360 0
G-R5:GLN-CA
20 4
40 24
60 32
80 8
100 0
120 0
140 4
160 0
180 0
200 8
220 20
240 12
260 28
280 8
300 8
320 8
340 0
360 0
GTP-M5:ALA-CA
20 0
40 0
60 0
80 0
100 0
120 0
140 0
160 0
180 0
200 0
220 0

240 0
260 0
280 3
300 0
320 0
340 0
360 0
G-P:LEU-CA
20 0
40 0
60 16
80 8
100 52
120 40
140 20
160 16
180 8
200 0
220 0
240 32
260 16
280 32
300 32
320 32
340 16
360 4
G-R5:ALA-S1
20 0
40 32
60 12
80 16
100 31
120 4
140 16
160 8
180 12
200 8
220 32
240 36
260 36
280 20
300 16
320 12
340 4
360 4
A-R6:GLU-S1
20 0
40 8
60 12
80 24
100 32
120 20
140 12
160 12
180 8
200 0
220 12
240 12

260 20
280 12
300 28
320 12
340 12
360 0
A-R6:PHE-S2
20 0
40 8
60 0
80 12
100 16
120 12
140 4
160 0
180 0
200 0
220 8
240 4
260 24
280 4
300 4
320 0
340 16
360 0
H2U-RIB:PRO-S1
20 0
40 0
60 0
80 0
100 0
120 0
140 0
160 0
180 0
200 0
220 0
240 0
260 0
280 0
300 3
320 0
340 0
360 0
C31-P:PHE-S2
20 0
40 0
60 0
80 0
100 0
120 0
140 0
160 0
180 0
200 0
220 3
240 3
260 0

280 0
300 0
320 0
340 0
360 0
C-Y:ARG-S2
20 18
40 21
60 60
80 93
100 72
120 60
140 26
160 21
180 12
200 24
220 36
240 51
260 78
280 69
300 39
320 21
340 9
360 33
G-P:GLN-S2
20 0
40 8
60 40
80 20
100 28
120 36
140 32
160 44
180 16
200 0
220 20
240 20
260 24
280 56
300 32
320 24
340 36
360 20
IU-P:LEU-S1
20 0
40 0
60 0
80 0
100 0
120 0
140 0
160 0
180 0
200 0
220 0
240 0
260 0
280 3

300 0
320 0
340 0
360 0
C-RIB:TYR-S2
20 0
40 3
60 6
80 12
100 15
120 9
140 15
160 3
180 0
200 0
220 9
240 9
260 21
280 21
300 9
320 15
340 12
360 6
G-R5:ASN-S2
20 12
40 16
60 19
80 23
100 32
120 28
140 27
160 12
180 4
200 12
220 12
240 44
260 24
280 52
300 24
320 8
340 0
360 8
G-R5:SER-S1
20 8
40 24
60 48
80 24
100 32
120 24
140 20
160 8
180 8
200 16
220 16
240 32
260 36
280 19
300 20

320   12
340   16
360   4
C-P:LYS-S1
20   3
40   6
60   33
80   30
100   51
120   51
140   78
160   30
180   24
200   0
220   12
240   24
260   39
280   90
300   51
320   102
340   54
360   36
A-P:TYR-S1
20   0
40   0
60   16
80   8
100   16
120   16
140   16
160   16
180   0
200   0
220   0
240   16
260   20
280   12
300   20
320   16
340   4
360   0
C-P:HIS-CA
20   0
40   9
60   6
80   3
100   30
120   15
140   18
160   15
180   3
200   0
220   0
240   3
260   24
280   9
300   12
320   24

340 3
360 3
H2U-MY:ARG-S2
20 0
40 0
60 0
80 0
100 3
120 0
140 0
160 0
180 0
200 0
220 0
240 0
260 3
280 0
300 3
320 0
340 3
360 0
G-R6:GLN-CA
20 0
40 8
60 8
80 28
100 16
120 4
140 0
160 8
180 0
200 0
220 12
240 8
260 36
280 12
300 4
320 0
340 0
360 0
U-P:THR-CA
20 0
40 6
60 6
80 9
100 9
120 24
140 15
160 6
180 3
200 0
220 6
240 18
260 24
280 24
300 18
320 24
340 12

360   6
G-P:PRO-CA
20   0
40   4
60   28
80   28
100   28
120   52
140   24
160   28
180   28
200   0
220   4
240   36
260   48
280   56
300   48
320   28
340   20
360   8
C-Y:ALA-CA
20   6
40   18
60   15
80   24
100   18
120   12
140   6
160   12
180   0
200   3
220   27
240   24
260   15
280   12
300   15
320   6
340   9
360   0
U-P:GLN-S1
20   0
40   0
60   12
80   18
100   12
120   18
140   3
160   9
180   3
200   0
220   6
240   9
260   6
280   12
300   27
320   24
340   9
360   6

C-P:TYR-CA
20 0
40 0
60 18
80 18
100 6
120 18
140 6
160 12
180 0
200 0
220 0
240 0
260 15
280 9
300 12
320 6
340 3
360 6
A-R6:PHE-S1
20 0
40 8
60 4
80 0
100 24
120 8
140 0
160 0
180 0
200 0
220 0
240 0
260 12
280 8
300 8
320 0
340 0
360 0
U-Y:THR-CA
20 0
40 6
60 12
80 9
100 9
120 15
140 3
160 0
180 3
200 3
220 0
240 3
260 10
280 6
300 6
320 6
340 6
360 0
A-R5:TRP-S2

20 0
40 0
60 16
80 0
100 12
120 4
140 0
160 8
180 0
200 0
220 4
240 8
260 4
280 12
300 4
320 4
340 0
360 0
A-RIB:ALA-CA
20 4
40 12
60 20
80 32
100 32
120 32
140 20
160 12
180 0
200 0
220 16
240 24
260 16
280 28
300 36
320 28
340 16
360 0
G-P:GLU-S2
20 0
40 8
60 16
80 20
100 48
120 44
140 44
160 48
180 4
200 0
220 16
240 32
260 8
280 36
300 44
320 36
340 24
360 12
G-P:PRO-S1
20 0

40 16
60 40
80 24
100 56
120 40
140 32
160 28
180 12
200 0
220 12
240 40
260 48
280 56
300 48
320 36
340 12
360 20
U-RIB:ARG-CA
20 3
40 6
60 15
80 15
100 27
120 39
140 27
160 15
180 3
200 3
220 9
240 33
260 12
280 15
300 33
320 9
340 15
360 3
A-R6:LEU-CA
20 0
40 4
60 8
80 32
100 28
120 28
140 12
160 0
180 4
200 0
220 8
240 0
260 20
280 24
300 28
320 19
340 4
360 0
A-RIB:PHE-CA
20 0
40 0

60   8
80   20
100   8
120   16
140   12
160   8
180   4
200   0
220   0
240   4
260   12
280   16
300   16
320   24
340   8
360   4
U-Y:PHE-S2
20   0
40   6
60   6
80   3
100   8
120   3
140   6
160   0
180   0
200   0
220   3
240   12
260   18
280   9
300   6
320   6
340   0
360   0
U-P:LYS-S2
20   6
40   6
60   21
80   54
100   81
120   54
140   51
160   51
180   3
200   0
220   12
240   24
260   42
280   69
300   45
320   48
340   33
360   12
A-P:ASN-CA
20   0
40   8
60   12

80 40
100 44
120 20
140 32
160 20
180 4
200 0
220 8
240 8
260 16
280 28
300 20
320 40
340 8
360 8
U-RIB:CYS-S1
20 0
40 0
60 0
80 0
100 0
120 3
140 3
160 6
180 0
200 0
220 0
240 0
260 0
280 3
300 0
320 0
340 0
360 0
A-RIB:TRP-S2
20 0
40 4
60 4
80 0
100 4
120 24
140 16
160 8
180 0
200 0
220 0
240 4
260 4
280 8
300 12
320 4
340 8
360 8
A-R6:TRP-S2
20 0
40 0
60 8
80 4

100   12
120   4
140   0
160   0
180   0
200   0
220   4
240   12
260   4
280   0
300   20
320   4
340   0
360   8
C-P:ASP-S1
20   0
40   15
60   24
80   27
100   48
120   30
140   30
160   24
180   6
200   0
220   12
240   27
260   18
280   24
300   36
320   12
340   18
360   3
G-R5:CYS-CA
20   0
40   4
60   0
80   4
100   0
120   8
140   0
160   4
180   0
200   0
220   0
240   0
260   4
280   0
300   0
320   0
340   0
360   0
FHU-MY:VAL-S1
20   0
40   0
60   0
80   0
100   3

120 0
140 0
160 0
180 3
200 0
220 0
240 0
260 0
280 0
300 0
320 0
340 3
360 0
G-R5:ASP-CA
20 4
40 8
60 20
80 15
100 12
120 0
140 16
160 16
180 0
200 4
220 16
240 20
260 16
280 16
300 24
320 8
340 12
360 0
A-RIB:ARG-S1
20 4
40 56
60 68
80 92
100 100
120 68
140 56
160 60
180 16
200 0
220 36
240 68
260 72
280 104
300 56
320 60
340 32
360 28
G-RIB:MET-CA
20 4
40 0
60 12
80 4
100 8
120 12

140 8
160 20
180 8
200 0
220 0
240 4
260 16
280 12
300 8
320 12
340 16
360 4
C-Y:TYR-S1
20 0
40 0
60 6
80 12
100 6
120 12
140 3
160 0
180 0
200 0
220 0
240 6
260 12
280 9
300 15
320 6
340 3
360 0
A-R5:ILE-CA
20 4
40 4
60 12
80 4
100 4
120 12
140 0
160 8
180 0
200 0
220 4
240 12
260 4
280 0
300 16
320 4
340 0
360 0
G-RIB:SER-CA
20 4
40 24
60 52
80 24
100 44
120 24
140 44

160 20
180 20
200 0
220 32
240 20
260 36
280 44
300 52
320 24
340 16
360 12
FHU-MY:THR-CA
20 0
40 0
60 0
80 3
100 0
120 0
140 0
160 0
180 0
200 0
220 3
240 0
260 3
280 0
300 3
320 0
340 0
360 0
C-RIB:GLN-S1
20 9
40 12
60 12
80 36
100 30
120 15
140 15
160 15
180 12
200 0
220 12
240 6
260 21
280 18
300 24
320 33
340 27
360 21
A-P:MET-S1
20 0
40 0
60 8
80 8
100 12
120 8
140 24
160 4

180 0
200 0
220 0
240 4
260 28
280 12
300 8
320 8
340 0
360 0
A-P:CYS-CA
20 0
40 4
60 0
80 4
100 8
120 4
140 4
160 0
180 0
200 0
220 0
240 4
260 8
280 4
300 0
320 0
340 0
360 4
IU-RIB:ALA-S1
20 0
40 0
60 0
80 0
100 3
120 0
140 0
160 0
180 0
200 0
220 0
240 0
260 0
280 0
300 3
320 0
340 0
360 0
G-R6:LYS-S1
20 4
40 8
60 36
80 28
100 44
120 32
140 16
160 20
180 0

200 0
220 4
240 24
260 48
280 56
300 44
320 32
340 12
360 4
QUO-M6:LEU-CA
20 0
40 0
60 0
80 0
100 0
120 0
140 0
160 0
180 0
200 0
220 0
240 0
260 4
280 0
300 0
320 0
340 0
360 0
A-R5:LEU-CA
20 8
40 4
60 12
80 28
100 24
120 16
140 4
160 4
180 0
200 0
220 4
240 4
260 16
280 8
300 20
320 8
340 12
360 0
A-P:PHE-CA
20 0
40 0
60 8
80 8
100 8
120 4
140 4
160 16
180 4
200 0

220   4
240   8
260   8
280   12
300   32
320   16
340   12
360   0
FHU-P:HIS-S2
20   0
40   0
60   0
80   0
100   0
120   0
140   0
160   0
180   0
200   0
220   0
240   0
260   0
280   6
300   0
320   0
340   0
360   0
G-P:ARG-S1
20   0
40   28
60   36
80   88
100   160
120   108
140   108
160   80
180   52
200   0
220   16
240   36
260   84
280   136
300   140
320   100
340   84
360   44
U-P:TRP-S1
20   0
40   0
60   6
80   3
100   15
120   3
140   3
160   3
180   3
200   0
220   0

240 0
260 3
280 12
300 15
320 6
340 3
360 0
IU-RIB:PRO-S1
20 0
40 0
60 0
80 0
100 0
120 3
140 0
160 0
180 0
200 0
220 0
240 0
260 0
280 0
300 0
320 0
340 0
360 0
G-RIB:GLN-CA
20 0
40 8
60 20
80 40
100 28
120 32
140 44
160 32
180 16
200 4
220 8
240 8
260 24
280 8
300 16
320 24
340 28
360 4
U-Y:TYR-CA
20 0
40 0
60 3
80 3
100 9
120 0
140 6
160 0
180 0
200 0
220 3
240 3

260 6
280 30
300 12
320 9
340 3
360 0
U-RIB:ILE-CA
20 0
40 0
60 0
80 9
100 6
120 15
140 12
160 3
180 0
200 0
220 3
240 0
260 3
280 12
300 3
320 9
340 3
360 3
DA-M5:THR-CA
20 0
40 0
60 0
80 0
100 0
120 0
140 0
160 0
180 0
200 0
220 0
240 0
260 0
280 0
300 0
320 3
340 0
360 0
FHU-RIB:TYR-S1
20 0
40 0
60 0
80 0
100 0
120 0
140 0
160 0
180 0
200 0
220 0
240 0
260 0

280 0
300 3
320 3
340 0
360 0
G-R6:GLN-S2
20 0
40 16
60 48
80 44
100 28
120 35
140 12
160 0
180 4
200 0
220 12
240 16
260 40
280 36
300 24
320 4
340 8
360 0
C-RIB:ILE-S1
20 0
40 3
60 9
80 9
100 21
120 12
140 6
160 12
180 0
200 0
220 0
240 9
260 21
280 18
300 15
320 9
340 6
360 0
A-R6:MET-S2
20 0
40 4
60 4
80 20
100 4
120 12
140 4
160 8
180 0
200 0
220 8
240 4
260 4
280 16

300 8
320 20
340 8
360 0
QUO-M5:ASN-S2
20 0
40 0
60 0
80 4
100 0
120 0
140 0
160 0
180 0
200 0
220 0
240 0
260 0
280 0
300 0
320 0
340 0
360 0
C31-P:GLN-S2
20 0
40 0
60 0
80 0
100 0
120 0
140 0
160 3
180 0
200 0
220 0
240 0
260 0
280 0
300 0
320 0
340 0
360 0
G-R5:TYR-S1
20 0
40 0
60 4
80 15
100 4
120 4
140 0
160 4
180 4
200 0
220 8
240 4
260 12
280 4
300 8

320 13
340 0
360 0
G-RIB:TRP-S2
20 0
40 0
60 4
80 12
100 12
120 12
140 4
160 4
180 4
200 0
220 0
240 4
260 12
280 4
300 20
320 12
340 4
360 0
IU-MY:ILE-S1
20 0
40 0
60 0
80 0
100 0
120 0
140 0
160 0
180 0
200 0
220 0
240 2
260 0
280 0
300 0
320 0
340 0
360 0
5BU-MY:ARG-S2
20 0
40 0
60 0
80 0
100 0
120 0
140 0
160 0
180 0
200 0
220 0
240 0
260 3
280 0
300 0
320 0

340 0
360 0
U-Y:LEU-S1
20 0
40 6
60 21
80 6
100 3
120 12
140 3
160 3
180 3
200 0
220 0
240 12
260 12
280 9
300 12
320 6
340 6
360 0
U-P:ASN-CA
20 0
40 3
60 6
80 15
100 15
120 30
140 24
160 12
180 3
200 0
220 0
240 6
260 18
280 18
300 18
320 9
340 9
360 0
U-P:ARG-S2
20 6
40 18
60 33
80 48
100 75
120 45
140 78
160 60
180 27
200 0
220 12
240 18
260 90
280 72
300 51
320 27
340 60

360 36
A-R6:LYS-S1
20 0
40 8
60 24
80 32
100 28
120 36
140 16
160 24
180 0
200 0
220 12
240 20
260 56
280 52
300 28
320 24
340 8
360 0
A-P:SER-S1
20 0
40 24
60 36
80 48
100 24
120 56
140 64
160 16
180 20
200 0
220 8
240 16
260 52
280 36
300 28
320 60
340 28
360 20
C-P:ASN-S2
20 0
40 9
60 24
80 6
100 42
120 15
140 39
160 24
180 9
200 0
220 3
240 30
260 24
280 57
300 42
320 24
340 36
360 9

G-R5:ILE-CA
20 4
40 0
60 4
80 3
100 12
120 0
140 0
160 0
180 0
200 4
220 8
240 8
260 8
280 12
300 8
320 0
340 8
360 0
A-R6:ILE-CA
20 0
40 4
60 8
80 8
100 0
120 8
140 12
160 8
180 4
200 0
220 0
240 4
260 8
280 12
300 16
320 4
340 8
360 4
FMU-RIB:ILE-CA
20 0
40 0
60 0
80 0
100 0
120 0
140 0
160 0
180 0
200 0
220 0
240 0
260 3
280 0
300 0
320 0
340 0
360 0
G-R6:ASN-CA

20 0
40 0
60 25
80 32
100 16
120 31
140 8
160 7
180 0
200 0
220 12
240 16
260 15
280 27
300 24
320 16
340 4
360 4
U-Y:LEU-S2
20 0
40 6
60 12
80 9
100 12
120 6
140 9
160 3
180 3
200 0
220 3
240 21
260 9
280 6
300 3
320 6
340 3
360 3
A-RIB:TYR-S2
20 0
40 8
60 8
80 28
100 20
120 24
140 12
160 8
180 4
200 0
220 4
240 8
260 32
280 12
300 24
320 16
340 0
360 0
G-P:TYR-S2
20 0

40 0
60 8
80 28
100 24
120 32
140 24
160 24
180 4
200 0
220 0
240 8
260 24
280 8
300 12
320 16
340 12
360 0
U-RIB:TRP-CA
20 0
40 0
60 9
80 3
100 0
120 3
140 3
160 3
180 0
200 0
220 3
240 6
260 6
280 3
300 6
320 0
340 3
360 0
A-R6:PRO-S1
20 0
40 4
60 8
80 20
100 40
120 28
140 28
160 20
180 4
200 0
220 12
240 24
260 12
280 36
300 23
320 24
340 4
360 4
G-P:GLN-CA
20 0
40 4

60   12
80   16
100   20
120   32
140   32
160   12
180   12
200   0
220   4
240   20
260   24
280   44
300   32
320   36
340   36
360   12
H2U-RIB:ASN-S2
20   0
40   0
60   0
80   0
100   0
120   0
140   0
160   0
180   0
200   0
220   3
240   0
260   3
280   0
300   3
320   0
340   0
360   0
C31-P:MET-S2
20   0
40   0
60   0
80   0
100   0
120   0
140   0
160   0
180   0
200   0
220   0
240   0
260   0
280   0
300   0
320   3
340   0
360   0
G-R6:ARG-S2
20   0
40   16
60   52

80   76
100   75
120   72
140   40
160   20
180   20
200   8
220   12
240   56
260   64
280   76
300   79
320   40
340   27
360   8
5BU-P:SER-CA
20   0
40   0
60   0
80   0
100   0
120   0
140   0
160   0
180   0
200   0
220   0
240   0
260   0
280   0
300   3
320   0
340   0
360   0
U34-P:ASN-S2
20   0
40   0
60   0
80   0
100   3
120   0
140   0
160   0
180   0
200   0
220   0
240   3
260   3
280   0
300   0
320   0
340   0
360   0
G-RIB:TYR-S1
20   0
40   0
60   8
80   8

100   8
120   12
140   4
160   12
180   0
200   0
220   4
240   4
260   20
280   24
300   8
320   0
340   16
360   0
C-P:ARG-S2
20   3
40   45
60   54
80   102
100   135
120   105
140   93
160   87
180   27
200   0
220   18
240   45
260   105
280   132
300   78
320   93
340   66
360   18
A-P:ARG-S2
20   0
40   56
60   72
80   120
100   132
120   92
140   96
160   112
180   28
200   0
220   28
240   68
260   112
280   132
300   88
320   96
340   88
360   20
FHU-MY:ASP-S2
20   0
40   0
60   0
80   0
100   3

120   0
140   0
160   0
180   0
200   0
220   0
240   0
260   0
280   3
300   3
320   0
340   0
360   0
G-P:MET-S1
20   0
40   0
60   8
80   12
100   12
120   8
140   12
160   4
180   4
200   0
220   0
240   8
260   4
280   8
300   4
320   12
340   8
360   0
A-R6:VAL-S1
20   0
40   8
60   12
80   40
100   24
120   24
140   16
160   16
180   0
200   0
220   8
240   12
260   20
280   12
300   36
320   12
340   8
360   0
A-RIB:PHE-S1
20   0
40   0
60   8
80   16
100   8
120   16

140 8
160 8
180 8
200 0
220 0
240 4
260 12
280 8
300 20
320 16
340 12
360 0
FMU-RIB:ARG-S1
20 0
40 0
60 0
80 0
100 0
120 3
140 0
160 0
180 0
200 0
220 0
240 3
260 0
280 0
300 0
320 0
340 0
360 0
FHU-MY:LEU-S1
20 0
40 0
60 0
80 3
100 0
120 0
140 3
160 3
180 0
200 0
220 0
240 0
260 3
280 0
300 0
320 0
340 3
360 0
A-R5:GLN-S1
20 0
40 12
60 12
80 16
100 12
120 24
140 12

160 8
180 8
200 4
220 12
240 8
260 24
280 4
300 16
320 12
340 0
360 0
C-RIB:GLN-S2
20 3
40 24
60 33
80 30
100 27
120 27
140 9
160 36
180 3
200 0
220 6
240 15
260 27
280 30
300 24
320 30
340 48
360 21
H2U-MY:LEU-S1
20 0
40 0
60 0
80 0
100 0
120 0
140 0
160 3
180 0
200 0
220 0
240 0
260 0
280 0
300 0
320 3
340 0
360 0
FMU-RIB:CYS-CA
20 0
40 0
60 0
80 0
100 0
120 0
140 0
160 3

180 0
200 0
220 0
240 0
260 0
280 0
300 0
320 0
340 0
360 0
G-P:ARG-CA
20 0
40 16
60 32
80 68
100 88
120 116
140 84
160 76
180 16
200 0
220 4
240 36
260 56
280 116
300 72
320 108
340 88
360 28
G-RIB:PHE-CA
20 0
40 8
60 8
80 16
100 24
120 24
140 8
160 8
180 0
200 0
220 0
240 12
260 8
280 20
300 4
320 12
340 12
360 12
A-R6:ASP-CA
20 0
40 8
60 4
80 8
100 24
120 12
140 16
160 12
180 0

200 0
220 0
240 4
260 12
280 8
300 32
320 8
340 4
360 4
DA-M5:GLU-S1
20 0
40 0
60 0
80 0
100 0
120 0
140 0
160 0
180 0
200 0
220 0
240 0
260 0
280 0
300 0
320 3
340 0
360 0
QUO-M6:ASN-S1
20 0
40 0
60 0
80 4
100 0
120 0
140 0
160 0
180 0
200 0
220 0
240 0
260 0
280 0
300 0
320 0
340 0
360 0
G-R6:TRP-CA
20 0
40 0
60 4
80 24
100 0
120 8
140 8
160 0
180 0
200 0

220   4
240   0
260   4
280   4
300   8
320   4
340   0
360   0
A-R6:ARG-S1
20   0
40   24
60   40
80   52
100   64
120   36
140   32
160   32
180   8
200   0
220   12
240   32
260   36
280   28
300   20
320   35
340   20
360   4
G-R6:ARG-CA
20   0
40   8
60   16
80   24
100   32
120   20
140   24
160   16
180   4
200   0
220   20
240   20
260   36
280   20
300   40
320   20
340   20
360   0
A-R5:ALA-CA
20   4
40   12
60   12
80   12
100   12
120   20
140   12
160   16
180   0
200   8
220   12

240   8
260   20
280   12
300   20
320   8
340   4
360   4
FMU-P:ALA-CA
20   0
40   0
60   0
80   0
100   0
120   0
140   0
160   0
180   0
200   0
220   0
240   3
260   0
280   0
300   0
320   0
340   0
360   0
FHU-RIB:THR-S1
20   0
40   0
60   0
80   3
100   0
120   0
140   0
160   0
180   0
200   0
220   0
240   0
260   3
280   3
300   0
320   0
340   0
360   0
QUO-M6:PHE-S2
20   0
40   0
60   0
80   0
100   0
120   0
140   0
160   0
180   0
200   0
220   0
240   0

260 4
280 0
300 4
320 0
340 0
360 0
FMU-P:ALA-S1
20 0
40 0
60 0
80 0
100 0
120 0
140 0
160 0
180 0
200 0
220 0
240 0
260 3
280 0
300 0
320 0
340 0
360 0
FMU-MY:PRO-CA
20 0
40 0
60 0
80 0
100 0
120 0
140 3
160 0
180 0
200 0
220 0
240 0
260 0
280 0
300 0
320 0
340 0
360 0
A-R5:ASP-CA
20 8
40 16
60 4
80 8
100 4
120 12
140 16
160 8
180 0
200 4
220 0
240 8
260 16

280   20
300   4
320   16
340   8
360   0
G-R6:TYR-CA
20   0
40   0
60   0
80   4
100   19
120   4
140   4
160   3
180   4
200   0
220   0
240   0
260   11
280   4
300   12
320   12
340   0
360   8
A-R5:ALA-S1
20   8
40   28
60   24
80   20
100   20
120   24
140   8
160   12
180   4
200   16
220   24
240   12
260   20
280   8
300   12
320   8
340   8
360   0
G-R5:TRP-S2
20   4
40   4
60   0
80   4
100   16
120   20
140   4
160   4
180   0
200   0
220   4
240   8
260   8
280   0

300 12
320 4
340 0
360 0
G-R6:GLU-CA
20 0
40 12
60 8
80 16
100 12
120 11
140 4
160 16
180 8
200 0
220 4
240 12
260 23
280 8
300 16
320 12
340 24
360 7
IU-RIB:GLN-S2
20 0
40 0
60 0
80 0
100 0
120 0
140 0
160 0
180 0
200 0
220 0
240 0
260 0
280 0
300 3
320 0
340 0
360 0
DA-M6:THR-S1
20 0
40 0
60 0
80 0
100 0
120 0
140 0
160 0
180 0
200 0
220 0
240 0
260 0
280 3
300 0

320   0
340   0
360   0
H2U-MY:PRO-CA
20   0
40   0
60   0
80   0
100   6
120   0
140   3
160   0
180   0
200   0
220   0
240   0
260   0
280   0
300   0
320   0
340   0
360   0
A-R5:ASP-S2
20   4
40   12
60   32
80   24
100   16
120   12
140   16
160   4
180   4
200   0
220   20
240   24
260   12
280   20
300   8
320   20
340   8
360   4
G-P:HIS-S1
20   0
40   8
60   12
80   36
100   28
120   24
140   20
160   4
180   4
200   0
220   16
240   4
260   20
280   20
300   40
320   12

340 20
360 4
H2U-P:ASN-S2
20 0
40 0
60 0
80 0
100 3
120 3
140 3
160 0
180 0
200 0
220 0
240 0
260 0
280 0
300 0
320 0
340 0
360 0
DA-M6:TYR-CA
20 0
40 0
60 0
80 0
100 0
120 3
140 0
160 0
180 0
200 0
220 0
240 0
260 0
280 0
300 3
320 0
340 0
360 0
A-P:PRO-CA
20 0
40 0
60 12
80 24
100 48
120 20
140 20
160 16
180 12
200 0
220 8
240 20
260 24
280 36
300 24
320 48
340 16

360   4
FHU-RIB:ASP-S2
20   0
40   0
60   0
80   0
100   0
120   0
140   0
160   3
180   0
200   0
220   0
240   0
260   0
280   0
300   0
320   0
340   3
360   0
U-P:VAL-CA
20   0
40   0
60   6
80   12
100   21
120   6
140   15
160   6
180   3
200   0
220   3
240   12
260   9
280   27
300   27
320   12
340   9
360   3
A-RIB:LYS-S2
20   16
40   48
60   84
80   40
100   76
120   40
140   40
160   24
180   8
200   36
220   60
240   96
260   68
280   80
300   60
320   36
340   68
360   16

H2U-MY:LYS-S1
20 0
40 0
60 0
80 0
100 0
120 0
140 0
160 0
180 0
200 0
220 0
240 0
260 3
280 0
300 0
320 0
340 0
360 0
U-P:TRP-S2
20 0
40 0
60 0
80 12
100 3
120 6
140 3
160 3
180 0
200 0
220 6
240 3
260 3
280 9
300 0
320 9
340 9
360 0
H2U-MY:ASN-S2
20 0
40 0
60 0
80 0
100 3
120 0
140 3
160 0
180 0
200 0
220 0
240 0
260 6
280 0
300 0
320 0
340 0
360 0
A-RIB:ASN-CA

20   0
40   4
60   16
80   16
100   36
120   20
140   36
160   8
180   8
200   0
220   8
240   36
260   24
280   24
300   20
320   36
340   12
360   24
U-Y:PRO-CA
20   0
40   6
60   18
80   15
100   3
120   3
140   9
160   3
180   3
200   0
220   0
240   9
260   9
280   12
300   18
320   6
340   9
360   3
G-R5:PRO-S1
20   20
40   16
60   36
80   20
100   16
120   16
140   4
160   8
180   0
200   8
220   44
240   27
260   20
280   16
300   20
320   4
340   12
360   0
C-RIB:ASN-S2
20   3

40   9
60   36
80   36
100   39
120   27
140   18
160   18
180   6
200   3
220   9
240   33
260   15
280   30
300   18
320   15
340   15
360   9
G-P:HIS-S2
20   0
40   8
60   28
80   24
100   40
120   28
140   20
160   12
180   0
200   0
220   12
240   12
260   24
280   60
300   28
320   32
340   20
360   8
A-RIB:TYR-S1
20   0
40   4
60   8
80   16
100   24
120   8
140   12
160   4
180   4
200   0
220   0
240   0
260   12
280   20
300   12
320   16
340   20
360   4
U31-P:SER-S1
20   0
40   0

60   0
80   0
100   0
120   0
140   0
160   0
180   0
200   0
220   0
240   0
260   0
280   0
300   3
320   0
340   0
360   0
G-R6:PHE-S2
20   4
40   0
60   8
80   16
100   16
120   4
140   4
160   4
180   0
200   0
220   4
240   16
260   8
280   11
300   4
320   0
340   3
360   0
FMU-RIB:ARG-S2
20   0
40   0
60   0
80   0
100   3
120   0
140   0
160   0
180   0
200   0
220   3
240   0
260   0
280   3
300   0
320   0
340   0
360   0
U-Y:GLN-S2
20   3
40   6
60   21

80 9
100 9
120 12
140 18
160 11
180 3
200 9
220 3
240 12
260 21
280 20
300 12
320 3
340 6
360 3
A-R5:ILE-S1
20 0
40 8
60 12
80 4
100 0
120 8
140 4
160 0
180 0
200 4
220 0
240 12
260 16
280 0
300 12
320 8
340 8
360 0
G-P:GLY-CA
20 4
40 20
60 48
80 92
100 72
120 76
140 68
160 40
180 28
200 0
220 32
240 76
260 60
280 104
300 88
320 56
340 24
360 8
G-R5:GLU-CA
20 4
40 8
60 8
80 4

100 4
120 8
140 3
160 8
180 4
200 0
220 4
240 16
260 12
280 15
300 4
320 15
340 12
360 8
G-R6:ILE-S1
20 4
40 0
60 4
80 0
100 12
120 8
140 0
160 4
180 0
200 0
220 0
240 8
260 7
280 8
300 7
320 0
340 0
360 4
U31-MY:THR-CA
20 0
40 0
60 0
80 0
100 0
120 0
140 0
160 0
180 3
200 0
220 0
240 0
260 0
280 0
300 0
320 3
340 0
360 0
FHU-RIB:SER-S1
20 0
40 0
60 0
80 0
100 6

120 0
140 3
160 0
180 0
200 0
220 0
240 0
260 0
280 0
300 0
320 3
340 0
360 0
FMU-MY:HIS-S1
20 0
40 0
60 0
80 0
100 0
120 0
140 0
160 0
180 0
200 0
220 0
240 3
260 0
280 0
300 0
320 0
340 0
360 0
A-P:GLU-CA
20 0
40 12
60 8
80 16
100 20
120 12
140 12
160 16
180 0
200 0
220 8
240 16
260 24
280 20
300 24
320 20
340 0
360 0
U31-RIB:GLN-S2
20 0
40 0
60 3
80 0
100 0
120 0

140 0
160 0
180 0
200 0
220 0
240 0
260 0
280 0
300 0
320 0
340 0
360 0
U-Y:ASP-CA
20 3
40 3
60 6
80 6
100 0
120 6
140 3
160 3
180 0
200 0
220 6
240 3
260 12
280 0
300 3
320 3
340 9
360 3
QUO-P:SER-S1
20 0
40 0
60 0
80 0
100 0
120 4
140 0
160 0
180 0
200 0
220 0
240 0
260 0
280 0
300 0
320 0
340 0
360 0
FHU-MY:ARG-S1
20 0
40 0
60 0
80 0
100 3
120 0
140 3

160 0
180 0
200 0
220 0
240 0
260 0
280 3
300 0
320 3
340 0
360 0
FHU-MY:THR-S1
20 0
40 0
60 0
80 0
100 3
120 0
140 0
160 0
180 0
200 0
220 3
240 0
260 0
280 6
300 0
320 0
340 0
360 0
IU-MY:LYS-CA
20 0
40 0
60 3
80 0
100 2
120 0
140 5
160 0
180 0
200 0
220 0
240 0
260 2
280 0
300 0
320 0
340 0
360 0
C-P:ILE-CA
20 0
40 0
60 18
80 12
100 6
120 36
140 12
160 6

180   3
200   0
220   0
240   0
260   24
280   9
300   21
320   18
340   12
360   3
FHU-RIB:TYR-S2
20   0
40   0
60   0
80   3
100   0
120   3
140   0
160   0
180   0
200   0
220   0
240   0
260   3
280   0
300   3
320   0
340   0
360   0
FHU-P:VAL-S1
20   0
40   0
60   0
80   0
100   0
120   3
140   0
160   0
180   0
200   0
220   0
240   0
260   0
280   0
300   0
320   3
340   0
360   0
G-R5:TYR-CA
20   0
40   8
60   0
80   15
100   0
120   4
140   4
160   4
180   4

200 4
220 0
240 8
260 12
280 0
300 7
320 14
340 0
360 0
G-R6:ASP-S1
20 0
40 16
60 8
80 24
100 15
120 32
140 12
160 12
180 4
200 0
220 12
240 16
260 28
280 48
300 20
320 8
340 24
360 8
FMU-P:ARG-S1
20 0
40 0
60 0
80 0
100 3
120 3
140 0
160 0
180 0
200 0
220 0
240 3
260 0
280 0
300 0
320 0
340 0
360 0
U-P:HIS-S1
20 0
40 6
60 9
80 12
100 6
120 6
140 12
160 12
180 6
200 0

220 0
240 15
260 21
280 15
300 9
320 15
340 9
360 0
QUO-M5:ARG-S2
20 0
40 0
60 0
80 0
100 4
120 0
140 0
160 0
180 4
200 0
220 0
240 0
260 0
280 0
300 0
320 0
340 0
360 0
C31-RIB:THR-CA
20 0
40 0
60 0
80 0
100 0
120 0
140 0
160 3
180 0
200 0
220 0
240 3
260 0
280 3
300 0
320 0
340 0
360 0
U-Y:HIS-S1
20 0
40 6
60 12
80 3
100 9
120 3
140 6
160 3
180 0
200 0
220 12

240   3
260   0
280   6
300   9
320   3
340   3
360   0
IU-RIB:ARG-S2
20   0
40   0
60   0
80   0
100   3
120   0
140   0
160   0
180   0
200   0
220   0
240   0
260   0
280   0
300   0
320   0
340   0
360   0
C-RIB:LEU-CA
20   0
40   6
60   15
80   24
100   24
120   21
140   18
160   12
180   12
200   0
220   0
240   12
260   21
280   48
300   36
320   27
340   24
360   0
U-RIB:ILE-S1
20   3
40   0
60   3
80   6
100   6
120   9
140   12
160   6
180   0
200   0
220   3
240   6

260   6
280   12
300   0
320   6
340   12
360   3
A-RIB:ASP-S2
20   0
40   0
60   8
80   32
100   20
120   24
140   4
160   8
180   12
200   0
220   12
240   16
260   32
280   40
300   40
320   40
340   24
360   0
H2U-RIB:GLU-S1
20   0
40   0
60   0
80   0
100   0
120   0
140   0
160   0
180   0
200   0
220   0
240   0
260   0
280   3
300   0
320   0
340   0
360   0
A-P:CYS-S1
20   0
40   0
60   4
80   0
100   4
120   0
140   0
160   0
180   4
200   0
220   0
240   0
260   12

280 8
300 4
320 0
340 0
360 0
G-R5:HIS-S1
20 8
40 24
60 24
80 23
100 24
120 16
140 8
160 0
180 0
200 0
220 8
240 12
260 27
280 8
300 16
320 16
340 0
360 0
FHU-P:LYS-S2
20 0
40 0
60 0
80 0
100 0
120 3
140 3
160 0
180 0
200 0
220 0
240 0
260 0
280 0
300 3
320 0
340 3
360 0
G-R5:GLU-S1
20 0
40 24
60 20
80 16
100 8
120 0
140 15
160 8
180 4
200 4
220 16
240 16
260 8
280 15

300 19
320 20
340 12
360 4
FMU-RIB:ALA-S1
20 0
40 0
60 0
80 3
100 0
120 0
140 0
160 0
180 0
200 0
220 0
240 0
260 0
280 0
300 0
320 0
340 0
360 0
QUO-M5:LEU-S1
20 0
40 0
60 0
80 0
100 0
120 0
140 0
160 0
180 0
200 0
220 0
240 4
260 0
280 4
300 0
320 0
340 0
360 0
G-RIB:GLU-S2
20 4
40 4
60 12
80 24
100 40
120 24
140 20
160 48
180 8
200 0
220 8
240 20
260 24
280 36
300 8

320 24
340 52
360 20
G-P:GLU-CA
20 0
40 4
60 4
80 8
100 12
120 56
140 36
160 16
180 4
200 0
220 12
240 0
260 28
280 20
300 20
320 24
340 28
360 0
G-P:CYS-CA
20 0
40 0
60 0
80 0
100 4
120 4
140 8
160 0
180 0
200 0
220 4
240 0
260 0
280 0
300 0
320 4
340 0
360 4
U-P:ASN-S2
20 0
40 6
60 6
80 21
100 30
120 27
140 27
160 12
180 3
200 3
220 9
240 12
260 24
280 21
300 18
320 27

340 3
360 3
C31-RIB:GLU-S1
20 0
40 0
60 0
80 3
100 0
120 0
140 0
160 0
180 0
200 0
220 0
240 0
260 0
280 0
300 0
320 0
340 0
360 0
U34-MY:ASN-S1
20 0
40 0
60 0
80 0
100 0
120 0
140 0
160 0
180 0
200 0
220 0
240 3
260 3
280 0
300 0
320 0
340 0
360 0
U-RIB:PHE-S1
20 0
40 0
60 6
80 3
100 12
120 21
140 18
160 3
180 0
200 0
220 0
240 3
260 3
280 6
300 18
320 9
340 6

360 3
U31-P:LEU-S2
20 0
40 0
60 0
80 0
100 0
120 0
140 0
160 0
180 0
200 0
220 0
240 0
260 0
280 0
300 3
320 0
340 0
360 0
U-Y:GLN-S1
20 0
40 3
60 21
80 6
100 3
120 18
140 6
160 14
180 3
200 0
220 6
240 12
260 21
280 5
300 12
320 12
340 0
360 6
C31-MY:GLU-S2
20 0
40 3
60 0
80 0
100 0
120 0
140 3
160 0
180 0
200 0
220 0
240 0
260 0
280 0
300 0
320 0
340 0
360 0

U-Y:PRO-S1
20 6
40 6
60 12
80 15
100 9
120 3
140 12
160 3
180 3
200 0
220 3
240 15
260 15
280 9
300 18
320 0
340 6
360 3
G-R6:ALA-S1
20 4
40 4
60 8
80 28
100 7
120 32
140 24
160 8
180 4
200 0
220 24
240 28
260 24
280 24
300 12
320 20
340 4
360 0
FMU-P:PHE-CA
20 0
40 0
60 0
80 0
100 0
120 3
140 0
160 0
180 0
200 0
220 0
240 0
260 0
280 0
300 0
320 0
340 0
360 0
IU-RIB:GLN-S1

20 0
40 0
60 0
80 0
100 0
120 0
140 0
160 0
180 0
200 0
220 0
240 0
260 0
280 0
300 3
320 0
340 0
360 0
U-P:GLN-CA
20 0
40 6
60 12
80 12
100 12
120 12
140 6
160 12
180 6
200 0
220 0
240 9
260 0
280 18
300 15
320 33
340 6
360 6
U-P:ARG-S1
20 0
40 3
60 9
80 39
100 48
120 45
140 57
160 36
180 21
200 3
220 12
240 18
260 42
280 63
300 63
320 45
340 45
360 12
U-P:PRO-S1
20 0

40   3
60   9
80   15
100   18
120   24
140   21
160   15
180   18
200   0
220   6
240   15
260   12
280   21
300   15
320   12
340   6
360   6
QUO-M5:GLU-S2
20   0
40   0
60   0
80   0
100   0
120   0
140   4
160   0
180   0
200   0
220   0
240   0
260   0
280   0
300   0
320   0
340   0
360   0
G-P:ILE-CA
20   0
40   0
60   16
80   12
100   16
120   28
140   20
160   20
180   0
200   0
220   0
240   8
260   40
280   4
300   12
320   20
340   8
360   0
C-P:GLU-S2
20   3
40   6

60 24
80 15
100 45
120 45
140 21
160 27
180 6
200 0
220 6
240 9
260 18
280 42
300 30
320 18
340 9
360 9
G-R5:THR-S1
20 4
40 12
60 20
80 8
100 28
120 8
140 4
160 4
180 0
200 4
220 28
240 28
260 28
280 16
300 12
320 20
340 4
360 0
A-RIB:ARG-S2
20 24
40 52
60 120
80 108
100 108
120 92
140 68
160 24
180 20
200 32
220 60
240 76
260 144
280 88
300 84
320 48
340 60
360 24
DA-M6:HIS-S1
20 0
40 0
60 0

80 0
100 0
120 0
140 0
160 0
180 0
200 0
220 0
240 0
260 3
280 0
300 0
320 0
340 0
360 0
U-RIB:GLN-S1
20 0
40 9
60 9
80 9
100 12
120 9
140 12
160 9
180 6
200 0
220 3
240 9
260 12
280 18
300 9
320 21
340 9
360 9
FMU-P:PHE-S2
20 0
40 0
60 0
80 3
100 0
120 0
140 0
160 0
180 0
200 0
220 0
240 0
260 0
280 0
300 0
320 0
340 0
360 0
U31-P:HIS-S1
20 0
40 0
60 0
80 0

100 0
120 0
140 0
160 0
180 0
200 0
220 0
240 0
260 0
280 0
300 0
320 3
340 0
360 0
C-Y:MET-S2
20 0
40 6
60 9
80 18
100 6
120 6
140 0
160 0
180 0
200 9
220 18
240 3
260 6
280 12
300 6
320 0
340 0
360 0
H2U-RIB:ARG-S2
20 0
40 0
60 0
80 0
100 0
120 0
140 0
160 0
180 0
200 0
220 0
240 0
260 3
280 0
300 0
320 3
340 0
360 0
G-P:ASP-S1
20 0
40 4
60 20
80 24
100 40

120 56
140 36
160 28
180 8
200 0
220 12
240 24
260 12
280 44
300 76
320 44
340 44
360 16
A-R5:VAL-S1
20 12
40 16
60 8
80 28
100 36
120 8
140 8
160 8
180 0
200 0
220 16
240 4
260 12
280 16
300 24
320 12
340 4
360 0
FHU-MY:ARG-S2
20 0
40 0
60 0
80 3
100 3
120 0
140 0
160 0
180 0
200 0
220 0
240 0
260 3
280 3
300 0
320 0
340 0
360 0
A-R5:ASN-S1
20 20
40 4
60 16
80 24
100 28
120 8

140   28
160   12
180   0
200   0
220   12
240   32
260   16
280   12
300   12
320   8
340   4
360   7
G-RIB:HIS-S1
20   4
40   8
60   4
80   20
100   12
120   12
140   36
160   8
180   16
200   0
220   4
240   16
260   32
280   24
300   16
320   28
340   32
360   4
U34-P:ASN-CA
20   0
40   0
60   0
80   6
100   3
120   0
140   0
160   0
180   0
200   0
220   0
240   0
260   3
280   0
300   0
320   0
340   0
360   0
H2U-MY:GLN-S2
20   0
40   0
60   0
80   0
100   0
120   0
140   0

160 0
180 0
200 0
220 0
240 0
260 0
280 0
300 0
320 0
340 0
360 3
A-RIB:GLU-S1
20 0
40 4
60 4
80 28
100 44
120 28
140 36
160 8
180 4
200 0
220 0
240 4
260 24
280 20
300 24
320 20
340 20
360 8
FMU-MY:CYS-S1
20 0
40 0
60 0
80 0
100 3
120 0
140 0
160 0
180 0
200 0
220 0
240 0
260 0
280 0
300 0
320 0
340 0
360 0
DA-M5:LYS-S2
20 0
40 0
60 0
80 0
100 0
120 0
140 0
160 0

180 0
200 0
220 0
240 0
260 0
280 3
300 0
320 0
340 0
360 0
U31-P:MET-CA
20 0
40 0
60 0
80 0
100 0
120 0
140 0
160 0
180 0
200 0
220 0
240 0
260 3
280 0
300 3
320 0
340 0
360 0
U-Y:LEU-CA
20 3
40 6
60 12
80 6
100 6
120 15
140 3
160 0
180 3
200 0
220 0
240 6
260 3
280 3
300 21
320 12
340 9
360 0
QUO-RIB:LEU-S1
20 0
40 0
60 0
80 0
100 0
120 0
140 0
160 0
180 0

200 0
220 0
240 0
260 8
280 0
300 0
320 0
340 0
360 0
IU-MY:LYS-S2
20 0
40 0
60 0
80 0
100 2
120 0
140 3
160 0
180 3
200 0
220 0
240 0
260 3
280 0
300 2
320 0
340 2
360 0
G-R5:TRP-S1
20 4
40 4
60 0
80 8
100 24
120 8
140 0
160 0
180 0
200 0
220 8
240 8
260 0
280 8
300 12
320 0
340 4
360 0
A-R6:TRP-S1
20 0
40 0
60 0
80 8
100 4
120 4
140 0
160 0
180 0
200 0

220 0
240 4
260 12
280 8
300 0
320 4
340 4
360 0
U-P:ILE-CA
20 0
40 0
60 6
80 0
100 15
120 6
140 12
160 3
180 0
200 0
220 0
240 3
260 6
280 3
300 3
320 15
340 9
360 0
C-Y:SER-S1
20 3
40 21
60 27
80 41
100 30
120 12
140 6
160 3
180 3
200 12
220 24
240 33
260 30
280 36
300 21
320 6
340 12
360 0
A-P:ASP-CA
20 4
40 0
60 12
80 32
100 16
120 48
140 8
160 20
180 8
200 0
220 8

240   16
260   20
280   32
300   32
320   12
340   28
360   8
G-RIB:PRO-CA
20   0
40   4
60   32
80   60
100   48
120   28
140   28
160   40
180   12
200   4
220   12
240   16
260   24
280   32
300   40
320   24
340   16
360   0
C-Y:HIS-CA
20   0
40   9
60   3
80   9
100   6
120   6
140   6
160   3
180   3
200   0
220   15
240   15
260   15
280   6
300   6
320   6
340   0
360   0
QUO-M5:ASP-S2
20   0
40   0
60   0
80   0
100   0
120   4
140   0
160   0
180   0
200   0
220   0
240   0

260 0
280 0
300 0
320 4
340 0
360 0
U-Y:ARG-S2
20 9
40 24
60 42
80 42
100 54
120 29
140 21
160 12
180 18
200 27
220 6
240 30
260 18
280 30
300 9
320 24
340 18
360 3
U31-RIB:ASN-CA
20 0
40 0
60 0
80 0
100 0
120 0
140 0
160 0
180 0
200 0
220 0
240 3
260 0
280 0
300 0
320 0
340 0
360 0
C-P:ASP-CA
20 6
40 0
60 15
80 27
100 27
120 36
140 21
160 27
180 0
200 0
220 9
240 24
260 9

280 30
300 27
320 15
340 12
360 0
A-P:PRO-S1
20 0
40 4
60 24
80 20
100 44
120 20
140 12
160 32
180 0
200 0
220 28
240 12
260 24
280 36
300 28
320 36
340 32
360 4
IU-RIB:ILE-CA
20 0
40 0
60 0
80 0
100 0
120 0
140 0
160 0
180 0
200 0
220 0
240 0
260 0
280 3
300 0
320 0
340 0
360 0
G-P:GLN-S1
20 0
40 24
60 16
80 24
100 24
120 32
140 28
160 16
180 28
200 0
220 4
240 20
260 40
280 28

300 36
320 28
340 52
360 12
FHU-RIB:LEU-S1
20 0
40 0
60 0
80 0
100 3
120 0
140 0
160 0
180 0
200 0
220 0
240 0
260 0
280 3
300 0
320 0
340 0
360 0
U-P:LEU-CA
20 0
40 3
60 3
80 9
100 12
120 18
140 9
160 0
180 6
200 0
220 6
240 3
260 3
280 27
300 24
320 15
340 3
360 0
G-R6:ALA-CA
20 0
40 4
60 8
80 28
100 19
120 24
140 20
160 16
180 4
200 0
220 24
240 20
260 28
280 12
300 36

320   8
340   4
360   0
IU-MY:GLU-S2
20   0
40   0
60   0
80   0
100   0
120   0
140   2
160   0
180   0
200   0
220   0
240   0
260   0
280   0
300   0
320   0
340   0
360   0
H2U-MY:ARG-CA
20   0
40   0
60   0
80   0
100   0
120   0
140   0
160   0
180   0
200   0
220   0
240   0
260   0
280   0
300   0
320   3
340   0
360   0
FHU-RIB:SER-CA
20   0
40   0
60   0
80   0
100   0
120   0
140   3
160   0
180   0
200   0
220   0
240   0
260   0
280   0
300   0
320   3

340   0
360   0
DA-M6:SER-CA
20   0
40   0
60   0
80   0
100   0
120   0
140   0
160   0
180   0
200   0
220   0
240   0
260   0
280   0
300   3
320   3
340   0
360   0
C-P:PRO-S1
20   0
40   21
60   15
80   27
100   24
120   33
140   15
160   18
180   0
200   0
220   6
240   21
260   18
280   36
300   21
320   12
340   27
360   12
QUO-M6:GLN-S1
20   0
40   0
60   0
80   0
100   0
120   4
140   0
160   0
180   0
200   0
220   0
240   0
260   0
280   0
300   0
320   0
340   0

360 0
DA-M6:LYS-S1
20 0
40 0
60 0
80 0
100 0
120 0
140 0
160 0
180 0
200 0
220 0
240 0
260 0
280 0
300 3
320 0
340 0
360 0
FMU-MY:ARG-S1
20 0
40 0
60 3
80 0
100 0
120 0
140 0
160 0
180 0
200 0
220 0
240 0
260 0
280 0
300 0
320 0
340 0
360 0
A-RIB:ILE-CA
20 0
40 0
60 12
80 28
100 20
120 12
140 12
160 8
180 4
200 0
220 4
240 0
260 12
280 24
300 12
320 16
340 4
360 12

QUO-P:PHE-S2
20 0
40 4
60 0
80 0
100 0
120 0
140 0
160 0
180 0
200 0
220 0
240 0
260 0
280 0
300 0
320 0
340 0
360 0
FHU-RIB:LEU-S2
20 0
40 0
60 0
80 0
100 3
120 3
140 0
160 0
180 0
200 0
220 0
240 0
260 0
280 6
300 3
320 0
340 0
360 0
DA-M6:TYR-S1
20 0
40 0
60 0
80 0
100 3
120 0
140 0
160 0
180 0
200 0
220 0
240 0
260 0
280 0
300 3
320 0
340 0
360 0
A-RIB:PHE-S2

20 0
40 4
60 4
80 28
100 20
120 12
140 16
160 8
180 8
200 0
220 0
240 0
260 16
280 12
300 24
320 0
340 8
360 12
G-R5:MET-CA
20 4
40 0
60 8
80 24
100 4
120 0
140 0
160 0
180 0
200 0
220 0
240 8
260 8
280 20
300 0
320 12
340 4
360 4
U-P:CYS-CA
20 0
40 0
60 0
80 3
100 3
120 6
140 0
160 3
180 0
200 0
220 0
240 0
260 0
280 0
300 0
320 0
340 0
360 0
G-R6:HIS-S1
20 4

40 12
60 12
80 24
100 24
120 28
140 4
160 0
180 0
200 0
220 8
240 19
260 32
280 20
300 20
320 12
340 0
360 4
C-P:PHE-S1
20 0
40 3
60 0
80 12
100 0
120 6
140 9
160 9
180 0
200 0
220 0
240 6
260 12
280 6
300 12
320 0
340 9
360 0
A-R5:CYS-S1
20 0
40 4
60 0
80 12
100 0
120 0
140 0
160 0
180 0
200 0
220 0
240 4
260 0
280 8
300 4
320 0
340 0
360 0
C-RIB:GLU-S2
20 0
40 0

60   18
80   24
100   18
120   27
140   12
160   42
180   9
200   0
220   0
240   21
260   36
280   21
300   9
320   15
340   15
360   12
U34-RIB:ASN-S1
20   0
40   0
60   3
80   3
100   0
120   0
140   0
160   0
180   0
200   0
220   0
240   0
260   0
280   3
300   0
320   0
340   0
360   0
G-RIB:TYR-CA
20   0
40   0
60   4
80   12
100   4
120   0
140   4
160   12
180   0
200   0
220   8
240   0
260   20
280   16
300   12
320   0
340   12
360   0
C31-P:TYR-S1
20   0
40   0
60   0

80 0
100 3
120 0
140 0
160 0
180 0
200 0
220 0
240 0
260 0
280 3
300 0
320 0
340 0
360 0
U31-RIB:ASP-S1
20 0
40 3
60 0
80 3
100 0
120 0
140 0
160 3
180 0
200 0
220 0
240 0
260 0
280 0
300 0
320 3
340 0
360 0
QUO-RIB:ASN-S2
20 0
40 0
60 0
80 0
100 0
120 0
140 0
160 4
180 0
200 0
220 0
240 0
260 0
280 0
300 0
320 0
340 0
360 0
U31-MY:ASP-CA
20 0
40 0
60 0
80 3

100 3
120 3
140 0
160 0
180 0
200 0
220 0
240 0
260 0
280 0
300 0
320 0
340 0
360 0
H2U-MY:ALA-CA
20 0
40 0
60 0
80 0
100 0
120 0
140 3
160 0
180 0
200 0
220 0
240 0
260 0
280 0
300 0
320 0
340 0
360 0
G-R5:ALA-CA
20 0
40 12
60 8
80 16
100 27
120 0
140 16
160 8
180 0
200 0
220 28
240 36
260 24
280 24
300 24
320 16
340 4
360 4
FMU-MY:PHE-CA
20 0
40 0
60 0
80 0
100 0

120 0
140 0
160 0
180 0
200 0
220 0
240 0
260 3
280 0
300 0
320 0
340 0
360 0
A-R6:LEU-S1
20 0
40 12
60 8
80 32
100 32
120 20
140 4
160 8
180 0
200 0
220 16
240 4
260 24
280 32
300 36
320 16
340 4
360 4
FMU-RIB:PHE-S1
20 0
40 0
60 0
80 0
100 0
120 0
140 0
160 0
180 0
200 0
220 0
240 3
260 0
280 0
300 0
320 0
340 3
360 0
U31-MY:THR-S1
20 0
40 0
60 0
80 3
100 0
120 0

140 0
160 3
180 0
200 0
220 0
240 0
260 0
280 0
300 0
320 3
340 0
360 0
C-Y:PHE-S2
20 6
40 0
60 6
80 6
100 3
120 9
140 3
160 0
180 0
200 0
220 3
240 12
260 9
280 9
300 6
320 0
340 0
360 0
H2U-RIB:LYS-S1
20 0
40 0
60 0
80 0
100 0
120 0
140 0
160 0
180 0
200 0
220 0
240 0
260 0
280 3
300 0
320 0
340 0
360 0
A-R6:LEU-S2
20 0
40 4
60 4
80 24
100 32
120 16
140 20

160 4
180 8
200 0
220 4
240 12
260 24
280 36
300 48
320 16
340 16
360 0
FMU-RIB:ARG-CA
20 0
40 0
60 0
80 0
100 0
120 3
140 0
160 0
180 0
200 0
220 0
240 0
260 0
280 0
300 0
320 0
340 0
360 0
A-R5:MET-S2
20 0
40 8
60 12
80 4
100 16
120 20
140 12
160 12
180 0
200 0
220 4
240 4
260 12
280 0
300 12
320 12
340 0
360 8
G-R6:HIS-S2
20 4
40 15
60 28
80 40
100 20
120 44
140 16
160 4

180 0
200 4
220 20
240 20
260 23
280 16
300 48
320 16
340 0
360 4
U-P:LYS-CA
20 0
40 0
60 18
80 39
100 36
120 27
140 42
160 39
180 12
200 0
220 3
240 24
260 24
280 24
300 30
320 48
340 24
360 9
A-P:ILE-S1
20 0
40 4
60 12
80 8
100 8
120 16
140 8
160 12
180 4
200 0
220 4
240 12
260 16
280 16
300 0
320 20
340 12
360 8
G-P:PHE-CA
20 0
40 0
60 8
80 16
100 24
120 8
140 20
160 20
180 0

200 0
220 8
240 16
260 20
280 8
300 16
320 12
340 8
360 0
IU-MY:THR-S1
20 0
40 0
60 0
80 0
100 0
120 3
140 0
160 0
180 0
200 0
220 0
240 0
260 0
280 0
300 0
320 0
340 2
360 0
G-R6:TYR-S1
20 0
40 0
60 8
80 4
100 15
120 8
140 0
160 7
180 0
200 0
220 8
240 0
260 8
280 15
300 8
320 12
340 4
360 8
OMC-RIB:LYS-S1
20 0
40 0
60 0
80 0
100 0
120 0
140 0
160 0
180 0
200 0

220 0
240 3
260 0
280 0
300 0
320 0
340 0
360 0
FHU-MY:CYS-S1
20 0
40 0
60 0
80 0
100 0
120 0
140 0
160 0
180 0
200 0
220 0
240 0
260 0
280 3
300 0
320 0
340 0
360 0
DA-M6:ASN-CA
20 0
40 0
60 0
80 0
100 0
120 0
140 0
160 0
180 0
200 0
220 0
240 3
260 0
280 0
300 0
320 0
340 0
360 0
C31-MY:THR-CA
20 0
40 0
60 0
80 0
100 0
120 0
140 0
160 0
180 0
200 0
220 0

240 0
260 3
280 0
300 0
320 0
340 0
360 0
QUO-M6:ARG-S2
20 0
40 0
60 0
80 0
100 0
120 4
140 0
160 0
180 0
200 0
220 0
240 0
260 0
280 0
300 0
320 0
340 0
360 4
U31-RIB:PHE-CA
20 0
40 0
60 0
80 0
100 0
120 3
140 0
160 0
180 0
200 0
220 0
240 0
260 0
280 0
300 0
320 0
340 0
360 0
QUO-M6:ARG-CA
20 0
40 0
60 0
80 0
100 0
120 0
140 4
160 0
180 0
200 0
220 0
240 0

260 0
280 0
300 0
320 0
340 0
360 0
C31-RIB:ALA-S1
20 0
40 0
60 0
80 0
100 0
120 0
140 3
160 0
180 0
200 0
220 0
240 0
260 0
280 0
300 3
320 0
340 0
360 0
U31-P:ASN-S2
20 0
40 0
60 0
80 0
100 3
120 0
140 0
160 0
180 0
200 0
220 0
240 0
260 0
280 0
300 0
320 0
340 0
360 0
C-RIB:HIS-CA
20 0
40 0
60 3
80 21
100 15
120 6
140 0
160 21
180 6
200 0
220 0
240 9
260 3

280   15
300   30
320   15
340   9
360   9
FHU-MY:LEU-CA
20   0
40   0
60   0
80   3
100   0
120   0
140   0
160   3
180   0
200   0
220   0
240   0
260   3
280   0
300   0
320   0
340   3
360   0
M2G-P:GLU-CA
20   0
40   0
60   0
80   0
100   0
120   0
140   0
160   0
180   0
200   0
220   0
240   0
260   0
280   0
300   0
320   0
340   0
360   4
U-RIB:TYR-CA
20   0
40   3
60   0
80   9
100   3
120   6
140   0
160   9
180   0
200   0
220   0
240   0
260   0
280   21

300 12
320 9
340 15
360 6
C-RIB:MET-S1
20 0
40 0
60 0
80 18
100 15
120 6
140 0
160 9
180 9
200 0
220 0
240 9
260 6
280 9
300 27
320 6
340 0
360 3
FMU-MY:ASN-CA
20 0
40 0
60 0
80 0
100 3
120 0
140 0
160 0
180 0
200 0
220 0
240 0
260 0
280 0
300 0
320 0
340 0
360 0
FMU-P:ASP-S1
20 0
40 0
60 0
80 0
100 0
120 0
140 3
160 0
180 0
200 0
220 0
240 0
260 0
280 0
300 0

320 0
340 0
360 0
U-RIB:ALA-S1
20 6
40 12
60 24
80 33
100 18
120 15
140 18
160 24
180 0
200 3
220 9
240 21
260 21
280 30
300 12
320 12
340 9
360 6
QUO-M5:ARG-CA
20 0
40 0
60 0
80 0
100 0
120 4
140 0
160 0
180 0
200 0
220 0
240 0
260 0
280 0
300 0
320 0
340 0
360 0
U-Y:ASP-S2
20 3
40 6
60 12
80 15
100 3
120 9
140 15
160 9
180 0
200 3
220 3
240 18
260 9
280 3
300 6
320 3

340 9
360 0
H2U-P:PRO-S1
20 0
40 0
60 0
80 0
100 3
120 0
140 0
160 0
180 0
200 0
220 0
240 0
260 0
280 0
300 0
320 0
340 0
360 0
U34-RIB:ASN-CA
20 0
40 0
60 0
80 3
100 0
120 0
140 0
160 0
180 0
200 0
220 0
240 3
260 3
280 3
300 0
320 0
340 0
360 0
FHU-P:LEU-CA
20 0
40 0
60 3
80 0
100 0
120 0
140 0
160 3
180 0
200 0
220 0
240 3
260 0
280 0
300 0
320 0
340 3

360 3
5BU-RIB:PRO-CA
20 0
40 3
60 0
80 0
100 0
120 0
140 0
160 0
180 0
200 0
220 0
240 0
260 0
280 0
300 0
320 0
340 0
360 0
G-R6:SER-S1
20 4
40 20
60 48
80 28
100 20
120 24
140 8
160 4
180 4
200 4
220 16
240 11
260 48
280 44
300 40
320 28
340 4
360 0
G-RIB:PHE-S1
20 4
40 8
60 0
80 16
100 16
120 32
140 8
160 4
180 4
200 0
220 0
240 12
260 12
280 16
300 8
320 4
340 24
360 8

IU-MY:ILE-CA
20 0
40 0
60 3
80 0
100 0
120 0
140 0
160 0
180 0
200 0
220 0
240 2
260 0
280 0
300 0
320 0
340 0
360 0
U-RIB:GLN-S2
20 0
40 12
60 9
80 15
100 24
120 18
140 12
160 12
180 6
200 0
220 6
240 27
260 12
280 21
300 15
320 9
340 18
360 0
U-Y:ARG-CA
20 3
40 3
60 15
80 36
100 24
120 15
140 18
160 3
180 3
200 0
220 6
240 6
260 6
280 9
300 15
320 9
340 15
360 0
U31-MY:MET-CA

20 0
40 0
60 0
80 3
100 0
120 0
140 0
160 0
180 0
200 0
220 0
240 0
260 0
280 0
300 0
320 0
340 0
360 0
U-P:TYR-CA
20 0
40 3
60 9
80 9
100 3
120 0
140 6
160 3
180 0
200 0
220 0
240 0
260 6
280 9
300 9
320 3
340 0
360 0
U34-RIB:SER-CA
20 0
40 0
60 0
80 0
100 0
120 0
140 0
160 0
180 0
200 0
220 0
240 0
260 0
280 0
300 3
320 0
340 0
360 0
G-R6:THR-CA
20 0

40 16
60 8
80 12
100 20
120 12
140 8
160 4
180 0
200 0
220 12
240 8
260 28
280 20
300 24
320 4
340 0
360 0
U34-MY:SER-CA
20 0
40 0
60 0
80 0
100 0
120 0
140 3
160 0
180 0
200 0
220 0
240 0
260 0
280 0
300 3
320 0
340 3
360 0
G-P:PHE-S2
20 0
40 8
60 20
80 16
100 20
120 24
140 12
160 20
180 0
200 0
220 4
240 4
260 4
280 8
300 4
320 20
340 4
360 4
A-RIB:LYS-S1
20 4
40 12

60 44
80 60
100 60
120 28
140 48
160 16
180 12
200 12
220 20
240 56
260 72
280 28
300 44
320 72
340 40
360 16
U-Y:ILE-S1
20 3
40 0
60 3
80 9
100 12
120 12
140 3
160 0
180 0
200 0
220 3
240 5
260 3
280 3
300 3
320 9
340 3
360 0
U31-MY:PHE-S1
20 0
40 0
60 0
80 0
100 0
120 0
140 0
160 0
180 0
200 0
220 0
240 0
260 0
280 3
300 0
320 0
340 0
360 0
C-Y:PHE-CA
20 0
40 0
60 6

80   3
100   12
120   3
140   3
160   6
180   0
200   0
220   3
240   0
260   3
280   6
300   3
320   3
340   3
360   3
QUO-M5:LEU-CA
20   0
40   0
60   0
80   0
100   0
120   0
140   0
160   0
180   0
200   0
220   0
240   0
260   0
280   4
300   0
320   0
340   0
360   0
A-RIB:ILE-S1
20   0
40   0
60   4
80   20
100   16
120   20
140   16
160   8
180   8
200   0
220   8
240   0
260   12
280   8
300   4
320   16
340   4
360   4
C31-MY:TYR-S2
20   0
40   0
60   6
80   0

100 0
120 0
140 0
160 0
180 0
200 0
220 0
240 0
260 0
280 0
300 0
320 0
340 0
360 0
A-R6:TRP-CA
20 0
40 0
60 4
80 4
100 4
120 4
140 0
160 0
180 0
200 0
220 0
240 4
260 12
280 4
300 4
320 0
340 4
360 0
H2U-MY:TRP-S2
20 0
40 0
60 0
80 0
100 0
120 0
140 0
160 0
180 0
200 0
220 0
240 0
260 0
280 0
300 3
320 0
340 0
360 0
IU-MY:LEU-S2
20 0
40 0
60 2
80 0
100 0

120 0
140 0
160 0
180 0
200 0
220 0
240 0
260 0
280 0
300 0
320 0
340 0
360 0
G-R5:LEU-S2
20 8
40 20
60 4
80 12
100 12
120 16
140 12
160 4
180 0
200 8
220 16
240 16
260 24
280 12
300 16
320 20
340 0
360 0
C31-P:SER-S1
20 0
40 0
60 0
80 0
100 0
120 0
140 0
160 0
180 0
200 0
220 0
240 0
260 0
280 0
300 0
320 3
340 0
360 0
IU-MY:SER-CA
20 0
40 0
60 0
80 2
100 0
120 0

140 0
160 0
180 0
200 0
220 0
240 0
260 0
280 0
300 0
320 0
340 0
360 0
U-RIB:GLY-CA
20 6
40 15
60 54
80 36
100 21
120 33
140 42
160 24
180 21
200 6
220 27
240 9
260 18
280 57
300 39
320 51
340 30
360 9
C-RIB:SER-S1
20 6
40 15
60 36
80 45
100 39
120 39
140 45
160 18
180 3
200 3
220 24
240 39
260 24
280 30
300 39
320 39
340 24
360 18
U34-P:PHE-S2
20 0
40 0
60 0
80 0
100 0
120 0
140 0

160   0
180   0
200   0
220   0
240   0
260   0
280   0
300   3
320   0
340   0
360   0
FHU-MY:TYR-S2
20   0
40   0
60   3
80   0
100   3
120   0
140   0
160   0
180   0
200   0
220   0
240   3
260   0
280   0
300   3
320   0
340   0
360   0
U31-RIB:ASP-CA
20   0
40   3
60   0
80   3
100   0
120   0
140   3
160   0
180   0
200   0
220   0
240   0
260   0
280   0
300   0
320   3
340   0
360   0
FMU-MY:ASP-CA
20   0
40   0
60   0
80   0
100   0
120   0
140   0
160   3

180 0
200 0
220 0
240 0
260 0
280 0
300 0
320 0
340 0
360 0
C-P:ASP-S2
20 0
40 21
60 12
80 39
100 48
120 36
140 36
160 39
180 3
200 0
220 21
240 24
260 30
280 24
300 33
320 12
340 12
360 6
A-RIB:GLU-S2
20 0
40 4
60 16
80 28
100 8
120 52
140 24
160 36
180 12
200 0
220 0
240 8
260 28
280 28
300 28
320 36
340 20
360 4
U-P:GLU-S1
20 0
40 3
60 3
80 9
100 6
120 27
140 18
160 9
180 3

200 0
220 3
240 6
260 15
280 9
300 15
320 18
340 18
360 0
U-RIB:TRP-S2
20 0
40 0
60 3
80 9
100 0
120 9
140 3
160 3
180 0
200 0
220 0
240 3
260 12
280 6
300 0
320 0
340 3
360 3
C31-P:ASP-S2
20 0
40 0
60 0
80 0
100 0
120 3
140 0
160 0
180 0
200 0
220 0
240 0
260 0
280 0
300 0
320 6
340 0
360 0
FHU-P:THR-CA
20 0
40 0
60 3
80 3
100 0
120 0
140 0
160 0
180 0
200 0

220 0
240 0
260 3
280 0
300 0
320 3
340 0
360 0
U31-RIB:MET-S2
20 0
40 0
60 0
80 0
100 0
120 3
140 0
160 0
180 0
200 0
220 0
240 0
260 0
280 0
300 0
320 0
340 0
360 0
DA-M6:HIS-CA
20 0
40 0
60 0
80 0
100 0
120 0
140 0
160 0
180 0
200 0
220 0
240 0
260 3
280 0
300 0
320 0
340 0
360 0
U31-P:ARG-S1
20 0
40 0
60 0
80 0
100 0
120 0
140 0
160 0
180 0
200 0
220 0

240 0
260 0
280 0
300 0
320 3
340 0
360 0
DA-M5:HIS-CA
20 0
40 0
60 0
80 0
100 0
120 0
140 0
160 0
180 0
200 0
220 0
240 0
260 3
280 0
300 0
320 0
340 0
360 0
DA-M5:HIS-S2
20 0
40 0
60 0
80 0
100 0
120 0
140 0
160 0
180 0
200 0
220 0
240 0
260 0
280 3
300 0
320 0
340 0
360 0
C-Y:PHE-S1
20 0
40 0
60 0
80 6
100 6
120 6
140 3
160 6
180 0
200 3
220 3
240 0

260 9
280 9
300 3
320 3
340 0
360 3
IU-RIB:ARG-CA
20 0
40 0
60 0
80 0
100 0
120 0
140 0
160 0
180 0
200 0
220 0
240 0
260 0
280 3
300 0
320 0
340 0
360 0
U31-RIB:ASP-S2
20 0
40 3
60 0
80 0
100 3
120 0
140 0
160 3
180 0
200 0
220 0
240 0
260 0
280 0
300 3
320 0
340 3
360 0
C-P:THR-S1
20 3
40 15
60 24
80 36
100 33
120 27
140 39
160 30
180 6
200 0
220 9
240 15
260 30

280 30
300 39
320 18
340 27
360 9
U31-RIB:THR-S1
20 0
40 0
60 0
80 0
100 3
120 3
140 0
160 0
180 0
200 0
220 0
240 0
260 0
280 0
300 0
320 0
340 0
360 0
C31-MY:LEU-S2
20 0
40 0
60 0
80 0
100 0
120 0
140 0
160 0
180 0
200 0
220 0
240 0
260 0
280 3
300 0
320 0
340 0
360 0
FMU-MY:ASP-S2
20 0
40 0
60 0
80 0
100 0
120 0
140 0
160 3
180 0
200 0
220 0
240 0
260 0
280 0

300 0
320 0
340 0
360 0
QUO-RIB:LYS-S2
20 0
40 0
60 0
80 0
100 0
120 4
140 0
160 0
180 0
200 0
220 0
240 0
260 0
280 0
300 0
320 0
340 0
360 0
FHU-MY:ALA-S1
20 0
40 3
60 0
80 0
100 0
120 3
140 0
160 3
180 0
200 0
220 0
240 0
260 0
280 0
300 3
320 0
340 0
360 0
C-Y:GLN-CA
20 0
40 9
60 3
80 12
100 6
120 6
140 6
160 0
180 0
200 3
220 18
240 18
260 15
280 3
300 6

320 0
340 3
360 0
FMU-MY:ASP-S1
20 0
40 0
60 0
80 0
100 0
120 0
140 0
160 3
180 0
200 0
220 0
240 0
260 0
280 0
300 0
320 0
340 0
360 0
FHU-MY:SER-S1
20 0
40 0
60 0
80 0
100 3
120 0
140 0
160 0
180 0
200 0
220 0
240 0
260 0
280 0
300 0
320 0
340 0
360 0
DA-M6:ASN-S1
20 0
40 0
60 0
80 0
100 0
120 0
140 3
160 0
180 0
200 0
220 0
240 3
260 0
280 0
300 0
320 0

340 0
360 0
C-RIB:LYS-CA
20 3
40 27
60 24
80 39
100 33
120 33
140 24
160 24
180 9
200 3
220 18
240 24
260 60
280 48
300 30
320 45
340 36
360 9
U-RIB:MET-S2
20 0
40 0
60 0
80 15
100 6
120 12
140 0
160 3
180 6
200 0
220 0
240 3
260 12
280 3
300 3
320 3
340 3
360 0
QUO-P:LEU-CA
20 0
40 0
60 0
80 0
100 8
120 0
140 0
160 0
180 0
200 0
220 0
240 0
260 0
280 0
300 0
320 0
340 0

360 0
GTP-M5:SER-S1
20 0
40 0
60 3
80 0
100 0
120 0
140 0
160 0
180 0
200 0
220 0
240 0
260 0
280 0
300 3
320 0
340 0
360 0
C-Y:PRO-S1
20 6
40 5
60 24
80 18
100 21
120 12
140 6
160 6
180 0
200 12
220 9
240 15
260 18
280 21
300 6
320 6
340 3
360 0
A-RIB:TRP-S1
20 0
40 4
60 0
80 0
100 8
120 8
140 16
160 4
180 4
200 0
220 0
240 0
260 8
280 8
300 8
320 8
340 12
360 0

DA-M6:LEU-S1
20 0
40 0
60 0
80 0
100 0
120 3
140 0
160 0
180 0
200 0
220 0
240 0
260 0
280 0
300 3
320 0
340 0
360 0
U-Y:TYR-S2
20 0
40 3
60 0
80 11
100 6
120 6
140 6
160 3
180 0
200 0
220 3
240 9
260 12
280 15
300 21
320 12
340 0
360 0
C-P:GLU-CA
20 0
40 3
60 12
80 9
100 18
120 21
140 15
160 6
180 12
200 0
220 9
240 3
260 9
280 27
300 27
320 24
340 15
360 3
C-Y:MET-CA

20 0
40 6
60 12
80 6
100 3
120 3
140 0
160 0
180 0
200 0
220 3
240 0
260 3
280 9
300 3
320 0
340 0
360 0
C31-RIB:GLN-S2
20 0
40 0
60 0
80 0
100 0
120 0
140 3
160 0
180 0
200 0
220 0
240 0
260 0
280 0
300 0
320 0
340 0
360 0
G-R5:GLN-S1
20 0
40 28
60 40
80 24
100 4
120 4
140 0
160 4
180 0
200 12
220 28
240 20
260 48
280 12
300 12
320 0
340 4
360 0
IU-MY:VAL-CA
20 0

40 0
60 0
80 0
100 2
120 2
140 0
160 0
180 0
200 0
220 0
240 0
260 0
280 0
300 0
320 0
340 0
360 0
GTP-M5:ALA-S1
20 0
40 0
60 0
80 0
100 0
120 0
140 0
160 0
180 0
200 0
220 0
240 0
260 0
280 3
300 0
320 0
340 0
360 0
G-R6:HIS-CA
20 0
40 0
60 12
80 16
100 12
120 20
140 4
160 0
180 0
200 0
220 12
240 15
260 32
280 8
300 24
320 8
340 0
360 0
G-R6:MET-CA
20 0
40 4

60 8
80 20
100 4
120 8
140 0
160 0
180 0
200 0
220 0
240 12
260 8
280 8
300 12
320 8
340 4
360 4
FMU-P:PHE-S1
20 0
40 0
60 0
80 0
100 3
120 0
140 0
160 0
180 0
200 0
220 0
240 0
260 0
280 0
300 0
320 0
340 0
360 0
C-RIB:PHE-CA
20 0
40 0
60 6
80 12
100 12
120 12
140 6
160 9
180 0
200 0
220 0
240 0
260 9
280 9
300 3
320 9
340 0
360 3
U-RIB:ASN-S2
20 3
40 9
60 12

80 27
100 21
120 24
140 21
160 21
180 3
200 0
220 15
240 18
260 12
280 18
300 15
320 24
340 33
360 9
U34-P:SER-CA
20 0
40 0
60 0
80 3
100 0
120 3
140 0
160 0
180 0
200 0
220 0
240 0
260 0
280 0
300 0
320 0
340 0
360 0
H2U-P:ASN-S1
20 0
40 0
60 0
80 0
100 3
120 3
140 3
160 0
180 0
200 0
220 0
240 0
260 0
280 0
300 0
320 0
340 0
360 0
C31-P:GLN-CA
20 0
40 0
60 0
80 0

100 0
120 0
140 0
160 3
180 0
200 0
220 0
240 0
260 0
280 0
300 0
320 0
340 0
360 0
QUO-P:LEU-S1
20 0
40 0
60 0
80 4
100 4
120 0
140 0
160 0
180 0
200 0
220 0
240 0
260 0
280 0
300 0
320 0
340 0
360 0
G-R6:TRP-S2
20 0
40 4
60 0
80 8
100 8
120 24
140 4
160 0
180 4
200 4
220 4
240 8
260 4
280 12
300 8
320 8
340 0
360 0
G-RIB:ASP-S2
20 0
40 4
60 32
80 48
100 52

120   44
140   16
160   36
180   12
200   4
220   0
240   24
260   32
280   40
300   24
320   48
340   40
360   24
I-RIB:ALA-S1
20   0
40   0
60   0
80   0
100   0
120   0
140   0
160   2
180   0
200   0
220   0
240   0
260   0
280   0
300   0
320   0
340   0
360   0
H2U-RIB:GLY-CA
20   0
40   0
60   0
80   0
100   0
120   0
140   0
160   0
180   0
200   0
220   0
240   0
260   0
280   0
300   0
320   3
340   0
360   0
H2U-P:LYS-S2
20   0
40   3
60   0
80   0
100   0
120   0

140   0
160   0
180   3
200   0
220   0
240   0
260   0
280   0
300   0
320   0
340   0
360   0
QUO-M6:LEU-S1
20   0
40   0
60   0
80   0
100   0
120   0
140   0
160   0
180   0
200   0
220   0
240   0
260   4
280   0
300   0
320   0
340   0
360   0
QUO-RIB:ASN-S1
20   0
40   0
60   0
80   0
100   0
120   0
140   0
160   4
180   0
200   0
220   0
240   0
260   0
280   0
300   0
320   0
340   0
360   0
H2U-MY:PHE-CA
20   0
40   0
60   0
80   0
100   0
120   0
140   3

160 0
180 0
200 0
220 0
240 0
260 0
280 0
300 0
320 0
340 0
360 0
FMU-P:ARG-S2
20 0
40 0
60 0
80 0
100 0
120 6
140 0
160 0
180 0
200 0
220 0
240 3
260 0
280 0
300 0
320 0
340 0
360 0
U31-MY:GLN-S1
20 0
40 0
60 3
80 0
100 0
120 0
140 0
160 0
180 0
200 0
220 0
240 0
260 0
280 0
300 0
320 0
340 0
360 0
OMC-MY:LYS-S2
20 0
40 0
60 3
80 0
100 0
120 0
140 0
160 0

180 0
200 0
220 0
240 0
260 0
280 0
300 0
320 0
340 0
360 0
U-RIB:LYS-CA
20 0
40 6
60 3
80 27
100 48
120 33
140 12
160 27
180 6
200 3
220 12
240 15
260 36
280 36
300 9
320 24
340 24
360 3
A-RIB:PRO-CA
20 0
40 4
60 16
80 20
100 32
120 44
140 12
160 20
180 16
200 0
220 0
240 16
260 36
280 16
300 24
320 40
340 12
360 0
H2U-P:PRO-CA
20 0
40 0
60 0
80 0
100 3
120 0
140 0
160 0
180 0

200 0
220 0
240 0
260 0
280 0
300 0
320 0
340 0
360 0
G-P:SER-S1
20 0
40 24
60 24
80 32
100 48
120 60
140 84
160 48
180 8
200 0
220 16
240 16
260 64
280 68
300 48
320 40
340 44
360 8
H2U-RIB:LEU-S2
20 0
40 0
60 0
80 0
100 0
120 0
140 0
160 0
180 0
200 0
220 0
240 3
260 0
280 0
300 0
320 0
340 0
360 0
H2U-P:GLU-S2
20 0
40 0
60 3
80 0
100 0
120 0
140 3
160 0
180 0
200 0

220 0
240 0
260 0
280 0
300 0
320 0
340 0
360 0
A-RIB:TRP-CA
20 0
40 0
60 4
80 0
100 8
120 16
140 8
160 4
180 4
200 0
220 0
240 4
260 8
280 8
300 4
320 8
340 8
360 0
QUO-M5:GLU-S1
20 0
40 0
60 0
80 0
100 0
120 0
140 4
160 0
180 0
200 0
220 0
240 0
260 0
280 0
300 0
320 0
340 0
360 0
QUO-M5:ASP-S1
20 0
40 0
60 0
80 0
100 0
120 4
140 0
160 0
180 0
200 0
220 0

240   0
260   0
280   0
300   0
320   4
340   0
360   0
FMU-RIB:ALA-CA
20   0
40   0
60   0
80   0
100   3
120   0
140   0
160   0
180   0
200   0
220   0
240   0
260   0
280   0
300   0
320   0
340   0
360   0
U-Y:TRP-S1
20   0
40   0
60   6
80   3
100   9
120   0
140   0
160   0
180   0
200   0
220   3
240   0
260   3
280   3
300   0
320   0
340   3
360   0
DA-M6:LYS-CA
20   0
40   0
60   0
80   0
100   0
120   0
140   0
160   0
180   0
200   0
220   0
240   0

260   0
280   3
300   0
320   0
340   0
360   0
DA-M5:ASN-CA
20   0
40   0
60   0
80   0
100   0
120   0
140   0
160   0
180   0
200   0
220   0
240   3
260   0
280   0
300   0
320   0
340   0
360   0
A-R6:LYS-CA
20   0
40   12
60   16
80   16
100   28
120   24
140   24
160   12
180   0
200   0
220   4
240   20
260   32
280   44
300   20
320   20
340   4
360   0
U31-P:TYR-S1
20   0
40   0
60   0
80   3
100   0
120   0
140   0
160   0
180   0
200   0
220   0
240   0
260   0

280   0
300   0
320   0
340   0
360   0
U-P:PHE-S1
20   0
40   6
60   3
80   3
100   3
120   6
140   6
160   3
180   3
200   0
220   3
240   3
260   6
280   15
300   6
320   3
340   0
360   3
FHU-P:GLY-CA
20   0
40   0
60   0
80   3
100   0
120   0
140   0
160   0
180   0
200   0
220   0
240   0
260   3
280   3
300   3
320   3
340   0
360   0
5BU-MY:PRO-CA
20   0
40   0
60   3
80   0
100   0
120   0
140   0
160   0
180   0
200   0
220   0
240   0
260   0
280   0

300 0
320 0
340 0
360 0
U-Y:ALA-S1
20 6
40 21
60 12
80 21
100 3
120 11
140 11
160 9
180 0
200 3
220 0
240 15
260 9
280 6
300 9
320 12
340 6
360 0
H2U-RIB:PRO-CA
20 0
40 0
60 0
80 0
100 0
120 0
140 0
160 0
180 0
200 0
220 0
240 0
260 0
280 0
300 3
320 0
340 0
360 0
IU-P:ARG-S1
20 0
40 0
60 3
80 0
100 0
120 0
140 0
160 0
180 0
200 0
220 0
240 0
260 0
280 0
300 0

320 0
340 3
360 0
IU-RIB:HIS-CA
20 0
40 0
60 0
80 0
100 0
120 0
140 0
160 0
180 0
200 0
220 0
240 3
260 0
280 0
300 0
320 0
340 0
360 0
U34-MY:VAL-CA
20 0
40 0
60 0
80 0
100 0
120 0
140 0
160 0
180 0
200 0
220 0
240 0
260 0
280 0
300 0
320 0
340 3
360 0
G-R6:THR-S1
20 0
40 4
60 12
80 20
100 32
120 24
140 12
160 4
180 0
200 0
220 12
240 36
260 12
280 20
300 28
320 16

340 4
360 0
C31-RIB:ASP-S1
20 0
40 0
60 0
80 0
100 0
120 0
140 0
160 0
180 0
200 0
220 0
240 0
260 0
280 0
300 0
320 3
340 0
360 0
C31-P:MET-S1
20 0
40 0
60 0
80 0
100 0
120 0
140 0
160 0
180 0
200 0
220 0
240 0
260 0
280 0
300 0
320 3
340 0
360 0
H2U-P:ARG-S2
20 0
40 0
60 0
80 3
100 0
120 0
140 0
160 0
180 0
200 0
220 0
240 0
260 0
280 0
300 0
320 0
340 0

360 0
G-R5:MET-S2
20 12
40 8
60 12
80 20
100 0
120 4
140 4
160 4
180 0
200 4
220 4
240 8
260 24
280 24
300 0
320 8
340 4
360 0
DA-M6:TYR-S2
20 0
40 0
60 0
80 0
100 0
120 3
140 0
160 0
180 0
200 0
220 0
240 0
260 0
280 0
300 0
320 0
340 0
360 0
QUO-RIB:PHE-S1
20 0
40 0
60 0
80 0
100 0
120 0
140 0
160 0
180 0
200 0
220 0
240 0
260 0
280 0
300 4
320 0
340 0
360 0

U34-P:TYR-S1
20 0
40 0
60 0
80 0
100 0
120 0
140 0
160 0
180 0
200 0
220 0
240 0
260 0
280 0
300 0
320 3
340 0
360 0
FMU-RIB:GLU-S2
20 0
40 0
60 0
80 0
100 0
120 0
140 0
160 0
180 0
200 0
220 0
240 0
260 0
280 0
300 0
320 0
340 3
360 0
QUO-M5:ASN-S1
20 0
40 0
60 0
80 4
100 0
120 0
140 0
160 0
180 0
200 0
220 0
240 0
260 0
280 0
300 0
320 0
340 0
360 0
G-P:CYS-S1

20 0
40 0
60 0
80 4
100 0
120 4
140 4
160 0
180 0
200 0
220 0
240 0
260 0
280 0
300 0
320 4
340 0
360 4
A-R5:PRO-CA
20 4
40 8
60 16
80 20
100 16
120 20
140 20
160 0
180 0
200 4
220 12
240 16
260 12
280 24
300 32
320 8
340 12
360 0
H2U-RIB:PHE-S1
20 0
40 0
60 0
80 0
100 0
120 0
140 0
160 0
180 0
200 0
220 0
240 0
260 0
280 3
300 0
320 0
340 0
360 0
C31-RIB:GLU-S2
20 0

40 0
60 3
80 0
100 0
120 3
140 0
160 0
180 0
200 0
220 0
240 0
260 0
280 0
300 0
320 0
340 0
360 0
U-RIB:GLU-CA
20 3
40 6
60 6
80 9
100 15
120 9
140 6
160 3
180 0
200 0
220 3
240 3
260 12
280 6
300 6
320 12
340 12
360 3
G-R6:TYR-S2
20 0
40 0
60 16
80 20
100 15
120 24
140 7
160 4
180 0
200 0
220 4
240 16
260 8
280 16
300 19
320 19
340 8
360 4
A-P:ALA-CA
20 0
40 8

60   24
80   36
100   44
120   28
140   36
160   20
180   16
200   0
220   0
240   16
260   32
280   24
300   40
320   44
340   32
360   8
QUO-M6:GLU-S2
20   0
40   0
60   0
80   0
100   0
120   0
140   0
160   4
180   0
200   0
220   0
240   0
260   0
280   0
300   0
320   0
340   0
360   0
GTP-M6:SER-S1
20   0
40   0
60   3
80   0
100   0
120   0
140   0
160   0
180   0
200   0
220   0
240   0
260   0
280   3
300   0
320   0
340   0
360   0
H2U-P:THR-S1
20   0
40   0
60   0

80 0
100 0
120 0
140 0
160 0
180 0
200 0
220 0
240 0
260 0
280 3
300 3
320 0
340 0
360 0
FHU-MY:TYR-CA
20 0
40 0
60 0
80 0
100 3
120 0
140 3
160 0
180 0
200 0
220 0
240 0
260 0
280 3
300 0
320 3
340 0
360 0
FMU-RIB:CYS-S1
20 0
40 0
60 0
80 0
100 0
120 0
140 0
160 3
180 0
200 0
220 0
240 0
260 0
280 0
300 0
320 0
340 0
360 0
U-P:HIS-CA
20 0
40 9
60 9
80 6

100 6
120 12
140 6
160 12
180 0
200 0
220 0
240 12
260 18
280 18
300 9
320 6
340 15
360 0
C31-P:TYR-S2
20 0
40 0
60 0
80 3
100 0
120 0
140 0
160 0
180 0
200 0
220 0
240 0
260 3
280 0
300 0
320 0
340 0
360 0
FHU-P:ALA-CA
20 0
40 0
60 0
80 0
100 0
120 0
140 0
160 0
180 0
200 0
220 0
240 3
260 0
280 0
300 0
320 0
340 0
360 0
FHU-MY:LEU-S2
20 0
40 0
60 0
80 3
100 0

120   0
140   0
160   3
180   0
200   0
220   0
240   0
260   0
280   3
300   0
320   0
340   3
360   0
C-P:GLU-S1
20   0
40   9
60   15
80   18
100   24
120   27
140   15
160   9
180   3
200   0
220   12
240   3
260   15
280   39
300   24
320   24
340   18
360   6
U31-RIB:GLU-S2
20   0
40   0
60   0
80   0
100   0
120   0
140   0
160   0
180   0
200   0
220   0
240   0
260   0
280   0
300   0
320   0
340   0
360   3
G-RIB:GLN-S1
20   4
40   8
60   20
80   36
100   24
120   28

140 28
160 40
180 24
200 0
220 12
240 12
260 24
280 16
300 28
320 20
340 16
360 32
FHU-P:ALA-S1
20 0
40 0
60 0
80 0
100 0
120 0
140 0
160 0
180 0
200 0
220 0
240 3
260 0
280 0
300 0
320 0
340 0
360 0
G-R6:LEU-CA
20 0
40 16
60 4
80 16
100 12
120 4
140 12
160 16
180 0
200 0
220 8
240 12
260 44
280 20
300 16
320 20
340 0
360 0
FHU-RIB:GLY-CA
20 0
40 6
60 3
80 0
100 0
120 0
140 0

160 0
180 0
200 0
220 0
240 3
260 0
280 0
300 0
320 0
340 0
360 0
C31-RIB:PHE-CA
20 0
40 0
60 0
80 0
100 3
120 3
140 0
160 0
180 0
200 0
220 0
240 0
260 0
280 0
300 0
320 0
340 0
360 0
FHU-RIB:THR-CA
20 0
40 0
60 0
80 3
100 0
120 0
140 0
160 0
180 0
200 0
220 0
240 0
260 3
280 3
300 0
320 0
340 0
360 0
FHU-RIB:LYS-CA
20 0
40 0
60 0
80 0
100 0
120 0
140 0
160 0

180   0
200   0
220   3
240   0
260   0
280   0
300   0
320   0
340   0
360   0
C31-P:GLU-CA
20   0
40   0
60   0
80   3
100   0
120   0
140   0
160   0
180   0
200   0
220   0
240   0
260   0
280   0
300   0
320   0
340   0
360   0
C-Y:CYS-CA
20   0
40   0
60   3
80   0
100   0
120   3
140   0
160   0
180   0
200   0
220   0
240   0
260   0
280   0
300   3
320   0
340   0
360   0
U-Y:TRP-CA
20   0
40   3
60   0
80   0
100   9
120   0
140   0
160   0
180   0

200 0
220 3
240 0
260 3
280 0
300 3
320 0
340 3
360 0
G-R5:VAL-S1
20 0
40 8
60 4
80 16
100 12
120 12
140 4
160 0
180 8
200 8
220 16
240 16
260 10
280 12
300 12
320 12
340 0
360 0
FHU-RIB:PRO-S1
20 0
40 0
60 0
80 0
100 0
120 0
140 0
160 3
180 0
200 0
220 0
240 0
260 0
280 0
300 0
320 0
340 0
360 0
DA-M5:SER-S1
20 0
40 0
60 0
80 0
100 0
120 0
140 0
160 0
180 0
200 0

220 0
240 0
260 0
280 3
300 0
320 0
340 0
360 3
DA-M6:GLN-S1
20 0
40 0
60 0
80 0
100 0
120 0
140 0
160 0
180 0
200 0
220 0
240 0
260 3
280 0
300 0
320 0
340 0
360 0
IU-P:ILE-CA
20 0
40 0
60 0
80 3
100 0
120 0
140 0
160 0
180 0
200 0
220 0
240 0
260 0
280 0
300 0
320 0
340 0
360 0
QUO-M6:LYS-S1
20 0
40 0
60 0
80 4
100 0
120 0
140 0
160 0
180 0
200 0
220 0

240   0
260   0
280   0
300   0
320   0
340   0
360   0
H2U-MY:TRP-S1
20   0
40   0
60   0
80   0
100   0
120   0
140   0
160   0
180   0
200   0
220   0
240   0
260   0
280   0
300   0
320   3
340   0
360   0
C31-MY:THR-S1
20   0
40   0
60   0
80   0
100   0
120   0
140   0
160   0
180   0
200   0
220   3
240   0
260   0
280   0
300   0
320   0
340   0
360   0
QUO-M6:PHE-S1
20   0
40   0
60   0
80   0
100   0
120   0
140   0
160   0
180   0
200   0
220   0
240   0

260   4
280   0
300   0
320   4
340   0
360   0
U34-RIB:ASN-S2
20   0
40   0
60   3
80   3
100   0
120   0
140   0
160   0
180   0
200   0
220   0
240   0
260   0
280   0
300   3
320   0
340   0
360   0
C31-MY:GLU-CA
20   0
40   0
60   0
80   0
100   0
120   3
140   0
160   0
180   0
200   0
220   0
240   0
260   0
280   0
300   0
320   0
340   0
360   0
FHU-P:THR-S1
20   0
40   0
60   0
80   3
100   0
120   0
140   0
160   0
180   0
200   0
220   0
240   3
260   0

280 0
300 0
320 3
340 0
360 0
H2U-RIB:ASN-S1
20 0
40 0
60 0
80 0
100 0
120 0
140 0
160 0
180 0
200 0
220 0
240 0
260 3
280 0
300 3
320 0
340 0
360 0
U-RIB:CYS-CA
20 0
40 0
60 0
80 0
100 0
120 0
140 6
160 3
180 3
200 0
220 3
240 0
260 6
280 0
300 0
320 0
340 0
360 0
FMU-RIB:ASN-S1
20 0
40 0
60 0
80 0
100 0
120 0
140 3
160 0
180 0
200 0
220 0
240 0
260 0
280 0

300   0
320   0
340   0
360   0
QUO-M5:PHE-S1
20   0
40   0
60   0
80   0
100   0
120   0
140   0
160   0
180   0
200   0
220   0
240   0
260   4
280   0
300   0
320   4
340   0
360   0
C31-P:ASP-CA
20   0
40   0
60   0
80   0
100   0
120   3
140   3
160   0
180   0
200   0
220   0
240   0
260   0
280   0
300   0
320   3
340   0
360   0
U31-MY:PHE-S2
20   0
40   0
60   0
80   0
100   0
120   0
140   0
160   0
180   0
200   0
220   0
240   0
260   0
280   3
300   0

320 0
340 0
360 0
H2U-P:PHE-S2
20 0
40 0
60 0
80 3
100 0
120 0
140 0
160 0
180 0
200 0
220 0
240 0
260 0
280 0
300 0
320 0
340 0
360 0
IU-P:LYS-CA
20 0
40 0
60 0
80 0
100 3
120 0
140 0
160 0
180 0
200 0
220 3
240 0
260 0
280 0
300 0
320 0
340 0
360 0
H2U-MY:LEU-CA
20 0
40 0
60 0
80 0
100 0
120 0
140 0
160 3
180 0
200 0
220 0
240 0
260 0
280 0
300 0
320 3

340 0
360 0
U34-P:ASN-S1
20 0
40 0
60 3
80 0
100 3
120 0
140 0
160 0
180 0
200 0
220 0
240 0
260 3
280 3
300 0
320 0
340 0
360 0
5BU-P:PRO-CA
20 0
40 0
60 0
80 0
100 0
120 0
140 0
160 0
180 0
200 0
220 0
240 0
260 0
280 0
300 3
320 0
340 0
360 0
FMU-MY:VAL-S1
20 0
40 0
60 0
80 0
100 0
120 0
140 0
160 0
180 0
200 0
220 0
240 3
260 0
280 0
300 0
320 0
340 0

360 3
U34-P:HIS-S1
20 0
40 0
60 0
80 0
100 0
120 0
140 0
160 3
180 0
200 0
220 0
240 0
260 0
280 0
300 0
320 0
340 0
360 0
C31-MY:ALA-CA
20 0
40 0
60 0
80 0
100 0
120 0
140 0
160 0
180 0
200 0
220 0
240 0
260 0
280 3
300 0
320 0
340 0
360 0
FMU-P:ILE-CA
20 0
40 0
60 0
80 0
100 3
120 0
140 0
160 0
180 0
200 0
220 0
240 0
260 0
280 0
300 0
320 0
340 0
360 0

FMU-RIB:VAL-S1
20 0
40 0
60 0
80 0
100 0
120 0
140 0
160 0
180 0
200 0
220 0
240 0
260 0
280 3
300 0
320 0
340 0
360 0
FHU-MY:LYS-S1
20 0
40 0
60 0
80 3
100 0
120 0
140 0
160 0
180 0
200 0
220 0
240 0
260 3
280 0
300 0
320 0
340 0
360 0
QUO-RIB:PHE-S2
20 0
40 0
60 0
80 0
100 0
120 0
140 0
160 0
180 0
200 0
220 0
240 0
260 0
280 0
300 8
320 0
340 0
360 0
U31-P:LEU-S1

20 0
40 0
60 0
80 0
100 0
120 0
140 0
160 0
180 0
200 0
220 0
240 0
260 0
280 3
300 0
320 0
340 0
360 0
FMU-MY:GLN-S2
20 0
40 0
60 0
80 0
100 0
120 0
140 0
160 0
180 0
200 0
220 0
240 0
260 0
280 0
300 3
320 0
340 0
360 0
DA-M5:GLN-CA
20 0
40 0
60 0
80 0
100 0
120 0
140 0
160 0
180 0
200 0
220 0
240 0
260 3
280 0
300 0
320 0
340 0
360 0
IU-MY:PRO-CA
20 0

40 0
60 0
80 3
100 0
120 0
140 0
160 0
180 0
200 0
220 0
240 0
260 0
280 2
300 0
320 0
340 0
360 0
H2U-MY:GLN-S1
20 0
40 0
60 0
80 0
100 0
120 0
140 0
160 0
180 0
200 0
220 0
240 0
260 0
280 0
300 0
320 0
340 0
360 3
G-RIB:CYS-CA
20 0
40 0
60 0
80 0
100 0
120 8
140 0
160 0
180 4
200 0
220 0
240 0
260 0
280 12
300 4
320 0
340 0
360 0
QUO-M5:ASP-CA
20 0
40 0

60 0
80 0
100 0
120 4
140 0
160 0
180 0
200 0
220 0
240 0
260 0
280 0
300 0
320 4
340 0
360 0
DA-M5:LYS-S1
20 0
40 0
60 0
80 0
100 0
120 0
140 0
160 0
180 0
200 0
220 0
240 0
260 0
280 3
300 0
320 0
340 0
360 0
DA-M5:MET-S2
20 0
40 0
60 0
80 0
100 0
120 0
140 0
160 0
180 0
200 0
220 3
240 0
260 0
280 0
300 0
320 0
340 0
360 0
U31-MY:ALA-CA
20 0
40 0
60 0

80 0
100 0
120 0
140 0
160 0
180 0
200 0
220 0
240 0
260 0
280 3
300 0
320 0
340 0
360 0
FHU-RIB:ARG-S2
20 0
40 0
60 3
80 0
100 0
120 0
140 0
160 0
180 3
200 0
220 0
240 3
260 3
280 0
300 0
320 0
340 0
360 3
FHU-P:SER-CA
20 0
40 0
60 0
80 0
100 0
120 3
140 0
160 3
180 0
200 0
220 0
240 0
260 0
280 0
300 0
320 0
340 0
360 0
FMU-MY:VAL-CA
20 0
40 0
60 0
80 0

100   0
120   0
140   0
160   0
180   3
200   0
220   0
240   0
260   3
280   0
300   0
320   0
340   0
360   0
C31-RIB:SER-S1
20   0
40   0
60   0
80   0
100   0
120   0
140   0
160   0
180   0
200   0
220   0
240   0
260   0
280   0
300   0
320   3
340   0
360   0
QUO-M6:ASP-S2
20   0
40   0
60   0
80   0
100   0
120   4
140   0
160   0
180   0
200   0
220   0
240   0
260   0
280   0
300   4
320   0
340   0
360   0
U34-MY:PHE-CA
20   0
40   0
60   0
80   0
100   0

120   3
140   0
160   0
180   0
200   0
220   0
240   0
260   0
280   0
300   0
320   0
340   0
360   0
U31-P:ASP-CA
20   0
40   0
60   0
80   0
100   0
120   0
140   0
160   0
180   0
200   0
220   0
240   0
260   3
280   0
300   3
320   0
340   0
360   0
G-R5:PHE-S2
20   0
40   8
60   4
80   8
100   8
120   0
140   8
160   0
180   0
200   0
220   16
240   8
260   23
280   4
300   4
320   0
340   0
360   0
U31-P:SER-CA
20   0
40   0
60   0
80   0
100   0
120   0

140 0
160 0
180 0
200 0
220 0
240 0
260 0
280 0
300 3
320 0
340 0
360 0
H2U-MY:GLU-S1
20 0
40 0
60 0
80 0
100 0
120 0
140 3
160 0
180 0
200 0
220 0
240 0
260 0
280 0
300 0
320 0
340 0
360 0
U34-P:ARG-S2
20 0
40 0
60 0
80 0
100 0
120 0
140 0
160 0
180 0
200 0
220 0
240 0
260 0
280 0
300 3
320 0
340 0
360 0
A-R6:SER-CA
20 4
40 8
60 12
80 12
100 8
120 24
140 32

160 12
180 0
200 0
220 12
240 20
260 20
280 16
300 20
320 16
340 20
360 8
C31-P:THR-S1
20 0
40 0
60 3
80 0
100 3
120 0
140 0
160 0
180 0
200 0
220 0
240 0
260 0
280 0
300 0
320 0
340 0
360 0
H2U-MY:GLU-CA
20 0
40 0
60 0
80 0
100 0
120 0
140 0
160 3
180 0
200 0
220 0
240 0
260 0
280 0
300 0
320 0
340 0
360 0
C31-P:PHE-CA
20 0
40 0
60 0
80 0
100 0
120 0
140 0
160 0

180 0
200 0
220 0
240 3
260 0
280 0
300 0
320 0
340 0
360 0
A-P:GLN-S2
20 0
40 20
60 16
80 56
100 20
120 20
140 28
160 20
180 12
200 0
220 8
240 12
260 36
280 40
300 8
320 32
340 16
360 8
U31-P:ASN-S1
20 0
40 0
60 0
80 0
100 3
120 0
140 0
160 0
180 0
200 0
220 0
240 0
260 0
280 0
300 0
320 0
340 0
360 0
IU-RIB:HIS-S1
20 0
40 0
60 0
80 0
100 0
120 0
140 0
160 0
180 0

200 0
220 0
240 3
260 0
280 0
300 0
320 0
340 0
360 0
FMU-MY:MET-CA
20 0
40 0
60 0
80 0
100 0
120 0
140 0
160 0
180 0
200 0
220 0
240 0
260 0
280 3
300 0
320 0
340 0
360 0
U31-MY:GLU-S2
20 0
40 0
60 0
80 0
100 0
120 0
140 0
160 3
180 0
200 0
220 0
240 0
260 3
280 0
300 0
320 0
340 0
360 0
C-Y:TRP-CA
20 0
40 0
60 3
80 2
100 8
120 0
140 3
160 0
180 0
200 0

220 0
240 3
260 0
280 3
300 0
320 3
340 3
360 0
QUO-M5:PHE-S2
20 0
40 0
60 0
80 0
100 0
120 0
140 0
160 0
180 0
200 0
220 0
240 0
260 4
280 0
300 0
320 4
340 0
360 0
FMU-RIB:MET-S2
20 0
40 0
60 0
80 0
100 0
120 0
140 0
160 0
180 0
200 0
220 0
240 0
260 0
280 0
300 0
320 3
340 0
360 0
FHU-MY:ASP-S1
20 0
40 0
60 0
80 0
100 3
120 0
140 3
160 0
180 0
200 0
220 0

240 0
260 0
280 3
300 0
320 3
340 0
360 0
G-P:VAL-S1
20 0
40 4
60 8
80 4
100 28
120 36
140 52
160 16
180 4
200 0
220 12
240 12
260 16
280 20
300 12
320 24
340 20
360 12
I-RIB:TRP-S1
20 0
40 0
60 0
80 0
100 0
120 0
140 2
160 0
180 0
200 0
220 0
240 0
260 0
280 0
300 0
320 0
340 0
360 0
I-RIB:ALA-CA
20 0
40 0
60 0
80 0
100 0
120 0
140 0
160 2
180 0
200 0
220 0
240 0

260 0
280 0
300 0
320 0
340 0
360 0
H2U-RIB:ARG-S1
20 0
40 0
60 0
80 0
100 0
120 0
140 0
160 0
180 0
200 0
220 0
240 0
260 3
280 0
300 0
320 0
340 0
360 0
5BU-P:ARG-CA
20 0
40 0
60 0
80 0
100 0
120 3
140 3
160 0
180 0
200 0
220 0
240 0
260 0
280 0
300 0
320 0
340 0
360 0
U34-MY:ASP-CA
20 0
40 0
60 0
80 0
100 3
120 0
140 0
160 0
180 0
200 0
220 0
240 0
260 0

280 0
300 0
320 0
340 0
360 0
U34-MY:SER-S1
20 0
40 0
60 0
80 0
100 0
120 0
140 0
160 0
180 0
200 0
220 0
240 0
260 0
280 0
300 3
320 0
340 0
360 3
A-RIB:MET-CA
20 0
40 4
60 4
80 20
100 4
120 16
140 8
160 4
180 0
200 0
220 8
240 4
260 12
280 16
300 12
320 4
340 12
360 0
4SU-P:GLU-S2
20 0
40 0
60 0
80 0
100 0
120 0
140 0
160 0
180 0
200 0
220 0
240 0
260 0
280 0

300 0
320 0
340 3
360 0
FMU-MY:PHE-S1
20 0
40 0
60 0
80 0
100 0
120 0
140 0
160 0
180 0
200 0
220 0
240 0
260 3
280 0
300 0
320 0
340 0
360 0
A-R5:CYS-CA
20 0
40 0
60 4
80 4
100 4
120 0
140 0
160 0
180 0
200 0
220 0
240 0
260 0
280 8
300 0
320 0
340 0
360 0
C31-P:PHE-S1
20 0
40 0
60 0
80 0
100 0
120 0
140 0
160 0
180 0
200 0
220 0
240 3
260 0
280 0
300 0

320   0
340   0
360   0
U34-RIB:SER-S1
20   0
40   0
60   0
80   3
100   0
120   0
140   0
160   0
180   0
200   0
220   0
240   0
260   0
280   3
300   0
320   0
340   0
360   0
C31-P:ALA-S1
20   0
40   0
60   0
80   0
100   0
120   0
140   0
160   0
180   0
200   0
220   0
240   0
260   0
280   3
300   0
320   0
340   0
360   0
M2G-P:GLY-CA
20   0
40   0
60   0
80   0
100   0
120   0
140   0
160   4
180   0
200   0
220   0
240   0
260   0
280   0
300   0
320   0

340 0
360 0
I-RIB:GLY-CA
20 0
40 0
60 0
80 0
100 0
120 0
140 0
160 2
180 0
200 0
220 0
240 0
260 0
280 0
300 0
320 0
340 0
360 0
IU-RIB:LYS-S2
20 0
40 0
60 0
80 0
100 0
120 0
140 0
160 3
180 0
200 0
220 3
240 0
260 0
280 0
300 0
320 0
340 0
360 0
DA-M5:ASN-S1
20 0
40 0
60 0
80 0
100 0
120 0
140 0
160 0
180 0
200 0
220 0
240 3
260 0
280 0
300 0
320 0
340 0

360 0
C-Y:TYR-CA
20 0
40 0
60 3
80 15
100 6
120 6
140 3
160 6
180 0
200 0
220 3
240 6
260 18
280 3
300 12
320 3
340 3
360 0
DA-M6:SER-S1
20 0
40 0
60 0
80 0
100 0
120 0
140 3
160 0
180 0
200 0
220 0
240 0
260 0
280 0
300 0
320 0
340 0
360 0
FHU-MY:GLY-CA
20 0
40 0
60 3
80 0
100 0
120 0
140 0
160 0
180 0
200 0
220 0
240 3
260 0
280 0
300 0
320 0
340 0
360 0

H2U-MY:ASN-S1
20 0
40 0
60 0
80 0
100 0
120 0
140 0
160 3
180 0
200 0
220 0
240 0
260 0
280 6
300 0
320 0
340 0
360 0
H2U-P:TRP-S1
20 0
40 0
60 0
80 0
100 3
120 0
140 0
160 0
180 0
200 0
220 0
240 0
260 0
280 0
300 0
320 0
340 0
360 0
DA-M5:LEU-S2
20 0
40 0
60 0
80 0
100 0
120 3
140 0
160 0
180 0
200 0
220 0
240 0
260 0
280 0
300 0
320 0
340 0
360 0
DA-M5:TYR-S1

20 0
40 0
60 0
80 0
100 3
120 0
140 0
160 0
180 0
200 0
220 0
240 0
260 0
280 0
300 3
320 0
340 0
360 0
H2U-P:GLU-CA
20 0
40 0
60 0
80 3
100 0
120 0
140 0
160 0
180 0
200 0
220 0
240 0
260 0
280 0
300 0
320 0
340 0
360 0
U34-MY:ASN-S2
20 0
40 0
60 0
80 0
100 0
120 0
140 0
160 0
180 0
200 0
220 3
240 0
260 3
280 0
300 0
320 0
340 0
360 0
DA-M5:ASN-S2
20 0

40 0
60 0
80 0
100 0
120 0
140 0
160 0
180 0
200 0
220 0
240 3
260 0
280 0
300 0
320 0
340 0
360 0
IU-RIB:LYS-CA
20 0
40 0
60 0
80 0
100 0
120 3
140 0
160 0
180 0
200 0
220 0
240 3
260 0
280 0
300 0
320 0
340 0
360 0
FMU-RIB:HIS-S1
20 0
40 0
60 0
80 0
100 0
120 0
140 0
160 3
180 0
200 0
220 0
240 0
260 0
280 0
300 0
320 0
340 0
360 0
H2U-P:LEU-S1
20 0
40 0

60 0
80 0
100 3
120 0
140 0
160 0
180 0
200 0
220 0
240 0
260 0
280 0
300 0
320 0
340 0
360 0
H2U-MY:GLN-CA
20 0
40 0
60 0
80 0
100 0
120 0
140 0
160 0
180 0
200 0
220 0
240 0
260 0
280 0
300 0
320 0
340 0
360 3
H2U-P:ASN-CA
20 0
40 0
60 0
80 0
100 0
120 3
140 3
160 0
180 0
200 0
220 0
240 0
260 0
280 0
300 0
320 0
340 0
360 0
C31-RIB:PHE-S2
20 0
40 0
60 0

80 0
100 3
120 3
140 0
160 0
180 0
200 0
220 0
240 0
260 0
280 0
300 0
320 0
340 0
360 0
GTP-M5:ASP-S2
20 0
40 0
60 0
80 0
100 0
120 0
140 0
160 0
180 0
200 0
220 0
240 0
260 0
280 0
300 3
320 0
340 0
360 0
C31-MY:PHE-CA
20 0
40 0
60 0
80 0
100 3
120 0
140 0
160 0
180 0
200 0
220 0
240 0
260 0
280 0
300 0
320 0
340 0
360 0
FMU-MY:GLU-CA
20 0
40 0
60 0
80 0

100   0
120   0
140   0
160   0
180   0
200   0
220   0
240   0
260   0
280   0
300   3
320   0
340   0
360   0
FHU-RIB:ILE-CA
20   0
40   0
60   0
80   0
100   0
120   0
140   0
160   0
180   0
200   0
220   0
240   0
260   0
280   0
300   3
320   3
340   0
360   0
5BU-P:ARG-S1
20   0
40   0
60   0
80   0
100   0
120   0
140   3
160   0
180   0
200   0
220   0
240   0
260   0
280   0
300   0
320   0
340   0
360   0
FMU-RIB:HIS-S2
20   0
40   0
60   0
80   0
100   0

120   0
140   3
160   0
180   0
200   0
220   0
240   0
260   0
280   0
300   0
320   0
340   0
360   0
A-RIB:CYS-CA
20   0
40   0
60   0
80   12
100   4
120   12
140   4
160   0
180   0
200   0
220   0
240   4
260   0
280   4
300   0
320   8
340   0
360   0
C31-RIB:LEU-S2
20   0
40   0
60   0
80   0
100   0
120   0
140   0
160   0
180   0
200   0
220   0
240   0
260   0
280   0
300   3
320   0
340   0
360   0
U31-MY:VAL-CA
20   0
40   0
60   0
80   0
100   0
120   3

140 0
160 0
180 0
200 0
220 0
240 0
260 0
280 0
300 0
320 0
340 0
360 0
U-Y:VAL-CA
20 0
40 6
60 3
80 6
100 12
120 0
140 6
160 3
180 0
200 0
220 0
240 12
260 9
280 15
300 3
320 9
340 6
360 0
I-RIB:TRP-S2
20 0
40 0
60 0
80 0
100 0
120 2
140 0
160 0
180 0
200 0
220 0
240 0
260 0
280 0
300 0
320 0
340 0
360 0
FHU-P:VAL-CA
20 0
40 0
60 0
80 0
100 0
120 3
140 0

160 0
180 0
200 0
220 0
240 0
260 0
280 0
300 0
320 3
340 0
360 0
C31-P:TYR-CA
20 0
40 0
60 0
80 0
100 3
120 0
140 0
160 0
180 0
200 0
220 0
240 0
260 0
280 3
300 0
320 0
340 0
360 0
U-Y:GLN-CA
20 0
40 3
60 15
80 3
100 0
120 3
140 6
160 8
180 0
200 6
220 0
240 6
260 6
280 6
300 6
320 12
340 0
360 0
G-RIB:CYS-S1
20 0
40 0
60 0
80 0
100 0
120 4
140 4
160 0

180 4
200 0
220 0
240 0
260 4
280 0
300 4
320 4
340 0
360 0
C-Y:MET-S1
20 0
40 9
60 6
80 9
100 9
120 0
140 0
160 0
180 0
200 3
220 6
240 6
260 3
280 9
300 3
320 0
340 0
360 0
IU-P:LEU-CA
20 0
40 0
60 0
80 0
100 0
120 0
140 3
160 0
180 0
200 0
220 0
240 0
260 0
280 0
300 3
320 0
340 0
360 0
C31-MY:LEU-CA
20 0
40 0
60 0
80 0
100 0
120 0
140 0
160 0
180 0

200 0
220 0
240 0
260 3
280 0
300 0
320 0
340 0
360 0
M2G-P:SER-S1
20 0
40 0
60 0
80 0
100 0
120 0
140 0
160 0
180 0
200 0
220 0
240 0
260 0
280 0
300 0
320 4
340 0
360 0
H2U-MY:PRO-S1
20 0
40 0
60 0
80 0
100 6
120 0
140 0
160 0
180 0
200 0
220 0
240 0
260 0
280 0
300 0
320 0
340 0
360 0
U31-P:GLN-S2
20 0
40 0
60 0
80 0
100 0
120 0
140 0
160 0
180 0
200 0

220   0
240   0
260   0
280   3
300   0
320   0
340   0
360   0
FMU-MY:GLU-S2
20   0
40   0
60   0
80   0
100   0
120   0
140   0
160   0
180   0
200   0
220   0
240   0
260   0
280   0
300   0
320   3
340   0
360   0
U31-RIB:MET-CA
20   0
40   0
60   3
80   0
100   0
120   0
140   0
160   0
180   0
200   0
220   0
240   0
260   0
280   0
300   0
320   0
340   0
360   0
FMU-MY:ARG-S2
20   0
40   3
60   0
80   0
100   0
120   0
140   0
160   0
180   0
200   0
220   0

240 0
260 0
280 0
300 0
320 0
340 0
360 0
C31-P:ASN-S2
20 0
40 0
60 0
80 0
100 0
120 3
140 0
160 0
180 0
200 0
220 0
240 0
260 0
280 0
300 0
320 0
340 0
360 0
FMU-MY:MET-S1
20 0
40 0
60 0
80 0
100 0
120 0
140 0
160 0
180 0
200 0
220 0
240 0
260 0
280 3
300 0
320 0
340 0
360 0
U-Y:PHE-S1
20 0
40 9
60 6
80 3
100 0
120 6
140 0
160 0
180 0
200 3
220 9
240 3

260 9
280 18
300 14
320 3
340 0
360 0
QUO-M6:ASP-S1
20 0
40 0
60 0
80 0
100 0
120 4
140 0
160 0
180 0
200 0
220 0
240 0
260 0
280 0
300 0
320 0
340 0
360 0
C31-P:GLN-S1
20 0
40 0
60 0
80 0
100 0
120 0
140 0
160 3
180 0
200 0
220 0
240 0
260 0
280 0
300 0
320 0
340 0
360 0
H2U-MY:PHE-S2
20 0
40 0
60 0
80 0
100 3
120 0
140 0
160 0
180 0
200 0
220 0
240 0
260 0

280 0
300 0
320 0
340 0
360 0
U34-P:GLU-S2
20 0
40 0
60 0
80 0
100 0
120 0
140 0
160 0
180 0
200 0
220 0
240 0
260 0
280 0
300 0
320 0
340 3
360 0
FHU-RIB:ARG-CA
20 0
40 0
60 0
80 0
100 0
120 0
140 0
160 0
180 0
200 0
220 0
240 0
260 0
280 0
300 0
320 3
340 3
360 0
FHU-P:ARG-S1
20 0
40 0
60 0
80 3
100 0
120 0
140 0
160 0
180 0
200 0
220 0
240 0
260 0
280 3

300 0
320 0
340 0
360 0
FHU-P:ASP-CA
20 0
40 0
60 0
80 0
100 0
120 0
140 3
160 6
180 0
200 0
220 0
240 0
260 0
280 0
300 0
320 0
340 0
360 0
U31-P:ASP-S2
20 0
40 0
60 0
80 0
100 3
120 0
140 0
160 0
180 0
200 0
220 0
240 0
260 3
280 0
300 3
320 0
340 0
360 0
C31-P:ALA-CA
20 0
40 0
60 0
80 0
100 0
120 0
140 0
160 0
180 0
200 0
220 0
240 0
260 0
280 0
300 3

320 0
340 0
360 0
U31-MY:ALA-S1
20 0
40 0
60 0
80 0
100 0
120 3
140 0
160 0
180 0
200 0
220 0
240 0
260 0
280 3
300 0
320 0
340 0
360 0
FMU-RIB:PHE-S2
20 0
40 0
60 0
80 0
100 0
120 0
140 0
160 0
180 0
200 0
220 0
240 0
260 3
280 0
300 0
320 0
340 3
360 0
A-R5:TRP-CA
20 0
40 4
60 0
80 4
100 4
120 4
140 4
160 0
180 0
200 0
220 4
240 8
260 16
280 4
300 0
320 4

340 0
360 0
GTP-M6:SER-CA
20 0
40 0
60 0
80 0
100 0
120 0
140 0
160 0
180 0
200 0
220 0
240 0
260 3
280 0
300 0
320 0
340 0
360 0
C31-RIB:TYR-S1
20 0
40 0
60 0
80 0
100 0
120 0
140 0
160 0
180 0
200 0
220 0
240 3
260 0
280 0
300 0
320 0
340 0
360 0
5BU-P:SER-S1
20 0
40 0
60 0
80 0
100 0
120 0
140 0
160 0
180 0
200 0
220 0
240 0
260 0
280 3
300 0
320 0
340 0

360 0
U31-RIB:TYR-S2
20 0
40 0
60 0
80 0
100 0
120 0
140 0
160 3
180 0
200 0
220 0
240 0
260 0
280 3
300 0
320 0
340 0
360 0
FMU-MY:ASN-S1
20 0
40 0
60 0
80 0
100 3
120 0
140 0
160 0
180 0
200 0
220 0
240 0
260 0
280 0
300 0
320 0
340 0
360 0
U34-P:PRO-S1
20 0
40 0
60 0
80 0
100 0
120 0
140 0
160 0
180 0
200 0
220 0
240 0
260 0
280 3
300 0
320 0
340 0
360 0

5BU-RIB:SER-S1
20 0
40 0
60 3
80 0
100 0
120 0
140 0
160 0
180 0
200 0
220 0
240 0
260 0
280 0
300 0
320 0
340 0
360 0
U31-P:GLN-S1
20 0
40 0
60 0
80 0
100 0
120 0
140 0
160 0
180 0
200 0
220 0
240 3
260 0
280 0
300 0
320 0
340 0
360 0
FHU-MY:SER-CA
20 0
40 0
60 0
80 0
100 0
120 3
140 0
160 0
180 0
200 0
220 0
240 3
260 0
280 0
300 0
320 0
340 0
360 0
QUO-M5:LEU-S2

20 0
40 0
60 0
80 0
100 0
120 0
140 0
160 0
180 0
200 0
220 0
240 4
260 0
280 4
300 0
320 0
340 0
360 0
I-P:TRP-S2
20 0
40 0
60 0
80 0
100 0
120 0
140 0
160 0
180 0
200 0
220 0
240 2
260 0
280 0
300 0
320 0
340 0
360 0
FMU-P:CYS-S1
20 0
40 0
60 0
80 0
100 0
120 0
140 0
160 0
180 0
200 3
220 0
240 0
260 0
280 0
300 0
320 0
340 0
360 0
IU-P:LYS-S2
20 0

40 0
60 0
80 0
100 0
120 0
140 3
160 0
180 0
200 0
220 0
240 0
260 0
280 0
300 3
320 0
340 0
360 0
GTP-M5:THR-S1
20 0
40 0
60 0
80 0
100 3
120 0
140 0
160 0
180 0
200 0
220 0
240 0
260 0
280 0
300 0
320 0
340 0
360 0
H2U-P:TRP-S2
20 0
40 0
60 0
80 0
100 3
120 0
140 0
160 0
180 0
200 0
220 0
240 0
260 0
280 0
300 0
320 0
340 0
360 0
FMU-MY:SER-S1
20 0
40 0

60 0
80 0
100 0
120 0
140 3
160 0
180 0
200 0
220 0
240 0
260 0
280 0
300 0
320 0
340 0
360 0
U31-RIB:ASN-S1
20 0
40 0
60 0
80 0
100 0
120 0
140 0
160 0
180 0
200 0
220 0
240 3
260 0
280 0
300 0
320 0
340 0
360 0
FHU-RIB:LYS-S1
20 0
40 3
60 0
80 0
100 0
120 0
140 0
160 0
180 0
200 0
220 0
240 3
260 0
280 0
300 0
320 0
340 0
360 0
FHU-RIB:LYS-S2
20 3
40 0
60 0

80 0
100 3
120 0
140 0
160 0
180 0
200 0
220 3
240 0
260 0
280 0
300 0
320 0
340 0
360 0
FHU-P:CYS-S1
20 0
40 0
60 0
80 0
100 3
120 0
140 0
160 0
180 0
200 0
220 0
240 0
260 0
280 0
300 0
320 0
340 0
360 0
FHU-P:ARG-S2
20 0
40 0
60 0
80 3
100 3
120 0
140 0
160 0
180 0
200 0
220 0
240 0
260 0
280 3
300 0
320 0
340 0
360 0
C31-MY:PHE-S1
20 0
40 0
60 0
80 0

100   3
120   0
140   0
160   0
180   0
200   0
220   3
240   0
260   0
280   0
300   0
320   0
340   0
360   0
GTP-M6:THR-S1
20   0
40   0
60   0
80   3
100   0
120   0
140   0
160   0
180   0
200   0
220   0
240   0
260   0
280   0
300   0
320   0
340   0
360   0
C31-MY:ALA-S1
20   0
40   0
60   0
80   0
100   0
120   0
140   0
160   0
180   0
200   0
220   0
240   0
260   0
280   3
300   0
320   0
340   0
360   0
5BU-P:ILE-S1
20   0
40   0
60   0
80   0
100   0

120 3
140 0
160 0
180 0
200 0
220 0
240 0
260 0
280 0
300 0
320 0
340 3
360 0
FMU-RIB:GLN-S1
20 0
40 0
60 0
80 0
100 0
120 0
140 0
160 0
180 0
200 0
220 0
240 0
260 0
280 3
300 0
320 0
340 0
360 0
IU-P:ILE-S1
20 0
40 0
60 0
80 0
100 0
120 0
140 0
160 0
180 0
200 0
220 0
240 0
260 3
280 0
300 0
320 0
340 0
360 0
IU-P:ALA-CA
20 0
40 0
60 3
80 0
100 0
120 0

140   0
160   0
180   0
200   0
220   0
240   0
260   0
280   0
300   0
320   0
340   0
360   0
C31-P:MET-CA
20   0
40   0
60   0
80   0
100   0
120   0
140   0
160   0
180   0
200   0
220   0
240   0
260   0
280   0
300   0
320   3
340   0
360   0
FHU-RIB:LEU-CA
20   0
40   0
60   0
80   0
100   3
120   0
140   0
160   0
180   0
200   0
220   0
240   0
260   0
280   3
300   0
320   0
340   0
360   0
C31-MY:PHE-S2
20   0
40   0
60   0
80   0
100   0
120   0
140   0

160 0
180 0
200 0
220 0
240 0
260 3
280 0
300 0
320 0
340 0
360 0
FMU-MY:MET-S2
20 0
40 0
60 0
80 0
100 0
120 0
140 0
160 0
180 0
200 0
220 0
240 0
260 0
280 0
300 3
320 3
340 0
360 0
C31-RIB:GLU-CA
20 0
40 0
60 0
80 0
100 0
120 0
140 0
160 0
180 0
200 0
220 0
240 0
260 3
280 0
300 0
320 0
340 0
360 0
U31-RIB:GLU-CA
20 0
40 0
60 0
80 0
100 0
120 0
140 0
160 0

180 0
200 0
220 0
240 0
260 0
280 0
300 0
320 0
340 3
360 0
U34-P:GLU-S1
20 0
40 0
60 0
80 0
100 0
120 0
140 0
160 0
180 0
200 0
220 0
240 0
260 0
280 0
300 0
320 0
340 0
360 3
IU-RIB:HIS-S2
20 0
40 0
60 0
80 0
100 0
120 0
140 0
160 0
180 0
200 0
220 3
240 0
260 0
280 0
300 0
320 0
340 0
360 0
FHU-RIB:ASP-S1
20 0
40 0
60 0
80 0
100 0
120 0
140 0
160 3
180 0

200 0
220 0
240 0
260 0
280 0
300 0
320 3
340 0
360 0
U31-P:ALA-S1
20 0
40 0
60 0
80 0
100 0
120 0
140 0
160 0
180 0
200 0
220 0
240 0
260 0
280 0
300 0
320 0
340 3
360 0
U31-P:GLN-CA
20 0
40 0
60 0
80 0
100 0
120 0
140 0
160 0
180 0
200 0
220 0
240 0
260 3
280 0
300 0
320 0
340 0
360 0
5BU-P:PRO-S1
20 0
40 0
60 0
80 0
100 0
120 0
140 0
160 0
180 0
200 0

220 0
240 0
260 0
280 3
300 0
320 0
340 0
360 0
H2U-MY:THR-CA
20 0
40 0
60 0
80 0
100 0
120 3
140 0
160 0
180 0
200 0
220 0
240 0
260 0
280 0
300 3
320 0
340 0
360 0
QUO-M6:ASN-CA
20 0
40 0
60 0
80 4
100 0
120 0
140 0
160 0
180 0
200 0
220 0
240 0
260 0
280 0
300 0
320 0
340 0
360 0
IU-MY:ALA-S1
20 0
40 0
60 0
80 0
100 3
120 2
140 0
160 0
180 0
200 0
220 0

240   0
260   0
280   0
300   0
320   0
340   0
360   0
DA-M5:LEU-S1
20   0
40   0
60   0
80   0
100   0
120   3
140   0
160   0
180   0
200   0
220   0
240   0
260   0
280   0
300   0
320   0
340   0
360   0
H2U-MY:LEU-S2
20   0
40   0
60   0
80   0
100   0
120   0
140   0
160   0
180   0
200   0
220   0
240   0
260   0
280   0
300   3
320   0
340   0
360   0
U-Y:CYS-CA
20   0
40   0
60   3
80   0
100   0
120   0
140   0
160   0
180   0
200   0
220   0
240   0

260 2
280 0
300 6
320 3
340 3
360 0
G-RIB:PHE-S2
20 0
40 0
60 0
80 4
100 4
120 28
140 16
160 4
180 8
200 0
220 0
240 12
260 20
280 32
300 0
320 12
340 20
360 4
DA-M5:TYR-CA
20 0
40 0
60 0
80 0
100 0
120 0
140 0
160 0
180 0
200 0
220 0
240 0
260 0
280 3
300 3
320 0
340 0
360 0
IU-MY:SER-S1
20 0
40 0
60 0
80 2
100 0
120 3
140 0
160 0
180 0
200 0
220 0
240 0
260 0

280 0
300 0
320 0
340 0
360 0
IU-RIB:ARG-S1
20 0
40 0
60 0
80 0
100 0
120 0
140 0
160 0
180 0
200 0
220 0
240 0
260 0
280 3
300 0
320 0
340 0
360 0
U31-MY:TYR-S2
20 0
40 0
60 0
80 0
100 0
120 0
140 0
160 0
180 0
200 0
220 0
240 0
260 0
280 3
300 0
320 0
340 0
360 0
DA-M6:HIS-S2
20 0
40 0
60 0
80 0
100 0
120 0
140 0
160 0
180 0
200 0
220 0
240 0
260 3
280 0

300 0
320 0
340 0
360 0
FMU-MY:GLN-CA
20 0
40 0
60 0
80 0
100 0
120 0
140 0
160 0
180 0
200 0
220 0
240 0
260 0
280 3
300 0
320 0
340 0
360 0
H2U-P:PHE-S1
20 0
40 0
60 0
80 3
100 0
120 0
140 0
160 0
180 0
200 0
220 0
240 0
260 0
280 0
300 0
320 0
340 0
360 0
DA-M6:ASN-S2
20 0
40 0
60 0
80 0
100 0
120 0
140 0
160 0
180 0
200 0
220 0
240 0
260 3
280 0
300 0

320 0
340 0
360 0
DA-M5:SER-CA
20 0
40 0
60 0
80 0
100 0
120 0
140 0
160 0
180 0
200 0
220 0
240 0
260 0
280 0
300 3
320 0
340 3
360 0
FHU-MY:ILE-S1
20 0
40 0
60 0
80 0
100 0
120 0
140 3
160 0
180 0
200 0
220 0
240 0
260 0
280 0
300 0
320 3
340 0
360 0
H2U-RIB:PHE-S2
20 0
40 0
60 0
80 0
100 0
120 0
140 0
160 0
180 0
200 0
220 0
240 0
260 3
280 0
300 0
320 0

340 0
360 0
C31-RIB:PHE-S1
20 0
40 0
60 0
80 0
100 3
120 3
140 0
160 0
180 0
200 0
220 0
240 0
260 0
280 0
300 0
320 0
340 0
360 0
U34-P:HIS-S2
20 0
40 0
60 0
80 0
100 0
120 0
140 0
160 3
180 0
200 0
220 0
240 0
260 0
280 3
300 0
320 0
340 0
360 0
U34-RIB:GLY-CA
20 0
40 0
60 0
80 0
100 0
120 3
140 0
160 0
180 0
200 0
220 0
240 0
260 0
280 0
300 0
320 0
340 0

360 0
OMC-P:LYS-S2
20 0
40 0
60 0
80 3
100 3
120 0
140 0
160 0
180 0
200 0
220 0
240 0
260 0
280 0
300 0
320 0
340 0
360 0
FHU-MY:ARG-CA
20 0
40 0
60 0
80 0
100 0
120 3
140 6
160 0
180 0
200 0
220 0
240 0
260 0
280 0
300 3
320 3
340 0
360 0
H2U-MY:TRP-CA
20 0
40 0
60 0
80 0
100 0
120 0
140 0
160 0
180 0
200 0
220 0
240 0
260 0
280 0
300 0
320 3
340 0
360 0

C31-MY:GLN-S1
20 0
40 0
60 0
80 0
100 0
120 0
140 0
160 0
180 0
200 0
220 0
240 0
260 0
280 0
300 3
320 0
340 0
360 0
DA-M6:GLU-S1
20 0
40 0
60 0
80 0
100 0
120 0
140 0
160 0
180 0
200 0
220 0
240 0
260 0
280 0
300 0
320 0
340 3
360 0
QUO-M6:PHE-CA
20 0
40 0
60 0
80 0
100 0
120 0
140 0
160 0
180 0
200 0
220 0
240 0
260 0
280 4
300 0
320 4
340 0
360 0
U34-MY:ASN-CA

20 0
40 0
60 0
80 0
100 0
120 0
140 0
160 0
180 0
200 0
220 0
240 3
260 0
280 0
300 0
320 0
340 0
360 0
QUO-M5:GLN-S2
20 0
40 0
60 0
80 0
100 4
120 0
140 0
160 0
180 0
200 0
220 0
240 0
260 0
280 0
300 0
320 0
340 0
360 0
5BU-RIB:ARG-S2
20 0
40 0
60 0
80 0
100 0
120 0
140 0
160 0
180 0
200 3
220 0
240 0
260 0
280 0
300 0
320 0
340 0
360 0
5BU-P:ARG-S2
20 0

40 0
60 0
80 0
100 0
120 0
140 0
160 3
180 0
200 0
220 0
240 0
260 0
280 0
300 0
320 0
340 0
360 0
IU-MY:HIS-CA
20 0
40 3
60 0
80 0
100 0
120 0
140 0
160 0
180 0
200 0
220 2
240 0
260 0
280 0
300 0
320 0
340 0
360 0
FHU-MY:ASP-CA
20 0
40 0
60 0
80 0
100 3
120 0
140 0
160 0
180 0
200 0
220 0
240 0
260 0
280 3
300 0
320 0
340 0
360 0
DA-M6:GLN-CA
20 0
40 0

60 0
80 0
100 0
120 0
140 0
160 0
180 0
200 0
220 0
240 0
260 3
280 0
300 0
320 0
340 0
360 0
5BU-RIB:PRO-S1
20 0
40 0
60 3
80 0
100 0
120 0
140 0
160 0
180 0
200 0
220 0
240 0
260 0
280 0
300 0
320 0
340 0
360 0
FMU-RIB:PHE-CA
20 0
40 0
60 0
80 0
100 0
120 0
140 0
160 0
180 0
200 0
220 3
240 0
260 0
280 0
300 0
320 0
340 0
360 0
C31-MY:SER-CA
20 0
40 0
60 0

80 0
100 0
120 0
140 0
160 0
180 0
200 0
220 0
240 3
260 0
280 0
300 0
320 0
340 0
360 0
H2U-MY:ILE-CA
20 0
40 0
60 0
80 0
100 0
120 0
140 0
160 3
180 0
200 0
220 0
240 0
260 0
280 0
300 0
320 0
340 0
360 0
FMU-MY:CYS-CA
20 0
40 0
60 0
80 0
100 0
120 3
140 0
160 0
180 0
200 0
220 0
240 0
260 0
280 0
300 0
320 0
340 0
360 0
H2U-MY:GLU-S2
20 0
40 0
60 0
80 0

100 3
120 0
140 0
160 0
180 0
200 0
220 0
240 0
260 0
280 0
300 0
320 0
340 0
360 0
H2U-MY:THR-S1
20 0
40 0
60 0
80 0
100 0
120 0
140 0
160 0
180 0
200 0
220 0
240 0
260 0
280 0
300 0
320 3
340 0
360 0
U31-MY:VAL-S1
20 0
40 0
60 0
80 0
100 0
120 3
140 0
160 0
180 0
200 0
220 0
240 0
260 0
280 0
300 0
320 0
340 0
360 0
QUO-M6:LYS-CA
20 0
40 0
60 0
80 4
100 0

120 0
140 0
160 0
180 0
200 0
220 0
240 0
260 0
280 0
300 0
320 0
340 0
360 0
DA-M6:LYS-S2
20 0
40 0
60 0
80 0
100 0
120 0
140 0
160 0
180 0
200 0
220 0
240 0
260 0
280 0
300 3
320 0
340 0
360 0
C31-P:GLU-S2
20 0
40 0
60 0
80 0
100 0
120 0
140 0
160 0
180 0
200 0
220 0
240 0
260 3
280 0
300 0
320 0
340 0
360 0
FMU-RIB:GLN-S2
20 0
40 0
60 0
80 0
100 0
120 0

140 0
160 0
180 0
200 0
220 0
240 0
260 0
280 0
300 3
320 0
340 0
360 0
DA-M5:HIS-S1
20 0
40 0
60 0
80 0
100 0
120 0
140 0
160 0
180 0
200 0
220 0
240 0
260 3
280 0
300 0
320 0
340 0
360 0
C31-RIB:ASP-CA
20 0
40 0
60 0
80 0
100 0
120 0
140 0
160 0
180 0
200 0
220 0
240 0
260 0
280 0
300 0
320 3
340 0
360 0
FHU-RIB:ALA-CA
20 0
40 0
60 0
80 3
100 0
120 3
140 0

160   0
180   0
200   0
220   0
240   0
260   0
280   0
300   3
320   0
340   0
360   0
U31-MY:TYR-CA
20   0
40   0
60   0
80   0
100   0
120   0
140   0
160   0
180   0
200   0
220   0
240   0
260   0
280   0
300   0
320   3
340   0
360   0
FHU-MY:PHE-S2
20   0
40   0
60   0
80   0
100   0
120   0
140   0
160   0
180   0
200   0
220   0
240   0
260   0
280   0
300   0
320   3
340   0
360   0
IU-RIB:ALA-CA
20   0
40   0
60   0
80   0
100   0
120   0
140   0
160   0

180   0
200   0
220   0
240   0
260   0
280   3
300   0
320   0
340   0
360   0
C31-P:ASP-S1
20   0
40   0
60   0
80   0
100   0
120   3
140   3
160   0
180   0
200   0
220   0
240   0
260   0
280   0
300   0
320   3
340   0
360   0
H2U-RIB:TRP-S2
20   0
40   0
60   0
80   0
100   0
120   0
140   0
160   0
180   0
200   0
220   0
240   3
260   0
280   0
300   0
320   0
340   0
360   0
QUO-RIB:GLN-S2
20   0
40   0
60   0
80   0
100   0
120   0
140   0
160   0
180   0

200 0
220 0
240 0
260 0
280 0
300 0
320 0
340 4
360 0
M2G-P:GLU-S2
20 0
40 0
60 0
80 0
100 0
120 0
140 0
160 0
180 0
200 0
220 0
240 0
260 0
280 0
300 0
320 0
340 4
360 0
FMU-P:GLN-CA
20 0
40 0
60 3
80 0
100 0
120 0
140 0
160 0
180 0
200 0
220 0
240 0
260 0
280 0
300 0
320 0
340 0
360 0
GTP-M5:SER-CA
20 0
40 0
60 0
80 0
100 3
120 0
140 0
160 0
180 0
200 0

220 0
240 0
260 0
280 0
300 0
320 0
340 0
360 0
IU-MY:THR-CA
20 0
40 0
60 0
80 0
100 0
120 3
140 2
160 0
180 0
200 0
220 0
240 0
260 0
280 0
300 0
320 0
340 0
360 0
H2U-RIB:GLU-CA
20 0
40 0
60 0
80 0
100 0
120 0
140 0
160 0
180 0
200 0
220 0
240 0
260 3
280 0
300 0
320 0
340 0
360 0
OMC-P:LYS-CA
20 0
40 0
60 0
80 0
100 3
120 0
140 0
160 0
180 0
200 0
220 0

240   0
260   0
280   0
300   0
320   0
340   0
360   0
DA-M6:THR-CA
20   0
40   0
60   0
80   0
100   0
120   0
140   0
160   0
180   0
200   0
220   0
240   0
260   0
280   0
300   3
320   0
340   3
360   0
GTP-M6:ASN-CA
20   0
40   3
60   0
80   0
100   0
120   0
140   0
160   0
180   0
200   0
220   0
240   0
260   0
280   0
300   0
320   0
340   0
360   0
U31-P:ASN-CA
20   0
40   0
60   0
80   0
100   3
120   0
140   0
160   0
180   0
200   0
220   0
240   0

260 0
280 0
300 0
320 0
340 0
360 0
QUO-P:LEU-S2
20 0
40 0
60 0
80 8
100 0
120 0
140 0
160 0
180 0
200 0
220 0
240 0
260 0
280 0
300 0
320 0
340 0
360 0
C31-RIB:TYR-S2
20 0
40 0
60 3
80 0
100 0
120 0
140 0
160 0
180 0
200 0
220 0
240 0
260 3
280 0
300 0
320 0
340 0
360 0
U31-MY:GLN-S2
20 0
40 0
60 3
80 0
100 0
120 0
140 0
160 0
180 0
200 0
220 0
240 0
260 0

280 0
300 0
320 0
340 0
360 0
5BU-P:ALA-S1
20 0
40 0
60 0
80 0
100 0
120 0
140 0
160 0
180 0
200 0
220 0
240 0
260 0
280 0
300 0
320 3
340 0
360 0
QUO-RIB:ASN-CA
20 0
40 0
60 0
80 0
100 0
120 0
140 0
160 4
180 0
200 0
220 0
240 0
260 0
280 0
300 0
320 0
340 0
360 0
IU-RIB:LYS-S1
20 0
40 0
60 0
80 0
100 0
120 0
140 0
160 0
180 0
200 0
220 0
240 3
260 0
280 0

300 0
320 0
340 3
360 0
IU-MY:LYS-S1
20 0
40 0
60 3
80 0
100 2
120 0
140 0
160 5
180 0
200 0
220 0
240 3
260 0
280 2
300 0
320 0
340 0
360 0
C31-RIB:LEU-S1
20 0
40 0
60 0
80 0
100 0
120 0
140 0
160 0
180 0
200 0
220 0
240 0
260 0
280 0
300 0
320 3
340 0
360 0
GTP-M5:ASP-S1
20 0
40 0
60 0
80 0
100 0
120 0
140 0
160 0
180 0
200 0
220 0
240 0
260 0
280 0
300 3

320 0
340 0
360 0
H2U-P:TRP-CA
20 0
40 0
60 0
80 0
100 3
120 0
140 0
160 0
180 0
200 0
220 0
240 0
260 0
280 0
300 0
320 0
340 0
360 0
U34-MY:PRO-CA
20 0
40 0
60 0
80 0
100 0
120 0
140 0
160 0
180 0
200 0
220 0
240 0
260 0
280 0
300 3
320 0
340 0
360 0
IU-P:HIS-CA
20 0
40 0
60 0
80 0
100 3
120 0
140 0
160 0
180 0
200 0
220 0
240 0
260 0
280 0
300 0
320 0

340 0
360 0
FHU-RIB:ASP-CA
20 0
40 0
60 0
80 0
100 0
120 0
140 3
160 0
180 0
200 0
220 0
240 0
260 0
280 0
300 0
320 3
340 0
360 0
IU-P:HIS-S2
20 0
40 0
60 0
80 0
100 0
120 3
140 0
160 0
180 0
200 0
220 0
240 0
260 0
280 0
300 0
320 0
340 0
360 0
FMU-MY:GLU-S1
20 0
40 0
60 0
80 0
100 0
120 0
140 0
160 0
180 0
200 0
220 0
240 0
260 0
280 0
300 3
320 0
340 0

360   0
C31-RIB:SER-CA
20   0
40   0
60   0
80   0
100   0
120   0
140   0
160   0
180   0
200   0
220   0
240   0
260   0
280   0
300   0
320   0
340   3
360   0
FHU-MY:GLN-S2
20   0
40   0
60   0
80   0
100   3
120   0
140   0
160   0
180   0
200   0
220   0
240   0
260   0
280   3
300   0
320   0
340   0
360   0
FHU-P:PRO-CA
20   0
40   0
60   0
80   0
100   0
120   3
140   0
160   0
180   0
200   0
220   0
240   0
260   0
280   0
300   0
320   0
340   0
360   0

QUO-RIB:LYS-S1
20 0
40 0
60 0
80 0
100 0
120 0
140 4
160 0
180 0
200 0
220 0
240 0
260 0
280 0
300 0
320 0
340 0
360 0
IU-P:SER-CA
20 0
40 0
60 0
80 0
100 3
120 0
140 0
160 0
180 0
200 0
220 0
240 0
260 0
280 0
300 0
320 0
340 0
360 0
FHU-P:ASP-S2
20 0
40 0
60 0
80 0
100 0
120 0
140 0
160 3
180 0
200 0
220 0
240 0
260 0
280 0
300 0
320 0
340 0
360 0
5BU-RIB:ILE-S1

20 0
40 0
60 0
80 0
100 0
120 0
140 0
160 0
180 0
200 0
220 0
240 3
260 0
280 0
300 0
320 0
340 0
360 0
DA-M6:GLU-S2
20 0
40 0
60 0
80 0
100 0
120 0
140 0
160 0
180 0
200 0
220 0
240 0
260 0
280 0
300 0
320 0
340 3
360 0
QUO-M6:GLU-S1
20 0
40 0
60 0
80 0
100 0
120 0
140 0
160 4
180 0
200 0
220 0
240 0
260 0
280 0
300 0
320 0
340 0
360 0
QUO-RIB:LEU-S2
20 0

40 0
60 0
80 0
100 0
120 0
140 0
160 0
180 0
200 0
220 0
240 0
260 8
280 0
300 0
320 0
340 0
360 0
IU-MY:MET-CA
20 0
40 0
60 0
80 0
100 0
120 0
140 0
160 2
180 0
200 0
220 0
240 0
260 0
280 0
300 0
320 0
340 0
360 0
IU-MY:ARG-S1
20 0
40 0
60 0
80 0
100 3
120 0
140 3
160 0
180 0
200 0
220 0
240 0
260 0
280 0
300 0
320 0
340 0
360 0
U31-MY:GLU-CA
20 0
40 0

60 0
80 0
100 0
120 3
140 0
160 0
180 0
200 0
220 0
240 0
260 0
280 0
300 0
320 0
340 0
360 0
GTP-M5:GLY-CA
20 0
40 0
60 3
80 0
100 0
120 0
140 0
160 0
180 0
200 0
220 0
240 0
260 0
280 0
300 0
320 0
340 0
360 0
H2U-MY:ASN-CA
20 0
40 0
60 0
80 0
100 0
120 0
140 3
160 0
180 0
200 0
220 0
240 0
260 0
280 3
300 0
320 0
340 0
360 0
FMU-MY:ALA-CA
20 0
40 0
60 0

80 0
100 3
120 0
140 0
160 0
180 0
200 0
220 0
240 0
260 0
280 0
300 0
320 0
340 0
360 0
IU-MY:LEU-S1
20 0
40 0
60 2
80 0
100 0
120 0
140 0
160 0
180 0
200 0
220 0
240 0
260 0
280 0
300 0
320 0
340 0
360 0
U34-P:TYR-S2
20 0
40 0
60 0
80 0
100 0
120 0
140 0
160 0
180 0
200 0
220 0
240 0
260 0
280 0
300 0
320 3
340 0
360 0
U31-MY:GLN-CA
20 0
40 0
60 3
80 0

100   0
120   0
140   0
160   0
180   0
200   0
220   0
240   0
260   0
280   0
300   0
320   0
340   0
360   0
QUO-M5:LYS-S2
20   0
40   0
60   0
80   4
100   0
120   0
140   0
160   0
180   0
200   0
220   0
240   0
260   0
280   0
300   0
320   0
340   0
360   0
FMU-P:ASP-S2
20   0
40   0
60   0
80   0
100   0
120   0
140   0
160   3
180   0
200   0
220   0
240   0
260   0
280   0
300   0
320   0
340   0
360   0
QUO-M5:ARG-S1
20   0
40   0
60   0
80   0
100   0

120   4
140   0
160   0
180   0
200   0
220   0
240   0
260   0
280   0
300   0
320   0
340   0
360   0
PSU-RIB:ARG-S2
20   0
40   0
60   0
80   0
100   0
120   3
140   0
160   0
180   0
200   0
220   0
240   0
260   0
280   0
300   0
320   0
340   0
360   0
M2G-P:GLU-S1
20   0
40   0
60   0
80   0
100   0
120   0
140   0
160   0
180   0
200   0
220   0
240   0
260   0
280   0
300   0
320   0
340   0
360   4
QUO-M6:GLN-S2
20   0
40   0
60   0
80   0
100   4
120   0

140   0
160   0
180   0
200   0
220   0
240   0
260   0
280   0
300   0
320   0
340   0
360   0
FMU-MY:ILE-CA
20   0
40   0
60   0
80   0
100   0
120   0
140   0
160   0
180   0
200   0
220   0
240   0
260   0
280   3
300   0
320   0
340   0
360   0
U31-RIB:ASN-S2
20   0
40   0
60   0
80   0
100   0
120   0
140   0
160   0
180   0
200   0
220   0
240   3
260   0
280   0
300   0
320   0
340   0
360   0
5BU-MY:PRO-S1
20   0
40   0
60   3
80   0
100   0
120   0
140   0

160 0
180 0
200 0
220 0
240 0
260 0
280 0
300 0
320 0
340 0
360 0
H2U-P:ARG-S1
20 0
40 0
60 0
80 3
100 0
120 0
140 0
160 0
180 0
200 0
220 0
240 0
260 0
280 0
300 0
320 0
340 0
360 0
H2U-MY:GLY-CA
20 0
40 0
60 0
80 0
100 0
120 3
140 0
160 0
180 0
200 0
220 0
240 0
260 0
280 0
300 0
320 0
340 0
360 0
U34-RIB:TYR-S2
20 0
40 0
60 0
80 0
100 0
120 0
140 0
160 0

180 0
200 0
220 0
240 0
260 0
280 0
300 3
320 0
340 0
360 0
IU-P:SER-S1
20 0
40 0
60 0
80 0
100 3
120 0
140 0
160 0
180 0
200 0
220 0
240 0
260 0
280 3
300 0
320 0
340 0
360 0
H2U-MY:LYS-S2
20 0
40 0
60 0
80 0
100 0
120 0
140 0
160 0
180 0
200 0
220 0
240 3
260 0
280 0
300 0
320 0
340 0
360 0
FMU-MY:SER-CA
20 0
40 0
60 0
80 0
100 0
120 0
140 3
160 0
180 0

200 0
220 0
240 0
260 0
280 0
300 0
320 0
340 0
360 0
H2U-MY:PHE-S1
20 0
40 0
60 0
80 0
100 0
120 0
140 3
160 0
180 0
200 0
220 0
240 0
260 0
280 0
300 0
320 0
340 0
360 0
FMU-MY:PRO-S1
20 0
40 0
60 0
80 0
100 0
120 0
140 3
160 0
180 0
200 0
220 0
240 0
260 0
280 0
300 0
320 0
340 0
360 0
QUO-M6:LYS-S2
20 0
40 0
60 4
80 0
100 0
120 0
140 0
160 0
180 0
200 0

220 0
240 0
260 0
280 0
300 0
320 0
340 0
360 0
FHU-RIB:TYR-CA
20 0
40 0
60 0
80 0
100 0
120 0
140 0
160 0
180 0
200 0
220 0
240 0
260 0
280 0
300 6
320 0
340 0
360 0
FHU-P:TYR-CA
20 0
40 6
60 0
80 0
100 0
120 0
140 0
160 0
180 3
200 0
220 0
240 0
260 0
280 0
300 0
320 0
340 0
360 0
GTP-M5:THR-CA
20 0
40 0
60 0
80 0
100 3
120 0
140 0
160 0
180 0
200 0
220 0

240   0
260   0
280   0
300   0
320   0
340   0
360   0
U31-MY:PHE-CA
20   0
40   0
60   0
80   0
100   0
120   0
140   0
160   0
180   0
200   0
220   0
240   0
260   0
280   0
300   3
320   0
340   0
360   0
U31-MY:MET-S1
20   0
40   0
60   0
80   3
100   0
120   0
140   0
160   0
180   0
200   0
220   0
240   0
260   0
280   0
300   0
320   0
340   0
360   0
FMU-RIB:ASP-S2
20   0
40   0
60   0
80   0
100   0
120   3
140   0
160   0
180   0
200   0
220   0
240   0

260   0
280   0
300   0
320   0
340   0
360   0
H2U-RIB:LYS-S2
20   0
40   0
60   0
80   0
100   0
120   0
140   0
160   0
180   0
200   0
220   0
240   0
260   0
280   0
300   3
320   0
340   0
360   0
DA-M5:THR-S1
20   0
40   0
60   0
80   0
100   0
120   0
140   0
160   0
180   0
200   0
220   0
240   0
260   0
280   0
300   0
320   3
340   0
360   0
FMU-MY:PHE-S2
20   0
40   0
60   0
80   0
100   0
120   0
140   0
160   0
180   0
200   0
220   0
240   0
260   6

280 0
300 0
320 0
340 0
360 0
U31-MY:TYR-S1
20 0
40 0
60 0
80 0
100 0
120 0
140 0
160 0
180 0
200 0
220 0
240 0
260 0
280 0
300 3
320 0
340 0
360 0
U34-P:GLU-CA
20 0
40 0
60 0
80 0
100 0
120 0
140 0
160 0
180 0
200 0
220 0
240 0
260 0
280 0
300 0
320 0
340 0
360 3
FMU-MY:ARG-CA
20 0
40 0
60 0
80 3
100 0
120 0
140 0
160 0
180 0
200 0
220 0
240 0
260 0
280 0

300 0
320 0
340 0
360 0
IU-MY:VAL-S1
20 0
40 0
60 0
80 0
100 2
120 0
140 0
160 0
180 0
200 0
220 0
240 0
260 0
280 0
300 0
320 0
340 0
360 0
QUO-M6:LEU-S2
20 0
40 0
60 0
80 0
100 0
120 0
140 0
160 0
180 0
200 0
220 0
240 4
260 4
280 0
300 0
320 0
340 0
360 0
FHU-P:PRO-S1
20 0
40 0
60 0
80 0
100 0
120 3
140 0
160 0
180 0
200 0
220 0
240 0
260 0
280 0
300 0

320 0
340 0
360 0
5BU-P:THR-CA
20 0
40 0
60 0
80 0
100 0
120 0
140 0
160 3
180 0
200 0
220 0
240 0
260 0
280 0
300 0
320 0
340 0
360 0
FHU-MY:ILE-CA
20 0
40 0
60 0
80 0
100 0
120 0
140 3
160 0
180 0
200 0
220 0
240 0
260 0
280 0
300 0
320 3
340 0
360 0
C31-MY:GLU-S1
20 0
40 0
60 0
80 0
100 0
120 0
140 3
160 0
180 0
200 0
220 0
240 0
260 0
280 0
300 0
320 0

340 0
360 0
IU-P:ARG-S2
20 0
40 0
60 0
80 0
100 0
120 0
140 0
160 0
180 0
200 0
220 0
240 3
260 0
280 0
300 0
320 0
340 0
360 0
C31-RIB:ASP-S2
20 0
40 6
60 0
80 0
100 0
120 0
140 0
160 0
180 0
200 0
220 0
240 0
260 0
280 0
300 0
320 0
340 0
360 0
DA-M5:GLU-S2
20 0
40 0
60 0
80 0
100 0
120 0
140 0
160 0
180 0
200 0
220 0
240 0
260 0
280 0
300 0
320 3
340 0

360 0
C31-MY:ASP-CA
20 0
40 0
60 0
80 0
100 0
120 0
140 0
160 0
180 0
200 0
220 0
240 3
260 0
280 0
300 0
320 0
340 0
360 0
U34-MY:TYR-S2
20 0
40 0
60 0
80 0
100 0
120 0
140 0
160 0
180 0
200 0
220 0
240 0
260 0
280 0
300 3
320 0
340 0
360 0
IU-MY:PRO-S1
20 0
40 0
60 0
80 3
100 0
120 0
140 0
160 0
180 0
200 0
220 0
240 0
260 0
280 0
300 2
320 0
340 0
360 0

C31-P:LEU-S2
20 0
40 3
60 0
80 0
100 0
120 0
140 0
160 0
180 0
200 0
220 0
240 0
260 0
280 0
300 0
320 0
340 0
360 0
5BU-MY:ILE-S1
20 0
40 0
60 0
80 0
100 0
120 0
140 0
160 0
180 0
200 0
220 0
240 0
260 0
280 0
300 3
320 0
340 0
360 0
IU-RIB:PRO-CA
20 0
40 0
60 0
80 0
100 0
120 0
140 3
160 0
180 0
200 0
220 0
240 0
260 0
280 0
300 0
320 0
340 0
360 0
U31-P:ARG-CA

20 0
40 0
60 0
80 0
100 0
120 0
140 0
160 0
180 0
200 0
220 0
240 0
260 0
280 0
300 0
320 0
340 3
360 0
DA-M5:ASP-S1
20 0
40 0
60 0
80 0
100 0
120 0
140 0
160 3
180 0
200 0
220 0
240 0
260 0
280 0
300 0
320 0
340 0
360 0
GTP-M6:GLY-CA
20 0
40 0
60 0
80 3
100 0
120 0
140 0
160 0
180 0
200 0
220 0
240 0
260 0
280 0
300 0
320 0
340 0
360 0
H2U-P:LEU-S2
20 0

40 0
60 0
80 0
100 3
120 0
140 0
160 0
180 0
200 0
220 0
240 0
260 0
280 0
300 0
320 0
340 0
360 0
GTP-M5:ASN-S1
20 3
40 0
60 0
80 0
100 0
120 0
140 0
160 0
180 0
200 0
220 0
240 0
260 0
280 0
300 0
320 0
340 0
360 0
FHU-RIB:ILE-S1
20 0
40 0
60 0
80 0
100 0
120 0
140 0
160 0
180 0
200 0
220 0
240 0
260 0
280 0
300 6
320 0
340 0
360 0
C31-MY:GLN-S2
20 0
40 0

60 0
80 0
100 0
120 0
140 0
160 0
180 0
200 0
220 0
240 0
260 0
280 0
300 3
320 0
340 0
360 0
QUO-M5:ASN-CA
20 0
40 0
60 0
80 4
100 0
120 0
140 0
160 0
180 0
200 0
220 0
240 0
260 0
280 0
300 0
320 0
340 0
360 0
IU-P:ASP-S2
20 0
40 0
60 0
80 0
100 0
120 0
140 3
160 0
180 0
200 0
220 0
240 0
260 0
280 0
300 0
320 0
340 0
360 0
QUO-M6:GLU-CA
20 0
40 0
60 0

80 0
100 0
120 0
140 4
160 0
180 0
200 0
220 0
240 0
260 0
280 0
300 0
320 0
340 0
360 0
FHU-P:ASP-S1
20 0
40 0
60 0
80 0
100 0
120 0
140 0
160 3
180 0
200 0
220 0
240 0
260 0
280 0
300 0
320 0
340 0
360 0
U31-P:ARG-S2
20 0
40 0
60 0
80 0
100 0
120 0
140 0
160 0
180 0
200 0
220 0
240 0
260 0
280 0
300 3
320 0
340 0
360 0
QUO-P:ASN-S2
20 0
40 0
60 0
80 0

100 0
120 0
140 0
160 0
180 0
200 4
220 0
240 0
260 0
280 0
300 0
320 0
340 0
360 0
U31-RIB:GLN-S1
20 0
40 0
60 0
80 3
100 0
120 0
140 0
160 0
180 0
200 0
220 0
240 0
260 0
280 0
300 0
320 0
340 0
360 0
C31-RIB:THR-S1
20 0
40 0
60 0
80 0
100 0
120 0
140 0
160 0
180 0
200 0
220 0
240 0
260 0
280 3
300 0
320 0
340 0
360 0
FHU-P:SER-S1
20 0
40 0
60 0
80 0
100 0

120   0
140   0
160   0
180   0
200   0
220   0
240   0
260   0
280   0
300   0
320   0
340   3
360   0
4SU-P:THR-S1
20   0
40   0
60   0
80   0
100   0
120   0
140   0
160   3
180   0
200   0
220   0
240   0
260   0
280   0
300   0
320   0
340   0
360   0
QUO-M6:ASN-S2
20   0
40   0
60   0
80   4
100   0
120   0
140   0
160   0
180   0
200   0
220   0
240   0
260   0
280   0
300   0
320   0
340   0
360   0
FMU-MY:GLN-S1
20   0
40   0
60   0
80   0
100   0
120   0

140 0
160 0
180 0
200 0
220 0
240 0
260 0
280 0
300 3
320 0
340 0
360 0
H2U-MY:ARG-S1
20 0
40 0
60 0
80 0
100 0
120 0
140 0
160 0
180 0
200 0
220 0
240 0
260 0
280 0
300 3
320 0
340 0
360 0
C31-P:ASN-S1
20 0
40 0
60 0
80 0
100 0
120 0
140 3
160 0
180 0
200 0
220 0
240 0
260 0
280 0
300 0
320 0
340 0
360 0
OMC-P:LYS-S1
20 0
40 0
60 0
80 0
100 3
120 0
140 0

160   0
180   0
200   0
220   0
240   0
260   0
280   0
300   0
320   0
340   0
360   0
U34-MY:ASP-S2
20   0
40   0
60   0
80   0
100   3
120   0
140   0
160   0
180   0
200   0
220   0
240   0
260   0
280   0
300   0
320   0
340   0
360   0
U31-RIB:GLN-CA
20   0
40   0
60   0
80   3
100   0
120   0
140   0
160   0
180   0
200   0
220   0
240   0
260   0
280   0
300   0
320   0
340   0
360   0
U31-RIB:PHE-S1
20   0
40   0
60   0
80   0
100   0
120   0
140   0
160   0

180 0
200 0
220 0
240 0
260 0
280 0
300 3
320 0
340 0
360 0
U34-P:ARG-S1
20 0
40 0
60 0
80 0
100 0
120 0
140 0
160 0
180 0
200 0
220 0
240 0
260 0
280 0
300 0
320 3
340 0
360 0
FMU-MY:ALA-S1
20 0
40 0
60 0
80 0
100 3
120 0
140 0
160 0
180 0
200 0
220 0
240 0
260 0
280 0
300 0
320 0
340 0
360 0
IU-P:HIS-S1
20 0
40 0
60 0
80 0
100 3
120 0
140 0
160 0
180 0

200 0
220 0
240 0
260 0
280 0
300 0
320 0
340 0
360 0
DA-M5:ASP-S2
20 0
40 0
60 0
80 0
100 0
120 0
140 0
160 3
180 0
200 0
220 0
240 0
260 0
280 0
300 0
320 0
340 0
360 0
FHU-RIB:PRO-CA
20 0
40 0
60 0
80 0
100 0
120 0
140 0
160 0
180 0
200 0
220 0
240 3
260 0
280 0
300 0
320 0
340 0
360 0
FMU-RIB:MET-S1
20 0
40 0
60 0
80 0
100 0
120 0
140 0
160 0
180 0
200 0

220   0
240   0
260   0
280   0
300   0
320   3
340   0
360   0
FMU-RIB:VAL-CA
20   0
40   0
60   0
80   0
100   0
120   0
140   0
160   0
180   0
200   0
220   0
240   0
260   0
280   3
300   0
320   0
340   0
360   0
QUO-M6:ASP-CA
20   0
40   0
60   0
80   0
100   0
120   4
140   0
160   0
180   0
200   0
220   0
240   0
260   0
280   0
300   0
320   0
340   0
360   0
H2U-P:THR-CA
20   0
40   0
60   0
80   0
100   0
120   6
140   0
160   0
180   0
200   0
220   0

240 0
260 0
280 0
300 0
320 0
340 0
360 0
H2U-RIB:PHE-CA
20 0
40 0
60 0
80 0
100 0
120 0
140 0
160 0
180 0
200 0
220 0
240 0
260 0
280 3
300 0
320 0
340 0
360 0
QUO-M5:PHE-CA
20 0
40 0
60 0
80 0
100 0
120 0
140 0
160 0
180 0
200 0
220 0
240 0
260 0
280 0
300 4
320 0
340 0
360 0
FMU-RIB:GLN-CA
20 0
40 0
60 0
80 0
100 0
120 0
140 0
160 0
180 0
200 0
220 0
240 0

260 0
280 3
300 0
320 0
340 0
360 0
C31-MY:LEU-S1
20 0
40 0
60 0
80 0
100 0
120 0
140 0
160 0
180 0
200 0
220 0
240 0
260 0
280 3
300 0
320 0
340 0
360 0
QUO-RIB:ASP-S1
20 0
40 0
60 0
80 0
100 0
120 0
140 4
160 0
180 0
200 0
220 0
240 0
260 0
280 0
300 0
320 0
340 0
360 0
FMU-RIB:GLU-S1
20 0
40 0
60 0
80 0
100 0
120 0
140 0
160 0
180 0
200 0
220 0
240 0
260 0

280 0
300 0
320 0
340 3
360 0
U31-MY:GLU-S1
20 0
40 0
60 0
80 0
100 0
120 0
140 3
160 0
180 0
200 0
220 0
240 0
260 0
280 0
300 0
320 0
340 0
360 0
G:HIS-S2
WoCr 622
Sug 1198
Hoo 609
C:GLU-CA
WoCr 53
Sug 50
Hoo 27
A:ASN-CA
WoCr 212
Sug 168
Hoo 85
C:LEU-CA
WoCr 105
Sug 114
Hoo 33
U:ASN-S2
WoCr 78
Sug 798
Hoo 473
G:ARG-CA
WoCr 399
Sug 1091
Hoo 212
C:SER-CA
WoCr 151
Sug 430
Hoo 185
A:GLU-CA
WoCr 196
Sug 214
Hoo 80
G:GLN-CA
WoCr 20

Sug 477
Hoo 96
C:ASP-S2
WoCr 234
Sug 1082
Hoo 232
C:VAL-S1
WoCr 88
Sug 193
Hoo 242
G:ALA-S1
WoCr 348
Sug 916
Hoo 385
C:GLN-S1
WoCr 160
Sug 624
Hoo 73
G:GLN-S1
WoCr 157
Sug 922
Hoo 204
U:LYS-S2
WoCr 208
Sug 929
Hoo 1367
C:ASN-S2
WoCr 62
Sug 740
Hoo 323
A:LYS-S2
WoCr 803
Sug 1272
Hoo 2365
G:LYS-S1
WoCr 390
Sug 891
Hoo 622
C:GLU-S1
WoCr 42
Sug 206
Hoo 112
A:TRP-S2
WoCr 111
Sug 534
Hoo 20
C:TRP-S1
WoCr 8
Sug 95
Hoo 9
G:TRP-CA
WoCr 52
Sug 173
Hoo 20
U:GLU-S1
WoCr 96
Sug 193
Hoo 86

G:GLU-S2
WoCr 365
Sug 1258
Hoo 270
A:PHE-S1
WoCr 44
Sug 278
Hoo 24
G:CYS-CA
WoCr 28
Sug 20
Hoo 0
A:ASN-S2
WoCr 769
Sug 1320
Hoo 350
G:ALA-CA
WoCr 196
Sug 311
Hoo 262
A:ASN-S1
WoCr 296
Sug 706
Hoo 174
G:LEU-CA
WoCr 168
Sug 278
Hoo 572
G:ASN-CA
WoCr 224
Sug 697
Hoo 218
A:TYR-S1
WoCr 131
Sug 466
Hoo 84
C:LEU-S2
WoCr 194
Sug 118
Hoo 30
U:MET-S1
WoCr 17
Sug 78
Hoo 6
C:TYR-S1
WoCr 39
Sug 75
Hoo 21
G:TRP-S1
WoCr 80
Sug 235
Hoo 131
U:THR-S1
WoCr 97
Sug 405
Hoo 36
C:MET-CA
WoCr 6

Sug   96
Hoo   38
C:LYS-S1
WoCr   255
Sug   732
Hoo   218
A:ILE-S1
WoCr   331
Sug   379
Hoo   20
G:HIS-CA
WoCr   112
Sug   353
Hoo   155
G:GLN-S2
WoCr   842
Sug   945
Hoo   704
C:LEU-S1
WoCr   104
Sug   510
Hoo   266
U:LYS-CA
WoCr   91
Sug   222
Hoo   155
A:MET-CA
WoCr   204
Sug   68
Hoo   40
G:ASP-CA
WoCr   377
Sug   285
Hoo   44
C:ALA-S1
WoCr   184
Sug   634
Hoo   240
U:PRO-S1
WoCr   54
Sug   550
Hoo   47
C:PHE-CA
WoCr   24
Sug   109
Hoo   75
G:VAL-S1
WoCr   390
Sug   382
Hoo   48
A:TRP-S1
WoCr   64
Sug   108
Hoo   24
U:GLU-S2
WoCr   47
Sug   152
Hoo   243

U:GLN-S1
WoCr 125
Sug 232
Hoo 102
G:ARG-S2
WoCr 1284
Sug 1876
Hoo 2121
G:TYR-S2
WoCr 209
Sug 323
Hoo 178
G:ASN-S2
WoCr 778
Sug 750
Hoo 855
A:HIS-CA
WoCr 187
Sug 376
Hoo 80
U:SER-S1
WoCr 39
Sug 765
Hoo 60
C:VAL-CA
WoCr 42
Sug 144
Hoo 27
G:GLY-CA
WoCr 459
Sug 1718
Hoo 1216
C:ASP-S1
WoCr 117
Sug 446
Hoo 63
A:HIS-S2
WoCr 387
Sug 866
Hoo 205
A:LEU-S2
WoCr 439
Sug 256
Hoo 284
U:TRP-CA
WoCr 3
Sug 6
Hoo 15
U:ASP-S2
WoCr 180
Sug 733
Hoo 145
A:TRP-CA
WoCr 24
Sug 52
Hoo 24
A:ALA-S1
WoCr 513

Sug 640
Hoo 668
C:TRP-S2
WoCr 123
Sug 360
Hoo 23
A:SER-S1
WoCr 449
Sug 949
Hoo 775
G:GLU-CA
WoCr 244
Sug 267
Hoo 68
U:VAL-CA
WoCr 85
Sug 122
Hoo 200
U:ASN-CA
WoCr 99
Sug 229
Hoo 67
C:ASP-CA
WoCr 144
Sug 431
Hoo 176
U:PHE-S2
WoCr 30
Sug 389
Hoo 2
U:TYR-S2
WoCr 33
Sug 41
Hoo 39
U:TYR-CA
WoCr 57
Sug 114
Hoo 9
C:LYS-CA
WoCr 187
Sug 718
Hoo 250
U:HIS-CA
WoCr 15
Sug 187
Hoo 21
C:GLU-S2
WoCr 248
Sug 696
Hoo 215
U:CYS-S1
WoCr 12
Sug 6
Hoo 5
A:ALA-CA
WoCr 235
Sug 317
Hoo 112

G:ARG-S1
WoCr 754
Sug 1256
Hoo 1319
U:CYS-CA
WoCr 74
Sug 3
Hoo 5
U:LEU-S2
WoCr 46
Sug 248
Hoo 71
A:ASP-S2
WoCr 325
Sug 568
Hoo 618
A:VAL-S1
WoCr 271
Sug 733
Hoo 183
U:ASN-S1
WoCr 105
Sug 252
Hoo 131
U:LEU-S1
WoCr 116
Sug 130
Hoo 27
A:GLN-CA
WoCr 176
Sug 128
Hoo 200
U:ARG-CA
WoCr 179
Sug 437
Hoo 370
U:VAL-S1
WoCr 12
Sug 127
Hoo 122
A:PRO-CA
WoCr 1272
Sug 253
Hoo 216
A:MET-S2
WoCr 151
Sug 234
Hoo 36
U:PHE-CA
WoCr 33
Sug 126
Hoo 5
U:MET-S2
WoCr 20
Sug 67
Hoo 97
A:PRO-S1
WoCr 521

Sug   826
Hoo   165
G:ASP-S1
WoCr   248
Sug   710
Hoo   135
A:GLN-S2
WoCr   429
Sug   614
Hoo   404
U:ALA-CA
WoCr   43
Sug   434
Hoo   229
A:ARG-S1
WoCr   380
Sug   748
Hoo   1089
U:PHE-S1
WoCr   30
Sug   87
Hoo   5
C:GLN-CA
WoCr   136
Sug   139
Hoo   12
G:SER-S1
WoCr   436
Sug   1375
Hoo   544
G:PHE-S1
WoCr   209
Sug   407
Hoo   16
G:SER-CA
WoCr   465
Sug   892
Hoo   378
U:MET-CA
WoCr   3
Sug   30
Hoo   9
C:ILE-S1
WoCr   15
Sug   258
Hoo   74
A:GLU-S2
WoCr   423
Sug   530
Hoo   384
U:HIS-S2
WoCr   639
Sug   408
Hoo   64
A:ARG-S2
WoCr   1501
Sug   2283
Hoo   2197

G:PHE-S2
WoCr   41
Sug   493
Hoo   68
G:CYS-S1
WoCr   24
Sug   8
Hoo   0
U:HIS-S1
WoCr   21
Sug   220
Hoo   21
A:PHE-S2
WoCr   198
Sug   350
Hoo   44
C:GLY-CA
WoCr   778
Sug   1917
Hoo   501
G:TRP-S2
WoCr   340
Sug   341
Hoo   28
U:GLU-CA
WoCr   47
Sug   83
Hoo   15
G:TYR-S1
WoCr   114
Sug   246
Hoo   164
U:GLN-S2
WoCr   240
Sug   452
Hoo   102
A:HIS-S1
WoCr   218
Sug   562
Hoo   72
G:LYS-CA
WoCr   403
Sug   682
Hoo   615
U:ILE-CA
WoCr   33
Sug   15
Hoo   39
A:MET-S1
WoCr   145
Sug   98
Hoo   116
C:TRP-CA
WoCr   11
Sug   11
Hoo   6
G:ASN-S1
WoCr   449

Sug 533
Hoo 637
G:PRO-CA
WoCr 159
Sug 879
Hoo 188
A:THR-CA
WoCr 156
Sug 223
Hoo 189
G:LEU-S1
WoCr 104
Sug 174
Hoo 92
A:ILE-CA
WoCr 113
Sug 243
Hoo 70
G:ILE-CA
WoCr 98
Sug 122
Hoo 36
A:ARG-CA
WoCr 446
Sug 761
Hoo 205
G:THR-CA
WoCr 72
Sug 401
Hoo 96
A:TYR-S2
WoCr 148
Sug 506
Hoo 201
C:HIS-S2
WoCr 86
Sug 475
Hoo 301
C:ASN-CA
WoCr 42
Sug 386
Hoo 67
U:GLY-CA
WoCr 166
Sug 1482
Hoo 94
A:GLY-CA
WoCr 586
Sug 1988
Hoo 1160
G:PRO-S1
WoCr 253
Sug 1723
Hoo 229
A:LEU-CA
WoCr 174
Sug 221
Hoo 56

C:HIS-CA
WoCr 71
Sug 333
Hoo 163
C:ARG-S1
WoCr 924
Sug 905
Hoo 990
C:THR-S1
WoCr 177
Sug 955
Hoo 180
C:CYS-S1
WoCr 3
Sug 6
Hoo 6
C:HIS-S1
WoCr 43
Sug 415
Hoo 30
A:TYR-CA
WoCr 213
Sug 136
Hoo 84
A:LEU-S1
WoCr 224
Sug 603
Hoo 104
A:CYS-S1
WoCr 16
Sug 16
Hoo 24
A:CYS-CA
WoCr 12
Sug 16
Hoo 16
C:PHE-S2
WoCr 28
Sug 57
Hoo 48
G:ASP-S2
WoCr 890
Sug 1360
Hoo 781
C:CYS-CA
WoCr 3
Sug 3
Hoo 3
G:LEU-S2
WoCr 307
Sug 683
Hoo 416
A:ASP-CA
WoCr 337
Sug 281
Hoo 141
U:LEU-CA
WoCr 45

Sug   36
Hoo   76
G:TYR-CA
WoCr   82
Sug   66
Hoo   21
U:LYS-S1
WoCr   114
Sug   420
Hoo   125
U:ARG-S2
WoCr   113
Sug   1518
Hoo   408
C:ARG-CA
WoCr   72
Sug   203
Hoo   616
C:ILE-CA
WoCr   21
Sug   184
Hoo   162
G:GLU-S1
WoCr   185
Sug   519
Hoo   82
G:ILE-S1
WoCr   137
Sug   201
Hoo   16
A:ASP-S1
WoCr   171
Sug   507
Hoo   261
U:SER-CA
WoCr   36
Sug   205
Hoo   113
U:PRO-CA
WoCr   223
Sug   213
Hoo   96
U:ILE-S1
WoCr   53
Sug   263
Hoo   193
C:TYR-CA
WoCr   27
Sug   48
Hoo   12
U:TRP-S1
WoCr   0
Sug   74
Hoo   18
C:LYS-S2
WoCr   339
Sug   1509
Hoo   1419

G:MET-CA
WoCr 72
Sug 209
Hoo 12
C:ARG-S2
WoCr 326
Sug 1271
Hoo 1743
G:HIS-S1
WoCr 226
Sug 535
Hoo 93
U:ARG-S1
WoCr 182
Sug 263
Hoo 619
G:THR-S1
WoCr 388
Sug 738
Hoo 220
C:PHE-S1
WoCr 80
Sug 18
Hoo 18
G:MET-S2
WoCr 154
Sug 553
Hoo 109
A:LYS-S1
WoCr 427
Sug 1178
Hoo 544
C:THR-CA
WoCr 77
Sug 562
Hoo 27
C:PRO-CA
WoCr 371
Sug 643
Hoo 42
C:GLN-S2
WoCr 618
Sug 974
Hoo 212
C:SER-S1
WoCr 148
Sug 621
Hoo 317
C:ALA-CA
WoCr 74
Sug 671
Hoo 211
G:PHE-CA
WoCr 27
Sug 235
Hoo 24
U:ALA-S1
WoCr 24

Sug 417
Hoo 190
C:MET-S2
WoCr 34
Sug 520
Hoo 15
C:MET-S1
WoCr 12
Sug 109
Hoo 9
A:GLN-S1
WoCr 751
Sug 292
Hoo 56
A:GLU-S1
WoCr 337
Sug 539
Hoo 96
U:ASP-S1
WoCr 491
Sug 322
Hoo 101
A:LYS-CA
WoCr 346
Sug 725
Hoo 219
U:ASP-CA
WoCr 18
Sug 73
Hoo 15
U:THR-CA
WoCr 24
Sug 35
Hoo 45
C:PRO-S1
WoCr 91
Sug 667
Hoo 334
G:MET-S1
WoCr 52
Sug 140
Hoo 16
C:ASN-S1
WoCr 338
Sug 682
Hoo 286
U:TRP-S2
WoCr 22
Sug 279
Hoo 112
C:TYR-S2
WoCr 39
Sug 148
Hoo 111
U:TYR-S1
WoCr 45
Sug 12
Hoo 150

A:PHE-CA
WoCr 68
Sug 193
Hoo 28
U:GLN-CA
WoCr 46
Sug 85
Hoo 226
A:SER-CA
WoCr 380
Sug 499
Hoo 92
G:LYS-S2
WoCr 785
Sug 2175
Hoo 2770
A:THR-S1
WoCr 349
Sug 692
Hoo 409
A:VAL-CA
WoCr 380
Sug 237
Hoo 138
G:VAL-CA
WoCr 146
Sug 96
Hoo 64
