# Supplementary material for: DARS-RNP and QUASI-RNP: New statistical potentials for protein-RNA docking
Source: BMC Bioinformatics. 2011 Aug 18;12:348. doi: 10.1186/1471-2105-12-348 (PMC3179970; doi:10.1186/1471-2105-12-348)
Supplement: Additional file 5 — Expected_DARS.pdf. Expected number of contacts in each distance, angle, and site bin, for each pair wise interaction in the DARS-RNP potential. [file 1471-2105-12-348-S5.PDF]

EXPECTED
A-RIB:GLN-S2
3 0
4 0
5 3
6 8
7 14
8 21
9 29
M2G-P:GLU-CA
3 0
4 0
5 0
6 0
7 0
8 0
9 0
U-Y:GLU-S1
3 0
4 0
5 1
6 0
7 11
8 20
9 33
C-Y:HIS-CA
3 0
4 0
5 0
6 0
7 1
8 4
9 8
FHU-RIB:ASP-S2
3 0
4 0
5 0
6 0
7 0
8 0
9 0
U31-P:HIS-CA
3 0
4 0
5 0
6 0
7 0
8 0
9 0
C31-RIB:GLN-S2
3 0
4 0
5 0
6 0
7 0
8 0
9 0
H2U-RIB:PHE-S2

3 0
4 0
5 0
6 0
7 0
8 0
9 0
FHU-MY:GLN-S2
3 0
4 0
5 0
6 0
7 0
8 0
9 0
H2U-MY:LEU-S2
3 0
4 0
5 0
6 0
7 0
8 0
9 0
QUO-M5:PHE-CA
3 0
4 0
5 0
6 0
7 0
8 0
9 0
FHU-RIB:THR-S1
3 0
4 0
5 0
6 0
7 0
8 0
9 0
G-RIB:ARG-S2
3 0
4 1
5 9
6 26
7 49
8 75
9 103
FHU-P:TYR-S2
3 0
4 0
5 0
6 0
7 0
8 0
9 0
G-P:TRP-CA
3 0
4 0

5 0
6 1
7 2
8 5
9 9
C-P:TRP-S1
3 0
4 0
5 0
6 1
7 2
8 4
9 6
C31-RIB:PHE-S1
3 0
4 0
5 0
6 0
7 0
8 0
9 0
U-Y:ARG-S2
3 0
4 0
5 2
6 6
7 13
8 22
9 34
U31-P:ARG-S2
3 0
4 0
5 0
6 0
7 0
8 0
9 0
DA-M6:SER-CA
3 0
4 0
5 0
6 0
7 0
8 0
9 0
M2G-P:SER-S1
3 0
4 0
5 0
6 0
7 0
8 0
9 0
DA-RIB:MET-S2
3 0
4 0
5 0
6 0

7 0
8 0
9 0
A-R5:HIS-CA
3 0
4 0
5 0
6 0
7 2
8 5
9 10
G-RIB:ILE-S1
3 0
4 0
5 1
6 4
7 10
8 17
9 27
C31-MY:LEU-S1
3 0
4 0
5 0
6 0
7 0
8 0
9 0
IU-RIB:VAL-S1
3 0
4 0
5 0
6 0
7 0
8 0
9 0
C-Y:ILE-S1
3 0
4 0
5 0
6 1
7 3
8 5
9 10
U-P:LYS-CA
3 0
4 0
5 1
6 5
7 13
8 22
9 33
A-P:TRP-S1
3 0
4 0
5 0
6 1
7 2
8 4

9   6
C-P:GLN-S2
3   0
4   0
5   3
6   8
7   14
8   20
9   26
QUO-RIB:PHE-S2
3   0
4   0
5   0
6   0
7   0
8   0
9   0
U-RIB:LYS-CA
3   0
4   0
5   1
6   4
7   11
8   20
9   32
C-P:VAL-S1
3   0
4   0
5   2
6   6
7   12
8   18
9   29
G-R5:LYS-CA
3   0
4   0
5   0
6   3
7   9
8   24
9   47
GTP-M5:ASN-S1
3   0
4   0
5   0
6   0
7   0
8   0
9   0
GTP-RIB:ARG-CA
3   0
4   0
5   0
6   0
7   0
8   0
9   0
IU-RIB:GLN-S1

3 0
4 0
5 0
6 0
7 0
8 0
9 0
DA-M5:ASN-S2
3 0
4 0
5 0
6 0
7 0
8 0
9 0
G-RIB:GLU-CA
3 0
4 0
5 2
6 10
7 28
8 56
9 93
DA-M6:GLN-S1
3 0
4 0
5 0
6 0
7 0
8 0
9 0
G-R6:ALA-S1
3 0
4 1
5 4
6 9
7 17
8 28
9 44
A-R5:LEU-CA
3 0
4 0
5 0
6 0
7 2
8 7
9 15
5BU-RIB:PRO-CA
3 0
4 0
5 0
6 0
7 0
8 0
9 0
FMU-RIB:ALA-CA
3 0
4 0

5 0
6 0
7 0
8 0
9 0
C-P:ARG-S2
3 0
4 2
5 8
6 21
7 36
8 50
9 68
IU-RIB:LYS-S2
3 0
4 0
5 0
6 0
7 0
8 0
9 0
QUO-P:LEU-CA
3 0
4 0
5 0
6 0
7 0
8 0
9 0
G-R5:VAL-CA
3 0
4 0
5 0
6 1
7 5
8 13
9 27
A-RIB:LEU-S1
3 0
4 0
5 1
6 3
7 8
8 15
9 24
A-P:VAL-S1
3 0
4 0
5 2
6 6
7 11
8 19
9 30
G-R5:HIS-S2
3 0
4 0
5 0
6 2

7 7
8 13
9 21
DA-RIB:ASN-S2
3 0
4 0
5 0
6 0
7 0
8 0
9 0
U-Y:SER-CA
3 0
4 0
5 0
6 2
7 6
8 11
9 18
5BU-P:ILE-CA
3 0
4 0
5 0
6 0
7 0
8 0
9 0
IU-MY:THR-CA
3 0
4 0
5 0
6 0
7 0
8 0
9 0
C31-RIB:SER-CA
3 0
4 0
5 0
6 0
7 0
8 0
9 0
FHU-P:THR-CA
3 0
4 0
5 0
6 0
7 0
8 0
9 0
FHU-P:ARG-S2
3 0
4 0
5 0
6 0
7 0
8 0

9 0
U-RIB:THR-S1
3 0
4 0
5 2
6 5
7 10
8 16
9 23
U-RIB:HIS-S2
3 0
4 0
5 0
6 3
7 6
8 9
9 12
G-R6:GLN-CA
3 0
4 0
5 0
6 1
7 4
8 11
9 19
FMU-RIB:VAL-S1
3 0
4 0
5 0
6 0
7 0
8 0
9 0
A-RIB:LYS-S2
3 0
4 0
5 9
6 21
7 36
8 51
9 69
C-P:LEU-CA
3 0
4 0
5 0
6 3
7 9
8 19
9 32
A-R6:ALA-S1
3 0
4 1
5 4
6 8
7 14
8 21
9 34
C-Y:ASN-S2

3 0
4 0
5 1
6 4
7 9
8 16
9 24
C31-MY:THR-CA
3 0
4 0
5 0
6 0
7 0
8 0
9 0
IU-MY:SER-S1
3 0
4 0
5 0
6 0
7 0
8 0
9 0
U-RIB:ALA-CA
3 0
4 0
5 1
6 4
7 9
8 16
9 25
U31-P:ARG-CA
3 0
4 0
5 0
6 0
7 0
8 0
9 0
G-P:PRO-S1
3 0
4 0
5 5
6 12
7 22
8 34
9 49
A-R5:ILE-CA
3 0
4 0
5 0
6 0
7 2
8 6
9 13
DA-M6:SER-S1
3 0
4 0

5 0
6 0
7 0
8 0
9 0
A-R6:HIS-S1
3 0
4 0
5 0
6 2
7 4
8 7
9 12
DA-RIB:ASN-S1
3 0
4 0
5 0
6 0
7 0
8 0
9 0
A-P:GLN-S1
3 0
4 0
5 1
6 5
7 11
8 17
9 25
C-RIB:ASN-S2
3 0
4 0
5 3
6 9
7 16
8 23
9 32
C31-RIB:PHE-CA
3 0
4 0
5 0
6 0
7 0
8 0
9 0
C31-P:PHE-S2
3 0
4 0
5 0
6 0
7 0
8 0
9 0
C31-P:GLN-CA
3 0
4 0
5 0
6 0

7 0
8 0
9 0
GTP-RIB:ARG-S2
3 0
4 0
5 0
6 0
7 0
8 0
9 0
G-RIB:HIS-S1
3 0
4 0
5 1
6 4
7 9
8 16
9 24
A-P:GLU-S2
3 0
4 0
5 0
6 26
7 42
8 60
9 80
IU-P:ASP-S2
3 0
4 0
5 0
6 0
7 0
8 0
9 0
G-P:ALA-S1
3 0
4 3
5 9
6 18
7 29
8 43
9 65
FMU-MY:ASP-S1
3 0
4 0
5 0
6 0
7 0
8 0
9 0
A-R5:TRP-S1
3 0
4 0
5 0
6 0
7 1
8 2

9 4
U31-P:ASP-S2
3 0
4 0
5 0
6 0
7 0
8 0
9 0
C-RIB:GLN-CA
3 0
4 0
5 0
6 2
7 6
8 13
9 22
U-RIB:ARG-S2
3 0
4 0
5 4
6 12
7 22
8 33
9 45
C-P:HIS-S2
3 0
4 0
5 1
6 4
7 8
8 12
9 17
FMU-RIB:PHE-S2
3 0
4 0
5 0
6 0
7 0
8 0
9 0
G-RIB:GLN-CA
3 0
4 0
5 0
6 3
7 10
8 19
9 32
G-R5:LEU-S2
3 0
4 0
5 0
6 2
7 6
8 11
9 21
U-RIB:PHE-CA

3   0
4   0
5   0
6   0
7   2
8   4
9   8
C31-P:ASP-S1
3   0
4   0
5   0
6   0
7   0
8   0
9   0
U-Y:GLN-CA
3   0
4   0
5   0
6   1
7   3
8   5
9   10
A-R6:TRP-CA
3   0
4   0
5   0
6   0
7   1
8   2
9   4
5BU-P:ARG-S2
3   0
4   0
5   0
6   0
7   0
8   0
9   0
A-R5:GLN-CA
3   0
4   0
5   0
6   0
7   2
8   7
9   14
C-RIB:ASP-S2
3   0
4   1
5   6
6   16
7   28
8   41
9   55
5BU-P:THR-CA
3   0
4   0

5 0
6 0
7 0
8 0
9 0
FMU-MY:ASP-S2
3 0
4 0
5 0
6 0
7 0
8 0
9 0
G-R5:TRP-S2
3 0
4 0
5 0
6 0
7 1
8 3
9 6
U-RIB:MET-S2
3 0
4 0
5 0
6 1
7 2
8 4
9 6
I-RIB:TRP-S1
3 0
4 0
5 0
6 0
7 0
8 0
9 0
G-P:GLN-S2
3 0
4 0
5 6
6 13
7 23
8 32
9 43
DA-M5:VAL-S1
3 0
4 0
5 0
6 0
7 0
8 0
9 0
U-RIB:ILE-CA
3 0
4 0
5 0
6 0

7 4
8 9
9 15
QUO-M6:ARG-S1
3 0
4 0
5 0
6 0
7 0
8 0
9 0
G-R5:LYS-S1
3 0
4 0
5 1
6 6
7 17
8 36
9 61
U31-P:ASN-CA
3 0
4 0
5 0
6 0
7 0
8 0
9 0
A-P:ILE-S1
3 0
4 0
5 1
6 3
7 7
8 10
9 17
U-P:LYS-S2
3 0
4 2
5 7
6 15
7 23
8 32
9 42
A-R6:GLN-S1
3 0
4 0
5 1
6 3
7 7
8 12
9 20
U31-RIB:THR-S1
3 0
4 0
5 0
6 0
7 0
8 0

9 0
C-P:TRP-CA
3 0
4 0
5 0
6 0
7 2
8 4
9 6
A-RIB:SER-S1
3 0
4 0
5 4
6 10
7 18
8 27
9 39
FMU-P:ALA-S1
3 0
4 0
5 0
6 0
7 0
8 0
9 0
U-Y:TRP-CA
3 0
4 0
5 0
6 0
7 0
8 1
9 2
C-Y:THR-S1
3 0
4 0
5 1
6 3
7 8
8 15
9 24
GTP-M6:THR-S1
3 0
4 0
5 0
6 0
7 0
8 0
9 0
C-RIB:TYR-S1
3 0
4 0
5 0
6 1
7 3
8 6
9 12
GTP-RIB:SER-CA

3 0
4 0
5 0
6 0
7 0
8 0
9 0
FMU-RIB:CYS-CA
3 0
4 0
5 0
6 0
7 0
8 0
9 0
QUO-M6:PHE-S1
3 0
4 0
5 0
6 0
7 0
8 0
9 0
U-P:ASP-S2
3 0
4 0
5 4
6 10
7 18
8 27
9 36
DA-M5:ASP-S1
3 0
4 0
5 0
6 0
7 0
8 0
9 0
QUO-M6:ASP-CA
3 0
4 0
5 0
6 0
7 0
8 0
9 0
A-P:LYS-S1
3 0
4 1
5 5
6 14
7 26
8 41
9 58
C-P:THR-CA
3 0
4 0

5   1
6   5
7   11
8   20
9   30
A-P:ASP-S1
3   0
4   0
5   5
6   13
7   25
8   38
9   55
G-P:TRP-S1
3   0
4   0
5   0
6   1
7   3
8   6
9   9
A-RIB:GLU-S1
3   0
4   0
5   3
6   12
7   29
8   48
9   72
C31-MY:LEU-CA
3   0
4   0
5   0
6   0
7   0
8   0
9   0
FMU-MY:ASP-CA
3   0
4   0
5   0
6   0
7   0
8   0
9   0
G-RIB:GLN-S1
3   0
4   0
5   1
6   6
7   15
8   25
9   37
FHU-RIB:TYR-S1
3   0
4   0
5   0
6   0

7   0
8   0
9   0
C-P:TYR-S1
3   0
4   0
5   0
6   1
7   3
8   7
9   11
G-R5:ARG-CA
3   0
4   0
5   0
6   2
7   8
8   19
9   39
FHU-RIB:ARG-S1
3   0
4   0
5   0
6   0
7   0
8   0
9   0
DA-M6:HIS-S2
3   0
4   0
5   0
6   0
7   0
8   0
9   0
C31-P:MET-CA
3   0
4   0
5   0
6   0
7   0
8   0
9   0
H2U-MY:TRP-S2
3   0
4   0
5   0
6   0
7   0
8   0
9   0
DA-RIB:ARG-S1
3   0
4   0
5   0
6   0
7   0
8   0

9 0
A-R6:LEU-S2
3 0
4 0
5 1
6 2
7 6
8 10
9 17
C-Y:PRO-CA
3 0
4 0
5 0
6 1
7 5
8 11
9 19
QUO-M5:LYS-S2
3 0
4 0
5 0
6 0
7 0
8 0
9 0
A-RIB:VAL-CA
3 0
4 0
5 0
6 3
7 9
8 19
9 32
GTP-RIB:ALA-S1
3 0
4 0
5 0
6 0
7 0
8 0
9 0
G-R5:PRO-CA
3 0
4 0
5 0
6 2
7 7
8 16
9 29
A-R5:PHE-CA
3 0
4 0
5 0
6 0
7 1
8 3
9 7
G-R6:ARG-CA

3 0
4 0
5 0
6 3
7 10
8 21
9 39
U-P:LEU-S1
3 0
4 0
5 0
6 2
7 5
8 10
9 17
C-Y:HIS-S1
3 0
4 0
5 0
6 1
7 2
8 5
9 9
G-R5:PHE-S1
3 0
4 0
5 0
6 0
7 1
8 4
9 8
U-Y:LEU-S2
3 0
4 0
5 0
6 1
7 3
8 5
9 10
U-RIB:TYR-S1
3 0
4 0
5 0
6 0
7 2
8 4
9 8
C31-MY:PHE-CA
3 0
4 0
5 0
6 0
7 0
8 0
9 0
U-Y:ARG-CA
3 0
4 0

5 0
6 1
7 4
8 9
9 17
U-RIB:MET-CA
3 0
4 0
5 0
6 0
7 2
8 3
9 6
FMU-RIB:GLN-CA
3 0
4 0
5 0
6 0
7 0
8 0
9 0
IU-RIB:PRO-CA
3 0
4 0
5 0
6 0
7 0
8 0
9 0
IU-P:ILE-CA
3 0
4 0
5 0
6 0
7 0
8 0
9 0
QUO-M6:ASN-CA
3 0
4 0
5 0
6 0
7 0
8 0
9 0
U-Y:HIS-S2
3 0
4 0
5 0
6 1
7 3
8 6
9 9
U34-P:TYR-S1
3 0
4 0
5 0
6 0

7 0
8 0
9 0
U34-P:GLU-S1
3 0
4 0
5 0
6 0
7 0
8 0
9 0
H2U-MY:ARG-S1
3 0
4 0
5 0
6 0
7 0
8 0
9 0
FMU-MY:PRO-S1
3 0
4 0
5 0
6 0
7 0
8 0
9 0
C-Y:GLU-S2
3 0
4 0
5 3
6 11
7 24
8 41
9 62
C31-P:ALA-S1
3 0
4 0
5 0
6 0
7 0
8 0
9 0
C-Y:ILE-CA
3 0
4 0
5 0
6 0
7 2
8 5
9 10
U-Y:VAL-S1
3 0
4 0
5 0
6 2
7 4
8 7

9   12
U31-RIB:GLN-S2
3   0
4   0
5   0
6   0
7   0
8   0
9   0
U31-RIB:GLU-CA
3   0
4   0
5   0
6   0
7   0
8   0
9   0
FHU-P:PRO-CA
3   0
4   0
5   0
6   0
7   0
8   0
9   0
DA-M5:THR-S1
3   0
4   0
5   0
6   0
7   0
8   0
9   0
G-R5:ASP-CA
3   0
4   0
5   0
6   3
7   10
8   22
9   44
U31-P:ASP-CA
3   0
4   0
5   0
6   0
7   0
8   0
9   0
A-P:TYR-S1
3   0
4   0
5   0
6   1
7   3
8   6
9   11
C-Y:THR-CA

3 0
4 0
5 0
6 1
7 5
8 10
9 20
C31-MY:SER-CA
3 0
4 0
5 0
6 0
7 0
8 0
9 0
FMU-P:ASP-S1
3 0
4 0
5 0
6 0
7 0
8 0
9 0
QUO-RIB:ASN-CA
3 0
4 0
5 0
6 0
7 0
8 0
9 0
C-RIB:GLU-S2
3 0
4 0
5 10
6 24
7 43
8 62
9 82
U-RIB:GLN-S2
3 0
4 0
5 2
6 6
7 10
8 15
9 20
GTP-M6:ASN-CA
3 0
4 0
5 0
6 0
7 0
8 0
9 0
M2G-P:GLY-CA
3 0
4 0

5 0
6 0
7 0
8 0
9 0
G-R6:ASN-S2
3 0
4 0
5 2
6 7
7 14
8 23
9 34
G-R5:MET-CA
3 0
4 0
5 0
6 0
7 1
8 4
9 8
C31-RIB:THR-CA
3 0
4 0
5 0
6 0
7 0
8 0
9 0
QUO-P:LEU-S1
3 0
4 0
5 0
6 0
7 0
8 0
9 0
U-P:SER-S1
3 0
4 1
5 3
6 7
7 12
8 19
9 27
U34-P:TYR-CA
3 0
4 0
5 0
6 0
7 0
8 0
9 0
DA-M5:GLN-CA
3 0
4 0
5 0
6 0

7 0
8 0
9 0
DA-RIB:ARG-CA
3 0
4 0
5 0
6 0
7 0
8 0
9 0
DA-RIB:MET-S1
3 0
4 0
5 0
6 0
7 0
8 0
9 0
G-P:LEU-CA
3 0
4 0
5 1
6 5
7 13
8 26
9 46
FHU-MY:ARG-S1
3 0
4 0
5 0
6 0
7 0
8 0
9 0
U-Y:SER-S1
3 0
4 0
5 1
6 4
7 7
8 13
9 20
A-R5:LYS-S2
3 0
4 0
5 4
6 12
7 25
8 42
9 61
U-RIB:PRO-CA
3 0
4 0
5 0
6 3
7 7
8 13

9   19
U-RIB:LYS-S1
3   0
4   0
5   0
6   7
7   16
8   26
9   37
A-R6:GLU-S2
3   0
4   0
5   7
6   17
7   30
8   46
9   62
IU-MY:THR-S1
3   0
4   0
5   0
6   0
7   0
8   0
9   0
H2U-MY:GLU-S2
3   0
4   0
5   0
6   0
7   0
8   0
9   0
A-RIB:TRP-S2
3   0
4   0
5   0
6   1
7   2
8   4
9   7
C-RIB:ARG-CA
3   0
4   0
5   1
6   6
7   15
8   28
9   46
FMU-RIB:VAL-CA
3   0
4   0
5   0
6   0
7   0
8   0
9   0
QUO-M6:PHE-S2

3 0
4 0
5 0
6 0
7 0
8 0
9 0
H2U-P:TRP-CA
3 0
4 0
5 0
6 0
7 0
8 0
9 0
QUO-M6:ASP-S2
3 0
4 0
5 0
6 0
7 0
8 0
9 0
U-Y:THR-S1
3 0
4 0
5 1
6 3
7 6
8 11
9 17
U31-MY:TYR-S2
3 0
4 0
5 0
6 0
7 0
8 0
9 0
FHU-RIB:ARG-CA
3 0
4 0
5 0
6 0
7 0
8 0
9 0
A-R6:PRO-CA
3 0
4 0
5 1
6 3
7 8
8 14
9 23
A-P:GLN-CA
3 0
4 0

5 0
6 3
7 8
8 14
9 23
A-R5:THR-S1
3 0
4 0
5 1
6 4
7 9
8 17
9 26
U34-P:ARG-S1
3 0
4 0
5 0
6 0
7 0
8 0
9 0
C-P:HIS-CA
3 0
4 0
5 0
6 2
7 5
8 9
9 15
IU-MY:TYR-CA
3 0
4 0
5 0
6 0
7 0
8 0
9 0
G-P:LYS-S2
3 0
4 6
5 18
6 37
7 57
8 77
9 101
IU-RIB:LEU-S1
3 0
4 0
5 0
6 0
7 0
8 0
9 0
5BU-MY:ARG-S2
3 0
4 0
5 0
6 0

7 0
8 0
9 0
A-R6:TYR-S2
3 0
4 0
5 0
6 1
7 4
8 6
9 10
QUO-M5:ARG-S2
3 0
4 0
5 0
6 0
7 0
8 0
9 0
5BU-RIB:ARG-S2
3 0
4 0
5 0
6 0
7 0
8 0
9 0
C-P:ASP-S2
3 0
4 0
5 7
6 17
7 27
8 41
9 56
G-R6:MET-S1
3 0
4 0
5 0
6 1
7 3
8 5
9 8
GTP-M5:ASP-S1
3 0
4 0
5 0
6 0
7 0
8 0
9 0
FMU-RIB:GLN-S1
3 0
4 0
5 0
6 0
7 0
8 0

9 0
DA-RIB:TYR-CA
3 0
4 0
5 0
6 0
7 0
8 0
9 0
5BU-P:SER-S1
3 0
4 0
5 0
6 0
7 0
8 0
9 0
C31-RIB:ALA-S1
3 0
4 0
5 0
6 0
7 0
8 0
9 0
A-P:ILE-CA
3 0
4 0
5 0
6 2
7 6
8 12
9 20
C-P:ASN-S1
3 0
4 0
5 2
6 7
7 13
8 21
9 29
C31-P:TYR-CA
3 0
4 0
5 0
6 0
7 0
8 0
9 0
G-R6:ASN-CA
3 0
4 0
5 0
6 2
7 7
8 15
9 25
QUO-M6:ASN-S1

3 0
4 0
5 0
6 0
7 0
8 0
9 0
IU-RIB:ILE-CA
3 0
4 0
5 0
6 0
7 0
8 0
9 0
G-RIB:PHE-S2
3 0
4 0
5 0
6 2
7 6
8 10
9 17
A-P:HIS-S2
3 0
4 0
5 2
6 5
7 9
8 14
9 20
FHU-MY:THR-S1
3 0
4 0
5 0
6 0
7 0
8 0
9 0
C31-RIB:THR-S1
3 0
4 0
5 0
6 0
7 0
8 0
9 0
A-R6:TRP-S2
3 0
4 0
5 0
6 0
7 2
8 3
9 5
A-P:GLU-CA
3 0
4 0

5 2
6 0
7 23
8 41
9 64
G-R6:GLU-S2
3 0
4 0
5 6
6 18
7 38
8 60
9 85
A-R6:MET-S1
3 0
4 0
5 0
6 1
7 2
8 4
9 7
QUO-M6:GLU-S1
3 0
4 0
5 0
6 0
7 0
8 0
9 0
C31-RIB:ASP-S1
3 0
4 0
5 0
6 0
7 0
8 0
9 0
U-RIB:ALA-S1
3 0
4 0
5 2
6 6
7 11
8 17
9 25
A-R5:CYS-S1
3 0
4 0
5 0
6 0
7 0
8 1
9 1
U-Y:TRP-S2
3 0
4 0
5 0
6 0

7 0
8 0
9 3
A-P:ASP-CA
3 0
4 0
5 2
6 0
7 20
8 34
9 50
G-R5:ASP-S1
3 0
4 0
5 0
6 6
7 15
8 31
9 54
U31-MY:THR-CA
3 0
4 0
5 0
6 0
7 0
8 0
9 0
U34-RIB:SER-S1
3 0
4 0
5 0
6 0
7 0
8 0
9 0
A-R6:ASN-CA
3 0
4 0
5 0
6 2
7 6
8 13
9 20
U-P:GLN-CA
3 0
4 0
5 0
6 2
7 6
8 11
9 16
QUO-RIB:ASN-S1
3 0
4 0
5 0
6 0
7 0
8 0

9 0
U-Y:ASN-CA
3 0
4 0
5 0
6 1
7 4
8 8
9 13
FMU-RIB:ARG-S2
3 0
4 0
5 0
6 0
7 0
8 0
9 0
C31-RIB:TYR-S2
3 0
4 0
5 0
6 0
7 0
8 0
9 0
H2U-MY:GLU-CA
3 0
4 0
5 0
6 0
7 0
8 0
9 0
A-R6:GLY-CA
3 0
4 1
5 4
6 10
7 19
8 32
9 48
C31-MY:ASP-CA
3 0
4 0
5 0
6 0
7 0
8 0
9 0
QUO-RIB:PHE-S1
3 0
4 0
5 0
6 0
7 0
8 0
9 0
U-P:GLY-CA

3 0
4 1
5 4
6 10
7 17
8 27
9 39
IU-P:HIS-S2
3 0
4 0
5 0
6 0
7 0
8 0
9 0
C-P:THR-S1
3 0
4 0
5 4
6 8
7 14
8 22
9 32
C-P:ILE-CA
3 0
4 0
5 0
6 2
7 7
8 13
9 21
U31-MY:VAL-S1
3 0
4 0
5 0
6 0
7 0
8 0
9 0
A-RIB:GLU-CA
3 0
4 0
5 1
6 7
7 19
8 38
9 61
QUO-M6:LYS-CA
3 0
4 0
5 0
6 0
7 0
8 0
9 0
A-RIB:ARG-CA
3 0
4 0

5   1
6   5
7   15
8   28
9   47
C31-P:LEU-S2
3   0
4   0
5   0
6   0
7   0
8   0
9   0
FMU-MY:GLN-S2
3   0
4   0
5   0
6   0
7   0
8   0
9   0
C-Y:LYS-CA
3   0
4   0
5   0
6   2
7   6
8   15
9   29
G-R6:TRP-CA
3   0
4   0
5   0
6   0
7   1
8   3
9   5
FMU-P:PHE-S1
3   0
4   0
5   0
6   0
7   0
8   0
9   0
A-R5:CYS-CA
3   0
4   0
5   0
6   0
7   0
8   1
9   2
C-RIB:LEU-CA
3   0
4   0
5   0
6   2

7   7
8   16
9   30
C-RIB:TYR-S2
3   0
4   0
5   0
6   2
7   6
8   9
9   14
C-Y:GLN-CA
3   0
4   0
5   0
6   0
7   2
8   6
9   12
G-R6:ASP-S2
3   0
4   0
5   4
6   12
7   25
8   39
9   58
G-R6:GLN-S2
3   0
4   0
5   2
6   6
7   13
8   21
9   30
A-R5:ASN-S1
3   0
4   0
5   0
6   2
7   7
8   14
9   23
G-P:ARG-S1
3   0
4   0
5   7
6   19
7   37
8   59
9   87
G-P:THR-CA
3   0
4   0
5   2
6   8
7   18
8   31

9 47
U34-MY:VAL-CA
3 0
4 0
5 0
6 0
7 0
8 0
9 0
FHU-P:ALA-CA
3 0
4 0
5 0
6 0
7 0
8 0
9 0
G-P:HIS-S1
3 0
4 0
5 1
6 5
7 10
8 17
9 26
C-RIB:ARG-S2
3 0
4 1
5 6
6 18
7 35
8 52
9 70
U31-P:LEU-S2
3 0
4 0
5 0
6 0
7 0
8 0
9 0
A-P:SER-S1
3 0
4 1
5 5
6 11
7 18
8 27
9 40
U-P:ASN-CA
3 0
4 0
5 0
6 3
7 7
8 12
9 18
U-RIB:SER-CA

3 0
4 0
5 1
6 4
7 10
8 17
9 25
GTP-RIB:ASN-S1
3 0
4 0
5 0
6 0
7 0
8 0
9 0
A-R6:ARG-S1
3 0
4 0
5 2
6 7
7 15
8 27
9 41
QUO-M6:GLU-CA
3 0
4 0
5 0
6 0
7 0
8 0
9 0
A-P:LEU-S1
3 0
4 0
5 1
6 3
7 8
8 15
9 25
U31-MY:GLU-S1
3 0
4 0
5 0
6 0
7 0
8 0
9 0
C-RIB:MET-S1
3 0
4 0
5 0
6 1
7 3
8 6
9 10
IU-MY:ILE-S1
3 0
4 0

5 0
6 0
7 0
8 0
9 0
FHU-MY:PHE-S2
3 0
4 0
5 0
6 0
7 0
8 0
9 0
A-P:CYS-S1
3 0
4 0
5 0
6 0
7 0
8 0
9 3
G-R5:SER-CA
3 0
4 0
5 0
6 3
7 10
8 23
9 40
U31-P:ARG-S1
3 0
4 0
5 0
6 0
7 0
8 0
9 0
H2U-MY:ALA-CA
3 0
4 0
5 0
6 0
7 0
8 0
9 0
U31-RIB:MET-S2
3 0
4 0
5 0
6 0
7 0
8 0
9 0
C-P:LYS-S1
3 0
4 1
5 5
6 14

7 27
8 42
9 59
U-RIB:TRP-S1
3 0
4 0
5 0
6 0
7 1
8 2
9 3
U-P:PHE-S1
3 0
4 0
5 0
6 0
7 2
8 5
9 8
FHU-MY:LEU-CA
3 0
4 0
5 0
6 0
7 0
8 0
9 0
C-Y:CYS-S1
3 0
4 0
5 0
6 0
7 0
8 0
9 1
U-RIB:LYS-S2
3 0
4 1
5 6
6 14
7 23
8 33
9 43
A-RIB:MET-S2
3 0
4 0
5 1
6 2
7 5
8 7
9 10
A-R5:GLN-S1
3 0
4 0
5 0
6 1
7 5
8 11

9   19
U31-MY:ALA-S1
3   0
4   0
5   0
6   0
7   0
8   0
9   0
C-Y:ASP-S2
3   0
4   0
5   2
6   7
7   15
8   27
9   42
U-Y:TYR-CA
3   0
4   0
5   0
6   0
7   1
8   3
9   6
C-P:SER-S1
3   0
4   1
5   5
6   11
7   18
8   27
9   37
G-R6:CYS-S1
3   0
4   0
5   0
6   0
7   1
8   0
9   2
U31-MY:VAL-CA
3   0
4   0
5   0
6   0
7   0
8   0
9   0
QUO-M6:LYS-S1
3   0
4   0
5   0
6   0
7   0
8   0
9   0
A-P:ASN-S2

3 0
4 0
5 4
6 9
7 16
8 23
9 32
U31-RIB:GLN-S1
3 0
4 0
5 0
6 0
7 0
8 0
9 0
IU-RIB:HIS-S2
3 0
4 0
5 0
6 0
7 0
8 0
9 0
FHU-RIB:ILE-CA
3 0
4 0
5 0
6 0
7 0
8 0
9 0
U-P:TYR-S1
3 0
4 0
5 0
6 1
7 2
8 5
9 8
FMU-P:GLN-CA
3 0
4 0
5 0
6 0
7 0
8 0
9 0
C-Y:LYS-S1
3 0
4 0
5 1
6 4
7 12
8 23
9 40
C-RIB:PHE-CA
3 0
4 0

5   0
6   1
7   4
8   8
9   14
H2U-P:GLU-S2
3   0
4   0
5   0
6   0
7   0
8   0
9   0
A-RIB:VAL-S1
3   0
4   0
5   1
6   5
7   11
8   18
9   29
H2U-RIB:LEU-S2
3   0
4   0
5   0
6   0
7   0
8   0
9   0
G-P:ARG-CA
3   0
4   0
5   3
6   12
7   27
8   48
9   74
A-RIB:TYR-CA
3   0
4   0
5   0
6   1
7   3
8   6
9   12
U-P:VAL-S1
3   0
4   0
5   0
6   4
7   8
8   13
9   20
5BU-P:ILE-S1
3   0
4   0
5   0
6   0

7   0
8   0
9   0
U-RIB:PHE-S1
3   0
4   0
5   0
6   0
7   2
8   4
9   7
G-RIB:MET-CA
3   0
4   0
5   0
6   0
7   5
8   10
9   15
IU-RIB:HIS-S1
3   0
4   0
5   0
6   0
7   0
8   0
9   0
C-Y:TRP-S2
3   0
4   0
5   0
6   0
7   1
8   2
9   4
A-R5:VAL-S1
3   0
4   0
5   0
6   2
7   5
8   11
9   20
A-P:MET-S1
3   0
4   0
5   0
6   2
7   4
8   6
9   10
A-RIB:ARG-S1
3   0
4   0
5   3
6   10
7   21
8   38

9 58
U-P:TYR-CA
3 0
4 0
5 0
6 1
7 2
8 5
9 8
U34-P:SER-CA
3 0
4 0
5 0
6 0
7 0
8 0
9 0
FMU-RIB:ARG-CA
3 0
4 0
5 0
6 0
7 0
8 0
9 0
FMU-RIB:ASN-S1
3 0
4 0
5 0
6 0
7 0
8 0
9 0
A-R5:THR-CA
3 0
4 0
5 0
6 1
7 5
8 12
9 22
A-P:CYS-CA
3 0
4 0
5 0
6 0
7 1
8 2
9 3
DA-M5:ASN-S1
3 0
4 0
5 0
6 0
7 0
8 0
9 0
C-RIB:LEU-S1

3 0
4 0
5 0
6 3
7 7
8 15
9 26
G-R5:PRO-S1
3 0
4 0
5 1
6 4
7 10
8 20
9 34
U-P:ASP-CA
3 0
4 0
5 1
6 5
7 12
8 21
9 32
FHU-P:LYS-CA
3 0
4 0
5 0
6 0
7 0
8 0
9 0
5BU-RIB:ILE-S1
3 0
4 0
5 0
6 0
7 0
8 0
9 0
G-P:GLU-S2
3 0
4 0
5 18
6 39
7 63
8 89
9 119
FHU-RIB:LEU-S1
3 0
4 0
5 0
6 0
7 0
8 0
9 0
QUO-RIB:ASP-S1
3 0
4 0

5 0
6 0
7 0
8 0
9 0
C-P:TYR-S2
3 0
4 0
5 0
6 2
7 6
8 9
9 14
A-R5:ASN-CA
3 0
4 0
5 0
6 1
7 4
8 10
9 20
A-P:GLN-S2
3 0
4 1
5 3
6 9
7 14
8 21
9 29
U-RIB:SER-S1
3 0
4 0
5 3
6 7
7 12
8 19
9 26
A-P:ALA-S1
3 0
4 2
5 5
6 11
7 18
8 28
9 43
FHU-MY:SER-S1
3 0
4 0
5 0
6 0
7 0
8 0
9 0
C31-P:THR-CA
3 0
4 0
5 0
6 0

7 0
8 0
9 0
A-RIB:ASN-S1
3 0
4 0
5 1
6 6
7 12
8 21
9 30
A-R6:GLN-CA
3 0
4 0
5 0
6 1
7 5
8 9
9 16
H2U-P:GLU-CA
3 0
4 0
5 0
6 0
7 0
8 0
9 0
G-R6:ILE-S1
3 0
4 0
5 0
6 0
7 5
8 9
9 15
A-P:VAL-CA
3 0
4 0
5 1
6 4
7 11
8 20
9 34
U-P:PHE-CA
3 0
4 0
5 0
6 1
7 3
8 5
9 9
A-RIB:MET-CA
3 0
4 0
5 0
6 1
7 3
8 6

9   10
G-R5:LYS-S2
3   0
4   1
5   6
6   16
7   33
8   56
9   82
A-R6:PHE-S1
3   0
4   0
5   0
6   0
7   2
8   4
9   7
DA-M5:GLU-S2
3   0
4   0
5   0
6   0
7   0
8   0
9   0
U-P:LYS-S1
3   0
4   0
5   3
6   9
7   17
8   26
9   38
G-RIB:LEU-S1
3   0
4   0
5   1
6   4
7   10
8   21
9   36
U34-RIB:ASN-S1
3   0
4   0
5   0
6   0
7   0
8   0
9   0
C31-P:ASP-CA
3   0
4   0
5   0
6   0
7   0
8   0
9   0
U34-P:SER-S1

3 0
4 0
5 0
6 0
7 0
8 0
9 0
FMU-MY:ARG-S1
3 0
4 0
5 0
6 0
7 0
8 0
9 0
C-P:TRP-S2
3 0
4 0
5 0
6 1
7 2
8 4
9 6
C31-RIB:ASP-S2
3 0
4 0
5 0
6 0
7 0
8 0
9 0
GTP-M5:ALA-CA
3 0
4 0
5 0
6 0
7 0
8 0
9 0
FHU-MY:ALA-CA
3 0
4 0
5 0
6 0
7 0
8 0
9 0
A-R6:PHE-S2
3 0
4 0
5 0
6 1
7 3
8 4
9 7
U31-P:ALA-S1
3 0
4 0

5 0
6 0
7 0
8 0
9 0
U34-MY:SER-S1
3 0
4 0
5 0
6 0
7 0
8 0
9 0
5BU-RIB:SER-S1
3 0
4 0
5 0
6 0
7 0
8 0
9 0
H2U-MY:TRP-CA
3 0
4 0
5 0
6 0
7 0
8 0
9 0
U-Y:TYR-S1
3 0
4 0
5 0
6 0
7 1
8 3
9 5
QUO-RIB:LYS-S1
3 0
4 0
5 0
6 0
7 0
8 0
9 0
H2U-MY:GLU-S1
3 0
4 0
5 0
6 0
7 0
8 0
9 0
A-R6:ARG-CA
3 0
4 0
5 1
6 4

7 10
8 20
9 33
C31-RIB:ASP-CA
3 0
4 0
5 0
6 0
7 0
8 0
9 0
U31-MY:MET-S1
3 0
4 0
5 0
6 0
7 0
8 0
9 0
DA-M6:GLU-S2
3 0
4 0
5 0
6 0
7 0
8 0
9 0
FHU-RIB:LYS-S1
3 0
4 0
5 0
6 0
7 0
8 0
9 0
QUO-M6:ASP-S1
3 0
4 0
5 0
6 0
7 0
8 0
9 0
A-R6:PHE-CA
3 0
4 0
5 0
6 0
7 2
8 4
9 8
G-R5:TRP-S1
3 0
4 0
5 0
6 0
7 1
8 3

9 5
U31-P:ASP-S1
3 0
4 0
5 0
6 0
7 0
8 0
9 0
G-P:LYS-S1
3 0
4 2
5 8
6 22
7 43
8 65
9 91
H2U-MY:TRP-S1
3 0
4 0
5 0
6 0
7 0
8 0
9 0
QUO-M5:LEU-S2
3 0
4 0
5 0
6 0
7 0
8 0
9 0
C-Y:TYR-CA
3 0
4 0
5 0
6 0
7 1
8 3
9 6
FHU-P:LEU-S2
3 0
4 0
5 0
6 0
7 0
8 0
9 0
A-R6:ALA-CA
3 0
4 0
5 2
6 6
7 12
8 20
9 32
U-P:PRO-CA

3 0
4 0
5 1
6 4
7 8
8 13
9 20
A-P:ARG-CA
3 0
4 0
5 1
6 7
7 16
8 30
9 48
A-P:TYR-S2
3 0
4 0
5 0
6 2
7 5
8 9
9 13
G-R6:GLY-CA
3 0
4 0
5 3
6 11
7 23
8 40
9 62
QUO-M5:ARG-S1
3 0
4 0
5 0
6 0
7 0
8 0
9 0
U-RIB:CYS-CA
3 0
4 0
5 0
6 0
7 0
8 1
9 2
U-P:ILE-S1
3 0
4 0
5 1
6 2
7 5
8 8
9 13
H2U-P:PHE-S1
3 0
4 0

5 0
6 0
7 0
8 0
9 0
G-R6:ASN-S1
3 0
4 0
5 1
6 4
7 10
8 19
9 29
H2U-RIB:ARG-S2
3 0
4 0
5 0
6 0
7 0
8 0
9 0
C-RIB:TYR-CA
3 0
4 0
5 0
6 1
7 3
8 7
9 12
G-RIB:ARG-S1
3 0
4 0
5 4
6 14
7 31
8 54
9 82
FHU-P:THR-S1
3 0
4 0
5 0
6 0
7 0
8 0
9 0
U-P:VAL-CA
3 0
4 0
5 0
6 3
7 8
8 14
9 23
DA-RIB:HIS-S2
3 0
4 0
5 0
6 0

7 0
8 0
9 0
C-Y:ASP-CA
3 0
4 0
5 0
6 2
7 7
8 16
9 30
A-R6:TRP-S1
3 0
4 0
5 0
6 0
7 1
8 0
9 5
GTP-RIB:GLY-CA
3 0
4 0
5 0
6 0
7 0
8 0
9 0
U31-MY:ASP-S1
3 0
4 0
5 0
6 0
7 0
8 0
9 0
FMU-MY:MET-CA
3 0
4 0
5 0
6 0
7 0
8 0
9 0
FHU-P:ASP-S2
3 0
4 0
5 0
6 0
7 0
8 0
9 0
U-P:ARG-CA
3 0
4 0
5 1
6 5
7 11
8 19

9   30
A-P:ASN-CA
3   0
4   0
5   1
6   4
7   11
8   19
9   29
DA-RIB:GLN-S2
3   0
4   0
5   0
6   0
7   0
8   0
9   0
U31-P:HIS-S1
3   0
4   0
5   0
6   0
7   0
8   0
9   0
QUO-P:SER-S1
3   0
4   0
5   0
6   0
7   0
8   0
9   0
A-R5:TYR-S2
3   0
4   0
5   0
6   1
7   2
8   5
9   10
C-Y:TYR-S2
3   0
4   0
5   0
6   1
7   2
8   5
9   8
DA-M5:ASN-CA
3   0
4   0
5   0
6   0
7   0
8   0
9   0
DA-RIB:VAL-S1

3 0
4 0
5 0
6 0
7 0
8 0
9 0
C-RIB:ALA-CA
3 0
4 0
5 2
6 7
7 16
8 26
9 41
C-P:PRO-CA
3 0
4 0
5 1
6 6
7 12
8 21
9 31
U-Y:ILE-CA
3 0
4 0
5 0
6 0
7 2
8 6
9 10
FHU-P:ARG-S1
3 0
4 0
5 0
6 0
7 0
8 0
9 0
U-Y:ALA-CA
3 0
4 0
5 0
6 2
7 5
8 10
9 17
A-RIB:HIS-S1
3 0
4 0
5 0
6 2
7 5
8 10
9 16
G-P:CYS-S1
3 0
4 0

5 0
6 0
7 1
8 0
9 5
U-Y:GLU-S2
3 0
4 0
5 3
6 9
7 17
8 28
9 41
A-R5:PRO-CA
3 0
4 0
5 0
6 2
7 5
8 12
9 22
A-RIB:CYS-CA
3 0
4 0
5 0
6 0
7 1
8 2
9 3
U34-P:HIS-S1
3 0
4 0
5 0
6 0
7 0
8 0
9 0
C31-P:THR-S1
3 0
4 0
5 0
6 0
7 0
8 0
9 0
GTP-M6:SER-CA
3 0
4 0
5 0
6 0
7 0
8 0
9 0
G-R5:GLN-S2
3 0
4 0
5 1
6 5

7   11
8   22
9   34
H2U-MY:LEU-S1
3   0
4   0
5   0
6   0
7   0
8   0
9   0
FMU-MY:PHE-S2
3   0
4   0
5   0
6   0
7   0
8   0
9   0
G-R6:PHE-S2
3   0
4   0
5   0
6   1
7   3
8   5
9   9
OMC-P:LYS-S1
3   0
4   0
5   0
6   0
7   0
8   0
9   0
C-RIB:MET-S2
3   0
4   0
5   1
6   2
7   4
8   7
9   10
A-RIB:GLY-CA
3   0
4   1
5   5
6   14
7   28
8   42
9   60
G-R5:TRP-CA
3   0
4   0
5   0
6   0
7   1
8   2

9   5
A-RIB:ASP-S2
3   0
4   1
5   6
6   15
7   28
8   42
9   59
5BU-MY:PRO-S1
3   0
4   0
5   0
6   0
7   0
8   0
9   0
C-P:GLU-CA
3   0
4   0
5   2
6   9
7   22
8   41
9   63
G-R5:ASP-S2
3   0
4   0
5   3
6   10
7   21
8   40
9   63
QUO-RIB:ASN-S2
3   0
4   0
5   0
6   0
7   0
8   0
9   0
FMU-MY:GLU-S2
3   0
4   0
5   0
6   0
7   0
8   0
9   0
QUO-RIB:LEU-S1
3   0
4   0
5   0
6   0
7   0
8   0
9   0
H2U-RIB:ARG-S1

3 0
4 0
5 0
6 0
7 0
8 0
9 0
M2G-P:GLU-S2
3 0
4 0
5 0
6 0
7 0
8 0
9 0
G-RIB:LEU-CA
3 0
4 0
5 0
6 3
7 9
8 22
9 40
A-P:THR-CA
3 0
4 0
5 1
6 5
7 11
8 19
9 30
U-Y:ASP-S1
3 0
4 0
5 1
6 3
7 8
8 15
9 25
C31-RIB:TYR-S1
3 0
4 0
5 0
6 0
7 0
8 0
9 0
C-RIB:HIS-CA
3 0
4 0
5 0
6 1
7 4
8 8
9 14
G-RIB:ASP-S1
3 0
4 0

5 4
6 15
7 32
8 51
9 75
U31-MY:PHE-S1
3 0
4 0
5 0
6 0
7 0
8 0
9 0
U-Y:ILE-S1
3 0
4 0
5 0
6 1
7 3
8 5
9 8
U-RIB:GLU-S1
3 0
4 0
5 2
6 8
7 20
8 32
9 46
U-P:HIS-S1
3 0
4 0
5 0
6 2
7 4
8 7
9 11
A-P:PRO-S1
3 0
4 0
5 3
6 8
7 14
8 23
9 32
A-R5:MET-CA
3 0
4 0
5 0
6 0
7 1
8 3
9 7
H2U-P:PRO-S1
3 0
4 0
5 0
6 0

7   0
8   0
9   0
A-P:HIS-CA
3   0
4   0
5   0
6   2
7   5
8   10
9   16
H2U-MY:ARG-CA
3   0
4   0
5   0
6   0
7   0
8   0
9   0
G-R6:PRO-CA
3   0
4   0
5   0
6   3
7   8
8   17
9   28
U31-RIB:ASP-S2
3   0
4   0
5   0
6   0
7   0
8   0
9   0
C-Y:MET-CA
3   0
4   0
5   0
6   0
7   1
8   3
9   5
A-R6:LEU-CA
3   0
4   0
5   0
6   1
7   4
8   9
9   19
FMU-MY:CYS-S1
3   0
4   0
5   0
6   0
7   0
8   0

9 0
C-Y:LYS-S2
3 0
4 1
5 4
6 11
7 22
8 37
9 54
A-R6:HIS-CA
3 0
4 0
5 0
6 1
7 3
8 6
9 11
A-R6:ASP-CA
3 0
4 0
5 1
6 5
7 13
8 23
9 37
C-P:ASP-CA
3 0
4 0
5 2
6 8
7 19
8 33
9 49
C-RIB:ALA-S1
3 0
4 1
5 5
6 10
7 18
8 27
9 40
A-RIB:HIS-S2
3 0
4 0
5 1
6 4
7 9
8 14
9 19
FHU-MY:TYR-CA
3 0
4 0
5 0
6 0
7 0
8 0
9 0
IU-P:SER-CA

3 0
4 0
5 0
6 0
7 0
8 0
9 0
U34-RIB:GLY-CA
3 0
4 0
5 0
6 0
7 0
8 0
9 0
A-RIB:THR-CA
3 0
4 0
5 0
6 3
7 10
8 19
9 31
C31-MY:GLU-S2
3 0
4 0
5 0
6 0
7 0
8 0
9 0
C-RIB:LYS-CA
3 0
4 0
5 1
6 6
7 17
8 31
9 49
G-R6:SER-S1
3 0
4 0
5 3
6 8
7 17
8 29
9 43
C31-P:ASN-S2
3 0
4 0
5 0
6 0
7 0
8 0
9 0
C-Y:ARG-CA
3 0
4 0

5 0
6 2
7 6
8 13
9 25
U-RIB:PRO-S1
3 0
4 0
5 1
6 5
7 9
8 14
9 21
IU-RIB:LEU-CA
3 0
4 0
5 0
6 0
7 0
8 0
9 0
U-RIB:HIS-CA
3 0
4 0
5 0
6 1
7 3
8 6
9 10
C-RIB:GLU-CA
3 0
4 0
5 1
6 8
7 21
8 39
9 63
A-P:PHE-CA
3 0
4 0
5 0
6 1
7 4
8 8
9 14
G-RIB:LYS-S1
3 0
4 0
5 5
6 17
7 35
8 60
9 88
U-P:ARG-S1
3 0
4 0
5 3
6 8

7 16
8 25
9 37
C-P:GLY-CA
3 0
4 1
5 6
6 15
7 26
8 40
9 58
C31-MY:GLN-S1
3 0
4 0
5 0
6 0
7 0
8 0
9 0
C31-MY:PHE-S2
3 0
4 0
5 0
6 0
7 0
8 0
9 0
H2U-MY:PRO-CA
3 0
4 0
5 0
6 0
7 0
8 0
9 0
FHU-MY:PRO-S1
3 0
4 0
5 0
6 0
7 0
8 0
9 0
A-RIB:CYS-S1
3 0
4 0
5 0
6 0
7 0
8 1
9 3
H2U-RIB:GLU-CA
3 0
4 0
5 0
6 0
7 0
8 0

9 0
IU-MY:VAL-S1
3 0
4 0
5 0
6 0
7 0
8 0
9 0
G-P:VAL-S1
3 0
4 0
5 4
6 10
7 18
8 30
9 46
DA-RIB:ASP-CA
3 0
4 0
5 0
6 0
7 0
8 0
9 0
A-P:PRO-CA
3 0
4 0
5 1
6 6
7 12
8 21
9 31
G-R5:SER-S1
3 0
4 0
5 2
6 6
7 16
8 30
9 47
FHU-P:VAL-CA
3 0
4 0
5 0
6 0
7 0
8 0
9 0
U34-P:HIS-S2
3 0
4 0
5 0
6 0
7 0
8 0
9 0
U-P:MET-S2

3 0
4 0
5 0
6 1
7 3
8 4
9 6
G-RIB:LYS-S2
3 0
4 0
5 14
6 31
7 53
8 76
9 101
FHU-RIB:LYS-S2
3 0
4 0
5 0
6 0
7 0
8 0
9 0
H2U-P:GLU-S1
3 0
4 0
5 0
6 0
7 0
8 0
9 0
G-R6:PRO-S1
3 0
4 0
5 1
6 5
7 12
8 20
9 32
U-RIB:GLU-CA
3 0
4 0
5 1
6 5
7 13
8 26
9 41
U-Y:ASP-CA
3 0
4 0
5 0
6 2
7 6
8 12
9 21
DA-RIB:TYR-S1
3 0
4 0

5 0
6 0
7 0
8 0
9 0
C-Y:PHE-S2
3 0
4 0
5 0
6 1
7 1
8 3
9 6
FMU-MY:MET-S1
3 0
4 0
5 0
6 0
7 0
8 0
9 0
C-P:PHE-S1
3 0
4 0
5 0
6 1
7 4
8 8
9 13
U-Y:PRO-S1
3 0
4 0
5 1
6 2
7 5
8 9
9 14
U-Y:GLU-CA
3 0
4 0
5 0
6 2
7 7
8 15
9 27
A-R5:TRP-CA
3 0
4 0
5 0
6 0
7 0
8 2
9 4
FHU-MY:CYS-S1
3 0
4 0
5 0
6 0

7 0
8 0
9 0
U-P:CYS-S1
3 0
4 0
5 0
6 0
7 0
8 1
9 1
IU-P:LEU-CA
3 0
4 0
5 0
6 0
7 0
8 0
9 0
U31-P:ASN-S1
3 0
4 0
5 0
6 0
7 0
8 0
9 0
H2U-MY:PHE-CA
3 0
4 0
5 0
6 0
7 0
8 0
9 0
G-R5:VAL-S1
3 0
4 0
5 1
6 3
7 7
8 16
9 28
DA-M6:LYS-CA
3 0
4 0
5 0
6 0
7 0
8 0
9 0
A-R5:MET-S2
3 0
4 0
5 0
6 1
7 3
8 4

9 7
U31-RIB:GLN-CA
3 0
4 0
5 0
6 0
7 0
8 0
9 0
IU-P:LYS-S1
3 0
4 0
5 0
6 0
7 0
8 0
9 0
U-P:GLN-S1
3 0
4 0
5 1
6 4
7 8
8 12
9 18
A-R5:LYS-S1
3 0
4 0
5 1
6 5
7 13
8 27
9 46
QUO-M6:LEU-S1
3 0
4 0
5 0
6 0
7 0
8 0
9 0
C-P:LYS-CA
3 0
4 0
5 2
6 8
7 19
8 34
9 52
I-P:TRP-S2
3 0
4 0
5 0
6 0
7 0
8 0
9 0
G-P:LYS-CA

3 0
4 0
5 4
6 14
7 31
8 53
9 82
FMU-P:ARG-S2
3 0
4 0
5 0
6 0
7 0
8 0
9 0
G-RIB:LYS-CA
3 0
4 0
5 2
6 10
7 25
8 45
9 74
G-RIB:MET-S1
3 0
4 0
5 0
6 2
7 5
8 9
9 15
FMU-MY:ILE-CA
3 0
4 0
5 0
6 0
7 0
8 0
9 0
DA-RIB:GLN-CA
3 0
4 0
5 0
6 0
7 0
8 0
9 0
FHU-MY:TYR-S1
3 0
4 0
5 0
6 0
7 0
8 0
9 0
OMC-MY:LYS-S2
3 0
4 0

5   0
6   0
7   0
8   0
9   0
G-P:TYR-S2
3   0
4   0
5   1
6   4
7   8
8   14
9   21
A-P:ASP-S2
3   0
4   0
5   8
6   17
7   29
8   43
9   58
U-Y:THR-CA
3   0
4   0
5   0
6   1
7   4
8   8
9   14
FMU-MY:VAL-CA
3   0
4   0
5   0
6   0
7   0
8   0
9   0
C31-P:GLU-S2
3   0
4   0
5   0
6   0
7   0
8   0
9   0
QUO-RIB:LYS-S2
3   0
4   0
5   0
6   0
7   0
8   0
9   0
A-R5:GLU-S2
3   0
4   0
5   4
6   12

7 27
8 45
9 68
U31-RIB:TYR-S2
3 0
4 0
5 0
6 0
7 0
8 0
9 0
U31-MY:GLN-S1
3 0
4 0
5 0
6 0
7 0
8 0
9 0
G-P:SER-S1
3 0
4 2
5 8
6 17
7 28
8 42
9 58
C-RIB:ARG-S1
3 0
4 0
5 3
6 10
7 22
8 37
9 56
DA-M5:MET-S2
3 0
4 0
5 0
6 0
7 0
8 0
9 0
C31-RIB:LEU-S1
3 0
4 0
5 0
6 0
7 0
8 0
9 0
C-RIB:GLU-S1
3 0
4 0
5 4
6 14
7 31
8 51

9 73
U-RIB:HIS-S1
3 0
4 0
5 0
6 1
7 4
8 7
9 11
G-R5:GLN-S1
3 0
4 0
5 0
6 2
7 6
8 14
9 25
IU-MY:GLU-S2
3 0
4 0
5 0
6 0
7 0
8 0
9 0
IU-MY:ARG-S2
3 0
4 0
5 0
6 0
7 0
8 0
9 0
A-R6:PRO-S1
3 0
4 0
5 2
6 5
7 11
8 17
9 24
C31-P:ALA-CA
3 0
4 0
5 0
6 0
7 0
8 0
9 0
IU-RIB:ALA-CA
3 0
4 0
5 0
6 0
7 0
8 0
9 0
A-P:THR-S1

3 0
4 1
5 4
6 9
7 15
8 23
9 33
A-R5:ARG-S1
3 0
4 0
5 1
6 4
7 11
8 24
9 42
GTP-RIB:ASN-S2
3 0
4 0
5 0
6 0
7 0
8 0
9 0
DA-M5:GLU-S1
3 0
4 0
5 0
6 0
7 0
8 0
9 0
U31-P:LEU-S1
3 0
4 0
5 0
6 0
7 0
8 0
9 0
U-RIB:VAL-S1
3 0
4 0
5 1
6 3
7 7
8 12
9 19
C-RIB:VAL-CA
3 0
4 0
5 0
6 3
7 10
8 19
9 31
C-RIB:ILE-S1
3 0
4 0

5   1
6   3
7   6
8   11
9   18
A-R6:CYS-S1
3   0
4   0
5   0
6   0
7   0
8   1
9   0
DA-M5:HIS-S2
3   0
4   0
5   0
6   0
7   0
8   0
9   0
FHU-P:TYR-CA
3   0
4   0
5   0
6   0
7   0
8   0
9   0
G-R6:LEU-CA
3   0
4   0
5   0
6   1
7   4
8   10
9   20
A-R5:ASP-S1
3   0
4   0
5   1
6   5
7   13
8   26
9   42
H2U-P:PRO-CA
3   0
4   0
5   0
6   0
7   0
8   0
9   0
DA-M6:LEU-S1
3   0
4   0
5   0
6   0

7 0
8 0
9 0
GTP-M5:SER-S1
3 0
4 0
5 0
6 0
7 0
8 0
9 0
C-Y:SER-S1
3 0
4 0
5 1
6 4
7 10
8 19
9 29
C-Y:MET-S1
3 0
4 0
5 0
6 0
7 1
8 3
9 6
G-R5:THR-CA
3 0
4 0
5 0
6 2
7 6
8 16
9 30
A-P:ARG-S1
3 0
4 0
5 4
6 12
7 24
8 39
9 59
A-R5:LYS-CA
3 0
4 0
5 0
6 2
7 7
8 19
9 36
U-P:GLU-S2
3 0
4 0
5 0
6 16
7 27
8 38

9 51
C31-P:SER-S1
3 0
4 0
5 0
6 0
7 0
8 0
9 0
DA-M6:ASN-CA
3 0
4 0
5 0
6 0
7 0
8 0
9 0
U-Y:PHE-S2
3 0
4 0
5 0
6 0
7 1
8 3
9 4
FHU-P:SER-CA
3 0
4 0
5 0
6 0
7 0
8 0
9 0
G-RIB:VAL-S1
3 0
4 0
5 2
6 8
7 17
8 27
9 43
C-Y:TYR-S1
3 0
4 0
5 0
6 0
7 1
8 3
9 6
OMC-P:LYS-CA
3 0
4 0
5 0
6 0
7 0
8 0
9 0
G-RIB:THR-S1

3 0
4 0
5 4
6 11
7 21
8 33
9 49
G-R5:CYS-CA
3 0
4 0
5 0
6 0
7 0
8 1
9 3
FMU-RIB:PHE-CA
3 0
4 0
5 0
6 0
7 0
8 0
9 0
U34-MY:PHE-CA
3 0
4 0
5 0
6 0
7 0
8 0
9 0
DA-M6:ASN-S1
3 0
4 0
5 0
6 0
7 0
8 0
9 0
C-RIB:THR-S1
3 0
4 0
5 3
6 8
7 15
8 22
9 33
FHU-P:LEU-S1
3 0
4 0
5 0
6 0
7 0
8 0
9 0
G-R6:LYS-CA
3 0
4 0

5 0
6 4
7 12
8 25
9 44
H2U-MY:THR-CA
3 0
4 0
5 0
6 0
7 0
8 0
9 0
U-Y:LYS-S2
3 0
4 0
5 0
6 9
7 16
8 25
9 36
FMU-MY:PHE-CA
3 0
4 0
5 0
6 0
7 0
8 0
9 0
C-P:VAL-CA
3 0
4 0
5 1
6 4
7 10
8 20
9 32
DA-M6:GLU-S1
3 0
4 0
5 0
6 0
7 0
8 0
9 0
C-P:PHE-CA
3 0
4 0
5 0
6 0
7 4
8 9
9 15
H2U-P:ARG-S2
3 0
4 0
5 0
6 0

7 0
8 0
9 0
U-Y:GLN-S2
3 0
4 0
5 1
6 3
7 7
8 11
9 16
G-P:ILE-CA
3 0
4 0
5 1
6 0
7 11
8 20
9 33
C-RIB:ILE-CA
3 0
4 0
5 0
6 1
7 6
8 12
9 21
DA-M5:TYR-CA
3 0
4 0
5 0
6 0
7 0
8 0
9 0
A-R5:ALA-CA
3 0
4 0
5 0
6 3
7 9
8 18
9 30
FHU-P:TYR-S1
3 0
4 0
5 0
6 0
7 0
8 0
9 0
IU-P:LYS-CA
3 0
4 0
5 0
6 0
7 0
8 0

9 0
IU-P:ALA-CA
3 0
4 0
5 0
6 0
7 0
8 0
9 0
H2U-MY:ASN-S1
3 0
4 0
5 0
6 0
7 0
8 0
9 0
U-P:ASN-S1
3 0
4 0
5 1
6 4
7 8
8 14
9 19
C31-P:MET-S1
3 0
4 0
5 0
6 0
7 0
8 0
9 0
U-Y:LEU-S1
3 0
4 0
5 0
6 1
7 2
8 5
9 9
U31-MY:PHE-S2
3 0
4 0
5 0
6 0
7 0
8 0
9 0
IU-P:ILE-S1
3 0
4 0
5 0
6 0
7 0
8 0
9 0
G-RIB:TRP-S1

3 0
4 0
5 0
6 1
7 3
8 5
9 9
A-R5:LEU-S1
3 0
4 0
5 0
6 1
7 3
8 7
9 15
G-P:ASN-S1
3 0
4 0
5 4
6 10
7 19
8 30
9 42
FMU-MY:VAL-S1
3 0
4 0
5 0
6 0
7 0
8 0
9 0
A-R6:TYR-CA
3 0
4 0
5 0
6 0
7 2
8 5
9 9
G-R6:GLU-S1
3 0
4 0
5 0
6 8
7 23
8 44
9 70
GTP-M5:GLY-CA
3 0
4 0
5 0
6 0
7 0
8 0
9 0
IU-RIB:ARG-S1
3 0
4 0

5 0
6 0
7 0
8 0
9 0
G-R5:HIS-CA
3 0
4 0
5 0
6 1
7 3
8 7
9 14
H2U-MY:PRO-S1
3 0
4 0
5 0
6 0
7 0
8 0
9 0
C31-MY:LEU-S2
3 0
4 0
5 0
6 0
7 0
8 0
9 0
FHU-RIB:ASP-S1
3 0
4 0
5 0
6 0
7 0
8 0
9 0
U-P:TRP-S2
3 0
4 0
5 0
6 0
7 1
8 3
9 4
C31-MY:GLU-S1
3 0
4 0
5 0
6 0
7 0
8 0
9 0
DA-M6:LYS-S2
3 0
4 0
5 0
6 0

7 0
8 0
9 0
5BU-P:ARG-CA
3 0
4 0
5 0
6 0
7 0
8 0
9 0
FMU-MY:GLN-S1
3 0
4 0
5 0
6 0
7 0
8 0
9 0
A-RIB:PRO-CA
3 0
4 0
5 1
6 5
7 12
8 20
9 32
QUO-RIB:LEU-S2
3 0
4 0
5 0
6 0
7 0
8 0
9 0
A-P:MET-CA
3 0
4 0
5 0
6 1
7 3
8 6
9 10
A-RIB:LYS-CA
3 0
4 0
5 1
6 6
7 17
8 31
9 49
U31-RIB:ASN-S1
3 0
4 0
5 0
6 0
7 0
8 0

9 0
FMU-P:PHE-CA
3 0
4 0
5 0
6 0
7 0
8 0
9 0
C31-RIB:PHE-S2
3 0
4 0
5 0
6 0
7 0
8 0
9 0
A-RIB:ARG-S2
3 0
4 0
5 6
6 19
7 35
8 54
9 73
U-RIB:LEU-CA
3 0
4 0
5 0
6 1
7 4
8 9
9 17
A-R5:ASP-CA
3 0
4 0
5 0
6 2
7 9
8 20
9 35
G-RIB:ASP-S2
3 0
4 0
5 9
6 21
7 39
8 58
9 82
A-R6:ASN-S2
3 0
4 0
5 2
6 6
7 12
8 18
9 25
A-R6:LYS-CA

3 0
4 0
5 1
6 5
7 12
8 23
9 39
G-R6:TYR-CA
3 0
4 0
5 0
6 0
7 2
8 5
9 9
C-RIB:ASN-S1
3 0
4 0
5 2
6 6
7 13
8 20
9 29
G-RIB:ARG-CA
3 0
4 0
5 1
6 8
7 21
8 41
9 68
C31-P:PHE-S1
3 0
4 0
5 0
6 0
7 0
8 0
9 0
G-R6:ASP-CA
3 0
4 0
5 0
6 4
7 12
8 26
9 44
GTP-RIB:SER-S1
3 0
4 0
5 0
6 0
7 0
8 0
9 0
G-R5:CYS-S1
3 0
4 0

5	0
6	0
7	0
8	1
9	2

C31-RIB:GLU-S2

3	0
4	0
5	0
6	0
7	0
8	0
9	0

G-P:PHE-S2

3	0
4	0
5	0
6	3
7	7
8	12
9	18

G-R5:LEU-S1

3	0
4	0
5	0
6	1
7	4
8	9
9	19

G-P:ASP-S2

3	0
4	0
5	11
6	25
7	42
8	60
9	84

G-R5:TYR-CA

3	0
4	0
5	0
6	0
7	1
8	4
9	9

FHU-MY:VAL-S1

3	0
4	0
5	0
6	0
7	0
8	0
9	0

FMU-RIB:ASP-S2

3	0
4	0
5	0
6	0

7   0
8   0
9   0
FMU-MY:MET-S2
3   0
4   0
5   0
6   0
7   0
8   0
9   0
FHU-MY:ASP-S1
3   0
4   0
5   0
6   0
7   0
8   0
9   0
FMU-RIB:CYS-S1
3   0
4   0
5   0
6   0
7   0
8   0
9   0
U-Y:ASN-S2
3   0
4   0
5   1
6   4
7   7
8   12
9   17
G-P:ASP-S1
3   0
4   1
5   7
6   19
7   35
8   55
9   77
G-P:GLY-CA
3   0
4   3
5   11
6   24
7   42
8   65
9   91
G-RIB:ILE-CA
3   0
4   0
5   0
6   3
7   9
8   18

9   30
H2U-RIB:PRO-CA
3   0
4   0
5   0
6   0
7   0
8   0
9   0
FMU-MY:SER-S1
3   0
4   0
5   0
6   0
7   0
8   0
9   0
G-R5:HIS-S1
3   0
4   0
5   0
6   1
7   4
8   9
9   16
A-R6:ILE-CA
3   0
4   0
5   0
6   1
7   4
8   9
9   15
G-RIB:SER-CA
3   0
4   0
5   3
6   11
7   24
8   38
9   55
G-R6:THR-CA
3   0
4   0
5   0
6   2
7   8
8   18
9   30
A-P:LEU-S2
3   0
4   0
5   1
6   5
7   9
8   16
9   24
FHU-RIB:LEU-S2

3 0
4 0
5 0
6 0
7 0
8 0
9 0
H2U-P:ASN-S1
3 0
4 0
5 0
6 0
7 0
8 0
9 0
QUO-M6:LEU-CA
3 0
4 0
5 0
6 0
7 0
8 0
9 0
U31-P:SER-CA
3 0
4 0
5 0
6 0
7 0
8 0
9 0
C31-P:ASN-S1
3 0
4 0
5 0
6 0
7 0
8 0
9 0
A-P:MET-S2
3 0
4 0
5 1
6 3
7 5
8 7
9 10
H2U-RIB:TRP-S2
3 0
4 0
5 0
6 0
7 0
8 0
9 0
IU-P:LYS-S2
3 0
4 0

5 0
6 0
7 0
8 0
9 0
GTP-RIB:ASN-CA
3 0
4 0
5 0
6 0
7 0
8 0
9 0
G-R6:LYS-S1
3 0
4 0
5 2
6 8
7 19
8 35
9 57
GTP-RIB:ARG-S1
3 0
4 0
5 0
6 0
7 0
8 0
9 0
U-RIB:GLY-CA
3 0
4 0
5 3
6 8
7 16
8 26
9 38
C31-P:MET-S2
3 0
4 0
5 0
6 0
7 0
8 0
9 0
C-Y:LEU-S1
3 0
4 0
5 0
6 1
7 3
8 6
9 13
G-R6:TYR-S1
3 0
4 0
5 0
6 1

7 2
8 5
9 9
U-P:TRP-CA
3 0
4 0
5 0
6 0
7 1
8 2
9 3
C31-P:PHE-CA
3 0
4 0
5 0
6 0
7 0
8 0
9 0
FHU-RIB:ALA-CA
3 0
4 0
5 0
6 0
7 0
8 0
9 0
C-Y:ARG-S1
3 0
4 0
5 1
6 4
7 9
8 20
9 35
G-P:TYR-CA
3 0
4 0
5 0
6 2
7 5
8 11
9 18
FMU-RIB:GLU-S1
3 0
4 0
5 0
6 0
7 0
8 0
9 0
U31-RIB:TYR-S1
3 0
4 0
5 0
6 0
7 0
8 0

9   0
U-RIB:TYR-S2
3   0
4   0
5   0
6   1
7   4
8   6
9   9
G-RIB:ASN-CA
3   0
4   0
5   0
6   5
7   14
8   25
9   38
DA-RIB:ASP-S1
3   0
4   0
5   0
6   0
7   0
8   0
9   0
U-RIB:LEU-S1
3   0
4   0
5   0
6   1
7   4
8   9
9   15
QUO-RIB:GLN-S2
3   0
4   0
5   0
6   0
7   0
8   0
9   0
FHU-MY:ILE-CA
3   0
4   0
5   0
6   0
7   0
8   0
9   0
A-R5:ARG-S2
3   0
4   0
5   3
6   9
7   21
8   39
9   59
U-RIB:TRP-CA

3 0
4 0
5 0
6 0
7 1
8 2
9 3
QUO-M5:GLN-S2
3 0
4 0
5 0
6 0
7 0
8 0
9 0
H2U-P:THR-S1
3 0
4 0
5 0
6 0
7 0
8 0
9 0
A-R6:ARG-S2
3 0
4 1
5 4
6 14
7 26
8 40
9 55
A-RIB:GLN-CA
3 0
4 0
5 0
6 2
7 7
8 13
9 22
IU-MY:LEU-S2
3 0
4 0
5 0
6 0
7 0
8 0
9 0
G-RIB:TYR-S2
3 0
4 0
5 0
6 3
7 8
8 13
9 20
H2U-MY:THR-S1
3 0
4 0

5 0
6 0
7 0
8 0
9 0
IU-RIB:SER-CA
3 0
4 0
5 0
6 0
7 0
8 0
9 0
G-R5:ASN-S1
3 0
4 0
5 0
6 3
7 9
8 19
9 30
G-R6:HIS-S1
3 0
4 0
5 0
6 2
7 5
8 9
9 16
FHU-RIB:PRO-S1
3 0
4 0
5 0
6 0
7 0
8 0
9 0
DA-M5:HIS-S1
3 0
4 0
5 0
6 0
7 0
8 0
9 0
A-P:LYS-S2
3 0
4 3
5 11
6 23
7 36
8 50
9 67
FHU-MY:TYR-S2
3 0
4 0
5 0
6 0

7 0
8 0
9 0
U34-MY:ASP-CA
3 0
4 0
5 0
6 0
7 0
8 0
9 0
A-R5:ASP-S2
3 0
4 0
5 2
6 8
7 18
8 32
9 48
C-P:ASP-S1
3 0
4 0
5 4
6 12
7 24
8 37
9 52
DA-M6:HIS-S1
3 0
4 0
5 0
6 0
7 0
8 0
9 0
DA-M6:LEU-S2
3 0
4 0
5 0
6 0
7 0
8 0
9 0
G-P:TRP-S2
3 0
4 0
5 0
6 1
7 4
8 7
9 9
A-R6:TYR-S1
3 0
4 0
5 0
6 0
7 2
8 5

9 8
DA-M6:TYR-S2
3 0
4 0
5 0
6 0
7 0
8 0
9 0
FHU-RIB:TYR-S2
3 0
4 0
5 0
6 0
7 0
8 0
9 0
G-R5:ASN-S2
3 0
4 0
5 1
6 5
7 13
8 23
9 36
FHU-RIB:GLY-CA
3 0
4 0
5 0
6 0
7 0
8 0
9 0
C31-P:GLU-S1
3 0
4 0
5 0
6 0
7 0
8 0
9 0
G-R5:GLN-CA
3 0
4 0
5 0
6 0
7 3
8 9
9 19
FHU-RIB:ALA-S1
3 0
4 0
5 0
6 0
7 0
8 0
9 0
C-Y:ASN-CA

3 0
4 0
5 0
6 1
7 4
8 9
9 17
U-Y:ASN-S1
3 0
4 0
5 0
6 2
7 5
8 10
9 15
C-P:ARG-CA
3 0
4 0
5 2
6 8
7 17
8 30
9 48
A-R6:VAL-S1
3 0
4 0
5 1
6 3
7 7
8 12
9 21
5BU-MY:ILE-S1
3 0
4 0
5 0
6 0
7 0
8 0
9 0
QUO-M5:ASN-S2
3 0
4 0
5 0
6 0
7 0
8 0
9 0
G-P:GLU-S1
3 0
4 0
5 9
6 25
7 48
8 77
9 109
A-R6:THR-CA
3 0
4 0

5 0
6 3
7 8
8 14
9 23
G-R5:ILE-S1
3 0
4 0
5 0
6 1
7 0
8 9
9 16
A-R6:SER-S1
3 0
4 0
5 3
6 8
7 13
8 21
9 31
U-Y:LYS-S1
3 0
4 0
5 1
6 4
7 10
8 18
9 28
U-Y:MET-CA
3 0
4 0
5 0
6 0
7 1
8 2
9 4
C-RIB:GLY-CA
3 0
4 1
5 5
6 14
7 26
8 40
9 58
DA-M6:ASN-S2
3 0
4 0
5 0
6 0
7 0
8 0
9 0
U31-MY:TYR-S1
3 0
4 0
5 0
6 0

7 0
8 0
9 0
A-R5:GLN-S2
3 0
4 0
5 1
6 4
7 9
8 16
9 25
FHU-P:CYS-S1
3 0
4 0
5 0
6 0
7 0
8 0
9 0
U-Y:ARG-S1
3 0
4 0
5 0
6 3
7 7
8 14
9 24
C-Y:GLY-CA
3 0
4 0
5 2
6 6
7 14
8 26
9 42
OMC-P:LYS-S2
3 0
4 0
5 0
6 0
7 0
8 0
9 0
C31-MY:PHE-S1
3 0
4 0
5 0
6 0
7 0
8 0
9 0
C-RIB:ASN-CA
3 0
4 0
5 0
6 4
7 10
8 18

9 27
A-R5:PHE-S2
3 0
4 0
5 0
6 0
7 2
8 4
9 7
A-R6:VAL-CA
3 0
4 0
5 0
6 2
7 6
8 12
9 23
U-RIB:PHE-S2
3 0
4 0
5 0
6 1
7 3
8 5
9 8
U34-MY:PRO-CA
3 0
4 0
5 0
6 0
7 0
8 0
9 0
C-Y:MET-S2
3 0
4 0
5 0
6 1
7 2
8 4
9 7
G-R5:TYR-S1
3 0
4 0
5 0
6 0
7 2
8 4
9 9
QUO-M5:PHE-S1
3 0
4 0
5 0
6 0
7 0
8 0
9 0
DA-RIB:ALA-CA

3 0
4 0
5 0
6 0
7 0
8 0
9 0
FHU-RIB:THR-CA
3 0
4 0
5 0
6 0
7 0
8 0
9 0
FMU-MY:ASN-CA
3 0
4 0
5 0
6 0
7 0
8 0
9 0
U31-P:ASN-S2
3 0
4 0
5 0
6 0
7 0
8 0
9 0
U-P:THR-S1
3 0
4 0
5 3
6 6
7 11
8 16
9 22
H2U-MY:GLN-S2
3 0
4 0
5 0
6 0
7 0
8 0
9 0
G-RIB:CYS-S1
3 0
4 0
5 0
6 0
7 1
8 0
9 4
H2U-P:ARG-S1
3 0
4 0

5   0
6   0
7   0
8   0
9   0
U-Y:PHE-S1
3   0
4   0
5   0
6   0
7   1
8   2
9   4
FHU-MY:ARG-S2
3   0
4   0
5   0
6   0
7   0
8   0
9   0
A-RIB:THR-S1
3   0
4   0
5   3
6   7
7   14
8   23
9   33
C31-P:TYR-S1
3   0
4   0
5   0
6   0
7   0
8   0
9   0
G-R6:THR-S1
3   0
4   0
5   0
6   6
7   14
8   23
9   34
IU-RIB:PRO-S1
3   0
4   0
5   0
6   0
7   0
8   0
9   0
H2U-P:ASN-CA
3   0
4   0
5   0
6   0

7 0
8 0
9 0
C-P:TYR-CA
3 0
4 0
5 0
6 0
7 3
8 7
9 12
C31-P:ASN-CA
3 0
4 0
5 0
6 0
7 0
8 0
9 0
U-Y:TRP-S1
3 0
4 0
5 0
6 0
7 0
8 1
9 2
C31-MY:TYR-S2
3 0
4 0
5 0
6 0
7 0
8 0
9 0
FHU-P:LYS-S1
3 0
4 0
5 0
6 0
7 0
8 0
9 0
A-R5:MET-S1
3 0
4 0
5 0
6 1
7 2
8 4
9 6
G-R6:LYS-S2
3 0
4 1
5 7
6 18
7 35
8 53

9 74
A-R6:ILE-S1
3 0
4 0
5 0
6 2
7 5
8 8
9 13
A-R6:GLU-CA
3 0
4 0
5 1
6 5
7 13
8 27
9 45
U31-MY:TYR-CA
3 0
4 0
5 0
6 0
7 0
8 0
9 0
FMU-RIB:MET-S2
3 0
4 0
5 0
6 0
7 0
8 0
9 0
U-Y:CYS-S1
3 0
4 0
5 0
6 0
7 0
8 0
9 1
H2U-MY:LYS-S2
3 0
4 0
5 0
6 0
7 0
8 0
9 0
U31-P:MET-CA
3 0
4 0
5 0
6 0
7 0
8 0
9 0
FMU-MY:GLU-S1

3 0
4 0
5 0
6 0
7 0
8 0
9 0
IU-P:HIS-S1
3 0
4 0
5 0
6 0
7 0
8 0
9 0
H2U-P:LEU-S1
3 0
4 0
5 0
6 0
7 0
8 0
9 0
A-P:TYR-CA
3 0
4 0
5 0
6 1
7 3
8 7
9 11
QUO-M6:ASN-S2
3 0
4 0
5 0
6 0
7 0
8 0
9 0
U-Y:LEU-CA
3 0
4 0
5 0
6 0
7 2
8 5
9 11
G-RIB:PRO-CA
3 0
4 0
5 1
6 7
7 16
8 29
9 46
A-R5:GLU-S1
3 0
4 0

5   1
6   5
7   15
8   32
9   54
A-RIB:TYR-S2
3   0
4   0
5   0
6   2
7   5
8   9
9   13
H2U-P:THR-CA
3   0
4   0
5   0
6   0
7   0
8   0
9   0
U-P:GLN-S2
3   0
4   0
5   3
6   7
7   11
8   15
9   20
C-P:ASN-S2
3   0
4   0
5   4
6   9
7   16
8   23
9   31
G-R5:GLU-S2
3   0
4   1
5   5
6   15
7   35
8   61
9   95
G-R6:GLN-S1
3   0
4   0
5   0
6   3
7   7
8   15
9   24
A-RIB:PHE-S2
3   0
4   0
5   0
6   2

7 4
8 7
9 11
DA-RIB:ALA-S1
3 0
4 0
5 0
6 0
7 0
8 0
9 0
A-R5:TYR-CA
3 0
4 0
5 0
6 0
7 1
8 3
9 7
A-R5:LEU-S2
3 0
4 0
5 0
6 1
7 4
8 9
9 16
U31-RIB:PHE-CA
3 0
4 0
5 0
6 0
7 0
8 0
9 0
H2U-MY:GLY-CA
3 0
4 0
5 0
6 0
7 0
8 0
9 0
PSU-RIB:ARG-S2
3 0
4 0
5 0
6 0
7 0
8 0
9 0
G-P:HIS-S2
3 0
4 0
5 3
6 7
7 14
8 21

9   30
C-P:LYS-S2
3   0
4   4
5   12
6   24
7   37
8   52
9   67
C-P:GLN-S1
3   0
4   0
5   1
6   5
7   10
8   16
9   23
U-P:GLU-CA
3   0
4   0
5   1
6   6
7   15
8   27
9   42
A-RIB:GLN-S1
3   0
4   0
5   0
6   4
7   10
8   17
9   26
IU-P:ARG-S1
3   0
4   0
5   0
6   0
7   0
8   0
9   0
U-RIB:TYR-CA
3   0
4   0
5   0
6   0
7   2
8   5
9   8
IU-MY:HIS-CA
3   0
4   0
5   0
6   0
7   0
8   0
9   0
DA-M5:TYR-S1

3 0
4 0
5 0
6 0
7 0
8 0
9 0
A-R6:THR-S1
3 0
4 0
5 2
6 6
7 11
8 17
9 26
C-Y:ASP-S1
3 0
4 0
5 1
6 4
7 11
8 22
9 36
FMU-P:PHE-S2
3 0
4 0
5 0
6 0
7 0
8 0
9 0
H2U-RIB:LYS-S1
3 0
4 0
5 0
6 0
7 0
8 0
9 0
U-RIB:ASN-S2
3 0
4 0
5 2
6 6
7 11
8 15
9 21
G-P:CYS-CA
3 0
4 0
5 0
6 0
7 0
8 3
9 6
QUO-M6:LEU-S2
3 0
4 0

5 0
6 0
7 0
8 0
9 0
C-RIB:PHE-S2
3 0
4 0
5 0
6 2
7 4
8 7
9 12
C-Y:ASN-S1
3 0
4 0
5 0
6 2
7 6
8 12
9 21
DA-RIB:LYS-S2
3 0
4 0
5 0
6 0
7 0
8 0
9 0
FMU-MY:GLN-CA
3 0
4 0
5 0
6 0
7 0
8 0
9 0
DA-M6:MET-S2
3 0
4 0
5 0
6 0
7 0
8 0
9 0
G-P:GLU-CA
3 0
4 0
5 4
6 15
7 35
8 62
9 98
H2U-RIB:PRO-S1
3 0
4 0
5 0
6 0

7 0
8 0
9 0
QUO-M5:ASN-CA
3 0
4 0
5 0
6 0
7 0
8 0
9 0
G-P:MET-S1
3 0
4 0
5 1
6 3
7 6
8 10
9 15
U31-MY:GLU-S2
3 0
4 0
5 0
6 0
7 0
8 0
9 0
A-RIB:ILE-S1
3 0
4 0
5 1
6 3
7 6
8 11
9 18
5BU-RIB:PRO-S1
3 0
4 0
5 0
6 0
7 0
8 0
9 0
U-Y:HIS-S1
3 0
4 0
5 0
6 1
7 2
8 4
9 7
U31-RIB:MET-S1
3 0
4 0
5 0
6 0
7 0
8 0

9   0
H2U-RIB:LYS-S2
3   0
4   0
5   0
6   0
7   0
8   0
9   0
G-RIB:VAL-CA
3   0
4   0
5   0
6   5
7   15
8   29
9   49
FHU-MY:LEU-S2
3   0
4   0
5   0
6   0
7   0
8   0
9   0
U34-MY:ASN-S1
3   0
4   0
5   0
6   0
7   0
8   0
9   0
A-RIB:MET-S1
3   0
4   0
5   0
6   1
7   3
8   6
9   10
G-RIB:GLY-CA
3   0
4   1
5   8
6   20
7   37
8   60
9   87
DA-M5:LEU-S1
3   0
4   0
5   0
6   0
7   0
8   0
9   0
U34-RIB:SER-CA

3 0
4 0
5 0
6 0
7 0
8 0
9 0
U-RIB:MET-S1
3 0
4 0
5 0
6 1
7 2
8 3
9 6
5BU-P:ALA-S1
3 0
4 0
5 0
6 0
7 0
8 0
9 0
U34-P:HIS-CA
3 0
4 0
5 0
6 0
7 0
8 0
9 0
C31-MY:ALA-S1
3 0
4 0
5 0
6 0
7 0
8 0
9 0
C-P:MET-S2
3 0
4 0
5 1
6 2
7 5
8 7
9 10
U-RIB:LEU-S2
3 0
4 0
5 0
6 2
7 5
8 9
9 15
U34-P:GLU-CA
3 0
4 0

5 0
6 0
7 0
8 0
9 0
U-Y:VAL-CA
3 0
4 0
5 0
6 1
7 3
8 7
9 13
H2U-RIB:ASN-S2
3 0
4 0
5 0
6 0
7 0
8 0
9 0
C-P:ALA-S1
3 0
4 2
5 5
6 11
7 18
8 27
9 40
C-Y:TRP-S1
3 0
4 0
5 0
6 0
7 1
8 1
9 3
DA-M5:ASP-S2
3 0
4 0
5 0
6 0
7 0
8 0
9 0
FMU-MY:ALA-CA
3 0
4 0
5 0
6 0
7 0
8 0
9 0
U31-P:GLN-CA
3 0
4 0
5 0
6 0

7 0
8 0
9 0
G-R5:MET-S1
3 0
4 0
5 0
6 1
7 2
8 5
9 9
H2U-RIB:GLY-CA
3 0
4 0
5 0
6 0
7 0
8 0
9 0
IU-P:HIS-CA
3 0
4 0
5 0
6 0
7 0
8 0
9 0
U-P:PHE-S2
3 0
4 0
5 0
6 1
7 3
8 5
9 8
C31-RIB:SER-S1
3 0
4 0
5 0
6 0
7 0
8 0
9 0
G-P:LEU-S1
3 0
4 0
5 2
6 6
7 13
8 24
9 40
G-P:ASN-S2
3 0
4 0
5 6
6 14
7 23
8 34

9 46
FHU-RIB:TYR-CA
3 0
4 0
5 0
6 0
7 0
8 0
9 0
C31-MY:GLU-CA
3 0
4 0
5 0
6 0
7 0
8 0
9 0
DA-RIB:ASN-CA
3 0
4 0
5 0
6 0
7 0
8 0
9 0
G-P:MET-CA
3 0
4 0
5 0
6 3
7 6
8 10
9 16
C-Y:VAL-CA
3 0
4 0
5 0
6 1
7 3
8 8
9 17
C-Y:PRO-S1
3 0
4 0
5 0
6 3
7 7
8 13
9 21
U31-P:MET-S2
3 0
4 0
5 0
6 0
7 0
8 0
9 0
U-Y:HIS-CA

3 0
4 0
5 0
6 0
7 2
8 3
9 6
IU-P:ARG-CA
3 0
4 0
5 0
6 0
7 0
8 0
9 0
G-R5:PHE-CA
3 0
4 0
5 0
6 0
7 1
8 4
9 9
A-R6:ASN-S1
3 0
4 0
5 1
6 4
7 9
8 15
9 23
G-P:SER-CA
3 0
4 0
5 4
6 12
7 24
8 38
9 55
FMU-RIB:ALA-S1
3 0
4 0
5 0
6 0
7 0
8 0
9 0
FMU-MY:GLU-CA
3 0
4 0
5 0
6 0
7 0
8 0
9 0
FMU-P:VAL-S1
3 0
4 0

5 0
6 0
7 0
8 0
9 0
A-RIB:SER-CA
3 0
4 0
5 2
6 7
7 14
8 24
9 36
G-RIB:ASN-S1
3 0
4 0
5 2
6 8
7 18
8 28
9 41
FMU-P:CYS-S1
3 0
4 0
5 0
6 0
7 0
8 0
9 0
DA-M6:GLN-CA
3 0
4 0
5 0
6 0
7 0
8 0
9 0
G-P:ARG-S2
3 0
4 3
5 14
6 32
7 54
8 77
9 103
C-P:ASN-CA
3 0
4 0
5 1
6 4
7 11
8 18
9 27
IU-RIB:ILE-S1
3 0
4 0
5 0
6 0

7 0
8 0
9 0
IU-RIB:LYS-CA
3 0
4 0
5 0
6 0
7 0
8 0
9 0
G-RIB:TYR-S1
3 0
4 0
5 0
6 2
7 5
8 9
9 17
U-RIB:ILE-S1
3 0
4 0
5 0
6 2
7 4
8 8
9 12
QUO-P:LEU-S2
3 0
4 0
5 0
6 0
7 0
8 0
9 0
GTP-M6:GLY-CA
3 0
4 0
5 0
6 0
7 0
8 0
9 0
QUO-M5:PHE-S2
3 0
4 0
5 0
6 0
7 0
8 0
9 0
A-P:PHE-S1
3 0
4 0
5 0
6 1
7 3
8 7

9 12
G-R6:PHE-CA
3 0
4 0
5 0
6 0
7 2
8 5
9 10
DA-M6:TYR-S1
3 0
4 0
5 0
6 0
7 0
8 0
9 0
H2U-MY:PHE-S1
3 0
4 0
5 0
6 0
7 0
8 0
9 0
A-P:SER-CA
3 0
4 0
5 2
6 7
7 15
8 25
9 36
C-Y:TRP-CA
3 0
4 0
5 0
6 0
7 0
8 1
9 3
QUO-M5:ASP-S2
3 0
4 0
5 0
6 0
7 0
8 0
9 0
G-RIB:CYS-CA
3 0
4 0
5 0
6 0
7 1
8 2
9 4
U31-MY:ASP-S2

3 0
4 0
5 0
6 0
7 0
8 0
9 0
U-Y:TYR-S2
3 0
4 0
5 0
6 1
7 2
8 4
9 7
QUO-M5:ASN-S1
3 0
4 0
5 0
6 0
7 0
8 0
9 0
DA-RIB:HIS-S1
3 0
4 0
5 0
6 0
7 0
8 0
9 0
U31-RIB:ASP-CA
3 0
4 0
5 0
6 0
7 0
8 0
9 0
A-P:ARG-S2
3 0
4 0
5 9
6 21
7 36
8 53
9 71
U34-P:ASN-S2
3 0
4 0
5 0
6 0
7 0
8 0
9 0
QUO-M5:ASP-CA
3 0
4 0

5 0
6 0
7 0
8 0
9 0
U-P:SER-CA
3 0
4 0
5 1
6 5
7 10
8 17
9 25
GTP-M6:ASN-S1
3 0
4 0
5 0
6 0
7 0
8 0
9 0
U31-RIB:MET-CA
3 0
4 0
5 0
6 0
7 0
8 0
9 0
U31-MY:MET-S2
3 0
4 0
5 0
6 0
7 0
8 0
9 0
U-P:THR-CA
3 0
4 0
5 1
6 4
7 8
8 14
9 22
IU-P:ARG-S2
3 0
4 0
5 0
6 0
7 0
8 0
9 0
QUO-M5:GLU-S1
3 0
4 0
5 0
6 0

7 0
8 0
9 0
G-R6:SER-CA
3 0
4 0
5 1
6 4
7 12
8 23
9 38
G-P:PRO-CA
3 0
4 0
5 3
6 9
7 18
8 32
9 47
IU-MY:SER-CA
3 0
4 0
5 0
6 0
7 0
8 0
9 0
QUO-M5:ARG-CA
3 0
4 0
5 0
6 0
7 0
8 0
9 0
A-R6:MET-S2
3 0
4 0
5 0
6 2
7 3
8 5
9 7
C-RIB:HIS-S2
3 0
4 0
5 1
6 4
7 8
8 13
9 17
A-RIB:TYR-S1
3 0
4 0
5 0
6 1
7 3
8 6

9   11
U31-MY:GLU-CA
3   0
4   0
5   0
6   0
7   0
8   0
9   0
C-P:MET-CA
3   0
4   0
5   0
6   1
7   4
8   7
9   11
A-RIB:PHE-S1
3   0
4   0
5   0
6   1
7   3
8   7
9   13
A-R5:PHE-S1
3   0
4   0
5   0
6   0
7   1
8   3
9   6
G-RIB:PRO-S1
3   0
4   0
5   3
6   10
7   20
8   33
9   48
FMU-P:ASP-CA
3   0
4   0
5   0
6   0
7   0
8   0
9   0
C31-MY:SER-S1
3   0
4   0
5   0
6   0
7   0
8   0
9   0
U-P:LEU-CA

3 0
4 0
5 0
6 2
7 5
8 11
9 20
A-RIB:ASP-S1
3 0
4 0
5 3
6 11
7 23
8 36
9 55
C-P:GLU-S2
3 0
4 0
5 0
6 25
7 43
8 60
9 79
U31-MY:THR-S1
3 0
4 0
5 0
6 0
7 0
8 0
9 0
C-Y:CYS-CA
3 0
4 0
5 0
6 0
7 0
8 0
9 1
C-P:ALA-CA
3 0
4 0
5 3
6 8
7 16
8 26
9 40
H2U-RIB:PHE-S1
3 0
4 0
5 0
6 0
7 0
8 0
9 0
U-P:CYS-CA
3 0
4 0

5 0
6 0
7 0
8 1
9 2
M2G-P:GLU-S1
3 0
4 0
5 0
6 0
7 0
8 0
9 0
U34-MY:ASP-S2
3 0
4 0
5 0
6 0
7 0
8 0
9 0
DA-M5:HIS-CA
3 0
4 0
5 0
6 0
7 0
8 0
9 0
DA-M5:LEU-S2
3 0
4 0
5 0
6 0
7 0
8 0
9 0
U31-P:GLN-S1
3 0
4 0
5 0
6 0
7 0
8 0
9 0
A-P:LYS-CA
3 0
4 0
5 2
6 8
7 19
8 33
9 50
G-R6:MET-S2
3 0
4 0
5 0
6 2

7   4
8   6
9   9
DA-RIB:MET-CA
3   0
4   0
5   0
6   0
7   0
8   0
9   0
A-R5:ASN-S2
3   0
4   0
5   1
6   4
7   10
8   18
9   27
IU-P:SER-S1
3   0
4   0
5   0
6   0
7   0
8   0
9   0
QUO-M6:GLN-S2
3   0
4   0
5   0
6   0
7   0
8   0
9   0
5BU-P:SER-CA
3   0
4   0
5   0
6   0
7   0
8   0
9   0
G-RIB:GLU-S2
3   0
4   0
5   13
6   34
7   61
8   88
9   120
U-Y:MET-S2
3   0
4   0
5   0
6   1
7   1
8   2

9 4
H2U-MY:LYS-S1
3 0
4 0
5 0
6 0
7 0
8 0
9 0
FMU-RIB:MET-S1
3 0
4 0
5 0
6 0
7 0
8 0
9 0
G-R6:ARG-S1
3 0
4 0
5 1
6 7
7 16
8 31
9 51
FMU-RIB:ARG-S1
3 0
4 0
5 0
6 0
7 0
8 0
9 0
C-RIB:LEU-S2
3 0
4 0
5 1
6 4
7 9
8 16
9 25
U31-RIB:ASP-S1
3 0
4 0
5 0
6 0
7 0
8 0
9 0
A-RIB:HIS-CA
3 0
4 0
5 0
6 2
7 4
8 9
9 16
FHU-MY:THR-CA

3 0
4 0
5 0
6 0
7 0
8 0
9 0
G-P:ASP-CA
3 0
4 0
5 4
6 13
7 28
8 49
9 73
IU-RIB:LYS-S1
3 0
4 0
5 0
6 0
7 0
8 0
9 0
FHU-P:ALA-S1
3 0
4 0
5 0
6 0
7 0
8 0
9 0
G-P:GLN-S1
3 0
4 0
5 3
6 8
7 16
8 27
9 39
FMU-MY:PRO-CA
3 0
4 0
5 0
6 0
7 0
8 0
9 0
U34-RIB:TYR-S2
3 0
4 0
5 0
6 0
7 0
8 0
9 0
C-RIB:LYS-S1
3 0
4 0

5   3
6   12
7   24
8   40
9   59
QUO-P:ASN-S2
3   0
4   0
5   0
6   0
7   0
8   0
9   0
C31-RIB:GLU-S1
3   0
4   0
5   0
6   0
7   0
8   0
9   0
A-RIB:LEU-S2
3   0
4   0
5   1
6   4
7   9
8   16
9   24
G-P:PHE-S1
3   0
4   0
5   0
6   3
7   6
8   12
9   19
G-RIB:THR-CA
3   0
4   0
5   1
6   5
7   14
8   28
9   45
A-RIB:ASP-CA
3   0
4   0
5   1
6   7
7   17
8   32
9   49
OMC-RIB:LYS-S1
3   0
4   0
5   0
6   0

7 0
8 0
9 0
C-Y:GLU-CA
3 0
4 0
5 0
6 2
7 7
8 18
9 36
A-R6:MET-CA
3 0
4 0
5 0
6 1
7 2
8 4
9 8
U34-MY:ASN-S2
3 0
4 0
5 0
6 0
7 0
8 0
9 0
G-RIB:TYR-CA
3 0
4 0
5 0
6 1
7 5
8 10
9 18
A-RIB:ASN-CA
3 0
4 0
5 0
6 3
7 10
8 18
9 28
FMU-P:ALA-CA
3 0
4 0
5 0
6 0
7 0
8 0
9 0
G-R5:ALA-CA
3 0
4 0
5 1
6 4
7 11
8 23

9 43
FHU-MY:LEU-S1
3 0
4 0
5 0
6 0
7 0
8 0
9 0
G-RIB:HIS-S2
3 0
4 0
5 2
6 7
7 13
8 21
9 28
U-P:HIS-S2
3 0
4 0
5 1
6 3
7 6
8 8
9 12
C-P:SER-CA
3 0
4 0
5 2
6 8
7 16
8 24
9 36
GTP-RIB:THR-S1
3 0
4 0
5 0
6 0
7 0
8 0
9 0
G-R6:TRP-S1
3 0
4 0
5 0
6 0
7 1
8 3
9 5
FHU-MY:ARG-CA
3 0
4 0
5 0
6 0
7 0
8 0
9 0
C-RIB:PHE-S1

3	0
4	0
5	0
6	1
7	3
8	7
9	12
A-P:HIS-S1
3	0
4	0
5	1
6	3
7	6
8	11
9	17
U-Y:ALA-S1
3	0
4	0
5	1
6	3
7	7
8	12
9	18
A-R5:GLY-CA
3	0
4	0
5	2
6	7
7	16
8	30
9	48
C-P:ARG-S1
3	0
4	1
5	4
6	12
7	24
8	39
9	57
DA-RIB:HIS-CA
3	0
4	0
5	0
6	0
7	0
8	0
9	0
FMU-MY:ARG-CA
3	0
4	0
5	0
6	0
7	0
8	0
9	0
I-RIB:TRP-S2
3	0
4	0

5 0
6 0
7 0
8 0
9 0
A-R6:HIS-S2
3 0
4 0
5 1
6 3
7 6
8 10
9 15
A-R6:ASP-S2
3 0
4 0
5 4
6 12
7 21
8 32
9 46
GTP-M5:ALA-S1
3 0
4 0
5 0
6 0
7 0
8 0
9 0
IU-RIB:HIS-CA
3 0
4 0
5 0
6 0
7 0
8 0
9 0
G-R5:MET-S2
3 0
4 0
5 0
6 1
7 3
8 7
9 11
FHU-MY:ALA-S1
3 0
4 0
5 0
6 0
7 0
8 0
9 0
I-RIB:GLY-CA
3 0
4 0
5 0
6 0

7 0
8 0
9 0
C-RIB:ASP-CA
3 0
4 0
5 1
6 7
7 18
8 32
9 49
G-R6:MET-CA
3 0
4 0
5 0
6 0
7 2
8 4
9 8
G-R6:TYR-S2
3 0
4 0
5 0
6 1
7 4
8 8
9 12
U31-MY:ASP-CA
3 0
4 0
5 0
6 0
7 0
8 0
9 0
U-P:ARG-S2
3 0
4 0
5 6
6 14
7 23
8 33
9 45
A-R6:GLN-S2
3 0
4 0
5 2
6 6
7 11
8 16
9 23
U-P:ASP-S1
3 0
4 0
5 2
6 7
7 15
8 24

9   34
A-P:LEU-CA
3   0
4   0
5   0
6   2
7   8
8   16
9   29
C-RIB:MET-CA
3   0
4   0
5   0
6   1
7   3
8   6
9   11
A-P:GLU-S1
3   0
4   0
5   6
6   16
7   32
8   51
9   72
FHU-RIB:ARG-S2
3   0
4   0
5   0
6   0
7   0
8   0
9   0
QUO-M6:ARG-S2
3   0
4   0
5   0
6   0
7   0
8   0
9   0
C-P:MET-S1
3   0
4   0
5   0
6   2
7   4
8   6
9   10
U31-P:MET-S1
3   0
4   0
5   0
6   0
7   0
8   0
9   0
OMC-RIB:LYS-S2

3 0
4 0
5 0
6 0
7 0
8 0
9 0
C-P:ILE-S1
3 0
4 0
5 1
6 3
7 7
8 11
9 18
C-Y:SER-CA
3 0
4 0
5 0
6 2
7 6
8 14
9 25
FHU-MY:SER-CA
3 0
4 0
5 0
6 0
7 0
8 0
9 0
U31-MY:MET-CA
3 0
4 0
5 0
6 0
7 0
8 0
9 0
G-R6:ILE-CA
3 0
4 0
5 0
6 1
7 4
8 9
9 17
C31-MY:THR-S1
3 0
4 0
5 0
6 0
7 0
8 0
9 0
C31-MY:GLN-S2
3 0
4 0

5 0
6 0
7 0
8 0
9 0
G-R5:ILE-CA
3 0
4 0
5 0
6 0
7 0
8 8
9 16
IU-MY:ALA-CA
3 0
4 0
5 0
6 0
7 0
8 0
9 0
IU-RIB:SER-S1
3 0
4 0
5 0
6 0
7 0
8 0
9 0
G-R5:ALA-S1
3 0
4 0
5 3
6 7
7 16
8 29
9 47
H2U-P:ASN-S2
3 0
4 0
5 0
6 0
7 0
8 0
9 0
IU-MY:ARG-S1
3 0
4 0
5 0
6 0
7 0
8 0
9 0
G-RIB:HIS-CA
3 0
4 0
5 0
6 2

7   7
8   14
9   23
C31-P:TYR-S2
3   0
4   0
5   0
6   0
7   0
8   0
9   0
IU-MY:ILE-CA
3   0
4   0
5   0
6   0
7   0
8   0
9   0
G-R6:LEU-S2
3   0
4   0
5   1
6   3
7   6
8   12
9   20
C-RIB:VAL-S1
3   0
4   0
5   2
6   5
7   11
8   19
9   29
U31-RIB:ASN-CA
3   0
4   0
5   0
6   0
7   0
8   0
9   0
FMU-P:ARG-S1
3   0
4   0
5   0
6   0
7   0
8   0
9   0
G-R5:GLU-CA
3   0
4   0
5   0
6   0
7   11
8   27

9 56
G-RIB:TRP-CA
3 0
4 0
5 0
6 1
7 2
8 5
9 8
QUO-M5:ASP-S1
3 0
4 0
5 0
6 0
7 0
8 0
9 0
G-P:THR-S1
3 0
4 1
5 6
6 13
7 22
8 34
9 50
G-R6:VAL-CA
3 0
4 0
5 0
6 2
7 7
8 16
9 29
G-R6:ALA-CA
3 0
4 0
5 1
6 6
7 14
8 25
9 40
DA-RIB:ARG-S2
3 0
4 0
5 0
6 0
7 0
8 0
9 0
U31-MY:ILE-S1
3 0
4 0
5 0
6 0
7 0
8 0
9 0
C-RIB:ASP-S1

3 0
4 0
5 3
6 11
7 23
8 37
9 52
C-Y:HIS-S2
3 0
4 0
5 0
6 1
7 4
8 7
9 12
C31-MY:ALA-CA
3 0
4 0
5 0
6 0
7 0
8 0
9 0
5BU-P:ARG-S1
3 0
4 0
5 0
6 0
7 0
8 0
9 0
IU-P:LEU-S1
3 0
4 0
5 0
6 0
7 0
8 0
9 0
H2U-MY:GLN-CA
3 0
4 0
5 0
6 0
7 0
8 0
9 0
C-Y:LEU-S2
3 0
4 0
5 0
6 1
7 4
8 8
9 14
H2U-P:LYS-S2
3 0
4 0

5 0
6 0
7 0
8 0
9 0
U34-P:GLU-S2
3 0
4 0
5 0
6 0
7 0
8 0
9 0
C-Y:GLU-S1
3 0
4 0
5 1
6 5
7 13
8 28
9 48
IU-MY:ALA-S1
3 0
4 0
5 0
6 0
7 0
8 0
9 0
H2U-P:TRP-S2
3 0
4 0
5 0
6 0
7 0
8 0
9 0
IU-MY:ARG-CA
3 0
4 0
5 0
6 0
7 0
8 0
9 0
I-RIB:ALA-CA
3 0
4 0
5 0
6 0
7 0
8 0
9 0
U34-P:ARG-S2
3 0
4 0
5 0
6 0

7 0
8 0
9 0
C31-P:GLN-S2
3 0
4 0
5 0
6 0
7 0
8 0
9 0
FHU-P:ASP-CA
3 0
4 0
5 0
6 0
7 0
8 0
9 0
IU-RIB:ARG-CA
3 0
4 0
5 0
6 0
7 0
8 0
9 0
C-Y:GLN-S2
3 0
4 0
5 1
6 3
7 7
8 13
9 21
FHU-RIB:LEU-CA
3 0
4 0
5 0
6 0
7 0
8 0
9 0
C-RIB:PRO-CA
3 0
4 0
5 1
6 5
7 11
8 20
9 31
A-P:PHE-S2
3 0
4 0
5 0
6 2
7 4
8 7

9 12
U-RIB:GLN-S1
3 0
4 0
5 0
6 3
7 7
8 13
9 19
C31-RIB:GLU-CA
3 0
4 0
5 0
6 0
7 0
8 0
9 0
G-P:PHE-CA
3 0
4 0
5 0
6 2
7 6
8 13
9 22
H2U-MY:PHE-S2
3 0
4 0
5 0
6 0
7 0
8 0
9 0
DA-RIB:SER-S1
3 0
4 0
5 0
6 0
7 0
8 0
9 0
A-R6:GLU-S1
3 0
4 0
5 3
6 9
7 21
8 35
9 54
C31-RIB:LEU-CA
3 0
4 0
5 0
6 0
7 0
8 0
9 0
G-RIB:ASP-CA

3 0
4 0
5 1
6 9
7 24
8 44
9 69
H2U-P:PHE-S2
3 0
4 0
5 0
6 0
7 0
8 0
9 0
C-RIB:SER-CA
3 0
4 0
5 2
6 7
7 16
8 26
9 38
U34-MY:ASN-CA
3 0
4 0
5 0
6 0
7 0
8 0
9 0
U-P:HIS-CA
3 0
4 0
5 0
6 0
7 3
8 7
9 10
U34-MY:SER-CA
3 0
4 0
5 0
6 0
7 0
8 0
9 0
G-R5:GLU-S1
3 0
4 0
5 1
6 7
7 19
8 42
9 74
DA-RIB:VAL-CA
3 0
4 0

5 0
6 0
7 0
8 0
9 0
H2U-RIB:PHE-CA
3 0
4 0
5 0
6 0
7 0
8 0
9 0
FMU-RIB:HIS-S2
3 0
4 0
5 0
6 0
7 0
8 0
9 0
QUO-M6:PHE-CA
3 0
4 0
5 0
6 0
7 0
8 0
9 0
H2U-MY:ASN-S2
3 0
4 0
5 0
6 0
7 0
8 0
9 0
DA-M6:THR-CA
3 0
4 0
5 0
6 0
7 0
8 0
9 0
GTP-RIB:THR-CA
3 0
4 0
5 0
6 0
7 0
8 0
9 0
A-P:ALA-CA
3 0
4 0
5 3
6 8

7   16
8   27
9   42
FMU-P:ASP-S2
3   0
4   0
5   0
6   0
7   0
8   0
9   0
DA-M5:THR-CA
3   0
4   0
5   0
6   0
7   0
8   0
9   0
G-R6:PHE-S1
3   0
4   0
5   0
6   1
7   2
8   4
9   8
U-Y:ASP-S2
3   0
4   0
5   2
6   5
7   11
8   18
9   29
G-RIB:ALA-CA
3   0
4   0
5   3
6   11
7   24
8   40
9   62
G-R6:CYS-CA
3   0
4   0
5   0
6   0
7   0
8   1
9   3
FMU-RIB:GLN-S2
3   0
4   0
5   0
6   0
7   0
8   0

9   0
G-RIB:SER-S1
3   0
4   1
5   7
6   16
7   28
8   42
9   60
IU-MY:LYS-CA
3   0
4   0
5   0
6   0
7   0
8   0
9   0
H2U-P:LEU-S2
3   0
4   0
5   0
6   0
7   0
8   0
9   0
QUO-M6:GLU-S2
3   0
4   0
5   0
6   0
7   0
8   0
9   0
U-P:MET-CA
3   0
4   0
5   0
6   1
7   2
8   4
9   7
A-P:ASN-S1
3   0
4   0
5   2
6   7
7   13
8   21
9   30
A-P:TRP-CA
3   0
4   0
5   0
6   0
7   2
8   3
9   6
C-P:GLU-S1

3 0
4 1
5 5
6 16
7 32
8 50
9 73
U-P:ALA-S1
3 0
4 1
5 3
6 6
7 11
8 17
9 26
U-RIB:CYS-S1
3 0
4 0
5 0
6 0
7 0
8 1
9 1
H2U-RIB:GLU-S1
3 0
4 0
5 0
6 0
7 0
8 0
9 0
C-RIB:PRO-S1
3 0
4 0
5 2
6 8
7 14
8 22
9 31
G-P:VAL-CA
3 0
4 0
5 0
6 7
7 18
8 33
9 53
U31-P:GLN-S2
3 0
4 0
5 0
6 0
7 0
8 0
9 0
U-Y:GLY-CA
3 0
4 0

5   1
6   5
7   10
8   17
9   28
A-RIB:ASN-S2
3   0
4   0
5   3
6   8
7   16
8   24
9   33
FHU-P:LEU-CA
3   0
4   0
5   0
6   0
7   0
8   0
9   0
A-R5:ARG-CA
3   0
4   0
5   0
6   2
7   6
8   15
9   30
IU-RIB:GLN-S2
3   0
4   0
5   0
6   0
7   0
8   0
9   0
IU-RIB:ARG-S2
3   0
4   0
5   0
6   0
7   0
8   0
9   0
QUO-M5:LYS-S1
3   0
4   0
5   0
6   0
7   0
8   0
9   0
FHU-MY:ASP-S2
3   0
4   0
5   0
6   0

7 0
8 0
9 0
QUO-M6:GLN-S1
3 0
4 0
5 0
6 0
7 0
8 0
9 0
QUO-M6:LYS-S2
3 0
4 0
5 0
6 0
7 0
8 0
9 0
G-RIB:GLN-S2
3 0
4 0
5 4
6 12
7 21
8 31
9 42
FMU-RIB:GLU-S2
3 0
4 0
5 0
6 0
7 0
8 0
9 0
U31-MY:ILE-CA
3 0
4 0
5 0
6 0
7 0
8 0
9 0
DA-M6:TYR-CA
3 0
4 0
5 0
6 0
7 0
8 0
9 0
IU-MY:LYS-S1
3 0
4 0
5 0
6 0
7 0
8 0

9 0
DA-M6:LYS-S1
3 0
4 0
5 0
6 0
7 0
8 0
9 0
C-RIB:LYS-S2
3 0
4 0
5 9
6 21
7 36
8 52
9 68
U-RIB:GLN-CA
3 0
4 0
5 0
6 1
7 5
8 9
9 16
FHU-P:PRO-S1
3 0
4 0
5 0
6 0
7 0
8 0
9 0
G-P:ALA-CA
3 0
4 1
5 5
6 14
7 27
8 43
9 66
U34-P:PHE-S2
3 0
4 0
5 0
6 0
7 0
8 0
9 0
U34-MY:TYR-S2
3 0
4 0
5 0
6 0
7 0
8 0
9 0
C-P:HIS-S1

3 0
4 0
5 1
6 3
7 5
8 10
9 15
H2U-MY:ILE-CA
3 0
4 0
5 0
6 0
7 0
8 0
9 0
G-P:MET-S2
3 0
4 0
5 2
6 4
7 7
8 11
9 15
G-P:GLN-CA
3 0
4 0
5 1
6 5
7 12
8 22
9 35
U31-MY:GLN-CA
3 0
4 0
5 0
6 0
7 0
8 0
9 0
A-RIB:PHE-CA
3 0
4 0
5 0
6 1
7 4
8 8
9 14
U-P:ASN-S2
3 0
4 0
5 2
6 6
7 10
8 15
9 21
U34-RIB:ASN-S2
3 0
4 0

5 0
6 0
7 0
8 0
9 0
U-P:PRO-S1
3 0
4 0
5 2
6 5
7 9
8 14
9 21
A-R5:PRO-S1
3 0
4 0
5 1
6 3
7 8
8 15
9 25
A-R5:HIS-S1
3 0
4 0
5 0
6 1
7 3
8 6
9 11
A-R6:CYS-CA
3 0
4 0
5 0
6 0
7 0
8 1
9 2
U-Y:PRO-CA
3 0
4 0
5 0
6 1
7 4
8 8
9 13
G-RIB:TRP-S2
3 0
4 0
5 0
6 1
7 3
8 6
9 9
G-R5:ARG-S1
3 0
4 0
5 1
6 5

7   14
8   30
9   55
U-RIB:VAL-CA
3   0
4   0
5   0
6   2
7   6
8   13
9   22
C-RIB:TRP-S1
3   0
4   0
5   0
6   1
7   2
8   3
9   6
QUO-RIB:LEU-CA
3   0
4   0
5   0
6   0
7   0
8   0
9   0
A-R6:ASP-S1
3   0
4   0
5   3
6   8
7   17
8   28
9   42
U-P:ILE-CA
3   0
4   0
5   0
6   2
7   5
8   9
9   15
DA-M6:THR-S1
3   0
4   0
5   0
6   0
7   0
8   0
9   0
G-P:TYR-S1
3   0
4   0
5   0
6   2
7   5
8   10

9   18
U-P:TYR-S2
3   0
4   0
5   0
6   2
7   4
8   7
9   9
A-RIB:GLU-S2
3   0
4   2
5   9
6   23
7   41
8   60
9   81
FMU-MY:ARG-S2
3   0
4   0
5   0
6   0
7   0
8   0
9   0
FMU-P:ILE-CA
3   0
4   0
5   0
6   0
7   0
8   0
9   0
G-R5:THR-S1
3   0
4   0
5   1
6   5
7   12
8   22
9   38
G-R6:HIS-S2
3   0
4   0
5   0
6   3
7   7
8   13
9   20
H2U-MY:LEU-CA
3   0
4   0
5   0
6   0
7   0
8   0
9   0
G-R6:TRP-S2

3 0
4 0
5 0
6 0
7 1
8 4
9 6
DA-M5:LYS-S2
3 0
4 0
5 0
6 0
7 0
8 0
9 0
G-RIB:ALA-S1
3 0
4 2
5 7
6 16
7 27
8 42
9 62
FHU-RIB:ASP-CA
3 0
4 0
5 0
6 0
7 0
8 0
9 0
C-Y:ALA-S1
3 0
4 0
5 1
6 5
7 10
8 18
9 29
C-Y:ARG-S2
3 0
4 0
5 2
6 8
7 18
8 33
9 51
4SU-P:GLU-S2
3 0
4 0
5 0
6 0
7 0
8 0
9 0
FHU-P:SER-S1
3 0
4 0

5   0
6   0
7   0
8   0
9   0
G-RIB:MET-S2
3   0
4   0
5   1
6   4
7   7
8   11
9   15
U-Y:LYS-CA
3   0
4   0
5   0
6   2
7   6
8   13
9   23
C-P:PRO-S1
3   0
4   0
5   3
6   8
7   14
8   22
9   31
IU-MY:PRO-CA
3   0
4   0
5   0
6   0
7   0
8   0
9   0
U-P:ALA-CA
3   0
4   0
5   2
6   5
7   10
8   16
9   26
H2U-MY:ASN-CA
3   0
4   0
5   0
6   0
7   0
8   0
9   0
GTP-M6:SER-S1
3   0
4   0
5   0
6   0

7 0
8 0
9 0
G-P:HIS-CA
3 0
4 0
5 0
6 3
7 8
8 16
9 26
G-RIB:LEU-S2
3 0
4 0
5 2
6 6
7 13
8 21
9 34
IU-MY:LEU-S1
3 0
4 0
5 0
6 0
7 0
8 0
9 0
A-R6:LYS-S2
3 0
4 0
5 7
6 16
7 28
8 41
9 55
C31-RIB:LEU-S2
3 0
4 0
5 0
6 0
7 0
8 0
9 0
QUO-M5:LEU-S1
3 0
4 0
5 0
6 0
7 0
8 0
9 0
A-R6:LEU-S1
3 0
4 0
5 0
6 2
7 4
8 9

9   17
G-R6:LEU-S1
3   0
4   0
5   0
6   2
7   5
8   10
9   18
FMU-MY:CYS-CA
3   0
4   0
5   0
6   0
7   0
8   0
9   0
G-R6:ARG-S2
3   0
4   0
5   4
6   14
7   30
8   50
9   71
C-RIB:GLN-S2
3   0
4   0
5   3
6   8
7   14
8   20
9   27
A-RIB:PRO-S1
3   0
4   0
5   2
6   8
7   15
8   23
9   33
IU-MY:VAL-CA
3   0
4   0
5   0
6   0
7   0
8   0
9   0
IU-MY:LEU-CA
3   0
4   0
5   0
6   0
7   0
8   0
9   0
C-Y:ALA-CA

3 0
4 0
5 0
6 3
7 7
8 14
9 25
C-RIB:TRP-CA
3 0
4 0
5 0
6 0
7 1
8 3
9 6
5BU-P:PRO-CA
3 0
4 0
5 0
6 0
7 0
8 0
9 0
FHU-P:HIS-S2
3 0
4 0
5 0
6 0
7 0
8 0
9 0
U-RIB:ASN-S1
3 0
4 0
5 1
6 4
7 8
8 14
9 20
C-P:LEU-S2
3 0
4 0
5 2
6 5
7 10
8 16
9 26
U34-MY:PHE-S1
3 0
4 0
5 0
6 0
7 0
8 0
9 0
U-RIB:ASP-CA
3 0
4 0

5 0
6 4
7 11
8 20
9 31
FMU-RIB:ILE-CA
3 0
4 0
5 0
6 0
7 0
8 0
9 0
H2U-P:TRP-S1
3 0
4 0
5 0
6 0
7 0
8 0
9 0
FMU-RIB:HIS-S1
3 0
4 0
5 0
6 0
7 0
8 0
9 0
FHU-P:LYS-S2
3 0
4 0
5 0
6 0
7 0
8 0
9 0
G-R5:TYR-S2
3 0
4 0
5 0
6 1
7 3
8 7
9 13
G-RIB:PHE-S1
3 0
4 0
5 0
6 1
7 4
8 10
9 17
A-R5:TRP-S2
3 0
4 0
5 0
6 0

7 1
8 2
9 5
FMU-MY:PHE-S1
3 0
4 0
5 0
6 0
7 0
8 0
9 0
FHU-P:ASP-S1
3 0
4 0
5 0
6 0
7 0
8 0
9 0
QUO-P:PHE-S2
3 0
4 0
5 0
6 0
7 0
8 0
9 0
U34-P:ASN-CA
3 0
4 0
5 0
6 0
7 0
8 0
9 0
H2U-RIB:GLU-S2
3 0
4 0
5 0
6 0
7 0
8 0
9 0
FHU-MY:GLY-CA
3 0
4 0
5 0
6 0
7 0
8 0
9 0
U31-P:SER-S1
3 0
4 0
5 0
6 0
7 0
8 0

9 0
FHU-RIB:ILE-S1
3 0
4 0
5 0
6 0
7 0
8 0
9 0
IU-MY:MET-CA
3 0
4 0
5 0
6 0
7 0
8 0
9 0
U31-RIB:ASN-S2
3 0
4 0
5 0
6 0
7 0
8 0
9 0
DA-RIB:GLN-S1
3 0
4 0
5 0
6 0
7 0
8 0
9 0
U31-RIB:GLU-S2
3 0
4 0
5 0
6 0
7 0
8 0
9 0
U-RIB:ASP-S1
3 0
4 0
5 2
6 7
7 14
8 24
9 34
FHU-P:VAL-S1
3 0
4 0
5 0
6 0
7 0
8 0
9 0
A-RIB:LYS-S1

3   0
4   0
5   3
6   11
7   24
8   40
9   58
FHU-MY:ASP-CA
3   0
4   0
5   0
6   0
7   0
8   0
9   0
C-RIB:SER-S1
3   0
4   0
5   4
6   11
7   19
8   29
9   39
U-RIB:GLU-S2
3   0
4   0
5   5
6   15
7   27
8   39
9   51
C-P:LEU-S1
3   0
4   0
5   1
6   4
7   9
8   17
9   27
G-R5:GLY-CA
3   0
4   0
5   2
6   9
7   22
8   40
9   67
A-RIB:TRP-CA
3   0
4   0
5   0
6   0
7   2
8   3
9   6
FMU-MY:SER-CA
3   0
4   0

5 0
6 0
7 0
8 0
9 0
GTP-M5:SER-CA
3 0
4 0
5 0
6 0
7 0
8 0
9 0
U-RIB:THR-CA
3 0
4 0
5 0
6 2
7 7
8 13
9 22
G-P:ILE-S1
3 0
4 0
5 2
6 6
7 11
8 18
9 29
G-R5:ASN-CA
3 0
4 0
5 0
6 1
7 6
8 13
9 26
DA-M5:SER-S1
3 0
4 0
5 0
6 0
7 0
8 0
9 0
IU-RIB:ALA-S1
3 0
4 0
5 0
6 0
7 0
8 0
9 0
IU-MY:PRO-S1
3 0
4 0
5 0
6 0

7 0
8 0
9 0
IU-MY:LYS-S2
3 0
4 0
5 0
6 0
7 0
8 0
9 0
I-RIB:ALA-S1
3 0
4 0
5 0
6 0
7 0
8 0
9 0
G-RIB:ASN-S2
3 0
4 1
5 4
6 12
7 22
8 34
9 45
FMU-MY:HIS-S1
3 0
4 0
5 0
6 0
7 0
8 0
9 0
G-R6:HIS-CA
3 0
4 0
5 0
6 1
7 4
8 8
9 14
FHU-RIB:SER-S1
3 0
4 0
5 0
6 0
7 0
8 0
9 0
U34-P:ASN-S1
3 0
4 0
5 0
6 0
7 0
8 0

9 0
QUO-M5:LEU-CA
3 0
4 0
5 0
6 0
7 0
8 0
9 0
U-P:TRP-S1
3 0
4 0
5 0
6 0
7 1
8 2
9 3
A-P:TRP-S2
3 0
4 0
5 0
6 1
7 3
8 5
9 7
G-R6:ASP-S1
3 0
4 0
5 2
6 8
7 19
8 32
9 51
A-RIB:TRP-S1
3 0
4 0
5 0
6 1
7 2
8 4
9 6
U34-RIB:ASN-CA
3 0
4 0
5 0
6 0
7 0
8 0
9 0
5BU-P:PRO-S1
3 0
4 0
5 0
6 0
7 0
8 0
9 0
A-R5:SER-CA

3 0
4 0
5 0
6 3
7 7
8 16
9 28
C-Y:VAL-S1
3 0
4 0
5 0
6 2
7 5
8 10
9 18
A-R5:HIS-S2
3 0
4 0
5 0
6 2
7 5
8 9
9 15
4SU-P:THR-S1
3 0
4 0
5 0
6 0
7 0
8 0
9 0
A-RIB:ALA-S1
3 0
4 1
5 5
6 10
7 18
8 28
9 42
H2U-RIB:ASN-S1
3 0
4 0
5 0
6 0
7 0
8 0
9 0
C-RIB:TRP-S2
3 0
4 0
5 0
6 1
7 2
8 4
9 6
G-P:ASN-CA
3 0
4 0

5   1
6   7
7   16
8   27
9   40
U31-MY:PHE-CA
3   0
4   0
5   0
6   0
7   0
8   0
9   0
A-R5:SER-S1
3   0
4   0
5   1
6   5
7   11
8   21
9   32
U-Y:PHE-CA
3   0
4   0
5   0
6   0
7   1
8   2
9   5
FHU-RIB:SER-CA
3   0
4   0
5   0
6   0
7   0
8   0
9   0
G-RIB:PHE-CA
3   0
4   0
5   0
6   2
7   5
8   10
9   19
DA-M5:LYS-S1
3   0
4   0
5   0
6   0
7   0
8   0
9   0
C-RIB:THR-CA
3   0
4   0
5   0
6   4

7 11
8 20
9 30
GTP-M5:THR-CA
3 0
4 0
5 0
6 0
7 0
8 0
9 0
U-RIB:ARG-S1
3 0
4 0
5 1
6 6
7 13
8 23
9 36
U-P:MET-S1
3 0
4 0
5 0
6 1
7 2
8 4
9 6
U31-P:TYR-S1
3 0
4 0
5 0
6 0
7 0
8 0
9 0
C-P:PHE-S2
3 0
4 0
5 0
6 2
7 5
8 8
9 12
FMU-RIB:PHE-S1
3 0
4 0
5 0
6 0
7 0
8 0
9 0
FHU-MY:LYS-S1
3 0
4 0
5 0
6 0
7 0
8 0

9   0
G-R5:PHE-S2
3   0
4   0
5   0
6   1
7   2
8   5
9   9
C31-P:ASP-S2
3   0
4   0
5   0
6   0
7   0
8   0
9   0
C-Y:PHE-S1
3   0
4   0
5   0
6   0
7   1
8   2
9   5
C-RIB:CYS-CA
3   0
4   0
5   0
6   0
7   0
8   1
9   3
H2U-MY:ARG-S2
3   0
4   0
5   0
6   0
7   0
8   0
9   0
G-R5:LEU-CA
3   0
4   0
5   0
6   1
7   3
8   8
9   19
G-R5:ARG-S2
3   0
4   0
5   3
6   11
7   27
8   50
9   79
A-RIB:ILE-CA

3 0
4 0
5 0
6 2
7 6
8 12
9 21
U34-P:TYR-S2
3 0
4 0
5 0
6 0
7 0
8 0
9 0
A-P:GLY-CA
3 0
4 1
5 6
6 16
7 28
8 43
9 61
DA-RIB:ASP-S2
3 0
4 0
5 0
6 0
7 0
8 0
9 0
C-P:CYS-S1
3 0
4 0
5 0
6 0
7 1
8 1
9 3
FHU-RIB:PRO-CA
3 0
4 0
5 0
6 0
7 0
8 0
9 0
G-P:LEU-S2
3 0
4 1
5 3
6 8
7 15
8 24
9 37
FMU-MY:ASN-S1
3 0
4 0

5 0
6 0
7 0
8 0
9 0
A-R6:SER-CA
3 0
4 0
5 1
6 5
7 11
8 18
9 28
G-R6:GLU-CA
3 0
4 0
5 0
6 4
7 13
8 30
9 55
IU-RIB:LEU-S2
3 0
4 0
5 0
6 0
7 0
8 0
9 0
U-RIB:ASN-CA
3 0
4 0
5 0
6 2
7 6
8 12
9 18
DA-M5:SER-CA
3 0
4 0
5 0
6 0
7 0
8 0
9 0
U31-MY:GLN-S2
3 0
4 0
5 0
6 0
7 0
8 0
9 0
A-RIB:ALA-CA
3 0
4 0
5 2
6 7

7 16
8 26
9 41
C31-P:GLN-S1
3 0
4 0
5 0
6 0
7 0
8 0
9 0
U-P:LEU-S2
3 0
4 0
5 0
6 3
7 6
8 10
9 16
FHU-RIB:LYS-CA
3 0
4 0
5 0
6 0
7 0
8 0
9 0
A-RIB:LEU-CA
3 0
4 0
5 0
6 2
7 7
8 15
9 27
U34-P:PRO-S1
3 0
4 0
5 0
6 0
7 0
8 0
9 0
A-R5:ALA-S1
3 0
4 0
5 0
6 6
7 12
8 21
9 33
FMU-MY:ALA-S1
3 0
4 0
5 0
6 0
7 0
8 0

9 0
C-Y:GLN-S1
3 0
4 0
5 0
6 1
7 4
8 9
9 15
A-R6:LYS-S1
3 0
4 0
5 2
6 8
7 18
8 30
9 45
U-RIB:ARG-CA
3 0
4 0
5 0
6 3
7 8
8 17
9 28
H2U-MY:GLN-S1
3 0
4 0
5 0
6 0
7 0
8 0
9 0
C-P:CYS-CA
3 0
4 0
5 0
6 0
7 1
8 1
9 3
FHU-MY:ILE-S1
3 0
4 0
5 0
6 0
7 0
8 0
9 0
U31-RIB:PHE-S1
3 0
4 0
5 0
6 0
7 0
8 0
9 0
GTP-M5:ASP-S2

3 0
4 0
5 0
6 0
7 0
8 0
9 0
C-RIB:GLN-S1
3 0
4 0
5 1
6 4
7 10
8 16
9 25
C-P:GLN-CA
3 0
4 0
5 0
6 2
7 7
8 13
9 21
U-P:GLU-S1
3 0
4 0
5 3
6 10
7 20
8 33
9 47
U-Y:GLN-S1
3 0
4 0
5 0
6 0
7 4
8 8
9 13
DA-M6:HIS-CA
3 0
4 0
5 0
6 0
7 0
8 0
9 0
U31-MY:ALA-CA
3 0
4 0
5 0
6 0
7 0
8 0
9 0
A-R5:ILE-S1
3 0
4 0

5 0
6 1
7 3
8 7
9 12
C-Y:LEU-CA
3 0
4 0
5 0
6 0
7 2
8 6
9 14
FHU-P:GLY-CA
3 0
4 0
5 0
6 0
7 0
8 0
9 0
C31-P:GLU-CA
3 0
4 0
5 0
6 0
7 0
8 0
9 0
C-RIB:HIS-S1
3 0
4 0
5 0
6 2
7 5
8 10
9 15
U-RIB:ASP-S2
3 0
4 0
5 3
6 10
7 18
8 27
9 36
C-Y:PHE-CA
3 0
4 0
5 0
6 0
7 1
8 3
9 6
G-R6:VAL-S1
3 0
4 0
5 1
6 4

7   9
8   16
9   27
C-RIB:CYS-S1
3   0
4   0
5   0
6   0
7   0
8   1
9   2
QUO-M5:GLU-S2
3   0
4   0
5   0
6   0
7   0
8   0
9   0
A-R5:VAL-CA
3   0
4   0
5   0
6   1
7   4
8   10
9   20
5BU-MY:PRO-CA
3   0
4   0
5   0
6   0
7   0
8   0
9   0
GTP-M5:THR-S1
3   0
4   0
5   0
6   0
7   0
8   0
9   0
G-RIB:GLU-S1
3   0
4   0
5   5
6   20
7   42
8   72
9   107
U-Y:CYS-CA
3   0
4   0
5   0
6   0
7   0
8   0

9   1
U-Y:MET-S1
3   0
4   0
5   0
6   0
7   1
8   2
9   4
A-R5:GLU-CA
3   0
4   0
5   0
6   2
7   9
8   22
9   42
U-RIB:TRP-S2
3   0
4   0
5   0
6   0
7   1
8   2
9   4
A-R5:TYR-S1
3   0
4   0
5   0
6   0
7   1
8   3
9   7
QUO-M6:ARG-CA
3   0
4   0
5   0
6   0
7   0
8   0
9   0
EXPECTED_ANG
U-RIB:U-Y:GLN-CA U-RIB:U-Y:GLN-CA_20 0.0
20   0
40   0
60   1
80   1
100   0
120   1
140   1
160   0
180   0
200   0
220   0
240   1
260   1
280   1
300   2

320   1
340   0
360   0
U-RIB:U-Y:VAL-CA U-RIB:U-Y:VAL-CA_140 -2634.41595178
20   0
40   0
60   1
80   2
100   2
120   0
140   2
160   1
180   0
200   0
220   0
240   1
260   1
280   2
300   2
320   1
340   1
360   0
GTP-RIB:GTP-M6:THR-S1 GTP-RIB:GTP-M6:THR-S1_160 0.0
20   0
40   0
60   0
80   0
100   0
120   0
140   0
160   0
180   0
200   0
220   0
240   0
260   0
280   0
300   0
320   0
340   0
360   0
C-P:C-RIB:ARG-S2 C-P:C-RIB:ARG-S2_320 -4295.4655249
20   0
40   4
60   11
80   15
100   15
120   14
140   13
160   10
180   5
200   0
220   4
240   11
260   15
280   15
300   14
320   13

340 11
360 5
G-P:G-RIB:ARG-S1 G-P:G-RIB:ARG-S1_180 -1423.38559066
20 0
40 4
60 11
80 15
100 16
120 15
140 13
160 10
180 4
200 0
220 4
240 10
260 14
280 16
300 15
320 13
340 10
360 4
FMU-RIB:FMU-MY:PRO-S1 FMU-RIB:FMU-MY:PRO-S1_60 0.0
20 0
40 0
60 0
80 0
100 0
120 0
140 0
160 0
180 0
200 0
220 0
240 0
260 0
280 0
300 0
320 0
340 0
360 0
U-RIB:U-P:ASN-CA U-RIB:U-P:ASN-CA_160 -3701.40432159
20 0
40 0
60 1
80 3
100 4
120 4
140 3
160 2
180 1
200 0
220 0
240 1
260 2
280 4
300 4
320 3
340 2

360 0
A-RIB:A-P:PRO-CA A-RIB:A-P:PRO-CA_320 -5012.56435953
20 0
40 0
60 3
80 5
100 7
120 7
140 6
160 4
180 1
200 0
220 0
240 2
260 5
280 7
300 7
320 6
340 4
360 1
H2U-P:H2U-RIB:ASN-S1 H2U-P:H2U-RIB:ASN-S1_20 0.0
20 0
40 0
60 0
80 0
100 0
120 0
140 0
160 0
180 0
200 0
220 0
240 0
260 0
280 0
300 0
320 0
340 0
360 0
IU-RIB:IU-MY:TYR-CA IU-RIB:IU-MY:TYR-CA_260 0.0
20 0
40 0
60 0
80 0
100 0
120 0
140 0
160 0
180 0
200 0
220 0
240 0
260 0
280 0
300 0
320 0
340 0
360 0

C-P:C-RIB:MET-S2 C-P:C-RIB:MET-S2_80 -4939.64447582
20 0
40 0
60 1
80 2
100 2
120 2
140 2
160 1
180 0
200 0
220 0
240 1
260 2
280 2
300 2
320 1
340 1
360 0
QUO-RIB:QUO-M6:LEU-CA QUO-RIB:QUO-M6:LEU-CA_100 0.0
20 0
40 0
60 0
80 0
100 0
120 0
140 0
160 0
180 0
200 0
220 0
240 0
260 0
280 0
300 0
320 0
340 0
360 0
G-RIB:G-P:SER-CA G-RIB:G-P:SER-CA_320 -3029.78641188
20 0
40 2
60 5
80 10
100 13
120 13
140 11
160 8
180 3
200 0
220 1
240 5
260 10
280 13
300 13
320 11
340 7
360 2
U-RIB:U-P:ALA-S1

20   0
40   1
60   3
80   5
100   5
120   6
140   5
160   3
180   1
200   0
220   1
240   3
260   5
280   6
300   6
320   5
340   4
360   1
C31-RIB:C31-P:GLU-S2 C31-RIB:C31-P:GLU-S2_20 0.0
20   0
40   0
60   0
80   0
100   0
120   0
140   0
160   0
180   0
200   0
220   0
240   0
260   0
280   0
300   0
320   0
340   0
360   0
A-RIB:A-R5:THR-S1 A-RIB:A-R5:THR-S1_280 -1123.7415582
20   0
40   2
60   4
80   5
100   5
120   4
140   3
160   2
180   0
200   0
220   2
240   4
260   5
280   5
300   4
320   3
340   2
360   0
A-P:A-RIB:ASP-CA A-P:A-RIB:ASP-CA_200 0.0
20   0

```
40   0
60   0
80   8
100   9
120   9
140   8
160   6
180   3
200   0
220   1
240   5
260   8
280   9
300   9
320   8
340   7
360   0
5BU-P:5BU-RIB:SER-S1 5BU-P:5BU-RIB:SER-S1_220 0.0
20   0
40   0
60   0
80   0
100   0
120   0
140   0
160   0
180   0
200   0
220   0
240   0
260   0
280   0
300   0
320   0
340   0
360   0
U-P:U-RIB:ASN-S1 U-P:U-RIB:ASN-S1_140 -2408.28624979
20   0
40   0
60   2
80   3
100   3
120   4
140   3
160   2
180   1
200   0
220   1
240   2
260   4
280   4
300   4
320   3
340   3
360   1
C-RIB:C-P:GLU-S2 C-RIB:C-P:GLU-S2_180 -297.10325965
20   0
40   4
```

60   11
80   17
100   21
120   20
140   18
160   13
180   5
200   0
220   4
240   11
260   17
280   21
300   20
320   17
340   12
360   5
FHU-RIB:FHU-MY:CYS-S1 FHU-RIB:FHU-MY:CYS-S1_320 0.0
20   0
40   0
60   0
80   0
100   0
120   0
140   0
160   0
180   0
200   0
220   0
240   0
260   0
280   0
300   0
320   0
340   0
360   0
A-RIB:A-R5:TRP-CA A-RIB:A-R5:TRP-CA_40 -5780.6133903
20   0
40   0
60   0
80   0
100   0
120   0
140   0
160   0
180   0
200   0
220   0
240   0
260   0
280   0
300   0
320   0
340   0
360   0
A-RIB:A-R6:LYS-S2 A-RIB:A-R6:LYS-S2_180 -1149.71208584
20   0
40   4
60   10

80   13
100   12
120   12
140   11
160   7
180   2
200   0
220   4
240   10
260   12
280   13
300   13
320   11
340   8
360   0
C-RIB:C-P:GLN-CA C-RIB:C-P:GLN-CA_200 0.0
20   0
40   0
60   1
80   3
100   4
120   4
140   4
160   2
180   1
200   0
220   0
240   1
260   3
280   4
300   5
320   4
340   2
360   1
C31-RIB:C31-P:ASP-S1 C31-RIB:C31-P:ASP-S1_300 0.0
20   0
40   0
60   0
80   0
100   0
120   0
140   0
160   0
180   0
200   0
220   0
240   0
260   0
280   0
300   0
320   0
340   0
360   0
H2U-RIB:H2U-MY:ARG-S1 H2U-RIB:H2U-MY:ARG-S1_40 0.0
20   0
40   0
60   0
80   0

100 0
120 0
140 0
160 0
180 0
200 0
220 0
240 0
260 0
280 0
300 0
320 0
340 0
360 0
DA-RIB:DA-M5:GLU-S2 DA-RIB:DA-M5:GLU-S2_240 0.0
20 0
40 0
60 0
80 0
100 0
120 0
140 0
160 0
180 0
200 0
220 0
240 0
260 0
280 0
300 0
320 0
340 0
360 0
G-RIB:G-R5:ASP-S2 G-RIB:G-R5:ASP-S2_280 -3714.08278774
20 0
40 7
60 12
80 13
100 10
120 8
140 7
160 5
180 1
200 0
220 7
240 12
260 13
280 10
300 8
320 7
340 5
360 2
FMU-RIB:FMU-P:VAL-S1 FMU-RIB:FMU-P:VAL-S1_220 0.0
20 0
40 0
60 0
80 0
100 0

120 0
140 0
160 0
180 0
200 0
220 0
240 0
260 0
280 0
300 0
320 0
340 0
360 0
U34-RIB:U34-P:HIS-CA U34-RIB:U34-P:HIS-CA_100 0.0
20 0
40 0
60 0
80 0
100 0
120 0
140 0
160 0
180 0
200 0
220 0
240 0
260 0
280 0
300 0
320 0
340 0
360 0
H2U-RIB:H2U-MY:LEU-CA H2U-RIB:H2U-MY:LEU-CA_160 -10958.5480241
20 0
40 0
60 0
80 0
100 0
120 0
140 0
160 0
180 0
200 0
220 0
240 0
260 0
280 0
300 0
320 0
340 0
360 0
IU-P:IU-RIB:LYS-S2 IU-P:IU-RIB:LYS-S2_260 0.0
20 0
40 0
60 0
80 0
100 0
120 0

140 0
160 0
180 0
200 0
220 0
240 0
260 0
280 0
300 0
320 0
340 0
360 0
U-RIB:U-P:MET-CA U-RIB:U-P:MET-CA_120 -4750.57470011
20 0
40 0
60 0
80 0
100 1
120 1
140 1
160 0
180 0
200 0
220 0
240 0
260 0
280 1
300 1
320 1
340 0
360 0
A-RIB:A-R5:ASN-S2 A-RIB:A-R5:ASN-S2_120 -3880.44239172
20 0
40 3
60 5
80 6
100 4
120 4
140 3
160 2
180 0
200 1
220 2
240 4
260 6
280 5
300 4
320 3
340 2
360 0
A-RIB:A-R6:ILE-CA A-RIB:A-R6:ILE-CA_360 -4479.42908716
20 0
40 0
60 1
80 2
100 0
120 3
140 2

160   1
180   0
200   0
220   0
240   1
260   2
280   3
300   2
320   2
340   1
360   0
U34-RIB:U34-MY:ASN-CA U34-RIB:U34-MY:ASN-CA_60 0.0
20   0
40   0
60   0
80   0
100   0
120   0
140   0
160   0
180   0
200   0
220   0
240   0
260   0
280   0
300   0
320   0
340   0
360   0
C-P:C-RIB:THR-CA C-P:C-RIB:THR-CA_140 -1932.10790908
20   0
40   1
60   3
80   5
100   5
120   6
140   5
160   3
180   1
200   0
220   1
240   3
260   5
280   6
300   6
320   5
340   4
360   1
IU-RIB:IU-P:ARG-CA IU-RIB:IU-P:ARG-CA_360 0.0
20   0
40   0
60   0
80   0
100   0
120   0
140   0
160   0

180   0
200   0
220   0
240   0
260   0
280   0
300   0
320   0
340   0
360   0
FHU-RIB:FHU-P:LYS-S1 FHU-RIB:FHU-P:LYS-S1_40 0.0
20   0
40   0
60   0
80   0
100   0
120   0
140   0
160   0
180   0
200   0
220   0
240   0
260   0
280   0
300   0
320   0
340   0
360   0
C-RIB:C-P:HIS-S2 C-RIB:C-P:HIS-S2_340 -1953.81856415
20   0
40   0
60   2
80   3
100   4
120   4
140   3
160   2
180   0
200   0
220   0
240   2
260   3
280   4
300   4
320   3
340   2
360   1
G-RIB:G-R5:PRO-S1 G-RIB:G-R5:PRO-S1_140 -207.942616763
20   1
40   4
60   6
80   6
100   6
120   4
140   3
160   2
180   0

200   1
220   3
240   6
260   6
280   5
300   4
320   3
340   2
360   0
C-RIB:C-P:LYS-S2 C-RIB:C-P:LYS-S2_220 -3572.38362745
20   0
40   4
60   9
80   15
100   18
120   17
140   15
160   12
180   4
200   0
220   4
240   9
260   15
280   18
300   17
320   15
340   12
360   4
U-P:U-RIB:CYS-S1 U-P:U-RIB:CYS-S1_240 0.0
20   0
40   0
60   0
80   0
100   0
120   0
140   0
160   0
180   0
200   0
220   0
240   0
260   0
280   0
300   0
320   0
340   0
360   0
C-P:C-RIB:VAL-CA C-P:C-RIB:VAL-CA_60 -2818.03421229
20   0
40   0
60   2
80   4
100   5
120   6
140   5
160   4
180   1
200   0

220   0
240   2
260   4
280   6
300   6
320   5
340   4
360   0
G-RIB:G-P:VAL-S1 G-RIB:G-P:VAL-S1_120 -2832.67234875
20   0
40   1
60   4
80   8
100   10
120   11
140   10
160   6
180   2
200   0
220   1
240   4
260   7
280   10
300   10
320   10
340   7
360   2
FHU-P:FHU-RIB:ILE-S1 FHU-P:FHU-RIB:ILE-S1_320 0.0
20   0
40   0
60   0
80   0
100   0
120   0
140   0
160   0
180   0
200   0
220   0
240   0
260   0
280   0
300   0
320   0
340   0
360   0
C-RIB:C-P:PHE-S1 C-RIB:C-P:PHE-S1_320 0.0
20   0
40   0
60   0
80   1
100   0
120   3
140   2
160   1
180   0
200   0
220   0

240   0
260   2
280   2
300   3
320   0
340   1
360   0
U-RIB:U-P:CYS-S1 U-RIB:U-P:CYS-S1_140 -4998.55275203
20   0
40   0
60   0
80   0
100   0
120   0
140   0
160   0
180   0
200   0
220   0
240   0
260   0
280   0
300   0
320   0
340   0
360   0
C31-P:C31-RIB:LEU-S2 C31-P:C31-RIB:LEU-S2_40 0.0
20   0
40   0
60   0
80   0
100   0
120   0
140   0
160   0
180   0
200   0
220   0
240   0
260   0
280   0
300   0
320   0
340   0
360   0
G-RIB:G-R6:ASP-S2 G-RIB:G-R6:ASP-S2_100 -2181.51735548
20   0
40   4
60   10
80   13
100   13
120   11
140   8
160   5
180   2
200   0
220   4
240   10

260 13
280 13
300 12
320 8
340 5
360 1
U31-RIB:U31-P:MET-CA U31-RIB:U31-P:MET-CA_80 0.0
20 0
40 0
60 0
80 0
100 0
120 0
140 0
160 0
180 0
200 0
220 0
240 0
260 0
280 0
300 0
320 0
340 0
360 0
FMU-P:FMU-RIB:VAL-S1 FMU-P:FMU-RIB:VAL-S1_100 0.0
20 0
40 0
60 0
80 0
100 0
120 0
140 0
160 0
180 0
200 0
220 0
240 0
260 0
280 0
300 0
320 0
340 0
360 0
G-RIB:G-P:MET-S1 G-RIB:G-P:MET-S1_300 -103.727468329
20 0
40 0
60 1
80 2
100 3
120 3
140 3
160 2
180 0
200 0
220 0
240 1
260 2

280   3
300   3
320   3
340   2
360   0
H2U-P:H2U-RIB:LEU-S2 H2U-P:H2U-RIB:LEU-S2_200 0.0
20   0
40   0
60   0
80   0
100   0
120   0
140   0
160   0
180   0
200   0
220   0
240   0
260   0
280   0
300   0
320   0
340   0
360   0
U-RIB:U-Y:THR-S1 U-RIB:U-Y:THR-S1_120 -3789.40185486
20   0
40   1
60   2
80   3
100   3
120   3
140   2
160   1
180   0
200   0
220   1
240   3
260   3
280   3
300   3
320   2
340   0
360   0
G-RIB:G-R6:ILE-S1 G-RIB:G-R6:ILE-S1_280 -2142.789278
20   0
40   0
60   2
80   0
100   3
120   3
140   0
160   1
180   0
200   0
220   0
240   2
260   3
280   3

```
300   3
320   0
340   0
360   0
FHU-RIB:FHU-MY:TYR-CA FHU-RIB:FHU-MY:TYR-CA_80 0.0
20   0
40   0
60   0
80   0
100   0
120   0
140   0
160   0
180   0
200   0
220   0
240   0
260   0
280   0
300   0
320   0
340   0
360   0
IU-RIB:IU-MY:PRO-CA IU-RIB:IU-MY:PRO-CA_100 0.0
20   0
40   0
60   0
80   0
100   0
120   0
140   0
160   0
180   0
200   0
220   0
240   0
260   0
280   0
300   0
320   0
340   0
360   0
QUO-RIB:QUO-M6:GLU-S1 QUO-RIB:QUO-M6:GLU-S1_160 -3434.83908349
20   0
40   0
60   0
80   0
100   0
120   0
140   0
160   0
180   0
200   0
220   0
240   0
260   0
280   0
300   0
```

320   0
340   0
360   0
U34-RIB:U34-P:HIS-S1 U34-RIB:U34-P:HIS-S1_360 0.0
20   0
40   0
60   0
80   0
100   0
120   0
140   0
160   0
180   0
200   0
220   0
240   0
260   0
280   0
300   0
320   0
340   0
360   0
QUO-P:QUO-RIB:PHE-S1 QUO-P:QUO-RIB:PHE-S1_100 0.0
20   0
40   0
60   0
80   0
100   0
120   0
140   0
160   0
180   0
200   0
220   0
240   0
260   0
280   0
300   0
320   0
340   0
360   0
A-P:A-RIB:MET-CA A-P:A-RIB:MET-CA_360 0.0
20   0
40   0
60   1
80   1
100   1
120   2
140   1
160   1
180   0
200   0
220   0
240   1
260   1
280   2
300   2
320   1

340   1
360   0
A-P:A-RIB:ILE-CA A-P:A-RIB:ILE-CA_140 -2774.63164431
20   0
40   0
60   1
80   3
100   3
120   4
140   3
160   2
180   1
200   0
220   0
240   0
260   3
280   3
300   4
320   3
340   2
360   1
C-RIB:C-Y:LYS-S1 C-RIB:C-Y:LYS-S1_60 -3915.94480219
20   1
40   3
60   6
80   6
100   5
120   5
140   4
160   3
180   1
200   1
220   4
240   6
260   7
280   6
300   5
320   4
340   3
360   1
G-RIB:G-R6:GLU-CA G-RIB:G-R6:GLU-CA_180 -3660.35899881
20   0
40   2
60   6
80   10
100   11
120   9
140   6
160   5
180   1
200   0
220   1
240   6
260   10
280   11
300   8
320   6
340   5

360 1
G-RIB:G-P:LYS-S1 G-RIB:G-P:LYS-S1_40 0.0
20 0
40 0
60 9
80 16
100 23
120 23
140 19
160 14
180 5
200 0
220 2
240 8
260 17
280 23
300 23
320 20
340 14
360 5
G-RIB:G-R6:GLN-S1 G-RIB:G-R6:GLN-S1_320 -2095.44068951
20 0
40 1
60 3
80 4
100 5
120 4
140 0
160 2
180 0
200 0
220 1
240 3
260 4
280 5
300 4
320 3
340 0
360 0
A-RIB:A-R6:PHE-S1 A-RIB:A-R6:PHE-S1_200 0.0
20 0
40 0
60 0
80 0
100 1
120 1
140 0
160 0
180 0
200 0
220 0
240 0
260 1
280 1
300 1
320 0
340 0
360 0

G-RIB:G-R6:ASN-S2 G-RIB:G-R6:ASN-S2_320 -3676.14933179
20 0
40 0
60 6
80 7
100 7
120 7
140 4
160 3
180 1
200 0
220 2
240 6
260 8
280 7
300 6
320 4
340 3
360 0
FMU-RIB:FMU-P:PHE-S1 FMU-RIB:FMU-P:PHE-S1_320 0.0
20 0
40 0
60 0
80 0
100 0
120 0
140 0
160 0
180 0
200 0
220 0
240 0
260 0
280 0
300 0
320 0
340 0
360 0
C31-RIB:C31-MY:LEU-S2 C31-RIB:C31-MY:LEU-S2_180 0.0
20 0
40 0
60 0
80 0
100 0
120 0
140 0
160 0
180 0
200 0
220 0
240 0
260 0
280 0
300 0
320 0
340 0
360 0
U34-RIB:U34-MY:PHE-CA U34-RIB:U34-MY:PHE-CA_60 0.0

20 0
40 0
60 0
80 0
100 0
120 0
140 0
160 0
180 0
200 0
220 0
240 0
260 0
280 0
300 0
320 0
340 0
360 0
C31-RIB:C31-MY:GLU-S2 C31-RIB:C31-MY:GLU-S2_180 0.0
20 0
40 0
60 0
80 0
100 0
120 0
140 0
160 0
180 0
200 0
220 0
240 0
260 0
280 0
300 0
320 0
340 0
360 0
H2U-RIB:H2U-MY:PHE-S2 H2U-RIB:H2U-MY:PHE-S2_320 0.0
20 0
40 0
60 0
80 0
100 0
120 0
140 0
160 0
180 0
200 0
220 0
240 0
260 0
280 0
300 0
320 0
340 0
360 0
A-RIB:A-P:ARG-CA A-RIB:A-P:ARG-CA_340 -5515.98680529
20 0

40 0
60 3
80 7
100 10
120 11
140 9
160 6
180 2
200 0
220 0
240 3
260 7
280 10
300 11
320 9
340 6
360 2
A-RIB:A-R6:GLN-S2 A-RIB:A-R6:GLN-S2_100 -3912.76881445
20 0
40 1
60 3
80 5
100 5
120 5
140 4
160 3
180 1
200 0
220 1
240 3
260 5
280 5
300 5
320 4
340 3
360 1
U-RIB:U-P:ARG-S2 U-RIB:U-P:ARG-S2_340 -5058.89576871
20 0
40 2
60 5
80 9
100 11
120 11
140 9
160 7
180 3
200 0
220 2
240 5
260 9
280 12
300 11
320 10
340 7
360 3
A-P:A-RIB:ARG-S1 A-P:A-RIB:ARG-S1_360 -5143.77814009
20 0
40 2

```
60   6
80   10
100   12
120   11
140   10
160   8
180   3
200   0
220   2
240   6
260   10
280   11
300   11
320   10
340   8
360   3
FHU-RIB:FHU-P:LEU-S1 FHU-RIB:FHU-P:LEU-S1_140 0.0
20   0
40   0
60   0
80   0
100   0
120   0
140   0
160   0
180   0
200   0
220   0
240   0
260   0
280   0
300   0
320   0
340   0
360   0
DA-RIB:DA-M5:GLU-S1 DA-RIB:DA-M5:GLU-S1_60 0.0
20   0
40   0
60   0
80   0
100   0
120   0
140   0
160   0
180   0
200   0
220   0
240   0
260   0
280   0
300   0
320   0
340   0
360   0
FHU-RIB:FHU-P:ASP-S2 FHU-RIB:FHU-P:ASP-S2_300 0.0
20   0
40   0
60   0
```

80   0
100   0
120   0
140   0
160   0
180   0
200   0
220   0
240   0
260   0
280   0
300   0
320   0
340   0
360   0
C-RIB:C-P:ARG-CA C-RIB:C-P:ARG-CA_360 -5331.30686418
20   0
40   1
60   3
80   7
100   10
120   11
140   10
160   6
180   2
200   0
220   1
240   3
260   7
280   10
300   11
320   10
340   6
360   2
QUO-P:QUO-RIB:PHE-S2 QUO-P:QUO-RIB:PHE-S2_260 0.0
20   0
40   0
60   0
80   0
100   0
120   0
140   0
160   0
180   0
200   0
220   0
240   0
260   0
280   0
300   0
320   0
340   0
360   0
U-P:U-RIB:ILE-S1 U-P:U-RIB:ILE-S1_240 -3342.43084561
20   0
40   0
60   1
80   2

100 2
120 2
140 2
160 1
180 0
200 0
220 0
240 1
260 2
280 2
300 0
320 2
340 1
360 0
FMU-P:FMU-RIB:GLN-CA FMU-P:FMU-RIB:GLN-CA_260 0.0
20 0
40 0
60 0
80 0
100 0
120 0
140 0
160 0
180 0
200 0
220 0
240 0
260 0
280 0
300 0
320 0
340 0
360 0
G-RIB:G-P:PHE-S1 G-RIB:G-P:PHE-S1_120 -1294.75570587
20 0
40 0
60 1
80 2
100 3
120 4
140 4
160 3
180 0
200 0
220 0
240 1
260 2
280 4
300 4
320 3
340 2
360 1
QUO-RIB:QUO-M6:GLU-S2 QUO-RIB:QUO-M6:GLU-S2_240 0.0
20 0
40 0
60 0
80 0
100 0

120 0
140 0
160 0
180 0
200 0
220 0
240 0
260 0
280 0
300 0
320 0
340 0
360 0
FHU-P:FHU-RIB:SER-CA FHU-P:FHU-RIB:SER-CA_120 0.0
20 0
40 0
60 0
80 0
100 0
120 0
140 0
160 0
180 0
200 0
220 0
240 0
260 0
280 0
300 0
320 0
340 0
360 0
5BU-RIB:5BU-P:PRO-S1 5BU-RIB:5BU-P:PRO-S1_20 0.0
20 0
40 0
60 0
80 0
100 0
120 0
140 0
160 0
180 0
200 0
220 0
240 0
260 0
280 0
300 0
320 0
340 0
360 0
G-P:G-RIB:TYR-S1 G-P:G-RIB:TYR-S1_200 0.0
20 0
40 0
60 1
80 2
100 3
120 3

140   2
160   1
180   0
200   0
220   0
240   1
260   2
280   3
300   3
320   0
340   2
360   0
C-RIB:C-Y:GLU-CA C-RIB:C-Y:GLU-CA_180 -2506.24991423
20   0
40   2
60   5
80   5
100   0
120   5
140   4
160   3
180   1
200   0
220   2
240   5
260   5
280   5
300   5
320   0
340   2
360   0
U-RIB:U-Y:ASP-S1 U-RIB:U-Y:ASP-S1_320 0.0
20   0
40   2
60   4
80   4
100   4
120   4
140   3
160   2
180   0
200   0
220   2
240   4
260   4
280   0
300   4
320   0
340   0
360   0
U31-RIB:U31-P:GLN-CA U31-RIB:U31-P:GLN-CA_340 0.0
20   0
40   0
60   0
80   0
100   0
120   0
140   0

160   0
180   0
200   0
220   0
240   0
260   0
280   0
300   0
320   0
340   0
360   0
G-RIB:G-R6:LYS-CA G-RIB:G-R6:LYS-CA_300 -1021.71912952
20   0
40   1
60   5
80   8
100   8
120   7
140   6
160   4
180   1
200   0
220   1
240   4
260   8
280   8
300   7
320   5
340   4
360   1
5BU-P:5BU-RIB:PRO-S1 5BU-P:5BU-RIB:PRO-S1_40 0.0
20   0
40   0
60   0
80   0
100   0
120   0
140   0
160   0
180   0
200   0
220   0
240   0
260   0
280   0
300   0
320   0
340   0
360   0
DA-RIB:DA-M5:MET-S2 DA-RIB:DA-M5:MET-S2_200 0.0
20   0
40   0
60   0
80   0
100   0
120   0
140   0
160   0

180   0
200   0
220   0
240   0
260   0
280   0
300   0
320   0
340   0
360   0
C31-RIB:C31-P:ASP-CA C31-RIB:C31-P:ASP-CA_200 0.0
20   0
40   0
60   0
80   0
100   0
120   0
140   0
160   0
180   0
200   0
220   0
240   0
260   0
280   0
300   0
320   0
340   0
360   0
A-RIB:A-R6:VAL-CA A-RIB:A-R6:VAL-CA_220 0.0
20   0
40   0
60   2
80   3
100   4
120   4
140   3
160   2
180   0
200   0
220   0
240   2
260   3
280   4
300   4
320   4
340   2
360   0
A-RIB:A-R6:VAL-S1 A-RIB:A-R6:VAL-S1_20 0.0
20   0
40   0
60   2
80   3
100   4
120   4
140   3
160   2
180   0

200 0
220 0
240 2
260 3
280 4
300 4
320 3
340 2
360 0
DA-RIB:DA-M5:ASP-S2 DA-RIB:DA-M5:ASP-S2_60 0.0
20 0
40 0
60 0
80 0
100 0
120 0
140 0
160 0
180 0
200 0
220 0
240 0
260 0
280 0
300 0
320 0
340 0
360 0
U-RIB:U-P:ASP-S2 U-RIB:U-P:ASP-S2_220 -4552.25187029
20 0
40 0
60 4
80 7
100 9
120 9
140 8
160 6
180 2
200 0
220 1
240 4
260 7
280 9
300 8
320 7
340 5
360 2
IU-RIB:IU-MY:SER-S1 IU-RIB:IU-MY:SER-S1_320 0.0
20 0
40 0
60 0
80 0
100 0
120 0
140 0
160 0
180 0
200 0

220   0
240   0
260   0
280   0
300   0
320   0
340   0
360   0
U31-RIB:U31-P:ASP-CA U31-RIB:U31-P:ASP-CA_280 0.0
20   0
40   0
60   0
80   0
100   0
120   0
140   0
160   0
180   0
200   0
220   0
240   0
260   0
280   0
300   0
320   0
340   0
360   0
FHU-RIB:FHU-P:TYR-S2 FHU-RIB:FHU-P:TYR-S2_180 0.0
20   0
40   0
60   0
80   0
100   0
120   0
140   0
160   0
180   0
200   0
220   0
240   0
260   0
280   0
300   0
320   0
340   0
360   0
U31-P:U31-RIB:PHE-CA U31-P:U31-RIB:PHE-CA_100 0.0
20   0
40   0
60   0
80   0
100   0
120   0
140   0
160   0
180   0
200   0
220   0

240   0
260   0
280   0
300   0
320   0
340   0
360   0
IU-RIB:IU-MY:LYS-S2 IU-RIB:IU-MY:LYS-S2_320 0.0
20   0
40   0
60   0
80   0
100   0
120   0
140   0
160   0
180   0
200   0
220   0
240   0
260   0
280   0
300   0
320   0
340   0
360   0
G-RIB:G-R5:TRP-S2 G-RIB:G-R5:TRP-S2_120 -7779.03320578
20   0
40   0
60   0
80   0
100   1
120   0
140   0
160   0
180   0
200   0
220   0
240   1
260   1
280   0
300   1
320   0
340   0
360   0
FMU-RIB:FMU-MY:ASN-CA FMU-RIB:FMU-MY:ASN-CA_160 0.0
20   0
40   0
60   0
80   0
100   0
120   0
140   0
160   0
180   0
200   0
220   0
240   0

260 0
280 0
300 0
320 0
340 0
360 0
FMU-RIB:FMU-P:ASP-S2 FMU-RIB:FMU-P:ASP-S2_100 0.0
20 0
40 0
60 0
80 0
100 0
120 0
140 0
160 0
180 0
200 0
220 0
240 0
260 0
280 0
300 0
320 0
340 0
360 0
DA-RIB:DA-M5:SER-S1 DA-RIB:DA-M5:SER-S1_200 0.0
20 0
40 0
60 0
80 0
100 0
120 0
140 0
160 0
180 0
200 0
220 0
240 0
260 0
280 0
300 0
320 0
340 0
360 0
C-RIB:C-Y:MET-CA C-RIB:C-Y:MET-CA_180 0.0
20 0
40 0
60 0
80 1
100 0
120 0
140 0
160 0
180 0
200 0
220 0
240 0
260 0

280   1
300   0
320   0
340   0
360   0
A-RIB:A-R6:PRO-S1 A-RIB:A-R6:PRO-S1_340 -434.949009012
20   0
40   1
60   3
80   5
100   5
120   5
140   4
160   3
180   1
200   0
220   1
240   4
260   5
280   5
300   5
320   4
340   3
360   1
A-P:A-RIB:ASN-S2 A-P:A-RIB:ASN-S2_120 -2188.06250763
20   0
40   2
60   4
80   7
100   6
120   6
140   6
160   5
180   2
200   0
220   2
240   5
260   7
280   7
300   7
320   6
340   5
360   2
G-RIB:G-R5:ILE-CA G-RIB:G-R5:ILE-CA_100 -3748.75946193
20   0
40   0
60   2
80   2
100   2
120   0
140   0
160   0
180   0
200   0
220   1
240   2
260   2
280   2

300   2
320   0
340   1
360   0
U-RIB:U-P:TYR-CA U-RIB:U-P:TYR-CA_20 0.0
20   0
40   0
60   0
80   1
100   1
120   0
140   1
160   1
180   0
200   0
220   0
240   0
260   1
280   1
300   1
320   1
340   0
360   0
OMC-RIB:OMC-P:LYS-S2 OMC-RIB:OMC-P:LYS-S2_160 0.0
20   0
40   0
60   0
80   0
100   0
120   0
140   0
160   0
180   0
200   0
220   0
240   0
260   0
280   0
300   0
320   0
340   0
360   0
IU-RIB:IU-P:ARG-S1 IU-RIB:IU-P:ARG-S1_220 0.0
20   0
40   0
60   0
80   0
100   0
120   0
140   0
160   0
180   0
200   0
220   0
240   0
260   0
280   0
300   0

320 0
340 0
360 0
U34-RIB:U34-P:ASN-CA U34-RIB:U34-P:ASN-CA_280 0.0
20 0
40 0
60 0
80 0
100 0
120 0
140 0
160 0
180 0
200 0
220 0
240 0
260 0
280 0
300 0
320 0
340 0
360 0
U31-RIB:U31-P:ASP-S2 U31-RIB:U31-P:ASP-S2_80 0.0
20 0
40 0
60 0
80 0
100 0
120 0
140 0
160 0
180 0
200 0
220 0
240 0
260 0
280 0
300 0
320 0
340 0
360 0
U-RIB:U-P:SER-S1 U-RIB:U-P:SER-S1_320 -5115.36892163
20 0
40 1
60 3
80 5
100 7
120 7
140 5
160 4
180 1
200 0
220 1
240 3
260 5
280 6
300 6
320 6

340   4
360   1
G-RIB:G-R5:ARG-S2 G-RIB:G-R5:ARG-S2_40 -3739.77612952
20   3
40   8
60   15
80   16
100   13
120   10
140   8
160   6
180   2
200   3
220   9
240   15
260   17
280   13
300   10
320   9
340   6
360   2
A-RIB:A-P:GLN-S1 A-RIB:A-P:GLN-S1_160 -2833.53611472
20   0
40   0
60   2
80   4
100   6
120   6
140   5
160   3
180   0
200   0
220   0
240   2
260   4
280   6
300   6
320   5
340   4
360   0
U31-RIB:U31-MY:VAL-CA U31-RIB:U31-MY:VAL-CA_260 0.0
20   0
40   0
60   0
80   0
100   0
120   0
140   0
160   0
180   0
200   0
220   0
240   0
260   0
280   0
300   0
320   0
340   0

360   0
C-RIB:C-Y:ASN-S1 C-RIB:C-Y:ASN-S1_100 0.0
20   0
40   2
60   3
80   3
100   0
120   2
140   2
160   1
180   0
200   0
220   2
240   4
260   4
280   3
300   2
320   0
340   1
360   0
U34-RIB:U34-P:ASN-S2 U34-RIB:U34-P:ASN-S2_260 -9692.86891111
20   0
40   0
60   0
80   0
100   0
120   0
140   0
160   0
180   0
200   0
220   0
240   0
260   0
280   0
300   0
320   0
340   0
360   0
G-P:G-RIB:ASN-CA G-P:G-RIB:ASN-CA_320 -3464.7514708
20   0
40   1
60   4
80   6
100   7
120   7
140   5
160   4
180   2
200   0
220   1
240   4
260   7
280   8
300   7
320   5
340   4
360   2

IU-RIB:IU-P:ILE-S1 IU-RIB:IU-P:ILE-S1_320 0.0
20 0
40 0
60 0
80 0
100 0
120 0
140 0
160 0
180 0
200 0
220 0
240 0
260 0
280 0
300 0
320 0
340 0
360 0
FMU-RIB:FMU-MY:ARG-S1 FMU-RIB:FMU-MY:ARG-S1_320 0.0
20 0
40 0
60 0
80 0
100 0
120 0
140 0
160 0
180 0
200 0
220 0
240 0
260 0
280 0
300 0
320 0
340 0
360 0
U34-RIB:U34-P:ARG-S2 U34-RIB:U34-P:ARG-S2_100 0.0
20 0
40 0
60 0
80 0
100 0
120 0
140 0
160 0
180 0
200 0
220 0
240 0
260 0
280 0
300 0
320 0
340 0
360 0
I-P:I-RIB:GLY-CA I-P:I-RIB:GLY-CA_120 0.0

20 0
40 0
60 0
80 0
100 0
120 0
140 0
160 0
180 0
200 0
220 0
240 0
260 0
280 0
300 0
320 0
340 0
360 0
FMU-P:FMU-RIB:CYS-CA FMU-P:FMU-RIB:CYS-CA_140 0.0
20 0
40 0
60 0
80 0
100 0
120 0
140 0
160 0
180 0
200 0
220 0
240 0
260 0
280 0
300 0
320 0
340 0
360 0
U34-RIB:U34-P:ARG-S1 U34-RIB:U34-P:ARG-S1_280 0.0
20 0
40 0
60 0
80 0
100 0
120 0
140 0
160 0
180 0
200 0
220 0
240 0
260 0
280 0
300 0
320 0
340 0
360 0
U-RIB:U-P:LEU-S2 U-RIB:U-P:LEU-S2_260 -4827.60824117
20 0

40 0
60 1
80 2
100 3
120 4
140 3
160 2
180 0
200 0
220 0
240 1
260 2
280 3
300 3
320 3
340 2
360 0
H2U-P:H2U-RIB:PHE-CA H2U-P:H2U-RIB:PHE-CA_120 0.0
20 0
40 0
60 0
80 0
100 0
120 0
140 0
160 0
180 0
200 0
220 0
240 0
260 0
280 0
300 0
320 0
340 0
360 0
A-RIB:A-P:GLY-CA A-RIB:A-P:GLY-CA_80 -3072.22857085
20 0
40 2
60 6
80 11
100 15
120 15
140 13
160 9
180 3
200 0
220 2
240 7
260 11
280 15
300 15
320 13
340 9
360 3
A-RIB:A-R6:PHE-CA A-RIB:A-R6:PHE-CA_300 -5215.83610458
20 0
40 0

```
60   0
80   1
100   1
120   1
140   1
160   0
180   0
200   0
220   0
240   0
260   1
280   1
300   1
320   0
340   0
360   0
FMU-RIB:FMU-MY:ARG-CA FMU-RIB:FMU-MY:ARG-CA_40 0.0
20   0
40   0
60   0
80   0
100   0
120   0
140   0
160   0
180   0
200   0
220   0
240   0
260   0
280   0
300   0
320   0
340   0
360   0
FHU-RIB:FHU-MY:LEU-CA FHU-RIB:FHU-MY:LEU-CA_300 0.0
20   0
40   0
60   0
80   0
100   0
120   0
140   0
160   0
180   0
200   0
220   0
240   0
260   0
280   0
300   0
320   0
340   0
360   0
G-RIB:G-R5:MET-S1 G-RIB:G-R5:MET-S1_140 0.0
20   0
40   0
60   1
```

80 1
100 0
120 1
140 0
160 0
180 0
200 0
220 1
240 0
260 1
280 1
300 1
320 0
340 0
360 0
QUO-RIB:QUO-M5:GLU-S2 QUO-RIB:QUO-M5:GLU-S2_120 0.0
20 0
40 0
60 0
80 0
100 0
120 0
140 0
160 0
180 0
200 0
220 0
240 0
260 0
280 0
300 0
320 0
340 0
360 0
A-RIB:A-R5:TRP-S2 A-RIB:A-R5:TRP-S2_360 0.0
20 0
40 0
60 0
80 0
100 0
120 0
140 0
160 0
180 0
200 0
220 0
240 0
260 0
280 0
300 0
320 0
340 0
360 0
FMU-P:FMU-RIB:GLU-S1 FMU-P:FMU-RIB:GLU-S1_240 0.0
20 0
40 0
60 0
80 0

100   0
120   0
140   0
160   0
180   0
200   0
220   0
240   0
260   0
280   0
300   0
320   0
340   0
360   0
H2U-RIB:H2U-MY:GLN-S1 H2U-RIB:H2U-MY:GLN-S1_120 0.0
20   0
40   0
60   0
80   0
100   0
120   0
140   0
160   0
180   0
200   0
220   0
240   0
260   0
280   0
300   0
320   0
340   0
360   0
U-RIB:U-Y:ALA-S1 U-RIB:U-Y:ALA-S1_260 -2337.27442659
20   0
40   2
60   3
80   3
100   3
120   3
140   2
160   1
180   0
200   0
220   0
240   3
260   3
280   3
300   3
320   3
340   2
360   0
U-RIB:U-Y:MET-S2 U-RIB:U-Y:MET-S2_320 -3967.16263592
20   0
40   0
60   0
80   0
100   0

120   0
140   0
160   0
180   0
200   0
220   0
240   0
260   1
280   0
300   0
320   0
340   0
360   0
A-RIB:A-P:LYS-S1 A-RIB:A-P:LYS-S1_40 -5147.30814875
20   0
40   2
60   6
80   10
100   14
120   14
140   13
160   9
180   3
200   0
220   0
240   6
260   10
280   14
300   14
320   12
340   9
360   3
C31-P:C31-RIB:PHE-S1 C31-P:C31-RIB:PHE-S1_360 0.0
20   0
40   0
60   0
80   0
100   0
120   0
140   0
160   0
180   0
200   0
220   0
240   0
260   0
280   0
300   0
320   0
340   0
360   0
C-RIB:C-Y:HIS-CA C-RIB:C-Y:HIS-CA_240 -6289.1964355
20   0
40   0
60   1
80   1
100   1
120   1

140   1
160   0
180   0
200   0
220   0
240   1
260   1
280   1
300   1
320   0
340   0
360   0
FHU-P:FHU-RIB:ALA-CA FHU-P:FHU-RIB:ALA-CA_60 0.0
20   0
40   0
60   0
80   0
100   0
120   0
140   0
160   0
180   0
200   0
220   0
240   0
260   0
280   0
300   0
320   0
340   0
360   0
G-P:G-RIB:LYS-CA G-P:G-RIB:LYS-CA_240 -2130.61087724
20   0
40   3
60   9
80   12
100   14
120   13
140   11
160   8
180   3
200   0
220   2
240   8
260   12
280   14
300   13
320   11
340   8
360   3
A-P:A-RIB:SER-S1 A-P:A-RIB:SER-S1_320 -3447.2587218
20   0
40   2
60   5
80   8
100   8
120   8
140   7

160 6
180 2
200 0
220 2
240 5
260 8
280 8
300 8
320 7
340 6
360 2
G-RIB:G-R5:LEU-S1 G-RIB:G-R5:LEU-S1_240 -1276.0244836
20 0
40 1
60 2
80 2
100 2
120 2
140 2
160 1
180 0
200 0
220 1
240 2
260 2
280 2
300 2
320 2
340 1
360 0
IU-P:IU-RIB:GLN-S1 IU-P:IU-RIB:GLN-S1_360 0.0
20 0
40 0
60 0
80 0
100 0
120 0
140 0
160 0
180 0
200 0
220 0
240 0
260 0
280 0
300 0
320 0
340 0
360 0
A-RIB:A-R5:LEU-CA A-RIB:A-R5:LEU-CA_340 -5998.52579908
20 0
40 1
60 1
80 2
100 2
120 2
140 1
160 0

180 0
200 0
220 1
240 1
260 2
280 2
300 2
320 1
340 1
360 0
H2U-P:H2U-RIB:LYS-S2 H2U-P:H2U-RIB:LYS-S2_300 -7325.38611082
20 0
40 0
60 0
80 0
100 0
120 0
140 0
160 0
180 0
200 0
220 0
240 0
260 0
280 0
300 0
320 0
340 0
360 0
C-RIB:C-P:LYS-S1 C-RIB:C-P:LYS-S1_140 -4482.91800487
20 0
40 2
60 6
80 10
100 14
120 15
140 12
160 9
180 3
200 0
220 1
240 6
260 10
280 14
300 14
320 13
340 9
360 3
C-RIB:C-P:CYS-CA C-RIB:C-P:CYS-CA_140 -3207.71413034
20 0
40 0
60 0
80 0
100 0
120 0
140 0
160 0
180 0

200 0
220 0
240 0
260 0
280 0
300 0
320 0
340 0
360 0
G-P:G-RIB:ASN-S2 G-P:G-RIB:ASN-S2_220 -3825.88181679
20 0
40 3
60 7
80 9
100 9
120 9
140 7
160 6
180 3
200 0
220 3
240 7
260 10
280 10
300 9
320 7
340 6
360 3
A-P:A-RIB:PRO-S1 A-P:A-RIB:PRO-S1_180 -1472.27478076
20 0
40 1
60 4
80 6
100 6
120 7
140 6
160 5
180 2
200 0
220 0
240 4
260 6
280 7
300 7
320 6
340 5
360 2
U34-RIB:U34-P:ASN-S1 U34-RIB:U34-P:ASN-S1_60 -14393.3871076
20 0
40 0
60 0
80 0
100 0
120 0
140 0
160 0
180 0
200 0

220   0
240   0
260   0
280   0
300   0
320   0
340   0
360   0
U-RIB:U-Y:GLN-S2 U-RIB:U-Y:GLN-S2_320 -481.652816045
20   0
40   1
60   3
80   3
100   3
120   2
140   2
160   1
180   0
200   0
220   1
240   3
260   3
280   3
300   3
320   2
340   1
360   0
C-RIB:C-Y:LEU-CA C-RIB:C-Y:LEU-CA_100 -3570.6381447
20   0
40   0
60   1
80   1
100   2
120   2
140   1
160   1
180   0
200   0
220   0
240   1
260   1
280   2
300   2
320   1
340   1
360   0
H2U-RIB:H2U-P:PRO-CA H2U-RIB:H2U-P:PRO-CA_80 0.0
20   0
40   0
60   0
80   0
100   0
120   0
140   0
160   0
180   0
200   0
220   0

240   0
260   0
280   0
300   0
320   0
340   0
360   0
G-RIB:G-P:PRO-S1 G-RIB:G-P:PRO-S1_240 -5026.65565432
20   0
40   2
60   5
80   9
100   12
120   12
140   11
160   7
180   2
200   0
220   1
240   5
260   8
280   11
300   12
320   10
340   7
360   2
C-P:C-RIB:GLN-CA C-P:C-RIB:GLN-CA_260 -4672.36206787
20   0
40   0
60   1
80   3
100   3
120   4
140   3
160   2
180   1
200   0
220   0
240   2
260   3
280   4
300   4
320   3
340   3
360   1
G-RIB:G-P:SER-S1 G-RIB:G-P:SER-S1_360 -2059.62461839
20   0
40   3
60   7
80   12
100   15
120   15
140   12
160   9
180   3
200   0
220   3
240   7

260   12
280   14
300   14
320   12
340   9
360   3
C-RIB:C-Y:ASP-CA C-RIB:C-Y:ASP-CA_140 -3654.39578858
20   0
40   2
60   4
80   5
100   4
120   4
140   3
160   2
180   0
200   0
220   2
240   4
260   4
280   4
300   4
320   3
340   2
360   0
U31-RIB:U31-P:MET-S2 U31-RIB:U31-P:MET-S2_220 0.0
20   0
40   0
60   0
80   0
100   0
120   0
140   0
160   0
180   0
200   0
220   0
240   0
260   0
280   0
300   0
320   0
340   0
360   0
QUO-RIB:QUO-M5:ASN-S1 QUO-RIB:QUO-M5:ASN-S1_40 0.0
20   0
40   0
60   0
80   0
100   0
120   0
140   0
160   0
180   0
200   0
220   0
240   0
260   0

280   0
300   0
320   0
340   0
360   0
A-RIB:A-P:MET-S2 A-RIB:A-P:MET-S2_100 -1216.61389274
20   0
40   0
60   1
80   2
100   2
120   2
140   2
160   1
180   0
200   0
220   0
240   1
260   2
280   2
300   2
320   2
340   1
360   0
H2U-P:H2U-RIB:ARG-S1 H2U-P:H2U-RIB:ARG-S1_300 0.0
20   0
40   0
60   0
80   0
100   0
120   0
140   0
160   0
180   0
200   0
220   0
240   0
260   0
280   0
300   0
320   0
340   0
360   0
U-RIB:U-Y:ASN-S1 U-RIB:U-Y:ASN-S1_140 -4202.18789753
20   0
40   1
60   2
80   3
100   3
120   2
140   2
160   1
180   0
200   0
220   1
240   2
260   2
280   3

300   3
320   2
340   1
360   0
H2U-RIB:H2U-MY:TRP-CA H2U-RIB:H2U-MY:TRP-CA_20 0.0
20   0
40   0
60   0
80   0
100   0
120   0
140   0
160   0
180   0
200   0
220   0
240   0
260   0
280   0
300   0
320   0
340   0
360   0
U-RIB:U-Y:VAL-S1 U-RIB:U-Y:VAL-S1_180 0.0
20   0
40   1
60   1
80   2
100   2
120   0
140   1
160   0
180   0
200   0
220   1
240   1
260   2
280   2
300   0
320   1
340   1
360   0
U34-RIB:U34-P:TYR-S2 U34-RIB:U34-P:TYR-S2_260 0.0
20   0
40   0
60   0
80   0
100   0
120   0
140   0
160   0
180   0
200   0
220   0
240   0
260   0
280   0
300   0

320   0
340   0
360   0
H2U-RIB:H2U-P:ARG-S1 H2U-RIB:H2U-P:ARG-S1_40 0.0
20   0
40   0
60   0
80   0
100   0
120   0
140   0
160   0
180   0
200   0
220   0
240   0
260   0
280   0
300   0
320   0
340   0
360   0
C-P:C-RIB:MET-CA C-P:C-RIB:MET-CA_340 0.0
20   0
40   0
60   0
80   1
100   2
120   2
140   1
160   0
180   0
200   0
220   0
240   1
260   1
280   2
300   2
320   1
340   0
360   0
C-P:C-RIB:PRO-S1 C-P:C-RIB:PRO-S1_160 -2296.77169378
20   0
40   1
60   4
80   5
100   6
120   6
140   6
160   4
180   2
200   0
220   1
240   4
260   6
280   7
300   7
320   6

340   4
360   2
C-RIB:C-P:ALA-S1 C-RIB:C-P:ALA-S1_360 -4211.21007465
20   0
40   2
60   5
80   7
100   9
120   9
140   8
160   6
180   2
200   0
220   2
240   5
260   7
280   9
300   10
320   8
340   6
360   2
C-RIB:C-Y:LEU-S1 C-RIB:C-Y:LEU-S1_60 -4100.36738431
20   0
40   1
60   1
80   1
100   2
120   2
140   1
160   1
180   0
200   0
220   1
240   1
260   1
280   2
300   2
320   1
340   1
360   0
C-P:C-RIB:MET-S1 C-P:C-RIB:MET-S1_220 0.0
20   0
40   0
60   0
80   1
100   1
120   2
140   0
160   1
180   0
200   0
220   0
240   1
260   1
280   2
300   2
320   1
340   0

360   0
H2U-RIB:H2U-P:THR-S1 H2U-RIB:H2U-P:THR-S1_220 0.0
20   0
40   0
60   0
80   0
100   0
120   0
140   0
160   0
180   0
200   0
220   0
240   0
260   0
280   0
300   0
320   0
340   0
360   0
C-P:C-RIB:GLY-CA C-P:C-RIB:GLY-CA_360 -2754.46703531
20   0
40   3
60   8
80   10
100   12
120   12
140   11
160   9
180   4
200   0
220   3
240   8
260   11
280   12
300   12
320   11
340   8
360   3
G-P:G-RIB:PHE-CA G-P:G-RIB:PHE-CA_300 -161.229709883
20   0
40   0
60   1
80   3
100   3
120   3
140   3
160   1
180   0
200   0
220   0
240   1
260   2
280   3
300   3
320   2
340   2
360   0

U31-RIB:U31-MY:ASP-S1 U31-RIB:U31-MY:ASP-S1_300 0.0
20 0
40 0
60 0
80 0
100 0
120 0
140 0
160 0
180 0
200 0
220 0
240 0
260 0
280 0
300 0
320 0
340 0
360 0
G-P:G-RIB:HIS-CA G-P:G-RIB:HIS-CA_40 -4419.72227863
20 0
40 0
60 2
80 4
100 4
120 4
140 3
160 0
180 1
200 0
220 0
240 2
260 4
280 4
300 4
320 3
340 2
360 1
A-RIB:A-R5:LYS-S2 A-RIB:A-R5:LYS-S2_80 -2988.95572696
20 2
40 7
60 11
80 14
100 11
120 9
140 8
160 5
180 0
200 2
220 7
240 11
260 14
280 11
300 10
320 8
340 6
360 0
A-RIB:A-R5:MET-S1 A-RIB:A-R5:MET-S1_320 -3507.75522019

```
20   0
40   0
60   1
80   1
100   1
120   1
140   0
160   0
180   0
200   0
220   0
240   1
260   1
280   0
300   1
320   0
340   0
360   0
QUO-P:QUO-RIB:ASN-CA QUO-P:QUO-RIB:ASN-CA_300 0.0
20   0
40   0
60   0
80   0
100   0
120   0
140   0
160   0
180   0
200   0
220   0
240   0
260   0
280   0
300   0
320   0
340   0
360   0
A-RIB:A-R6:GLN-S1 A-RIB:A-R6:GLN-S1_40 -4063.19384856
20   0
40   0
60   2
80   3
100   4
120   4
140   3
160   2
180   0
200   0
220   0
240   2
260   4
280   4
300   4
320   3
340   2
360   0
U-P:U-RIB:GLN-S1 U-P:U-RIB:GLN-S1_120 -2133.73535767
20   0
```

40   0
60   2
80   3
100   3
120   3
140   2
160   2
180   1
200   0
220   1
240   2
260   3
280   3
300   3
320   3
340   2
360   1
C31-RIB:C31-P:GLN-CA C31-RIB:C31-P:GLN-CA_300 0.0
20   0
40   0
60   0
80   0
100   0
120   0
140   0
160   0
180   0
200   0
220   0
240   0
260   0
280   0
300   0
320   0
340   0
360   0
C-RIB:C-P:ASN-S2 C-RIB:C-P:ASN-S2_280 -4795.33228737
20   0
40   1
60   4
80   6
100   8
120   7
140   6
160   5
180   2
200   0
220   1
240   4
260   6
280   8
300   7
320   6
340   5
360   2
GTP-RIB:GTP-M6:SER-S1 GTP-RIB:GTP-M6:SER-S1_220 0.0
20   0
40   0

60 0
80 0
100 0
120 0
140 0
160 0
180 0
200 0
220 0
240 0
260 0
280 0
300 0
320 0
340 0
360 0
G-RIB:G-R5:HIS-CA G-RIB:G-R5:HIS-CA_200 -6744.07519606
20 0
40 1
60 2
80 2
100 2
120 1
140 1
160 0
180 0
200 0
220 1
240 2
260 2
280 2
300 2
320 1
340 1
360 0
FHU-RIB:FHU-P:ASP-S1 FHU-RIB:FHU-P:ASP-S1_260 0.0
20 0
40 0
60 0
80 0
100 0
120 0
140 0
160 0
180 0
200 0
220 0
240 0
260 0
280 0
300 0
320 0
340 0
360 0
A-P:A-RIB:PHE-S1 A-P:A-RIB:PHE-S1_60 -5055.08096788
20 0
40 0
60 1

80   1
100   2
120   2
140   2
160   1
180   0
200   0
220   0
240   1
260   1
280   2
300   2
320   2
340   1
360   0
U31-P:U31-RIB:GLU-S2 U31-P:U31-RIB:GLU-S2_40 0.0
20   0
40   0
60   0
80   0
100   0
120   0
140   0
160   0
180   0
200   0
220   0
240   0
260   0
280   0
300   0
320   0
340   0
360   0
U-P:U-RIB:ASN-CA U-P:U-RIB:ASN-CA_100 -2443.46288544
20   0
40   0
60   1
80   3
100   3
120   3
140   2
160   2
180   1
200   0
220   0
240   2
260   3
280   3
300   3
320   3
340   2
360   1
C-RIB:C-Y:TRP-CA C-RIB:C-Y:TRP-CA_280 -4479.42908716
20   0
40   0
60   0
80   0

100 0
120 0
140 0
160 0
180 0
200 0
220 0
240 0
260 0
280 0
300 0
320 0
340 0
360 0
U-RIB:U-Y:HIS-CA U-RIB:U-Y:HIS-CA_180 0.0
20 0
40 0
60 0
80 0
100 1
120 1
140 1
160 0
180 0
200 0
220 0
240 0
260 0
280 1
300 1
320 0
340 0
360 0
M2G-RIB:M2G-P:GLY-CA M2G-RIB:M2G-P:GLY-CA_300 0.0
20 0
40 0
60 0
80 0
100 0
120 0
140 0
160 0
180 0
200 0
220 0
240 0
260 0
280 0
300 0
320 0
340 0
360 0
A-P:A-RIB:SER-CA A-P:A-RIB:SER-CA_100 -3915.82324141
20 0
40 1
60 4
80 6
100 7

120   7
140   6
160   5
180   2
200   0
220   1
240   4
260   6
280   7
300   7
320   6
340   5
360   2
DA-RIB:DA-M5:SER-CA DA-RIB:DA-M5:SER-CA_340 -8038.17962263
20   0
40   0
60   0
80   0
100   0
120   0
140   0
160   0
180   0
200   0
220   0
240   0
260   0
280   0
300   0
320   0
340   0
360   0
U31-P:U31-RIB:TYR-S1 U31-P:U31-RIB:TYR-S1_120 0.0
20   0
40   0
60   0
80   0
100   0
120   0
140   0
160   0
180   0
200   0
220   0
240   0
260   0
280   0
300   0
320   0
340   0
360   0
C-RIB:C-Y:PHE-S1 C-RIB:C-Y:PHE-S1_320 -3369.6323556
20   0
40   0
60   0
80   0
100   0
120   0

140 0
160 0
180 0
200 0
220 0
240 0
260 0
280 1
300 0
320 0
340 0
360 0
H2U-RIB:H2U-MY:LEU-S1 H2U-RIB:H2U-MY:LEU-S1_120 0.0
20 0
40 0
60 0
80 0
100 0
120 0
140 0
160 0
180 0
200 0
220 0
240 0
260 0
280 0
300 0
320 0
340 0
360 0
U31-RIB:U31-MY:TYR-S2 U31-RIB:U31-MY:TYR-S2_160 0.0
20 0
40 0
60 0
80 0
100 0
120 0
140 0
160 0
180 0
200 0
220 0
240 0
260 0
280 0
300 0
320 0
340 0
360 0
FHU-RIB:FHU-P:SER-S1 FHU-RIB:FHU-P:SER-S1_200 0.0
20 0
40 0
60 0
80 0
100 0
120 0
140 0

160 0
180 0
200 0
220 0
240 0
260 0
280 0
300 0
320 0
340 0
360 0
C31-P:C31-RIB:THR-CA C31-P:C31-RIB:THR-CA_40 0.0
20 0
40 0
60 0
80 0
100 0
120 0
140 0
160 0
180 0
200 0
220 0
240 0
260 0
280 0
300 0
320 0
340 0
360 0
C-RIB:C-P:GLY-CA C-RIB:C-P:GLY-CA_60 -2781.51066392
20 0
40 2
60 6
80 11
100 14
120 14
140 12
160 8
180 3
200 0
220 2
240 6
260 10
280 14
300 14
320 12
340 8
360 3
QUO-RIB:QUO-M5:LEU-S2 QUO-RIB:QUO-M5:LEU-S2_180 0.0
20 0
40 0
60 0
80 0
100 0
120 0
140 0
160 0

180   0
200   0
220   0
240   0
260   0
280   0
300   0
320   0
340   0
360   0
G-RIB:G-R6:LYS-S1 G-RIB:G-R6:LYS-S1_60 -3512.29575547
20   0
40   2
60   8
80   12
100   12
120   10
140   8
160   5
180   0
200   0
220   2
240   8
260   11
280   12
300   10
320   7
340   5
360   1
G-RIB:G-R6:GLU-S1 G-RIB:G-R6:GLU-S1_140 305.01187182
20   0
40   3
60   10
80   15
100   14
120   12
140   9
160   6
180   2
200   0
220   3
240   10
260   14
280   14
300   12
320   9
340   6
360   2
FHU-P:FHU-RIB:ILE-CA FHU-P:FHU-RIB:ILE-CA_280 0.0
20   0
40   0
60   0
80   0
100   0
120   0
140   0
160   0
180   0

200   0
220   0
240   0
260   0
280   0
300   0
320   0
340   0
360   0
G-RIB:G-R5:THR-CA G-RIB:G-R5:THR-CA_340 0.0
20   0
40   1
60   3
80   4
100   4
120   4
140   3
160   2
180   0
200   0
220   2
240   4
260   5
280   5
300   4
320   3
340   0
360   0
U-RIB:U-Y:TRP-S1 U-RIB:U-Y:TRP-S1_340 -6176.78606863
20   0
40   0
60   0
80   0
100   0
120   0
140   0
160   0
180   0
200   0
220   0
240   0
260   0
280   0
300   0
320   0
340   0
360   0
U-RIB:U-Y:THR-CA U-RIB:U-Y:THR-CA_200 -6166.8153805
20   0
40   0
60   1
80   2
100   3
120   2
140   2
160   0
180   0
200   0

220   0
240   1
260   2
280   2
300   2
320   2
340   1
360   0
5BU-RIB:5BU-P:SER-CA 5BU-RIB:5BU-P:SER-CA_240 0.0
20   0
40   0
60   0
80   0
100   0
120   0
140   0
160   0
180   0
200   0
220   0
240   0
260   0
280   0
300   0
320   0
340   0
360   0
G-RIB:G-P:PHE-CA G-RIB:G-P:PHE-CA_60 -4368.63360977
20   0
40   0
60   1
80   2
100   4
120   5
140   4
160   3
180   0
200   0
220   0
240   1
260   2
280   4
300   4
320   4
340   3
360   0
U31-RIB:U31-MY:GLU-S1 U31-RIB:U31-MY:GLU-S1_120 0.0
20   0
40   0
60   0
80   0
100   0
120   0
140   0
160   0
180   0
200   0
220   0

240 0
260 0
280 0
300 0
320 0
340 0
360 0
A-RIB:A-P:SER-CA A-RIB:A-P:SER-CA_40 -2909.86043635
20 0
40 1
60 3
80 7
100 8
120 9
140 7
160 5
180 2
200 0
220 1
240 3
260 6
280 8
300 8
320 6
340 5
360 2
U31-P:U31-RIB:MET-S1 U31-P:U31-RIB:MET-S1_60 0.0
20 0
40 0
60 0
80 0
100 0
120 0
140 0
160 0
180 0
200 0
220 0
240 0
260 0
280 0
300 0
320 0
340 0
360 0
DA-RIB:DA-M6:SER-S1 DA-RIB:DA-M6:SER-S1_60 0.0
20 0
40 0
60 0
80 0
100 0
120 0
140 0
160 0
180 0
200 0
220 0
240 0

260 0
280 0
300 0
320 0
340 0
360 0
G-RIB:G-P:GLN-CA G-RIB:G-P:GLN-CA_280 -4289.14872454
20 0
40 0
60 2
80 5
100 7
120 9
140 6
160 4
180 1
200 0
220 0
240 2
260 5
280 7
300 8
320 7
340 5
360 1
FHU-RIB:FHU-P:LYS-S2 FHU-RIB:FHU-P:LYS-S2_180 0.0
20 0
40 0
60 0
80 0
100 0
120 0
140 0
160 0
180 0
200 0
220 0
240 0
260 0
280 0
300 0
320 0
340 0
360 0
FHU-RIB:FHU-MY:TYR-S2 FHU-RIB:FHU-MY:TYR-S2_260 0.0
20 0
40 0
60 0
80 0
100 0
120 0
140 0
160 0
180 0
200 0
220 0
240 0
260 0

280   0
300   0
320   0
340   0
360   0
A-RIB:A-P:PHE-S1 A-RIB:A-P:PHE-S1_20 0.0
20   0
40   0
60   0
80   1
100   2
120   3
140   2
160   1
180   0
200   0
220   0
240   0
260   1
280   2
300   2
320   2
340   1
360   0
G-RIB:G-R5:VAL-S1 G-RIB:G-R5:VAL-S1_360 0.0
20   0
40   3
60   4
80   4
100   4
120   3
140   3
160   0
180   0
200   1
220   3
240   5
260   4
280   4
300   4
320   3
340   0
360   0
FHU-RIB:FHU-P:THR-CA FHU-RIB:FHU-P:THR-CA_20 0.0
20   0
40   0
60   0
80   0
100   0
120   0
140   0
160   0
180   0
200   0
220   0
240   0
260   0
280   0

300   0
320   0
340   0
360   0
U-RIB:U-Y:ARG-CA U-RIB:U-Y:ARG-CA_300 -3749.32200311
20   0
40   1
60   1
80   2
100   3
120   3
140   2
160   1
180   0
200   0
220   1
240   1
260   2
280   2
300   3
320   2
340   1
360   0
A-RIB:A-R6:HIS-S1 A-RIB:A-R6:HIS-S1_280 -5899.26735495
20   0
40   0
60   1
80   2
100   2
120   2
140   2
160   1
180   0
200   0
220   0
240   1
260   2
280   2
300   2
320   2
340   1
360   0
A-RIB:A-P:HIS-S1 A-RIB:A-P:HIS-S1_260 -2694.93954378
20   0
40   0
60   1
80   2
100   4
120   4
140   3
160   2
180   0
200   0
220   0
240   1
260   2
280   3
300   4

320   3
340   2
360   0
C-P:C-RIB:CYS-CA C-P:C-RIB:CYS-CA_240 0.0
20   0
40   0
60   0
80   0
100   0
120   0
140   0
160   0
180   0
200   0
220   0
240   0
260   0
280   0
300   0
320   0
340   0
360   0
A-RIB:A-P:GLN-S2
20   0
40   1
60   4
80   6
100   7
120   7
140   6
160   4
180   1
200   0
220   1
240   3
260   5
280   7
300   7
320   6
340   5
360   1
G-RIB:G-P:GLU-CA G-RIB:G-P:GLU-CA_220 -4678.59139273
20   0
40   1
60   6
80   15
100   22
120   23
140   20
160   13
180   5
200   0
220   1
240   0
260   14
280   21
300   23
320   20

340 14
360 0
FHU-RIB:FHU-P:SER-CA FHU-RIB:FHU-P:SER-CA_140 0.0
20 0
40 0
60 0
80 0
100 0
120 0
140 0
160 0
180 0
200 0
220 0
240 0
260 0
280 0
300 0
320 0
340 0
360 0
A-RIB:A-P:PHE-CA A-RIB:A-P:PHE-CA_160 -5429.88509892
20 0
40 0
60 1
80 1
100 3
120 3
140 2
160 1
180 0
200 0
220 0
240 0
260 2
280 2
300 3
320 2
340 1
360 0
DA-RIB:DA-M6:GLN-CA DA-RIB:DA-M6:GLN-CA_80 0.0
20 0
40 0
60 0
80 0
100 0
120 0
140 0
160 0
180 0
200 0
220 0
240 0
260 0
280 0
300 0
320 0
340 0

```
360   0
U34-RIB:U34-P:SER-CA U34-RIB:U34-P:SER-CA_340 0.0
20   0
40   0
60   0
80   0
100   0
120   0
140   0
160   0
180   0
200   0
220   0
240   0
260   0
280   0
300   0
320   0
340   0
360   0
H2U-RIB:H2U-MY:GLN-S2 H2U-RIB:H2U-MY:GLN-S2_240 0.0
20   0
40   0
60   0
80   0
100   0
120   0
140   0
160   0
180   0
200   0
220   0
240   0
260   0
280   0
300   0
320   0
340   0
360   0
G-RIB:G-R6:LEU-S1 G-RIB:G-R6:LEU-S1_220 -5894.96452437
20   0
40   0
60   1
80   3
100   0
120   3
140   3
160   1
180   0
200   0
220   0
240   2
260   2
280   3
300   3
320   2
340   0
360   0
```

H2U-RIB:H2U-P:TRP-S2 H2U-RIB:H2U-P:TRP-S2_20 0.0
20 0
40 0
60 0
80 0
100 0
120 0
140 0
160 0
180 0
200 0
220 0
240 0
260 0
280 0
300 0
320 0
340 0
360 0
G-P:G-RIB:ILE-S1 G-P:G-RIB:ILE-S1_360 -3154.04316562
20 0
40 1
60 0
80 4
100 5
120 5
140 4
160 0
180 0
200 0
220 1
240 0
260 4
280 4
300 5
320 4
340 2
360 1
DA-RIB:DA-M6:HIS-CA DA-RIB:DA-M6:HIS-CA_120 0.0
20 0
40 0
60 0
80 0
100 0
120 0
140 0
160 0
180 0
200 0
220 0
240 0
260 0
280 0
300 0
320 0
340 0
360 0
A-P:A-RIB:MET-S2 A-P:A-RIB:MET-S2_180 0.0

20 0
40 0
60 1
80 2
100 2
120 2
140 2
160 1
180 0
200 0
220 0
240 1
260 2
280 2
300 2
320 2
340 1
360 0
G-RIB:G-P:PRO-CA G-RIB:G-P:PRO-CA_80 -2970.15403295
20 0
40 1
60 4
80 8
100 12
120 12
140 10
160 7
180 2
200 0
220 1
240 3
260 7
280 10
300 11
320 9
340 6
360 2
C-RIB:C-Y:TYR-S2 C-RIB:C-Y:TYR-S2_280 -5404.15194685
20 0
40 0
60 1
80 1
100 1
120 1
140 1
160 0
180 0
200 0
220 0
240 1
260 1
280 1
300 1
320 1
340 0
360 0
C31-RIB:C31-P:ALA-CA C31-RIB:C31-P:ALA-CA_260 0.0
20 0

40   0
60   0
80   0
100   0
120   0
140   0
160   0
180   0
200   0
220   0
240   0
260   0
280   0
300   0
320   0
340   0
360   0
C-RIB:C-Y:TYR-CA C-RIB:C-Y:TYR-CA_60 -3602.24653071
20   0
40   0
60   0
80   0
100   0
120   0
140   0
160   0
180   0
200   0
220   0
240   0
260   0
280   0
300   1
320   0
340   0
360   0
DA-RIB:DA-M6:THR-S1 DA-RIB:DA-M6:THR-S1_300 0.0
20   0
40   0
60   0
80   0
100   0
120   0
140   0
160   0
180   0
200   0
220   0
240   0
260   0
280   0
300   0
320   0
340   0
360   0
U-RIB:U-P:HIS-S2 U-RIB:U-P:HIS-S2_160 -3797.03733904
20   0
40   0

60   1
80   2
100   3
120   3
140   2
160   1
180   0
200   0
220   0
240   1
260   2
280   3
300   3
320   2
340   2
360   0
U31-RIB:U31-MY:PHE-S1 U31-RIB:U31-MY:PHE-S1_220 0.0
20   0
40   0
60   0
80   0
100   0
120   0
140   0
160   0
180   0
200   0
220   0
240   0
260   0
280   0
300   0
320   0
340   0
360   0
U34-RIB:U34-MY:ASP-CA U34-RIB:U34-MY:ASP-CA_80 0.0
20   0
40   0
60   0
80   0
100   0
120   0
140   0
160   0
180   0
200   0
220   0
240   0
260   0
280   0
300   0
320   0
340   0
360   0
G-RIB:G-P:THR-CA G-RIB:G-P:THR-CA_20 0.0
20   0
40   0
60   3

80   7
100   11
120   11
140   9
160   6
180   2
200   0
220   0
240   3
260   7
280   10
300   11
320   10
340   7
360   2
U-RIB:U-Y:ASP-CA U-RIB:U-Y:ASP-CA_360 -3075.65555985
20   0
40   1
60   2
80   3
100   0
120   3
140   3
160   1
180   0
200   0
220   1
240   2
260   3
280   0
300   3
320   3
340   2
360   0
C-RIB:C-Y:GLN-CA C-RIB:C-Y:GLN-CA_180 0.0
20   0
40   0
60   1
80   1
100   1
120   1
140   1
160   0
180   0
200   0
220   0
240   1
260   1
280   2
300   1
320   0
340   1
360   0
G-P:G-RIB:TRP-CA G-P:G-RIB:TRP-CA_280 -6184.79160921
20   0
40   0
60   0
80   1

```
100   1
120   0
140   1
160   1
180   0
200   0
220   0
240   0
260   1
280   1
300   1
320   1
340   0
360   0
C-P:C-RIB:HIS-CA C-P:C-RIB:HIS-CA_360 -5789.14253007
20   0
40   0
60   1
80   2
100   2
120   2
140   0
160   1
180   0
200   0
220   0
240   1
260   2
280   2
300   2
320   2
340   2
360   0
U31-P:U31-RIB:MET-S2 U31-P:U31-RIB:MET-S2_340 0.0
20   0
40   0
60   0
80   0
100   0
120   0
140   0
160   0
180   0
200   0
220   0
240   0
260   0
280   0
300   0
320   0
340   0
360   0
U-RIB:U-P:ALA-CA U-RIB:U-P:ALA-CA_160 -4933.18473572
20   0
40   0
60   2
80   4
100   5
```

120   6
140   5
160   3
180   1
200   0
220   0
240   2
260   4
280   6
300   6
320   5
340   3
360   1
FHU-P:FHU-RIB:TYR-S1 FHU-P:FHU-RIB:TYR-S1_20 0.0
20   0
40   0
60   0
80   0
100   0
120   0
140   0
160   0
180   0
200   0
220   0
240   0
260   0
280   0
300   0
320   0
340   0
360   0
U-RIB:U-Y:CYS-CA U-RIB:U-Y:CYS-CA_320 -5849.68302389
20   0
40   0
60   0
80   0
100   0
120   0
140   0
160   0
180   0
200   0
220   0
240   0
260   0
280   0
300   0
320   0
340   0
360   0
C-P:C-RIB:ASP-S1 C-P:C-RIB:ASP-S1_20 0.0
20   0
40   0
60   6
80   10
100   11
120   11

140   9
160   7
180   3
200   0
220   2
240   7
260   10
280   11
300   11
320   9
340   8
360   4
DA-RIB:DA-M5:LEU-S1 DA-RIB:DA-M5:LEU-S1_340 0.0
20   0
40   0
60   0
80   0
100   0
120   0
140   0
160   0
180   0
200   0
220   0
240   0
260   0
280   0
300   0
320   0
340   0
360   0
FHU-RIB:FHU-P:ARG-S2 FHU-RIB:FHU-P:ARG-S2_80 -7629.15943592
20   0
40   0
60   0
80   0
100   0
120   0
140   0
160   0
180   0
200   0
220   0
240   0
260   0
280   0
300   0
320   0
340   0
360   0
5BU-RIB:5BU-P:ILE-CA 5BU-RIB:5BU-P:ILE-CA_60 0.0
20   0
40   0
60   0
80   0
100   0
120   0
140   0

160   0
180   0
200   0
220   0
240   0
260   0
280   0
300   0
320   0
340   0
360   0
DA-RIB:DA-M5:ASN-S1 DA-RIB:DA-M5:ASN-S1_300 0.0
20   0
40   0
60   0
80   0
100   0
120   0
140   0
160   0
180   0
200   0
220   0
240   0
260   0
280   0
300   0
320   0
340   0
360   0
5BU-RIB:5BU-P:THR-CA 5BU-RIB:5BU-P:THR-CA_120 0.0
20   0
40   0
60   0
80   0
100   0
120   0
140   0
160   0
180   0
200   0
220   0
240   0
260   0
280   0
300   0
320   0
340   0
360   0
G-RIB:G-P:GLY-CA G-RIB:G-P:GLY-CA_180 -4103.66221545
20   0
40   3
60   10
80   18
100   23
120   23
140   20
160   14

180   5
200   0
220   3
240   9
260   17
280   23
300   24
320   20
340   13
360   5
G-RIB:G-R5:ILE-S1 G-RIB:G-R5:ILE-S1_140 -1721.13890095
20   0
40   1
60   2
80   2
100   2
120   2
140   1
160   0
180   0
200   0
220   1
240   2
260   2
280   2
300   2
320   0
340   0
360   0
FMU-RIB:FMU-MY:GLU-S2 FMU-RIB:FMU-MY:GLU-S2_300 0.0
20   0
40   0
60   0
80   0
100   0
120   0
140   0
160   0
180   0
200   0
220   0
240   0
260   0
280   0
300   0
320   0
340   0
360   0
C-RIB:C-Y:GLN-S2 C-RIB:C-Y:GLN-S2_20 -5777.77685576
20   0
40   2
60   4
80   4
100   3
120   2
140   2
160   0
180   0

200 0
220 2
240 4
260 4
280 3
300 3
320 0
340 1
360 0
H2U-RIB:H2U-MY:GLY-CA H2U-RIB:H2U-MY:GLY-CA_20 0.0
20 0
40 0
60 0
80 0
100 0
120 0
140 0
160 0
180 0
200 0
220 0
240 0
260 0
280 0
300 0
320 0
340 0
360 0
A-RIB:A-P:LEU-S1 A-RIB:A-P:LEU-S1_120 -751.079386846
20 0
40 0
60 1
80 4
100 5
120 5
140 5
160 3
180 1
200 0
220 0
240 2
260 4
280 5
300 5
320 5
340 3
360 0
U-RIB:U-Y:GLN-S1 U-RIB:U-Y:GLN-S1_60 -5927.17740615
20 0
40 1
60 1
80 2
100 2
120 2
140 1
160 1
180 0
200 0

220 0
240 2
260 2
280 2
300 2
320 1
340 0
360 0
G-RIB:G-R5:LYS-S2 G-RIB:G-R5:LYS-S2_160 -3425.65319972
20 3
40 11
60 17
80 19
100 15
120 11
140 10
160 7
180 2
200 3
220 11
240 17
260 19
280 15
300 11
320 9
340 7
360 2
U-RIB:U-Y:SER-S1 U-RIB:U-Y:SER-S1_240 -2360.0058948
20 0
40 2
60 3
80 4
100 4
120 3
140 3
160 2
180 0
200 0
220 2
240 3
260 4
280 3
300 3
320 3
340 2
360 0
U-RIB:U-P:ILE-S1 U-RIB:U-P:ILE-S1_160 -4097.48799682
20 0
40 0
60 1
80 2
100 2
120 3
140 2
160 1
180 0
200 0
220 0

240   0
260   2
280   2
300   3
320   0
340   1
360   0
GTP-RIB:GTP-M6:ASN-CA GTP-RIB:GTP-M6:ASN-CA_80 0.0
20   0
40   0
60   0
80   0
100   0
120   0
140   0
160   0
180   0
200   0
220   0
240   0
260   0
280   0
300   0
320   0
340   0
360   0
FMU-P:FMU-RIB:CYS-S1 FMU-P:FMU-RIB:CYS-S1_220 0.0
20   0
40   0
60   0
80   0
100   0
120   0
140   0
160   0
180   0
200   0
220   0
240   0
260   0
280   0
300   0
320   0
340   0
360   0
U-P:U-RIB:TRP-S1 U-P:U-RIB:TRP-S1_180 0.0
20   0
40   0
60   0
80   0
100   0
120   0
140   0
160   0
180   0
200   0
220   0
240   0

260   0
280   0
300   0
320   0
340   0
360   0
FMU-RIB:FMU-MY:VAL-S1 FMU-RIB:FMU-MY:VAL-S1_260 0.0
20   0
40   0
60   0
80   0
100   0
120   0
140   0
160   0
180   0
200   0
220   0
240   0
260   0
280   0
300   0
320   0
340   0
360   0
4SU-RIB:4SU-P:THR-S1 4SU-RIB:4SU-P:THR-S1_360 0.0
20   0
40   0
60   0
80   0
100   0
120   0
140   0
160   0
180   0
200   0
220   0
240   0
260   0
280   0
300   0
320   0
340   0
360   0
A-RIB:A-P:ASN-CA A-RIB:A-P:ASN-CA_240 -3290.46547238
20   0
40   0
60   2
80   5
100   6
120   7
140   5
160   3
180   1
200   0
220   0
240   2
260   5

280   6
300   6
320   5
340   3
360   1
G-RIB:G-P:ARG-S2 G-RIB:G-P:ARG-S2_80 -4894.71494268
20   0
40   4
60   12
80   21
100   28
120   26
140   23
160   17
180   7
200   0
220   4
240   12
260   21
280   28
300   27
320   23
340   17
360   7
DA-RIB:DA-M6:GLN-S1 DA-RIB:DA-M6:GLN-S1_280 0.0
20   0
40   0
60   0
80   0
100   0
120   0
140   0
160   0
180   0
200   0
220   0
240   0
260   0
280   0
300   0
320   0
340   0
360   0
G-RIB:G-R5:TYR-S1 G-RIB:G-R5:TYR-S1_340 0.0
20   0
40   0
60   1
80   1
100   1
120   1
140   0
160   0
180   0
200   0
220   0
240   1
260   1
280   1

300 1
320 1
340 0
360 0
G-RIB:G-R6:ILE-CA G-RIB:G-R6:ILE-CA_220 0.0
20 0
40 0
60 1
80 0
100 3
120 3
140 0
160 1
180 0
200 0
220 0
240 1
260 3
280 3
300 3
320 2
340 0
360 0
H2U-RIB:H2U-MY:GLU-CA H2U-RIB:H2U-MY:GLU-CA_80 0.0
20 0
40 0
60 0
80 0
100 0
120 0
140 0
160 0
180 0
200 0
220 0
240 0
260 0
280 0
300 0
320 0
340 0
360 0
G-RIB:G-P:TRP-S2 G-RIB:G-P:TRP-S2_200 0.0
20 0
40 0
60 0
80 1
100 2
120 2
140 2
160 1
180 0
200 0
220 0
240 0
260 1
280 2
300 2

320   2
340   1
360   0
U31-RIB:U31-P:ASN-S2 U31-RIB:U31-P:ASN-S2_260 0.0
20   0
40   0
60   0
80   0
100   0
120   0
140   0
160   0
180   0
200   0
220   0
240   0
260   0
280   0
300   0
320   0
340   0
360   0
G-RIB:G-R6:VAL-CA G-RIB:G-R6:VAL-CA_260 -3029.9968244
20   0
40   0
60   3
80   4
100   5
120   5
140   3
160   0
180   1
200   0
220   0
240   3
260   4
280   6
300   5
320   3
340   2
360   0
U-RIB:U-P:ASP-CA U-RIB:U-P:ASP-CA_220 0.0
20   0
40   0
60   2
80   5
100   7
120   7
140   6
160   4
180   1
200   0
220   0
240   2
260   5
280   7
300   7
320   6

340   4
360   1
A-RIB:A-R5:TYR-CA A-RIB:A-R5:TYR-CA_20 0.0
20   0
40   0
60   0
80   1
100   1
120   1
140   0
160   0
180   0
200   0
220   0
240   0
260   1
280   1
300   1
320   0
340   0
360   0
C-P:C-RIB:ILE-CA C-P:C-RIB:ILE-CA_240 0.0
20   0
40   0
60   1
80   3
100   4
120   4
140   3
160   2
180   1
200   0
220   0
240   0
260   3
280   4
300   4
320   3
340   2
360   0
U-P:U-RIB:SER-S1 U-P:U-RIB:SER-S1_200 -6929.8684788
20   0
40   1
60   3
80   5
100   5
120   5
140   5
160   4
180   1
200   0
220   2
240   4
260   5
280   5
300   5
320   5
340   3

360   1
U-P:U-RIB:ARG-CA U-P:U-RIB:ARG-CA_280 -2435.50448677
20   0
40   1
60   3
80   4
100   5
120   5
140   4
160   3
180   1
200   0
220   1
240   3
260   4
280   5
300   5
320   4
340   3
360   1
C31-RIB:C31-P:SER-S1 C31-RIB:C31-P:SER-S1_320 -12675.9675659
20   0
40   0
60   0
80   0
100   0
120   0
140   0
160   0
180   0
200   0
220   0
240   0
260   0
280   0
300   0
320   0
340   0
360   0
U31-RIB:U31-P:LEU-S1 U31-RIB:U31-P:LEU-S1_20 0.0
20   0
40   0
60   0
80   0
100   0
120   0
140   0
160   0
180   0
200   0
220   0
240   0
260   0
280   0
300   0
320   0
340   0
360   0

A-RIB:A-R5:ALA-S1 A-RIB:A-R5:ALA-S1_260 -2853.18150164
20 1
40 3
60 5
80 6
100 6
120 5
140 4
160 3
180 1
200 1
220 4
240 5
260 6
280 6
300 5
320 4
340 3
360 0
A-RIB:A-P:PRO-S1 A-RIB:A-P:PRO-S1_360 -1886.59488962
20 0
40 1
60 4
80 6
100 8
120 7
140 7
160 5
180 0
200 0
220 1
240 4
260 6
280 7
300 7
320 6
340 4
360 1
C-P:C-RIB:LYS-S2 C-P:C-RIB:LYS-S2_260 -3084.72182716
20 1
40 6
60 12
80 15
100 15
120 14
140 13
160 11
180 5
200 1
220 5
240 12
260 15
280 15
300 14
320 12
340 11
360 5
G-P:G-RIB:PRO-CA G-P:G-RIB:PRO-CA_100 -4351.83549352

20 0
40 1
60 5
80 7
100 8
120 8
140 7
160 5
180 2
200 0
220 2
240 5
260 8
280 9
300 8
320 7
340 5
360 0
C-P:C-RIB:VAL-S1 C-P:C-RIB:VAL-S1_320 -3953.54879587
20 0
40 1
60 3
80 5
100 5
120 5
140 5
160 4
180 0
200 0
220 0
240 3
260 5
280 6
300 6
320 5
340 4
360 1
C-RIB:C-Y:LYS-CA C-RIB:C-Y:LYS-CA_320 -1209.2026461
20 0
40 2
60 3
80 4
100 3
120 4
140 3
160 2
180 1
200 0
220 2
240 4
260 4
280 4
300 4
320 3
340 2
360 1
G-RIB:G-R5:GLU-S1 G-RIB:G-R5:GLU-S1_260 1160.07779585
20 0

40   7
60   13
80   13
100   10
120   0
140   8
160   5
180   2
200   2
220   7
240   12
260   12
280   11
300   9
320   8
340   5
360   2
C31-P:C31-RIB:ALA-S1 C31-P:C31-RIB:ALA-S1_20 0.0
20   0
40   0
60   0
80   0
100   0
120   0
140   0
160   0
180   0
200   0
220   0
240   0
260   0
280   0
300   0
320   0
340   0
360   0
A-RIB:A-R6:LEU-S2 A-RIB:A-R6:LEU-S2_160 -1664.71330295
20   0
40   0
60   2
80   3
100   3
120   3
140   3
160   2
180   0
200   0
220   0
240   2
260   3
280   3
300   3
320   3
340   2
360   0
U34-RIB:U34-MY:TYR-S2 U34-RIB:U34-MY:TYR-S2_360 0.0
20   0
40   0

60   0
80   0
100   0
120   0
140   0
160   0
180   0
200   0
220   0
240   0
260   0
280   0
300   0
320   0
340   0
360   0
QUO-RIB:QUO-M5:ARG-CA QUO-RIB:QUO-M5:ARG-CA_360 0.0
20   0
40   0
60   0
80   0
100   0
120   0
140   0
160   0
180   0
200   0
220   0
240   0
260   0
280   0
300   0
320   0
340   0
360   0
FMU-RIB:FMU-MY:ASP-S1 FMU-RIB:FMU-MY:ASP-S1_160 -12675.9675659
20   0
40   0
60   0
80   0
100   0
120   0
140   0
160   0
180   0
200   0
220   0
240   0
260   0
280   0
300   0
320   0
340   0
360   0
QUO-RIB:QUO-P:LEU-S1 QUO-RIB:QUO-P:LEU-S1_40 0.0
20   0
40   0
60   0

80   0
100   0
120   0
140   0
160   0
180   0
200   0
220   0
240   0
260   0
280   0
300   0
320   0
340   0
360   0
A-RIB:A-R6:LYS-CA A-RIB:A-R6:LYS-CA_80 -2274.56737547
20   0
40   0
60   3
80   6
100   7
120   7
140   6
160   5
180   0
200   0
220   1
240   4
260   6
280   8
300   7
320   6
340   5
360   0
U-P:U-RIB:HIS-CA U-P:U-RIB:HIS-CA_340 -5911.73989417
20   0
40   0
60   1
80   1
100   2
120   2
140   2
160   1
180   0
200   0
220   0
240   0
260   1
280   2
300   2
320   1
340   1
360   0
A-RIB:A-R6:ASP-CA A-RIB:A-R6:ASP-CA_360 -2045.44190848
20   0
40   1
60   4
80   6

100   7
120   7
140   6
160   4
180   0
200   0
220   0
240   4
260   7
280   8
300   7
320   5
340   4
360   1
U31-RIB:U31-MY:ALA-S1 U31-RIB:U31-MY:ALA-S1_220 0.0
20   0
40   0
60   0
80   0
100   0
120   0
140   0
160   0
180   0
200   0
220   0
240   0
260   0
280   0
300   0
320   0
340   0
360   0
U-RIB:U-P:TYR-S2 U-RIB:U-P:TYR-S2_40 0.0
20   0
40   0
60   1
80   2
100   2
120   2
140   2
160   1
180   0
200   0
220   0
240   0
260   1
280   2
300   2
320   2
340   1
360   0
C-RIB:C-P:GLU-CA C-RIB:C-P:GLU-CA_40 -2022.91709284
20   0
40   1
60   5
80   10
100   14

120   15
140   12
160   8
180   3
200   0
220   1
240   4
260   9
280   14
300   15
320   12
340   8
360   3
U-RIB:U-Y:PHE-CA U-RIB:U-Y:PHE-CA_160 0.0
20   0
40   0
60   0
80   0
100   0
120   0
140   0
160   0
180   0
200   0
220   0
240   0
260   0
280   0
300   0
320   0
340   0
360   0
A-RIB:A-R5:LEU-S2 A-RIB:A-R5:LEU-S2_320 -6093.28735419
20   0
40   0
60   2
80   3
100   2
120   2
140   1
160   0
180   0
200   0
220   1
240   2
260   2
280   2
300   2
320   2
340   1
360   0
C-RIB:C-Y:ALA-S1 C-RIB:C-Y:ALA-S1_120 -1888.3640526
20   1
40   3
60   5
80   5
100   4
120   4

140   3
160   2
180   0
200   1
220   3
240   5
260   5
280   5
300   4
320   3
340   2
360   0
G-P:G-RIB:HIS-S1 G-P:G-RIB:HIS-S1_360 -2390.88345546
20   0
40   1
60   2
80   4
100   5
120   4
140   4
160   3
180   1
200   0
220   1
240   2
260   4
280   4
300   4
320   4
340   3
360   1
GTP-RIB:GTP-M5:ASP-S1 GTP-RIB:GTP-M5:ASP-S1_80 0.0
20   0
40   0
60   0
80   0
100   0
120   0
140   0
160   0
180   0
200   0
220   0
240   0
260   0
280   0
300   0
320   0
340   0
360   0
A-RIB:A-R6:LEU-CA A-RIB:A-R6:LEU-CA_240 0.0
20   0
40   0
60   1
80   2
100   3
120   3
140   3

160   0
180   0
200   0
220   0
240   0
260   2
280   3
300   3
320   3
340   2
360   0
G-RIB:G-R6:PRO-S1 G-RIB:G-R6:PRO-S1_240 -2683.93562049
20   0
40   2
60   5
80   7
100   6
120   6
140   4
160   3
180   0
200   0
220   1
240   5
260   6
280   6
300   6
320   4
340   2
360   0
A-RIB:A-R5:GLN-S1 A-RIB:A-R5:GLN-S1_340 0.0
20   0
40   1
60   2
80   3
100   3
120   3
140   2
160   1
180   0
200   0
220   1
240   3
260   3
280   3
300   3
320   2
340   0
360   0
C-RIB:C-P:THR-CA C-RIB:C-P:THR-CA_100 -3497.06114012
20   0
40   0
60   2
80   4
100   7
120   7
140   6
160   4

180   1
200   0
220   0
240   2
260   4
280   7
300   7
320   6
340   4
360   1
U31-RIB:U31-MY:GLU-CA U31-RIB:U31-MY:GLU-CA_20 0.0
20   0
40   0
60   0
80   0
100   0
120   0
140   0
160   0
180   0
200   0
220   0
240   0
260   0
280   0
300   0
320   0
340   0
360   0
U31-RIB:U31-MY:GLN-S1 U31-RIB:U31-MY:GLN-S1_280 0.0
20   0
40   0
60   0
80   0
100   0
120   0
140   0
160   0
180   0
200   0
220   0
240   0
260   0
280   0
300   0
320   0
340   0
360   0
C-RIB:C-Y:ILE-S1 C-RIB:C-Y:ILE-S1_160 0.0
20   0
40   1
60   1
80   1
100   1
120   1
140   1
160   0
180   0

200   0
220   1
240   1
260   1
280   1
300   1
320   1
340   0
360   0
G-RIB:G-R5:SER-S1 G-RIB:G-R5:SER-S1_200 -4881.43785566
20   2
40   6
60   9
80   9
100   7
120   6
140   4
160   3
180   1
200   2
220   6
240   9
260   10
280   7
300   5
320   4
340   3
360   1
FHU-P:FHU-RIB:ASP-S1 FHU-P:FHU-RIB:ASP-S1_140 0.0
20   0
40   0
60   0
80   0
100   0
120   0
140   0
160   0
180   0
200   0
220   0
240   0
260   0
280   0
300   0
320   0
340   0
360   0
U-P:U-RIB:ALA-S1 U-P:U-RIB:ALA-S1_340 -2274.43810913
20   0
40   1
60   3
80   5
100   5
120   5
140   4
160   3
180   0
200   0

```
220   1
240   3
260   5
280   5
300   5
320   4
340   3
360   1
C-RIB:C-P:VAL-CA C-RIB:C-P:VAL-CA_180 -3309.23611258
20   0
40   0
60   2
80   4
100   7
120   7
140   6
160   4
180   1
200   0
220   0
240   2
260   4
280   7
300   7
320   6
340   4
360   1
U-RIB:U-Y:ILE-S1 U-RIB:U-Y:ILE-S1_220 -3800.86392306
20   0
40   0
60   1
80   1
100   1
120   1
140   1
160   0
180   0
200   0
220   0
240   1
260   1
280   1
300   1
320   1
340   1
360   0
A-P:A-RIB:TYR-S1 A-P:A-RIB:TYR-S1_60 -5806.28939889
20   0
40   0
60   0
80   1
100   2
120   2
140   1
160   1
180   0
200   0
220   0
```

240 0
260 1
280 2
300 2
320 2
340 1
360 0
C-P:C-RIB:TRP-CA C-P:C-RIB:TRP-CA_80 -2746.94741601
20 0
40 0
60 0
80 0
100 1
120 1
140 0
160 0
180 0
200 0
220 0
240 0
260 0
280 1
300 0
320 0
340 0
360 0
U31-RIB:U31-MY:PHE-S2 U31-RIB:U31-MY:PHE-S2_140 0.0
20 0
40 0
60 0
80 0
100 0
120 0
140 0
160 0
180 0
200 0
220 0
240 0
260 0
280 0
300 0
320 0
340 0
360 0
IU-RIB:IU-P:LYS-CA IU-RIB:IU-P:LYS-CA_260 0.0
20 0
40 0
60 0
80 0
100 0
120 0
140 0
160 0
180 0
200 0
220 0
240 0

260 0
280 0
300 0
320 0
340 0
360 0
H2U-RIB:H2U-MY:GLU-S1 H2U-RIB:H2U-MY:GLU-S1_280 0.0
20 0
40 0
60 0
80 0
100 0
120 0
140 0
160 0
180 0
200 0
220 0
240 0
260 0
280 0
300 0
320 0
340 0
360 0
QUO-P:QUO-RIB:ASN-S1 QUO-P:QUO-RIB:ASN-S1_340 0.0
20 0
40 0
60 0
80 0
100 0
120 0
140 0
160 0
180 0
200 0
220 0
240 0
260 0
280 0
300 0
320 0
340 0
360 0
U34-RIB:U34-P:TYR-CA U34-RIB:U34-P:TYR-CA_260 0.0
20 0
40 0
60 0
80 0
100 0
120 0
140 0
160 0
180 0
200 0
220 0
240 0
260 0

280   0
300   0
320   0
340   0
360   0
C31-P:C31-RIB:ASP-S2 C31-P:C31-RIB:ASP-S2_120 0.0
20   0
40   0
60   0
80   0
100   0
120   0
140   0
160   0
180   0
200   0
220   0
240   0
260   0
280   0
300   0
320   0
340   0
360   0
C-P:C-RIB:HIS-S2 C-P:C-RIB:HIS-S2_180 -5364.24648805
20   0
40   0
60   2
80   3
100   3
120   3
140   3
160   2
180   1
200   0
220   0
240   2
260   3
280   3
300   3
320   3
340   3
360   1
QUO-RIB:QUO-M5:ASP-S2 QUO-RIB:QUO-M5:ASP-S2_40 0.0
20   0
40   0
60   0
80   0
100   0
120   0
140   0
160   0
180   0
200   0
220   0
240   0
260   0
280   0

300 0
320 0
340 0
360 0
H2U-RIB:H2U-MY:LYS-S2 H2U-RIB:H2U-MY:LYS-S2_120 0.0
20 0
40 0
60 0
80 0
100 0
120 0
140 0
160 0
180 0
200 0
220 0
240 0
260 0
280 0
300 0
320 0
340 0
360 0
H2U-P:H2U-RIB:PHE-S1 H2U-P:H2U-RIB:PHE-S1_140 0.0
20 0
40 0
60 0
80 0
100 0
120 0
140 0
160 0
180 0
200 0
220 0
240 0
260 0
280 0
300 0
320 0
340 0
360 0
QUO-RIB:QUO-M6:LYS-S2 QUO-RIB:QUO-M6:LYS-S2_100 0.0
20 0
40 0
60 0
80 0
100 0
120 0
140 0
160 0
180 0
200 0
220 0
240 0
260 0
280 0
300 0

320 0
340 0
360 0
G-RIB:G-R6:PHE-S1 G-RIB:G-R6:PHE-S1_280 -2177.59854723
20 0
40 0
60 0
80 1
100 1
120 1
140 1
160 1
180 0
200 0
220 0
240 0
260 1
280 1
300 1
320 0
340 0
360 0
U31-RIB:U31-MY:GLN-S2 U31-RIB:U31-MY:GLN-S2_160 0.0
20 0
40 0
60 0
80 0
100 0
120 0
140 0
160 0
180 0
200 0
220 0
240 0
260 0
280 0
300 0
320 0
340 0
360 0
G-P:G-RIB:LEU-S2 G-P:G-RIB:LEU-S2_120 -297.10325965
20 0
40 0
60 4
80 6
100 7
120 7
140 6
160 4
180 1
200 0
220 1
240 4
260 6
280 6
300 6
320 5

340 3
360 1
C-P:C-RIB:ASN-S1 C-P:C-RIB:ASN-S1_220 -4818.92972913
20 0
40 1
60 4
80 5
100 6
120 5
140 5
160 4
180 2
200 0
220 1
240 4
260 5
280 6
300 6
320 5
340 4
360 2
G-RIB:G-P:HIS-CA G-RIB:G-P:HIS-CA_60 -5418.82386333
20 0
40 0
60 1
80 4
100 5
120 6
140 5
160 3
180 1
200 0
220 0
240 1
260 4
280 5
300 5
320 4
340 3
360 0
G-RIB:G-R6:HIS-S2 G-RIB:G-R6:HIS-S2_320 -4155.07690533
20 0
40 1
60 3
80 4
100 4
120 3
140 2
160 2
180 0
200 0
220 1
240 3
260 4
280 4
300 4
320 2
340 0

360 0
G-RIB:G-P:ASN-S2 G-RIB:G-P:ASN-S2_320 -2599.13576695
20 0
40 2
60 6
80 10
100 12
120 11
140 10
160 7
180 3
200 0
220 2
240 5
260 9
280 11
300 11
320 9
340 8
360 2
U-RIB:U-Y:ARG-S1 U-RIB:U-Y:ARG-S1_340 -5393.98508598
20 0
40 2
60 3
80 3
100 4
120 4
140 3
160 2
180 0
200 0
220 2
240 3
260 4
280 4
300 4
320 3
340 2
360 0
G-RIB:G-R5:PHE-S1 G-RIB:G-R5:PHE-S1_20 -6669.83105986
20 0
40 0
60 1
80 1
100 1
120 0
140 1
160 0
180 0
200 0
220 0
240 1
260 1
280 1
300 1
320 0
340 0
360 0

U-RIB:U-P:TYR-S1 U-RIB:U-P:TYR-S1_120 -955.560809652
20 0
40 0
60 0
80 1
100 1
120 2
140 1
160 0
180 0
200 0
220 0
240 0
260 1
280 1
300 1
320 0
340 0
360 0
C-P:C-RIB:ALA-S1 C-P:C-RIB:ALA-S1_180 -4506.77383301
20 0
40 2
60 5
80 7
100 8
120 9
140 8
160 6
180 2
200 0
220 2
240 5
260 7
280 8
300 8
320 8
340 6
360 2
A-RIB:A-R6:ARG-CA A-RIB:A-R6:ARG-CA_220 -6352.73101036
20 0
40 0
60 3
80 5
100 6
120 7
140 5
160 4
180 0
200 0
220 0
240 3
260 5
280 7
300 6
320 5
340 4
360 0
IU-RIB:IU-P:ASP-S2 IU-RIB:IU-P:ASP-S2_60 0.0

20 0
40 0
60 0
80 0
100 0
120 0
140 0
160 0
180 0
200 0
220 0
240 0
260 0
280 0
300 0
320 0
340 0
360 0
G-RIB:G-R6:CYS-S1 G-RIB:G-R6:CYS-S1_280 0.0
20 0
40 0
60 0
80 0
100 0
120 0
140 0
160 0
180 0
200 0
220 0
240 0
260 0
280 0
300 0
320 0
340 0
360 0
G-RIB:G-R5:ASP-CA G-RIB:G-R5:ASP-CA_360 0.0
20 0
40 3
60 6
80 7
100 6
120 0
140 5
160 3
180 0
200 0
220 3
240 6
260 7
280 6
300 5
320 5
340 3
360 0
GTP-RIB:GTP-M5:SER-S1 GTP-RIB:GTP-M5:SER-S1_240 0.0
20 0

40 0
60 0
80 0
100 0
120 0
140 0
160 0
180 0
200 0
220 0
240 0
260 0
280 0
300 0
320 0
340 0
360 0
U34-P:U34-RIB:ASN-CA U34-P:U34-RIB:ASN-CA_260 -11963.1740541
20 0
40 0
60 0
80 0
100 0
120 0
140 0
160 0
180 0
200 0
220 0
240 0
260 0
280 0
300 0
320 0
340 0
360 0
G-P:G-RIB:ASP-S2 G-P:G-RIB:ASP-S2_180 -1846.24894251
20 0
40 5
60 12
80 17
100 17
120 16
140 14
160 12
180 5
200 1
220 0
240 13
260 17
280 17
300 17
320 14
340 12
360 5
H2U-RIB:H2U-P:TRP-S1 H2U-RIB:H2U-P:TRP-S1_140 0.0
20 0
40 0

60   0
80   0
100   0
120   0
140   0
160   0
180   0
200   0
220   0
240   0
260   0
280   0
300   0
320   0
340   0
360   0
QUO-RIB:QUO-M6:ASP-S1 QUO-RIB:QUO-M6:ASP-S1_200 0.0
20   0
40   0
60   0
80   0
100   0
120   0
140   0
160   0
180   0
200   0
220   0
240   0
260   0
280   0
300   0
320   0
340   0
360   0
G-RIB:G-P:ARG-S1 G-RIB:G-P:ARG-S1_140 -4272.85580174
20   0
40   2
60   7
80   14
100   20
120   22
140   19
160   13
180   5
200   0
220   2
240   7
260   15
280   21
300   22
320   19
340   13
360   5
C-RIB:C-Y:THR-CA C-RIB:C-Y:THR-CA_200 -8452.09164244
20   0
40   1
60   2

80   3
100   3
120   2
140   2
160   1
180   0
200   0
220   1
240   3
260   3
280   3
300   3
320   2
340   1
360   0
C-P:C-RIB:TRP-S1 C-P:C-RIB:TRP-S1_100 -5988.31989091
20   0
40   0
60   0
80   1
100   1
120   1
140   0
160   0
180   0
200   0
220   0
240   0
260   1
280   1
300   0
320   0
340   0
360   0
U31-P:U31-RIB:ASP-S1 U31-P:U31-RIB:ASP-S1_360 0.0
20   0
40   0
60   0
80   0
100   0
120   0
140   0
160   0
180   0
200   0
220   0
240   0
260   0
280   0
300   0
320   0
340   0
360   0
G-RIB:G-P:LYS-S2 G-RIB:G-P:LYS-S2_220 -2417.03369985
20   1
40   6
60   13
80   23

100   28
120   26
140   24
160   18
180   7
200   0
220   6
240   14
260   23
280   28
300   26
320   23
340   18
360   7
FMU-RIB:FMU-MY:GLU-S1 FMU-RIB:FMU-MY:GLU-S1_280 0.0
20   0
40   0
60   0
80   0
100   0
120   0
140   0
160   0
180   0
200   0
220   0
240   0
260   0
280   0
300   0
320   0
340   0
360   0
G-P:G-RIB:VAL-CA G-P:G-RIB:VAL-CA_160 -2658.4482707
20   0
40   0
60   4
80   7
100   9
120   8
140   7
160   5
180   0
200   0
220   1
240   4
260   7
280   9
300   9
320   8
340   5
360   2
C-P:C-RIB:LEU-S1 C-P:C-RIB:LEU-S1_320 -3998.58845943
20   0
40   0
60   2
80   4
100   5

120   5
140   4
160   3
180   1
200   0
220   0
240   2
260   4
280   4
300   5
320   4
340   3
360   1
IU-P:IU-RIB:LYS-CA IU-P:IU-RIB:LYS-CA_320 0.0
20   0
40   0
60   0
80   0
100   0
120   0
140   0
160   0
180   0
200   0
220   0
240   0
260   0
280   0
300   0
320   0
340   0
360   0
DA-RIB:DA-M5:THR-S1 DA-RIB:DA-M5:THR-S1_40 0.0
20   0
40   0
60   0
80   0
100   0
120   0
140   0
160   0
180   0
200   0
220   0
240   0
260   0
280   0
300   0
320   0
340   0
360   0
U-RIB:U-Y:TYR-S1 U-RIB:U-Y:TYR-S1_20 0.0
20   0
40   0
60   0
80   0
100   0
120   1

140   0
160   0
180   0
200   0
220   0
240   0
260   0
280   1
300   1
320   0
340   0
360   0
C31-RIB:C31-P:GLN-S2 C31-RIB:C31-P:GLN-S2_340 0.0
20   0
40   0
60   0
80   0
100   0
120   0
140   0
160   0
180   0
200   0
220   0
240   0
260   0
280   0
300   0
320   0
340   0
360   0
U34-P:U34-RIB:ASN-S2 U34-P:U34-RIB:ASN-S2_40 0.0
20   0
40   0
60   0
80   0
100   0
120   0
140   0
160   0
180   0
200   0
220   0
240   0
260   0
280   0
300   0
320   0
340   0
360   0
C-RIB:C-Y:LYS-S2 C-RIB:C-Y:LYS-S2_80 -4485.44083764
20   2
40   6
60   11
80   12
100   9
120   7
140   6

160   4
180   1
200   2
220   7
240   11
260   14
280   10
300   7
320   6
340   5
360   2
DA-RIB:DA-M6:ASN-S1 DA-RIB:DA-M6:ASN-S1_60 0.0
20   0
40   0
60   0
80   0
100   0
120   0
140   0
160   0
180   0
200   0
220   0
240   0
260   0
280   0
300   0
320   0
340   0
360   0
G-RIB:G-R5:ARG-CA G-RIB:G-R5:ARG-CA_80 -2595.41239739
20   0
40   3
60   5
80   5
100   5
120   5
140   4
160   3
180   1
200   0
220   3
240   5
260   5
280   5
300   5
320   4
340   2
360   1
G-RIB:G-P:THR-S1 G-RIB:G-P:THR-S1_160 -3117.651695
20   0
40   0
60   5
80   9
100   12
120   12
140   11
160   7

180   3
200   0
220   0
240   5
260   9
280   12
300   12
320   11
340   8
360   3
U-RIB:U-P:GLN-S1 U-RIB:U-P:GLN-S1_160 -2823.19074414
20   0
40   0
60   2
80   3
100   4
120   4
140   4
160   2
180   1
200   0
220   0
240   1
260   3
280   4
300   4
320   3
340   2
360   1
A-RIB:A-R6:GLU-CA A-RIB:A-R6:GLU-CA_240 -2533.07945754
20   0
40   1
60   0
80   8
100   9
120   8
140   7
160   5
180   0
200   0
220   1
240   4
260   8
280   9
300   8
320   7
340   0
360   0
U-P:U-RIB:SER-CA U-P:U-RIB:SER-CA_40 -5152.25862523
20   0
40   1
60   3
80   4
100   5
120   4
140   4
160   3
180   1

200   0
220   1
240   3
260   4
280   5
300   4
320   4
340   3
360   1
U-P:U-RIB:LYS-CA U-P:U-RIB:LYS-CA_140 -2015.91987651
20   0
40   1
60   3
80   5
100   5
120   5
140   5
160   4
180   1
200   0
220   1
240   3
260   5
280   6
300   6
320   5
340   4
360   1
A-RIB:A-R5:CYS-CA A-RIB:A-R5:CYS-CA_160 0.0
20   0
40   0
60   0
80   0
100   0
120   0
140   0
160   0
180   0
200   0
220   0
240   0
260   0
280   0
300   0
320   0
340   0
360   0
G-RIB:G-R6:HIS-CA G-RIB:G-R6:HIS-CA_340 0.0
20   0
40   0
60   1
80   2
100   3
120   2
140   1
160   0
180   0
200   0

```
220   0
240   1
260   2
280   3
300   2
320   2
340   0
360   0
C-P:C-RIB:GLN-S1 C-P:C-RIB:GLN-S1_280 -3091.42352699
20   0
40   1
60   2
80   4
100   4
120   4
140   4
160   3
180   1
200   0
220   1
240   2
260   4
280   5
300   5
320   4
340   3
360   1
IU-RIB:IU-MY:ARG-CA IU-RIB:IU-MY:ARG-CA_300 0.0
20   0
40   0
60   0
80   0
100   0
120   0
140   0
160   0
180   0
200   0
220   0
240   0
260   0
280   0
300   0
320   0
340   0
360   0
A-RIB:A-R6:TRP-S2 A-RIB:A-R6:TRP-S2_140 0.0
20   0
40   0
60   0
80   1
100   1
120   1
140   0
160   0
180   0
200   0
220   0
```

240   0
260   1
280   0
300   1
320   0
340   0
360   0
C31-P:C31-RIB:TYR-S2 C31-P:C31-RIB:TYR-S2_220 0.0
20   0
40   0
60   0
80   0
100   0
120   0
140   0
160   0
180   0
200   0
220   0
240   0
260   0
280   0
300   0
320   0
340   0
360   0
FHU-RIB:FHU-P:LEU-CA FHU-RIB:FHU-P:LEU-CA_260 0.0
20   0
40   0
60   0
80   0
100   0
120   0
140   0
160   0
180   0
200   0
220   0
240   0
260   0
280   0
300   0
320   0
340   0
360   0
OMC-RIB:OMC-P:LYS-CA OMC-RIB:OMC-P:LYS-CA_160 0.0
20   0
40   0
60   0
80   0
100   0
120   0
140   0
160   0
180   0
200   0
220   0
240   0

260 0
280 0
300 0
320 0
340 0
360 0
IU-RIB:IU-MY:ILE-S1 IU-RIB:IU-MY:ILE-S1_220 0.0
20 0
40 0
60 0
80 0
100 0
120 0
140 0
160 0
180 0
200 0
220 0
240 0
260 0
280 0
300 0
320 0
340 0
360 0
U-P:U-RIB:TYR-S2 U-P:U-RIB:TYR-S2_20 0.0
20 0
40 0
60 0
80 1
100 1
120 1
140 1
160 1
180 0
200 0
220 0
240 1
260 2
280 2
300 2
320 1
340 1
360 0
A-RIB:A-R5:GLN-S2 A-RIB:A-R5:GLN-S2_300 -2693.10215945
20 0
40 2
60 4
80 5
100 5
120 3
140 3
160 2
180 0
200 0
220 2
240 4
260 5

280   5
300   4
320   3
340   2
360   0
U-P:U-RIB:GLN-CA U-P:U-RIB:GLN-CA_220 -5793.41813537
20   0
40   0
60   1
80   2
100   3
120   3
140   2
160   1
180   0
200   0
220   0
240   1
260   2
280   3
300   3
320   2
340   1
360   0
A-RIB:A-R5:ALA-CA A-RIB:A-R5:ALA-CA_100 -1921.77192038
20   0
40   2
60   4
80   5
100   5
120   4
140   3
160   2
180   0
200   0
220   2
240   4
260   5
280   5
300   4
320   3
340   2
360   1
FMU-RIB:FMU-MY:SER-S1 FMU-RIB:FMU-MY:SER-S1_360 0.0
20   0
40   0
60   0
80   0
100   0
120   0
140   0
160   0
180   0
200   0
220   0
240   0
260   0
280   0

300 0
320 0
340 0
360 0
H2U-RIB:H2U-P:ARG-S2 H2U-RIB:H2U-P:ARG-S2_360 0.0
20 0
40 0
60 0
80 0
100 0
120 0
140 0
160 0
180 0
200 0
220 0
240 0
260 0
280 0
300 0
320 0
340 0
360 0
G-RIB:G-R6:TYR-CA G-RIB:G-R6:TYR-CA_320 -5275.87361652
20 0
40 0
60 0
80 1
100 1
120 1
140 1
160 1
180 0
200 0
220 0
240 0
260 1
280 1
300 1
320 1
340 0
360 0
FHU-P:FHU-RIB:LYS-S1 FHU-P:FHU-RIB:LYS-S1_40 -12675.9675659
20 0
40 0
60 0
80 0
100 0
120 0
140 0
160 0
180 0
200 0
220 0
240 0
260 0
280 0
300 0

320 0
340 0
360 0
U31-RIB:U31-P:ARG-S1 U31-RIB:U31-P:ARG-S1_240 0.0
20 0
40 0
60 0
80 0
100 0
120 0
140 0
160 0
180 0
200 0
220 0
240 0
260 0
280 0
300 0
320 0
340 0
360 0
C-RIB:C-Y:HIS-S1 C-RIB:C-Y:HIS-S1_180 -5576.40292368
20 0
40 0
60 1
80 1
100 1
120 1
140 1
160 0
180 0
200 0
220 1
240 1
260 1
280 1
300 1
320 1
340 0
360 0
G-RIB:G-R6:TYR-S1 G-RIB:G-R6:TYR-S1_40 0.0
20 0
40 0
60 1
80 1
100 1
120 1
140 0
160 1
180 0
200 0
220 0
240 0
260 1
280 2
300 1
320 1

340   0
360   0
A-RIB:A-P:LEU-S2 A-RIB:A-P:LEU-S2_100 -2002.44770176
20   0
40   1
60   2
80   4
100   5
120   5
140   4
160   3
180   0
200   0
220   0
240   2
260   4
280   5
300   5
320   5
340   3
360   1
FHU-RIB:FHU-P:LYS-CA FHU-RIB:FHU-P:LYS-CA_260 0.0
20   0
40   0
60   0
80   0
100   0
120   0
140   0
160   0
180   0
200   0
220   0
240   0
260   0
280   0
300   0
320   0
340   0
360   0
A-RIB:A-R5:HIS-S2 A-RIB:A-R5:HIS-S2_260 -6500.38310379
20   0
40   1
60   2
80   3
100   2
120   2
140   2
160   1
180   0
200   0
220   1
240   2
260   3
280   2
300   2
320   1
340   1

360 0
G-RIB:G-P:GLN-S2 G-RIB:G-P:GLN-S2_300 -2655.73296889
20 0
40 2
60 5
80 9
100 11
120 11
140 9
160 7
180 2
200 0
220 2
240 5
260 9
280 11
300 10
320 9
340 7
360 2
G-RIB:G-R6:ASN-CA G-RIB:G-R6:ASN-CA_160 -2631.68418452
20 0
40 0
60 2
80 4
100 5
120 4
140 3
160 2
180 0
200 0
220 0
240 3
260 5
280 5
300 4
320 3
340 2
360 0
QUO-RIB:QUO-M5:LEU-S1 QUO-RIB:QUO-M5:LEU-S1_140 0.0
20 0
40 0
60 0
80 0
100 0
120 0
140 0
160 0
180 0
200 0
220 0
240 0
260 0
280 0
300 0
320 0
340 0
360 0

DA-RIB:DA-M6:SER-CA DA-RIB:DA-M6:SER-CA_320 -6553.73679946
20 0
40 0
60 0
80 0
100 0
120 0
140 0
160 0
180 0
200 0
220 0
240 0
260 0
280 0
300 0
320 0
340 0
360 0
C-P:C-RIB:ARG-S1 C-P:C-RIB:ARG-S1_60 -5644.48857923
20 0
40 2
60 7
80 10
100 11
120 11
140 10
160 7
180 3
200 0
220 2
240 7
260 10
280 11
300 11
320 10
340 7
360 3
G-RIB:G-R6:TYR-S2 G-RIB:G-R6:TYR-S2_240 -5143.60177161
20 0
40 0
60 1
80 2
100 2
120 2
140 1
160 1
180 0
200 0
220 0
240 2
260 2
280 2
300 2
320 1
340 1
360 0
G-P:G-RIB:ARG-S2 G-P:G-RIB:ARG-S2_100 -3686.65926338

20   1
40   7
60   17
80   22
100   22
120   20
140   17
160   14
180   6
200   1
220   7
240   17
260   21
280   22
300   20
320   16
340   14
360   6
C-RIB:C-Y:ARG-S1 C-RIB:C-Y:ARG-S1_280 -5159.70293526
20   1
40   3
60   5
80   6
100   5
120   5
140   3
160   2
180   1
200   1
220   3
240   6
260   6
280   5
300   5
320   4
340   2
360   0
FHU-RIB:FHU-MY:LYS-S1 FHU-RIB:FHU-MY:LYS-S1_260 -8772.24118483
20   0
40   0
60   0
80   0
100   0
120   0
140   0
160   0
180   0
200   0
220   0
240   0
260   0
280   0
300   0
320   0
340   0
360   0
U31-RIB:U31-P:MET-S1 U31-RIB:U31-P:MET-S1_40 0.0
20   0

```
40   0
60   0
80   0
100   0
120   0
140   0
160   0
180   0
200   0
220   0
240   0
260   0
280   0
300   0
320   0
340   0
360   0
H2U-RIB:H2U-P:LYS-S2 H2U-RIB:H2U-P:LYS-S2_360 0.0
20   0
40   0
60   0
80   0
100   0
120   0
140   0
160   0
180   0
200   0
220   0
240   0
260   0
280   0
300   0
320   0
340   0
360   0
G-P:G-RIB:LYS-S1 G-P:G-RIB:LYS-S1_20 -3567.15086383
20   0
40   5
60   12
80   17
100   17
120   16
140   13
160   10
180   5
200   0
220   5
240   12
260   17
280   17
300   16
320   14
340   10
360   5
G-RIB:G-P:ASP-CA G-RIB:G-P:ASP-CA_300 -3440.90312906
20   0
40   0
```

60   0
80   12
100   17
120   18
140   15
160   10
180   3
200   0
220   1
240   6
260   11
280   16
300   17
320   15
340   10
360   4
FMU-RIB:FMU-P:PHE-CA FMU-RIB:FMU-P:PHE-CA_100 0.0
20   0
40   0
60   0
80   0
100   0
120   0
140   0
160   0
180   0
200   0
220   0
240   0
260   0
280   0
300   0
320   0
340   0
360   0
GTP-RIB:GTP-M5:ALA-S1 GTP-RIB:GTP-M5:ALA-S1_80 0.0
20   0
40   0
60   0
80   0
100   0
120   0
140   0
160   0
180   0
200   0
220   0
240   0
260   0
280   0
300   0
320   0
340   0
360   0
G-RIB:G-R6:VAL-S1 G-RIB:G-R6:VAL-S1_140 0.0
20   0
40   1
60   0

80 4
100 5
120 5
140 0
160 0
180 1
200 0
220 1
240 3
260 5
280 5
300 5
320 4
340 2
360 0
FHU-RIB:FHU-MY:THR-CA FHU-RIB:FHU-MY:THR-CA_100 0.0
20 0
40 0
60 0
80 0
100 0
120 0
140 0
160 0
180 0
200 0
220 0
240 0
260 0
280 0
300 0
320 0
340 0
360 0
A-RIB:A-P:TRP-CA A-RIB:A-P:TRP-CA_280 -2463.47144092
20 0
40 0
60 0
80 1
100 1
120 1
140 1
160 0
180 0
200 0
220 0
240 0
260 0
280 1
300 1
320 1
340 0
360 0
A-RIB:A-P:ASN-S2 A-RIB:A-P:ASN-S2_240 -5431.73344862
20 0
40 1
60 4
80 7

100   8
120   8
140   7
160   5
180   2
200   0
220   0
240   4
260   6
280   8
300   8
320   7
340   5
360   0
G-RIB:G-R5:ASP-S1 G-RIB:G-R5:ASP-S1_220 -3590.78582195
20   0
40   5
60   9
80   10
100   8
120   7
140   6
160   4
180   0
200   1
220   5
240   9
260   10
280   8
300   7
320   5
340   4
360   1
C-RIB:C-P:ARG-S1 C-RIB:C-P:ARG-S1_100 -5073.1166751
20   0
40   1
60   5
80   10
100   13
120   14
140   12
160   8
180   3
200   0
220   1
240   5
260   10
280   14
300   14
320   12
340   8
360   3
FMU-P:FMU-RIB:ARG-CA FMU-P:FMU-RIB:ARG-CA_360 0.0
20   0
40   0
60   0
80   0
100   0

120   0
140   0
160   0
180   0
200   0
220   0
240   0
260   0
280   0
300   0
320   0
340   0
360   0
U-RIB:U-P:LYS-S2 U-RIB:U-P:LYS-S2_220 -3575.00415933
20   0
40   2
60   6
80   10
100   11
120   11
140   9
160   7
180   2
200   0
220   2
240   6
260   9
280   11
300   10
320   9
340   7
360   2
C-P:C-RIB:LEU-CA C-P:C-RIB:LEU-CA_20 0.0
20   0
40   0
60   2
80   4
100   5
120   5
140   5
160   3
180   1
200   0
220   0
240   2
260   4
280   5
300   5
320   5
340   3
360   0
C-RIB:C-P:LEU-S2 C-RIB:C-P:LEU-S2_180 0.0
20   0
40   0
60   2
80   4
100   5
120   6

140   5
160   4
180   0
200   0
220   0
240   2
260   4
280   5
300   6
320   5
340   3
360   1
IU-RIB:IU-MY:MET-CA IU-RIB:IU-MY:MET-CA_360 0.0
20   0
40   0
60   0
80   0
100   0
120   0
140   0
160   0
180   0
200   0
220   0
240   0
260   0
280   0
300   0
320   0
340   0
360   0
QUO-RIB:QUO-M6:LYS-S1 QUO-RIB:QUO-M6:LYS-S1_60 0.0
20   0
40   0
60   0
80   0
100   0
120   0
140   0
160   0
180   0
200   0
220   0
240   0
260   0
280   0
300   0
320   0
340   0
360   0
C31-RIB:C31-MY:ASP-CA C31-RIB:C31-MY:ASP-CA_160 0.0
20   0
40   0
60   0
80   0
100   0
120   0
140   0

160   0
180   0
200   0
220   0
240   0
260   0
280   0
300   0
320   0
340   0
360   0
FHU-RIB:FHU-P:ASP-CA FHU-RIB:FHU-P:ASP-CA_220 0.0
20   0
40   0
60   0
80   0
100   0
120   0
140   0
160   0
180   0
200   0
220   0
240   0
260   0
280   0
300   0
320   0
340   0
360   0
C-RIB:C-P:ARG-S2 C-RIB:C-P:ARG-S2_180 -4426.2857597
20   0
40   3
60   8
80   14
100   18
120   17
140   14
160   11
180   4
200   0
220   3
240   9
260   14
280   18
300   17
320   15
340   11
360   4
A-P:A-RIB:LYS-CA A-P:A-RIB:LYS-CA_20 -8606.99912219
20   0
40   1
60   5
80   8
100   9
120   9
140   8
160   7

180 3
200 0
220 1
240 5
260 8
280 9
300 9
320 9
340 6
360 3
A-RIB:A-R6:GLN-CA A-RIB:A-R6:GLN-CA_280 0.0
20 0
40 0
60 1
80 2
100 3
120 3
140 2
160 1
180 0
200 0
220 0
240 1
260 2
280 0
300 3
320 2
340 2
360 0
G-RIB:G-R6:MET-S2 G-RIB:G-R6:MET-S2_80 -4048.33813931
20 0
40 0
60 2
80 2
100 2
120 2
140 0
160 0
180 0
200 0
220 0
240 1
260 2
280 2
300 2
320 1
340 0
360 0
FHU-RIB:FHU-MY:ALA-CA FHU-RIB:FHU-MY:ALA-CA_300 -9953.9219942
20 0
40 0
60 0
80 0
100 0
120 0
140 0
160 0
180 0

200 0
220 0
240 0
260 0
280 0
300 0
320 0
340 0
360 0
U-P:U-RIB:MET-S2 U-P:U-RIB:MET-S2_40 0.0
20 0
40 0
60 0
80 1
100 1
120 1
140 0
160 0
180 0
200 0
220 0
240 0
260 1
280 1
300 1
320 1
340 0
360 0
U-P:U-RIB:ALA-CA U-P:U-RIB:ALA-CA_100 -3815.74800857
20 0
40 1
60 3
80 4
100 5
120 5
140 4
160 3
180 1
200 0
220 1
240 2
260 4
280 5
300 5
320 4
340 3
360 1
G-P:G-RIB:TYR-S2 G-P:G-RIB:TYR-S2_260 -4494.58334024
20 0
40 0
60 2
80 3
100 4
120 3
140 3
160 2
180 1
200 0

220 0
240 2
260 3
280 4
300 4
320 3
340 2
360 0
C-RIB:C-Y:MET-S1 C-RIB:C-Y:MET-S1_40 -6227.2503926
20 0
40 0
60 1
80 1
100 1
120 0
140 0
160 0
180 0
200 0
220 0
240 0
260 1
280 1
300 0
320 0
340 0
360 0
C-P:C-RIB:THR-S1 C-P:C-RIB:THR-S1_220 -3732.87363902
20 0
40 2
60 4
80 6
100 7
120 6
140 6
160 4
180 2
200 0
220 1
240 4
260 6
280 7
300 7
320 6
340 5
360 2
A-RIB:A-R5:ASP-CA A-RIB:A-R5:ASP-CA_340 -2631.42823568
20 0
40 2
60 4
80 6
100 6
120 5
140 4
160 2
180 0
200 0
220 0

```
240   5
260   6
280   6
300   5
320   4
340   2
360   0
FMU-RIB:FMU-MY:PHE-S1 FMU-RIB:FMU-MY:PHE-S1_140 0.0
20   0
40   0
60   0
80   0
100   0
120   0
140   0
160   0
180   0
200   0
220   0
240   0
260   0
280   0
300   0
320   0
340   0
360   0
IU-P:IU-RIB:ILE-CA IU-P:IU-RIB:ILE-CA_140 0.0
20   0
40   0
60   0
80   0
100   0
120   0
140   0
160   0
180   0
200   0
220   0
240   0
260   0
280   0
300   0
320   0
340   0
360   0
H2U-RIB:H2U-MY:ILE-CA H2U-RIB:H2U-MY:ILE-CA_60 0.0
20   0
40   0
60   0
80   0
100   0
120   0
140   0
160   0
180   0
200   0
220   0
240   0
```

260 0
280 0
300 0
320 0
340 0
360 0
C-P:C-RIB:ASN-CA C-P:C-RIB:ASN-CA_40 -3110.18869943
20 0
40 0
60 2
80 4
100 5
120 5
140 4
160 3
180 1
200 0
220 0
240 2
260 4
280 5
300 5
320 4
340 3
360 1
U-RIB:U-P:LEU-S1 U-RIB:U-P:LEU-S1_140 -1373.28507834
20 0
40 0
60 1
80 2
100 3
120 4
140 3
160 2
180 0
200 0
220 0
240 1
260 2
280 3
300 3
320 3
340 2
360 0
C-P:C-RIB:GLU-S1 C-P:C-RIB:GLU-S1_320 249.629310002
20 0
40 3
60 9
80 13
100 15
120 14
140 13
160 11
180 5
200 0
220 0
240 9
260 13

280   15
300   14
320   13
340   10
360   5
U31-P:U31-RIB:GLN-CA U31-P:U31-RIB:GLN-CA_180 0.0
20   0
40   0
60   0
80   0
100   0
120   0
140   0
160   0
180   0
200   0
220   0
240   0
260   0
280   0
300   0
320   0
340   0
360   0
A-RIB:A-P:CYS-CA A-RIB:A-P:CYS-CA_200 0.0
20   0
40   0
60   0
80   0
100   0
120   0
140   0
160   0
180   0
200   0
220   0
240   0
260   0
280   0
300   0
320   0
340   0
360   0
H2U-RIB:H2U-MY:TRP-S1 H2U-RIB:H2U-MY:TRP-S1_320 -17115.4326793
20   0
40   0
60   0
80   0
100   0
120   0
140   0
160   0
180   0
200   0
220   0
240   0
260   0
280   0

300   0
320   0
340   0
360   0
G-RIB:G-R5:SER-CA G-RIB:G-R5:SER-CA_340 -3506.90479538
20   1
40   4
60   7
80   7
100   5
120   5
140   4
160   2
180   1
200   1
220   4
240   7
260   7
280   5
300   5
320   3
340   2
360   0
C-RIB:C-P:PRO-CA C-RIB:C-P:PRO-CA_20 0.0
20   0
40   1
60   3
80   5
100   7
120   7
140   6
160   4
180   1
200   0
220   0
240   3
260   5
280   6
300   7
320   6
340   4
360   1
G-RIB:G-P:LEU-S2 G-RIB:G-P:LEU-S2_100 -3133.89904932
20   0
40   0
60   3
80   6
100   7
120   9
140   8
160   5
180   2
200   0
220   1
240   3
260   6
280   8
300   9

320   8
340   5
360   2
FMU-P:FMU-RIB:ASP-S2 FMU-P:FMU-RIB:ASP-S2_200 0.0
20   0
40   0
60   0
80   0
100   0
120   0
140   0
160   0
180   0
200   0
220   0
240   0
260   0
280   0
300   0
320   0
340   0
360   0
FMU-P:FMU-RIB:MET-S1 FMU-P:FMU-RIB:MET-S1_320 -2722.04557167
20   0
40   0
60   0
80   0
100   0
120   0
140   0
160   0
180   0
200   0
220   0
240   0
260   0
280   0
300   0
320   0
340   0
360   0
A-P:A-RIB:PHE-S2 A-P:A-RIB:PHE-S2_300 -5631.90600802
20   0
40   0
60   1
80   2
100   2
120   2
140   2
160   1
180   0
200   0
220   0
240   0
260   2
280   2
300   2
320   0

340 1
360 0
IU-RIB:IU-MY:PRO-S1 IU-RIB:IU-MY:PRO-S1_140 0.0
20 0
40 0
60 0
80 0
100 0
120 0
140 0
160 0
180 0
200 0
220 0
240 0
260 0
280 0
300 0
320 0
340 0
360 0
U31-RIB:U31-P:GLN-S1 U31-RIB:U31-P:GLN-S1_260 0.0
20 0
40 0
60 0
80 0
100 0
120 0
140 0
160 0
180 0
200 0
220 0
240 0
260 0
280 0
300 0
320 0
340 0
360 0
U-RIB:U-P:PHE-S2 U-RIB:U-P:PHE-S2_200 0.0
20 0
40 0
60 0
80 0
100 2
120 2
140 1
160 1
180 0
200 0
220 0
240 0
260 1
280 2
300 2
320 0
340 0

360   0
A-RIB:A-R5:MET-S2 A-RIB:A-R5:MET-S2_360 -9190.03981352
20   0
40   0
60   1
80   1
100   1
120   1
140   1
160   0
180   0
200   0
220   0
240   1
260   1
280   0
300   1
320   1
340   0
360   0
DA-RIB:DA-M6:GLU-S2 DA-RIB:DA-M6:GLU-S2_220 0.0
20   0
40   0
60   0
80   0
100   0
120   0
140   0
160   0
180   0
200   0
220   0
240   0
260   0
280   0
300   0
320   0
340   0
360   0
C-RIB:C-P:TYR-S2 C-RIB:C-P:TYR-S2_40 0.0
20   0
40   0
60   1
80   2
100   3
120   3
140   3
160   2
180   0
200   0
220   0
240   1
260   2
280   3
300   3
320   2
340   1
360   0

C-P:C-RIB:PHE-CA C-P:C-RIB:PHE-CA_120 -3295.14481778
20 0
40 0
60 1
80 2
100 3
120 3
140 2
160 1
180 0
200 0
220 0
240 0
260 2
280 2
300 2
320 2
340 0
360 0
OMC-P:OMC-RIB:LYS-S1 OMC-P:OMC-RIB:LYS-S1_40 0.0
20 0
40 0
60 0
80 0
100 0
120 0
140 0
160 0
180 0
200 0
220 0
240 0
260 0
280 0
300 0
320 0
340 0
360 0
C-P:C-RIB:GLN-S2 C-P:C-RIB:GLN-S2_20 -4717.0915871
20 0
40 1
60 4
80 5
100 5
120 6
140 5
160 4
180 2
200 0
220 1
240 4
260 5
280 6
300 5
320 5
340 4
360 2
C-RIB:C-Y:THR-S1 C-RIB:C-Y:THR-S1_240 -4692.82172322

20   0
40   2
60   4
80   4
100   4
120   3
140   3
160   2
180   0
200   0
220   2
240   4
260   4
280   4
300   3
320   3
340   2
360   0
IU-P:IU-RIB:HIS-S2 IU-P:IU-RIB:HIS-S2_160 0.0
20   0
40   0
60   0
80   0
100   0
120   0
140   0
160   0
180   0
200   0
220   0
240   0
260   0
280   0
300   0
320   0
340   0
360   0
C-P:C-RIB:ASP-CA C-P:C-RIB:ASP-CA_340 -3386.85371185
20   0
40   1
60   5
80   8
100   9
120   9
140   8
160   6
180   3
200   0
220   1
240   5
260   8
280   10
300   9
320   8
340   6
360   3
H2U-RIB:H2U-P:ASN-S1 H2U-RIB:H2U-P:ASN-S1_20 0.0
20   0

40   0
60   0
80   0
100   0
120   0
140   0
160   0
180   0
200   0
220   0
240   0
260   0
280   0
300   0
320   0
340   0
360   0
U34-RIB:U34-P:SER-S1 U34-RIB:U34-P:SER-S1_200 0.0
20   0
40   0
60   0
80   0
100   0
120   0
140   0
160   0
180   0
200   0
220   0
240   0
260   0
280   0
300   0
320   0
340   0
360   0
G-P:G-RIB:PHE-S1 G-P:G-RIB:PHE-S1_120 -5463.36821193
20   0
40   0
60   0
80   2
100   3
120   3
140   2
160   1
180   0
200   0
220   0
240   1
260   2
280   3
300   3
320   2
340   1
360   0
G-RIB:G-R6:TRP-S2 G-RIB:G-R6:TRP-S2_340 0.0
20   0
40   0

```
60   0
80   1
100   1
120   0
140   0
160   0
180   0
200   0
220   0
240   0
260   1
280   1
300   1
320   0
340   0
360   0
G-P:G-RIB:GLN-CA G-P:G-RIB:GLN-CA_180 -5871.66819614
20   0
40   1
60   3
80   5
100   6
120   5
140   4
160   3
180   1
200   0
220   1
240   3
260   5
280   6
300   6
320   4
340   3
360   1
C31-RIB:C31-MY:LEU-CA C31-RIB:C31-MY:LEU-CA_160 0.0
20   0
40   0
60   0
80   0
100   0
120   0
140   0
160   0
180   0
200   0
220   0
240   0
260   0
280   0
300   0
320   0
340   0
360   0
FMU-P:FMU-RIB:PHE-S1 FMU-P:FMU-RIB:PHE-S1_140 0.0
20   0
40   0
60   0
```

```
80   0
100   0
120   0
140   0
160   0
180   0
200   0
220   0
240   0
260   0
280   0
300   0
320   0
340   0
360   0
QUO-RIB:QUO-M6:GLU-CA QUO-RIB:QUO-M6:GLU-CA_320 0.0
20   0
40   0
60   0
80   0
100   0
120   0
140   0
160   0
180   0
200   0
220   0
240   0
260   0
280   0
300   0
320   0
340   0
360   0
G-P:G-RIB:GLU-CA G-P:G-RIB:GLU-CA_20 0.0
20   0
40   0
60   9
80   14
100   17
120   17
140   14
160   10
180   5
200   0
220   3
240   9
260   15
280   17
300   16
320   14
340   10
360   5
FMU-P:FMU-RIB:GLN-S2 FMU-P:FMU-RIB:GLN-S2_360 0.0
20   0
40   0
60   0
80   0
```

```
100   0
120   0
140   0
160   0
180   0
200   0
220   0
240   0
260   0
280   0
300   0
320   0
340   0
360   0
C31-P:C31-RIB:GLU-CA C31-P:C31-RIB:GLU-CA_220 0.0
20   0
40   0
60   0
80   0
100   0
120   0
140   0
160   0
180   0
200   0
220   0
240   0
260   0
280   0
300   0
320   0
340   0
360   0
C31-RIB:C31-P:GLN-S1 C31-RIB:C31-P:GLN-S1_200 0.0
20   0
40   0
60   0
80   0
100   0
120   0
140   0
160   0
180   0
200   0
220   0
240   0
260   0
280   0
300   0
320   0
340   0
360   0
U-RIB:U-P:LYS-S1 U-RIB:U-P:LYS-S1_140 -3791.74872944
20   0
40   1
60   4
80   7
100   9
```

120   9
140   8
160   6
180   2
200   0
220   1
240   3
260   7
280   9
300   9
320   8
340   6
360   2
U31-RIB:U31-MY:THR-S1 U31-RIB:U31-MY:THR-S1_160 -9953.9219942
20   0
40   0
60   0
80   0
100   0
120   0
140   0
160   0
180   0
200   0
220   0
240   0
260   0
280   0
300   0
320   0
340   0
360   0
C-P:C-RIB:ALA-CA C-P:C-RIB:ALA-CA_340 -4086.29024978
20   0
40   1
60   4
80   7
100   8
120   8
140   7
160   6
180   2
200   0
220   1
240   4
260   7
280   8
300   8
320   7
340   5
360   2
A-RIB:A-R5:LYS-CA A-RIB:A-R5:LYS-CA_300 -2648.05573407
20   0
40   2
60   4
80   5
100   5
120   5

140   4
160   2
180   0
200   0
220   2
240   4
260   5
280   5
300   5
320   4
340   2
360   0
C31-RIB:C31-MY:GLU-S1 C31-RIB:C31-MY:GLU-S1_280 0.0
20   0
40   0
60   0
80   0
100   0
120   0
140   0
160   0
180   0
200   0
220   0
240   0
260   0
280   0
300   0
320   0
340   0
360   0
U31-RIB:U31-P:GLN-S2 U31-RIB:U31-P:GLN-S2_40 0.0
20   0
40   0
60   0
80   0
100   0
120   0
140   0
160   0
180   0
200   0
220   0
240   0
260   0
280   0
300   0
320   0
340   0
360   0
A-RIB:A-P:LEU-CA A-RIB:A-P:LEU-CA_160 -3657.64666942
20   0
40   0
60   1
80   3
100   5
120   6
140   5

160   3
180   0
200   0
220   0
240   1
260   4
280   5
300   6
320   5
340   3
360   1
G-RIB:G-P:ASN-S1 G-RIB:G-P:ASN-S1_60 -5392.37737909
20   0
40   1
60   4
80   8
100   10
120   10
140   9
160   6
180   2
200   0
220   1
240   4
260   8
280   10
300   10
320   8
340   6
360   2
DA-RIB:DA-M5:ASN-CA DA-RIB:DA-M5:ASN-CA_340 0.0
20   0
40   0
60   0
80   0
100   0
120   0
140   0
160   0
180   0
200   0
220   0
240   0
260   0
280   0
300   0
320   0
340   0
360   0
FHU-RIB:FHU-MY:THR-S1 FHU-RIB:FHU-MY:THR-S1_60 0.0
20   0
40   0
60   0
80   0
100   0
120   0
140   0
160   0

```
180   0
200   0
220   0
240   0
260   0
280   0
300   0
320   0
340   0
360   0
C-RIB:C-P:CYS-S1 C-RIB:C-P:CYS-S1_140 0.0
20   0
40   0
60   0
80   0
100   0
120   0
140   0
160   0
180   0
200   0
220   0
240   0
260   0
280   0
300   0
320   0
340   0
360   0
A-RIB:A-P:ASP-S2 A-RIB:A-P:ASP-S2_240 -2531.35822603
20   0
40   3
60   7
80   12
100   15
120   15
140   13
160   9
180   0
200   0
220   0
240   7
260   11
280   14
300   15
320   12
340   9
360   3
DA-RIB:DA-M6:HIS-S1 DA-RIB:DA-M6:HIS-S1_100 0.0
20   0
40   0
60   0
80   0
100   0
120   0
140   0
160   0
180   0
```

200   0
220   0
240   0
260   0
280   0
300   0
320   0
340   0
360   0
C31-RIB:C31-MY:THR-S1 C31-RIB:C31-MY:THR-S1_220 -10405.6624229
20   0
40   0
60   0
80   0
100   0
120   0
140   0
160   0
180   0
200   0
220   0
240   0
260   0
280   0
300   0
320   0
340   0
360   0
G-RIB:G-R5:TRP-S1 G-RIB:G-R5:TRP-S1_240 -5353.47380735
20   0
40   0
60   0
80   1
100   0
120   0
140   0
160   0
180   0
200   0
220   0
240   0
260   0
280   1
300   0
320   0
340   0
360   0
A-P:A-RIB:GLU-S2 A-P:A-RIB:GLU-S2_40 1192.25258129
20   0
40   6
60   13
80   17
100   17
120   17
140   16
160   13
180   5
200   0

220 0
240 12
260 17
280 18
300 17
320 15
340 13
360 5
DA-RIB:DA-M6:ASN-CA DA-RIB:DA-M6:ASN-CA_360 0.0
20 0
40 0
60 0
80 0
100 0
120 0
140 0
160 0
180 0
200 0
220 0
240 0
260 0
280 0
300 0
320 0
340 0
360 0
U-RIB:U-P:ARG-CA U-RIB:U-P:ARG-CA_160 -4174.20349505
20 0
40 0
60 2
80 4
100 6
120 7
140 6
160 4
180 1
200 0
220 0
240 2
260 4
280 6
300 7
320 6
340 4
360 1
DA-RIB:DA-M6:LEU-S2 DA-RIB:DA-M6:LEU-S2_20 0.0
20 0
40 0
60 0
80 0
100 0
120 0
140 0
160 0
180 0
200 0
220 0

240 0
260 0
280 0
300 0
320 0
340 0
360 0
FHU-RIB:FHU-P:TYR-S1 FHU-RIB:FHU-P:TYR-S1_180 -11963.1740541
20 0
40 0
60 0
80 0
100 0
120 0
140 0
160 0
180 0
200 0
220 0
240 0
260 0
280 0
300 0
320 0
340 0
360 0
FMU-RIB:FMU-MY:GLN-S2 FMU-RIB:FMU-MY:GLN-S2_60 0.0
20 0
40 0
60 0
80 0
100 0
120 0
140 0
160 0
180 0
200 0
220 0
240 0
260 0
280 0
300 0
320 0
340 0
360 0
A-RIB:A-R5:ARG-CA A-RIB:A-R5:ARG-CA_180 0.0
20 0
40 2
60 4
80 4
100 4
120 4
140 3
160 2
180 0
200 0
220 2
240 3

260 4
280 4
300 4
320 3
340 2
360 0
IU-P:IU-RIB:ALA-S1 IU-P:IU-RIB:ALA-S1_260 0.0
20 0
40 0
60 0
80 0
100 0
120 0
140 0
160 0
180 0
200 0
220 0
240 0
260 0
280 0
300 0
320 0
340 0
360 0
U-RIB:U-Y:ILE-CA U-RIB:U-Y:ILE-CA_280 -4096.04955692
20 0
40 0
60 1
80 1
100 1
120 2
140 1
160 0
180 0
200 0
220 0
240 1
260 1
280 1
300 1
320 1
340 1
360 0
G-RIB:G-P:MET-CA G-RIB:G-P:MET-CA_340 -4920.61641895
20 0
40 0
60 1
80 2
100 3
120 4
140 3
160 0
180 0
200 0
220 0
240 1
260 2

280   3
300   4
320   3
340   2
360   0
G-P:G-RIB:ALA-CA G-P:G-RIB:ALA-CA_100 -3149.2211479
20   0
40   3
60   8
80   11
100   12
120   12
140   10
160   7
180   3
200   0
220   3
240   8
260   11
280   12
300   12
320   10
340   8
360   3
FHU-P:FHU-RIB:ARG-CA FHU-P:FHU-RIB:ARG-CA_260 0.0
20   0
40   0
60   0
80   0
100   0
120   0
140   0
160   0
180   0
200   0
220   0
240   0
260   0
280   0
300   0
320   0
340   0
360   0
C31-RIB:C31-P:PHE-S2 C31-RIB:C31-P:PHE-S2_20 0.0
20   0
40   0
60   0
80   0
100   0
120   0
140   0
160   0
180   0
200   0
220   0
240   0
260   0
280   0

300   0
320   0
340   0
360   0
G-RIB:G-R5:THR-S1 G-RIB:G-R5:THR-S1_40 -2724.52452401
20   1
40   3
60   5
80   7
100   6
120   5
140   4
160   3
180   0
200   1
220   4
240   6
260   7
280   6
300   5
320   4
340   3
360   0
C-RIB:C-Y:ARG-S2 C-RIB:C-Y:ARG-S2_280 -4948.30726153
20   1
40   5
60   10
80   11
100   9
120   7
140   5
160   3
180   1
200   1
220   5
240   10
260   11
280   9
300   7
320   5
340   4
360   1
FHU-P:FHU-RIB:PRO-CA FHU-P:FHU-RIB:PRO-CA_80 0.0
20   0
40   0
60   0
80   0
100   0
120   0
140   0
160   0
180   0
200   0
220   0
240   0
260   0
280   0
300   0

320   0
340   0
360   0
U31-P:U31-RIB:ASN-CA U31-P:U31-RIB:ASN-CA_320 0.0
20   0
40   0
60   0
80   0
100   0
120   0
140   0
160   0
180   0
200   0
220   0
240   0
260   0
280   0
300   0
320   0
340   0
360   0
G-RIB:G-P:LYS-CA G-RIB:G-P:LYS-CA_180 -4805.96869178
20   0
40   1
60   6
80   12
100   19
120   20
140   16
160   11
180   4
200   0
220   1
240   6
260   12
280   19
300   20
320   17
340   11
360   4
G-RIB:G-R6:LEU-CA G-RIB:G-R6:LEU-CA_80 -4065.58970305
20   0
40   0
60   1
80   3
100   3
120   3
140   3
160   2
180   0
200   0
220   0
240   1
260   3
280   3
300   3
320   2

```
340   0
360   0
A-RIB:A-R6:THR-S1 A-RIB:A-R6:THR-S1_60 -2741.94698514
20   0
40   1
60   3
80   5
100   6
120   5
140   4
160   3
180   1
200   0
220   1
240   3
260   5
280   6
300   5
320   4
340   3
360   1
FHU-P:FHU-RIB:THR-CA FHU-P:FHU-RIB:THR-CA_20 0.0
20   0
40   0
60   0
80   0
100   0
120   0
140   0
160   0
180   0
200   0
220   0
240   0
260   0
280   0
300   0
320   0
340   0
360   0
H2U-RIB:H2U-MY:PRO-S1 H2U-RIB:H2U-MY:PRO-S1_340 0.0
20   0
40   0
60   0
80   0
100   0
120   0
140   0
160   0
180   0
200   0
220   0
240   0
260   0
280   0
300   0
320   0
340   0
```

360 0
FHU-RIB:FHU-MY:LEU-S1 FHU-RIB:FHU-MY:LEU-S1_140 -11174.1372141
20 0
40 0
60 0
80 0
100 0
120 0
140 0
160 0
180 0
200 0
220 0
240 0
260 0
280 0
300 0
320 0
340 0
360 0
U-RIB:U-P:TRP-S1 U-RIB:U-P:TRP-S1_60 -6553.73679946
20 0
40 0
60 0
80 0
100 0
120 0
140 0
160 0
180 0
200 0
220 0
240 0
260 0
280 0
300 0
320 0
340 0
360 0
C31-RIB:C31-MY:ALA-S1 C31-RIB:C31-MY:ALA-S1_120 0.0
20 0
40 0
60 0
80 0
100 0
120 0
140 0
160 0
180 0
200 0
220 0
240 0
260 0
280 0
300 0
320 0
340 0
360 0

FHU-P:FHU-RIB:ASP-CA FHU-P:FHU-RIB:ASP-CA_100 0.0
20 0
40 0
60 0
80 0
100 0
120 0
140 0
160 0
180 0
200 0
220 0
240 0
260 0
280 0
300 0
320 0
340 0
360 0
G-P:G-RIB:PHE-S2 G-P:G-RIB:PHE-S2_240 -4414.81105273
20 0
40 0
60 0
80 3
100 3
120 3
140 2
160 1
180 0
200 0
220 0
240 2
260 3
280 3
300 0
320 2
340 2
360 0
C-P:C-RIB:ILE-S1 C-P:C-RIB:ILE-S1_60 -3692.2241975
20 0
40 0
60 2
80 3
100 3
120 3
140 3
160 2
180 0
200 0
220 0
240 2
260 2
280 3
300 3
320 3
340 2
360 0
G-P:G-RIB:TRP-S2 G-P:G-RIB:TRP-S2_20 0.0

20   0
40   0
60   1
80   1
100   2
120   1
140   1
160   1
180   0
200   0
220   0
240   1
260   1
280   1
300   1
320   1
340   1
360   0
C-RIB:C-Y:PRO-CA C-RIB:C-Y:PRO-CA_40 -6088.94429846
20   0
40   1
60   2
80   3
100   3
120   2
140   2
160   1
180   0
200   0
220   1
240   3
260   3
280   3
300   2
320   2
340   1
360   0
A-RIB:A-P:THR-CA A-RIB:A-P:THR-CA_340 302.820175387
20   0
40   0
60   2
80   4
100   7
120   7
140   6
160   4
180   1
200   0
220   0
240   2
260   5
280   6
300   7
320   6
340   4
360   1
FHU-RIB:FHU-MY:ALA-S1 FHU-RIB:FHU-MY:ALA-S1_140 0.0
20   0

40 0
60 0
80 0
100 0
120 0
140 0
160 0
180 0
200 0
220 0
240 0
260 0
280 0
300 0
320 0
340 0
360 0
IU-RIB:IU-P:ILE-CA IU-RIB:IU-P:ILE-CA_140 0.0
20 0
40 0
60 0
80 0
100 0
120 0
140 0
160 0
180 0
200 0
220 0
240 0
260 0
280 0
300 0
320 0
340 0
360 0
A-RIB:A-R6:CYS-CA A-RIB:A-R6:CYS-CA_40 0.0
20 0
40 0
60 0
80 0
100 0
120 0
140 0
160 0
180 0
200 0
220 0
240 0
260 0
280 0
300 0
320 0
340 0
360 0
U-P:U-RIB:HIS-S2
20 0
40 0

60   1
80   2
100   2
120   2
140   2
160   1
180   0
200   0
220   0
240   1
260   2
280   2
300   2
320   2
340   1
360   0
G-P:G-RIB:GLN-S1 G-P:G-RIB:GLN-S1_40 -3757.21101983
20   0
40   1
60   4
80   7
100   7
120   7
140   5
160   4
180   2
200   0
220   1
240   5
260   7
280   7
300   7
320   6
340   4
360   2
5BU-P:5BU-RIB:ILE-S1 5BU-P:5BU-RIB:ILE-S1_220 0.0
20   0
40   0
60   0
80   0
100   0
120   0
140   0
160   0
180   0
200   0
220   0
240   0
260   0
280   0
300   0
320   0
340   0
360   0
A-RIB:A-R6:PRO-CA A-RIB:A-R6:PRO-CA_220 -6493.40690212
20   0
40   0
60   2

80   4
100   5
120   4
140   4
160   2
180   0
200   0
220   0
240   3
260   4
280   4
300   4
320   3
340   2
360   1
C-P:C-RIB:TRP-S2 C-P:C-RIB:TRP-S2_300 -1940.22712767
20   0
40   0
60   0
80   1
100   1
120   1
140   0
160   0
180   0
200   0
220   0
240   0
260   1
280   1
300   1
320   1
340   0
360   0
U-RIB:U-Y:HIS-S2 U-RIB:U-Y:HIS-S2_360 -6963.9503457
20   0
40   0
60   1
80   2
100   1
120   1
140   1
160   0
180   0
200   0
220   0
240   1
260   1
280   1
300   1
320   1
340   0
360   0
G-RIB:G-R6:GLU-S2 G-RIB:G-R6:GLU-S2_20 -2915.88161223
20   1
40   6
60   16
80   20

```
100   20
120   17
140   12
160   7
180   2
200   0
220   6
240   15
260   20
280   19
300   18
320   12
340   7
360   2
A-RIB:A-R6:TRP-CA A-RIB:A-R6:TRP-CA_340 -4494.58334024
20   0
40   0
60   0
80   0
100   1
120   0
140   0
160   0
180   0
200   0
220   0
240   0
260   0
280   0
300   1
320   0
340   0
360   0
U34-P:U34-RIB:SER-CA U34-P:U34-RIB:SER-CA_260 0.0
20   0
40   0
60   0
80   0
100   0
120   0
140   0
160   0
180   0
200   0
220   0
240   0
260   0
280   0
300   0
320   0
340   0
360   0
C-P:C-RIB:CYS-S1 C-P:C-RIB:CYS-S1_120 0.0
20   0
40   0
60   0
80   0
100   0
```

120   0
140   0
160   0
180   0
200   0
220   0
240   0
260   0
280   0
300   0
320   0
340   0
360   0
FHU-RIB:FHU-P:HIS-S2 FHU-RIB:FHU-P:HIS-S2_280 -12123.0819647
20   0
40   0
60   0
80   0
100   0
120   0
140   0
160   0
180   0
200   0
220   0
240   0
260   0
280   0
300   0
320   0
340   0
360   0
I-RIB:I-P:TRP-S2 I-RIB:I-P:TRP-S2_120 0.0
20   0
40   0
60   0
80   0
100   0
120   0
140   0
160   0
180   0
200   0
220   0
240   0
260   0
280   0
300   0
320   0
340   0
360   0
FHU-P:FHU-RIB:ARG-S2 FHU-P:FHU-RIB:ARG-S2_80 0.0
20   0
40   0
60   0
80   0
100   0
120   0

140 0
160 0
180 0
200 0
220 0
240 0
260 0
280 0
300 0
320 0
340 0
360 0
IU-RIB:IU-MY:ILE-CA IU-RIB:IU-MY:ILE-CA_280 0.0
20 0
40 0
60 0
80 0
100 0
120 0
140 0
160 0
180 0
200 0
220 0
240 0
260 0
280 0
300 0
320 0
340 0
360 0
U-RIB:U-Y:GLY-CA U-RIB:U-Y:GLY-CA_280 -3410.41867009
20 0
40 2
60 4
80 5
100 5
120 4
140 4
160 2
180 1
200 0
220 2
240 4
260 5
280 5
300 4
320 4
340 2
360 1
A-P:A-RIB:CYS-CA A-P:A-RIB:CYS-CA_40 0.0
20 0
40 0
60 0
80 0
100 0
120 0
140 0

160   0
180   0
200   0
220   0
240   0
260   0
280   0
300   0
320   0
340   0
360   0
C31-RIB:C31-P:THR-CA C31-RIB:C31-P:THR-CA_60 -9139.98330992
20   0
40   0
60   0
80   0
100   0
120   0
140   0
160   0
180   0
200   0
220   0
240   0
260   0
280   0
300   0
320   0
340   0
360   0
H2U-RIB:H2U-MY:GLU-S2 H2U-RIB:H2U-MY:GLU-S2_20 0.0
20   0
40   0
60   0
80   0
100   0
120   0
140   0
160   0
180   0
200   0
220   0
240   0
260   0
280   0
300   0
320   0
340   0
360   0
A-RIB:A-R6:ILE-S1 A-RIB:A-R6:ILE-S1_120 -861.713765161
20   0
40   0
60   1
80   2
100   0
120   2
140   2
160   0

180   0
200   0
220   0
240   1
260   2
280   3
300   3
320   2
340   1
360   0
U-RIB:U-P:GLN-S2 U-RIB:U-P:GLN-S2_40 -3938.65946441
20   0
40   1
60   2
80   4
100   5
120   5
140   4
160   3
180   1
200   0
220   0
240   2
260   4
280   5
300   5
320   4
340   3
360   0
U-RIB:U-P:LYS-CA U-RIB:U-P:LYS-CA_220 -3774.20111604
20   0
40   0
60   2
80   5
100   7
120   8
140   6
160   4
180   1
200   0
220   0
240   2
260   5
280   7
300   8
320   7
340   5
360   1
C31-RIB:C31-MY:PHE-CA C31-RIB:C31-MY:PHE-CA_300 0.0
20   0
40   0
60   0
80   0
100   0
120   0
140   0
160   0
180   0

200 0
220 0
240 0
260 0
280 0
300 0
320 0
340 0
360 0
U-P:U-RIB:TYR-S1 U-P:U-RIB:TYR-S1_200 0.0
20 0
40 0
60 0
80 1
100 1
120 1
140 1
160 1
180 0
200 0
220 0
240 0
260 0
280 1
300 1
320 1
340 1
360 0
FHU-RIB:FHU-P:CYS-S1 FHU-RIB:FHU-P:CYS-S1_360 0.0
20 0
40 0
60 0
80 0
100 0
120 0
140 0
160 0
180 0
200 0
220 0
240 0
260 0
280 0
300 0
320 0
340 0
360 0
A-RIB:A-P:ALA-CA A-RIB:A-P:ALA-CA_120 -2541.20997919
20 0
40 1
60 4
80 7
100 9
120 10
140 9
160 6
180 2
200 0

220   0
240   4
260   6
280   9
300   10
320   8
340   6
360   2
G-RIB:G-R5:PRO-CA G-RIB:G-R5:PRO-CA_240 -3636.5917887
20   0
40   2
60   4
80   5
100   4
120   4
140   3
160   0
180   0
200   0
220   2
240   4
260   5
280   4
300   3
320   3
340   2
360   0
G-P:G-RIB:TYR-CA G-P:G-RIB:TYR-CA_260 -4892.7917859
20   0
40   0
60   1
80   2
100   3
120   0
140   2
160   1
180   0
200   0
220   0
240   0
260   2
280   3
300   3
320   0
340   2
360   0
H2U-RIB:H2U-MY:LEU-S2 H2U-RIB:H2U-MY:LEU-S2_240 0.0
20   0
40   0
60   0
80   0
100   0
120   0
140   0
160   0
180   0
200   0
220   0

240 0
260 0
280 0
300 0
320 0
340 0
360 0
A-P:A-RIB:HIS-S2 A-P:A-RIB:HIS-S2_360 -2352.16840754
20 0
40 0
60 2
80 4
100 4
120 4
140 3
160 3
180 1
200 0
220 1
240 3
260 3
280 4
300 3
320 3
340 3
360 1
A-P:A-RIB:GLN-S2
20 0
40 2
60 4
80 6
100 6
120 6
140 6
160 4
180 1
200 0
220 1
240 4
260 6
280 6
300 6
320 5
340 4
360 2
A-RIB:A-P:TYR-S1 A-RIB:A-P:TYR-S1_160 -6174.28963249
20 0
40 0
60 0
80 1
100 2
120 2
140 2
160 1
180 0
200 0
220 0
240 0

260   1
280   2
300   2
320   2
340   1
360   0
G-RIB:G-P:TRP-S1 G-RIB:G-P:TRP-S1_340 0.0
20   0
40   0
60   0
80   0
100   1
120   0
140   2
160   1
180   0
200   0
220   0
240   0
260   1
280   2
300   2
320   2
340   0
360   0
U31-RIB:U31-MY:ILE-CA U31-RIB:U31-MY:ILE-CA_120 0.0
20   0
40   0
60   0
80   0
100   0
120   0
140   0
160   0
180   0
200   0
220   0
240   0
260   0
280   0
300   0
320   0
340   0
360   0
H2U-P:H2U-RIB:PRO-S1 H2U-P:H2U-RIB:PRO-S1_20 0.0
20   0
40   0
60   0
80   0
100   0
120   0
140   0
160   0
180   0
200   0
220   0
240   0
260   0

280   0
300   0
320   0
340   0
360   0
A-P:A-RIB:PHE-CA A-P:A-RIB:PHE-CA_180 -4619.27443751
20   0
40   0
60   1
80   2
100   2
120   2
140   2
160   1
180   0
200   0
220   0
240   1
260   2
280   2
300   2
320   2
340   1
360   0
G-RIB:G-R6:ARG-CA G-RIB:G-R6:ARG-CA_40 -4412.35908553
20   0
40   1
60   4
80   6
100   8
120   6
140   5
160   3
180   1
200   0
220   1
240   3
260   6
280   7
300   6
320   5
340   3
360   0
FMU-RIB:FMU-MY:CYS-CA FMU-RIB:FMU-MY:CYS-CA_200 0.0
20   0
40   0
60   0
80   0
100   0
120   0
140   0
160   0
180   0
200   0
220   0
240   0
260   0
280   0

```
300   0
320   0
340   0
360   0
U-P:U-RIB:LYS-S2 U-P:U-RIB:LYS-S2_300 -3156.4576982
20   0
40   3
60   7
80   10
100   9
120   9
140   7
160   6
180   2
200   1
220   3
240   8
260   9
280   10
300   9
320   8
340   6
360   2
C31-P:C31-RIB:LEU-S1 C31-P:C31-RIB:LEU-S1_100 0.0
20   0
40   0
60   0
80   0
100   0
120   0
140   0
160   0
180   0
200   0
220   0
240   0
260   0
280   0
300   0
320   0
340   0
360   0
U31-RIB:U31-MY:GLU-S2 U31-RIB:U31-MY:GLU-S2_240 0.0
20   0
40   0
60   0
80   0
100   0
120   0
140   0
160   0
180   0
200   0
220   0
240   0
260   0
280   0
300   0
```

320 0
340 0
360 0
FHU-RIB:FHU-P:PRO-CA FHU-RIB:FHU-P:PRO-CA_100 0.0
20 0
40 0
60 0
80 0
100 0
120 0
140 0
160 0
180 0
200 0
220 0
240 0
260 0
280 0
300 0
320 0
340 0
360 0
A-P:A-RIB:HIS-CA A-P:A-RIB:HIS-CA_140 -973.846594906
20 0
40 0
60 1
80 2
100 3
120 2
140 2
160 2
180 1
200 0
220 0
240 1
260 2
280 2
300 3
320 2
340 2
360 0
U-RIB:U-Y:CYS-S1 U-RIB:U-Y:CYS-S1_40 0.0
20 0
40 0
60 0
80 0
100 0
120 0
140 0
160 0
180 0
200 0
220 0
240 0
260 0
280 0
300 0
320 0

340 0
360 0
5BU-RIB:5BU-P:PRO-CA 5BU-RIB:5BU-P:PRO-CA_120 0.0
20 0
40 0
60 0
80 0
100 0
120 0
140 0
160 0
180 0
200 0
220 0
240 0
260 0
280 0
300 0
320 0
340 0
360 0
C-RIB:C-P:LYS-CA C-RIB:C-P:LYS-CA_100 -3599.33744138
20 0
40 1
60 4
80 7
100 11
120 12
140 10
160 7
180 2
200 0
220 1
240 3
260 7
280 11
300 12
320 10
340 7
360 2
A-RIB:A-R5:TYR-S2 A-RIB:A-R5:TYR-S2_120 -5734.74315778
20 0
40 0
60 1
80 1
100 1
120 1
140 1
160 0
180 0
200 0
220 0
240 1
260 1
280 1
300 1
320 0
340 0

```
360   0
U-RIB:U-Y:TYR-S2 U-RIB:U-Y:TYR-S2_300 -6681.61412773
20   0
40   0
60   0
80   1
100   1
120   1
140   1
160   0
180   0
200   0
220   0
240   1
260   1
280   1
300   1
320   1
340   0
360   0
FHU-P:FHU-RIB:ALA-S1 FHU-P:FHU-RIB:ALA-S1_280 -10245.7545123
20   0
40   0
60   0
80   0
100   0
120   0
140   0
160   0
180   0
200   0
220   0
240   0
260   0
280   0
300   0
320   0
340   0
360   0
FMU-RIB:FMU-P:CYS-S1 FMU-RIB:FMU-P:CYS-S1_160 0.0
20   0
40   0
60   0
80   0
100   0
120   0
140   0
160   0
180   0
200   0
220   0
240   0
260   0
280   0
300   0
320   0
340   0
360   0
```

A-RIB:A-R5:VAL-S1 A-RIB:A-R5:VAL-S1_300 -5035.30661985
20 0
40 2
60 3
80 3
100 3
120 3
140 2
160 1
180 0
200 0
220 1
240 3
260 3
280 3
300 3
320 2
340 1
360 0
QUO-RIB:QUO-M5:LYS-S2 QUO-RIB:QUO-M5:LYS-S2_240 0.0
20 0
40 0
60 0
80 0
100 0
120 0
140 0
160 0
180 0
200 0
220 0
240 0
260 0
280 0
300 0
320 0
340 0
360 0
H2U-RIB:H2U-MY:PHE-S1 H2U-RIB:H2U-MY:PHE-S1_340 0.0
20 0
40 0
60 0
80 0
100 0
120 0
140 0
160 0
180 0
200 0
220 0
240 0
260 0
280 0
300 0
320 0
340 0
360 0
G-RIB:G-R6:LEU-S2 G-RIB:G-R6:LEU-S2_80 -2965.26837326

```
20   0
40   1
60   2
80   3
100   4
120   3
140   2
160   1
180   0
200   0
220   1
240   2
260   3
280   4
300   4
320   3
340   0
360   0
A-RIB:A-R5:ARG-S1 A-RIB:A-R5:ARG-S1_240 -4262.19674726
20   1
40   3
60   5
80   7
100   7
120   6
140   5
160   3
180   1
200   1
220   3
240   6
260   7
280   7
300   6
320   5
340   0
360   1
A-RIB:A-R6:TYR-S2 A-RIB:A-R6:TYR-S2_180 0.0
20   0
40   0
60   1
80   2
100   2
120   2
140   1
160   1
180   0
200   0
220   0
240   1
260   2
280   2
300   2
320   1
340   1
360   0
FHU-P:FHU-RIB:PRO-S1 FHU-P:FHU-RIB:PRO-S1_200 0.0
20   0
```

40   0
60   0
80   0
100   0
120   0
140   0
160   0
180   0
200   0
220   0
240   0
260   0
280   0
300   0
320   0
340   0
360   0
G-RIB:G-R6:PHE-S2 G-RIB:G-R6:PHE-S2_20 -12675.9675659
20   0
40   0
60   1
80   1
100   1
120   2
140   1
160   1
180   0
200   0
220   0
240   1
260   1
280   1
300   1
320   0
340   1
360   0
U-P:U-RIB:GLU-CA U-P:U-RIB:GLU-CA_220 -2191.06034545
20   0
40   1
60   4
80   6
100   7
120   8
140   6
160   5
180   0
200   0
220   1
240   4
260   6
280   8
300   8
320   7
340   5
360   2
QUO-RIB:QUO-M6:ARG-CA QUO-RIB:QUO-M6:ARG-CA_80 0.0
20   0
40   0

60   0
80   0
100   0
120   0
140   0
160   0
180   0
200   0
220   0
240   0
260   0
280   0
300   0
320   0
340   0
360   0
FMU-RIB:FMU-P:ASP-CA FMU-RIB:FMU-P:ASP-CA_20 0.0
20   0
40   0
60   0
80   0
100   0
120   0
140   0
160   0
180   0
200   0
220   0
240   0
260   0
280   0
300   0
320   0
340   0
360   0
IU-P:IU-RIB:ARG-CA IU-P:IU-RIB:ARG-CA_280 -10095.5441196
20   0
40   0
60   0
80   0
100   0
120   0
140   0
160   0
180   0
200   0
220   0
240   0
260   0
280   0
300   0
320   0
340   0
360   0
FHU-RIB:FHU-P:ARG-S1 FHU-RIB:FHU-P:ARG-S1_340 0.0
20   0
40   0
60   0

```
80   0
100   0
120   0
140   0
160   0
180   0
200   0
220   0
240   0
260   0
280   0
300   0
320   0
340   0
360   0
IU-P:IU-RIB:HIS-CA IU-P:IU-RIB:HIS-CA_60 0.0
20   0
40   0
60   0
80   0
100   0
120   0
140   0
160   0
180   0
200   0
220   0
240   0
260   0
280   0
300   0
320   0
340   0
360   0
C-P:C-RIB:SER-CA C-P:C-RIB:SER-CA_100 -3979.16279715
20   0
40   1
60   4
80   7
100   7
120   8
140   7
160   5
180   2
200   0
220   1
240   4
260   7
280   7
300   7
320   7
340   5
360   2
A-RIB:A-P:MET-S1 A-RIB:A-P:MET-S1_260 -6783.415561
20   0
40   0
60   1
80   1
```

100   2
120   2
140   2
160   1
180   0
200   0
220   0
240   1
260   1
280   2
300   2
320   2
340   0
360   0
U-P:U-RIB:GLU-S2 U-P:U-RIB:GLU-S2_280 -2262.88313243
20   0
40   0
60   8
80   11
100   12
120   11
140   9
160   8
180   3
200   0
220   3
240   8
260   11
280   12
300   11
320   9
340   8
360   3
U-RIB:U-P:PHE-CA U-RIB:U-P:PHE-CA_120 -3369.6323556
20   0
40   0
60   0
80   1
100   0
120   2
140   1
160   1
180   0
200   0
220   0
240   0
260   1
280   2
300   0
320   1
340   0
360   0
G-RIB:G-P:TYR-CA G-RIB:G-P:TYR-CA_100 -3967.9818499
20   0
40   0
60   1
80   2
100   4

120 4
140 3
160 2
180 0
200 0
220 0
240 1
260 2
280 4
300 4
320 3
340 2
360 0
C31-P:C31-RIB:GLU-S2 C31-P:C31-RIB:GLU-S2_180 0.0
20 0
40 0
60 0
80 0
100 0
120 0
140 0
160 0
180 0
200 0
220 0
240 0
260 0
280 0
300 0
320 0
340 0
360 0
H2U-RIB:H2U-MY:ASN-S2 H2U-RIB:H2U-MY:ASN-S2_280 0.0
20 0
40 0
60 0
80 0
100 0
120 0
140 0
160 0
180 0
200 0
220 0
240 0
260 0
280 0
300 0
320 0
340 0
360 0
C-RIB:C-Y:CYS-CA C-RIB:C-Y:CYS-CA_320 0.0
20 0
40 0
60 0
80 0
100 0
120 0

140 0
160 0
180 0
200 0
220 0
240 0
260 0
280 0
300 0
320 0
340 0
360 0
G-RIB:G-R5:HIS-S2 G-RIB:G-R5:HIS-S2_80 -5638.47528979
20 0
40 2
60 4
80 4
100 3
120 2
140 2
160 1
180 0
200 0
220 2
240 3
260 4
280 3
300 2
320 0
340 1
360 0
C-RIB:C-Y:VAL-S1 C-RIB:C-Y:VAL-S1_360 0.0
20 0
40 2
60 3
80 3
100 2
120 2
140 2
160 0
180 0
200 0
220 2
240 3
260 3
280 2
300 2
320 2
340 0
360 0
C-P:C-RIB:ASP-S2 C-P:C-RIB:ASP-S2_40 -1048.36988641
20 0
40 3
60 8
80 11
100 12
120 12
140 10

160   8
180   4
200   0
220   3
240   8
260   12
280   12
300   12
320   11
340   9
360   4
G-P:G-RIB:LYS-S2 G-P:G-RIB:LYS-S2_180 1298.65998101
20   2
40   9
60   19
80   23
100   22
120   20
140   17
160   15
180   6
200   2
220   9
240   18
260   22
280   22
300   20
320   17
340   14
360   7
A-P:A-RIB:GLU-CA A-P:A-RIB:GLU-CA_300 -2096.88415746
20   0
40   2
60   0
80   9
100   11
120   11
140   10
160   7
180   0
200   0
220   0
240   0
260   10
280   12
300   12
320   10
340   8
360   3
C-RIB:C-Y:HIS-S2 C-RIB:C-Y:HIS-S2_220 -4487.83667125
20   0
40   1
60   2
80   2
100   2
120   1
140   1
160   1

180   0
200   0
220   1
240   2
260   2
280   2
300   1
320   0
340   1
360   0
QUO-RIB:QUO-M5:GLN-S2 QUO-RIB:QUO-M5:GLN-S2_200 0.0
20   0
40   0
60   0
80   0
100   0
120   0
140   0
160   0
180   0
200   0
220   0
240   0
260   0
280   0
300   0
320   0
340   0
360   0
U-RIB:U-P:TRP-S2 U-RIB:U-P:TRP-S2_160 -3950.83498108
20   0
40   0
60   0
80   0
100   1
120   1
140   0
160   0
180   0
200   0
220   0
240   0
260   0
280   1
300   0
320   0
340   0
360   0
FMU-RIB:FMU-MY:GLU-CA FMU-RIB:FMU-MY:GLU-CA_180 0.0
20   0
40   0
60   0
80   0
100   0
120   0
140   0
160   0
180   0

200   0
220   0
240   0
260   0
280   0
300   0
320   0
340   0
360   0
C31-RIB:C31-MY:GLN-S1 C31-RIB:C31-MY:GLN-S1_20 0.0
20   0
40   0
60   0
80   0
100   0
120   0
140   0
160   0
180   0
200   0
220   0
240   0
260   0
280   0
300   0
320   0
340   0
360   0
H2U-P:H2U-RIB:GLY-CA H2U-P:H2U-RIB:GLY-CA_280 0.0
20   0
40   0
60   0
80   0
100   0
120   0
140   0
160   0
180   0
200   0
220   0
240   0
260   0
280   0
300   0
320   0
340   0
360   0
QUO-RIB:QUO-M5:PHE-S2 QUO-RIB:QUO-M5:PHE-S2_180 0.0
20   0
40   0
60   0
80   0
100   0
120   0
140   0
160   0
180   0
200   0

220   0
240   0
260   0
280   0
300   0
320   0
340   0
360   0
G-RIB:G-R5:MET-CA G-RIB:G-R5:MET-CA_240 -4710.86356709
20   0
40   0
60   1
80   1
100   1
120   0
140   0
160   0
180   0
200   0
220   0
240   1
260   1
280   1
300   0
320   0
340   0
360   0
G-RIB:G-R5:GLN-CA G-RIB:G-R5:GLN-CA_100 0.0
20   0
40   1
60   2
80   2
100   0
120   0
140   2
160   0
180   0
200   0
220   1
240   2
260   2
280   2
300   2
320   2
340   0
360   0
G-RIB:G-R6:ASP-S1 G-RIB:G-R6:ASP-S1_80 -1938.8720756
20   0
40   2
60   7
80   10
100   11
120   9
140   7
160   4
180   1
200   0
220   2

240 7
260 11
280 11
300 9
320 7
340 4
360 1
IU-RIB:IU-MY:LYS-S1 IU-RIB:IU-MY:LYS-S1_220 0.0
20 0
40 0
60 0
80 0
100 0
120 0
140 0
160 0
180 0
200 0
220 0
240 0
260 0
280 0
300 0
320 0
340 0
360 0
U-P:U-RIB:ILE-CA U-P:U-RIB:ILE-CA_280 -3625.32822005
20 0
40 0
60 0
80 2
100 3
120 3
140 2
160 1
180 0
200 0
220 0
240 0
260 2
280 2
300 2
320 2
340 1
360 0
FMU-P:FMU-RIB:VAL-CA FMU-P:FMU-RIB:VAL-CA_80 0.0
20 0
40 0
60 0
80 0
100 0
120 0
140 0
160 0
180 0
200 0
220 0
240 0

260   0
280   0
300   0
320   0
340   0
360   0
A-P:A-RIB:VAL-CA A-P:A-RIB:VAL-CA_180 -2079.6177973
20   0
40   0
60   3
80   4
100   5
120   6
140   5
160   4
180   1
200   0
220   0
240   2
260   5
280   6
300   6
320   5
340   4
360   2
QUO-RIB:QUO-M6:LEU-S1 QUO-RIB:QUO-M6:LEU-S1_180 0.0
20   0
40   0
60   0
80   0
100   0
120   0
140   0
160   0
180   0
200   0
220   0
240   0
260   0
280   0
300   0
320   0
340   0
360   0
G-RIB:G-R6:ARG-S1 G-RIB:G-R6:ARG-S1_200 0.0
20   0
40   2
60   7
80   9
100   10
120   9
140   7
160   4
180   0
200   0
220   2
240   6
260   10

280   10
300   9
320   7
340   5
360   1
U-RIB:U-Y:GLU-CA U-RIB:U-Y:GLU-CA_320 562.776682819
20   0
40   1
60   3
80   4
100   5
120   5
140   3
160   2
180   0
200   0
220   1
240   3
260   4
280   4
300   4
320   3
340   0
360   0
FMU-RIB:FMU-MY:MET-S1 FMU-RIB:FMU-MY:MET-S1_360 0.0
20   0
40   0
60   0
80   0
100   0
120   0
140   0
160   0
180   0
200   0
220   0
240   0
260   0
280   0
300   0
320   0
340   0
360   0
M2G-RIB:M2G-P:GLU-S2 M2G-RIB:M2G-P:GLU-S2_60 0.0
20   0
40   0
60   0
80   0
100   0
120   0
140   0
160   0
180   0
200   0
220   0
240   0
260   0
280   0

300 0
320 0
340 0
360 0
C-RIB:C-P:MET-S1 C-RIB:C-P:MET-S1_140 -2552.09234415
20 0
40 0
60 1
80 1
100 2
120 2
140 2
160 1
180 0
200 0
220 0
240 0
260 1
280 2
300 2
320 2
340 1
360 0
G-P:G-RIB:SER-CA G-P:G-RIB:SER-CA_300 -3774.20111604
20 0
40 2
60 7
80 10
100 10
120 10
140 9
160 7
180 3
200 0
220 2
240 7
260 10
280 11
300 11
320 9
340 7
360 3
FHU-RIB:FHU-MY:PRO-S1 FHU-RIB:FHU-MY:PRO-S1_280 0.0
20 0
40 0
60 0
80 0
100 0
120 0
140 0
160 0
180 0
200 0
220 0
240 0
260 0
280 0
300 0

320 0
340 0
360 0
GTP-RIB:GTP-M5:ASN-S1 GTP-RIB:GTP-M5:ASN-S1_320 0.0
20 0
40 0
60 0
80 0
100 0
120 0
140 0
160 0
180 0
200 0
220 0
240 0
260 0
280 0
300 0
320 0
340 0
360 0
U34-RIB:U34-MY:SER-CA U34-RIB:U34-MY:SER-CA_340 -14393.3871076
20 0
40 0
60 0
80 0
100 0
120 0
140 0
160 0
180 0
200 0
220 0
240 0
260 0
280 0
300 0
320 0
340 0
360 0
C31-P:C31-RIB:GLN-S2 C31-P:C31-RIB:GLN-S2_340 0.0
20 0
40 0
60 0
80 0
100 0
120 0
140 0
160 0
180 0
200 0
220 0
240 0
260 0
280 0
300 0
320 0

340   0
360   0
U-P:U-RIB:ARG-S1 U-P:U-RIB:ARG-S1_360 -1236.93978734
20   0
40   1
60   4
80   6
100   7
120   7
140   5
160   4
180   1
200   0
220   1
240   4
260   6
280   7
300   6
320   6
340   4
360   1
IU-RIB:IU-MY:LEU-S1 IU-RIB:IU-MY:LEU-S1_40 0.0
20   0
40   0
60   0
80   0
100   0
120   0
140   0
160   0
180   0
200   0
220   0
240   0
260   0
280   0
300   0
320   0
340   0
360   0
G-RIB:G-P:TYR-S1 G-RIB:G-P:TYR-S1_340 -4304.45904713
20   0
40   0
60   1
80   2
100   3
120   4
140   3
160   2
180   0
200   0
220   0
240   1
260   2
280   4
300   4
320   3
340   2

360   0
U-RIB:U-P:MET-S1 U-RIB:U-P:MET-S1_60 -3878.66349061
20   0
40   0
60   0
80   1
100   1
120   1
140   0
160   0
180   0
200   0
220   0
240   0
260   1
280   1
300   1
320   1
340   1
360   0
FMU-RIB:FMU-MY:ILE-CA FMU-RIB:FMU-MY:ILE-CA_140 0.0
20   0
40   0
60   0
80   0
100   0
120   0
140   0
160   0
180   0
200   0
220   0
240   0
260   0
280   0
300   0
320   0
340   0
360   0
A-P:A-RIB:ASN-S1 A-P:A-RIB:ASN-S1_360 -4884.77184942
20   0
40   1
60   3
80   5
100   6
120   5
140   5
160   4
180   2
200   0
220   1
240   4
260   6
280   6
300   6
320   5
340   4
360   2

G-P:G-RIB:TRP-S1 G-P:G-RIB:TRP-S1_240 -6981.16871003
20 0
40 0
60 1
80 1
100 1
120 1
140 1
160 1
180 0
200 0
220 0
240 0
260 1
280 1
300 1
320 1
340 1
360 0
U34-P:U34-RIB:SER-S1 U34-P:U34-RIB:SER-S1_140 0.0
20 0
40 0
60 0
80 0
100 0
120 0
140 0
160 0
180 0
200 0
220 0
240 0
260 0
280 0
300 0
320 0
340 0
360 0
U31-RIB:U31-P:LEU-S2 U31-RIB:U31-P:LEU-S2_120 0.0
20 0
40 0
60 0
80 0
100 0
120 0
140 0
160 0
180 0
200 0
220 0
240 0
260 0
280 0
300 0
320 0
340 0
360 0
5BU-RIB:5BU-P:ALA-S1 5BU-RIB:5BU-P:ALA-S1_260 0.0

```
20   0
40   0
60   0
80   0
100   0
120   0
140   0
160   0
180   0
200   0
220   0
240   0
260   0
280   0
300   0
320   0
340   0
360   0
FMU-RIB:FMU-MY:PHE-CA FMU-RIB:FMU-MY:PHE-CA_80 0.0
20   0
40   0
60   0
80   0
100   0
120   0
140   0
160   0
180   0
200   0
220   0
240   0
260   0
280   0
300   0
320   0
340   0
360   0
IU-RIB:IU-P:HIS-CA IU-RIB:IU-P:HIS-CA_340 0.0
20   0
40   0
60   0
80   0
100   0
120   0
140   0
160   0
180   0
200   0
220   0
240   0
260   0
280   0
300   0
320   0
340   0
360   0
A-RIB:A-R5:GLU-CA A-RIB:A-R5:GLU-CA_120 -3902.86191338
20   0
```

40 2
60 0
80 6
100 7
120 6
140 5
160 3
180 1
200 0
220 2
240 5
260 6
280 7
300 6
320 4
340 0
360 1
IU-RIB:IU-P:LYS-S2 IU-RIB:IU-P:LYS-S2_140 -9241.12848238
20 0
40 0
60 0
80 0
100 0
120 0
140 0
160 0
180 0
200 0
220 0
240 0
260 0
280 0
300 0
320 0
340 0
360 0
U31-RIB:U31-P:ASP-S1 U31-RIB:U31-P:ASP-S1_160 0.0
20 0
40 0
60 0
80 0
100 0
120 0
140 0
160 0
180 0
200 0
220 0
240 0
260 0
280 0
300 0
320 0
340 0
360 0
GTP-RIB:GTP-M6:ASN-S1 GTP-RIB:GTP-M6:ASN-S1_200 0.0
20 0
40 0

60 0
80 0
100 0
120 0
140 0
160 0
180 0
200 0
220 0
240 0
260 0
280 0
300 0
320 0
340 0
360 0
U34-RIB:U34-MY:PRO-CA U34-RIB:U34-MY:PRO-CA_20 0.0
20 0
40 0
60 0
80 0
100 0
120 0
140 0
160 0
180 0
200 0
220 0
240 0
260 0
280 0
300 0
320 0
340 0
360 0
G-RIB:G-R5:ALA-S1 G-RIB:G-R5:ALA-S1_20 0.0
20 0
40 6
60 8
80 8
100 7
120 6
140 5
160 4
180 1
200 2
220 6
240 8
260 9
280 8
300 6
320 5
340 4
360 1
G-RIB:G-P:CYS-CA G-RIB:G-P:CYS-CA_200 0.0
20 0
40 0
60 0

80 0
100 1
120 1
140 1
160 0
180 0
200 0
220 0
240 0
260 0
280 0
300 0
320 1
340 0
360 0
C-RIB:C-P:THR-S1 C-RIB:C-P:THR-S1_20 -6078.84000914
20 0
40 1
60 4
80 6
100 8
120 7
140 6
160 5
180 1
200 0
220 1
240 3
260 6
280 8
300 7
320 7
340 5
360 1
QUO-RIB:QUO-M5:ASP-S1 QUO-RIB:QUO-M5:ASP-S1_100 0.0
20 0
40 0
60 0
80 0
100 0
120 0
140 0
160 0
180 0
200 0
220 0
240 0
260 0
280 0
300 0
320 0
340 0
360 0
QUO-RIB:QUO-M5:GLU-S1 QUO-RIB:QUO-M5:GLU-S1_120 0.0
20 0
40 0
60 0
80 0

```
100   0
120   0
140   0
160   0
180   0
200   0
220   0
240   0
260   0
280   0
300   0
320   0
340   0
360   0
U31-RIB:U31-P:ARG-CA U31-RIB:U31-P:ARG-CA_20 0.0
20   0
40   0
60   0
80   0
100   0
120   0
140   0
160   0
180   0
200   0
220   0
240   0
260   0
280   0
300   0
320   0
340   0
360   0
FHU-RIB:FHU-MY:ARG-CA FHU-RIB:FHU-MY:ARG-CA_200 0.0
20   0
40   0
60   0
80   0
100   0
120   0
140   0
160   0
180   0
200   0
220   0
240   0
260   0
280   0
300   0
320   0
340   0
360   0
FHU-RIB:FHU-P:VAL-CA FHU-RIB:FHU-P:VAL-CA_180 0.0
20   0
40   0
60   0
80   0
100   0
```

120   0
140   0
160   0
180   0
200   0
220   0
240   0
260   0
280   0
300   0
320   0
340   0
360   0
U-RIB:U-Y:HIS-S1 U-RIB:U-Y:HIS-S1_260 0.0
20   0
40   0
60   1
80   1
100   1
120   1
140   0
160   0
180   0
200   0
220   0
240   0
260   0
280   1
300   1
320   1
340   0
360   0
U-P:U-RIB:VAL-S1 U-P:U-RIB:VAL-S1_80 -3680.52305341
20   0
40   1
60   2
80   3
100   3
120   3
140   3
160   2
180   1
200   0
220   0
240   2
260   3
280   4
300   4
320   3
340   2
360   0
U-RIB:U-Y:TRP-S2 U-RIB:U-Y:TRP-S2_160 0.0
20   0
40   0
60   0
80   0
100   0
120   0

140 0
160 0
180 0
200 0
220 0
240 0
260 0
280 0
300 0
320 0
340 0
360 0
H2U-RIB:H2U-P:PRO-S1 H2U-RIB:H2U-P:PRO-S1_200 0.0
20 0
40 0
60 0
80 0
100 0
120 0
140 0
160 0
180 0
200 0
220 0
240 0
260 0
280 0
300 0
320 0
340 0
360 0
FMU-P:FMU-RIB:ARG-S1 FMU-P:FMU-RIB:ARG-S1_160 0.0
20 0
40 0
60 0
80 0
100 0
120 0
140 0
160 0
180 0
200 0
220 0
240 0
260 0
280 0
300 0
320 0
340 0
360 0
A-P:A-RIB:GLY-CA A-P:A-RIB:GLY-CA_200 -4464.36695776
20 0
40 3
60 8
80 12
100 13
120 12
140 11

160   10
180   4
200   0
220   3
240   8
260   11
280   12
300   12
320   11
340   9
360   4
G-RIB:G-P:ASP-S2 G-RIB:G-P:ASP-S2_80 -1175.31772586
20   0
40   4
60   10
80   17
100   22
120   21
140   19
160   14
180   5
200   0
220   3
240   10
260   17
280   21
300   20
320   18
340   13
360   5
FMU-RIB:FMU-MY:CYS-S1 FMU-RIB:FMU-MY:CYS-S1_160 0.0
20   0
40   0
60   0
80   0
100   0
120   0
140   0
160   0
180   0
200   0
220   0
240   0
260   0
280   0
300   0
320   0
340   0
360   0
H2U-RIB:H2U-P:GLU-CA H2U-RIB:H2U-P:GLU-CA_40 0.0
20   0
40   0
60   0
80   0
100   0
120   0
140   0
160   0

180 0
200 0
220 0
240 0
260 0
280 0
300 0
320 0
340 0
360 0
C31-RIB:C31-P:LEU-S2 C31-RIB:C31-P:LEU-S2_220 0.0
20 0
40 0
60 0
80 0
100 0
120 0
140 0
160 0
180 0
200 0
220 0
240 0
260 0
280 0
300 0
320 0
340 0
360 0
G-P:G-RIB:SER-S1 G-P:G-RIB:SER-S1_200 -6385.1198723
20 0
40 4
60 9
80 12
100 12
120 12
140 11
160 8
180 3
200 0
220 4
240 9
260 12
280 13
300 12
320 11
340 9
360 4
C-RIB:C-Y:GLU-S1 C-RIB:C-Y:GLU-S1_360 0.0
20 1
40 4
60 8
80 9
100 7
120 0
140 5
160 4
180 1

200 0
220 4
240 8
260 8
280 7
300 0
320 5
340 3
360 0
IU-RIB:IU-MY:ALA-S1 IU-RIB:IU-MY:ALA-S1_360 0.0
20 0
40 0
60 0
80 0
100 0
120 0
140 0
160 0
180 0
200 0
220 0
240 0
260 0
280 0
300 0
320 0
340 0
360 0
QUO-RIB:QUO-M6:ASP-CA QUO-RIB:QUO-M6:ASP-CA_20 0.0
20 0
40 0
60 0
80 0
100 0
120 0
140 0
160 0
180 0
200 0
220 0
240 0
260 0
280 0
300 0
320 0
340 0
360 0
G-RIB:G-P:ASN-CA G-RIB:G-P:ASN-CA_200 -13388.7610777
20 0
40 0
60 3
80 6
100 10
120 9
140 8
160 5
180 2
200 0

220 0
240 3
260 6
280 9
300 10
320 8
340 5
360 2
A-RIB:A-R6:GLU-S1 A-RIB:A-R6:GLU-S1_320 -569.458991793
20 0
40 2
60 6
80 11
100 12
120 10
140 9
160 7
180 2
200 0
220 2
240 7
260 11
280 12
300 11
320 9
340 6
360 0
FHU-P:FHU-RIB:THR-S1 FHU-P:FHU-RIB:THR-S1_340 0.0
20 0
40 0
60 0
80 0
100 0
120 0
140 0
160 0
180 0
200 0
220 0
240 0
260 0
280 0
300 0
320 0
340 0
360 0
IU-RIB:IU-MY:THR-CA IU-RIB:IU-MY:THR-CA_80 0.0
20 0
40 0
60 0
80 0
100 0
120 0
140 0
160 0
180 0
200 0
220 0

240   0
260   0
280   0
300   0
320   0
340   0
360   0
U-P:U-RIB:GLN-S2 U-P:U-RIB:GLN-S2_260 -2369.8377461
20   0
40   1
60   3
80   4
100   4
120   4
140   3
160   2
180   1
200   0
220   1
240   3
260   4
280   4
300   4
320   3
340   3
360   0
G-RIB:G-R5:GLN-S1 G-RIB:G-R5:GLN-S1_80 -4188.15618794
20   0
40   2
60   4
80   4
100   3
120   3
140   0
160   2
180   0
200   0
220   2
240   4
260   4
280   3
300   3
320   0
340   1
360   0
U31-P:U31-RIB:MET-CA U31-P:U31-RIB:MET-CA_320 0.0
20   0
40   0
60   0
80   0
100   0
120   0
140   0
160   0
180   0
200   0
220   0
240   0

260   0
280   0
300   0
320   0
340   0
360   0
C31-RIB:C31-P:MET-S2 C31-RIB:C31-P:MET-S2_20 0.0
20   0
40   0
60   0
80   0
100   0
120   0
140   0
160   0
180   0
200   0
220   0
240   0
260   0
280   0
300   0
320   0
340   0
360   0
A-RIB:A-P:VAL-CA A-RIB:A-P:VAL-CA_220 0.0
20   0
40   0
60   2
80   4
100   7
120   7
140   6
160   4
180   1
200   0
220   0
240   2
260   4
280   7
300   7
320   7
340   5
360   1
C31-RIB:C31-P:GLU-S1 C31-RIB:C31-P:GLU-S1_40 0.0
20   0
40   0
60   0
80   0
100   0
120   0
140   0
160   0
180   0
200   0
220   0
240   0
260   0

280   0
300   0
320   0
340   0
360   0
QUO-P:QUO-RIB:LYS-S1 QUO-P:QUO-RIB:LYS-S1_320 0.0
20   0
40   0
60   0
80   0
100   0
120   0
140   0
160   0
180   0
200   0
220   0
240   0
260   0
280   0
300   0
320   0
340   0
360   0
U31-P:U31-RIB:PHE-S1 U31-P:U31-RIB:PHE-S1_300 -9571.98090386
20   0
40   0
60   0
80   0
100   0
120   0
140   0
160   0
180   0
200   0
220   0
240   0
260   0
280   0
300   0
320   0
340   0
360   0
FHU-RIB:FHU-P:GLY-CA FHU-RIB:FHU-P:GLY-CA_80 -8949.29596427
20   0
40   0
60   0
80   0
100   0
120   0
140   0
160   0
180   0
200   0
220   0
240   0
260   0
280   0

300   0
320   0
340   0
360   0
C-RIB:C-P:LEU-CA C-RIB:C-P:LEU-CA_160 -977.520002037
20   0
40   0
60   1
80   4
100   6
120   7
140   6
160   4
180   0
200   0
220   0
240   1
260   4
280   6
300   7
320   6
340   4
360   1
C-P:C-RIB:GLU-CA C-P:C-RIB:GLU-CA_180 -1088.41074686
20   0
40   1
60   6
80   9
100   11
120   12
140   11
160   8
180   3
200   0
220   2
240   5
260   10
280   12
300   12
320   11
340   8
360   3
C31-RIB:C31-P:ASP-S2 C31-RIB:C31-P:ASP-S2_80 0.0
20   0
40   0
60   0
80   0
100   0
120   0
140   0
160   0
180   0
200   0
220   0
240   0
260   0
280   0
300   0

320 0
340 0
360 0
A-RIB:A-P:CYS-S1 A-RIB:A-P:CYS-S1_100 -4248.77776777
20 0
40 0
60 0
80 0
100 0
120 0
140 0
160 0
180 0
200 0
220 0
240 0
260 0
280 0
300 0
320 0
340 0
360 0
FHU-P:FHU-RIB:TYR-CA FHU-P:FHU-RIB:TYR-CA_300 -14393.3871076
20 0
40 0
60 0
80 0
100 0
120 0
140 0
160 0
180 0
200 0
220 0
240 0
260 0
280 0
300 0
320 0
340 0
360 0
C31-RIB:C31-MY:LEU-S1 C31-RIB:C31-MY:LEU-S1_20 0.0
20 0
40 0
60 0
80 0
100 0
120 0
140 0
160 0
180 0
200 0
220 0
240 0
260 0
280 0
300 0
320 0

340 0
360 0
FMU-P:FMU-RIB:GLU-S2 FMU-P:FMU-RIB:GLU-S2_180 0.0
20 0
40 0
60 0
80 0
100 0
120 0
140 0
160 0
180 0
200 0
220 0
240 0
260 0
280 0
300 0
320 0
340 0
360 0
A-RIB:A-P:GLU-CA A-RIB:A-P:GLU-CA_300 -1049.63112983
20 0
40 1
60 5
80 9
100 14
120 15
140 12
160 8
180 0
200 0
220 1
240 4
260 9
280 13
300 15
320 13
340 0
360 0
U-P:U-RIB:MET-S1 U-P:U-RIB:MET-S1_20 0.0
20 0
40 0
60 0
80 1
100 1
120 1
140 0
160 0
180 0
200 0
220 0
240 0
260 1
280 1
300 1
320 1
340 0

360 0
A-RIB:A-P:ASN-S1 A-RIB:A-P:ASN-S1_260 -3895.65747119
20 0
40 1
60 3
80 6
100 7
120 7
140 6
160 4
180 1
200 0
220 1
240 2
260 5
280 7
300 6
320 6
340 4
360 1
H2U-RIB:H2U-P:LEU-S2 H2U-RIB:H2U-P:LEU-S2_300 0.0
20 0
40 0
60 0
80 0
100 0
120 0
140 0
160 0
180 0
200 0
220 0
240 0
260 0
280 0
300 0
320 0
340 0
360 0
IU-RIB:IU-MY:LYS-CA IU-RIB:IU-MY:LYS-CA_260 -9042.80565256
20 0
40 0
60 0
80 0
100 0
120 0
140 0
160 0
180 0
200 0
220 0
240 0
260 0
280 0
300 0
320 0
340 0
360 0

U34-RIB:U34-P:TYR-S1 U34-RIB:U34-P:TYR-S1_320 -14393.3871076
20 0
40 0
60 0
80 0
100 0
120 0
140 0
160 0
180 0
200 0
220 0
240 0
260 0
280 0
300 0
320 0
340 0
360 0
5BU-RIB:5BU-MY:ARG-S2 5BU-RIB:5BU-MY:ARG-S2_200 0.0
20 0
40 0
60 0
80 0
100 0
120 0
140 0
160 0
180 0
200 0
220 0
240 0
260 0
280 0
300 0
320 0
340 0
360 0
G-RIB:G-R5:GLY-CA G-RIB:G-R5:GLY-CA_160 -2896.66513079
20 2
40 7
60 11
80 12
100 11
120 9
140 8
160 6
180 2
200 2
220 7
240 11
260 12
280 11
300 9
320 7
340 5
360 2
A-RIB:A-R5:LEU-S1 A-RIB:A-R5:LEU-S1_60 -1675.65244944

```
20   0
40   1
60   2
80   2
100   2
120   2
140   0
160   1
180   0
200   0
220   1
240   1
260   2
280   2
300   2
320   1
340   1
360   0
C-RIB:C-Y:SER-CA C-RIB:C-Y:SER-CA_320 -3065.19905509
20   0
40   2
60   4
80   4
100   3
120   3
140   2
160   1
180   0
200   0
220   2
240   5
260   4
280   3
300   3
320   2
340   1
360   0
A-RIB:A-R6:MET-S1 A-RIB:A-R6:MET-S1_120 -4816.04363369
20   0
40   0
60   0
80   1
100   1
120   1
140   1
160   1
180   0
200   0
220   0
240   0
260   0
280   1
300   1
320   1
340   0
360   0
DA-RIB:DA-M6:LYS-CA DA-RIB:DA-M6:LYS-CA_40 0.0
20   0
```

40   0
60   0
80   0
100   0
120   0
140   0
160   0
180   0
200   0
220   0
240   0
260   0
280   0
300   0
320   0
340   0
360   0
IU-P:IU-RIB:ARG-S2 IU-P:IU-RIB:ARG-S2_40 0.0
20   0
40   0
60   0
80   0
100   0
120   0
140   0
160   0
180   0
200   0
220   0
240   0
260   0
280   0
300   0
320   0
340   0
360   0
G-RIB:G-R5:GLU-S2 G-RIB:G-R5:GLU-S2_200 238.965222759
20   4
40   12
60   19
80   20
100   16
120   12
140   10
160   7
180   3
200   4
220   12
240   19
260   21
280   16
300   12
320   10
340   8
360   0
G-RIB:G-R5:LEU-CA G-RIB:G-R5:LEU-CA_140 -5591.14833115
20   0
40   1

60   1
80   2
100   2
120   2
140   2
160   1
180   0
200   0
220   1
240   2
260   2
280   2
300   2
320   2
340   0
360   0
A-P:A-RIB:VAL-S1 A-P:A-RIB:VAL-S1_340 -3792.45322551
20   0
40   1
60   3
80   5
100   5
120   5
140   5
160   4
180   1
200   0
220   0
240   3
260   5
280   5
300   6
320   5
340   4
360   1
5BU-RIB:5BU-P:ARG-S2 5BU-RIB:5BU-P:ARG-S2_260 0.0
20   0
40   0
60   0
80   0
100   0
120   0
140   0
160   0
180   0
200   0
220   0
240   0
260   0
280   0
300   0
320   0
340   0
360   0
H2U-P:H2U-RIB:LYS-S1 H2U-P:H2U-RIB:LYS-S1_160 0.0
20   0
40   0
60   0

80   0
100   0
120   0
140   0
160   0
180   0
200   0
220   0
240   0
260   0
280   0
300   0
320   0
340   0
360   0
FMU-RIB:FMU-MY:ARG-S2 FMU-RIB:FMU-MY:ARG-S2_360 0.0
20   0
40   0
60   0
80   0
100   0
120   0
140   0
160   0
180   0
200   0
220   0
240   0
260   0
280   0
300   0
320   0
340   0
360   0
FMU-RIB:FMU-MY:ASP-CA FMU-RIB:FMU-MY:ASP-CA_20 0.0
20   0
40   0
60   0
80   0
100   0
120   0
140   0
160   0
180   0
200   0
220   0
240   0
260   0
280   0
300   0
320   0
340   0
360   0
U31-RIB:U31-MY:ASP-S2 U31-RIB:U31-MY:ASP-S2_200 0.0
20   0
40   0
60   0
80   0

100 0
120 0
140 0
160 0
180 0
200 0
220 0
240 0
260 0
280 0
300 0
320 0
340 0
360 0
QUO-P:QUO-RIB:ASN-S2 QUO-P:QUO-RIB:ASN-S2_180 0.0
20 0
40 0
60 0
80 0
100 0
120 0
140 0
160 0
180 0
200 0
220 0
240 0
260 0
280 0
300 0
320 0
340 0
360 0
G-RIB:G-P:HIS-S2 G-RIB:G-P:HIS-S2_240 -2999.67204536
20 0
40 1
60 3
80 6
100 7
120 7
140 6
160 4
180 0
200 0
220 1
240 3
260 6
280 7
300 7
320 6
340 4
360 1
C31-P:C31-RIB:ASP-CA C31-P:C31-RIB:ASP-CA_140 0.0
20 0
40 0
60 0
80 0
100 0

120   0
140   0
160   0
180   0
200   0
220   0
240   0
260   0
280   0
300   0
320   0
340   0
360   0
C31-P:C31-RIB:PHE-S2 C31-P:C31-RIB:PHE-S2_340 0.0
20   0
40   0
60   0
80   0
100   0
120   0
140   0
160   0
180   0
200   0
220   0
240   0
260   0
280   0
300   0
320   0
340   0
360   0
A-RIB:A-R6:HIS-S2 A-RIB:A-R6:HIS-S2_160 -3606.66864978
20   0
40   0
60   2
80   3
100   3
120   2
140   2
160   1
180   0
200   0
220   0
240   2
260   3
280   3
300   3
320   2
340   1
360   0
G-RIB:G-P:ILE-S1 G-RIB:G-P:ILE-S1_240 -4354.22826305
20   0
40   0
60   2
80   4
100   6
120   7

140   6
160   4
180   0
200   0
220   0
240   2
260   4
280   6
300   7
320   6
340   4
360   1
U-RIB:U-P:HIS-CA U-RIB:U-P:HIS-CA_220 0.0
20   0
40   0
60   0
80   1
100   2
120   2
140   2
160   1
180   0
200   0
220   0
240   0
260   1
280   2
300   2
320   2
340   1
360   0
A-P:A-RIB:ILE-S1 A-P:A-RIB:ILE-S1_320 -3765.69150497
20   0
40   0
60   2
80   3
100   3
120   4
140   3
160   2
180   1
200   0
220   0
240   0
260   2
280   3
300   3
320   3
340   2
360   1
QUO-RIB:QUO-M5:LEU-CA QUO-RIB:QUO-M5:LEU-CA_320 0.0
20   0
40   0
60   0
80   0
100   0
120   0
140   0

160   0
180   0
200   0
220   0
240   0
260   0
280   0
300   0
320   0
340   0
360   0
C-RIB:C-P:TRP-CA C-RIB:C-P:TRP-CA_60 -4836.31725772
20   0
40   0
60   0
80   0
100   0
120   1
140   1
160   0
180   0
200   0
220   0
240   0
260   1
280   1
300   0
320   1
340   0
360   0
H2U-RIB:H2U-P:THR-CA H2U-RIB:H2U-P:THR-CA_100 0.0
20   0
40   0
60   0
80   0
100   0
120   0
140   0
160   0
180   0
200   0
220   0
240   0
260   0
280   0
300   0
320   0
340   0
360   0
U31-P:U31-RIB:ASP-S2 U31-P:U31-RIB:ASP-S2_220 0.0
20   0
40   0
60   0
80   0
100   0
120   0
140   0
160   0

180   0
200   0
220   0
240   0
260   0
280   0
300   0
320   0
340   0
360   0
GTP-RIB:GTP-M6:SER-CA GTP-RIB:GTP-M6:SER-CA_20 0.0
20   0
40   0
60   0
80   0
100   0
120   0
140   0
160   0
180   0
200   0
220   0
240   0
260   0
280   0
300   0
320   0
340   0
360   0
C-P:C-RIB:HIS-S1 C-P:C-RIB:HIS-S1_20 0.0
20   0
40   0
60   1
80   2
100   3
120   3
140   2
160   2
180   0
200   0
220   0
240   1
260   2
280   2
300   3
320   2
340   2
360   1
G-P:G-RIB:MET-S2 G-P:G-RIB:MET-S2_360 -3515.42220719
20   0
40   1
60   2
80   3
100   3
120   2
140   2
160   2
180   0

200   0
220   0
240   2
260   3
280   3
300   3
320   3
340   2
360   0
FMU-RIB:FMU-P:ILE-CA FMU-RIB:FMU-P:ILE-CA_340 0.0
20   0
40   0
60   0
80   0
100   0
120   0
140   0
160   0
180   0
200   0
220   0
240   0
260   0
280   0
300   0
320   0
340   0
360   0
DA-RIB:DA-M5:THR-CA DA-RIB:DA-M5:THR-CA_300 0.0
20   0
40   0
60   0
80   0
100   0
120   0
140   0
160   0
180   0
200   0
220   0
240   0
260   0
280   0
300   0
320   0
340   0
360   0
U-RIB:U-P:LEU-CA U-RIB:U-P:LEU-CA_60 -2173.12748558
20   0
40   0
60   1
80   2
100   4
120   4
140   4
160   0
180   1
200   0

```
220   0
240   1
260   2
280   3
300   4
320   3
340   2
360   0
H2U-P:H2U-RIB:GLU-S1 H2U-P:H2U-RIB:GLU-S1_100 0.0
20   0
40   0
60   0
80   0
100   0
120   0
140   0
160   0
180   0
200   0
220   0
240   0
260   0
280   0
300   0
320   0
340   0
360   0
IU-RIB:IU-MY:VAL-CA IU-RIB:IU-MY:VAL-CA_40 0.0
20   0
40   0
60   0
80   0
100   0
120   0
140   0
160   0
180   0
200   0
220   0
240   0
260   0
280   0
300   0
320   0
340   0
360   0
FHU-RIB:FHU-MY:LEU-S2 FHU-RIB:FHU-MY:LEU-S2_240 0.0
20   0
40   0
60   0
80   0
100   0
120   0
140   0
160   0
180   0
200   0
220   0
```

240   0
260   0
280   0
300   0
320   0
340   0
360   0
G-RIB:G-R5:ALA-CA G-RIB:G-R5:ALA-CA_360 -2746.94741601
20   0
40   4
60   6
80   6
100   6
120   0
140   4
160   3
180   0
200   0
220   4
240   6
260   7
280   6
300   6
320   5
340   3
360   1
U31-RIB:U31-MY:MET-CA U31-RIB:U31-MY:MET-CA_320 0.0
20   0
40   0
60   0
80   0
100   0
120   0
140   0
160   0
180   0
200   0
220   0
240   0
260   0
280   0
300   0
320   0
340   0
360   0
U-RIB:U-P:ASN-S1 U-RIB:U-P:ASN-S1_360 -2390.88345546
20   0
40   0
60   2
80   3
100   5
120   4
140   4
160   3
180   1
200   0
220   0
240   1

```
260   3
280   4
300   4
320   4
340   2
360   1
C-RIB:C-P:HIS-S1 C-RIB:C-P:HIS-S1_220 0.0
20   0
40   0
60   1
80   2
100   3
120   3
140   3
160   2
180   0
200   0
220   0
240   1
260   2
280   3
300   3
320   3
340   2
360   0
U-RIB:U-Y:LEU-S2 U-RIB:U-Y:LEU-S2_140 -4136.64493803
20   0
40   0
60   1
80   1
100   1
120   1
140   1
160   1
180   0
200   0
220   0
240   1
260   1
280   1
300   1
320   1
340   1
360   0
IU-RIB:IU-MY:SER-CA IU-RIB:IU-MY:SER-CA_140 0.0
20   0
40   0
60   0
80   0
100   0
120   0
140   0
160   0
180   0
200   0
220   0
240   0
260   0
```

280 0
300 0
320 0
340 0
360 0
QUO-RIB:QUO-P:LEU-S2 QUO-RIB:QUO-P:LEU-S2_80 -18832.852221
20 0
40 0
60 0
80 0
100 0
120 0
140 0
160 0
180 0
200 0
220 0
240 0
260 0
280 0
300 0
320 0
340 0
360 0
C31-RIB:C31-P:TYR-CA C31-RIB:C31-P:TYR-CA_340 0.0
20 0
40 0
60 0
80 0
100 0
120 0
140 0
160 0
180 0
200 0
220 0
240 0
260 0
280 0
300 0
320 0
340 0
360 0
U-RIB:U-P:SER-CA U-RIB:U-P:SER-CA_240 -5289.05495459
20 0
40 0
60 2
80 4
100 6
120 6
140 5
160 3
180 1
200 0
220 0
240 2
260 4
280 5

300   6
320   5
340   3
360   1
G-RIB:G-P:ASP-S1 G-RIB:G-P:ASP-S1_340 -3217.59863543
20   0
40   2
60   8
80   14
100   19
120   20
140   17
160   12
180   4
200   0
220   2
240   7
260   14
280   19
300   19
320   17
340   12
360   4
GTP-RIB:GTP-M5:THR-CA GTP-RIB:GTP-M5:THR-CA_140 0.0
20   0
40   0
60   0
80   0
100   0
120   0
140   0
160   0
180   0
200   0
220   0
240   0
260   0
280   0
300   0
320   0
340   0
360   0
IU-RIB:IU-P:ARG-S2 IU-RIB:IU-P:ARG-S2_200 0.0
20   0
40   0
60   0
80   0
100   0
120   0
140   0
160   0
180   0
200   0
220   0
240   0
260   0
280   0
300   0

320   0
340   0
360   0
G-RIB:G-P:VAL-CA G-RIB:G-P:VAL-CA_220 -5619.90737607
20   0
40   0
60   3
80   7
100   11
120   12
140   11
160   7
180   2
200   0
220   0
240   0
260   7
280   11
300   12
320   11
340   7
360   0
A-RIB:A-R6:TYR-S1 A-RIB:A-R6:TYR-S1_40 0.0
20   0
40   0
60   0
80   1
100   1
120   1
140   1
160   1
180   0
200   0
220   0
240   0
260   1
280   1
300   1
320   1
340   1
360   0
C31-RIB:C31-MY:SER-CA C31-RIB:C31-MY:SER-CA_260 0.0
20   0
40   0
60   0
80   0
100   0
120   0
140   0
160   0
180   0
200   0
220   0
240   0
260   0
280   0
300   0
320   0

340 0
360 0
C-RIB:C-P:TRP-S1 C-RIB:C-P:TRP-S1_160 -2999.67204536
20 0
40 0
60 0
80 1
100 1
120 1
140 1
160 0
180 0
200 0
220 0
240 0
260 1
280 1
300 0
320 1
340 0
360 0
U34-RIB:U34-P:GLU-CA U34-RIB:U34-P:GLU-CA_60 0.0
20 0
40 0
60 0
80 0
100 0
120 0
140 0
160 0
180 0
200 0
220 0
240 0
260 0
280 0
300 0
320 0
340 0
360 0
U-P:U-RIB:THR-S1 U-P:U-RIB:THR-S1_120 -2265.66378163
20 0
40 1
60 3
80 4
100 5
120 4
140 4
160 3
180 1
200 0
220 1
240 3
260 4
280 4
300 4
320 4
340 3

```
360   0
QUO-RIB:QUO-P:SER-S1 QUO-RIB:QUO-P:SER-S1_300 0.0
20   0
40   0
60   0
80   0
100   0
120   0
140   0
160   0
180   0
200   0
220   0
240   0
260   0
280   0
300   0
320   0
340   0
360   0
U-RIB:U-P:VAL-CA U-RIB:U-P:VAL-CA_160 -1412.40766992
20   0
40   0
60   1
80   3
100   5
120   5
140   4
160   3
180   1
200   0
220   0
240   1
260   3
280   4
300   5
320   4
340   3
360   1
A-P:A-RIB:THR-CA A-P:A-RIB:THR-CA_340 -4091.02158343
20   0
40   1
60   3
80   5
100   6
120   5
140   5
160   4
180   1
200   0
220   1
240   3
260   4
280   5
300   6
320   5
340   4
360   1
```

```
IU-RIB:IU-MY:ARG-S2 IU-RIB:IU-MY:ARG-S2_80 0.0
20   0
40   0
60   0
80   0
100   0
120   0
140   0
160   0
180   0
200   0
220   0
240   0
260   0
280   0
300   0
320   0
340   0
360   0
A-RIB:A-R6:ASN-S2 A-RIB:A-R6:ASN-S2_80 -3477.74246755
20   0
40   1
60   4
80   5
100   5
120   5
140   4
160   3
180   1
200   0
220   1
240   4
260   6
280   6
300   5
320   4
340   3
360   1
DA-RIB:DA-M5:ASN-S2 DA-RIB:DA-M5:ASN-S2_200 0.0
20   0
40   0
60   0
80   0
100   0
120   0
140   0
160   0
180   0
200   0
220   0
240   0
260   0
280   0
300   0
320   0
340   0
360   0
H2U-RIB:H2U-MY:ASN-S1 H2U-RIB:H2U-MY:ASN-S1_300 0.0
```

20 0
40 0
60 0
80 0
100 0
120 0
140 0
160 0
180 0
200 0
220 0
240 0
260 0
280 0
300 0
320 0
340 0
360 0
G-RIB:G-R6:ASP-CA G-RIB:G-R6:ASP-CA_140 946.973058471
20 0
40 1
60 5
80 8
100 9
120 7
140 5
160 4
180 1
200 0
220 1
240 5
260 8
280 9
300 7
320 5
340 4
360 1
U-P:U-RIB:VAL-CA U-P:U-RIB:VAL-CA_240 0.0
20 0
40 0
60 2
80 3
100 3
120 4
140 3
160 0
180 1
200 0
220 0
240 0
260 3
280 4
300 4
320 3
340 2
360 1
H2U-RIB:H2U-MY:ASN-CA H2U-RIB:H2U-MY:ASN-CA_140 -8236.50245246
20 0

```
40   0
60   0
80   0
100   0
120   0
140   0
160   0
180   0
200   0
220   0
240   0
260   0
280   0
300   0
320   0
340   0
360   0
H2U-RIB:H2U-P:PHE-S2 H2U-RIB:H2U-P:PHE-S2_180 0.0
20   0
40   0
60   0
80   0
100   0
120   0
140   0
160   0
180   0
200   0
220   0
240   0
260   0
280   0
300   0
320   0
340   0
360   0
C31-RIB:C31-MY:PHE-S1 C31-RIB:C31-MY:PHE-S1_80 0.0
20   0
40   0
60   0
80   0
100   0
120   0
140   0
160   0
180   0
200   0
220   0
240   0
260   0
280   0
300   0
320   0
340   0
360   0
U-P:U-RIB:LEU-CA U-P:U-RIB:LEU-CA_220 0.0
20   0
40   0
```

60   1
80   0
100   3
120   3
140   2
160   1
180   0
200   0
220   0
240   1
260   2
280   3
300   3
320   2
340   1
360   0
C-RIB:C-P:ALA-CA C-RIB:C-P:ALA-CA_180 -2460.12544088
20   0
40   1
60   3
80   7
100   9
120   9
140   8
160   5
180   2
200   0
220   1
240   4
260   7
280   9
300   9
320   8
340   5
360   0
U-RIB:U-Y:PRO-S1 U-RIB:U-Y:PRO-S1_300 -4827.60824117
20   0
40   1
60   2
80   3
100   2
120   2
140   2
160   1
180   0
200   0
220   1
240   2
260   3
280   2
300   2
320   0
340   1
360   0
A-P:A-RIB:ALA-CA A-P:A-RIB:ALA-CA_140 -2423.61461383
20   0
40   1
60   4

80   7
100   8
120   8
140   7
160   6
180   0
200   0
220   1
240   4
260   7
280   8
300   8
320   7
340   6
360   0
C-RIB:C-Y:TRP-S2 C-RIB:C-Y:TRP-S2_260 -3598.71450922
20   0
40   0
60   0
80   0
100   0
120   0
140   0
160   0
180   0
200   0
220   0
240   0
260   0
280   0
300   0
320   0
340   0
360   0
G-RIB:G-P:LEU-CA G-RIB:G-P:LEU-CA_140 -1862.30857069
20   0
40   0
60   2
80   5
100   8
120   10
140   9
160   6
180   1
200   0
220   0
240   2
260   6
280   9
300   10
320   9
340   6
360   2
H2U-RIB:H2U-MY:THR-CA H2U-RIB:H2U-MY:THR-CA_180 0.0
20   0
40   0
60   0
80   0

100 0
120 0
140 0
160 0
180 0
200 0
220 0
240 0
260 0
280 0
300 0
320 0
340 0
360 0
FMU-P:FMU-RIB:ARG-S2 FMU-P:FMU-RIB:ARG-S2_120 0.0
20 0
40 0
60 0
80 0
100 0
120 0
140 0
160 0
180 0
200 0
220 0
240 0
260 0
280 0
300 0
320 0
340 0
360 0
G-RIB:G-R6:ARG-S2 G-RIB:G-R6:ARG-S2_320 -3300.30232541
20 0
40 4
60 12
80 16
100 16
120 14
140 10
160 7
180 2
200 0
220 4
240 13
260 16
280 16
300 14
320 10
340 7
360 2
IU-P:IU-RIB:PRO-CA IU-P:IU-RIB:PRO-CA_300 0.0
20 0
40 0
60 0
80 0
100 0

```
120   0
140   0
160   0
180   0
200   0
220   0
240   0
260   0
280   0
300   0
320   0
340   0
360   0
C31-RIB:C31-MY:THR-CA C31-RIB:C31-MY:THR-CA_140 0.0
20   0
40   0
60   0
80   0
100   0
120   0
140   0
160   0
180   0
200   0
220   0
240   0
260   0
280   0
300   0
320   0
340   0
360   0
U34-RIB:U34-P:GLU-S1 U34-RIB:U34-P:GLU-S1_360 -15398.0131375
20   0
40   0
60   0
80   0
100   0
120   0
140   0
160   0
180   0
200   0
220   0
240   0
260   0
280   0
300   0
320   0
340   0
360   0
FMU-P:FMU-RIB:HIS-S2 FMU-P:FMU-RIB:HIS-S2_60 0.0
20   0
40   0
60   0
80   0
100   0
120   0
```

140 0
160 0
180 0
200 0
220 0
240 0
260 0
280 0
300 0
320 0
340 0
360 0
QUO-RIB:QUO-M5:PHE-CA QUO-RIB:QUO-M5:PHE-CA_240 0.0
20 0
40 0
60 0
80 0
100 0
120 0
140 0
160 0
180 0
200 0
220 0
240 0
260 0
280 0
300 0
320 0
340 0
360 0
C-RIB:C-Y:ASN-CA C-RIB:C-Y:ASN-CA_360 -5845.30930227
20 0
40 1
60 2
80 2
100 2
120 2
140 2
160 0
180 0
200 0
220 0
240 2
260 2
280 3
300 2
320 2
340 1
360 0
G-RIB:G-R5:VAL-CA G-RIB:G-R5:VAL-CA_160 -1524.43907918
20 0
40 1
60 3
80 3
100 3
120 3
140 3

160   2
180   0
200   0
220   2
240   3
260   4
280   4
300   3
320   3
340   2
360   0
A-P:A-RIB:GLN-CA A-P:A-RIB:GLN-CA_300 -2301.47175081
20   0
40   0
60   2
80   3
100   4
120   4
140   3
160   2
180   1
200   0
220   0
240   2
260   3
280   4
300   4
320   3
340   3
360   1
DA-RIB:DA-M5:TYR-CA DA-RIB:DA-M5:TYR-CA_360 0.0
20   0
40   0
60   0
80   0
100   0
120   0
140   0
160   0
180   0
200   0
220   0
240   0
260   0
280   0
300   0
320   0
340   0
360   0
A-RIB:A-P:GLU-S1 A-RIB:A-P:GLU-S1_260 -1449.19149064
20   0
40   2
60   7
80   13
100   17
120   17
140   15
160   11

180   0
200   0
220   2
240   6
260   13
280   17
300   18
320   15
340   11
360   4
U31-RIB:U31-P:HIS-S1 U31-RIB:U31-P:HIS-S1_240 0.0
20   0
40   0
60   0
80   0
100   0
120   0
140   0
160   0
180   0
200   0
220   0
240   0
260   0
280   0
300   0
320   0
340   0
360   0
QUO-RIB:QUO-M5:ASN-CA QUO-RIB:QUO-M5:ASN-CA_360 0.0
20   0
40   0
60   0
80   0
100   0
120   0
140   0
160   0
180   0
200   0
220   0
240   0
260   0
280   0
300   0
320   0
340   0
360   0
I-P:I-RIB:TRP-S1 I-P:I-RIB:TRP-S1_320 0.0
20   0
40   0
60   0
80   0
100   0
120   0
140   0
160   0
180   0

200   0
220   0
240   0
260   0
280   0
300   0
320   0
340   0
360   0
FHU-P:FHU-RIB:SER-S1 FHU-P:FHU-RIB:SER-S1_160 0.0
20   0
40   0
60   0
80   0
100   0
120   0
140   0
160   0
180   0
200   0
220   0
240   0
260   0
280   0
300   0
320   0
340   0
360   0
U-RIB:U-Y:LYS-S2 U-RIB:U-Y:LYS-S2_180 -5083.83617638
20   1
40   4
60   7
80   8
100   7
120   6
140   5
160   3
180   1
200   1
220   4
240   7
260   8
280   7
300   6
320   5
340   3
360   1
G-RIB:G-R6:SER-CA G-RIB:G-R6:SER-CA_320 -2851.90829314
20   0
40   2
60   5
80   7
100   8
120   6
140   4
160   3
180   0
200   0

220   2
240   5
260   8
280   8
300   6
320   5
340   3
360   1
C-RIB:C-Y:PHE-S2 C-RIB:C-Y:PHE-S2_340 0.0
20   0
40   0
60   0
80   1
100   1
120   1
140   0
160   0
180   0
200   0
220   0
240   1
260   1
280   1
300   1
320   0
340   0
360   0
A-P:A-RIB:ARG-S2 A-P:A-RIB:ARG-S2_260 -5531.08159582
20   0
40   4
60   11
80   15
100   16
120   14
140   14
160   11
180   5
200   0
220   4
240   11
260   15
280   15
300   15
320   14
340   11
360   4
U-P:U-RIB:LEU-S1 U-P:U-RIB:LEU-S1_120 -3862.12171377
20   0
40   0
60   1
80   2
100   2
120   3
140   2
160   1
180   0
200   0
220   0

240   0
260   2
280   3
300   3
320   2
340   1
360   0
FHU-P:FHU-RIB:LYS-CA FHU-P:FHU-RIB:LYS-CA_360 0.0
20   0
40   0
60   0
80   0
100   0
120   0
140   0
160   0
180   0
200   0
220   0
240   0
260   0
280   0
300   0
320   0
340   0
360   0
A-RIB:A-R6:LEU-S1 A-RIB:A-R6:LEU-S1_340 -1696.44798754
20   0
40   0
60   1
80   2
100   3
120   3
140   3
160   2
180   0
200   0
220   0
240   1
260   2
280   3
300   3
320   3
340   2
360   0
DA-RIB:DA-M6:HIS-S2 DA-RIB:DA-M6:HIS-S2_60 0.0
20   0
40   0
60   0
80   0
100   0
120   0
140   0
160   0
180   0
200   0
220   0
240   0

260   0
280   0
300   0
320   0
340   0
360   0
OMC-RIB:OMC-P:LYS-S1 OMC-RIB:OMC-P:LYS-S1_340 0.0
20   0
40   0
60   0
80   0
100   0
120   0
140   0
160   0
180   0
200   0
220   0
240   0
260   0
280   0
300   0
320   0
340   0
360   0
FMU-RIB:FMU-MY:PHE-S2 FMU-RIB:FMU-MY:PHE-S2_80 0.0
20   0
40   0
60   0
80   0
100   0
120   0
140   0
160   0
180   0
200   0
220   0
240   0
260   0
280   0
300   0
320   0
340   0
360   0
C31-P:C31-RIB:SER-CA C31-P:C31-RIB:SER-CA_200 0.0
20   0
40   0
60   0
80   0
100   0
120   0
140   0
160   0
180   0
200   0
220   0
240   0
260   0

280   0
300   0
320   0
340   0
360   0
G-RIB:G-R5:LYS-S1 G-RIB:G-R5:LYS-S1_60 -3336.70864294
20   1
40   6
60   10
80   11
100   9
120   8
140   7
160   5
180   2
200   1
220   5
240   10
260   10
280   9
300   8
320   7
340   4
360   0
A-P:A-RIB:TYR-S2 A-P:A-RIB:TYR-S2_300 -5395.02592307
20   0
40   0
60   1
80   2
100   2
120   2
140   2
160   1
180   0
200   0
220   0
240   1
260   2
280   2
300   2
320   2
340   0
360   0
A-RIB:A-P:SER-S1 A-RIB:A-P:SER-S1_100 -2101.70179976
20   0
40   2
60   5
80   8
100   10
120   9
140   8
160   6
180   2
200   0
220   2
240   4
260   7
280   9

300   9
320   8
340   5
360   2
U31-RIB:U31-MY:ILE-S1 U31-RIB:U31-MY:ILE-S1_180 0.0
20   0
40   0
60   0
80   0
100   0
120   0
140   0
160   0
180   0
200   0
220   0
240   0
260   0
280   0
300   0
320   0
340   0
360   0
U31-P:U31-RIB:ASP-CA U31-P:U31-RIB:ASP-CA_160 0.0
20   0
40   0
60   0
80   0
100   0
120   0
140   0
160   0
180   0
200   0
220   0
240   0
260   0
280   0
300   0
320   0
340   0
360   0
U-P:U-RIB:PHE-S2 U-P:U-RIB:PHE-S2_300 -5898.31052441
20   0
40   0
60   1
80   1
100   1
120   1
140   1
160   0
180   0
200   0
220   0
240   0
260   0
280   1
300   1

320   1
340   0
360   0
U-P:U-RIB:ARG-S2 U-P:U-RIB:ARG-S2_280 -4045.97444368
20   0
40   3
60   7
80   9
100   10
120   9
140   8
160   6
180   2
200   0
220   3
240   7
260   9
280   9
300   9
320   8
340   6
360   2
C-RIB:C-P:TYR-S1 C-RIB:C-P:TYR-S1_220 0.0
20   0
40   0
60   0
80   1
100   2
120   2
140   2
160   1
180   0
200   0
220   0
240   0
260   1
280   2
300   2
320   2
340   1
360   0
QUO-RIB:QUO-M5:LYS-S1 QUO-RIB:QUO-M5:LYS-S1_220 0.0
20   0
40   0
60   0
80   0
100   0
120   0
140   0
160   0
180   0
200   0
220   0
240   0
260   0
280   0
300   0
320   0

340   0
360   0
H2U-P:H2U-RIB:ASN-S2 H2U-P:H2U-RIB:ASN-S2_280 0.0
20   0
40   0
60   0
80   0
100   0
120   0
140   0
160   0
180   0
200   0
220   0
240   0
260   0
280   0
300   0
320   0
340   0
360   0
IU-RIB:IU-MY:VAL-S1 IU-RIB:IU-MY:VAL-S1_260 0.0
20   0
40   0
60   0
80   0
100   0
120   0
140   0
160   0
180   0
200   0
220   0
240   0
260   0
280   0
300   0
320   0
340   0
360   0
C-RIB:C-P:PHE-S2 C-RIB:C-P:PHE-S2_160 -3105.84564371
20   0
40   0
60   1
80   2
100   2
120   3
140   2
160   1
180   0
200   0
220   0
240   1
260   2
280   2
300   3
320   2
340   1

360   0
C-RIB:C-P:ASP-S1 C-RIB:C-P:ASP-S1_40 -4815.46681779
20   0
40   2
60   6
80   10
100   13
120   13
140   10
160   7
180   3
200   0
220   1
240   5
260   10
280   12
300   12
320   10
340   7
360   3
QUO-RIB:QUO-M6:ASP-S2 QUO-RIB:QUO-M6:ASP-S2_120 -3434.83908349
20   0
40   0
60   0
80   0
100   0
120   0
140   0
160   0
180   0
200   0
220   0
240   0
260   0
280   0
300   0
320   0
340   0
360   0
U-RIB:U-P:ASP-S1 U-RIB:U-P:ASP-S1_140 -3234.99197638
20   0
40   0
60   3
80   6
100   8
120   8
140   7
160   0
180   1
200   0
220   1
240   3
260   6
280   8
300   8
320   7
340   5
360   2

A-RIB:A-R6:MET-S2 A-RIB:A-R6:MET-S2_160 -4902.62931523
20 0
40 0
60 1
80 1
100 1
120 1
140 1
160 1
180 0
200 0
220 0
240 1
260 1
280 1
300 1
320 1
340 0
360 0
H2U-P:H2U-RIB:GLU-S2 H2U-P:H2U-RIB:GLU-S2_160 0.0
20 0
40 0
60 0
80 0
100 0
120 0
140 0
160 0
180 0
200 0
220 0
240 0
260 0
280 0
300 0
320 0
340 0
360 0
C-RIB:C-P:MET-S2 C-RIB:C-P:MET-S2_240 -5711.34626385
20 0
40 0
60 1
80 2
100 2
120 2
140 2
160 0
180 0
200 0
220 0
240 1
260 2
280 2
300 2
320 2
340 1
360 0
U31-RIB:U31-MY:MET-S1 U31-RIB:U31-MY:MET-S1_80 -9456.71767237

```
20   0
40   0
60   0
80   0
100   0
120   0
140   0
160   0
180   0
200   0
220   0
240   0
260   0
280   0
300   0
320   0
340   0
360   0
G-RIB:G-P:GLN-S1 G-RIB:G-P:GLN-S1_140 -3003.23795529
20   0
40   1
60   3
80   7
100   9
120   9
140   8
160   5
180   2
200   0
220   1
240   3
260   7
280   9
300   9
320   8
340   6
360   2
C-RIB:C-P:PRO-S1 C-RIB:C-P:PRO-S1_320 -1396.03145264
20   0
40   1
60   3
80   6
100   8
120   7
140   6
160   4
180   0
200   0
220   1
240   3
260   6
280   7
300   7
320   6
340   4
360   1
FHU-RIB:FHU-MY:SER-CA FHU-RIB:FHU-MY:SER-CA_360 0.0
20   0
```

```
40   0
60   0
80   0
100   0
120   0
140   0
160   0
180   0
200   0
220   0
240   0
260   0
280   0
300   0
320   0
340   0
360   0
C-RIB:C-Y:SER-S1 C-RIB:C-Y:SER-S1_260 -3905.28232243
20   1
40   4
60   6
80   6
100   4
120   3
140   2
160   2
180   0
200   1
220   4
240   6
260   6
280   4
300   4
320   3
340   2
360   0
C-RIB:C-P:LEU-S1 C-RIB:C-P:LEU-S1_180 -1787.78527919
20   0
40   0
60   2
80   4
100   6
120   6
140   5
160   3
180   1
200   0
220   0
240   2
260   4
280   5
300   6
320   5
340   3
360   1
H2U-RIB:H2U-P:GLU-S1 H2U-RIB:H2U-P:GLU-S1_300 0.0
20   0
40   0
```

60 0
80 0
100 0
120 0
140 0
160 0
180 0
200 0
220 0
240 0
260 0
280 0
300 0
320 0
340 0
360 0
U34-RIB:U34-MY:ASN-S2 U34-RIB:U34-MY:ASN-S2_20 0.0
20 0
40 0
60 0
80 0
100 0
120 0
140 0
160 0
180 0
200 0
220 0
240 0
260 0
280 0
300 0
320 0
340 0
360 0
A-P:A-RIB:TRP-CA A-P:A-RIB:TRP-CA_220 0.0
20 0
40 0
60 0
80 0
100 1
120 1
140 1
160 0
180 0
200 0
220 0
240 0
260 1
280 1
300 1
320 1
340 0
360 0
A-RIB:A-R6:TRP-S1 A-RIB:A-R6:TRP-S1_220 0.0
20 0
40 0
60 0

80 0
100 1
120 1
140 0
160 0
180 0
200 0
220 0
240 0
260 0
280 0
300 0
320 0
340 0
360 0
GTP-RIB:GTP-M5:ALA-CA GTP-RIB:GTP-M5:ALA-CA_360 0.0
20 0
40 0
60 0
80 0
100 0
120 0
140 0
160 0
180 0
200 0
220 0
240 0
260 0
280 0
300 0
320 0
340 0
360 0
U-P:U-RIB:GLY-CA U-P:U-RIB:GLY-CA_40 -4338.47851963
20 0
40 2
60 5
80 7
100 8
120 8
140 7
160 5
180 2
200 0
220 2
240 5
260 7
280 7
300 7
320 7
340 5
360 2
C31-RIB:C31-P:ASN-S1 C31-RIB:C31-P:ASN-S1_320 0.0
20 0
40 0
60 0
80 0

100 0
120 0
140 0
160 0
180 0
200 0
220 0
240 0
260 0
280 0
300 0
320 0
340 0
360 0
U-P:U-RIB:THR-CA U-P:U-RIB:THR-CA_320 -3169.57746513
20 0
40 0
60 2
80 3
100 4
120 4
140 3
160 2
180 0
200 0
220 0
240 2
260 3
280 4
300 4
320 3
340 2
360 1
FHU-RIB:FHU-P:VAL-S1 FHU-RIB:FHU-P:VAL-S1_80 0.0
20 0
40 0
60 0
80 0
100 0
120 0
140 0
160 0
180 0
200 0
220 0
240 0
260 0
280 0
300 0
320 0
340 0
360 0
U-P:U-RIB:ASP-S2 U-P:U-RIB:ASP-S2_60 -123.180952108
20 0
40 2
60 5
80 7
100 8

120   7
140   6
160   5
180   2
200   0
220   2
240   5
260   7
280   8
300   7
320   7
340   5
360   2
U34-RIB:U34-P:HIS-S2 U34-RIB:U34-P:HIS-S2_280 -11671.3415359
20   0
40   0
60   0
80   0
100   0
120   0
140   0
160   0
180   0
200   0
220   0
240   0
260   0
280   0
300   0
320   0
340   0
360   0
FMU-RIB:FMU-P:ASP-S1 FMU-RIB:FMU-P:ASP-S1_300 0.0
20   0
40   0
60   0
80   0
100   0
120   0
140   0
160   0
180   0
200   0
220   0
240   0
260   0
280   0
300   0
320   0
340   0
360   0
U-RIB:U-P:THR-S1 U-RIB:U-P:THR-S1_20 0.0
20   0
40   1
60   2
80   4
100   5
120   5

140   4
160   3
180   1
200   0
220   0
240   2
260   4
280   5
300   5
320   5
340   3
360   1
A-P:A-RIB:LEU-S1 A-P:A-RIB:LEU-S1_300 -2127.88018522
20   0
40   0
60   2
80   4
100   4
120   4
140   4
160   3
180   1
200   0
220   0
240   0
260   4
280   4
300   5
320   4
340   3
360   1
A-RIB:A-R5:PHE-S1 A-RIB:A-R5:PHE-S1_320 0.0
20   0
40   0
60   0
80   1
100   1
120   1
140   0
160   0
180   0
200   0
220   0
240   0
260   1
280   1
300   1
320   0
340   0
360   0
C-RIB:C-P:ILE-S1 C-RIB:C-P:ILE-S1_340 0.0
20   0
40   0
60   1
80   2
100   3
120   4
140   4

160   2
180   0
200   0
220   0
240   1
260   3
280   4
300   4
320   3
340   0
360   1
A-RIB:A-P:THR-S1 A-RIB:A-P:THR-S1_300 -4344.6710182
20   0
40   1
60   3
80   6
100   8
120   7
140   7
160   5
180   2
200   0
220   1
240   3
260   6
280   7
300   8
320   7
340   5
360   2
FHU-RIB:FHU-MY:ASP-CA FHU-RIB:FHU-MY:ASP-CA_140 0.0
20   0
40   0
60   0
80   0
100   0
120   0
140   0
160   0
180   0
200   0
220   0
240   0
260   0
280   0
300   0
320   0
340   0
360   0
U31-P:U31-RIB:TYR-S2 U31-P:U31-RIB:TYR-S2_300 0.0
20   0
40   0
60   0
80   0
100   0
120   0
140   0
160   0

180   0
200   0
220   0
240   0
260   0
280   0
300   0
320   0
340   0
360   0
A-P:A-RIB:LYS-S2 A-P:A-RIB:LYS-S2_320 -2441.2496538
20   1
40   5
60   11
80   15
100   15
120   15
140   14
160   11
180   4
200   1
220   5
240   11
260   15
280   15
300   15
320   13
340   11
360   4
A-RIB:A-R6:GLU-S2 A-RIB:A-R6:GLU-S2_140 -663.728291544
20   0
40   4
60   11
80   14
100   15
120   14
140   12
160   8
180   2
200   0
220   4
240   11
260   15
280   14
300   14
320   11
340   8
360   2
A-RIB:A-R5:PHE-S2 A-RIB:A-R5:PHE-S2_80 -4964.63207387
20   0
40   0
60   0
80   1
100   1
120   1
140   0
160   0
180   0

200 0
220 0
240 0
260 1
280 1
300 1
320 0
340 0
360 0
C-RIB:C-P:ASN-CA C-RIB:C-P:ASN-CA_40 -3609.32571454
20 0
40 0
60 2
80 4
100 6
120 6
140 5
160 4
180 1
200 0
220 0
240 2
260 4
280 6
300 6
320 5
340 3
360 1
G-RIB:G-P:LEU-S1 G-RIB:G-P:LEU-S1_100 -3316.31192697
20 0
40 0
60 2
80 5
100 8
120 8
140 8
160 6
180 2
200 0
220 0
240 2
260 5
280 8
300 9
320 8
340 5
360 2
QUO-RIB:QUO-M6:PHE-CA QUO-RIB:QUO-M6:PHE-CA_340 0.0
20 0
40 0
60 0
80 0
100 0
120 0
140 0
160 0
180 0
200 0

220 0
240 0
260 0
280 0
300 0
320 0
340 0
360 0
A-RIB:A-P:ARG-S1 A-RIB:A-P:ARG-S1_360 -5328.26485931
20 0
40 1
60 5
80 10
100 13
120 14
140 12
160 8
180 3
200 0
220 1
240 5
260 10
280 13
300 14
320 12
340 8
360 3
U-P:U-RIB:TRP-S2 U-P:U-RIB:TRP-S2_100 0.0
20 0
40 0
60 0
80 0
100 0
120 0
140 0
160 0
180 0
200 0
220 0
240 0
260 0
280 0
300 0
320 0
340 0
360 0
G-P:G-RIB:ASN-S1 G-P:G-RIB:ASN-S1_300 -3571.80166323
20 0
40 2
60 5
80 8
100 8
120 7
140 6
160 5
180 0
200 0
220 2

240   6
260   8
280   9
300   8
320   6
340   5
360   2
DA-RIB:DA-M5:TYR-S1 DA-RIB:DA-M5:TYR-S1_20 0.0
20   0
40   0
60   0
80   0
100   0
120   0
140   0
160   0
180   0
200   0
220   0
240   0
260   0
280   0
300   0
320   0
340   0
360   0
A-RIB:A-P:MET-CA A-RIB:A-P:MET-CA_20 0.0
20   0
40   0
60   0
80   1
100   2
120   2
140   2
160   1
180   0
200   0
220   0
240   0
260   1
280   2
300   2
320   2
340   0
360   0
A-RIB:A-R5:ARG-S2 A-RIB:A-R5:ARG-S2_20 -6289.1964355
20   1
40   6
60   10
80   12
100   11
120   9
140   7
160   5
180   1
200   2
220   6
240   10

260   13
280   11
300   8
320   7
340   5
360   1
C31-RIB:C31-MY:SER-S1 C31-RIB:C31-MY:SER-S1_20 0.0
20   0
40   0
60   0
80   0
100   0
120   0
140   0
160   0
180   0
200   0
220   0
240   0
260   0
280   0
300   0
320   0
340   0
360   0
QUO-RIB:QUO-M5:ASP-CA QUO-RIB:QUO-M5:ASP-CA_260 0.0
20   0
40   0
60   0
80   0
100   0
120   0
140   0
160   0
180   0
200   0
220   0
240   0
260   0
280   0
300   0
320   0
340   0
360   0
U31-RIB:U31-MY:PHE-CA U31-RIB:U31-MY:PHE-CA_140 0.0
20   0
40   0
60   0
80   0
100   0
120   0
140   0
160   0
180   0
200   0
220   0
240   0
260   0

280   0
300   0
320   0
340   0
360   0
U-RIB:U-P:ASN-S2 U-RIB:U-P:ASN-S2_20 0.0
20   0
40   1
60   2
80   4
100   5
120   5
140   4
160   3
180   1
200   0
220   1
240   2
260   4
280   5
300   5
320   4
340   3
360   1
FHU-P:FHU-RIB:ASP-S2 FHU-P:FHU-RIB:ASP-S2_80 0.0
20   0
40   0
60   0
80   0
100   0
120   0
140   0
160   0
180   0
200   0
220   0
240   0
260   0
280   0
300   0
320   0
340   0
360   0
G-RIB:G-P:TRP-CA G-RIB:G-P:TRP-CA_300 -4194.32035243
20   0
40   0
60   0
80   1
100   2
120   2
140   0
160   1
180   0
200   0
220   0
240   0
260   1
280   2

300   2
320   2
340   0
360   0
QUO-RIB:QUO-M6:ARG-S2 QUO-RIB:QUO-M6:ARG-S2_240 0.0
20   0
40   0
60   0
80   0
100   0
120   0
140   0
160   0
180   0
200   0
220   0
240   0
260   0
280   0
300   0
320   0
340   0
360   0
U-RIB:U-P:CYS-CA U-RIB:U-P:CYS-CA_80 -5832.2342711
20   0
40   0
60   0
80   0
100   0
120   0
140   0
160   0
180   0
200   0
220   0
240   0
260   0
280   0
300   0
320   0
340   0
360   0
IU-RIB:IU-P:ALA-CA IU-RIB:IU-P:ALA-CA_340 0.0
20   0
40   0
60   0
80   0
100   0
120   0
140   0
160   0
180   0
200   0
220   0
240   0
260   0
280   0
300   0

320   0
340   0
360   0
H2U-RIB:H2U-MY:THR-S1 H2U-RIB:H2U-MY:THR-S1_80 0.0
20   0
40   0
60   0
80   0
100   0
120   0
140   0
160   0
180   0
200   0
220   0
240   0
260   0
280   0
300   0
320   0
340   0
360   0
A-RIB:A-P:TRP-S2 A-RIB:A-P:TRP-S2_120 0.0
20   0
40   0
60   0
80   1
100   1
120   0
140   1
160   0
180   0
200   0
220   0
240   0
260   0
280   1
300   1
320   1
340   0
360   0
QUO-RIB:QUO-M6:ARG-S1 QUO-RIB:QUO-M6:ARG-S1_180 -3434.83908349
20   0
40   0
60   0
80   0
100   0
120   0
140   0
160   0
180   0
200   0
220   0
240   0
260   0
280   0
300   0
320   0

340   0
360   0
G-P:G-RIB:ASP-S1 G-P:G-RIB:ASP-S1_80 -198.529314482
20   0
40   4
60   10
80   14
100   15
120   15
140   12
160   10
180   5
200   0
220   4
240   10
260   15
280   15
300   14
320   13
340   10
360   5
C31-RIB:C31-MY:GLU-CA C31-RIB:C31-MY:GLU-CA_20 0.0
20   0
40   0
60   0
80   0
100   0
120   0
140   0
160   0
180   0
200   0
220   0
240   0
260   0
280   0
300   0
320   0
340   0
360   0
A-RIB:A-R6:THR-CA A-RIB:A-R6:THR-CA_220 -6642.55321383
20   0
40   0
60   2
80   4
100   5
120   5
140   4
160   2
180   0
200   0
220   0
240   2
260   4
280   5
300   4
320   4
340   2

360   0
H2U-P:H2U-RIB:GLU-CA H2U-P:H2U-RIB:GLU-CA_140 0.0
20   0
40   0
60   0
80   0
100   0
120   0
140   0
160   0
180   0
200   0
220   0
240   0
260   0
280   0
300   0
320   0
340   0
360   0
G-RIB:G-P:PHE-S2 G-RIB:G-P:PHE-S2_140 -2559.04245433
20   0
40   0
60   1
80   3
100   4
120   4
140   4
160   2
180   0
200   0
220   0
240   1
260   3
280   4
300   4
320   4
340   2
360   1
QUO-P:QUO-RIB:LEU-CA QUO-P:QUO-RIB:LEU-CA_20 0.0
20   0
40   0
60   0
80   0
100   0
120   0
140   0
160   0
180   0
200   0
220   0
240   0
260   0
280   0
300   0
320   0
340   0
360   0

G-RIB:G-R6:THR-CA G-RIB:G-R6:THR-CA_360 0.0
20 0
40 0
60 3
80 5
100 6
120 5
140 4
160 3
180 0
200 0
220 0
240 3
260 6
280 6
300 5
320 4
340 0
360 0
A-P:A-RIB:PRO-CA A-P:A-RIB:PRO-CA_220 0.0
20 0
40 1
60 3
80 5
100 6
120 6
140 5
160 4
180 2
200 0
220 0
240 3
260 5
280 6
300 6
320 5
340 4
360 0
G-P:G-RIB:ALA-S1 G-P:G-RIB:ALA-S1_340 -2912.15249262
20 1
40 5
60 9
80 12
100 13
120 12
140 11
160 8
180 3
200 1
220 5
240 9
260 12
280 13
300 13
320 11
340 8
360 3
C-RIB:C-Y:ASP-S2 C-RIB:C-Y:ASP-S2_140 -2280.65051353

20   1
40   5
60   9
80   9
100   7
120   5
140   4
160   3
180   1
200   1
220   5
240   8
260   9
280   7
300   5
320   5
340   3
360   0
FHU-P:FHU-RIB:GLY-CA FHU-P:FHU-RIB:GLY-CA_80 0.0
20   0
40   0
60   0
80   0
100   0
120   0
140   0
160   0
180   0
200   0
220   0
240   0
260   0
280   0
300   0
320   0
340   0
360   0
A-RIB:A-R5:SER-CA A-RIB:A-R5:SER-CA_180 -7778.31803399
20   0
40   2
60   4
80   4
100   5
120   4
140   3
160   2
180   0
200   0
220   2
240   4
260   5
280   4
300   4
320   3
340   2
360   0
U-RIB:U-Y:GLU-S2 U-RIB:U-Y:GLU-S2_120 2043.8804576
20   1

40   4
60   8
80   9
100   7
120   6
140   5
160   4
180   1
200   1
220   0
240   8
260   9
280   7
300   6
320   0
340   4
360   0
FMU-RIB:FMU-MY:ALA-S1 FMU-RIB:FMU-MY:ALA-S1_340 0.0
20   0
40   0
60   0
80   0
100   0
120   0
140   0
160   0
180   0
200   0
220   0
240   0
260   0
280   0
300   0
320   0
340   0
360   0
U31-RIB:U31-P:ARG-S2 U31-RIB:U31-P:ARG-S2_240 0.0
20   0
40   0
60   0
80   0
100   0
120   0
140   0
160   0
180   0
200   0
220   0
240   0
260   0
280   0
300   0
320   0
340   0
360   0
U31-RIB:U31-MY:VAL-S1 U31-RIB:U31-MY:VAL-S1_80 0.0
20   0
40   0

60   0
80   0
100   0
120   0
140   0
160   0
180   0
200   0
220   0
240   0
260   0
280   0
300   0
320   0
340   0
360   0
C31-P:C31-RIB:ASP-S1 C31-P:C31-RIB:ASP-S1_60 0.0
20   0
40   0
60   0
80   0
100   0
120   0
140   0
160   0
180   0
200   0
220   0
240   0
260   0
280   0
300   0
320   0
340   0
360   0
A-RIB:A-R5:ASN-S1 A-RIB:A-R5:ASN-S1_360 -5601.16899922
20   0
40   2
60   3
80   4
100   4
120   3
140   2
160   1
180   0
200   0
220   2
240   3
260   4
280   4
300   3
320   2
340   1
360   0
GTP-RIB:GTP-M5:ASP-S2 GTP-RIB:GTP-M5:ASP-S2_60 0.0
20   0
40   0
60   0

80   0
100   0
120   0
140   0
160   0
180   0
200   0
220   0
240   0
260   0
280   0
300   0
320   0
340   0
360   0
5BU-P:5BU-RIB:PRO-CA 5BU-P:5BU-RIB:PRO-CA_260 0.0
20   0
40   0
60   0
80   0
100   0
120   0
140   0
160   0
180   0
200   0
220   0
240   0
260   0
280   0
300   0
320   0
340   0
360   0
M2G-RIB:M2G-P:GLU-S1 M2G-RIB:M2G-P:GLU-S1_300 0.0
20   0
40   0
60   0
80   0
100   0
120   0
140   0
160   0
180   0
200   0
220   0
240   0
260   0
280   0
300   0
320   0
340   0
360   0
DA-RIB:DA-M5:VAL-S1 DA-RIB:DA-M5:VAL-S1_80 0.0
20   0
40   0
60   0
80   0

100 0
120 0
140 0
160 0
180 0
200 0
220 0
240 0
260 0
280 0
300 0
320 0
340 0
360 0
FMU-RIB:FMU-P:ALA-CA FMU-RIB:FMU-P:ALA-CA_300 0.0
20 0
40 0
60 0
80 0
100 0
120 0
140 0
160 0
180 0
200 0
220 0
240 0
260 0
280 0
300 0
320 0
340 0
360 0
QUO-RIB:QUO-M6:LEU-S2 QUO-RIB:QUO-M6:LEU-S2_120 0.0
20 0
40 0
60 0
80 0
100 0
120 0
140 0
160 0
180 0
200 0
220 0
240 0
260 0
280 0
300 0
320 0
340 0
360 0
FMU-RIB:FMU-MY:ASN-S1 FMU-RIB:FMU-MY:ASN-S1_320 0.0
20 0
40 0
60 0
80 0
100 0

120   0
140   0
160   0
180   0
200   0
220   0
240   0
260   0
280   0
300   0
320   0
340   0
360   0
A-RIB:A-R5:ASP-S2 A-RIB:A-R5:ASP-S2_80 -2039.79147808
20   1
40   5
60   8
80   10
100   9
120   7
140   6
160   4
180   1
200   0
220   5
240   9
260   10
280   9
300   7
320   6
340   4
360   1
U34-RIB:U34-P:GLU-S2 U34-RIB:U34-P:GLU-S2_120 0.0
20   0
40   0
60   0
80   0
100   0
120   0
140   0
160   0
180   0
200   0
220   0
240   0
260   0
280   0
300   0
320   0
340   0
360   0
DA-RIB:DA-M6:THR-CA DA-RIB:DA-M6:THR-CA_40 0.0
20   0
40   0
60   0
80   0
100   0
120   0

140   0
160   0
180   0
200   0
220   0
240   0
260   0
280   0
300   0
320   0
340   0
360   0
U-RIB:U-P:MET-S2 U-RIB:U-P:MET-S2_260 -5680.49016574
20   0
40   0
60   0
80   1
100   1
120   0
140   1
160   1
180   0
200   0
220   0
240   0
260   1
280   1
300   1
320   1
340   1
360   0
A-RIB:A-P:ALA-S1 A-RIB:A-P:ALA-S1_320 -2701.24951151
20   0
40   2
60   5
80   7
100   9
120   10
140   9
160   6
180   2
200   0
220   0
240   5
260   7
280   10
300   10
320   9
340   6
360   2
U-RIB:U-P:GLU-S2 U-RIB:U-P:GLU-S2_320 -2448.78272142
20   0
40   3
60   7
80   11
100   14
120   13
140   11

160 8
180 3
200 0
220 2
240 7
260 11
280 13
300 13
320 11
340 8
360 3
A-P:A-RIB:LYS-S1 A-P:A-RIB:LYS-S1_300 -3233.3301972
20 0
40 3
60 7
80 10
100 12
120 11
140 10
160 8
180 3
200 0
220 3
240 7
260 10
280 11
300 11
320 10
340 8
360 4
A-RIB:A-R6:ASP-S1 A-RIB:A-R6:ASP-S1_320 -1971.59963109
20 0
40 1
60 6
80 8
100 9
120 8
140 7
160 5
180 0
200 0
220 1
240 6
260 8
280 9
300 8
320 7
340 5
360 1
U34-P:U34-RIB:GLY-CA U34-P:U34-RIB:GLY-CA_340 0.0
20 0
40 0
60 0
80 0
100 0
120 0
140 0
160 0

180   0
200   0
220   0
240   0
260   0
280   0
300   0
320   0
340   0
360   0
A-P:A-RIB:TRP-S2 A-P:A-RIB:TRP-S2_180 0.0
20   0
40   0
60   0
80   0
100   1
120   1
140   1
160   0
180   0
200   0
220   0
240   0
260   1
280   1
300   1
320   1
340   1
360   0
C-RIB:C-Y:GLN-S1 C-RIB:C-Y:GLN-S1_160 -1925.81130428
20   0
40   1
60   2
80   2
100   2
120   2
140   1
160   1
180   0
200   0
220   1
240   2
260   2
280   2
300   0
320   2
340   1
360   0
H2U-RIB:H2U-MY:LYS-S1 H2U-RIB:H2U-MY:LYS-S1_300 0.0
20   0
40   0
60   0
80   0
100   0
120   0
140   0
160   0
180   0

200 0
220 0
240 0
260 0
280 0
300 0
320 0
340 0
360 0
G-P:G-RIB:MET-S1 G-P:G-RIB:MET-S1_80 -2541.70359662
20 0
40 0
60 2
80 2
100 2
120 2
140 2
160 1
180 0
200 0
220 0
240 2
260 2
280 3
300 3
320 2
340 1
360 0
G-RIB:G-R5:ARG-S1 G-RIB:G-R5:ARG-S1_260 -3848.66919679
20 1
40 5
60 8
80 8
100 8
120 7
140 6
160 4
180 1
200 1
220 5
240 8
260 9
280 8
300 7
320 6
340 4
360 1
U-RIB:U-Y:SER-CA U-RIB:U-Y:SER-CA_300 -952.647566923
20 0
40 1
60 2
80 3
100 3
120 3
140 2
160 1
180 0
200 0

220   1
240   2
260   3
280   3
300   3
320   2
340   1
360   0
U-RIB:U-Y:ASN-CA U-RIB:U-Y:ASN-CA_240 -4328.03403618
20   0
40   0
60   1
80   2
100   2
120   2
140   1
160   1
180   0
200   0
220   0
240   1
260   2
280   2
300   2
320   2
340   1
360   0
FHU-RIB:FHU-MY:TYR-S1 FHU-RIB:FHU-MY:TYR-S1_60 0.0
20   0
40   0
60   0
80   0
100   0
120   0
140   0
160   0
180   0
200   0
220   0
240   0
260   0
280   0
300   0
320   0
340   0
360   0
A-RIB:A-R6:SER-S1 A-RIB:A-R6:SER-S1_60 -3955.41626927
20   0
40   2
60   4
80   6
100   7
120   6
140   5
160   3
180   1
200   0
220   2

240   4
260   6
280   7
300   6
320   5
340   4
360   1
5BU-RIB:5BU-MY:PRO-S1 5BU-RIB:5BU-MY:PRO-S1_120 0.0
20   0
40   0
60   0
80   0
100   0
120   0
140   0
160   0
180   0
200   0
220   0
240   0
260   0
280   0
300   0
320   0
340   0
360   0
IU-RIB:IU-P:LYS-S1 IU-RIB:IU-P:LYS-S1_160 0.0
20   0
40   0
60   0
80   0
100   0
120   0
140   0
160   0
180   0
200   0
220   0
240   0
260   0
280   0
300   0
320   0
340   0
360   0
A-RIB:A-R6:ARG-S1 A-RIB:A-R6:ARG-S1_100 -4824.62157277
20   0
40   1
60   5
80   8
100   9
120   8
140   7
160   5
180   1
200   0
220   1
240   5

260   8
280   9
300   8
320   7
340   5
360   1
IU-RIB:IU-MY:GLU-S2 IU-RIB:IU-MY:GLU-S2_320 0.0
20   0
40   0
60   0
80   0
100   0
120   0
140   0
160   0
180   0
200   0
220   0
240   0
260   0
280   0
300   0
320   0
340   0
360   0
G-RIB:G-R5:TYR-CA G-RIB:G-R5:TYR-CA_360 0.0
20   0
40   0
60   0
80   1
100   0
120   1
140   1
160   0
180   0
200   0
220   0
240   1
260   1
280   0
300   1
320   1
340   0
360   0
G-P:G-RIB:GLU-S1 G-P:G-RIB:GLU-S1_360 -2636.80872131
20   0
40   0
60   14
80   20
100   21
120   20
140   17
160   14
180   7
200   0
220   5
240   14
260   20

```
280   21
300   20
320   17
340   14
360   6
U31-RIB:U31-MY:THR-CA U31-RIB:U31-MY:THR-CA_60 0.0
20   0
40   0
60   0
80   0
100   0
120   0
140   0
160   0
180   0
200   0
220   0
240   0
260   0
280   0
300   0
320   0
340   0
360   0
DA-RIB:DA-M6:TYR-S2 DA-RIB:DA-M6:TYR-S2_260 0.0
20   0
40   0
60   0
80   0
100   0
120   0
140   0
160   0
180   0
200   0
220   0
240   0
260   0
280   0
300   0
320   0
340   0
360   0
U-P:U-RIB:MET-CA U-P:U-RIB:MET-CA_280 -4955.461011
20   0
40   0
60   0
80   1
100   1
120   1
140   0
160   0
180   0
200   0
220   0
240   0
260   1
280   1
```

300   1
320   0
340   0
360   0
IU-P:IU-RIB:PRO-S1 IU-P:IU-RIB:PRO-S1_180 0.0
20   0
40   0
60   0
80   0
100   0
120   0
140   0
160   0
180   0
200   0
220   0
240   0
260   0
280   0
300   0
320   0
340   0
360   0
U-RIB:U-P:TRP-CA U-RIB:U-P:TRP-CA_120 -5705.14422643
20   0
40   0
60   0
80   0
100   0
120   0
140   0
160   0
180   0
200   0
220   0
240   0
260   0
280   0
300   0
320   0
340   0
360   0
U-P:U-RIB:HIS-S1 U-P:U-RIB:HIS-S1_360 0.0
20   0
40   0
60   1
80   1
100   2
120   2
140   1
160   1
180   0
200   0
220   0
240   0
260   1
280   2
300   2

320   1
340   1
360   0
DA-RIB:DA-M5:LEU-S2 DA-RIB:DA-M5:LEU-S2_120 -8168.61566735
20   0
40   0
60   0
80   0
100   0
120   0
140   0
160   0
180   0
200   0
220   0
240   0
260   0
280   0
300   0
320   0
340   0
360   0
C31-P:C31-RIB:GLU-S1 C31-P:C31-RIB:GLU-S1_180 0.0
20   0
40   0
60   0
80   0
100   0
120   0
140   0
160   0
180   0
200   0
220   0
240   0
260   0
280   0
300   0
320   0
340   0
360   0
H2U-RIB:H2U-P:ASN-CA H2U-RIB:H2U-P:ASN-CA_120 -9953.9219942
20   0
40   0
60   0
80   0
100   0
120   0
140   0
160   0
180   0
200   0
220   0
240   0
260   0
280   0
300   0
320   0

```
340   0
360   0
U31-P:U31-RIB:THR-S1 U31-P:U31-RIB:THR-S1_300 0.0
20   0
40   0
60   0
80   0
100   0
120   0
140   0
160   0
180   0
200   0
220   0
240   0
260   0
280   0
300   0
320   0
340   0
360   0
U-RIB:U-Y:PHE-S2 U-RIB:U-Y:PHE-S2_80 -3008.0004991
20   0
40   0
60   0
80   0
100   0
120   0
140   0
160   0
180   0
200   0
220   0
240   0
260   0
280   0
300   0
320   0
340   0
360   0
DA-RIB:DA-M6:ASN-S2 DA-RIB:DA-M6:ASN-S2_180 0.0
20   0
40   0
60   0
80   0
100   0
120   0
140   0
160   0
180   0
200   0
220   0
240   0
260   0
280   0
300   0
320   0
340   0
```

360 0
H2U-RIB:H2U-MY:ARG-S2 H2U-RIB:H2U-MY:ARG-S2_260 -9241.12848238
20 0
40 0
60 0
80 0
100 0
120 0
140 0
160 0
180 0
200 0
220 0
240 0
260 0
280 0
300 0
320 0
340 0
360 0
FHU-RIB:FHU-P:THR-S1 FHU-RIB:FHU-P:THR-S1_340 0.0
20 0
40 0
60 0
80 0
100 0
120 0
140 0
160 0
180 0
200 0
220 0
240 0
260 0
280 0
300 0
320 0
340 0
360 0
A-RIB:A-R6:HIS-CA A-RIB:A-R6:HIS-CA_20 0.0
20 0
40 0
60 1
80 1
100 2
120 2
140 1
160 1
180 0
200 0
220 0
240 1
260 2
280 2
300 2
320 1
340 1
360 0

H2U-P:H2U-RIB:PHE-S2 H2U-P:H2U-RIB:PHE-S2_340 0.0
20 0
40 0
60 0
80 0
100 0
120 0
140 0
160 0
180 0
200 0
220 0
240 0
260 0
280 0
300 0
320 0
340 0
360 0
U-RIB:U-Y:ASN-S2 U-RIB:U-Y:ASN-S2_320 -3688.56165041
20 0
40 1
60 3
80 3
100 3
120 3
140 2
160 2
180 0
200 0
220 1
240 3
260 3
280 3
300 3
320 2
340 2
360 0
G-P:G-RIB:LEU-S1 G-P:G-RIB:LEU-S1_60 -4141.03415557
20 0
40 1
60 3
80 6
100 7
120 7
140 5
160 3
180 1
200 0
220 1
240 3
260 5
280 6
300 6
320 5
340 3
360 1
U-RIB:U-P:PRO-CA U-RIB:U-P:PRO-CA_240 -5097.37034081

```
20   0
40   0
60   2
80   3
100   5
120   4
140   4
160   3
180   1
200   0
220   0
240   1
260   3
280   4
300   4
320   4
340   2
360   0
IU-P:IU-RIB:LYS-S1 IU-P:IU-RIB:LYS-S1_40 0.0
20   0
40   0
60   0
80   0
100   0
120   0
140   0
160   0
180   0
200   0
220   0
240   0
260   0
280   0
300   0
320   0
340   0
360   0
G-RIB:G-P:HIS-S1 G-RIB:G-P:HIS-S1_80 -5219.22790888
20   0
40   0
60   2
80   4
100   6
120   6
140   5
160   3
180   1
200   0
220   0
240   2
260   4
280   6
300   6
320   5
340   3
360   1
C-P:C-RIB:LYS-S1 C-P:C-RIB:LYS-S1_60 -5626.13941273
20   0
```

40   3
60   7
80   11
100   12
120   11
140   10
160   8
180   4
200   0
220   3
240   8
260   10
280   12
300   12
320   10
340   8
360   3
IU-RIB:IU-P:LEU-CA IU-RIB:IU-P:LEU-CA_300 -9042.80565256
20   0
40   0
60   0
80   0
100   0
120   0
140   0
160   0
180   0
200   0
220   0
240   0
260   0
280   0
300   0
320   0
340   0
360   0
QUO-RIB:QUO-M5:ASN-S2 QUO-RIB:QUO-M5:ASN-S2_20 0.0
20   0
40   0
60   0
80   0
100   0
120   0
140   0
160   0
180   0
200   0
220   0
240   0
260   0
280   0
300   0
320   0
340   0
360   0
C-RIB:C-P:HIS-CA C-RIB:C-P:HIS-CA_340 -1018.29106292
20   0
40   0

60   1
80   2
100   3
120   3
140   3
160   2
180   0
200   0
220   0
240   1
260   2
280   3
300   3
320   2
340   1
360   0
FHU-RIB:FHU-MY:PHE-S2 FHU-RIB:FHU-MY:PHE-S2_160 0.0
20   0
40   0
60   0
80   0
100   0
120   0
140   0
160   0
180   0
200   0
220   0
240   0
260   0
280   0
300   0
320   0
340   0
360   0
U-RIB:U-P:PRO-S1 U-RIB:U-P:PRO-S1_360 -3820.08599507
20   0
40   1
60   2
80   4
100   5
120   5
140   4
160   3
180   1
200   0
220   0
240   2
260   4
280   5
300   5
320   4
340   3
360   1
U-P:U-RIB:LEU-S2 U-P:U-RIB:LEU-S2_160 -1427.79041432
20   0
40   0
60   0

80   2
100   2
120   2
140   2
160   1
180   0
200   0
220   0
240   1
260   2
280   3
300   3
320   2
340   1
360   0
A-RIB:A-P:PHE-S2 A-RIB:A-P:PHE-S2_120 -2735.71060466
20   0
40   0
60   1
80   2
100   2
120   2
140   2
160   1
180   0
200   0
220   0
240   1
260   2
280   2
300   2
320   2
340   1
360   0
FMU-RIB:FMU-MY:MET-CA FMU-RIB:FMU-MY:MET-CA_160 0.0
20   0
40   0
60   0
80   0
100   0
120   0
140   0
160   0
180   0
200   0
220   0
240   0
260   0
280   0
300   0
320   0
340   0
360   0
A-RIB:A-R6:PHE-S2 A-RIB:A-R6:PHE-S2_140 -2566.01211478
20   0
40   0
60   0
80   1

100 1
120 1
140 1
160 0
180 0
200 0
220 0
240 1
260 1
280 1
300 1
320 0
340 0
360 0
G-RIB:G-R6:GLY-CA G-RIB:G-R6:GLY-CA_80 -3699.7493871
20 0
40 4
60 9
80 13
100 14
120 12
140 9
160 6
180 2
200 0
220 3
240 9
260 12
280 13
300 11
320 9
340 6
360 2
U-P:U-RIB:ASP-S1 U-P:U-RIB:ASP-S1_120 -2258.97490527
20 0
40 1
60 4
80 6
100 7
120 7
140 6
160 4
180 2
200 0
220 1
240 4
260 6
280 7
300 6
320 6
340 4
360 2
A-RIB:A-R5:TYR-S1 A-RIB:A-R5:TYR-S1_280 -4481.31830772
20 0
40 0
60 1
80 1
100 1

120   1
140   0
160   0
180   0
200   0
220   0
240   0
260   1
280   1
300   1
320   0
340   0
360   0
C31-P:C31-RIB:SER-S1 C31-P:C31-RIB:SER-S1_300 0.0
20   0
40   0
60   0
80   0
100   0
120   0
140   0
160   0
180   0
200   0
220   0
240   0
260   0
280   0
300   0
320   0
340   0
360   0
DA-RIB:DA-M5:ASP-S1 DA-RIB:DA-M5:ASP-S1_220 0.0
20   0
40   0
60   0
80   0
100   0
120   0
140   0
160   0
180   0
200   0
220   0
240   0
260   0
280   0
300   0
320   0
340   0
360   0
U34-P:U34-RIB:ASN-S1 U34-P:U34-RIB:ASN-S1_100 0.0
20   0
40   0
60   0
80   0
100   0
120   0

140   0
160   0
180   0
200   0
220   0
240   0
260   0
280   0
300   0
320   0
340   0
360   0
G-P:G-RIB:HIS-S2 G-P:G-RIB:HIS-S2_280 -4301.33259542
20   0
40   1
60   4
80   5
100   6
120   5
140   5
160   4
180   2
200   0
220   1
240   4
260   5
280   6
300   5
320   5
340   4
360   1
A-RIB:A-P:ARG-S2 A-RIB:A-P:ARG-S2_180 -4405.01770932
20   0
40   3
60   9
80   15
100   18
120   17
140   16
160   11
180   4
200   0
220   3
240   9
260   15
280   19
300   17
320   15
340   11
360   4
FHU-P:FHU-RIB:ARG-S1 FHU-P:FHU-RIB:ARG-S1_140 0.0
20   0
40   0
60   0
80   0
100   0
120   0
140   0

160 0
180 0
200 0
220 0
240 0
260 0
280 0
300 0
320 0
340 0
360 0
A-RIB:A-R6:ALA-S1 A-RIB:A-R6:ALA-S1_360 -2048.27196322
20 0
40 2
60 4
80 6
100 7
120 7
140 6
160 4
180 0
200 0
220 2
240 4
260 6
280 7
300 7
320 6
340 4
360 1
A-RIB:A-R5:CYS-S1 A-RIB:A-R5:CYS-S1_100 0.0
20 0
40 0
60 0
80 0
100 0
120 0
140 0
160 0
180 0
200 0
220 0
240 0
260 0
280 0
300 0
320 0
340 0
360 0
U-P:U-RIB:GLU-S1 U-P:U-RIB:GLU-S1_360 -106.312458293
20 0
40 2
60 6
80 8
100 9
120 9
140 8
160 6

180 0
200 0
220 0
240 6
260 8
280 9
300 9
320 8
340 6
360 2
FHU-P:FHU-RIB:LYS-S2 FHU-P:FHU-RIB:LYS-S2_220 -11410.2884529
20 0
40 0
60 0
80 0
100 0
120 0
140 0
160 0
180 0
200 0
220 0
240 0
260 0
280 0
300 0
320 0
340 0
360 0
C-P:C-RIB:TYR-CA C-P:C-RIB:TYR-CA_20 0.0
20 0
40 0
60 1
80 1
100 2
120 2
140 0
160 0
180 0
200 0
220 0
240 0
260 1
280 2
300 2
320 2
340 1
360 0
G-P:G-RIB:VAL-S1 G-P:G-RIB:VAL-S1_80 -2801.34921553
20 0
40 2
60 5
80 7
100 8
120 8
140 7
160 5
180 2

200   0
220   2
240   5
260   8
280   9
300   8
320   7
340   5
360   2
U31-RIB:U31-P:ASN-S1 U31-RIB:U31-P:ASN-S1_360 0.0
20   0
40   0
60   0
80   0
100   0
120   0
140   0
160   0
180   0
200   0
220   0
240   0
260   0
280   0
300   0
320   0
340   0
360   0
IU-RIB:IU-P:HIS-S1 IU-RIB:IU-P:HIS-S1_140 0.0
20   0
40   0
60   0
80   0
100   0
120   0
140   0
160   0
180   0
200   0
220   0
240   0
260   0
280   0
300   0
320   0
340   0
360   0
C31-RIB:C31-P:MET-S1 C31-RIB:C31-P:MET-S1_20 0.0
20   0
40   0
60   0
80   0
100   0
120   0
140   0
160   0
180   0
200   0

220 0
240 0
260 0
280 0
300 0
320 0
340 0
360 0
QUO-RIB:QUO-M6:PHE-S2 QUO-RIB:QUO-M6:PHE-S2_240 0.0
20 0
40 0
60 0
80 0
100 0
120 0
140 0
160 0
180 0
200 0
220 0
240 0
260 0
280 0
300 0
320 0
340 0
360 0
FHU-RIB:FHU-MY:SER-S1 FHU-RIB:FHU-MY:SER-S1_100 -10405.6624229
20 0
40 0
60 0
80 0
100 0
120 0
140 0
160 0
180 0
200 0
220 0
240 0
260 0
280 0
300 0
320 0
340 0
360 0
FMU-P:FMU-RIB:PHE-S2 FMU-P:FMU-RIB:PHE-S2_360 0.0
20 0
40 0
60 0
80 0
100 0
120 0
140 0
160 0
180 0
200 0
220 0

240   0
260   0
280   0
300   0
320   0
340   0
360   0
A-RIB:A-R5:HIS-S1 A-RIB:A-R5:HIS-S1_60 -3863.88877387
20   0
40   1
60   1
80   2
100   2
120   1
140   0
160   0
180   0
200   0
220   0
240   1
260   2
280   1
300   1
320   1
340   0
360   0
H2U-RIB:H2U-MY:ARG-CA H2U-RIB:H2U-MY:ARG-CA_220 0.0
20   0
40   0
60   0
80   0
100   0
120   0
140   0
160   0
180   0
200   0
220   0
240   0
260   0
280   0
300   0
320   0
340   0
360   0
C-RIB:C-P:SER-S1 C-RIB:C-P:SER-S1_40 -5319.16118959
20   0
40   2
60   5
80   8
100   9
120   10
140   8
160   5
180   2
200   0
220   2
240   5

260   7
280   9
300   8
320   7
340   5
360   2
C-RIB:C-Y:GLU-S2 C-RIB:C-Y:GLU-S2_120 -326.765601479
20   2
40   8
60   14
80   15
100   10
120   7
140   7
160   5
180   2
200   2
220   7
240   13
260   14
280   10
300   8
320   7
340   5
360   2
U-RIB:U-P:VAL-S1 U-RIB:U-P:VAL-S1_260 -3149.62262447
20   0
40   0
60   2
80   3
100   4
120   4
140   4
160   3
180   1
200   0
220   0
240   1
260   3
280   4
300   4
320   4
340   3
360   0
U34-RIB:U34-MY:ASP-S2 U34-RIB:U34-MY:ASP-S2_80 0.0
20   0
40   0
60   0
80   0
100   0
120   0
140   0
160   0
180   0
200   0
220   0
240   0
260   0

280   0
300   0
320   0
340   0
360   0
5BU-RIB:5BU-MY:ILE-S1 5BU-RIB:5BU-MY:ILE-S1_340 0.0
20   0
40   0
60   0
80   0
100   0
120   0
140   0
160   0
180   0
200   0
220   0
240   0
260   0
280   0
300   0
320   0
340   0
360   0
G-RIB:G-R6:HIS-S1 G-RIB:G-R6:HIS-S1_40 -7108.80444629
20   0
40   0
60   2
80   3
100   3
120   2
140   2
160   0
180   0
200   0
220   0
240   2
260   3
280   3
300   2
320   2
340   0
360   0
C-RIB:C-P:GLN-S1 C-RIB:C-P:GLN-S1_200 0.0
20   0
40   0
60   2
80   4
100   5
120   5
140   4
160   3
180   1
200   0
220   0
240   2
260   4
280   5

300   5
320   5
340   3
360   1
FMU-P:FMU-RIB:ALA-S1 FMU-P:FMU-RIB:ALA-S1_60 0.0
20   0
40   0
60   0
80   0
100   0
120   0
140   0
160   0
180   0
200   0
220   0
240   0
260   0
280   0
300   0
320   0
340   0
360   0
C-RIB:C-Y:ARG-CA C-RIB:C-Y:ARG-CA_140 -4479.42908716
20   0
40   2
60   3
80   3
100   4
120   3
140   2
160   1
180   0
200   0
220   2
240   3
260   4
280   4
300   3
320   3
340   1
360   0
A-RIB:A-R5:PRO-CA A-RIB:A-R5:PRO-CA_300 -5562.23196166
20   0
40   1
60   3
80   4
100   3
120   3
140   2
160   0
180   0
200   0
220   1
240   3
260   4
280   3
300   3

320   2
340   1
360   0
C-RIB:C-Y:CYS-S1 C-RIB:C-Y:CYS-S1_300 -7430.8366061
20   0
40   0
60   0
80   0
100   0
120   0
140   0
160   0
180   0
200   0
220   0
240   0
260   0
280   0
300   0
320   0
340   0
360   0
DA-RIB:DA-M5:HIS-CA DA-RIB:DA-M5:HIS-CA_20 0.0
20   0
40   0
60   0
80   0
100   0
120   0
140   0
160   0
180   0
200   0
220   0
240   0
260   0
280   0
300   0
320   0
340   0
360   0
C-RIB:C-Y:MET-S2 C-RIB:C-Y:MET-S2_120 -4538.03067851
20   0
40   0
60   1
80   1
100   1
120   0
140   0
160   0
180   0
200   0
220   0
240   1
260   1
280   1
300   1
320   0

340 0
360 0
C31-P:C31-RIB:TYR-S1 C31-P:C31-RIB:TYR-S1_260 0.0
20 0
40 0
60 0
80 0
100 0
120 0
140 0
160 0
180 0
200 0
220 0
240 0
260 0
280 0
300 0
320 0
340 0
360 0
IU-RIB:IU-MY:THR-S1 IU-RIB:IU-MY:THR-S1_320 0.0
20 0
40 0
60 0
80 0
100 0
120 0
140 0
160 0
180 0
200 0
220 0
240 0
260 0
280 0
300 0
320 0
340 0
360 0
QUO-P:QUO-RIB:ASP-S1 QUO-P:QUO-RIB:ASP-S1_20 0.0
20 0
40 0
60 0
80 0
100 0
120 0
140 0
160 0
180 0
200 0
220 0
240 0
260 0
280 0
300 0
320 0
340 0

```
360   0
G-P:G-RIB:GLY-CA G-P:G-RIB:GLY-CA_40 -3406.91627212
20   0
40   6
60   13
80   17
100   18
120   16
140   14
160   11
180   5
200   1
220   5
240   13
260   18
280   18
300   16
320   14
340   12
360   5
U-P:U-RIB:PRO-CA U-P:U-RIB:PRO-CA_200 0.0
20   0
40   0
60   2
80   3
100   4
120   3
140   3
160   2
180   1
200   0
220   0
240   2
260   3
280   4
300   4
320   3
340   0
360   1
C-RIB:C-Y:ASN-S2 C-RIB:C-Y:ASN-S2_360 0.0
20   0
40   3
60   5
80   5
100   3
120   3
140   2
160   2
180   0
200   0
220   3
240   5
260   5
280   4
300   3
320   2
340   1
360   0
```

U-P:U-RIB:ASN-S2 U-P:U-RIB:ASN-S2_300 -2768.06012877
20 0
40 1
60 3
80 4
100 4
120 4
140 3
160 3
180 1
200 0
220 1
240 3
260 4
280 4
300 4
320 4
340 3
360 1
I-P:I-RIB:ALA-CA I-P:I-RIB:ALA-CA_80 0.0
20 0
40 0
60 0
80 0
100 0
120 0
140 0
160 0
180 0
200 0
220 0
240 0
260 0
280 0
300 0
320 0
340 0
360 0
A-RIB:A-R5:ILE-S1 A-RIB:A-R5:ILE-S1_60 -4833.41084958
20 0
40 1
60 1
80 2
100 0
120 1
140 1
160 0
180 0
200 0
220 0
240 1
260 2
280 0
300 1
320 1
340 0
360 0
U-P:U-RIB:PHE-S1 U-P:U-RIB:PHE-S1_20 0.0

20   0
40   0
60   0
80   1
100   1
120   1
140   1
160   0
180   0
200   0
220   0
240   0
260   1
280   1
300   1
320   1
340   0
360   0
A-RIB:A-P:ILE-CA A-RIB:A-P:ILE-CA_100 -2670.55241131
20   0
40   0
60   1
80   3
100   4
120   4
140   3
160   2
180   0
200   0
220   0
240   1
260   3
280   4
300   4
320   3
340   2
360   0
U-RIB:U-Y:MET-CA U-RIB:U-Y:MET-CA_100 0.0
20   0
40   0
60   0
80   0
100   0
120   0
140   0
160   0
180   0
200   0
220   0
240   0
260   0
280   0
300   0
320   0
340   0
360   0
G-P:G-RIB:THR-CA G-P:G-RIB:THR-CA_60 -1037.04749356
20   0

```
40   1
60   5
80   7
100   8
120   8
140   6
160   4
180   2
200   0
220   1
240   4
260   7
280   8
300   8
320   7
340   5
360   2
U-P:U-RIB:PRO-S1 U-P:U-RIB:PRO-S1_40 0.0
20   0
40   0
60   3
80   4
100   4
120   4
140   3
160   3
180   1
200   0
220   1
240   3
260   4
280   4
300   4
320   4
340   3
360   1
H2U-P:H2U-RIB:TRP-S2 H2U-P:H2U-RIB:TRP-S2_160 0.0
20   0
40   0
60   0
80   0
100   0
120   0
140   0
160   0
180   0
200   0
220   0
240   0
260   0
280   0
300   0
320   0
340   0
360   0
C31-RIB:C31-MY:GLN-S2 C31-RIB:C31-MY:GLN-S2_60 0.0
20   0
40   0
```

60 0
80 0
100 0
120 0
140 0
160 0
180 0
200 0
220 0
240 0
260 0
280 0
300 0
320 0
340 0
360 0
G-RIB:G-R5:MET-S2 G-RIB:G-R5:MET-S2_320 -4635.31146861
20 0
40 1
60 2
80 2
100 0
120 1
140 1
160 0
180 0
200 0
220 1
240 1
260 2
280 1
300 0
320 1
340 0
360 0
FMU-P:FMU-RIB:GLN-S1 FMU-P:FMU-RIB:GLN-S1_160 0.0
20 0
40 0
60 0
80 0
100 0
120 0
140 0
160 0
180 0
200 0
220 0
240 0
260 0
280 0
300 0
320 0
340 0
360 0
G-P:G-RIB:ASP-CA G-P:G-RIB:ASP-CA_140 -2312.24833608
20 0
40 2
60 7

80   11
100   13
120   13
140   11
160   8
180   4
200   0
220   2
240   7
260   12
280   13
300   13
320   11
340   8
360   3
A-RIB:A-R5:THR-CA A-RIB:A-R5:THR-CA_20 -7739.29813062
20   0
40   1
60   2
80   3
100   4
120   3
140   2
160   1
180   0
200   0
220   1
240   2
260   3
280   4
300   3
320   2
340   1
360   0
FHU-P:FHU-RIB:LEU-S2 FHU-P:FHU-RIB:LEU-S2_100 -12294.0264755
20   0
40   0
60   0
80   0
100   0
120   0
140   0
160   0
180   0
200   0
220   0
240   0
260   0
280   0
300   0
320   0
340   0
360   0
G-RIB:G-P:MET-S2 G-RIB:G-P:MET-S2_220 -6035.99664791
20   0
40   0
60   2
80   3

```
100   4
120   4
140   3
160   2
180   0
200   0
220   0
240   1
260   3
280   4
300   3
320   3
340   0
360   0
A-RIB:A-R5:GLU-S1 A-RIB:A-R5:GLU-S1_140 -3023.69220865
20   0
40   4
60   8
80   10
100   9
120   8
140   7
160   4
180   0
200   1
220   4
240   8
260   10
280   9
300   7
320   6
340   4
360   1
FHU-RIB:FHU-MY:GLN-S2 FHU-RIB:FHU-MY:GLN-S2_140 0.0
20   0
40   0
60   0
80   0
100   0
120   0
140   0
160   0
180   0
200   0
220   0
240   0
260   0
280   0
300   0
320   0
340   0
360   0
U-RIB:U-Y:ARG-S2 U-RIB:U-Y:ARG-S2_80 -4310.49979614
20   1
40   3
60   6
80   7
100   6
```

120   5
140   4
160   3
180   1
200   1
220   3
240   6
260   7
280   6
300   5
320   4
340   3
360   1
U-P:U-RIB:CYS-CA U-P:U-RIB:CYS-CA_240 0.0
20   0
40   0
60   0
80   0
100   0
120   0
140   0
160   0
180   0
200   0
220   0
240   0
260   0
280   0
300   0
320   0
340   0
360   0
U31-RIB:U31-MY:ALA-CA U31-RIB:U31-MY:ALA-CA_340 0.0
20   0
40   0
60   0
80   0
100   0
120   0
140   0
160   0
180   0
200   0
220   0
240   0
260   0
280   0
300   0
320   0
340   0
360   0
C31-RIB:C31-P:MET-CA C31-RIB:C31-P:MET-CA_360 0.0
20   0
40   0
60   0
80   0
100   0
120   0

140   0
160   0
180   0
200   0
220   0
240   0
260   0
280   0
300   0
320   0
340   0
360   0
A-RIB:A-P:LYS-S2 A-RIB:A-P:LYS-S2_80 -4560.04342594
20   1
40   4
60   9
80   15
100   18
120   17
140   15
160   11
180   4
200   0
220   4
240   9
260   14
280   18
300   17
320   15
340   11
360   4
G-P:G-RIB:GLU-S2 G-P:G-RIB:GLU-S2_120 20.5620487636
20   2
40   9
60   20
80   27
100   26
120   24
140   21
160   18
180   8
200   0
220   9
240   19
260   26
280   26
300   24
320   21
340   18
360   8
QUO-RIB:QUO-M5:ARG-S2 QUO-RIB:QUO-M5:ARG-S2_40 0.0
20   0
40   0
60   0
80   0
100   0
120   0
140   0

160   0
180   0
200   0
220   0
240   0
260   0
280   0
300   0
320   0
340   0
360   0
C-RIB:C-P:ILE-CA C-RIB:C-P:ILE-CA_120 -4970.76502912
20   0
40   0
60   1
80   3
100   4
120   4
140   4
160   2
180   0
200   0
220   0
240   0
260   3
280   4
300   5
320   4
340   3
360   1
FHU-RIB:FHU-MY:VAL-S1 FHU-RIB:FHU-MY:VAL-S1_260 0.0
20   0
40   0
60   0
80   0
100   0
120   0
140   0
160   0
180   0
200   0
220   0
240   0
260   0
280   0
300   0
320   0
340   0
360   0
A-RIB:A-P:GLN-CA A-RIB:A-P:GLN-CA_220 -8168.61566735
20   0
40   0
60   1
80   3
100   5
120   5
140   4
160   3

180   0
200   0
220   0
240   1
260   3
280   5
300   5
320   4
340   3
360   1
A-RIB:A-R5:GLN-CA A-RIB:A-R5:GLN-CA_260 -5218.54917647
20   0
40   0
60   1
80   2
100   2
120   2
140   1
160   0
180   0
200   0
220   1
240   1
260   2
280   2
300   2
320   1
340   0
360   0
C-P:C-RIB:PHE-S2 C-P:C-RIB:PHE-S2_360 -4025.17193636
20   0
40   0
60   1
80   2
100   2
120   2
140   2
160   1
180   0
200   0
220   0
240   0
260   2
280   2
300   2
320   2
340   1
360   0
C-P:C-RIB:LEU-S2 C-P:C-RIB:LEU-S2_140 -4318.57710616
20   0
40   1
60   2
80   4
100   5
120   5
140   4
160   3
180   1

200   0
220   0
240   3
260   4
280   5
300   5
320   4
340   3
360   1
U31-RIB:U31-P:ALA-S1 U31-RIB:U31-P:ALA-S1_260 0.0
20   0
40   0
60   0
80   0
100   0
120   0
140   0
160   0
180   0
200   0
220   0
240   0
260   0
280   0
300   0
320   0
340   0
360   0
C-RIB:C-P:TYR-CA C-RIB:C-P:TYR-CA_260 -5376.15078665
20   0
40   0
60   0
80   1
100   2
120   2
140   2
160   1
180   0
200   0
220   0
240   0
260   1
280   2
300   2
320   2
340   1
360   0
A-P:A-RIB:TYR-CA A-P:A-RIB:TYR-CA_280 -4041.99857255
20   0
40   0
60   0
80   1
100   2
120   2
140   2
160   1
180   0
200   0

220   0
240   0
260   1
280   2
300   2
320   2
340   1
360   0
G-RIB:G-P:GLU-S1 G-RIB:G-P:GLU-S1_100 -416.926526204
20   0
40   3
60   10
80   19
100   27
120   27
140   23
160   17
180   6
200   0
220   3
240   10
260   19
280   27
300   27
320   23
340   16
360   6
FHU-RIB:FHU-P:ALA-CA FHU-RIB:FHU-P:ALA-CA_180 0.0
20   0
40   0
60   0
80   0
100   0
120   0
140   0
160   0
180   0
200   0
220   0
240   0
260   0
280   0
300   0
320   0
340   0
360   0
A-RIB:A-P:ILE-S1 A-RIB:A-P:ILE-S1_260 -4044.10995969
20   0
40   0
60   1
80   2
100   3
120   4
140   3
160   2
180   0
200   0
220   0

```
240   1
260   3
280   3
300   0
320   3
340   2
360   0
A-P:A-RIB:TRP-S1 A-P:A-RIB:TRP-S1_260 -4571.77689375
20   0
40   0
60   0
80   0
100   1
120   1
140   1
160   0
180   0
200   0
220   0
240   0
260   1
280   1
300   1
320   1
340   1
360   0
C31-RIB:C31-P:TYR-S1 C31-RIB:C31-P:TYR-S1_260 0.0
20   0
40   0
60   0
80   0
100   0
120   0
140   0
160   0
180   0
200   0
220   0
240   0
260   0
280   0
300   0
320   0
340   0
360   0
A-P:A-RIB:ALA-S1 A-P:A-RIB:ALA-S1_240 -3579.37788095
20   0
40   2
60   5
80   7
100   9
120   9
140   8
160   6
180   0
200   0
220   2
240   5
```

260 8
280 8
300 9
320 8
340 6
360 2
A-RIB:A-R5:PRO-S1 A-RIB:A-R5:PRO-S1_140 -4653.07292572
20 0
40 2
60 4
80 4
100 4
120 3
140 3
160 0
180 0
200 0
220 2
240 4
260 5
280 4
300 3
320 3
340 2
360 0
U31-RIB:U31-P:HIS-CA U31-RIB:U31-P:HIS-CA_40 0.0
20 0
40 0
60 0
80 0
100 0
120 0
140 0
160 0
180 0
200 0
220 0
240 0
260 0
280 0
300 0
320 0
340 0
360 0
DA-RIB:DA-M6:LYS-S2 DA-RIB:DA-M6:LYS-S2_60 0.0
20 0
40 0
60 0
80 0
100 0
120 0
140 0
160 0
180 0
200 0
220 0
240 0
260 0

```
280   0
300   0
320   0
340   0
360   0
C31-RIB:C31-P:ASN-CA C31-RIB:C31-P:ASN-CA_360 0.0
20   0
40   0
60   0
80   0
100   0
120   0
140   0
160   0
180   0
200   0
220   0
240   0
260   0
280   0
300   0
320   0
340   0
360   0
5BU-RIB:5BU-P:ARG-CA 5BU-RIB:5BU-P:ARG-CA_340 0.0
20   0
40   0
60   0
80   0
100   0
120   0
140   0
160   0
180   0
200   0
220   0
240   0
260   0
280   0
300   0
320   0
340   0
360   0
A-RIB:A-R5:PHE-CA A-RIB:A-R5:PHE-CA_360 0.0
20   0
40   0
60   1
80   0
100   1
120   1
140   0
160   0
180   0
200   0
220   0
240   1
260   1
280   1
```

```
300   1
320   0
340   0
360   0
C-RIB:C-Y:TRP-S1 C-RIB:C-Y:TRP-S1_40 -6804.47143909
20   0
40   0
60   0
80   0
100   0
120   0
140   0
160   0
180   0
200   0
220   0
240   0
260   0
280   0
300   0
320   0
340   0
360   0
IU-RIB:IU-MY:LEU-S2 IU-RIB:IU-MY:LEU-S2_100 0.0
20   0
40   0
60   0
80   0
100   0
120   0
140   0
160   0
180   0
200   0
220   0
240   0
260   0
280   0
300   0
320   0
340   0
360   0
A-RIB:A-R6:ASP-S2 A-RIB:A-R6:ASP-S2_320 -865.224516943
20   0
40   2
60   7
80   10
100   10
120   10
140   8
160   6
180   2
200   0
220   2
240   8
260   10
280   10
300   10
```

320   8
340   5
360   2
U31-RIB:U31-MY:TYR-CA U31-RIB:U31-MY:TYR-CA_120 0.0
20   0
40   0
60   0
80   0
100   0
120   0
140   0
160   0
180   0
200   0
220   0
240   0
260   0
280   0
300   0
320   0
340   0
360   0
C-RIB:C-Y:ALA-CA C-RIB:C-Y:ALA-CA_120 -2880.6323878
20   0
40   2
60   4
80   4
100   3
120   3
140   3
160   2
180   0
200   0
220   2
240   4
260   4
280   4
300   3
320   3
340   2
360   0
C-RIB:C-P:ASP-CA C-RIB:C-P:ASP-CA_360 0.0
20   0
40   0
60   4
80   8
100   11
120   12
140   9
160   7
180   0
200   0
220   1
240   4
260   8
280   11
300   11
320   9

340   6
360   0
A-P:A-RIB:CYS-S1 A-P:A-RIB:CYS-S1_240 -6660.70503608
20   0
40   0
60   0
80   0
100   0
120   0
140   0
160   0
180   0
200   0
220   0
240   0
260   0
280   0
300   0
320   0
340   0
360   0
A-P:A-RIB:ASP-S1 A-P:A-RIB:ASP-S1_140 -1614.53067328
20   0
40   0
60   0
80   10
100   10
120   10
140   10
160   8
180   0
200   0
220   0
240   7
260   10
280   11
300   11
320   10
340   8
360   3
G-RIB:G-R6:ALA-CA G-RIB:G-R6:ALA-CA_60 -1135.95789621
20   0
40   2
60   5
80   7
100   8
120   8
140   6
160   4
180   1
200   0
220   1
240   5
260   7
280   8
300   7
320   6
340   4

```
360   0
FMU-RIB:FMU-MY:ALA-CA FMU-RIB:FMU-MY:ALA-CA_120 0.0
20   0
40   0
60   0
80   0
100   0
120   0
140   0
160   0
180   0
200   0
220   0
240   0
260   0
280   0
300   0
320   0
340   0
360   0
C-P:C-RIB:PRO-CA C-P:C-RIB:PRO-CA_240 -3376.07634533
20   0
40   1
60   3
80   5
100   6
120   6
140   5
160   4
180   2
200   0
220   1
240   3
260   5
280   6
300   6
320   5
340   4
360   2
G-RIB:G-P:ILE-CA G-RIB:G-P:ILE-CA_100 -1978.47262483
20   0
40   0
60   2
80   4
100   7
120   8
140   6
160   4
180   0
200   0
220   0
240   2
260   5
280   7
300   7
320   6
340   4
360   0
```

FHU-RIB:FHU-P:ALA-S1 FHU-RIB:FHU-P:ALA-S1_300 0.0
20 0
40 0
60 0
80 0
100 0
120 0
140 0
160 0
180 0
200 0
220 0
240 0
260 0
280 0
300 0
320 0
340 0
360 0
FMU-RIB:FMU-P:ARG-S1 FMU-RIB:FMU-P:ARG-S1_100 -13680.5935958
20 0
40 0
60 0
80 0
100 0
120 0
140 0
160 0
180 0
200 0
220 0
240 0
260 0
280 0
300 0
320 0
340 0
360 0
A-RIB:A-R6:GLY-CA A-RIB:A-R6:GLY-CA_160 -3446.84372931
20 0
40 2
60 6
80 9
100 10
120 10
140 8
160 5
180 0
200 0
220 2
240 6
260 10
280 11
300 10
320 8
340 6
360 2
U-RIB:U-P:ARG-S1 U-RIB:U-P:ARG-S1_40 -2651.21406803

20 0
40 1
60 3
80 6
100 8
120 8
140 8
160 5
180 2
200 0
220 1
240 3
260 6
280 8
300 9
320 8
340 5
360 2
A-P:A-RIB:GLN-S1 A-P:A-RIB:GLN-S1_140 -4626.13474458
20 0
40 1
60 3
80 4
100 5
120 5
140 4
160 3
180 1
200 0
220 0
240 3
260 5
280 5
300 5
320 4
340 4
360 1
G-RIB:G-P:CYS-S1 G-RIB:G-P:CYS-S1_320 -3415.0962487
20 0
40 0
60 0
80 0
100 0
120 1
140 1
160 0
180 0
200 0
220 0
240 0
260 0
280 0
300 0
320 1
340 0
360 0
H2U-RIB:H2U-P:GLU-S2 H2U-RIB:H2U-P:GLU-S2_120 0.0
20 0

40   0
60   0
80   0
100   0
120   0
140   0
160   0
180   0
200   0
220   0
240   0
260   0
280   0
300   0
320   0
340   0
360   0
C-P:C-RIB:SER-S1 C-P:C-RIB:SER-S1_120 -3628.21143683
20   0
40   2
60   5
80   7
100   8
120   9
140   8
160   6
180   3
200   0
220   2
240   6
260   8
280   8
300   8
320   8
340   6
360   3
QUO-RIB:QUO-M6:ASN-S2 QUO-RIB:QUO-M6:ASN-S2_320 0.0
20   0
40   0
60   0
80   0
100   0
120   0
140   0
160   0
180   0
200   0
220   0
240   0
260   0
280   0
300   0
320   0
340   0
360   0
U34-RIB:U34-P:PHE-S2 U34-RIB:U34-P:PHE-S2_240 0.0
20   0
40   0

60   0
80   0
100   0
120   0
140   0
160   0
180   0
200   0
220   0
240   0
260   0
280   0
300   0
320   0
340   0
360   0
U-RIB:U-Y:TRP-CA U-RIB:U-Y:TRP-CA_140 0.0
20   0
40   0
60   0
80   0
100   0
120   0
140   0
160   0
180   0
200   0
220   0
240   0
260   0
280   0
300   0
320   0
340   0
360   0
IU-P:IU-RIB:HIS-S1 IU-P:IU-RIB:HIS-S1_300 0.0
20   0
40   0
60   0
80   0
100   0
120   0
140   0
160   0
180   0
200   0
220   0
240   0
260   0
280   0
300   0
320   0
340   0
360   0
A-RIB:A-P:VAL-S1 A-RIB:A-P:VAL-S1_20 0.0
20   0
40   1
60   2

80   5
100   6
120   7
140   6
160   4
180   1
200   0
220   0
240   2
260   4
280   6
300   7
320   6
340   4
360   1
A-P:A-RIB:LEU-S2 A-P:A-RIB:LEU-S2_120 -2813.53103344
20   0
40   1
60   2
80   4
100   5
120   5
140   4
160   3
180   1
200   0
220   0
240   2
260   4
280   5
300   5
320   4
340   3
360   1
U31-RIB:U31-MY:MET-S2 U31-RIB:U31-MY:MET-S2_140 0.0
20   0
40   0
60   0
80   0
100   0
120   0
140   0
160   0
180   0
200   0
220   0
240   0
260   0
280   0
300   0
320   0
340   0
360   0
U34-RIB:U34-MY:SER-S1 U34-RIB:U34-MY:SER-S1_320 0.0
20   0
40   0
60   0
80   0

100   0
120   0
140   0
160   0
180   0
200   0
220   0
240   0
260   0
280   0
300   0
320   0
340   0
360   0
G-RIB:G-P:TYR-S2 G-RIB:G-P:TYR-S2_300 -2052.99591633
20   0
40   0
60   1
80   3
100   5
120   5
140   4
160   3
180   1
200   0
220   0
240   2
260   3
280   4
300   5
320   4
340   3
360   0
U-RIB:U-Y:GLU-S1 U-RIB:U-Y:GLU-S1_300 -165.199343826
20   0
40   2
60   5
80   6
100   6
120   0
140   4
160   3
180   1
200   0
220   2
240   5
260   6
280   5
300   5
320   0
340   3
360   0
PSU-P:PSU-RIB:ARG-S2 PSU-P:PSU-RIB:ARG-S2_120 -11410.2884529
20   0
40   0
60   0
80   0
100   0

```
120   0
140   0
160   0
180   0
200   0
220   0
240   0
260   0
280   0
300   0
320   0
340   0
360   0
A-P:A-RIB:ASN-CA A-P:A-RIB:ASN-CA_340 -2622.48664296
20   0
40   0
60   2
80   4
100   5
120   5
140   4
160   4
180   1
200   0
220   0
240   3
260   5
280   5
300   5
320   4
340   4
360   1
U-RIB:U-P:GLY-CA U-RIB:U-P:GLY-CA_160 -4572.98347408
20   0
40   1
60   4
80   7
100   9
120   10
140   8
160   6
180   2
200   0
220   1
240   4
260   7
280   9
300   9
320   8
340   6
360   2
5BU-RIB:5BU-P:SER-S1 5BU-RIB:5BU-P:SER-S1_300 0.0
20   0
40   0
60   0
80   0
100   0
120   0
```

140   0
160   0
180   0
200   0
220   0
240   0
260   0
280   0
300   0
320   0
340   0
360   0
G-RIB:G-R6:MET-CA G-RIB:G-R6:MET-CA_340 -4290.42097754
20   0
40   0
60   0
80   1
100   1
120   1
140   0
160   0
180   0
200   0
220   0
240   1
260   1
280   1
300   1
320   1
340   0
360   0
5BU-RIB:5BU-P:ILE-S1 5BU-RIB:5BU-P:ILE-S1_360 0.0
20   0
40   0
60   0
80   0
100   0
120   0
140   0
160   0
180   0
200   0
220   0
240   0
260   0
280   0
300   0
320   0
340   0
360   0
I-P:I-RIB:ALA-S1 I-P:I-RIB:ALA-S1_260 0.0
20   0
40   0
60   0
80   0
100   0
120   0
140   0

160 0
180 0
200 0
220 0
240 0
260 0
280 0
300 0
320 0
340 0
360 0
C31-RIB:C31-MY:TYR-S2 C31-RIB:C31-MY:TYR-S2_180 0.0
20 0
40 0
60 0
80 0
100 0
120 0
140 0
160 0
180 0
200 0
220 0
240 0
260 0
280 0
300 0
320 0
340 0
360 0
G-RIB:G-R6:TRP-CA G-RIB:G-R6:TRP-CA_340 0.0
20 0
40 0
60 0
80 0
100 0
120 0
140 0
160 0
180 0
200 0
220 0
240 0
260 1
280 1
300 0
320 0
340 0
360 0
U-RIB:U-Y:LEU-S1 U-RIB:U-Y:LEU-S1_200 0.0
20 0
40 0
60 0
80 1
100 1
120 1
140 1
160 0

```
180   0
200   0
220   0
240   1
260   1
280   1
300   1
320   1
340   0
360   0
U31-RIB:U31-MY:GLN-CA U31-RIB:U31-MY:GLN-CA_280 0.0
20   0
40   0
60   0
80   0
100   0
120   0
140   0
160   0
180   0
200   0
220   0
240   0
260   0
280   0
300   0
320   0
340   0
360   0
DA-RIB:DA-M5:HIS-S2 DA-RIB:DA-M5:HIS-S2_80 0.0
20   0
40   0
60   0
80   0
100   0
120   0
140   0
160   0
180   0
200   0
220   0
240   0
260   0
280   0
300   0
320   0
340   0
360   0
G-RIB:G-R6:ASN-S1 G-RIB:G-R6:ASN-S1_60 -4091.2172876
20   0
40   1
60   4
80   6
100   6
120   5
140   3
160   2
180   1
```

200   0
220   1
240   4
260   6
280   6
300   5
320   3
340   2
360   0
C31-RIB:C31-P:TYR-S2 C31-RIB:C31-P:TYR-S2_300 0.0
20   0
40   0
60   0
80   0
100   0
120   0
140   0
160   0
180   0
200   0
220   0
240   0
260   0
280   0
300   0
320   0
340   0
360   0
G-RIB:G-R5:PHE-S2 G-RIB:G-R5:PHE-S2_240 -4074.3943769
20   0
40   0
60   1
80   1
100   1
120   0
140   1
160   0
180   0
200   0
220   0
240   1
260   1
280   1
300   1
320   0
340   0
360   0
C-RIB:C-P:PHE-CA C-RIB:C-P:PHE-CA_140 -12.4196383149
20   0
40   0
60   1
80   1
100   3
120   3
140   2
160   1
180   0
200   0

220	0
240	1
260	2
280	3
300	3
320	0
340	1
360	0
QUO-RIB:QUO-P:PHE-S2 QUO-RIB:QUO-P:PHE-S2_120 0.0
20	0
40	0
60	0
80	0
100	0
120	0
140	0
160	0
180	0
200	0
220	0
240	0
260	0
280	0
300	0
320	0
340	0
360	0
U-RIB:U-Y:LEU-CA U-RIB:U-Y:LEU-CA_120 -4901.13626749
20	0
40	0
60	0
80	1
100	1
120	2
140	1
160	0
180	0
200	0
220	0
240	0
260	1
280	2
300	2
320	1
340	1
360	0
QUO-RIB:QUO-M6:LYS-CA QUO-RIB:QUO-M6:LYS-CA_180 0.0
20	0
40	0
60	0
80	0
100	0
120	0
140	0
160	0
180	0
200	0
220	0

240 0
260 0
280 0
300 0
320 0
340 0
360 0
FMU-RIB:FMU-MY:ASP-S2 FMU-RIB:FMU-MY:ASP-S2_260 0.0
20 0
40 0
60 0
80 0
100 0
120 0
140 0
160 0
180 0
200 0
220 0
240 0
260 0
280 0
300 0
320 0
340 0
360 0
FHU-RIB:FHU-P:TYR-CA FHU-RIB:FHU-P:TYR-CA_280 0.0
20 0
40 0
60 0
80 0
100 0
120 0
140 0
160 0
180 0
200 0
220 0
240 0
260 0
280 0
300 0
320 0
340 0
360 0
A-P:A-RIB:GLU-S1 A-P:A-RIB:GLU-S1_120 -1709.64465611
20 0
40 3
60 9
80 13
100 14
120 14
140 13
160 10
180 4
200 0
220 0
240 9

260   13
280   15
300   14
320   12
340   10
360   4
A-RIB:A-R5:LYS-S1 A-RIB:A-R5:LYS-S1_60 -3392.7239734
20   1
40   4
60   7
80   7
100   7
120   7
140   5
160   3
180   0
200   1
220   4
240   7
260   8
280   8
300   7
320   5
340   4
360   1
C-RIB:C-P:SER-CA C-RIB:C-P:SER-CA_300 -2860.46331834
20   0
40   1
60   4
80   6
100   9
120   9
140   7
160   5
180   2
200   0
220   1
240   4
260   7
280   8
300   8
320   7
340   4
360   1
FMU-RIB:FMU-P:ALA-S1 FMU-RIB:FMU-P:ALA-S1_40 0.0
20   0
40   0
60   0
80   0
100   0
120   0
140   0
160   0
180   0
200   0
220   0
240   0
260   0

280   0
300   0
320   0
340   0
360   0
U31-RIB:U31-P:TYR-S1 U31-RIB:U31-P:TYR-S1_360 0.0
20   0
40   0
60   0
80   0
100   0
120   0
140   0
160   0
180   0
200   0
220   0
240   0
260   0
280   0
300   0
320   0
340   0
360   0
A-RIB:A-P:TYR-S2 A-RIB:A-P:TYR-S2_80 -5664.18936579
20   0
40   0
60   1
80   2
100   3
120   3
140   2
160   1
180   0
200   0
220   0
240   1
260   2
280   2
300   3
320   2
340   1
360   0
U31-RIB:U31-P:ASN-CA U31-RIB:U31-P:ASN-CA_40 0.0
20   0
40   0
60   0
80   0
100   0
120   0
140   0
160   0
180   0
200   0
220   0
240   0
260   0
280   0

300   0
320   0
340   0
360   0
A-RIB:A-R6:ASN-CA A-RIB:A-R6:ASN-CA_340 -3187.45103339
20   0
40   0
60   2
80   4
100   4
120   3
140   3
160   2
180   0
200   0
220   0
240   2
260   4
280   4
300   4
320   3
340   2
360   0
A-P:A-RIB:ASP-S2 A-P:A-RIB:ASP-S2_120 -1738.15365986
20   0
40   0
60   8
80   11
100   12
120   11
140   11
160   9
180   4
200   0
220   4
240   9
260   12
280   12
300   12
320   11
340   10
360   0
U34-RIB:U34-MY:ASN-S1 U34-RIB:U34-MY:ASN-S1_40 0.0
20   0
40   0
60   0
80   0
100   0
120   0
140   0
160   0
180   0
200   0
220   0
240   0
260   0
280   0
300   0

320   0
340   0
360   0
U-P:U-RIB:TYR-CA U-P:U-RIB:TYR-CA_20 0.0
20   0
40   0
60   0
80   1
100   1
120   1
140   0
160   1
180   0
200   0
220   0
240   0
260   0
280   1
300   1
320   1
340   1
360   0
A-RIB:A-R6:SER-CA A-RIB:A-R6:SER-CA_120 -3463.08016372
20   0
40   1
60   3
80   5
100   5
120   5
140   4
160   3
180   0
200   0
220   1
240   3
260   5
280   6
300   5
320   4
340   3
360   1
IU-RIB:IU-MY:ARG-S1 IU-RIB:IU-MY:ARG-S1_160 0.0
20   0
40   0
60   0
80   0
100   0
120   0
140   0
160   0
180   0
200   0
220   0
240   0
260   0
280   0
300   0
320   0

340   0
360   0
FMU-RIB:FMU-MY:HIS-S1 FMU-RIB:FMU-MY:HIS-S1_60 0.0
20   0
40   0
60   0
80   0
100   0
120   0
140   0
160   0
180   0
200   0
220   0
240   0
260   0
280   0
300   0
320   0
340   0
360   0
DA-RIB:DA-M5:LYS-S2 DA-RIB:DA-M5:LYS-S2_20 0.0
20   0
40   0
60   0
80   0
100   0
120   0
140   0
160   0
180   0
200   0
220   0
240   0
260   0
280   0
300   0
320   0
340   0
360   0
FMU-RIB:FMU-MY:SER-CA FMU-RIB:FMU-MY:SER-CA_40 0.0
20   0
40   0
60   0
80   0
100   0
120   0
140   0
160   0
180   0
200   0
220   0
240   0
260   0
280   0
300   0
320   0
340   0

```
360   0
QUO-P:QUO-RIB:LEU-S1 QUO-P:QUO-RIB:LEU-S1_80 0.0
20   0
40   0
60   0
80   0
100   0
120   0
140   0
160   0
180   0
200   0
220   0
240   0
260   0
280   0
300   0
320   0
340   0
360   0
IU-P:IU-RIB:GLN-S2 IU-P:IU-RIB:GLN-S2_340 0.0
20   0
40   0
60   0
80   0
100   0
120   0
140   0
160   0
180   0
200   0
220   0
240   0
260   0
280   0
300   0
320   0
340   0
360   0
QUO-P:QUO-RIB:LYS-S2 QUO-P:QUO-RIB:LYS-S2_240 0.0
20   0
40   0
60   0
80   0
100   0
120   0
140   0
160   0
180   0
200   0
220   0
240   0
260   0
280   0
300   0
320   0
340   0
360   0
```

```
IU-RIB:IU-P:SER-S1 IU-RIB:IU-P:SER-S1_340 0.0
20   0
40   0
60   0
80   0
100   0
120   0
140   0
160   0
180   0
200   0
220   0
240   0
260   0
280   0
300   0
320   0
340   0
360   0
U-RIB:U-Y:ALA-CA U-RIB:U-Y:ALA-CA_280 -2724.52452401
20   0
40   1
60   2
80   2
100   3
120   3
140   2
160   1
180   0
200   0
220   1
240   2
260   2
280   2
300   3
320   2
340   1
360   0
FMU-RIB:FMU-MY:PRO-CA FMU-RIB:FMU-MY:PRO-CA_280 0.0
20   0
40   0
60   0
80   0
100   0
120   0
140   0
160   0
180   0
200   0
220   0
240   0
260   0
280   0
300   0
320   0
340   0
360   0
FMU-P:FMU-RIB:MET-S2 FMU-P:FMU-RIB:MET-S2_200 0.0
```

20 0
40 0
60 0
80 0
100 0
120 0
140 0
160 0
180 0
200 0
220 0
240 0
260 0
280 0
300 0
320 0
340 0
360 0
U31-P:U31-RIB:GLN-S1 U31-P:U31-RIB:GLN-S1_180 0.0
20 0
40 0
60 0
80 0
100 0
120 0
140 0
160 0
180 0
200 0
220 0
240 0
260 0
280 0
300 0
320 0
340 0
360 0
U31-P:U31-RIB:GLU-CA U31-P:U31-RIB:GLU-CA_240 0.0
20 0
40 0
60 0
80 0
100 0
120 0
140 0
160 0
180 0
200 0
220 0
240 0
260 0
280 0
300 0
320 0
340 0
360 0
A-RIB:A-R5:SER-S1 A-RIB:A-R5:SER-S1_300 -3253.80607157
20 1

40   3
60   5
80   6
100   6
120   5
140   4
160   2
180   0
200   1
220   3
240   5
260   6
280   5
300   5
320   3
340   2
360   0
A-RIB:A-P:TYR-CA A-RIB:A-P:TYR-CA_80 -4836.31725772
20   0
40   0
60   0
80   1
100   2
120   2
140   2
160   1
180   0
200   0
220   0
240   0
260   1
280   2
300   2
320   2
340   1
360   0
QUO-RIB:QUO-M6:GLN-S1 QUO-RIB:QUO-M6:GLN-S1_300 0.0
20   0
40   0
60   0
80   0
100   0
120   0
140   0
160   0
180   0
200   0
220   0
240   0
260   0
280   0
300   0
320   0
340   0
360   0
H2U-RIB:H2U-P:PHE-S1 H2U-RIB:H2U-P:PHE-S1_60 0.0
20   0
40   0

```
60   0
80   0
100   0
120   0
140   0
160   0
180   0
200   0
220   0
240   0
260   0
280   0
300   0
320   0
340   0
360   0
G-RIB:G-P:GLU-S2 G-RIB:G-P:GLU-S2_40 -414.362553975
20   0
40   6
60   15
80   25
100   31
120   31
140   26
160   20
180   8
200   0
220   6
240   15
260   26
280   32
300   30
320   27
340   20
360   8
U-RIB:U-Y:PRO-CA U-RIB:U-Y:PRO-CA_320 -2954.35932908
20   0
40   1
60   2
80   2
100   2
120   2
140   2
160   1
180   0
200   0
220   0
240   2
260   2
280   2
300   2
320   1
340   1
360   0
FHU-RIB:FHU-MY:ASP-S2 FHU-RIB:FHU-MY:ASP-S2_300 -8378.12457782
20   0
40   0
60   0
```

80 0
100 0
120 0
140 0
160 0
180 0
200 0
220 0
240 0
260 0
280 0
300 0
320 0
340 0
360 0
H2U-RIB:H2U-P:ASN-S2 H2U-RIB:H2U-P:ASN-S2_140 -10576.6069338
20 0
40 0
60 0
80 0
100 0
120 0
140 0
160 0
180 0
200 0
220 0
240 0
260 0
280 0
300 0
320 0
340 0
360 0
GTP-RIB:GTP-M5:GLY-CA GTP-RIB:GTP-M5:GLY-CA_100 0.0
20 0
40 0
60 0
80 0
100 0
120 0
140 0
160 0
180 0
200 0
220 0
240 0
260 0
280 0
300 0
320 0
340 0
360 0
G-RIB:G-R6:PHE-CA G-RIB:G-R6:PHE-CA_120 -1885.26884588
20 0
40 0
60 0
80 1

100   0
120   1
140   1
160   0
180   0
200   0
220   0
240   0
260   1
280   1
300   1
320   0
340   0
360   0
C-P:C-RIB:GLU-S2 C-P:C-RIB:GLU-S2_120 -1016.63067575
20   0
40   0
60   13
80   17
100   18
120   17
140   16
160   14
180   7
200   0
220   0
240   13
260   17
280   18
300   17
320   16
340   13
360   6
C31-RIB:C31-P:PHE-CA C31-RIB:C31-P:PHE-CA_40 0.0
20   0
40   0
60   0
80   0
100   0
120   0
140   0
160   0
180   0
200   0
220   0
240   0
260   0
280   0
300   0
320   0
340   0
360   0
FHU-P:FHU-RIB:LEU-CA FHU-P:FHU-RIB:LEU-CA_140 0.0
20   0
40   0
60   0
80   0
100   0

120   0
140   0
160   0
180   0
200   0
220   0
240   0
260   0
280   0
300   0
320   0
340   0
360   0
C-RIB:C-Y:PHE-CA C-RIB:C-Y:PHE-CA_140 -3309.23611258
20   0
40   0
60   0
80   0
100   1
120   1
140   0
160   0
180   0
200   0
220   0
240   0
260   1
280   1
300   1
320   0
340   0
360   0
U-RIB:U-P:GLN-CA U-RIB:U-P:GLN-CA_60 -5590.16256919
20   0
40   0
60   1
80   2
100   3
120   4
140   3
160   2
180   0
200   0
220   0
240   1
260   0
280   3
300   3
320   3
340   2
360   0
FMU-RIB:FMU-P:PHE-S2 FMU-RIB:FMU-P:PHE-S2_320 0.0
20   0
40   0
60   0
80   0
100   0
120   0

140   0
160   0
180   0
200   0
220   0
240   0
260   0
280   0
300   0
320   0
340   0
360   0
DA-RIB:DA-M5:LYS-S1 DA-RIB:DA-M5:LYS-S1_320 0.0
20   0
40   0
60   0
80   0
100   0
120   0
140   0
160   0
180   0
200   0
220   0
240   0
260   0
280   0
300   0
320   0
340   0
360   0
G-P:G-RIB:GLN-S2 G-P:G-RIB:GLN-S2_80 -1825.80193554
20   0
40   3
60   7
80   9
100   8
120   8
140   7
160   6
180   2
200   0
220   3
240   7
260   9
280   9
300   8
320   7
340   6
360   2
C31-P:C31-RIB:LEU-CA C31-P:C31-RIB:LEU-CA_360 0.0
20   0
40   0
60   0
80   0
100   0
120   0
140   0

160 0
180 0
200 0
220 0
240 0
260 0
280 0
300 0
320 0
340 0
360 0
G-P:G-RIB:CYS-S1 G-P:G-RIB:CYS-S1_120 -4037.78118829
20 0
40 0
60 0
80 0
100 0
120 0
140 0
160 0
180 0
200 0
220 0
240 0
260 0
280 0
300 0
320 0
340 0
360 0
G-RIB:G-R6:GLN-S2 G-RIB:G-R6:GLN-S2_280 -4098.20753005
20 0
40 2
60 5
80 7
100 6
120 6
140 4
160 0
180 1
200 0
220 2
240 5
260 7
280 6
300 6
320 4
340 2
360 0
A-RIB:A-P:HIS-CA A-RIB:A-P:HIS-CA_20 0.0
20 0
40 0
60 1
80 2
100 3
120 4
140 3
160 2

180   0
200   0
220   0
240   1
260   2
280   3
300   3
320   3
340   2
360   0
A-RIB:A-R5:ASN-CA A-RIB:A-R5:ASN-CA_180 0.0
20   0
40   1
60   2
80   3
100   3
120   2
140   2
160   0
180   0
200   0
220   1
240   2
260   3
280   3
300   2
320   2
340   1
360   0
G-RIB:G-R5:ASN-S1 G-RIB:G-R5:ASN-S1_40 -5134.15503143
20   0
40   3
60   5
80   6
100   4
120   4
140   3
160   2
180   0
200   1
220   3
240   5
260   6
280   4
300   4
320   3
340   2
360   1
IU-RIB:IU-MY:LEU-CA IU-RIB:IU-MY:LEU-CA_160 0.0
20   0
40   0
60   0
80   0
100   0
120   0
140   0
160   0
180   0

200   0
220   0
240   0
260   0
280   0
300   0
320   0
340   0
360   0
C-RIB:C-P:VAL-S1 C-RIB:C-P:VAL-S1_340 -1755.70541677
20   0
40   1
60   3
80   5
100   6
120   7
140   6
160   4
180   1
200   0
220   1
240   2
260   5
280   6
300   7
320   6
340   4
360   1
U-RIB:U-P:PHE-S1 U-RIB:U-P:PHE-S1_20 0.0
20   0
40   0
60   0
80   1
100   1
120   1
140   1
160   1
180   0
200   0
220   0
240   0
260   1
280   1
300   2
320   1
340   0
360   0
G-RIB:G-R5:HIS-S1 G-RIB:G-R5:HIS-S1_40 -6909.64214072
20   0
40   1
60   2
80   3
100   2
120   2
140   2
160   0
180   0
200   0

220   1
240   2
260   2
280   2
300   2
320   2
340   0
360   0
C31-RIB:C31-MY:PHE-S2 C31-RIB:C31-MY:PHE-S2_60 0.0
20   0
40   0
60   0
80   0
100   0
120   0
140   0
160   0
180   0
200   0
220   0
240   0
260   0
280   0
300   0
320   0
340   0
360   0
C-RIB:C-P:ASN-S1 C-RIB:C-P:ASN-S1_320 -4292.96744503
20   0
40   1
60   3
80   5
100   7
120   7
140   6
160   4
180   1
200   0
220   1
240   3
260   5
280   7
300   7
320   5
340   4
360   1
QUO-RIB:QUO-M5:ARG-S1 QUO-RIB:QUO-M5:ARG-S1_300 0.0
20   0
40   0
60   0
80   0
100   0
120   0
140   0
160   0
180   0
200   0
220   0

```
240   0
260   0
280   0
300   0
320   0
340   0
360   0
C-RIB:C-P:ASP-S2 C-RIB:C-P:ASP-S2_120 -2286.87853354
20   0
40   3
60   7
80   12
100   14
120   14
140   12
160   9
180   3
200   0
220   2
240   7
260   12
280   14
300   13
320   11
340   8
360   3
QUO-RIB:QUO-M6:PHE-S1 QUO-RIB:QUO-M6:PHE-S1_320 -3434.83908349
20   0
40   0
60   0
80   0
100   0
120   0
140   0
160   0
180   0
200   0
220   0
240   0
260   0
280   0
300   0
320   0
340   0
360   0
U31-RIB:U31-MY:TYR-S1 U31-RIB:U31-MY:TYR-S1_120 0.0
20   0
40   0
60   0
80   0
100   0
120   0
140   0
160   0
180   0
200   0
220   0
240   0
```

```
260   0
280   0
300   0
320   0
340   0
360   0
QUO-RIB:QUO-M6:ASN-CA QUO-RIB:QUO-M6:ASN-CA_280 0.0
20   0
40   0
60   0
80   0
100   0
120   0
140   0
160   0
180   0
200   0
220   0
240   0
260   0
280   0
300   0
320   0
340   0
360   0
DA-RIB:DA-M6:MET-S2 DA-RIB:DA-M6:MET-S2_120 0.0
20   0
40   0
60   0
80   0
100   0
120   0
140   0
160   0
180   0
200   0
220   0
240   0
260   0
280   0
300   0
320   0
340   0
360   0
QUO-RIB:QUO-M5:PHE-S1 QUO-RIB:QUO-M5:PHE-S1_160 0.0
20   0
40   0
60   0
80   0
100   0
120   0
140   0
160   0
180   0
200   0
220   0
240   0
260   0
```

280   0
300   0
320   0
340   0
360   0
FHU-P:FHU-RIB:LEU-S1 FHU-P:FHU-RIB:LEU-S1_100 -12294.0264755
20   0
40   0
60   0
80   0
100   0
120   0
140   0
160   0
180   0
200   0
220   0
240   0
260   0
280   0
300   0
320   0
340   0
360   0
A-RIB:A-R6:LYS-S1 A-RIB:A-R6:LYS-S1_20 0.0
20   0
40   1
60   5
80   8
100   9
120   9
140   8
160   5
180   0
200   0
220   1
240   6
260   9
280   9
300   10
320   8
340   6
360   0
C-RIB:C-P:MET-CA
20   0
40   0
60   0
80   1
100   2
120   2
140   2
160   0
180   0
200   0
220   0
240   0
260   1
280   2

300   2
320   2
340   1
360   0
C-RIB:C-Y:GLY-CA C-RIB:C-Y:GLY-CA_220 -4251.83856205
20   1
40   4
60   7
80   8
100   6
120   6
140   5
160   3
180   1
200   1
220   4
240   7
260   8
280   7
300   6
320   5
340   0
360   1
C-P:C-RIB:TYR-S2 C-P:C-RIB:TYR-S2_140 -4361.42046739
20   0
40   0
60   1
80   2
100   2
120   3
140   2
160   1
180   0
200   0
220   0
240   1
260   2
280   3
300   3
320   2
340   1
360   1
FMU-RIB:FMU-P:GLN-CA FMU-RIB:FMU-P:GLN-CA_260 0.0
20   0
40   0
60   0
80   0
100   0
120   0
140   0
160   0
180   0
200   0
220   0
240   0
260   0
280   0
300   0

320   0
340   0
360   0
A-RIB:A-R5:HIS-CA A-RIB:A-R5:HIS-CA_240 -6560.72612048
20   0
40   0
60   1
80   1
100   1
120   1
140   0
160   0
180   0
200   0
220   0
240   1
260   1
280   1
300   1
320   1
340   0
360   0
U-RIB:U-Y:TYR-CA U-RIB:U-Y:TYR-CA_60 -3645.02824375
20   0
40   0
60   0
80   0
100   0
120   0
140   0
160   0
180   0
200   0
220   0
240   0
260   0
280   1
300   1
320   0
340   0
360   0
IU-P:IU-RIB:ILE-S1 IU-P:IU-RIB:ILE-S1_60 0.0
20   0
40   0
60   0
80   0
100   0
120   0
140   0
160   0
180   0
200   0
220   0
240   0
260   0
280   0
300   0
320   0

340   0
360   0
H2U-RIB:H2U-MY:GLN-CA H2U-RIB:H2U-MY:GLN-CA_200 0.0
20   0
40   0
60   0
80   0
100   0
120   0
140   0
160   0
180   0
200   0
220   0
240   0
260   0
280   0
300   0
320   0
340   0
360   0
FMU-RIB:FMU-MY:GLN-CA FMU-RIB:FMU-MY:GLN-CA_40 0.0
20   0
40   0
60   0
80   0
100   0
120   0
140   0
160   0
180   0
200   0
220   0
240   0
260   0
280   0
300   0
320   0
340   0
360   0
C-RIB:C-Y:ASP-S1 C-RIB:C-Y:ASP-S1_240 -2449.00462932
20   1
40   3
60   6
80   7
100   6
120   5
140   4
160   2
180   0
200   1
220   4
240   6
260   6
280   5
300   5
320   4
340   3

360 1
H2U-RIB:H2U-MY:ALA-CA H2U-RIB:H2U-MY:ALA-CA_320 0.0
20 0
40 0
60 0
80 0
100 0
120 0
140 0
160 0
180 0
200 0
220 0
240 0
260 0
280 0
300 0
320 0
340 0
360 0
C-RIB:C-P:TRP-S2 C-RIB:C-P:TRP-S2_100 -4920.61641895
20 0
40 0
60 0
80 1
100 1
120 1
140 1
160 0
180 0
200 0
220 0
240 0
260 1
280 0
300 1
320 0
340 0
360 0
QUO-RIB:QUO-M6:GLN-S2 QUO-RIB:QUO-M6:GLN-S2_60 0.0
20 0
40 0
60 0
80 0
100 0
120 0
140 0
160 0
180 0
200 0
220 0
240 0
260 0
280 0
300 0
320 0
340 0
360 0

OMC-RIB:OMC-MY:LYS-S2 OMC-RIB:OMC-MY:LYS-S2_220 0.0
20 0
40 0
60 0
80 0
100 0
120 0
140 0
160 0
180 0
200 0
220 0
240 0
260 0
280 0
300 0
320 0
340 0
360 0
5BU-RIB:5BU-MY:PRO-CA 5BU-RIB:5BU-MY:PRO-CA_40 0.0
20 0
40 0
60 0
80 0
100 0
120 0
140 0
160 0
180 0
200 0
220 0
240 0
260 0
280 0
300 0
320 0
340 0
360 0
IU-RIB:IU-P:HIS-S2 IU-RIB:IU-P:HIS-S2_60 0.0
20 0
40 0
60 0
80 0
100 0
120 0
140 0
160 0
180 0
200 0
220 0
240 0
260 0
280 0
300 0
320 0
340 0
360 0
FMU-P:FMU-RIB:ASN-S1 FMU-P:FMU-RIB:ASN-S1_340 0.0

```
20   0
40   0
60   0
80   0
100   0
120   0
140   0
160   0
180   0
200   0
220   0
240   0
260   0
280   0
300   0
320   0
340   0
360   0
FMU-P:FMU-RIB:ILE-CA FMU-P:FMU-RIB:ILE-CA_220 0.0
20   0
40   0
60   0
80   0
100   0
120   0
140   0
160   0
180   0
200   0
220   0
240   0
260   0
280   0
300   0
320   0
340   0
360   0
FHU-RIB:FHU-MY:ARG-S1 FHU-RIB:FHU-MY:ARG-S1_20 0.0
20   0
40   0
60   0
80   0
100   0
120   0
140   0
160   0
180   0
200   0
220   0
240   0
260   0
280   0
300   0
320   0
340   0
360   0
U31-RIB:U31-P:SER-CA U31-RIB:U31-P:SER-CA_40 0.0
20   0
```

40   0
60   0
80   0
100   0
120   0
140   0
160   0
180   0
200   0
220   0
240   0
260   0
280   0
300   0
320   0
340   0
360   0
5BU-P:5BU-RIB:ARG-S2 5BU-P:5BU-RIB:ARG-S2_260 0.0
20   0
40   0
60   0
80   0
100   0
120   0
140   0
160   0
180   0
200   0
220   0
240   0
260   0
280   0
300   0
320   0
340   0
360   0
U31-RIB:U31-P:SER-S1 U31-RIB:U31-P:SER-S1_80 0.0
20   0
40   0
60   0
80   0
100   0
120   0
140   0
160   0
180   0
200   0
220   0
240   0
260   0
280   0
300   0
320   0
340   0
360   0
A-P:A-RIB:ARG-CA A-P:A-RIB:ARG-CA_360 -5002.28143995
20   0
40   1

60   4
80   7
100   9
120   9
140   8
160   6
180   2
200   0
220   1
240   4
260   7
280   9
300   9
320   8
340   6
360   2
U-P:U-RIB:PHE-CA U-P:U-RIB:PHE-CA_220 0.0
20   0
40   0
60   0
80   1
100   1
120   1
140   1
160   0
180   0
200   0
220   0
240   0
260   0
280   1
300   1
320   1
340   0
360   0
U-RIB:U-P:ILE-CA U-RIB:U-P:ILE-CA_20 0.0
20   0
40   0
60   1
80   0
100   3
120   3
140   2
160   1
180   0
200   0
220   0
240   1
260   2
280   3
300   3
320   3
340   2
360   0
FHU-RIB:FHU-MY:ILE-CA FHU-RIB:FHU-MY:ILE-CA_80 0.0
20   0
40   0
60   0

```
80   0
100   0
120   0
140   0
160   0
180   0
200   0
220   0
240   0
260   0
280   0
300   0
320   0
340   0
360   0
G-RIB:G-R6:TRP-S1 G-RIB:G-R6:TRP-S1_80 -6045.45357792
20   0
40   0
60   0
80   1
100   0
120   1
140   0
160   0
180   0
200   0
220   0
240   0
260   1
280   1
300   1
320   0
340   0
360   0
G-RIB:G-R5:ASN-S2 G-RIB:G-R5:ASN-S2_340 0.0
20   1
40   4
60   7
80   7
100   6
120   4
140   4
160   3
180   1
200   1
220   4
240   7
260   8
280   6
300   4
320   4
340   0
360   1
QUO-P:QUO-RIB:GLN-S2 QUO-P:QUO-RIB:GLN-S2_180 0.0
20   0
40   0
60   0
80   0
```

100   0
120   0
140   0
160   0
180   0
200   0
220   0
240   0
260   0
280   0
300   0
320   0
340   0
360   0
A-RIB:A-R6:ALA-CA A-RIB:A-R6:ALA-CA_340 -2464.58777915
20   0
40   1
60   3
80   5
100   6
120   6
140   0
160   4
180   1
200   0
220   1
240   3
260   0
280   6
300   6
320   6
340   4
360   1
G-RIB:G-R6:ALA-S1 G-RIB:G-R6:ALA-S1_320 -2540.66711358
20   0
40   3
60   6
80   9
100   9
120   9
140   7
160   4
180   1
200   0
220   3
240   6
260   8
280   9
300   9
320   7
340   4
360   0
FMU-RIB:FMU-MY:MET-S2 FMU-RIB:FMU-MY:MET-S2_80 0.0
20   0
40   0
60   0
80   0
100   0

120   0
140   0
160   0
180   0
200   0
220   0
240   0
260   0
280   0
300   0
320   0
340   0
360   0
C-RIB:C-P:GLN-S2 C-RIB:C-P:GLN-S2_320 -3427.41707298
20   0
40   1
60   3
80   5
100   7
120   6
140   5
160   4
180   1
200   0
220   1
240   3
260   5
280   7
300   6
320   6
340   4
360   1
U31-P:U31-RIB:GLN-S2 U31-P:U31-RIB:GLN-S2_120 0.0
20   0
40   0
60   0
80   0
100   0
120   0
140   0
160   0
180   0
200   0
220   0
240   0
260   0
280   0
300   0
320   0
340   0
360   0
C-P:C-RIB:ARG-CA C-P:C-RIB:ARG-CA_120 -4046.48742284
20   0
40   1
60   4
80   7
100   9
120   9

140   8
160   5
180   2
200   0
220   1
240   5
260   7
280   8
300   9
320   8
340   5
360   2
C31-RIB:C31-P:THR-S1 C31-RIB:C31-P:THR-S1_40 0.0
20   0
40   0
60   0
80   0
100   0
120   0
140   0
160   0
180   0
200   0
220   0
240   0
260   0
280   0
300   0
320   0
340   0
360   0
I-P:I-RIB:TRP-S2 I-P:I-RIB:TRP-S2_200 0.0
20   0
40   0
60   0
80   0
100   0
120   0
140   0
160   0
180   0
200   0
220   0
240   0
260   0
280   0
300   0
320   0
340   0
360   0
U31-RIB:U31-MY:ASP-CA U31-RIB:U31-MY:ASP-CA_300 0.0
20   0
40   0
60   0
80   0
100   0
120   0
140   0

160   0
180   0
200   0
220   0
240   0
260   0
280   0
300   0
320   0
340   0
360   0
A-RIB:A-P:ASP-CA A-RIB:A-P:ASP-CA_360 -2552.09234415
20   0
40   0
60   4
80   8
100   11
120   12
140   10
160   7
180   3
200   0
220   1
240   4
260   8
280   11
300   12
320   10
340   7
360   2
C31-P:C31-RIB:PHE-CA C31-P:C31-RIB:PHE-CA_180 0.0
20   0
40   0
60   0
80   0
100   0
120   0
140   0
160   0
180   0
200   0
220   0
240   0
260   0
280   0
300   0
320   0
340   0
360   0
U-RIB:U-Y:MET-S1 U-RIB:U-Y:MET-S1_40 -4925.13367208
20   0
40   0
60   0
80   0
100   0
120   0
140   0
160   0

180   0
200   0
220   0
240   0
260   0
280   0
300   0
320   0
340   0
360   0
H2U-P:H2U-RIB:PRO-CA H2U-P:H2U-RIB:PRO-CA_220 0.0
20   0
40   0
60   0
80   0
100   0
120   0
140   0
160   0
180   0
200   0
220   0
240   0
260   0
280   0
300   0
320   0
340   0
360   0
FHU-RIB:FHU-MY:ARG-S2 FHU-RIB:FHU-MY:ARG-S2_280 -8306.30179085
20   0
40   0
60   0
80   0
100   0
120   0
140   0
160   0
180   0
200   0
220   0
240   0
260   0
280   0
300   0
320   0
340   0
360   0
G-P:G-RIB:THR-S1 G-P:G-RIB:THR-S1_160 -2841.84362512
20   0
40   3
60   7
80   9
100   10
120   9
140   8
160   6
180   2

200   0
220   3
240   7
260   9
280   10
300   10
320   8
340   6
360   2
H2U-RIB:H2U-P:LEU-S1 H2U-RIB:H2U-P:LEU-S1_340 0.0
20   0
40   0
60   0
80   0
100   0
120   0
140   0
160   0
180   0
200   0
220   0
240   0
260   0
280   0
300   0
320   0
340   0
360   0
A-RIB:A-R5:VAL-CA A-RIB:A-R5:VAL-CA_160 -5957.60904214
20   0
40   1
60   2
80   3
100   3
120   2
140   2
160   1
180   0
200   0
220   1
240   2
260   3
280   3
300   3
320   2
340   1
360   0
A-RIB:A-R6:TYR-CA A-RIB:A-R6:TYR-CA_260 -6396.55674627
20   0
40   0
60   0
80   1
100   1
120   1
140   0
160   0
180   0
200   0

220   0
240   0
260   1
280   1
300   1
320   1
340   1
360   0
G-RIB:G-R5:TRP-CA G-RIB:G-R5:TRP-CA_60 0.0
20   0
40   0
60   0
80   0
100   0
120   0
140   0
160   0
180   0
200   0
220   0
240   0
260   0
280   0
300   0
320   0
340   0
360   0
A-RIB:A-P:HIS-S2 A-RIB:A-P:HIS-S2_160 -2230.36594645
20   0
40   0
60   2
80   4
100   5
120   5
140   4
160   3
180   1
200   0
220   0
240   2
260   3
280   4
300   5
320   4
340   3
360   1
C31-RIB:C31-P:PHE-S1 C31-RIB:C31-P:PHE-S1_80 0.0
20   0
40   0
60   0
80   0
100   0
120   0
140   0
160   0
180   0
200   0
220   0

240   0
260   0
280   0
300   0
320   0
340   0
360   0
C31-RIB:C31-P:ASN-S2 C31-RIB:C31-P:ASN-S2_200 0.0
20   0
40   0
60   0
80   0
100   0
120   0
140   0
160   0
180   0
200   0
220   0
240   0
260   0
280   0
300   0
320   0
340   0
360   0
DA-RIB:DA-M6:GLU-S1 DA-RIB:DA-M6:GLU-S1_140 0.0
20   0
40   0
60   0
80   0
100   0
120   0
140   0
160   0
180   0
200   0
220   0
240   0
260   0
280   0
300   0
320   0
340   0
360   0
A-RIB:A-R5:ILE-CA A-RIB:A-R5:ILE-CA_160 -5332.06794932
20   0
40   0
60   1
80   2
100   2
120   2
140   0
160   0
180   0
200   0
220   0
240   1

260 2
280 0
300 1
320 1
340 0
360 0
GTP-RIB:GTP-M5:THR-S1 GTP-RIB:GTP-M5:THR-S1_80 0.0
20 0
40 0
60 0
80 0
100 0
120 0
140 0
160 0
180 0
200 0
220 0
240 0
260 0
280 0
300 0
320 0
340 0
360 0
M2G-RIB:M2G-P:GLU-CA M2G-RIB:M2G-P:GLU-CA_260 0.0
20 0
40 0
60 0
80 0
100 0
120 0
140 0
160 0
180 0
200 0
220 0
240 0
260 0
280 0
300 0
320 0
340 0
360 0
G-P:G-RIB:PRO-S1 G-P:G-RIB:PRO-S1_60 -4717.0915871
20 0
40 3
60 6
80 8
100 9
120 9
140 8
160 6
180 2
200 0
220 3
240 7
260 9

280   10
300   10
320   8
340   6
360   2
A-P:A-RIB:HIS-S1 A-P:A-RIB:HIS-S1_360 -3318.67502935
20   0
40   0
60   1
80   2
100   3
120   3
140   2
160   2
180   1
200   0
220   0
240   1
260   3
280   3
300   3
320   2
340   2
360   1
U34-RIB:U34-MY:PHE-S1 U34-RIB:U34-MY:PHE-S1_160 0.0
20   0
40   0
60   0
80   0
100   0
120   0
140   0
160   0
180   0
200   0
220   0
240   0
260   0
280   0
300   0
320   0
340   0
360   0
QUO-RIB:QUO-P:LEU-CA QUO-RIB:QUO-P:LEU-CA_80 0.0
20   0
40   0
60   0
80   0
100   0
120   0
140   0
160   0
180   0
200   0
220   0
240   0
260   0
280   0

300   0
320   0
340   0
360   0
G-P:G-RIB:ILE-CA G-P:G-RIB:ILE-CA_120 -4154.24865439
20   0
40   0
60   0
80   5
100   6
120   5
140   4
160   3
180   0
200   0
220   0
240   0
260   4
280   5
300   5
320   4
340   3
360   0
FHU-RIB:FHU-P:PRO-S1 FHU-RIB:FHU-P:PRO-S1_160 0.0
20   0
40   0
60   0
80   0
100   0
120   0
140   0
160   0
180   0
200   0
220   0
240   0
260   0
280   0
300   0
320   0
340   0
360   0
A-RIB:A-P:ASP-S1 A-RIB:A-P:ASP-S1_320 -2161.24394887
20   0
40   2
60   6
80   10
100   13
120   13
140   12
160   8
180   3
200   0
220   2
240   5
260   9
280   12
300   13

320   11
340   8
360   3
A-RIB:A-R6:ARG-S2 A-RIB:A-R6:ARG-S2_140 -4386.2738095
20   0
40   3
60   9
80   13
100   12
120   11
140   10
160   6
180   2
200   0
220   3
240   9
260   13
280   12
300   12
320   10
340   7
360   2
OMC-P:OMC-RIB:LYS-S2 OMC-P:OMC-RIB:LYS-S2_220 0.0
20   0
40   0
60   0
80   0
100   0
120   0
140   0
160   0
180   0
200   0
220   0
240   0
260   0
280   0
300   0
320   0
340   0
360   0
DA-RIB:DA-M6:LYS-S1 DA-RIB:DA-M6:LYS-S1_240 0.0
20   0
40   0
60   0
80   0
100   0
120   0
140   0
160   0
180   0
200   0
220   0
240   0
260   0
280   0
300   0
320   0

340   0
360   0
DA-RIB:DA-M5:HIS-S1 DA-RIB:DA-M5:HIS-S1_320 0.0
20   0
40   0
60   0
80   0
100   0
120   0
140   0
160   0
180   0
200   0
220   0
240   0
260   0
280   0
300   0
320   0
340   0
360   0
G-RIB:G-R5:ASN-CA G-RIB:G-R5:ASN-CA_300 -3574.34876648
20   0
40   1
60   3
80   4
100   3
120   3
140   2
160   1
180   0
200   0
220   2
240   4
260   4
280   0
300   3
320   3
340   1
360   0
C31-RIB:C31-MY:ALA-CA C31-RIB:C31-MY:ALA-CA_120 0.0
20   0
40   0
60   0
80   0
100   0
120   0
140   0
160   0
180   0
200   0
220   0
240   0
260   0
280   0
300   0
320   0
340   0

360 0
G-RIB:G-R5:PHE-CA G-RIB:G-R5:PHE-CA_60 -4840.68327234
20 0
40 0
60 1
80 1
100 1
120 0
140 0
160 0
180 0
200 0
220 0
240 1
260 1
280 1
300 0
320 1
340 0
360 0
DA-RIB:DA-M6:TYR-CA DA-RIB:DA-M6:TYR-CA_180 0.0
20 0
40 0
60 0
80 0
100 0
120 0
140 0
160 0
180 0
200 0
220 0
240 0
260 0
280 0
300 0
320 0
340 0
360 0
DA-RIB:DA-M6:LEU-S1 DA-RIB:DA-M6:LEU-S1_240 0.0
20 0
40 0
60 0
80 0
100 0
120 0
140 0
160 0
180 0
200 0
220 0
240 0
260 0
280 0
300 0
320 0
340 0
360 0

QUO-RIB:QUO-M6:ASN-S1 QUO-RIB:QUO-M6:ASN-S1_60 0.0
20 0
40 0
60 0
80 0
100 0
120 0
140 0
160 0
180 0
200 0
220 0
240 0
260 0
280 0
300 0
320 0
340 0
360 0
A-RIB:A-R6:MET-CA A-RIB:A-R6:MET-CA_80 -4276.46199607
20 0
40 0
60 0
80 1
100 1
120 1
140 1
160 0
180 0
200 0
220 0
240 0
260 1
280 1
300 1
320 1
340 0
360 0
G-RIB:G-R6:GLN-CA G-RIB:G-R6:GLN-CA_140 0.0
20 0
40 0
60 1
80 3
100 3
120 3
140 0
160 1
180 0
200 0
220 0
240 2
260 3
280 3
300 3
320 0
340 0
360 0
GTP-RIB:GTP-M5:SER-CA GTP-RIB:GTP-M5:SER-CA_300 0.0

20 0
40 0
60 0
80 0
100 0
120 0
140 0
160 0
180 0
200 0
220 0
240 0
260 0
280 0
300 0
320 0
340 0
360 0
G-P:G-RIB:LEU-CA G-P:G-RIB:LEU-CA_340 -2724.52452401
20 0
40 1
60 3
80 6
100 8
120 7
140 6
160 4
180 1
200 0
220 0
240 3
260 5
280 6
300 7
320 6
340 3
360 1
G-RIB:G-R6:PRO-CA G-RIB:G-R6:PRO-CA_180 0.0
20 0
40 1
60 3
80 5
100 6
120 5
140 3
160 2
180 0
200 0
220 1
240 3
260 5
280 5
300 0
320 3
340 2
360 0
A-RIB:A-R5:GLU-S2 A-RIB:A-R5:GLU-S2_320 -826.012490697
20 0

40   7
60   12
80   15
100   13
120   10
140   9
160   6
180   2
200   2
220   7
240   12
260   15
280   12
300   10
320   8
340   6
360   2
FMU-RIB:FMU-MY:VAL-CA FMU-RIB:FMU-MY:VAL-CA_280 0.0
20   0
40   0
60   0
80   0
100   0
120   0
140   0
160   0
180   0
200   0
220   0
240   0
260   0
280   0
300   0
320   0
340   0
360   0
IU-P:IU-RIB:ARG-S1 IU-P:IU-RIB:ARG-S1_360 0.0
20   0
40   0
60   0
80   0
100   0
120   0
140   0
160   0
180   0
200   0
220   0
240   0
260   0
280   0
300   0
320   0
340   0
360   0
C-RIB:C-Y:LEU-S2 C-RIB:C-Y:LEU-S2_300 -4282.26868412
20   0
40   1

60 2
80 2
100 2
120 2
140 1
160 0
180 0
200 0
220 1
240 2
260 2
280 2
300 2
320 0
340 0
360 0
C-RIB:C-P:GLU-S1
20 0
40 2
60 7
80 13
100 18
120 18
140 15
160 10
180 4
200 0
220 2
240 7
260 13
280 17
300 17
320 14
340 10
360 4
G-P:G-RIB:ARG-CA G-P:G-RIB:ARG-CA_340 -4327.60339176
20 0
40 2
60 7
80 11
100 13
120 12
140 10
160 7
180 3
200 0
220 2
240 7
260 10
280 12
300 12
320 10
340 7
360 2
FHU-P:FHU-RIB:TYR-S2 FHU-P:FHU-RIB:TYR-S2_300 -11671.3415359
20 0
40 0
60 0

80   0
100   0
120   0
140   0
160   0
180   0
200   0
220   0
240   0
260   0
280   0
300   0
320   0
340   0
360   0
C31-RIB:C31-P:ALA-S1 C31-RIB:C31-P:ALA-S1_20 0.0
20   0
40   0
60   0
80   0
100   0
120   0
140   0
160   0
180   0
200   0
220   0
240   0
260   0
280   0
300   0
320   0
340   0
360   0
A-P:A-RIB:LEU-CA A-P:A-RIB:LEU-CA_300 -3994.81399612
20   0
40   0
60   2
80   4
100   5
120   5
140   4
160   3
180   1
200   0
220   0
240   0
260   3
280   4
300   5
320   4
340   3
360   1
U-RIB:U-Y:ASP-S2 U-RIB:U-Y:ASP-S2_160 -3001.52039505
20   1
40   3
60   5
80   6

100 5
120 4
140 4
160 2
180 0
200 0
220 3
240 5
260 6
280 5
300 5
320 4
340 2
360 0
U34-RIB:U34-MY:VAL-CA U34-RIB:U34-MY:VAL-CA_220 0.0
20 0
40 0
60 0
80 0
100 0
120 0
140 0
160 0
180 0
200 0
220 0
240 0
260 0
280 0
300 0
320 0
340 0
360 0
FMU-RIB:FMU-MY:GLN-S1 FMU-RIB:FMU-MY:GLN-S1_280 0.0
20 0
40 0
60 0
80 0
100 0
120 0
140 0
160 0
180 0
200 0
220 0
240 0
260 0
280 0
300 0
320 0
340 0
360 0
G-RIB:G-R5:GLN-S2 G-RIB:G-R5:GLN-S2_120 -3220.17921001
20 1
40 4
60 6
80 7
100 5

120 4
140 3
160 0
180 0
200 1
220 4
240 6
260 7
280 5
300 4
320 3
340 2
360 1
G-P:G-RIB:MET-CA G-P:G-RIB:MET-CA_320 -3553.25061611
20 0
40 0
60 1
80 2
100 2
120 2
140 2
160 1
180 0
200 0
220 0
240 1
260 2
280 3
300 3
320 2
340 1
360 0
FMU-P:FMU-RIB:HIS-S1 FMU-P:FMU-RIB:HIS-S1_160 -17115.4326793
20 0
40 0
60 0
80 0
100 0
120 0
140 0
160 0
180 0
200 0
220 0
240 0
260 0
280 0
300 0
320 0
340 0
360 0
G-RIB:G-P:ALA-S1 G-RIB:G-P:ALA-S1_180 -1821.14701007
20 0
40 3
60 7
80 12
100 15
120 16

140   15
160   10
180   3
200   0
220   3
240   7
260   13
280   15
300   16
320   14
340   11
360   3
A-RIB:A-R5:ASP-S1 A-RIB:A-R5:ASP-S1_140 -1118.38539237
20   0
40   3
60   7
80   8
100   0
120   6
140   5
160   3
180   0
200   1
220   3
240   7
260   9
280   7
300   6
320   4
340   3
360   1
A-RIB:A-R5:GLY-CA A-RIB:A-R5:GLY-CA_360 -5903.33801208
20   1
40   5
60   8
80   9
100   8
120   7
140   5
160   4
180   1
200   1
220   5
240   8
260   9
280   8
300   7
320   6
340   4
360   1
H2U-RIB:H2U-MY:PHE-CA H2U-RIB:H2U-MY:PHE-CA_220 0.0
20   0
40   0
60   0
80   0
100   0
120   0
140   0

```
160   0
180   0
200   0
220   0
240   0
260   0
280   0
300   0
320   0
340   0
360   0
G-RIB:G-R6:LYS-S2 G-RIB:G-R6:LYS-S2_80 -2870.61502328
20   0
40   6
60   14
80   18
100   17
120   16
140   11
160   7
180   2
200   0
220   6
240   14
260   18
280   17
300   15
320   11
340   7
360   0
G-RIB:G-R5:LYS-CA G-RIB:G-R5:LYS-CA_240 -2281.16891768
20   1
40   4
60   6
80   6
100   6
120   6
140   5
160   3
180   1
200   1
220   4
240   6
260   6
280   7
300   6
320   5
340   3
360   1
C-P:C-RIB:LYS-CA C-P:C-RIB:LYS-CA_180 -2917.89192687
20   0
40   1
60   5
80   7
100   9
120   9
140   8
160   6
```

180   2
200   0
220   1
240   5
260   7
280   9
300   10
320   8
340   6
360   2
U34-RIB:U34-P:PRO-S1 U34-RIB:U34-P:PRO-S1_20 0.0
20   0
40   0
60   0
80   0
100   0
120   0
140   0
160   0
180   0
200   0
220   0
240   0
260   0
280   0
300   0
320   0
340   0
360   0
A-RIB:A-P:LYS-CA A-RIB:A-P:LYS-CA_300 -3424.94800187
20   0
40   1
60   4
80   8
100   11
120   12
140   10
160   7
180   2
200   0
220   1
240   4
260   7
280   11
300   12
320   10
340   7
360   2
C-RIB:C-Y:VAL-CA C-RIB:C-Y:VAL-CA_360 0.0
20   0
40   1
60   2
80   2
100   2
120   2
140   2
160   1
180   0

200 0
220 1
240 2
260 2
280 2
300 2
320 2
340 0
360 0
FHU-RIB:FHU-P:LEU-S2 FHU-RIB:FHU-P:LEU-S2_60 0.0
20 0
40 0
60 0
80 0
100 0
120 0
140 0
160 0
180 0
200 0
220 0
240 0
260 0
280 0
300 0
320 0
340 0
360 0
G-RIB:G-R5:TYR-S2 G-RIB:G-R5:TYR-S2_80 -3109.1022216
20 0
40 1
60 2
80 2
100 2
120 1
140 0
160 0
180 0
200 0
220 0
240 2
260 2
280 1
300 1
320 1
340 1
360 0
G-RIB:G-R5:CYS-CA G-RIB:G-R5:CYS-CA_120 -6965.6668076
20 0
40 0
60 0
80 0
100 0
120 0
140 0
160 0
180 0
200 0

220   0
240   0
260   0
280   0
300   0
320   0
340   0
360   0
G-RIB:G-R6:SER-S1 G-RIB:G-R6:SER-S1_80 -2716.56527317
20   0
40   3
60   7
80   9
100   9
120   8
140   6
160   4
180   1
200   0
220   3
240   7
260   9
280   9
300   8
320   6
340   4
360   0
FHU-RIB:FHU-MY:GLY-CA FHU-RIB:FHU-MY:GLY-CA_260 0.0
20   0
40   0
60   0
80   0
100   0
120   0
140   0
160   0
180   0
200   0
220   0
240   0
260   0
280   0
300   0
320   0
340   0
360   0
FHU-RIB:FHU-MY:ILE-S1 FHU-RIB:FHU-MY:ILE-S1_180 0.0
20   0
40   0
60   0
80   0
100   0
120   0
140   0
160   0
180   0
200   0
220   0

240 0
260 0
280 0
300 0
320 0
340 0
360 0
M2G-RIB:M2G-P:SER-S1 M2G-RIB:M2G-P:SER-S1_40 0.0
20 0
40 0
60 0
80 0
100 0
120 0
140 0
160 0
180 0
200 0
220 0
240 0
260 0
280 0
300 0
320 0
340 0
360 0
IU-RIB:IU-MY:ALA-CA IU-RIB:IU-MY:ALA-CA_360 0.0
20 0
40 0
60 0
80 0
100 0
120 0
140 0
160 0
180 0
200 0
220 0
240 0
260 0
280 0
300 0
320 0
340 0
360 0
C-RIB:C-Y:PRO-S1 C-RIB:C-Y:PRO-S1_320 -1895.45336404
20 0
40 2
60 4
80 4
100 3
120 3
140 2
160 1
180 0
200 0
220 2
240 4

260   4
280   3
300   2
320   2
340   1
360   0
C-P:C-RIB:PHE-S1 C-P:C-RIB:PHE-S1_40 -5253.4037977
20   0
40   0
60   1
80   2
100   2
120   3
140   2
160   1
180   0
200   0
220   0
240   0
260   1
280   2
300   2
320   2
340   1
360   0
A-RIB:A-R5:MET-CA A-RIB:A-R5:MET-CA_280 0.0
20   0
40   0
60   0
80   1
100   1
120   1
140   0
160   0
180   0
200   0
220   0
240   0
260   1
280   0
300   1
320   0
340   0
360   0
U31-P:U31-RIB:ASN-S2 U31-P:U31-RIB:ASN-S2_120 0.0
20   0
40   0
60   0
80   0
100   0
120   0
140   0
160   0
180   0
200   0
220   0
240   0
260   0

```
280   0
300   0
320   0
340   0
360   0
A-RIB:A-R6:ASN-S1 A-RIB:A-R6:ASN-S1_40 0.0
20   0
40   0
60   3
80   5
100   5
120   4
140   3
160   2
180   0
200   0
220   1
240   3
260   5
280   5
300   4
320   4
340   2
360   0
C31-P:C31-RIB:THR-S1 C31-P:C31-RIB:THR-S1_220 0.0
20   0
40   0
60   0
80   0
100   0
120   0
140   0
160   0
180   0
200   0
220   0
240   0
260   0
280   0
300   0
320   0
340   0
360   0
IU-RIB:IU-P:LEU-S1 IU-RIB:IU-P:LEU-S1_360 0.0
20   0
40   0
60   0
80   0
100   0
120   0
140   0
160   0
180   0
200   0
220   0
240   0
260   0
280   0
```

300   0
320   0
340   0
360   0
U-RIB:U-Y:LYS-CA U-RIB:U-Y:LYS-CA_160 -734.916093214
20   0
40   1
60   2
80   3
100   4
120   4
140   3
160   2
180   0
200   0
220   1
240   2
260   3
280   4
300   4
320   3
340   2
360   0
IU-RIB:IU-P:SER-CA IU-RIB:IU-P:SER-CA_40 0.0
20   0
40   0
60   0
80   0
100   0
120   0
140   0
160   0
180   0
200   0
220   0
240   0
260   0
280   0
300   0
320   0
340   0
360   0
G-RIB:G-R6:MET-S1 G-RIB:G-R6:MET-S1_140 0.0
20   0
40   0
60   0
80   1
100   1
120   1
140   0
160   0
180   0
200   0
220   0
240   1
260   1
280   1
300   1

320   1
340   0
360   0
G-RIB:G-P:ALA-CA G-RIB:G-P:ALA-CA_300 -2660.86437295
20   0
40   0
60   6
80   11
100   15
120   16
140   14
160   10
180   0
200   0
220   1
240   6
260   11
280   15
300   16
320   14
340   10
360   3
A-P:A-RIB:THR-S1 A-P:A-RIB:THR-S1_340 -4372.24807089
20   0
40   2
60   4
80   6
100   7
120   6
140   6
160   5
180   2
200   0
220   2
240   4
260   6
280   6
300   6
320   6
340   5
360   2
4SU-RIB:4SU-P:GLU-S2 4SU-RIB:4SU-P:GLU-S2_40 0.0
20   0
40   0
60   0
80   0
100   0
120   0
140   0
160   0
180   0
200   0
220   0
240   0
260   0
280   0
300   0
320   0

340   0
360   0
H2U-RIB:H2U-MY:PRO-CA H2U-RIB:H2U-MY:PRO-CA_220 0.0
20   0
40   0
60   0
80   0
100   0
120   0
140   0
160   0
180   0
200   0
220   0
240   0
260   0
280   0
300   0
320   0
340   0
360   0
H2U-RIB:H2U-P:TRP-CA H2U-RIB:H2U-P:TRP-CA_60 0.0
20   0
40   0
60   0
80   0
100   0
120   0
140   0
160   0
180   0
200   0
220   0
240   0
260   0
280   0
300   0
320   0
340   0
360   0
GTP-RIB:GTP-M6:GLY-CA GTP-RIB:GTP-M6:GLY-CA_220 0.0
20   0
40   0
60   0
80   0
100   0
120   0
140   0
160   0
180   0
200   0
220   0
240   0
260   0
280   0
300   0
320   0
340   0

360 0
C-P:C-RIB:ASN-S2 C-P:C-RIB:ASN-S2_180 -2059.62461839
20 0
40 2
60 4
80 6
100 6
120 6
140 6
160 5
180 2
200 0
220 2
240 5
260 7
280 7
300 6
320 6
340 5
360 2
G-RIB:G-R5:LEU-S2 G-RIB:G-R5:LEU-S2_20 -5302.93363265
20 0
40 2
60 3
80 3
100 3
120 3
140 2
160 1
180 0
200 0
220 2
240 3
260 3
280 3
300 3
320 2
340 0
360 0
QUO-RIB:QUO-P:ASN-S2 QUO-RIB:QUO-P:ASN-S2_180 0.0
20 0
40 0
60 0
80 0
100 0
120 0
140 0
160 0
180 0
200 0
220 0
240 0
260 0
280 0
300 0
320 0
340 0
360 0

A-RIB:A-P:TRP-S1 A-RIB:A-P:TRP-S1_300 -2417.03369985
20 0
40 0
60 0
80 1
100 1
120 1
140 0
160 0
180 0
200 0
220 0
240 0
260 0
280 1
300 1
320 1
340 0
360 0
DA-RIB:DA-M6:TYR-S1 DA-RIB:DA-M6:TYR-S1_300 -9346.57897766
20 0
40 0
60 0
80 0
100 0
120 0
140 0
160 0
180 0
200 0
220 0
240 0
260 0
280 0
300 0
320 0
340 0
360 0
IU-P:IU-RIB:ALA-CA IU-P:IU-RIB:ALA-CA_300 0.0
20 0
40 0
60 0
80 0
100 0
120 0
140 0
160 0
180 0
200 0
220 0
240 0
260 0
280 0
300 0
320 0
340 0
360 0
U-P:U-RIB:ASP-CA U-P:U-RIB:ASP-CA_120 -3394.29043855

20   0
40   0
60   3
80   5
100   6
120   6
140   5
160   3
180   1
200   0
220   1
240   3
260   5
280   6
300   5
320   5
340   3
360   1
U-RIB:U-Y:PHE-S1 U-RIB:U-Y:PHE-S1_160 0.0
20   0
40   0
60   0
80   0
100   0
120   0
140   0
160   0
180   0
200   0
220   0
240   0
260   0
280   0
300   0
320   0
340   0
360   0
U-RIB:U-Y:LYS-S1 U-RIB:U-Y:LYS-S1_120 -2147.45147699
20   0
40   2
60   4
80   5
100   5
120   5
140   4
160   3
180   1
200   0
220   2
240   4
260   5
280   5
300   5
320   4
340   3
360   1
U-RIB:U-P:GLU-S1 U-RIB:U-P:GLU-S1_140 -1555.18986367
20   0

40 1
60 5
80 8
100 11
120 11
140 9
160 6
180 2
200 0
220 1
240 4
260 8
280 11
300 11
320 9
340 6
360 0
U-P:U-RIB:TRP-CA U-P:U-RIB:TRP-CA_320 0.0
20 0
40 0
60 0
80 0
100 0
120 0
140 0
160 0
180 0
200 0
220 0
240 0
260 0
280 0
300 0
320 0
340 0
360 0
C31-RIB:C31-P:GLU-CA C31-RIB:C31-P:GLU-CA_140 0.0
20 0
40 0
60 0
80 0
100 0
120 0
140 0
160 0
180 0
200 0
220 0
240 0
260 0
280 0
300 0
320 0
340 0
360 0
G-RIB:G-R6:THR-S1 G-RIB:G-R6:THR-S1_240 -4642.47249283
20 0
40 2

60   5
80   7
100   7
120   7
140   5
160   3
180   0
200   0
220   2
240   5
260   7
280   7
300   7
320   5
340   3
360   0
U-RIB:U-P:HIS-S1 U-RIB:U-P:HIS-S1_160 -5093.49588708
20   0
40   0
60   0
80   1
100   2
120   2
140   2
160   1
180   0
200   0
220   0
240   1
260   1
280   2
300   2
320   2
340   1
360   0
U-P:U-RIB:LYS-S1 U-P:U-RIB:LYS-S1_320 -3677.2420386
20   0
40   2
60   5
80   7
100   7
120   7
140   6
160   5
180   2
200   0
220   2
240   5
260   7
280   7
300   7
320   6
340   5
360   2
U31-P:U31-RIB:ASN-S1 U31-P:U31-RIB:ASN-S1_20 0.0
20   0
40   0
60   0

80   0
100   0
120   0
140   0
160   0
180   0
200   0
220   0
240   0
260   0
280   0
300   0
320   0
340   0
360   0
DA-RIB:DA-M5:GLN-CA DA-RIB:DA-M5:GLN-CA_200 0.0
20   0
40   0
60   0
80   0
100   0
120   0
140   0
160   0
180   0
200   0
220   0
240   0
260   0
280   0
300   0
320   0
340   0
360   0
A-RIB:A-R5:TRP-S1 A-RIB:A-R5:TRP-S1_120 -4283.43165652
20   0
40   0
60   0
80   0
100   0
120   0
140   0
160   0
180   0
200   0
220   0
240   0
260   0
280   0
300   0
320   0
340   0
360   0
FMU-P:FMU-RIB:ALA-CA FMU-P:FMU-RIB:ALA-CA_360 0.0
20   0
40   0
60   0
80   0

100   0
120   0
140   0
160   0
180   0
200   0
220   0
240   0
260   0
280   0
300   0
320   0
340   0
360   0
C-RIB:C-Y:ILE-CA C-RIB:C-Y:ILE-CA_260 -3429.888607
20   0
40   0
60   1
80   1
100   1
120   1
140   1
160   0
180   0
200   0
220   0
240   1
260   1
280   1
300   1
320   1
340   0
360   0
G-P:G-RIB:CYS-CA G-P:G-RIB:CYS-CA_360 0.0
20   0
40   0
60   0
80   0
100   0
120   0
140   0
160   0
180   0
200   0
220   0
240   0
260   0
280   1
300   0
320   0
340   0
360   0
FMU-P:FMU-RIB:PHE-CA FMU-P:FMU-RIB:PHE-CA_40 0.0
20   0
40   0
60   0
80   0
100   0

120   0
140   0
160   0
180   0
200   0
220   0
240   0
260   0
280   0
300   0
320   0
340   0
360   0
FMU-RIB:FMU-P:ARG-S2 FMU-RIB:FMU-P:ARG-S2_260 0.0
20   0
40   0
60   0
80   0
100   0
120   0
140   0
160   0
180   0
200   0
220   0
240   0
260   0
280   0
300   0
320   0
340   0
360   0
U-RIB:U-P:THR-CA U-RIB:U-P:THR-CA_80 -2054.88802231
20   0
40   0
60   2
80   3
100   5
120   5
140   4
160   3
180   1
200   0
220   0
240   2
260   3
280   5
300   5
320   4
340   3
360   1
C-P:C-RIB:TYR-S1 C-P:C-RIB:TYR-S1_260 -5328.87296157
20   0
40   0
60   1
80   1
100   2
120   2

140   2
160   1
180   0
200   0
220   0
240   0
260   1
280   2
300   2
320   2
340   1
360   0
U34-P:U34-RIB:TYR-S2 U34-P:U34-RIB:TYR-S2_40 0.0
20   0
40   0
60   0
80   0
100   0
120   0
140   0
160   0
180   0
200   0
220   0
240   0
260   0
280   0
300   0
320   0
340   0
360   0
A-P:A-RIB:MET-S1 A-P:A-RIB:MET-S1_120 -4271.8264238
20   0
40   0
60   0
80   1
100   2
120   2
140   1
160   1
180   0
200   0
220   0
240   1
260   1
280   2
300   2
320   1
340   1
360   0
H2U-RIB:H2U-MY:TRP-S2 H2U-RIB:H2U-MY:TRP-S2_360 0.0
20   0
40   0
60   0
80   0
100   0
120   0
140   0

160   0
180   0
200   0
220   0
240   0
260   0
280   0
300   0
320   0
340   0
360   0
5BU-RIB:5BU-P:ARG-S1 5BU-RIB:5BU-P:ARG-S1_340 0.0
20   0
40   0
60   0
80   0
100   0
120   0
140   0
160   0
180   0
200   0
220   0
240   0
260   0
280   0
300   0
320   0
340   0
360   0
G-RIB:G-R5:CYS-S1 G-RIB:G-R5:CYS-S1_160 -6071.6478048
20   0
40   0
60   0
80   0
100   0
120   0
140   0
160   0
180   0
200   0
220   0
240   0
260   0
280   0
300   0
320   0
340   0
360   0
FHU-RIB:FHU-MY:ASP-S1 FHU-RIB:FHU-MY:ASP-S1_80 0.0
20   0
40   0
60   0
80   0
100   0
120   0
140   0
160   0

```
180   0
200   0
220   0
240   0
260   0
280   0
300   0
320   0
340   0
360   0
G-RIB:G-R5:GLU-CA G-RIB:G-R5:GLU-CA_240 -1763.68652742
20   0
40   4
60   7
80   8
100   8
120   7
140   6
160   4
180   1
200   0
220   4
240   7
260   8
280   8
300   7
320   6
340   4
360   1
H2U-P:H2U-RIB:ARG-S2 H2U-P:H2U-RIB:ARG-S2_220 0.0
20   0
40   0
60   0
80   0
100   0
120   0
140   0
160   0
180   0
200   0
220   0
240   0
260   0
280   0
300   0
320   0
340   0
360   0
C-RIB:C-Y:TYR-S1 C-RIB:C-Y:TYR-S1_60 -5267.20724652
20   0
40   0
60   0
80   0
100   0
120   0
140   0
160   0
180   0
```

200   0
220   0
240   0
260   0
280   0
300   0
320   0
340   0
360   0
A-RIB:A-R6:CYS-S1 A-RIB:A-R6:CYS-S1_260 -6519.08291071
20   0
40   0
60   0
80   0
100   0
120   0
140   0
160   0
180   0
200   0
220   0
240   0
260   0
280   0
300   0
320   0
340   0
360   0
A-RIB:A-P:GLU-S2 A-RIB:A-P:GLU-S2_140 -1728.45614198
20   0
40   4
60   10
80   17
100   20
120   21
140   17
160   13
180   5
200   0
220   4
240   10
260   17
280   21
300   20
320   18
340   13
360   5
QUO-P:QUO-RIB:LEU-S2 QUO-P:QUO-RIB:LEU-S2_300 0.0
20   0
40   0
60   0
80   0
100   0
120   0
140   0
160   0
180   0
200   0

220 0
240 0
260 0
280 0
300 0
320 0
340 0
360 0
IU-RIB:IU-MY:HIS-CA IU-RIB:IU-MY:HIS-CA_120 0.0
20 0
40 0
60 0
80 0
100 0
120 0
140 0
160 0
180 0
200 0
220 0
240 0
260 0
280 0
300 0
320 0
340 0
360 0
G-RIB:G-P:ARG-CA G-RIB:G-P:ARG-CA_160 -4897.68213357
20 0
40 1
60 5
80 10
100 15
120 18
140 15
160 10
180 4
200 0
220 1
240 5
260 11
280 16
300 18
320 16
340 10
360 4
U-RIB:U-P:GLU-CA U-RIB:U-P:GLU-CA_160 -1136.93781026
20 0
40 0
60 0
80 6
100 9
120 9
140 8
160 5
180 2
200 0
220 0

```
240   3
260   6
280   9
300   10
320   8
340   5
360   0
G-RIB:G-R6:CYS-CA G-RIB:G-R6:CYS-CA_220 0.0
20   0
40   0
60   0
80   0
100   0
120   0
140   0
160   0
180   0
200   0
220   0
240   0
260   0
280   0
300   0
320   0
340   0
360   0
EXPECTED_SITE
G:HIS-S2
WoCr   95
Sug   150
Hoo   112
C:GLU-CA
WoCr   45
Sug   25
Hoo   17
A:ASN-CA
WoCr   48
Sug   30
Hoo   24
C:LEU-CA
WoCr   18
Sug   18
Hoo   6
U:ASN-S2
WoCr   14
Sug   147
Hoo   92
G:ARG-CA
WoCr   86
Sug   212
Hoo   44
C:SER-CA
WoCr   37
Sug   73
Hoo   42
A:GLU-CA
WoCr   78
Sug   107
```

Hoo 36
G:GLN-CA
WoCr 26
Sug 50
Hoo 38
C:ASP-S2
WoCr 88
Sug 363
Hoo 98
C:VAL-S1
WoCr 26
Sug 37
Hoo 73
G:ALA-S1
WoCr 205
Sug 328
Hoo 105
C:GLN-S1
WoCr 47
Sug 75
Hoo 21
G:GLN-S1
WoCr 68
Sug 103
Hoo 91
U:LYS-S2
WoCr 64
Sug 234
Hoo 217
C:ASN-S2
WoCr 22
Sug 141
Hoo 75
A:LYS-S2
WoCr 323
Sug 391
Hoo 358
G:LYS-S1
WoCr 123
Sug 360
Hoo 95
C:GLU-S1
WoCr 30
Sug 119
Hoo 104
C:TRP-S1
WoCr 2
Sug 11
Hoo 1
G:TRP-CA
WoCr 7
Sug 18
Hoo 5
U:GLU-S1
WoCr 109
Sug 143
Hoo 105
G:GLU-S2

WoCr 220
Sug 740
Hoo 264
A:PHE-S1
WoCr 12
Sug 34
Hoo 5
G:CYS-CA
WoCr 5
Sug 4
Hoo 0
A:ASN-S2
WoCr 167
Sug 212
Hoo 99
G:ALA-CA
WoCr 95
Sug 100
Hoo 68
A:ASN-S1
WoCr 63
Sug 145
Hoo 51
G:LEU-CA
WoCr 40
Sug 43
Hoo 90
G:ASN-CA
WoCr 49
Sug 130
Hoo 43
A:TYR-S1
WoCr 16
Sug 38
Hoo 5
C:LEU-S2
WoCr 54
Sug 19
Hoo 7
U:MET-S1
WoCr 8
Sug 8
Hoo 2
C:TYR-S1
WoCr 4
Sug 10
Hoo 3
G:TRP-S1
WoCr 7
Sug 27
Hoo 22
U:THR-S1
WoCr 35
Sug 98
Hoo 11
C:MET-CA
WoCr 3
Sug 13

Hoo 9
C:LYS-S1
WoCr 70
Sug 175
Hoo 57
A:ILE-S1
WoCr 91
Sug 78
Hoo 9
C:ARG-S1
WoCr 128
Sug 149
Hoo 100
G:HIS-CA
WoCr 26
Sug 47
Hoo 34
G:GLN-S2
WoCr 264
Sug 164
Hoo 200
C:LEU-S1
WoCr 23
Sug 67
Hoo 52
U:LYS-CA
WoCr 42
Sug 59
Hoo 33
A:MET-CA
WoCr 26
Sug 10
Hoo 5
G:ASP-CA
WoCr 123
Sug 93
Hoo 43
C:ALA-S1
WoCr 67
Sug 131
Hoo 50
U:PRO-S1
WoCr 11
Sug 123
Hoo 20
C:PHE-CA
WoCr 4
Sug 30
Hoo 13
G:VAL-S1
WoCr 126
Sug 142
Hoo 28
A:TRP-S1
WoCr 18
Sug 14
Hoo 4
U:GLU-S2

WoCr 41
Sug 130
Hoo 269
U:GLN-S1
WoCr 26
Sug 36
Hoo 23
G:ARG-S2
WoCr 279
Sug 554
Hoo 308
G:TYR-S2
WoCr 27
Sug 53
Hoo 37
G:ASN-S2
WoCr 178
Sug 186
Hoo 191
A:HIS-CA
WoCr 27
Sug 33
Hoo 9
U:SER-S1
WoCr 16
Sug 163
Hoo 15
C:VAL-CA
WoCr 11
Sug 24
Hoo 8
G:GLY-CA
WoCr 130
Sug 353
Hoo 276
C:ASP-S1
WoCr 46
Sug 131
Hoo 18
A:HIS-S2
WoCr 52
Sug 130
Hoo 25
A:LEU-S2
WoCr 54
Sug 55
Hoo 36
U:TRP-CA
WoCr 1
Sug 1
Hoo 1
U:ASP-S2
WoCr 104
Sug 309
Hoo 117
A:TRP-CA
WoCr 7
Sug 5

Hoo 3
A:ALA-S1
WoCr 230
Sug 214
Hoo 159
G:HIS-S1
WoCr 45
Sug 63
Hoo 17
A:SER-S1
WoCr 109
Sug 224
Hoo 140
G:GLU-CA
WoCr 124
Sug 167
Hoo 55
U:VAL-CA
WoCr 42
Sug 27
Hoo 47
U:ASN-CA
WoCr 22
Sug 35
Hoo 11
C:ASP-CA
WoCr 38
Sug 124
Hoo 50
U:PHE-S2
WoCr 3
Sug 27
Hoo 3
U:TYR-S2
WoCr 5
Sug 5
Hoo 5
U:TYR-CA
WoCr 4
Sug 18
Hoo 3
C:LYS-CA
WoCr 44
Sug 138
Hoo 68
U:HIS-CA
WoCr 5
Sug 29
Hoo 4
C:GLU-S2
WoCr 137
Sug 405
Hoo 211
A:ALA-CA
WoCr 79
Sug 131
Hoo 26
G:ARG-S1

WoCr 176
Sug 353
Hoo 171
U:CYS-CA
WoCr 11
Sug 0
Hoo 0
U:LEU-S2
WoCr 16
Sug 29
Hoo 14
A:ASP-S2
WoCr 167
Sug 245
Hoo 257
U:ASN-S1
WoCr 22
Sug 39
Hoo 25
G:MET-S2
WoCr 33
Sug 73
Hoo 34
A:GLN-CA
WoCr 35
Sug 26
Hoo 42
U:ARG-CA
WoCr 45
Sug 94
Hoo 46
U:VAL-S1
WoCr 9
Sug 30
Hoo 35
A:PRO-CA
WoCr 259
Sug 65
Hoo 32
U:HIS-S1
WoCr 5
Sug 32
Hoo 4
U:PHE-CA
WoCr 3
Sug 9
Hoo 3
U:MET-S2
WoCr 7
Sug 11
Hoo 16
A:PRO-S1
WoCr 107
Sug 171
Hoo 36
G:ASP-S1
WoCr 79
Sug 253

Hoo 118
A:GLN-S2
WoCr 77
Sug 131
Hoo 104
U:ALA-CA
WoCr 16
Sug 88
Hoo 44
A:ARG-S1
WoCr 92
Sug 133
Hoo 139
U:CYS-S1
WoCr 1
Sug 0
Hoo 0
U:PHE-S1
WoCr 3
Sug 4
Hoo 2
C:GLN-CA
WoCr 36
Sug 16
Hoo 6
G:SER-S1
WoCr 154
Sug 380
Hoo 130
G:PHE-S1
WoCr 43
Sug 50
Hoo 8
G:SER-CA
WoCr 185
Sug 247
Hoo 85
U:MET-CA
WoCr 2
Sug 2
Hoo 2
C:ILE-S1
WoCr 7
Sug 47
Hoo 9
A:GLU-S2
WoCr 264
Sug 277
Hoo 243
U:HIS-S2
WoCr 163
Sug 54
Hoo 10
A:ARG-S2
WoCr 304
Sug 471
Hoo 257
G:PHE-S2

WoCr 15
Sug 57
Hoo 22
G:CYS-S1
WoCr 5
Sug 3
Hoo 0
A:PHE-S2
WoCr 38
Sug 38
Hoo 6
C:GLY-CA
WoCr 200
Sug 329
Hoo 107
G:TRP-S2
WoCr 32
Sug 51
Hoo 6
U:GLU-CA
WoCr 43
Sug 59
Hoo 16
G:TYR-S1
WoCr 19
Sug 40
Hoo 48
U:GLN-S2
WoCr 50
Sug 90
Hoo 28
A:HIS-S1
WoCr 40
Sug 58
Hoo 10
G:LYS-CA
WoCr 112
Sug 226
Hoo 91
U:ILE-CA
WoCr 8
Sug 5
Hoo 12
A:MET-S1
WoCr 17
Sug 22
Hoo 16
C:TRP-CA
WoCr 2
Sug 2
Hoo 1
G:ASN-S1
WoCr 94
Sug 106
Hoo 136
G:PRO-CA
WoCr 47
Sug 174

Hoo   138
A:THR-CA
WoCr   42
Sug   39
Hoo   29
G:LEU-S1
WoCr   28
Sug   35
Hoo   19
A:ILE-CA
WoCr   29
Sug   61
Hoo   37
G:ILE-CA
WoCr   53
Sug   35
Hoo   15
A:ARG-CA
WoCr   83
Sug   105
Hoo   28
C:HIS-S2
WoCr   19
Sug   55
Hoo   82
C:ASN-CA
WoCr   10
Sug   85
Hoo   22
U:GLY-CA
WoCr   54
Sug   220
Hoo   33
A:GLY-CA
WoCr   149
Sug   360
Hoo   168
G:PRO-S1
WoCr   82
Sug   376
Hoo   92
A:LEU-CA
WoCr   30
Sug   29
Hoo   11
C:HIS-CA
WoCr   12
Sug   30
Hoo   44
C:THR-S1
WoCr   53
Sug   173
Hoo   61
C:CYS-S1
WoCr   1
Sug   1
Hoo   0
C:HIS-S1

WoCr 7
Sug 38
Hoo 4
A:TYR-CA
WoCr 29
Sug 10
Hoo 5
A:LEU-S1
WoCr 35
Sug 77
Hoo 11
A:CYS-S1
WoCr 4
Sug 2
Hoo 1
A:CYS-CA
WoCr 4
Sug 3
Hoo 1
C:PHE-S2
WoCr 11
Sug 6
Hoo 10
G:ASP-S2
WoCr 285
Sug 473
Hoo 312
C:CYS-CA
WoCr 1
Sug 0
Hoo 1
G:LEU-S2
WoCr 86
Sug 175
Hoo 71
A:ASP-CA
WoCr 171
Sug 145
Hoo 56
U:LEU-CA
WoCr 8
Sug 5
Hoo 17
G:TYR-CA
WoCr 13
Sug 12
Hoo 8
U:LYS-S1
WoCr 38
Sug 112
Hoo 22
U:ARG-S2
WoCr 28
Sug 303
Hoo 62
C:ILE-CA
WoCr 7
Sug 26

Hoo 24
G:GLU-S1
WoCr 96
Sug 321
Hoo 76
G:ILE-S1
WoCr 67
Sug 77
Hoo 16
A:ASP-S1
WoCr 84
Sug 258
Hoo 118
U:SER-CA
WoCr 13
Sug 46
Hoo 24
U:PRO-CA
WoCr 40
Sug 57
Hoo 23
U:ILE-S1
WoCr 18
Sug 76
Hoo 36
C:TYR-CA
WoCr 4
Sug 4
Hoo 3
U:TRP-S1
WoCr 0
Sug 12
Hoo 1
C:LYS-S2
WoCr 105
Sug 444
Hoo 249
G:MET-CA
WoCr 11
Sug 23
Hoo 8
C:ARG-S2
WoCr 58
Sug 266
Hoo 172
U:ARG-S1
WoCr 44
Sug 58
Hoo 80
C:PHE-S1
WoCr 13
Sug 4
Hoo 2
A:LYS-S1
WoCr 136
Sug 266
Hoo 101
A:VAL-S1

WoCr 48
Sug 139
Hoo 45
A:PHE-CA
WoCr 12
Sug 32
Hoo 5
C:THR-CA
WoCr 23
Sug 97
Hoo 10
A:TRP-S2
WoCr 17
Sug 57
Hoo 4
C:PRO-CA
WoCr 101
Sug 112
Hoo 9
A:MET-S2
WoCr 16
Sug 40
Hoo 7
C:SER-S1
WoCr 31
Sug 126
Hoo 65
C:ALA-CA
WoCr 29
Sug 124
Hoo 44
G:PHE-CA
WoCr 13
Sug 27
Hoo 8
C:ARG-CA
WoCr 17
Sug 40
Hoo 69
U:ALA-S1
WoCr 14
Sug 108
Hoo 36
C:MET-S2
WoCr 5
Sug 68
Hoo 3
C:MET-S1
WoCr 3
Sug 13
Hoo 3
A:GLN-S1
WoCr 141
Sug 67
Hoo 17
A:GLU-S1
WoCr 192
Sug 252

Hoo 50
U:ASP-S1
WoCr 323
Sug 120
Hoo 134
A:LYS-CA
WoCr 102
Sug 172
Hoo 54
U:ASP-CA
WoCr 14
Sug 29
Hoo 13
U:THR-CA
WoCr 11
Sug 9
Hoo 11
C:TRP-S2
WoCr 14
Sug 40
Hoo 1
G:MET-S1
WoCr 12
Sug 15
Hoo 10
C:GLN-S2
WoCr 146
Sug 136
Hoo 62
U:TRP-S2
WoCr 16
Sug 32
Hoo 18
C:ASN-S1
WoCr 114
Sug 115
Hoo 82
A:TYR-S2
WoCr 34
Sug 63
Hoo 14
C:TYR-S2
WoCr 7
Sug 30
Hoo 14
U:TYR-S1
WoCr 4
Sug 3
Hoo 35
U:GLN-CA
WoCr 12
Sug 17
Hoo 65
A:SER-CA
WoCr 80
Sug 131
Hoo 24
G:THR-S1

WoCr 165
Sug 227
Hoo 76
G:LYS-S2
WoCr 270
Sug 794
Hoo 446
A:THR-S1
WoCr 114
Sug 134
Hoo 70
A:VAL-CA
WoCr 86
Sug 66
Hoo 23
G:THR-CA
WoCr 44
Sug 85
Hoo 32
U:LEU-S1
WoCr 28
Sug 14
Hoo 5
C:PRO-S1
WoCr 36
Sug 134
Hoo 82
G:VAL-CA
WoCr 46
Sug 38
Hoo 25
