# Supplementary material for: DARS-RNP and QUASI-RNP: New statistical potentials for protein-RNA docking
Source: BMC Bioinformatics. 2011 Aug 18;12:348. doi: 10.1186/1471-2105-12-348 (PMC3179970; doi:10.1186/1471-2105-12-348)
Supplement: Additional file 6 — Expected_QUASI.pdf. Expected number of contacts in each distance, angle, and site bin, for each pair wise interaction in the QUASI-RNP potential. [file 1471-2105-12-348-S6.PDF]

EXPECTED
A-RIB:GLN-S2
3 0
4 0
5 3
6 5
7 8
8 12
9 19
M2G-P:GLU-CA
3 0
4 0
5 0
6 0
7 0
8 0
9 0
U-Y:GLU-S1
3 0
4 0
5 3
6 0
7 10
8 15
9 24
C-Y:HIS-CA
3 0
4 0
5 0
6 2
7 4
8 6
9 10
FHU-RIB:ASP-S2
3 0
4 0
5 0
6 0
7 0
8 0
9 0
U31-P:HIS-CA
3 0
4 0
5 0
6 0
7 0
8 0
9 0
C31-RIB:GLN-S2
3 0
4 0
5 0
6 0
7 0
8 0
9 0
H2U-RIB:PHE-S2

3 0
4 0
5 0
6 0
7 0
8 0
9 0
FHU-MY:GLN-S2
3 0
4 0
5 0
6 0
7 0
8 0
9 0
H2U-MY:LEU-S2
3 0
4 0
5 0
6 0
7 0
8 0
9 0
QUO-M5:PHE-CA
3 0
4 0
5 0
6 0
7 0
8 0
9 0
FHU-RIB:THR-S1
3 0
4 0
5 0
6 0
7 0
8 0
9 0
G-RIB:ARG-S2
3 0
4 0
5 7
6 12
7 19
8 30
9 45
FHU-P:TYR-S2
3 0
4 0
5 0
6 0
7 0
8 0
9 0
G-P:TRP-CA
3 0
4 0

5 1
6 2
7 3
8 5
9 9
C-P:TRP-S1
3 0
4 0
5 0
6 1
7 2
8 3
9 5
C31-RIB:PHE-S1
3 0
4 0
5 0
6 0
7 0
8 0
9 0
U-Y:ARG-S2
3 0
4 0
5 3
6 5
7 8
8 13
9 20
U31-P:ARG-S2
3 0
4 0
5 0
6 0
7 0
8 0
9 0
DA-M6:SER-CA
3 0
4 0
5 0
6 0
7 0
8 0
9 0
M2G-P:SER-S1
3 0
4 0
5 0
6 0
7 0
8 0
9 0
DA-RIB:MET-S2
3 0
4 0
5 0
6 0

7 0
8 0
9 0
A-R5:HIS-CA
3 0
4 0
5 0
6 3
7 5
8 8
9 13
G-RIB:ILE-S1
3 0
4 0
5 6
6 10
7 15
8 24
9 37
C31-MY:LEU-S1
3 0
4 0
5 0
6 0
7 0
8 0
9 0
IU-RIB:VAL-S1
3 0
4 0
5 0
6 0
7 0
8 0
9 0
C-Y:ILE-S1
3 0
4 0
5 3
6 6
7 10
8 15
9 24
U-P:LYS-CA
3 0
4 0
5 3
6 5
7 8
8 13
9 20
A-P:TRP-S1
3 0
4 0
5 1
6 1
7 3
8 4

9   7
C-P:GLN-S2
3   0
4   0
5   2
6   4
7   6
8   10
9   15
QUO-RIB:PHE-S2
3   0
4   0
5   0
6   0
7   0
8   0
9   0
U-RIB:LYS-CA
3   0
4   0
5   3
6   5
7   8
8   13
9   20
C-P:VAL-S1
3   0
4   0
5   5
6   8
7   13
8   20
9   31
G-R5:LYS-CA
3   0
4   0
5   7
6   12
7   19
8   31
9   47
GTP-M5:ASN-S1
3   0
4   0
5   0
6   0
7   0
8   0
9   0
GTP-RIB:ARG-CA
3   0
4   0
5   0
6   0
7   0
8   0
9   0
IU-RIB:GLN-S1

3 0
4 0
5 0
6 0
7 0
8 0
9 0
DA-M5:ASN-S2
3 0
4 0
5 0
6 0
7 0
8 0
9 0
G-RIB:GLU-CA
3 0
4 0
5 8
6 14
7 22
8 36
9 55
DA-M6:GLN-S1
3 0
4 0
5 0
6 0
7 0
8 0
9 0
G-R6:ALA-S1
3 0
4 1
5 8
6 15
7 23
8 36
9 56
A-R5:LEU-CA
3 0
4 0
5 0
6 12
7 19
8 31
9 47
5BU-RIB:PRO-CA
3 0
4 0
5 0
6 0
7 0
8 0
9 0
FMU-RIB:ALA-CA
3 0
4 0

5 0
6 0
7 0
8 0
9 0
C-P:ARG-S2
3 0
4 0
5 4
6 7
7 12
8 19
9 29
IU-RIB:LYS-S2
3 0
4 0
5 0
6 0
7 0
8 0
9 0
QUO-P:LEU-CA
3 0
4 0
5 0
6 0
7 0
8 0
9 0
G-R5:VAL-CA
3 0
4 0
5 0
6 13
7 20
8 32
9 50
A-RIB:LEU-S1
3 0
4 0
5 7
6 12
7 19
8 31
9 47
A-P:VAL-S1
3 0
4 0
5 6
6 10
7 16
8 26
9 39
G-R5:HIS-S2
3 0
4 0
5 0
6 4

7 6
8 10
9 16
DA-RIB:ASN-S2
3 0
4 0
5 0
6 0
7 0
8 0
9 0
U-Y:SER-CA
3 0
4 0
5 2
6 4
7 6
8 10
9 16
5BU-P:ILE-CA
3 0
4 0
5 0
6 0
7 0
8 0
9 0
IU-MY:THR-CA
3 0
4 0
5 0
6 0
7 0
8 0
9 0
C31-RIB:SER-CA
3 0
4 0
5 0
6 0
7 0
8 0
9 0
FHU-P:THR-CA
3 0
4 0
5 0
6 0
7 0
8 0
9 0
FHU-P:ARG-S2
3 0
4 0
5 0
6 0
7 0
8 0

9 0
U-RIB:THR-S1
3 0
4 0
5 2
6 4
7 6
8 9
9 14
U-RIB:HIS-S2
3 0
4 0
5 1
6 1
7 2
8 4
9 7
G-R6:GLN-CA
3 0
4 0
5 0
6 6
7 10
8 16
9 24
FMU-RIB:VAL-S1
3 0
4 0
5 0
6 0
7 0
8 0
9 0
A-RIB:LYS-S2
3 0
4 0
5 5
6 10
7 15
8 24
9 37
C-P:LEU-CA
3 0
4 0
5 6
6 10
7 15
8 24
9 37
A-R6:ALA-S1
3 0
4 0
5 7
6 12
7 18
8 29
9 44
C-Y:ASN-S2

3 0
4 0
5 2
6 4
7 7
8 11
9 17
C31-MY:THR-CA
3 0
4 0
5 0
6 0
7 0
8 0
9 0
IU-MY:SER-S1
3 0
4 0
5 0
6 0
7 0
8 0
9 0
U-RIB:ALA-CA
3 0
4 0
5 3
6 6
7 10
8 16
9 24
U31-P:ARG-CA
3 0
4 0
5 0
6 0
7 0
8 0
9 0
G-P:PRO-S1
3 0
4 0
5 4
6 8
7 12
8 20
9 31
A-R5:ILE-CA
3 0
4 0
5 0
6 8
7 12
8 19
9 30
DA-M6:SER-S1
3 0
4 0

5 0
6 0
7 0
8 0
9 0
A-R6:HIS-S1
3 0
4 0
5 2
6 3
7 5
8 8
9 13
DA-RIB:ASN-S1
3 0
4 0
5 0
6 0
7 0
8 0
9 0
A-P:GLN-S1
3 0
4 0
5 3
6 5
7 8
8 12
9 19
C-RIB:ASN-S2
3 0
4 0
5 2
6 4
7 7
8 11
9 17
C31-RIB:PHE-CA
3 0
4 0
5 0
6 0
7 0
8 0
9 0
C31-P:PHE-S2
3 0
4 0
5 0
6 0
7 0
8 0
9 0
C31-P:GLN-CA
3 0
4 0
5 0
6 0

7 0
8 0
9 0
GTP-RIB:ARG-S2
3 0
4 0
5 0
6 0
7 0
8 0
9 0
G-RIB:HIS-S1
3 0
4 0
5 2
6 4
7 6
8 10
9 16
A-P:GLU-S2
3 0
4 0
5 0
6 11
7 18
8 28
9 43
IU-P:ASP-S2
3 0
4 0
5 0
6 0
7 0
8 0
9 0
G-P:ALA-S1
3 0
4 0
5 8
6 15
7 23
8 36
9 55
FMU-MY:ASP-S1
3 0
4 0
5 0
6 0
7 0
8 0
9 0
A-R5:TRP-S1
3 0
4 0
5 0
6 1
7 3
8 4

9 7
U31-P:ASP-S2
3 0
4 0
5 0
6 0
7 0
8 0
9 0
C-RIB:GLN-CA
3 0
4 0
5 2
6 4
7 6
8 10
9 15
U-RIB:ARG-S2
3 0
4 0
5 3
6 5
7 8
8 13
9 20
C-P:HIS-S2
3 0
4 0
5 1
6 2
7 4
8 6
9 10
FMU-RIB:PHE-S2
3 0
4 0
5 0
6 0
7 0
8 0
9 0
G-RIB:GLN-CA
3 0
4 0
5 3
6 6
7 10
8 16
9 24
G-R5:LEU-S2
3 0
4 0
5 9
6 16
7 24
8 39
9 59
U-RIB:PHE-CA

3 0
4 0
5 1
6 3
7 4
8 7
9 11
C31-P:ASP-S1
3 0
4 0
5 0
6 0
7 0
8 0
9 0
U-Y:GLN-CA
3 0
4 0
5 1
6 2
7 4
8 7
9 10
A-R6:TRP-CA
3 0
4 0
5 0
6 0
7 3
8 4
9 7
5BU-P:ARG-S2
3 0
4 0
5 0
6 0
7 0
8 0
9 0
A-R5:GLN-CA
3 0
4 0
5 3
6 5
7 8
8 12
9 19
C-RIB:ASP-S2
3 0
4 0
5 4
6 7
7 10
8 17
9 26
5BU-P:THR-CA
3 0
4 0

5 0
6 0
7 0
8 0
9 0
FMU-MY:ASP-S2
3 0
4 0
5 0
6 0
7 0
8 0
9 0
G-R5:TRP-S2
3 0
4 0
5 1
6 2
7 3
8 5
9 9
U-RIB:MET-S2
3 0
4 0
5 1
6 2
7 3
8 4
9 7
I-RIB:TRP-S1
3 0
4 0
5 0
6 0
7 0
8 0
9 0
G-P:GLN-S2
3 0
4 0
5 3
6 6
7 9
8 15
9 23
DA-M5:VAL-S1
3 0
4 0
5 0
6 0
7 0
8 0
9 0
U-RIB:ILE-CA
3 0
4 0
5 0
6 0

7 6
8 10
9 16
QUO-M6:ARG-S1
3 0
4 0
5 0
6 0
7 0
8 0
9 0
G-R5:LYS-S1
3 0
4 0
5 7
6 12
7 19
8 31
9 47
U31-P:ASN-CA
3 0
4 0
5 0
6 0
7 0
8 0
9 0
A-P:ILE-S1
3 0
4 0
5 4
6 8
7 12
8 19
9 30
U-P:LYS-S2
3 0
4 0
5 3
6 5
7 8
8 13
9 20
A-R6:GLN-S1
3 0
4 0
5 3
6 5
7 8
8 12
9 19
U31-RIB:THR-S1
3 0
4 0
5 0
6 0
7 0
8 0

9 0
C-P:TRP-CA
3 0
4 0
5 0
6 1
7 2
8 3
9 5
A-RIB:SER-S1
3 0
4 0
5 4
6 8
7 12
8 19
9 29
FMU-P:ALA-S1
3 0
4 0
5 0
6 0
7 0
8 0
9 0
U-Y:TRP-CA
3 0
4 0
5 0
6 1
7 1
8 2
9 4
C-Y:THR-S1
3 0
4 0
5 3
6 5
7 9
8 14
9 21
GTP-M6:THR-S1
3 0
4 0
5 0
6 0
7 0
8 0
9 0
C-RIB:TYR-S1
3 0
4 0
5 2
6 3
7 5
8 9
9 14
GTP-RIB:SER-CA

3 0
4 0
5 0
6 0
7 0
8 0
9 0
FMU-RIB:CYS-CA
3 0
4 0
5 0
6 0
7 0
8 0
9 0
QUO-M6:PHE-S1
3 0
4 0
5 0
6 0
7 0
8 0
9 0
U-P:ASP-S2
3 0
4 0
5 2
6 4
7 7
8 11
9 17
DA-M5:ASP-S1
3 0
4 0
5 0
6 0
7 0
8 0
9 0
QUO-M6:ASP-CA
3 0
4 0
5 0
6 0
7 0
8 0
9 0
A-P:LYS-S1
3 0
4 0
5 6
6 10
7 15
8 24
9 37
C-P:THR-CA
3 0
4 0

5 3
6 5
7 9
8 14
9 21
A-P:ASP-S1
3 0
4 0
5 5
6 9
7 13
8 21
9 33
G-P:TRP-S1
3 0
4 0
5 1
6 2
7 3
8 5
9 9
A-RIB:GLU-S1
3 0
4 0
5 6
6 11
7 18
8 28
9 43
C31-MY:LEU-CA
3 0
4 0
5 0
6 0
7 0
8 0
9 0
FMU-MY:ASP-CA
3 0
4 0
5 0
6 0
7 0
8 0
9 0
G-RIB:GLN-S1
3 0
4 0
5 3
6 6
7 10
8 16
9 24
FHU-RIB:TYR-S1
3 0
4 0
5 0
6 0

7 0
8 0
9 0
C-P:TYR-S1
3 0
4 0
5 2
6 3
7 5
8 9
9 14
G-R5:ARG-CA
3 0
4 0
5 7
6 12
7 19
8 30
9 46
FHU-RIB:ARG-S1
3 0
4 0
5 0
6 0
7 0
8 0
9 0
DA-M6:HIS-S2
3 0
4 0
5 0
6 0
7 0
8 0
9 0
C31-P:MET-CA
3 0
4 0
5 0
6 0
7 0
8 0
9 0
H2U-MY:TRP-S2
3 0
4 0
5 0
6 0
7 0
8 0
9 0
DA-RIB:ARG-S1
3 0
4 0
5 0
6 0
7 0
8 0

9 0
A-R6:LEU-S2
3 0
4 0
5 7
6 12
7 19
8 31
9 47
C-Y:PRO-CA
3 0
4 0
5 3
6 5
7 8
8 13
9 20
QUO-M5:LYS-S2
3 0
4 0
5 0
6 0
7 0
8 0
9 0
A-RIB:VAL-CA
3 0
4 0
5 6
6 10
7 16
8 26
9 39
GTP-RIB:ALA-S1
3 0
4 0
5 0
6 0
7 0
8 0
9 0
G-R5:PRO-CA
3 0
4 0
5 5
6 8
7 13
8 20
9 31
A-R5:PHE-CA
3 0
4 0
5 0
6 5
7 8
8 13
9 20
G-R6:ARG-CA

3 0
4 0
5 7
6 12
7 19
8 30
9 46
U-P:LEU-S1
3 0
4 0
5 0
6 7
7 10
8 17
9 26
C-Y:HIS-S1
3 0
4 0
5 1
6 2
7 4
8 6
9 10
G-R5:PHE-S1
3 0
4 0
5 0
6 6
7 10
8 16
9 25
U-Y:LEU-S2
3 0
4 0
5 4
6 7
7 10
8 17
9 26
U-RIB:TYR-S1
3 0
4 0
5 0
6 2
7 4
8 6
9 9
C31-MY:PHE-CA
3 0
4 0
5 0
6 0
7 0
8 0
9 0
U-Y:ARG-CA
3 0
4 0

5 3
6 5
7 8
8 13
9 20
U-RIB:MET-CA
3 0
4 0
5 1
6 2
7 3
8 4
9 7
FMU-RIB:GLN-CA
3 0
4 0
5 0
6 0
7 0
8 0
9 0
IU-RIB:PRO-CA
3 0
4 0
5 0
6 0
7 0
8 0
9 0
IU-P:ILE-CA
3 0
4 0
5 0
6 0
7 0
8 0
9 0
QUO-M6:ASN-CA
3 0
4 0
5 0
6 0
7 0
8 0
9 0
U-Y:HIS-S2
3 0
4 0
5 1
6 1
7 2
8 4
9 7
U34-P:TYR-S1
3 0
4 0
5 0
6 0

7 0
8 0
9 0
U34-P:GLU-S1
3 0
4 0
5 0
6 0
7 0
8 0
9 0
H2U-MY:ARG-S1
3 0
4 0
5 0
6 0
7 0
8 0
9 0
FMU-MY:PRO-S1
3 0
4 0
5 0
6 0
7 0
8 0
9 0
C-Y:GLU-S2
3 0
4 0
5 5
6 9
7 14
8 22
9 34
C31-P:ALA-S1
3 0
4 0
5 0
6 0
7 0
8 0
9 0
C-Y:ILE-CA
3 0
4 0
5 0
6 6
7 10
8 15
9 24
U-Y:VAL-S1
3 0
4 0
5 3
6 5
7 9
8 14

9 21
U31-RIB:GLN-S2
3 0
4 0
5 0
6 0
7 0
8 0
9 0
U31-RIB:GLU-CA
3 0
4 0
5 0
6 0
7 0
8 0
9 0
FHU-P:PRO-CA
3 0
4 0
5 0
6 0
7 0
8 0
9 0
DA-M5:THR-S1
3 0
4 0
5 0
6 0
7 0
8 0
9 0
G-R5:ASP-CA
3 0
4 0
5 0
6 11
7 17
8 27
9 41
U31-P:ASP-CA
3 0
4 0
5 0
6 0
7 0
8 0
9 0
A-P:TYR-S1
3 0
4 0
5 2
6 4
7 7
8 11
9 17
C-Y:THR-CA

3 0
4 0
5 0
6 5
7 9
8 14
9 21
C31-MY:SER-CA
3 0
4 0
5 0
6 0
7 0
8 0
9 0
FMU-P:ASP-S1
3 0
4 0
5 0
6 0
7 0
8 0
9 0
QUO-RIB:ASN-CA
3 0
4 0
5 0
6 0
7 0
8 0
9 0
C-RIB:GLU-S2
3 0
4 0
5 5
6 9
7 14
8 22
9 34
U-RIB:GLN-S2
3 0
4 0
5 1
6 2
7 4
8 6
9 10
GTP-M6:ASN-CA
3 0
4 0
5 0
6 0
7 0
8 0
9 0
M2G-P:GLY-CA
3 0
4 0

5 0
6 0
7 0
8 0
9 0
G-R6:ASN-S2
3 0
4 0
5 4
6 7
7 11
8 17
9 26
G-R5:MET-CA
3 0
4 0
5 2
6 4
7 7
8 11
9 16
C31-RIB:THR-CA
3 0
4 0
5 0
6 0
7 0
8 0
9 0
QUO-P:LEU-S1
3 0
4 0
5 0
6 0
7 0
8 0
9 0
U-P:SER-S1
3 0
4 0
5 2
6 4
7 6
8 10
9 16
U34-P:TYR-CA
3 0
4 0
5 0
6 0
7 0
8 0
9 0
DA-M5:GLN-CA
3 0
4 0
5 0
6 0

7 0
8 0
9 0
DA-RIB:ARG-CA
3 0
4 0
5 0
6 0
7 0
8 0
9 0
DA-RIB:MET-S1
3 0
4 0
5 0
6 0
7 0
8 0
9 0
G-P:LEU-CA
3 0
4 0
5 9
6 16
7 24
8 38
9 59
FHU-MY:ARG-S1
3 0
4 0
5 0
6 0
7 0
8 0
9 0
U-Y:SER-S1
3 0
4 0
5 2
6 4
7 6
8 10
9 16
A-R5:LYS-S2
3 0
4 0
5 5
6 10
7 15
8 24
9 37
U-RIB:PRO-CA
3 0
4 0
5 0
6 3
7 5
8 9

9   13
U-RIB:LYS-S1
3   0
4   0
5   0
6   5
7   8
8   13
9   20
A-R6:GLU-S2
3   0
4   0
5   6
6   11
7   18
8   28
9   43
IU-MY:THR-S1
3   0
4   0
5   0
6   0
7   0
8   0
9   0
H2U-MY:GLU-S2
3   0
4   0
5   0
6   0
7   0
8   0
9   0
A-RIB:TRP-S2
3   0
4   0
5   1
6   1
7   3
8   4
9   7
C-RIB:ARG-CA
3   0
4   0
5   4
6   7
7   12
8   19
9   29
FMU-RIB:VAL-CA
3   0
4   0
5   0
6   0
7   0
8   0
9   0
QUO-M6:PHE-S2

3 0
4 0
5 0
6 0
7 0
8 0
9 0
H2U-P:TRP-CA
3 0
4 0
5 0
6 0
7 0
8 0
9 0
QUO-M6:ASP-S2
3 0
4 0
5 0
6 0
7 0
8 0
9 0
U-Y:THR-S1
3 0
4 0
5 2
6 4
7 6
8 9
9 14
U31-MY:TYR-S2
3 0
4 0
5 0
6 0
7 0
8 0
9 0
FHU-RIB:ARG-CA
3 0
4 0
5 0
6 0
7 0
8 0
9 0
A-R6:PRO-CA
3 0
4 0
5 3
6 6
7 10
8 16
9 25
A-P:GLN-CA
3 0
4 0

5 3
6 5
7 8
8 12
9 19
A-R5:THR-S1
3 0
4 0
5 4
6 7
7 11
8 17
9 27
U34-P:ARG-S1
3 0
4 0
5 0
6 0
7 0
8 0
9 0
C-P:HIS-CA
3 0
4 0
5 1
6 2
7 4
8 6
9 10
IU-MY:TYR-CA
3 0
4 0
5 0
6 0
7 0
8 0
9 0
G-P:LYS-S2
3 0
4 0
5 7
6 12
7 19
8 30
9 46
IU-RIB:LEU-S1
3 0
4 0
5 0
6 0
7 0
8 0
9 0
5BU-MY:ARG-S2
3 0
4 0
5 0
6 0

7 0
8 0
9 0
A-R6:TYR-S2
3 0
4 0
5 2
6 4
7 7
8 11
9 17
QUO-M5:ARG-S2
3 0
4 0
5 0
6 0
7 0
8 0
9 0
5BU-RIB:ARG-S2
3 0
4 0
5 0
6 0
7 0
8 0
9 0
C-P:ASP-S2
3 0
4 0
5 4
6 7
7 10
8 17
9 25
G-R6:MET-S1
3 0
4 0
5 2
6 4
7 7
8 11
9 16
GTP-M5:ASP-S1
3 0
4 0
5 0
6 0
7 0
8 0
9 0
FMU-RIB:GLN-S1
3 0
4 0
5 0
6 0
7 0
8 0

9 0
DA-RIB:TYR-CA
3 0
4 0
5 0
6 0
7 0
8 0
9 0
5BU-P:SER-S1
3 0
4 0
5 0
6 0
7 0
8 0
9 0
C31-RIB:ALA-S1
3 0
4 0
5 0
6 0
7 0
8 0
9 0
A-P:ILE-CA
3 0
4 0
5 4
6 8
7 12
8 19
9 30
C-P:ASN-S1
3 0
4 0
5 2
6 4
7 7
8 11
9 17
C31-P:TYR-CA
3 0
4 0
5 0
6 0
7 0
8 0
9 0
G-R6:ASN-CA
3 0
4 0
5 0
6 7
7 11
8 17
9 26
QUO-M6:ASN-S1

3 0
4 0
5 0
6 0
7 0
8 0
9 0
IU-RIB:ILE-CA
3 0
4 0
5 0
6 0
7 0
8 0
9 0
G-RIB:PHE-S2
3 0
4 0
5 4
6 6
7 10
8 16
9 25
A-P:HIS-S2
3 0
4 0
5 2
6 3
7 5
8 8
9 13
FHU-MY:THR-S1
3 0
4 0
5 0
6 0
7 0
8 0
9 0
C31-RIB:THR-S1
3 0
4 0
5 0
6 0
7 0
8 0
9 0
A-R6:TRP-S2
3 0
4 0
5 1
6 1
7 3
8 4
9 7
A-P:GLU-CA
3 0
4 0

5 6
6 0
7 18
8 28
9 43
G-R6:GLU-S2
3 0
4 0
5 8
6 14
7 22
8 35
9 54
A-R6:MET-S1
3 0
4 0
5 2
6 3
7 5
8 8
9 13
QUO-M6:GLU-S1
3 0
4 0
5 0
6 0
7 0
8 0
9 0
C31-RIB:ASP-S1
3 0
4 0
5 0
6 0
7 0
8 0
9 0
U-RIB:ALA-S1
3 0
4 0
5 3
6 6
7 10
8 16
9 24
A-R5:CYS-S1
3 0
4 0
5 0
6 1
7 2
8 3
9 5
U-Y:TRP-S2
3 0
4 0
5 0
6 1

7 1
8 0
9 4
A-P:ASP-CA
3 0
4 0
5 5
6 0
7 13
8 21
9 33
G-R5:ASP-S1
3 0
4 0
5 0
6 11
7 17
8 27
9 41
U31-MY:THR-CA
3 0
4 0
5 0
6 0
7 0
8 0
9 0
U34-RIB:SER-S1
3 0
4 0
5 0
6 0
7 0
8 0
9 0
A-R6:ASN-CA
3 0
4 0
5 3
6 5
7 8
8 14
9 21
U-P:GLN-CA
3 0
4 0
5 1
6 2
7 4
8 7
9 10
QUO-RIB:ASN-S1
3 0
4 0
5 0
6 0
7 0
8 0

9 0
U-Y:ASN-CA
3 0
4 0
5 1
6 3
7 4
8 7
9 11
FMU-RIB:ARG-S2
3 0
4 0
5 0
6 0
7 0
8 0
9 0
C31-RIB:TYR-S2
3 0
4 0
5 0
6 0
7 0
8 0
9 0
H2U-MY:GLU-CA
3 0
4 0
5 0
6 0
7 0
8 0
9 0
A-R6:GLY-CA
3 0
4 0
5 6
6 10
7 15
8 25
9 38
C31-MY:ASP-CA
3 0
4 0
5 0
6 0
7 0
8 0
9 0
QUO-RIB:PHE-S1
3 0
4 0
5 0
6 0
7 0
8 0
9 0
U-P:GLY-CA

3 0
4 0
5 3
6 5
7 8
8 13
9 21
IU-P:HIS-S2
3 0
4 0
5 0
6 0
7 0
8 0
9 0
C-P:THR-S1
3 0
4 0
5 3
6 5
7 9
8 14
9 21
C-P:ILE-CA
3 0
4 0
5 0
6 6
7 9
8 15
9 23
U31-MY:VAL-S1
3 0
4 0
5 0
6 0
7 0
8 0
9 0
A-RIB:GLU-CA
3 0
4 0
5 7
6 11
7 18
8 28
9 43
QUO-M6:LYS-CA
3 0
4 0
5 0
6 0
7 0
8 0
9 0
A-RIB:ARG-CA
3 0
4 0

5 5
6 9
7 15
8 24
9 36
C31-P:LEU-S2
3 0
4 0
5 0
6 0
7 0
8 0
9 0
FMU-MY:GLN-S2
3 0
4 0
5 0
6 0
7 0
8 0
9 0
C-Y:LYS-CA
3 0
4 0
5 0
6 8
7 12
8 19
9 30
G-R6:TRP-CA
3 0
4 0
5 1
6 2
7 3
8 5
9 9
FMU-P:PHE-S1
3 0
4 0
5 0
6 0
7 0
8 0
9 0
A-R5:CYS-CA
3 0
4 0
5 0
6 1
7 2
8 3
9 5
C-RIB:LEU-CA
3 0
4 0
5 6
6 10

7 15
8 24
9 38
C-RIB:TYR-S2
3 0
4 0
5 2
6 3
7 5
8 9
9 14
C-Y:GLN-CA
3 0
4 0
5 0
6 4
7 6
8 10
9 15
G-R6:ASP-S2
3 0
4 0
5 6
6 11
7 17
8 26
9 40
G-R6:GLN-S2
3 0
4 0
5 3
6 6
7 10
8 15
9 24
A-R5:ASN-S1
3 0
4 0
5 3
6 5
7 8
8 14
9 21
G-P:ARG-S1
3 0
4 0
5 7
6 12
7 19
8 29
9 45
G-P:THR-CA
3 0
4 0
5 5
6 9
7 14
8 22

9 33
U34-MY:VAL-CA
3 0
4 0
5 0
6 0
7 0
8 0
9 0
FHU-P:ALA-CA
3 0
4 0
5 0
6 0
7 0
8 0
9 0
G-P:HIS-S1
3 0
4 0
5 2
6 4
7 6
8 10
9 16
C-RIB:ARG-S2
3 0
4 0
5 4
6 7
7 12
8 19
9 29
U31-P:LEU-S2
3 0
4 0
5 0
6 0
7 0
8 0
9 0
A-P:SER-S1
3 0
4 0
5 4
6 8
7 12
8 19
9 29
U-P:ASN-CA
3 0
4 0
5 1
6 3
7 4
8 7
9 11
U-RIB:SER-CA

3 0
4 0
5 2
6 4
7 6
8 10
9 16
GTP-RIB:ASN-S1
3 0
4 0
5 0
6 0
7 0
8 0
9 0
A-R6:ARG-S1
3 0
4 0
5 5
6 9
7 15
8 24
9 36
QUO-M6:GLU-CA
3 0
4 0
5 0
6 0
7 0
8 0
9 0
A-P:LEU-S1
3 0
4 0
5 7
6 12
7 19
8 31
9 47
U31-MY:GLU-S1
3 0
4 0
5 0
6 0
7 0
8 0
9 0
C-RIB:MET-S1
3 0
4 0
5 1
6 2
7 4
8 7
9 10
IU-MY:ILE-S1
3 0
4 0

5 0
6 0
7 0
8 0
9 0
FHU-MY:PHE-S2
3 0
4 0
5 0
6 0
7 0
8 0
9 0
A-P:CYS-S1
3 0
4 0
5 0
6 0
7 0
8 0
9 5
G-R5:SER-CA
3 0
4 0
5 0
6 10
7 15
8 24
9 37
U31-P:ARG-S1
3 0
4 0
5 0
6 0
7 0
8 0
9 0
H2U-MY:ALA-CA
3 0
4 0
5 0
6 0
7 0
8 0
9 0
U31-RIB:MET-S2
3 0
4 0
5 0
6 0
7 0
8 0
9 0
C-P:LYS-S1
3 0
4 0
5 4
6 8

7 12
8 19
9 30
U-RIB:TRP-S1
3 0
4 0
5 0
6 1
7 1
8 2
9 4
U-P:PHE-S1
3 0
4 0
5 1
6 0
7 4
8 7
9 11
FHU-MY:LEU-CA
3 0
4 0
5 0
6 0
7 0
8 0
9 0
C-Y:CYS-S1
3 0
4 0
5 0
6 0
7 0
8 3
9 4
U-RIB:LYS-S2
3 0
4 0
5 3
6 5
7 8
8 13
9 20
A-RIB:MET-S2
3 0
4 0
5 2
6 3
7 5
8 8
9 13
A-R5:GLN-S1
3 0
4 0
5 3
6 5
7 8
8 12

9 19
U31-MY:ALA-S1
3 0
4 0
5 0
6 0
7 0
8 0
9 0
C-Y:ASP-S2
3 0
4 0
5 4
6 7
7 10
8 17
9 26
U-Y:TYR-CA
3 0
4 0
5 1
6 2
7 4
8 6
9 9
C-P:SER-S1
3 0
4 0
5 3
6 6
7 9
8 15
9 23
G-R6:CYS-S1
3 0
4 0
5 0
6 2
7 3
8 0
9 7
U31-MY:VAL-CA
3 0
4 0
5 0
6 0
7 0
8 0
9 0
QUO-M6:LYS-S1
3 0
4 0
5 0
6 0
7 0
8 0
9 0
A-P:ASN-S2

3 0
4 0
5 3
6 5
7 8
8 13
9 21
U31-RIB:GLN-S1
3 0
4 0
5 0
6 0
7 0
8 0
9 0
IU-RIB:HIS-S2
3 0
4 0
5 0
6 0
7 0
8 0
9 0
FHU-RIB:ILE-CA
3 0
4 0
5 0
6 0
7 0
8 0
9 0
U-P:TYR-S1
3 0
4 0
5 0
6 2
7 4
8 6
9 9
FMU-P:GLN-CA
3 0
4 0
5 0
6 0
7 0
8 0
9 0
C-Y:LYS-S1
3 0
4 0
5 4
6 8
7 12
8 19
9 30
C-RIB:PHE-CA
3 0
4 0

5 0
6 4
7 6
8 10
9 16
H2U-P:GLU-S2
3 0
4 0
5 0
6 0
7 0
8 0
9 0
A-RIB:VAL-S1
3 0
4 0
5 6
6 10
7 16
8 26
9 39
H2U-RIB:LEU-S2
3 0
4 0
5 0
6 0
7 0
8 0
9 0
G-P:ARG-CA
3 0
4 0
5 7
6 12
7 19
8 29
9 45
A-RIB:TYR-CA
3 0
4 0
5 2
6 4
7 7
8 11
9 17
U-P:VAL-S1
3 0
4 0
5 0
6 5
7 9
8 14
9 21
5BU-P:ILE-S1
3 0
4 0
5 0
6 0

7 0
8 0
9 0
U-RIB:PHE-S1
3 0
4 0
5 1
6 3
7 4
8 7
9 11
G-RIB:MET-CA
3 0
4 0
5 2
6 0
7 7
8 11
9 16
IU-RIB:HIS-S1
3 0
4 0
5 0
6 0
7 0
8 0
9 0
C-Y:TRP-S2
3 0
4 0
5 0
6 1
7 2
8 3
9 5
A-R5:VAL-S1
3 0
4 0
5 6
6 10
7 16
8 26
9 39
A-P:MET-S1
3 0
4 0
5 2
6 3
7 5
8 8
9 13
A-RIB:ARG-S1
3 0
4 0
5 5
6 9
7 15
8 24

9 36
U-P:TYR-CA
3 0
4 0
5 0
6 2
7 4
8 6
9 9
U34-P:SER-CA
3 0
4 0
5 0
6 0
7 0
8 0
9 0
FMU-RIB:ARG-CA
3 0
4 0
5 0
6 0
7 0
8 0
9 0
FMU-RIB:ASN-S1
3 0
4 0
5 0
6 0
7 0
8 0
9 0
A-R5:THR-CA
3 0
4 0
5 0
6 7
7 11
8 17
9 27
A-P:CYS-CA
3 0
4 0
5 0
6 1
7 2
8 3
9 5
DA-M5:ASN-S1
3 0
4 0
5 0
6 0
7 0
8 0
9 0
C-RIB:LEU-S1

3 0
4 0
5 6
6 10
7 15
8 24
9 38
G-R5:PRO-S1
3 0
4 0
5 5
6 8
7 13
8 20
9 31
U-P:ASP-CA
3 0
4 0
5 2
6 4
7 7
8 11
9 18
FHU-P:LYS-CA
3 0
4 0
5 0
6 0
7 0
8 0
9 0
5BU-RIB:ILE-S1
3 0
4 0
5 0
6 0
7 0
8 0
9 0
G-P:GLU-S2
3 0
4 0
5 8
6 14
7 22
8 35
9 53
FHU-RIB:LEU-S1
3 0
4 0
5 0
6 0
7 0
8 0
9 0
QUO-RIB:ASP-S1
3 0
4 0

5 0
6 0
7 0
8 0
9 0
C-P:TYR-S2
3 0
4 0
5 2
6 3
7 5
8 9
9 14
A-R5:ASN-CA
3 0
4 0
5 0
6 5
7 8
8 14
9 21
A-P:GLN-S2
3 0
4 0
5 3
6 5
7 8
8 12
9 19
U-RIB:SER-S1
3 0
4 0
5 2
6 4
7 6
8 10
9 16
A-P:ALA-S1
3 0
4 0
5 7
6 12
7 18
8 29
9 44
FHU-MY:SER-S1
3 0
4 0
5 0
6 0
7 0
8 0
9 0
C31-P:THR-CA
3 0
4 0
5 0
6 0

7 0
8 0
9 0
A-RIB:ASN-S1
3 0
4 0
5 3
6 5
7 8
8 14
9 21
A-R6:GLN-CA
3 0
4 0
5 0
6 5
7 8
8 12
9 19
H2U-P:GLU-CA
3 0
4 0
5 0
6 0
7 0
8 0
9 0
G-R6:ILE-S1
3 0
4 0
5 6
6 0
7 15
8 24
9 37
A-P:VAL-CA
3 0
4 0
5 6
6 10
7 16
8 26
9 39
U-P:PHE-CA
3 0
4 0
5 1
6 3
7 4
8 7
9 11
A-RIB:MET-CA
3 0
4 0
5 2
6 3
7 5
8 8

9 13
G-R5:LYS-S2
3 0
4 0
5 7
6 12
7 19
8 30
9 46
A-R6:PHE-S1
3 0
4 0
5 3
6 0
7 8
8 13
9 20
DA-M5:GLU-S2
3 0
4 0
5 0
6 0
7 0
8 0
9 0
U-P:LYS-S1
3 0
4 0
5 3
6 5
7 8
8 13
9 20
G-RIB:LEU-S1
3 0
4 0
5 9
6 16
7 24
8 39
9 59
U34-RIB:ASN-S1
3 0
4 0
5 0
6 0
7 0
8 0
9 0
C31-P:ASP-CA
3 0
4 0
5 0
6 0
7 0
8 0
9 0
U34-P:SER-S1

3 0
4 0
5 0
6 0
7 0
8 0
9 0
FMU-MY:ARG-S1
3 0
4 0
5 0
6 0
7 0
8 0
9 0
C-P:TRP-S2
3 0
4 0
5 0
6 1
7 2
8 3
9 5
C31-RIB:ASP-S2
3 0
4 0
5 0
6 0
7 0
8 0
9 0
GTP-M5:ALA-CA
3 0
4 0
5 0
6 0
7 0
8 0
9 0
FHU-MY:ALA-CA
3 0
4 0
5 0
6 0
7 0
8 0
9 0
A-R6:PHE-S2
3 0
4 0
5 3
6 5
7 8
8 13
9 20
U31-P:ALA-S1
3 0
4 0

5 0
6 0
7 0
8 0
9 0
U34-MY:SER-S1
3 0
4 0
5 0
6 0
7 0
8 0
9 0
5BU-RIB:SER-S1
3 0
4 0
5 0
6 0
7 0
8 0
9 0
H2U-MY:TRP-CA
3 0
4 0
5 0
6 0
7 0
8 0
9 0
U-Y:TYR-S1
3 0
4 0
5 1
6 0
7 4
8 6
9 9
QUO-RIB:LYS-S1
3 0
4 0
5 0
6 0
7 0
8 0
9 0
H2U-MY:GLU-S1
3 0
4 0
5 0
6 0
7 0
8 0
9 0
A-R6:ARG-CA
3 0
4 0
5 5
6 9

7   15
8   24
9   36
C31-RIB:ASP-CA
3   0
4   0
5   0
6   0
7   0
8   0
9   0
U31-MY:MET-S1
3   0
4   0
5   0
6   0
7   0
8   0
9   0
DA-M6:GLU-S2
3   0
4   0
5   0
6   0
7   0
8   0
9   0
FHU-RIB:LYS-S1
3   0
4   0
5   0
6   0
7   0
8   0
9   0
QUO-M6:ASP-S1
3   0
4   0
5   0
6   0
7   0
8   0
9   0
A-R6:PHE-CA
3   0
4   0
5   3
6   5
7   8
8   13
9   20
G-R5:TRP-S1
3   0
4   0
5   0
6   2
7   3
8   5

9 9
U31-P:ASP-S1
3 0
4 0
5 0
6 0
7 0
8 0
9 0
G-P:LYS-S1
3 0
4 0
5 7
6 12
7 19
8 30
9 46
H2U-MY:TRP-S1
3 0
4 0
5 0
6 0
7 0
8 0
9 0
QUO-M5:LEU-S2
3 0
4 0
5 0
6 0
7 0
8 0
9 0
C-Y:TYR-CA
3 0
4 0
5 2
6 3
7 5
8 9
9 14
FHU-P:LEU-S2
3 0
4 0
5 0
6 0
7 0
8 0
9 0
A-R6:ALA-CA
3 0
4 0
5 7
6 12
7 18
8 29
9 44
U-P:PRO-CA

3 0
4 0
5 2
6 3
7 5
8 9
9 13
A-P:ARG-CA
3 0
4 0
5 5
6 9
7 15
8 24
9 36
A-P:TYR-S2
3 0
4 0
5 2
6 4
7 7
8 11
9 17
G-R6:GLY-CA
3 0
4 0
5 7
6 13
7 20
8 31
9 48
QUO-M5:ARG-S1
3 0
4 0
5 0
6 0
7 0
8 0
9 0
U-RIB:CYS-CA
3 0
4 0
5 0
6 0
7 0
8 2
9 3
U-P:ILE-S1
3 0
4 0
5 2
6 4
7 6
8 10
9 16
H2U-P:PHE-S1
3 0
4 0

5 0
6 0
7 0
8 0
9 0
G-R6:ASN-S1
3 0
4 0
5 4
6 7
7 11
8 17
9 26
H2U-RIB:ARG-S2
3 0
4 0
5 0
6 0
7 0
8 0
9 0
C-RIB:TYR-CA
3 0
4 0
5 0
6 3
7 5
8 9
9 14
G-RIB:ARG-S1
3 0
4 0
5 7
6 12
7 19
8 30
9 46
FHU-P:THR-S1
3 0
4 0
5 0
6 0
7 0
8 0
9 0
U-P:VAL-CA
3 0
4 0
5 0
6 5
7 9
8 14
9 21
DA-RIB:HIS-S2
3 0
4 0
5 0
6 0

7 0
8 0
9 0
C-Y:ASP-CA
3 0
4 0
5 0
6 7
7 11
8 17
9 26
A-R6:TRP-S1
3 0
4 0
5 1
6 1
7 3
8 0
9 7
GTP-RIB:GLY-CA
3 0
4 0
5 0
6 0
7 0
8 0
9 0
U31-MY:ASP-S1
3 0
4 0
5 0
6 0
7 0
8 0
9 0
FMU-MY:MET-CA
3 0
4 0
5 0
6 0
7 0
8 0
9 0
FHU-P:ASP-S2
3 0
4 0
5 0
6 0
7 0
8 0
9 0
U-P:ARG-CA
3 0
4 0
5 3
6 5
7 8
8 13

9 20
A-P:ASN-CA
3 0
4 0
5 3
6 5
7 8
8 14
9 21
DA-RIB:GLN-S2
3 0
4 0
5 0
6 0
7 0
8 0
9 0
U31-P:HIS-S1
3 0
4 0
5 0
6 0
7 0
8 0
9 0
QUO-P:SER-S1
3 0
4 0
5 0
6 0
7 0
8 0
9 0
A-R5:TYR-S2
3 0
4 0
5 2
6 4
7 7
8 11
9 17
C-Y:TYR-S2
3 0
4 0
5 2
6 3
7 5
8 9
9 14
DA-M5:ASN-CA
3 0
4 0
5 0
6 0
7 0
8 0
9 0
DA-RIB:VAL-S1

3 0
4 0
5 0
6 0
7 0
8 0
9 0
C-RIB:ALA-CA
3 0
4 0
5 5
6 9
7 14
8 23
9 35
C-P:PRO-CA
3 0
4 0
5 3
6 5
7 8
8 13
9 19
U-Y:ILE-CA
3 0
4 0
5 0
6 0
7 6
8 10
9 16
FHU-P:ARG-S1
3 0
4 0
5 0
6 0
7 0
8 0
9 0
U-Y:ALA-CA
3 0
4 0
5 3
6 6
7 10
8 16
9 24
A-RIB:HIS-S1
3 0
4 0
5 2
6 3
7 5
8 8
9 13
G-P:CYS-S1
3 0
4 0

5 0
6 2
7 3
8 0
9 7
U-Y:GLU-S2
3 0
4 0
5 3
6 6
7 9
8 15
9 23
A-R5:PRO-CA
3 0
4 0
5 3
6 6
7 10
8 16
9 25
A-RIB:CYS-CA
3 0
4 0
5 0
6 1
7 2
8 3
9 5
U34-P:HIS-S1
3 0
4 0
5 0
6 0
7 0
8 0
9 0
C31-P:THR-S1
3 0
4 0
5 0
6 0
7 0
8 0
9 0
GTP-M6:SER-CA
3 0
4 0
5 0
6 0
7 0
8 0
9 0
G-R5:GLN-S2
3 0
4 0
5 3
6 6

7 10
8 15
9 24
H2U-MY:LEU-S1
3 0
4 0
5 0
6 0
7 0
8 0
9 0
FMU-MY:PHE-S2
3 0
4 0
5 0
6 0
7 0
8 0
9 0
G-R6:PHE-S2
3 0
4 0
5 0
6 6
7 10
8 16
9 25
OMC-P:LYS-S1
3 0
4 0
5 0
6 0
7 0
8 0
9 0
C-RIB:MET-S2
3 0
4 0
5 1
6 2
7 4
8 7
9 10
A-RIB:GLY-CA
3 0
4 0
5 6
6 10
7 15
8 25
9 38
G-R5:TRP-CA
3 0
4 0
5 0
6 2
7 3
8 5

9 9
A-RIB:ASP-S2
3 0
4 0
5 5
6 8
7 13
8 21
9 32
5BU-MY:PRO-S1
3 0
4 0
5 0
6 0
7 0
8 0
9 0
C-P:GLU-CA
3 0
4 0
5 5
6 9
7 14
8 22
9 34
G-R5:ASP-S2
3 0
4 0
5 6
6 11
7 17
8 26
9 40
QUO-RIB:ASN-S2
3 0
4 0
5 0
6 0
7 0
8 0
9 0
FMU-MY:GLU-S2
3 0
4 0
5 0
6 0
7 0
8 0
9 0
QUO-RIB:LEU-S1
3 0
4 0
5 0
6 0
7 0
8 0
9 0
H2U-RIB:ARG-S1

3 0
4 0
5 0
6 0
7 0
8 0
9 0
M2G-P:GLU-S2
3 0
4 0
5 0
6 0
7 0
8 0
9 0
G-RIB:LEU-CA
3 0
4 0
5 9
6 16
7 24
8 39
9 59
A-P:THR-CA
3 0
4 0
5 4
6 7
7 11
8 17
9 27
U-Y:ASP-S1
3 0
4 0
5 2
6 4
7 7
8 11
9 18
C31-RIB:TYR-S1
3 0
4 0
5 0
6 0
7 0
8 0
9 0
C-RIB:HIS-CA
3 0
4 0
5 1
6 2
7 4
8 6
9 10
G-RIB:ASP-S1
3 0
4 0

5 6
6 11
7 17
8 27
9 41
U31-MY:PHE-S1
3 0
4 0
5 0
6 0
7 0
8 0
9 0
U-Y:ILE-S1
3 0
4 0
5 2
6 4
7 6
8 10
9 16
U-RIB:GLU-S1
3 0
4 0
5 3
6 6
7 10
8 15
9 24
U-P:HIS-S1
3 0
4 0
5 0
6 1
7 3
8 4
9 7
A-P:PRO-S1
3 0
4 0
5 3
6 6
7 10
8 16
9 25
A-R5:MET-CA
3 0
4 0
5 2
6 3
7 5
8 8
9 13
H2U-P:PRO-S1
3 0
4 0
5 0
6 0

7 0
8 0
9 0
A-P:HIS-CA
3 0
4 0
5 2
6 3
7 5
8 8
9 13
H2U-MY:ARG-CA
3 0
4 0
5 0
6 0
7 0
8 0
9 0
G-R6:PRO-CA
3 0
4 0
5 5
6 8
7 13
8 20
9 31
U31-RIB:ASP-S2
3 0
4 0
5 0
6 0
7 0
8 0
9 0
C-Y:MET-CA
3 0
4 0
5 1
6 0
7 4
8 7
9 10
A-R6:LEU-CA
3 0
4 0
5 7
6 12
7 19
8 31
9 47
FMU-MY:CYS-S1
3 0
4 0
5 0
6 0
7 0
8 0

9   0
C-Y:LYS-S2
3   0
4   0
5   4
6   8
7   12
8   19
9   29
A-R6:HIS-CA
3   0
4   0
5   2
6   3
7   5
8   8
9   13
A-R6:ASP-CA
3   0
4   0
5   5
6   9
7   13
8   21
9   33
C-P:ASP-CA
3   0
4   0
5   4
6   7
7   11
8   17
9   26
C-RIB:ALA-S1
3   0
4   0
5   5
6   9
7   14
8   23
9   35
A-RIB:HIS-S2
3   0
4   0
5   2
6   3
7   5
8   8
9   13
FHU-MY:TYR-CA
3   0
4   0
5   0
6   0
7   0
8   0
9   0
IU-P:SER-CA

3 0
4 0
5 0
6 0
7 0
8 0
9 0
U34-RIB:GLY-CA
3 0
4 0
5 0
6 0
7 0
8 0
9 0
A-RIB:THR-CA
3 0
4 0
5 4
6 7
7 11
8 17
9 27
C31-MY:GLU-S2
3 0
4 0
5 0
6 0
7 0
8 0
9 0
C-RIB:LYS-CA
3 0
4 0
5 4
6 8
7 12
8 19
9 30
G-R6:SER-S1
3 0
4 0
5 5
6 10
7 15
8 24
9 37
C31-P:ASN-S2
3 0
4 0
5 0
6 0
7 0
8 0
9 0
C-Y:ARG-CA
3 0
4 0

5 0
6 7
7 12
8 19
9 29
U-RIB:PRO-S1
3 0
4 0
5 2
6 3
7 5
8 9
9 13
IU-RIB:LEU-CA
3 0
4 0
5 0
6 0
7 0
8 0
9 0
U-RIB:HIS-CA
3 0
4 0
5 1
6 1
7 3
8 4
9 7
C-RIB:GLU-CA
3 0
4 0
5 5
6 9
7 14
8 23
9 35
A-P:PHE-CA
3 0
4 0
5 0
6 5
7 8
8 13
9 20
G-RIB:LYS-S1
3 0
4 0
5 7
6 12
7 19
8 31
9 47
U-P:ARG-S1
3 0
4 0
5 3
6 5

7 8
8 13
9 20
C-P:GLY-CA
3 0
4 0
5 4
6 8
7 12
8 20
9 30
C31-MY:GLN-S1
3 0
4 0
5 0
6 0
7 0
8 0
9 0
C31-MY:PHE-S2
3 0
4 0
5 0
6 0
7 0
8 0
9 0
H2U-MY:PRO-CA
3 0
4 0
5 0
6 0
7 0
8 0
9 0
FHU-MY:PRO-S1
3 0
4 0
5 0
6 0
7 0
8 0
9 0
A-RIB:CYS-S1
3 0
4 0
5 0
6 1
7 2
8 3
9 5
H2U-RIB:GLU-CA
3 0
4 0
5 0
6 0
7 0
8 0

9 0
IU-MY:VAL-S1
3 0
4 0
5 0
6 0
7 0
8 0
9 0
G-P:VAL-S1
3 0
4 0
5 7
6 13
7 20
8 32
9 49
DA-RIB:ASP-CA
3 0
4 0
5 0
6 0
7 0
8 0
9 0
A-P:PRO-CA
3 0
4 0
5 3
6 6
7 10
8 16
9 25
G-R5:SER-S1
3 0
4 0
5 5
6 10
7 15
8 24
9 37
FHU-P:VAL-CA
3 0
4 0
5 0
6 0
7 0
8 0
9 0
U34-P:HIS-S2
3 0
4 0
5 0
6 0
7 0
8 0
9 0
U-P:MET-S2

3 0
4 0
5 0
6 2
7 3
8 4
9 7
G-RIB:LYS-S2
3 0
4 0
5 7
6 12
7 19
8 30
9 46
FHU-RIB:LYS-S2
3 0
4 0
5 0
6 0
7 0
8 0
9 0
H2U-P:GLU-S1
3 0
4 0
5 0
6 0
7 0
8 0
9 0
G-R6:PRO-S1
3 0
4 0
5 5
6 8
7 13
8 20
9 31
U-RIB:GLU-CA
3 0
4 0
5 3
6 6
7 10
8 15
9 24
U-Y:ASP-CA
3 0
4 0
5 0
6 4
7 7
8 12
9 18
DA-RIB:TYR-S1
3 0
4 0

5 0
6 0
7 0
8 0
9 0
C-Y:PHE-S2
3 0
4 0
5 2
6 4
7 6
8 10
9 16
FMU-MY:MET-S1
3 0
4 0
5 0
6 0
7 0
8 0
9 0
C-P:PHE-S1
3 0
4 0
5 0
6 4
7 6
8 10
9 16
U-Y:PRO-S1
3 0
4 0
5 2
6 3
7 5
8 9
9 13
U-Y:GLU-CA
3 0
4 0
5 0
6 6
7 10
8 15
9 24
A-R5:TRP-CA
3 0
4 0
5 0
6 1
7 3
8 4
9 7
FHU-MY:CYS-S1
3 0
4 0
5 0
6 0

7	0
8	0
9	0

U-P:CYS-S1

3	0
4	0
5	0
6	0
7	1
8	2
9	3

IU-P:LEU-CA

3	0
4	0
5	0
6	0
7	0
8	0
9	0

U31-P:ASN-S1

3	0
4	0
5	0
6	0
7	0
8	0
9	0

H2U-MY:PHE-CA

3	0
4	0
5	0
6	0
7	0
8	0
9	0

G-R5:VAL-S1

3	0
4	0
5	7
6	13
7	20
8	32
9	49

DA-M6:LYS-CA

3	0
4	0
5	0
6	0
7	0
8	0
9	0

A-R5:MET-S2

3	0
4	0
5	2
6	3
7	5
8	8

9 13
U31-RIB:GLN-CA
3 0
4 0
5 0
6 0
7 0
8 0
9 0
IU-P:LYS-S1
3 0
4 0
5 0
6 0
7 0
8 0
9 0
U-P:GLN-S1
3 0
4 0
5 1
6 2
7 4
8 7
9 10
A-R5:LYS-S1
3 0
4 0
5 6
6 10
7 15
8 24
9 37
QUO-M6:LEU-S1
3 0
4 0
5 0
6 0
7 0
8 0
9 0
C-P:LYS-CA
3 0
4 0
5 4
6 8
7 12
8 19
9 30
I-P:TRP-S2
3 0
4 0
5 0
6 0
7 0
8 0
9 0
G-P:LYS-CA

3 0
4 0
5 7
6 12
7 19
8 30
9 47
FMU-P:ARG-S2
3 0
4 0
5 0
6 0
7 0
8 0
9 0
G-RIB:LYS-CA
3 0
4 0
5 7
6 12
7 19
8 31
9 47
G-RIB:MET-S1
3 0
4 0
5 0
6 4
7 7
8 11
9 16
FMU-MY:ILE-CA
3 0
4 0
5 0
6 0
7 0
8 0
9 0
DA-RIB:GLN-CA
3 0
4 0
5 0
6 0
7 0
8 0
9 0
FHU-MY:TYR-S1
3 0
4 0
5 0
6 0
7 0
8 0
9 0
OMC-MY:LYS-S2
3 0
4 0

5 0
6 0
7 0
8 0
9 0
G-P:TYR-S2
3 0
4 0
5 3
6 5
7 9
8 14
9 21
A-P:ASP-S2
3 0
4 0
5 5
6 8
7 13
8 21
9 32
U-Y:THR-CA
3 0
4 0
5 2
6 4
7 6
8 9
9 14
FMU-MY:VAL-CA
3 0
4 0
5 0
6 0
7 0
8 0
9 0
C31-P:GLU-S2
3 0
4 0
5 0
6 0
7 0
8 0
9 0
QUO-RIB:LYS-S2
3 0
4 0
5 0
6 0
7 0
8 0
9 0
A-R5:GLU-S2
3 0
4 0
5 6
6 11

7 18
8 28
9 43
U31-RIB:TYR-S2
3 0
4 0
5 0
6 0
7 0
8 0
9 0
U31-MY:GLN-S1
3 0
4 0
5 0
6 0
7 0
8 0
9 0
G-P:SER-S1
3 0
4 0
5 5
6 10
7 15
8 24
9 37
C-RIB:ARG-S1
3 0
4 0
5 4
6 7
7 12
8 19
9 29
DA-M5:MET-S2
3 0
4 0
5 0
6 0
7 0
8 0
9 0
C31-RIB:LEU-S1
3 0
4 0
5 0
6 0
7 0
8 0
9 0
C-RIB:GLU-S1
3 0
4 0
5 5
6 9
7 14
8 22

9 35
U-RIB:HIS-S1
3 0
4 0
5 1
6 1
7 3
8 4
9 7
G-R5:GLN-S1
3 0
4 0
5 3
6 6
7 10
8 16
9 24
IU-MY:GLU-S2
3 0
4 0
5 0
6 0
7 0
8 0
9 0
IU-MY:ARG-S2
3 0
4 0
5 0
6 0
7 0
8 0
9 0
A-R6:PRO-S1
3 0
4 0
5 3
6 6
7 10
8 16
9 25
C31-P:ALA-CA
3 0
4 0
5 0
6 0
7 0
8 0
9 0
IU-RIB:ALA-CA
3 0
4 0
5 0
6 0
7 0
8 0
9 0
A-P:THR-S1

3 0
4 0
5 4
6 7
7 11
8 17
9 27
A-R5:ARG-S1
3 0
4 0
5 5
6 9
7 15
8 24
9 36
GTP-RIB:ASN-S2
3 0
4 0
5 0
6 0
7 0
8 0
9 0
DA-M5:GLU-S1
3 0
4 0
5 0
6 0
7 0
8 0
9 0
U31-P:LEU-S1
3 0
4 0
5 0
6 0
7 0
8 0
9 0
U-RIB:VAL-S1
3 0
4 0
5 3
6 5
7 9
8 14
9 21
C-RIB:VAL-CA
3 0
4 0
5 5
6 8
7 13
8 20
9 31
C-RIB:ILE-S1
3 0
4 0

5 3
6 6
7 10
8 15
9 24
A-R6:CYS-S1
3 0
4 0
5 0
6 1
7 2
8 3
9 0
DA-M5:HIS-S2
3 0
4 0
5 0
6 0
7 0
8 0
9 0
FHU-P:TYR-CA
3 0
4 0
5 0
6 0
7 0
8 0
9 0
G-R6:LEU-CA
3 0
4 0
5 9
6 16
7 24
8 39
9 59
A-R5:ASP-S1
3 0
4 0
5 5
6 9
7 13
8 21
9 33
H2U-P:PRO-CA
3 0
4 0
5 0
6 0
7 0
8 0
9 0
DA-M6:LEU-S1
3 0
4 0
5 0
6 0

7 0
8 0
9 0
GTP-M5:SER-S1
3 0
4 0
5 0
6 0
7 0
8 0
9 0
C-Y:SER-S1
3 0
4 0
5 3
6 6
7 9
8 15
9 23
C-Y:MET-S1
3 0
4 0
5 1
6 2
7 4
8 7
9 10
G-R5:THR-CA
3 0
4 0
5 0
6 9
7 14
8 22
9 34
A-P:ARG-S1
3 0
4 0
5 5
6 9
7 15
8 24
9 36
A-R5:LYS-CA
3 0
4 0
5 0
6 10
7 15
8 24
9 37
U-P:GLU-S2
3 0
4 0
5 0
6 6
7 9
8 15

9 23
C31-P:SER-S1
3 0
4 0
5 0
6 0
7 0
8 0
9 0
DA-M6:ASN-CA
3 0
4 0
5 0
6 0
7 0
8 0
9 0
U-Y:PHE-S2
3 0
4 0
5 1
6 3
7 4
8 7
9 11
FHU-P:SER-CA
3 0
4 0
5 0
6 0
7 0
8 0
9 0
G-RIB:VAL-S1
3 0
4 0
5 7
6 13
7 20
8 32
9 49
C-Y:TYR-S1
3 0
4 0
5 2
6 3
7 5
8 9
9 14
OMC-P:LYS-CA
3 0
4 0
5 0
6 0
7 0
8 0
9 0
G-RIB:THR-S1

3 0
4 0
5 5
6 9
7 14
8 22
9 34
G-R5:CYS-CA
3 0
4 0
5 0
6 0
7 0
8 4
9 7
FMU-RIB:PHE-CA
3 0
4 0
5 0
6 0
7 0
8 0
9 0
U34-MY:PHE-CA
3 0
4 0
5 0
6 0
7 0
8 0
9 0
DA-M6:ASN-S1
3 0
4 0
5 0
6 0
7 0
8 0
9 0
C-RIB:THR-S1
3 0
4 0
5 3
6 5
7 9
8 14
9 21
FHU-P:LEU-S1
3 0
4 0
5 0
6 0
7 0
8 0
9 0
G-R6:LYS-CA
3 0
4 0

5   7
6   12
7   19
8   31
9   47
H2U-MY:THR-CA
3   0
4   0
5   0
6   0
7   0
8   0
9   0
U-Y:LYS-S2
3   0
4   0
5   0
6   5
7   8
8   13
9   20
FMU-MY:PHE-CA
3   0
4   0
5   0
6   0
7   0
8   0
9   0
C-P:VAL-CA
3   0
4   0
5   5
6   8
7   13
8   20
9   31
DA-M6:GLU-S1
3   0
4   0
5   0
6   0
7   0
8   0
9   0
C-P:PHE-CA
3   0
4   0
5   0
6   0
7   6
8   10
9   16
H2U-P:ARG-S2
3   0
4   0
5   0
6   0

7 0
8 0
9 0
U-Y:GLN-S2
3 0
4 0
5 1
6 2
7 4
8 6
9 10
G-P:ILE-CA
3 0
4 0
5 5
6 0
7 15
8 24
9 37
C-RIB:ILE-CA
3 0
4 0
5 0
6 6
7 10
8 15
9 24
DA-M5:TYR-CA
3 0
4 0
5 0
6 0
7 0
8 0
9 0
A-R5:ALA-CA
3 0
4 0
5 7
6 12
7 18
8 29
9 44
FHU-P:TYR-S1
3 0
4 0
5 0
6 0
7 0
8 0
9 0
IU-P:LYS-CA
3 0
4 0
5 0
6 0
7 0
8 0

9 0
IU-P:ALA-CA
3 0
4 0
5 0
6 0
7 0
8 0
9 0
H2U-MY:ASN-S1
3 0
4 0
5 0
6 0
7 0
8 0
9 0
U-P:ASN-S1
3 0
4 0
5 1
6 3
7 4
8 7
9 11
C31-P:MET-S1
3 0
4 0
5 0
6 0
7 0
8 0
9 0
U-Y:LEU-S1
3 0
4 0
5 0
6 7
7 10
8 17
9 26
U31-MY:PHE-S2
3 0
4 0
5 0
6 0
7 0
8 0
9 0
IU-P:ILE-S1
3 0
4 0
5 0
6 0
7 0
8 0
9 0
G-RIB:TRP-S1

3 0
4 0
5 1
6 2
7 3
8 5
9 9
A-R5:LEU-S1
3 0
4 0
5 0
6 12
7 19
8 31
9 47
G-P:ASN-S1
3 0
4 0
5 4
6 7
7 11
8 17
9 26
FMU-MY:VAL-S1
3 0
4 0
5 0
6 0
7 0
8 0
9 0
A-R6:TYR-CA
3 0
4 0
5 2
6 4
7 7
8 11
9 17
G-R6:GLU-S1
3 0
4 0
5 0
6 14
7 22
8 36
9 54
GTP-M5:GLY-CA
3 0
4 0
5 0
6 0
7 0
8 0
9 0
IU-RIB:ARG-S1
3 0
4 0

5 0
6 0
7 0
8 0
9 0
G-R5:HIS-CA
3 0
4 0
5 2
6 4
7 6
8 10
9 16
H2U-MY:PRO-S1
3 0
4 0
5 0
6 0
7 0
8 0
9 0
C31-MY:LEU-S2
3 0
4 0
5 0
6 0
7 0
8 0
9 0
FHU-RIB:ASP-S1
3 0
4 0
5 0
6 0
7 0
8 0
9 0
U-P:TRP-S2
3 0
4 0
5 0
6 1
7 1
8 2
9 3
C31-MY:GLU-S1
3 0
4 0
5 0
6 0
7 0
8 0
9 0
DA-M6:LYS-S2
3 0
4 0
5 0
6 0

7 0
8 0
9 0
5BU-P:ARG-CA
3 0
4 0
5 0
6 0
7 0
8 0
9 0
FMU-MY:GLN-S1
3 0
4 0
5 0
6 0
7 0
8 0
9 0
A-RIB:PRO-CA
3 0
4 0
5 3
6 6
7 10
8 16
9 25
QUO-RIB:LEU-S2
3 0
4 0
5 0
6 0
7 0
8 0
9 0
A-P:MET-CA
3 0
4 0
5 2
6 3
7 5
8 8
9 13
A-RIB:LYS-CA
3 0
4 0
5 6
6 10
7 15
8 24
9 37
U31-RIB:ASN-S1
3 0
4 0
5 0
6 0
7 0
8 0

9 0
FMU-P:PHE-CA
3 0
4 0
5 0
6 0
7 0
8 0
9 0
C31-RIB:PHE-S2
3 0
4 0
5 0
6 0
7 0
8 0
9 0
A-RIB:ARG-S2
3 0
4 0
5 5
6 9
7 15
8 23
9 36
U-RIB:LEU-CA
3 0
4 0
5 4
6 7
7 10
8 17
9 26
A-R5:ASP-CA
3 0
4 0
5 5
6 9
7 13
8 21
9 33
G-RIB:ASP-S2
3 0
4 0
5 6
6 11
7 17
8 26
9 40
A-R6:ASN-S2
3 0
4 0
5 3
6 5
7 8
8 13
9 21
A-R6:LYS-CA

3 0
4 0
5 6
6 10
7 15
8 24
9 37
G-R6:TYR-CA
3 0
4 0
5 0
6 6
7 9
8 14
9 22
C-RIB:ASN-S1
3 0
4 0
5 2
6 4
7 7
8 11
9 17
G-RIB:ARG-CA
3 0
4 0
5 7
6 12
7 19
8 30
9 46
C31-P:PHE-S1
3 0
4 0
5 0
6 0
7 0
8 0
9 0
G-R6:ASP-CA
3 0
4 0
5 0
6 11
7 17
8 27
9 41
GTP-RIB:SER-S1
3 0
4 0
5 0
6 0
7 0
8 0
9 0
G-R5:CYS-S1
3 0
4 0

5 0
6 0
7 3
8 4
9 7
C31-RIB:GLU-S2
3 0
4 0
5 0
6 0
7 0
8 0
9 0
G-P:PHE-S2
3 0
4 0
5 0
6 6
7 10
8 16
9 25
G-R5:LEU-S1
3 0
4 0
5 9
6 16
7 24
8 39
9 59
G-P:ASP-S2
3 0
4 0
5 6
6 10
7 16
8 26
9 40
G-R5:TYR-CA
3 0
4 0
5 3
6 0
7 9
8 14
9 22
FHU-MY:VAL-S1
3 0
4 0
5 0
6 0
7 0
8 0
9 0
FMU-RIB:ASP-S2
3 0
4 0
5 0
6 0

7 0
8 0
9 0
FMU-MY:MET-S2
3 0
4 0
5 0
6 0
7 0
8 0
9 0
FHU-MY:ASP-S1
3 0
4 0
5 0
6 0
7 0
8 0
9 0
FMU-RIB:CYS-S1
3 0
4 0
5 0
6 0
7 0
8 0
9 0
U-Y:ASN-S2
3 0
4 0
5 1
6 3
7 4
8 7
9 11
G-P:ASP-S1
3 0
4 0
5 6
6 11
7 17
8 27
9 41
G-P:GLY-CA
3 0
4 0
5 7
6 12
7 19
8 31
9 47
G-RIB:ILE-CA
3 0
4 0
5 6
6 10
7 15
8 24

9 37
H2U-RIB:PRO-CA
3 0
4 0
5 0
6 0
7 0
8 0
9 0
FMU-MY:SER-S1
3 0
4 0
5 0
6 0
7 0
8 0
9 0
G-R5:HIS-S1
3 0
4 0
5 2
6 4
7 6
8 10
9 16
A-R6:ILE-CA
3 0
4 0
5 4
6 8
7 12
8 19
9 30
G-RIB:SER-CA
3 0
4 0
5 5
6 10
7 15
8 24
9 37
G-R6:THR-CA
3 0
4 0
5 0
6 9
7 14
8 22
9 34
A-P:LEU-S2
3 0
4 0
5 7
6 12
7 19
8 31
9 47
FHU-RIB:LEU-S2

3 0
4 0
5 0
6 0
7 0
8 0
9 0
H2U-P:ASN-S1
3 0
4 0
5 0
6 0
7 0
8 0
9 0
QUO-M6:LEU-CA
3 0
4 0
5 0
6 0
7 0
8 0
9 0
U31-P:SER-CA
3 0
4 0
5 0
6 0
7 0
8 0
9 0
C31-P:ASN-S1
3 0
4 0
5 0
6 0
7 0
8 0
9 0
A-P:MET-S2
3 0
4 0
5 2
6 3
7 5
8 8
9 13
H2U-RIB:TRP-S2
3 0
4 0
5 0
6 0
7 0
8 0
9 0
IU-P:LYS-S2
3 0
4 0

5 0
6 0
7 0
8 0
9 0
GTP-RIB:ASN-CA
3 0
4 0
5 0
6 0
7 0
8 0
9 0
G-R6:LYS-S1
3 0
4 0
5 7
6 12
7 19
8 31
9 47
GTP-RIB:ARG-S1
3 0
4 0
5 0
6 0
7 0
8 0
9 0
U-RIB:GLY-CA
3 0
4 0
5 3
6 5
7 8
8 13
9 21
C31-P:MET-S2
3 0
4 0
5 0
6 0
7 0
8 0
9 0
C-Y:LEU-S1
3 0
4 0
5 6
6 10
7 15
8 24
9 38
G-R6:TYR-S1
3 0
4 0
5 3
6 6

7 9
8 14
9 22
U-P:TRP-CA
3 0
4 0
5 0
6 1
7 1
8 2
9 3
C31-P:PHE-CA
3 0
4 0
5 0
6 0
7 0
8 0
9 0
FHU-RIB:ALA-CA
3 0
4 0
5 0
6 0
7 0
8 0
9 0
C-Y:ARG-S1
3 0
4 0
5 4
6 7
7 12
8 19
9 29
G-P:TYR-CA
3 0
4 0
5 3
6 5
7 9
8 14
9 21
FMU-RIB:GLU-S1
3 0
4 0
5 0
6 0
7 0
8 0
9 0
U31-RIB:TYR-S1
3 0
4 0
5 0
6 0
7 0
8 0

9 0
U-RIB:TYR-S2
3 0
4 0
5 1
6 2
7 4
8 6
9 9
G-RIB:ASN-CA
3 0
4 0
5 4
6 7
7 11
8 17
9 26
DA-RIB:ASP-S1
3 0
4 0
5 0
6 0
7 0
8 0
9 0
U-RIB:LEU-S1
3 0
4 0
5 4
6 7
7 10
8 17
9 26
QUO-RIB:GLN-S2
3 0
4 0
5 0
6 0
7 0
8 0
9 0
FHU-MY:ILE-CA
3 0
4 0
5 0
6 0
7 0
8 0
9 0
A-R5:ARG-S2
3 0
4 0
5 5
6 9
7 15
8 23
9 36
U-RIB:TRP-CA

3 0
4 0
5 0
6 1
7 1
8 2
9 4
QUO-M5:GLN-S2
3 0
4 0
5 0
6 0
7 0
8 0
9 0
H2U-P:THR-S1
3 0
4 0
5 0
6 0
7 0
8 0
9 0
A-R6:ARG-S2
3 0
4 0
5 5
6 9
7 15
8 23
9 36
A-RIB:GLN-CA
3 0
4 0
5 3
6 5
7 8
8 12
9 19
IU-MY:LEU-S2
3 0
4 0
5 0
6 0
7 0
8 0
9 0
G-RIB:TYR-S2
3 0
4 0
5 0
6 6
7 9
8 14
9 22
H2U-MY:THR-S1
3 0
4 0

5 0
6 0
7 0
8 0
9 0
IU-RIB:SER-CA
3 0
4 0
5 0
6 0
7 0
8 0
9 0
G-R5:ASN-S1
3 0
4 0
5 4
6 7
7 11
8 17
9 26
G-R6:HIS-S1
3 0
4 0
5 2
6 4
7 6
8 10
9 16
FHU-RIB:PRO-S1
3 0
4 0
5 0
6 0
7 0
8 0
9 0
DA-M5:HIS-S1
3 0
4 0
5 0
6 0
7 0
8 0
9 0
A-P:LYS-S2
3 0
4 0
5 5
6 10
7 15
8 24
9 37
FHU-MY:TYR-S2
3 0
4 0
5 0
6 0

7 0
8 0
9 0
U34-MY:ASP-CA
3 0
4 0
5 0
6 0
7 0
8 0
9 0
A-R5:ASP-S2
3 0
4 0
5 5
6 8
7 13
8 21
9 32
C-P:ASP-S1
3 0
4 0
5 4
6 7
7 11
8 17
9 26
DA-M6:HIS-S1
3 0
4 0
5 0
6 0
7 0
8 0
9 0
DA-M6:LEU-S2
3 0
4 0
5 0
6 0
7 0
8 0
9 0
G-P:TRP-S2
3 0
4 0
5 0
6 2
7 3
8 5
9 9
A-R6:TYR-S1
3 0
4 0
5 2
6 4
7 7
8 11

9   17
DA-M6:TYR-S2
3   0
4   0
5   0
6   0
7   0
8   0
9   0
FHU-RIB:TYR-S2
3   0
4   0
5   0
6   0
7   0
8   0
9   0
G-R5:ASN-S2
3   0
4   0
5   4
6   7
7   11
8   17
9   26
FHU-RIB:GLY-CA
3   0
4   0
5   0
6   0
7   0
8   0
9   0
C31-P:GLU-S1
3   0
4   0
5   0
6   0
7   0
8   0
9   0
G-R5:GLN-CA
3   0
4   0
5   0
6   0
7   10
8   16
9   24
FHU-RIB:ALA-S1
3   0
4   0
5   0
6   0
7   0
8   0
9   0
C-Y:ASN-CA

3 0
4 0
5 2
6 4
7 7
8 11
9 17
U-Y:ASN-S1
3 0
4 0
5 1
6 3
7 4
8 7
9 11
C-P:ARG-CA
3 0
4 0
5 4
6 7
7 12
8 19
9 29
A-R6:VAL-S1
3 0
4 0
5 6
6 10
7 16
8 26
9 39
5BU-MY:ILE-S1
3 0
4 0
5 0
6 0
7 0
8 0
9 0
QUO-M5:ASN-S2
3 0
4 0
5 0
6 0
7 0
8 0
9 0
G-P:GLU-S1
3 0
4 0
5 8
6 14
7 22
8 35
9 54
A-R6:THR-CA
3 0
4 0

5	4
6	7
7	11
8	17
9	27

G-R5:ILE-S1

3	0
4	0
5	6
6	10
7	0
8	24
9	37

A-R6:SER-S1

3	0
4	0
5	4
6	8
7	12
8	19
9	29

U-Y:LYS-S1

3	0
4	0
5	3
6	5
7	8
8	13
9	20

U-Y:MET-CA

3	0
4	0
5	1
6	2
7	3
8	4
9	7

C-RIB:GLY-CA

3	0
4	0
5	4
6	8
7	12
8	20
9	30

DA-M6:ASN-S2

3	0
4	0
5	0
6	0
7	0
8	0
9	0

U31-MY:TYR-S1

3	0
4	0
5	0
6	0

7 0
8 0
9 0
A-R5:GLN-S2
3 0
4 0
5 3
6 5
7 8
8 12
9 19
FHU-P:CYS-S1
3 0
4 0
5 0
6 0
7 0
8 0
9 0
U-Y:ARG-S1
3 0
4 0
5 3
6 5
7 8
8 13
9 20
C-Y:GLY-CA
3 0
4 0
5 4
6 8
7 12
8 20
9 30
OMC-P:LYS-S2
3 0
4 0
5 0
6 0
7 0
8 0
9 0
C31-MY:PHE-S1
3 0
4 0
5 0
6 0
7 0
8 0
9 0
C-RIB:ASN-CA
3 0
4 0
5 2
6 4
7 7
8 11

9 17
A-R5:PHE-S2
3 0
4 0
5 3
6 5
7 8
8 13
9 20
A-R6:VAL-CA
3 0
4 0
5 6
6 10
7 16
8 26
9 39
U-RIB:PHE-S2
3 0
4 0
5 1
6 3
7 4
8 7
9 11
U34-MY:PRO-CA
3 0
4 0
5 0
6 0
7 0
8 0
9 0
C-Y:MET-S2
3 0
4 0
5 1
6 2
7 4
8 7
9 10
G-R5:TYR-S1
3 0
4 0
5 0
6 6
7 9
8 14
9 22
QUO-M5:PHE-S1
3 0
4 0
5 0
6 0
7 0
8 0
9 0
DA-RIB:ALA-CA

3 0
4 0
5 0
6 0
7 0
8 0
9 0
FHU-RIB:THR-CA
3 0
4 0
5 0
6 0
7 0
8 0
9 0
FMU-MY:ASN-CA
3 0
4 0
5 0
6 0
7 0
8 0
9 0
U31-P:ASN-S2
3 0
4 0
5 0
6 0
7 0
8 0
9 0
U-P:THR-S1
3 0
4 0
5 2
6 4
7 6
8 9
9 14
H2U-MY:GLN-S2
3 0
4 0
5 0
6 0
7 0
8 0
9 0
G-RIB:CYS-S1
3 0
4 0
5 0
6 0
7 3
8 0
9 7
H2U-P:ARG-S1
3 0
4 0

5 0
6 0
7 0
8 0
9 0
U-Y:PHE-S1
3 0
4 0
5 1
6 3
7 4
8 7
9 11
FHU-MY:ARG-S2
3 0
4 0
5 0
6 0
7 0
8 0
9 0
A-RIB:THR-S1
3 0
4 0
5 4
6 7
7 11
8 17
9 27
C31-P:TYR-S1
3 0
4 0
5 0
6 0
7 0
8 0
9 0
G-R6:THR-S1
3 0
4 0
5 0
6 9
7 14
8 22
9 34
IU-RIB:PRO-S1
3 0
4 0
5 0
6 0
7 0
8 0
9 0
H2U-P:ASN-CA
3 0
4 0
5 0
6 0

7 0
8 0
9 0
C-P:TYR-CA
3 0
4 0
5 2
6 0
7 5
8 9
9 14
C31-P:ASN-CA
3 0
4 0
5 0
6 0
7 0
8 0
9 0
U-Y:TRP-S1
3 0
4 0
5 0
6 1
7 1
8 2
9 4
C31-MY:TYR-S2
3 0
4 0
5 0
6 0
7 0
8 0
9 0
FHU-P:LYS-S1
3 0
4 0
5 0
6 0
7 0
8 0
9 0
A-R5:MET-S1
3 0
4 0
5 2
6 3
7 5
8 8
9 13
G-R6:LYS-S2
3 0
4 0
5 7
6 12
7 19
8 30

9   46
A-R6:ILE-S1
3   0
4   0
5   4
6   8
7   12
8   19
9   30
A-R6:GLU-CA
3   0
4   0
5   7
6   11
7   18
8   28
9   43
U31-MY:TYR-CA
3   0
4   0
5   0
6   0
7   0
8   0
9   0
FMU-RIB:MET-S2
3   0
4   0
5   0
6   0
7   0
8   0
9   0
U-Y:CYS-S1
3   0
4   0
5   0
6   0
7   0
8   2
9   3
H2U-MY:LYS-S2
3   0
4   0
5   0
6   0
7   0
8   0
9   0
U31-P:MET-CA
3   0
4   0
5   0
6   0
7   0
8   0
9   0
FMU-MY:GLU-S1

3 0
4 0
5 0
6 0
7 0
8 0
9 0
IU-P:HIS-S1
3 0
4 0
5 0
6 0
7 0
8 0
9 0
H2U-P:LEU-S1
3 0
4 0
5 0
6 0
7 0
8 0
9 0
A-P:TYR-CA
3 0
4 0
5 2
6 4
7 7
8 11
9 17
QUO-M6:ASN-S2
3 0
4 0
5 0
6 0
7 0
8 0
9 0
U-Y:LEU-CA
3 0
4 0
5 0
6 0
7 10
8 17
9 26
G-RIB:PRO-CA
3 0
4 0
5 5
6 8
7 13
8 20
9 31
A-R5:GLU-S1
3 0
4 0

5   6
6   11
7   18
8   28
9   43
A-RIB:TYR-S2
3   0
4   0
5   2
6   4
7   7
8   11
9   17
H2U-P:THR-CA
3   0
4   0
5   0
6   0
7   0
8   0
9   0
U-P:GLN-S2
3   0
4   0
5   1
6   2
7   4
8   6
9   10
C-P:ASN-S2
3   0
4   0
5   2
6   4
7   7
8   11
9   16
G-R5:GLU-S2
3   0
4   0
5   8
6   14
7   22
8   35
9   54
G-R6:GLN-S1
3   0
4   0
5   0
6   6
7   10
8   16
9   24
A-RIB:PHE-S2
3   0
4   0
5   3
6   5

7 8
8 13
9 20
DA-RIB:ALA-S1
3 0
4 0
5 0
6 0
7 0
8 0
9 0
A-R5:TYR-CA
3 0
4 0
5 0
6 4
7 7
8 11
9 17
A-R5:LEU-S2
3 0
4 0
5 7
6 12
7 19
8 31
9 47
U31-RIB:PHE-CA
3 0
4 0
5 0
6 0
7 0
8 0
9 0
H2U-MY:GLY-CA
3 0
4 0
5 0
6 0
7 0
8 0
9 0
PSU-RIB:ARG-S2
3 0
4 0
5 0
6 0
7 0
8 0
9 0
G-P:HIS-S2
3 0
4 0
5 2
6 4
7 6
8 10

9   16
C-P:LYS-S2
3   0
4   0
5   4
6   8
7   12
8   19
9   29
C-P:GLN-S1
3   0
4   0
5   2
6   4
7   6
8   10
9   15
U-P:GLU-CA
3   0
4   0
5   3
6   6
7   10
8   15
9   24
A-RIB:GLN-S1
3   0
4   0
5   0
6   5
7   8
8   12
9   19
IU-P:ARG-S1
3   0
4   0
5   0
6   0
7   0
8   0
9   0
U-RIB:TYR-CA
3   0
4   0
5   1
6   2
7   4
8   6
9   9
IU-MY:HIS-CA
3   0
4   0
5   0
6   0
7   0
8   0
9   0
DA-M5:TYR-S1

3 0
4 0
5 0
6 0
7 0
8 0
9 0
A-R6:THR-S1
3 0
4 0
5 4
6 7
7 11
8 17
9 27
C-Y:ASP-S1
3 0
4 0
5 4
6 7
7 11
8 17
9 26
FMU-P:PHE-S2
3 0
4 0
5 0
6 0
7 0
8 0
9 0
H2U-RIB:LYS-S1
3 0
4 0
5 0
6 0
7 0
8 0
9 0
U-RIB:ASN-S2
3 0
4 0
5 1
6 3
7 4
8 7
9 11
G-P:CYS-CA
3 0
4 0
5 0
6 0
7 0
8 4
9 7
QUO-M6:LEU-S2
3 0
4 0

5 0
6 0
7 0
8 0
9 0
C-RIB:PHE-S2
3 0
4 0
5 2
6 4
7 6
8 10
9 16
C-Y:ASN-S1
3 0
4 0
5 2
6 4
7 7
8 11
9 17
DA-RIB:LYS-S2
3 0
4 0
5 0
6 0
7 0
8 0
9 0
FMU-MY:GLN-CA
3 0
4 0
5 0
6 0
7 0
8 0
9 0
DA-M6:MET-S2
3 0
4 0
5 0
6 0
7 0
8 0
9 0
G-P:GLU-CA
3 0
4 0
5 8
6 14
7 22
8 35
9 54
H2U-RIB:PRO-S1
3 0
4 0
5 0
6 0

7 0
8 0
9 0
QUO-M5:ASN-CA
3 0
4 0
5 0
6 0
7 0
8 0
9 0
G-P:MET-S1
3 0
4 0
5 2
6 4
7 6
8 10
9 16
U31-MY:GLU-S2
3 0
4 0
5 0
6 0
7 0
8 0
9 0
A-RIB:ILE-S1
3 0
4 0
5 4
6 8
7 12
8 19
9 30
5BU-RIB:PRO-S1
3 0
4 0
5 0
6 0
7 0
8 0
9 0
U-Y:HIS-S1
3 0
4 0
5 1
6 1
7 3
8 4
9 7
U31-RIB:MET-S1
3 0
4 0
5 0
6 0
7 0
8 0

9 0
H2U-RIB:LYS-S2
3 0
4 0
5 0
6 0
7 0
8 0
9 0
G-RIB:VAL-CA
3 0
4 0
5 7
6 13
7 20
8 32
9 50
FHU-MY:LEU-S2
3 0
4 0
5 0
6 0
7 0
8 0
9 0
U34-MY:ASN-S1
3 0
4 0
5 0
6 0
7 0
8 0
9 0
A-RIB:MET-S1
3 0
4 0
5 0
6 3
7 5
8 8
9 13
G-RIB:GLY-CA
3 0
4 0
5 7
6 13
7 20
8 31
9 48
DA-M5:LEU-S1
3 0
4 0
5 0
6 0
7 0
8 0
9 0
U34-RIB:SER-CA

3 0
4 0
5 0
6 0
7 0
8 0
9 0
U-RIB:MET-S1
3 0
4 0
5 1
6 2
7 3
8 4
9 7
5BU-P:ALA-S1
3 0
4 0
5 0
6 0
7 0
8 0
9 0
U34-P:HIS-CA
3 0
4 0
5 0
6 0
7 0
8 0
9 0
C31-MY:ALA-S1
3 0
4 0
5 0
6 0
7 0
8 0
9 0
C-P:MET-S2
3 0
4 0
5 1
6 2
7 4
8 7
9 10
U-RIB:LEU-S2
3 0
4 0
5 4
6 7
7 10
8 17
9 26
U34-P:GLU-CA
3 0
4 0

5 0
6 0
7 0
8 0
9 0
U-Y:VAL-CA
3 0
4 0
5 0
6 5
7 9
8 14
9 21
H2U-RIB:ASN-S2
3 0
4 0
5 0
6 0
7 0
8 0
9 0
C-P:ALA-S1
3 0
4 0
5 5
6 9
7 14
8 23
9 35
C-Y:TRP-S1
3 0
4 0
5 0
6 1
7 2
8 3
9 5
DA-M5:ASP-S2
3 0
4 0
5 0
6 0
7 0
8 0
9 0
FMU-MY:ALA-CA
3 0
4 0
5 0
6 0
7 0
8 0
9 0
U31-P:GLN-CA
3 0
4 0
5 0
6 0

7   0
8   0
9   0
G-R5:MET-S1
3   0
4   0
5   0
6   4
7   7
8   11
9   16
H2U-RIB:GLY-CA
3   0
4   0
5   0
6   0
7   0
8   0
9   0
IU-P:HIS-CA
3   0
4   0
5   0
6   0
7   0
8   0
9   0
U-P:PHE-S2
3   0
4   0
5   0
6   3
7   4
8   7
9   11
C31-RIB:SER-S1
3   0
4   0
5   0
6   0
7   0
8   0
9   0
G-P:LEU-S1
3   0
4   0
5   9
6   16
7   24
8   38
9   59
G-P:ASN-S2
3   0
4   0
5   4
6   7
7   11
8   17

9   26
FHU-RIB:TYR-CA
3   0
4   0
5   0
6   0
7   0
8   0
9   0
C31-MY:GLU-CA
3   0
4   0
5   0
6   0
7   0
8   0
9   0
DA-RIB:ASN-CA
3   0
4   0
5   0
6   0
7   0
8   0
9   0
G-P:MET-CA
3   0
4   0
5   2
6   4
7   6
8   10
9   16
C-Y:VAL-CA
3   0
4   0
5   5
6   8
7   13
8   20
9   31
C-Y:PRO-S1
3   0
4   0
5   3
6   5
7   8
8   13
9   20
U31-P:MET-S2
3   0
4   0
5   0
6   0
7   0
8   0
9   0
U-Y:HIS-CA

3 0
4 0
5 0
6 1
7 3
8 4
9 7
IU-P:ARG-CA
3 0
4 0
5 0
6 0
7 0
8 0
9 0
G-R5:PHE-CA
3 0
4 0
5 0
6 0
7 10
8 16
9 25
A-R6:ASN-S1
3 0
4 0
5 3
6 5
7 8
8 14
9 21
G-P:SER-CA
3 0
4 0
5 5
6 10
7 15
8 24
9 37
FMU-RIB:ALA-S1
3 0
4 0
5 0
6 0
7 0
8 0
9 0
FMU-MY:GLU-CA
3 0
4 0
5 0
6 0
7 0
8 0
9 0
FMU-P:VAL-S1
3 0
4 0

5 0
6 0
7 0
8 0
9 0
A-RIB:SER-CA
3 0
4 0
5 4
6 8
7 12
8 19
9 29
G-RIB:ASN-S1
3 0
4 0
5 4
6 7
7 11
8 17
9 26
FMU-P:CYS-S1
3 0
4 0
5 0
6 0
7 0
8 0
9 0
DA-M6:GLN-CA
3 0
4 0
5 0
6 0
7 0
8 0
9 0
G-P:ARG-S2
3 0
4 0
5 7
6 12
7 18
8 29
9 45
C-P:ASN-CA
3 0
4 0
5 2
6 4
7 7
8 11
9 17
IU-RIB:ILE-S1
3 0
4 0
5 0
6 0

7 0
8 0
9 0
IU-RIB:LYS-CA
3 0
4 0
5 0
6 0
7 0
8 0
9 0
G-RIB:TYR-S1
3 0
4 0
5 3
6 6
7 9
8 14
9 22
U-RIB:ILE-S1
3 0
4 0
5 2
6 4
7 6
8 10
9 16
QUO-P:LEU-S2
3 0
4 0
5 0
6 0
7 0
8 0
9 0
GTP-M6:GLY-CA
3 0
4 0
5 0
6 0
7 0
8 0
9 0
QUO-M5:PHE-S2
3 0
4 0
5 0
6 0
7 0
8 0
9 0
A-P:PHE-S1
3 0
4 0
5 3
6 5
7 8
8 13

9   20
G-R6:PHE-CA
3   0
4   0
5   0
6   6
7   10
8   16
9   25
DA-M6:TYR-S1
3   0
4   0
5   0
6   0
7   0
8   0
9   0
H2U-MY:PHE-S1
3   0
4   0
5   0
6   0
7   0
8   0
9   0
A-P:SER-CA
3   0
4   0
5   4
6   8
7   12
8   19
9   29
C-Y:TRP-CA
3   0
4   0
5   0
6   1
7   2
8   3
9   5
QUO-M5:ASP-S2
3   0
4   0
5   0
6   0
7   0
8   0
9   0
G-RIB:CYS-CA
3   0
4   0
5   1
6   0
7   3
8   4
9   7
U31-MY:ASP-S2

3 0
4 0
5 0
6 0
7 0
8 0
9 0
U-Y:TYR-S2
3 0
4 0
5 1
6 2
7 4
8 6
9 9
QUO-M5:ASN-S1
3 0
4 0
5 0
6 0
7 0
8 0
9 0
DA-RIB:HIS-S1
3 0
4 0
5 0
6 0
7 0
8 0
9 0
U31-RIB:ASP-CA
3 0
4 0
5 0
6 0
7 0
8 0
9 0
A-P:ARG-S2
3 0
4 0
5 5
6 9
7 15
8 23
9 36
U34-P:ASN-S2
3 0
4 0
5 0
6 0
7 0
8 0
9 0
QUO-M5:ASP-CA
3 0
4 0

5 0
6 0
7 0
8 0
9 0
U-P:SER-CA
3 0
4 0
5 2
6 4
7 6
8 10
9 16
GTP-M6:ASN-S1
3 0
4 0
5 0
6 0
7 0
8 0
9 0
U31-RIB:MET-CA
3 0
4 0
5 0
6 0
7 0
8 0
9 0
U31-MY:MET-S2
3 0
4 0
5 0
6 0
7 0
8 0
9 0
U-P:THR-CA
3 0
4 0
5 2
6 4
7 6
8 9
9 14
IU-P:ARG-S2
3 0
4 0
5 0
6 0
7 0
8 0
9 0
QUO-M5:GLU-S1
3 0
4 0
5 0
6 0

7 0
8 0
9 0
G-R6:SER-CA
3 0
4 0
5 5
6 10
7 15
8 24
9 37
G-P:PRO-CA
3 0
4 0
5 4
6 8
7 12
8 20
9 31
IU-MY:SER-CA
3 0
4 0
5 0
6 0
7 0
8 0
9 0
QUO-M5:ARG-CA
3 0
4 0
5 0
6 0
7 0
8 0
9 0
A-R6:MET-S2
3 0
4 0
5 2
6 3
7 5
8 8
9 13
C-RIB:HIS-S2
3 0
4 0
5 1
6 2
7 4
8 6
9 10
A-RIB:TYR-S1
3 0
4 0
5 2
6 4
7 7
8 11

9 17
U31-MY:GLU-CA
3 0
4 0
5 0
6 0
7 0
8 0
9 0
C-P:MET-CA
3 0
4 0
5 1
6 2
7 4
8 7
9 10
A-RIB:PHE-S1
3 0
4 0
5 3
6 5
7 8
8 13
9 20
A-R5:PHE-S1
3 0
4 0
5 3
6 5
7 8
8 13
9 20
G-RIB:PRO-S1
3 0
4 0
5 5
6 8
7 13
8 20
9 31
FMU-P:ASP-CA
3 0
4 0
5 0
6 0
7 0
8 0
9 0
C31-MY:SER-S1
3 0
4 0
5 0
6 0
7 0
8 0
9 0
U-P:LEU-CA

3 0
4 0
5 0
6 7
7 10
8 17
9 26
A-RIB:ASP-S1
3 0
4 0
5 5
6 9
7 13
8 21
9 33
C-P:GLU-S2
3 0
4 0
5 0
6 9
7 14
8 22
9 34
U31-MY:THR-S1
3 0
4 0
5 0
6 0
7 0
8 0
9 0
C-Y:CYS-CA
3 0
4 0
5 0
6 1
7 0
8 0
9 4
C-P:ALA-CA
3 0
4 0
5 5
6 9
7 14
8 23
9 35
H2U-RIB:PHE-S1
3 0
4 0
5 0
6 0
7 0
8 0
9 0
U-P:CYS-CA
3 0
4 0

5 0
6 0
7 0
8 2
9 3
M2G-P:GLU-S1
3 0
4 0
5 0
6 0
7 0
8 0
9 0
U34-MY:ASP-S2
3 0
4 0
5 0
6 0
7 0
8 0
9 0
DA-M5:HIS-CA
3 0
4 0
5 0
6 0
7 0
8 0
9 0
DA-M5:LEU-S2
3 0
4 0
5 0
6 0
7 0
8 0
9 0
U31-P:GLN-S1
3 0
4 0
5 0
6 0
7 0
8 0
9 0
A-P:LYS-CA
3 0
4 0
5 6
6 10
7 15
8 24
9 37
G-R6:MET-S2
3 0
4 0
5 2
6 4

7 7
8 11
9 16
DA-RIB:MET-CA
3 0
4 0
5 0
6 0
7 0
8 0
9 0
A-R5:ASN-S2
3 0
4 0
5 3
6 5
7 8
8 13
9 21
IU-P:SER-S1
3 0
4 0
5 0
6 0
7 0
8 0
9 0
QUO-M6:GLN-S2
3 0
4 0
5 0
6 0
7 0
8 0
9 0
5BU-P:SER-CA
3 0
4 0
5 0
6 0
7 0
8 0
9 0
G-RIB:GLU-S2
3 0
4 0
5 8
6 14
7 22
8 35
9 54
U-Y:MET-S2
3 0
4 0
5 1
6 2
7 3
8 4

9 7
H2U-MY:LYS-S1
3 0
4 0
5 0
6 0
7 0
8 0
9 0
FMU-RIB:MET-S1
3 0
4 0
5 0
6 0
7 0
8 0
9 0
G-R6:ARG-S1
3 0
4 0
5 7
6 12
7 19
8 30
9 46
FMU-RIB:ARG-S1
3 0
4 0
5 0
6 0
7 0
8 0
9 0
C-RIB:LEU-S2
3 0
4 0
5 6
6 10
7 15
8 24
9 37
U31-RIB:ASP-S1
3 0
4 0
5 0
6 0
7 0
8 0
9 0
A-RIB:HIS-CA
3 0
4 0
5 2
6 3
7 5
8 8
9 13
FHU-MY:THR-CA

3 0
4 0
5 0
6 0
7 0
8 0
9 0
G-P:ASP-CA
3 0
4 0
5 6
6 11
7 17
8 27
9 41
IU-RIB:LYS-S1
3 0
4 0
5 0
6 0
7 0
8 0
9 0
FHU-P:ALA-S1
3 0
4 0
5 0
6 0
7 0
8 0
9 0
G-P:GLN-S1
3 0
4 0
5 3
6 6
7 10
8 15
9 24
FMU-MY:PRO-CA
3 0
4 0
5 0
6 0
7 0
8 0
9 0
U34-RIB:TYR-S2
3 0
4 0
5 0
6 0
7 0
8 0
9 0
C-RIB:LYS-S1
3 0
4 0

5 4
6 8
7 12
8 19
9 30
QUO-P:ASN-S2
3 0
4 0
5 0
6 0
7 0
8 0
9 0
C31-RIB:GLU-S1
3 0
4 0
5 0
6 0
7 0
8 0
9 0
A-RIB:LEU-S2
3 0
4 0
5 7
6 12
7 19
8 31
9 47
G-P:PHE-S1
3 0
4 0
5 4
6 6
7 10
8 16
9 25
G-RIB:THR-CA
3 0
4 0
5 5
6 9
7 14
8 22
9 34
A-RIB:ASP-CA
3 0
4 0
5 5
6 9
7 13
8 21
9 33
OMC-RIB:LYS-S1
3 0
4 0
5 0
6 0

7 0
8 0
9 0
C-Y:GLU-CA
3 0
4 0
5 5
6 9
7 14
8 23
9 35
A-R6:MET-CA
3 0
4 0
5 2
6 3
7 5
8 8
9 13
U34-MY:ASN-S2
3 0
4 0
5 0
6 0
7 0
8 0
9 0
G-RIB:TYR-CA
3 0
4 0
5 0
6 6
7 9
8 14
9 22
A-RIB:ASN-CA
3 0
4 0
5 3
6 5
7 8
8 14
9 21
FMU-P:ALA-CA
3 0
4 0
5 0
6 0
7 0
8 0
9 0
G-R5:ALA-CA
3 0
4 1
5 8
6 15
7 23
8 36

9   56
FHU-MY:LEU-S1
3   0
4   0
5   0
6   0
7   0
8   0
9   0
G-RIB:HIS-S2
3   0
4   0
5   2
6   4
7   6
8   10
9   16
U-P:HIS-S2
3   0
4   0
5   1
6   1
7   2
8   4
9   7
C-P:SER-CA
3   0
4   0
5   3
6   6
7   9
8   15
9   23
GTP-RIB:THR-S1
3   0
4   0
5   0
6   0
7   0
8   0
9   0
G-R6:TRP-S1
3   0
4   0
5   1
6   0
7   3
8   5
9   9
FHU-MY:ARG-CA
3   0
4   0
5   0
6   0
7   0
8   0
9   0
C-RIB:PHE-S1

3 0
4 0
5 2
6 4
7 6
8 10
9 16
A-P:HIS-S1
3 0
4 0
5 2
6 3
7 5
8 8
9 13
U-Y:ALA-S1
3 0
4 0
5 3
6 6
7 10
8 16
9 24
A-R5:GLY-CA
3 0
4 0
5 6
6 10
7 15
8 25
9 38
C-P:ARG-S1
3 0
4 0
5 4
6 7
7 12
8 19
9 29
DA-RIB:HIS-CA
3 0
4 0
5 0
6 0
7 0
8 0
9 0
FMU-MY:ARG-CA
3 0
4 0
5 0
6 0
7 0
8 0
9 0
I-RIB:TRP-S2
3 0
4 0

5 0
6 0
7 0
8 0
9 0
A-R6:HIS-S2
3 0
4 0
5 2
6 3
7 5
8 8
9 13
A-R6:ASP-S2
3 0
4 0
5 5
6 8
7 13
8 21
9 32
GTP-M5:ALA-S1
3 0
4 0
5 0
6 0
7 0
8 0
9 0
IU-RIB:HIS-CA
3 0
4 0
5 0
6 0
7 0
8 0
9 0
G-R5:MET-S2
3 0
4 0
5 2
6 4
7 7
8 11
9 16
FHU-MY:ALA-S1
3 0
4 0
5 0
6 0
7 0
8 0
9 0
I-RIB:GLY-CA
3 0
4 0
5 0
6 0

7 0
8 0
9 0
C-RIB:ASP-CA
3 0
4 0
5 4
6 7
7 11
8 17
9 26
G-R6:MET-CA
3 0
4 0
5 2
6 4
7 7
8 11
9 16
G-R6:TYR-S2
3 0
4 0
5 3
6 6
7 9
8 14
9 22
U31-MY:ASP-CA
3 0
4 0
5 0
6 0
7 0
8 0
9 0
U-P:ARG-S2
3 0
4 0
5 3
6 5
7 8
8 13
9 20
A-R6:GLN-S2
3 0
4 0
5 3
6 5
7 8
8 12
9 19
U-P:ASP-S1
3 0
4 0
5 2
6 4
7 7
8 11

9 18
A-P:LEU-CA
3 0
4 0
5 7
6 12
7 19
8 31
9 47
C-RIB:MET-CA
3 0
4 0
5 1
6 2
7 4
8 7
9 10
A-P:GLU-S1
3 0
4 0
5 6
6 11
7 18
8 28
9 43
FHU-RIB:ARG-S2
3 0
4 0
5 0
6 0
7 0
8 0
9 0
QUO-M6:ARG-S2
3 0
4 0
5 0
6 0
7 0
8 0
9 0
C-P:MET-S1
3 0
4 0
5 1
6 2
7 4
8 7
9 10
U31-P:MET-S1
3 0
4 0
5 0
6 0
7 0
8 0
9 0
OMC-RIB:LYS-S2

3 0
4 0
5 0
6 0
7 0
8 0
9 0
C-P:ILE-S1
3 0
4 0
5 3
6 6
7 9
8 15
9 23
C-Y:SER-CA
3 0
4 0
5 3
6 6
7 9
8 15
9 23
FHU-MY:SER-CA
3 0
4 0
5 0
6 0
7 0
8 0
9 0
U31-MY:MET-CA
3 0
4 0
5 0
6 0
7 0
8 0
9 0
G-R6:ILE-CA
3 0
4 0
5 0
6 10
7 15
8 24
9 37
C31-MY:THR-S1
3 0
4 0
5 0
6 0
7 0
8 0
9 0
C31-MY:GLN-S2
3 0
4 0

5 0
6 0
7 0
8 0
9 0
G-R5:ILE-CA
3 0
4 0
5 0
6 10
7 0
8 24
9 37
IU-MY:ALA-CA
3 0
4 0
5 0
6 0
7 0
8 0
9 0
IU-RIB:SER-S1
3 0
4 0
5 0
6 0
7 0
8 0
9 0
G-R5:ALA-S1
3 0
4 1
5 8
6 15
7 23
8 36
9 56
H2U-P:ASN-S2
3 0
4 0
5 0
6 0
7 0
8 0
9 0
IU-MY:ARG-S1
3 0
4 0
5 0
6 0
7 0
8 0
9 0
G-RIB:HIS-CA
3 0
4 0
5 2
6 4

7 6
8 10
9 16
C31-P:TYR-S2
3 0
4 0
5 0
6 0
7 0
8 0
9 0
IU-MY:ILE-CA
3 0
4 0
5 0
6 0
7 0
8 0
9 0
G-R6:LEU-S2
3 0
4 1
5 9
6 16
7 24
8 39
9 59
C-RIB:VAL-S1
3 0
4 0
5 5
6 8
7 13
8 20
9 31
U31-RIB:ASN-CA
3 0
4 0
5 0
6 0
7 0
8 0
9 0
FMU-P:ARG-S1
3 0
4 0
5 0
6 0
7 0
8 0
9 0
G-R5:GLU-CA
3 0
4 0
5 8
6 0
7 22
8 36

9   55
G-RIB:TRP-CA
3   0
4   0
5   1
6   2
7   3
8   5
9   9
QUO-M5:ASP-S1
3   0
4   0
5   0
6   0
7   0
8   0
9   0
G-P:THR-S1
3   0
4   0
5   5
6   9
7   14
8   22
9   33
G-R6:VAL-CA
3   0
4   0
5   0
6   13
7   20
8   32
9   50
G-R6:ALA-CA
3   0
4   0
5   8
6   15
7   23
8   36
9   56
DA-RIB:ARG-S2
3   0
4   0
5   0
6   0
7   0
8   0
9   0
U31-MY:ILE-S1
3   0
4   0
5   0
6   0
7   0
8   0
9   0
C-RIB:ASP-S1

3 0
4 0
5 4
6 7
7 11
8 17
9 26
C-Y:HIS-S2
3 0
4 0
5 0
6 2
7 4
8 6
9 10
C31-MY:ALA-CA
3 0
4 0
5 0
6 0
7 0
8 0
9 0
5BU-P:ARG-S1
3 0
4 0
5 0
6 0
7 0
8 0
9 0
IU-P:LEU-S1
3 0
4 0
5 0
6 0
7 0
8 0
9 0
H2U-MY:GLN-CA
3 0
4 0
5 0
6 0
7 0
8 0
9 0
C-Y:LEU-S2
3 0
4 0
5 6
6 10
7 15
8 24
9 37
H2U-P:LYS-S2
3 0
4 0

5	0
6	0
7	0
8	0
9	0

U34-P:GLU-S2

3	0
4	0
5	0
6	0
7	0
8	0
9	0

C-Y:GLU-S1

3	0
4	0
5	5
6	9
7	14
8	22
9	35

IU-MY:ALA-S1

3	0
4	0
5	0
6	0
7	0
8	0
9	0

H2U-P:TRP-S2

3	0
4	0
5	0
6	0
7	0
8	0
9	0

IU-MY:ARG-CA

3	0
4	0
5	0
6	0
7	0
8	0
9	0

I-RIB:ALA-CA

3	0
4	0
5	0
6	0
7	0
8	0
9	0

U34-P:ARG-S2

3	0
4	0
5	0
6	0

7 0
8 0
9 0
C31-P:GLN-S2
3 0
4 0
5 0
6 0
7 0
8 0
9 0
FHU-P:ASP-CA
3 0
4 0
5 0
6 0
7 0
8 0
9 0
IU-RIB:ARG-CA
3 0
4 0
5 0
6 0
7 0
8 0
9 0
C-Y:GLN-S2
3 0
4 0
5 2
6 4
7 6
8 10
9 15
FHU-RIB:LEU-CA
3 0
4 0
5 0
6 0
7 0
8 0
9 0
C-RIB:PRO-CA
3 0
4 0
5 3
6 5
7 8
8 13
9 20
A-P:PHE-S2
3 0
4 0
5 0
6 5
7 8
8 13

9 20
U-RIB:GLN-S1
3 0
4 0
5 1
6 2
7 4
8 7
9 10
C31-RIB:GLU-CA
3 0
4 0
5 0
6 0
7 0
8 0
9 0
G-P:PHE-CA
3 0
4 0
5 4
6 6
7 10
8 16
9 25
H2U-MY:PHE-S2
3 0
4 0
5 0
6 0
7 0
8 0
9 0
DA-RIB:SER-S1
3 0
4 0
5 0
6 0
7 0
8 0
9 0
A-R6:GLU-S1
3 0
4 0
5 6
6 11
7 18
8 28
9 43
C31-RIB:LEU-CA
3 0
4 0
5 0
6 0
7 0
8 0
9 0
G-RIB:ASP-CA

3 0
4 0
5 6
6 11
7 17
8 27
9 41
H2U-P:PHE-S2
3 0
4 0
5 0
6 0
7 0
8 0
9 0
C-RIB:SER-CA
3 0
4 0
5 3
6 6
7 9
8 15
9 23
U34-MY:ASN-CA
3 0
4 0
5 0
6 0
7 0
8 0
9 0
U-P:HIS-CA
3 0
4 0
5 1
6 0
7 3
8 4
9 7
U34-MY:SER-CA
3 0
4 0
5 0
6 0
7 0
8 0
9 0
G-R5:GLU-S1
3 0
4 0
5 8
6 14
7 22
8 36
9 54
DA-RIB:VAL-CA
3 0
4 0

5 0
6 0
7 0
8 0
9 0
H2U-RIB:PHE-CA
3 0
4 0
5 0
6 0
7 0
8 0
9 0
FMU-RIB:HIS-S2
3 0
4 0
5 0
6 0
7 0
8 0
9 0
QUO-M6:PHE-CA
3 0
4 0
5 0
6 0
7 0
8 0
9 0
H2U-MY:ASN-S2
3 0
4 0
5 0
6 0
7 0
8 0
9 0
DA-M6:THR-CA
3 0
4 0
5 0
6 0
7 0
8 0
9 0
GTP-RIB:THR-CA
3 0
4 0
5 0
6 0
7 0
8 0
9 0
A-P:ALA-CA
3 0
4 0
5 7
6 12

7   18
8   29
9   44
FMU-P:ASP-S2
3   0
4   0
5   0
6   0
7   0
8   0
9   0
DA-M5:THR-CA
3   0
4   0
5   0
6   0
7   0
8   0
9   0
G-R6:PHE-S1
3   0
4   0
5   4
6   6
7   10
8   16
9   25
U-Y:ASP-S2
3   0
4   0
5   2
6   4
7   7
8   11
9   17
G-RIB:ALA-CA
3   0
4   0
5   8
6   15
7   23
8   36
9   56
G-R6:CYS-CA
3   0
4   0
5   0
6   0
7   3
8   4
9   7
FMU-RIB:GLN-S2
3   0
4   0
5   0
6   0
7   0
8   0

9 0
G-RIB:SER-S1
3 0
4 0
5 5
6 10
7 15
8 24
9 37
IU-MY:LYS-CA
3 0
4 0
5 0
6 0
7 0
8 0
9 0
H2U-P:LEU-S2
3 0
4 0
5 0
6 0
7 0
8 0
9 0
QUO-M6:GLU-S2
3 0
4 0
5 0
6 0
7 0
8 0
9 0
U-P:MET-CA
3 0
4 0
5 1
6 2
7 3
8 4
9 7
A-P:ASN-S1
3 0
4 0
5 3
6 5
7 8
8 14
9 21
A-P:TRP-CA
3 0
4 0
5 1
6 1
7 3
8 4
9 7
C-P:GLU-S1

3 0
4 0
5 5
6 9
7 14
8 22
9 34
U-P:ALA-S1
3 0
4 0
5 3
6 6
7 10
8 16
9 24
U-RIB:CYS-S1
3 0
4 0
5 0
6 0
7 1
8 2
9 3
H2U-RIB:GLU-S1
3 0
4 0
5 0
6 0
7 0
8 0
9 0
C-RIB:PRO-S1
3 0
4 0
5 3
6 5
7 8
8 13
9 20
G-P:VAL-CA
3 0
4 0
5 0
6 13
7 20
8 32
9 49
U31-P:GLN-S2
3 0
4 0
5 0
6 0
7 0
8 0
9 0
U-Y:GLY-CA
3 0
4 0

5 3
6 5
7 8
8 13
9 21
A-RIB:ASN-S2
3 0
4 0
5 3
6 5
7 8
8 13
9 21
FHU-P:LEU-CA
3 0
4 0
5 0
6 0
7 0
8 0
9 0
A-R5:ARG-CA
3 0
4 0
5 0
6 9
7 15
8 24
9 36
IU-RIB:GLN-S2
3 0
4 0
5 0
6 0
7 0
8 0
9 0
IU-RIB:ARG-S2
3 0
4 0
5 0
6 0
7 0
8 0
9 0
QUO-M5:LYS-S1
3 0
4 0
5 0
6 0
7 0
8 0
9 0
FHU-MY:ASP-S2
3 0
4 0
5 0
6 0

7 0
8 0
9 0
QUO-M6:GLN-S1
3 0
4 0
5 0
6 0
7 0
8 0
9 0
QUO-M6:LYS-S2
3 0
4 0
5 0
6 0
7 0
8 0
9 0
G-RIB:GLN-S2
3 0
4 0
5 3
6 6
7 10
8 15
9 24
FMU-RIB:GLU-S2
3 0
4 0
5 0
6 0
7 0
8 0
9 0
U31-MY:ILE-CA
3 0
4 0
5 0
6 0
7 0
8 0
9 0
DA-M6:TYR-CA
3 0
4 0
5 0
6 0
7 0
8 0
9 0
IU-MY:LYS-S1
3 0
4 0
5 0
6 0
7 0
8 0

9 0
DA-M6:LYS-S1
3 0
4 0
5 0
6 0
7 0
8 0
9 0
C-RIB:LYS-S2
3 0
4 0
5 4
6 8
7 12
8 19
9 29
U-RIB:GLN-CA
3 0
4 0
5 0
6 2
7 4
8 7
9 10
FHU-P:PRO-S1
3 0
4 0
5 0
6 0
7 0
8 0
9 0
G-P:ALA-CA
3 0
4 0
5 8
6 15
7 23
8 36
9 55
U34-P:PHE-S2
3 0
4 0
5 0
6 0
7 0
8 0
9 0
U34-MY:TYR-S2
3 0
4 0
5 0
6 0
7 0
8 0
9 0
C-P:HIS-S1

3 0
4 0
5 1
6 2
7 4
8 6
9 10
H2U-MY:ILE-CA
3 0
4 0
5 0
6 0
7 0
8 0
9 0
G-P:MET-S2
3 0
4 0
5 2
6 4
7 6
8 10
9 16
G-P:GLN-CA
3 0
4 0
5 3
6 6
7 10
8 15
9 24
U31-MY:GLN-CA
3 0
4 0
5 0
6 0
7 0
8 0
9 0
A-RIB:PHE-CA
3 0
4 0
5 3
6 5
7 8
8 13
9 20
U-P:ASN-S2
3 0
4 0
5 1
6 3
7 4
8 7
9 11
U34-RIB:ASN-S2
3 0
4 0

5 0
6 0
7 0
8 0
9 0
U-P:PRO-S1
3 0
4 0
5 2
6 3
7 5
8 9
9 13
A-R5:PRO-S1
3 0
4 0
5 3
6 6
7 10
8 16
9 25
A-R5:HIS-S1
3 0
4 0
5 2
6 3
7 5
8 8
9 13
A-R6:CYS-CA
3 0
4 0
5 0
6 0
7 2
8 3
9 5
U-Y:PRO-CA
3 0
4 0
5 2
6 3
7 5
8 9
9 13
G-RIB:TRP-S2
3 0
4 0
5 1
6 2
7 3
8 5
9 9
G-R5:ARG-S1
3 0
4 0
5 7
6 12

7 19
8 30
9 46
U-RIB:VAL-CA
3 0
4 0
5 0
6 5
7 9
8 14
9 21
C-RIB:TRP-S1
3 0
4 0
5 0
6 1
7 2
8 3
9 5
QUO-RIB:LEU-CA
3 0
4 0
5 0
6 0
7 0
8 0
9 0
A-R6:ASP-S1
3 0
4 0
5 5
6 9
7 13
8 21
9 33
U-P:ILE-CA
3 0
4 0
5 0
6 4
7 6
8 10
9 16
DA-M6:THR-S1
3 0
4 0
5 0
6 0
7 0
8 0
9 0
G-P:TYR-S1
3 0
4 0
5 3
6 5
7 9
8 14

9 21
U-P:TYR-S2
3 0
4 0
5 0
6 2
7 4
8 6
9 9
A-RIB:GLU-S2
3 0
4 0
5 6
6 11
7 18
8 28
9 43
FMU-MY:ARG-S2
3 0
4 0
5 0
6 0
7 0
8 0
9 0
FMU-P:ILE-CA
3 0
4 0
5 0
6 0
7 0
8 0
9 0
G-R5:THR-S1
3 0
4 0
5 5
6 9
7 14
8 22
9 34
G-R6:HIS-S2
3 0
4 0
5 0
6 4
7 6
8 10
9 16
H2U-MY:LEU-CA
3 0
4 0
5 0
6 0
7 0
8 0
9 0
G-R6:TRP-S2

3 0
4 0
5 1
6 2
7 3
8 5
9 9
DA-M5:LYS-S2
3 0
4 0
5 0
6 0
7 0
8 0
9 0
G-RIB:ALA-S1
3 0
4 1
5 8
6 15
7 23
8 36
9 56
FHU-RIB:ASP-CA
3 0
4 0
5 0
6 0
7 0
8 0
9 0
C-Y:ALA-S1
3 0
4 0
5 5
6 9
7 14
8 23
9 35
C-Y:ARG-S2
3 0
4 0
5 4
6 7
7 12
8 19
9 29
4SU-P:GLU-S2
3 0
4 0
5 0
6 0
7 0
8 0
9 0
FHU-P:SER-S1
3 0
4 0

5 0
6 0
7 0
8 0
9 0
G-RIB:MET-S2
3 0
4 0
5 2
6 4
7 7
8 11
9 16
U-Y:LYS-CA
3 0
4 0
5 3
6 5
7 8
8 13
9 20
C-P:PRO-S1
3 0
4 0
5 3
6 5
7 8
8 13
9 19
IU-MY:PRO-CA
3 0
4 0
5 0
6 0
7 0
8 0
9 0
U-P:ALA-CA
3 0
4 0
5 3
6 6
7 10
8 16
9 24
H2U-MY:ASN-CA
3 0
4 0
5 0
6 0
7 0
8 0
9 0
GTP-M6:SER-S1
3 0
4 0
5 0
6 0

7   0
8   0
9   0
G-P:HIS-CA
3   0
4   0
5   0
6   4
7   6
8   10
9   16
G-RIB:LEU-S2
3   0
4   0
5   9
6   16
7   24
8   39
9   59
IU-MY:LEU-S1
3   0
4   0
5   0
6   0
7   0
8   0
9   0
A-R6:LYS-S2
3   0
4   0
5   5
6   10
7   15
8   24
9   37
C31-RIB:LEU-S2
3   0
4   0
5   0
6   0
7   0
8   0
9   0
QUO-M5:LEU-S1
3   0
4   0
5   0
6   0
7   0
8   0
9   0
A-R6:LEU-S1
3   0
4   0
5   7
6   12
7   19
8   31

9   47
G-R6:LEU-S1
3   0
4   1
5   9
6   16
7   24
8   39
9   59
FMU-MY:CYS-CA
3   0
4   0
5   0
6   0
7   0
8   0
9   0
G-R6:ARG-S2
3   0
4   0
5   7
6   12
7   19
8   30
9   45
C-RIB:GLN-S2
3   0
4   0
5   2
6   4
7   6
8   10
9   15
A-RIB:PRO-S1
3   0
4   0
5   3
6   6
7   10
8   16
9   25
IU-MY:VAL-CA
3   0
4   0
5   0
6   0
7   0
8   0
9   0
IU-MY:LEU-CA
3   0
4   0
5   0
6   0
7   0
8   0
9   0
C-Y:ALA-CA

3 0
4 0
5 5
6 9
7 14
8 23
9 35
C-RIB:TRP-CA
3 0
4 0
5 0
6 1
7 2
8 3
9 5
5BU-P:PRO-CA
3 0
4 0
5 0
6 0
7 0
8 0
9 0
FHU-P:HIS-S2
3 0
4 0
5 0
6 0
7 0
8 0
9 0
U-RIB:ASN-S1
3 0
4 0
5 1
6 3
7 4
8 7
9 11
C-P:LEU-S2
3 0
4 0
5 6
6 10
7 15
8 24
9 37
U34-MY:PHE-S1
3 0
4 0
5 0
6 0
7 0
8 0
9 0
U-RIB:ASP-CA
3 0
4 0

5 2
6 4
7 7
8 12
9 18
FMU-RIB:ILE-CA
3 0
4 0
5 0
6 0
7 0
8 0
9 0
H2U-P:TRP-S1
3 0
4 0
5 0
6 0
7 0
8 0
9 0
FMU-RIB:HIS-S1
3 0
4 0
5 0
6 0
7 0
8 0
9 0
FHU-P:LYS-S2
3 0
4 0
5 0
6 0
7 0
8 0
9 0
G-R5:TYR-S2
3 0
4 0
5 3
6 6
7 9
8 14
9 22
G-RIB:PHE-S1
3 0
4 0
5 4
6 6
7 10
8 16
9 25
A-R5:TRP-S2
3 0
4 0
5 0
6 1

7 3
8 4
9 7
FMU-MY:PHE-S1
3 0
4 0
5 0
6 0
7 0
8 0
9 0
FHU-P:ASP-S1
3 0
4 0
5 0
6 0
7 0
8 0
9 0
QUO-P:PHE-S2
3 0
4 0
5 0
6 0
7 0
8 0
9 0
U34-P:ASN-CA
3 0
4 0
5 0
6 0
7 0
8 0
9 0
H2U-RIB:GLU-S2
3 0
4 0
5 0
6 0
7 0
8 0
9 0
FHU-MY:GLY-CA
3 0
4 0
5 0
6 0
7 0
8 0
9 0
U31-P:SER-S1
3 0
4 0
5 0
6 0
7 0
8 0

9 0
FHU-RIB:ILE-S1
3 0
4 0
5 0
6 0
7 0
8 0
9 0
IU-MY:MET-CA
3 0
4 0
5 0
6 0
7 0
8 0
9 0
U31-RIB:ASN-S2
3 0
4 0
5 0
6 0
7 0
8 0
9 0
DA-RIB:GLN-S1
3 0
4 0
5 0
6 0
7 0
8 0
9 0
U31-RIB:GLU-S2
3 0
4 0
5 0
6 0
7 0
8 0
9 0
U-RIB:ASP-S1
3 0
4 0
5 2
6 4
7 7
8 11
9 18
FHU-P:VAL-S1
3 0
4 0
5 0
6 0
7 0
8 0
9 0
A-RIB:LYS-S1

3 0
4 0
5 6
6 10
7 15
8 24
9 37
FHU-MY:ASP-CA
3 0
4 0
5 0
6 0
7 0
8 0
9 0
C-RIB:SER-S1
3 0
4 0
5 3
6 6
7 9
8 15
9 23
U-RIB:GLU-S2
3 0
4 0
5 3
6 6
7 9
8 15
9 23
C-P:LEU-S1
3 0
4 0
5 6
6 10
7 15
8 24
9 37
G-R5:GLY-CA
3 0
4 0
5 7
6 13
7 20
8 31
9 48
A-RIB:TRP-CA
3 0
4 0
5 0
6 1
7 3
8 4
9 7
FMU-MY:SER-CA
3 0
4 0

5 0
6 0
7 0
8 0
9 0
GTP-M5:SER-CA
3 0
4 0
5 0
6 0
7 0
8 0
9 0
U-RIB:THR-CA
3 0
4 0
5 0
6 4
7 6
8 9
9 14
G-P:ILE-S1
3 0
4 0
5 5
6 10
7 15
8 24
9 37
G-R5:ASN-CA
3 0
4 0
5 4
6 7
7 11
8 17
9 26
DA-M5:SER-S1
3 0
4 0
5 0
6 0
7 0
8 0
9 0
IU-RIB:ALA-S1
3 0
4 0
5 0
6 0
7 0
8 0
9 0
IU-MY:PRO-S1
3 0
4 0
5 0
6 0

7 0
8 0
9 0
IU-MY:LYS-S2
3 0
4 0
5 0
6 0
7 0
8 0
9 0
I-RIB:ALA-S1
3 0
4 0
5 0
6 0
7 0
8 0
9 0
G-RIB:ASN-S2
3 0
4 0
5 4
6 7
7 11
8 17
9 26
FMU-MY:HIS-S1
3 0
4 0
5 0
6 0
7 0
8 0
9 0
G-R6:HIS-CA
3 0
4 0
5 2
6 4
7 6
8 10
9 16
FHU-RIB:SER-S1
3 0
4 0
5 0
6 0
7 0
8 0
9 0
U34-P:ASN-S1
3 0
4 0
5 0
6 0
7 0
8 0

9 0
QUO-M5:LEU-CA
3 0
4 0
5 0
6 0
7 0
8 0
9 0
U-P:TRP-S1
3 0
4 0
5 0
6 1
7 1
8 2
9 3
A-P:TRP-S2
3 0
4 0
5 0
6 1
7 3
8 4
9 7
G-R6:ASP-S1
3 0
4 0
5 6
6 11
7 17
8 27
9 41
A-RIB:TRP-S1
3 0
4 0
5 1
6 1
7 3
8 4
9 7
U34-RIB:ASN-CA
3 0
4 0
5 0
6 0
7 0
8 0
9 0
5BU-P:PRO-S1
3 0
4 0
5 0
6 0
7 0
8 0
9 0
A-R5:SER-CA

3 0
4 0
5 0
6 8
7 12
8 19
9 29
C-Y:VAL-S1
3 0
4 0
5 5
6 8
7 13
8 20
9 31
A-R5:HIS-S2
3 0
4 0
5 2
6 3
7 5
8 8
9 13
4SU-P:THR-S1
3 0
4 0
5 0
6 0
7 0
8 0
9 0
A-RIB:ALA-S1
3 0
4 0
5 7
6 12
7 18
8 29
9 44
H2U-RIB:ASN-S1
3 0
4 0
5 0
6 0
7 0
8 0
9 0
C-RIB:TRP-S2
3 0
4 0
5 0
6 1
7 2
8 3
9 5
G-P:ASN-CA
3 0
4 0

5 4
6 7
7 11
8 17
9 26
U31-MY:PHE-CA
3 0
4 0
5 0
6 0
7 0
8 0
9 0
A-R5:SER-S1
3 0
4 0
5 4
6 8
7 12
8 19
9 29
U-Y:PHE-CA
3 0
4 0
5 0
6 3
7 4
8 7
9 11
FHU-RIB:SER-CA
3 0
4 0
5 0
6 0
7 0
8 0
9 0
G-RIB:PHE-CA
3 0
4 0
5 4
6 6
7 10
8 16
9 25
DA-M5:LYS-S1
3 0
4 0
5 0
6 0
7 0
8 0
9 0
C-RIB:THR-CA
3 0
4 0
5 3
6 5

7 9
8 14
9 21
GTP-M5:THR-CA
3 0
4 0
5 0
6 0
7 0
8 0
9 0
U-RIB:ARG-S1
3 0
4 0
5 3
6 5
7 8
8 13
9 20
U-P:MET-S1
3 0
4 0
5 1
6 2
7 3
8 4
9 7
U31-P:TYR-S1
3 0
4 0
5 0
6 0
7 0
8 0
9 0
C-P:PHE-S2
3 0
4 0
5 0
6 4
7 6
8 10
9 16
FMU-RIB:PHE-S1
3 0
4 0
5 0
6 0
7 0
8 0
9 0
FHU-MY:LYS-S1
3 0
4 0
5 0
6 0
7 0
8 0

9   0
G-R5:PHE-S2
3   0
4   0
5   0
6   6
7   10
8   16
9   25
C31-P:ASP-S2
3   0
4   0
5   0
6   0
7   0
8   0
9   0
C-Y:PHE-S1
3   0
4   0
5   2
6   4
7   6
8   10
9   16
C-RIB:CYS-CA
3   0
4   0
5   0
6   1
7   0
8   3
9   4
H2U-MY:ARG-S2
3   0
4   0
5   0
6   0
7   0
8   0
9   0
G-R5:LEU-CA
3   0
4   0
5   9
6   16
7   24
8   39
9   59
G-R5:ARG-S2
3   0
4   0
5   7
6   12
7   19
8   30
9   45
A-RIB:ILE-CA

3 0
4 0
5 4
6 8
7 12
8 19
9 30
U34-P:TYR-S2
3 0
4 0
5 0
6 0
7 0
8 0
9 0
A-P:GLY-CA
3 0
4 0
5 6
6 10
7 15
8 25
9 38
DA-RIB:ASP-S2
3 0
4 0
5 0
6 0
7 0
8 0
9 0
C-P:CYS-S1
3 0
4 0
5 0
6 1
7 1
8 3
9 4
FHU-RIB:PRO-CA
3 0
4 0
5 0
6 0
7 0
8 0
9 0
G-P:LEU-S2
3 0
4 1
5 9
6 16
7 24
8 38
9 58
FMU-MY:ASN-S1
3 0
4 0

5 0
6 0
7 0
8 0
9 0
A-R6:SER-CA
3 0
4 0
5 4
6 8
7 12
8 19
9 29
G-R6:GLU-CA
3 0
4 0
5 0
6 14
7 22
8 36
9 55
IU-RIB:LEU-S2
3 0
4 0
5 0
6 0
7 0
8 0
9 0
U-RIB:ASN-CA
3 0
4 0
5 1
6 3
7 4
8 7
9 11
DA-M5:SER-CA
3 0
4 0
5 0
6 0
7 0
8 0
9 0
U31-MY:GLN-S2
3 0
4 0
5 0
6 0
7 0
8 0
9 0
A-RIB:ALA-CA
3 0
4 0
5 7
6 12

7   18
8   29
9   44
C31-P:GLN-S1
3   0
4   0
5   0
6   0
7   0
8   0
9   0
U-P:LEU-S2
3   0
4   0
5   0
6   7
7   10
8   17
9   26
FHU-RIB:LYS-CA
3   0
4   0
5   0
6   0
7   0
8   0
9   0
A-RIB:LEU-CA
3   0
4   0
5   0
6   12
7   19
8   31
9   47
U34-P:PRO-S1
3   0
4   0
5   0
6   0
7   0
8   0
9   0
A-R5:ALA-S1
3   0
4   0
5   0
6   12
7   18
8   29
9   44
FMU-MY:ALA-S1
3   0
4   0
5   0
6   0
7   0
8   0

9 0
C-Y:GLN-S1
3 0
4 0
5 0
6 4
7 6
8 10
9 15
A-R6:LYS-S1
3 0
4 0
5 6
6 10
7 15
8 24
9 37
U-RIB:ARG-CA
3 0
4 0
5 3
6 5
7 8
8 13
9 20
H2U-MY:GLN-S1
3 0
4 0
5 0
6 0
7 0
8 0
9 0
C-P:CYS-CA
3 0
4 0
5 0
6 0
7 1
8 3
9 4
FHU-MY:ILE-S1
3 0
4 0
5 0
6 0
7 0
8 0
9 0
U31-RIB:PHE-S1
3 0
4 0
5 0
6 0
7 0
8 0
9 0
GTP-M5:ASP-S2

3 0
4 0
5 0
6 0
7 0
8 0
9 0
C-RIB:GLN-S1
3 0
4 0
5 2
6 4
7 6
8 10
9 15
C-P:GLN-CA
3 0
4 0
5 2
6 4
7 6
8 10
9 15
U-P:GLU-S1
3 0
4 0
5 3
6 6
7 10
8 15
9 24
U-Y:GLN-S1
3 0
4 0
5 0
6 0
7 4
8 7
9 10
DA-M6:HIS-CA
3 0
4 0
5 0
6 0
7 0
8 0
9 0
U31-MY:ALA-CA
3 0
4 0
5 0
6 0
7 0
8 0
9 0
A-R5:ILE-S1
3 0
4 0

5   4
6   8
7   12
8   19
9   30
C-Y:LEU-CA
3   0
4   0
5   6
6   10
7   15
8   24
9   38
FHU-P:GLY-CA
3   0
4   0
5   0
6   0
7   0
8   0
9   0
C31-P:GLU-CA
3   0
4   0
5   0
6   0
7   0
8   0
9   0
C-RIB:HIS-S1
3   0
4   0
5   1
6   2
7   4
8   6
9   10
U-RIB:ASP-S2
3   0
4   0
5   2
6   4
7   7
8   11
9   17
C-Y:PHE-CA
3   0
4   0
5   0
6   4
7   6
8   10
9   16
G-R6:VAL-S1
3   0
4   0
5   7
6   13

7 20
8 32
9 49
C-RIB:CYS-S1
3 0
4 0
5 0
6 0
7 1
8 3
9 4
QUO-M5:GLU-S2
3 0
4 0
5 0
6 0
7 0
8 0
9 0
A-R5:VAL-CA
3 0
4 0
5 6
6 10
7 16
8 26
9 39
5BU-MY:PRO-CA
3 0
4 0
5 0
6 0
7 0
8 0
9 0
GTP-M5:THR-S1
3 0
4 0
5 0
6 0
7 0
8 0
9 0
G-RIB:GLU-S1
3 0
4 0
5 8
6 14
7 22
8 36
9 54
U-Y:CYS-CA
3 0
4 0
5 0
6 0
7 0
8 2

9   3
U-Y:MET-S1
3   0
4   0
5   1
6   2
7   3
8   4
9   7
A-R5:GLU-CA
3   0
4   0
5   7
6   11
7   18
8   28
9   43
U-RIB:TRP-S2
3   0
4   0
5   0
6   1
7   1
8   2
9   4
A-R5:TYR-S1
3   0
4   0
5   2
6   4
7   7
8   11
9   17
QUO-M6:ARG-CA
3   0
4   0
5   0
6   0
7   0
8   0
9   0
EXPECTED_ANG
5BU-P:PRO-S1
20   0
40   0
60   0
80   0
100   0
120   0
140   0
160   0
180   0
200   0
220   0
240   0
260   0
280   0
300   0

320 0
340 0
360 0
C-Y:ASP-S1
20 0
40 2
60 4
80 5
100 6
120 5
140 4
160 3
180 0
200 0
220 2
240 4
260 5
280 6
300 5
320 4
340 3
360 1
C-RIB:CYS-S1
20 0
40 0
60 0
80 0
100 1
120 0
140 0
160 0
180 0
200 0
220 0
240 0
260 1
280 1
300 0
320 0
340 0
360 0
A-P:VAL-CA
20 0
40 0
60 6
80 8
100 9
120 8
140 6
160 4
180 1
200 0
220 0
240 6
260 8
280 9
300 8
320 7

340 5
360 2
OMC-RIB:LYS-S2
20 0
40 0
60 0
80 0
100 0
120 0
140 0
160 0
180 0
200 0
220 0
240 0
260 0
280 0
300 0
320 0
340 0
360 0
C-RIB:GLU-S1
20 0
40 2
60 5
80 7
100 7
120 7
140 5
160 4
180 1
200 0
220 0
240 5
260 7
280 7
300 7
320 6
340 4
360 1
GTP-M5:ASP-S1
20 0
40 0
60 0
80 0
100 0
120 0
140 0
160 0
180 0
200 0
220 0
240 0
260 0
280 0
300 0
320 0
340 0

360 0
G-RIB:SER-S1
20 0
40 3
60 5
80 7
100 8
120 7
140 6
160 4
180 1
200 0
220 3
240 5
260 7
280 8
300 7
320 6
340 4
360 1
C-RIB:VAL-S1
20 0
40 2
60 5
80 6
100 7
120 6
140 5
160 3
180 0
200 0
220 0
240 4
260 6
280 7
300 6
320 5
340 4
360 1
G-R5:CYS-S1
20 0
40 0
60 0
80 0
100 0
120 1
140 0
160 0
180 0
200 0
220 0
240 0
260 0
280 0
300 0
320 0
340 0
360 0

M2G-P:GLU-S1
20 0
40 0
60 0
80 0
100 0
120 0
140 0
160 0
180 0
200 0
220 0
240 0
260 0
280 0
300 0
320 0
340 0
360 0
U-Y:ASN-S2
20 0
40 0
60 1
80 2
100 2
120 2
140 1
160 1
180 0
200 0
220 1
240 1
260 2
280 2
300 2
320 2
340 1
360 0
A-RIB:ASN-S1
20 0
40 1
60 3
80 4
100 4
120 4
140 3
160 2
180 1
200 0
220 1
240 3
260 4
280 4
300 4
320 3
340 2
360 1
C-Y:LYS-CA

20   0
40   2
60   4
80   6
100   6
120   6
140   5
160   3
180   1
200   0
220   2
240   4
260   6
280   6
300   6
320   5
340   3
360   1
A-R6:ILE-S1
20   0
40   2
60   4
80   6
100   0
120   6
140   4
160   0
180   1
200   0
220   0
240   4
260   6
280   6
300   6
320   5
340   3
360   0
H2U-MY:GLN-S1
20   0
40   0
60   0
80   0
100   0
120   0
140   0
160   0
180   0
200   0
220   0
240   0
260   0
280   0
300   0
320   0
340   0
360   0
C-Y:CYS-S1
20   0

40 0
60 0
80 1
100 0
120 0
140 0
160 0
180 0
200 0
220 0
240 0
260 0
280 0
300 0
320 0
340 0
360 0
FHU-MY:SER-CA
20 0
40 0
60 0
80 0
100 0
120 0
140 0
160 0
180 0
200 0
220 0
240 0
260 0
280 0
300 0
320 0
340 0
360 0
FHU-MY:ARG-S2
20 0
40 0
60 0
80 0
100 0
120 0
140 0
160 0
180 0
200 0
220 0
240 0
260 0
280 0
300 0
320 0
340 0
360 0
C31-P:GLU-S2
20 0
40 0

60 0
80 0
100 0
120 0
140 0
160 0
180 0
200 0
220 0
240 0
260 0
280 0
300 0
320 0
340 0
360 0
G-RIB:TRP-CA
20 0
40 0
60 0
80 1
100 2
120 0
140 1
160 1
180 0
200 0
220 0
240 1
260 1
280 2
300 1
320 1
340 0
360 0
U31-MY:ASP-S1
20 0
40 0
60 0
80 0
100 0
120 0
140 0
160 0
180 0
200 0
220 0
240 0
260 0
280 0
300 0
320 0
340 0
360 0
A-R6:GLU-S2
20 0
40 3
60 6

80 9
100 9
120 8
140 7
160 5
180 2
200 0
220 3
240 6
260 9
280 9
300 9
320 7
340 5
360 2
C-P:ARG-S1
20 0
40 2
60 4
80 6
100 6
120 5
140 4
160 3
180 1
200 0
220 2
240 4
260 6
280 6
300 6
320 5
340 3
360 1
FHU-RIB:THR-S1
20 0
40 0
60 0
80 0
100 0
120 0
140 0
160 0
180 0
200 0
220 0
240 0
260 0
280 0
300 0
320 0
340 0
360 0
C-P:MET-S2
20 0
40 0
60 1
80 2

100 2
120 2
140 1
160 0
180 0
200 0
220 0
240 1
260 2
280 2
300 2
320 1
340 1
360 0
C-P:VAL-S1
20 0
40 2
60 4
80 6
100 7
120 6
140 5
160 3
180 1
200 0
220 2
240 4
260 6
280 7
300 6
320 5
340 4
360 1
U-Y:ARG-S1
20 0
40 1
60 3
80 4
100 4
120 4
140 3
160 2
180 0
200 0
220 1
240 3
260 4
280 4
300 4
320 3
340 2
360 1
G-R5:ASP-S2
20 0
40 3
60 6
80 8
100 9

120 8
140 6
160 5
180 2
200 0
220 3
240 6
260 8
280 9
300 8
320 7
340 5
360 2
C-P:LYS-CA
20 0
40 2
60 4
80 6
100 6
120 6
140 5
160 3
180 1
200 0
220 2
240 4
260 6
280 6
300 6
320 5
340 3
360 1
H2U-P:ASN-CA
20 0
40 0
60 0
80 0
100 0
120 0
140 0
160 0
180 0
200 0
220 0
240 0
260 0
280 0
300 0
320 0
340 0
360 0
G-P:LEU-S1
20 0
40 4
60 9
80 12
100 13
120 12

140 9
160 7
180 2
200 1
220 5
240 9
260 12
280 13
300 12
320 10
340 7
360 3
FMU-RIB:GLN-S2
20 0
40 0
60 0
80 0
100 0
120 0
140 0
160 0
180 0
200 0
220 0
240 0
260 0
280 0
300 0
320 0
340 0
360 0
QUO-M5:GLU-S2
20 0
40 0
60 0
80 0
100 0
120 0
140 0
160 0
180 0
200 0
220 0
240 0
260 0
280 0
300 0
320 0
340 0
360 0
U31-MY:ILE-CA
20 0
40 0
60 0
80 0
100 0
120 0
140 0

160 0
180 0
200 0
220 0
240 0
260 0
280 0
300 0
320 0
340 0
360 0
QUO-RIB:LEU-S2
20 0
40 0
60 0
80 0
100 0
120 0
140 0
160 0
180 0
200 0
220 0
240 0
260 0
280 0
300 0
320 0
340 0
360 0
U-Y:LYS-S1
20 0
40 1
60 3
80 4
100 4
120 4
140 3
160 2
180 1
200 0
220 1
240 3
260 4
280 4
300 4
320 3
340 2
360 1
U31-MY:VAL-CA
20 0
40 0
60 0
80 0
100 0
120 0
140 0
160 0

180 0
200 0
220 0
240 0
260 0
280 0
300 0
320 0
340 0
360 0
M2G-P:SER-S1
20 0
40 0
60 0
80 0
100 0
120 0
140 0
160 0
180 0
200 0
220 0
240 0
260 0
280 0
300 0
320 0
340 0
360 0
U-RIB:ASP-CA
20 0
40 1
60 2
80 3
100 4
120 3
140 3
160 2
180 0
200 0
220 1
240 2
260 3
280 4
300 3
320 3
340 2
360 0
A-RIB:GLU-CA
20 0
40 3
60 0
80 9
100 9
120 8
140 7
160 5
180 0

200 0
220 0
240 0
260 9
280 9
300 9
320 7
340 5
360 2
IU-P:ALA-CA
20 0
40 0
60 0
80 0
100 0
120 0
140 0
160 0
180 0
200 0
220 0
240 0
260 0
280 0
300 0
320 0
340 0
360 0
C31-P:ASN-S1
20 0
40 0
60 0
80 0
100 0
120 0
140 0
160 0
180 0
200 0
220 0
240 0
260 0
280 0
300 0
320 0
340 0
360 0
A-R6:ASP-S1
20 0
40 2
60 5
80 6
100 7
120 6
140 5
160 4
180 0
200 0

220 2
240 5
260 7
280 7
300 6
320 5
340 4
360 1
5BU-P:ILE-S1
20 0
40 0
60 0
80 0
100 0
120 0
140 0
160 0
180 0
200 0
220 0
240 0
260 0
280 0
300 0
320 0
340 0
360 0
C31-P:GLU-S1
20 0
40 0
60 0
80 0
100 0
120 0
140 0
160 0
180 0
200 0
220 0
240 0
260 0
280 0
300 0
320 0
340 0
360 0
U34-MY:PHE-CA
20 0
40 0
60 0
80 0
100 0
120 0
140 0
160 0
180 0
200 0
220 0

240 0
260 0
280 0
300 0
320 0
340 0
360 0
U-Y:MET-S2
20 0
40 0
60 1
80 1
100 1
120 1
140 1
160 0
180 0
200 0
220 0
240 1
260 1
280 1
300 0
320 1
340 0
360 0
IU-RIB:ALA-S1
20 0
40 0
60 0
80 0
100 0
120 0
140 0
160 0
180 0
200 0
220 0
240 0
260 0
280 0
300 0
320 0
340 0
360 0
QUO-M6:ASP-CA
20 0
40 0
60 0
80 0
100 0
120 0
140 0
160 0
180 0
200 0
220 0
240 0

260 0
280 0
300 0
320 0
340 0
360 0
A-P:TYR-S2
20 0
40 1
60 2
80 3
100 4
120 3
140 2
160 2
180 0
200 0
220 1
240 2
260 3
280 4
300 3
320 3
340 2
360 0
C-RIB:THR-S1
20 0
40 1
60 3
80 4
100 4
120 4
140 3
160 2
180 1
200 0
220 1
240 3
260 4
280 4
300 4
320 3
340 2
360 1
FHU-RIB:ASP-CA
20 0
40 0
60 0
80 0
100 0
120 0
140 0
160 0
180 0
200 0
220 0
240 0
260 0

280 0
300 0
320 0
340 0
360 0
FHU-P:SER-CA
20 0
40 0
60 0
80 0
100 0
120 0
140 0
160 0
180 0
200 0
220 0
240 0
260 0
280 0
300 0
320 0
340 0
360 0
IU-MY:LEU-S2
20 0
40 0
60 0
80 0
100 0
120 0
140 0
160 0
180 0
200 0
220 0
240 0
260 0
280 0
300 0
320 0
340 0
360 0
A-P:ASP-S1
20 0
40 2
60 5
80 6
100 7
120 6
140 5
160 4
180 1
200 0
220 2
240 5
260 7
280 7

300 6
320 5
340 4
360 1
FHU-RIB:ASP-S2
20 0
40 0
60 0
80 0
100 0
120 0
140 0
160 0
180 0
200 0
220 0
240 0
260 0
280 0
300 0
320 0
340 0
360 0
C-RIB:ILE-CA
20 0
40 1
60 3
80 5
100 5
120 4
140 3
160 3
180 1
200 0
220 2
240 0
260 5
280 5
300 5
320 4
340 3
360 1
U31-RIB:GLN-S2
20 0
40 0
60 0
80 0
100 0
120 0
140 0
160 0
180 0
200 0
220 0
240 0
260 0
280 0
300 0

320 0
340 0
360 0
C31-MY:SER-CA
20 0
40 0
60 0
80 0
100 0
120 0
140 0
160 0
180 0
200 0
220 0
240 0
260 0
280 0
300 0
320 0
340 0
360 0
U31-RIB:MET-S1
20 0
40 0
60 0
80 0
100 0
120 0
140 0
160 0
180 0
200 0
220 0
240 0
260 0
280 0
300 0
320 0
340 0
360 0
OMC-RIB:LYS-S1
20 0
40 0
60 0
80 0
100 0
120 0
140 0
160 0
180 0
200 0
220 0
240 0
260 0
280 0
300 0
320 0

340 0
360 0
QUO-M6:PHE-CA
20 0
40 0
60 0
80 0
100 0
120 0
140 0
160 0
180 0
200 0
220 0
240 0
260 0
280 0
300 0
320 0
340 0
360 0
U34-MY:PRO-CA
20 0
40 0
60 0
80 0
100 0
120 0
140 0
160 0
180 0
200 0
220 0
240 0
260 0
280 0
300 0
320 0
340 0
360 0
FHU-RIB:SER-CA
20 0
40 0
60 0
80 0
100 0
120 0
140 0
160 0
180 0
200 0
220 0
240 0
260 0
280 0
300 0
320 0
340 0

360 0
OMC-MY:LYS-S2
20 0
40 0
60 0
80 0
100 0
120 0
140 0
160 0
180 0
200 0
220 0
240 0
260 0
280 0
300 0
320 0
340 0
360 0
GTP-M5:ASN-S1
20 0
40 0
60 0
80 0
100 0
120 0
140 0
160 0
180 0
200 0
220 0
240 0
260 0
280 0
300 0
320 0
340 0
360 0
G-R6:PRO-CA
20 0
40 2
60 4
80 6
100 7
120 6
140 5
160 3
180 0
200 0
220 2
240 4
260 6
280 7
300 0
320 5
340 4
360 1

U-RIB:MET-S1
20 0
40 0
60 1
80 1
100 1
120 1
140 1
160 0
180 0
200 0
220 0
240 1
260 1
280 1
300 1
320 1
340 0
360 0
C31-MY:TYR-S2
20 0
40 0
60 0
80 0
100 0
120 0
140 0
160 0
180 0
200 0
220 0
240 0
260 0
280 0
300 0
320 0
340 0
360 0
FMU-P:ASP-CA
20 0
40 0
60 0
80 0
100 0
120 0
140 0
160 0
180 0
200 0
220 0
240 0
260 0
280 0
300 0
320 0
340 0
360 0
FHU-P:LEU-S1

20 0
40 0
60 0
80 0
100 0
120 0
140 0
160 0
180 0
200 0
220 0
240 0
260 0
280 0
300 0
320 0
340 0
360 0
U31-RIB:GLU-CA
20 0
40 0
60 0
80 0
100 0
120 0
140 0
160 0
180 0
200 0
220 0
240 0
260 0
280 0
300 0
320 0
340 0
360 0
H2U-MY:ASN-CA
20 0
40 0
60 0
80 0
100 0
120 0
140 0
160 0
180 0
200 0
220 0
240 0
260 0
280 0
300 0
320 0
340 0
360 0
G-RIB:LYS-CA
20 1

40 3
60 7
80 9
100 10
120 9
140 7
160 5
180 2
200 0
220 4
240 7
260 10
280 10
300 9
320 8
340 6
360 2
C31-RIB:SER-S1
20 0
40 0
60 0
80 0
100 0
120 0
140 0
160 0
180 0
200 0
220 0
240 0
260 0
280 0
300 0
320 0
340 0
360 0
FHU-RIB:ILE-S1
20 0
40 0
60 0
80 0
100 0
120 0
140 0
160 0
180 0
200 0
220 0
240 0
260 0
280 0
300 0
320 0
340 0
360 0
A-RIB:THR-S1
20 0
40 2

60 4
80 5
100 6
120 5
140 4
160 3
180 1
200 0
220 2
240 4
260 5
280 6
300 5
320 4
340 3
360 1
U-Y:THR-S1
20 0
40 1
60 2
80 3
100 3
120 3
140 2
160 1
180 0
200 0
220 1
240 2
260 3
280 3
300 3
320 2
340 0
360 0
A-RIB:SER-CA
20 0
40 2
60 4
80 6
100 6
120 6
140 4
160 3
180 1
200 0
220 2
240 4
260 6
280 6
300 6
320 5
340 3
360 1
A-R5:ASN-CA
20 0
40 1
60 3

80   4
100   4
120   4
140   3
160   0
180   0
200   0
220   1
240   3
260   4
280   4
300   4
320   3
340   2
360   1
A-RIB:ALA-CA
20   1
40   3
60   7
80   9
100   10
120   9
140   7
160   5
180   0
200   0
220   3
240   7
260   9
280   10
300   9
320   7
340   5
360   0
A-P:GLN-S1
20   0
40   1
60   3
80   4
100   4
120   3
140   3
160   2
180   0
200   0
220   1
240   3
260   4
280   4
300   4
320   3
340   2
360   0
FHU-MY:PRO-S1
20   0
40   0
60   0
80   0

100 0
120 0
140 0
160 0
180 0
200 0
220 0
240 0
260 0
280 0
300 0
320 0
340 0
360 0
U34-RIB:SER-CA
20 0
40 0
60 0
80 0
100 0
120 0
140 0
160 0
180 0
200 0
220 0
240 0
260 0
280 0
300 0
320 0
340 0
360 0
DA-M6:SER-S1
20 0
40 0
60 0
80 0
100 0
120 0
140 0
160 0
180 0
200 0
220 0
240 0
260 0
280 0
300 0
320 0
340 0
360 0
U-P:LEU-S2
20 0
40 2
60 4
80 5
100 5

120 5
140 4
160 3
180 0
200 0
220 2
240 4
260 5
280 5
300 5
320 4
340 3
360 0
FHU-P:ASP-S1
20 0
40 0
60 0
80 0
100 0
120 0
140 0
160 0
180 0
200 0
220 0
240 0
260 0
280 0
300 0
320 0
340 0
360 0
QUO-M6:GLU-S1
20 0
40 0
60 0
80 0
100 0
120 0
140 0
160 0
180 0
200 0
220 0
240 0
260 0
280 0
300 0
320 0
340 0
360 0
QUO-M6:ASN-CA
20 0
40 0
60 0
80 0
100 0
120 0

140 0
160 0
180 0
200 0
220 0
240 0
260 0
280 0
300 0
320 0
340 0
360 0
G-P:SER-CA
20 0
40 3
60 5
80 7
100 8
120 7
140 6
160 4
180 1
200 0
220 3
240 5
260 7
280 8
300 7
320 6
340 4
360 1
C-RIB:SER-CA
20 0
40 1
60 3
80 5
100 5
120 4
140 3
160 2
180 1
200 0
220 2
240 3
260 5
280 5
300 4
320 4
340 3
360 1
C-P:CYS-S1
20 0
40 0
60 0
80 0
100 0
120 0
140 0

160 0
180 0
200 0
220 0
240 0
260 0
280 1
300 0
320 0
340 0
360 0
G-RIB:ASN-S2
20 0
40 2
60 4
80 5
100 6
120 5
140 4
160 3
180 1
200 0
220 2
240 4
260 5
280 6
300 5
320 4
340 3
360 1
C31-MY:THR-CA
20 0
40 0
60 0
80 0
100 0
120 0
140 0
160 0
180 0
200 0
220 0
240 0
260 0
280 0
300 0
320 0
340 0
360 0
U-RIB:LEU-S2
20 0
40 0
60 0
80 5
100 5
120 5
140 4
160 3

180   1
200   0
220   2
240   4
260   5
280   5
300   5
320   4
340   3
360   1
C-Y:THR-S1
20   0
40   1
60   3
80   4
100   4
120   4
140   3
160   2
180   1
200   0
220   1
240   3
260   4
280   4
300   4
320   3
340   2
360   1
H2U-RIB:GLU-S1
20   0
40   0
60   0
80   0
100   0
120   0
140   0
160   0
180   0
200   0
220   0
240   0
260   0
280   0
300   0
320   0
340   0
360   0
U-RIB:GLU-S2
20   0
40   0
60   3
80   5
100   5
120   4
140   3
160   2
180   1

200   0
220   2
240   3
260   5
280   5
300   4
320   4
340   3
360   1
A-P:TYR-CA
20   0
40   0
60   2
80   3
100   4
120   3
140   2
160   2
180   0
200   0
220   1
240   2
260   3
280   4
300   3
320   3
340   2
360   0
G-P:LYS-S2
20   1
40   3
60   7
80   9
100   10
120   9
140   7
160   5
180   2
200   0
220   3
240   7
260   9
280   10
300   9
320   8
340   5
360   2
G-P:ILE-S1
20   0
40   3
60   5
80   7
100   8
120   7
140   6
160   4
180   0
200   0

220   3
240   5
260   7
280   8
300   7
320   6
340   4
360   1
U31-MY:TYR-S2
20   0
40   0
60   0
80   0
100   0
120   0
140   0
160   0
180   0
200   0
220   0
240   0
260   0
280   0
300   0
320   0
340   0
360   0
C31-RIB:PHE-S1
20   0
40   0
60   0
80   0
100   0
120   0
140   0
160   0
180   0
200   0
220   0
240   0
260   0
280   0
300   0
320   0
340   0
360   0
C-RIB:PHE-S2
20   0
40   0
60   2
80   3
100   3
120   3
140   2
160   2
180   0
200   0
220   0

240 0
260 3
280 3
300 3
320 2
340 2
360 0
U-RIB:ASN-S1
20 0
40 0
60 1
80 2
100 2
120 2
140 1
160 1
180 0
200 0
220 1
240 1
260 2
280 2
300 2
320 2
340 1
360 0
DA-M5:ASN-CA
20 0
40 0
60 0
80 0
100 0
120 0
140 0
160 0
180 0
200 0
220 0
240 0
260 0
280 0
300 0
320 0
340 0
360 0
U-P:LYS-S2
20 0
40 1
60 3
80 4
100 4
120 4
140 3
160 2
180 1
200 0
220 1
240 3

260 4
280 4
300 4
320 3
340 2
360 1
U31-MY:MET-S2
20 0
40 0
60 0
80 0
100 0
120 0
140 0
160 0
180 0
200 0
220 0
240 0
260 0
280 0
300 0
320 0
340 0
360 0
U-Y:TRP-S1
20 0
40 0
60 0
80 0
100 0
120 0
140 0
160 0
180 0
200 0
220 0
240 0
260 0
280 0
300 0
320 0
340 0
360 0
FHU-P:LYS-CA
20 0
40 0
60 0
80 0
100 0
120 0
140 0
160 0
180 0
200 0
220 0
240 0
260 0

280 0
300 0
320 0
340 0
360 0
U-P:GLY-CA
20 0
40 1
60 3
80 4
100 4
120 4
140 3
160 2
180 1
200 0
220 1
240 3
260 4
280 4
300 4
320 3
340 2
360 1
U-P:TRP-CA
20 0
40 0
60 0
80 0
100 0
120 0
140 0
160 0
180 0
200 0
220 0
240 0
260 0
280 0
300 0
320 0
340 0
360 0
DA-M5:SER-S1
20 0
40 0
60 0
80 0
100 0
120 0
140 0
160 0
180 0
200 0
220 0
240 0
260 0
280 0

300 0
320 0
340 0
360 0
C-RIB:TRP-S2
20 0
40 0
60 0
80 1
100 1
120 1
140 0
160 0
180 0
200 0
220 0
240 0
260 1
280 1
300 1
320 1
340 0
360 0
U31-P:ARG-S2
20 0
40 0
60 0
80 0
100 0
120 0
140 0
160 0
180 0
200 0
220 0
240 0
260 0
280 0
300 0
320 0
340 0
360 0
G-R5:THR-CA
20 0
40 2
60 5
80 7
100 7
120 6
140 5
160 4
180 0
200 0
220 2
240 5
260 7
280 7
300 7

320 6
340 0
360 0
QUO-P:LEU-CA
20 0
40 0
60 0
80 0
100 0
120 0
140 0
160 0
180 0
200 0
220 0
240 0
260 0
280 0
300 0
320 0
340 0
360 0
C31-RIB:ALA-S1
20 0
40 0
60 0
80 0
100 0
120 0
140 0
160 0
180 0
200 0
220 0
240 0
260 0
280 0
300 0
320 0
340 0
360 0
G-R5:GLU-S2
20 1
40 4
60 8
80 11
100 12
120 11
140 8
160 6
180 2
200 1
220 4
240 8
260 11
280 12
300 11
320 9

340 7
360 0
FHU-MY:ASP-S2
20 0
40 0
60 0
80 0
100 0
120 0
140 0
160 0
180 0
200 0
220 0
240 0
260 0
280 0
300 0
320 0
340 0
360 0
G-R5:SER-S1
20 0
40 3
60 5
80 7
100 8
120 7
140 6
160 4
180 1
200 0
220 3
240 5
260 7
280 8
300 7
320 6
340 4
360 1
U-Y:ASP-S1
20 0
40 1
60 2
80 3
100 4
120 3
140 3
160 2
180 0
200 0
220 1
240 2
260 3
280 0
300 3
320 0
340 0

360 0
U31-RIB:ASN-S2
20 0
40 0
60 0
80 0
100 0
120 0
140 0
160 0
180 0
200 0
220 0
240 0
260 0
280 0
300 0
320 0
340 0
360 0
IU-MY:MET-CA
20 0
40 0
60 0
80 0
100 0
120 0
140 0
160 0
180 0
200 0
220 0
240 0
260 0
280 0
300 0
320 0
340 0
360 0
U-RIB:THR-CA
20 0
40 1
60 2
80 3
100 3
120 3
140 2
160 1
180 0
200 0
220 1
240 2
260 3
280 3
300 3
320 2
340 1
360 0

U-RIB:MET-CA
20 0
40 0
60 1
80 1
100 1
120 1
140 0
160 0
180 0
200 0
220 0
240 0
260 1
280 1
300 1
320 0
340 0
360 0
G-R5:PHE-CA
20 0
40 2
60 4
80 5
100 5
120 0
140 0
160 3
180 0
200 0
220 2
240 4
260 5
280 5
300 0
320 4
340 0
360 0
A-P:HIS-S1
20 0
40 1
60 2
80 2
100 2
120 2
140 2
160 1
180 0
200 0
220 1
240 2
260 2
280 2
300 2
320 2
340 1
360 0
GTP-M5:THR-S1

20 0
40 0
60 0
80 0
100 0
120 0
140 0
160 0
180 0
200 0
220 0
240 0
260 0
280 0
300 0
320 0
340 0
360 0
C31-MY:ALA-S1
20 0
40 0
60 0
80 0
100 0
120 0
140 0
160 0
180 0
200 0
220 0
240 0
260 0
280 0
300 0
320 0
340 0
360 0
C-RIB:LYS-S2
20 0
40 2
60 4
80 6
100 6
120 6
140 4
160 3
180 1
200 0
220 2
240 4
260 6
280 6
300 6
320 5
340 3
360 1
G-R6:PRO-S1
20 0

40   2
60   4
80   6
100   7
120   6
140   5
160   3
180   0
200   0
220   2
240   4
260   6
280   7
300   6
320   5
340   4
360   0
A-R6:MET-CA
20   0
40   0
60   2
80   2
100   3
120   2
140   2
160   0
180   0
200   0
220   0
240   2
260   2
280   3
300   2
320   2
340   1
360   0
FHU-RIB:ALA-S1
20   0
40   0
60   0
80   0
100   0
120   0
140   0
160   0
180   0
200   0
220   0
240   0
260   0
280   0
300   0
320   0
340   0
360   0
U34-MY:ASP-CA
20   0
40   0

60 0
80 0
100 0
120 0
140 0
160 0
180 0
200 0
220 0
240 0
260 0
280 0
300 0
320 0
340 0
360 0
U-RIB:GLN-CA
20 0
40 0
60 1
80 2
100 2
120 2
140 1
160 1
180 0
200 0
220 0
240 1
260 2
280 2
300 2
320 1
340 1
360 0
DA-M5:LYS-S1
20 0
40 0
60 0
80 0
100 0
120 0
140 0
160 0
180 0
200 0
220 0
240 0
260 0
280 0
300 0
320 0
340 0
360 0
G-RIB:ASP-CA
20 0
40 3
60 6

80   8
100   9
120   8
140   6
160   5
180   2
200   0
220   3
240   6
260   8
280   9
300   8
320   7
340   5
360   2
H2U-P:ASN-S2
20   0
40   0
60   0
80   0
100   0
120   0
140   0
160   0
180   0
200   0
220   0
240   0
260   0
280   0
300   0
320   0
340   0
360   0
FHU-RIB:ALA-CA
20   0
40   0
60   0
80   0
100   0
120   0
140   0
160   0
180   0
200   0
220   0
240   0
260   0
280   0
300   0
320   0
340   0
360   0
U-Y:CYS-S1
20   0
40   0
60   0
80   0

100 0
120 0
140 0
160 0
180 0
200 0
220 0
240 0
260 0
280 0
300 0
320 0
340 0
360 0
U31-P:TYR-S1
20 0
40 0
60 0
80 0
100 0
120 0
140 0
160 0
180 0
200 0
220 0
240 0
260 0
280 0
300 0
320 0
340 0
360 0
GTP-M6:ASN-CA
20 0
40 0
60 0
80 0
100 0
120 0
140 0
160 0
180 0
200 0
220 0
240 0
260 0
280 0
300 0
320 0
340 0
360 0
C-RIB:GLN-CA
20 0
40 1
60 2
80 3
100 3

120 3
140 2
160 1
180 0
200 0
220 1
240 2
260 3
280 3
300 3
320 2
340 2
360 0
A-RIB:LEU-CA
20 0
40 3
60 7
80 10
100 10
120 9
140 7
160 5
180 2
200 0
220 4
240 0
260 10
280 10
300 9
320 8
340 6
360 2
U-RIB:VAL-CA
20 0
40 0
60 3
80 4
100 4
120 4
140 3
160 0
180 1
200 0
220 0
240 0
260 4
280 4
300 4
320 3
340 2
360 1
GTP-M5:SER-CA
20 0
40 0
60 0
80 0
100 0
120 0

140 0
160 0
180 0
200 0
220 0
240 0
260 0
280 0
300 0
320 0
340 0
360 0
U-RIB:TYR-S1
20 0
40 0
60 0
80 2
100 2
120 1
140 1
160 1
180 0
200 0
220 0
240 0
260 0
280 2
300 2
320 1
340 1
360 0
A-P:PHE-S2
20 0
40 1
60 3
80 4
100 4
120 4
140 3
160 2
180 1
200 0
220 1
240 3
260 4
280 4
300 4
320 3
340 2
360 0
IU-P:ILE-S1
20 0
40 0
60 0
80 0
100 0
120 0
140 0

160 0
180 0
200 0
220 0
240 0
260 0
280 0
300 0
320 0
340 0
360 0
G-RIB:ILE-S1
20 0
40 3
60 0
80 7
100 8
120 7
140 6
160 0
180 0
200 0
220 3
240 0
260 7
280 8
300 7
320 6
340 4
360 1
A-R5:THR-S1
20 0
40 2
60 4
80 5
100 6
120 5
140 4
160 3
180 1
200 0
220 2
240 4
260 5
280 6
300 5
320 4
340 3
360 0
C-Y:TYR-S2
20 0
40 1
60 2
80 2
100 3
120 2
140 2
160 1

180 0
200 0
220 1
240 2
260 2
280 3
300 2
320 2
340 1
360 0
G-R5:GLU-S1
20 0
40 4
60 8
80 11
100 12
120 0
140 9
160 6
180 2
200 1
220 4
240 8
260 11
280 12
300 11
320 9
340 7
360 2
C31-RIB:GLU-S1
20 0
40 0
60 0
80 0
100 0
120 0
140 0
160 0
180 0
200 0
220 0
240 0
260 0
280 0
300 0
320 0
340 0
360 0
A-R6:TRP-S1
20 0
40 0
60 0
80 1
100 1
120 1
140 0
160 0
180 0

200 0
220 0
240 1
260 1
280 1
300 0
320 1
340 0
360 0
G-RIB:HIS-S2
20 0
40 1
60 2
80 3
100 3
120 3
140 2
160 2
180 0
200 0
220 1
240 2
260 3
280 3
300 3
320 2
340 2
360 0
G-P:PHE-S1
20 0
40 0
60 3
80 5
100 5
120 5
140 4
160 3
180 0
200 0
220 0
240 3
260 5
280 5
300 5
320 4
340 3
360 1
IU-RIB:PRO-S1
20 0
40 0
60 0
80 0
100 0
120 0
140 0
160 0
180 0
200 0

220 0
240 0
260 0
280 0
300 0
320 0
340 0
360 0
FMU-RIB:ALA-CA
20 0
40 0
60 0
80 0
100 0
120 0
140 0
160 0
180 0
200 0
220 0
240 0
260 0
280 0
300 0
320 0
340 0
360 0
FHU-MY:ALA-CA
20 0
40 0
60 0
80 0
100 0
120 0
140 0
160 0
180 0
200 0
220 0
240 0
260 0
280 0
300 0
320 0
340 0
360 0
QUO-RIB:ASN-S2
20 0
40 0
60 0
80 0
100 0
120 0
140 0
160 0
180 0
200 0
220 0

240 0
260 0
280 0
300 0
320 0
340 0
360 0
A-RIB:SER-S1
20 0
40 2
60 4
80 6
100 6
120 6
140 4
160 3
180 1
200 0
220 2
240 4
260 6
280 6
300 6
320 5
340 3
360 1
A-RIB:MET-S1
20 0
40 0
60 0
80 2
100 3
120 2
140 2
160 1
180 0
200 0
220 1
240 2
260 2
280 3
300 2
320 2
340 1
360 0
U-RIB:PHE-S2
20 0
40 0
60 1
80 2
100 2
120 2
140 1
160 1
180 0
200 0
220 0
240 1

260 0
280 2
300 2
320 2
340 1
360 0
C31-P:ALA-CA
20 0
40 0
60 0
80 0
100 0
120 0
140 0
160 0
180 0
200 0
220 0
240 0
260 0
280 0
300 0
320 0
340 0
360 0
C-P:PRO-S1
20 0
40 1
60 3
80 4
100 4
120 4
140 3
160 2
180 0
200 0
220 1
240 3
260 4
280 4
300 4
320 3
340 2
360 1
U31-RIB:THR-S1
20 0
40 0
60 0
80 0
100 0
120 0
140 0
160 0
180 0
200 0
220 0
240 0
260 0

280 0
300 0
320 0
340 0
360 0
C-P:LYS-S2
20 0
40 2
60 4
80 6
100 6
120 6
140 4
160 3
180 1
200 0
220 2
240 4
260 6
280 6
300 6
320 5
340 3
360 1
QUO-M5:LEU-S1
20 0
40 0
60 0
80 0
100 0
120 0
140 0
160 0
180 0
200 0
220 0
240 0
260 0
280 0
300 0
320 0
340 0
360 0
G-P:THR-S1
20 0
40 0
60 5
80 7
100 7
120 6
140 5
160 4
180 1
200 0
220 0
240 5
260 7
280 7

300 7
320 5
340 4
360 1
G-R6:LYS-S2
20 0
40 3
60 7
80 9
100 10
120 9
140 7
160 5
180 2
200 0
220 4
240 7
260 9
280 10
300 9
320 8
340 6
360 0
C-RIB:HIS-S2
20 0
40 0
60 1
80 2
100 2
120 2
140 1
160 1
180 0
200 0
220 0
240 1
260 2
280 2
300 2
320 1
340 1
360 0
C-RIB:GLU-CA
20 0
40 2
60 5
80 7
100 7
120 7
140 5
160 4
180 1
200 0
220 3
240 5
260 7
280 7
300 7

320 6
340 4
360 1
U31-MY:PHE-S2
20 0
40 0
60 0
80 0
100 0
120 0
140 0
160 0
180 0
200 0
220 0
240 0
260 0
280 0
300 0
320 0
340 0
360 0
H2U-MY:LEU-S2
20 0
40 0
60 0
80 0
100 0
120 0
140 0
160 0
180 0
200 0
220 0
240 0
260 0
280 0
300 0
320 0
340 0
360 0
C-RIB:ARG-S1
20 0
40 2
60 4
80 6
100 6
120 5
140 4
160 3
180 1
200 0
220 2
240 4
260 6
280 6
300 6
320 5

340 3
360 1
U-P:LYS-CA
20 0
40 0
60 3
80 4
100 4
120 4
140 3
160 2
180 1
200 0
220 1
240 3
260 4
280 4
300 4
320 3
340 2
360 1
DA-M5:GLN-CA
20 0
40 0
60 0
80 0
100 0
120 0
140 0
160 0
180 0
200 0
220 0
240 0
260 0
280 0
300 0
320 0
340 0
360 0
G-R6:VAL-CA
20 0
40 0
60 7
80 10
100 11
120 10
140 8
160 0
180 2
200 0
220 4
240 7
260 10
280 11
300 10
320 8
340 6

360 0
FHU-P:ALA-S1
20 0
40 0
60 0
80 0
100 0
120 0
140 0
160 0
180 0
200 0
220 0
240 0
260 0
280 0
300 0
320 0
340 0
360 0
C-RIB:MET-S1
20 0
40 0
60 0
80 2
100 2
120 2
140 0
160 1
180 0
200 0
220 0
240 1
260 2
280 2
300 2
320 1
340 0
360 0
A-R6:SER-S1
20 0
40 2
60 4
80 6
100 6
120 6
140 4
160 3
180 1
200 0
220 2
240 4
260 6
280 6
300 6
320 5
340 3
360 1

A-P:ILE-CA
20   0
40   0
60   4
80   6
100   6
120   6
140   4
160   3
180   1
200   0
220   2
240   4
260   6
280   6
300   6
320   5
340   3
360   1
DA-M6:TYR-S1
20   0
40   0
60   0
80   0
100   0
120   0
140   0
160   0
180   0
200   0
220   0
240   0
260   0
280   0
300   0
320   0
340   0
360   0
QUO-RIB:PHE-S2
20   0
40   0
60   0
80   0
100   0
120   0
140   0
160   0
180   0
200   0
220   0
240   0
260   0
280   0
300   0
320   0
340   0
360   0
DA-M5:TYR-CA

20 0
40 0
60 0
80 0
100 0
120 0
140 0
160 0
180 0
200 0
220 0
240 0
260 0
280 0
300 0
320 0
340 0
360 0
FHU-P:GLY-CA
20 0
40 0
60 0
80 0
100 0
120 0
140 0
160 0
180 0
200 0
220 0
240 0
260 0
280 0
300 0
320 0
340 0
360 0
5BU-P:ALA-S1
20 0
40 0
60 0
80 0
100 0
120 0
140 0
160 0
180 0
200 0
220 0
240 0
260 0
280 0
300 0
320 0
340 0
360 0
FMU-RIB:GLU-S1
20 0

40 0
60 0
80 0
100 0
120 0
140 0
160 0
180 0
200 0
220 0
240 0
260 0
280 0
300 0
320 0
340 0
360 0
C31-P:ASN-CA
20 0
40 0
60 0
80 0
100 0
120 0
140 0
160 0
180 0
200 0
220 0
240 0
260 0
280 0
300 0
320 0
340 0
360 0
U-Y:PHE-CA
20 0
40 0
60 1
80 2
100 0
120 2
140 1
160 0
180 0
200 0
220 0
240 1
260 2
280 2
300 2
320 2
340 0
360 0
U-RIB:PRO-CA
20 0
40 1

60 2
80 2
100 3
120 2
140 2
160 1
180 0
200 0
220 0
240 2
260 2
280 3
300 2
320 2
340 0
360 0
C-P:PHE-CA
20 0
40 0
60 2
80 3
100 3
120 3
140 2
160 2
180 0
200 0
220 0
240 2
260 3
280 3
300 3
320 0
340 2
360 0
U34-P:ASN-CA
20 0
40 0
60 0
80 0
100 0
120 0
140 0
160 0
180 0
200 0
220 0
240 0
260 0
280 0
300 0
320 0
340 0
360 0
C-RIB:TYR-CA
20 0
40 0
60 2

80 2
100 3
120 2
140 0
160 0
180 0
200 0
220 1
240 2
260 2
280 3
300 2
320 2
340 1
360 0
U34-P:SER-S1
20 0
40 0
60 0
80 0
100 0
120 0
140 0
160 0
180 0
200 0
220 0
240 0
260 0
280 0
300 0
320 0
340 0
360 0
C-RIB:HIS-S1
20 0
40 0
60 1
80 2
100 2
120 2
140 1
160 1
180 0
200 0
220 0
240 1
260 2
280 2
300 2
320 1
340 1
360 0
C31-P:TYR-S2
20 0
40 0
60 0
80 0

100 0
120 0
140 0
160 0
180 0
200 0
220 0
240 0
260 0
280 0
300 0
320 0
340 0
360 0
C-P:TRP-S1
20 0
40 0
60 0
80 1
100 1
120 1
140 0
160 0
180 0
200 0
220 0
240 0
260 1
280 1
300 0
320 1
340 0
360 0
H2U-P:LEU-S1
20 0
40 0
60 0
80 0
100 0
120 0
140 0
160 0
180 0
200 0
220 0
240 0
260 0
280 0
300 0
320 0
340 0
360 0
U-Y:MET-CA
20 0
40 0
60 1
80 1
100 0

120   0
140   0
160   0
180   0
200   0
220   0
240   1
260   1
280   0
300   1
320   1
340   0
360   0
U-RIB:ARG-S1
20   0
40   1
60   3
80   4
100   4
120   4
140   3
160   2
180   0
200   0
220   1
240   3
260   4
280   4
300   4
320   3
340   2
360   1
G-R5:ILE-CA
20   0
40   0
60   5
80   7
100   8
120   0
140   0
160   0
180   0
200   0
220   3
240   5
260   7
280   8
300   7
320   0
340   4
360   0
C31-RIB:LEU-S2
20   0
40   0
60   0
80   0
100   0
120   0

140 0
160 0
180 0
200 0
220 0
240 0
260 0
280 0
300 0
320 0
340 0
360 0
FMU-RIB:ILE-CA
20 0
40 0
60 0
80 0
100 0
120 0
140 0
160 0
180 0
200 0
220 0
240 0
260 0
280 0
300 0
320 0
340 0
360 0
G-RIB:ASP-S1
20 0
40 3
60 6
80 8
100 9
120 8
140 6
160 5
180 2
200 0
220 3
240 6
260 8
280 9
300 8
320 7
340 5
360 2
U-RIB:TRP-S1
20 0
40 0
60 0
80 0
100 0
120 0
140 0

160 0
180 0
200 0
220 0
240 0
260 0
280 0
300 0
320 0
340 0
360 0
G-P:TRP-S1
20 0
40 0
60 1
80 0
100 2
120 0
140 1
160 1
180 0
200 0
220 0
240 1
260 1
280 2
300 1
320 1
340 0
360 0
FMU-MY:GLN-S1
20 0
40 0
60 0
80 0
100 0
120 0
140 0
160 0
180 0
200 0
220 0
240 0
260 0
280 0
300 0
320 0
340 0
360 0
FHU-P:THR-S1
20 0
40 0
60 0
80 0
100 0
120 0
140 0
160 0

180 0
200 0
220 0
240 0
260 0
280 0
300 0
320 0
340 0
360 0
U31-RIB:GLN-CA
20 0
40 0
60 0
80 0
100 0
120 0
140 0
160 0
180 0
200 0
220 0
240 0
260 0
280 0
300 0
320 0
340 0
360 0
C-Y:LEU-CA
20 0
40 3
60 5
80 7
100 8
120 7
140 6
160 4
180 0
200 0
220 3
240 5
260 8
280 8
300 7
320 6
340 4
360 1
FMU-RIB:MET-S1
20 0
40 0
60 0
80 0
100 0
120 0
140 0
160 0
180 0

200 0
220 0
240 0
260 0
280 0
300 0
320 0
340 0
360 0
A-RIB:HIS-CA
20 0
40 1
60 2
80 2
100 2
120 2
140 2
160 1
180 0
200 0
220 0
240 2
260 2
280 2
300 2
320 2
340 1
360 0
FMU-MY:PHE-S1
20 0
40 0
60 0
80 0
100 0
120 0
140 0
160 0
180 0
200 0
220 0
240 0
260 0
280 0
300 0
320 0
340 0
360 0
U34-MY:SER-S1
20 0
40 0
60 0
80 0
100 0
120 0
140 0
160 0
180 0
200 0

220   0
240   0
260   0
280   0
300   0
320   0
340   0
360   0
A-RIB:HIS-S1
20   0
40   1
60   2
80   2
100   2
120   2
140   2
160   1
180   0
200   0
220   0
240   2
260   2
280   2
300   2
320   2
340   1
360   0
FMU-MY:SER-CA
20   0
40   0
60   0
80   0
100   0
120   0
140   0
160   0
180   0
200   0
220   0
240   0
260   0
280   0
300   0
320   0
340   0
360   0
U-P:ILE-CA
20   0
40   0
60   2
80   0
100   3
120   3
140   2
160   2
180   0
200   0
220   0

240 2
260 3
280 3
300 3
320 2
340 2
360 0
H2U-RIB:PHE-CA
20 0
40 0
60 0
80 0
100 0
120 0
140 0
160 0
180 0
200 0
220 0
240 0
260 0
280 0
300 0
320 0
340 0
360 0
C-P:PRO-CA
20 0
40 1
60 3
80 4
100 4
120 4
140 3
160 2
180 0
200 0
220 0
240 3
260 4
280 4
300 4
320 3
340 2
360 1
C31-P:MET-S2
20 0
40 0
60 0
80 0
100 0
120 0
140 0
160 0
180 0
200 0
220 0
240 0

260 0
280 0
300 0
320 0
340 0
360 0
G-P:ASN-S2
20 0
40 2
60 4
80 5
100 6
120 5
140 4
160 3
180 1
200 0
220 2
240 4
260 5
280 6
300 5
320 4
340 3
360 1
DA-M6:HIS-CA
20 0
40 0
60 0
80 0
100 0
120 0
140 0
160 0
180 0
200 0
220 0
240 0
260 0
280 0
300 0
320 0
340 0
360 0
A-R5:TRP-S1
20 0
40 0
60 0
80 1
100 1
120 1
140 1
160 0
180 0
200 0
220 0
240 1
260 1

280 1
300 1
320 1
340 0
360 0
4SU-P:GLU-S2
20 0
40 0
60 0
80 0
100 0
120 0
140 0
160 0
180 0
200 0
220 0
240 0
260 0
280 0
300 0
320 0
340 0
360 0
A-P:HIS-CA
20 0
40 1
60 2
80 2
100 2
120 2
140 2
160 1
180 0
200 0
220 1
240 2
260 2
280 2
300 2
320 2
340 1
360 0
A-RIB:THR-CA
20 0
40 2
60 4
80 5
100 6
120 5
140 4
160 3
180 1
200 0
220 2
240 4
260 5
280 6

300 5
320 4
340 3
360 1
A-RIB:GLN-S2
20 0
40 1
60 3
80 4
100 4
120 3
140 3
160 2
180 0
200 0
220 1
240 3
260 4
280 4
300 4
320 3
340 2
360 0
FMU-MY:CYS-CA
20 0
40 0
60 0
80 0
100 0
120 0
140 0
160 0
180 0
200 0
220 0
240 0
260 0
280 0
300 0
320 0
340 0
360 0
U34-RIB:ASN-S1
20 0
40 0
60 0
80 0
100 0
120 0
140 0
160 0
180 0
200 0
220 0
240 0
260 0
280 0
300 0

320 0
340 0
360 0
A-R5:MET-S1
20 0
40 0
60 2
80 2
100 3
120 2
140 2
160 0
180 0
200 0
220 1
240 2
260 2
280 0
300 2
320 2
340 1
360 0
U-P:ASN-S1
20 0
40 0
60 1
80 2
100 2
120 2
140 1
160 1
180 0
200 0
220 1
240 1
260 2
280 2
300 2
320 2
340 1
360 0
G-RIB:ARG-S2
20 1
40 3
60 7
80 9
100 10
120 9
140 7
160 5
180 2
200 0
220 3
240 7
260 9
280 10
300 9
320 8

340 5
360 2
C-Y:ARG-CA
20 0
40 2
60 4
80 6
100 6
120 5
140 4
160 3
180 1
200 0
220 2
240 4
260 6
280 6
300 6
320 5
340 3
360 0
G-R5:HIS-CA
20 0
40 1
60 2
80 3
100 3
120 3
140 2
160 0
180 0
200 0
220 1
240 2
260 3
280 3
300 3
320 2
340 2
360 0
A-RIB:CYS-S1
20 0
40 0
60 0
80 0
100 1
120 1
140 0
160 0
180 0
200 0
220 0
240 0
260 0
280 0
300 1
320 0
340 0

360 0
C-RIB:ASN-S1
20 0
40 1
60 2
80 3
100 3
120 3
140 2
160 2
180 0
200 0
220 1
240 2
260 3
280 3
300 3
320 3
340 2
360 0
A-R6:ALA-S1
20 0
40 3
60 7
80 9
100 10
120 9
140 7
160 5
180 0
200 0
220 3
240 7
260 9
280 10
300 9
320 7
340 5
360 2
U-RIB:SER-S1
20 0
40 1
60 2
80 3
100 3
120 3
140 2
160 2
180 0
200 0
220 1
240 2
260 3
280 3
300 3
320 2
340 2
360 0

QUO-M5:ASN-CA
20   0
40   0
60   0
80   0
100   0
120   0
140   0
160   0
180   0
200   0
220   0
240   0
260   0
280   0
300   0
320   0
340   0
360   0
C-RIB:LEU-S1
20   0
40   3
60   5
80   7
100   8
120   7
140   6
160   4
180   1
200   0
220   0
240   5
260   8
280   8
300   7
320   6
340   4
360   1
A-RIB:ASP-CA
20   0
40   0
60   0
80   6
100   7
120   6
140   5
160   4
180   1
200   0
220   2
240   5
260   7
280   7
300   6
320   5
340   4
360   0
IU-P:ARG-S1

20 0
40 0
60 0
80 0
100 0
120 0
140 0
160 0
180 0
200 0
220 0
240 0
260 0
280 0
300 0
320 0
340 0
360 0
G-R5:GLN-S2
20 0
40 1
60 3
80 5
100 5
120 4
140 3
160 0
180 0
200 0
220 2
240 3
260 5
280 5
300 5
320 4
340 3
360 1
G-R5:ASN-S1
20 0
40 2
60 4
80 5
100 6
120 5
140 4
160 3
180 1
200 0
220 2
240 4
260 5
280 6
300 5
320 4
340 3
360 1
FMU-MY:ILE-CA
20 0

40   0
60   0
80   0
100   0
120   0
140   0
160   0
180   0
200   0
220   0
240   0
260   0
280   0
300   0
320   0
340   0
360   0
C-Y:ALA-S1
20   0
40   2
60   5
80   7
100   8
120   7
140   5
160   4
180   0
200   0
220   3
240   5
260   7
280   8
300   7
320   6
340   4
360   0
C-P:ILE-S1
20   0
40   1
60   3
80   5
100   5
120   4
140   3
160   3
180   1
200   0
220   2
240   3
260   5
280   5
300   5
320   4
340   0
360   1
5BU-MY:ILE-S1
20   0
40   0

60 0
80 0
100 0
120 0
140 0
160 0
180 0
200 0
220 0
240 0
260 0
280 0
300 0
320 0
340 0
360 0
U-RIB:GLN-S2
20 0
40 0
60 1
80 2
100 2
120 2
140 1
160 1
180 0
200 0
220 0
240 1
260 2
280 2
300 2
320 1
340 1
360 0
C-P:HIS-S2
20 0
40 0
60 1
80 2
100 2
120 2
140 1
160 1
180 0
200 0
220 0
240 1
260 2
280 2
300 2
320 1
340 1
360 0
G-RIB:GLY-CA
20 1
40 3
60 7

80 10
100 10
120 9
140 7
160 6
180 2
200 0
220 4
240 7
260 10
280 10
300 10
320 8
340 6
360 2
U-P:HIS-S2
20 0
40 0
60 1
80 1
100 1
120 1
140 1
160 0
180 0
200 0
220 0
240 1
260 1
280 1
300 1
320 1
340 0
360 0
H2U-MY:PRO-S1
20 0
40 0
60 0
80 0
100 0
120 0
140 0
160 0
180 0
200 0
220 0
240 0
260 0
280 0
300 0
320 0
340 0
360 0
G-R6:CYS-CA
20 0
40 0
60 0
80 1

100 0
120 1
140 1
160 0
180 0
200 0
220 0
240 0
260 0
280 0
300 1
320 0
340 0
360 0
C-Y:THR-CA
20 0
40 1
60 3
80 4
100 4
120 4
140 3
160 2
180 1
200 0
220 1
240 3
260 4
280 4
300 4
320 3
340 2
360 0
A-P:GLU-S1
20 0
40 3
60 6
80 9
100 9
120 8
140 7
160 5
180 0
200 0
220 3
240 6
260 9
280 9
300 9
320 7
340 5
360 2
IU-RIB:ARG-S1
20 0
40 0
60 0
80 0
100 0

120 0
140 0
160 0
180 0
200 0
220 0
240 0
260 0
280 0
300 0
320 0
340 0
360 0
C-Y:LYS-S2
20 0
40 2
60 4
80 6
100 6
120 6
140 4
160 3
180 1
200 0
220 2
240 4
260 6
280 6
300 6
320 5
340 3
360 1
A-P:MET-S2
20 0
40 1
60 2
80 2
100 3
120 2
140 2
160 1
180 0
200 0
220 0
240 2
260 2
280 3
300 2
320 2
340 1
360 0
DA-M5:GLU-S1
20 0
40 0
60 0
80 0
100 0
120 0

140 0
160 0
180 0
200 0
220 0
240 0
260 0
280 0
300 0
320 0
340 0
360 0
U34-P:HIS-S1
20 0
40 0
60 0
80 0
100 0
120 0
140 0
160 0
180 0
200 0
220 0
240 0
260 0
280 0
300 0
320 0
340 0
360 0
U34-MY:VAL-CA
20 0
40 0
60 0
80 0
100 0
120 0
140 0
160 0
180 0
200 0
220 0
240 0
260 0
280 0
300 0
320 0
340 0
360 0
FHU-MY:ALA-S1
20 0
40 0
60 0
80 0
100 0
120 0
140 0

160 0
180 0
200 0
220 0
240 0
260 0
280 0
300 0
320 0
340 0
360 0
IU-P:LYS-S1
20 0
40 0
60 0
80 0
100 0
120 0
140 0
160 0
180 0
200 0
220 0
240 0
260 0
280 0
300 0
320 0
340 0
360 0
QUO-M6:ASN-S1
20 0
40 0
60 0
80 0
100 0
120 0
140 0
160 0
180 0
200 0
220 0
240 0
260 0
280 0
300 0
320 0
340 0
360 0
A-R5:ARG-S2
20 0
40 2
60 5
80 7
100 8
120 7
140 6
160 4

180 1
200 0
220 3
240 5
260 7
280 8
300 7
320 6
340 4
360 1
H2U-MY:GLU-S2
20 0
40 0
60 0
80 0
100 0
120 0
140 0
160 0
180 0
200 0
220 0
240 0
260 0
280 0
300 0
320 0
340 0
360 0
C-Y:LEU-S1
20 0
40 3
60 5
80 7
100 8
120 7
140 6
160 4
180 0
200 0
220 3
240 5
260 8
280 8
300 7
320 6
340 4
360 1
U34-P:HIS-CA
20 0
40 0
60 0
80 0
100 0
120 0
140 0
160 0
180 0

200 0
220 0
240 0
260 0
280 0
300 0
320 0
340 0
360 0
H2U-RIB:PRO-CA
20 0
40 0
60 0
80 0
100 0
120 0
140 0
160 0
180 0
200 0
220 0
240 0
260 0
280 0
300 0
320 0
340 0
360 0
C-RIB:ASP-S2
20 0
40 2
60 4
80 5
100 5
120 5
140 4
160 3
180 1
200 0
220 2
240 4
260 5
280 5
300 5
320 4
340 3
360 1
4SU-P:THR-S1
20 0
40 0
60 0
80 0
100 0
120 0
140 0
160 0
180 0
200 0

220 0
240 0
260 0
280 0
300 0
320 0
340 0
360 0
FMU-RIB:CYS-S1
20 0
40 0
60 0
80 0
100 0
120 0
140 0
160 0
180 0
200 0
220 0
240 0
260 0
280 0
300 0
320 0
340 0
360 0
FHU-RIB:ARG-S1
20 0
40 0
60 0
80 0
100 0
120 0
140 0
160 0
180 0
200 0
220 0
240 0
260 0
280 0
300 0
320 0
340 0
360 0
FMU-MY:PHE-CA
20 0
40 0
60 0
80 0
100 0
120 0
140 0
160 0
180 0
200 0
220 0

240 0
260 0
280 0
300 0
320 0
340 0
360 0
C-P:TYR-S1
20 0
40 1
60 2
80 2
100 3
120 2
140 2
160 1
180 0
200 0
220 0
240 2
260 2
280 3
300 2
320 2
340 1
360 0
IU-MY:SER-S1
20 0
40 0
60 0
80 0
100 0
120 0
140 0
160 0
180 0
200 0
220 0
240 0
260 0
280 0
300 0
320 0
340 0
360 0
A-RIB:HIS-S2
20 0
40 0
60 2
80 2
100 2
120 2
140 2
160 1
180 0
200 0
220 1
240 2

260 2
280 2
300 2
320 2
340 1
360 0
IU-P:SER-S1
20 0
40 0
60 0
80 0
100 0
120 0
140 0
160 0
180 0
200 0
220 0
240 0
260 0
280 0
300 0
320 0
340 0
360 0
G-R6:GLY-CA
20 1
40 3
60 7
80 10
100 10
120 9
140 7
160 6
180 2
200 0
220 4
240 7
260 10
280 10
300 10
320 8
340 6
360 2
C-Y:GLN-S1
20 0
40 1
60 2
80 3
100 3
120 3
140 2
160 1
180 0
200 0
220 1
240 2
260 3

280 3
300 0
320 2
340 2
360 0
C-Y:LYS-S1
20 0
40 2
60 4
80 6
100 6
120 6
140 5
160 3
180 1
200 0
220 2
240 4
260 6
280 6
300 6
320 5
340 3
360 1
FMU-MY:PRO-CA
20 0
40 0
60 0
80 0
100 0
120 0
140 0
160 0
180 0
200 0
220 0
240 0
260 0
280 0
300 0
320 0
340 0
360 0
A-R5:ARG-CA
20 0
40 2
60 5
80 7
100 8
120 7
140 6
160 4
180 0
200 0
220 3
240 5
260 7
280 8

300   7
320   6
340   4
360   1
C-P:MET-S1
20   0
40   0
60   1
80   2
100   2
120   2
140   1
160   1
180   0
200   0
220   0
240   1
260   2
280   2
300   2
320   1
340   1
360   0
G-RIB:ASN-S1
20   0
40   2
60   4
80   5
100   6
120   5
140   4
160   3
180   0
200   0
220   2
240   4
260   5
280   6
300   5
320   4
340   3
360   1
U-Y:THR-CA
20   0
40   1
60   2
80   3
100   3
120   3
140   2
160   0
180   0
200   0
220   0
240   2
260   3
280   3
300   3

320   2
340   1
360   0
U-P:ASN-S2
20   0
40   0
60   1
80   2
100   2
120   2
140   1
160   1
180   0
200   0
220   1
240   1
260   2
280   2
300   2
320   2
340   1
360   0
FMU-P:ALA-CA
20   0
40   0
60   0
80   0
100   0
120   0
140   0
160   0
180   0
200   0
220   0
240   0
260   0
280   0
300   0
320   0
340   0
360   0
G-RIB:ASN-CA
20   0
40   2
60   4
80   5
100   6
120   5
140   4
160   3
180   1
200   0
220   2
240   4
260   5
280   6
300   5
320   4

340 3
360 1
C-P:VAL-CA
20 0
40 2
60 5
80 6
100 7
120 6
140 5
160 3
180 1
200 0
220 2
240 4
260 6
280 7
300 6
320 5
340 4
360 1
A-R6:CYS-S1
20 0
40 0
60 0
80 1
100 0
120 0
140 0
160 0
180 0
200 0
220 0
240 0
260 1
280 1
300 0
320 1
340 0
360 0
C-Y:ASN-CA
20 0
40 1
60 2
80 3
100 3
120 3
140 2
160 0
180 0
200 0
220 0
240 2
260 3
280 3
300 3
320 3
340 2

360 0
FHU-RIB:PRO-CA
20 0
40 0
60 0
80 0
100 0
120 0
140 0
160 0
180 0
200 0
220 0
240 0
260 0
280 0
300 0
320 0
340 0
360 0
G-P:MET-S2
20 0
40 0
60 2
80 3
100 3
120 3
140 2
160 2
180 0
200 0
220 1
240 2
260 3
280 3
300 3
320 2
340 0
360 0
U-P:TRP-S2
20 0
40 0
60 0
80 0
100 0
120 0
140 0
160 0
180 0
200 0
220 0
240 0
260 0
280 0
300 0
320 0
340 0
360 0

C-P:LEU-S2
20 0
40 3
60 5
80 7
100 8
120 7
140 6
160 4
180 0
200 0
220 3
240 5
260 8
280 8
300 7
320 6
340 4
360 1
G-RIB:VAL-S1
20 0
40 4
60 7
80 10
100 11
120 10
140 8
160 6
180 2
200 0
220 4
240 7
260 10
280 11
300 10
320 8
340 6
360 2
DA-M5:VAL-S1
20 0
40 0
60 0
80 0
100 0
120 0
140 0
160 0
180 0
200 0
220 0
240 0
260 0
280 0
300 0
320 0
340 0
360 0
DA-M6:GLU-S2

20 0
40 0
60 0
80 0
100 0
120 0
140 0
160 0
180 0
200 0
220 0
240 0
260 0
280 0
300 0
320 0
340 0
360 0
A-R6:HIS-S2
20 0
40 1
60 2
80 2
100 2
120 2
140 2
160 1
180 0
200 0
220 1
240 2
260 2
280 2
300 2
320 2
340 1
360 0
C-Y:ILE-S1
20 0
40 1
60 3
80 5
100 5
120 4
140 3
160 0
180 0
200 0
220 2
240 3
260 5
280 5
300 5
320 4
340 0
360 0
G-P:ASP-CA
20 0

40 0
60 0
80 8
100 9
120 8
140 6
160 5
180 2
200 0
220 3
240 6
260 8
280 9
300 8
320 7
340 5
360 2
GTP-M6:ASN-S1
20 0
40 0
60 0
80 0
100 0
120 0
140 0
160 0
180 0
200 0
220 0
240 0
260 0
280 0
300 0
320 0
340 0
360 0
G-RIB:ILE-CA
20 0
40 3
60 0
80 7
100 8
120 7
140 6
160 4
180 0
200 0
220 3
240 0
260 7
280 8
300 7
320 6
340 4
360 0
G-R6:ARG-S1
20 0
40 3

60   7
80   9
100   10
120   9
140   7
160   5
180   0
200   0
220   3
240   7
260   9
280   10
300   9
320   8
340   5
360   2
C-RIB:ARG-CA
20   0
40   2
60   4
80   6
100   6
120   5
140   4
160   3
180   1
200   0
220   2
240   4
260   6
280   6
300   6
320   5
340   3
360   1
U-P:CYS-S1
20   0
40   0
60   0
80   0
100   0
120   0
140   0
160   0
180   0
200   0
220   0
240   0
260   0
280   0
300   0
320   0
340   0
360   0
U31-MY:GLU-S1
20   0
40   0
60   0

80 0
100 0
120 0
140 0
160 0
180 0
200 0
220 0
240 0
260 0
280 0
300 0
320 0
340 0
360 0
G-R5:LYS-S1
20 1
40 3
60 7
80 9
100 10
120 9
140 7
160 5
180 2
200 0
220 4
240 7
260 10
280 10
300 9
320 8
340 6
360 0
IU-P:ARG-S2
20 0
40 0
60 0
80 0
100 0
120 0
140 0
160 0
180 0
200 0
220 0
240 0
260 0
280 0
300 0
320 0
340 0
360 0
G-R5:ILE-S1
20 0
40 3
60 5
80 7

100 8
120 7
140 6
160 0
180 0
200 0
220 3
240 5
260 7
280 8
300 7
320 0
340 0
360 0
A-RIB:TYR-CA
20 0
40 0
60 2
80 3
100 4
120 3
140 2
160 2
180 0
200 0
220 1
240 2
260 3
280 4
300 3
320 3
340 2
360 0
U31-RIB:ASN-CA
20 0
40 0
60 0
80 0
100 0
120 0
140 0
160 0
180 0
200 0
220 0
240 0
260 0
280 0
300 0
320 0
340 0
360 0
H2U-RIB:GLY-CA
20 0
40 0
60 0
80 0
100 0

120 0
140 0
160 0
180 0
200 0
220 0
240 0
260 0
280 0
300 0
320 0
340 0
360 0
A-P:LEU-S2
20 0
40 3
60 7
80 9
100 10
120 9
140 7
160 5
180 0
200 0
220 4
240 7
260 10
280 10
300 9
320 8
340 6
360 2
U31-MY:ASP-S2
20 0
40 0
60 0
80 0
100 0
120 0
140 0
160 0
180 0
200 0
220 0
240 0
260 0
280 0
300 0
320 0
340 0
360 0
A-RIB:ARG-CA
20 0
40 2
60 5
80 7
100 8
120 7

140   6
160   4
180   1
200   0
220   3
240   5
260   7
280   8
300   7
320   6
340   4
360   1
U34-P:TYR-CA
20   0
40   0
60   0
80   0
100   0
120   0
140   0
160   0
180   0
200   0
220   0
240   0
260   0
280   0
300   0
320   0
340   0
360   0
A-R5:SER-CA
20   0
40   2
60   4
80   6
100   6
120   6
140   4
160   3
180   1
200   0
220   2
240   4
260   6
280   6
300   6
320   5
340   3
360   1
A-P:ASN-S1
20   0
40   1
60   3
80   4
100   4
120   4
140   3

160 2
180 1
200 0
220 1
240 3
260 4
280 4
300 4
320 3
340 2
360 1
DA-M6:ASN-S1
20 0
40 0
60 0
80 0
100 0
120 0
140 0
160 0
180 0
200 0
220 0
240 0
260 0
280 0
300 0
320 0
340 0
360 0
C-Y:VAL-S1
20 0
40 2
60 5
80 6
100 7
120 6
140 5
160 0
180 0
200 0
220 2
240 4
260 6
280 7
300 6
320 5
340 0
360 0
C-RIB:TYR-S1
20 0
40 0
60 2
80 2
100 3
120 2
140 2
160 1

180 0
200 0
220 1
240 0
260 2
280 3
300 2
320 2
340 1
360 0
5BU-MY:PRO-S1
20 0
40 0
60 0
80 0
100 0
120 0
140 0
160 0
180 0
200 0
220 0
240 0
260 0
280 0
300 0
320 0
340 0
360 0
U-Y:PRO-S1
20 0
40 1
60 2
80 2
100 3
120 2
140 2
160 1
180 0
200 0
220 1
240 2
260 2
280 3
300 2
320 0
340 1
360 0
G-R5:ARG-CA
20 1
40 3
60 7
80 9
100 10
120 9
140 7
160 5
180 2

200 0
220 3
240 7
260 9
280 10
300 9
320 8
340 5
360 2
FHU-P:ARG-S1
20 0
40 0
60 0
80 0
100 0
120 0
140 0
160 0
180 0
200 0
220 0
240 0
260 0
280 0
300 0
320 0
340 0
360 0
U-P:ILE-S1
20 0
40 0
60 2
80 3
100 3
120 3
140 2
160 2
180 0
200 0
220 1
240 0
260 3
280 3
300 3
320 0
340 2
360 0
U-Y:LYS-CA
20 0
40 1
60 3
80 4
100 4
120 4
140 3
160 2
180 1
200 0

220   1
240   3
260   4
280   4
300   4
320   3
340   2
360   0
H2U-MY:THR-CA
20   0
40   0
60   0
80   0
100   0
120   0
140   0
160   0
180   0
200   0
220   0
240   0
260   0
280   0
300   0
320   0
340   0
360   0
G-R5:ARG-S1
20   1
40   3
60   7
80   9
100   10
120   9
140   7
160   5
180   2
200   0
220   3
240   7
260   9
280   10
300   9
320   8
340   5
360   2
C31-P:SER-S1
20   0
40   0
60   0
80   0
100   0
120   0
140   0
160   0
180   0
200   0
220   0

240 0
260 0
280 0
300 0
320 0
340 0
360 0
G-P:ARG-S2
20 0
40 3
60 7
80 9
100 10
120 9
140 7
160 5
180 2
200 0
220 3
240 7
260 9
280 10
300 9
320 8
340 5
360 2
C-Y:ASN-S2
20 0
40 1
60 2
80 3
100 3
120 3
140 2
160 2
180 0
200 0
220 1
240 2
260 3
280 3
300 3
320 3
340 2
360 0
G-R6:ASP-S2
20 0
40 3
60 6
80 8
100 9
120 8
140 6
160 5
180 2
200 0
220 3
240 6

260   8
280   9
300   8
320   7
340   5
360   2
U-Y:ASN-S1
20   0
40   0
60   1
80   2
100   2
120   2
140   1
160   1
180   0
200   0
220   1
240   1
260   2
280   2
300   2
320   2
340   1
360   0
FMU-MY:VAL-S1
20   0
40   0
60   0
80   0
100   0
120   0
140   0
160   0
180   0
200   0
220   0
240   0
260   0
280   0
300   0
320   0
340   0
360   0
A-R5:VAL-CA
20   0
40   3
60   6
80   8
100   9
120   8
140   6
160   4
180   0
200   0
220   3
240   6
260   8

280 9
300 8
320 7
340 5
360 0
G-P:ARG-S1
20 0
40 3
60 7
80 9
100 10
120 9
140 7
160 5
180 2
200 0
220 3
240 7
260 9
280 10
300 9
320 8
340 5
360 2
C-RIB:PHE-S1
20 0
40 1
60 2
80 3
100 3
120 3
140 2
160 2
180 0
200 0
220 0
240 0
260 3
280 3
300 3
320 2
340 2
360 0
U-RIB:PRO-S1
20 0
40 0
60 2
80 2
100 3
120 2
140 2
160 1
180 0
200 0
220 1
240 2
260 2
280 3

300 2
320 2
340 1
360 0
U-Y:MET-S1
20 0
40 0
60 1
80 0
100 1
120 0
140 0
160 0
180 0
200 0
220 0
240 1
260 1
280 1
300 0
320 1
340 0
360 0
FMU-RIB:ARG-S1
20 0
40 0
60 0
80 0
100 0
120 0
140 0
160 0
180 0
200 0
220 0
240 0
260 0
280 0
300 0
320 0
340 0
360 0
C31-P:GLN-S2
20 0
40 0
60 0
80 0
100 0
120 0
140 0
160 0
180 0
200 0
220 0
240 0
260 0
280 0
300 0

320 0
340 0
360 0
A-R6:PHE-CA
20 0
40 1
60 3
80 4
100 4
120 4
140 3
160 0
180 0
200 0
220 0
240 3
260 4
280 4
300 4
320 0
340 0
360 0
DA-M6:LEU-S2
20 0
40 0
60 0
80 0
100 0
120 0
140 0
160 0
180 0
200 0
220 0
240 0
260 0
280 0
300 0
320 0
340 0
360 0
GTP-M6:SER-S1
20 0
40 0
60 0
80 0
100 0
120 0
140 0
160 0
180 0
200 0
220 0
240 0
260 0
280 0
300 0
320 0

340 0
360 0
U-RIB:ALA-CA
20 0
40 2
60 3
80 5
100 5
120 5
140 4
160 3
180 1
200 0
220 2
240 3
260 5
280 5
300 5
320 4
340 3
360 1
U-Y:GLU-S2
20 0
40 1
60 3
80 5
100 5
120 4
140 3
160 2
180 1
200 0
220 0
240 3
260 5
280 5
300 4
320 0
340 3
360 0
C31-P:PHE-S1
20 0
40 0
60 0
80 0
100 0
120 0
140 0
160 0
180 0
200 0
220 0
240 0
260 0
280 0
300 0
320 0
340 0

360   0
G-RIB:LEU-S2
20   0
40   0
60   9
80   12
100   13
120   12
140   9
160   7
180   2
200   1
220   5
240   9
260   12
280   13
300   12
320   10
340   7
360   3
QUO-M5:ASN-S1
20   0
40   0
60   0
80   0
100   0
120   0
140   0
160   0
180   0
200   0
220   0
240   0
260   0
280   0
300   0
320   0
340   0
360   0
A-P:ASN-S2
20   0
40   1
60   3
80   4
100   4
120   4
140   3
160   2
180   1
200   0
220   0
240   3
260   4
280   4
300   4
320   3
340   2
360   0

C-P:ASN-CA
20 0
40 1
60 2
80 3
100 3
120 3
140 2
160 2
180 0
200 0
220 1
240 2
260 3
280 3
300 3
320 3
340 2
360 0
A-R6:MET-S1
20 0
40 0
60 2
80 2
100 3
120 2
140 2
160 1
180 0
200 0
220 0
240 2
260 0
280 3
300 2
320 2
340 1
360 0
C-P:CYS-CA
20 0
40 0
60 0
80 0
100 0
120 0
140 0
160 0
180 0
200 0
220 0
240 0
260 1
280 1
300 0
320 0
340 0
360 0
H2U-RIB:TRP-S2

20 0
40 0
60 0
80 0
100 0
120 0
140 0
160 0
180 0
200 0
220 0
240 0
260 0
280 0
300 0
320 0
340 0
360 0
U34-P:GLU-S1
20 0
40 0
60 0
80 0
100 0
120 0
140 0
160 0
180 0
200 0
220 0
240 0
260 0
280 0
300 0
320 0
340 0
360 0
DA-M5:THR-CA
20 0
40 0
60 0
80 0
100 0
120 0
140 0
160 0
180 0
200 0
220 0
240 0
260 0
280 0
300 0
320 0
340 0
360 0
C-RIB:TRP-CA
20 0

40 0
60 0
80 1
100 1
120 1
140 0
160 0
180 0
200 0
220 0
240 0
260 0
280 1
300 0
320 0
340 0
360 0
C-Y:TRP-S1
20 0
40 0
60 0
80 1
100 1
120 1
140 0
160 0
180 0
200 0
220 0
240 0
260 1
280 1
300 0
320 1
340 0
360 0
IU-MY:ARG-CA
20 0
40 0
60 0
80 0
100 0
120 0
140 0
160 0
180 0
200 0
220 0
240 0
260 0
280 0
300 0
320 0
340 0
360 0
A-P:TRP-S2
20 0
40 0

60 1
80 1
100 1
120 0
140 1
160 0
180 0
200 0
220 0
240 1
260 0
280 1
300 1
320 1
340 0
360 0
C-RIB:ASN-CA
20 0
40 1
60 2
80 3
100 3
120 3
140 2
160 2
180 0
200 0
220 1
240 2
260 3
280 3
300 3
320 3
340 2
360 0
A-P:PHE-S1
20 0
40 0
60 3
80 4
100 4
120 4
140 3
160 2
180 0
200 0
220 1
240 3
260 4
280 4
300 4
320 3
340 2
360 1
H2U-P:TRP-S2
20 0
40 0
60 0

80 0
100 0
120 0
140 0
160 0
180 0
200 0
220 0
240 0
260 0
280 0
300 0
320 0
340 0
360 0
G-RIB:GLU-S1
20 0
40 0
60 8
80 11
100 12
120 11
140 9
160 6
180 2
200 1
220 4
240 8
260 11
280 12
300 11
320 9
340 7
360 2
A-R5:GLN-S2
20 0
40 1
60 3
80 4
100 4
120 3
140 3
160 2
180 0
200 0
220 1
240 3
260 4
280 4
300 4
320 3
340 2
360 0
C31-RIB:LEU-CA
20 0
40 0
60 0
80 0

100 0
120 0
140 0
160 0
180 0
200 0
220 0
240 0
260 0
280 0
300 0
320 0
340 0
360 0
C-Y:SER-CA
20 0
40 1
60 3
80 5
100 5
120 4
140 3
160 2
180 1
200 0
220 2
240 3
260 5
280 5
300 4
320 4
340 3
360 0
A-R6:CYS-CA
20 0
40 0
60 0
80 1
100 0
120 0
140 0
160 0
180 0
200 0
220 0
240 0
260 0
280 1
300 1
320 0
340 0
360 0
A-R6:PRO-CA
20 0
40 0
60 3
80 5
100 5

120 5
140 4
160 3
180 1
200 0
220 2
240 3
260 5
280 5
300 5
320 4
340 3
360 1
DA-M6:HIS-S2
20 0
40 0
60 0
80 0
100 0
120 0
140 0
160 0
180 0
200 0
220 0
240 0
260 0
280 0
300 0
320 0
340 0
360 0
A-P:LEU-CA
20 0
40 3
60 7
80 9
100 10
120 9
140 7
160 5
180 0
200 0
220 4
240 7
260 10
280 10
300 9
320 8
340 6
360 2
FMU-MY:ARG-S2
20 0
40 0
60 0
80 0
100 0
120 0

140 0
160 0
180 0
200 0
220 0
240 0
260 0
280 0
300 0
320 0
340 0
360 0
U31-MY:TYR-S1
20 0
40 0
60 0
80 0
100 0
120 0
140 0
160 0
180 0
200 0
220 0
240 0
260 0
280 0
300 0
320 0
340 0
360 0
DA-M6:ASN-S2
20 0
40 0
60 0
80 0
100 0
120 0
140 0
160 0
180 0
200 0
220 0
240 0
260 0
280 0
300 0
320 0
340 0
360 0
IU-MY:ARG-S2
20 0
40 0
60 0
80 0
100 0
120 0
140 0

160 0
180 0
200 0
220 0
240 0
260 0
280 0
300 0
320 0
340 0
360 0
U31-P:ASP-S1
20 0
40 0
60 0
80 0
100 0
120 0
140 0
160 0
180 0
200 0
220 0
240 0
260 0
280 0
300 0
320 0
340 0
360 0
U-Y:HIS-S2
20 0
40 0
60 1
80 1
100 1
120 1
140 1
160 0
180 0
200 0
220 0
240 1
260 1
280 1
300 1
320 1
340 0
360 0
A-R5:HIS-S2
20 0
40 1
60 2
80 2
100 2
120 2
140 2
160 1

180 0
200 0
220 1
240 2
260 2
280 2
300 2
320 2
340 1
360 0
GTP-M5:GLY-CA
20 0
40 0
60 0
80 0
100 0
120 0
140 0
160 0
180 0
200 0
220 0
240 0
260 0
280 0
300 0
320 0
340 0
360 0
C-P:ALA-S1
20 0
40 2
60 5
80 7
100 8
120 7
140 5
160 4
180 1
200 0
220 3
240 5
260 7
280 8
300 7
320 6
340 4
360 1
U34-P:ASN-S2
20 0
40 0
60 0
80 0
100 0
120 0
140 0
160 0
180 0

200 0
220 0
240 0
260 0
280 0
300 0
320 0
340 0
360 0
QUO-M5:LYS-S1
20 0
40 0
60 0
80 0
100 0
120 0
140 0
160 0
180 0
200 0
220 0
240 0
260 0
280 0
300 0
320 0
340 0
360 0
IU-P:LYS-CA
20 0
40 0
60 0
80 0
100 0
120 0
140 0
160 0
180 0
200 0
220 0
240 0
260 0
280 0
300 0
320 0
340 0
360 0
QUO-M5:LYS-S2
20 0
40 0
60 0
80 0
100 0
120 0
140 0
160 0
180 0
200 0

220 0
240 0
260 0
280 0
300 0
320 0
340 0
360 0
C31-MY:GLN-S2
20 0
40 0
60 0
80 0
100 0
120 0
140 0
160 0
180 0
200 0
220 0
240 0
260 0
280 0
300 0
320 0
340 0
360 0
A-P:LYS-S1
20 0
40 3
60 5
80 7
100 8
120 7
140 6
160 4
180 1
200 0
220 0
240 5
260 8
280 8
300 7
320 6
340 4
360 1
G-P:TYR-CA
20 0
40 0
60 3
80 4
100 4
120 4
140 3
160 2
180 1
200 0
220 0

240 3
260 4
280 4
300 4
320 3
340 2
360 0
U-RIB:LEU-CA
20 0
40 0
60 4
80 0
100 5
120 5
140 4
160 3
180 1
200 0
220 0
240 4
260 5
280 5
300 5
320 4
340 3
360 0
U34-P:TYR-S2
20 0
40 0
60 0
80 0
100 0
120 0
140 0
160 0
180 0
200 0
220 0
240 0
260 0
280 0
300 0
320 0
340 0
360 0
G-RIB:MET-S2
20 0
40 1
60 2
80 3
100 3
120 3
140 2
160 2
180 0
200 0
220 0
240 2

260   3
280   3
300   3
320   3
340   2
360   0
G-R5:GLN-CA
20   0
40   1
60   3
80   5
100   0
120   0
140   4
160   0
180   0
200   0
220   2
240   3
260   5
280   5
300   5
320   4
340   0
360   0
H2U-RIB:GLU-S2
20   0
40   0
60   0
80   0
100   0
120   0
140   0
160   0
180   0
200   0
220   0
240   0
260   0
280   0
300   0
320   0
340   0
360   0
A-R5:PHE-S2
20   0
40   1
60   0
80   4
100   4
120   4
140   0
160   0
180   1
200   0
220   1
240   0
260   4

280 4
300 4
320 0
340 0
360 1
G-P:LEU-CA
20 0
40 0
60 9
80 12
100 13
120 12
140 9
160 7
180 2
200 0
220 0
240 9
260 12
280 13
300 12
320 10
340 7
360 3
C-P:ASP-CA
20 0
40 0
60 4
80 5
100 6
120 5
140 4
160 3
180 0
200 0
220 2
240 4
260 5
280 6
300 5
320 4
340 3
360 0
H2U-MY:LEU-S1
20 0
40 0
60 0
80 0
100 0
120 0
140 0
160 0
180 0
200 0
220 0
240 0
260 0
280 0

300   0
320   0
340   0
360   0
C-P:MET-CA
20   0
40   0
60   1
80   2
100   2
120   2
140   1
160   0
180   0
200   0
220   0
240   1
260   2
280   2
300   2
320   1
340   1
360   0
FHU-MY:ILE-S1
20   0
40   0
60   0
80   0
100   0
120   0
140   0
160   0
180   0
200   0
220   0
240   0
260   0
280   0
300   0
320   0
340   0
360   0
C-RIB:MET-CA
20   0
40   0
60   0
80   2
100   2
120   2
140   1
160   0
180   0
200   0
220   0
240   1
260   2
280   2
300   2

320 1
340 0
360 0
H2U-RIB:PRO-S1
20 0
40 0
60 0
80 0
100 0
120 0
140 0
160 0
180 0
200 0
220 0
240 0
260 0
280 0
300 0
320 0
340 0
360 0
H2U-P:PRO-CA
20 0
40 0
60 0
80 0
100 0
120 0
140 0
160 0
180 0
200 0
220 0
240 0
260 0
280 0
300 0
320 0
340 0
360 0
A-R5:ASP-S1
20 0
40 2
60 5
80 6
100 0
120 6
140 5
160 4
180 0
200 0
220 2
240 5
260 7
280 7
300 6
320 5

340 4
360 1
C31-RIB:THR-CA
20 0
40 0
60 0
80 0
100 0
120 0
140 0
160 0
180 0
200 0
220 0
240 0
260 0
280 0
300 0
320 0
340 0
360 0
G-RIB:LEU-CA
20 0
40 4
60 9
80 12
100 13
120 12
140 9
160 7
180 2
200 0
220 5
240 9
260 12
280 13
300 12
320 10
340 7
360 3
FMU-RIB:ARG-S2
20 0
40 0
60 0
80 0
100 0
120 0
140 0
160 0
180 0
200 0
220 0
240 0
260 0
280 0
300 0
320 0
340 0

360 0
G-P:GLN-S2
20 0
40 1
60 3
80 5
100 5
120 4
140 3
160 2
180 1
200 0
220 2
240 3
260 5
280 5
300 4
320 4
340 3
360 1
U-P:SER-S1
20 0
40 1
60 2
80 3
100 3
120 3
140 2
160 2
180 0
200 0
220 1
240 2
260 3
280 3
300 3
320 2
340 2
360 0
U31-P:MET-S1
20 0
40 0
60 0
80 0
100 0
120 0
140 0
160 0
180 0
200 0
220 0
240 0
260 0
280 0
300 0
320 0
340 0
360 0

G-RIB:PHE-CA
20 0
40 2
60 4
80 5
100 5
120 5
140 4
160 3
180 0
200 0
220 0
240 4
260 5
280 5
300 5
320 4
340 3
360 1
QUO-RIB:LYS-S2
20 0
40 0
60 0
80 0
100 0
120 0
140 0
160 0
180 0
200 0
220 0
240 0
260 0
280 0
300 0
320 0
340 0
360 0
G-R5:ASN-S2
20 0
40 2
60 4
80 5
100 6
120 5
140 4
160 3
180 1
200 0
220 2
240 4
260 5
280 6
300 5
320 4
340 0
360 1
G-R5:LYS-CA

20   1
40   3
60   7
80   9
100   10
120   9
140   7
160   5
180   2
200   0
220   4
240   7
260   10
280   10
300   9
320   8
340   6
360   2
G-R5:VAL-S1
20   0
40   4
60   7
80   10
100   11
120   10
140   8
160   0
180   2
200   1
220   4
240   7
260   10
280   11
300   10
320   8
340   0
360   0
A-R5:GLN-CA
20   0
40   0
60   3
80   4
100   4
120   3
140   3
160   2
180   0
200   0
220   1
240   3
260   4
280   4
300   4
320   3
340   0
360   0
A-P:TYR-S1
20   0

40   0
60   2
80   3
100   4
120   3
140   2
160   2
180   0
200   0
220   0
240   2
260   3
280   4
300   3
320   3
340   2
360   0
C-P:HIS-CA
20   0
40   0
60   1
80   2
100   2
120   2
140   1
160   1
180   0
200   0
220   0
240   1
260   2
280   2
300   2
320   1
340   1
360   0
A-R5:SER-S1
20   0
40   2
60   4
80   6
100   6
120   6
140   4
160   3
180   1
200   0
220   2
240   4
260   6
280   6
300   6
320   5
340   3
360   0
QUO-M5:PHE-S1
20   0
40   0

60 0
80 0
100 0
120 0
140 0
160 0
180 0
200 0
220 0
240 0
260 0
280 0
300 0
320 0
340 0
360 0
H2U-MY:ARG-S2
20 0
40 0
60 0
80 0
100 0
120 0
140 0
160 0
180 0
200 0
220 0
240 0
260 0
280 0
300 0
320 0
340 0
360 0
G-R6:GLN-CA
20 0
40 1
60 3
80 5
100 5
120 4
140 0
160 3
180 0
200 0
220 2
240 3
260 5
280 5
300 5
320 0
340 0
360 0
H2U-MY:PHE-S2
20 0
40 0
60 0

80 0
100 0
120 0
140 0
160 0
180 0
200 0
220 0
240 0
260 0
280 0
300 0
320 0
340 0
360 0
A-P:ALA-S1
20 0
40 3
60 7
80 9
100 10
120 9
140 7
160 5
180 2
200 0
220 0
240 7
260 9
280 10
300 9
320 7
340 5
360 2
G-R6:ASP-CA
20 0
40 3
60 6
80 8
100 9
120 8
140 6
160 5
180 2
200 0
220 3
240 6
260 8
280 9
300 8
320 7
340 5
360 2
A-R6:ASN-S2
20 0
40 1
60 3
80 4

100 4
120 4
140 3
160 2
180 1
200 0
220 1
240 3
260 4
280 4
300 4
320 3
340 2
360 1
U-P:TYR-S1
20 0
40 0
60 1
80 2
100 2
120 1
140 1
160 0
180 0
200 0
220 0
240 0
260 2
280 2
300 2
320 0
340 0
360 0
GTP-M5:ASP-S2
20 0
40 0
60 0
80 0
100 0
120 0
140 0
160 0
180 0
200 0
220 0
240 0
260 0
280 0
300 0
320 0
340 0
360 0
G-R6:GLU-S2
20 1
40 4
60 8
80 11
100 12

120   11
140   8
160   6
180   2
200   0
220   4
240   8
260   11
280   12
300   11
320   9
340   7
360   2
QUO-M6:PHE-S2
20   0
40   0
60   0
80   0
100   0
120   0
140   0
160   0
180   0
200   0
220   0
240   0
260   0
280   0
300   0
320   0
340   0
360   0
C-Y:GLU-S1
20   0
40   2
60   5
80   7
100   7
120   0
140   5
160   4
180   1
200   0
220   3
240   5
260   7
280   7
300   0
320   6
340   4
360   0
QUO-M6:LEU-S1
20   0
40   0
60   0
80   0
100   0
120   0

140 0
160 0
180 0
200 0
220 0
240 0
260 0
280 0
300 0
320 0
340 0
360 0
FHU-P:LEU-CA
20 0
40 0
60 0
80 0
100 0
120 0
140 0
160 0
180 0
200 0
220 0
240 0
260 0
280 0
300 0
320 0
340 0
360 0
FMU-RIB:ALA-S1
20 0
40 0
60 0
80 0
100 0
120 0
140 0
160 0
180 0
200 0
220 0
240 0
260 0
280 0
300 0
320 0
340 0
360 0
U-Y:GLN-S2
20 0
40 0
60 1
80 2
100 2
120 2
140 1

160   1
180   0
200   0
220   0
240   1
260   2
280   2
300   2
320   1
340   1
360   0
A-R6:LEU-CA
20   0
40   3
60   7
80   10
100   10
120   9
140   7
160   0
180   2
200   0
220   4
240   0
260   10
280   10
300   9
320   8
340   6
360   0
A-RIB:PHE-CA
20   0
40   0
60   3
80   4
100   4
120   4
140   3
160   2
180   1
200   0
220   0
240   3
260   4
280   4
300   4
320   3
340   2
360   1
A-P:CYS-CA
20   0
40   0
60   0
80   1
100   1
120   1
140   0
160   0

180 0
200 0
220 0
240 0
260 1
280 1
300 0
320 0
340 0
360 0
FMU-MY:PHE-S2
20 0
40 0
60 0
80 0
100 0
120 0
140 0
160 0
180 0
200 0
220 0
240 0
260 0
280 0
300 0
320 0
340 0
360 0
H2U-RIB:ASN-S2
20 0
40 0
60 0
80 0
100 0
120 0
140 0
160 0
180 0
200 0
220 0
240 0
260 0
280 0
300 0
320 0
340 0
360 0
A-R6:ARG-CA
20 0
40 0
60 5
80 7
100 8
120 7
140 6
160 4
180 0

200 0
220 3
240 5
260 7
280 8
300 7
320 6
340 4
360 0
C-Y:ARG-S1
20 0
40 2
60 4
80 6
100 6
120 5
140 4
160 3
180 1
200 0
220 2
240 4
260 6
280 6
300 6
320 5
340 3
360 1
U34-MY:ASN-S1
20 0
40 0
60 0
80 0
100 0
120 0
140 0
160 0
180 0
200 0
220 0
240 0
260 0
280 0
300 0
320 0
340 0
360 0
G-R6:MET-S2
20 0
40 1
60 2
80 3
100 3
120 3
140 0
160 0
180 0
200 0

220 0
240 2
260 3
280 3
300 3
320 3
340 2
360 0
G-P:GLY-CA
20 1
40 3
60 7
80 9
100 10
120 9
140 7
160 5
180 2
200 0
220 4
240 7
260 10
280 10
300 9
320 8
340 6
360 2
A-R6:TRP-S2
20 0
40 0
60 1
80 1
100 1
120 1
140 0
160 0
180 0
200 0
220 0
240 1
260 1
280 0
300 1
320 1
340 0
360 0
IU-P:ARG-CA
20 0
40 0
60 0
80 0
100 0
120 0
140 0
160 0
180 0
200 0
220 0

240 0
260 0
280 0
300 0
320 0
340 0
360 0
QUO-M6:ARG-S1
20 0
40 0
60 0
80 0
100 0
120 0
140 0
160 0
180 0
200 0
220 0
240 0
260 0
280 0
300 0
320 0
340 0
360 0
C31-RIB:TYR-S2
20 0
40 0
60 0
80 0
100 0
120 0
140 0
160 0
180 0
200 0
220 0
240 0
260 0
280 0
300 0
320 0
340 0
360 0
U-Y:ASN-CA
20 0
40 0
60 1
80 2
100 2
120 2
140 1
160 1
180 0
200 0
220 1
240 1

260 2
280 2
300 2
320 2
340 1
360 0
FMU-MY:ASN-CA
20 0
40 0
60 0
80 0
100 0
120 0
140 0
160 0
180 0
200 0
220 0
240 0
260 0
280 0
300 0
320 0
340 0
360 0
C-RIB:GLN-S2
20 0
40 1
60 2
80 3
100 3
120 3
140 2
160 1
180 0
200 0
220 1
240 2
260 3
280 3
300 3
320 2
340 1
360 0
G-R5:CYS-CA
20 0
40 0
60 0
80 1
100 0
120 1
140 0
160 0
180 0
200 0
220 0
240 0
260 1

280 0
300 0
320 0
340 0
360 0
U-P:ASP-S2
20 0
40 0
60 2
80 3
100 4
120 3
140 2
160 2
180 0
200 0
220 1
240 2
260 3
280 4
300 3
320 3
340 2
360 0
IU-RIB:ARG-S2
20 0
40 0
60 0
80 0
100 0
120 0
140 0
160 0
180 0
200 0
220 0
240 0
260 0
280 0
300 0
320 0
340 0
360 0
FHU-RIB:LEU-S1
20 0
40 0
60 0
80 0
100 0
120 0
140 0
160 0
180 0
200 0
220 0
240 0
260 0
280 0

300 0
320 0
340 0
360 0
C-P:TRP-S2
20 0
40 0
60 0
80 1
100 1
120 1
140 0
160 0
180 0
200 0
220 0
240 0
260 1
280 0
300 1
320 0
340 0
360 0
U31-MY:GLN-CA
20 0
40 0
60 0
80 0
100 0
120 0
140 0
160 0
180 0
200 0
220 0
240 0
260 0
280 0
300 0
320 0
340 0
360 0
IU-RIB:LYS-CA
20 0
40 0
60 0
80 0
100 0
120 0
140 0
160 0
180 0
200 0
220 0
240 0
260 0
280 0
300 0

320 0
340 0
360 0
A-RIB:ARG-S1
20 0
40 2
60 5
80 7
100 8
120 7
140 6
160 4
180 1
200 0
220 3
240 5
260 7
280 8
300 7
320 6
340 4
360 1
A-R6:TYR-S1
20 0
40 0
60 2
80 3
100 4
120 3
140 2
160 2
180 0
200 0
220 0
240 2
260 3
280 4
300 3
320 3
340 2
360 0
G-P:VAL-CA
20 0
40 4
60 7
80 10
100 11
120 10
140 8
160 6
180 2
200 0
220 4
240 0
260 10
280 11
300 10
320 8

340 6
360 0
U31-P:GLN-S1
20 0
40 0
60 0
80 0
100 0
120 0
140 0
160 0
180 0
200 0
220 0
240 0
260 0
280 0
300 0
320 0
340 0
360 0
DA-M5:MET-S2
20 0
40 0
60 0
80 0
100 0
120 0
140 0
160 0
180 0
200 0
220 0
240 0
260 0
280 0
300 0
320 0
340 0
360 0
QUO-M6:LEU-S2
20 0
40 0
60 0
80 0
100 0
120 0
140 0
160 0
180 0
200 0
220 0
240 0
260 0
280 0
300 0
320 0
340 0

360   0
U-Y:SER-S1
20   0
40   1
60   2
80   3
100   3
120   3
140   2
160   2
180   0
200   0
220   1
240   2
260   3
280   3
300   3
320   2
340   2
360   0
U-P:THR-S1
20   0
40   1
60   2
80   3
100   3
120   3
140   2
160   1
180   0
200   0
220   1
240   2
260   3
280   3
300   3
320   2
340   1
360   0
C31-P:GLN-S1
20   0
40   0
60   0
80   0
100   0
120   0
140   0
160   0
180   0
200   0
220   0
240   0
260   0
280   0
300   0
320   0
340   0
360   0

C-Y:GLY-CA
20 0
40 2
60 4
80 6
100 6
120 6
140 5
160 3
180 1
200 0
220 2
240 4
260 6
280 6
300 6
320 5
340 0
360 1
U-RIB:ILE-CA
20 0
40 0
60 0
80 3
100 3
120 3
140 2
160 2
180 0
200 0
220 1
240 0
260 3
280 3
300 3
320 2
340 2
360 0
5BU-P:THR-CA
20 0
40 0
60 0
80 0
100 0
120 0
140 0
160 0
180 0
200 0
220 0
240 0
260 0
280 0
300 0
320 0
340 0
360 0
FHU-P:THR-CA

20 0
40 0
60 0
80 0
100 0
120 0
140 0
160 0
180 0
200 0
220 0
240 0
260 0
280 0
300 0
320 0
340 0
360 0
A-R5:HIS-S1
20 0
40 1
60 2
80 2
100 2
120 2
140 0
160 1
180 0
200 0
220 1
240 2
260 2
280 2
300 2
320 2
340 0
360 0
A-P:ARG-S1
20 0
40 2
60 5
80 7
100 8
120 7
140 6
160 4
180 1
200 0
220 3
240 5
260 7
280 8
300 7
320 6
340 4
360 1
C-Y:PRO-CA
20 0

40 1
60 3
80 4
100 4
120 4
140 3
160 2
180 0
200 0
220 1
240 3
260 4
280 4
300 4
320 3
340 2
360 0
U31-RIB:TYR-S1
20 0
40 0
60 0
80 0
100 0
120 0
140 0
160 0
180 0
200 0
220 0
240 0
260 0
280 0
300 0
320 0
340 0
360 0
FHU-P:TYR-CA
20 0
40 0
60 0
80 0
100 0
120 0
140 0
160 0
180 0
200 0
220 0
240 0
260 0
280 0
300 0
320 0
340 0
360 0
QUO-M6:LEU-CA
20 0
40 0

60 0
80 0
100 0
120 0
140 0
160 0
180 0
200 0
220 0
240 0
260 0
280 0
300 0
320 0
340 0
360 0
QUO-M6:ASP-S1
20 0
40 0
60 0
80 0
100 0
120 0
140 0
160 0
180 0
200 0
220 0
240 0
260 0
280 0
300 0
320 0
340 0
360 0
FMU-MY:ALA-S1
20 0
40 0
60 0
80 0
100 0
120 0
140 0
160 0
180 0
200 0
220 0
240 0
260 0
280 0
300 0
320 0
340 0
360 0
G-R5:SER-CA
20 0
40 3
60 5

80 7
100 8
120 7
140 6
160 4
180 1
200 0
220 3
240 5
260 7
280 8
300 7
320 6
340 4
360 0
A-RIB:LEU-S2
20 0
40 3
60 7
80 9
100 10
120 9
140 7
160 5
180 2
200 0
220 0
240 7
260 10
280 10
300 9
320 8
340 6
360 2
G-RIB:ARG-S1
20 1
40 3
60 7
80 9
100 10
120 9
140 7
160 5
180 2
200 0
220 3
240 7
260 9
280 10
300 9
320 8
340 5
360 2
U-Y:ALA-CA
20 0
40 2
60 3
80 5

100 5
120 5
140 4
160 3
180 0
200 0
220 2
240 3
260 5
280 5
300 5
320 4
340 3
360 0
U-Y:TYR-CA
20 0
40 0
60 1
80 2
100 2
120 0
140 1
160 0
180 0
200 0
220 0
240 1
260 2
280 2
300 2
320 1
340 1
360 0
OMC-P:LYS-S2
20 0
40 0
60 0
80 0
100 0
120 0
140 0
160 0
180 0
200 0
220 0
240 0
260 0
280 0
300 0
320 0
340 0
360 0
G-RIB:LYS-S2
20 1
40 3
60 7
80 9
100 10

120 9
140 7
160 5
180 2
200 0
220 4
240 7
260 9
280 10
300 9
320 8
340 6
360 2
G-R5:HIS-S2
20 0
40 1
60 2
80 3
100 3
120 3
140 2
160 2
180 0
200 0
220 1
240 2
260 3
280 3
300 3
320 0
340 2
360 0
C-RIB:ARG-S2
20 0
40 2
60 4
80 6
100 6
120 5
140 4
160 3
180 1
200 0
220 2
240 4
260 6
280 6
300 6
320 5
340 3
360 1
G-RIB:PRO-S1
20 0
40 2
60 4
80 6
100 7
120 6

140   5
160   3
180   1
200   0
220   2
240   4
260   6
280   7
300   6
320   5
340   4
360   1
FMU-RIB:GLN-S1
20   0
40   0
60   0
80   0
100   0
120   0
140   0
160   0
180   0
200   0
220   0
240   0
260   0
280   0
300   0
320   0
340   0
360   0
G-P:LEU-S2
20   0
40   4
60   9
80   12
100   13
120   11
140   9
160   7
180   2
200   0
220   5
240   9
260   12
280   13
300   12
320   10
340   7
360   3
A-P:GLU-S2
20   0
40   3
60   6
80   9
100   9
120   8
140   7

160 5
180 2
200 0
220 3
240 6
260 9
280 9
300 9
320 7
340 5
360 2
FHU-RIB:TYR-S1
20 0
40 0
60 0
80 0
100 0
120 0
140 0
160 0
180 0
200 0
220 0
240 0
260 0
280 0
300 0
320 0
340 0
360 0
IU-MY:PRO-CA
20 0
40 0
60 0
80 0
100 0
120 0
140 0
160 0
180 0
200 0
220 0
240 0
260 0
280 0
300 0
320 0
340 0
360 0
G-R6:GLN-S2
20 0
40 1
60 3
80 5
100 5
120 4
140 3
160 0

180 1
200 0
220 2
240 3
260 5
280 5
300 5
320 4
340 3
360 0
C-RIB:ILE-S1
20 0
40 1
60 3
80 5
100 5
120 4
140 3
160 3
180 0
200 0
220 0
240 3
260 5
280 5
300 5
320 4
340 3
360 0
C-P:ALA-CA
20 0
40 2
60 5
80 7
100 8
120 7
140 5
160 4
180 1
200 0
220 3
240 5
260 7
280 8
300 7
320 6
340 4
360 0
QUO-M5:ASN-S2
20 0
40 0
60 0
80 0
100 0
120 0
140 0
160 0
180 0

200 0
220 0
240 0
260 0
280 0
300 0
320 0
340 0
360 0
IU-RIB:PRO-CA
20 0
40 0
60 0
80 0
100 0
120 0
140 0
160 0
180 0
200 0
220 0
240 0
260 0
280 0
300 0
320 0
340 0
360 0
IU-MY:ILE-S1
20 0
40 0
60 0
80 0
100 0
120 0
140 0
160 0
180 0
200 0
220 0
240 0
260 0
280 0
300 0
320 0
340 0
360 0
C31-RIB:PHE-S2
20 0
40 0
60 0
80 0
100 0
120 0
140 0
160 0
180 0
200 0

220   0
240   0
260   0
280   0
300   0
320   0
340   0
360   0
G-R6:GLU-S1
20   0
40   4
60   8
80   11
100   12
120   11
140   9
160   6
180   2
200   0
220   4
240   8
260   11
280   12
300   11
320   9
340   7
360   2
U31-P:SER-CA
20   0
40   0
60   0
80   0
100   0
120   0
140   0
160   0
180   0
200   0
220   0
240   0
260   0
280   0
300   0
320   0
340   0
360   0
U31-RIB:GLU-S2
20   0
40   0
60   0
80   0
100   0
120   0
140   0
160   0
180   0
200   0
220   0

240   0
260   0
280   0
300   0
320   0
340   0
360   0
G-RIB:LYS-S1
20   1
40   3
60   7
80   9
100   10
120   9
140   7
160   5
180   2
200   0
220   4
240   7
260   10
280   10
300   9
320   8
340   6
360   2
FHU-P:ASP-CA
20   0
40   0
60   0
80   0
100   0
120   0
140   0
160   0
180   0
200   0
220   0
240   0
260   0
280   0
300   0
320   0
340   0
360   0
FMU-MY:SER-S1
20   0
40   0
60   0
80   0
100   0
120   0
140   0
160   0
180   0
200   0
220   0
240   0

260 0
280 0
300 0
320 0
340 0
360 0
A-P:SER-S1
20 0
40 2
60 4
80 6
100 6
120 6
140 4
160 3
180 1
200 0
220 2
240 4
260 6
280 6
300 6
320 5
340 3
360 1
DA-M6:ASN-CA
20 0
40 0
60 0
80 0
100 0
120 0
140 0
160 0
180 0
200 0
220 0
240 0
260 0
280 0
300 0
320 0
340 0
360 0
C31-MY:GLU-S1
20 0
40 0
60 0
80 0
100 0
120 0
140 0
160 0
180 0
200 0
220 0
240 0
260 0

280 0
300 0
320 0
340 0
360 0
DA-M6:SER-CA
20 0
40 0
60 0
80 0
100 0
120 0
140 0
160 0
180 0
200 0
220 0
240 0
260 0
280 0
300 0
320 0
340 0
360 0
H2U-MY:LYS-S2
20 0
40 0
60 0
80 0
100 0
120 0
140 0
160 0
180 0
200 0
220 0
240 0
260 0
280 0
300 0
320 0
340 0
360 0
A-R6:ILE-CA
20 0
40 2
60 4
80 6
100 0
120 6
140 4
160 3
180 1
200 0
220 0
240 4
260 6
280 6

300 6
320 5
340 3
360 1
U-RIB:LYS-S1
20 0
40 1
60 3
80 4
100 4
120 4
140 3
160 2
180 1
200 0
220 1
240 3
260 4
280 4
300 4
320 3
340 2
360 1
U-RIB:HIS-S1
20 0
40 0
60 1
80 1
100 1
120 1
140 1
160 0
180 0
200 0
220 0
240 0
260 1
280 1
300 1
320 1
340 0
360 0
C-Y:TYR-S1
20 0
40 0
60 2
80 2
100 3
120 2
140 2
160 0
180 0
200 0
220 0
240 2
260 2
280 3
300 2

320 2
340 1
360 0
G-RIB:LEU-S1
20 0
40 4
60 9
80 12
100 13
120 12
140 9
160 7
180 2
200 0
220 5
240 9
260 12
280 13
300 12
320 10
340 7
360 3
G-R6:MET-S1
20 0
40 1
60 0
80 3
100 3
120 3
140 0
160 0
180 0
200 0
220 1
240 2
260 3
280 3
300 3
320 3
340 2
360 0
A-R5:GLU-CA
20 1
40 3
60 0
80 9
100 9
120 8
140 7
160 5
180 2
200 0
220 3
240 6
260 9
280 9
300 9
320 7

340   0
360   2
U-RIB:TRP-CA
20   0
40   0
60   0
80   0
100   0
120   0
140   0
160   0
180   0
200   0
220   0
240   0
260   0
280   0
300   0
320   0
340   0
360   0
A-P:ARG-CA
20   0
40   2
60   5
80   7
100   8
120   7
140   6
160   4
180   1
200   0
220   3
240   5
260   7
280   8
300   7
320   6
340   4
360   1
U-P:GLU-S2
20   0
40   1
60   3
80   4
100   5
120   4
140   3
160   2
180   1
200   0
220   2
240   3
260   5
280   5
300   4
320   4
340   3

360 1
QUO-M6:GLU-CA
20 0
40 0
60 0
80 0
100 0
120 0
140 0
160 0
180 0
200 0
220 0
240 0
260 0
280 0
300 0
320 0
340 0
360 0
A-P:ASP-S2
20 0
40 2
60 5
80 6
100 7
120 6
140 5
160 4
180 0
200 0
220 0
240 5
260 6
280 7
300 6
320 5
340 4
360 1
U31-P:HIS-CA
20 0
40 0
60 0
80 0
100 0
120 0
140 0
160 0
180 0
200 0
220 0
240 0
260 0
280 0
300 0
320 0
340 0
360 0

H2U-MY:ARG-S1
20 0
40 0
60 0
80 0
100 0
120 0
140 0
160 0
180 0
200 0
220 0
240 0
260 0
280 0
300 0
320 0
340 0
360 0
G-P:GLN-CA
20 0
40 1
60 3
80 5
100 5
120 4
140 4
160 3
180 1
200 0
220 2
240 3
260 5
280 5
300 5
320 4
340 3
360 1
C-P:TYR-CA
20 0
40 0
60 2
80 2
100 3
120 2
140 2
160 1
180 0
200 0
220 0
240 0
260 2
280 3
300 2
320 2
340 1
360 0
G-R6:ARG-S2

20   0
40   3
60   7
80   9
100   10
120   9
140   7
160   5
180   2
200   0
220   3
240   7
260   9
280   10
300   9
320   8
340   5
360   2
C-P:SER-S1
20   0
40   1
60   3
80   5
100   5
120   4
140   3
160   2
180   1
200   0
220   2
240   3
260   5
280   5
300   4
320   4
340   3
360   1
G-R6:CYS-S1
20   0
40   0
60   0
80   0
100   0
120   0
140   1
160   0
180   0
200   0
220   0
240   1
260   0
280   0
300   1
320   0
340   0
360   0
G-RIB:GLN-S2
20   0

40 1
60 3
80 5
100 5
120 4
140 3
160 3
180 1
200 0
220 2
240 3
260 5
280 5
300 5
320 4
340 3
360 1
C-RIB:ALA-CA
20 0
40 2
60 5
80 7
100 8
120 7
140 5
160 4
180 1
200 0
220 3
240 5
260 7
280 8
300 7
320 6
340 4
360 1
U34-P:ARG-S1
20 0
40 0
60 0
80 0
100 0
120 0
140 0
160 0
180 0
200 0
220 0
240 0
260 0
280 0
300 0
320 0
340 0
360 0
A-R6:THR-CA
20 0
40 2

60   4
80   5
100   6
120   5
140   4
160   3
180   1
200   0
220   2
240   4
260   5
280   6
300   5
320   4
340   3
360   0
C-Y:ILE-CA
20   0
40   1
60   3
80   5
100   5
120   4
140   3
160   3
180   0
200   0
220   2
240   3
260   5
280   5
300   5
320   4
340   3
360   0
U-P:LYS-S1
20   0
40   1
60   3
80   4
100   4
120   4
140   3
160   2
180   1
200   0
220   1
240   3
260   4
280   4
300   4
320   3
340   2
360   1
FMU-MY:MET-S1
20   0
40   0
60   0

80 0
100 0
120 0
140 0
160 0
180 0
200 0
220 0
240 0
260 0
280 0
300 0
320 0
340 0
360 0
G-R6:LEU-S1
20 1
40 4
60 9
80 12
100 0
120 12
140 9
160 7
180 0
200 0
220 5
240 9
260 12
280 13
300 12
320 10
340 0
360 0
C31-RIB:ASP-S2
20 0
40 0
60 0
80 0
100 0
120 0
140 0
160 0
180 0
200 0
220 0
240 0
260 0
280 0
300 0
320 0
340 0
360 0
FHU-MY:TYR-S1
20 0
40 0
60 0
80 0

100 0
120 0
140 0
160 0
180 0
200 0
220 0
240 0
260 0
280 0
300 0
320 0
340 0
360 0
A-R6:GLN-CA
20 0
40 0
60 3
80 4
100 4
120 3
140 3
160 2
180 0
200 0
220 0
240 3
260 4
280 0
300 4
320 3
340 2
360 0
FHU-P:VAL-S1
20 0
40 0
60 0
80 0
100 0
120 0
140 0
160 0
180 0
200 0
220 0
240 0
260 0
280 0
300 0
320 0
340 0
360 0
A-R5:LEU-S1
20 0
40 3
60 7
80 10
100 10

120 9
140 0
160 5
180 0
200 0
220 4
240 7
260 10
280 10
300 9
320 8
340 6
360 0
PSU-RIB:ARG-S2
20 0
40 0
60 0
80 0
100 0
120 0
140 0
160 0
180 0
200 0
220 0
240 0
260 0
280 0
300 0
320 0
340 0
360 0
G-P:THR-CA
20 0
40 2
60 5
80 7
100 7
120 6
140 5
160 4
180 1
200 0
220 2
240 5
260 7
280 7
300 7
320 5
340 4
360 1
IU-MY:ALA-CA
20 0
40 0
60 0
80 0
100 0
120 0

140 0
160 0
180 0
200 0
220 0
240 0
260 0
280 0
300 0
320 0
340 0
360 0
C-Y:GLU-CA
20 0
40 2
60 5
80 7
100 0
120 7
140 5
160 4
180 1
200 0
220 3
240 5
260 7
280 7
300 7
320 0
340 4
360 0
IU-P:HIS-S2
20 0
40 0
60 0
80 0
100 0
120 0
140 0
160 0
180 0
200 0
220 0
240 0
260 0
280 0
300 0
320 0
340 0
360 0
C-Y:VAL-CA
20 0
40 2
60 5
80 6
100 7
120 6
140 5

160 3
180 0
200 0
220 2
240 4
260 6
280 7
300 6
320 5
340 0
360 0
U-P:SER-CA
20 0
40 1
60 2
80 3
100 3
120 3
140 2
160 2
180 0
200 0
220 1
240 2
260 3
280 3
300 3
320 2
340 2
360 0
U-RIB:ASP-S2
20 0
40 1
60 2
80 3
100 4
120 3
140 2
160 2
180 0
200 0
220 1
240 2
260 3
280 4
300 3
320 3
340 2
360 0
C31-P:PHE-CA
20 0
40 0
60 0
80 0
100 0
120 0
140 0
160 0

180 0
200 0
220 0
240 0
260 0
280 0
300 0
320 0
340 0
360 0
C31-MY:LEU-CA
20 0
40 0
60 0
80 0
100 0
120 0
140 0
160 0
180 0
200 0
220 0
240 0
260 0
280 0
300 0
320 0
340 0
360 0
IU-RIB:ILE-S1
20 0
40 0
60 0
80 0
100 0
120 0
140 0
160 0
180 0
200 0
220 0
240 0
260 0
280 0
300 0
320 0
340 0
360 0
A-RIB:ASP-S2
20 0
40 0
60 5
80 6
100 7
120 6
140 5
160 4
180 1

200 0
220 2
240 5
260 6
280 7
300 6
320 5
340 4
360 0
H2U-MY:GLU-S1
20 0
40 0
60 0
80 0
100 0
120 0
140 0
160 0
180 0
200 0
220 0
240 0
260 0
280 0
300 0
320 0
340 0
360 0
QUO-M6:ARG-CA
20 0
40 0
60 0
80 0
100 0
120 0
140 0
160 0
180 0
200 0
220 0
240 0
260 0
280 0
300 0
320 0
340 0
360 0
G-R6:SER-CA
20 0
40 3
60 5
80 7
100 8
120 7
140 6
160 4
180 0
200 0

220   3
240   5
260   7
280   8
300   7
320   6
340   4
360   1
A-P:CYS-S1
20   0
40   0
60   0
80   0
100   1
120   0
140   0
160   0
180   0
200   0
220   0
240   0
260   1
280   1
300   1
320   0
340   0
360   0
A-R5:TYR-S1
20   0
40   1
60   2
80   3
100   4
120   3
140   2
160   0
180   0
200   0
220   1
240   2
260   3
280   4
300   3
320   3
340   2
360   0
IU-P:SER-CA
20   0
40   0
60   0
80   0
100   0
120   0
140   0
160   0
180   0
200   0
220   0

240 0
260 0
280 0
300 0
320 0
340 0
360 0
C-RIB:THR-CA
20 0
40 1
60 3
80 4
100 4
120 4
140 3
160 2
180 1
200 0
220 1
240 3
260 4
280 4
300 4
320 3
340 2
360 1
FHU-P:LYS-S2
20 0
40 0
60 0
80 0
100 0
120 0
140 0
160 0
180 0
200 0
220 0
240 0
260 0
280 0
300 0
320 0
340 0
360 0
U-P:THR-CA
20 0
40 1
60 2
80 3
100 3
120 3
140 2
160 1
180 0
200 0
220 1
240 2

260   3
280   3
300   3
320   2
340   1
360   0
G-R5:ASP-S1
20   0
40   3
60   6
80   8
100   9
120   8
140   6
160   5
180   0
200   0
220   3
240   6
260   8
280   9
300   8
320   7
340   5
360   2
C-RIB:GLY-CA
20   0
40   2
60   4
80   6
100   6
120   6
140   5
160   3
180   1
200   0
220   2
240   4
260   6
280   6
300   6
320   5
340   3
360   1
G-P:PRO-CA
20   0
40   2
60   4
80   6
100   7
120   6
140   5
160   3
180   1
200   0
220   2
240   4
260   6

280 7
300 6
320 5
340 4
360 1
G-R6:ILE-CA
20 0
40 0
60 5
80 0
100 8
120 7
140 0
160 4
180 1
200 0
220 0
240 5
260 7
280 8
300 7
320 6
340 0
360 0
FHU-MY:THR-S1
20 0
40 0
60 0
80 0
100 0
120 0
140 0
160 0
180 0
200 0
220 0
240 0
260 0
280 0
300 0
320 0
340 0
360 0
A-R5:ALA-CA
20 1
40 3
60 7
80 9
100 10
120 9
140 7
160 5
180 0
200 0
220 3
240 7
260 9
280 10

300 9
320 7
340 5
360 2
A-R6:TYR-CA
20 0
40 1
60 2
80 3
100 4
120 3
140 0
160 2
180 0
200 0
220 1
240 2
260 3
280 4
300 3
320 3
340 2
360 0
QUO-M5:ARG-S2
20 0
40 0
60 0
80 0
100 0
120 0
140 0
160 0
180 0
200 0
220 0
240 0
260 0
280 0
300 0
320 0
340 0
360 0
A-R6:TRP-CA
20 0
40 0
60 1
80 1
100 1
120 1
140 0
160 0
180 0
200 0
220 0
240 1
260 1
280 1
300 1

320 0
340 0
360 0
G-R6:HIS-S1
20 0
40 1
60 2
80 3
100 3
120 3
140 2
160 0
180 0
200 0
220 1
240 2
260 3
280 3
300 3
320 2
340 0
360 0
C31-P:TYR-CA
20 0
40 0
60 0
80 0
100 0
120 0
140 0
160 0
180 0
200 0
220 0
240 0
260 0
280 0
300 0
320 0
340 0
360 0
U-P:MET-CA
20 0
40 0
60 1
80 0
100 1
120 1
140 1
160 0
180 0
200 0
220 0
240 1
260 0
280 1
300 1
320 1

340 0
360 0
DA-M5:ASP-S2
20 0
40 0
60 0
80 0
100 0
120 0
140 0
160 0
180 0
200 0
220 0
240 0
260 0
280 0
300 0
320 0
340 0
360 0
FHU-P:PRO-CA
20 0
40 0
60 0
80 0
100 0
120 0
140 0
160 0
180 0
200 0
220 0
240 0
260 0
280 0
300 0
320 0
340 0
360 0
U-P:ASP-S1
20 0
40 0
60 2
80 3
100 4
120 3
140 3
160 0
180 0
200 0
220 1
240 2
260 3
280 4
300 3
320 3
340 2

360 0
A-R5:ALA-S1
20 1
40 3
60 7
80 9
100 10
120 9
140 7
160 5
180 2
200 0
220 3
240 7
260 9
280 10
300 9
320 7
340 5
360 0
QUO-RIB:PHE-S1
20 0
40 0
60 0
80 0
100 0
120 0
140 0
160 0
180 0
200 0
220 0
240 0
260 0
280 0
300 0
320 0
340 0
360 0
C31-P:ASN-S2
20 0
40 0
60 0
80 0
100 0
120 0
140 0
160 0
180 0
200 0
220 0
240 0
260 0
280 0
300 0
320 0
340 0
360 0

A-R5:LYS-CA
20 0
40 3
60 5
80 7
100 8
120 7
140 6
160 4
180 1
200 0
220 3
240 5
260 8
280 8
300 7
320 6
340 4
360 0
U34-P:TYR-S1
20 0
40 0
60 0
80 0
100 0
120 0
140 0
160 0
180 0
200 0
220 0
240 0
260 0
280 0
300 0
320 0
340 0
360 0
H2U-MY:ALA-CA
20 0
40 0
60 0
80 0
100 0
120 0
140 0
160 0
180 0
200 0
220 0
240 0
260 0
280 0
300 0
320 0
340 0
360 0
G-R5:LEU-S1

20 0
40 4
60 9
80 12
100 13
120 12
140 9
160 7
180 0
200 1
220 5
240 9
260 12
280 13
300 12
320 10
340 7
360 0
IU-RIB:GLN-S2
20 0
40 0
60 0
80 0
100 0
120 0
140 0
160 0
180 0
200 0
220 0
240 0
260 0
280 0
300 0
320 0
340 0
360 0
U-P:ARG-S2
20 0
40 1
60 3
80 4
100 4
120 4
140 3
160 2
180 0
200 0
220 1
240 3
260 4
280 4
300 4
320 3
340 2
360 1
U-P:PHE-CA
20 0

40 0
60 1
80 2
100 0
120 2
140 1
160 1
180 0
200 0
220 0
240 1
260 2
280 2
300 0
320 2
340 0
360 0
C31-MY:GLN-S1
20 0
40 0
60 0
80 0
100 0
120 0
140 0
160 0
180 0
200 0
220 0
240 0
260 0
280 0
300 0
320 0
340 0
360 0
A-P:ASN-CA
20 0
40 1
60 3
80 4
100 4
120 4
140 3
160 2
180 1
200 0
220 1
240 3
260 4
280 4
300 4
320 3
340 2
360 1
FHU-RIB:PRO-S1
20 0
40 0

60 0
80 0
100 0
120 0
140 0
160 0
180 0
200 0
220 0
240 0
260 0
280 0
300 0
320 0
340 0
360 0
A-P:HIS-S2
20 0
40 1
60 2
80 2
100 2
120 2
140 2
160 1
180 0
200 0
220 1
240 2
260 2
280 2
300 2
320 2
340 1
360 0
IU-MY:LYS-S1
20 0
40 0
60 0
80 0
100 0
120 0
140 0
160 0
180 0
200 0
220 0
240 0
260 0
280 0
300 0
320 0
340 0
360 0
A-R6:THR-S1
20 0
40 2
60 4

80 5
100 6
120 5
140 4
160 3
180 1
200 0
220 2
240 4
260 5
280 6
300 5
320 4
340 3
360 1
U-P:MET-S1
20 0
40 0
60 1
80 1
100 1
120 1
140 0
160 0
180 0
200 0
220 0
240 1
260 1
280 1
300 1
320 1
340 0
360 0
I-RIB:TRP-S1
20 0
40 0
60 0
80 0
100 0
120 0
140 0
160 0
180 0
200 0
220 0
240 0
260 0
280 0
300 0
320 0
340 0
360 0
U34-RIB:ASN-CA
20 0
40 0
60 0
80 0

100 0
120 0
140 0
160 0
180 0
200 0
220 0
240 0
260 0
280 0
300 0
320 0
340 0
360 0
GTP-M5:ALA-CA
20 0
40 0
60 0
80 0
100 0
120 0
140 0
160 0
180 0
200 0
220 0
240 0
260 0
280 0
300 0
320 0
340 0
360 0
IU-P:ASP-S2
20 0
40 0
60 0
80 0
100 0
120 0
140 0
160 0
180 0
200 0
220 0
240 0
260 0
280 0
300 0
320 0
340 0
360 0
FHU-P:LYS-S1
20 0
40 0
60 0
80 0
100 0

120 0
140 0
160 0
180 0
200 0
220 0
240 0
260 0
280 0
300 0
320 0
340 0
360 0
G-R5:LYS-S2
20 1
40 3
60 7
80 9
100 10
120 9
140 7
160 5
180 2
200 0
220 4
240 7
260 9
280 10
300 9
320 8
340 6
360 2
G-R5:ARG-S2
20 1
40 3
60 7
80 9
100 10
120 9
140 7
160 5
180 2
200 0
220 3
240 7
260 9
280 10
300 9
320 8
340 5
360 2
G-P:HIS-S1
20 0
40 1
60 2
80 3
100 3
120 3

140 2
160 2
180 0
200 0
220 1
240 2
260 3
280 3
300 3
320 2
340 2
360 0
IU-MY:LYS-S2
20 0
40 0
60 0
80 0
100 0
120 0
140 0
160 0
180 0
200 0
220 0
240 0
260 0
280 0
300 0
320 0
340 0
360 0
U-P:ALA-CA
20 0
40 2
60 3
80 5
100 5
120 5
140 4
160 3
180 1
200 0
220 2
240 3
260 5
280 5
300 5
320 4
340 3
360 1
C31-MY:THR-S1
20 0
40 0
60 0
80 0
100 0
120 0
140 0

160 0
180 0
200 0
220 0
240 0
260 0
280 0
300 0
320 0
340 0
360 0
A-R5:LEU-CA
20 1
40 3
60 7
80 10
100 10
120 9
140 7
160 5
180 0
200 0
220 4
240 7
260 10
280 10
300 9
320 8
340 6
360 0
G-R6:GLN-S1
20 0
40 1
60 3
80 5
100 5
120 4
140 0
160 3
180 0
200 0
220 2
240 3
260 5
280 5
300 5
320 4
340 0
360 0
C31-RIB:THR-S1
20 0
40 0
60 0
80 0
100 0
120 0
140 0
160 0

180   0
200   0
220   0
240   0
260   0
280   0
300   0
320   0
340   0
360   0
U34-P:SER-CA
20   0
40   0
60   0
80   0
100   0
120   0
140   0
160   0
180   0
200   0
220   0
240   0
260   0
280   0
300   0
320   0
340   0
360   0
A-R5:LYS-S2
20   0
40   3
60   5
80   7
100   8
120   7
140   6
160   4
180   0
200   0
220   3
240   5
260   7
280   8
300   7
320   6
340   4
360   0
FMU-MY:PRO-S1
20   0
40   0
60   0
80   0
100   0
120   0
140   0
160   0
180   0

200 0
220 0
240 0
260 0
280 0
300 0
320 0
340 0
360 0
I-RIB:ALA-CA
20 0
40 0
60 0
80 0
100 0
120 0
140 0
160 0
180 0
200 0
220 0
240 0
260 0
280 0
300 0
320 0
340 0
360 0
IU-MY:PRO-S1
20 0
40 0
60 0
80 0
100 0
120 0
140 0
160 0
180 0
200 0
220 0
240 0
260 0
280 0
300 0
320 0
340 0
360 0
A-P:MET-CA
20 0
40 0
60 2
80 2
100 3
120 2
140 2
160 1
180 0
200 0

220 0
240 2
260 2
280 3
300 2
320 2
340 0
360 0
DA-M6:TYR-CA
20 0
40 0
60 0
80 0
100 0
120 0
140 0
160 0
180 0
200 0
220 0
240 0
260 0
280 0
300 0
320 0
340 0
360 0
A-P:PRO-CA
20 0
40 0
60 3
80 5
100 5
120 5
140 4
160 3
180 1
200 0
220 2
240 3
260 5
280 5
300 5
320 4
340 3
360 1
IU-MY:LEU-S1
20 0
40 0
60 0
80 0
100 0
120 0
140 0
160 0
180 0
200 0
220 0

240 0
260 0
280 0
300 0
320 0
340 0
360 0
5BU-P:PRO-CA
20 0
40 0
60 0
80 0
100 0
120 0
140 0
160 0
180 0
200 0
220 0
240 0
260 0
280 0
300 0
320 0
340 0
360 0
DA-M6:TYR-S2
20 0
40 0
60 0
80 0
100 0
120 0
140 0
160 0
180 0
200 0
220 0
240 0
260 0
280 0
300 0
320 0
340 0
360 0
G-P:MET-CA
20 0
40 0
60 2
80 3
100 3
120 3
140 2
160 0
180 0
200 0
220 1
240 2

260 3
280 3
300 3
320 2
340 2
360 0
U31-MY:ASP-CA
20 0
40 0
60 0
80 0
100 0
120 0
140 0
160 0
180 0
200 0
220 0
240 0
260 0
280 0
300 0
320 0
340 0
360 0
U-Y:TRP-S2
20 0
40 0
60 0
80 0
100 0
120 0
140 0
160 0
180 0
200 0
220 0
240 0
260 0
280 0
300 0
320 0
340 0
360 0
U34-MY:ASP-S2
20 0
40 0
60 0
80 0
100 0
120 0
140 0
160 0
180 0
200 0
220 0
240 0
260 0

280 0
300 0
320 0
340 0
360 0
A-RIB:TYR-S2
20 0
40 1
60 2
80 3
100 4
120 3
140 2
160 2
180 0
200 0
220 1
240 2
260 3
280 4
300 3
320 3
340 0
360 0
FHU-P:SER-S1
20 0
40 0
60 0
80 0
100 0
120 0
140 0
160 0
180 0
200 0
220 0
240 0
260 0
280 0
300 0
320 0
340 0
360 0
A-P:GLY-CA
20 0
40 3
60 6
80 8
100 8
120 7
140 6
160 4
180 1
200 0
220 3
240 5
260 8
280 8

300   7
320   6
340   4
360   1
C-P:LEU-S1
20   0
40   3
60   5
80   7
100   8
120   7
140   6
160   4
180   1
200   0
220   3
240   5
260   8
280   8
300   7
320   6
340   4
360   1
IU-RIB:HIS-CA
20   0
40   0
60   0
80   0
100   0
120   0
140   0
160   0
180   0
200   0
220   0
240   0
260   0
280   0
300   0
320   0
340   0
360   0
QUO-M6:ASN-S2
20   0
40   0
60   0
80   0
100   0
120   0
140   0
160   0
180   0
200   0
220   0
240   0
260   0
280   0
300   0

320 0
340 0
360 0
U-Y:PRO-CA
20 0
40 1
60 2
80 2
100 3
120 2
140 2
160 1
180 0
200 0
220 0
240 2
260 2
280 3
300 2
320 2
340 1
360 0
G-P:ALA-CA
20 0
40 0
60 8
80 11
100 12
120 11
140 9
160 6
180 0
200 0
220 4
240 8
260 11
280 12
300 11
320 9
340 7
360 2
G-R6:PHE-S1
20 0
40 0
60 4
80 5
100 5
120 5
140 4
160 3
180 0
200 0
220 2
240 4
260 5
280 5
300 5
320 0

340 0
360 0
U31-P:SER-S1
20 0
40 0
60 0
80 0
100 0
120 0
140 0
160 0
180 0
200 0
220 0
240 0
260 0
280 0
300 0
320 0
340 0
360 0
FMU-MY:ASN-S1
20 0
40 0
60 0
80 0
100 0
120 0
140 0
160 0
180 0
200 0
220 0
240 0
260 0
280 0
300 0
320 0
340 0
360 0
U31-P:HIS-S1
20 0
40 0
60 0
80 0
100 0
120 0
140 0
160 0
180 0
200 0
220 0
240 0
260 0
280 0
300 0
320 0
340 0

360 0
QUO-M5:ASP-CA
20 0
40 0
60 0
80 0
100 0
120 0
140 0
160 0
180 0
200 0
220 0
240 0
260 0
280 0
300 0
320 0
340 0
360 0
U31-P:LEU-S2
20 0
40 0
60 0
80 0
100 0
120 0
140 0
160 0
180 0
200 0
220 0
240 0
260 0
280 0
300 0
320 0
340 0
360 0
A-P:THR-S1
20 0
40 2
60 4
80 5
100 6
120 5
140 4
160 3
180 1
200 0
220 2
240 4
260 5
280 6
300 5
320 4
340 3
360 1

FHU-RIB:LYS-S2
20 0
40 0
60 0
80 0
100 0
120 0
140 0
160 0
180 0
200 0
220 0
240 0
260 0
280 0
300 0
320 0
340 0
360 0
U-RIB:THR-S1
20 0
40 1
60 2
80 3
100 3
120 3
140 2
160 1
180 0
200 0
220 1
240 2
260 3
280 3
300 3
320 2
340 1
360 0
A-R6:GLN-S1
20 0
40 1
60 3
80 4
100 4
120 3
140 3
160 2
180 0
200 0
220 1
240 3
260 4
280 4
300 4
320 3
340 2
360 1
M2G-P:GLY-CA

20 0
40 0
60 0
80 0
100 0
120 0
140 0
160 0
180 0
200 0
220 0
240 0
260 0
280 0
300 0
320 0
340 0
360 0
U-Y:HIS-CA
20 0
40 0
60 1
80 0
100 1
120 1
140 1
160 0
180 0
200 0
220 0
240 1
260 0
280 1
300 1
320 1
340 0
360 0
FMU-RIB:CYS-CA
20 0
40 0
60 0
80 0
100 0
120 0
140 0
160 0
180 0
200 0
220 0
240 0
260 0
280 0
300 0
320 0
340 0
360 0
FMU-RIB:PHE-S1
20 0

40 0
60 0
80 0
100 0
120 0
140 0
160 0
180 0
200 0
220 0
240 0
260 0
280 0
300 0
320 0
340 0
360 0
G-RIB:HIS-CA
20 0
40 1
60 2
80 3
100 3
120 3
140 2
160 0
180 0
200 0
220 1
240 2
260 3
280 3
300 3
320 2
340 2
360 0
A-RIB:LYS-CA
20 0
40 3
60 5
80 7
100 8
120 7
140 6
160 4
180 1
200 0
220 3
240 5
260 8
280 8
300 7
320 6
340 4
360 1
U-P:ASN-CA
20 0
40 0

60 1
80 2
100 2
120 2
140 1
160 1
180 0
200 0
220 0
240 1
260 2
280 2
300 2
320 2
340 1
360 0
G-RIB:TRP-S1
20 0
40 0
60 1
80 1
100 2
120 1
140 1
160 1
180 0
200 0
220 0
240 1
260 1
280 2
300 1
320 1
340 1
360 0
5BU-MY:ARG-S2
20 0
40 0
60 0
80 0
100 0
120 0
140 0
160 0
180 0
200 0
220 0
240 0
260 0
280 0
300 0
320 0
340 0
360 0
G-RIB:GLN-CA
20 0
40 1
60 3

80 5
100 5
120 4
140 4
160 3
180 1
200 0
220 2
240 3
260 5
280 5
300 5
320 4
340 3
360 1
G-R5:GLU-CA
20 1
40 4
60 8
80 11
100 12
120 11
140 9
160 6
180 2
200 0
220 4
240 8
260 11
280 12
300 11
320 9
340 7
360 2
A-R5:ASN-S2
20 0
40 1
60 3
80 4
100 4
120 4
140 3
160 2
180 0
200 0
220 1
240 3
260 4
280 4
300 4
320 3
340 2
360 1
U34-P:HIS-S2
20 0
40 0
60 0
80 0

100 0
120 0
140 0
160 0
180 0
200 0
220 0
240 0
260 0
280 0
300 0
320 0
340 0
360 0
A-R6:ALA-CA
20 0
40 3
60 7
80 9
100 10
120 9
140 0
160 5
180 2
200 0
220 3
240 7
260 0
280 10
300 9
320 7
340 5
360 2
GTP-M5:SER-S1
20 0
40 0
60 0
80 0
100 0
120 0
140 0
160 0
180 0
200 0
220 0
240 0
260 0
280 0
300 0
320 0
340 0
360 0
U-RIB:GLU-CA
20 0
40 1
60 3
80 5
100 5

120   4
140   4
160   3
180   0
200   0
220   2
240   3
260   5
280   5
300   5
320   4
340   3
360   1
C-Y:ASP-S2
20   0
40   2
60   4
80   5
100   5
120   5
140   4
160   3
180   1
200   0
220   2
240   4
260   5
280   5
300   5
320   4
340   3
360   0
DA-M5:LYS-S2
20   0
40   0
60   0
80   0
100   0
120   0
140   0
160   0
180   0
200   0
220   0
240   0
260   0
280   0
300   0
320   0
340   0
360   0
5BU-RIB:PRO-S1
20   0
40   0
60   0
80   0
100   0
120   0

140 0
160 0
180 0
200 0
220 0
240 0
260 0
280 0
300 0
320 0
340 0
360 0
C-Y:LEU-S2
20 0
40 3
60 5
80 7
100 8
120 7
140 6
160 0
180 1
200 0
220 3
240 5
260 8
280 8
300 7
320 0
340 0
360 0
C-Y:PHE-S2
20 0
40 0
60 2
80 3
100 3
120 3
140 2
160 0
180 0
200 0
220 1
240 2
260 3
280 3
300 3
320 0
340 0
360 0
G-R6:TYR-S1
20 0
40 0
60 3
80 4
100 5
120 4
140 0

160 2
180 0
200 0
220 1
240 0
260 4
280 5
300 4
320 3
340 2
360 1
G-RIB:ALA-S1
20 1
40 4
60 8
80 11
100 12
120 11
140 9
160 7
180 2
200 1
220 4
240 8
260 11
280 12
300 11
320 10
340 7
360 2
C-P:ASN-S2
20 0
40 1
60 2
80 3
100 3
120 3
140 2
160 2
180 0
200 0
220 1
240 2
260 3
280 3
300 3
320 3
340 2
360 0
A-P:LYS-CA
20 0
40 3
60 5
80 7
100 8
120 7
140 6
160 4

180 1
200 0
220 3
240 5
260 8
280 8
300 7
320 6
340 4
360 1
DA-M5:GLU-S2
20 0
40 0
60 0
80 0
100 0
120 0
140 0
160 0
180 0
200 0
220 0
240 0
260 0
280 0
300 0
320 0
340 0
360 0
QUO-M5:GLN-S2
20 0
40 0
60 0
80 0
100 0
120 0
140 0
160 0
180 0
200 0
220 0
240 0
260 0
280 0
300 0
320 0
340 0
360 0
QUO-M6:GLU-S2
20 0
40 0
60 0
80 0
100 0
120 0
140 0
160 0
180 0

200 0
220 0
240 0
260 0
280 0
300 0
320 0
340 0
360 0
A-P:LEU-S1
20 0
40 3
60 7
80 9
100 10
120 9
140 7
160 5
180 2
200 0
220 4
240 7
260 10
280 10
300 9
320 8
340 6
360 0
G-R6:ASN-CA
20 0
40 0
60 4
80 5
100 6
120 5
140 4
160 3
180 0
200 0
220 2
240 4
260 5
280 6
300 5
320 4
340 3
360 1
FMU-RIB:ARG-CA
20 0
40 0
60 0
80 0
100 0
120 0
140 0
160 0
180 0
200 0

220 0
240 0
260 0
280 0
300 0
320 0
340 0
360 0
U-RIB:LEU-S1
20 0
40 0
60 4
80 5
100 5
120 5
140 4
160 3
180 1
200 0
220 0
240 0
260 5
280 5
300 5
320 4
340 3
360 1
QUO-P:SER-S1
20 0
40 0
60 0
80 0
100 0
120 0
140 0
160 0
180 0
200 0
220 0
240 0
260 0
280 0
300 0
320 0
340 0
360 0
C-Y:GLN-S2
20 0
40 1
60 2
80 3
100 3
120 3
140 2
160 0
180 0
200 0
220 1

240 2
260 3
280 3
300 3
320 0
340 1
360 0
QUO-RIB:ASN-S1
20 0
40 0
60 0
80 0
100 0
120 0
140 0
160 0
180 0
200 0
220 0
240 0
260 0
280 0
300 0
320 0
340 0
360 0
A-R5:TYR-S2
20 0
40 1
60 2
80 3
100 4
120 3
140 2
160 2
180 0
200 0
220 1
240 2
260 3
280 4
300 3
320 0
340 2
360 0
U-P:PHE-S2
20 0
40 0
60 1
80 0
100 2
120 2
140 1
160 1
180 0
200 0
220 0
240 1

260 2
280 2
300 2
320 0
340 0
360 0
A-P:TRP-CA
20 0
40 0
60 1
80 1
100 1
120 1
140 1
160 0
180 0
200 0
220 0
240 0
260 1
280 1
300 1
320 1
340 0
360 0
U31-MY:PHE-S1
20 0
40 0
60 0
80 0
100 0
120 0
140 0
160 0
180 0
200 0
220 0
240 0
260 0
280 0
300 0
320 0
340 0
360 0
C-P:LEU-CA
20 0
40 3
60 5
80 7
100 8
120 7
140 6
160 4
180 0
200 0
220 3
240 5
260 8

280 8
300 7
320 6
340 4
360 1
FMU-RIB:HIS-S2
20 0
40 0
60 0
80 0
100 0
120 0
140 0
160 0
180 0
200 0
220 0
240 0
260 0
280 0
300 0
320 0
340 0
360 0
G-R5:ASP-CA
20 0
40 3
60 6
80 8
100 9
120 0
140 6
160 5
180 0
200 0
220 3
240 6
260 8
280 9
300 8
320 7
340 5
360 0
U34-MY:PHE-S1
20 0
40 0
60 0
80 0
100 0
120 0
140 0
160 0
180 0
200 0
220 0
240 0
260 0
280 0

300 0
320 0
340 0
360 0
G-R5:TYR-CA
20 0
40 1
60 0
80 4
100 0
120 4
140 3
160 2
180 1
200 0
220 0
240 3
260 4
280 0
300 4
320 3
340 0
360 0
G-RIB:TYR-S1
20 0
40 0
60 3
80 4
100 5
120 4
140 3
160 2
180 0
200 0
220 1
240 3
260 4
280 5
300 4
320 0
340 2
360 0
5BU-P:SER-CA
20 0
40 0
60 0
80 0
100 0
120 0
140 0
160 0
180 0
200 0
220 0
240 0
260 0
280 0
300 0

320 0
340 0
360 0
C-RIB:ASP-CA
20 0
40 2
60 4
80 5
100 6
120 5
140 4
160 3
180 1
200 0
220 2
240 4
260 5
280 6
300 5
320 4
340 3
360 1
A-R6:GLY-CA
20 0
40 3
60 6
80 8
100 8
120 7
140 6
160 4
180 0
200 0
220 3
240 5
260 8
280 8
300 8
320 6
340 4
360 1
U-Y:ARG-S2
20 0
40 1
60 3
80 4
100 4
120 4
140 3
160 2
180 0
200 0
220 1
240 3
260 4
280 4
300 4
320 3

340 2
360 1
A-R6:VAL-S1
20 0
40 3
60 6
80 8
100 9
120 8
140 6
160 4
180 0
200 0
220 3
240 6
260 8
280 9
300 8
320 7
340 5
360 0
A-P:GLN-CA
20 0
40 0
60 3
80 4
100 4
120 3
140 3
160 2
180 0
200 0
220 1
240 3
260 4
280 4
300 4
320 3
340 2
360 1
FMU-P:ARG-S1
20 0
40 0
60 0
80 0
100 0
120 0
140 0
160 0
180 0
200 0
220 0
240 0
260 0
280 0
300 0
320 0
340 0

360 0
QUO-M5:ASP-S1
20 0
40 0
60 0
80 0
100 0
120 0
140 0
160 0
180 0
200 0
220 0
240 0
260 0
280 0
300 0
320 0
340 0
360 0
FHU-MY:LEU-S1
20 0
40 0
60 0
80 0
100 0
120 0
140 0
160 0
180 0
200 0
220 0
240 0
260 0
280 0
300 0
320 0
340 0
360 0
A-R6:GLN-S2
20 0
40 1
60 3
80 4
100 4
120 3
140 3
160 2
180 0
200 0
220 1
240 3
260 4
280 4
300 4
320 3
340 2
360 0

U-RIB:PHE-CA
20 0
40 0
60 1
80 2
100 2
120 2
140 1
160 1
180 0
200 0
220 0
240 1
260 0
280 2
300 2
320 2
340 1
360 0
G-P:MET-S1
20 0
40 0
60 2
80 3
100 3
120 3
140 2
160 2
180 0
200 0
220 0
240 2
260 3
280 3
300 3
320 2
340 2
360 0
G-RIB:ALA-CA
20 1
40 4
60 8
80 11
100 12
120 11
140 9
160 7
180 2
200 1
220 4
240 8
260 11
280 12
300 11
320 10
340 7
360 2
U-RIB:ILE-S1

20 0
40 0
60 2
80 3
100 3
120 3
140 2
160 2
180 0
200 0
220 1
240 2
260 3
280 3
300 0
320 2
340 2
360 0
U-P:MET-S2
20 0
40 0
60 1
80 1
100 1
120 0
140 1
160 0
180 0
200 0
220 0
240 1
260 1
280 1
300 1
320 1
340 0
360 0
OMC-P:LYS-CA
20 0
40 0
60 0
80 0
100 0
120 0
140 0
160 0
180 0
200 0
220 0
240 0
260 0
280 0
300 0
320 0
340 0
360 0
U-Y:LEU-S1
20 0

40 2
60 4
80 5
100 5
120 5
140 4
160 3
180 1
200 0
220 0
240 4
260 5
280 5
300 5
320 4
340 3
360 0
IU-P:HIS-S1
20 0
40 0
60 0
80 0
100 0
120 0
140 0
160 0
180 0
200 0
220 0
240 0
260 0
280 0
300 0
320 0
340 0
360 0
DA-M6:LYS-S1
20 0
40 0
60 0
80 0
100 0
120 0
140 0
160 0
180 0
200 0
220 0
240 0
260 0
280 0
300 0
320 0
340 0
360 0
U-P:GLN-S1
20 0
40 0

60 1
80 2
100 2
120 2
140 1
160 1
180 0
200 0
220 0
240 1
260 2
280 2
300 2
320 1
340 1
360 0
U31-RIB:PHE-CA
20 0
40 0
60 0
80 0
100 0
120 0
140 0
160 0
180 0
200 0
220 0
240 0
260 0
280 0
300 0
320 0
340 0
360 0
A-R6:PHE-S1
20 0
40 1
60 3
80 0
100 4
120 4
140 0
160 0
180 0
200 0
220 0
240 0
260 4
280 4
300 4
320 0
340 0
360 0
G-R5:PRO-CA
20 0
40 2
60 4

80 6
100 7
120 6
140 5
160 0
180 0
200 0
220 2
240 4
260 6
280 7
300 6
320 5
340 4
360 1
A-R5:ASP-CA
20 0
40 2
60 5
80 6
100 7
120 6
140 5
160 4
180 0
200 0
220 0
240 5
260 7
280 7
300 6
320 5
340 4
360 0
G-R5:HIS-S1
20 0
40 1
60 2
80 3
100 3
120 3
140 2
160 0
180 0
200 0
220 1
240 2
260 3
280 3
300 3
320 2
340 0
360 0
C31-P:ALA-S1
20 0
40 0
60 0
80 0

100 0
120 0
140 0
160 0
180 0
200 0
220 0
240 0
260 0
280 0
300 0
320 0
340 0
360 0
IU-RIB:GLN-S1
20 0
40 0
60 0
80 0
100 0
120 0
140 0
160 0
180 0
200 0
220 0
240 0
260 0
280 0
300 0
320 0
340 0
360 0
A-RIB:PHE-S2
20 0
40 1
60 3
80 4
100 4
120 4
140 3
160 2
180 1
200 0
220 0
240 0
260 4
280 4
300 4
320 0
340 2
360 1
FMU-P:CYS-S1
20 0
40 0
60 0
80 0
100 0

120 0
140 0
160 0
180 0
200 0
220 0
240 0
260 0
280 0
300 0
320 0
340 0
360 0
C-P:TRP-CA
20 0
40 0
60 0
80 1
100 0
120 1
140 0
160 0
180 0
200 0
220 0
240 0
260 1
280 1
300 0
320 1
340 0
360 0
FMU-MY:GLU-CA
20 0
40 0
60 0
80 0
100 0
120 0
140 0
160 0
180 0
200 0
220 0
240 0
260 0
280 0
300 0
320 0
340 0
360 0
G-R6:GLU-CA
20 0
40 4
60 8
80 11
100 12
120 11

140   9
160   6
180   2
200   0
220   4
240   8
260   11
280   12
300   11
320   9
340   7
360   2
A-R6:ASN-CA
20   0
40   0
60   3
80   4
100   4
120   4
140   3
160   2
180   1
200   0
220   1
240   3
260   4
280   4
300   4
320   3
340   2
360   0
A-R6:ASP-CA
20   0
40   2
60   5
80   6
100   7
120   6
140   5
160   4
180   0
200   0
220   0
240   5
260   7
280   7
300   6
320   5
340   4
360   1
C-Y:ASN-S1
20   0
40   1
60   2
80   3
100   0
120   3
140   2

160	2
180	0
200	0
220	1
240	2
260	3
280	3
300	3
320	0
340	2
360	0

FHU-MY:TYR-S2

20	0
40	0
60	0
80	0
100	0
120	0
140	0
160	0
180	0
200	0
220	0
240	0
260	0
280	0
300	0
320	0
340	0
360	0

5BU-P:ARG-CA

20	0
40	0
60	0
80	0
100	0
120	0
140	0
160	0
180	0
200	0
220	0
240	0
260	0
280	0
300	0
320	0
340	0
360	0

U-P:ALA-S1

20	0
40	2
60	3
80	5
100	5
120	5
140	4
160	3

180 1
200 0
220 2
240 3
260 5
280 5
300 5
320 4
340 3
360 1
H2U-P:ARG-S1
20 0
40 0
60 0
80 0
100 0
120 0
140 0
160 0
180 0
200 0
220 0
240 0
260 0
280 0
300 0
320 0
340 0
360 0
A-R5:CYS-S1
20 0
40 0
60 0
80 1
100 0
120 0
140 0
160 0
180 0
200 0
220 0
240 0
260 0
280 1
300 1
320 0
340 0
360 0
G-R5:TRP-S2
20 0
40 0
60 0
80 1
100 2
120 1
140 1
160 1
180 0

200 0
220 0
240 1
260 1
280 0
300 1
320 1
340 0
360 0
H2U-MY:TRP-S2
20 0
40 0
60 0
80 0
100 0
120 0
140 0
160 0
180 0
200 0
220 0
240 0
260 0
280 0
300 0
320 0
340 0
360 0
IU-MY:ALA-S1
20 0
40 0
60 0
80 0
100 0
120 0
140 0
160 0
180 0
200 0
220 0
240 0
260 0
280 0
300 0
320 0
340 0
360 0
FMU-P:PHE-S2
20 0
40 0
60 0
80 0
100 0
120 0
140 0
160 0
180 0
200 0

220 0
240 0
260 0
280 0
300 0
320 0
340 0
360 0
H2U-MY:PRO-CA
20 0
40 0
60 0
80 0
100 0
120 0
140 0
160 0
180 0
200 0
220 0
240 0
260 0
280 0
300 0
320 0
340 0
360 0
U-Y:HIS-S1
20 0
40 0
60 1
80 1
100 1
120 1
140 1
160 0
180 0
200 0
220 0
240 1
260 0
280 1
300 1
320 1
340 0
360 0
G-R6:ALA-S1
20 1
40 4
60 8
80 11
100 12
120 11
140 9
160 7
180 2
200 0
220 4

240 8
260 11
280 12
300 11
320 10
340 7
360 0
C-P:THR-CA
20 0
40 1
60 3
80 4
100 4
120 4
140 3
160 2
180 1
200 0
220 1
240 3
260 4
280 4
300 4
320 3
340 2
360 1
A-RIB:ASP-S1
20 0
40 0
60 0
80 6
100 7
120 6
140 5
160 4
180 0
200 0
220 0
240 5
260 7
280 7
300 6
320 5
340 4
360 1
U31-P:ARG-S1
20 0
40 0
60 0
80 0
100 0
120 0
140 0
160 0
180 0
200 0
220 0
240 0

260 0
280 0
300 0
320 0
340 0
360 0
C31-P:TYR-S1
20 0
40 0
60 0
80 0
100 0
120 0
140 0
160 0
180 0
200 0
220 0
240 0
260 0
280 0
300 0
320 0
340 0
360 0
H2U-RIB:LYS-S1
20 0
40 0
60 0
80 0
100 0
120 0
140 0
160 0
180 0
200 0
220 0
240 0
260 0
280 0
300 0
320 0
340 0
360 0
U-P:VAL-S1
20 0
40 1
60 3
80 4
100 4
120 4
140 3
160 2
180 1
200 0
220 1
240 3
260 4

280 4
300 4
320 3
340 2
360 0
IU-P:ILE-CA
20 0
40 0
60 0
80 0
100 0
120 0
140 0
160 0
180 0
200 0
220 0
240 0
260 0
280 0
300 0
320 0
340 0
360 0
IU-MY:VAL-S1
20 0
40 0
60 0
80 0
100 0
120 0
140 0
160 0
180 0
200 0
220 0
240 0
260 0
280 0
300 0
320 0
340 0
360 0
QUO-P:ASN-S2
20 0
40 0
60 0
80 0
100 0
120 0
140 0
160 0
180 0
200 0
220 0
240 0
260 0
280 0

300 0
320 0
340 0
360 0
FMU-P:PHE-CA
20 0
40 0
60 0
80 0
100 0
120 0
140 0
160 0
180 0
200 0
220 0
240 0
260 0
280 0
300 0
320 0
340 0
360 0
H2U-P:LEU-S2
20 0
40 0
60 0
80 0
100 0
120 0
140 0
160 0
180 0
200 0
220 0
240 0
260 0
280 0
300 0
320 0
340 0
360 0
A-R5:ILE-CA
20 0
40 2
60 4
80 6
100 6
120 6
140 0
160 3
180 0
200 0
220 2
240 4
260 6
280 0
300 6

320 5
340 0
360 0
U-P:PRO-S1
20 0
40 1
60 2
80 2
100 3
120 2
140 2
160 1
180 0
200 0
220 1
240 2
260 2
280 3
300 2
320 2
340 1
360 0
U-RIB:ASN-CA
20 0
40 0
60 1
80 2
100 2
120 2
140 1
160 1
180 0
200 0
220 1
240 1
260 2
280 2
300 2
320 2
340 1
360 0
C-RIB:GLU-S2
20 0
40 0
60 5
80 7
100 7
120 7
140 5
160 4
180 1
200 0
220 0
240 5
260 7
280 7
300 7
320 6

340 4
360 1
QUO-RIB:ASN-CA
20 0
40 0
60 0
80 0
100 0
120 0
140 0
160 0
180 0
200 0
220 0
240 0
260 0
280 0
300 0
320 0
340 0
360 0
C31-MY:LEU-S2
20 0
40 0
60 0
80 0
100 0
120 0
140 0
160 0
180 0
200 0
220 0
240 0
260 0
280 0
300 0
320 0
340 0
360 0
G-R6:PHE-S2
20 0
40 0
60 4
80 5
100 5
120 5
140 4
160 3
180 0
200 0
220 2
240 4
260 5
280 5
300 5
320 0
340 3

360 0
A-R6:PHE-S2
20 0
40 1
60 0
80 4
100 4
120 4
140 3
160 0
180 0
200 0
220 1
240 3
260 4
280 4
300 4
320 0
340 2
360 0
DA-M6:HIS-S1
20 0
40 0
60 0
80 0
100 0
120 0
140 0
160 0
180 0
200 0
220 0
240 0
260 0
280 0
300 0
320 0
340 0
360 0
U-RIB:GLN-S1
20 0
40 0
60 1
80 2
100 2
120 2
140 1
160 1
180 0
200 0
220 0
240 1
260 2
280 2
300 2
320 1
340 1
360 0

IU-MY:ILE-CA
20 0
40 0
60 0
80 0
100 0
120 0
140 0
160 0
180 0
200 0
220 0
240 0
260 0
280 0
300 0
320 0
340 0
360 0
A-RIB:PRO-S1
20 0
40 2
60 3
80 5
100 5
120 5
140 4
160 3
180 1
200 0
220 0
240 3
260 5
280 5
300 5
320 4
340 3
360 1
DA-M6:MET-S2
20 0
40 0
60 0
80 0
100 0
120 0
140 0
160 0
180 0
200 0
220 0
240 0
260 0
280 0
300 0
320 0
340 0
360 0
G-R6:LEU-S2

20 1
40 4
60 9
80 12
100 13
120 12
140 9
160 7
180 0
200 0
220 5
240 9
260 12
280 13
300 12
320 10
340 0
360 0
5BU-P:SER-S1
20 0
40 0
60 0
80 0
100 0
120 0
140 0
160 0
180 0
200 0
220 0
240 0
260 0
280 0
300 0
320 0
340 0
360 0
G-R6:ARG-CA
20 0
40 3
60 7
80 9
100 10
120 9
140 7
160 5
180 2
200 0
220 3
240 7
260 9
280 10
300 9
320 8
340 5
360 0
IU-P:LEU-S1
20 0

40 0
60 0
80 0
100 0
120 0
140 0
160 0
180 0
200 0
220 0
240 0
260 0
280 0
300 0
320 0
340 0
360 0
G-P:ASP-S1
20 0
40 3
60 6
80 8
100 9
120 8
140 6
160 5
180 2
200 0
220 3
240 6
260 8
280 9
300 8
320 7
340 5
360 2
G-R5:PRO-S1
20 0
40 2
60 4
80 6
100 7
120 6
140 5
160 3
180 0
200 0
220 2
240 4
260 6
280 7
300 6
320 5
340 4
360 0
A-R5:VAL-S1
20 0
40 3

60 6
80 8
100 9
120 8
140 6
160 4
180 0
200 0
220 3
240 6
260 8
280 9
300 8
320 7
340 5
360 0
G-RIB:TRP-S2
20 0
40 0
60 1
80 1
100 2
120 1
140 1
160 1
180 0
200 0
220 0
240 1
260 1
280 2
300 1
320 1
340 1
360 0
FHU-P:PRO-S1
20 0
40 0
60 0
80 0
100 0
120 0
140 0
160 0
180 0
200 0
220 0
240 0
260 0
280 0
300 0
320 0
340 0
360 0
C31-RIB:PHE-CA
20 0
40 0
60 0

80 0
100 0
120 0
140 0
160 0
180 0
200 0
220 0
240 0
260 0
280 0
300 0
320 0
340 0
360 0
DA-M5:HIS-CA
20 0
40 0
60 0
80 0
100 0
120 0
140 0
160 0
180 0
200 0
220 0
240 0
260 0
280 0
300 0
320 0
340 0
360 0
A-RIB:GLU-S1
20 0
40 3
60 6
80 9
100 9
120 8
140 7
160 5
180 2
200 0
220 0
240 6
260 9
280 9
300 9
320 7
340 5
360 2
H2U-RIB:LEU-S2
20 0
40 0
60 0
80 0

100 0
120 0
140 0
160 0
180 0
200 0
220 0
240 0
260 0
280 0
300 0
320 0
340 0
360 0
U31-P:MET-CA
20 0
40 0
60 0
80 0
100 0
120 0
140 0
160 0
180 0
200 0
220 0
240 0
260 0
280 0
300 0
320 0
340 0
360 0
C31-MY:LEU-S1
20 0
40 0
60 0
80 0
100 0
120 0
140 0
160 0
180 0
200 0
220 0
240 0
260 0
280 0
300 0
320 0
340 0
360 0
A-P:TRP-S1
20 0
40 0
60 0
80 1
100 1

120   1
140   0
160   0
180   0
200   0
220   0
240   0
260   0
280   1
300   1
320   1
340   0
360   0
A-P:GLU-CA
20   0
40   3
60   6
80   9
100   9
120   8
140   7
160   5
180   0
200   0
220   3
240   6
260   9
280   9
300   9
320   7
340   0
360   0
G-R5:TRP-S1
20   0
40   0
60   0
80   1
100   2
120   1
140   0
160   0
180   0
200   0
220   0
240   1
260   0
280   2
300   1
320   0
340   1
360   0
U34-MY:TYR-S2
20   0
40   0
60   0
80   0
100   0
120   0

140 0
160 0
180 0
200 0
220 0
240 0
260 0
280 0
300 0
320 0
340 0
360 0
C-P:SER-CA
20 0
40 1
60 3
80 5
100 5
120 4
140 3
160 2
180 1
200 0
220 2
240 3
260 5
280 5
300 4
320 4
340 3
360 1
U-RIB:PHE-S1
20 0
40 0
60 1
80 2
100 2
120 2
140 1
160 1
180 0
200 0
220 0
240 1
260 2
280 2
300 2
320 2
340 1
360 0
C-RIB:TRP-S1
20 0
40 0
60 0
80 1
100 1
120 1
140 0

160 0
180 0
200 0
220 0
240 0
260 1
280 1
300 0
320 0
340 0
360 0
A-R6:GLU-CA
20 1
40 3
60 0
80 9
100 9
120 8
140 7
160 5
180 0
200 0
220 3
240 6
260 9
280 9
300 9
320 7
340 0
360 0
U-Y:GLU-CA
20 0
40 1
60 3
80 5
100 5
120 4
140 4
160 3
180 1
200 0
220 2
240 3
260 5
280 5
300 5
320 4
340 0
360 0
H2U-P:PHE-S2
20 0
40 0
60 0
80 0
100 0
120 0
140 0
160 0

180 0
200 0
220 0
240 0
260 0
280 0
300 0
320 0
340 0
360 0
DA-M5:HIS-S2
20 0
40 0
60 0
80 0
100 0
120 0
140 0
160 0
180 0
200 0
220 0
240 0
260 0
280 0
300 0
320 0
340 0
360 0
C-P:GLN-S2
20 0
40 1
60 2
80 3
100 3
120 3
140 2
160 1
180 0
200 0
220 1
240 2
260 3
280 3
300 3
320 2
340 1
360 0
IU-P:HIS-CA
20 0
40 0
60 0
80 0
100 0
120 0
140 0
160 0
180 0

200 0
220 0
240 0
260 0
280 0
300 0
320 0
340 0
360 0
A-R6:GLU-S1
20 0
40 3
60 6
80 9
100 9
120 8
140 7
160 5
180 2
200 0
220 3
240 6
260 9
280 9
300 9
320 7
340 5
360 0
U34-RIB:GLY-CA
20 0
40 0
60 0
80 0
100 0
120 0
140 0
160 0
180 0
200 0
220 0
240 0
260 0
280 0
300 0
320 0
340 0
360 0
G-RIB:VAL-CA
20 0
40 0
60 7
80 10
100 11
120 10
140 8
160 6
180 0
200 0

220 4
240 7
260 10
280 11
300 10
320 8
340 6
360 2
A-RIB:ALA-S1
20 0
40 3
60 7
80 9
100 10
120 9
140 7
160 5
180 0
200 0
220 3
240 7
260 9
280 10
300 9
320 7
340 5
360 2
A-P:PRO-S1
20 0
40 2
60 3
80 5
100 5
120 5
140 4
160 3
180 0
200 0
220 2
240 3
260 5
280 5
300 5
320 4
340 3
360 1
C-P:ARG-S2
20 0
40 2
60 4
80 6
100 6
120 5
140 4
160 3
180 1
200 0
220 2

240   4
260   6
280   6
300   6
320   5
340   3
360   1
G-P:GLN-S1
20   0
40   1
60   3
80   5
100   5
120   4
140   4
160   3
180   1
200   0
220   2
240   3
260   5
280   5
300   5
320   4
340   3
360   1
U-RIB:GLU-S1
20   0
40   1
60   3
80   5
100   5
120   4
140   3
160   3
180   0
200   0
220   0
240   3
260   5
280   5
300   5
320   4
340   3
360   1
C-Y:CYS-CA
20   0
40   0
60   0
80   0
100   0
120   0
140   0
160   0
180   0
200   0
220   0
240   0

260 0
280 0
300 0
320 0
340 0
360 0
QUO-RIB:LEU-CA
20 0
40 0
60 0
80 0
100 0
120 0
140 0
160 0
180 0
200 0
220 0
240 0
260 0
280 0
300 0
320 0
340 0
360 0
U-P:LEU-CA
20 0
40 2
60 4
80 5
100 5
120 5
140 4
160 0
180 1
200 0
220 2
240 4
260 5
280 5
300 5
320 4
340 3
360 0
G-R5:VAL-CA
20 0
40 4
60 7
80 10
100 11
120 10
140 8
160 6
180 2
200 1
220 4
240 7
260 10

280 11
300 10
320 8
340 6
360 0
QUO-M5:PHE-CA
20 0
40 0
60 0
80 0
100 0
120 0
140 0
160 0
180 0
200 0
220 0
240 0
260 0
280 0
300 0
320 0
340 0
360 0
C31-RIB:TYR-S1
20 0
40 0
60 0
80 0
100 0
120 0
140 0
160 0
180 0
200 0
220 0
240 0
260 0
280 0
300 0
320 0
340 0
360 0
FHU-RIB:THR-CA
20 0
40 0
60 0
80 0
100 0
120 0
140 0
160 0
180 0
200 0
220 0
240 0
260 0
280 0

300 0
320 0
340 0
360 0
QUO-M6:ARG-S2
20 0
40 0
60 0
80 0
100 0
120 0
140 0
160 0
180 0
200 0
220 0
240 0
260 0
280 0
300 0
320 0
340 0
360 0
QUO-P:LEU-S1
20 0
40 0
60 0
80 0
100 0
120 0
140 0
160 0
180 0
200 0
220 0
240 0
260 0
280 0
300 0
320 0
340 0
360 0
C-P:LYS-S1
20 0
40 2
60 4
80 6
100 6
120 6
140 4
160 3
180 1
200 0
220 2
240 4
260 6
280 6
300 6

320 5
340 3
360 1
FMU-RIB:PHE-CA
20 0
40 0
60 0
80 0
100 0
120 0
140 0
160 0
180 0
200 0
220 0
240 0
260 0
280 0
300 0
320 0
340 0
360 0
C-Y:GLU-S2
20 0
40 2
60 5
80 7
100 7
120 7
140 5
160 4
180 1
200 0
220 2
240 5
260 7
280 7
300 7
320 6
340 4
360 1
DA-M5:SER-CA
20 0
40 0
60 0
80 0
100 0
120 0
140 0
160 0
180 0
200 0
220 0
240 0
260 0
280 0
300 0
320 0

340 0
360 0
U-P:LEU-S1
20 0
40 2
60 4
80 5
100 5
120 5
140 4
160 3
180 0
200 0
220 2
240 4
260 5
280 5
300 5
320 4
340 3
360 0
IU-MY:THR-CA
20 0
40 0
60 0
80 0
100 0
120 0
140 0
160 0
180 0
200 0
220 0
240 0
260 0
280 0
300 0
320 0
340 0
360 0
C-RIB:TYR-S2
20 0
40 1
60 2
80 2
100 3
120 2
140 2
160 1
180 0
200 0
220 1
240 2
260 2
280 3
300 2
320 2
340 1

360 0
IU-MY:GLU-S2
20 0
40 0
60 0
80 0
100 0
120 0
140 0
160 0
180 0
200 0
220 0
240 0
260 0
280 0
300 0
320 0
340 0
360 0
IU-RIB:ALA-CA
20 0
40 0
60 0
80 0
100 0
120 0
140 0
160 0
180 0
200 0
220 0
240 0
260 0
280 0
300 0
320 0
340 0
360 0
G-RIB:GLU-S2
20 1
40 4
60 8
80 11
100 12
120 11
140 8
160 6
180 2
200 0
220 4
240 8
260 11
280 12
300 11
320 9
340 7
360 2

A-RIB:GLN-CA
20 0
40 0
60 3
80 4
100 4
120 3
140 3
160 2
180 0
200 0
220 1
240 3
260 4
280 4
300 4
320 3
340 2
360 1
FHU-MY:GLN-S2
20 0
40 0
60 0
80 0
100 0
120 0
140 0
160 0
180 0
200 0
220 0
240 0
260 0
280 0
300 0
320 0
340 0
360 0
FHU-P:VAL-CA
20 0
40 0
60 0
80 0
100 0
120 0
140 0
160 0
180 0
200 0
220 0
240 0
260 0
280 0
300 0
320 0
340 0
360 0
G-P:CYS-CA

20   0
40   0
60   0
80   0
100   1
120   1
140   1
160   0
180   0
200   0
220   0
240   0
260   0
280   0
300   0
320   1
340   0
360   0
U-P:VAL-CA
20   0
40   0
60   3
80   4
100   4
120   4
140   3
160   2
180   1
200   0
220   1
240   3
260   4
280   4
300   4
320   3
340   2
360   1
FMU-MY:ASP-CA
20   0
40   0
60   0
80   0
100   0
120   0
140   0
160   0
180   0
200   0
220   0
240   0
260   0
280   0
300   0
320   0
340   0
360   0
G-R6:TRP-S1
20   0

40 0
60 1
80 1
100 2
120 1
140 1
160 0
180 0
200 0
220 0
240 1
260 1
280 2
300 1
320 1
340 1
360 0
G-R6:TRP-CA
20 0
40 0
60 1
80 1
100 0
120 1
140 1
160 0
180 0
200 0
220 0
240 0
260 1
280 2
300 1
320 1
340 0
360 0
FMU-P:ASP-S1
20 0
40 0
60 0
80 0
100 0
120 0
140 0
160 0
180 0
200 0
220 0
240 0
260 0
280 0
300 0
320 0
340 0
360 0
A-RIB:LEU-S1
20 0
40 3

60 7
80 10
100 10
120 9
140 7
160 5
180 2
200 0
220 4
240 0
260 10
280 10
300 9
320 8
340 6
360 2
H2U-P:THR-CA
20 0
40 0
60 0
80 0
100 0
120 0
140 0
160 0
180 0
200 0
220 0
240 0
260 0
280 0
300 0
320 0
340 0
360 0
U31-MY:ALA-CA
20 0
40 0
60 0
80 0
100 0
120 0
140 0
160 0
180 0
200 0
220 0
240 0
260 0
280 0
300 0
320 0
340 0
360 0
U-P:TYR-S2
20 0
40 0
60 1

80 2
100 2
120 1
140 1
160 1
180 0
200 0
220 0
240 1
260 2
280 2
300 2
320 1
340 1
360 0
C31-RIB:SER-CA
20 0
40 0
60 0
80 0
100 0
120 0
140 0
160 0
180 0
200 0
220 0
240 0
260 0
280 0
300 0
320 0
340 0
360 0
U31-P:ALA-S1
20 0
40 0
60 0
80 0
100 0
120 0
140 0
160 0
180 0
200 0
220 0
240 0
260 0
280 0
300 0
320 0
340 0
360 0
U-Y:PHE-S2
20 0
40 0
60 1
80 2

100   2
120   2
140   1
160   0
180   0
200   0
220   0
240   1
260   2
280   2
300   2
320   2
340   0
360   0
DA-M6:GLN-S1
20   0
40   0
60   0
80   0
100   0
120   0
140   0
160   0
180   0
200   0
220   0
240   0
260   0
280   0
300   0
320   0
340   0
360   0
G-RIB:TYR-CA
20   0
40   0
60   3
80   4
100   5
120   0
140   3
160   2
180   0
200   0
220   1
240   0
260   4
280   5
300   4
320   0
340   2
360   0
QUO-M5:PHE-S2
20   0
40   0
60   0
80   0
100   0

120   0
140   0
160   0
180   0
200   0
220   0
240   0
260   0
280   0
300   0
320   0
340   0
360   0
FHU-RIB:LEU-S2
20   0
40   0
60   0
80   0
100   0
120   0
140   0
160   0
180   0
200   0
220   0
240   0
260   0
280   0
300   0
320   0
340   0
360   0
H2U-RIB:PHE-S1
20   0
40   0
60   0
80   0
100   0
120   0
140   0
160   0
180   0
200   0
220   0
240   0
260   0
280   0
300   0
320   0
340   0
360   0
G-R6:VAL-S1
20   0
40   4
60   0
80   10
100   11
120   10

140 0
160 0
180 2
200 0
220 4
240 7
260 10
280 11
300 10
320 8
340 6
360 2
5BU-MY:PRO-CA
20 0
40 0
60 0
80 0
100 0
120 0
140 0
160 0
180 0
200 0
220 0
240 0
260 0
280 0
300 0
320 0
340 0
360 0
H2U-RIB:GLU-CA
20 0
40 0
60 0
80 0
100 0
120 0
140 0
160 0
180 0
200 0
220 0
240 0
260 0
280 0
300 0
320 0
340 0
360 0
H2U-MY:LYS-S1
20 0
40 0
60 0
80 0
100 0
120 0
140 0

160   0
180   0
200   0
220   0
240   0
260   0
280   0
300   0
320   0
340   0
360   0
U-P:TRP-S1
20   0
40   0
60   0
80   0
100   0
120   0
140   0
160   0
180   0
200   0
220   0
240   0
260   0
280   0
300   0
320   0
340   0
360   0
FHU-RIB:SER-S1
20   0
40   0
60   0
80   0
100   0
120   0
140   0
160   0
180   0
200   0
220   0
240   0
260   0
280   0
300   0
320   0
340   0
360   0
G-R5:THR-S1
20   0
40   2
60   5
80   7
100   7
120   6
140   5
160   4

180 0
200 0
220 2
240 5
260 7
280 7
300 7
320 6
340 4
360 0
G-P:HIS-S2
20 0
40 1
60 2
80 3
100 3
120 3
140 2
160 2
180 0
200 0
220 1
240 2
260 3
280 3
300 3
320 2
340 2
360 0
H2U-P:PHE-S1
20 0
40 0
60 0
80 0
100 0
120 0
140 0
160 0
180 0
200 0
220 0
240 0
260 0
280 0
300 0
320 0
340 0
360 0
U-P:HIS-S1
20 0
40 0
60 1
80 1
100 1
120 1
140 1
160 0
180 0

200 0
220 0
240 1
260 1
280 1
300 1
320 1
340 0
360 0
C31-P:THR-S1
20 0
40 0
60 0
80 0
100 0
120 0
140 0
160 0
180 0
200 0
220 0
240 0
260 0
280 0
300 0
320 0
340 0
360 0
FHU-P:TYR-S2
20 0
40 0
60 0
80 0
100 0
120 0
140 0
160 0
180 0
200 0
220 0
240 0
260 0
280 0
300 0
320 0
340 0
360 0
I-RIB:ALA-S1
20 0
40 0
60 0
80 0
100 0
120 0
140 0
160 0
180 0
200 0

220 0
240 0
260 0
280 0
300 0
320 0
340 0
360 0
A-R5:ILE-S1
20 0
40 2
60 4
80 6
100 0
120 6
140 4
160 0
180 0
200 0
220 0
240 4
260 6
280 0
300 6
320 5
340 3
360 0
A-R6:LEU-S1
20 0
40 3
60 7
80 10
100 10
120 9
140 7
160 5
180 0
200 0
220 4
240 7
260 10
280 10
300 9
320 8
340 6
360 2
C31-MY:GLU-CA
20 0
40 0
60 0
80 0
100 0
120 0
140 0
160 0
180 0
200 0
220 0

240   0
260   0
280   0
300   0
320   0
340   0
360   0
U-RIB:SER-CA
20   0
40   1
60   2
80   3
100   3
120   3
140   2
160   2
180   0
200   0
220   1
240   2
260   3
280   3
300   3
320   2
340   2
360   0
IU-MY:ARG-S1
20   0
40   0
60   0
80   0
100   0
120   0
140   0
160   0
180   0
200   0
220   0
240   0
260   0
280   0
300   0
320   0
340   0
360   0
U31-MY:THR-S1
20   0
40   0
60   0
80   0
100   0
120   0
140   0
160   0
180   0
200   0
220   0
240   0

260   0
280   0
300   0
320   0
340   0
360   0
U-Y:LEU-CA
20   0
40   2
60   4
80   5
100   5
120   5
140   4
160   0
180   1
200   0
220   0
240   4
260   5
280   5
300   5
320   4
340   3
360   0
U-P:GLU-CA
20   0
40   1
60   0
80   5
100   5
120   4
140   4
160   3
180   1
200   0
220   2
240   3
260   5
280   5
300   5
320   4
340   3
360   0
U31-MY:MET-CA
20   0
40   0
60   0
80   0
100   0
120   0
140   0
160   0
180   0
200   0
220   0
240   0
260   0

280 0
300 0
320 0
340 0
360 0
G-P:HIS-CA
20 0
40 1
60 2
80 3
100 3
120 3
140 2
160 2
180 0
200 0
220 0
240 2
260 3
280 3
300 3
320 2
340 2
360 0
G-R5:ASN-CA
20 0
40 2
60 4
80 5
100 6
120 5
140 4
160 3
180 1
200 0
220 2
240 4
260 5
280 0
300 5
320 4
340 3
360 1
FMU-MY:VAL-CA
20 0
40 0
60 0
80 0
100 0
120 0
140 0
160 0
180 0
200 0
220 0
240 0
260 0
280 0

300 0
320 0
340 0
360 0
A-R6:LEU-S2
20 0
40 3
60 7
80 9
100 10
120 9
140 7
160 5
180 2
200 0
220 4
240 7
260 10
280 10
300 9
320 8
340 6
360 0
IU-MY:LYS-CA
20 0
40 0
60 0
80 0
100 0
120 0
140 0
160 0
180 0
200 0
220 0
240 0
260 0
280 0
300 0
320 0
340 0
360 0
G-R6:PHE-CA
20 0
40 2
60 4
80 5
100 0
120 5
140 4
160 0
180 0
200 0
220 0
240 4
260 5
280 5
300 5

320 0
340 0
360 0
FMU-MY:GLU-S1
20 0
40 0
60 0
80 0
100 0
120 0
140 0
160 0
180 0
200 0
220 0
240 0
260 0
280 0
300 0
320 0
340 0
360 0
A-RIB:TRP-S2
20 0
40 0
60 1
80 0
100 1
120 1
140 1
160 0
180 0
200 0
220 0
240 1
260 1
280 1
300 1
320 1
340 0
360 0
A-P:ASP-CA
20 0
40 0
60 5
80 6
100 7
120 6
140 5
160 4
180 1
200 0
220 2
240 5
260 7
280 7
300 6
320 5

340 4
360 1
I-RIB:GLY-CA
20 0
40 0
60 0
80 0
100 0
120 0
140 0
160 0
180 0
200 0
220 0
240 0
260 0
280 0
300 0
320 0
340 0
360 0
C-P:PHE-S2
20 0
40 0
60 2
80 3
100 3
120 3
140 2
160 2
180 0
200 0
220 1
240 2
260 3
280 3
300 3
320 2
340 2
360 0
C-P:THR-S1
20 0
40 1
60 3
80 4
100 4
120 4
140 3
160 2
180 1
200 0
220 1
240 3
260 4
280 4
300 4
320 3
340 2

360 1
U-RIB:ARG-S2
20 0
40 1
60 3
80 4
100 4
120 4
140 3
160 2
180 0
200 0
220 1
240 3
260 4
280 4
300 4
320 3
340 2
360 1
IU-MY:THR-S1
20 0
40 0
60 0
80 0
100 0
120 0
140 0
160 0
180 0
200 0
220 0
240 0
260 0
280 0
300 0
320 0
340 0
360 0
G-P:ALA-S1
20 0
40 4
60 8
80 11
100 12
120 11
140 9
160 6
180 2
200 1
220 4
240 8
260 11
280 12
300 11
320 9
340 7
360 2

QUO-M5:ASP-S2
20 0
40 0
60 0
80 0
100 0
120 0
140 0
160 0
180 0
200 0
220 0
240 0
260 0
280 0
300 0
320 0
340 0
360 0
G-R5:TYR-S2
20 0
40 1
60 3
80 4
100 5
120 4
140 0
160 0
180 0
200 0
220 0
240 3
260 4
280 5
300 4
320 3
340 2
360 1
U31-RIB:ASP-S2
20 0
40 0
60 0
80 0
100 0
120 0
140 0
160 0
180 0
200 0
220 0
240 0
260 0
280 0
300 0
320 0
340 0
360 0
U31-RIB:MET-CA

20 0
40 0
60 0
80 0
100 0
120 0
140 0
160 0
180 0
200 0
220 0
240 0
260 0
280 0
300 0
320 0
340 0
360 0
C-Y:PRO-S1
20 0
40 1
60 3
80 4
100 4
120 4
140 3
160 2
180 0
200 0
220 1
240 3
260 4
280 4
300 4
320 3
340 2
360 0
FHU-MY:CYS-S1
20 0
40 0
60 0
80 0
100 0
120 0
140 0
160 0
180 0
200 0
220 0
240 0
260 0
280 0
300 0
320 0
340 0
360 0
A-RIB:GLN-S1
20 0

40 1
60 3
80 4
100 4
120 3
140 3
160 2
180 0
200 0
220 0
240 3
260 4
280 4
300 4
320 3
340 2
360 1
G-RIB:THR-CA
20 0
40 2
60 5
80 7
100 7
120 6
140 5
160 4
180 1
200 0
220 2
240 5
260 7
280 7
300 7
320 6
340 4
360 1
FMU-MY:ALA-CA
20 0
40 0
60 0
80 0
100 0
120 0
140 0
160 0
180 0
200 0
220 0
240 0
260 0
280 0
300 0
320 0
340 0
360 0
FMU-MY:HIS-S1
20 0
40 0

60 0
80 0
100 0
120 0
140 0
160 0
180 0
200 0
220 0
240 0
260 0
280 0
300 0
320 0
340 0
360 0
C31-MY:ALA-CA
20 0
40 0
60 0
80 0
100 0
120 0
140 0
160 0
180 0
200 0
220 0
240 0
260 0
280 0
300 0
320 0
340 0
360 0
A-R5:PHE-CA
20 0
40 0
60 3
80 0
100 4
120 4
140 3
160 0
180 0
200 0
220 1
240 3
260 4
280 4
300 4
320 0
340 0
360 0
U-RIB:VAL-S1
20 0
40 1
60 3

80   4
100   4
120   4
140   3
160   2
180   1
200   0
220   0
240   3
260   4
280   4
300   4
320   3
340   2
360   0
U-RIB:ASP-S1
20   0
40   1
60   2
80   3
100   4
120   3
140   3
160   2
180   0
200   0
220   1
240   2
260   3
280   4
300   3
320   3
340   2
360   0
FMU-MY:GLU-S2
20   0
40   0
60   0
80   0
100   0
120   0
140   0
160   0
180   0
200   0
220   0
240   0
260   0
280   0
300   0
320   0
340   0
360   0
C-RIB:HIS-CA
20   0
40   0
60   1
80   2

100 2
120 2
140 0
160 1
180 0
200 0
220 0
240 1
260 2
280 2
300 2
320 1
340 1
360 0
FHU-MY:LEU-CA
20 0
40 0
60 0
80 0
100 0
120 0
140 0
160 0
180 0
200 0
220 0
240 0
260 0
280 0
300 0
320 0
340 0
360 0
C-RIB:ALA-S1
20 0
40 2
60 5
80 7
100 8
120 7
140 5
160 4
180 1
200 0
220 3
240 5
260 7
280 8
300 7
320 6
340 4
360 1
GTP-M5:ALA-S1
20 0
40 0
60 0
80 0
100 0

120   0
140   0
160   0
180   0
200   0
220   0
240   0
260   0
280   0
300   0
320   0
340   0
360   0
G-P:GLU-S2
20   0
40   4
60   8
80   11
100   12
120   10
140   8
160   6
180   2
200   0
220   4
240   8
260   11
280   12
300   11
320   9
340   6
360   2
C-RIB:LYS-S1
20   0
40   2
60   4
80   6
100   6
120   6
140   5
160   3
180   1
200   0
220   2
240   4
260   6
280   6
300   6
320   5
340   3
360   1
U-RIB:TYR-S2
20   0
40   0
60   0
80   2
100   2
120   1

140 1
160 1
180 0
200 0
220 0
240 1
260 2
280 2
300 2
320 1
340 1
360 0
FMU-RIB:GLU-S2
20 0
40 0
60 0
80 0
100 0
120 0
140 0
160 0
180 0
200 0
220 0
240 0
260 0
280 0
300 0
320 0
340 0
360 0
FMU-RIB:PHE-S2
20 0
40 0
60 0
80 0
100 0
120 0
140 0
160 0
180 0
200 0
220 0
240 0
260 0
280 0
300 0
320 0
340 0
360 0
IU-RIB:ARG-CA
20 0
40 0
60 0
80 0
100 0
120 0
140 0

160 0
180 0
200 0
220 0
240 0
260 0
280 0
300 0
320 0
340 0
360 0
QUO-M6:GLN-S1
20 0
40 0
60 0
80 0
100 0
120 0
140 0
160 0
180 0
200 0
220 0
240 0
260 0
280 0
300 0
320 0
340 0
360 0
G-R5:ALA-S1
20 0
40 4
60 8
80 11
100 12
120 11
140 9
160 7
180 2
200 1
220 4
240 8
260 11
280 12
300 11
320 10
340 7
360 2
FMU-P:VAL-S1
20 0
40 0
60 0
80 0
100 0
120 0
140 0
160 0

180 0
200 0
220 0
240 0
260 0
280 0
300 0
320 0
340 0
360 0
U-P:ARG-CA
20 0
40 1
60 3
80 4
100 4
120 4
140 3
160 2
180 0
200 0
220 1
240 3
260 4
280 4
300 4
320 3
340 2
360 1
FMU-P:PHE-S1
20 0
40 0
60 0
80 0
100 0
120 0
140 0
160 0
180 0
200 0
220 0
240 0
260 0
280 0
300 0
320 0
340 0
360 0
A-RIB:GLU-S2
20 0
40 3
60 6
80 9
100 9
120 8
140 7
160 5
180 2

200 0
220 0
240 6
260 9
280 9
300 9
320 7
340 5
360 2
U-RIB:ALA-S1
20 0
40 2
60 3
80 5
100 5
120 5
140 4
160 3
180 0
200 0
220 2
240 3
260 5
280 5
300 5
320 4
340 3
360 1
C-RIB:ASP-S1
20 0
40 0
60 4
80 5
100 6
120 5
140 4
160 3
180 1
200 0
220 2
240 4
260 5
280 6
300 5
320 4
340 3
360 1
A-R6:ASN-S1
20 0
40 0
60 3
80 4
100 4
120 4
140 3
160 2
180 0
200 0

220 1
240 3
260 4
280 4
300 4
320 3
340 2
360 1
OMC-P:LYS-S1
20 0
40 0
60 0
80 0
100 0
120 0
140 0
160 0
180 0
200 0
220 0
240 0
260 0
280 0
300 0
320 0
340 0
360 0
H2U-MY:PHE-S1
20 0
40 0
60 0
80 0
100 0
120 0
140 0
160 0
180 0
200 0
220 0
240 0
260 0
280 0
300 0
320 0
340 0
360 0
U31-MY:PHE-CA
20 0
40 0
60 0
80 0
100 0
120 0
140 0
160 0
180 0
200 0
220 0

240 0
260 0
280 0
300 0
320 0
340 0
360 0
A-RIB:ASN-S2
20 0
40 1
60 3
80 4
100 4
120 4
140 3
160 2
180 1
200 0
220 1
240 3
260 4
280 4
300 4
320 3
340 2
360 1
G-P:ASN-CA
20 0
40 2
60 4
80 5
100 6
120 5
140 4
160 3
180 1
200 0
220 2
240 4
260 5
280 6
300 5
320 4
340 3
360 1
H2U-P:GLU-CA
20 0
40 0
60 0
80 0
100 0
120 0
140 0
160 0
180 0
200 0
220 0
240 0

260 0
280 0
300 0
320 0
340 0
360 0
U31-RIB:ASP-S1
20 0
40 0
60 0
80 0
100 0
120 0
140 0
160 0
180 0
200 0
220 0
240 0
260 0
280 0
300 0
320 0
340 0
360 0
A-P:MET-S1
20 0
40 0
60 2
80 2
100 3
120 2
140 2
160 1
180 0
200 0
220 0
240 2
260 2
280 3
300 2
320 2
340 0
360 0
U-P:ARG-S1
20 0
40 1
60 3
80 4
100 4
120 4
140 3
160 2
180 0
200 0
220 1
240 3
260 4

280 4
300 4
320 3
340 2
360 1
A-RIB:VAL-CA
20 0
40 3
60 6
80 8
100 9
120 8
140 6
160 4
180 1
200 0
220 0
240 6
260 8
280 9
300 8
320 7
340 5
360 2
C-Y:HIS-S2
20 0
40 0
60 1
80 2
100 2
120 2
140 1
160 1
180 0
200 0
220 0
240 1
260 2
280 2
300 2
320 0
340 1
360 0
C-Y:MET-S2
20 0
40 0
60 1
80 2
100 2
120 2
140 0
160 0
180 0
200 0
220 0
240 1
260 2
280 2

300 2
320 0
340 0
360 0
A-R5:TYR-CA
20 0
40 1
60 2
80 3
100 4
120 3
140 2
160 0
180 0
200 0
220 1
240 2
260 3
280 4
300 3
320 3
340 2
360 0
U-Y:GLN-S1
20 0
40 0
60 1
80 2
100 2
120 2
140 1
160 1
180 0
200 0
220 0
240 1
260 2
280 2
300 2
320 1
340 0
360 0
G-RIB:HIS-S1
20 0
40 1
60 2
80 3
100 3
120 3
140 2
160 2
180 0
200 0
220 1
240 2
260 3
280 3
300 3

320 2
340 2
360 0
C31-MY:ASP-CA
20 0
40 0
60 0
80 0
100 0
120 0
140 0
160 0
180 0
200 0
220 0
240 0
260 0
280 0
300 0
320 0
340 0
360 0
FMU-P:ALA-S1
20 0
40 0
60 0
80 0
100 0
120 0
140 0
160 0
180 0
200 0
220 0
240 0
260 0
280 0
300 0
320 0
340 0
360 0
U31-MY:GLN-S1
20 0
40 0
60 0
80 0
100 0
120 0
140 0
160 0
180 0
200 0
220 0
240 0
260 0
280 0
300 0
320 0

340 0
360 0
FHU-MY:ARG-CA
20 0
40 0
60 0
80 0
100 0
120 0
140 0
160 0
180 0
200 0
220 0
240 0
260 0
280 0
300 0
320 0
340 0
360 0
5BU-P:ILE-CA
20 0
40 0
60 0
80 0
100 0
120 0
140 0
160 0
180 0
200 0
220 0
240 0
260 0
280 0
300 0
320 0
340 0
360 0
A-R5:MET-S2
20 0
40 1
60 2
80 2
100 3
120 2
140 2
160 1
180 0
200 0
220 1
240 2
260 2
280 0
300 2
320 2
340 0

360 0
A-R5:TRP-S2
20 0
40 0
60 1
80 0
100 1
120 1
140 0
160 0
180 0
200 0
220 0
240 1
260 1
280 1
300 1
320 1
340 0
360 0
C-RIB:LEU-CA
20 0
40 3
60 5
80 7
100 8
120 7
140 6
160 4
180 1
200 0
220 0
240 5
260 8
280 8
300 7
320 6
340 4
360 0
FMU-MY:ASP-S1
20 0
40 0
60 0
80 0
100 0
120 0
140 0
160 0
180 0
200 0
220 0
240 0
260 0
280 0
300 0
320 0
340 0
360 0

U34-P:GLU-CA
20 0
40 0
60 0
80 0
100 0
120 0
140 0
160 0
180 0
200 0
220 0
240 0
260 0
280 0
300 0
320 0
340 0
360 0
5BU-P:ARG-S1
20 0
40 0
60 0
80 0
100 0
120 0
140 0
160 0
180 0
200 0
220 0
240 0
260 0
280 0
300 0
320 0
340 0
360 0
DA-M5:LEU-S2
20 0
40 0
60 0
80 0
100 0
120 0
140 0
160 0
180 0
200 0
220 0
240 0
260 0
280 0
300 0
320 0
340 0
360 0
QUO-M5:LEU-CA

20 0
40 0
60 0
80 0
100 0
120 0
140 0
160 0
180 0
200 0
220 0
240 0
260 0
280 0
300 0
320 0
340 0
360 0
FMU-MY:MET-S2
20 0
40 0
60 0
80 0
100 0
120 0
140 0
160 0
180 0
200 0
220 0
240 0
260 0
280 0
300 0
320 0
340 0
360 0
G-R6:ASP-S1
20 0
40 3
60 6
80 8
100 9
120 8
140 6
160 5
180 2
200 0
220 3
240 6
260 8
280 9
300 8
320 7
340 5
360 2
DA-M6:GLU-S1
20 0

40 0
60 0
80 0
100 0
120 0
140 0
160 0
180 0
200 0
220 0
240 0
260 0
280 0
300 0
320 0
340 0
360 0
H2U-MY:GLN-CA
20 0
40 0
60 0
80 0
100 0
120 0
140 0
160 0
180 0
200 0
220 0
240 0
260 0
280 0
300 0
320 0
340 0
360 0
H2U-MY:ARG-CA
20 0
40 0
60 0
80 0
100 0
120 0
140 0
160 0
180 0
200 0
220 0
240 0
260 0
280 0
300 0
320 0
340 0
360 0
C-RIB:CYS-CA
20 0
40 0

60 0
80 1
100 1
120 0
140 0
160 0
180 0
200 0
220 0
240 0
260 1
280 1
300 0
320 0
340 0
360 0
A-R5:ASN-S1
20 0
40 1
60 3
80 4
100 4
120 4
140 3
160 2
180 0
200 0
220 1
240 3
260 4
280 4
300 4
320 3
340 2
360 1
U31-P:ASN-S2
20 0
40 0
60 0
80 0
100 0
120 0
140 0
160 0
180 0
200 0
220 0
240 0
260 0
280 0
300 0
320 0
340 0
360 0
U-P:GLN-S2
20 0
40 0
60 1

80   2
100   2
120   2
140   1
160   1
180   0
200   0
220   0
240   1
260   2
280   2
300   2
320   1
340   1
360   0
H2U-MY:ASN-S2
20   0
40   0
60   0
80   0
100   0
120   0
140   0
160   0
180   0
200   0
220   0
240   0
260   0
280   0
300   0
320   0
340   0
360   0
G-RIB:GLU-CA
20   0
40   0
60   8
80   11
100   12
120   11
140   9
160   6
180   2
200   0
220   4
240   8
260   11
280   12
300   11
320   9
340   7
360   2
U31-RIB:ASP-CA
20   0
40   0
60   0
80   0

100 0
120 0
140 0
160 0
180 0
200 0
220 0
240 0
260 0
280 0
300 0
320 0
340 0
360 0
C31-MY:GLU-S2
20 0
40 0
60 0
80 0
100 0
120 0
140 0
160 0
180 0
200 0
220 0
240 0
260 0
280 0
300 0
320 0
340 0
360 0
G-R5:MET-CA
20 0
40 0
60 2
80 3
100 3
120 0
140 0
160 0
180 0
200 0
220 0
240 2
260 3
280 3
300 0
320 3
340 2
360 0
U-P:CYS-CA
20 0
40 0
60 0
80 0
100 0

120 0
140 0
160 0
180 0
200 0
220 0
240 0
260 0
280 0
300 0
320 0
340 0
360 0
IU-MY:TYR-CA
20 0
40 0
60 0
80 0
100 0
120 0
140 0
160 0
180 0
200 0
220 0
240 0
260 0
280 0
300 0
320 0
340 0
360 0
FHU-RIB:LEU-CA
20 0
40 0
60 0
80 0
100 0
120 0
140 0
160 0
180 0
200 0
220 0
240 0
260 0
280 0
300 0
320 0
340 0
360 0
FHU-P:ASP-S2
20 0
40 0
60 0
80 0
100 0
120 0

140 0
160 0
180 0
200 0
220 0
240 0
260 0
280 0
300 0
320 0
340 0
360 0
FHU-P:LEU-S2
20 0
40 0
60 0
80 0
100 0
120 0
140 0
160 0
180 0
200 0
220 0
240 0
260 0
280 0
300 0
320 0
340 0
360 0
C-P:PHE-S1
20 0
40 1
60 0
80 3
100 0
120 3
140 2
160 2
180 0
200 0
220 0
240 2
260 3
280 3
300 3
320 0
340 2
360 0
QUO-M5:ARG-CA
20 0
40 0
60 0
80 0
100 0
120 0
140 0

160 0
180 0
200 0
220 0
240 0
260 0
280 0
300 0
320 0
340 0
360 0
U-Y:ASP-S2
20 0
40 1
60 2
80 3
100 4
120 3
140 2
160 2
180 0
200 0
220 1
240 2
260 3
280 4
300 3
320 3
340 2
360 0
U-RIB:CYS-S1
20 0
40 0
60 0
80 0
100 0
120 0
140 0
160 0
180 0
200 0
220 0
240 0
260 0
280 0
300 0
320 0
340 0
360 0
G-P:LYS-S1
20 0
40 0
60 7
80 9
100 10
120 9
140 7
160 5

180 2
200 0
220 4
240 7
260 9
280 10
300 9
320 8
340 6
360 2
C-RIB:PRO-CA
20 0
40 1
60 3
80 4
100 4
120 4
140 3
160 2
180 0
200 0
220 1
240 3
260 4
280 4
300 4
320 3
340 2
360 1
C31-P:ASP-S2
20 0
40 0
60 0
80 0
100 0
120 0
140 0
160 0
180 0
200 0
220 0
240 0
260 0
280 0
300 0
320 0
340 0
360 0
H2U-P:PRO-S1
20 0
40 0
60 0
80 0
100 0
120 0
140 0
160 0
180 0

200 0
220 0
240 0
260 0
280 0
300 0
320 0
340 0
360 0
U-Y:TYR-S1
20 0
40 0
60 1
80 2
100 2
120 1
140 1
160 0
180 0
200 0
220 0
240 1
260 2
280 2
300 2
320 1
340 0
360 0
FHU-MY:PHE-S2
20 0
40 0
60 0
80 0
100 0
120 0
140 0
160 0
180 0
200 0
220 0
240 0
260 0
280 0
300 0
320 0
340 0
360 0
5BU-RIB:PRO-CA
20 0
40 0
60 0
80 0
100 0
120 0
140 0
160 0
180 0
200 0

220   0
240   0
260   0
280   0
300   0
320   0
340   0
360   0
G-P:ILE-CA
20   0
40   0
60   5
80   7
100   8
120   7
140   6
160   4
180   0
200   0
220   0
240   5
260   7
280   8
300   7
320   6
340   4
360   0
G-RIB:SER-CA
20   0
40   3
60   5
80   7
100   8
120   7
140   6
160   4
180   1
200   0
220   3
240   5
260   7
280   8
300   7
320   6
340   4
360   1
C-P:GLU-S2
20   0
40   2
60   5
80   7
100   7
120   7
140   5
160   4
180   1
200   0
220   2

240 5
260 7
280 7
300 7
320 6
340 4
360 1
C-Y:HIS-S1
20 0
40 0
60 1
80 2
100 2
120 2
140 1
160 1
180 0
200 0
220 0
240 1
260 2
280 2
300 2
320 1
340 1
360 0
A-R6:LYS-CA
20 0
40 3
60 5
80 7
100 8
120 7
140 6
160 4
180 0
200 0
220 3
240 5
260 8
280 8
300 7
320 6
340 4
360 0
U31-P:ASN-S1
20 0
40 0
60 0
80 0
100 0
120 0
140 0
160 0
180 0
200 0
220 0
240 0

260 0
280 0
300 0
320 0
340 0
360 0
G-R5:LEU-CA
20 0
40 4
60 9
80 12
100 13
120 12
140 9
160 7
180 0
200 0
220 5
240 9
260 12
280 13
300 12
320 10
340 0
360 3
DA-M6:THR-CA
20 0
40 0
60 0
80 0
100 0
120 0
140 0
160 0
180 0
200 0
220 0
240 0
260 0
280 0
300 0
320 0
340 0
360 0
H2U-RIB:LYS-S2
20 0
40 0
60 0
80 0
100 0
120 0
140 0
160 0
180 0
200 0
220 0
240 0
260 0

280 0
300 0
320 0
340 0
360 0
U-Y:ASP-CA
20 0
40 1
60 2
80 3
100 0
120 3
140 3
160 2
180 0
200 0
220 1
240 2
260 3
280 0
300 3
320 3
340 2
360 0
G-R6:HIS-CA
20 0
40 0
60 2
80 3
100 3
120 3
140 2
160 0
180 0
200 0
220 1
240 2
260 3
280 3
300 3
320 2
340 0
360 0
U-P:TYR-CA
20 0
40 0
60 1
80 2
100 2
120 0
140 1
160 1
180 0
200 0
220 0
240 0
260 2
280 2

300 2
320 1
340 0
360 0
G-RIB:ARG-CA
20 0
40 3
60 7
80 9
100 10
120 9
140 7
160 5
180 2
200 0
220 3
240 7
260 9
280 10
300 9
320 8
340 5
360 2
G-R6:THR-CA
20 0
40 2
60 5
80 7
100 7
120 6
140 5
160 4
180 0
200 0
220 2
240 5
260 7
280 7
300 7
320 6
340 0
360 0
U-Y:GLY-CA
20 0
40 1
60 3
80 4
100 4
120 4
140 3
160 2
180 1
200 0
220 1
240 3
260 4
280 4
300 4

320 3
340 2
360 1
A-R6:ARG-S2
20 0
40 2
60 5
80 7
100 8
120 7
140 6
160 4
180 1
200 0
220 3
240 5
260 7
280 8
300 7
320 6
340 4
360 1
C31-P:ASP-CA
20 0
40 0
60 0
80 0
100 0
120 0
140 0
160 0
180 0
200 0
220 0
240 0
260 0
280 0
300 0
320 0
340 0
360 0
U34-MY:ASN-S2
20 0
40 0
60 0
80 0
100 0
120 0
140 0
160 0
180 0
200 0
220 0
240 0
260 0
280 0
300 0
320 0

340 0
360 0
IU-MY:LEU-CA
20 0
40 0
60 0
80 0
100 0
120 0
140 0
160 0
180 0
200 0
220 0
240 0
260 0
280 0
300 0
320 0
340 0
360 0
G-R6:HIS-S2
20 0
40 1
60 2
80 3
100 3
120 3
140 2
160 2
180 0
200 0
220 1
240 2
260 3
280 3
300 3
320 2
340 0
360 0
A-P:VAL-S1
20 0
40 3
60 6
80 8
100 9
120 8
140 6
160 4
180 1
200 0
220 3
240 6
260 8
280 9
300 8
320 7
340 5

360 2
U-Y:LEU-S2
20 0
40 2
60 4
80 5
100 5
120 5
140 4
160 3
180 1
200 0
220 2
240 4
260 5
280 5
300 5
320 4
340 3
360 1
U31-P:LEU-S1
20 0
40 0
60 0
80 0
100 0
120 0
140 0
160 0
180 0
200 0
220 0
240 0
260 0
280 0
300 0
320 0
340 0
360 0
G-R6:LYS-CA
20 0
40 3
60 7
80 9
100 10
120 9
140 7
160 5
180 2
200 0
220 4
240 7
260 10
280 10
300 9
320 8
340 6
360 2

U-RIB:MET-S2
20 0
40 0
60 0
80 1
100 1
120 1
140 0
160 0
180 0
200 0
220 0
240 1
260 1
280 1
300 1
320 1
340 0
360 0
FHU-MY:ASP-S1
20 0
40 0
60 0
80 0
100 0
120 0
140 0
160 0
180 0
200 0
220 0
240 0
260 0
280 0
300 0
320 0
340 0
360 0
G-R5:GLY-CA
20 1
40 3
60 7
80 10
100 10
120 9
140 7
160 6
180 2
200 0
220 4
240 7
260 10
280 10
300 10
320 8
340 6
360 2
5BU-P:ARG-S2

20 0
40 0
60 0
80 0
100 0
120 0
140 0
160 0
180 0
200 0
220 0
240 0
260 0
280 0
300 0
320 0
340 0
360 0
H2U-MY:GLU-CA
20 0
40 0
60 0
80 0
100 0
120 0
140 0
160 0
180 0
200 0
220 0
240 0
260 0
280 0
300 0
320 0
340 0
360 0
FMU-MY:CYS-S1
20 0
40 0
60 0
80 0
100 0
120 0
140 0
160 0
180 0
200 0
220 0
240 0
260 0
280 0
300 0
320 0
340 0
360 0
U-Y:SER-CA
20 0

40 1
60 2
80 3
100 3
120 3
140 2
160 2
180 0
200 0
220 1
240 2
260 3
280 3
300 3
320 2
340 2
360 0
FMU-P:ASP-S2
20 0
40 0
60 0
80 0
100 0
120 0
140 0
160 0
180 0
200 0
220 0
240 0
260 0
280 0
300 0
320 0
340 0
360 0
G-R6:ALA-CA
20 0
40 4
60 8
80 11
100 12
120 11
140 9
160 7
180 2
200 0
220 4
240 8
260 11
280 12
300 11
320 10
340 7
360 0
A-RIB:GLY-CA
20 0
40 3

60 6
80 8
100 8
120 7
140 6
160 4
180 1
200 0
220 3
240 5
260 8
280 8
300 8
320 6
340 4
360 1
U-Y:CYS-CA
20 0
40 0
60 0
80 0
100 0
120 0
140 0
160 0
180 0
200 0
220 0
240 0
260 0
280 0
300 0
320 0
340 0
360 0
IU-MY:HIS-CA
20 0
40 0
60 0
80 0
100 0
120 0
140 0
160 0
180 0
200 0
220 0
240 0
260 0
280 0
300 0
320 0
340 0
360 0
C-P:GLU-CA
20 0
40 2
60 5

80 7
100 7
120 7
140 5
160 4
180 1
200 0
220 3
240 5
260 7
280 7
300 7
320 6
340 4
360 1
G-R5:TRP-CA
20 0
40 0
60 0
80 1
100 2
120 1
140 0
160 0
180 0
200 0
220 0
240 0
260 1
280 2
300 1
320 0
340 0
360 0
M2G-P:GLU-CA
20 0
40 0
60 0
80 0
100 0
120 0
140 0
160 0
180 0
200 0
220 0
240 0
260 0
280 0
300 0
320 0
340 0
360 0
C31-RIB:GLN-S2
20 0
40 0
60 0
80 0

100 0
120 0
140 0
160 0
180 0
200 0
220 0
240 0
260 0
280 0
300 0
320 0
340 0
360 0
IU-MY:SER-CA
20 0
40 0
60 0
80 0
100 0
120 0
140 0
160 0
180 0
200 0
220 0
240 0
260 0
280 0
300 0
320 0
340 0
360 0
U-RIB:HIS-S2
20 0
40 0
60 1
80 1
100 1
120 1
140 1
160 0
180 0
200 0
220 0
240 1
260 1
280 1
300 1
320 1
340 0
360 0
U34-P:PHE-S2
20 0
40 0
60 0
80 0
100 0

120 0
140 0
160 0
180 0
200 0
220 0
240 0
260 0
280 0
300 0
320 0
340 0
360 0
U-RIB:TYR-CA
20 0
40 0
60 0
80 2
100 2
120 1
140 0
160 1
180 0
200 0
220 0
240 0
260 0
280 2
300 2
320 1
340 1
360 0
DA-M5:TYR-S1
20 0
40 0
60 0
80 0
100 0
120 0
140 0
160 0
180 0
200 0
220 0
240 0
260 0
280 0
300 0
320 0
340 0
360 0
H2U-RIB:ARG-S1
20 0
40 0
60 0
80 0
100 0
120 0

140 0
160 0
180 0
200 0
220 0
240 0
260 0
280 0
300 0
320 0
340 0
360 0
QUO-M5:ARG-S1
20 0
40 0
60 0
80 0
100 0
120 0
140 0
160 0
180 0
200 0
220 0
240 0
260 0
280 0
300 0
320 0
340 0
360 0
A-R5:THR-CA
20 0
40 2
60 4
80 5
100 6
120 5
140 4
160 3
180 0
200 0
220 2
240 4
260 5
280 6
300 5
320 4
340 3
360 0
FHU-P:ALA-CA
20 0
40 0
60 0
80 0
100 0
120 0
140 0

160 0
180 0
200 0
220 0
240 0
260 0
280 0
300 0
320 0
340 0
360 0
QUO-RIB:LEU-S1
20 0
40 0
60 0
80 0
100 0
120 0
140 0
160 0
180 0
200 0
220 0
240 0
260 0
280 0
300 0
320 0
340 0
360 0
FHU-MY:LEU-S2
20 0
40 0
60 0
80 0
100 0
120 0
140 0
160 0
180 0
200 0
220 0
240 0
260 0
280 0
300 0
320 0
340 0
360 0
G-RIB:TYR-S2
20 0
40 1
60 3
80 4
100 5
120 4
140 3
160 2

180 1
200 0
220 1
240 3
260 4
280 5
300 4
320 3
340 2
360 0
DA-M6:THR-S1
20 0
40 0
60 0
80 0
100 0
120 0
140 0
160 0
180 0
200 0
220 0
240 0
260 0
280 0
300 0
320 0
340 0
360 0
QUO-M5:GLU-S1
20 0
40 0
60 0
80 0
100 0
120 0
140 0
160 0
180 0
200 0
220 0
240 0
260 0
280 0
300 0
320 0
340 0
360 0
H2U-MY:TRP-S1
20 0
40 0
60 0
80 0
100 0
120 0
140 0
160 0
180 0

200 0
220 0
240 0
260 0
280 0
300 0
320 0
340 0
360 0
C-Y:GLN-CA
20 0
40 1
60 2
80 3
100 3
120 3
140 2
160 0
180 0
200 0
220 1
240 2
260 3
280 3
300 3
320 0
340 2
360 0
C31-P:GLN-CA
20 0
40 0
60 0
80 0
100 0
120 0
140 0
160 0
180 0
200 0
220 0
240 0
260 0
280 0
300 0
320 0
340 0
360 0
QUO-RIB:LYS-S1
20 0
40 0
60 0
80 0
100 0
120 0
140 0
160 0
180 0
200 0

220 0
240 0
260 0
280 0
300 0
320 0
340 0
360 0
IU-RIB:LYS-S2
20 0
40 0
60 0
80 0
100 0
120 0
140 0
160 0
180 0
200 0
220 0
240 0
260 0
280 0
300 0
320 0
340 0
360 0
G-RIB:PHE-S1
20 0
40 2
60 0
80 5
100 5
120 5
140 4
160 3
180 1
200 0
220 0
240 4
260 5
280 5
300 5
320 4
340 3
360 1
U31-P:GLN-S2
20 0
40 0
60 0
80 0
100 0
120 0
140 0
160 0
180 0
200 0
220 0

240 0
260 0
280 0
300 0
320 0
340 0
360 0
FMU-MY:ASP-S2
20 0
40 0
60 0
80 0
100 0
120 0
140 0
160 0
180 0
200 0
220 0
240 0
260 0
280 0
300 0
320 0
340 0
360 0
U31-MY:GLU-CA
20 0
40 0
60 0
80 0
100 0
120 0
140 0
160 0
180 0
200 0
220 0
240 0
260 0
280 0
300 0
320 0
340 0
360 0
A-RIB:TYR-S1
20 0
40 1
60 2
80 3
100 4
120 3
140 2
160 2
180 0
200 0
220 0
240 0

260   3
280   4
300   3
320   3
340   2
360   0
G-P:GLU-CA
20   0
40   4
60   8
80   11
100   12
120   11
140   9
160   6
180   2
200   0
220   4
240   0
260   11
280   12
300   11
320   9
340   7
360   0
DA-M5:ASN-S1
20   0
40   0
60   0
80   0
100   0
120   0
140   0
160   0
180   0
200   0
220   0
240   0
260   0
280   0
300   0
320   0
340   0
360   0
A-RIB:TRP-S1
20   0
40   0
60   0
80   0
100   1
120   1
140   1
160   0
180   0
200   0
220   0
240   0
260   1

280 1
300 1
320 1
340 0
360 0
U31-P:ASP-CA
20 0
40 0
60 0
80 0
100 0
120 0
140 0
160 0
180 0
200 0
220 0
240 0
260 0
280 0
300 0
320 0
340 0
360 0
U-Y:TRP-CA
20 0
40 0
60 0
80 0
100 0
120 0
140 0
160 0
180 0
200 0
220 0
240 0
260 0
280 0
300 0
320 0
340 0
360 0
FMU-P:ILE-CA
20 0
40 0
60 0
80 0
100 0
120 0
140 0
160 0
180 0
200 0
220 0
240 0
260 0
280 0

300 0
320 0
340 0
360 0
QUO-RIB:ASP-S1
20 0
40 0
60 0
80 0
100 0
120 0
140 0
160 0
180 0
200 0
220 0
240 0
260 0
280 0
300 0
320 0
340 0
360 0
C31-P:MET-CA
20 0
40 0
60 0
80 0
100 0
120 0
140 0
160 0
180 0
200 0
220 0
240 0
260 0
280 0
300 0
320 0
340 0
360 0
A-P:PHE-CA
20 0
40 0
60 3
80 4
100 4
120 4
140 3
160 2
180 1
200 0
220 1
240 3
260 4
280 4
300 4

320 3
340 2
360 0
H2U-P:GLU-S2
20 0
40 0
60 0
80 0
100 0
120 0
140 0
160 0
180 0
200 0
220 0
240 0
260 0
280 0
300 0
320 0
340 0
360 0
FHU-P:TYR-S1
20 0
40 0
60 0
80 0
100 0
120 0
140 0
160 0
180 0
200 0
220 0
240 0
260 0
280 0
300 0
320 0
340 0
360 0
IU-RIB:ILE-CA
20 0
40 0
60 0
80 0
100 0
120 0
140 0
160 0
180 0
200 0
220 0
240 0
260 0
280 0
300 0
320 0

340 0
360 0
A-R5:ASP-S2
20 0
40 2
60 5
80 6
100 7
120 6
140 5
160 4
180 1
200 0
220 2
240 5
260 6
280 7
300 6
320 5
340 4
360 1
C31-P:MET-S1
20 0
40 0
60 0
80 0
100 0
120 0
140 0
160 0
180 0
200 0
220 0
240 0
260 0
280 0
300 0
320 0
340 0
360 0
H2U-P:GLU-S1
20 0
40 0
60 0
80 0
100 0
120 0
140 0
160 0
180 0
200 0
220 0
240 0
260 0
280 0
300 0
320 0
340 0

360 0
A-R5:MET-CA
20 0
40 1
60 2
80 2
100 3
120 2
140 2
160 1
180 0
200 0
220 1
240 2
260 2
280 0
300 2
320 2
340 1
360 0
A-RIB:ASN-CA
20 0
40 1
60 3
80 4
100 4
120 4
140 3
160 2
180 1
200 0
220 1
240 3
260 4
280 4
300 4
320 3
340 2
360 1
G-RIB:PRO-CA
20 0
40 2
60 4
80 6
100 7
120 6
140 5
160 3
180 1
200 0
220 2
240 4
260 6
280 7
300 6
320 5
340 4
360 0

C-RIB:SER-S1
20 0
40 1
60 3
80 5
100 5
120 4
140 3
160 2
180 1
200 0
220 2
240 3
260 5
280 5
300 4
320 4
340 3
360 1
C-RIB:PHE-CA
20 0
40 0
60 2
80 3
100 3
120 3
140 2
160 2
180 0
200 0
220 0
240 0
260 3
280 3
300 3
320 2
340 0
360 0
A-RIB:ILE-CA
20 0
40 0
60 4
80 6
100 6
120 6
140 4
160 3
180 1
200 0
220 2
240 0
260 6
280 6
300 6
320 5
340 3
360 1
G-R5:ALA-CA

20 0
40 4
60 8
80 11
100 12
120 0
140 9
160 7
180 0
200 0
220 4
240 8
260 11
280 12
300 11
320 10
340 7
360 2
QUO-M6:LYS-S1
20 0
40 0
60 0
80 0
100 0
120 0
140 0
160 0
180 0
200 0
220 0
240 0
260 0
280 0
300 0
320 0
340 0
360 0
FMU-MY:MET-CA
20 0
40 0
60 0
80 0
100 0
120 0
140 0
160 0
180 0
200 0
220 0
240 0
260 0
280 0
300 0
320 0
340 0
360 0
FHU-MY:TYR-CA
20 0

40 0
60 0
80 0
100 0
120 0
140 0
160 0
180 0
200 0
220 0
240 0
260 0
280 0
300 0
320 0
340 0
360 0
A-R6:ASP-S2
20 0
40 2
60 5
80 6
100 7
120 6
140 5
160 4
180 1
200 0
220 2
240 5
260 6
280 7
300 6
320 5
340 4
360 1
A-R6:MET-S2
20 0
40 1
60 2
80 2
100 3
120 2
140 2
160 1
180 0
200 0
220 1
240 2
260 2
280 3
300 2
320 2
340 1
360 0
A-P:LYS-S2
20 0
40 3

60   5
80   7
100   8
120   7
140   6
160   4
180   1
200   0
220   3
240   5
260   7
280   8
300   7
320   6
340   4
360   1
A-P:ARG-S2
20   0
40   2
60   5
80   7
100   8
120   7
140   6
160   4
180   1
200   0
220   3
240   5
260   7
280   8
300   7
320   6
340   4
360   1
A-R6:TYR-S2
20   0
40   1
60   2
80   3
100   4
120   3
140   2
160   2
180   0
200   0
220   1
240   2
260   3
280   4
300   3
320   3
340   2
360   0
G-P:SER-S1
20   0
40   3
60   5

80 7
100 8
120 7
140 6
160 4
180 1
200 0
220 3
240 5
260 7
280 8
300 7
320 6
340 4
360 1
G-R6:ILE-S1
20 0
40 0
60 5
80 0
100 8
120 7
140 0
160 4
180 0
200 0
220 0
240 5
260 7
280 8
300 7
320 0
340 0
360 1
A-RIB:PHE-S1
20 0
40 0
60 3
80 4
100 4
120 4
140 3
160 2
180 1
200 0
220 0
240 3
260 4
280 4
300 4
320 3
340 2
360 0
FHU-RIB:TYR-CA
20 0
40 0
60 0
80 0

100 0
120 0
140 0
160 0
180 0
200 0
220 0
240 0
260 0
280 0
300 0
320 0
340 0
360 0
U-P:GLN-CA
20 0
40 0
60 1
80 2
100 2
120 2
140 1
160 1
180 0
200 0
220 0
240 1
260 0
280 2
300 2
320 1
340 1
360 0
IU-RIB:HIS-S2
20 0
40 0
60 0
80 0
100 0
120 0
140 0
160 0
180 0
200 0
220 0
240 0
260 0
280 0
300 0
320 0
340 0
360 0
U31-MY:VAL-S1
20 0
40 0
60 0
80 0
100 0

120 0
140 0
160 0
180 0
200 0
220 0
240 0
260 0
280 0
300 0
320 0
340 0
360 0
C-Y:ALA-CA
20 0
40 2
60 5
80 7
100 8
120 7
140 5
160 4
180 0
200 0
220 3
240 5
260 7
280 8
300 7
320 6
340 4
360 0
FHU-RIB:TYR-S2
20 0
40 0
60 0
80 0
100 0
120 0
140 0
160 0
180 0
200 0
220 0
240 0
260 0
280 0
300 0
320 0
340 0
360 0
QUO-P:PHE-S2
20 0
40 0
60 0
80 0
100 0
120 0

140 0
160 0
180 0
200 0
220 0
240 0
260 0
280 0
300 0
320 0
340 0
360 0
H2U-P:TRP-S1
20 0
40 0
60 0
80 0
100 0
120 0
140 0
160 0
180 0
200 0
220 0
240 0
260 0
280 0
300 0
320 0
340 0
360 0
U-P:PHE-S1
20 0
40 0
60 1
80 2
100 2
120 2
140 1
160 1
180 0
200 0
220 0
240 1
260 2
280 2
300 2
320 2
340 0
360 0
G-R6:SER-S1
20 0
40 3
60 5
80 7
100 8
120 7
140 6

160 4
180 1
200 0
220 3
240 5
260 7
280 8
300 7
320 6
340 4
360 0
FMU-RIB:ASP-S2
20 0
40 0
60 0
80 0
100 0
120 0
140 0
160 0
180 0
200 0
220 0
240 0
260 0
280 0
300 0
320 0
340 0
360 0
A-RIB:LYS-S1
20 0
40 3
60 5
80 7
100 8
120 7
140 6
160 4
180 1
200 0
220 3
240 5
260 8
280 8
300 7
320 6
340 4
360 1
FHU-MY:ILE-CA
20 0
40 0
60 0
80 0
100 0
120 0
140 0
160 0

180 0
200 0
220 0
240 0
260 0
280 0
300 0
320 0
340 0
360 0
C-P:TYR-S2
20 0
40 0
60 2
80 2
100 3
120 2
140 2
160 1
180 0
200 0
220 1
240 2
260 2
280 3
300 2
320 2
340 1
360 0
H2U-P:THR-S1
20 0
40 0
60 0
80 0
100 0
120 0
140 0
160 0
180 0
200 0
220 0
240 0
260 0
280 0
300 0
320 0
340 0
360 0
A-R5:GLN-S1
20 0
40 1
60 3
80 4
100 4
120 3
140 3
160 2
180 0

200 0
220 1
240 3
260 4
280 4
300 4
320 3
340 0
360 0
U34-RIB:TYR-S2
20 0
40 0
60 0
80 0
100 0
120 0
140 0
160 0
180 0
200 0
220 0
240 0
260 0
280 0
300 0
320 0
340 0
360 0
G-R5:MET-S2
20 0
40 1
60 2
80 3
100 0
120 3
140 2
160 2
180 0
200 0
220 1
240 2
260 3
280 3
300 0
320 3
340 2
360 0
G-R5:GLN-S1
20 0
40 1
60 3
80 5
100 5
120 4
140 0
160 3
180 0
200 0

220 2
240 3
260 5
280 5
300 5
320 0
340 3
360 0
H2U-MY:THR-S1
20 0
40 0
60 0
80 0
100 0
120 0
140 0
160 0
180 0
200 0
220 0
240 0
260 0
280 0
300 0
320 0
340 0
360 0
H2U-P:LYS-S2
20 0
40 0
60 0
80 0
100 0
120 0
140 0
160 0
180 0
200 0
220 0
240 0
260 0
280 0
300 0
320 0
340 0
360 0
FMU-P:ARG-S2
20 0
40 0
60 0
80 0
100 0
120 0
140 0
160 0
180 0
200 0
220 0

240 0
260 0
280 0
300 0
320 0
340 0
360 0
G-P:CYS-S1
20 0
40 0
60 0
80 1
100 0
120 1
140 1
160 0
180 0
200 0
220 0
240 0
260 0
280 0
300 0
320 1
340 0
360 0
C-Y:PHE-CA
20 0
40 0
60 2
80 3
100 3
120 3
140 2
160 2
180 0
200 0
220 1
240 0
260 3
280 3
300 3
320 2
340 2
360 0
FMU-MY:GLN-S2
20 0
40 0
60 0
80 0
100 0
120 0
140 0
160 0
180 0
200 0
220 0
240 0

260 0
280 0
300 0
320 0
340 0
360 0
U-Y:GLN-CA
20 0
40 0
60 1
80 2
100 0
120 2
140 1
160 1
180 0
200 0
220 0
240 1
260 2
280 2
300 2
320 1
340 0
360 0
FMU-RIB:MET-S2
20 0
40 0
60 0
80 0
100 0
120 0
140 0
160 0
180 0
200 0
220 0
240 0
260 0
280 0
300 0
320 0
340 0
360 0
C31-RIB:GLU-S2
20 0
40 0
60 0
80 0
100 0
120 0
140 0
160 0
180 0
200 0
220 0
240 0
260 0

280 0
300 0
320 0
340 0
360 0
G-P:PHE-S2
20 0
40 2
60 3
80 5
100 5
120 5
140 4
160 3
180 0
200 0
220 2
240 3
260 5
280 5
300 5
320 4
340 3
360 1
QUO-RIB:GLN-S2
20 0
40 0
60 0
80 0
100 0
120 0
140 0
160 0
180 0
200 0
220 0
240 0
260 0
280 0
300 0
320 0
340 0
360 0
A-RIB:MET-CA
20 0
40 1
60 2
80 2
100 3
120 2
140 2
160 1
180 0
200 0
220 1
240 2
260 2
280 3

300 2
320 2
340 1
360 0
A-P:ALA-CA
20 0
40 3
60 7
80 9
100 10
120 9
140 7
160 5
180 2
200 0
220 0
240 7
260 9
280 10
300 9
320 7
340 5
360 2
DA-M5:HIS-S1
20 0
40 0
60 0
80 0
100 0
120 0
140 0
160 0
180 0
200 0
220 0
240 0
260 0
280 0
300 0
320 0
340 0
360 0
I-RIB:TRP-S2
20 0
40 0
60 0
80 0
100 0
120 0
140 0
160 0
180 0
200 0
220 0
240 0
260 0
280 0
300 0

320 0
340 0
360 0
C31-MY:PHE-S2
20 0
40 0
60 0
80 0
100 0
120 0
140 0
160 0
180 0
200 0
220 0
240 0
260 0
280 0
300 0
320 0
340 0
360 0
C-P:GLY-CA
20 0
40 2
60 4
80 6
100 6
120 6
140 5
160 3
180 1
200 0
220 2
240 4
260 6
280 6
300 6
320 5
340 3
360 1
U-RIB:ASN-S2
20 0
40 0
60 1
80 2
100 2
120 2
140 1
160 1
180 0
200 0
220 1
240 1
260 2
280 2
300 2
320 2

340 1
360 0
FHU-MY:ARG-S1
20 0
40 0
60 0
80 0
100 0
120 0
140 0
160 0
180 0
200 0
220 0
240 0
260 0
280 0
300 0
320 0
340 0
360 0
G-P:ASP-S2
20 0
40 3
60 6
80 8
100 9
120 8
140 6
160 5
180 1
200 0
220 3
240 6
260 8
280 9
300 8
320 7
340 5
360 2
G-R6:MET-CA
20 0
40 1
60 2
80 3
100 3
120 3
140 0
160 0
180 0
200 0
220 0
240 2
260 3
280 3
300 3
320 3
340 2

360 0
IU-RIB:HIS-S1
20 0
40 0
60 0
80 0
100 0
120 0
140 0
160 0
180 0
200 0
220 0
240 0
260 0
280 0
300 0
320 0
340 0
360 0
C-Y:TRP-S2
20 0
40 0
60 0
80 1
100 1
120 1
140 0
160 0
180 0
200 0
220 0
240 0
260 1
280 1
300 1
320 1
340 0
360 0
QUO-M6:GLN-S2
20 0
40 0
60 0
80 0
100 0
120 0
140 0
160 0
180 0
200 0
220 0
240 0
260 0
280 0
300 0
320 0
340 0
360 0

QUO-P:LEU-S2
20 0
40 0
60 0
80 0
100 0
120 0
140 0
160 0
180 0
200 0
220 0
240 0
260 0
280 0
300 0
320 0
340 0
360 0
A-R5:GLY-CA
20 0
40 3
60 6
80 8
100 8
120 7
140 6
160 4
180 1
200 0
220 3
240 5
260 8
280 8
300 8
320 6
340 4
360 1
FMU-RIB:ASN-S1
20 0
40 0
60 0
80 0
100 0
120 0
140 0
160 0
180 0
200 0
220 0
240 0
260 0
280 0
300 0
320 0
340 0
360 0
FMU-RIB:GLN-CA

20 0
40 0
60 0
80 0
100 0
120 0
140 0
160 0
180 0
200 0
220 0
240 0
260 0
280 0
300 0
320 0
340 0
360 0
U31-RIB:PHE-S1
20 0
40 0
60 0
80 0
100 0
120 0
140 0
160 0
180 0
200 0
220 0
240 0
260 0
280 0
300 0
320 0
340 0
360 0
A-R5:GLU-S2
20 0
40 3
60 6
80 9
100 9
120 8
140 7
160 5
180 2
200 0
220 3
240 6
260 9
280 9
300 9
320 7
340 5
360 2
G-R6:ASN-S1
20 0

40   2
60   4
80   5
100   6
120   5
140   4
160   3
180   1
200   0
220   2
240   4
260   5
280   6
300   5
320   4
340   3
360   0
C-RIB:MET-S2
20   0
40   0
60   1
80   2
100   2
120   2
140   1
160   1
180   0
200   0
220   0
240   1
260   2
280   2
300   2
320   1
340   1
360   0
FMU-RIB:HIS-S1
20   0
40   0
60   0
80   0
100   0
120   0
140   0
160   0
180   0
200   0
220   0
240   0
260   0
280   0
300   0
320   0
340   0
360   0
GTP-M6:SER-CA
20   0
40   0

60 0
80 0
100 0
120 0
140 0
160 0
180 0
200 0
220 0
240 0
260 0
280 0
300 0
320 0
340 0
360 0
FHU-RIB:LYS-S1
20 0
40 0
60 0
80 0
100 0
120 0
140 0
160 0
180 0
200 0
220 0
240 0
260 0
280 0
300 0
320 0
340 0
360 0
U31-P:MET-S2
20 0
40 0
60 0
80 0
100 0
120 0
140 0
160 0
180 0
200 0
220 0
240 0
260 0
280 0
300 0
320 0
340 0
360 0
U-RIB:TRP-S2
20 0
40 0
60 0

80 0
100 0
120 0
140 0
160 0
180 0
200 0
220 0
240 0
260 0
280 0
300 0
320 0
340 0
360 0
G-R6:LEU-CA
20 0
40 4
60 9
80 12
100 13
120 12
140 9
160 7
180 0
200 0
220 5
240 9
260 12
280 13
300 12
320 10
340 0
360 0
FHU-RIB:GLY-CA
20 0
40 0
60 0
80 0
100 0
120 0
140 0
160 0
180 0
200 0
220 0
240 0
260 0
280 0
300 0
320 0
340 0
360 0
FHU-P:CYS-S1
20 0
40 0
60 0
80 0

100 0
120 0
140 0
160 0
180 0
200 0
220 0
240 0
260 0
280 0
300 0
320 0
340 0
360 0
C-Y:MET-S1
20 0
40 0
60 1
80 2
100 2
120 0
140 0
160 0
180 0
200 0
220 0
240 1
260 2
280 2
300 2
320 0
340 0
360 0
DA-M6:LYS-S2
20 0
40 0
60 0
80 0
100 0
120 0
140 0
160 0
180 0
200 0
220 0
240 0
260 0
280 0
300 0
320 0
340 0
360 0
A-RIB:ARG-S2
20 0
40 2
60 5
80 7
100 8

120 7
140 6
160 4
180 1
200 0
220 3
240 5
260 7
280 8
300 7
320 6
340 4
360 1
U-RIB:ARG-CA
20 0
40 1
60 3
80 4
100 4
120 4
140 3
160 2
180 0
200 0
220 1
240 3
260 4
280 4
300 4
320 3
340 2
360 1
H2U-MY:PHE-CA
20 0
40 0
60 0
80 0
100 0
120 0
140 0
160 0
180 0
200 0
220 0
240 0
260 0
280 0
300 0
320 0
340 0
360 0
G-R6:THR-S1
20 0
40 2
60 5
80 7
100 7
120 6

140 5
160 4
180 0
200 0
220 2
240 5
260 7
280 7
300 7
320 6
340 4
360 0
G-R5:TYR-S1
20 0
40 0
60 3
80 4
100 5
120 4
140 0
160 2
180 1
200 0
220 1
240 3
260 4
280 5
300 4
320 3
340 0
360 0
U31-MY:GLU-S2
20 0
40 0
60 0
80 0
100 0
120 0
140 0
160 0
180 0
200 0
220 0
240 0
260 0
280 0
300 0
320 0
340 0
360 0
G-P:ARG-CA
20 0
40 3
60 7
80 9
100 10
120 9
140 7

160   5
180   2
200   0
220   3
240   7
260   9
280   10
300   9
320   8
340   5
360   2
G-RIB:ASP-S2
20   0
40   3
60   6
80   8
100   9
120   8
140   6
160   5
180   2
200   0
220   0
240   6
260   8
280   9
300   8
320   7
340   5
360   2
C-P:ASN-S1
20   0
40   1
60   2
80   3
100   3
120   3
140   2
160   2
180   0
200   0
220   1
240   2
260   3
280   3
300   3
320   3
340   2
360   0
U34-P:ARG-S2
20   0
40   0
60   0
80   0
100   0
120   0
140   0
160   0

180 0
200 0
220 0
240 0
260 0
280 0
300 0
320 0
340 0
360 0
H2U-P:ARG-S2
20 0
40 0
60 0
80 0
100 0
120 0
140 0
160 0
180 0
200 0
220 0
240 0
260 0
280 0
300 0
320 0
340 0
360 0
C-RIB:ASN-S2
20 0
40 1
60 2
80 3
100 3
120 3
140 2
160 2
180 0
200 0
220 1
240 2
260 3
280 3
300 3
320 3
340 2
360 0
U31-P:ASN-CA
20 0
40 0
60 0
80 0
100 0
120 0
140 0
160 0
180 0

200 0
220 0
240 0
260 0
280 0
300 0
320 0
340 0
360 0
U34-MY:SER-CA
20 0
40 0
60 0
80 0
100 0
120 0
140 0
160 0
180 0
200 0
220 0
240 0
260 0
280 0
300 0
320 0
340 0
360 0
C-RIB:LYS-CA
20 0
40 2
60 4
80 6
100 6
120 6
140 5
160 3
180 1
200 0
220 2
240 4
260 6
280 6
300 6
320 5
340 3
360 1
U34-P:PRO-S1
20 0
40 0
60 0
80 0
100 0
120 0
140 0
160 0
180 0
200 0

220 0
240 0
260 0
280 0
300 0
320 0
340 0
360 0
A-R5:PRO-S1
20 0
40 2
60 3
80 5
100 5
120 5
140 4
160 0
180 0
200 0
220 2
240 3
260 5
280 5
300 5
320 4
340 3
360 1
I-P:TRP-S2
20 0
40 0
60 0
80 0
100 0
120 0
140 0
160 0
180 0
200 0
220 0
240 0
260 0
280 0
300 0
320 0
340 0
360 0
A-R5:ARG-S1
20 0
40 2
60 5
80 7
100 8
120 7
140 6
160 4
180 1
200 0
220 3

240 5
260 7
280 8
300 7
320 6
340 0
360 1
QUO-M6:LYS-S2
20 0
40 0
60 0
80 0
100 0
120 0
140 0
160 0
180 0
200 0
220 0
240 0
260 0
280 0
300 0
320 0
340 0
360 0
C-P:ASP-S1
20 0
40 2
60 4
80 5
100 6
120 5
140 4
160 3
180 1
200 0
220 2
240 4
260 5
280 6
300 5
320 4
340 3
360 1
FHU-MY:SER-S1
20 0
40 0
60 0
80 0
100 0
120 0
140 0
160 0
180 0
200 0
220 0
240 0

260 0
280 0
300 0
320 0
340 0
360 0
A-P:ILE-S1
20 0
40 2
60 4
80 6
100 6
120 6
140 4
160 3
180 1
200 0
220 2
240 4
260 6
280 6
300 0
320 5
340 3
360 1
U-Y:ILE-S1
20 0
40 0
60 2
80 3
100 3
120 3
140 2
160 0
180 0
200 0
220 1
240 2
260 3
280 3
300 3
320 2
340 2
360 0
G-P:LYS-CA
20 0
40 3
60 7
80 9
100 10
120 9
140 7
160 5
180 2
200 0
220 4
240 7
260 9

280 10
300 9
320 8
340 6
360 2
G-P:GLU-S1
20 0
40 4
60 8
80 11
100 12
120 11
140 9
160 6
180 2
200 0
220 4
240 8
260 11
280 12
300 11
320 9
340 7
360 2
C-RIB:PRO-S1
20 0
40 1
60 3
80 4
100 4
120 4
140 3
160 2
180 0
200 0
220 1
240 3
260 4
280 4
300 4
320 3
340 2
360 1
A-R6:LYS-S1
20 0
40 3
60 5
80 7
100 8
120 7
140 6
160 4
180 0
200 0
220 3
240 5
260 8
280 8

300 7
320 6
340 4
360 0
C-Y:MET-CA
20 0
40 0
60 1
80 2
100 2
120 2
140 0
160 0
180 0
200 0
220 0
240 0
260 2
280 2
300 2
320 0
340 0
360 0
A-RIB:TRP-CA
20 0
40 0
60 1
80 0
100 1
120 1
140 1
160 0
180 0
200 0
220 0
240 1
260 1
280 1
300 1
320 1
340 0
360 0
H2U-P:ASN-S1
20 0
40 0
60 0
80 0
100 0
120 0
140 0
160 0
180 0
200 0
220 0
240 0
260 0
280 0
300 0

320 0
340 0
360 0
A-R6:PRO-S1
20 0
40 2
60 3
80 5
100 5
120 5
140 4
160 3
180 1
200 0
220 2
240 3
260 5
280 5
300 5
320 4
340 3
360 1
C-P:GLU-S1
20 0
40 2
60 5
80 7
100 7
120 7
140 5
160 4
180 1
200 0
220 3
240 5
260 7
280 7
300 7
320 6
340 4
360 1
DA-M6:LEU-S1
20 0
40 0
60 0
80 0
100 0
120 0
140 0
160 0
180 0
200 0
220 0
240 0
260 0
280 0
300 0
320 0

340 0
360 0
C-P:GLN-S1
20 0
40 1
60 2
80 3
100 3
120 3
140 2
160 1
180 0
200 0
220 1
240 2
260 3
280 3
300 3
320 2
340 2
360 0
C-RIB:GLN-S1
20 0
40 1
60 2
80 3
100 3
120 3
140 2
160 1
180 0
200 0
220 1
240 2
260 3
280 3
300 3
320 2
340 2
360 0
U-RIB:HIS-CA
20 0
40 0
60 1
80 1
100 1
120 1
140 1
160 0
180 0
200 0
220 0
240 0
260 1
280 1
300 1
320 1
340 0

360 0
G-R6:TYR-CA
20 0
40 0
60 0
80 4
100 5
120 4
140 3
160 2
180 1
200 0
220 0
240 0
260 4
280 5
300 4
320 3
340 0
360 1
A-P:THR-CA
20 0
40 2
60 4
80 5
100 6
120 5
140 4
160 3
180 1
200 0
220 2
240 4
260 5
280 6
300 5
320 4
340 3
360 1
H2U-RIB:PHE-S2
20 0
40 0
60 0
80 0
100 0
120 0
140 0
160 0
180 0
200 0
220 0
240 0
260 0
280 0
300 0
320 0
340 0
360 0

U-P:ASP-CA
20   0
40   1
60   2
80   3
100   4
120   3
140   3
160   2
180   0
200   0
220   0
240   2
260   3
280   4
300   3
320   3
340   2
360   0
A-RIB:VAL-S1
20   0
40   3
60   6
80   8
100   9
120   8
140   6
160   4
180   1
200   0
220   0
240   6
260   8
280   9
300   8
320   7
340   5
360   2
QUO-M6:LYS-CA
20   0
40   0
60   0
80   0
100   0
120   0
140   0
160   0
180   0
200   0
220   0
240   0
260   0
280   0
300   0
320   0
340   0
360   0
U31-RIB:MET-S2

20 0
40 0
60 0
80 0
100 0
120 0
140 0
160 0
180 0
200 0
220 0
240 0
260 0
280 0
300 0
320 0
340 0
360 0
H2U-P:TRP-CA
20 0
40 0
60 0
80 0
100 0
120 0
140 0
160 0
180 0
200 0
220 0
240 0
260 0
280 0
300 0
320 0
340 0
360 0
G-P:TYR-S2
20 0
40 0
60 3
80 4
100 4
120 4
140 3
160 2
180 1
200 0
220 0
240 3
260 4
280 4
300 4
320 3
340 2
360 0
G-RIB:CYS-S1
20 0

40 0
60 0
80 0
100 0
120 1
140 1
160 0
180 0
200 0
220 0
240 0
260 1
280 0
300 1
320 1
340 0
360 0
A-R5:PRO-CA
20 0
40 2
60 3
80 5
100 5
120 5
140 4
160 0
180 0
200 0
220 2
240 3
260 5
280 5
300 5
320 4
340 3
360 0
DA-M5:THR-S1
20 0
40 0
60 0
80 0
100 0
120 0
140 0
160 0
180 0
200 0
220 0
240 0
260 0
280 0
300 0
320 0
340 0
360 0
QUO-M5:LEU-S2
20 0
40 0

60 0
80 0
100 0
120 0
140 0
160 0
180 0
200 0
220 0
240 0
260 0
280 0
300 0
320 0
340 0
360 0
H2U-MY:GLN-S2
20 0
40 0
60 0
80 0
100 0
120 0
140 0
160 0
180 0
200 0
220 0
240 0
260 0
280 0
300 0
320 0
340 0
360 0
A-P:GLN-S2
20 0
40 1
60 3
80 4
100 4
120 3
140 3
160 2
180 0
200 0
220 1
240 3
260 4
280 4
300 4
320 3
340 2
360 0
G-P:TYR-S1
20 0
40 0
60 3

80 4
100 4
120 4
140 3
160 2
180 0
200 0
220 0
240 3
260 4
280 4
300 4
320 3
340 2
360 1
H2U-MY:LEU-CA
20 0
40 0
60 0
80 0
100 0
120 0
140 0
160 0
180 0
200 0
220 0
240 0
260 0
280 0
300 0
320 0
340 0
360 0
G-RIB:THR-S1
20 0
40 2
60 5
80 7
100 7
120 6
140 5
160 4
180 1
200 0
220 2
240 5
260 7
280 7
300 7
320 6
340 4
360 1
U34-P:GLU-S2
20 0
40 0
60 0
80 0

100 0
120 0
140 0
160 0
180 0
200 0
220 0
240 0
260 0
280 0
300 0
320 0
340 0
360 0
FMU-MY:ARG-S1
20 0
40 0
60 0
80 0
100 0
120 0
140 0
160 0
180 0
200 0
220 0
240 0
260 0
280 0
300 0
320 0
340 0
360 0
G-R5:LEU-S2
20 1
40 4
60 9
80 12
100 13
120 12
140 9
160 7
180 0
200 1
220 5
240 9
260 12
280 13
300 12
320 10
340 0
360 0
G-RIB:MET-CA
20 0
40 0
60 2
80 3
100 3

120 3
140 2
160 2
180 0
200 0
220 0
240 2
260 3
280 3
300 3
320 3
340 2
360 0
H2U-RIB:ASN-S1
20 0
40 0
60 0
80 0
100 0
120 0
140 0
160 0
180 0
200 0
220 0
240 0
260 0
280 0
300 0
320 0
340 0
360 0
U31-P:ARG-CA
20 0
40 0
60 0
80 0
100 0
120 0
140 0
160 0
180 0
200 0
220 0
240 0
260 0
280 0
300 0
320 0
340 0
360 0
U-P:HIS-CA
20 0
40 0
60 1
80 1
100 1
120 1

140 1
160 0
180 0
200 0
220 0
240 1
260 1
280 1
300 1
320 1
340 0
360 0
C31-P:PHE-S2
20 0
40 0
60 0
80 0
100 0
120 0
140 0
160 0
180 0
200 0
220 0
240 0
260 0
280 0
300 0
320 0
340 0
360 0
C-P:HIS-S1
20 0
40 0
60 1
80 2
100 2
120 2
140 1
160 1
180 0
200 0
220 0
240 1
260 2
280 2
300 2
320 1
340 1
360 0
DA-M5:ASP-S1
20 0
40 0
60 0
80 0
100 0
120 0
140 0

160 0
180 0
200 0
220 0
240 0
260 0
280 0
300 0
320 0
340 0
360 0
GTP-M6:GLY-CA
20 0
40 0
60 0
80 0
100 0
120 0
140 0
160 0
180 0
200 0
220 0
240 0
260 0
280 0
300 0
320 0
340 0
360 0
U-Y:VAL-S1
20 0
40 1
60 3
80 4
100 4
120 0
140 3
160 0
180 0
200 0
220 1
240 3
260 4
280 4
300 0
320 3
340 2
360 0
C-Y:PHE-S1
20 0
40 0
60 0
80 3
100 3
120 3
140 2
160 2

180 0
200 0
220 1
240 0
260 3
280 3
300 3
320 2
340 0
360 0
A-R6:LYS-S2
20 0
40 3
60 5
80 7
100 8
120 7
140 6
160 4
180 1
200 0
220 3
240 5
260 7
280 8
300 7
320 6
340 4
360 0
U-Y:TYR-S2
20 0
40 0
60 0
80 2
100 2
120 1
140 1
160 1
180 0
200 0
220 0
240 1
260 2
280 2
300 2
320 1
340 0
360 0
A-RIB:MET-S2
20 0
40 1
60 2
80 2
100 3
120 2
140 2
160 1
180 0

200 0
220 1
240 2
260 2
280 3
300 2
320 2
340 1
360 0
C31-RIB:GLU-CA
20 0
40 0
60 0
80 0
100 0
120 0
140 0
160 0
180 0
200 0
220 0
240 0
260 0
280 0
300 0
320 0
340 0
360 0
QUO-M6:ASP-S2
20 0
40 0
60 0
80 0
100 0
120 0
140 0
160 0
180 0
200 0
220 0
240 0
260 0
280 0
300 0
320 0
340 0
360 0
U31-MY:MET-S1
20 0
40 0
60 0
80 0
100 0
120 0
140 0
160 0
180 0
200 0

220 0
240 0
260 0
280 0
300 0
320 0
340 0
360 0
C-Y:SER-S1
20 0
40 1
60 3
80 5
100 5
120 4
140 3
160 2
180 1
200 0
220 2
240 3
260 5
280 5
300 4
320 4
340 3
360 0
A-RIB:ILE-S1
20 0
40 0
60 4
80 6
100 6
120 6
140 4
160 3
180 1
200 0
220 2
240 0
260 6
280 6
300 6
320 5
340 3
360 1
G-P:VAL-S1
20 0
40 4
60 7
80 10
100 11
120 10
140 8
160 6
180 2
200 0
220 4

240 7
260 10
280 11
300 10
320 8
340 6
360 2
G-P:PHE-CA
20 0
40 0
60 3
80 5
100 5
120 5
140 4
160 3
180 0
200 0
220 2
240 3
260 5
280 5
300 5
320 4
340 3
360 0
GTP-M6:THR-S1
20 0
40 0
60 0
80 0
100 0
120 0
140 0
160 0
180 0
200 0
220 0
240 0
260 0
280 0
300 0
320 0
340 0
360 0
U31-P:GLN-CA
20 0
40 0
60 0
80 0
100 0
120 0
140 0
160 0
180 0
200 0
220 0
240 0

260   0
280   0
300   0
320   0
340   0
360   0
G-RIB:GLN-S1
20   0
40   1
60   3
80   5
100   5
120   4
140   4
160   3
180   1
200   0
220   2
240   3
260   5
280   5
300   5
320   4
340   3
360   1
QUO-M6:PHE-S1
20   0
40   0
60   0
80   0
100   0
120   0
140   0
160   0
180   0
200   0
220   0
240   0
260   0
280   0
300   0
320   0
340   0
360   0
FHU-P:ARG-S2
20   0
40   0
60   0
80   0
100   0
120   0
140   0
160   0
180   0
200   0
220   0
240   0
260   0

280 0
300 0
320 0
340 0
360 0
G-R6:TRP-S2
20 0
40 0
60 0
80 1
100 2
120 1
140 1
160 0
180 0
200 0
220 0
240 1
260 1
280 2
300 1
320 1
340 0
360 0
A-RIB:LYS-S2
20 0
40 3
60 5
80 7
100 8
120 7
140 6
160 4
180 1
200 0
220 3
240 5
260 7
280 8
300 7
320 6
340 4
360 1
C-Y:ARG-S2
20 0
40 2
60 4
80 6
100 6
120 5
140 4
160 3
180 1
200 0
220 2
240 4
260 6
280 6

300 6
320 5
340 3
360 1
C-Y:ASP-CA
20 0
40 2
60 4
80 5
100 6
120 5
140 4
160 3
180 0
200 0
220 2
240 4
260 5
280 6
300 5
320 4
340 3
360 1
U-P:GLU-S1
20 0
40 1
60 3
80 5
100 5
120 4
140 3
160 3
180 1
200 0
220 2
240 3
260 5
280 5
300 5
320 4
340 3
360 0
A-R6:HIS-S1
20 0
40 0
60 2
80 2
100 2
120 2
140 2
160 1
180 0
200 0
220 1
240 2
260 2
280 2
300 2

320 2
340 1
360 0
U34-RIB:SER-S1
20 0
40 0
60 0
80 0
100 0
120 0
140 0
160 0
180 0
200 0
220 0
240 0
260 0
280 0
300 0
320 0
340 0
360 0
A-R6:VAL-CA
20 0
40 3
60 6
80 8
100 9
120 8
140 6
160 4
180 1
200 0
220 0
240 6
260 8
280 9
300 8
320 7
340 5
360 0
DA-M5:ASN-S2
20 0
40 0
60 0
80 0
100 0
120 0
140 0
160 0
180 0
200 0
220 0
240 0
260 0
280 0
300 0
320 0

340 0
360 0
5BU-RIB:SER-S1
20 0
40 0
60 0
80 0
100 0
120 0
140 0
160 0
180 0
200 0
220 0
240 0
260 0
280 0
300 0
320 0
340 0
360 0
G-R5:MET-S1
20 0
40 0
60 2
80 3
100 0
120 3
140 0
160 0
180 0
200 0
220 1
240 0
260 3
280 3
300 3
320 3
340 2
360 0
G-P:TRP-CA
20 0
40 0
60 1
80 1
100 2
120 1
140 0
160 1
180 0
200 0
220 0
240 1
260 1
280 2
300 1
320 1
340 0

360 0
H2U-MY:ASN-S1
20 0
40 0
60 0
80 0
100 0
120 0
140 0
160 0
180 0
200 0
220 0
240 0
260 0
280 0
300 0
320 0
340 0
360 0
FHU-MY:VAL-S1
20 0
40 0
60 0
80 0
100 0
120 0
140 0
160 0
180 0
200 0
220 0
240 0
260 0
280 0
300 0
320 0
340 0
360 0
C-Y:TRP-CA
20 0
40 0
60 0
80 1
100 1
120 0
140 0
160 0
180 0
200 0
220 0
240 0
260 0
280 1
300 0
320 1
340 0
360 0

FHU-MY:LYS-S1
20   0
40   0
60   0
80   0
100   0
120   0
140   0
160   0
180   0
200   0
220   0
240   0
260   0
280   0
300   0
320   0
340   0
360   0
G-P:ASN-S1
20   0
40   2
60   4
80   5
100   6
120   5
140   4
160   3
180   1
200   0
220   2
240   4
260   5
280   6
300   5
320   4
340   3
360   1
C-RIB:VAL-CA
20   0
40   2
60   5
80   6
100   7
120   6
140   5
160   3
180   1
200   0
220   2
240   4
260   6
280   7
300   6
320   5
340   4
360   0
U-Y:LYS-S2

20   0
40   1
60   3
80   4
100   4
120   4
140   3
160   2
180   1
200   0
220   1
240   3
260   4
280   4
300   4
320   3
340   2
360   1
DA-M6:LYS-CA
20   0
40   0
60   0
80   0
100   0
120   0
140   0
160   0
180   0
200   0
220   0
240   0
260   0
280   0
300   0
320   0
340   0
360   0
C31-MY:SER-S1
20   0
40   0
60   0
80   0
100   0
120   0
140   0
160   0
180   0
200   0
220   0
240   0
260   0
280   0
300   0
320   0
340   0
360   0
FHU-RIB:ARG-S2
20   0

40 0
60 0
80 0
100 0
120 0
140 0
160 0
180 0
200 0
220 0
240 0
260 0
280 0
300 0
320 0
340 0
360 0
U34-MY:ASN-CA
20 0
40 0
60 0
80 0
100 0
120 0
140 0
160 0
180 0
200 0
220 0
240 0
260 0
280 0
300 0
320 0
340 0
360 0
C-Y:HIS-CA
20 0
40 0
60 1
80 2
100 2
120 2
140 1
160 1
180 0
200 0
220 0
240 1
260 2
280 2
300 2
320 1
340 0
360 0
U31-MY:TYR-CA
20 0
40 0

60   0
80   0
100   0
120   0
140   0
160   0
180   0
200   0
220   0
240   0
260   0
280   0
300   0
320   0
340   0
360   0
U-RIB:GLY-CA
20   0
40   1
60   3
80   4
100   4
120   4
140   3
160   2
180   1
200   0
220   1
240   3
260   4
280   4
300   4
320   3
340   2
360   1
GTP-M5:THR-CA
20   0
40   0
60   0
80   0
100   0
120   0
140   0
160   0
180   0
200   0
220   0
240   0
260   0
280   0
300   0
320   0
340   0
360   0
G-R6:LYS-S1
20   1
40   3
60   7

80 9
100 10
120 9
140 7
160 5
180 0
200 0
220 4
240 7
260 10
280 10
300 9
320 8
340 6
360 2
U-Y:ARG-CA
20 0
40 1
60 3
80 4
100 4
120 4
140 3
160 2
180 0
200 0
220 1
240 3
260 4
280 4
300 4
320 3
340 2
360 0
A-R6:ARG-S1
20 0
40 2
60 5
80 7
100 8
120 7
140 6
160 4
180 1
200 0
220 3
240 5
260 7
280 8
300 7
320 6
340 4
360 1
C31-MY:PHE-CA
20 0
40 0
60 0
80 0

100   0
120   0
140   0
160   0
180   0
200   0
220   0
240   0
260   0
280   0
300   0
320   0
340   0
360   0
C-P:ILE-CA
20   0
40   0
60   3
80   5
100   5
120   4
140   3
160   3
180   1
200   0
220   0
240   0
260   5
280   5
300   5
320   4
340   3
360   1
FHU-RIB:ILE-CA
20   0
40   0
60   0
80   0
100   0
120   0
140   0
160   0
180   0
200   0
220   0
240   0
260   0
280   0
300   0
320   0
340   0
360   0
IU-RIB:LYS-S1
20   0
40   0
60   0
80   0
100   0

120 0
140 0
160 0
180 0
200 0
220 0
240 0
260 0
280 0
300 0
320 0
340 0
360 0
DA-M5:LEU-S1
20 0
40 0
60 0
80 0
100 0
120 0
140 0
160 0
180 0
200 0
220 0
240 0
260 0
280 0
300 0
320 0
340 0
360 0
U34-P:ASN-S1
20 0
40 0
60 0
80 0
100 0
120 0
140 0
160 0
180 0
200 0
220 0
240 0
260 0
280 0
300 0
320 0
340 0
360 0
U-RIB:LYS-CA
20 0
40 1
60 3
80 4
100 4
120 4

140 3
160 2
180 1
200 0
220 1
240 3
260 4
280 4
300 4
320 3
340 2
360 1
U-Y:PHE-S1
20 0
40 0
60 1
80 2
100 0
120 2
140 0
160 0
180 0
200 0
220 0
240 1
260 2
280 2
300 2
320 2
340 0
360 0
U31-MY:ILE-S1
20 0
40 0
60 0
80 0
100 0
120 0
140 0
160 0
180 0
200 0
220 0
240 0
260 0
280 0
300 0
320 0
340 0
360 0
U31-MY:ALA-S1
20 0
40 0
60 0
80 0
100 0
120 0
140 0

160 0
180 0
200 0
220 0
240 0
260 0
280 0
300 0
320 0
340 0
360 0
U31-P:ASP-S2
20 0
40 0
60 0
80 0
100 0
120 0
140 0
160 0
180 0
200 0
220 0
240 0
260 0
280 0
300 0
320 0
340 0
360 0
C-Y:TYR-CA
20 0
40 0
60 2
80 2
100 3
120 2
140 2
160 1
180 0
200 0
220 1
240 2
260 2
280 3
300 2
320 2
340 1
360 0
G-R5:PHE-S2
20 0
40 2
60 4
80 5
100 5
120 0
140 4
160 0

180 0
200 0
220 2
240 4
260 5
280 5
300 5
320 0
340 0
360 0
U31-RIB:ASN-S1
20 0
40 0
60 0
80 0
100 0
120 0
140 0
160 0
180 0
200 0
220 0
240 0
260 0
280 0
300 0
320 0
340 0
360 0
FMU-RIB:VAL-S1
20 0
40 0
60 0
80 0
100 0
120 0
140 0
160 0
180 0
200 0
220 0
240 0
260 0
280 0
300 0
320 0
340 0
360 0
U31-RIB:GLN-S1
20 0
40 0
60 0
80 0
100 0
120 0
140 0
160 0
180 0

200 0
220 0
240 0
260 0
280 0
300 0
320 0
340 0
360 0
G-RIB:PHE-S2
20 0
40 0
60 0
80 5
100 5
120 5
140 4
160 3
180 1
200 0
220 0
240 4
260 5
280 5
300 0
320 4
340 3
360 1
C-RIB:LEU-S2
20 0
40 3
60 5
80 7
100 8
120 7
140 6
160 4
180 1
200 0
220 0
240 5
260 8
280 8
300 7
320 6
340 4
360 1
C31-P:LEU-S2
20 0
40 0
60 0
80 0
100 0
120 0
140 0
160 0
180 0
200 0

220 0
240 0
260 0
280 0
300 0
320 0
340 0
360 0
H2U-RIB:ARG-S2
20 0
40 0
60 0
80 0
100 0
120 0
140 0
160 0
180 0
200 0
220 0
240 0
260 0
280 0
300 0
320 0
340 0
360 0
G-R6:ASN-S2
20 0
40 0
60 4
80 5
100 6
120 5
140 4
160 3
180 1
200 0
220 2
240 4
260 5
280 6
300 5
320 4
340 3
360 0
A-R5:HIS-CA
20 0
40 1
60 2
80 2
100 2
120 2
140 0
160 1
180 0
200 0
220 1

240 2
260 2
280 2
300 2
320 2
340 1
360 0
U-Y:ALA-S1
20 0
40 2
60 3
80 5
100 5
120 5
140 4
160 3
180 0
200 0
220 0
240 3
260 5
280 5
300 5
320 4
340 3
360 0
A-R5:TRP-CA
20 0
40 0
60 0
80 1
100 1
120 1
140 1
160 0
180 0
200 0
220 0
240 1
260 1
280 1
300 0
320 1
340 0
360 0
5BU-RIB:ARG-S2
20 0
40 0
60 0
80 0
100 0
120 0
140 0
160 0
180 0
200 0
220 0
240 0

260 0
280 0
300 0
320 0
340 0
360 0
H2U-MY:TRP-CA
20 0
40 0
60 0
80 0
100 0
120 0
140 0
160 0
180 0
200 0
220 0
240 0
260 0
280 0
300 0
320 0
340 0
360 0
U-Y:GLU-S1
20 0
40 1
60 3
80 5
100 5
120 0
140 3
160 3
180 1
200 0
220 2
240 3
260 5
280 5
300 5
320 0
340 3
360 0
FHU-MY:THR-CA
20 0
40 0
60 0
80 0
100 0
120 0
140 0
160 0
180 0
200 0
220 0
240 0
260 0

280 0
300 0
320 0
340 0
360 0
A-RIB:PRO-CA
20 0
40 2
60 3
80 5
100 5
120 5
140 4
160 3
180 1
200 0
220 0
240 3
260 5
280 5
300 5
320 4
340 3
360 0
IU-P:LYS-S2
20 0
40 0
60 0
80 0
100 0
120 0
140 0
160 0
180 0
200 0
220 0
240 0
260 0
280 0
300 0
320 0
340 0
360 0
A-R6:SER-CA
20 0
40 2
60 4
80 6
100 6
120 6
140 4
160 3
180 0
200 0
220 2
240 4
260 6
280 6

300 6
320 5
340 3
360 1
U31-RIB:TYR-S2
20 0
40 0
60 0
80 0
100 0
120 0
140 0
160 0
180 0
200 0
220 0
240 0
260 0
280 0
300 0
320 0
340 0
360 0
C31-RIB:ASP-S1
20 0
40 0
60 0
80 0
100 0
120 0
140 0
160 0
180 0
200 0
220 0
240 0
260 0
280 0
300 0
320 0
340 0
360 0
M2G-P:GLU-S2
20 0
40 0
60 0
80 0
100 0
120 0
140 0
160 0
180 0
200 0
220 0
240 0
260 0
280 0
300 0

320 0
340 0
360 0
FMU-MY:GLN-CA
20 0
40 0
60 0
80 0
100 0
120 0
140 0
160 0
180 0
200 0
220 0
240 0
260 0
280 0
300 0
320 0
340 0
360 0
C-P:ASP-S2
20 0
40 2
60 4
80 5
100 5
120 5
140 4
160 3
180 1
200 0
220 2
240 4
260 5
280 5
300 5
320 4
340 3
360 1
FHU-RIB:ASP-S1
20 0
40 0
60 0
80 0
100 0
120 0
140 0
160 0
180 0
200 0
220 0
240 0
260 0
280 0
300 0
320 0

340   0
360   0
A-RIB:CYS-CA
20   0
40   0
60   0
80   1
100   1
120   1
140   0
160   0
180   0
200   0
220   0
240   0
260   0
280   1
300   0
320   1
340   0
360   0
U-P:PRO-CA
20   0
40   1
60   2
80   2
100   3
120   2
140   2
160   1
180   0
200   0
220   1
240   2
260   2
280   3
300   2
320   2
340   1
360   0
FMU-P:GLN-CA
20   0
40   0
60   0
80   0
100   0
120   0
140   0
160   0
180   0
200   0
220   0
240   0
260   0
280   0
300   0
320   0
340   0

360 0
U31-MY:THR-CA
20 0
40 0
60 0
80 0
100 0
120 0
140 0
160 0
180 0
200 0
220 0
240 0
260 0
280 0
300 0
320 0
340 0
360 0
G-RIB:CYS-CA
20 0
40 0
60 0
80 0
100 0
120 1
140 0
160 0
180 0
200 0
220 0
240 0
260 0
280 1
300 1
320 0
340 0
360 0
U34-RIB:ASN-S2
20 0
40 0
60 0
80 0
100 0
120 0
140 0
160 0
180 0
200 0
220 0
240 0
260 0
280 0
300 0
320 0
340 0
360 0

FHU-MY:ASP-CA
20 0
40 0
60 0
80 0
100 0
120 0
140 0
160 0
180 0
200 0
220 0
240 0
260 0
280 0
300 0
320 0
340 0
360 0
C31-MY:PHE-S1
20 0
40 0
60 0
80 0
100 0
120 0
140 0
160 0
180 0
200 0
220 0
240 0
260 0
280 0
300 0
320 0
340 0
360 0
U31-MY:GLN-S2
20 0
40 0
60 0
80 0
100 0
120 0
140 0
160 0
180 0
200 0
220 0
240 0
260 0
280 0
300 0
320 0
340 0
360 0
A-R5:GLU-S1

20   0
40   3
60   6
80   9
100   9
120   8
140   7
160   5
180   0
200   0
220   3
240   6
260   9
280   9
300   9
320   7
340   5
360   2
A-R5:CYS-CA
20   0
40   0
60   0
80   1
100   1
120   0
140   0
160   0
180   0
200   0
220   0
240   0
260   0
280   1
300   0
320   0
340   0
360   0
G-P:TRP-S2
20   0
40   0
60   1
80   1
100   2
120   1
140   1
160   1
180   0
200   0
220   0
240   1
260   1
280   2
300   1
320   1
340   1
360   0
A-R5:PHE-S1
20   0

40 1
60 3
80 4
100 4
120 4
140 0
160 0
180 0
200 0
220 1
240 3
260 4
280 4
300 4
320 0
340 0
360 0
U-RIB:CYS-CA
20 0
40 0
60 0
80 0
100 0
120 0
140 0
160 0
180 0
200 0
220 0
240 0
260 0
280 0
300 0
320 0
340 0
360 0
FMU-MY:ARG-CA
20 0
40 0
60 0
80 0
100 0
120 0
140 0
160 0
180 0
200 0
220 0
240 0
260 0
280 0
300 0
320 0
340 0
360 0
C-P:ARG-CA
20 0
40 2

60 4
80 6
100 6
120 5
140 4
160 3
180 1
200 0
220 2
240 4
260 6
280 6
300 6
320 5
340 3
360 1
DA-M6:GLN-CA
20 0
40 0
60 0
80 0
100 0
120 0
140 0
160 0
180 0
200 0
220 0
240 0
260 0
280 0
300 0
320 0
340 0
360 0
C31-RIB:ASP-CA
20 0
40 0
60 0
80 0
100 0
120 0
140 0
160 0
180 0
200 0
220 0
240 0
260 0
280 0
300 0
320 0
340 0
360 0
A-P:SER-CA
20 0
40 2
60 4

80 6
100 6
120 6
140 4
160 3
180 1
200 0
220 2
240 4
260 6
280 6
300 6
320 5
340 3
360 1
G-P:PRO-S1
20 0
40 2
60 4
80 6
100 7
120 6
140 5
160 3
180 1
200 0
220 2
240 4
260 6
280 7
300 6
320 5
340 4
360 1
IU-MY:VAL-CA
20 0
40 0
60 0
80 0
100 0
120 0
140 0
160 0
180 0
200 0
220 0
240 0
260 0
280 0
300 0
320 0
340 0
360 0
U-Y:VAL-CA
20 0
40 1
60 3
80 4

100 4
120 0
140 3
160 2
180 0
200 0
220 0
240 3
260 4
280 4
300 4
320 3
340 2
360 0
U-Y:ILE-CA
20 0
40 0
60 2
80 3
100 3
120 3
140 2
160 2
180 0
200 0
220 0
240 2
260 3
280 3
300 3
320 2
340 2
360 0
FMU-RIB:VAL-CA
20 0
40 0
60 0
80 0
100 0
120 0
140 0
160 0
180 0
200 0
220 0
240 0
260 0
280 0
300 0
320 0
340 0
360 0
FHU-RIB:LYS-CA
20 0
40 0
60 0
80 0
100 0

120 0
140 0
160 0
180 0
200 0
220 0
240 0
260 0
280 0
300 0
320 0
340 0
360 0
A-R6:HIS-CA
20 0
40 0
60 2
80 2
100 2
120 2
140 2
160 1
180 0
200 0
220 0
240 2
260 2
280 2
300 2
320 2
340 1
360 0
G-RIB:MET-S1
20 0
40 1
60 2
80 3
100 3
120 3
140 2
160 2
180 0
200 0
220 1
240 2
260 3
280 3
300 3
320 3
340 2
360 0
G-R6:TYR-S2
20 0
40 0
60 3
80 4
100 5
120 4

140 3
160 2
180 0
200 0
220 1
240 3
260 4
280 5
300 4
320 3
340 2
360 1
FHU-MY:GLY-CA
20 0
40 0
60 0
80 0
100 0
120 0
140 0
160 0
180 0
200 0
220 0
240 0
260 0
280 0
300 0
320 0
340 0
360 0
C-P:GLN-CA
20 0
40 1
60 2
80 3
100 3
120 3
140 2
160 1
180 0
200 0
220 1
240 2
260 3
280 3
300 3
320 2
340 2
360 0
FHU-P:HIS-S2
20 0
40 0
60 0
80 0
100 0
120 0
140 0

160 0
180 0
200 0
220 0
240 0
260 0
280 0
300 0
320 0
340 0
360 0
C31-P:ASP-S1
20 0
40 0
60 0
80 0
100 0
120 0
140 0
160 0
180 0
200 0
220 0
240 0
260 0
280 0
300 0
320 0
340 0
360 0
C31-P:THR-CA
20 0
40 0
60 0
80 0
100 0
120 0
140 0
160 0
180 0
200 0
220 0
240 0
260 0
280 0
300 0
320 0
340 0
360 0
H2U-MY:ILE-CA
20 0
40 0
60 0
80 0
100 0
120 0
140 0
160 0

180 0
200 0
220 0
240 0
260 0
280 0
300 0
320 0
340 0
360 0
FHU-RIB:ARG-CA
20 0
40 0
60 0
80 0
100 0
120 0
140 0
160 0
180 0
200 0
220 0
240 0
260 0
280 0
300 0
320 0
340 0
360 0
IU-P:LEU-CA
20 0
40 0
60 0
80 0
100 0
120 0
140 0
160 0
180 0
200 0
220 0
240 0
260 0
280 0
300 0
320 0
340 0
360 0
C31-P:GLU-CA
20 0
40 0
60 0
80 0
100 0
120 0
140 0
160 0
180 0

200 0
220 0
240 0
260 0
280 0
300 0
320 0
340 0
360 0
U-RIB:LYS-S2
20 0
40 1
60 3
80 4
100 4
120 4
140 3
160 2
180 1
200 0
220 1
240 3
260 4
280 4
300 4
320 3
340 2
360 1
H2U-MY:GLY-CA
20 0
40 0
60 0
80 0
100 0
120 0
140 0
160 0
180 0
200 0
220 0
240 0
260 0
280 0
300 0
320 0
340 0
360 0
5BU-RIB:ILE-S1
20 0
40 0
60 0
80 0
100 0
120 0
140 0
160 0
180 0
200 0

220 0
240 0
260 0
280 0
300 0
320 0
340 0
360 0
C31-RIB:LEU-S1
20 0
40 0
60 0
80 0
100 0
120 0
140 0
160 0
180 0
200 0
220 0
240 0
260 0
280 0
300 0
320 0
340 0
360 0
G-R5:PHE-S1
20 0
40 0
60 4
80 5
100 5
120 0
140 4
160 3
180 0
200 0
220 2
240 4
260 5
280 5
300 5
320 4
340 0
360 0
A-R5:LYS-S1
20 0
40 3
60 5
80 7
100 8
120 7
140 6
160 4
180 0
200 0
220 3

240   5
260   8
280   8
300   7
320   6
340   4
360   1
A-R5:LEU-S2
20   0
40   0
60   7
80   9
100   10
120   9
140   7
160   0
180   2
200   0
220   4
240   7
260   10
280   10
300   9
320   8
340   6
360   0
EXPECTED_SITE
G:HIS-S2
WoCr   31
Sug   68
Hoo   35
C:GLU-CA
WoCr   67
Sug   147
Hoo   76
A:ASN-CA
WoCr   40
Sug   90
Hoo   47
C:LEU-CA
WoCr   72
Sug   159
Hoo   83
U:ASN-S2
WoCr   22
Sug   49
Hoo   25
G:ARG-CA
WoCr   87
Sug   193
Hoo   100
C:SER-CA
WoCr   45
Sug   100
Hoo   52
A:GLU-CA
WoCr   83
Sug   184

Hoo   96
G:GLN-CA
WoCr   46
Sug   102
Hoo   53
C:ASP-S2
WoCr   49
Sug   109
Hoo   57
C:VAL-S1
WoCr   60
Sug   133
Hoo   69
G:ALA-S1
WoCr   107
Sug   236
Hoo   123
C:GLN-S1
WoCr   29
Sug   65
Hoo   34
G:GLN-S1
WoCr   46
Sug   102
Hoo   53
U:LYS-S2
WoCr   39
Sug   86
Hoo   44
C:ASN-S2
WoCr   32
Sug   71
Hoo   37
A:LYS-S2
WoCr   70
Sug   156
Hoo   81
G:LYS-S1
WoCr   90
Sug   199
Hoo   103
C:GLU-S1
WoCr   66
Sug   147
Hoo   76
C:TRP-S1
WoCr   11
Sug   24
Hoo   12
G:TRP-CA
WoCr   17
Sug   38
Hoo   19
U:GLU-S1
WoCr   46
Sug   101
Hoo   52
G:GLU-S2

WoCr 103
Sug 227
Hoo 118
A:PHE-S1
WoCr 39
Sug 85
Hoo 44
G:CYS-CA
WoCr 14
Sug 31
Hoo 0
A:ASN-S2
WoCr 40
Sug 89
Hoo 46
G:ALA-CA
WoCr 107
Sug 236
Hoo 123
A:ASN-S1
WoCr 40
Sug 90
Hoo 47
G:LEU-CA
WoCr 113
Sug 250
Hoo 130
G:ASN-CA
WoCr 51
Sug 113
Hoo 58
A:TYR-S1
WoCr 33
Sug 74
Hoo 38
C:LEU-S2
WoCr 72
Sug 159
Hoo 83
U:MET-S1
WoCr 14
Sug 31
Hoo 16
C:TYR-S1
WoCr 26
Sug 59
Hoo 30
G:TRP-S1
WoCr 17
Sug 38
Hoo 19
U:THR-S1
WoCr 28
Sug 62
Hoo 32
C:MET-CA
WoCr 20
Sug 45

Hoo 23
C:LYS-S1
WoCr 57
Sug 127
Hoo 66
A:ILE-S1
WoCr 57
Sug 126
Hoo 65
C:ARG-S1
WoCr 56
Sug 123
Hoo 64
G:HIS-CA
WoCr 31
Sug 69
Hoo 36
G:GLN-S2
WoCr 46
Sug 101
Hoo 52
C:LEU-S1
WoCr 72
Sug 159
Hoo 83
U:LYS-CA
WoCr 39
Sug 87
Hoo 45
A:MET-CA
WoCr 25
Sug 56
Hoo 29
G:ASP-CA
WoCr 79
Sug 175
Hoo 91
C:ALA-S1
WoCr 68
Sug 150
Hoo 78
U:PRO-S1
WoCr 26
Sug 57
Hoo 30
C:PHE-CA
WoCr 31
Sug 68
Hoo 35
G:VAL-S1
WoCr 95
Sug 210
Hoo 109
A:TRP-S1
WoCr 13
Sug 30
Hoo 15
U:GLU-S2

WoCr 45
Sug 100
Hoo 52
U:GLN-S1
WoCr 20
Sug 45
Hoo 23
G:ARG-S2
WoCr 87
Sug 192
Hoo 100
G:TYR-S2
WoCr 42
Sug 92
Hoo 48
G:ASN-S2
WoCr 50
Sug 112
Hoo 58
A:HIS-CA
WoCr 25
Sug 55
Hoo 28
U:SER-S1
WoCr 31
Sug 69
Hoo 36
C:VAL-CA
WoCr 60
Sug 133
Hoo 69
G:GLY-CA
WoCr 91
Sug 202
Hoo 105
C:ASP-S1
WoCr 50
Sug 111
Hoo 58
A:HIS-S2
WoCr 24
Sug 54
Hoo 28
A:LEU-S2
WoCr 90
Sug 199
Hoo 104
U:TRP-CA
WoCr 7
Sug 16
Hoo 8
U:ASP-S2
WoCr 34
Sug 75
Hoo 39
A:TRP-CA
WoCr 13
Sug 30

Hoo 15
A:ALA-S1
WoCr 85
Sug 188
Hoo 98
G:HIS-S1
WoCr 31
Sug 69
Hoo 36
A:SER-S1
WoCr 57
Sug 125
Hoo 65
G:GLU-CA
WoCr 105
Sug 231
Hoo 120
U:VAL-CA
WoCr 41
Sug 92
Hoo 48
U:ASN-CA
WoCr 22
Sug 49
Hoo 25
C:ASP-CA
WoCr 50
Sug 111
Hoo 58
U:PHE-S2
WoCr 21
Sug 47
Hoo 24
U:TYR-S2
WoCr 18
Sug 40
Hoo 21
U:TYR-CA
WoCr 18
Sug 40
Hoo 21
C:LYS-CA
WoCr 57
Sug 127
Hoo 66
U:HIS-CA
WoCr 13
Sug 30
Hoo 15
C:GLU-S2
WoCr 65
Sug 145
Hoo 75
A:ALA-CA
WoCr 85
Sug 188
Hoo 98
G:ARG-S1

WoCr   87
Sug   193
Hoo   100
U:CYS-CA
WoCr   6
Sug   13
Hoo   7
U:LEU-S2
WoCr   49
Sug   110
Hoo   57
A:ASP-S2
WoCr   62
Sug   137
Hoo   71
U:ASN-S1
WoCr   22
Sug   49
Hoo   25
G:MET-S2
WoCr   32
Sug   70
Hoo   36
A:GLN-CA
WoCr   37
Sug   81
Hoo   42
U:ARG-CA
WoCr   38
Sug   85
Hoo   44
U:VAL-S1
WoCr   41
Sug   92
Hoo   48
A:PRO-CA
WoCr   47
Sug   105
Hoo   54
U:HIS-S1
WoCr   13
Sug   30
Hoo   15
U:PHE-CA
WoCr   21
Sug   47
Hoo   24
U:MET-S2
WoCr   14
Sug   31
Hoo   16
A:PRO-S1
WoCr   47
Sug   105
Hoo   54
G:ASP-S1
WoCr   79
Sug   175

Hoo   91
A:GLN-S2
WoCr   36
Sug   80
Hoo   42
U:ALA-CA
WoCr   47
Sug   103
Hoo   54
A:ARG-S1
WoCr   70
Sug   154
Hoo   80
U:CYS-S1
WoCr   6
Sug   13
Hoo   7
U:PHE-S1
WoCr   21
Sug   47
Hoo   24
C:GLN-CA
WoCr   29
Sug   65
Hoo   34
G:SER-S1
WoCr   71
Sug   157
Hoo   82
G:PHE-S1
WoCr   48
Sug   107
Hoo   56
G:SER-CA
WoCr   71
Sug   157
Hoo   82
U:MET-CA
WoCr   14
Sug   31
Hoo   16
C:ILE-S1
WoCr   45
Sug   101
Hoo   52
A:GLU-S2
WoCr   82
Sug   181
Hoo   94
U:HIS-S2
WoCr   13
Sug   30
Hoo   15
A:ARG-S2
WoCr   69
Sug   153
Hoo   80
G:PHE-S2

WoCr 48
Sug 107
Hoo 56
G:CYS-S1
WoCr 14
Sug 31
Hoo 0
A:PHE-S2
WoCr 38
Sug 85
Hoo 44
C:GLY-CA
WoCr 58
Sug 128
Hoo 67
G:TRP-S2
WoCr 17
Sug 38
Hoo 19
U:GLU-CA
WoCr 46
Sug 101
Hoo 53
G:TYR-S1
WoCr 42
Sug 93
Hoo 48
U:GLN-S2
WoCr 20
Sug 44
Hoo 23
A:HIS-S1
WoCr 25
Sug 55
Hoo 28
G:LYS-CA
WoCr 90
Sug 199
Hoo 104
U:ILE-CA
WoCr 31
Sug 69
Hoo 36
A:MET-S1
WoCr 25
Sug 56
Hoo 29
C:TRP-CA
WoCr 11
Sug 24
Hoo 12
G:ASN-S1
WoCr 51
Sug 113
Hoo 58
G:PRO-CA
WoCr 59
Sug 131

Hoo 68
A:THR-CA
WoCr 51
Sug 114
Hoo 59
G:LEU-S1
WoCr 113
Sug 250
Hoo 130
A:ILE-CA
WoCr 57
Sug 126
Hoo 65
G:ILE-CA
WoCr 72
Sug 158
Hoo 82
A:ARG-CA
WoCr 70
Sug 154
Hoo 80
C:HIS-S2
WoCr 19
Sug 43
Hoo 22
C:ASN-CA
WoCr 32
Sug 72
Hoo 37
U:GLY-CA
WoCr 40
Sug 88
Hoo 46
A:GLY-CA
WoCr 73
Sug 161
Hoo 84
G:PRO-S1
WoCr 59
Sug 131
Hoo 68
A:LEU-CA
WoCr 90
Sug 200
Hoo 104
C:HIS-CA
WoCr 20
Sug 44
Hoo 23
C:THR-S1
WoCr 41
Sug 91
Hoo 47
C:CYS-S1
WoCr 9
Sug 20
Hoo 10
C:HIS-S1

WoCr 20
Sug 44
Hoo 23
A:TYR-CA
WoCr 33
Sug 74
Hoo 38
A:LEU-S1
WoCr 90
Sug 200
Hoo 104
A:CYS-S1
WoCr 11
Sug 25
Hoo 13
A:CYS-CA
WoCr 11
Sug 25
Hoo 13
C:PHE-S2
WoCr 31
Sug 68
Hoo 35
G:ASP-S2
WoCr 78
Sug 171
Hoo 89
C:CYS-CA
WoCr 9
Sug 20
Hoo 10
G:LEU-S2
WoCr 113
Sug 250
Hoo 130
A:ASP-CA
WoCr 63
Sug 139
Hoo 72
U:LEU-CA
WoCr 50
Sug 110
Hoo 57
G:TYR-CA
WoCr 42
Sug 93
Hoo 48
U:LYS-S1
WoCr 39
Sug 87
Hoo 45
U:ARG-S2
WoCr 38
Sug 84
Hoo 44
C:ILE-CA
WoCr 45
Sug 101

Hoo 52
G:GLU-S1
WoCr 104
Sug 230
Hoo 120
G:ILE-S1
WoCr 72
Sug 158
Hoo 82
A:ASP-S1
WoCr 63
Sug 139
Hoo 72
U:SER-CA
WoCr 31
Sug 69
Hoo 36
U:PRO-CA
WoCr 26
Sug 57
Hoo 30
U:ILE-S1
WoCr 31
Sug 69
Hoo 36
C:TYR-CA
WoCr 26
Sug 59
Hoo 30
U:TRP-S1
WoCr 0
Sug 16
Hoo 8
C:LYS-S2
WoCr 56
Sug 124
Hoo 65
G:MET-CA
WoCr 32
Sug 71
Hoo 37
C:ARG-S2
WoCr 55
Sug 122
Hoo 64
U:ARG-S1
WoCr 38
Sug 85
Hoo 44
C:PHE-S1
WoCr 31
Sug 68
Hoo 35
A:LYS-S1
WoCr 72
Sug 159
Hoo 82
A:VAL-S1

WoCr 76
Sug 167
Hoo 87
A:PHE-CA
WoCr 39
Sug 85
Hoo 44
C:THR-CA
WoCr 41
Sug 91
Hoo 47
A:TRP-S2
WoCr 13
Sug 30
Hoo 15
C:PRO-CA
WoCr 38
Sug 84
Hoo 43
A:MET-S2
WoCr 25
Sug 56
Hoo 29
C:SER-S1
WoCr 45
Sug 100
Hoo 52
C:ALA-CA
WoCr 68
Sug 150
Hoo 78
G:PHE-CA
WoCr 48
Sug 107
Hoo 56
C:ARG-CA
WoCr 56
Sug 123
Hoo 64
U:ALA-S1
WoCr 47
Sug 103
Hoo 54
C:MET-S2
WoCr 20
Sug 45
Hoo 23
C:MET-S1
WoCr 20
Sug 45
Hoo 23
A:GLN-S1
WoCr 37
Sug 81
Hoo 42
A:GLU-S1
WoCr 83
Sug 184

Hoo 96
U:ASP-S1
WoCr 34
Sug 76
Hoo 40
A:LYS-CA
WoCr 72
Sug 159
Hoo 83
U:ASP-CA
WoCr 34
Sug 76
Hoo 40
U:THR-CA
WoCr 28
Sug 62
Hoo 32
C:TRP-S2
WoCr 11
Sug 24
Hoo 12
G:MET-S1
WoCr 32
Sug 71
Hoo 37
C:GLN-S2
WoCr 29
Sug 64
Hoo 33
U:TRP-S2
WoCr 7
Sug 16
Hoo 8
C:ASN-S1
WoCr 32
Sug 72
Hoo 37
A:TYR-S2
WoCr 33
Sug 74
Hoo 38
C:TYR-S2
WoCr 26
Sug 59
Hoo 30
U:TYR-S1
WoCr 18
Sug 40
Hoo 21
U:GLN-CA
WoCr 20
Sug 45
Hoo 23
A:SER-CA
WoCr 57
Sug 125
Hoo 65
G:THR-S1

```
WoCr   64
Sug   142
Hoo   74
G:LYS-S2
WoCr   88
Sug   195
Hoo   102
A:THR-S1
WoCr   51
Sug   114
Hoo   59
A:VAL-CA
WoCr   76
Sug   167
Hoo   87
G:THR-CA
WoCr   64
Sug   142
Hoo   74
U:LEU-S1
WoCr   50
Sug   110
Hoo   57
C:PRO-S1
WoCr   38
Sug   84
Hoo   43
G:VAL-CA
WoCr   95
Sug   210
Hoo   109
```
